# Transcriptome Profiling of Testis during Sexual Maturation Stages in *Eriocheir sinensis* Using Illumina Sequencing

**DOI:** 10.1371/journal.pone.0033735

**Published:** 2012-03-19

**Authors:** Lin He, Qun Wang, Xinkun Jin, Ying Wang, Lili Chen, Lihua Liu, Yang Wang

**Affiliations:** School of Life Sciences, East China Normal University, Shanghai, China; Auburn University, United States of America

## Abstract

The testis is a highly specialized tissue that plays dual roles in ensuring fertility by producing spermatozoa and hormones. Spermatogenesis is a complex process, resulting in the production of mature sperm from primordial germ cells. Significant structural and biochemical changes take place in the seminiferous epithelium of the adult testis during spermatogenesis. The gene expression pattern of testis in Chinese mitten crab (*Eriocheir sinensis*) has not been extensively studied, and limited genetic research has been performed on this species. The advent of high-throughput sequencing technologies enables the generation of genomic resources within a short period of time and at minimal cost. In the present study, we performed *de novo* transcriptome sequencing to produce a comprehensive transcript dataset for testis of *E. sinensis*. In two runs, we produced 25,698,778 sequencing reads corresponding with 2.31 Gb total nucleotides. These reads were assembled into 342,753 contigs or 141,861 scaffold sequences, which identified 96,311 unigenes. Based on similarity searches with known proteins, 39,995 unigenes were annotated based on having a Blast hit in the non-redundant database or ESTscan results with a cut-off E-value above 10^−5^. This is the first report of a mitten crab transcriptome using high-throughput sequencing technology, and all these testes transcripts can help us understand the molecular mechanisms involved in spermatogenesis and testis maturation.

## Introduction

The Chinese mitten crab (*Eriocheir sinensis*) (Henri Milne Edwards 1854) is one of the most important aquaculture species in China and has high commercial value as a food source [1]. *E. sinensis* is a catadromous crustacean with a life-span of about two years. During its complex life cycle, the crab spends most of its life in rivers and lakes [2]. The adults migrate downstream towards estuarine waters, where they reach maturity and mate from November to March before moving into high salinity regions of estuaries where they release the larvae during early spring [3]. This species reproduces only once and dies shortly after. Relative to mammals, mitten crabs require more complicated environments to induce mating and spawning, and unique regulatory mechanisms are involved in crustacean reproduction. Sexual precosity has been reported in cultured Chinese mitten crab populations since development of their intensive aquaculture in the early 1980s [4]. Precocious crabs mature and die early at a small size, that can lead catastrophic losses for farmers and this seriously impacts development of crab aquaculture. The molecular mechanisms underlying Chinese mitten crab sexual precosity remain unclear. Therefore, genetic mechanisms involved in growth, reproduction and immune response of *E. sinensis* are currently active research areas for this economically important aquaculture species.

The testis provides the environment to produce genetically unique male gametes during spermatogenesis. Stringent temporal and spatial expression of genes during both transcriptional and translational processes during protein synthesis is of fundamental importance to ensure the highly ordered processes of spermatogenesis [5]. Spermatogenesis is a highly complex temporal event during which a relatively undifferentiated diploid cell, a spermatogonium, slowly evolves into a highly specialized haploid cell called a spermatozoon [6]. Different genes are expressed in different phases of spermatogenesis that produce proteins with restricted patterns of expression. The expression of these genes is influenced by extrinsic cues, but is determined primarily by intrinsic, genetic programing of spermatogenic cells. Many studies on genes that regulate spermatogenesis have been carried out especially in mammals, but little attention has been paid to such genes in crustaceans. In previous work in our lab, we had identified a total number of 6,287 high quality expressed sequence tags (ESTs), including 3,297 from hepatopancreas [7] and 2,990 from testis [8] from *E. sinensis* based on two cDNA libraries representing 3,829 unigenes, from healthy male mitten crabs at different developmental stages. We then compared relationships between gene expression in the hepatopancreas and in the testis in *E. sinensis* [9]. From this analysis identified some genes involved with regulation of reproduction in the mitten crab, including elongation factor-1 alpha, DNA-binding nuclear protein p8, H2A histone family, endonuclease and reverse transcriptase-like protein. Based on our *E. sinensis* EST libraries, we have also focused on studying the expression levels of many reproduction or immune related genes in different tissues and different developmental stages in specific tissues, including leptin receptor [10], selenoprotein M [11] and cathepsin L [12], that will help us to better understand their functions.

Transcriptome sequencing can yield the subset of genes from the genome that functionally active in a selected tissue and species of interest. In non-model organisms lacking existing genomic resources, for example a fully sequenced genome, obtaining a transcriptome is an effective way to evaluate gene expression and allows comparative studies at the whole genome level [13], [14], [15]. In this report we present a comprehensive analysis of the transcriptome generated from *E. sinensis* and provide a general view of the potential molecular mechanisms involved in male reproduction for this species. This analysis was based on construction of an annotated testis transcriptome library by *de novo* assembly of hundreds of millions of short raw DNA reads generated from high-throughput technology (Illumina/Solexa) without prior genomic sequence information. Global approaches of this type can pave the way to development of a more complete understanding of the complex gene and protein networks that drive the biological and reproductive processes of spermatogenesis.

## Methods

### Animals

Healthy sexually mature male mitten crabs (*E. sinensis*, 150–200 g) that had reached the stage of rapid testis development, were obtained from a commercial crab farm (Caojing Town special aquaculture farm in Jinshan District) near Shanghai, China in October, November and December between 2010. Male crabs were placed in an ice bath for 1–2 min until they were lightly anesthetized. Testes were then removed surgically and immediately frozen in liquid nitrogen and stored at −80°C until required. Testes tissue of three different individuals were selected in October, November and December in 2010 respectively, nine pairs of testis tissue were pooled as one sample for RNA extraction.

### RNA extraction and cDNA library preparation

Total RNA was extracted using QIAzol Lysis Reagent (Qiagen, Shanghai, China) and then purified on RNeasy spin columns (Qiagen) as per the manufacturer's instructions. The RNA integrity (RNA Integrity Score is 6.8) and quantity were determined on an Agilent 2100 Bioanalyzer (Agilent, Shanghai, China) before cDNA synthesis.

Oligo (dT) linked beads were used to isolate poly (A) mRNA after total RNA had been collected from the testes samples. Short mRNA fragments were used as templates to synthesize the first-strand cDNA with random hexamers. The second-strand cDNA was synthesized using buffer, dNTPs, RNase H and DNA polymerase I. The paired-end library was synthesized using the Genomic Sample Prep kit (Illumina, Shenzhen, China) according to the manufacturer's instructions. Short fragments were purified using a QiaQuick PCR extraction kit (Qiagen, Shanghai, China) and resolved with EB buffer for end reparation and adding of poly (A). Following this, the short fragments were connected with sequencing adapters. After agarose gel electrophoresis, suitable fragments were selected for PCR amplification as templates. A mixed cDNA sample representing sexually mature stages undergoing rapid development of testis tissues in adult *E. sinensis* was prepared and sequenced using the Illumina HiSeq™ 2000 and Solexa sequencing technology.

### Assembly

Transcriptome *de novo* assembly was carried out with the short read assembling program SOAPdenovo [16]. All subsequent analyses were based on clean reads. The reads of certain lengths of overlap with no uncalled bases (N) were combined in contigs to form longer fragments. Contigs were then connected using N to represent the unknown sequence between each pair of contigs to form scaffolds. Paired-end reads were used for gap filling of scaffolds to obtain sequences with the smallest number of N's. Such sequences were defined as unigenes. In the final step, Blastx alignments (E-value<10^−5^) between unigenes and sequences in protein databases, including the National Center for Biotechnology Information (NCBI) non-redundant (nr) database, Swiss-Prot, Kyoto Encyclopedia of Genes and Genomes (KEGG) and Clusters of Orthologous Groups (COG) were performed to identify the direction sequence of unigenes. If results of different databases were conflicting, a priority order of alignments from the nr, Swiss-Prot, KEGG and COG databases was followed to decide the sequence direction. When a unigene happened to be unaligned to any sequence in the above databases, the software program ESTScan [17] was used to define the sequence direction. For unigenes with determined sequence directions, we identified their sequences from the 5′ end to 3′ end; for those with undetermined directions, we provided their sequence based on the assembly software.

### Homology searches and functional unigene annotation

Annotation provides information on expression and function of a unigene. In the functional annotation, unigene sequences were first aligned using Blastx to the nr, Swiss-Prot, KEGG and COG protein databases (E-value<10^−5^), retrieving proteins with the highest sequence similarity with the given unigenes along with their protein functional annotations. Homology searches were carried out by query of the NCBI non-redundant protein database using the Blastx algorithm (E-value<10^−5^) [18]. After nr annotation, we used the Blast2GO program [19] to obtain Gene Ontology (GO) annotations, and WEGO software [20] was used to perform GO functional classification of all unigenes in order to understand the distribution of gene functions at the macro level.

Using EC (Enzyme Commission number) terms, biochemical pathway information was collected by downloading relevant maps from the KEGG database (http://www.genome.jp/kegg/) [21]. This database contains systematic analysis of inner-cell metabolic pathways and functions of individual gene products. These pathways are useful in studies of complex biological behaviors. After obtaining the KEGG pathway annotations, unigenes were aligned to the COG database to predict and classify potential functions based on known orthologous gene products. Every protein in COG is assumed to evolve from an ancestor protein, and the whole database is built on coding proteins with complete genomes as well as systematic evolutionary relationships of bacteria, algae and eukaryotic organisms [22].

### Data deposition

*De novo* assembly sequence data from *E. sinensis* were deposited in the National Center for Biotechnology Information (NCBI, USA, http://www.ncbi.nlm.nih.gov/). The full data set is also available from Qun Wang on request (qwang@bio.ecnu.edu.cn).

## Results

### Transcriptome sequencing output, assembly and expression annotation

**Figure 1:**
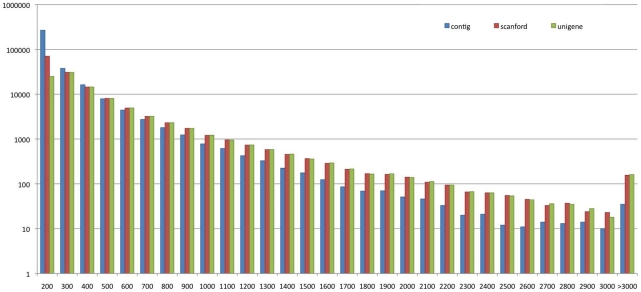
Statistics of Illumina short read assembly quality. The length distributions of *de novo* assemblies of contigs, scaffolds and unigenes are shown (X-axes indicates sequence size (nt), Y-axes indicates number of assembled contigs, scaffolds and unigenes).

*De novo* assembly sequence data from *E. sinensis* were deposited in the Transcriptome Shotgun Assembly (TSA) database with accession numbers JR707930 - JR778295. Illumina high-throughput second generation sequencing produced 25,698,778 clean reads representing with a total of 2,312,890,020 (2.31 Gb) nucleotides (Table 1). Average read size, Q20 percentage and GC content were 90 bp, 91.30%, and 49.17%, respectively. From these short reads, 264,636 contigs were assembled, with a median length of 191 bp. From the contigs, 141,861 scaffolds were constructed using SOAPdenovo, with a median length of 300 bp. The quality of Illumina short read sequence assemblies results are shown in Figure 1, and 96,311 unigenes were obtained, with a median length of 382 bp (Table S1). Altogether we obtained an invaluable resource for further studies of gene functions, protein products and comparative genomics.

**Table 1:** Summary of the transcriptome.

Name	Number of sequences	Mean lengh (bp)
Total reads	25,698,778	90
Total nucleotides (nt)	2,312,890,020	-
Total number of contigs	342,753	191
Total number of scaffolds	141,861	300
Total number of unigenes	96311	382
Sequences with E-value<10^−5^	39,995	-

Distinct gene sequences were first searched with Blastx against the NCBI nr database with a cut-off E-value set at 10^−5^. Using this approach, 29,007 unigenes (30.1% of all unigenes) returned results above the cut-off value. Similarly, up to 10,988 (11.4%) unigenes were annotated via ESTscan analysis. Since no genome or EST information existed for *Eriocheir* species, 58.5% of the unigenes could not be matched to known genes. It is likely that many of the genes of unknown function and/or unknown protein product would share common functions with known genes within the same cluster in the GO clustering analysis.

### GO assignments

**Figure 2:**
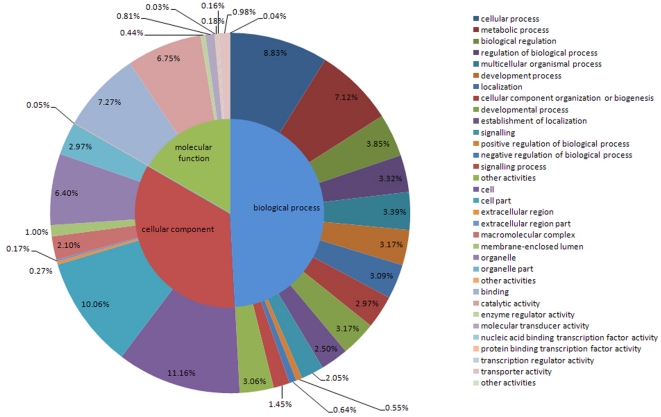
Distribution of GO classifications. Transcripts were classified into three main categories: biological process, cellular component and molecular function.

A total of 96,311 unigenes from *E. sinensis* were assigned for GO analysis based on matches with sequences where the function was known previously. Among these, 60,929 unigenes (63.3% of total) were annotated successfully with confident matches. As many as 29,951 unigenes were found to be involved in biological processes, including cellular process (5383 transcripts with percentages of 17.97%), metabolic process (4341; 14.49%), biological regulation (2347; 7.84%), regulation of biological process (2021; 6.75%), multicellular organismal process (2063; 6.89%), developmental process (1930; 6.44%), localization (1885; 6.29%), cellular component organization or biogenesis (1812; 6.05%), establishment of localization (1524; 5.09%), signaling (1245; 4.16%), reproduction (466; 15.56%), reproductive process (451; 15.06%) and signaling process (883; 2.95%), as well as other activities (1862; 6.22%) (Figure 2).

Moreover, 20,826 transcripts were classified according to a cellular component and could be divided into cell (6,798; 32.64%), cell part (6,132; 29.44%), organelle (3,896; 18.71%), organelle part (1,809; 8.69%), macromolecular complex (1,279; 6.14%), membrane-enclosed lumen (613; 2.94%), extracellular region (165; 0.79%), extracellular region part (106; 0.51%) and others (28; 0.14%), respectively. GO analysis also showed that 10,152 transcripts had potential molecular functions, including binding (4428; 43.62%), catalytic activity (4116; 40.54%), structural molecule activity (11; 0.11%), transporter activity (597; 5.88%), molecular transducer activity (496; 4.89%), enzyme regulator activity (266; 2.62%), transcription regulator activity (96; 0.95%), nucleic acid binding transcription factor activity (20;0.20%), protein binding transcription factor activity (109; 1.07%) and others (24; 0.24%). For Chinese mitten crab, growth and reproduction traits are of particular interest and the GOs for related genes were highlighted and listed in Table S2.

### Metabolic pathways by KEGG analysis

A total of 19,355 unigenes were associated with 220 predicted KEGG metabolic pathways, and the number of unigenes in different pathways ranged from 1 to 2275 (Table S3). The top 25 pathways with highest EST numbers are shown in Table 2, and two major pathways (metabolic pathways and regulation of actin cytoskeleton) included over 3,600 unigenes. The most important pathways that may be relevant to spermatogenesis or reproduction included regulation of actin cytoskeleton (1345), adherens junction (997), focal adhesion (643), chemokine signaling pathway (705), MAPK signaling pathway (527), ubiquitin-mediated proteolysis (400), Fc gamma R-mediated phagocytosis (823), endocytosis (428), splicesome (1145), purine metabolism (518), pyrimidine metabolism (391) and other anti-hyperthermia stress and anti-oxidative stress pathways or gene families. These predicted pathways are likely to be useful in future investigations focussing on their functions in *E. sinensis*.

**Table 2:** Top 25 pathways with the highest EST numbers.

No.	Pathways	Number of ESTs	Pathway ID
1	Metabolic pathways	2275(11.75%)	ko01100
2	Regulation actin cytoskeleton	1345 (6.95%)	ko04810
3	Spliceosome	1145 (5.92%)	ko03040
4	Amoebiasis	1010 (5.22%)	ko05146
5	Adherens junction	997 (5.15%)	ko04520
6	Vibrio cholerae infection	864 (4.46%)	ko05110
7	Phototransduction	848 (4.38%)	ko04744
8	Fc gamma R-mediated phagocytosis	823 (4.25%)	ko04666
9	Olfactory transduction	809 (4.18%)	ko04740
10	Shigellosis	793 (4.1%)	ko05131
11	Bacterial invasion of epithelial cells	788 (4.07%)	ko05100
12	Pathogenic Escherichia coli infection	778 (4.02%)	ko05130
13	Pathways in cancer	741 (3.83%)	ko05200
14	Chemokine signaling pathway	705 (3.64%)	ko04062
15	Focal adhesion	643 (3.32%)	ko04510
16	Huntington's disease	624 (3.22%)	ko05016
17	MAPK signaling pathway	527 (2.72%)	ko04010
18	Purine metabolism	518 (2.68%)	ko00230
19	Protein processing in endoplasmic reticulum	482 (2.49%)	ko04141
20	Endocytosis	428 (2.21%)	ko04144
21	Ubiquitin mediated proteolysis	400 (2.07%)	ko04120
22	Pyrimidine metabolism	391 (2.02%)	ko00240
23	Dorso-ventral axis formation	384 (1.98%)	ko04320
24	Dilated cardiomyopathy	379 (1.96%)	ko05414
25	Hypertrophic cardiomyopathy (HCM)	374 (1.93%)	ko05410

**Figure 3:**
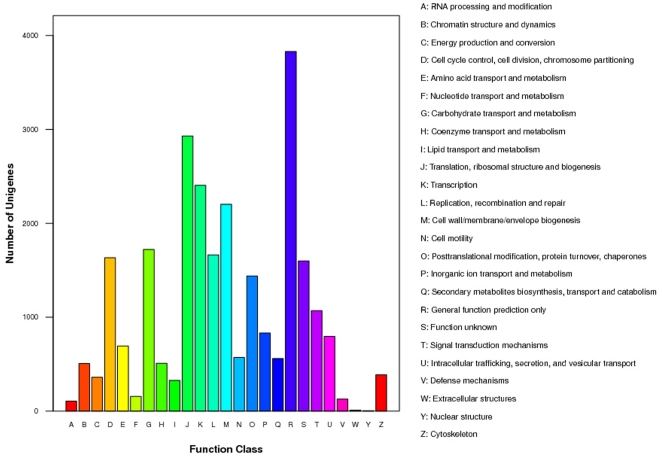
Histogram presentation of clusters of orthologous groups (COG) classification.

Functional annotation using the COG database classified 26,422 unigenes into 25 categories. The highest represented biological processes included “cell cycle control, cell division, chromosome partitioning” (1,632), “post-translational modification, protein turnover, chaperones” (1,439), “transcription” (2,405), “replication, recombination and repair” (1,662), “translation, ribosomal structure and biogenesis” (2,930) and “cell wall/membrane/envelope biogenesis” (2,203) (Figure 3). In total, 3,829 unigenes were assigned into the “general function prediction only” category, and 1,598 unigenes were assigned to the “function unknown” category. Therefore, the inferred functions of 20.5% of unigenes were not resolved.

### Functional genes involved in spermatogenesis and testis development

Brachyuran spermatozoa are characterized by a globular shape, an absence of a flagellum, and the presence of a variable number of radial arms (RA). The mature sperm of the Chinese mitten crab *E. sinensis* is composed of a spherical acrosome, nuclear cup, and about twenty RAs, decondensed chromatin, a thin cytoplasmic layer, and a complex globular acrosome, that is penetrated centrally by the perforatorium. [23]. The spermiogenesis of the Chinese mitten crab *E. sinensis* also involves typical acrosome formation and distinct nuclear structure reorganization [24]. During the early phases of spermatogenesis the nucleus of primary spermatocytes contains typical meiotic figures, and decondensation of chromatin in the mid-spermatid also occurrs. All of these developmental processes involve transcription, replication, recombination and repair, translation, cell division, chromosome partitioning and cell wall/membrane/envelope biogenesis, that were identified in the COG classifications of our dataset.

Transcriptomes analysis provided us an invaluable resource for further studies on the specific mechanisms of spermatogenesis and fertilization in the complex life cycle of *E. sinensis*. Here we reported the most important functional genes involved in spermatogenesis and testis development, including cyclin-related unigenes, ERK1/ERK2 MAPKs; RhoGEF, Rho, Rac, Cdc42, cadherin and PAR3; the DEAD box family of ATP-dependent RNA helicases, DDX51, DDX55, dead box protein 73D (Dbp73), Dbp80, eukaryotic initiation factor 4A (eIF4A); and Ubiquitin Mediated Proteolysis pathway. In the discussion part we tried to elucidate their probably role in reproduction of *E. sinensis* in detail.

## Discussion

In the testis transcriptome of *E. sinensis* described here, the predominant gene clusters were found to be involved with various cellular and metabolic biological processes and functions, including molecular binding and catalytic activities, as well as for forming structural components of the cell or cell organelles. Over 2 million Illumina reads were assembled into 96,311 unigenes, and 29,007 and 10,988 CDSs were predicted by Blastx and ESTScan, respectively. Both gene annotation and pathway analyses helped predict potential genes and their likely specific roles at the whole transcriptome level. Applying Blast analysis and functional annotation (e.g., GO, Swissprot and KEGG) using the assembled gene models from catalogs of other species, we have sampled an extensive and diverse expressed gene catalog for *E. sinensis* representing a large proportion of the genes expressed in testis. Enrichment analyses of GO functions and KEGG pathways lend support to the biological significance of transcriptome profiles derived from short-read sequencing technology. This approach will assist in the discovery and annotation of novel genes that play key roles in spermatogenesis, sperm deposition and transport during crustacean reproduction.

The goal of spermatogenesis is to produce a genetically unique male gamete that can fertilize an ovum ultimately produce offspring and the process involves series of intricate, cellular, proliferative and developmental phases. The testes provide a location to produce and to accumulate spermatozoa, that are then transported via accessory sex glands that modify spermatozoa activity during the different stages. Like other developmental processes, that of testes is strictly regulated in a spatial and temporal manner, requiring specific genes to be turned on and off at specific times and in specific locations [25]. Important signaling pathways potentially involved in regulation of reproduction were also identified based on the KEGG analysis. Here we discuss the most important signaling pathways involved in the various processes during testis development, including mitotic proliferation of spermatogonia, entry into meiosis, recombination, reduction of spermatocyte division, differentiation of haploid spermatids, and elongation and release of spermatozoa. Other genes relevant to this process include cell cycle regulation, regulation of actin cytoskeleton, DEAD box family genes related to spermatogenesis, ubiquitin-mediated proteolysis, heat shock proteins (HSPs) and peroxiredoxin involved in anti-oxidant system. Furthermore, insect hormone biosynthesis, steroid hormone biosynthesis, apoptosis, p53 signaling pathway, DNA replication, oocyte meiosis, neurotrophin signaling pathway, calcium signaling pathway and Wnt signaling pathway, genes may be involved that modulate molecular mechanisms by second messenger signal transductions. These are all potentially important pathways functioning in reproduction, and will be discussed in here.

### Cell cycle proteins involved with spermatogenesis

Spermatogenesis is a highly ordered developmental process of continuous germ cell maturation, and significant structural and biochemical changes take place in the seminiferous epithelium of the adult testis during spermatogenesis [6]. The male meiotic cell cycle reaches completion and haploid spermatids, derived from secondary spermatocytes, start a complex cell differentiation program to form viable sperm. These processes involve both mitotic and meiotic divisions and extensive cellular remodeling through cell cycle phases, that require coordinated activation and inactivation of specific protein kinases [26]. Key among these are the serine/threonine protein kinase complexes composed of a regulatory subunit, cyclin and a catalytic subunit, cyclin-dependent kinase (Cdk). Cdk is activated and deactivated in a timely manner by its cyclin partners and Cdk inhibitor (CkI). Cdk–cyclin complexes are linked to the apoptotic machinery by p53 protein family members, that in turn can regulate the activity of CkI at the transcriptional level [27]. Here we identified 50 cyclin-related unigenes in *E. sinensis*, including G1/S-specific cyclin-D1, cyclin-D3, cyclin A, cyclin B, cyclin-C, cyclin-G2, cyclin H, cyclin-L1, cyclin-L2, cyclin-K and cyclin-Y. The association of cyclin B with Cdk1 is required to regulate phosphorylation state, that in turn controls kinase activity, and cyclin A, cyclin B, cyclin H were confirmed to have important roles in spermatogenesis. In mammals, CDC2/cyclin B1 kinase activity in pachytene spermatocytes is required for the G2→M transition during prophase I. The G2→M transition also requires cyclin A, which may partner with Cdk1 or Cdk2 during meiosis [28]. Cyclins and cyclin-dependent kinases, polo-like and aurora kinases, and the mitogen-activated protein kinase (MAPK) signaling pathway regulate the transit of primary spermatocytes across the blood-testis barrier and contribute to its remodeling during germ cell divisions [29]. The ERK1/ERK2 MAPKs are transiently activated during mitosis, and MAPK activation has been implicated in the spindle assembly checkpoint and in establishing the timing of unperturbed mitosis [30]. All of these pathways are yet to be studied extensively in *E. sinensis*., or other crustacean, so the transcriptome results here may identify the mechanism of testis development at the signal pathway level.

### Function of actin in spermatogenesis

In the post-natal testis, pre-spermatogonia abandon their central location in the testicular cords and migrate to the margin of the cords. Without losing their physical association with somatic Sertoli cells, these pre-spermatogonia initiate a mitotic amplification cell cycle, generating large numbers of interconnected spermatogonia and self-renewing cells located along the wall of the seminiferous tubule [31]. Testis-specific junction adherens, such as the dynamic Sertoli-germ cell adherens junctions and the intercellular junctions of the Sertoli cells, are actin-based junctional structures that are important not only in mechanical adhesion of the cells, but also in morphogenesis and differentiation of germ cells [32]. Spermatogonia do not separate completely after meiosis but remain joined by intercellular bridges, that persist during all stages of spermatogenesis. This facilitates biochemical interactions and synchronizes germ cell maturation. We identified many gene transcripts encoding actin and actin binding proteins in our dataset, that are likely to contribute to regulation of the actin cytoskeleton pathway (Pathway ID: ko04520), including 1,345 unigenes involved in adherens junction (Pathway ID: ko04110) and focal adhesion (Pathway ID: ko04510). The most important genes identified in adherens junction role, included RhoGEF, Rho, Rac, Cdc42, cadherin and PAR3; and those identified in the focal adhesion pathway included Rap1, B-Raf, MEK1, ERK1/2, Elk1 and MLC.

Recently a new model for sperm head elongation based on the acrosome-acroplaxome-manchette complex was proposed for *Drosophila*, and F-actin assembly is considered to be crucial during sperm individualization [33]. Kierszenbaum *et al.* suggested that the acroplaxome, an assembly of an F-actin-keratin-containing cytoskeletal plate, is present in the subacrosomal space in mammalian spermatids [34]. It anchors the developing acrosome to the nuclear envelope during shaping of the spermatid head to secure the acrosome at the corresponding nuclear pole. The actin polymerization and dynamic reorganization of the F-actin cytoskeleton in this special cytoskeletal plate likely allows the developing acrosome to adapt to nuclear envelope shaping. Actin-binding proteins provide another perspective on the role of actin in spermatogenesis. Most actin-binding proteins are found in the actin-rich site, and they bind to actin filaments, modulating their properties and activities [35], especially during assembly and disassembly. The fact that a large number of actin-binding proteins exist in testes suggests an important role for actin dynamics in sperm function. Several newly identified actin-binding proteins include profilin IV [36], acrosome expressed protein 1 (AEP1) [37] and dishevelled-1 [38], but their relative importance and precise function in regulating spermatid morphological changes by reorganizing actin cytoskeleton remain to be determined. In a study of gametogenesis by Maier *et al.*, activity-regulated cytoskeleton-associated protein (ARC), an effector molecule that associates with the actin cytoskeleton, is believed to support a role for actin cytoskeleton in the acrosome formation, the sperm acrosome reaction and in maintaining sperm cell motility. ARC co-localizes with the developing acrosome in spermatids and is present in the acrosomal region of mature sperm, while it is lost to varying degrees during sperm capacitation and in acrosome-reacted sperm [39]. F-actin can play an important role during spermatogenesis, because all kinds of sperm contain actin, although the specific role of actin during spermatogenesis may vary among species, but the mechanisms underlying it are likely to share many similarities.

### DEAD box family genes involved in spermatogenesis

The DEAD (Asp-Glu-Ala-Asp)-box family of RNA helicases modulate RNA structures, which is a crucial step in many fundamental biological processes. This class of proteins participates in several aspects of RNA metabolism and translational events, including pre-mRNA splicing, ribosome biogenesis, nucleo-cytoplasmic transport, translation and RNA decay that ultimately regulate organelle gene expression for specific biological functions [40]. Gene expression in germ cells requires temporal uncoupling of transcription and translation. Two-thirds of the mRNAs in the adult mammalian testis are associated with specific proteins, forming messenger ribonuclear protein (mRNP) particles, and are stored in the cytoplasm of spermatids for translation at specific times when required for progression and completion of spermatogenesis [41].

In the current study, we identified the DEAD box family of ATP-dependent RNA helicases, DDX51, DDX55, dead box protein 73D (Dbp73), Dbp80, eukaryotic initiation factor 4A (eIF4A), other DEAD box proteins that function in germ cell development and reproductive regulation, as well as Piwi-like proteins responsible for maintaining the stability of cells division rates in germ cells in the *E. sinensis* transcriptome. In addition, the vasa-like protein is so far the only one known to be specifically expressed in the germ cell lineage. Its helicase activity is required for translation of at least two mRNAs involved in germ cell migration and development [42]. Piwi proteins bind to and are required for accumulation of small RNAs known as piRNAs. In mice, the three Piwi-related proteins, Miwi, Mili and Miwi2, function primarily in spermatogenesis, and only Miwi2 and Mili have an apparent role in transposon silencing [43]. Indeed, *Drosophila* PIWI is found in the nucleus, where it promotes transcriptionally permissive histone modifications, as well as the expression of a nuclear piRNA that silences a master regulatory locus [44]. We had identified the *vasa* gene in the original *E. sinensis* EST library analysis and studied its expression levels during sexually maturation of *E. sinensis*. This protein was expressed in the gonads specifically. Using qRT-PCR analysis, we showed that *Es-vasa* mRNA transcripts were at their highest levels during periods of rapid development of the gonads (stage III-2 inovaries and spermatocyte stage in testes).

### Ubiquitin-dependent proteolytic system in the testis

Essentially, the success of female and male gametogenesis depends on a balance between two critical processes: the regulation of cell division cycles and the apoptotic dismissal of gamete precursors. Spermatogenic cells also undergo a programmed cell death process or apoptosis. The omnipresent ubiquitin–proteasome system (UPS) is an ATP-dependent enzymatic machinery that targets substrate proteins for degradation by the 26S proteasome by tagging them with an isopeptide chain composed of covalently linked molecules of ubiquitin, a small chaperone protein. Post-translational modification by small ubiquitin-like modifiers or SUMO proteins has been implicated as an important regulatory event in several cellular processes, including transcriptional regulation, protein stability, stress-induced responses, cell cycle progression and the DNA repair process [45]. Sperm proteasomes are essential for successful fertilization, and they function as targets and/or regulators of sperm capacitation. E3 ubiquitin-protein ligase (Ubr2) localizes to meiotic chromatin regions and functions together with the ubiquitin conjugating (E2) enzyme HR6B in histone H2A ubiquitylation during male meiosis [46]. In ascidians, the ubiquitin–proteasome system participates in fertilization, particularly in degradation of the proteinaceous egg coat [47]. Currengtly, no studies have examined the function of ubiquitin-conjugating enzymes in the developing ovary and testis in crustaceans. Most reports of the ubiquitin-proteasome system in crustaceans relate to molting [48]. Shen B *et al.* demonstrated however that ubiquitin conjugating enzymes UBE2r/UBC3/CDC34 were differentially expressed in developing ovary and testis, and may play an important role in oogenesis and spermatogenesis in crustaceans [49].

In the present testis transcriptome data set, we identified many ubiquitin-related genes and constructed the Ubiquitin Mediated Proteolysis pathway by KEGG analysis, that included ubiquitin-activating enzyme E1, E3 ubiquitin-protein ligase RING2, ubiquitin-conjugating enzyme E2, ubiquitin carboxyl-terminal hydrolase, ubiquitin-like modifier-activating enzyme 5, ubiquitin fusion degradation protein 1, ubiquitin-fold modifier 1, E3 ubiquitin-protein ligase mind-bomb and ubiquitin specific peptidase. Other proteins that regulate the ubiquitin-mediated proteolysis pathway were also observed in our study, including the SUMO-activating enzyme, E3 SUMO-protein ligases, RanBP2 and NSE2, and the Cullin protein family. The Cullin-RING ubiquitin-ligase CRL4 controls the cell cycle and response to DNA damage checkpoints, ensuring genomic integrity. Inactivation of the Cul4 component of the CRL4 E3 ligase complex in *Caenorhabditis elegans* by RNA interference has been shown to result in massive mitotic DNA re-replication in blast cells, largely due to failed degradation of the DNA licensing protein, CDT-1, and premature spermatogenesis [50]. Identification of all the genes involved in the ubiquitin-mediated proteolysis pathway that regulates post-translational protein modification in spermiogenesis will provide us with a background of gene interactions in the ubiquitin system.

### Anti-hyperthermia stress and anti-oxidative stress genes in testes

A wide variety of environmental stressors induce cells to rapidly synthesize a distinct set of proteins known as heat shock proteins (HSPs) [51]. HSPs act as molecular chaperones involved in protein folding, assembly and transport, and they play critical roles in the regulation of cell growth, survival and differentiation [52]. Some HSPs have been shown to be involved in basic cellular functions, including as trafficking and translocating proteins in membranes [53]. Recently, several HSPs have been identified with potentially important functions in the male testis [54] [55]. For example, *hsp-70* is strongly expressed in spermatogenic cells in normal testicular tissue with lower expression noted in tissue showing spermatogenic arrest at the spermatocyte and round spermatid stage [56]. In mammalian spermatocytes, cell division cycle protein 2 (CDC2)/cyclin B1 and the chaperone heat shock protein A2 (HSPA2) are required for the G2→M transition in prophase I, while HSPA2 is necessary for activation of CDC2 (CDK1) to form the active CDC2/cyclin B1 complex [57] [58]. This may provide a link between synapsed chromosomes and the cell cycle component CDC2. The chaperone activity of HSPA2 as well as that of other HSP proteins is controlled by their nucleotide-binding domain, that binds and hydrolyses ATP [59]. HSP90 is present in the cytoplasm of all male germ cell types during mouse spermatogenesis, but it is detected mainly in spermatogonia and elongated spermatids in rabbit testis [60]. Here we identified four HSPs in the *E. sinensis* testis transcriptome dataset, including HSP70 (HSPA), HSP40, HSP90-2 and HSP60, as well as other small HSPs. Compared with mammals however, the number of HSPs identified in *E. sinensis* was limited, and this may be related to the evolutionary distance between the species.

All aerobic organisms have evolved efficient and specific defense systems to detoxify dangerous oxidants rapidly in particular hydrogen peroxide and superoxide [61]. The peroxisomal metabolic pathways are essential for normal spermatogenesis, while oxidative stress is also needed for several sperm-specific physiological processes including capacitation, acrosome reaction and sperm-oocyte fusion [62]. Here we identified 206 unigenes involved in the peroxisome pathway, such as peroxiredoxin (PRDX 3) and extracellular superoxide dismutase (SOD). The emergence of unprocessed peroxiredoxin (PRDX 3 or 4) at the spermiogenic stage indicates that important processing enzyme activity is suppressed at lower temperatures during spermiogenesis in the testes. This suggests that the temperature sensitivity of spermatogenesis can, in part, be explained by alteration in enzyme processing activity [63]. PRDX 4 is involved potentially in acrosome formation during spermiogenesis of rats in the membrane-bound form. It is present in the lumen of the endoplasmic reticulum, Golgi bodies and perinuclear space in young rat testes. The conversion of the soluble form to the membrane-bound form of the enzyme may have a role in acrosome formation during vesicular reorganization during spermiogenesis [64]. Peroxiredoxin 2 (PRDX2) is a highly efficient redox protein that neutralizes hydrogen peroxide, resulting in protection of cells from oxidative damage and in regulation of peroxide-mediated signal transduction events [65]. Such mechanisms protect sperm cells by decreasing DNA damage and inhibiting apoptosis during spermatogenesis, that would otherwise leads to accumulation of damaged cells in the ejaculate.

For many farmed aquatic species like mitten crab, economically important traits particularly growth and reproduction are of particular interest. Sequence information and annotations based on Blast, GO and KEGG analyses all provided valuable information for studying the molecular basis of these important traits in *E. sinensis*. Transcripts that putatively influence growth (GO: 0042065) and reproduction (GO: 0030154) are shown in Table S2. Among them, genes encoding different groups of growth factors and specific receptors involved in cell growth were identified. These include epidermal growth factor domains and receptors, transforming growth factors and receptors, insulin-like growth factor receptors and fibroblast growth factor and receptors. Transcripts of other proteins related directly to reproduction were also observed in our study, including fertilin or ADAM 10 and 11, sperm protamine, cathepsin, testis-expressed sequence 264, testis-specific protein kinase 1, testis development protein NYD-SP11, testis kinesin-like protein KIFC1, mitogen-activated protein kinase (MAPK), SP17 (sperm protein), A-kinase anchor protein (AKAP), reproductive homeobox 8 (Rhox8), and male reproductive-related LIM protein. These critical reproduction specific genes and proteins regulate the development of testes or spermatogenesis, but their sepcific functions vary in different species/taxonomic groups. Overall, functional analyses of our Illumina dataset identified many candidate genes potentially involved in reproduction, and we propose to focus on functional studies in the future work to understand the molecular basis of reproduction in crustacean species. Our results illustrate the utility of Illumina second generation sequencing as a basis for defining metabolic pathways and tissue specific functional genomics in a non-model species.

## Supporting Information

Table S1 **Sequences with significant BLAST matches against Nr and Swiss-Prot database.**
(XLS)

Table S2 **GOs of growth and reproduction related genes.**
(XLS)

Table S3 **KEGG biochemical mappings for** ***E. sinensis*** **.**
(DOC)
