# Correction: Transcriptome Profiling of Testis during Sexual Maturation Stages in *Eriocheir sinensis* Using Illumina Sequencing

**DOI:** 10.1371/annotation/9c2ccae9-3f9e-474c-9e48-f1a2c02ccf22

**Published:** 2012-08-09

**Authors:** Lin He, Qun Wang, Xingkun Jin, Ying Wang, Lili Chen, Lihua Liu, Yang Wang

The third author's name was spelled incorrectly. The correct name is: Xingkun Jin.

