# Supplementary material for: Transcriptome Profiling of Testis during Sexual Maturation Stages in Eriocheir sinensis Using Illumina Sequencing
Source: PLoS One. 2012 Mar 19;7(3):e33735. doi: 10.1371/journal.pone.0033735 (PMC3307765; doi:10.1371/journal.pone.0033735)
Supplement: Supplementary file 1 [file pone.0033735.s003.doc]

JingChaoZuZhi2-Unigene.fa



|  |  |  |  |
| --- | --- | --- | --- |
| **1. JingChaoZuZhi2-Unigene.fa** | | | |
| **#** | **Pathway** | **Count (19355)** | **Pathway ID** |
| 1 | Metabolic pathways | 2275 | ko01100 |
| 2 | Regulation of actin cytoskeleton | 1345 | ko04810 |
| 3 | Spliceosome | 1145 | ko03040 |
| 4 | Amoebiasis | 1010 | ko05146 |
| 5 | Adherens junction | 997 | ko04520 |
| 6 | Vibrio cholerae infection | 864 | ko05110 |
| 7 | Phototransduction | 848 | ko04744 |
| 8 | Fc gamma R-mediated phagocytosis | 823 | ko04666 |
| 9 | Olfactory transduction | 809 | ko04740 |
| 10 | Shigellosis | 793 | ko05131 |
| 11 | Bacterial invasion of epithelial cells | 788 | ko05100 |
| 12 | Pathogenic Escherichia coli infection | 778 | ko05130 |
| 13 | Pathways in cancer | 741 | ko05200 |
| 14 | Chemokine signaling pathway | 705 | ko04062 |
| 15 | Focal adhesion | 643 | ko04510 |
| 16 | Huntington's disease | 624 | ko05016 |
| 17 | MAPK signaling pathway | 527 | ko04010 |
| 18 | Purine metabolism | 518 | ko00230 |
| 19 | Protein processing in endoplasmic reticulum | 482 | ko04141 |
| 20 | Endocytosis | 428 | ko04144 |
| 21 | Ubiquitin mediated proteolysis | 400 | ko04120 |
| 22 | Pyrimidine metabolism | 391 | ko00240 |
| 23 | Dorso-ventral axis formation | 384 | ko04320 |
| 24 | Dilated cardiomyopathy | 379 | ko05414 |
| 25 | Hypertrophic cardiomyopathy (HCM) | 374 | ko05410 |
| 26 | Tight junction | 361 | ko04530 |
| 27 | Phagosome | 353 | ko04145 |
| 28 | Lysosome | 352 | ko04142 |
| 29 | Wnt signaling pathway | 351 | ko04310 |
| 30 | Salivary secretion | 349 | ko04970 |
| 31 | Amyotrophic lateral sclerosis (ALS) | 348 | ko05014 |
| 32 | Alzheimer's disease | 344 | ko05010 |
| 33 | Vascular smooth muscle contraction | 338 | ko04270 |
| 34 | ECM-receptor interaction | 336 | ko04512 |
| 35 | Calcium signaling pathway | 320 | ko04020 |
| 36 | Neuroactive ligand-receptor interaction | 316 | ko04080 |
| 37 | Lysine degradation | 305 | ko00310 |
| 38 | Cell cycle | 293 | ko04110 |
| 39 | Axon guidance | 270 | ko04360 |
| 40 | Viral myocarditis | 257 | ko05416 |
| 41 | Leukocyte transendothelial migration | 253 | ko04670 |
| 42 | Cardiac muscle contraction | 250 | ko04260 |
| 43 | RNA polymerase | 249 | ko03020 |
| 44 | Insulin signaling pathway | 237 | ko04910 |
| 45 | Hedgehog signaling pathway | 236 | ko04340 |
| 46 | Basal cell carcinoma | 226 | ko05217 |
| 47 | Pancreatic secretion | 223 | ko04972 |
| 48 | Peroxisome | 206 | ko04146 |
| 49 | RNA degradation | 202 | ko03018 |
| 50 | Neurotrophin signaling pathway | 196 | ko04722 |
| 51 | Phosphatidylinositol signaling system | 194 | ko04070 |
| 52 | T cell receptor signaling pathway | 192 | ko04660 |
| 53 | Arrhythmogenic right ventricular cardiomyopathy (ARVC) | 188 | ko05412 |
| 54 | Melanogenesis | 187 | ko04916 |
| 55 | Vasopressin-regulated water reabsorption | 186 | ko04962 |
| 56 | Gap junction | 183 | ko04540 |
| 57 | Prostate cancer | 179 | ko05215 |
| 58 | VEGF signaling pathway | 178 | ko04370 |
| 59 | Long-term potentiation | 178 | ko04720 |
| 60 | Parkinson's disease | 176 | ko05012 |
| 61 | Progesterone-mediated oocyte maturation | 176 | ko04914 |
| 62 | Gastric acid secretion | 172 | ko04971 |
| 63 | Notch signaling pathway | 172 | ko04330 |
| 64 | Jak-STAT signaling pathway | 171 | ko04630 |
| 65 | Oocyte meiosis | 171 | ko04114 |
| 66 | Oxidative phosphorylation | 170 | ko00190 |
| 67 | Natural killer cell mediated cytotoxicity | 167 | ko04650 |
| 68 | GnRH signaling pathway | 164 | ko04912 |
| 69 | Small cell lung cancer | 164 | ko05222 |
| 70 | Inositol phosphate metabolism | 158 | ko00562 |
| 71 | B cell receptor signaling pathway | 153 | ko04662 |
| 72 | Glycolysis / Gluconeogenesis | 151 | ko00010 |
| 73 | Renal cell carcinoma | 146 | ko05211 |
| 74 | ErbB signaling pathway | 133 | ko04012 |
| 75 | Chagas disease | 132 | ko05142 |
| 76 | Cell adhesion molecules (CAMs) | 132 | ko04514 |
| 77 | Basal transcription factors | 128 | ko03022 |
| 78 | Glycerophospholipid metabolism | 127 | ko00564 |
| 79 | TGF-beta signaling pathway | 126 | ko04350 |
| 80 | Valine, leucine and isoleucine degradation | 125 | ko00280 |
| 81 | Riboflavin metabolism | 123 | ko00740 |
| 82 | Prion diseases | 123 | ko05020 |
| 83 | Amino sugar and nucleotide sugar metabolism | 119 | ko00520 |
| 84 | Long-term depression | 118 | ko04730 |
| 85 | Phototransduction - fly | 118 | ko04745 |
| 86 | Systemic lupus erythematosus | 117 | ko05322 |
| 87 | Fatty acid metabolism | 117 | ko00071 |
| 88 | ABC transporters | 116 | ko02010 |
| 89 | Nucleotide excision repair | 115 | ko03420 |
| 90 | Colorectal cancer | 112 | ko05210 |
| 91 | Adipocytokine signaling pathway | 109 | ko04920 |
| 92 | PPAR signaling pathway | 107 | ko03320 |
| 93 | Ribosome | 107 | ko03010 |
| 94 | Chronic myeloid leukemia | 107 | ko05220 |
| 95 | Aminoacyl-tRNA biosynthesis | 106 | ko00970 |
| 96 | Starch and sucrose metabolism | 105 | ko00500 |
| 97 | Epithelial cell signaling in Helicobacter pylori infection | 103 | ko05120 |
| 98 | DNA replication | 103 | ko03030 |
| 99 | Base excision repair | 101 | ko03410 |
| 100 | Drug metabolism - other enzymes | 100 | ko00983 |
| 101 | Glutathione metabolism | 99 | ko00480 |
| 102 | Fc epsilon RI signaling pathway | 98 | ko04664 |
| 103 | Glycerolipid metabolism | 98 | ko00561 |
| 104 | Antigen processing and presentation | 98 | ko04612 |
| 105 | Hematopoietic cell lineage | 96 | ko04640 |
| 106 | Endometrial cancer | 94 | ko05213 |
| 107 | Tyrosine metabolism | 93 | ko00350 |
| 108 | Arginine and proline metabolism | 93 | ko00330 |
| 109 | Propanoate metabolism | 92 | ko00640 |
| 110 | Type II diabetes mellitus | 92 | ko04930 |
| 111 | mTOR signaling pathway | 91 | ko04150 |
| 112 | N-Glycan biosynthesis | 89 | ko00510 |
| 113 | Citrate cycle (TCA cycle) | 89 | ko00020 |
| 114 | Cytokine-cytokine receptor interaction | 89 | ko04060 |
| 115 | Pyruvate metabolism | 88 | ko00620 |
| 116 | NOD-like receptor signaling pathway | 86 | ko04621 |
| 117 | Complement and coagulation cascades | 86 | ko04610 |
| 118 | Pancreatic cancer | 86 | ko05212 |
| 119 | beta-Alanine metabolism | 83 | ko00410 |
| 120 | Glioma | 83 | ko05214 |
| 121 | Toll-like receptor signaling pathway | 82 | ko04620 |
| 122 | p53 signaling pathway | 81 | ko04115 |
| 123 | Apoptosis | 81 | ko04210 |
| 124 | Galactose metabolism | 80 | ko00052 |
| 125 | Glycine, serine and threonine metabolism | 79 | ko00260 |
| 126 | Fructose and mannose metabolism | 79 | ko00051 |
| 127 | Alanine, aspartate and glutamate metabolism | 77 | ko00250 |
| 128 | Tryptophan metabolism | 76 | ko00380 |
| 129 | Proteasome | 76 | ko03050 |
| 130 | Non-small cell lung cancer | 75 | ko05223 |
| 131 | Cysteine and methionine metabolism | 73 | ko00270 |
| 132 | Malaria | 72 | ko05144 |
| 133 | MAPK signaling pathway - fly | 72 | ko04013 |
| 134 | Circadian rhythm - mammal | 69 | ko04710 |
| 135 | Butanoate metabolism | 69 | ko00650 |
| 136 | Acute myeloid leukemia | 67 | ko05221 |
| 137 | RIG-I-like receptor signaling pathway | 67 | ko04622 |
| 138 | Aldosterone-regulated sodium reabsorption | 67 | ko04960 |
| 139 | Mismatch repair | 67 | ko03430 |
| 140 | Retinol metabolism | 66 | ko00830 |
| 141 | Metabolism of xenobiotics by cytochrome P450 | 65 | ko00980 |
| 142 | Drug metabolism - cytochrome P450 | 65 | ko00982 |
| 143 | Circadian rhythm - fly | 65 | ko04711 |
| 144 | Leishmaniasis | 63 | ko05140 |
| 145 | Arachidonic acid metabolism | 61 | ko00590 |
| 146 | Nicotinate and nicotinamide metabolism | 58 | ko00760 |
| 147 | Linoleic acid metabolism | 56 | ko00591 |
| 148 | Limonene and pinene degradation | 56 | ko00903 |
| 149 | Biosynthesis of unsaturated fatty acids | 56 | ko01040 |
| 150 | Sphingolipid metabolism | 56 | ko00600 |
| 151 | Ether lipid metabolism | 55 | ko00565 |
| 152 | Pentose phosphate pathway | 52 | ko00030 |
| 153 | Homologous recombination | 52 | ko03440 |
| 154 | Melanoma | 51 | ko05218 |
| 155 | Cytosolic DNA-sensing pathway | 49 | ko04623 |
| 156 | Collecting duct acid secretion | 48 | ko04966 |
| 157 | alpha-Linolenic acid metabolism | 48 | ko00592 |
| 158 | Other glycan degradation | 48 | ko00511 |
| 159 | Renin-angiotensin system | 47 | ko04614 |
| 160 | Thyroid cancer | 46 | ko05216 |
| 161 | SNARE interactions in vesicular transport | 46 | ko04130 |
| 162 | Glycosaminoglycan biosynthesis - chondroitin sulfate | 44 | ko00532 |
| 163 | Bladder cancer | 44 | ko05219 |
| 164 | Glycosylphosphatidylinositol(GPI)-anchor biosynthesis | 43 | ko00563 |
| 165 | Proximal tubule bicarbonate reclamation | 42 | ko04964 |
| 166 | Porphyrin and chlorophyll metabolism | 41 | ko00860 |
| 167 | Glycosaminoglycan degradation | 41 | ko00531 |
| 168 | Pentose and glucuronate interconversions | 41 | ko00040 |
| 169 | Glycosaminoglycan biosynthesis - heparan sulfate | 41 | ko00534 |
| 170 | O-Glycan biosynthesis | 40 | ko00512 |
| 171 | One carbon pool by folate | 40 | ko00670 |
| 172 | Taste transduction | 40 | ko04742 |
| 173 | Protein export | 39 | ko03060 |
| 174 | Steroid hormone biosynthesis | 39 | ko00140 |
| 175 | Ascorbate and aldarate metabolism | 39 | ko00053 |
| 176 | Valine, leucine and isoleucine biosynthesis | 39 | ko00290 |
| 177 | Regulation of autophagy | 38 | ko04140 |
| 178 | Selenoamino acid metabolism | 35 | ko00450 |
| 179 | Histidine metabolism | 35 | ko00340 |
| 180 | Pantothenate and CoA biosynthesis | 34 | ko00770 |
| 181 | Nitrogen metabolism | 34 | ko00910 |
| 182 | Non-homologous end-joining | 34 | ko03450 |
| 183 | Maturity onset diabetes of the young | 33 | ko04950 |
| 184 | Autoimmune thyroid disease | 30 | ko05320 |
| 185 | Terpenoid backbone biosynthesis | 30 | ko00900 |
| 186 | Glyoxylate and dicarboxylate metabolism | 28 | ko00630 |
| 187 | Phenylalanine metabolism | 28 | ko00360 |
| 188 | Type I diabetes mellitus | 25 | ko04940 |
| 189 | Fatty acid biosynthesis | 24 | ko00061 |
| 190 | Steroid biosynthesis | 23 | ko00100 |
| 191 | Fatty acid elongation in mitochondria | 23 | ko00062 |
| 192 | Glycosphingolipid biosynthesis - globo series | 22 | ko00603 |
| 193 | Primary immunodeficiency | 20 | ko05340 |
| 194 | Sulfur metabolism | 20 | ko00920 |
| 195 | Folate biosynthesis | 20 | ko00790 |
| 196 | Ubiquinone and other terpenoid-quinone biosynthesis | 19 | ko00130 |
| 197 | Glycosaminoglycan biosynthesis - keratan sulfate | 18 | ko00533 |
| 198 | Glycosphingolipid biosynthesis - lacto and neolacto series | 18 | ko00601 |
| 199 | Glycosphingolipid biosynthesis - ganglio series | 17 | ko00604 |
| 200 | Thiamine metabolism | 17 | ko00730 |
| 201 | O-Mannosyl glycan biosynthesis | 15 | ko00514 |
| 202 | Primary bile acid biosynthesis | 14 | ko00120 |
| 203 | Synthesis and degradation of ketone bodies | 14 | ko00072 |
| 204 | Cyanoamino acid metabolism | 13 | ko00460 |
| 205 | Caffeine metabolism | 12 | ko00232 |
| 206 | Allograft rejection | 12 | ko05330 |
| 207 | Graft-versus-host disease | 12 | ko05332 |
| 208 | Taurine and hypotaurine metabolism | 11 | ko00430 |
| 209 | Lysine biosynthesis | 9 | ko00300 |
| 210 | Insect hormone biosynthesis | 7 | ko00981 |
| 211 | Phenylalanine, tyrosine and tryptophan biosynthesis | 7 | ko00400 |
| 212 | Butirosin and neomycin biosynthesis | 7 | ko00524 |
| 213 | Lipoic acid metabolism | 6 | ko00785 |
| 214 | Asthma | 6 | ko05310 |
| 215 | D-Glutamine and D-glutamate metabolism | 5 | ko00471 |
| 216 | Vitamin B6 metabolism | 5 | ko00750 |
| 217 | Biotin metabolism | 3 | ko00780 |
| 218 | D-Arginine and D-ornithine metabolism | 2 | ko00472 |
| 219 | Intestinal immune network for IgA production | 2 | ko04672 |
| 220 | Polyketide sugar unit biosynthesis | 1 | ko00523 |

 

|  |  |  |
| --- | --- | --- |
| **#** | **Pathway** | **Differentially expressed genes** |
| 1 | Metabolic pathways (no map in kegg database) | Unigene10002\_JingChaoZuZhi2, Unigene10005\_JingChaoZuZhi2, Unigene10011\_JingChaoZuZhi2, Unigene10032\_JingChaoZuZhi2, Unigene10082\_JingChaoZuZhi2, Unigene10122\_JingChaoZuZhi2, Unigene10129\_JingChaoZuZhi2, Unigene10133\_JingChaoZuZhi2, Unigene10268\_JingChaoZuZhi2, Unigene10302\_JingChaoZuZhi2, Unigene10315\_JingChaoZuZhi2, Unigene10340\_JingChaoZuZhi2, Unigene1037\_JingChaoZuZhi2, Unigene10384\_JingChaoZuZhi2, Unigene10394\_JingChaoZuZhi2, Unigene103\_JingChaoZuZhi2, Unigene10451\_JingChaoZuZhi2, Unigene1045\_JingChaoZuZhi2, Unigene10514\_JingChaoZuZhi2, Unigene10542\_JingChaoZuZhi2, Unigene10578\_JingChaoZuZhi2, Unigene1071\_JingChaoZuZhi2, Unigene10763\_JingChaoZuZhi2, Unigene10769\_JingChaoZuZhi2, Unigene10782\_JingChaoZuZhi2, Unigene10802\_JingChaoZuZhi2, Unigene10806\_JingChaoZuZhi2, Unigene10827\_JingChaoZuZhi2, Unigene10829\_JingChaoZuZhi2, Unigene1082\_JingChaoZuZhi2, Unigene10860\_JingChaoZuZhi2, Unigene10883\_JingChaoZuZhi2, Unigene10888\_JingChaoZuZhi2, Unigene10895\_JingChaoZuZhi2, Unigene10939\_JingChaoZuZhi2, Unigene10976\_JingChaoZuZhi2, Unigene10979\_JingChaoZuZhi2, Unigene11055\_JingChaoZuZhi2, Unigene11074\_JingChaoZuZhi2, Unigene11119\_JingChaoZuZhi2, Unigene11134\_JingChaoZuZhi2, Unigene11150\_JingChaoZuZhi2, Unigene11206\_JingChaoZuZhi2, Unigene11260\_JingChaoZuZhi2, Unigene11303\_JingChaoZuZhi2, Unigene11312\_JingChaoZuZhi2, Unigene11372\_JingChaoZuZhi2, Unigene11433\_JingChaoZuZhi2, Unigene11462\_JingChaoZuZhi2, Unigene11466\_JingChaoZuZhi2, Unigene11491\_JingChaoZuZhi2, Unigene11496\_JingChaoZuZhi2, Unigene11536\_JingChaoZuZhi2, Unigene1154\_JingChaoZuZhi2, Unigene11564\_JingChaoZuZhi2, Unigene1164\_JingChaoZuZhi2, Unigene11681\_JingChaoZuZhi2, Unigene11700\_JingChaoZuZhi2, Unigene11719\_JingChaoZuZhi2, Unigene11721\_JingChaoZuZhi2, Unigene11730\_JingChaoZuZhi2, Unigene11803\_JingChaoZuZhi2, Unigene11808\_JingChaoZuZhi2, Unigene11872\_JingChaoZuZhi2, Unigene11892\_JingChaoZuZhi2, Unigene11963\_JingChaoZuZhi2, Unigene11995\_JingChaoZuZhi2, Unigene12083\_JingChaoZuZhi2, Unigene12101\_JingChaoZuZhi2, Unigene12129\_JingChaoZuZhi2, Unigene12217\_JingChaoZuZhi2, Unigene12260\_JingChaoZuZhi2, Unigene12263\_JingChaoZuZhi2, Unigene12286\_JingChaoZuZhi2, Unigene12292\_JingChaoZuZhi2, Unigene1229\_JingChaoZuZhi2, Unigene12354\_JingChaoZuZhi2, Unigene12356\_JingChaoZuZhi2, Unigene12380\_JingChaoZuZhi2, Unigene12387\_JingChaoZuZhi2, Unigene12404\_JingChaoZuZhi2, Unigene12419\_JingChaoZuZhi2, Unigene12454\_JingChaoZuZhi2, Unigene12516\_JingChaoZuZhi2, Unigene1252\_JingChaoZuZhi2, Unigene12560\_JingChaoZuZhi2, Unigene12617\_JingChaoZuZhi2, Unigene12639\_JingChaoZuZhi2, Unigene12675\_JingChaoZuZhi2, Unigene12700\_JingChaoZuZhi2, Unigene12736\_JingChaoZuZhi2, Unigene12745\_JingChaoZuZhi2, Unigene12758\_JingChaoZuZhi2, Unigene12768\_JingChaoZuZhi2, Unigene12885\_JingChaoZuZhi2, Unigene12966\_JingChaoZuZhi2, Unigene12979\_JingChaoZuZhi2, Unigene13003\_JingChaoZuZhi2, Unigene13056\_JingChaoZuZhi2, Unigene13068\_JingChaoZuZhi2, Unigene13096\_JingChaoZuZhi2, Unigene13103\_JingChaoZuZhi2, Unigene1313\_JingChaoZuZhi2, Unigene13144\_JingChaoZuZhi2, Unigene13149\_JingChaoZuZhi2, Unigene13160\_JingChaoZuZhi2, Unigene13176\_JingChaoZuZhi2, Unigene13191\_JingChaoZuZhi2, Unigene13227\_JingChaoZuZhi2, Unigene1323\_JingChaoZuZhi2, Unigene13280\_JingChaoZuZhi2, Unigene13289\_JingChaoZuZhi2, Unigene13298\_JingChaoZuZhi2, Unigene13307\_JingChaoZuZhi2, Unigene13337\_JingChaoZuZhi2, Unigene13388\_JingChaoZuZhi2, Unigene13419\_JingChaoZuZhi2, Unigene13420\_JingChaoZuZhi2, Unigene13429\_JingChaoZuZhi2, Unigene13435\_JingChaoZuZhi2, Unigene13452\_JingChaoZuZhi2, Unigene13455\_JingChaoZuZhi2, Unigene13538\_JingChaoZuZhi2, Unigene13565\_JingChaoZuZhi2, Unigene13587\_JingChaoZuZhi2, Unigene13648\_JingChaoZuZhi2, Unigene13693\_JingChaoZuZhi2, Unigene13702\_JingChaoZuZhi2, Unigene13740\_JingChaoZuZhi2, Unigene13795\_JingChaoZuZhi2, Unigene13803\_JingChaoZuZhi2, Unigene13837\_JingChaoZuZhi2, Unigene13869\_JingChaoZuZhi2, Unigene13878\_JingChaoZuZhi2, Unigene13936\_JingChaoZuZhi2, Unigene1393\_JingChaoZuZhi2, Unigene13946\_JingChaoZuZhi2, Unigene13966\_JingChaoZuZhi2, Unigene13972\_JingChaoZuZhi2, Unigene13988\_JingChaoZuZhi2, Unigene14041\_JingChaoZuZhi2, Unigene14068\_JingChaoZuZhi2, Unigene14104\_JingChaoZuZhi2, Unigene14132\_JingChaoZuZhi2, Unigene14135\_JingChaoZuZhi2, Unigene14158\_JingChaoZuZhi2, Unigene14203\_JingChaoZuZhi2, Unigene14212\_JingChaoZuZhi2, Unigene14339\_JingChaoZuZhi2, Unigene14357\_JingChaoZuZhi2, Unigene14360\_JingChaoZuZhi2, Unigene14388\_JingChaoZuZhi2, Unigene14414\_JingChaoZuZhi2, Unigene14419\_JingChaoZuZhi2, Unigene14424\_JingChaoZuZhi2, Unigene14446\_JingChaoZuZhi2, Unigene14448\_JingChaoZuZhi2, Unigene144\_JingChaoZuZhi2, Unigene14533\_JingChaoZuZhi2, Unigene14551\_JingChaoZuZhi2, Unigene14603\_JingChaoZuZhi2, Unigene14630\_JingChaoZuZhi2, Unigene14631\_JingChaoZuZhi2, Unigene1468\_JingChaoZuZhi2, Unigene14716\_JingChaoZuZhi2, Unigene1478\_JingChaoZuZhi2, Unigene14816\_JingChaoZuZhi2, Unigene14871\_JingChaoZuZhi2, Unigene14878\_JingChaoZuZhi2, Unigene14913\_JingChaoZuZhi2, Unigene14977\_JingChaoZuZhi2, Unigene15002\_JingChaoZuZhi2, Unigene15115\_JingChaoZuZhi2, Unigene15172\_JingChaoZuZhi2, Unigene15198\_JingChaoZuZhi2, Unigene15201\_JingChaoZuZhi2, Unigene15206\_JingChaoZuZhi2, Unigene15216\_JingChaoZuZhi2, Unigene15256\_JingChaoZuZhi2, Unigene15288\_JingChaoZuZhi2, Unigene15290\_JingChaoZuZhi2, Unigene15358\_JingChaoZuZhi2, Unigene15363\_JingChaoZuZhi2, Unigene15418\_JingChaoZuZhi2, Unigene15425\_JingChaoZuZhi2, Unigene15503\_JingChaoZuZhi2, Unigene15537\_JingChaoZuZhi2, Unigene15539\_JingChaoZuZhi2, Unigene15542\_JingChaoZuZhi2, Unigene15591\_JingChaoZuZhi2, Unigene1561\_JingChaoZuZhi2, Unigene15659\_JingChaoZuZhi2, Unigene15728\_JingChaoZuZhi2, Unigene15755\_JingChaoZuZhi2, Unigene1576\_JingChaoZuZhi2, Unigene15770\_JingChaoZuZhi2, Unigene15780\_JingChaoZuZhi2, Unigene15802\_JingChaoZuZhi2, Unigene15856\_JingChaoZuZhi2, Unigene15867\_JingChaoZuZhi2, Unigene15892\_JingChaoZuZhi2, Unigene15927\_JingChaoZuZhi2, Unigene15929\_JingChaoZuZhi2, Unigene15942\_JingChaoZuZhi2, Unigene15992\_JingChaoZuZhi2, Unigene16010\_JingChaoZuZhi2, Unigene16073\_JingChaoZuZhi2, Unigene16107\_JingChaoZuZhi2, Unigene16149\_JingChaoZuZhi2, Unigene16176\_JingChaoZuZhi2, Unigene16232\_JingChaoZuZhi2, Unigene16281\_JingChaoZuZhi2, Unigene1628\_JingChaoZuZhi2, Unigene16293\_JingChaoZuZhi2, Unigene16295\_JingChaoZuZhi2, Unigene16317\_JingChaoZuZhi2, Unigene16352\_JingChaoZuZhi2, Unigene16415\_JingChaoZuZhi2, Unigene16460\_JingChaoZuZhi2, Unigene16501\_JingChaoZuZhi2, Unigene16542\_JingChaoZuZhi2, Unigene16549\_JingChaoZuZhi2, Unigene16594\_JingChaoZuZhi2, Unigene16646\_JingChaoZuZhi2, Unigene16663\_JingChaoZuZhi2, Unigene16681\_JingChaoZuZhi2, Unigene16711\_JingChaoZuZhi2, Unigene16738\_JingChaoZuZhi2, Unigene16762\_JingChaoZuZhi2, Unigene16786\_JingChaoZuZhi2, Unigene16788\_JingChaoZuZhi2, Unigene16795\_JingChaoZuZhi2, Unigene16845\_JingChaoZuZhi2, Unigene16863\_JingChaoZuZhi2, Unigene16868\_JingChaoZuZhi2, Unigene16892\_JingChaoZuZhi2, Unigene16899\_JingChaoZuZhi2, Unigene16938\_JingChaoZuZhi2, Unigene16945\_JingChaoZuZhi2, Unigene16991\_JingChaoZuZhi2, Unigene16996\_JingChaoZuZhi2, Unigene17015\_JingChaoZuZhi2, Unigene17028\_JingChaoZuZhi2, Unigene17035\_JingChaoZuZhi2, Unigene17049\_JingChaoZuZhi2, Unigene17065\_JingChaoZuZhi2, Unigene17078\_JingChaoZuZhi2, Unigene17082\_JingChaoZuZhi2, Unigene17084\_JingChaoZuZhi2, Unigene17099\_JingChaoZuZhi2, Unigene17138\_JingChaoZuZhi2, Unigene17151\_JingChaoZuZhi2, Unigene17162\_JingChaoZuZhi2, Unigene17217\_JingChaoZuZhi2, Unigene17221\_JingChaoZuZhi2, Unigene17235\_JingChaoZuZhi2, Unigene17278\_JingChaoZuZhi2, Unigene17330\_JingChaoZuZhi2, Unigene1738\_JingChaoZuZhi2, Unigene17395\_JingChaoZuZhi2, Unigene173\_JingChaoZuZhi2, Unigene17401\_JingChaoZuZhi2, Unigene1744\_JingChaoZuZhi2, Unigene17452\_JingChaoZuZhi2, Unigene17467\_JingChaoZuZhi2, Unigene17636\_JingChaoZuZhi2, Unigene17655\_JingChaoZuZhi2, Unigene17667\_JingChaoZuZhi2, Unigene17691\_JingChaoZuZhi2, Unigene17709\_JingChaoZuZhi2, Unigene17861\_JingChaoZuZhi2, Unigene17889\_JingChaoZuZhi2, Unigene17972\_JingChaoZuZhi2, Unigene17995\_JingChaoZuZhi2, Unigene17997\_JingChaoZuZhi2, Unigene18000\_JingChaoZuZhi2, Unigene18017\_JingChaoZuZhi2, Unigene18029\_JingChaoZuZhi2, Unigene18099\_JingChaoZuZhi2, Unigene18114\_JingChaoZuZhi2, Unigene18117\_JingChaoZuZhi2, Unigene18123\_JingChaoZuZhi2, Unigene18132\_JingChaoZuZhi2, Unigene18163\_JingChaoZuZhi2, Unigene18176\_JingChaoZuZhi2, Unigene18179\_JingChaoZuZhi2, Unigene18187\_JingChaoZuZhi2, Unigene18188\_JingChaoZuZhi2, Unigene18214\_JingChaoZuZhi2, Unigene18229\_JingChaoZuZhi2, Unigene18268\_JingChaoZuZhi2, Unigene18308\_JingChaoZuZhi2, Unigene18313\_JingChaoZuZhi2, Unigene18326\_JingChaoZuZhi2, Unigene18335\_JingChaoZuZhi2, Unigene18348\_JingChaoZuZhi2, Unigene18351\_JingChaoZuZhi2, Unigene18358\_JingChaoZuZhi2, Unigene18409\_JingChaoZuZhi2, Unigene18461\_JingChaoZuZhi2, Unigene18464\_JingChaoZuZhi2, Unigene18475\_JingChaoZuZhi2, Unigene18508\_JingChaoZuZhi2, Unigene18510\_JingChaoZuZhi2, Unigene18525\_JingChaoZuZhi2, Unigene18537\_JingChaoZuZhi2, Unigene18553\_JingChaoZuZhi2, Unigene18569\_JingChaoZuZhi2, Unigene18613\_JingChaoZuZhi2, Unigene18665\_JingChaoZuZhi2, Unigene18666\_JingChaoZuZhi2, Unigene18721\_JingChaoZuZhi2, Unigene18776\_JingChaoZuZhi2, Unigene18788\_JingChaoZuZhi2, Unigene1878\_JingChaoZuZhi2, Unigene18846\_JingChaoZuZhi2, Unigene18868\_JingChaoZuZhi2, Unigene18874\_JingChaoZuZhi2, Unigene18921\_JingChaoZuZhi2, Unigene18929\_JingChaoZuZhi2, Unigene18935\_JingChaoZuZhi2, Unigene18991\_JingChaoZuZhi2, Unigene19006\_JingChaoZuZhi2, Unigene19015\_JingChaoZuZhi2, Unigene19050\_JingChaoZuZhi2, Unigene19051\_JingChaoZuZhi2, Unigene19126\_JingChaoZuZhi2, Unigene19140\_JingChaoZuZhi2, Unigene19181\_JingChaoZuZhi2, Unigene19186\_JingChaoZuZhi2, Unigene19226\_JingChaoZuZhi2, Unigene1922\_JingChaoZuZhi2, Unigene19246\_JingChaoZuZhi2, Unigene19251\_JingChaoZuZhi2, Unigene19256\_JingChaoZuZhi2, Unigene19280\_JingChaoZuZhi2, Unigene19303\_JingChaoZuZhi2, Unigene19355\_JingChaoZuZhi2, Unigene19398\_JingChaoZuZhi2, Unigene19410\_JingChaoZuZhi2, Unigene19428\_JingChaoZuZhi2, Unigene19436\_JingChaoZuZhi2, Unigene19458\_JingChaoZuZhi2, Unigene19475\_JingChaoZuZhi2, Unigene19476\_JingChaoZuZhi2, Unigene19497\_JingChaoZuZhi2, Unigene19510\_JingChaoZuZhi2, Unigene19530\_JingChaoZuZhi2, Unigene19539\_JingChaoZuZhi2, Unigene19555\_JingChaoZuZhi2, Unigene19565\_JingChaoZuZhi2, Unigene19582\_JingChaoZuZhi2, Unigene19619\_JingChaoZuZhi2, Unigene19634\_JingChaoZuZhi2, Unigene19643\_JingChaoZuZhi2, Unigene19644\_JingChaoZuZhi2, Unigene19645\_JingChaoZuZhi2, Unigene19647\_JingChaoZuZhi2, Unigene19667\_JingChaoZuZhi2, Unigene196\_JingChaoZuZhi2, Unigene19728\_JingChaoZuZhi2, Unigene19750\_JingChaoZuZhi2, Unigene19751\_JingChaoZuZhi2, Unigene19778\_JingChaoZuZhi2, Unigene19811\_JingChaoZuZhi2, Unigene19838\_JingChaoZuZhi2, Unigene19853\_JingChaoZuZhi2, Unigene19858\_JingChaoZuZhi2, Unigene19862\_JingChaoZuZhi2, Unigene19893\_JingChaoZuZhi2, Unigene19904\_JingChaoZuZhi2, Unigene19907\_JingChaoZuZhi2, Unigene19911\_JingChaoZuZhi2, Unigene19928\_JingChaoZuZhi2, Unigene19938\_JingChaoZuZhi2, Unigene19950\_JingChaoZuZhi2, Unigene19978\_JingChaoZuZhi2, Unigene19979\_JingChaoZuZhi2, Unigene20010\_JingChaoZuZhi2, Unigene20049\_JingChaoZuZhi2, Unigene20050\_JingChaoZuZhi2, Unigene20066\_JingChaoZuZhi2, Unigene20071\_JingChaoZuZhi2, Unigene20087\_JingChaoZuZhi2, Unigene20099\_JingChaoZuZhi2, Unigene20114\_JingChaoZuZhi2, Unigene20129\_JingChaoZuZhi2, Unigene2012\_JingChaoZuZhi2, Unigene20143\_JingChaoZuZhi2, Unigene20150\_JingChaoZuZhi2, Unigene20163\_JingChaoZuZhi2, Unigene20172\_JingChaoZuZhi2, Unigene20209\_JingChaoZuZhi2, Unigene20219\_JingChaoZuZhi2, Unigene20264\_JingChaoZuZhi2, Unigene20270\_JingChaoZuZhi2, Unigene20314\_JingChaoZuZhi2, Unigene20357\_JingChaoZuZhi2, Unigene20393\_JingChaoZuZhi2, Unigene20404\_JingChaoZuZhi2, Unigene20411\_JingChaoZuZhi2, Unigene20422\_JingChaoZuZhi2, Unigene20441\_JingChaoZuZhi2, Unigene20452\_JingChaoZuZhi2, Unigene20453\_JingChaoZuZhi2, Unigene2046\_JingChaoZuZhi2, Unigene20504\_JingChaoZuZhi2, Unigene20531\_JingChaoZuZhi2, Unigene20559\_JingChaoZuZhi2, Unigene2058\_JingChaoZuZhi2, Unigene20597\_JingChaoZuZhi2, Unigene20612\_JingChaoZuZhi2, Unigene20631\_JingChaoZuZhi2, Unigene20658\_JingChaoZuZhi2, Unigene20676\_JingChaoZuZhi2, Unigene20684\_JingChaoZuZhi2, Unigene20711\_JingChaoZuZhi2, Unigene20742\_JingChaoZuZhi2, Unigene20760\_JingChaoZuZhi2, Unigene20786\_JingChaoZuZhi2, Unigene20802\_JingChaoZuZhi2, Unigene20814\_JingChaoZuZhi2, Unigene20857\_JingChaoZuZhi2, Unigene20862\_JingChaoZuZhi2, Unigene20896\_JingChaoZuZhi2, Unigene20936\_JingChaoZuZhi2, Unigene20950\_JingChaoZuZhi2, Unigene20959\_JingChaoZuZhi2, Unigene20962\_JingChaoZuZhi2, Unigene20979\_JingChaoZuZhi2, Unigene20986\_JingChaoZuZhi2, Unigene21020\_JingChaoZuZhi2, Unigene21024\_JingChaoZuZhi2, Unigene21025\_JingChaoZuZhi2, Unigene21049\_JingChaoZuZhi2, Unigene21054\_JingChaoZuZhi2, Unigene21065\_JingChaoZuZhi2, Unigene21092\_JingChaoZuZhi2, Unigene21094\_JingChaoZuZhi2, Unigene21099\_JingChaoZuZhi2, Unigene21122\_JingChaoZuZhi2, Unigene21158\_JingChaoZuZhi2, Unigene21176\_JingChaoZuZhi2, Unigene21230\_JingChaoZuZhi2, Unigene21270\_JingChaoZuZhi2, Unigene21281\_JingChaoZuZhi2, Unigene21282\_JingChaoZuZhi2, Unigene21295\_JingChaoZuZhi2, Unigene21318\_JingChaoZuZhi2, Unigene2132\_JingChaoZuZhi2, Unigene21363\_JingChaoZuZhi2, Unigene21392\_JingChaoZuZhi2, Unigene21407\_JingChaoZuZhi2, Unigene21428\_JingChaoZuZhi2, Unigene21490\_JingChaoZuZhi2, Unigene21503\_JingChaoZuZhi2, Unigene21507\_JingChaoZuZhi2, Unigene21510\_JingChaoZuZhi2, Unigene21541\_JingChaoZuZhi2, Unigene21557\_JingChaoZuZhi2, Unigene2155\_JingChaoZuZhi2, Unigene21628\_JingChaoZuZhi2, Unigene2162\_JingChaoZuZhi2, Unigene21648\_JingChaoZuZhi2, Unigene21678\_JingChaoZuZhi2, Unigene21718\_JingChaoZuZhi2, Unigene21754\_JingChaoZuZhi2, Unigene21756\_JingChaoZuZhi2, Unigene21759\_JingChaoZuZhi2, Unigene21767\_JingChaoZuZhi2, Unigene21805\_JingChaoZuZhi2, Unigene21806\_JingChaoZuZhi2, Unigene21820\_JingChaoZuZhi2, Unigene21821\_JingChaoZuZhi2, Unigene21828\_JingChaoZuZhi2, Unigene21859\_JingChaoZuZhi2, Unigene21877\_JingChaoZuZhi2, Unigene2190\_JingChaoZuZhi2, Unigene21917\_JingChaoZuZhi2, Unigene21949\_JingChaoZuZhi2, Unigene21977\_JingChaoZuZhi2, Unigene21986\_JingChaoZuZhi2, Unigene21993\_JingChaoZuZhi2, Unigene22003\_JingChaoZuZhi2, Unigene22065\_JingChaoZuZhi2, Unigene22072\_JingChaoZuZhi2, Unigene22075\_JingChaoZuZhi2, Unigene22086\_JingChaoZuZhi2, Unigene22123\_JingChaoZuZhi2, Unigene22143\_JingChaoZuZhi2, Unigene22156\_JingChaoZuZhi2, Unigene22197\_JingChaoZuZhi2, Unigene22198\_JingChaoZuZhi2, Unigene22211\_JingChaoZuZhi2, Unigene22257\_JingChaoZuZhi2, Unigene22297\_JingChaoZuZhi2, Unigene22309\_JingChaoZuZhi2, Unigene2230\_JingChaoZuZhi2, Unigene22310\_JingChaoZuZhi2, Unigene22325\_JingChaoZuZhi2, Unigene22334\_JingChaoZuZhi2, Unigene22339\_JingChaoZuZhi2, Unigene22352\_JingChaoZuZhi2, Unigene22355\_JingChaoZuZhi2, Unigene2235\_JingChaoZuZhi2, Unigene22360\_JingChaoZuZhi2, Unigene22376\_JingChaoZuZhi2, Unigene22387\_JingChaoZuZhi2, Unigene22392\_JingChaoZuZhi2, Unigene22403\_JingChaoZuZhi2, Unigene22416\_JingChaoZuZhi2, Unigene22451\_JingChaoZuZhi2, Unigene22462\_JingChaoZuZhi2, Unigene22474\_JingChaoZuZhi2, Unigene22492\_JingChaoZuZhi2, Unigene22498\_JingChaoZuZhi2, Unigene22516\_JingChaoZuZhi2, Unigene22545\_JingChaoZuZhi2, Unigene22546\_JingChaoZuZhi2, Unigene2254\_JingChaoZuZhi2, Unigene22579\_JingChaoZuZhi2, Unigene22582\_JingChaoZuZhi2, Unigene22591\_JingChaoZuZhi2, Unigene22601\_JingChaoZuZhi2, Unigene22680\_JingChaoZuZhi2, Unigene22684\_JingChaoZuZhi2, Unigene22727\_JingChaoZuZhi2, Unigene22747\_JingChaoZuZhi2, Unigene2275\_JingChaoZuZhi2, Unigene22780\_JingChaoZuZhi2, Unigene22793\_JingChaoZuZhi2, Unigene22824\_JingChaoZuZhi2, Unigene22830\_JingChaoZuZhi2, Unigene22833\_JingChaoZuZhi2, Unigene22838\_JingChaoZuZhi2, Unigene22842\_JingChaoZuZhi2, Unigene22870\_JingChaoZuZhi2, Unigene22904\_JingChaoZuZhi2, Unigene22908\_JingChaoZuZhi2, Unigene22914\_JingChaoZuZhi2, Unigene22923\_JingChaoZuZhi2, Unigene2306\_JingChaoZuZhi2, Unigene23105\_JingChaoZuZhi2, Unigene23172\_JingChaoZuZhi2, Unigene23178\_JingChaoZuZhi2, Unigene23389\_JingChaoZuZhi2, Unigene2342\_JingChaoZuZhi2, Unigene23447\_JingChaoZuZhi2, Unigene23475\_JingChaoZuZhi2, Unigene23504\_JingChaoZuZhi2, Unigene2354\_JingChaoZuZhi2, Unigene23634\_JingChaoZuZhi2, Unigene23700\_JingChaoZuZhi2, Unigene23842\_JingChaoZuZhi2, Unigene23882\_JingChaoZuZhi2, Unigene240\_JingChaoZuZhi2, Unigene24146\_JingChaoZuZhi2, Unigene24204\_JingChaoZuZhi2, Unigene2424\_JingChaoZuZhi2, Unigene24261\_JingChaoZuZhi2, Unigene24264\_JingChaoZuZhi2, Unigene2431\_JingChaoZuZhi2, Unigene24676\_JingChaoZuZhi2, Unigene2474\_JingChaoZuZhi2, Unigene2480\_JingChaoZuZhi2, Unigene2487\_JingChaoZuZhi2, Unigene248\_JingChaoZuZhi2, Unigene24985\_JingChaoZuZhi2, Unigene2506\_JingChaoZuZhi2, Unigene25108\_JingChaoZuZhi2, Unigene25123\_JingChaoZuZhi2, Unigene2514\_JingChaoZuZhi2, Unigene25228\_JingChaoZuZhi2, Unigene2530\_JingChaoZuZhi2, Unigene25319\_JingChaoZuZhi2, Unigene2535\_JingChaoZuZhi2, Unigene25361\_JingChaoZuZhi2, Unigene2553\_JingChaoZuZhi2, Unigene25672\_JingChaoZuZhi2, Unigene25744\_JingChaoZuZhi2, Unigene25992\_JingChaoZuZhi2, Unigene26084\_JingChaoZuZhi2, Unigene26104\_JingChaoZuZhi2, Unigene26170\_JingChaoZuZhi2, Unigene26274\_JingChaoZuZhi2, Unigene26507\_JingChaoZuZhi2, Unigene26787\_JingChaoZuZhi2, Unigene26869\_JingChaoZuZhi2, Unigene26903\_JingChaoZuZhi2, Unigene26954\_JingChaoZuZhi2, Unigene27011\_JingChaoZuZhi2, Unigene2705\_JingChaoZuZhi2, Unigene2707\_JingChaoZuZhi2, Unigene2711\_JingChaoZuZhi2, Unigene27219\_JingChaoZuZhi2, Unigene27220\_JingChaoZuZhi2, Unigene27301\_JingChaoZuZhi2, Unigene2730\_JingChaoZuZhi2, Unigene27353\_JingChaoZuZhi2, Unigene2761\_JingChaoZuZhi2, Unigene28123\_JingChaoZuZhi2, Unigene28252\_JingChaoZuZhi2, Unigene28256\_JingChaoZuZhi2, Unigene28844\_JingChaoZuZhi2, Unigene28889\_JingChaoZuZhi2, Unigene289\_JingChaoZuZhi2, Unigene2909\_JingChaoZuZhi2, Unigene29151\_JingChaoZuZhi2, Unigene2958\_JingChaoZuZhi2, Unigene29994\_JingChaoZuZhi2, Unigene29995\_JingChaoZuZhi2, Unigene30051\_JingChaoZuZhi2, Unigene30402\_JingChaoZuZhi2, Unigene3061\_JingChaoZuZhi2, Unigene30697\_JingChaoZuZhi2, Unigene30780\_JingChaoZuZhi2, Unigene30782\_JingChaoZuZhi2, Unigene30799\_JingChaoZuZhi2, Unigene30849\_JingChaoZuZhi2, Unigene3098\_JingChaoZuZhi2, Unigene31032\_JingChaoZuZhi2, Unigene31036\_JingChaoZuZhi2, Unigene31040\_JingChaoZuZhi2, Unigene31068\_JingChaoZuZhi2, Unigene31166\_JingChaoZuZhi2, Unigene3116\_JingChaoZuZhi2, Unigene31282\_JingChaoZuZhi2, Unigene3128\_JingChaoZuZhi2, Unigene31327\_JingChaoZuZhi2, Unigene31336\_JingChaoZuZhi2, Unigene31355\_JingChaoZuZhi2, Unigene31402\_JingChaoZuZhi2, Unigene3162\_JingChaoZuZhi2, Unigene3187\_JingChaoZuZhi2, Unigene32031\_JingChaoZuZhi2, Unigene322\_JingChaoZuZhi2, Unigene32391\_JingChaoZuZhi2, Unigene32472\_JingChaoZuZhi2, Unigene32589\_JingChaoZuZhi2, Unigene3273\_JingChaoZuZhi2, Unigene32746\_JingChaoZuZhi2, Unigene32752\_JingChaoZuZhi2, Unigene32848\_JingChaoZuZhi2, Unigene3292\_JingChaoZuZhi2, Unigene32951\_JingChaoZuZhi2, Unigene32999\_JingChaoZuZhi2, Unigene33026\_JingChaoZuZhi2, Unigene33161\_JingChaoZuZhi2, Unigene33238\_JingChaoZuZhi2, Unigene33308\_JingChaoZuZhi2, Unigene3336\_JingChaoZuZhi2, Unigene33470\_JingChaoZuZhi2, Unigene33523\_JingChaoZuZhi2, Unigene3366\_JingChaoZuZhi2, Unigene33822\_JingChaoZuZhi2, Unigene3384\_JingChaoZuZhi2, Unigene33875\_JingChaoZuZhi2, Unigene34150\_JingChaoZuZhi2, Unigene34300\_JingChaoZuZhi2, Unigene34338\_JingChaoZuZhi2, Unigene34397\_JingChaoZuZhi2, Unigene3457\_JingChaoZuZhi2, Unigene35136\_JingChaoZuZhi2, Unigene35187\_JingChaoZuZhi2, Unigene35298\_JingChaoZuZhi2, Unigene35366\_JingChaoZuZhi2, Unigene35450\_JingChaoZuZhi2, Unigene35544\_JingChaoZuZhi2, Unigene35605\_JingChaoZuZhi2, Unigene35687\_JingChaoZuZhi2, Unigene35740\_JingChaoZuZhi2, Unigene3580\_JingChaoZuZhi2, Unigene35848\_JingChaoZuZhi2, Unigene3592\_JingChaoZuZhi2, Unigene36078\_JingChaoZuZhi2, Unigene36089\_JingChaoZuZhi2, Unigene36183\_JingChaoZuZhi2, Unigene36230\_JingChaoZuZhi2, Unigene3632\_JingChaoZuZhi2, Unigene36386\_JingChaoZuZhi2, Unigene3643\_JingChaoZuZhi2, Unigene36522\_JingChaoZuZhi2, Unigene36714\_JingChaoZuZhi2, Unigene36790\_JingChaoZuZhi2, Unigene36819\_JingChaoZuZhi2, Unigene3682\_JingChaoZuZhi2, Unigene36973\_JingChaoZuZhi2, Unigene36996\_JingChaoZuZhi2, Unigene3700\_JingChaoZuZhi2, Unigene3714\_JingChaoZuZhi2, Unigene37224\_JingChaoZuZhi2, Unigene37317\_JingChaoZuZhi2, Unigene37457\_JingChaoZuZhi2, Unigene37471\_JingChaoZuZhi2, Unigene37504\_JingChaoZuZhi2, Unigene37516\_JingChaoZuZhi2, Unigene37548\_JingChaoZuZhi2, Unigene37589\_JingChaoZuZhi2, Unigene37689\_JingChaoZuZhi2, Unigene3769\_JingChaoZuZhi2, Unigene37825\_JingChaoZuZhi2, Unigene37828\_JingChaoZuZhi2, Unigene38105\_JingChaoZuZhi2, Unigene38162\_JingChaoZuZhi2, Unigene38285\_JingChaoZuZhi2, Unigene38309\_JingChaoZuZhi2, Unigene38324\_JingChaoZuZhi2, Unigene38448\_JingChaoZuZhi2, Unigene3850\_JingChaoZuZhi2, Unigene38514\_JingChaoZuZhi2, Unigene3852\_JingChaoZuZhi2, Unigene385\_JingChaoZuZhi2, Unigene38622\_JingChaoZuZhi2, Unigene3871\_JingChaoZuZhi2, Unigene38753\_JingChaoZuZhi2, Unigene38871\_JingChaoZuZhi2, Unigene38886\_JingChaoZuZhi2, Unigene38924\_JingChaoZuZhi2, Unigene38940\_JingChaoZuZhi2, Unigene3910\_JingChaoZuZhi2, Unigene39201\_JingChaoZuZhi2, Unigene3936\_JingChaoZuZhi2, Unigene3938\_JingChaoZuZhi2, Unigene39505\_JingChaoZuZhi2, Unigene39577\_JingChaoZuZhi2, Unigene39624\_JingChaoZuZhi2, Unigene39661\_JingChaoZuZhi2, Unigene3974\_JingChaoZuZhi2, Unigene39829\_JingChaoZuZhi2, Unigene4050\_JingChaoZuZhi2, Unigene40527\_JingChaoZuZhi2, Unigene40598\_JingChaoZuZhi2, Unigene40607\_JingChaoZuZhi2, Unigene4072\_JingChaoZuZhi2, Unigene40865\_JingChaoZuZhi2, Unigene40890\_JingChaoZuZhi2, Unigene40914\_JingChaoZuZhi2, Unigene4111\_JingChaoZuZhi2, Unigene41155\_JingChaoZuZhi2, Unigene41227\_JingChaoZuZhi2, Unigene41333\_JingChaoZuZhi2, Unigene41427\_JingChaoZuZhi2, Unigene414\_JingChaoZuZhi2, Unigene41503\_JingChaoZuZhi2, Unigene41513\_JingChaoZuZhi2, Unigene4160\_JingChaoZuZhi2, Unigene41663\_JingChaoZuZhi2, Unigene41683\_JingChaoZuZhi2, Unigene41739\_JingChaoZuZhi2, Unigene41982\_JingChaoZuZhi2, Unigene42036\_JingChaoZuZhi2, Unigene42056\_JingChaoZuZhi2, Unigene4207\_JingChaoZuZhi2, Unigene42157\_JingChaoZuZhi2, Unigene42329\_JingChaoZuZhi2, Unigene42346\_JingChaoZuZhi2, Unigene42364\_JingChaoZuZhi2, Unigene42373\_JingChaoZuZhi2, Unigene42414\_JingChaoZuZhi2, Unigene42610\_JingChaoZuZhi2, Unigene42637\_JingChaoZuZhi2, Unigene42651\_JingChaoZuZhi2, Unigene42670\_JingChaoZuZhi2, Unigene42718\_JingChaoZuZhi2, Unigene42806\_JingChaoZuZhi2, Unigene42809\_JingChaoZuZhi2, Unigene42846\_JingChaoZuZhi2, Unigene42887\_JingChaoZuZhi2, Unigene42890\_JingChaoZuZhi2, Unigene43048\_JingChaoZuZhi2, Unigene43072\_JingChaoZuZhi2, Unigene43097\_JingChaoZuZhi2, Unigene43293\_JingChaoZuZhi2, Unigene4334\_JingChaoZuZhi2, Unigene43410\_JingChaoZuZhi2, Unigene4346\_JingChaoZuZhi2, Unigene43658\_JingChaoZuZhi2, Unigene43723\_JingChaoZuZhi2, Unigene4375\_JingChaoZuZhi2, Unigene4377\_JingChaoZuZhi2, Unigene43926\_JingChaoZuZhi2, Unigene43\_JingChaoZuZhi2, Unigene44000\_JingChaoZuZhi2, Unigene44078\_JingChaoZuZhi2, Unigene44116\_JingChaoZuZhi2, Unigene4413\_JingChaoZuZhi2, Unigene44152\_JingChaoZuZhi2, Unigene44195\_JingChaoZuZhi2, Unigene44196\_JingChaoZuZhi2, Unigene4423\_JingChaoZuZhi2, Unigene44246\_JingChaoZuZhi2, Unigene44252\_JingChaoZuZhi2, Unigene4426\_JingChaoZuZhi2, Unigene44278\_JingChaoZuZhi2, Unigene4428\_JingChaoZuZhi2, Unigene44292\_JingChaoZuZhi2, Unigene4431\_JingChaoZuZhi2, Unigene44544\_JingChaoZuZhi2, Unigene44590\_JingChaoZuZhi2, Unigene4461\_JingChaoZuZhi2, Unigene44745\_JingChaoZuZhi2, Unigene44775\_JingChaoZuZhi2, Unigene44797\_JingChaoZuZhi2, Unigene45176\_JingChaoZuZhi2, Unigene45407\_JingChaoZuZhi2, Unigene4542\_JingChaoZuZhi2, Unigene45512\_JingChaoZuZhi2, Unigene45532\_JingChaoZuZhi2, Unigene45669\_JingChaoZuZhi2, Unigene45682\_JingChaoZuZhi2, Unigene45708\_JingChaoZuZhi2, Unigene45887\_JingChaoZuZhi2, Unigene45888\_JingChaoZuZhi2, Unigene460\_JingChaoZuZhi2, Unigene46190\_JingChaoZuZhi2, Unigene4621\_JingChaoZuZhi2, Unigene46223\_JingChaoZuZhi2, Unigene46280\_JingChaoZuZhi2, Unigene46297\_JingChaoZuZhi2, Unigene46382\_JingChaoZuZhi2, Unigene46463\_JingChaoZuZhi2, Unigene46569\_JingChaoZuZhi2, Unigene46619\_JingChaoZuZhi2, Unigene46661\_JingChaoZuZhi2, Unigene46680\_JingChaoZuZhi2, Unigene46843\_JingChaoZuZhi2, Unigene46849\_JingChaoZuZhi2, Unigene47032\_JingChaoZuZhi2, Unigene47051\_JingChaoZuZhi2, Unigene47095\_JingChaoZuZhi2, Unigene4713\_JingChaoZuZhi2, Unigene47202\_JingChaoZuZhi2, Unigene47206\_JingChaoZuZhi2, Unigene47262\_JingChaoZuZhi2, Unigene47300\_JingChaoZuZhi2, Unigene4738\_JingChaoZuZhi2, Unigene47407\_JingChaoZuZhi2, Unigene47424\_JingChaoZuZhi2, Unigene4763\_JingChaoZuZhi2, Unigene4767\_JingChaoZuZhi2, Unigene47722\_JingChaoZuZhi2, Unigene47794\_JingChaoZuZhi2, Unigene47798\_JingChaoZuZhi2, Unigene47814\_JingChaoZuZhi2, Unigene47824\_JingChaoZuZhi2, Unigene47888\_JingChaoZuZhi2, Unigene47901\_JingChaoZuZhi2, Unigene4795\_JingChaoZuZhi2, Unigene48004\_JingChaoZuZhi2, Unigene48046\_JingChaoZuZhi2, Unigene48070\_JingChaoZuZhi2, Unigene48095\_JingChaoZuZhi2, Unigene48142\_JingChaoZuZhi2, Unigene4816\_JingChaoZuZhi2, Unigene48173\_JingChaoZuZhi2, Unigene4818\_JingChaoZuZhi2, Unigene48331\_JingChaoZuZhi2, Unigene48343\_JingChaoZuZhi2, Unigene4854\_JingChaoZuZhi2, Unigene48626\_JingChaoZuZhi2, Unigene48636\_JingChaoZuZhi2, Unigene48660\_JingChaoZuZhi2, Unigene486\_JingChaoZuZhi2, Unigene48770\_JingChaoZuZhi2, Unigene4889\_JingChaoZuZhi2, Unigene48948\_JingChaoZuZhi2, Unigene48958\_JingChaoZuZhi2, Unigene4900\_JingChaoZuZhi2, Unigene490\_JingChaoZuZhi2, Unigene49331\_JingChaoZuZhi2, Unigene49337\_JingChaoZuZhi2, Unigene49345\_JingChaoZuZhi2, Unigene4954\_JingChaoZuZhi2, Unigene49623\_JingChaoZuZhi2, Unigene49664\_JingChaoZuZhi2, Unigene49766\_JingChaoZuZhi2, Unigene49770\_JingChaoZuZhi2, Unigene49819\_JingChaoZuZhi2, Unigene49963\_JingChaoZuZhi2, Unigene49985\_JingChaoZuZhi2, Unigene49987\_JingChaoZuZhi2, Unigene50147\_JingChaoZuZhi2, Unigene50173\_JingChaoZuZhi2, Unigene50259\_JingChaoZuZhi2, Unigene50284\_JingChaoZuZhi2, Unigene50305\_JingChaoZuZhi2, Unigene50371\_JingChaoZuZhi2, Unigene50422\_JingChaoZuZhi2, Unigene50456\_JingChaoZuZhi2, Unigene50485\_JingChaoZuZhi2, Unigene50538\_JingChaoZuZhi2, Unigene5057\_JingChaoZuZhi2, Unigene5068\_JingChaoZuZhi2, Unigene50833\_JingChaoZuZhi2, Unigene50908\_JingChaoZuZhi2, Unigene50927\_JingChaoZuZhi2, Unigene50937\_JingChaoZuZhi2, Unigene50977\_JingChaoZuZhi2, Unigene51012\_JingChaoZuZhi2, Unigene5113\_JingChaoZuZhi2, Unigene51191\_JingChaoZuZhi2, Unigene51478\_JingChaoZuZhi2, Unigene51504\_JingChaoZuZhi2, Unigene51532\_JingChaoZuZhi2, Unigene51540\_JingChaoZuZhi2, Unigene51567\_JingChaoZuZhi2, Unigene51610\_JingChaoZuZhi2, Unigene51652\_JingChaoZuZhi2, Unigene51678\_JingChaoZuZhi2, Unigene51682\_JingChaoZuZhi2, Unigene51688\_JingChaoZuZhi2, Unigene51741\_JingChaoZuZhi2, Unigene5182\_JingChaoZuZhi2, Unigene51835\_JingChaoZuZhi2, Unigene51848\_JingChaoZuZhi2, Unigene51906\_JingChaoZuZhi2, Unigene5200\_JingChaoZuZhi2, Unigene52036\_JingChaoZuZhi2, Unigene52040\_JingChaoZuZhi2, Unigene52057\_JingChaoZuZhi2, Unigene52142\_JingChaoZuZhi2, Unigene52259\_JingChaoZuZhi2, Unigene52317\_JingChaoZuZhi2, Unigene52383\_JingChaoZuZhi2, Unigene523\_JingChaoZuZhi2, Unigene5240\_JingChaoZuZhi2, Unigene52572\_JingChaoZuZhi2, Unigene52598\_JingChaoZuZhi2, Unigene52712\_JingChaoZuZhi2, Unigene52751\_JingChaoZuZhi2, Unigene52889\_JingChaoZuZhi2, Unigene52898\_JingChaoZuZhi2, Unigene52911\_JingChaoZuZhi2, Unigene52974\_JingChaoZuZhi2, Unigene52981\_JingChaoZuZhi2, Unigene52\_JingChaoZuZhi2, Unigene53030\_JingChaoZuZhi2, Unigene53167\_JingChaoZuZhi2, Unigene53240\_JingChaoZuZhi2, Unigene53362\_JingChaoZuZhi2, Unigene53419\_JingChaoZuZhi2, Unigene53447\_JingChaoZuZhi2, Unigene5345\_JingChaoZuZhi2, Unigene53467\_JingChaoZuZhi2, Unigene53484\_JingChaoZuZhi2, Unigene53510\_JingChaoZuZhi2, Unigene53627\_JingChaoZuZhi2, Unigene53809\_JingChaoZuZhi2, Unigene53812\_JingChaoZuZhi2, Unigene53831\_JingChaoZuZhi2, Unigene53875\_JingChaoZuZhi2, Unigene53966\_JingChaoZuZhi2, Unigene54009\_JingChaoZuZhi2, Unigene54075\_JingChaoZuZhi2, Unigene54236\_JingChaoZuZhi2, Unigene5423\_JingChaoZuZhi2, Unigene542\_JingChaoZuZhi2, Unigene54350\_JingChaoZuZhi2, Unigene54367\_JingChaoZuZhi2, Unigene54387\_JingChaoZuZhi2, Unigene5449\_JingChaoZuZhi2, Unigene54539\_JingChaoZuZhi2, Unigene5457\_JingChaoZuZhi2, Unigene54669\_JingChaoZuZhi2, Unigene54707\_JingChaoZuZhi2, Unigene54768\_JingChaoZuZhi2, Unigene54783\_JingChaoZuZhi2, Unigene5484\_JingChaoZuZhi2, Unigene5496\_JingChaoZuZhi2, Unigene54974\_JingChaoZuZhi2, Unigene55009\_JingChaoZuZhi2, Unigene55063\_JingChaoZuZhi2, Unigene55114\_JingChaoZuZhi2, Unigene5518\_JingChaoZuZhi2, Unigene55268\_JingChaoZuZhi2, Unigene552\_JingChaoZuZhi2, Unigene55355\_JingChaoZuZhi2, Unigene55368\_JingChaoZuZhi2, Unigene55371\_JingChaoZuZhi2, Unigene55482\_JingChaoZuZhi2, Unigene5552\_JingChaoZuZhi2, Unigene55583\_JingChaoZuZhi2, Unigene55617\_JingChaoZuZhi2, Unigene55656\_JingChaoZuZhi2, Unigene55700\_JingChaoZuZhi2, Unigene55725\_JingChaoZuZhi2, Unigene55745\_JingChaoZuZhi2, Unigene55751\_JingChaoZuZhi2, Unigene55810\_JingChaoZuZhi2, Unigene55906\_JingChaoZuZhi2, Unigene55918\_JingChaoZuZhi2, Unigene55983\_JingChaoZuZhi2, Unigene56089\_JingChaoZuZhi2, Unigene56290\_JingChaoZuZhi2, Unigene56370\_JingChaoZuZhi2, Unigene5640\_JingChaoZuZhi2, Unigene56557\_JingChaoZuZhi2, Unigene5663\_JingChaoZuZhi2, Unigene56660\_JingChaoZuZhi2, Unigene56745\_JingChaoZuZhi2, Unigene56889\_JingChaoZuZhi2, Unigene56961\_JingChaoZuZhi2, Unigene57015\_JingChaoZuZhi2, Unigene57061\_JingChaoZuZhi2, Unigene57130\_JingChaoZuZhi2, Unigene5714\_JingChaoZuZhi2, Unigene57410\_JingChaoZuZhi2, Unigene5750\_JingChaoZuZhi2, Unigene57511\_JingChaoZuZhi2, Unigene57596\_JingChaoZuZhi2, Unigene57635\_JingChaoZuZhi2, Unigene57726\_JingChaoZuZhi2, Unigene5782\_JingChaoZuZhi2, Unigene57841\_JingChaoZuZhi2, Unigene57878\_JingChaoZuZhi2, Unigene57931\_JingChaoZuZhi2, Unigene57967\_JingChaoZuZhi2, Unigene57970\_JingChaoZuZhi2, Unigene58141\_JingChaoZuZhi2, Unigene58214\_JingChaoZuZhi2, Unigene58224\_JingChaoZuZhi2, Unigene58354\_JingChaoZuZhi2, Unigene58437\_JingChaoZuZhi2, Unigene58502\_JingChaoZuZhi2, Unigene5852\_JingChaoZuZhi2, Unigene58621\_JingChaoZuZhi2, Unigene5875\_JingChaoZuZhi2, Unigene58763\_JingChaoZuZhi2, Unigene58963\_JingChaoZuZhi2, Unigene5897\_JingChaoZuZhi2, Unigene59000\_JingChaoZuZhi2, Unigene5900\_JingChaoZuZhi2, Unigene59038\_JingChaoZuZhi2, Unigene59180\_JingChaoZuZhi2, Unigene59210\_JingChaoZuZhi2, Unigene5938\_JingChaoZuZhi2, Unigene59427\_JingChaoZuZhi2, Unigene59449\_JingChaoZuZhi2, Unigene5944\_JingChaoZuZhi2, Unigene59480\_JingChaoZuZhi2, Unigene59493\_JingChaoZuZhi2, Unigene59502\_JingChaoZuZhi2, Unigene59548\_JingChaoZuZhi2, Unigene59570\_JingChaoZuZhi2, Unigene5957\_JingChaoZuZhi2, Unigene59684\_JingChaoZuZhi2, Unigene59697\_JingChaoZuZhi2, Unigene59731\_JingChaoZuZhi2, Unigene59735\_JingChaoZuZhi2, Unigene59764\_JingChaoZuZhi2, Unigene59836\_JingChaoZuZhi2, Unigene59837\_JingChaoZuZhi2, Unigene59979\_JingChaoZuZhi2, Unigene6009\_JingChaoZuZhi2, Unigene60108\_JingChaoZuZhi2, Unigene60117\_JingChaoZuZhi2, Unigene60177\_JingChaoZuZhi2, Unigene60210\_JingChaoZuZhi2, Unigene60260\_JingChaoZuZhi2, Unigene60326\_JingChaoZuZhi2, Unigene60406\_JingChaoZuZhi2, Unigene60781\_JingChaoZuZhi2, Unigene6078\_JingChaoZuZhi2, Unigene60894\_JingChaoZuZhi2, Unigene60905\_JingChaoZuZhi2, Unigene60906\_JingChaoZuZhi2, Unigene60942\_JingChaoZuZhi2, Unigene6096\_JingChaoZuZhi2, Unigene60970\_JingChaoZuZhi2, Unigene61121\_JingChaoZuZhi2, Unigene61162\_JingChaoZuZhi2, Unigene6117\_JingChaoZuZhi2, Unigene61189\_JingChaoZuZhi2, Unigene61304\_JingChaoZuZhi2, Unigene6134\_JingChaoZuZhi2, Unigene61380\_JingChaoZuZhi2, Unigene61651\_JingChaoZuZhi2, Unigene61659\_JingChaoZuZhi2, Unigene61664\_JingChaoZuZhi2, Unigene6166\_JingChaoZuZhi2, Unigene61679\_JingChaoZuZhi2, Unigene61702\_JingChaoZuZhi2, Unigene61708\_JingChaoZuZhi2, Unigene61836\_JingChaoZuZhi2, Unigene61\_JingChaoZuZhi2, Unigene62191\_JingChaoZuZhi2, Unigene6219\_JingChaoZuZhi2, Unigene62246\_JingChaoZuZhi2, Unigene62337\_JingChaoZuZhi2, Unigene6233\_JingChaoZuZhi2, Unigene62415\_JingChaoZuZhi2, Unigene6246\_JingChaoZuZhi2, Unigene6247\_JingChaoZuZhi2, Unigene6250\_JingChaoZuZhi2, Unigene62515\_JingChaoZuZhi2, Unigene62545\_JingChaoZuZhi2, Unigene62568\_JingChaoZuZhi2, Unigene62603\_JingChaoZuZhi2, Unigene62804\_JingChaoZuZhi2, Unigene62887\_JingChaoZuZhi2, Unigene62892\_JingChaoZuZhi2, Unigene62924\_JingChaoZuZhi2, Unigene62962\_JingChaoZuZhi2, Unigene62994\_JingChaoZuZhi2, Unigene63067\_JingChaoZuZhi2, Unigene630\_JingChaoZuZhi2, Unigene63112\_JingChaoZuZhi2, Unigene63170\_JingChaoZuZhi2, Unigene63175\_JingChaoZuZhi2, Unigene63261\_JingChaoZuZhi2, Unigene63341\_JingChaoZuZhi2, Unigene63374\_JingChaoZuZhi2, Unigene63375\_JingChaoZuZhi2, Unigene63384\_JingChaoZuZhi2, Unigene63447\_JingChaoZuZhi2, Unigene63458\_JingChaoZuZhi2, Unigene63485\_JingChaoZuZhi2, Unigene634\_JingChaoZuZhi2, Unigene63649\_JingChaoZuZhi2, Unigene63705\_JingChaoZuZhi2, Unigene63715\_JingChaoZuZhi2, Unigene63747\_JingChaoZuZhi2, Unigene63807\_JingChaoZuZhi2, Unigene63935\_JingChaoZuZhi2, Unigene63968\_JingChaoZuZhi2, Unigene6396\_JingChaoZuZhi2, Unigene64058\_JingChaoZuZhi2, Unigene64169\_JingChaoZuZhi2, Unigene64217\_JingChaoZuZhi2, Unigene64315\_JingChaoZuZhi2, Unigene64393\_JingChaoZuZhi2, Unigene64434\_JingChaoZuZhi2, Unigene64489\_JingChaoZuZhi2, Unigene64615\_JingChaoZuZhi2, Unigene64627\_JingChaoZuZhi2, Unigene64643\_JingChaoZuZhi2, Unigene64673\_JingChaoZuZhi2, Unigene64846\_JingChaoZuZhi2, Unigene64862\_JingChaoZuZhi2, Unigene64903\_JingChaoZuZhi2, Unigene6494\_JingChaoZuZhi2, Unigene64991\_JingChaoZuZhi2, Unigene65006\_JingChaoZuZhi2, Unigene65187\_JingChaoZuZhi2, Unigene65222\_JingChaoZuZhi2, Unigene65278\_JingChaoZuZhi2, Unigene65330\_JingChaoZuZhi2, Unigene65405\_JingChaoZuZhi2, Unigene65441\_JingChaoZuZhi2, Unigene65445\_JingChaoZuZhi2, Unigene65475\_JingChaoZuZhi2, Unigene65498\_JingChaoZuZhi2, Unigene65515\_JingChaoZuZhi2, Unigene65525\_JingChaoZuZhi2, Unigene65555\_JingChaoZuZhi2, Unigene65632\_JingChaoZuZhi2, Unigene65721\_JingChaoZuZhi2, Unigene6573\_JingChaoZuZhi2, Unigene65800\_JingChaoZuZhi2, Unigene65807\_JingChaoZuZhi2, Unigene65835\_JingChaoZuZhi2, Unigene65960\_JingChaoZuZhi2, Unigene66246\_JingChaoZuZhi2, Unigene662\_JingChaoZuZhi2, Unigene66337\_JingChaoZuZhi2, Unigene66456\_JingChaoZuZhi2, Unigene66496\_JingChaoZuZhi2, Unigene66535\_JingChaoZuZhi2, Unigene66540\_JingChaoZuZhi2, Unigene66615\_JingChaoZuZhi2, Unigene66806\_JingChaoZuZhi2, Unigene66815\_JingChaoZuZhi2, Unigene66850\_JingChaoZuZhi2, Unigene6685\_JingChaoZuZhi2, Unigene66888\_JingChaoZuZhi2, Unigene66954\_JingChaoZuZhi2, Unigene67191\_JingChaoZuZhi2, Unigene67318\_JingChaoZuZhi2, Unigene67328\_JingChaoZuZhi2, Unigene67337\_JingChaoZuZhi2, Unigene6739\_JingChaoZuZhi2, Unigene67411\_JingChaoZuZhi2, Unigene67412\_JingChaoZuZhi2, Unigene67588\_JingChaoZuZhi2, Unigene67589\_JingChaoZuZhi2, Unigene67613\_JingChaoZuZhi2, Unigene67655\_JingChaoZuZhi2, Unigene67673\_JingChaoZuZhi2, Unigene67767\_JingChaoZuZhi2, Unigene6783\_JingChaoZuZhi2, Unigene6790\_JingChaoZuZhi2, Unigene67946\_JingChaoZuZhi2, Unigene67964\_JingChaoZuZhi2, Unigene67968\_JingChaoZuZhi2, Unigene6797\_JingChaoZuZhi2, Unigene67981\_JingChaoZuZhi2, Unigene68001\_JingChaoZuZhi2, Unigene68015\_JingChaoZuZhi2, Unigene68024\_JingChaoZuZhi2, Unigene6802\_JingChaoZuZhi2, Unigene68045\_JingChaoZuZhi2, Unigene68097\_JingChaoZuZhi2, Unigene68129\_JingChaoZuZhi2, Unigene68151\_JingChaoZuZhi2, Unigene68159\_JingChaoZuZhi2, Unigene68200\_JingChaoZuZhi2, Unigene68356\_JingChaoZuZhi2, Unigene68497\_JingChaoZuZhi2, Unigene684\_JingChaoZuZhi2, Unigene68502\_JingChaoZuZhi2, Unigene68509\_JingChaoZuZhi2, Unigene68599\_JingChaoZuZhi2, Unigene685\_JingChaoZuZhi2, Unigene6862\_JingChaoZuZhi2, Unigene6868\_JingChaoZuZhi2, Unigene68698\_JingChaoZuZhi2, Unigene68716\_JingChaoZuZhi2, Unigene68832\_JingChaoZuZhi2, Unigene68920\_JingChaoZuZhi2, Unigene68962\_JingChaoZuZhi2, Unigene69092\_JingChaoZuZhi2, Unigene69155\_JingChaoZuZhi2, Unigene69176\_JingChaoZuZhi2, Unigene69209\_JingChaoZuZhi2, Unigene69249\_JingChaoZuZhi2, Unigene69255\_JingChaoZuZhi2, Unigene69271\_JingChaoZuZhi2, Unigene69314\_JingChaoZuZhi2, Unigene69398\_JingChaoZuZhi2, Unigene6945\_JingChaoZuZhi2, Unigene69536\_JingChaoZuZhi2, Unigene6955\_JingChaoZuZhi2, Unigene69571\_JingChaoZuZhi2, Unigene69579\_JingChaoZuZhi2, Unigene69601\_JingChaoZuZhi2, Unigene69639\_JingChaoZuZhi2, Unigene69752\_JingChaoZuZhi2, Unigene69875\_JingChaoZuZhi2, Unigene69922\_JingChaoZuZhi2, Unigene69978\_JingChaoZuZhi2, Unigene70140\_JingChaoZuZhi2, Unigene70190\_JingChaoZuZhi2, Unigene70225\_JingChaoZuZhi2, Unigene70244\_JingChaoZuZhi2, Unigene70308\_JingChaoZuZhi2, Unigene70315\_JingChaoZuZhi2, Unigene70342\_JingChaoZuZhi2, Unigene70355\_JingChaoZuZhi2, Unigene70488\_JingChaoZuZhi2, Unigene7059\_JingChaoZuZhi2, Unigene70615\_JingChaoZuZhi2, Unigene70620\_JingChaoZuZhi2, Unigene70647\_JingChaoZuZhi2, Unigene70659\_JingChaoZuZhi2, Unigene70672\_JingChaoZuZhi2, Unigene70703\_JingChaoZuZhi2, Unigene70779\_JingChaoZuZhi2, Unigene70824\_JingChaoZuZhi2, Unigene7082\_JingChaoZuZhi2, Unigene70893\_JingChaoZuZhi2, Unigene70921\_JingChaoZuZhi2, Unigene71103\_JingChaoZuZhi2, Unigene7110\_JingChaoZuZhi2, Unigene71152\_JingChaoZuZhi2, Unigene71153\_JingChaoZuZhi2, Unigene71196\_JingChaoZuZhi2, Unigene71235\_JingChaoZuZhi2, Unigene71246\_JingChaoZuZhi2, Unigene71248\_JingChaoZuZhi2, Unigene71418\_JingChaoZuZhi2, Unigene71482\_JingChaoZuZhi2, Unigene71639\_JingChaoZuZhi2, Unigene71724\_JingChaoZuZhi2, Unigene71728\_JingChaoZuZhi2, Unigene7176\_JingChaoZuZhi2, Unigene7178\_JingChaoZuZhi2, Unigene7185\_JingChaoZuZhi2, Unigene71936\_JingChaoZuZhi2, Unigene71976\_JingChaoZuZhi2, Unigene72016\_JingChaoZuZhi2, Unigene72098\_JingChaoZuZhi2, Unigene72136\_JingChaoZuZhi2, Unigene72384\_JingChaoZuZhi2, Unigene7242\_JingChaoZuZhi2, Unigene72461\_JingChaoZuZhi2, Unigene72466\_JingChaoZuZhi2, Unigene72474\_JingChaoZuZhi2, Unigene72476\_JingChaoZuZhi2, Unigene72508\_JingChaoZuZhi2, Unigene72565\_JingChaoZuZhi2, Unigene72674\_JingChaoZuZhi2, Unigene72686\_JingChaoZuZhi2, Unigene72767\_JingChaoZuZhi2, Unigene72813\_JingChaoZuZhi2, Unigene7283\_JingChaoZuZhi2, Unigene72849\_JingChaoZuZhi2, Unigene72901\_JingChaoZuZhi2, Unigene72924\_JingChaoZuZhi2, Unigene72974\_JingChaoZuZhi2, Unigene729\_JingChaoZuZhi2, Unigene73018\_JingChaoZuZhi2, Unigene73037\_JingChaoZuZhi2, Unigene7314\_JingChaoZuZhi2, Unigene73150\_JingChaoZuZhi2, Unigene7326\_JingChaoZuZhi2, Unigene73283\_JingChaoZuZhi2, Unigene73297\_JingChaoZuZhi2, Unigene73451\_JingChaoZuZhi2, Unigene73543\_JingChaoZuZhi2, Unigene73561\_JingChaoZuZhi2, Unigene73632\_JingChaoZuZhi2, Unigene73740\_JingChaoZuZhi2, Unigene73757\_JingChaoZuZhi2, Unigene73765\_JingChaoZuZhi2, Unigene73853\_JingChaoZuZhi2, Unigene73857\_JingChaoZuZhi2, Unigene73913\_JingChaoZuZhi2, Unigene73953\_JingChaoZuZhi2, Unigene74062\_JingChaoZuZhi2, Unigene7407\_JingChaoZuZhi2, Unigene74216\_JingChaoZuZhi2, Unigene74274\_JingChaoZuZhi2, Unigene7436\_JingChaoZuZhi2, Unigene74409\_JingChaoZuZhi2, Unigene74505\_JingChaoZuZhi2, Unigene74517\_JingChaoZuZhi2, Unigene74587\_JingChaoZuZhi2, Unigene74590\_JingChaoZuZhi2, Unigene74594\_JingChaoZuZhi2, Unigene74612\_JingChaoZuZhi2, Unigene74643\_JingChaoZuZhi2, Unigene74725\_JingChaoZuZhi2, Unigene74736\_JingChaoZuZhi2, Unigene74771\_JingChaoZuZhi2, Unigene7493\_JingChaoZuZhi2, Unigene75050\_JingChaoZuZhi2, Unigene7508\_JingChaoZuZhi2, Unigene75124\_JingChaoZuZhi2, Unigene75125\_JingChaoZuZhi2, Unigene75288\_JingChaoZuZhi2, Unigene7528\_JingChaoZuZhi2, Unigene7537\_JingChaoZuZhi2, Unigene75416\_JingChaoZuZhi2, Unigene75459\_JingChaoZuZhi2, Unigene75469\_JingChaoZuZhi2, Unigene75514\_JingChaoZuZhi2, Unigene75529\_JingChaoZuZhi2, Unigene75535\_JingChaoZuZhi2, Unigene75617\_JingChaoZuZhi2, Unigene75625\_JingChaoZuZhi2, Unigene75690\_JingChaoZuZhi2, Unigene75743\_JingChaoZuZhi2, Unigene75749\_JingChaoZuZhi2, Unigene75828\_JingChaoZuZhi2, Unigene75891\_JingChaoZuZhi2, Unigene75912\_JingChaoZuZhi2, Unigene75919\_JingChaoZuZhi2, Unigene7591\_JingChaoZuZhi2, Unigene75945\_JingChaoZuZhi2, Unigene75948\_JingChaoZuZhi2, Unigene76000\_JingChaoZuZhi2, Unigene76052\_JingChaoZuZhi2, Unigene76057\_JingChaoZuZhi2, Unigene76068\_JingChaoZuZhi2, Unigene7608\_JingChaoZuZhi2, Unigene76109\_JingChaoZuZhi2, Unigene76128\_JingChaoZuZhi2, Unigene76182\_JingChaoZuZhi2, Unigene76261\_JingChaoZuZhi2, Unigene76331\_JingChaoZuZhi2, Unigene76337\_JingChaoZuZhi2, Unigene76369\_JingChaoZuZhi2, Unigene76385\_JingChaoZuZhi2, Unigene7641\_JingChaoZuZhi2, Unigene76424\_JingChaoZuZhi2, Unigene76438\_JingChaoZuZhi2, Unigene76458\_JingChaoZuZhi2, Unigene76480\_JingChaoZuZhi2, Unigene76502\_JingChaoZuZhi2, Unigene76514\_JingChaoZuZhi2, Unigene76593\_JingChaoZuZhi2, Unigene76647\_JingChaoZuZhi2, Unigene76651\_JingChaoZuZhi2, Unigene76668\_JingChaoZuZhi2, Unigene76706\_JingChaoZuZhi2, Unigene76769\_JingChaoZuZhi2, Unigene76824\_JingChaoZuZhi2, Unigene76865\_JingChaoZuZhi2, Unigene76868\_JingChaoZuZhi2, Unigene76892\_JingChaoZuZhi2, Unigene76896\_JingChaoZuZhi2, Unigene76903\_JingChaoZuZhi2, Unigene76908\_JingChaoZuZhi2, Unigene76915\_JingChaoZuZhi2, Unigene76957\_JingChaoZuZhi2, Unigene77016\_JingChaoZuZhi2, Unigene77041\_JingChaoZuZhi2, Unigene77149\_JingChaoZuZhi2, Unigene77192\_JingChaoZuZhi2, Unigene77193\_JingChaoZuZhi2, Unigene77197\_JingChaoZuZhi2, Unigene77249\_JingChaoZuZhi2, Unigene77256\_JingChaoZuZhi2, Unigene77259\_JingChaoZuZhi2, Unigene77344\_JingChaoZuZhi2, Unigene77374\_JingChaoZuZhi2, Unigene77449\_JingChaoZuZhi2, Unigene77454\_JingChaoZuZhi2, Unigene77482\_JingChaoZuZhi2, Unigene77528\_JingChaoZuZhi2, Unigene77559\_JingChaoZuZhi2, Unigene77631\_JingChaoZuZhi2, Unigene77649\_JingChaoZuZhi2, Unigene7767\_JingChaoZuZhi2, Unigene77749\_JingChaoZuZhi2, Unigene77779\_JingChaoZuZhi2, Unigene77844\_JingChaoZuZhi2, Unigene77892\_JingChaoZuZhi2, Unigene77903\_JingChaoZuZhi2, Unigene77994\_JingChaoZuZhi2, Unigene7799\_JingChaoZuZhi2, Unigene78070\_JingChaoZuZhi2, Unigene78071\_JingChaoZuZhi2, Unigene78078\_JingChaoZuZhi2, Unigene78083\_JingChaoZuZhi2, Unigene7810\_JingChaoZuZhi2, Unigene78168\_JingChaoZuZhi2, Unigene7817\_JingChaoZuZhi2, Unigene78189\_JingChaoZuZhi2, Unigene78224\_JingChaoZuZhi2, Unigene78226\_JingChaoZuZhi2, Unigene78268\_JingChaoZuZhi2, Unigene78415\_JingChaoZuZhi2, Unigene78417\_JingChaoZuZhi2, Unigene7842\_JingChaoZuZhi2, Unigene78435\_JingChaoZuZhi2, Unigene78518\_JingChaoZuZhi2, Unigene78578\_JingChaoZuZhi2, Unigene78619\_JingChaoZuZhi2, Unigene78625\_JingChaoZuZhi2, Unigene78672\_JingChaoZuZhi2, Unigene78726\_JingChaoZuZhi2, Unigene78752\_JingChaoZuZhi2, Unigene78757\_JingChaoZuZhi2, Unigene78781\_JingChaoZuZhi2, Unigene78813\_JingChaoZuZhi2, Unigene7881\_JingChaoZuZhi2, Unigene78854\_JingChaoZuZhi2, Unigene78885\_JingChaoZuZhi2, Unigene78939\_JingChaoZuZhi2, Unigene78992\_JingChaoZuZhi2, Unigene78998\_JingChaoZuZhi2, Unigene7899\_JingChaoZuZhi2, Unigene79033\_JingChaoZuZhi2, Unigene79036\_JingChaoZuZhi2, Unigene79066\_JingChaoZuZhi2, Unigene79135\_JingChaoZuZhi2, Unigene79149\_JingChaoZuZhi2, Unigene79166\_JingChaoZuZhi2, Unigene79196\_JingChaoZuZhi2, Unigene79198\_JingChaoZuZhi2, Unigene79203\_JingChaoZuZhi2, Unigene79210\_JingChaoZuZhi2, Unigene79211\_JingChaoZuZhi2, Unigene79234\_JingChaoZuZhi2, Unigene79239\_JingChaoZuZhi2, Unigene79246\_JingChaoZuZhi2, Unigene79258\_JingChaoZuZhi2, Unigene79268\_JingChaoZuZhi2, Unigene79288\_JingChaoZuZhi2, Unigene79414\_JingChaoZuZhi2, Unigene7947\_JingChaoZuZhi2, Unigene7948\_JingChaoZuZhi2, Unigene79515\_JingChaoZuZhi2, Unigene79523\_JingChaoZuZhi2, Unigene79539\_JingChaoZuZhi2, Unigene79577\_JingChaoZuZhi2, Unigene79590\_JingChaoZuZhi2, Unigene79613\_JingChaoZuZhi2, Unigene79637\_JingChaoZuZhi2, Unigene7964\_JingChaoZuZhi2, Unigene7966\_JingChaoZuZhi2, Unigene79698\_JingChaoZuZhi2, Unigene7985\_JingChaoZuZhi2, Unigene79879\_JingChaoZuZhi2, Unigene79892\_JingChaoZuZhi2, Unigene79895\_JingChaoZuZhi2, Unigene79906\_JingChaoZuZhi2, Unigene7990\_JingChaoZuZhi2, Unigene79976\_JingChaoZuZhi2, Unigene79999\_JingChaoZuZhi2, Unigene80008\_JingChaoZuZhi2, Unigene80032\_JingChaoZuZhi2, Unigene8011\_JingChaoZuZhi2, Unigene80141\_JingChaoZuZhi2, Unigene80142\_JingChaoZuZhi2, Unigene80205\_JingChaoZuZhi2, Unigene80211\_JingChaoZuZhi2, Unigene80222\_JingChaoZuZhi2, Unigene80265\_JingChaoZuZhi2, Unigene80285\_JingChaoZuZhi2, Unigene80305\_JingChaoZuZhi2, Unigene80328\_JingChaoZuZhi2, Unigene80334\_JingChaoZuZhi2, Unigene80375\_JingChaoZuZhi2, Unigene80397\_JingChaoZuZhi2, Unigene80446\_JingChaoZuZhi2, Unigene80504\_JingChaoZuZhi2, Unigene80528\_JingChaoZuZhi2, Unigene80590\_JingChaoZuZhi2, Unigene80622\_JingChaoZuZhi2, Unigene80635\_JingChaoZuZhi2, Unigene80636\_JingChaoZuZhi2, Unigene80736\_JingChaoZuZhi2, Unigene8075\_JingChaoZuZhi2, Unigene80809\_JingChaoZuZhi2, Unigene80848\_JingChaoZuZhi2, Unigene80881\_JingChaoZuZhi2, Unigene80895\_JingChaoZuZhi2, Unigene80932\_JingChaoZuZhi2, Unigene80948\_JingChaoZuZhi2, Unigene80971\_JingChaoZuZhi2, Unigene81110\_JingChaoZuZhi2, Unigene81192\_JingChaoZuZhi2, Unigene81293\_JingChaoZuZhi2, Unigene81297\_JingChaoZuZhi2, Unigene81346\_JingChaoZuZhi2, Unigene81481\_JingChaoZuZhi2, Unigene814\_JingChaoZuZhi2, Unigene81511\_JingChaoZuZhi2, Unigene81536\_JingChaoZuZhi2, Unigene8153\_JingChaoZuZhi2, Unigene81575\_JingChaoZuZhi2, Unigene81597\_JingChaoZuZhi2, Unigene81615\_JingChaoZuZhi2, Unigene81630\_JingChaoZuZhi2, Unigene81640\_JingChaoZuZhi2, Unigene81646\_JingChaoZuZhi2, Unigene81654\_JingChaoZuZhi2, Unigene81661\_JingChaoZuZhi2, Unigene81674\_JingChaoZuZhi2, Unigene81706\_JingChaoZuZhi2, Unigene81708\_JingChaoZuZhi2, Unigene81722\_JingChaoZuZhi2, Unigene8175\_JingChaoZuZhi2, Unigene81770\_JingChaoZuZhi2, Unigene81774\_JingChaoZuZhi2, Unigene81825\_JingChaoZuZhi2, Unigene81878\_JingChaoZuZhi2, Unigene81935\_JingChaoZuZhi2, Unigene81947\_JingChaoZuZhi2, Unigene81951\_JingChaoZuZhi2, Unigene81997\_JingChaoZuZhi2, Unigene82129\_JingChaoZuZhi2, Unigene8213\_JingChaoZuZhi2, Unigene82158\_JingChaoZuZhi2, Unigene82161\_JingChaoZuZhi2, Unigene82170\_JingChaoZuZhi2, Unigene82186\_JingChaoZuZhi2, Unigene82234\_JingChaoZuZhi2, Unigene8223\_JingChaoZuZhi2, Unigene82258\_JingChaoZuZhi2, Unigene8225\_JingChaoZuZhi2, Unigene82270\_JingChaoZuZhi2, Unigene82297\_JingChaoZuZhi2, Unigene8235\_JingChaoZuZhi2, Unigene82389\_JingChaoZuZhi2, Unigene82393\_JingChaoZuZhi2, Unigene82414\_JingChaoZuZhi2, Unigene82435\_JingChaoZuZhi2, Unigene82464\_JingChaoZuZhi2, Unigene82503\_JingChaoZuZhi2, Unigene82594\_JingChaoZuZhi2, Unigene82645\_JingChaoZuZhi2, Unigene82671\_JingChaoZuZhi2, Unigene82744\_JingChaoZuZhi2, Unigene8278\_JingChaoZuZhi2, Unigene82808\_JingChaoZuZhi2, Unigene82817\_JingChaoZuZhi2, Unigene82818\_JingChaoZuZhi2, Unigene82835\_JingChaoZuZhi2, Unigene82868\_JingChaoZuZhi2, Unigene82901\_JingChaoZuZhi2, Unigene83023\_JingChaoZuZhi2, Unigene83101\_JingChaoZuZhi2, Unigene8310\_JingChaoZuZhi2, Unigene83110\_JingChaoZuZhi2, Unigene83131\_JingChaoZuZhi2, Unigene83132\_JingChaoZuZhi2, Unigene83134\_JingChaoZuZhi2, Unigene83138\_JingChaoZuZhi2, Unigene83182\_JingChaoZuZhi2, Unigene83218\_JingChaoZuZhi2, Unigene83268\_JingChaoZuZhi2, Unigene83273\_JingChaoZuZhi2, Unigene83277\_JingChaoZuZhi2, Unigene83297\_JingChaoZuZhi2, Unigene83308\_JingChaoZuZhi2, Unigene83318\_JingChaoZuZhi2, Unigene83328\_JingChaoZuZhi2, Unigene83364\_JingChaoZuZhi2, Unigene83409\_JingChaoZuZhi2, Unigene83411\_JingChaoZuZhi2, Unigene83440\_JingChaoZuZhi2, Unigene83448\_JingChaoZuZhi2, Unigene83490\_JingChaoZuZhi2, Unigene83531\_JingChaoZuZhi2, Unigene83564\_JingChaoZuZhi2, Unigene83570\_JingChaoZuZhi2, Unigene83623\_JingChaoZuZhi2, Unigene83626\_JingChaoZuZhi2, Unigene83716\_JingChaoZuZhi2, Unigene83795\_JingChaoZuZhi2, Unigene8379\_JingChaoZuZhi2, Unigene83846\_JingChaoZuZhi2, Unigene83851\_JingChaoZuZhi2, Unigene83854\_JingChaoZuZhi2, Unigene83898\_JingChaoZuZhi2, Unigene83913\_JingChaoZuZhi2, Unigene83939\_JingChaoZuZhi2, Unigene83966\_JingChaoZuZhi2, Unigene83981\_JingChaoZuZhi2, Unigene84021\_JingChaoZuZhi2, Unigene84050\_JingChaoZuZhi2, Unigene84063\_JingChaoZuZhi2, Unigene84076\_JingChaoZuZhi2, Unigene84124\_JingChaoZuZhi2, Unigene84149\_JingChaoZuZhi2, Unigene84154\_JingChaoZuZhi2, Unigene84181\_JingChaoZuZhi2, Unigene84203\_JingChaoZuZhi2, Unigene84222\_JingChaoZuZhi2, Unigene84224\_JingChaoZuZhi2, Unigene84279\_JingChaoZuZhi2, Unigene84292\_JingChaoZuZhi2, Unigene84343\_JingChaoZuZhi2, Unigene84345\_JingChaoZuZhi2, Unigene84371\_JingChaoZuZhi2, Unigene84378\_JingChaoZuZhi2, Unigene84385\_JingChaoZuZhi2, Unigene84416\_JingChaoZuZhi2, Unigene84417\_JingChaoZuZhi2, Unigene84420\_JingChaoZuZhi2, Unigene8442\_JingChaoZuZhi2, Unigene84436\_JingChaoZuZhi2, Unigene84523\_JingChaoZuZhi2, Unigene84531\_JingChaoZuZhi2, Unigene84532\_JingChaoZuZhi2, Unigene84539\_JingChaoZuZhi2, Unigene84546\_JingChaoZuZhi2, Unigene8454\_JingChaoZuZhi2, Unigene84626\_JingChaoZuZhi2, Unigene84663\_JingChaoZuZhi2, Unigene84682\_JingChaoZuZhi2, Unigene84701\_JingChaoZuZhi2, Unigene84785\_JingChaoZuZhi2, Unigene84790\_JingChaoZuZhi2, Unigene84831\_JingChaoZuZhi2, Unigene84839\_JingChaoZuZhi2, Unigene84851\_JingChaoZuZhi2, Unigene84876\_JingChaoZuZhi2, Unigene84894\_JingChaoZuZhi2, Unigene8506\_JingChaoZuZhi2, Unigene8508\_JingChaoZuZhi2, Unigene85107\_JingChaoZuZhi2, Unigene8512\_JingChaoZuZhi2, Unigene85131\_JingChaoZuZhi2, Unigene8517\_JingChaoZuZhi2, Unigene85224\_JingChaoZuZhi2, Unigene85304\_JingChaoZuZhi2, Unigene85353\_JingChaoZuZhi2, Unigene85355\_JingChaoZuZhi2, Unigene85365\_JingChaoZuZhi2, Unigene85366\_JingChaoZuZhi2, Unigene85391\_JingChaoZuZhi2, Unigene85428\_JingChaoZuZhi2, Unigene85449\_JingChaoZuZhi2, Unigene85473\_JingChaoZuZhi2, Unigene85477\_JingChaoZuZhi2, Unigene85539\_JingChaoZuZhi2, Unigene85584\_JingChaoZuZhi2, Unigene85591\_JingChaoZuZhi2, Unigene85640\_JingChaoZuZhi2, Unigene85687\_JingChaoZuZhi2, Unigene85693\_JingChaoZuZhi2, Unigene85742\_JingChaoZuZhi2, Unigene85745\_JingChaoZuZhi2, Unigene85769\_JingChaoZuZhi2, Unigene85789\_JingChaoZuZhi2, Unigene85814\_JingChaoZuZhi2, Unigene85937\_JingChaoZuZhi2, Unigene85939\_JingChaoZuZhi2, Unigene85949\_JingChaoZuZhi2, Unigene85989\_JingChaoZuZhi2, Unigene85996\_JingChaoZuZhi2, Unigene86007\_JingChaoZuZhi2, Unigene86034\_JingChaoZuZhi2, Unigene86035\_JingChaoZuZhi2, Unigene86058\_JingChaoZuZhi2, Unigene8606\_JingChaoZuZhi2, Unigene86120\_JingChaoZuZhi2, Unigene86141\_JingChaoZuZhi2, Unigene86157\_JingChaoZuZhi2, Unigene86198\_JingChaoZuZhi2, Unigene86210\_JingChaoZuZhi2, Unigene86213\_JingChaoZuZhi2, Unigene86271\_JingChaoZuZhi2, Unigene86295\_JingChaoZuZhi2, Unigene86307\_JingChaoZuZhi2, Unigene86319\_JingChaoZuZhi2, Unigene86332\_JingChaoZuZhi2, Unigene8641\_JingChaoZuZhi2, Unigene8643\_JingChaoZuZhi2, Unigene86463\_JingChaoZuZhi2, Unigene86528\_JingChaoZuZhi2, Unigene86545\_JingChaoZuZhi2, Unigene86566\_JingChaoZuZhi2, Unigene86568\_JingChaoZuZhi2, Unigene86582\_JingChaoZuZhi2, Unigene86585\_JingChaoZuZhi2, Unigene86607\_JingChaoZuZhi2, Unigene86608\_JingChaoZuZhi2, Unigene86708\_JingChaoZuZhi2, Unigene86715\_JingChaoZuZhi2, Unigene86734\_JingChaoZuZhi2, Unigene86770\_JingChaoZuZhi2, Unigene86806\_JingChaoZuZhi2, Unigene86821\_JingChaoZuZhi2, Unigene86826\_JingChaoZuZhi2, Unigene8689\_JingChaoZuZhi2, Unigene86915\_JingChaoZuZhi2, Unigene86967\_JingChaoZuZhi2, Unigene86987\_JingChaoZuZhi2, Unigene86995\_JingChaoZuZhi2, Unigene86998\_JingChaoZuZhi2, Unigene87007\_JingChaoZuZhi2, Unigene87015\_JingChaoZuZhi2, Unigene87018\_JingChaoZuZhi2, Unigene87025\_JingChaoZuZhi2, Unigene87044\_JingChaoZuZhi2, Unigene87046\_JingChaoZuZhi2, Unigene87054\_JingChaoZuZhi2, Unigene87060\_JingChaoZuZhi2, Unigene87092\_JingChaoZuZhi2, Unigene8712\_JingChaoZuZhi2, Unigene87210\_JingChaoZuZhi2, Unigene87222\_JingChaoZuZhi2, Unigene87230\_JingChaoZuZhi2, Unigene87332\_JingChaoZuZhi2, Unigene87347\_JingChaoZuZhi2, Unigene87349\_JingChaoZuZhi2, Unigene8737\_JingChaoZuZhi2, Unigene87389\_JingChaoZuZhi2, Unigene87422\_JingChaoZuZhi2, Unigene87433\_JingChaoZuZhi2, Unigene87478\_JingChaoZuZhi2, Unigene87480\_JingChaoZuZhi2, Unigene87485\_JingChaoZuZhi2, Unigene87524\_JingChaoZuZhi2, Unigene87559\_JingChaoZuZhi2, Unigene8755\_JingChaoZuZhi2, Unigene87583\_JingChaoZuZhi2, Unigene87599\_JingChaoZuZhi2, Unigene87607\_JingChaoZuZhi2, Unigene87776\_JingChaoZuZhi2, Unigene87796\_JingChaoZuZhi2, Unigene87820\_JingChaoZuZhi2, Unigene87847\_JingChaoZuZhi2, Unigene87853\_JingChaoZuZhi2, Unigene87855\_JingChaoZuZhi2, Unigene87856\_JingChaoZuZhi2, Unigene87857\_JingChaoZuZhi2, Unigene87893\_JingChaoZuZhi2, Unigene87931\_JingChaoZuZhi2, Unigene87948\_JingChaoZuZhi2, Unigene87960\_JingChaoZuZhi2, Unigene87966\_JingChaoZuZhi2, Unigene87997\_JingChaoZuZhi2, Unigene87999\_JingChaoZuZhi2, Unigene87\_JingChaoZuZhi2, Unigene88016\_JingChaoZuZhi2, Unigene88045\_JingChaoZuZhi2, Unigene88047\_JingChaoZuZhi2, Unigene88068\_JingChaoZuZhi2, Unigene88090\_JingChaoZuZhi2, Unigene8809\_JingChaoZuZhi2, Unigene88135\_JingChaoZuZhi2, Unigene88157\_JingChaoZuZhi2, Unigene88179\_JingChaoZuZhi2, Unigene881\_JingChaoZuZhi2, Unigene88238\_JingChaoZuZhi2, Unigene88262\_JingChaoZuZhi2, Unigene8826\_JingChaoZuZhi2, Unigene88283\_JingChaoZuZhi2, Unigene88312\_JingChaoZuZhi2, Unigene88377\_JingChaoZuZhi2, Unigene8837\_JingChaoZuZhi2, Unigene88383\_JingChaoZuZhi2, Unigene88385\_JingChaoZuZhi2, Unigene88393\_JingChaoZuZhi2, Unigene883\_JingChaoZuZhi2, Unigene88429\_JingChaoZuZhi2, Unigene88475\_JingChaoZuZhi2, Unigene88523\_JingChaoZuZhi2, Unigene88528\_JingChaoZuZhi2, Unigene88620\_JingChaoZuZhi2, Unigene88704\_JingChaoZuZhi2, Unigene88709\_JingChaoZuZhi2, Unigene88773\_JingChaoZuZhi2, Unigene88774\_JingChaoZuZhi2, Unigene88795\_JingChaoZuZhi2, Unigene88801\_JingChaoZuZhi2, Unigene88819\_JingChaoZuZhi2, Unigene88876\_JingChaoZuZhi2, Unigene88914\_JingChaoZuZhi2, Unigene88932\_JingChaoZuZhi2, Unigene88933\_JingChaoZuZhi2, Unigene88958\_JingChaoZuZhi2, Unigene88996\_JingChaoZuZhi2, Unigene889\_JingChaoZuZhi2, Unigene89026\_JingChaoZuZhi2, Unigene89040\_JingChaoZuZhi2, Unigene89042\_JingChaoZuZhi2, Unigene89115\_JingChaoZuZhi2, Unigene89128\_JingChaoZuZhi2, Unigene89144\_JingChaoZuZhi2, Unigene89186\_JingChaoZuZhi2, Unigene89199\_JingChaoZuZhi2, Unigene89210\_JingChaoZuZhi2, Unigene89232\_JingChaoZuZhi2, Unigene89233\_JingChaoZuZhi2, Unigene89238\_JingChaoZuZhi2, Unigene89245\_JingChaoZuZhi2, Unigene89265\_JingChaoZuZhi2, Unigene89277\_JingChaoZuZhi2, Unigene89279\_JingChaoZuZhi2, Unigene89280\_JingChaoZuZhi2, Unigene89289\_JingChaoZuZhi2, Unigene89310\_JingChaoZuZhi2, Unigene89341\_JingChaoZuZhi2, Unigene89347\_JingChaoZuZhi2, Unigene89375\_JingChaoZuZhi2, Unigene89444\_JingChaoZuZhi2, Unigene89496\_JingChaoZuZhi2, Unigene89504\_JingChaoZuZhi2, Unigene8952\_JingChaoZuZhi2, Unigene89595\_JingChaoZuZhi2, Unigene89607\_JingChaoZuZhi2, Unigene89639\_JingChaoZuZhi2, Unigene89653\_JingChaoZuZhi2, Unigene89670\_JingChaoZuZhi2, Unigene8970\_JingChaoZuZhi2, Unigene89723\_JingChaoZuZhi2, Unigene89725\_JingChaoZuZhi2, Unigene8973\_JingChaoZuZhi2, Unigene89746\_JingChaoZuZhi2, Unigene89752\_JingChaoZuZhi2, Unigene89753\_JingChaoZuZhi2, Unigene89766\_JingChaoZuZhi2, Unigene89769\_JingChaoZuZhi2, Unigene89779\_JingChaoZuZhi2, Unigene89783\_JingChaoZuZhi2, Unigene89788\_JingChaoZuZhi2, Unigene89805\_JingChaoZuZhi2, Unigene89813\_JingChaoZuZhi2, Unigene8984\_JingChaoZuZhi2, Unigene89879\_JingChaoZuZhi2, Unigene89882\_JingChaoZuZhi2, Unigene89887\_JingChaoZuZhi2, Unigene89941\_JingChaoZuZhi2, Unigene89976\_JingChaoZuZhi2, Unigene90008\_JingChaoZuZhi2, Unigene90034\_JingChaoZuZhi2, Unigene90062\_JingChaoZuZhi2, Unigene9006\_JingChaoZuZhi2, Unigene90077\_JingChaoZuZhi2, Unigene90104\_JingChaoZuZhi2, Unigene90127\_JingChaoZuZhi2, Unigene90131\_JingChaoZuZhi2, Unigene90139\_JingChaoZuZhi2, Unigene90147\_JingChaoZuZhi2, Unigene90180\_JingChaoZuZhi2, Unigene90188\_JingChaoZuZhi2, Unigene90190\_JingChaoZuZhi2, Unigene90215\_JingChaoZuZhi2, Unigene90268\_JingChaoZuZhi2, Unigene90317\_JingChaoZuZhi2, Unigene90348\_JingChaoZuZhi2, Unigene90358\_JingChaoZuZhi2, Unigene90376\_JingChaoZuZhi2, Unigene90392\_JingChaoZuZhi2, Unigene90393\_JingChaoZuZhi2, Unigene90531\_JingChaoZuZhi2, Unigene90550\_JingChaoZuZhi2, Unigene90556\_JingChaoZuZhi2, Unigene90557\_JingChaoZuZhi2, Unigene90612\_JingChaoZuZhi2, Unigene90616\_JingChaoZuZhi2, Unigene90638\_JingChaoZuZhi2, Unigene90656\_JingChaoZuZhi2, Unigene90658\_JingChaoZuZhi2, Unigene90730\_JingChaoZuZhi2, Unigene90773\_JingChaoZuZhi2, Unigene90793\_JingChaoZuZhi2, Unigene90832\_JingChaoZuZhi2, Unigene90838\_JingChaoZuZhi2, Unigene90859\_JingChaoZuZhi2, Unigene90862\_JingChaoZuZhi2, Unigene90875\_JingChaoZuZhi2, Unigene90891\_JingChaoZuZhi2, Unigene90914\_JingChaoZuZhi2, Unigene90919\_JingChaoZuZhi2, Unigene90926\_JingChaoZuZhi2, Unigene90935\_JingChaoZuZhi2, Unigene90949\_JingChaoZuZhi2, Unigene90961\_JingChaoZuZhi2, Unigene9097\_JingChaoZuZhi2, Unigene90999\_JingChaoZuZhi2, Unigene91020\_JingChaoZuZhi2, Unigene91039\_JingChaoZuZhi2, Unigene91050\_JingChaoZuZhi2, Unigene91051\_JingChaoZuZhi2, Unigene91081\_JingChaoZuZhi2, Unigene91087\_JingChaoZuZhi2, Unigene91115\_JingChaoZuZhi2, Unigene91122\_JingChaoZuZhi2, Unigene91175\_JingChaoZuZhi2, Unigene91180\_JingChaoZuZhi2, Unigene91187\_JingChaoZuZhi2, Unigene91194\_JingChaoZuZhi2, Unigene91254\_JingChaoZuZhi2, Unigene91270\_JingChaoZuZhi2, Unigene9127\_JingChaoZuZhi2, Unigene9128\_JingChaoZuZhi2, Unigene91329\_JingChaoZuZhi2, Unigene91360\_JingChaoZuZhi2, Unigene91369\_JingChaoZuZhi2, Unigene9140\_JingChaoZuZhi2, Unigene91429\_JingChaoZuZhi2, Unigene91430\_JingChaoZuZhi2, Unigene91458\_JingChaoZuZhi2, Unigene91460\_JingChaoZuZhi2, Unigene91469\_JingChaoZuZhi2, Unigene91490\_JingChaoZuZhi2, Unigene91513\_JingChaoZuZhi2, Unigene91530\_JingChaoZuZhi2, Unigene91541\_JingChaoZuZhi2, Unigene91556\_JingChaoZuZhi2, Unigene91654\_JingChaoZuZhi2, Unigene91662\_JingChaoZuZhi2, Unigene91663\_JingChaoZuZhi2, Unigene9167\_JingChaoZuZhi2, Unigene91687\_JingChaoZuZhi2, Unigene91700\_JingChaoZuZhi2, Unigene91720\_JingChaoZuZhi2, Unigene91739\_JingChaoZuZhi2, Unigene91751\_JingChaoZuZhi2, Unigene91755\_JingChaoZuZhi2, Unigene91777\_JingChaoZuZhi2, Unigene91798\_JingChaoZuZhi2, Unigene91818\_JingChaoZuZhi2, Unigene91845\_JingChaoZuZhi2, Unigene91854\_JingChaoZuZhi2, Unigene91855\_JingChaoZuZhi2, Unigene91914\_JingChaoZuZhi2, Unigene91921\_JingChaoZuZhi2, Unigene91943\_JingChaoZuZhi2, Unigene91948\_JingChaoZuZhi2, Unigene91950\_JingChaoZuZhi2, Unigene91969\_JingChaoZuZhi2, Unigene91973\_JingChaoZuZhi2, Unigene91995\_JingChaoZuZhi2, Unigene92017\_JingChaoZuZhi2, Unigene92027\_JingChaoZuZhi2, Unigene92035\_JingChaoZuZhi2, Unigene92037\_JingChaoZuZhi2, Unigene92063\_JingChaoZuZhi2, Unigene9207\_JingChaoZuZhi2, Unigene92087\_JingChaoZuZhi2, Unigene92088\_JingChaoZuZhi2, Unigene92135\_JingChaoZuZhi2, Unigene92138\_JingChaoZuZhi2, Unigene92149\_JingChaoZuZhi2, Unigene92153\_JingChaoZuZhi2, Unigene92172\_JingChaoZuZhi2, Unigene92196\_JingChaoZuZhi2, Unigene92224\_JingChaoZuZhi2, Unigene92240\_JingChaoZuZhi2, Unigene92251\_JingChaoZuZhi2, Unigene92256\_JingChaoZuZhi2, Unigene92263\_JingChaoZuZhi2, Unigene92272\_JingChaoZuZhi2, Unigene92281\_JingChaoZuZhi2, Unigene92312\_JingChaoZuZhi2, Unigene92321\_JingChaoZuZhi2, Unigene92368\_JingChaoZuZhi2, Unigene92394\_JingChaoZuZhi2, Unigene92395\_JingChaoZuZhi2, Unigene92418\_JingChaoZuZhi2, Unigene92429\_JingChaoZuZhi2, Unigene92442\_JingChaoZuZhi2, Unigene92452\_JingChaoZuZhi2, Unigene92462\_JingChaoZuZhi2, Unigene92489\_JingChaoZuZhi2, Unigene92503\_JingChaoZuZhi2, Unigene92517\_JingChaoZuZhi2, Unigene92534\_JingChaoZuZhi2, Unigene92539\_JingChaoZuZhi2, Unigene92569\_JingChaoZuZhi2, Unigene9256\_JingChaoZuZhi2, Unigene92607\_JingChaoZuZhi2, Unigene92638\_JingChaoZuZhi2, Unigene92659\_JingChaoZuZhi2, Unigene92681\_JingChaoZuZhi2, Unigene92737\_JingChaoZuZhi2, Unigene92742\_JingChaoZuZhi2, Unigene92757\_JingChaoZuZhi2, Unigene92778\_JingChaoZuZhi2, Unigene92799\_JingChaoZuZhi2, Unigene92803\_JingChaoZuZhi2, Unigene92815\_JingChaoZuZhi2, Unigene92868\_JingChaoZuZhi2, Unigene92869\_JingChaoZuZhi2, Unigene92908\_JingChaoZuZhi2, Unigene92910\_JingChaoZuZhi2, Unigene92942\_JingChaoZuZhi2, Unigene92976\_JingChaoZuZhi2, Unigene92978\_JingChaoZuZhi2, Unigene92983\_JingChaoZuZhi2, Unigene92989\_JingChaoZuZhi2, Unigene92990\_JingChaoZuZhi2, Unigene93043\_JingChaoZuZhi2, Unigene93067\_JingChaoZuZhi2, Unigene93088\_JingChaoZuZhi2, Unigene93095\_JingChaoZuZhi2, Unigene93106\_JingChaoZuZhi2, Unigene93143\_JingChaoZuZhi2, Unigene9314\_JingChaoZuZhi2, Unigene9319\_JingChaoZuZhi2, Unigene93212\_JingChaoZuZhi2, Unigene93255\_JingChaoZuZhi2, Unigene93264\_JingChaoZuZhi2, Unigene93265\_JingChaoZuZhi2, Unigene93270\_JingChaoZuZhi2, Unigene93275\_JingChaoZuZhi2, Unigene93334\_JingChaoZuZhi2, Unigene93354\_JingChaoZuZhi2, Unigene93355\_JingChaoZuZhi2, Unigene93406\_JingChaoZuZhi2, Unigene93414\_JingChaoZuZhi2, Unigene93416\_JingChaoZuZhi2, Unigene93422\_JingChaoZuZhi2, Unigene93425\_JingChaoZuZhi2, Unigene93427\_JingChaoZuZhi2, Unigene93445\_JingChaoZuZhi2, Unigene93471\_JingChaoZuZhi2, Unigene93486\_JingChaoZuZhi2, Unigene93519\_JingChaoZuZhi2, Unigene93589\_JingChaoZuZhi2, Unigene93591\_JingChaoZuZhi2, Unigene93604\_JingChaoZuZhi2, Unigene93666\_JingChaoZuZhi2, Unigene93681\_JingChaoZuZhi2, Unigene93701\_JingChaoZuZhi2, Unigene93710\_JingChaoZuZhi2, Unigene93731\_JingChaoZuZhi2, Unigene93785\_JingChaoZuZhi2, Unigene93804\_JingChaoZuZhi2, Unigene93828\_JingChaoZuZhi2, Unigene93837\_JingChaoZuZhi2, Unigene93843\_JingChaoZuZhi2, Unigene93877\_JingChaoZuZhi2, Unigene93886\_JingChaoZuZhi2, Unigene93904\_JingChaoZuZhi2, Unigene93924\_JingChaoZuZhi2, Unigene93928\_JingChaoZuZhi2, Unigene93936\_JingChaoZuZhi2, Unigene93940\_JingChaoZuZhi2, Unigene93980\_JingChaoZuZhi2, Unigene93993\_JingChaoZuZhi2, Unigene94001\_JingChaoZuZhi2, Unigene94009\_JingChaoZuZhi2, Unigene94021\_JingChaoZuZhi2, Unigene94029\_JingChaoZuZhi2, Unigene94086\_JingChaoZuZhi2, Unigene94118\_JingChaoZuZhi2, Unigene94125\_JingChaoZuZhi2, Unigene94128\_JingChaoZuZhi2, Unigene94157\_JingChaoZuZhi2, Unigene9415\_JingChaoZuZhi2, Unigene94274\_JingChaoZuZhi2, Unigene94353\_JingChaoZuZhi2, Unigene94364\_JingChaoZuZhi2, Unigene94393\_JingChaoZuZhi2, Unigene94406\_JingChaoZuZhi2, Unigene94416\_JingChaoZuZhi2, Unigene94422\_JingChaoZuZhi2, Unigene94457\_JingChaoZuZhi2, Unigene94464\_JingChaoZuZhi2, Unigene94536\_JingChaoZuZhi2, Unigene94552\_JingChaoZuZhi2, Unigene94565\_JingChaoZuZhi2, Unigene94568\_JingChaoZuZhi2, Unigene94585\_JingChaoZuZhi2, Unigene94595\_JingChaoZuZhi2, Unigene94607\_JingChaoZuZhi2, Unigene94614\_JingChaoZuZhi2, Unigene9461\_JingChaoZuZhi2, Unigene94630\_JingChaoZuZhi2, Unigene94647\_JingChaoZuZhi2, Unigene94651\_JingChaoZuZhi2, Unigene9466\_JingChaoZuZhi2, Unigene94691\_JingChaoZuZhi2, Unigene94697\_JingChaoZuZhi2, Unigene94726\_JingChaoZuZhi2, Unigene94745\_JingChaoZuZhi2, Unigene94759\_JingChaoZuZhi2, Unigene94772\_JingChaoZuZhi2, Unigene94775\_JingChaoZuZhi2, Unigene94777\_JingChaoZuZhi2, Unigene94803\_JingChaoZuZhi2, Unigene94808\_JingChaoZuZhi2, Unigene94826\_JingChaoZuZhi2, Unigene94831\_JingChaoZuZhi2, Unigene94837\_JingChaoZuZhi2, Unigene94838\_JingChaoZuZhi2, Unigene94892\_JingChaoZuZhi2, Unigene94894\_JingChaoZuZhi2, Unigene94898\_JingChaoZuZhi2, Unigene94902\_JingChaoZuZhi2, Unigene94908\_JingChaoZuZhi2, Unigene94910\_JingChaoZuZhi2, Unigene94913\_JingChaoZuZhi2, Unigene94925\_JingChaoZuZhi2, Unigene94932\_JingChaoZuZhi2, Unigene94946\_JingChaoZuZhi2, Unigene94957\_JingChaoZuZhi2, Unigene94962\_JingChaoZuZhi2, Unigene95002\_JingChaoZuZhi2, Unigene95016\_JingChaoZuZhi2, Unigene95041\_JingChaoZuZhi2, Unigene95057\_JingChaoZuZhi2, Unigene95069\_JingChaoZuZhi2, Unigene95081\_JingChaoZuZhi2, Unigene95083\_JingChaoZuZhi2, Unigene95090\_JingChaoZuZhi2, Unigene95102\_JingChaoZuZhi2, Unigene95132\_JingChaoZuZhi2, Unigene95141\_JingChaoZuZhi2, Unigene95155\_JingChaoZuZhi2, Unigene95159\_JingChaoZuZhi2, Unigene95160\_JingChaoZuZhi2, Unigene95162\_JingChaoZuZhi2, Unigene95174\_JingChaoZuZhi2, Unigene95179\_JingChaoZuZhi2, Unigene95191\_JingChaoZuZhi2, Unigene95192\_JingChaoZuZhi2, Unigene95198\_JingChaoZuZhi2, Unigene9521\_JingChaoZuZhi2, Unigene95237\_JingChaoZuZhi2, Unigene9523\_JingChaoZuZhi2, Unigene95269\_JingChaoZuZhi2, Unigene9528\_JingChaoZuZhi2, Unigene95296\_JingChaoZuZhi2, Unigene95309\_JingChaoZuZhi2, Unigene95325\_JingChaoZuZhi2, Unigene95331\_JingChaoZuZhi2, Unigene95343\_JingChaoZuZhi2, Unigene95345\_JingChaoZuZhi2, Unigene95350\_JingChaoZuZhi2, Unigene95361\_JingChaoZuZhi2, Unigene95378\_JingChaoZuZhi2, Unigene95384\_JingChaoZuZhi2, Unigene95394\_JingChaoZuZhi2, Unigene95399\_JingChaoZuZhi2, Unigene95445\_JingChaoZuZhi2, Unigene95464\_JingChaoZuZhi2, Unigene95469\_JingChaoZuZhi2, Unigene95473\_JingChaoZuZhi2, Unigene95476\_JingChaoZuZhi2, Unigene95480\_JingChaoZuZhi2, Unigene95481\_JingChaoZuZhi2, Unigene95495\_JingChaoZuZhi2, Unigene95505\_JingChaoZuZhi2, Unigene95509\_JingChaoZuZhi2, Unigene9550\_JingChaoZuZhi2, Unigene95523\_JingChaoZuZhi2, Unigene95525\_JingChaoZuZhi2, Unigene95535\_JingChaoZuZhi2, Unigene95541\_JingChaoZuZhi2, Unigene95552\_JingChaoZuZhi2, Unigene95561\_JingChaoZuZhi2, Unigene95563\_JingChaoZuZhi2, Unigene95575\_JingChaoZuZhi2, Unigene95591\_JingChaoZuZhi2, Unigene95593\_JingChaoZuZhi2, Unigene95594\_JingChaoZuZhi2, Unigene95596\_JingChaoZuZhi2, Unigene95612\_JingChaoZuZhi2, Unigene95616\_JingChaoZuZhi2, Unigene95618\_JingChaoZuZhi2, Unigene95622\_JingChaoZuZhi2, Unigene95648\_JingChaoZuZhi2, Unigene95655\_JingChaoZuZhi2, Unigene95656\_JingChaoZuZhi2, Unigene95657\_JingChaoZuZhi2, Unigene95658\_JingChaoZuZhi2, Unigene95663\_JingChaoZuZhi2, Unigene95668\_JingChaoZuZhi2, Unigene95673\_JingChaoZuZhi2, Unigene95677\_JingChaoZuZhi2, Unigene95693\_JingChaoZuZhi2, Unigene9569\_JingChaoZuZhi2, Unigene95737\_JingChaoZuZhi2, Unigene95739\_JingChaoZuZhi2, Unigene95742\_JingChaoZuZhi2, Unigene95746\_JingChaoZuZhi2, Unigene95771\_JingChaoZuZhi2, Unigene95789\_JingChaoZuZhi2, Unigene95806\_JingChaoZuZhi2, Unigene9580\_JingChaoZuZhi2, Unigene95818\_JingChaoZuZhi2, Unigene95845\_JingChaoZuZhi2, Unigene95846\_JingChaoZuZhi2, Unigene95855\_JingChaoZuZhi2, Unigene95865\_JingChaoZuZhi2, Unigene95868\_JingChaoZuZhi2, Unigene95887\_JingChaoZuZhi2, Unigene95888\_JingChaoZuZhi2, Unigene95901\_JingChaoZuZhi2, Unigene95908\_JingChaoZuZhi2, Unigene95911\_JingChaoZuZhi2, Unigene95939\_JingChaoZuZhi2, Unigene95941\_JingChaoZuZhi2, Unigene95953\_JingChaoZuZhi2, Unigene95958\_JingChaoZuZhi2, Unigene95959\_JingChaoZuZhi2, Unigene95978\_JingChaoZuZhi2, Unigene95985\_JingChaoZuZhi2, Unigene95986\_JingChaoZuZhi2, Unigene95992\_JingChaoZuZhi2, Unigene95998\_JingChaoZuZhi2, Unigene96002\_JingChaoZuZhi2, Unigene96021\_JingChaoZuZhi2, Unigene96023\_JingChaoZuZhi2, Unigene96031\_JingChaoZuZhi2, Unigene96039\_JingChaoZuZhi2, Unigene96060\_JingChaoZuZhi2, Unigene96076\_JingChaoZuZhi2, Unigene96100\_JingChaoZuZhi2, Unigene96106\_JingChaoZuZhi2, Unigene96119\_JingChaoZuZhi2, Unigene96124\_JingChaoZuZhi2, Unigene96165\_JingChaoZuZhi2, Unigene96171\_JingChaoZuZhi2, Unigene96178\_JingChaoZuZhi2, Unigene96179\_JingChaoZuZhi2, Unigene96197\_JingChaoZuZhi2, Unigene96220\_JingChaoZuZhi2, Unigene96229\_JingChaoZuZhi2, Unigene96240\_JingChaoZuZhi2, Unigene96266\_JingChaoZuZhi2, Unigene96268\_JingChaoZuZhi2, Unigene96275\_JingChaoZuZhi2, Unigene96284\_JingChaoZuZhi2, Unigene9631\_JingChaoZuZhi2, Unigene9634\_JingChaoZuZhi2, Unigene963\_JingChaoZuZhi2, Unigene9748\_JingChaoZuZhi2, Unigene9750\_JingChaoZuZhi2, Unigene9753\_JingChaoZuZhi2, Unigene9836\_JingChaoZuZhi2, Unigene9973\_JingChaoZuZhi2, Unigene9977\_JingChaoZuZhi2, Unigene9\_JingChaoZuZhi2 |
| 2 | Regulation of actin cytoskeleton | Unigene10016\_JingChaoZuZhi2, Unigene10027\_JingChaoZuZhi2, Unigene10047\_JingChaoZuZhi2, Unigene10051\_JingChaoZuZhi2, Unigene10053\_JingChaoZuZhi2, Unigene10078\_JingChaoZuZhi2, Unigene10080\_JingChaoZuZhi2, Unigene10128\_JingChaoZuZhi2, Unigene10164\_JingChaoZuZhi2, Unigene1017\_JingChaoZuZhi2, Unigene10208\_JingChaoZuZhi2, Unigene10218\_JingChaoZuZhi2, Unigene10235\_JingChaoZuZhi2, Unigene10259\_JingChaoZuZhi2, Unigene10381\_JingChaoZuZhi2, Unigene10422\_JingChaoZuZhi2, Unigene10443\_JingChaoZuZhi2, Unigene10456\_JingChaoZuZhi2, Unigene10492\_JingChaoZuZhi2, Unigene10498\_JingChaoZuZhi2, Unigene10576\_JingChaoZuZhi2, Unigene10582\_JingChaoZuZhi2, Unigene1060\_JingChaoZuZhi2, Unigene10628\_JingChaoZuZhi2, Unigene10637\_JingChaoZuZhi2, Unigene10733\_JingChaoZuZhi2, Unigene10765\_JingChaoZuZhi2, Unigene10772\_JingChaoZuZhi2, Unigene10803\_JingChaoZuZhi2, Unigene10857\_JingChaoZuZhi2, Unigene10922\_JingChaoZuZhi2, Unigene10936\_JingChaoZuZhi2, Unigene10974\_JingChaoZuZhi2, Unigene10979\_JingChaoZuZhi2, Unigene10\_JingChaoZuZhi2, Unigene11112\_JingChaoZuZhi2, Unigene1111\_JingChaoZuZhi2, Unigene11211\_JingChaoZuZhi2, Unigene1121\_JingChaoZuZhi2, Unigene11240\_JingChaoZuZhi2, Unigene11258\_JingChaoZuZhi2, Unigene11446\_JingChaoZuZhi2, Unigene11475\_JingChaoZuZhi2, Unigene11545\_JingChaoZuZhi2, Unigene1155\_JingChaoZuZhi2, Unigene11569\_JingChaoZuZhi2, Unigene115\_JingChaoZuZhi2, Unigene11626\_JingChaoZuZhi2, Unigene1162\_JingChaoZuZhi2, Unigene11729\_JingChaoZuZhi2, Unigene11810\_JingChaoZuZhi2, Unigene11889\_JingChaoZuZhi2, Unigene11902\_JingChaoZuZhi2, Unigene11918\_JingChaoZuZhi2, Unigene11932\_JingChaoZuZhi2, Unigene11961\_JingChaoZuZhi2, Unigene11982\_JingChaoZuZhi2, Unigene11995\_JingChaoZuZhi2, Unigene12011\_JingChaoZuZhi2, Unigene12060\_JingChaoZuZhi2, Unigene12090\_JingChaoZuZhi2, Unigene12105\_JingChaoZuZhi2, Unigene12148\_JingChaoZuZhi2, Unigene12235\_JingChaoZuZhi2, Unigene12285\_JingChaoZuZhi2, Unigene12303\_JingChaoZuZhi2, Unigene12310\_JingChaoZuZhi2, Unigene12326\_JingChaoZuZhi2, Unigene12343\_JingChaoZuZhi2, Unigene12372\_JingChaoZuZhi2, Unigene12420\_JingChaoZuZhi2, Unigene12447\_JingChaoZuZhi2, Unigene12549\_JingChaoZuZhi2, Unigene12554\_JingChaoZuZhi2, Unigene12595\_JingChaoZuZhi2, Unigene12632\_JingChaoZuZhi2, Unigene12644\_JingChaoZuZhi2, Unigene12815\_JingChaoZuZhi2, Unigene12937\_JingChaoZuZhi2, Unigene12940\_JingChaoZuZhi2, Unigene13036\_JingChaoZuZhi2, Unigene13085\_JingChaoZuZhi2, Unigene13097\_JingChaoZuZhi2, Unigene13149\_JingChaoZuZhi2, Unigene13180\_JingChaoZuZhi2, Unigene13196\_JingChaoZuZhi2, Unigene13249\_JingChaoZuZhi2, Unigene1325\_JingChaoZuZhi2, Unigene13478\_JingChaoZuZhi2, Unigene13485\_JingChaoZuZhi2, Unigene13559\_JingChaoZuZhi2, Unigene13572\_JingChaoZuZhi2, Unigene13587\_JingChaoZuZhi2, Unigene13588\_JingChaoZuZhi2, Unigene13607\_JingChaoZuZhi2, Unigene13618\_JingChaoZuZhi2, Unigene13650\_JingChaoZuZhi2, Unigene13720\_JingChaoZuZhi2, Unigene13766\_JingChaoZuZhi2, Unigene13847\_JingChaoZuZhi2, Unigene13890\_JingChaoZuZhi2, Unigene13894\_JingChaoZuZhi2, Unigene13901\_JingChaoZuZhi2, Unigene13904\_JingChaoZuZhi2, Unigene13909\_JingChaoZuZhi2, Unigene13927\_JingChaoZuZhi2, Unigene13952\_JingChaoZuZhi2, Unigene13960\_JingChaoZuZhi2, Unigene14050\_JingChaoZuZhi2, Unigene14068\_JingChaoZuZhi2, Unigene14071\_JingChaoZuZhi2, Unigene14159\_JingChaoZuZhi2, Unigene14193\_JingChaoZuZhi2, Unigene14246\_JingChaoZuZhi2, Unigene14289\_JingChaoZuZhi2, Unigene14396\_JingChaoZuZhi2, Unigene14401\_JingChaoZuZhi2, Unigene14450\_JingChaoZuZhi2, Unigene14476\_JingChaoZuZhi2, Unigene14534\_JingChaoZuZhi2, Unigene14610\_JingChaoZuZhi2, Unigene14682\_JingChaoZuZhi2, Unigene14714\_JingChaoZuZhi2, Unigene14715\_JingChaoZuZhi2, Unigene1482\_JingChaoZuZhi2, Unigene14845\_JingChaoZuZhi2, Unigene14912\_JingChaoZuZhi2, Unigene14970\_JingChaoZuZhi2, Unigene15030\_JingChaoZuZhi2, Unigene15088\_JingChaoZuZhi2, Unigene15095\_JingChaoZuZhi2, Unigene15096\_JingChaoZuZhi2, Unigene1515\_JingChaoZuZhi2, Unigene15176\_JingChaoZuZhi2, Unigene15212\_JingChaoZuZhi2, Unigene15228\_JingChaoZuZhi2, Unigene15251\_JingChaoZuZhi2, Unigene15286\_JingChaoZuZhi2, Unigene15288\_JingChaoZuZhi2, Unigene15319\_JingChaoZuZhi2, Unigene15333\_JingChaoZuZhi2, Unigene15345\_JingChaoZuZhi2, Unigene15374\_JingChaoZuZhi2, Unigene15381\_JingChaoZuZhi2, Unigene15402\_JingChaoZuZhi2, Unigene15421\_JingChaoZuZhi2, Unigene15492\_JingChaoZuZhi2, Unigene15497\_JingChaoZuZhi2, Unigene15534\_JingChaoZuZhi2, Unigene15557\_JingChaoZuZhi2, Unigene15560\_JingChaoZuZhi2, Unigene15615\_JingChaoZuZhi2, Unigene15649\_JingChaoZuZhi2, Unigene15650\_JingChaoZuZhi2, Unigene15651\_JingChaoZuZhi2, Unigene15656\_JingChaoZuZhi2, Unigene15659\_JingChaoZuZhi2, Unigene15731\_JingChaoZuZhi2, Unigene15736\_JingChaoZuZhi2, Unigene15769\_JingChaoZuZhi2, Unigene15772\_JingChaoZuZhi2, Unigene15857\_JingChaoZuZhi2, Unigene15879\_JingChaoZuZhi2, Unigene15986\_JingChaoZuZhi2, Unigene16004\_JingChaoZuZhi2, Unigene16103\_JingChaoZuZhi2, Unigene16121\_JingChaoZuZhi2, Unigene1616\_JingChaoZuZhi2, Unigene16176\_JingChaoZuZhi2, Unigene16182\_JingChaoZuZhi2, Unigene16210\_JingChaoZuZhi2, Unigene16318\_JingChaoZuZhi2, Unigene16324\_JingChaoZuZhi2, Unigene16388\_JingChaoZuZhi2, Unigene16402\_JingChaoZuZhi2, Unigene16427\_JingChaoZuZhi2, Unigene16467\_JingChaoZuZhi2, Unigene16481\_JingChaoZuZhi2, Unigene1648\_JingChaoZuZhi2, Unigene1651\_JingChaoZuZhi2, Unigene16591\_JingChaoZuZhi2, Unigene16597\_JingChaoZuZhi2, Unigene16606\_JingChaoZuZhi2, Unigene16655\_JingChaoZuZhi2, Unigene16691\_JingChaoZuZhi2, Unigene16729\_JingChaoZuZhi2, Unigene16730\_JingChaoZuZhi2, Unigene16733\_JingChaoZuZhi2, Unigene16738\_JingChaoZuZhi2, Unigene16778\_JingChaoZuZhi2, Unigene16837\_JingChaoZuZhi2, Unigene16869\_JingChaoZuZhi2, Unigene16873\_JingChaoZuZhi2, Unigene16950\_JingChaoZuZhi2, Unigene16998\_JingChaoZuZhi2, Unigene17068\_JingChaoZuZhi2, Unigene17101\_JingChaoZuZhi2, Unigene17122\_JingChaoZuZhi2, Unigene17128\_JingChaoZuZhi2, Unigene17227\_JingChaoZuZhi2, Unigene17231\_JingChaoZuZhi2, Unigene17247\_JingChaoZuZhi2, Unigene17501\_JingChaoZuZhi2, Unigene17547\_JingChaoZuZhi2, Unigene17744\_JingChaoZuZhi2, Unigene17764\_JingChaoZuZhi2, Unigene17770\_JingChaoZuZhi2, Unigene17789\_JingChaoZuZhi2, Unigene17800\_JingChaoZuZhi2, Unigene17821\_JingChaoZuZhi2, Unigene17829\_JingChaoZuZhi2, Unigene17845\_JingChaoZuZhi2, Unigene17894\_JingChaoZuZhi2, Unigene18010\_JingChaoZuZhi2, Unigene18051\_JingChaoZuZhi2, Unigene18095\_JingChaoZuZhi2, Unigene18237\_JingChaoZuZhi2, Unigene18256\_JingChaoZuZhi2, Unigene18259\_JingChaoZuZhi2, Unigene18260\_JingChaoZuZhi2, Unigene18269\_JingChaoZuZhi2, Unigene18334\_JingChaoZuZhi2, Unigene18348\_JingChaoZuZhi2, Unigene18360\_JingChaoZuZhi2, Unigene18402\_JingChaoZuZhi2, Unigene18426\_JingChaoZuZhi2, Unigene18438\_JingChaoZuZhi2, Unigene18441\_JingChaoZuZhi2, Unigene18491\_JingChaoZuZhi2, Unigene18505\_JingChaoZuZhi2, Unigene18545\_JingChaoZuZhi2, Unigene18611\_JingChaoZuZhi2, Unigene18662\_JingChaoZuZhi2, Unigene18672\_JingChaoZuZhi2, Unigene18685\_JingChaoZuZhi2, Unigene18699\_JingChaoZuZhi2, Unigene18790\_JingChaoZuZhi2, Unigene187\_JingChaoZuZhi2, Unigene18835\_JingChaoZuZhi2, Unigene18892\_JingChaoZuZhi2, Unigene18932\_JingChaoZuZhi2, Unigene19001\_JingChaoZuZhi2, Unigene19004\_JingChaoZuZhi2, Unigene19021\_JingChaoZuZhi2, Unigene19086\_JingChaoZuZhi2, Unigene19137\_JingChaoZuZhi2, Unigene19193\_JingChaoZuZhi2, Unigene19204\_JingChaoZuZhi2, Unigene19223\_JingChaoZuZhi2, Unigene19224\_JingChaoZuZhi2, Unigene19319\_JingChaoZuZhi2, Unigene19365\_JingChaoZuZhi2, Unigene19391\_JingChaoZuZhi2, Unigene19396\_JingChaoZuZhi2, Unigene19404\_JingChaoZuZhi2, Unigene19465\_JingChaoZuZhi2, Unigene19466\_JingChaoZuZhi2, Unigene19512\_JingChaoZuZhi2, Unigene1951\_JingChaoZuZhi2, Unigene19533\_JingChaoZuZhi2, Unigene19559\_JingChaoZuZhi2, Unigene19701\_JingChaoZuZhi2, Unigene19750\_JingChaoZuZhi2, Unigene19883\_JingChaoZuZhi2, Unigene19979\_JingChaoZuZhi2, Unigene20025\_JingChaoZuZhi2, Unigene20030\_JingChaoZuZhi2, Unigene20049\_JingChaoZuZhi2, Unigene20083\_JingChaoZuZhi2, Unigene20159\_JingChaoZuZhi2, Unigene20188\_JingChaoZuZhi2, Unigene201\_JingChaoZuZhi2, Unigene20220\_JingChaoZuZhi2, Unigene20223\_JingChaoZuZhi2, Unigene20239\_JingChaoZuZhi2, Unigene20356\_JingChaoZuZhi2, Unigene20373\_JingChaoZuZhi2, Unigene20394\_JingChaoZuZhi2, Unigene20410\_JingChaoZuZhi2, Unigene20447\_JingChaoZuZhi2, Unigene20464\_JingChaoZuZhi2, Unigene20468\_JingChaoZuZhi2, Unigene20474\_JingChaoZuZhi2, Unigene20479\_JingChaoZuZhi2, Unigene20557\_JingChaoZuZhi2, Unigene20580\_JingChaoZuZhi2, Unigene20608\_JingChaoZuZhi2, Unigene20629\_JingChaoZuZhi2, Unigene20720\_JingChaoZuZhi2, Unigene20727\_JingChaoZuZhi2, Unigene20758\_JingChaoZuZhi2, Unigene20794\_JingChaoZuZhi2, Unigene20820\_JingChaoZuZhi2, Unigene20837\_JingChaoZuZhi2, Unigene2092\_JingChaoZuZhi2, Unigene20943\_JingChaoZuZhi2, Unigene21057\_JingChaoZuZhi2, Unigene21118\_JingChaoZuZhi2, Unigene21137\_JingChaoZuZhi2, Unigene2115\_JingChaoZuZhi2, Unigene21225\_JingChaoZuZhi2, Unigene21311\_JingChaoZuZhi2, Unigene21336\_JingChaoZuZhi2, Unigene21487\_JingChaoZuZhi2, Unigene21513\_JingChaoZuZhi2, Unigene21536\_JingChaoZuZhi2, Unigene21537\_JingChaoZuZhi2, Unigene21543\_JingChaoZuZhi2, Unigene21636\_JingChaoZuZhi2, Unigene21713\_JingChaoZuZhi2, Unigene21809\_JingChaoZuZhi2, Unigene21818\_JingChaoZuZhi2, Unigene21860\_JingChaoZuZhi2, Unigene21898\_JingChaoZuZhi2, Unigene21932\_JingChaoZuZhi2, Unigene21933\_JingChaoZuZhi2, Unigene21936\_JingChaoZuZhi2, Unigene22024\_JingChaoZuZhi2, Unigene22045\_JingChaoZuZhi2, Unigene22055\_JingChaoZuZhi2, Unigene22091\_JingChaoZuZhi2, Unigene22119\_JingChaoZuZhi2, Unigene22128\_JingChaoZuZhi2, Unigene22168\_JingChaoZuZhi2, Unigene22174\_JingChaoZuZhi2, Unigene22183\_JingChaoZuZhi2, Unigene22189\_JingChaoZuZhi2, Unigene22216\_JingChaoZuZhi2, Unigene22220\_JingChaoZuZhi2, Unigene22229\_JingChaoZuZhi2, Unigene22243\_JingChaoZuZhi2, Unigene22251\_JingChaoZuZhi2, Unigene22262\_JingChaoZuZhi2, Unigene2226\_JingChaoZuZhi2, Unigene22277\_JingChaoZuZhi2, Unigene22329\_JingChaoZuZhi2, Unigene22372\_JingChaoZuZhi2, Unigene22379\_JingChaoZuZhi2, Unigene22414\_JingChaoZuZhi2, Unigene22455\_JingChaoZuZhi2, Unigene22482\_JingChaoZuZhi2, Unigene22485\_JingChaoZuZhi2, Unigene22487\_JingChaoZuZhi2, Unigene22517\_JingChaoZuZhi2, Unigene22551\_JingChaoZuZhi2, Unigene22558\_JingChaoZuZhi2, Unigene22586\_JingChaoZuZhi2, Unigene2258\_JingChaoZuZhi2, Unigene22644\_JingChaoZuZhi2, Unigene22652\_JingChaoZuZhi2, Unigene22674\_JingChaoZuZhi2, Unigene22680\_JingChaoZuZhi2, Unigene22737\_JingChaoZuZhi2, Unigene22804\_JingChaoZuZhi2, Unigene22878\_JingChaoZuZhi2, Unigene22880\_JingChaoZuZhi2, Unigene22895\_JingChaoZuZhi2, Unigene2289\_JingChaoZuZhi2, Unigene22909\_JingChaoZuZhi2, Unigene22928\_JingChaoZuZhi2, Unigene2308\_JingChaoZuZhi2, Unigene23164\_JingChaoZuZhi2, Unigene23447\_JingChaoZuZhi2, Unigene23924\_JingChaoZuZhi2, Unigene23978\_JingChaoZuZhi2, Unigene24392\_JingChaoZuZhi2, Unigene24425\_JingChaoZuZhi2, Unigene2445\_JingChaoZuZhi2, Unigene2456\_JingChaoZuZhi2, Unigene24600\_JingChaoZuZhi2, Unigene2465\_JingChaoZuZhi2, Unigene2503\_JingChaoZuZhi2, Unigene2510\_JingChaoZuZhi2, Unigene25164\_JingChaoZuZhi2, Unigene2517\_JingChaoZuZhi2, Unigene25350\_JingChaoZuZhi2, Unigene2539\_JingChaoZuZhi2, Unigene2544\_JingChaoZuZhi2, Unigene25693\_JingChaoZuZhi2, Unigene25962\_JingChaoZuZhi2, Unigene26086\_JingChaoZuZhi2, Unigene26987\_JingChaoZuZhi2, Unigene27063\_JingChaoZuZhi2, Unigene2728\_JingChaoZuZhi2, Unigene2790\_JingChaoZuZhi2, Unigene28064\_JingChaoZuZhi2, Unigene28243\_JingChaoZuZhi2, Unigene2857\_JingChaoZuZhi2, Unigene28793\_JingChaoZuZhi2, Unigene28926\_JingChaoZuZhi2, Unigene29005\_JingChaoZuZhi2, Unigene2901\_JingChaoZuZhi2, Unigene29183\_JingChaoZuZhi2, Unigene29201\_JingChaoZuZhi2, Unigene29312\_JingChaoZuZhi2, Unigene2968\_JingChaoZuZhi2, Unigene30281\_JingChaoZuZhi2, Unigene30593\_JingChaoZuZhi2, Unigene30726\_JingChaoZuZhi2, Unigene30787\_JingChaoZuZhi2, Unigene30851\_JingChaoZuZhi2, Unigene30852\_JingChaoZuZhi2, Unigene31234\_JingChaoZuZhi2, Unigene3131\_JingChaoZuZhi2, Unigene31612\_JingChaoZuZhi2, Unigene31660\_JingChaoZuZhi2, Unigene31912\_JingChaoZuZhi2, Unigene31918\_JingChaoZuZhi2, Unigene3191\_JingChaoZuZhi2, Unigene3202\_JingChaoZuZhi2, Unigene3235\_JingChaoZuZhi2, Unigene32373\_JingChaoZuZhi2, Unigene32417\_JingChaoZuZhi2, Unigene324\_JingChaoZuZhi2, Unigene3259\_JingChaoZuZhi2, Unigene3292\_JingChaoZuZhi2, Unigene33044\_JingChaoZuZhi2, Unigene33173\_JingChaoZuZhi2, Unigene3321\_JingChaoZuZhi2, Unigene3352\_JingChaoZuZhi2, Unigene33683\_JingChaoZuZhi2, Unigene3388\_JingChaoZuZhi2, Unigene34142\_JingChaoZuZhi2, Unigene34180\_JingChaoZuZhi2, Unigene34463\_JingChaoZuZhi2, Unigene34660\_JingChaoZuZhi2, Unigene34785\_JingChaoZuZhi2, Unigene3493\_JingChaoZuZhi2, Unigene35016\_JingChaoZuZhi2, Unigene35080\_JingChaoZuZhi2, Unigene35243\_JingChaoZuZhi2, Unigene3550\_JingChaoZuZhi2, Unigene35698\_JingChaoZuZhi2, Unigene35927\_JingChaoZuZhi2, Unigene36084\_JingChaoZuZhi2, Unigene36257\_JingChaoZuZhi2, Unigene36342\_JingChaoZuZhi2, Unigene36361\_JingChaoZuZhi2, Unigene3646\_JingChaoZuZhi2, Unigene36511\_JingChaoZuZhi2, Unigene36631\_JingChaoZuZhi2, Unigene3671\_JingChaoZuZhi2, Unigene3693\_JingChaoZuZhi2, Unigene36974\_JingChaoZuZhi2, Unigene36976\_JingChaoZuZhi2, Unigene37029\_JingChaoZuZhi2, Unigene37164\_JingChaoZuZhi2, Unigene37176\_JingChaoZuZhi2, Unigene37739\_JingChaoZuZhi2, Unigene37818\_JingChaoZuZhi2, Unigene37958\_JingChaoZuZhi2, Unigene3813\_JingChaoZuZhi2, Unigene3854\_JingChaoZuZhi2, Unigene39102\_JingChaoZuZhi2, Unigene3912\_JingChaoZuZhi2, Unigene39216\_JingChaoZuZhi2, Unigene39661\_JingChaoZuZhi2, Unigene39876\_JingChaoZuZhi2, Unigene40143\_JingChaoZuZhi2, Unigene4015\_JingChaoZuZhi2, Unigene40200\_JingChaoZuZhi2, Unigene40281\_JingChaoZuZhi2, Unigene4029\_JingChaoZuZhi2, Unigene4044\_JingChaoZuZhi2, Unigene40919\_JingChaoZuZhi2, Unigene41040\_JingChaoZuZhi2, Unigene41118\_JingChaoZuZhi2, Unigene41148\_JingChaoZuZhi2, Unigene411\_JingChaoZuZhi2, Unigene41213\_JingChaoZuZhi2, Unigene41571\_JingChaoZuZhi2, Unigene41700\_JingChaoZuZhi2, Unigene4186\_JingChaoZuZhi2, Unigene42032\_JingChaoZuZhi2, Unigene42146\_JingChaoZuZhi2, Unigene42248\_JingChaoZuZhi2, Unigene42256\_JingChaoZuZhi2, Unigene42321\_JingChaoZuZhi2, Unigene4254\_JingChaoZuZhi2, Unigene42565\_JingChaoZuZhi2, Unigene43041\_JingChaoZuZhi2, Unigene43128\_JingChaoZuZhi2, Unigene43147\_JingChaoZuZhi2, Unigene43169\_JingChaoZuZhi2, Unigene43249\_JingChaoZuZhi2, Unigene43260\_JingChaoZuZhi2, Unigene43322\_JingChaoZuZhi2, Unigene4337\_JingChaoZuZhi2, Unigene43483\_JingChaoZuZhi2, Unigene43512\_JingChaoZuZhi2, Unigene43574\_JingChaoZuZhi2, Unigene44190\_JingChaoZuZhi2, Unigene44454\_JingChaoZuZhi2, Unigene44516\_JingChaoZuZhi2, Unigene44731\_JingChaoZuZhi2, Unigene45073\_JingChaoZuZhi2, Unigene45134\_JingChaoZuZhi2, Unigene45367\_JingChaoZuZhi2, Unigene45449\_JingChaoZuZhi2, Unigene4547\_JingChaoZuZhi2, Unigene45593\_JingChaoZuZhi2, Unigene45622\_JingChaoZuZhi2, Unigene45670\_JingChaoZuZhi2, Unigene45760\_JingChaoZuZhi2, Unigene45782\_JingChaoZuZhi2, Unigene45901\_JingChaoZuZhi2, Unigene4599\_JingChaoZuZhi2, Unigene45\_JingChaoZuZhi2, Unigene46046\_JingChaoZuZhi2, Unigene46256\_JingChaoZuZhi2, Unigene46407\_JingChaoZuZhi2, Unigene46413\_JingChaoZuZhi2, Unigene46755\_JingChaoZuZhi2, Unigene4713\_JingChaoZuZhi2, Unigene47461\_JingChaoZuZhi2, Unigene47578\_JingChaoZuZhi2, Unigene4758\_JingChaoZuZhi2, Unigene475\_JingChaoZuZhi2, Unigene47749\_JingChaoZuZhi2, Unigene47949\_JingChaoZuZhi2, Unigene48280\_JingChaoZuZhi2, Unigene4829\_JingChaoZuZhi2, Unigene48309\_JingChaoZuZhi2, Unigene4873\_JingChaoZuZhi2, Unigene48751\_JingChaoZuZhi2, Unigene4878\_JingChaoZuZhi2, Unigene48843\_JingChaoZuZhi2, Unigene48853\_JingChaoZuZhi2, Unigene4886\_JingChaoZuZhi2, Unigene48962\_JingChaoZuZhi2, Unigene4935\_JingChaoZuZhi2, Unigene49387\_JingChaoZuZhi2, Unigene49798\_JingChaoZuZhi2, Unigene4985\_JingChaoZuZhi2, Unigene49973\_JingChaoZuZhi2, Unigene49\_JingChaoZuZhi2, Unigene50540\_JingChaoZuZhi2, Unigene50563\_JingChaoZuZhi2, Unigene50668\_JingChaoZuZhi2, Unigene50673\_JingChaoZuZhi2, Unigene5069\_JingChaoZuZhi2, Unigene50713\_JingChaoZuZhi2, Unigene5072\_JingChaoZuZhi2, Unigene50744\_JingChaoZuZhi2, Unigene51035\_JingChaoZuZhi2, Unigene5108\_JingChaoZuZhi2, Unigene51090\_JingChaoZuZhi2, Unigene51115\_JingChaoZuZhi2, Unigene5119\_JingChaoZuZhi2, Unigene5125\_JingChaoZuZhi2, Unigene5126\_JingChaoZuZhi2, Unigene51467\_JingChaoZuZhi2, Unigene51496\_JingChaoZuZhi2, Unigene51567\_JingChaoZuZhi2, Unigene51619\_JingChaoZuZhi2, Unigene51705\_JingChaoZuZhi2, Unigene51848\_JingChaoZuZhi2, Unigene51895\_JingChaoZuZhi2, Unigene51966\_JingChaoZuZhi2, Unigene5196\_JingChaoZuZhi2, Unigene52035\_JingChaoZuZhi2, Unigene5204\_JingChaoZuZhi2, Unigene52069\_JingChaoZuZhi2, Unigene52073\_JingChaoZuZhi2, Unigene52100\_JingChaoZuZhi2, Unigene52125\_JingChaoZuZhi2, Unigene52456\_JingChaoZuZhi2, Unigene524\_JingChaoZuZhi2, Unigene52507\_JingChaoZuZhi2, Unigene52638\_JingChaoZuZhi2, Unigene5265\_JingChaoZuZhi2, Unigene52727\_JingChaoZuZhi2, Unigene5298\_JingChaoZuZhi2, Unigene53131\_JingChaoZuZhi2, Unigene53138\_JingChaoZuZhi2, Unigene5335\_JingChaoZuZhi2, Unigene5341\_JingChaoZuZhi2, Unigene53484\_JingChaoZuZhi2, Unigene53500\_JingChaoZuZhi2, Unigene53524\_JingChaoZuZhi2, Unigene53751\_JingChaoZuZhi2, Unigene53917\_JingChaoZuZhi2, Unigene5394\_JingChaoZuZhi2, Unigene54081\_JingChaoZuZhi2, Unigene5418\_JingChaoZuZhi2, Unigene54470\_JingChaoZuZhi2, Unigene54744\_JingChaoZuZhi2, Unigene54918\_JingChaoZuZhi2, Unigene54974\_JingChaoZuZhi2, Unigene54980\_JingChaoZuZhi2, Unigene54986\_JingChaoZuZhi2, Unigene55009\_JingChaoZuZhi2, Unigene55019\_JingChaoZuZhi2, Unigene55239\_JingChaoZuZhi2, Unigene55326\_JingChaoZuZhi2, Unigene55385\_JingChaoZuZhi2, Unigene55764\_JingChaoZuZhi2, Unigene55839\_JingChaoZuZhi2, Unigene56421\_JingChaoZuZhi2, Unigene56456\_JingChaoZuZhi2, Unigene56560\_JingChaoZuZhi2, Unigene56660\_JingChaoZuZhi2, Unigene56709\_JingChaoZuZhi2, Unigene56897\_JingChaoZuZhi2, Unigene57200\_JingChaoZuZhi2, Unigene5723\_JingChaoZuZhi2, Unigene57240\_JingChaoZuZhi2, Unigene57327\_JingChaoZuZhi2, Unigene57488\_JingChaoZuZhi2, Unigene57597\_JingChaoZuZhi2, Unigene57751\_JingChaoZuZhi2, Unigene5782\_JingChaoZuZhi2, Unigene5796\_JingChaoZuZhi2, Unigene57988\_JingChaoZuZhi2, Unigene5809\_JingChaoZuZhi2, Unigene58169\_JingChaoZuZhi2, Unigene58322\_JingChaoZuZhi2, Unigene58474\_JingChaoZuZhi2, Unigene58675\_JingChaoZuZhi2, Unigene5896\_JingChaoZuZhi2, Unigene59101\_JingChaoZuZhi2, Unigene59146\_JingChaoZuZhi2, Unigene5914\_JingChaoZuZhi2, Unigene59265\_JingChaoZuZhi2, Unigene59336\_JingChaoZuZhi2, Unigene59433\_JingChaoZuZhi2, Unigene5946\_JingChaoZuZhi2, Unigene59684\_JingChaoZuZhi2, Unigene59933\_JingChaoZuZhi2, Unigene60121\_JingChaoZuZhi2, Unigene60180\_JingChaoZuZhi2, Unigene6028\_JingChaoZuZhi2, Unigene60551\_JingChaoZuZhi2, Unigene60586\_JingChaoZuZhi2, Unigene60607\_JingChaoZuZhi2, Unigene6072\_JingChaoZuZhi2, Unigene60769\_JingChaoZuZhi2, Unigene60806\_JingChaoZuZhi2, Unigene60930\_JingChaoZuZhi2, Unigene60990\_JingChaoZuZhi2, Unigene61110\_JingChaoZuZhi2, Unigene61244\_JingChaoZuZhi2, Unigene613\_JingChaoZuZhi2, Unigene61474\_JingChaoZuZhi2, Unigene61526\_JingChaoZuZhi2, Unigene6158\_JingChaoZuZhi2, Unigene6170\_JingChaoZuZhi2, Unigene6171\_JingChaoZuZhi2, Unigene6174\_JingChaoZuZhi2, Unigene61879\_JingChaoZuZhi2, Unigene61931\_JingChaoZuZhi2, Unigene62100\_JingChaoZuZhi2, Unigene6215\_JingChaoZuZhi2, Unigene62238\_JingChaoZuZhi2, Unigene62257\_JingChaoZuZhi2, Unigene6233\_JingChaoZuZhi2, Unigene6239\_JingChaoZuZhi2, Unigene62431\_JingChaoZuZhi2, Unigene62545\_JingChaoZuZhi2, Unigene62606\_JingChaoZuZhi2, Unigene62695\_JingChaoZuZhi2, Unigene62716\_JingChaoZuZhi2, Unigene62786\_JingChaoZuZhi2, Unigene6291\_JingChaoZuZhi2, Unigene63052\_JingChaoZuZhi2, Unigene63145\_JingChaoZuZhi2, Unigene63384\_JingChaoZuZhi2, Unigene63424\_JingChaoZuZhi2, Unigene63584\_JingChaoZuZhi2, Unigene63604\_JingChaoZuZhi2, Unigene63615\_JingChaoZuZhi2, Unigene6361\_JingChaoZuZhi2, Unigene63812\_JingChaoZuZhi2, Unigene6382\_JingChaoZuZhi2, Unigene63873\_JingChaoZuZhi2, Unigene63925\_JingChaoZuZhi2, Unigene64208\_JingChaoZuZhi2, Unigene6438\_JingChaoZuZhi2, Unigene64416\_JingChaoZuZhi2, Unigene64452\_JingChaoZuZhi2, Unigene64458\_JingChaoZuZhi2, Unigene64495\_JingChaoZuZhi2, Unigene64650\_JingChaoZuZhi2, Unigene64678\_JingChaoZuZhi2, Unigene6467\_JingChaoZuZhi2, Unigene64692\_JingChaoZuZhi2, Unigene64697\_JingChaoZuZhi2, Unigene64838\_JingChaoZuZhi2, Unigene64843\_JingChaoZuZhi2, Unigene6493\_JingChaoZuZhi2, Unigene6498\_JingChaoZuZhi2, Unigene65002\_JingChaoZuZhi2, Unigene65022\_JingChaoZuZhi2, Unigene65129\_JingChaoZuZhi2, Unigene65150\_JingChaoZuZhi2, Unigene65187\_JingChaoZuZhi2, Unigene65326\_JingChaoZuZhi2, Unigene65375\_JingChaoZuZhi2, Unigene6539\_JingChaoZuZhi2, Unigene65648\_JingChaoZuZhi2, Unigene65773\_JingChaoZuZhi2, Unigene66013\_JingChaoZuZhi2, Unigene6605\_JingChaoZuZhi2, Unigene66077\_JingChaoZuZhi2, Unigene66087\_JingChaoZuZhi2, Unigene66231\_JingChaoZuZhi2, Unigene66253\_JingChaoZuZhi2, Unigene6634\_JingChaoZuZhi2, Unigene66453\_JingChaoZuZhi2, Unigene6651\_JingChaoZuZhi2, Unigene66559\_JingChaoZuZhi2, Unigene66590\_JingChaoZuZhi2, Unigene6676\_JingChaoZuZhi2, Unigene66813\_JingChaoZuZhi2, Unigene66913\_JingChaoZuZhi2, Unigene66986\_JingChaoZuZhi2, Unigene67005\_JingChaoZuZhi2, Unigene67063\_JingChaoZuZhi2, Unigene67070\_JingChaoZuZhi2, Unigene67081\_JingChaoZuZhi2, Unigene67191\_JingChaoZuZhi2, Unigene67249\_JingChaoZuZhi2, Unigene67279\_JingChaoZuZhi2, Unigene67542\_JingChaoZuZhi2, Unigene67572\_JingChaoZuZhi2, Unigene6769\_JingChaoZuZhi2, Unigene67777\_JingChaoZuZhi2, Unigene67780\_JingChaoZuZhi2, Unigene67817\_JingChaoZuZhi2, Unigene67847\_JingChaoZuZhi2, Unigene67958\_JingChaoZuZhi2, Unigene6796\_JingChaoZuZhi2, Unigene6797\_JingChaoZuZhi2, Unigene68056\_JingChaoZuZhi2, Unigene68108\_JingChaoZuZhi2, Unigene68134\_JingChaoZuZhi2, Unigene68139\_JingChaoZuZhi2, Unigene68159\_JingChaoZuZhi2, Unigene6815\_JingChaoZuZhi2, Unigene6828\_JingChaoZuZhi2, Unigene68527\_JingChaoZuZhi2, Unigene6864\_JingChaoZuZhi2, Unigene6865\_JingChaoZuZhi2, Unigene68672\_JingChaoZuZhi2, Unigene6872\_JingChaoZuZhi2, Unigene68845\_JingChaoZuZhi2, Unigene69005\_JingChaoZuZhi2, Unigene69092\_JingChaoZuZhi2, Unigene69100\_JingChaoZuZhi2, Unigene69141\_JingChaoZuZhi2, Unigene6914\_JingChaoZuZhi2, Unigene69221\_JingChaoZuZhi2, Unigene69386\_JingChaoZuZhi2, Unigene69562\_JingChaoZuZhi2, Unigene69565\_JingChaoZuZhi2, Unigene69657\_JingChaoZuZhi2, Unigene69658\_JingChaoZuZhi2, Unigene69775\_JingChaoZuZhi2, Unigene69786\_JingChaoZuZhi2, Unigene70045\_JingChaoZuZhi2, Unigene70063\_JingChaoZuZhi2, Unigene70068\_JingChaoZuZhi2, Unigene70100\_JingChaoZuZhi2, Unigene7016\_JingChaoZuZhi2, Unigene70329\_JingChaoZuZhi2, Unigene7032\_JingChaoZuZhi2, Unigene70373\_JingChaoZuZhi2, Unigene70505\_JingChaoZuZhi2, Unigene70522\_JingChaoZuZhi2, Unigene70549\_JingChaoZuZhi2, Unigene70970\_JingChaoZuZhi2, Unigene71043\_JingChaoZuZhi2, Unigene7116\_JingChaoZuZhi2, Unigene7118\_JingChaoZuZhi2, Unigene71213\_JingChaoZuZhi2, Unigene71241\_JingChaoZuZhi2, Unigene71247\_JingChaoZuZhi2, Unigene71385\_JingChaoZuZhi2, Unigene71486\_JingChaoZuZhi2, Unigene7151\_JingChaoZuZhi2, Unigene71546\_JingChaoZuZhi2, Unigene71592\_JingChaoZuZhi2, Unigene71662\_JingChaoZuZhi2, Unigene71755\_JingChaoZuZhi2, Unigene71774\_JingChaoZuZhi2, Unigene71808\_JingChaoZuZhi2, Unigene71939\_JingChaoZuZhi2, Unigene7206\_JingChaoZuZhi2, Unigene72249\_JingChaoZuZhi2, Unigene72304\_JingChaoZuZhi2, Unigene72334\_JingChaoZuZhi2, Unigene72351\_JingChaoZuZhi2, Unigene72503\_JingChaoZuZhi2, Unigene72521\_JingChaoZuZhi2, Unigene7268\_JingChaoZuZhi2, Unigene72708\_JingChaoZuZhi2, Unigene72832\_JingChaoZuZhi2, Unigene73257\_JingChaoZuZhi2, Unigene7326\_JingChaoZuZhi2, Unigene73446\_JingChaoZuZhi2, Unigene73475\_JingChaoZuZhi2, Unigene73550\_JingChaoZuZhi2, Unigene73606\_JingChaoZuZhi2, Unigene73620\_JingChaoZuZhi2, Unigene7373\_JingChaoZuZhi2, Unigene73817\_JingChaoZuZhi2, Unigene73887\_JingChaoZuZhi2, Unigene73919\_JingChaoZuZhi2, Unigene73983\_JingChaoZuZhi2, Unigene7398\_JingChaoZuZhi2, Unigene74050\_JingChaoZuZhi2, Unigene741\_JingChaoZuZhi2, Unigene74212\_JingChaoZuZhi2, Unigene74221\_JingChaoZuZhi2, Unigene74259\_JingChaoZuZhi2, Unigene74298\_JingChaoZuZhi2, Unigene74474\_JingChaoZuZhi2, Unigene74475\_JingChaoZuZhi2, Unigene74599\_JingChaoZuZhi2, Unigene74719\_JingChaoZuZhi2, Unigene74730\_JingChaoZuZhi2, Unigene74854\_JingChaoZuZhi2, Unigene7489\_JingChaoZuZhi2, Unigene75009\_JingChaoZuZhi2, Unigene7508\_JingChaoZuZhi2, Unigene75112\_JingChaoZuZhi2, Unigene75119\_JingChaoZuZhi2, Unigene75146\_JingChaoZuZhi2, Unigene75167\_JingChaoZuZhi2, Unigene75317\_JingChaoZuZhi2, Unigene75415\_JingChaoZuZhi2, Unigene75491\_JingChaoZuZhi2, Unigene75641\_JingChaoZuZhi2, Unigene75781\_JingChaoZuZhi2, Unigene75788\_JingChaoZuZhi2, Unigene75840\_JingChaoZuZhi2, Unigene75859\_JingChaoZuZhi2, Unigene75888\_JingChaoZuZhi2, Unigene75902\_JingChaoZuZhi2, Unigene75946\_JingChaoZuZhi2, Unigene75989\_JingChaoZuZhi2, Unigene76002\_JingChaoZuZhi2, Unigene76069\_JingChaoZuZhi2, Unigene76104\_JingChaoZuZhi2, Unigene76111\_JingChaoZuZhi2, Unigene76269\_JingChaoZuZhi2, Unigene76302\_JingChaoZuZhi2, Unigene76303\_JingChaoZuZhi2, Unigene7634\_JingChaoZuZhi2, Unigene76515\_JingChaoZuZhi2, Unigene76536\_JingChaoZuZhi2, Unigene7663\_JingChaoZuZhi2, Unigene76776\_JingChaoZuZhi2, Unigene76785\_JingChaoZuZhi2, Unigene76819\_JingChaoZuZhi2, Unigene76859\_JingChaoZuZhi2, Unigene76871\_JingChaoZuZhi2, Unigene76917\_JingChaoZuZhi2, Unigene77054\_JingChaoZuZhi2, Unigene77061\_JingChaoZuZhi2, Unigene77071\_JingChaoZuZhi2, Unigene77200\_JingChaoZuZhi2, Unigene77406\_JingChaoZuZhi2, Unigene77413\_JingChaoZuZhi2, Unigene77510\_JingChaoZuZhi2, Unigene77520\_JingChaoZuZhi2, Unigene77528\_JingChaoZuZhi2, Unigene77712\_JingChaoZuZhi2, Unigene7794\_JingChaoZuZhi2, Unigene77954\_JingChaoZuZhi2, Unigene77955\_JingChaoZuZhi2, Unigene78009\_JingChaoZuZhi2, Unigene78225\_JingChaoZuZhi2, Unigene78288\_JingChaoZuZhi2, Unigene78325\_JingChaoZuZhi2, Unigene78361\_JingChaoZuZhi2, Unigene78390\_JingChaoZuZhi2, Unigene78410\_JingChaoZuZhi2, Unigene78490\_JingChaoZuZhi2, Unigene7850\_JingChaoZuZhi2, Unigene78521\_JingChaoZuZhi2, Unigene78555\_JingChaoZuZhi2, Unigene78574\_JingChaoZuZhi2, Unigene78636\_JingChaoZuZhi2, Unigene78909\_JingChaoZuZhi2, Unigene79030\_JingChaoZuZhi2, Unigene79058\_JingChaoZuZhi2, Unigene79118\_JingChaoZuZhi2, Unigene7911\_JingChaoZuZhi2, Unigene79141\_JingChaoZuZhi2, Unigene79274\_JingChaoZuZhi2, Unigene79358\_JingChaoZuZhi2, Unigene79411\_JingChaoZuZhi2, Unigene79433\_JingChaoZuZhi2, Unigene79443\_JingChaoZuZhi2, Unigene79568\_JingChaoZuZhi2, Unigene79653\_JingChaoZuZhi2, Unigene79681\_JingChaoZuZhi2, Unigene79726\_JingChaoZuZhi2, Unigene79789\_JingChaoZuZhi2, Unigene79824\_JingChaoZuZhi2, Unigene79864\_JingChaoZuZhi2, Unigene79871\_JingChaoZuZhi2, Unigene7987\_JingChaoZuZhi2, Unigene79882\_JingChaoZuZhi2, Unigene79940\_JingChaoZuZhi2, Unigene8006\_JingChaoZuZhi2, Unigene80095\_JingChaoZuZhi2, Unigene80143\_JingChaoZuZhi2, Unigene80204\_JingChaoZuZhi2, Unigene80212\_JingChaoZuZhi2, Unigene80215\_JingChaoZuZhi2, Unigene80246\_JingChaoZuZhi2, Unigene80251\_JingChaoZuZhi2, Unigene80331\_JingChaoZuZhi2, Unigene80403\_JingChaoZuZhi2, Unigene80619\_JingChaoZuZhi2, Unigene80893\_JingChaoZuZhi2, Unigene80980\_JingChaoZuZhi2, Unigene8099\_JingChaoZuZhi2, Unigene81055\_JingChaoZuZhi2, Unigene81075\_JingChaoZuZhi2, Unigene81145\_JingChaoZuZhi2, Unigene81167\_JingChaoZuZhi2, Unigene81253\_JingChaoZuZhi2, Unigene81271\_JingChaoZuZhi2, Unigene81327\_JingChaoZuZhi2, Unigene81372\_JingChaoZuZhi2, Unigene81434\_JingChaoZuZhi2, Unigene81437\_JingChaoZuZhi2, Unigene81440\_JingChaoZuZhi2, Unigene81443\_JingChaoZuZhi2, Unigene81450\_JingChaoZuZhi2, Unigene81463\_JingChaoZuZhi2, Unigene81471\_JingChaoZuZhi2, Unigene81561\_JingChaoZuZhi2, Unigene81592\_JingChaoZuZhi2, Unigene81598\_JingChaoZuZhi2, Unigene81599\_JingChaoZuZhi2, Unigene81686\_JingChaoZuZhi2, Unigene81719\_JingChaoZuZhi2, Unigene81757\_JingChaoZuZhi2, Unigene81780\_JingChaoZuZhi2, Unigene81803\_JingChaoZuZhi2, Unigene81825\_JingChaoZuZhi2, Unigene81903\_JingChaoZuZhi2, Unigene81904\_JingChaoZuZhi2, Unigene8191\_JingChaoZuZhi2, Unigene8192\_JingChaoZuZhi2, Unigene82047\_JingChaoZuZhi2, Unigene82061\_JingChaoZuZhi2, Unigene82070\_JingChaoZuZhi2, Unigene82111\_JingChaoZuZhi2, Unigene82117\_JingChaoZuZhi2, Unigene82143\_JingChaoZuZhi2, Unigene82215\_JingChaoZuZhi2, Unigene82297\_JingChaoZuZhi2, Unigene82329\_JingChaoZuZhi2, Unigene8237\_JingChaoZuZhi2, Unigene82389\_JingChaoZuZhi2, Unigene82489\_JingChaoZuZhi2, Unigene8248\_JingChaoZuZhi2, Unigene82513\_JingChaoZuZhi2, Unigene82517\_JingChaoZuZhi2, Unigene82521\_JingChaoZuZhi2, Unigene82578\_JingChaoZuZhi2, Unigene8261\_JingChaoZuZhi2, Unigene82662\_JingChaoZuZhi2, Unigene82688\_JingChaoZuZhi2, Unigene82760\_JingChaoZuZhi2, Unigene82842\_JingChaoZuZhi2, Unigene82921\_JingChaoZuZhi2, Unigene83079\_JingChaoZuZhi2, Unigene83214\_JingChaoZuZhi2, Unigene83433\_JingChaoZuZhi2, Unigene83487\_JingChaoZuZhi2, Unigene8350\_JingChaoZuZhi2, Unigene83582\_JingChaoZuZhi2, Unigene83583\_JingChaoZuZhi2, Unigene83588\_JingChaoZuZhi2, Unigene83663\_JingChaoZuZhi2, Unigene83744\_JingChaoZuZhi2, Unigene83766\_JingChaoZuZhi2, Unigene83782\_JingChaoZuZhi2, Unigene83825\_JingChaoZuZhi2, Unigene83872\_JingChaoZuZhi2, Unigene8392\_JingChaoZuZhi2, Unigene83984\_JingChaoZuZhi2, Unigene8406\_JingChaoZuZhi2, Unigene84076\_JingChaoZuZhi2, Unigene84109\_JingChaoZuZhi2, Unigene84152\_JingChaoZuZhi2, Unigene84168\_JingChaoZuZhi2, Unigene84180\_JingChaoZuZhi2, Unigene841\_JingChaoZuZhi2, Unigene84212\_JingChaoZuZhi2, Unigene84282\_JingChaoZuZhi2, Unigene8443\_JingChaoZuZhi2, Unigene84510\_JingChaoZuZhi2, Unigene84519\_JingChaoZuZhi2, Unigene84533\_JingChaoZuZhi2, Unigene8455\_JingChaoZuZhi2, Unigene84566\_JingChaoZuZhi2, Unigene84748\_JingChaoZuZhi2, Unigene84760\_JingChaoZuZhi2, Unigene84924\_JingChaoZuZhi2, Unigene84944\_JingChaoZuZhi2, Unigene84945\_JingChaoZuZhi2, Unigene84971\_JingChaoZuZhi2, Unigene84978\_JingChaoZuZhi2, Unigene84993\_JingChaoZuZhi2, Unigene8500\_JingChaoZuZhi2, Unigene85122\_JingChaoZuZhi2, Unigene85132\_JingChaoZuZhi2, Unigene85193\_JingChaoZuZhi2, Unigene851\_JingChaoZuZhi2, Unigene85214\_JingChaoZuZhi2, Unigene85314\_JingChaoZuZhi2, Unigene85387\_JingChaoZuZhi2, Unigene85407\_JingChaoZuZhi2, Unigene85503\_JingChaoZuZhi2, Unigene85574\_JingChaoZuZhi2, Unigene85577\_JingChaoZuZhi2, Unigene85578\_JingChaoZuZhi2, Unigene85598\_JingChaoZuZhi2, Unigene85770\_JingChaoZuZhi2, Unigene85902\_JingChaoZuZhi2, Unigene85923\_JingChaoZuZhi2, Unigene86020\_JingChaoZuZhi2, Unigene86091\_JingChaoZuZhi2, Unigene86145\_JingChaoZuZhi2, Unigene86206\_JingChaoZuZhi2, Unigene8621\_JingChaoZuZhi2, Unigene86349\_JingChaoZuZhi2, Unigene86368\_JingChaoZuZhi2, Unigene86470\_JingChaoZuZhi2, Unigene86526\_JingChaoZuZhi2, Unigene86546\_JingChaoZuZhi2, Unigene86579\_JingChaoZuZhi2, Unigene8658\_JingChaoZuZhi2, Unigene8661\_JingChaoZuZhi2, Unigene86661\_JingChaoZuZhi2, Unigene86669\_JingChaoZuZhi2, Unigene866\_JingChaoZuZhi2, Unigene86713\_JingChaoZuZhi2, Unigene86741\_JingChaoZuZhi2, Unigene86759\_JingChaoZuZhi2, Unigene86789\_JingChaoZuZhi2, Unigene86958\_JingChaoZuZhi2, Unigene87020\_JingChaoZuZhi2, Unigene87082\_JingChaoZuZhi2, Unigene8709\_JingChaoZuZhi2, Unigene87191\_JingChaoZuZhi2, Unigene87203\_JingChaoZuZhi2, Unigene87256\_JingChaoZuZhi2, Unigene87362\_JingChaoZuZhi2, Unigene87365\_JingChaoZuZhi2, Unigene87459\_JingChaoZuZhi2, Unigene87463\_JingChaoZuZhi2, Unigene87478\_JingChaoZuZhi2, Unigene87488\_JingChaoZuZhi2, Unigene87528\_JingChaoZuZhi2, Unigene87533\_JingChaoZuZhi2, Unigene87626\_JingChaoZuZhi2, Unigene87677\_JingChaoZuZhi2, Unigene876\_JingChaoZuZhi2, Unigene87774\_JingChaoZuZhi2, Unigene87825\_JingChaoZuZhi2, Unigene87851\_JingChaoZuZhi2, Unigene87853\_JingChaoZuZhi2, Unigene87922\_JingChaoZuZhi2, Unigene87946\_JingChaoZuZhi2, Unigene87958\_JingChaoZuZhi2, Unigene87984\_JingChaoZuZhi2, Unigene8799\_JingChaoZuZhi2, Unigene88059\_JingChaoZuZhi2, Unigene88090\_JingChaoZuZhi2, Unigene88092\_JingChaoZuZhi2, Unigene88185\_JingChaoZuZhi2, Unigene88215\_JingChaoZuZhi2, Unigene88216\_JingChaoZuZhi2, Unigene88307\_JingChaoZuZhi2, Unigene88326\_JingChaoZuZhi2, Unigene8833\_JingChaoZuZhi2, Unigene88342\_JingChaoZuZhi2, Unigene88391\_JingChaoZuZhi2, Unigene88400\_JingChaoZuZhi2, Unigene88406\_JingChaoZuZhi2, Unigene88442\_JingChaoZuZhi2, Unigene88470\_JingChaoZuZhi2, Unigene88484\_JingChaoZuZhi2, Unigene88511\_JingChaoZuZhi2, Unigene88608\_JingChaoZuZhi2, Unigene8860\_JingChaoZuZhi2, Unigene88683\_JingChaoZuZhi2, Unigene88706\_JingChaoZuZhi2, Unigene88708\_JingChaoZuZhi2, Unigene88738\_JingChaoZuZhi2, Unigene88741\_JingChaoZuZhi2, Unigene88778\_JingChaoZuZhi2, Unigene88779\_JingChaoZuZhi2, Unigene88781\_JingChaoZuZhi2, Unigene88797\_JingChaoZuZhi2, Unigene88830\_JingChaoZuZhi2, Unigene88837\_JingChaoZuZhi2, Unigene8887\_JingChaoZuZhi2, Unigene88925\_JingChaoZuZhi2, Unigene88945\_JingChaoZuZhi2, Unigene88979\_JingChaoZuZhi2, Unigene88988\_JingChaoZuZhi2, Unigene889\_JingChaoZuZhi2, Unigene89013\_JingChaoZuZhi2, Unigene89045\_JingChaoZuZhi2, Unigene89060\_JingChaoZuZhi2, Unigene8907\_JingChaoZuZhi2, Unigene89093\_JingChaoZuZhi2, Unigene89121\_JingChaoZuZhi2, Unigene89323\_JingChaoZuZhi2, Unigene89329\_JingChaoZuZhi2, Unigene89337\_JingChaoZuZhi2, Unigene89352\_JingChaoZuZhi2, Unigene89367\_JingChaoZuZhi2, Unigene89392\_JingChaoZuZhi2, Unigene89409\_JingChaoZuZhi2, Unigene89431\_JingChaoZuZhi2, Unigene89541\_JingChaoZuZhi2, Unigene89544\_JingChaoZuZhi2, Unigene89584\_JingChaoZuZhi2, Unigene89596\_JingChaoZuZhi2, Unigene89606\_JingChaoZuZhi2, Unigene89728\_JingChaoZuZhi2, Unigene89746\_JingChaoZuZhi2, Unigene89769\_JingChaoZuZhi2, Unigene89834\_JingChaoZuZhi2, Unigene89867\_JingChaoZuZhi2, Unigene89956\_JingChaoZuZhi2, Unigene89959\_JingChaoZuZhi2, Unigene90027\_JingChaoZuZhi2, Unigene90038\_JingChaoZuZhi2, Unigene9005\_JingChaoZuZhi2, Unigene90066\_JingChaoZuZhi2, Unigene90071\_JingChaoZuZhi2, Unigene90209\_JingChaoZuZhi2, Unigene90341\_JingChaoZuZhi2, Unigene90356\_JingChaoZuZhi2, Unigene9038\_JingChaoZuZhi2, Unigene90401\_JingChaoZuZhi2, Unigene90454\_JingChaoZuZhi2, Unigene9049\_JingChaoZuZhi2, Unigene9050\_JingChaoZuZhi2, Unigene90564\_JingChaoZuZhi2, Unigene90626\_JingChaoZuZhi2, Unigene90714\_JingChaoZuZhi2, Unigene90767\_JingChaoZuZhi2, Unigene90779\_JingChaoZuZhi2, Unigene90953\_JingChaoZuZhi2, Unigene90995\_JingChaoZuZhi2, Unigene91030\_JingChaoZuZhi2, Unigene91099\_JingChaoZuZhi2, Unigene91148\_JingChaoZuZhi2, Unigene91163\_JingChaoZuZhi2, Unigene91179\_JingChaoZuZhi2, Unigene91233\_JingChaoZuZhi2, Unigene91289\_JingChaoZuZhi2, Unigene91321\_JingChaoZuZhi2, Unigene91379\_JingChaoZuZhi2, Unigene91390\_JingChaoZuZhi2, Unigene91456\_JingChaoZuZhi2, Unigene91475\_JingChaoZuZhi2, Unigene91487\_JingChaoZuZhi2, Unigene91529\_JingChaoZuZhi2, Unigene91553\_JingChaoZuZhi2, Unigene91574\_JingChaoZuZhi2, Unigene91612\_JingChaoZuZhi2, Unigene91631\_JingChaoZuZhi2, Unigene91680\_JingChaoZuZhi2, Unigene91710\_JingChaoZuZhi2, Unigene91717\_JingChaoZuZhi2, Unigene91839\_JingChaoZuZhi2, Unigene91848\_JingChaoZuZhi2, Unigene91871\_JingChaoZuZhi2, Unigene91906\_JingChaoZuZhi2, Unigene91944\_JingChaoZuZhi2, Unigene91958\_JingChaoZuZhi2, Unigene91991\_JingChaoZuZhi2, Unigene92091\_JingChaoZuZhi2, Unigene92104\_JingChaoZuZhi2, Unigene92112\_JingChaoZuZhi2, Unigene92141\_JingChaoZuZhi2, Unigene92309\_JingChaoZuZhi2, Unigene92329\_JingChaoZuZhi2, Unigene92346\_JingChaoZuZhi2, Unigene92372\_JingChaoZuZhi2, Unigene92395\_JingChaoZuZhi2, Unigene92448\_JingChaoZuZhi2, Unigene92466\_JingChaoZuZhi2, Unigene92471\_JingChaoZuZhi2, Unigene92485\_JingChaoZuZhi2, Unigene92492\_JingChaoZuZhi2, Unigene92577\_JingChaoZuZhi2, Unigene92580\_JingChaoZuZhi2, Unigene9260\_JingChaoZuZhi2, Unigene92671\_JingChaoZuZhi2, Unigene92673\_JingChaoZuZhi2, Unigene92681\_JingChaoZuZhi2, Unigene92700\_JingChaoZuZhi2, Unigene92722\_JingChaoZuZhi2, Unigene92769\_JingChaoZuZhi2, Unigene92780\_JingChaoZuZhi2, Unigene92832\_JingChaoZuZhi2, Unigene92838\_JingChaoZuZhi2, Unigene92929\_JingChaoZuZhi2, Unigene92951\_JingChaoZuZhi2, Unigene92995\_JingChaoZuZhi2, Unigene93010\_JingChaoZuZhi2, Unigene93073\_JingChaoZuZhi2, Unigene93133\_JingChaoZuZhi2, Unigene93246\_JingChaoZuZhi2, Unigene93279\_JingChaoZuZhi2, Unigene93374\_JingChaoZuZhi2, Unigene93552\_JingChaoZuZhi2, Unigene93562\_JingChaoZuZhi2, Unigene93573\_JingChaoZuZhi2, Unigene93582\_JingChaoZuZhi2, Unigene93592\_JingChaoZuZhi2, Unigene93610\_JingChaoZuZhi2, Unigene93631\_JingChaoZuZhi2, Unigene93702\_JingChaoZuZhi2, Unigene93717\_JingChaoZuZhi2, Unigene93742\_JingChaoZuZhi2, Unigene93768\_JingChaoZuZhi2, Unigene93780\_JingChaoZuZhi2, Unigene93782\_JingChaoZuZhi2, Unigene93807\_JingChaoZuZhi2, Unigene93821\_JingChaoZuZhi2, Unigene93853\_JingChaoZuZhi2, Unigene93865\_JingChaoZuZhi2, Unigene93884\_JingChaoZuZhi2, Unigene93979\_JingChaoZuZhi2, Unigene93990\_JingChaoZuZhi2, Unigene94083\_JingChaoZuZhi2, Unigene94110\_JingChaoZuZhi2, Unigene94120\_JingChaoZuZhi2, Unigene94129\_JingChaoZuZhi2, Unigene94134\_JingChaoZuZhi2, Unigene94149\_JingChaoZuZhi2, Unigene94194\_JingChaoZuZhi2, Unigene94208\_JingChaoZuZhi2, Unigene94238\_JingChaoZuZhi2, Unigene94260\_JingChaoZuZhi2, Unigene94298\_JingChaoZuZhi2, Unigene94370\_JingChaoZuZhi2, Unigene94401\_JingChaoZuZhi2, Unigene94404\_JingChaoZuZhi2, Unigene94456\_JingChaoZuZhi2, Unigene94471\_JingChaoZuZhi2, Unigene94520\_JingChaoZuZhi2, Unigene94544\_JingChaoZuZhi2, Unigene94546\_JingChaoZuZhi2, Unigene94561\_JingChaoZuZhi2, Unigene94584\_JingChaoZuZhi2, Unigene94647\_JingChaoZuZhi2, Unigene94656\_JingChaoZuZhi2, Unigene94685\_JingChaoZuZhi2, Unigene94687\_JingChaoZuZhi2, Unigene94711\_JingChaoZuZhi2, Unigene94723\_JingChaoZuZhi2, Unigene94792\_JingChaoZuZhi2, Unigene94795\_JingChaoZuZhi2, Unigene94815\_JingChaoZuZhi2, Unigene94823\_JingChaoZuZhi2, Unigene94878\_JingChaoZuZhi2, Unigene94891\_JingChaoZuZhi2, Unigene94903\_JingChaoZuZhi2, Unigene94904\_JingChaoZuZhi2, Unigene9490\_JingChaoZuZhi2, Unigene94922\_JingChaoZuZhi2, Unigene94950\_JingChaoZuZhi2, Unigene94968\_JingChaoZuZhi2, Unigene95014\_JingChaoZuZhi2, Unigene95020\_JingChaoZuZhi2, Unigene95031\_JingChaoZuZhi2, Unigene95059\_JingChaoZuZhi2, Unigene95071\_JingChaoZuZhi2, Unigene95088\_JingChaoZuZhi2, Unigene95098\_JingChaoZuZhi2, Unigene95119\_JingChaoZuZhi2, Unigene95120\_JingChaoZuZhi2, Unigene95169\_JingChaoZuZhi2, Unigene951\_JingChaoZuZhi2, Unigene95203\_JingChaoZuZhi2, Unigene95212\_JingChaoZuZhi2, Unigene95243\_JingChaoZuZhi2, Unigene95359\_JingChaoZuZhi2, Unigene95371\_JingChaoZuZhi2, Unigene9540\_JingChaoZuZhi2, Unigene95456\_JingChaoZuZhi2, Unigene95486\_JingChaoZuZhi2, Unigene95565\_JingChaoZuZhi2, Unigene95589\_JingChaoZuZhi2, Unigene95633\_JingChaoZuZhi2, Unigene95686\_JingChaoZuZhi2, Unigene95782\_JingChaoZuZhi2, Unigene95807\_JingChaoZuZhi2, Unigene95812\_JingChaoZuZhi2, Unigene95822\_JingChaoZuZhi2, Unigene95875\_JingChaoZuZhi2, Unigene95879\_JingChaoZuZhi2, Unigene95881\_JingChaoZuZhi2, Unigene95882\_JingChaoZuZhi2, Unigene95907\_JingChaoZuZhi2, Unigene95938\_JingChaoZuZhi2, Unigene95945\_JingChaoZuZhi2, Unigene95954\_JingChaoZuZhi2, Unigene95972\_JingChaoZuZhi2, Unigene95982\_JingChaoZuZhi2, Unigene95987\_JingChaoZuZhi2, Unigene95994\_JingChaoZuZhi2, Unigene96012\_JingChaoZuZhi2, Unigene96033\_JingChaoZuZhi2, Unigene96061\_JingChaoZuZhi2, Unigene96083\_JingChaoZuZhi2, Unigene96097\_JingChaoZuZhi2, Unigene96121\_JingChaoZuZhi2, Unigene96170\_JingChaoZuZhi2, Unigene96200\_JingChaoZuZhi2, Unigene96217\_JingChaoZuZhi2, Unigene96236\_JingChaoZuZhi2, Unigene96243\_JingChaoZuZhi2, Unigene96247\_JingChaoZuZhi2, Unigene96288\_JingChaoZuZhi2, Unigene96292\_JingChaoZuZhi2, Unigene96294\_JingChaoZuZhi2, Unigene96303\_JingChaoZuZhi2, Unigene96308\_JingChaoZuZhi2, Unigene9688\_JingChaoZuZhi2, Unigene9722\_JingChaoZuZhi2, Unigene9739\_JingChaoZuZhi2, Unigene9743\_JingChaoZuZhi2, Unigene9807\_JingChaoZuZhi2, Unigene9833\_JingChaoZuZhi2, Unigene9902\_JingChaoZuZhi2, Unigene9924\_JingChaoZuZhi2, Unigene9988\_JingChaoZuZhi2, Unigene9992\_JingChaoZuZhi2 |
| 3 | Spliceosome | Unigene1000\_JingChaoZuZhi2, Unigene10117\_JingChaoZuZhi2, Unigene10177\_JingChaoZuZhi2, Unigene10221\_JingChaoZuZhi2, Unigene1025\_JingChaoZuZhi2, Unigene10310\_JingChaoZuZhi2, Unigene10337\_JingChaoZuZhi2, Unigene10341\_JingChaoZuZhi2, Unigene10380\_JingChaoZuZhi2, Unigene10462\_JingChaoZuZhi2, Unigene1047\_JingChaoZuZhi2, Unigene10589\_JingChaoZuZhi2, Unigene10616\_JingChaoZuZhi2, Unigene10664\_JingChaoZuZhi2, Unigene10733\_JingChaoZuZhi2, Unigene1080\_JingChaoZuZhi2, Unigene10969\_JingChaoZuZhi2, Unigene11122\_JingChaoZuZhi2, Unigene11125\_JingChaoZuZhi2, Unigene1121\_JingChaoZuZhi2, Unigene11241\_JingChaoZuZhi2, Unigene11266\_JingChaoZuZhi2, Unigene11278\_JingChaoZuZhi2, Unigene11286\_JingChaoZuZhi2, Unigene11373\_JingChaoZuZhi2, Unigene11419\_JingChaoZuZhi2, Unigene1143\_JingChaoZuZhi2, Unigene11454\_JingChaoZuZhi2, Unigene11468\_JingChaoZuZhi2, Unigene11489\_JingChaoZuZhi2, Unigene11632\_JingChaoZuZhi2, Unigene11699\_JingChaoZuZhi2, Unigene11758\_JingChaoZuZhi2, Unigene11815\_JingChaoZuZhi2, Unigene11849\_JingChaoZuZhi2, Unigene1187\_JingChaoZuZhi2, Unigene11970\_JingChaoZuZhi2, Unigene11982\_JingChaoZuZhi2, Unigene12033\_JingChaoZuZhi2, Unigene12268\_JingChaoZuZhi2, Unigene12331\_JingChaoZuZhi2, Unigene12338\_JingChaoZuZhi2, Unigene12461\_JingChaoZuZhi2, Unigene1247\_JingChaoZuZhi2, Unigene12514\_JingChaoZuZhi2, Unigene12519\_JingChaoZuZhi2, Unigene12529\_JingChaoZuZhi2, Unigene12559\_JingChaoZuZhi2, Unigene12584\_JingChaoZuZhi2, Unigene12637\_JingChaoZuZhi2, Unigene12701\_JingChaoZuZhi2, Unigene12705\_JingChaoZuZhi2, Unigene1273\_JingChaoZuZhi2, Unigene12746\_JingChaoZuZhi2, Unigene12748\_JingChaoZuZhi2, Unigene12757\_JingChaoZuZhi2, Unigene12800\_JingChaoZuZhi2, Unigene12825\_JingChaoZuZhi2, Unigene12828\_JingChaoZuZhi2, Unigene12862\_JingChaoZuZhi2, Unigene12874\_JingChaoZuZhi2, Unigene12892\_JingChaoZuZhi2, Unigene12900\_JingChaoZuZhi2, Unigene12976\_JingChaoZuZhi2, Unigene13013\_JingChaoZuZhi2, Unigene13021\_JingChaoZuZhi2, Unigene13037\_JingChaoZuZhi2, Unigene13066\_JingChaoZuZhi2, Unigene13078\_JingChaoZuZhi2, Unigene13097\_JingChaoZuZhi2, Unigene13115\_JingChaoZuZhi2, Unigene1311\_JingChaoZuZhi2, Unigene1314\_JingChaoZuZhi2, Unigene13210\_JingChaoZuZhi2, Unigene13225\_JingChaoZuZhi2, Unigene13344\_JingChaoZuZhi2, Unigene13386\_JingChaoZuZhi2, Unigene13464\_JingChaoZuZhi2, Unigene1350\_JingChaoZuZhi2, Unigene1352\_JingChaoZuZhi2, Unigene13551\_JingChaoZuZhi2, Unigene13561\_JingChaoZuZhi2, Unigene13673\_JingChaoZuZhi2, Unigene13700\_JingChaoZuZhi2, Unigene13717\_JingChaoZuZhi2, Unigene1371\_JingChaoZuZhi2, Unigene1374\_JingChaoZuZhi2, Unigene13773\_JingChaoZuZhi2, Unigene13775\_JingChaoZuZhi2, Unigene13778\_JingChaoZuZhi2, Unigene1377\_JingChaoZuZhi2, Unigene13831\_JingChaoZuZhi2, Unigene1393\_JingChaoZuZhi2, Unigene13975\_JingChaoZuZhi2, Unigene13982\_JingChaoZuZhi2, Unigene14074\_JingChaoZuZhi2, Unigene14148\_JingChaoZuZhi2, Unigene1419\_JingChaoZuZhi2, Unigene14250\_JingChaoZuZhi2, Unigene14292\_JingChaoZuZhi2, Unigene14333\_JingChaoZuZhi2, Unigene1438\_JingChaoZuZhi2, Unigene14409\_JingChaoZuZhi2, Unigene14420\_JingChaoZuZhi2, Unigene14450\_JingChaoZuZhi2, Unigene1453\_JingChaoZuZhi2, Unigene145\_JingChaoZuZhi2, Unigene14610\_JingChaoZuZhi2, Unigene14647\_JingChaoZuZhi2, Unigene14659\_JingChaoZuZhi2, Unigene1473\_JingChaoZuZhi2, Unigene14749\_JingChaoZuZhi2, Unigene14755\_JingChaoZuZhi2, Unigene14820\_JingChaoZuZhi2, Unigene14845\_JingChaoZuZhi2, Unigene14852\_JingChaoZuZhi2, Unigene14942\_JingChaoZuZhi2, Unigene14970\_JingChaoZuZhi2, Unigene1511\_JingChaoZuZhi2, Unigene15163\_JingChaoZuZhi2, Unigene15220\_JingChaoZuZhi2, Unigene15223\_JingChaoZuZhi2, Unigene1527\_JingChaoZuZhi2, Unigene15383\_JingChaoZuZhi2, Unigene15394\_JingChaoZuZhi2, Unigene1540\_JingChaoZuZhi2, Unigene1542\_JingChaoZuZhi2, Unigene15444\_JingChaoZuZhi2, Unigene15463\_JingChaoZuZhi2, Unigene15559\_JingChaoZuZhi2, Unigene15583\_JingChaoZuZhi2, Unigene15659\_JingChaoZuZhi2, Unigene15685\_JingChaoZuZhi2, Unigene1569\_JingChaoZuZhi2, Unigene15731\_JingChaoZuZhi2, Unigene15795\_JingChaoZuZhi2, Unigene157\_JingChaoZuZhi2, Unigene15843\_JingChaoZuZhi2, Unigene15889\_JingChaoZuZhi2, Unigene158\_JingChaoZuZhi2, Unigene15906\_JingChaoZuZhi2, Unigene15994\_JingChaoZuZhi2, Unigene16029\_JingChaoZuZhi2, Unigene16103\_JingChaoZuZhi2, Unigene16171\_JingChaoZuZhi2, Unigene16242\_JingChaoZuZhi2, Unigene1625\_JingChaoZuZhi2, Unigene16271\_JingChaoZuZhi2, Unigene16328\_JingChaoZuZhi2, Unigene16348\_JingChaoZuZhi2, Unigene16356\_JingChaoZuZhi2, Unigene1635\_JingChaoZuZhi2, Unigene16508\_JingChaoZuZhi2, Unigene16565\_JingChaoZuZhi2, Unigene16569\_JingChaoZuZhi2, Unigene16572\_JingChaoZuZhi2, Unigene16605\_JingChaoZuZhi2, Unigene16640\_JingChaoZuZhi2, Unigene16655\_JingChaoZuZhi2, Unigene16667\_JingChaoZuZhi2, Unigene16681\_JingChaoZuZhi2, Unigene1678\_JingChaoZuZhi2, Unigene16818\_JingChaoZuZhi2, Unigene16820\_JingChaoZuZhi2, Unigene16901\_JingChaoZuZhi2, Unigene16921\_JingChaoZuZhi2, Unigene16950\_JingChaoZuZhi2, Unigene16953\_JingChaoZuZhi2, Unigene17058\_JingChaoZuZhi2, Unigene17101\_JingChaoZuZhi2, Unigene17112\_JingChaoZuZhi2, Unigene17167\_JingChaoZuZhi2, Unigene17191\_JingChaoZuZhi2, Unigene17227\_JingChaoZuZhi2, Unigene17229\_JingChaoZuZhi2, Unigene17236\_JingChaoZuZhi2, Unigene17264\_JingChaoZuZhi2, Unigene17265\_JingChaoZuZhi2, Unigene17297\_JingChaoZuZhi2, Unigene17393\_JingChaoZuZhi2, Unigene173\_JingChaoZuZhi2, Unigene17403\_JingChaoZuZhi2, Unigene17420\_JingChaoZuZhi2, Unigene17429\_JingChaoZuZhi2, Unigene17436\_JingChaoZuZhi2, Unigene17498\_JingChaoZuZhi2, Unigene17693\_JingChaoZuZhi2, Unigene1769\_JingChaoZuZhi2, Unigene17737\_JingChaoZuZhi2, Unigene17741\_JingChaoZuZhi2, Unigene17772\_JingChaoZuZhi2, Unigene17790\_JingChaoZuZhi2, Unigene17796\_JingChaoZuZhi2, Unigene17868\_JingChaoZuZhi2, Unigene17887\_JingChaoZuZhi2, Unigene17891\_JingChaoZuZhi2, Unigene17926\_JingChaoZuZhi2, Unigene17932\_JingChaoZuZhi2, Unigene17965\_JingChaoZuZhi2, Unigene17979\_JingChaoZuZhi2, Unigene17988\_JingChaoZuZhi2, Unigene179\_JingChaoZuZhi2, Unigene18066\_JingChaoZuZhi2, Unigene18099\_JingChaoZuZhi2, Unigene18113\_JingChaoZuZhi2, Unigene18186\_JingChaoZuZhi2, Unigene18199\_JingChaoZuZhi2, Unigene18211\_JingChaoZuZhi2, Unigene18262\_JingChaoZuZhi2, Unigene18287\_JingChaoZuZhi2, Unigene1829\_JingChaoZuZhi2, Unigene18359\_JingChaoZuZhi2, Unigene18360\_JingChaoZuZhi2, Unigene18372\_JingChaoZuZhi2, Unigene183\_JingChaoZuZhi2, Unigene18577\_JingChaoZuZhi2, Unigene18585\_JingChaoZuZhi2, Unigene18587\_JingChaoZuZhi2, Unigene18671\_JingChaoZuZhi2, Unigene1869\_JingChaoZuZhi2, Unigene18816\_JingChaoZuZhi2, Unigene18870\_JingChaoZuZhi2, Unigene18887\_JingChaoZuZhi2, Unigene18960\_JingChaoZuZhi2, Unigene18964\_JingChaoZuZhi2, Unigene19000\_JingChaoZuZhi2, Unigene190\_JingChaoZuZhi2, Unigene19131\_JingChaoZuZhi2, Unigene19270\_JingChaoZuZhi2, Unigene19301\_JingChaoZuZhi2, Unigene19319\_JingChaoZuZhi2, Unigene19355\_JingChaoZuZhi2, Unigene19368\_JingChaoZuZhi2, Unigene19374\_JingChaoZuZhi2, Unigene19384\_JingChaoZuZhi2, Unigene19388\_JingChaoZuZhi2, Unigene19396\_JingChaoZuZhi2, Unigene19436\_JingChaoZuZhi2, Unigene19440\_JingChaoZuZhi2, Unigene19532\_JingChaoZuZhi2, Unigene19533\_JingChaoZuZhi2, Unigene19545\_JingChaoZuZhi2, Unigene19625\_JingChaoZuZhi2, Unigene19627\_JingChaoZuZhi2, Unigene19648\_JingChaoZuZhi2, Unigene1971\_JingChaoZuZhi2, Unigene19746\_JingChaoZuZhi2, Unigene19789\_JingChaoZuZhi2, Unigene19829\_JingChaoZuZhi2, Unigene19866\_JingChaoZuZhi2, Unigene1987\_JingChaoZuZhi2, Unigene19925\_JingChaoZuZhi2, Unigene19927\_JingChaoZuZhi2, Unigene19934\_JingChaoZuZhi2, Unigene19986\_JingChaoZuZhi2, Unigene19995\_JingChaoZuZhi2, Unigene20003\_JingChaoZuZhi2, Unigene20030\_JingChaoZuZhi2, Unigene2003\_JingChaoZuZhi2, Unigene20091\_JingChaoZuZhi2, Unigene2009\_JingChaoZuZhi2, Unigene20119\_JingChaoZuZhi2, Unigene20165\_JingChaoZuZhi2, Unigene2018\_JingChaoZuZhi2, Unigene20200\_JingChaoZuZhi2, Unigene20209\_JingChaoZuZhi2, Unigene20360\_JingChaoZuZhi2, Unigene20424\_JingChaoZuZhi2, Unigene20516\_JingChaoZuZhi2, Unigene20529\_JingChaoZuZhi2, Unigene20539\_JingChaoZuZhi2, Unigene20678\_JingChaoZuZhi2, Unigene20690\_JingChaoZuZhi2, Unigene20720\_JingChaoZuZhi2, Unigene20758\_JingChaoZuZhi2, Unigene20811\_JingChaoZuZhi2, Unigene20837\_JingChaoZuZhi2, Unigene20888\_JingChaoZuZhi2, Unigene20940\_JingChaoZuZhi2, Unigene20943\_JingChaoZuZhi2, Unigene20952\_JingChaoZuZhi2, Unigene20996\_JingChaoZuZhi2, Unigene21026\_JingChaoZuZhi2, Unigene2102\_JingChaoZuZhi2, Unigene21057\_JingChaoZuZhi2, Unigene21077\_JingChaoZuZhi2, Unigene21103\_JingChaoZuZhi2, Unigene21183\_JingChaoZuZhi2, Unigene21186\_JingChaoZuZhi2, Unigene21188\_JingChaoZuZhi2, Unigene21227\_JingChaoZuZhi2, Unigene21270\_JingChaoZuZhi2, Unigene21290\_JingChaoZuZhi2, Unigene21323\_JingChaoZuZhi2, Unigene21400\_JingChaoZuZhi2, Unigene21469\_JingChaoZuZhi2, Unigene21477\_JingChaoZuZhi2, Unigene21504\_JingChaoZuZhi2, Unigene21514\_JingChaoZuZhi2, Unigene21536\_JingChaoZuZhi2, Unigene21552\_JingChaoZuZhi2, Unigene21565\_JingChaoZuZhi2, Unigene21573\_JingChaoZuZhi2, Unigene21577\_JingChaoZuZhi2, Unigene21579\_JingChaoZuZhi2, Unigene21730\_JingChaoZuZhi2, Unigene21764\_JingChaoZuZhi2, Unigene2176\_JingChaoZuZhi2, Unigene2180\_JingChaoZuZhi2, Unigene21868\_JingChaoZuZhi2, Unigene21870\_JingChaoZuZhi2, Unigene21871\_JingChaoZuZhi2, Unigene21914\_JingChaoZuZhi2, Unigene21925\_JingChaoZuZhi2, Unigene22006\_JingChaoZuZhi2, Unigene2200\_JingChaoZuZhi2, Unigene22010\_JingChaoZuZhi2, Unigene22291\_JingChaoZuZhi2, Unigene22308\_JingChaoZuZhi2, Unigene2231\_JingChaoZuZhi2, Unigene22409\_JingChaoZuZhi2, Unigene22532\_JingChaoZuZhi2, Unigene22533\_JingChaoZuZhi2, Unigene22534\_JingChaoZuZhi2, Unigene22545\_JingChaoZuZhi2, Unigene22618\_JingChaoZuZhi2, Unigene22626\_JingChaoZuZhi2, Unigene22657\_JingChaoZuZhi2, Unigene22664\_JingChaoZuZhi2, Unigene22707\_JingChaoZuZhi2, Unigene22733\_JingChaoZuZhi2, Unigene22845\_JingChaoZuZhi2, Unigene22849\_JingChaoZuZhi2, Unigene22869\_JingChaoZuZhi2, Unigene22870\_JingChaoZuZhi2, Unigene22889\_JingChaoZuZhi2, Unigene22978\_JingChaoZuZhi2, Unigene23016\_JingChaoZuZhi2, Unigene23361\_JingChaoZuZhi2, Unigene23518\_JingChaoZuZhi2, Unigene24167\_JingChaoZuZhi2, Unigene24632\_JingChaoZuZhi2, Unigene25675\_JingChaoZuZhi2, Unigene25958\_JingChaoZuZhi2, Unigene26095\_JingChaoZuZhi2, Unigene26142\_JingChaoZuZhi2, Unigene2622\_JingChaoZuZhi2, Unigene26244\_JingChaoZuZhi2, Unigene26419\_JingChaoZuZhi2, Unigene26523\_JingChaoZuZhi2, Unigene2693\_JingChaoZuZhi2, Unigene27217\_JingChaoZuZhi2, Unigene274\_JingChaoZuZhi2, Unigene2751\_JingChaoZuZhi2, Unigene27587\_JingChaoZuZhi2, Unigene27639\_JingChaoZuZhi2, Unigene28357\_JingChaoZuZhi2, Unigene28596\_JingChaoZuZhi2, Unigene28772\_JingChaoZuZhi2, Unigene29216\_JingChaoZuZhi2, Unigene29281\_JingChaoZuZhi2, Unigene293\_JingChaoZuZhi2, Unigene2969\_JingChaoZuZhi2, Unigene29847\_JingChaoZuZhi2, Unigene29950\_JingChaoZuZhi2, Unigene30213\_JingChaoZuZhi2, Unigene3075\_JingChaoZuZhi2, Unigene30845\_JingChaoZuZhi2, Unigene30947\_JingChaoZuZhi2, Unigene31148\_JingChaoZuZhi2, Unigene31580\_JingChaoZuZhi2, Unigene31634\_JingChaoZuZhi2, Unigene3166\_JingChaoZuZhi2, Unigene32211\_JingChaoZuZhi2, Unigene32374\_JingChaoZuZhi2, Unigene32498\_JingChaoZuZhi2, Unigene3253\_JingChaoZuZhi2, Unigene3297\_JingChaoZuZhi2, Unigene3333\_JingChaoZuZhi2, Unigene33536\_JingChaoZuZhi2, Unigene33732\_JingChaoZuZhi2, Unigene33805\_JingChaoZuZhi2, Unigene33862\_JingChaoZuZhi2, Unigene34055\_JingChaoZuZhi2, Unigene34256\_JingChaoZuZhi2, Unigene3462\_JingChaoZuZhi2, Unigene3470\_JingChaoZuZhi2, Unigene34777\_JingChaoZuZhi2, Unigene35069\_JingChaoZuZhi2, Unigene3596\_JingChaoZuZhi2, Unigene35990\_JingChaoZuZhi2, Unigene36059\_JingChaoZuZhi2, Unigene3634\_JingChaoZuZhi2, Unigene36411\_JingChaoZuZhi2, Unigene3688\_JingChaoZuZhi2, Unigene3705\_JingChaoZuZhi2, Unigene37342\_JingChaoZuZhi2, Unigene37431\_JingChaoZuZhi2, Unigene3762\_JingChaoZuZhi2, Unigene3764\_JingChaoZuZhi2, Unigene37684\_JingChaoZuZhi2, Unigene37817\_JingChaoZuZhi2, Unigene37884\_JingChaoZuZhi2, Unigene37970\_JingChaoZuZhi2, Unigene38121\_JingChaoZuZhi2, Unigene38199\_JingChaoZuZhi2, Unigene38298\_JingChaoZuZhi2, Unigene3858\_JingChaoZuZhi2, Unigene38615\_JingChaoZuZhi2, Unigene38765\_JingChaoZuZhi2, Unigene38917\_JingChaoZuZhi2, Unigene39191\_JingChaoZuZhi2, Unigene39197\_JingChaoZuZhi2, Unigene4044\_JingChaoZuZhi2, Unigene40728\_JingChaoZuZhi2, Unigene40922\_JingChaoZuZhi2, Unigene41120\_JingChaoZuZhi2, Unigene41149\_JingChaoZuZhi2, Unigene41367\_JingChaoZuZhi2, Unigene416\_JingChaoZuZhi2, Unigene42273\_JingChaoZuZhi2, Unigene42422\_JingChaoZuZhi2, Unigene4254\_JingChaoZuZhi2, Unigene42655\_JingChaoZuZhi2, Unigene427\_JingChaoZuZhi2, Unigene4291\_JingChaoZuZhi2, Unigene429\_JingChaoZuZhi2, Unigene43169\_JingChaoZuZhi2, Unigene4317\_JingChaoZuZhi2, Unigene43249\_JingChaoZuZhi2, Unigene43264\_JingChaoZuZhi2, Unigene43348\_JingChaoZuZhi2, Unigene434\_JingChaoZuZhi2, Unigene43512\_JingChaoZuZhi2, Unigene43515\_JingChaoZuZhi2, Unigene43582\_JingChaoZuZhi2, Unigene43592\_JingChaoZuZhi2, Unigene43726\_JingChaoZuZhi2, Unigene4375\_JingChaoZuZhi2, Unigene43822\_JingChaoZuZhi2, Unigene4397\_JingChaoZuZhi2, Unigene44205\_JingChaoZuZhi2, Unigene44516\_JingChaoZuZhi2, Unigene44969\_JingChaoZuZhi2, Unigene45244\_JingChaoZuZhi2, Unigene45367\_JingChaoZuZhi2, Unigene45383\_JingChaoZuZhi2, Unigene4546\_JingChaoZuZhi2, Unigene45662\_JingChaoZuZhi2, Unigene457\_JingChaoZuZhi2, Unigene4588\_JingChaoZuZhi2, Unigene4589\_JingChaoZuZhi2, Unigene4639\_JingChaoZuZhi2, Unigene46415\_JingChaoZuZhi2, Unigene46820\_JingChaoZuZhi2, Unigene47022\_JingChaoZuZhi2, Unigene4730\_JingChaoZuZhi2, Unigene4801\_JingChaoZuZhi2, Unigene4807\_JingChaoZuZhi2, Unigene48093\_JingChaoZuZhi2, Unigene48130\_JingChaoZuZhi2, Unigene48226\_JingChaoZuZhi2, Unigene48228\_JingChaoZuZhi2, Unigene48369\_JingChaoZuZhi2, Unigene4840\_JingChaoZuZhi2, Unigene48455\_JingChaoZuZhi2, Unigene49151\_JingChaoZuZhi2, Unigene49413\_JingChaoZuZhi2, Unigene49603\_JingChaoZuZhi2, Unigene49609\_JingChaoZuZhi2, Unigene50026\_JingChaoZuZhi2, Unigene500\_JingChaoZuZhi2, Unigene5021\_JingChaoZuZhi2, Unigene50278\_JingChaoZuZhi2, Unigene5033\_JingChaoZuZhi2, Unigene50503\_JingChaoZuZhi2, Unigene50514\_JingChaoZuZhi2, Unigene50660\_JingChaoZuZhi2, Unigene50865\_JingChaoZuZhi2, Unigene51057\_JingChaoZuZhi2, Unigene51269\_JingChaoZuZhi2, Unigene51554\_JingChaoZuZhi2, Unigene5158\_JingChaoZuZhi2, Unigene5159\_JingChaoZuZhi2, Unigene5161\_JingChaoZuZhi2, Unigene51679\_JingChaoZuZhi2, Unigene51703\_JingChaoZuZhi2, Unigene51820\_JingChaoZuZhi2, Unigene52266\_JingChaoZuZhi2, Unigene5227\_JingChaoZuZhi2, Unigene52366\_JingChaoZuZhi2, Unigene52449\_JingChaoZuZhi2, Unigene52629\_JingChaoZuZhi2, Unigene5263\_JingChaoZuZhi2, Unigene52694\_JingChaoZuZhi2, Unigene52804\_JingChaoZuZhi2, Unigene5283\_JingChaoZuZhi2, Unigene5311\_JingChaoZuZhi2, Unigene53228\_JingChaoZuZhi2, Unigene53269\_JingChaoZuZhi2, Unigene5341\_JingChaoZuZhi2, Unigene53491\_JingChaoZuZhi2, Unigene53622\_JingChaoZuZhi2, Unigene53689\_JingChaoZuZhi2, Unigene53828\_JingChaoZuZhi2, Unigene53915\_JingChaoZuZhi2, Unigene53918\_JingChaoZuZhi2, Unigene54085\_JingChaoZuZhi2, Unigene5418\_JingChaoZuZhi2, Unigene54862\_JingChaoZuZhi2, Unigene55100\_JingChaoZuZhi2, Unigene5529\_JingChaoZuZhi2, Unigene55999\_JingChaoZuZhi2, Unigene56293\_JingChaoZuZhi2, Unigene5636\_JingChaoZuZhi2, Unigene5650\_JingChaoZuZhi2, Unigene56510\_JingChaoZuZhi2, Unigene56715\_JingChaoZuZhi2, Unigene5674\_JingChaoZuZhi2, Unigene56799\_JingChaoZuZhi2, Unigene5687\_JingChaoZuZhi2, Unigene57037\_JingChaoZuZhi2, Unigene5731\_JingChaoZuZhi2, Unigene57676\_JingChaoZuZhi2, Unigene5782\_JingChaoZuZhi2, Unigene57888\_JingChaoZuZhi2, Unigene5824\_JingChaoZuZhi2, Unigene58394\_JingChaoZuZhi2, Unigene58405\_JingChaoZuZhi2, Unigene58470\_JingChaoZuZhi2, Unigene58558\_JingChaoZuZhi2, Unigene58680\_JingChaoZuZhi2, Unigene58717\_JingChaoZuZhi2, Unigene58746\_JingChaoZuZhi2, Unigene5882\_JingChaoZuZhi2, Unigene5897\_JingChaoZuZhi2, Unigene59018\_JingChaoZuZhi2, Unigene5904\_JingChaoZuZhi2, Unigene59118\_JingChaoZuZhi2, Unigene59250\_JingChaoZuZhi2, Unigene59510\_JingChaoZuZhi2, Unigene59513\_JingChaoZuZhi2, Unigene59629\_JingChaoZuZhi2, Unigene5990\_JingChaoZuZhi2, Unigene59925\_JingChaoZuZhi2, Unigene5992\_JingChaoZuZhi2, Unigene59952\_JingChaoZuZhi2, Unigene59990\_JingChaoZuZhi2, Unigene60151\_JingChaoZuZhi2, Unigene60198\_JingChaoZuZhi2, Unigene60551\_JingChaoZuZhi2, Unigene60690\_JingChaoZuZhi2, Unigene60806\_JingChaoZuZhi2, Unigene60854\_JingChaoZuZhi2, Unigene60891\_JingChaoZuZhi2, Unigene60987\_JingChaoZuZhi2, Unigene61064\_JingChaoZuZhi2, Unigene6120\_JingChaoZuZhi2, Unigene61330\_JingChaoZuZhi2, Unigene6135\_JingChaoZuZhi2, Unigene61457\_JingChaoZuZhi2, Unigene61474\_JingChaoZuZhi2, Unigene61602\_JingChaoZuZhi2, Unigene61664\_JingChaoZuZhi2, Unigene61728\_JingChaoZuZhi2, Unigene61880\_JingChaoZuZhi2, Unigene61929\_JingChaoZuZhi2, Unigene619\_JingChaoZuZhi2, Unigene62272\_JingChaoZuZhi2, Unigene62376\_JingChaoZuZhi2, Unigene62419\_JingChaoZuZhi2, Unigene6266\_JingChaoZuZhi2, Unigene62762\_JingChaoZuZhi2, Unigene62954\_JingChaoZuZhi2, Unigene63182\_JingChaoZuZhi2, Unigene63229\_JingChaoZuZhi2, Unigene63247\_JingChaoZuZhi2, Unigene63253\_JingChaoZuZhi2, Unigene63394\_JingChaoZuZhi2, Unigene63412\_JingChaoZuZhi2, Unigene63442\_JingChaoZuZhi2, Unigene63562\_JingChaoZuZhi2, Unigene63819\_JingChaoZuZhi2, Unigene64027\_JingChaoZuZhi2, Unigene64059\_JingChaoZuZhi2, Unigene64118\_JingChaoZuZhi2, Unigene64280\_JingChaoZuZhi2, Unigene64329\_JingChaoZuZhi2, Unigene64452\_JingChaoZuZhi2, Unigene64499\_JingChaoZuZhi2, Unigene64690\_JingChaoZuZhi2, Unigene64728\_JingChaoZuZhi2, Unigene64838\_JingChaoZuZhi2, Unigene64903\_JingChaoZuZhi2, Unigene64948\_JingChaoZuZhi2, Unigene65005\_JingChaoZuZhi2, Unigene6519\_JingChaoZuZhi2, Unigene65272\_JingChaoZuZhi2, Unigene65464\_JingChaoZuZhi2, Unigene65662\_JingChaoZuZhi2, Unigene6566\_JingChaoZuZhi2, Unigene65681\_JingChaoZuZhi2, Unigene65856\_JingChaoZuZhi2, Unigene65875\_JingChaoZuZhi2, Unigene65884\_JingChaoZuZhi2, Unigene65950\_JingChaoZuZhi2, Unigene66343\_JingChaoZuZhi2, Unigene66590\_JingChaoZuZhi2, Unigene66765\_JingChaoZuZhi2, Unigene66773\_JingChaoZuZhi2, Unigene668\_JingChaoZuZhi2, Unigene66952\_JingChaoZuZhi2, Unigene67088\_JingChaoZuZhi2, Unigene67194\_JingChaoZuZhi2, Unigene67245\_JingChaoZuZhi2, Unigene67286\_JingChaoZuZhi2, Unigene67525\_JingChaoZuZhi2, Unigene6753\_JingChaoZuZhi2, Unigene6776\_JingChaoZuZhi2, Unigene67958\_JingChaoZuZhi2, Unigene6799\_JingChaoZuZhi2, Unigene68104\_JingChaoZuZhi2, Unigene68174\_JingChaoZuZhi2, Unigene6818\_JingChaoZuZhi2, Unigene6825\_JingChaoZuZhi2, Unigene68307\_JingChaoZuZhi2, Unigene6830\_JingChaoZuZhi2, Unigene68411\_JingChaoZuZhi2, Unigene68547\_JingChaoZuZhi2, Unigene68571\_JingChaoZuZhi2, Unigene68822\_JingChaoZuZhi2, Unigene68977\_JingChaoZuZhi2, Unigene69141\_JingChaoZuZhi2, Unigene6914\_JingChaoZuZhi2, Unigene69178\_JingChaoZuZhi2, Unigene69239\_JingChaoZuZhi2, Unigene69375\_JingChaoZuZhi2, Unigene69395\_JingChaoZuZhi2, Unigene69438\_JingChaoZuZhi2, Unigene69444\_JingChaoZuZhi2, Unigene69535\_JingChaoZuZhi2, Unigene69655\_JingChaoZuZhi2, Unigene69733\_JingChaoZuZhi2, Unigene702\_JingChaoZuZhi2, Unigene70328\_JingChaoZuZhi2, Unigene70929\_JingChaoZuZhi2, Unigene71051\_JingChaoZuZhi2, Unigene7116\_JingChaoZuZhi2, Unigene71260\_JingChaoZuZhi2, Unigene71460\_JingChaoZuZhi2, Unigene71546\_JingChaoZuZhi2, Unigene71650\_JingChaoZuZhi2, Unigene71874\_JingChaoZuZhi2, Unigene71877\_JingChaoZuZhi2, Unigene72099\_JingChaoZuZhi2, Unigene72374\_JingChaoZuZhi2, Unigene72464\_JingChaoZuZhi2, Unigene72466\_JingChaoZuZhi2, Unigene72676\_JingChaoZuZhi2, Unigene72716\_JingChaoZuZhi2, Unigene72754\_JingChaoZuZhi2, Unigene7276\_JingChaoZuZhi2, Unigene72990\_JingChaoZuZhi2, Unigene73015\_JingChaoZuZhi2, Unigene73107\_JingChaoZuZhi2, Unigene73118\_JingChaoZuZhi2, Unigene73133\_JingChaoZuZhi2, Unigene73369\_JingChaoZuZhi2, Unigene73507\_JingChaoZuZhi2, Unigene73641\_JingChaoZuZhi2, Unigene73708\_JingChaoZuZhi2, Unigene7412\_JingChaoZuZhi2, Unigene74216\_JingChaoZuZhi2, Unigene74233\_JingChaoZuZhi2, Unigene74402\_JingChaoZuZhi2, Unigene744\_JingChaoZuZhi2, Unigene74552\_JingChaoZuZhi2, Unigene74607\_JingChaoZuZhi2, Unigene74717\_JingChaoZuZhi2, Unigene74719\_JingChaoZuZhi2, Unigene74785\_JingChaoZuZhi2, Unigene74814\_JingChaoZuZhi2, Unigene74832\_JingChaoZuZhi2, Unigene74900\_JingChaoZuZhi2, Unigene74943\_JingChaoZuZhi2, Unigene75021\_JingChaoZuZhi2, Unigene75096\_JingChaoZuZhi2, Unigene75237\_JingChaoZuZhi2, Unigene75566\_JingChaoZuZhi2, Unigene75699\_JingChaoZuZhi2, Unigene75709\_JingChaoZuZhi2, Unigene75839\_JingChaoZuZhi2, Unigene75926\_JingChaoZuZhi2, Unigene75939\_JingChaoZuZhi2, Unigene75968\_JingChaoZuZhi2, Unigene76142\_JingChaoZuZhi2, Unigene7641\_JingChaoZuZhi2, Unigene76536\_JingChaoZuZhi2, Unigene76545\_JingChaoZuZhi2, Unigene76649\_JingChaoZuZhi2, Unigene76676\_JingChaoZuZhi2, Unigene76753\_JingChaoZuZhi2, Unigene76793\_JingChaoZuZhi2, Unigene76844\_JingChaoZuZhi2, Unigene76914\_JingChaoZuZhi2, Unigene76957\_JingChaoZuZhi2, Unigene76969\_JingChaoZuZhi2, Unigene76993\_JingChaoZuZhi2, Unigene76995\_JingChaoZuZhi2, Unigene77028\_JingChaoZuZhi2, Unigene7716\_JingChaoZuZhi2, Unigene77205\_JingChaoZuZhi2, Unigene77243\_JingChaoZuZhi2, Unigene77298\_JingChaoZuZhi2, Unigene77305\_JingChaoZuZhi2, Unigene77369\_JingChaoZuZhi2, Unigene77446\_JingChaoZuZhi2, Unigene77568\_JingChaoZuZhi2, Unigene77724\_JingChaoZuZhi2, Unigene77885\_JingChaoZuZhi2, Unigene77913\_JingChaoZuZhi2, Unigene78009\_JingChaoZuZhi2, Unigene7800\_JingChaoZuZhi2, Unigene78172\_JingChaoZuZhi2, Unigene7824\_JingChaoZuZhi2, Unigene78288\_JingChaoZuZhi2, Unigene7828\_JingChaoZuZhi2, Unigene78350\_JingChaoZuZhi2, Unigene78410\_JingChaoZuZhi2, Unigene78477\_JingChaoZuZhi2, Unigene78517\_JingChaoZuZhi2, Unigene78547\_JingChaoZuZhi2, Unigene78563\_JingChaoZuZhi2, Unigene78656\_JingChaoZuZhi2, Unigene78707\_JingChaoZuZhi2, Unigene78733\_JingChaoZuZhi2, Unigene79239\_JingChaoZuZhi2, Unigene79333\_JingChaoZuZhi2, Unigene79388\_JingChaoZuZhi2, Unigene79433\_JingChaoZuZhi2, Unigene79468\_JingChaoZuZhi2, Unigene79642\_JingChaoZuZhi2, Unigene7973\_JingChaoZuZhi2, Unigene79800\_JingChaoZuZhi2, Unigene79902\_JingChaoZuZhi2, Unigene79965\_JingChaoZuZhi2, Unigene80137\_JingChaoZuZhi2, Unigene80180\_JingChaoZuZhi2, Unigene80317\_JingChaoZuZhi2, Unigene80449\_JingChaoZuZhi2, Unigene80459\_JingChaoZuZhi2, Unigene80471\_JingChaoZuZhi2, Unigene80481\_JingChaoZuZhi2, Unigene80559\_JingChaoZuZhi2, Unigene80653\_JingChaoZuZhi2, Unigene80836\_JingChaoZuZhi2, Unigene8087\_JingChaoZuZhi2, Unigene80893\_JingChaoZuZhi2, Unigene81075\_JingChaoZuZhi2, Unigene81122\_JingChaoZuZhi2, Unigene81172\_JingChaoZuZhi2, Unigene8123\_JingChaoZuZhi2, Unigene81259\_JingChaoZuZhi2, Unigene81284\_JingChaoZuZhi2, Unigene81359\_JingChaoZuZhi2, Unigene81383\_JingChaoZuZhi2, Unigene81437\_JingChaoZuZhi2, Unigene81440\_JingChaoZuZhi2, Unigene81491\_JingChaoZuZhi2, Unigene81651\_JingChaoZuZhi2, Unigene81664\_JingChaoZuZhi2, Unigene81671\_JingChaoZuZhi2, Unigene81738\_JingChaoZuZhi2, Unigene81803\_JingChaoZuZhi2, Unigene81844\_JingChaoZuZhi2, Unigene81884\_JingChaoZuZhi2, Unigene81955\_JingChaoZuZhi2, Unigene82058\_JingChaoZuZhi2, Unigene82067\_JingChaoZuZhi2, Unigene82089\_JingChaoZuZhi2, Unigene82132\_JingChaoZuZhi2, Unigene821\_JingChaoZuZhi2, Unigene82345\_JingChaoZuZhi2, Unigene82367\_JingChaoZuZhi2, Unigene82396\_JingChaoZuZhi2, Unigene82430\_JingChaoZuZhi2, Unigene82468\_JingChaoZuZhi2, Unigene82513\_JingChaoZuZhi2, Unigene82536\_JingChaoZuZhi2, Unigene8280\_JingChaoZuZhi2, Unigene82836\_JingChaoZuZhi2, Unigene828\_JingChaoZuZhi2, Unigene83068\_JingChaoZuZhi2, Unigene83092\_JingChaoZuZhi2, Unigene83105\_JingChaoZuZhi2, Unigene83176\_JingChaoZuZhi2, Unigene83280\_JingChaoZuZhi2, Unigene83285\_JingChaoZuZhi2, Unigene83405\_JingChaoZuZhi2, Unigene83494\_JingChaoZuZhi2, Unigene83524\_JingChaoZuZhi2, Unigene83578\_JingChaoZuZhi2, Unigene83584\_JingChaoZuZhi2, Unigene83611\_JingChaoZuZhi2, Unigene83620\_JingChaoZuZhi2, Unigene83631\_JingChaoZuZhi2, Unigene83639\_JingChaoZuZhi2, Unigene83678\_JingChaoZuZhi2, Unigene83699\_JingChaoZuZhi2, Unigene83732\_JingChaoZuZhi2, Unigene83770\_JingChaoZuZhi2, Unigene83796\_JingChaoZuZhi2, Unigene83881\_JingChaoZuZhi2, Unigene84032\_JingChaoZuZhi2, Unigene84075\_JingChaoZuZhi2, Unigene84199\_JingChaoZuZhi2, Unigene84212\_JingChaoZuZhi2, Unigene84235\_JingChaoZuZhi2, Unigene84246\_JingChaoZuZhi2, Unigene84517\_JingChaoZuZhi2, Unigene84564\_JingChaoZuZhi2, Unigene84566\_JingChaoZuZhi2, Unigene84576\_JingChaoZuZhi2, Unigene84628\_JingChaoZuZhi2, Unigene84638\_JingChaoZuZhi2, Unigene8464\_JingChaoZuZhi2, Unigene84745\_JingChaoZuZhi2, Unigene84774\_JingChaoZuZhi2, Unigene84804\_JingChaoZuZhi2, Unigene84835\_JingChaoZuZhi2, Unigene84960\_JingChaoZuZhi2, Unigene84972\_JingChaoZuZhi2, Unigene84993\_JingChaoZuZhi2, Unigene8500\_JingChaoZuZhi2, Unigene85033\_JingChaoZuZhi2, Unigene85076\_JingChaoZuZhi2, Unigene85142\_JingChaoZuZhi2, Unigene85168\_JingChaoZuZhi2, Unigene85208\_JingChaoZuZhi2, Unigene85214\_JingChaoZuZhi2, Unigene85239\_JingChaoZuZhi2, Unigene85334\_JingChaoZuZhi2, Unigene85338\_JingChaoZuZhi2, Unigene85377\_JingChaoZuZhi2, Unigene85383\_JingChaoZuZhi2, Unigene8538\_JingChaoZuZhi2, Unigene85431\_JingChaoZuZhi2, Unigene85486\_JingChaoZuZhi2, Unigene85560\_JingChaoZuZhi2, Unigene858\_JingChaoZuZhi2, Unigene85942\_JingChaoZuZhi2, Unigene85961\_JingChaoZuZhi2, Unigene85992\_JingChaoZuZhi2, Unigene86020\_JingChaoZuZhi2, Unigene86030\_JingChaoZuZhi2, Unigene86114\_JingChaoZuZhi2, Unigene86230\_JingChaoZuZhi2, Unigene86291\_JingChaoZuZhi2, Unigene86293\_JingChaoZuZhi2, Unigene862\_JingChaoZuZhi2, Unigene86309\_JingChaoZuZhi2, Unigene86316\_JingChaoZuZhi2, Unigene86387\_JingChaoZuZhi2, Unigene86402\_JingChaoZuZhi2, Unigene86471\_JingChaoZuZhi2, Unigene86662\_JingChaoZuZhi2, Unigene86830\_JingChaoZuZhi2, Unigene86840\_JingChaoZuZhi2, Unigene87023\_JingChaoZuZhi2, Unigene870\_JingChaoZuZhi2, Unigene87105\_JingChaoZuZhi2, Unigene87118\_JingChaoZuZhi2, Unigene8711\_JingChaoZuZhi2, Unigene87145\_JingChaoZuZhi2, Unigene87217\_JingChaoZuZhi2, Unigene87238\_JingChaoZuZhi2, Unigene87319\_JingChaoZuZhi2, Unigene87455\_JingChaoZuZhi2, Unigene87478\_JingChaoZuZhi2, Unigene87506\_JingChaoZuZhi2, Unigene87556\_JingChaoZuZhi2, Unigene87619\_JingChaoZuZhi2, Unigene8762\_JingChaoZuZhi2, Unigene87644\_JingChaoZuZhi2, Unigene87689\_JingChaoZuZhi2, Unigene87861\_JingChaoZuZhi2, Unigene87882\_JingChaoZuZhi2, Unigene87922\_JingChaoZuZhi2, Unigene88010\_JingChaoZuZhi2, Unigene88063\_JingChaoZuZhi2, Unigene88064\_JingChaoZuZhi2, Unigene88072\_JingChaoZuZhi2, Unigene88162\_JingChaoZuZhi2, Unigene88220\_JingChaoZuZhi2, Unigene88351\_JingChaoZuZhi2, Unigene88361\_JingChaoZuZhi2, Unigene88444\_JingChaoZuZhi2, Unigene88447\_JingChaoZuZhi2, Unigene88450\_JingChaoZuZhi2, Unigene88482\_JingChaoZuZhi2, Unigene88503\_JingChaoZuZhi2, Unigene88581\_JingChaoZuZhi2, Unigene88590\_JingChaoZuZhi2, Unigene88686\_JingChaoZuZhi2, Unigene88779\_JingChaoZuZhi2, Unigene88797\_JingChaoZuZhi2, Unigene88812\_JingChaoZuZhi2, Unigene88837\_JingChaoZuZhi2, Unigene88921\_JingChaoZuZhi2, Unigene88972\_JingChaoZuZhi2, Unigene88997\_JingChaoZuZhi2, Unigene89031\_JingChaoZuZhi2, Unigene89072\_JingChaoZuZhi2, Unigene89106\_JingChaoZuZhi2, Unigene89120\_JingChaoZuZhi2, Unigene89174\_JingChaoZuZhi2, Unigene89244\_JingChaoZuZhi2, Unigene89249\_JingChaoZuZhi2, Unigene89276\_JingChaoZuZhi2, Unigene89287\_JingChaoZuZhi2, Unigene89293\_JingChaoZuZhi2, Unigene89308\_JingChaoZuZhi2, Unigene89422\_JingChaoZuZhi2, Unigene89442\_JingChaoZuZhi2, Unigene89454\_JingChaoZuZhi2, Unigene89636\_JingChaoZuZhi2, Unigene89655\_JingChaoZuZhi2, Unigene89703\_JingChaoZuZhi2, Unigene89712\_JingChaoZuZhi2, Unigene89736\_JingChaoZuZhi2, Unigene89785\_JingChaoZuZhi2, Unigene89804\_JingChaoZuZhi2, Unigene89908\_JingChaoZuZhi2, Unigene90009\_JingChaoZuZhi2, Unigene9000\_JingChaoZuZhi2, Unigene90159\_JingChaoZuZhi2, Unigene90174\_JingChaoZuZhi2, Unigene90209\_JingChaoZuZhi2, Unigene90265\_JingChaoZuZhi2, Unigene90303\_JingChaoZuZhi2, Unigene90324\_JingChaoZuZhi2, Unigene90379\_JingChaoZuZhi2, Unigene90391\_JingChaoZuZhi2, Unigene90419\_JingChaoZuZhi2, Unigene90443\_JingChaoZuZhi2, Unigene90459\_JingChaoZuZhi2, Unigene90463\_JingChaoZuZhi2, Unigene90484\_JingChaoZuZhi2, Unigene90537\_JingChaoZuZhi2, Unigene90547\_JingChaoZuZhi2, Unigene90606\_JingChaoZuZhi2, Unigene90620\_JingChaoZuZhi2, Unigene90682\_JingChaoZuZhi2, Unigene90900\_JingChaoZuZhi2, Unigene90932\_JingChaoZuZhi2, Unigene91003\_JingChaoZuZhi2, Unigene91166\_JingChaoZuZhi2, Unigene9119\_JingChaoZuZhi2, Unigene91266\_JingChaoZuZhi2, Unigene91307\_JingChaoZuZhi2, Unigene91361\_JingChaoZuZhi2, Unigene91427\_JingChaoZuZhi2, Unigene91449\_JingChaoZuZhi2, Unigene91482\_JingChaoZuZhi2, Unigene91500\_JingChaoZuZhi2, Unigene91518\_JingChaoZuZhi2, Unigene91628\_JingChaoZuZhi2, Unigene91703\_JingChaoZuZhi2, Unigene91710\_JingChaoZuZhi2, Unigene9173\_JingChaoZuZhi2, Unigene91746\_JingChaoZuZhi2, Unigene91789\_JingChaoZuZhi2, Unigene91814\_JingChaoZuZhi2, Unigene91862\_JingChaoZuZhi2, Unigene91871\_JingChaoZuZhi2, Unigene91895\_JingChaoZuZhi2, Unigene91912\_JingChaoZuZhi2, Unigene91931\_JingChaoZuZhi2, Unigene91944\_JingChaoZuZhi2, Unigene91948\_JingChaoZuZhi2, Unigene91951\_JingChaoZuZhi2, Unigene91980\_JingChaoZuZhi2, Unigene92082\_JingChaoZuZhi2, Unigene92093\_JingChaoZuZhi2, Unigene9212\_JingChaoZuZhi2, Unigene92204\_JingChaoZuZhi2, Unigene92230\_JingChaoZuZhi2, Unigene92249\_JingChaoZuZhi2, Unigene92293\_JingChaoZuZhi2, Unigene92304\_JingChaoZuZhi2, Unigene92313\_JingChaoZuZhi2, Unigene92315\_JingChaoZuZhi2, Unigene92328\_JingChaoZuZhi2, Unigene92374\_JingChaoZuZhi2, Unigene92403\_JingChaoZuZhi2, Unigene92491\_JingChaoZuZhi2, Unigene92545\_JingChaoZuZhi2, Unigene92589\_JingChaoZuZhi2, Unigene92606\_JingChaoZuZhi2, Unigene92650\_JingChaoZuZhi2, Unigene9266\_JingChaoZuZhi2, Unigene92687\_JingChaoZuZhi2, Unigene92716\_JingChaoZuZhi2, Unigene92727\_JingChaoZuZhi2, Unigene92788\_JingChaoZuZhi2, Unigene92792\_JingChaoZuZhi2, Unigene92832\_JingChaoZuZhi2, Unigene92889\_JingChaoZuZhi2, Unigene92945\_JingChaoZuZhi2, Unigene92986\_JingChaoZuZhi2, Unigene92989\_JingChaoZuZhi2, Unigene93073\_JingChaoZuZhi2, Unigene9308\_JingChaoZuZhi2, Unigene93117\_JingChaoZuZhi2, Unigene9321\_JingChaoZuZhi2, Unigene93239\_JingChaoZuZhi2, Unigene93250\_JingChaoZuZhi2, Unigene93251\_JingChaoZuZhi2, Unigene93352\_JingChaoZuZhi2, Unigene93370\_JingChaoZuZhi2, Unigene93493\_JingChaoZuZhi2, Unigene93515\_JingChaoZuZhi2, Unigene93558\_JingChaoZuZhi2, Unigene93647\_JingChaoZuZhi2, Unigene9365\_JingChaoZuZhi2, Unigene93683\_JingChaoZuZhi2, Unigene93703\_JingChaoZuZhi2, Unigene93730\_JingChaoZuZhi2, Unigene93732\_JingChaoZuZhi2, Unigene93784\_JingChaoZuZhi2, Unigene93786\_JingChaoZuZhi2, Unigene93834\_JingChaoZuZhi2, Unigene93853\_JingChaoZuZhi2, Unigene93884\_JingChaoZuZhi2, Unigene93904\_JingChaoZuZhi2, Unigene93957\_JingChaoZuZhi2, Unigene93958\_JingChaoZuZhi2, Unigene93973\_JingChaoZuZhi2, Unigene93987\_JingChaoZuZhi2, Unigene93991\_JingChaoZuZhi2, Unigene93998\_JingChaoZuZhi2, Unigene94026\_JingChaoZuZhi2, Unigene94035\_JingChaoZuZhi2, Unigene94051\_JingChaoZuZhi2, Unigene94062\_JingChaoZuZhi2, Unigene9406\_JingChaoZuZhi2, Unigene94090\_JingChaoZuZhi2, Unigene94122\_JingChaoZuZhi2, Unigene94154\_JingChaoZuZhi2, Unigene94224\_JingChaoZuZhi2, Unigene94232\_JingChaoZuZhi2, Unigene94233\_JingChaoZuZhi2, Unigene94246\_JingChaoZuZhi2, Unigene94304\_JingChaoZuZhi2, Unigene94336\_JingChaoZuZhi2, Unigene94341\_JingChaoZuZhi2, Unigene94342\_JingChaoZuZhi2, Unigene94362\_JingChaoZuZhi2, Unigene94374\_JingChaoZuZhi2, Unigene94395\_JingChaoZuZhi2, Unigene94404\_JingChaoZuZhi2, Unigene94413\_JingChaoZuZhi2, Unigene94471\_JingChaoZuZhi2, Unigene94483\_JingChaoZuZhi2, Unigene94498\_JingChaoZuZhi2, Unigene94517\_JingChaoZuZhi2, Unigene94533\_JingChaoZuZhi2, Unigene94548\_JingChaoZuZhi2, Unigene94566\_JingChaoZuZhi2, Unigene94570\_JingChaoZuZhi2, Unigene94603\_JingChaoZuZhi2, Unigene94635\_JingChaoZuZhi2, Unigene94695\_JingChaoZuZhi2, Unigene94701\_JingChaoZuZhi2, Unigene94721\_JingChaoZuZhi2, Unigene94730\_JingChaoZuZhi2, Unigene94774\_JingChaoZuZhi2, Unigene94778\_JingChaoZuZhi2, Unigene94789\_JingChaoZuZhi2, Unigene94790\_JingChaoZuZhi2, Unigene94808\_JingChaoZuZhi2, Unigene94811\_JingChaoZuZhi2, Unigene94915\_JingChaoZuZhi2, Unigene94990\_JingChaoZuZhi2, Unigene95015\_JingChaoZuZhi2, Unigene95068\_JingChaoZuZhi2, Unigene95158\_JingChaoZuZhi2, Unigene95162\_JingChaoZuZhi2, Unigene95196\_JingChaoZuZhi2, Unigene95201\_JingChaoZuZhi2, Unigene95202\_JingChaoZuZhi2, Unigene95267\_JingChaoZuZhi2, Unigene95284\_JingChaoZuZhi2, Unigene95287\_JingChaoZuZhi2, Unigene95297\_JingChaoZuZhi2, Unigene95311\_JingChaoZuZhi2, Unigene95334\_JingChaoZuZhi2, Unigene95335\_JingChaoZuZhi2, Unigene95371\_JingChaoZuZhi2, Unigene95427\_JingChaoZuZhi2, Unigene95465\_JingChaoZuZhi2, Unigene95581\_JingChaoZuZhi2, Unigene95597\_JingChaoZuZhi2, Unigene95624\_JingChaoZuZhi2, Unigene95644\_JingChaoZuZhi2, Unigene95654\_JingChaoZuZhi2, Unigene95714\_JingChaoZuZhi2, Unigene9574\_JingChaoZuZhi2, Unigene95752\_JingChaoZuZhi2, Unigene95783\_JingChaoZuZhi2, Unigene95784\_JingChaoZuZhi2, Unigene95831\_JingChaoZuZhi2, Unigene95897\_JingChaoZuZhi2, Unigene95903\_JingChaoZuZhi2, Unigene95904\_JingChaoZuZhi2, Unigene95932\_JingChaoZuZhi2, Unigene95984\_JingChaoZuZhi2, Unigene96014\_JingChaoZuZhi2, Unigene96034\_JingChaoZuZhi2, Unigene96035\_JingChaoZuZhi2, Unigene96051\_JingChaoZuZhi2, Unigene96055\_JingChaoZuZhi2, Unigene96066\_JingChaoZuZhi2, Unigene96076\_JingChaoZuZhi2, Unigene96082\_JingChaoZuZhi2, Unigene96132\_JingChaoZuZhi2, Unigene96136\_JingChaoZuZhi2, Unigene96140\_JingChaoZuZhi2, Unigene96176\_JingChaoZuZhi2, Unigene96180\_JingChaoZuZhi2, Unigene96195\_JingChaoZuZhi2, Unigene96219\_JingChaoZuZhi2, Unigene96254\_JingChaoZuZhi2, Unigene9626\_JingChaoZuZhi2, Unigene9721\_JingChaoZuZhi2, Unigene9746\_JingChaoZuZhi2, Unigene9788\_JingChaoZuZhi2, Unigene9789\_JingChaoZuZhi2, Unigene9791\_JingChaoZuZhi2, Unigene9804\_JingChaoZuZhi2, Unigene9929\_JingChaoZuZhi2, Unigene9948\_JingChaoZuZhi2, Unigene9953\_JingChaoZuZhi2, Unigene9972\_JingChaoZuZhi2, Unigene9992\_JingChaoZuZhi2 |
| 4 | Amoebiasis | Unigene10078\_JingChaoZuZhi2, Unigene10128\_JingChaoZuZhi2, Unigene10149\_JingChaoZuZhi2, Unigene10318\_JingChaoZuZhi2, Unigene10320\_JingChaoZuZhi2, Unigene10362\_JingChaoZuZhi2, Unigene10368\_JingChaoZuZhi2, Unigene10380\_JingChaoZuZhi2, Unigene10597\_JingChaoZuZhi2, Unigene10630\_JingChaoZuZhi2, Unigene10758\_JingChaoZuZhi2, Unigene10999\_JingChaoZuZhi2, Unigene11048\_JingChaoZuZhi2, Unigene11060\_JingChaoZuZhi2, Unigene11064\_JingChaoZuZhi2, Unigene11095\_JingChaoZuZhi2, Unigene11125\_JingChaoZuZhi2, Unigene11130\_JingChaoZuZhi2, Unigene1115\_JingChaoZuZhi2, Unigene11320\_JingChaoZuZhi2, Unigene11408\_JingChaoZuZhi2, Unigene11483\_JingChaoZuZhi2, Unigene11524\_JingChaoZuZhi2, Unigene1154\_JingChaoZuZhi2, Unigene11560\_JingChaoZuZhi2, Unigene11668\_JingChaoZuZhi2, Unigene11747\_JingChaoZuZhi2, Unigene11830\_JingChaoZuZhi2, Unigene11889\_JingChaoZuZhi2, Unigene11961\_JingChaoZuZhi2, Unigene11969\_JingChaoZuZhi2, Unigene11982\_JingChaoZuZhi2, Unigene11996\_JingChaoZuZhi2, Unigene12011\_JingChaoZuZhi2, Unigene12052\_JingChaoZuZhi2, Unigene12066\_JingChaoZuZhi2, Unigene12091\_JingChaoZuZhi2, Unigene12193\_JingChaoZuZhi2, Unigene12244\_JingChaoZuZhi2, Unigene12253\_JingChaoZuZhi2, Unigene12296\_JingChaoZuZhi2, Unigene12297\_JingChaoZuZhi2, Unigene12311\_JingChaoZuZhi2, Unigene12327\_JingChaoZuZhi2, Unigene12329\_JingChaoZuZhi2, Unigene12361\_JingChaoZuZhi2, Unigene12376\_JingChaoZuZhi2, Unigene12543\_JingChaoZuZhi2, Unigene1256\_JingChaoZuZhi2, Unigene12595\_JingChaoZuZhi2, Unigene12652\_JingChaoZuZhi2, Unigene12655\_JingChaoZuZhi2, Unigene12665\_JingChaoZuZhi2, Unigene12669\_JingChaoZuZhi2, Unigene12728\_JingChaoZuZhi2, Unigene12850\_JingChaoZuZhi2, Unigene12897\_JingChaoZuZhi2, Unigene12904\_JingChaoZuZhi2, Unigene12906\_JingChaoZuZhi2, Unigene12912\_JingChaoZuZhi2, Unigene12913\_JingChaoZuZhi2, Unigene12972\_JingChaoZuZhi2, Unigene12985\_JingChaoZuZhi2, Unigene13016\_JingChaoZuZhi2, Unigene13036\_JingChaoZuZhi2, Unigene13078\_JingChaoZuZhi2, Unigene13112\_JingChaoZuZhi2, Unigene13225\_JingChaoZuZhi2, Unigene13228\_JingChaoZuZhi2, Unigene13269\_JingChaoZuZhi2, Unigene13297\_JingChaoZuZhi2, Unigene13381\_JingChaoZuZhi2, Unigene13389\_JingChaoZuZhi2, Unigene13390\_JingChaoZuZhi2, Unigene13464\_JingChaoZuZhi2, Unigene13487\_JingChaoZuZhi2, Unigene13493\_JingChaoZuZhi2, Unigene13607\_JingChaoZuZhi2, Unigene13720\_JingChaoZuZhi2, Unigene13770\_JingChaoZuZhi2, Unigene13864\_JingChaoZuZhi2, Unigene13866\_JingChaoZuZhi2, Unigene13875\_JingChaoZuZhi2, Unigene13880\_JingChaoZuZhi2, Unigene13890\_JingChaoZuZhi2, Unigene13914\_JingChaoZuZhi2, Unigene13925\_JingChaoZuZhi2, Unigene13941\_JingChaoZuZhi2, Unigene13961\_JingChaoZuZhi2, Unigene13966\_JingChaoZuZhi2, Unigene13975\_JingChaoZuZhi2, Unigene13978\_JingChaoZuZhi2, Unigene13992\_JingChaoZuZhi2, Unigene14056\_JingChaoZuZhi2, Unigene14074\_JingChaoZuZhi2, Unigene14109\_JingChaoZuZhi2, Unigene1413\_JingChaoZuZhi2, Unigene14252\_JingChaoZuZhi2, Unigene14322\_JingChaoZuZhi2, Unigene14338\_JingChaoZuZhi2, Unigene14360\_JingChaoZuZhi2, Unigene1437\_JingChaoZuZhi2, Unigene14393\_JingChaoZuZhi2, Unigene14409\_JingChaoZuZhi2, Unigene14438\_JingChaoZuZhi2, Unigene14492\_JingChaoZuZhi2, Unigene14610\_JingChaoZuZhi2, Unigene14684\_JingChaoZuZhi2, Unigene14706\_JingChaoZuZhi2, Unigene14717\_JingChaoZuZhi2, Unigene14770\_JingChaoZuZhi2, Unigene14792\_JingChaoZuZhi2, Unigene147\_JingChaoZuZhi2, Unigene14903\_JingChaoZuZhi2, Unigene14912\_JingChaoZuZhi2, Unigene14914\_JingChaoZuZhi2, Unigene14928\_JingChaoZuZhi2, Unigene14948\_JingChaoZuZhi2, Unigene14963\_JingChaoZuZhi2, Unigene15010\_JingChaoZuZhi2, Unigene15088\_JingChaoZuZhi2, Unigene15129\_JingChaoZuZhi2, Unigene15146\_JingChaoZuZhi2, Unigene15160\_JingChaoZuZhi2, Unigene15228\_JingChaoZuZhi2, Unigene15229\_JingChaoZuZhi2, Unigene15276\_JingChaoZuZhi2, Unigene15304\_JingChaoZuZhi2, Unigene15331\_JingChaoZuZhi2, Unigene15475\_JingChaoZuZhi2, Unigene15536\_JingChaoZuZhi2, Unigene15651\_JingChaoZuZhi2, Unigene15677\_JingChaoZuZhi2, Unigene15763\_JingChaoZuZhi2, Unigene15803\_JingChaoZuZhi2, Unigene15814\_JingChaoZuZhi2, Unigene15840\_JingChaoZuZhi2, Unigene15879\_JingChaoZuZhi2, Unigene16008\_JingChaoZuZhi2, Unigene16064\_JingChaoZuZhi2, Unigene1606\_JingChaoZuZhi2, Unigene16072\_JingChaoZuZhi2, Unigene1616\_JingChaoZuZhi2, Unigene16240\_JingChaoZuZhi2, Unigene16266\_JingChaoZuZhi2, Unigene16283\_JingChaoZuZhi2, Unigene16300\_JingChaoZuZhi2, Unigene16312\_JingChaoZuZhi2, Unigene16410\_JingChaoZuZhi2, Unigene16578\_JingChaoZuZhi2, Unigene16591\_JingChaoZuZhi2, Unigene16596\_JingChaoZuZhi2, Unigene16780\_JingChaoZuZhi2, Unigene16785\_JingChaoZuZhi2, Unigene16791\_JingChaoZuZhi2, Unigene16833\_JingChaoZuZhi2, Unigene16844\_JingChaoZuZhi2, Unigene16855\_JingChaoZuZhi2, Unigene16903\_JingChaoZuZhi2, Unigene16970\_JingChaoZuZhi2, Unigene16991\_JingChaoZuZhi2, Unigene169\_JingChaoZuZhi2, Unigene17015\_JingChaoZuZhi2, Unigene17026\_JingChaoZuZhi2, Unigene1710\_JingChaoZuZhi2, Unigene17190\_JingChaoZuZhi2, Unigene1719\_JingChaoZuZhi2, Unigene17219\_JingChaoZuZhi2, Unigene17243\_JingChaoZuZhi2, Unigene17247\_JingChaoZuZhi2, Unigene17280\_JingChaoZuZhi2, Unigene17288\_JingChaoZuZhi2, Unigene17382\_JingChaoZuZhi2, Unigene17425\_JingChaoZuZhi2, Unigene1746\_JingChaoZuZhi2, Unigene1749\_JingChaoZuZhi2, Unigene17501\_JingChaoZuZhi2, Unigene17506\_JingChaoZuZhi2, Unigene17513\_JingChaoZuZhi2, Unigene17537\_JingChaoZuZhi2, Unigene17605\_JingChaoZuZhi2, Unigene17675\_JingChaoZuZhi2, Unigene17735\_JingChaoZuZhi2, Unigene17754\_JingChaoZuZhi2, Unigene17758\_JingChaoZuZhi2, Unigene17764\_JingChaoZuZhi2, Unigene17810\_JingChaoZuZhi2, Unigene17983\_JingChaoZuZhi2, Unigene18042\_JingChaoZuZhi2, Unigene18075\_JingChaoZuZhi2, Unigene18089\_JingChaoZuZhi2, Unigene18101\_JingChaoZuZhi2, Unigene18107\_JingChaoZuZhi2, Unigene18126\_JingChaoZuZhi2, Unigene18211\_JingChaoZuZhi2, Unigene18292\_JingChaoZuZhi2, Unigene18307\_JingChaoZuZhi2, Unigene18333\_JingChaoZuZhi2, Unigene18361\_JingChaoZuZhi2, Unigene18375\_JingChaoZuZhi2, Unigene18435\_JingChaoZuZhi2, Unigene18441\_JingChaoZuZhi2, Unigene18465\_JingChaoZuZhi2, Unigene18483\_JingChaoZuZhi2, Unigene18488\_JingChaoZuZhi2, Unigene18515\_JingChaoZuZhi2, Unigene18588\_JingChaoZuZhi2, Unigene18656\_JingChaoZuZhi2, Unigene18672\_JingChaoZuZhi2, Unigene18682\_JingChaoZuZhi2, Unigene18683\_JingChaoZuZhi2, Unigene18730\_JingChaoZuZhi2, Unigene18733\_JingChaoZuZhi2, Unigene18780\_JingChaoZuZhi2, Unigene18781\_JingChaoZuZhi2, Unigene19049\_JingChaoZuZhi2, Unigene19143\_JingChaoZuZhi2, Unigene19207\_JingChaoZuZhi2, Unigene19339\_JingChaoZuZhi2, Unigene19349\_JingChaoZuZhi2, Unigene19365\_JingChaoZuZhi2, Unigene19459\_JingChaoZuZhi2, Unigene19511\_JingChaoZuZhi2, Unigene19512\_JingChaoZuZhi2, Unigene19618\_JingChaoZuZhi2, Unigene19640\_JingChaoZuZhi2, Unigene19662\_JingChaoZuZhi2, Unigene19742\_JingChaoZuZhi2, Unigene19768\_JingChaoZuZhi2, Unigene19790\_JingChaoZuZhi2, Unigene19800\_JingChaoZuZhi2, Unigene19879\_JingChaoZuZhi2, Unigene19908\_JingChaoZuZhi2, Unigene19948\_JingChaoZuZhi2, Unigene19961\_JingChaoZuZhi2, Unigene19973\_JingChaoZuZhi2, Unigene20051\_JingChaoZuZhi2, Unigene20067\_JingChaoZuZhi2, Unigene20101\_JingChaoZuZhi2, Unigene20130\_JingChaoZuZhi2, Unigene20141\_JingChaoZuZhi2, Unigene20204\_JingChaoZuZhi2, Unigene20211\_JingChaoZuZhi2, Unigene20243\_JingChaoZuZhi2, Unigene20255\_JingChaoZuZhi2, Unigene20298\_JingChaoZuZhi2, Unigene20365\_JingChaoZuZhi2, Unigene20367\_JingChaoZuZhi2, Unigene20407\_JingChaoZuZhi2, Unigene20433\_JingChaoZuZhi2, Unigene20529\_JingChaoZuZhi2, Unigene20575\_JingChaoZuZhi2, Unigene20641\_JingChaoZuZhi2, Unigene20646\_JingChaoZuZhi2, Unigene20705\_JingChaoZuZhi2, Unigene20716\_JingChaoZuZhi2, Unigene20748\_JingChaoZuZhi2, Unigene20768\_JingChaoZuZhi2, Unigene20773\_JingChaoZuZhi2, Unigene20794\_JingChaoZuZhi2, Unigene20796\_JingChaoZuZhi2, Unigene20805\_JingChaoZuZhi2, Unigene20843\_JingChaoZuZhi2, Unigene20892\_JingChaoZuZhi2, Unigene20893\_JingChaoZuZhi2, Unigene20913\_JingChaoZuZhi2, Unigene20921\_JingChaoZuZhi2, Unigene20937\_JingChaoZuZhi2, Unigene20942\_JingChaoZuZhi2, Unigene20973\_JingChaoZuZhi2, Unigene21016\_JingChaoZuZhi2, Unigene21023\_JingChaoZuZhi2, Unigene21028\_JingChaoZuZhi2, Unigene21108\_JingChaoZuZhi2, Unigene21118\_JingChaoZuZhi2, Unigene21137\_JingChaoZuZhi2, Unigene21175\_JingChaoZuZhi2, Unigene21233\_JingChaoZuZhi2, Unigene21253\_JingChaoZuZhi2, Unigene21399\_JingChaoZuZhi2, Unigene21401\_JingChaoZuZhi2, Unigene21443\_JingChaoZuZhi2, Unigene21460\_JingChaoZuZhi2, Unigene21472\_JingChaoZuZhi2, Unigene21479\_JingChaoZuZhi2, Unigene21482\_JingChaoZuZhi2, Unigene21490\_JingChaoZuZhi2, Unigene21524\_JingChaoZuZhi2, Unigene21531\_JingChaoZuZhi2, Unigene21550\_JingChaoZuZhi2, Unigene21556\_JingChaoZuZhi2, Unigene21571\_JingChaoZuZhi2, Unigene21575\_JingChaoZuZhi2, Unigene21578\_JingChaoZuZhi2, Unigene21602\_JingChaoZuZhi2, Unigene21609\_JingChaoZuZhi2, Unigene21623\_JingChaoZuZhi2, Unigene21658\_JingChaoZuZhi2, Unigene21697\_JingChaoZuZhi2, Unigene21723\_JingChaoZuZhi2, Unigene21724\_JingChaoZuZhi2, Unigene2173\_JingChaoZuZhi2, Unigene21780\_JingChaoZuZhi2, Unigene21804\_JingChaoZuZhi2, Unigene21815\_JingChaoZuZhi2, Unigene2184\_JingChaoZuZhi2, Unigene21863\_JingChaoZuZhi2, Unigene21888\_JingChaoZuZhi2, Unigene21963\_JingChaoZuZhi2, Unigene21973\_JingChaoZuZhi2, Unigene22024\_JingChaoZuZhi2, Unigene22057\_JingChaoZuZhi2, Unigene22071\_JingChaoZuZhi2, Unigene22073\_JingChaoZuZhi2, Unigene22075\_JingChaoZuZhi2, Unigene22193\_JingChaoZuZhi2, Unigene22201\_JingChaoZuZhi2, Unigene22221\_JingChaoZuZhi2, Unigene2223\_JingChaoZuZhi2, Unigene22245\_JingChaoZuZhi2, Unigene22277\_JingChaoZuZhi2, Unigene22278\_JingChaoZuZhi2, Unigene22305\_JingChaoZuZhi2, Unigene22310\_JingChaoZuZhi2, Unigene22313\_JingChaoZuZhi2, Unigene22396\_JingChaoZuZhi2, Unigene22399\_JingChaoZuZhi2, Unigene22418\_JingChaoZuZhi2, Unigene22419\_JingChaoZuZhi2, Unigene22420\_JingChaoZuZhi2, Unigene22426\_JingChaoZuZhi2, Unigene22430\_JingChaoZuZhi2, Unigene22442\_JingChaoZuZhi2, Unigene22445\_JingChaoZuZhi2, Unigene22455\_JingChaoZuZhi2, Unigene22464\_JingChaoZuZhi2, Unigene22472\_JingChaoZuZhi2, Unigene22491\_JingChaoZuZhi2, Unigene2250\_JingChaoZuZhi2, Unigene22517\_JingChaoZuZhi2, Unigene22530\_JingChaoZuZhi2, Unigene22538\_JingChaoZuZhi2, Unigene22556\_JingChaoZuZhi2, Unigene22572\_JingChaoZuZhi2, Unigene22574\_JingChaoZuZhi2, Unigene22594\_JingChaoZuZhi2, Unigene22624\_JingChaoZuZhi2, Unigene22637\_JingChaoZuZhi2, Unigene22638\_JingChaoZuZhi2, Unigene22640\_JingChaoZuZhi2, Unigene22643\_JingChaoZuZhi2, Unigene22647\_JingChaoZuZhi2, Unigene22686\_JingChaoZuZhi2, Unigene22743\_JingChaoZuZhi2, Unigene22770\_JingChaoZuZhi2, Unigene22779\_JingChaoZuZhi2, Unigene22788\_JingChaoZuZhi2, Unigene22794\_JingChaoZuZhi2, Unigene22828\_JingChaoZuZhi2, Unigene22839\_JingChaoZuZhi2, Unigene22851\_JingChaoZuZhi2, Unigene22852\_JingChaoZuZhi2, Unigene22857\_JingChaoZuZhi2, Unigene22863\_JingChaoZuZhi2, Unigene22865\_JingChaoZuZhi2, Unigene22880\_JingChaoZuZhi2, Unigene22886\_JingChaoZuZhi2, Unigene22901\_JingChaoZuZhi2, Unigene22908\_JingChaoZuZhi2, Unigene22917\_JingChaoZuZhi2, Unigene22920\_JingChaoZuZhi2, Unigene22922\_JingChaoZuZhi2, Unigene22925\_JingChaoZuZhi2, Unigene22928\_JingChaoZuZhi2, Unigene22931\_JingChaoZuZhi2, Unigene229\_JingChaoZuZhi2, Unigene2324\_JingChaoZuZhi2, Unigene2342\_JingChaoZuZhi2, Unigene2345\_JingChaoZuZhi2, Unigene2379\_JingChaoZuZhi2, Unigene2456\_JingChaoZuZhi2, Unigene24772\_JingChaoZuZhi2, Unigene25014\_JingChaoZuZhi2, Unigene2517\_JingChaoZuZhi2, Unigene2525\_JingChaoZuZhi2, Unigene25310\_JingChaoZuZhi2, Unigene26438\_JingChaoZuZhi2, Unigene26496\_JingChaoZuZhi2, Unigene26730\_JingChaoZuZhi2, Unigene26782\_JingChaoZuZhi2, Unigene2721\_JingChaoZuZhi2, Unigene27933\_JingChaoZuZhi2, Unigene27\_JingChaoZuZhi2, Unigene28208\_JingChaoZuZhi2, Unigene28467\_JingChaoZuZhi2, Unigene29201\_JingChaoZuZhi2, Unigene29488\_JingChaoZuZhi2, Unigene2960\_JingChaoZuZhi2, Unigene31090\_JingChaoZuZhi2, Unigene3176\_JingChaoZuZhi2, Unigene3206\_JingChaoZuZhi2, Unigene324\_JingChaoZuZhi2, Unigene32827\_JingChaoZuZhi2, Unigene3286\_JingChaoZuZhi2, Unigene3300\_JingChaoZuZhi2, Unigene3324\_JingChaoZuZhi2, Unigene332\_JingChaoZuZhi2, Unigene3362\_JingChaoZuZhi2, Unigene3422\_JingChaoZuZhi2, Unigene34589\_JingChaoZuZhi2, Unigene3469\_JingChaoZuZhi2, Unigene34807\_JingChaoZuZhi2, Unigene34929\_JingChaoZuZhi2, Unigene35624\_JingChaoZuZhi2, Unigene36479\_JingChaoZuZhi2, Unigene3763\_JingChaoZuZhi2, Unigene3799\_JingChaoZuZhi2, Unigene3872\_JingChaoZuZhi2, Unigene39325\_JingChaoZuZhi2, Unigene39674\_JingChaoZuZhi2, Unigene39832\_JingChaoZuZhi2, Unigene3994\_JingChaoZuZhi2, Unigene41089\_JingChaoZuZhi2, Unigene4112\_JingChaoZuZhi2, Unigene41333\_JingChaoZuZhi2, Unigene4174\_JingChaoZuZhi2, Unigene43059\_JingChaoZuZhi2, Unigene4339\_JingChaoZuZhi2, Unigene43889\_JingChaoZuZhi2, Unigene44454\_JingChaoZuZhi2, Unigene4474\_JingChaoZuZhi2, Unigene451\_JingChaoZuZhi2, Unigene4529\_JingChaoZuZhi2, Unigene45468\_JingChaoZuZhi2, Unigene4599\_JingChaoZuZhi2, Unigene4618\_JingChaoZuZhi2, Unigene46407\_JingChaoZuZhi2, Unigene46581\_JingChaoZuZhi2, Unigene46628\_JingChaoZuZhi2, Unigene48408\_JingChaoZuZhi2, Unigene4841\_JingChaoZuZhi2, Unigene48673\_JingChaoZuZhi2, Unigene48751\_JingChaoZuZhi2, Unigene48843\_JingChaoZuZhi2, Unigene49253\_JingChaoZuZhi2, Unigene4975\_JingChaoZuZhi2, Unigene499\_JingChaoZuZhi2, Unigene5035\_JingChaoZuZhi2, Unigene5073\_JingChaoZuZhi2, Unigene5124\_JingChaoZuZhi2, Unigene5146\_JingChaoZuZhi2, Unigene51512\_JingChaoZuZhi2, Unigene52004\_JingChaoZuZhi2, Unigene52122\_JingChaoZuZhi2, Unigene5349\_JingChaoZuZhi2, Unigene53503\_JingChaoZuZhi2, Unigene53917\_JingChaoZuZhi2, Unigene54367\_JingChaoZuZhi2, Unigene54679\_JingChaoZuZhi2, Unigene54744\_JingChaoZuZhi2, Unigene54777\_JingChaoZuZhi2, Unigene54986\_JingChaoZuZhi2, Unigene5529\_JingChaoZuZhi2, Unigene5534\_JingChaoZuZhi2, Unigene5554\_JingChaoZuZhi2, Unigene55913\_JingChaoZuZhi2, Unigene563\_JingChaoZuZhi2, Unigene57338\_JingChaoZuZhi2, Unigene5733\_JingChaoZuZhi2, Unigene5739\_JingChaoZuZhi2, Unigene57488\_JingChaoZuZhi2, Unigene57749\_JingChaoZuZhi2, Unigene5814\_JingChaoZuZhi2, Unigene5820\_JingChaoZuZhi2, Unigene58405\_JingChaoZuZhi2, Unigene5863\_JingChaoZuZhi2, Unigene58670\_JingChaoZuZhi2, Unigene58967\_JingChaoZuZhi2, Unigene589\_JingChaoZuZhi2, Unigene5903\_JingChaoZuZhi2, Unigene59101\_JingChaoZuZhi2, Unigene59510\_JingChaoZuZhi2, Unigene5959\_JingChaoZuZhi2, Unigene59604\_JingChaoZuZhi2, Unigene59797\_JingChaoZuZhi2, Unigene60026\_JingChaoZuZhi2, Unigene60650\_JingChaoZuZhi2, Unigene6074\_JingChaoZuZhi2, Unigene60988\_JingChaoZuZhi2, Unigene61043\_JingChaoZuZhi2, Unigene610\_JingChaoZuZhi2, Unigene6121\_JingChaoZuZhi2, Unigene61390\_JingChaoZuZhi2, Unigene61406\_JingChaoZuZhi2, Unigene61657\_JingChaoZuZhi2, Unigene61675\_JingChaoZuZhi2, Unigene61702\_JingChaoZuZhi2, Unigene62251\_JingChaoZuZhi2, Unigene6262\_JingChaoZuZhi2, Unigene62835\_JingChaoZuZhi2, Unigene62919\_JingChaoZuZhi2, Unigene63547\_JingChaoZuZhi2, Unigene63589\_JingChaoZuZhi2, Unigene6392\_JingChaoZuZhi2, Unigene63\_JingChaoZuZhi2, Unigene64164\_JingChaoZuZhi2, Unigene64169\_JingChaoZuZhi2, Unigene6433\_JingChaoZuZhi2, Unigene64529\_JingChaoZuZhi2, Unigene64626\_JingChaoZuZhi2, Unigene6468\_JingChaoZuZhi2, Unigene64750\_JingChaoZuZhi2, Unigene64830\_JingChaoZuZhi2, Unigene6495\_JingChaoZuZhi2, Unigene65431\_JingChaoZuZhi2, Unigene65445\_JingChaoZuZhi2, Unigene6563\_JingChaoZuZhi2, Unigene65663\_JingChaoZuZhi2, Unigene65862\_JingChaoZuZhi2, Unigene66105\_JingChaoZuZhi2, Unigene66160\_JingChaoZuZhi2, Unigene6625\_JingChaoZuZhi2, Unigene66540\_JingChaoZuZhi2, Unigene66700\_JingChaoZuZhi2, Unigene66757\_JingChaoZuZhi2, Unigene6699\_JingChaoZuZhi2, Unigene67052\_JingChaoZuZhi2, Unigene6735\_JingChaoZuZhi2, Unigene67365\_JingChaoZuZhi2, Unigene68262\_JingChaoZuZhi2, Unigene68495\_JingChaoZuZhi2, Unigene68867\_JingChaoZuZhi2, Unigene68906\_JingChaoZuZhi2, Unigene69062\_JingChaoZuZhi2, Unigene69071\_JingChaoZuZhi2, Unigene69445\_JingChaoZuZhi2, Unigene6979\_JingChaoZuZhi2, Unigene70155\_JingChaoZuZhi2, Unigene70156\_JingChaoZuZhi2, Unigene70319\_JingChaoZuZhi2, Unigene70353\_JingChaoZuZhi2, Unigene70493\_JingChaoZuZhi2, Unigene70539\_JingChaoZuZhi2, Unigene705\_JingChaoZuZhi2, Unigene7062\_JingChaoZuZhi2, Unigene7068\_JingChaoZuZhi2, Unigene70696\_JingChaoZuZhi2, Unigene70723\_JingChaoZuZhi2, Unigene7074\_JingChaoZuZhi2, Unigene7083\_JingChaoZuZhi2, Unigene71079\_JingChaoZuZhi2, Unigene71144\_JingChaoZuZhi2, Unigene71172\_JingChaoZuZhi2, Unigene71361\_JingChaoZuZhi2, Unigene7139\_JingChaoZuZhi2, Unigene7162\_JingChaoZuZhi2, Unigene71738\_JingChaoZuZhi2, Unigene7186\_JingChaoZuZhi2, Unigene71928\_JingChaoZuZhi2, Unigene72015\_JingChaoZuZhi2, Unigene72219\_JingChaoZuZhi2, Unigene72397\_JingChaoZuZhi2, Unigene72550\_JingChaoZuZhi2, Unigene72643\_JingChaoZuZhi2, Unigene7306\_JingChaoZuZhi2, Unigene73445\_JingChaoZuZhi2, Unigene73470\_JingChaoZuZhi2, Unigene73554\_JingChaoZuZhi2, Unigene7407\_JingChaoZuZhi2, Unigene74199\_JingChaoZuZhi2, Unigene74502\_JingChaoZuZhi2, Unigene74795\_JingChaoZuZhi2, Unigene7480\_JingChaoZuZhi2, Unigene75030\_JingChaoZuZhi2, Unigene75045\_JingChaoZuZhi2, Unigene75123\_JingChaoZuZhi2, Unigene751\_JingChaoZuZhi2, Unigene75235\_JingChaoZuZhi2, Unigene75244\_JingChaoZuZhi2, Unigene75422\_JingChaoZuZhi2, Unigene754\_JingChaoZuZhi2, Unigene75793\_JingChaoZuZhi2, Unigene75821\_JingChaoZuZhi2, Unigene75857\_JingChaoZuZhi2, Unigene76592\_JingChaoZuZhi2, Unigene7670\_JingChaoZuZhi2, Unigene76805\_JingChaoZuZhi2, Unigene76863\_JingChaoZuZhi2, Unigene76917\_JingChaoZuZhi2, Unigene77061\_JingChaoZuZhi2, Unigene77072\_JingChaoZuZhi2, Unigene77149\_JingChaoZuZhi2, Unigene77539\_JingChaoZuZhi2, Unigene77543\_JingChaoZuZhi2, Unigene77549\_JingChaoZuZhi2, Unigene77596\_JingChaoZuZhi2, Unigene77644\_JingChaoZuZhi2, Unigene77816\_JingChaoZuZhi2, Unigene77871\_JingChaoZuZhi2, Unigene7793\_JingChaoZuZhi2, Unigene78078\_JingChaoZuZhi2, Unigene78235\_JingChaoZuZhi2, Unigene78429\_JingChaoZuZhi2, Unigene78715\_JingChaoZuZhi2, Unigene787\_JingChaoZuZhi2, Unigene78909\_JingChaoZuZhi2, Unigene78987\_JingChaoZuZhi2, Unigene79077\_JingChaoZuZhi2, Unigene79109\_JingChaoZuZhi2, Unigene79203\_JingChaoZuZhi2, Unigene79529\_JingChaoZuZhi2, Unigene79560\_JingChaoZuZhi2, Unigene79778\_JingChaoZuZhi2, Unigene79810\_JingChaoZuZhi2, Unigene79828\_JingChaoZuZhi2, Unigene79902\_JingChaoZuZhi2, Unigene79987\_JingChaoZuZhi2, Unigene80069\_JingChaoZuZhi2, Unigene80120\_JingChaoZuZhi2, Unigene80193\_JingChaoZuZhi2, Unigene80225\_JingChaoZuZhi2, Unigene80247\_JingChaoZuZhi2, Unigene80312\_JingChaoZuZhi2, Unigene80412\_JingChaoZuZhi2, Unigene80475\_JingChaoZuZhi2, Unigene80608\_JingChaoZuZhi2, Unigene80653\_JingChaoZuZhi2, Unigene80818\_JingChaoZuZhi2, Unigene8094\_JingChaoZuZhi2, Unigene81148\_JingChaoZuZhi2, Unigene81225\_JingChaoZuZhi2, Unigene8133\_JingChaoZuZhi2, Unigene81393\_JingChaoZuZhi2, Unigene81599\_JingChaoZuZhi2, Unigene81601\_JingChaoZuZhi2, Unigene81700\_JingChaoZuZhi2, Unigene81793\_JingChaoZuZhi2, Unigene81964\_JingChaoZuZhi2, Unigene81\_JingChaoZuZhi2, Unigene82040\_JingChaoZuZhi2, Unigene82120\_JingChaoZuZhi2, Unigene82442\_JingChaoZuZhi2, Unigene82734\_JingChaoZuZhi2, Unigene82761\_JingChaoZuZhi2, Unigene8288\_JingChaoZuZhi2, Unigene828\_JingChaoZuZhi2, Unigene82918\_JingChaoZuZhi2, Unigene82\_JingChaoZuZhi2, Unigene83094\_JingChaoZuZhi2, Unigene83252\_JingChaoZuZhi2, Unigene83276\_JingChaoZuZhi2, Unigene83303\_JingChaoZuZhi2, Unigene83345\_JingChaoZuZhi2, Unigene8338\_JingChaoZuZhi2, Unigene83507\_JingChaoZuZhi2, Unigene83935\_JingChaoZuZhi2, Unigene83973\_JingChaoZuZhi2, Unigene84190\_JingChaoZuZhi2, Unigene84252\_JingChaoZuZhi2, Unigene84257\_JingChaoZuZhi2, Unigene84270\_JingChaoZuZhi2, Unigene84352\_JingChaoZuZhi2, Unigene843\_JingChaoZuZhi2, Unigene84658\_JingChaoZuZhi2, Unigene84671\_JingChaoZuZhi2, Unigene84829\_JingChaoZuZhi2, Unigene84880\_JingChaoZuZhi2, Unigene84883\_JingChaoZuZhi2, Unigene84966\_JingChaoZuZhi2, Unigene85055\_JingChaoZuZhi2, Unigene85066\_JingChaoZuZhi2, Unigene85069\_JingChaoZuZhi2, Unigene85099\_JingChaoZuZhi2, Unigene8510\_JingChaoZuZhi2, Unigene85198\_JingChaoZuZhi2, Unigene8520\_JingChaoZuZhi2, Unigene85215\_JingChaoZuZhi2, Unigene85335\_JingChaoZuZhi2, Unigene85375\_JingChaoZuZhi2, Unigene85405\_JingChaoZuZhi2, Unigene85490\_JingChaoZuZhi2, Unigene85503\_JingChaoZuZhi2, Unigene85504\_JingChaoZuZhi2, Unigene85520\_JingChaoZuZhi2, Unigene85781\_JingChaoZuZhi2, Unigene85923\_JingChaoZuZhi2, Unigene8607\_JingChaoZuZhi2, Unigene86107\_JingChaoZuZhi2, Unigene86131\_JingChaoZuZhi2, Unigene86206\_JingChaoZuZhi2, Unigene86243\_JingChaoZuZhi2, Unigene86271\_JingChaoZuZhi2, Unigene86296\_JingChaoZuZhi2, Unigene86362\_JingChaoZuZhi2, Unigene86504\_JingChaoZuZhi2, Unigene86526\_JingChaoZuZhi2, Unigene86630\_JingChaoZuZhi2, Unigene86669\_JingChaoZuZhi2, Unigene86941\_JingChaoZuZhi2, Unigene8710\_JingChaoZuZhi2, Unigene87160\_JingChaoZuZhi2, Unigene87169\_JingChaoZuZhi2, Unigene87191\_JingChaoZuZhi2, Unigene87203\_JingChaoZuZhi2, Unigene87228\_JingChaoZuZhi2, Unigene87246\_JingChaoZuZhi2, Unigene87332\_JingChaoZuZhi2, Unigene87400\_JingChaoZuZhi2, Unigene87448\_JingChaoZuZhi2, Unigene87474\_JingChaoZuZhi2, Unigene87551\_JingChaoZuZhi2, Unigene87587\_JingChaoZuZhi2, Unigene87607\_JingChaoZuZhi2, Unigene87653\_JingChaoZuZhi2, Unigene87741\_JingChaoZuZhi2, Unigene87818\_JingChaoZuZhi2, Unigene87903\_JingChaoZuZhi2, Unigene87917\_JingChaoZuZhi2, Unigene87946\_JingChaoZuZhi2, Unigene87952\_JingChaoZuZhi2, Unigene87963\_JingChaoZuZhi2, Unigene88059\_JingChaoZuZhi2, Unigene88087\_JingChaoZuZhi2, Unigene88089\_JingChaoZuZhi2, Unigene88115\_JingChaoZuZhi2, Unigene88185\_JingChaoZuZhi2, Unigene88203\_JingChaoZuZhi2, Unigene88235\_JingChaoZuZhi2, Unigene88283\_JingChaoZuZhi2, Unigene88385\_JingChaoZuZhi2, Unigene88391\_JingChaoZuZhi2, Unigene8839\_JingChaoZuZhi2, Unigene88546\_JingChaoZuZhi2, Unigene88618\_JingChaoZuZhi2, Unigene88661\_JingChaoZuZhi2, Unigene88672\_JingChaoZuZhi2, Unigene88740\_JingChaoZuZhi2, Unigene88761\_JingChaoZuZhi2, Unigene88773\_JingChaoZuZhi2, Unigene88806\_JingChaoZuZhi2, Unigene88886\_JingChaoZuZhi2, Unigene88990\_JingChaoZuZhi2, Unigene89045\_JingChaoZuZhi2, Unigene89057\_JingChaoZuZhi2, Unigene89087\_JingChaoZuZhi2, Unigene89094\_JingChaoZuZhi2, Unigene89127\_JingChaoZuZhi2, Unigene89192\_JingChaoZuZhi2, Unigene89251\_JingChaoZuZhi2, Unigene89305\_JingChaoZuZhi2, Unigene89321\_JingChaoZuZhi2, Unigene89413\_JingChaoZuZhi2, Unigene89572\_JingChaoZuZhi2, Unigene89597\_JingChaoZuZhi2, Unigene89628\_JingChaoZuZhi2, Unigene896\_JingChaoZuZhi2, Unigene89710\_JingChaoZuZhi2, Unigene89946\_JingChaoZuZhi2, Unigene8996\_JingChaoZuZhi2, Unigene89973\_JingChaoZuZhi2, Unigene90003\_JingChaoZuZhi2, Unigene90078\_JingChaoZuZhi2, Unigene90082\_JingChaoZuZhi2, Unigene90084\_JingChaoZuZhi2, Unigene90173\_JingChaoZuZhi2, Unigene90201\_JingChaoZuZhi2, Unigene90261\_JingChaoZuZhi2, Unigene90284\_JingChaoZuZhi2, Unigene9028\_JingChaoZuZhi2, Unigene90442\_JingChaoZuZhi2, Unigene90494\_JingChaoZuZhi2, Unigene9052\_JingChaoZuZhi2, Unigene9053\_JingChaoZuZhi2, Unigene90600\_JingChaoZuZhi2, Unigene90609\_JingChaoZuZhi2, Unigene90724\_JingChaoZuZhi2, Unigene90742\_JingChaoZuZhi2, Unigene90828\_JingChaoZuZhi2, Unigene90830\_JingChaoZuZhi2, Unigene90889\_JingChaoZuZhi2, Unigene90892\_JingChaoZuZhi2, Unigene90933\_JingChaoZuZhi2, Unigene90953\_JingChaoZuZhi2, Unigene90955\_JingChaoZuZhi2, Unigene91138\_JingChaoZuZhi2, Unigene91252\_JingChaoZuZhi2, Unigene91307\_JingChaoZuZhi2, Unigene9132\_JingChaoZuZhi2, Unigene91350\_JingChaoZuZhi2, Unigene91359\_JingChaoZuZhi2, Unigene91446\_JingChaoZuZhi2, Unigene91527\_JingChaoZuZhi2, Unigene91529\_JingChaoZuZhi2, Unigene91534\_JingChaoZuZhi2, Unigene91584\_JingChaoZuZhi2, Unigene91592\_JingChaoZuZhi2, Unigene91613\_JingChaoZuZhi2, Unigene91685\_JingChaoZuZhi2, Unigene91686\_JingChaoZuZhi2, Unigene91710\_JingChaoZuZhi2, Unigene91733\_JingChaoZuZhi2, Unigene91739\_JingChaoZuZhi2, Unigene91848\_JingChaoZuZhi2, Unigene91952\_JingChaoZuZhi2, Unigene91983\_JingChaoZuZhi2, Unigene92015\_JingChaoZuZhi2, Unigene92046\_JingChaoZuZhi2, Unigene92084\_JingChaoZuZhi2, Unigene92101\_JingChaoZuZhi2, Unigene92112\_JingChaoZuZhi2, Unigene92139\_JingChaoZuZhi2, Unigene92170\_JingChaoZuZhi2, Unigene9220\_JingChaoZuZhi2, Unigene92227\_JingChaoZuZhi2, Unigene92245\_JingChaoZuZhi2, Unigene92271\_JingChaoZuZhi2, Unigene92379\_JingChaoZuZhi2, Unigene92417\_JingChaoZuZhi2, Unigene92529\_JingChaoZuZhi2, Unigene92536\_JingChaoZuZhi2, Unigene92548\_JingChaoZuZhi2, Unigene92566\_JingChaoZuZhi2, Unigene92596\_JingChaoZuZhi2, Unigene92607\_JingChaoZuZhi2, Unigene92627\_JingChaoZuZhi2, Unigene92749\_JingChaoZuZhi2, Unigene92786\_JingChaoZuZhi2, Unigene92831\_JingChaoZuZhi2, Unigene92901\_JingChaoZuZhi2, Unigene92905\_JingChaoZuZhi2, Unigene92912\_JingChaoZuZhi2, Unigene92920\_JingChaoZuZhi2, Unigene93012\_JingChaoZuZhi2, Unigene93016\_JingChaoZuZhi2, Unigene93025\_JingChaoZuZhi2, Unigene93032\_JingChaoZuZhi2, Unigene93047\_JingChaoZuZhi2, Unigene93070\_JingChaoZuZhi2, Unigene93081\_JingChaoZuZhi2, Unigene93093\_JingChaoZuZhi2, Unigene93111\_JingChaoZuZhi2, Unigene93115\_JingChaoZuZhi2, Unigene93195\_JingChaoZuZhi2, Unigene93207\_JingChaoZuZhi2, Unigene93229\_JingChaoZuZhi2, Unigene93238\_JingChaoZuZhi2, Unigene93333\_JingChaoZuZhi2, Unigene93343\_JingChaoZuZhi2, Unigene93362\_JingChaoZuZhi2, Unigene93412\_JingChaoZuZhi2, Unigene93444\_JingChaoZuZhi2, Unigene93470\_JingChaoZuZhi2, Unigene93492\_JingChaoZuZhi2, Unigene93516\_JingChaoZuZhi2, Unigene93520\_JingChaoZuZhi2, Unigene93583\_JingChaoZuZhi2, Unigene93594\_JingChaoZuZhi2, Unigene93646\_JingChaoZuZhi2, Unigene93702\_JingChaoZuZhi2, Unigene93717\_JingChaoZuZhi2, Unigene93734\_JingChaoZuZhi2, Unigene93746\_JingChaoZuZhi2, Unigene93760\_JingChaoZuZhi2, Unigene93765\_JingChaoZuZhi2, Unigene93798\_JingChaoZuZhi2, Unigene93807\_JingChaoZuZhi2, Unigene9380\_JingChaoZuZhi2, Unigene93837\_JingChaoZuZhi2, Unigene93857\_JingChaoZuZhi2, Unigene93874\_JingChaoZuZhi2, Unigene93902\_JingChaoZuZhi2, Unigene93930\_JingChaoZuZhi2, Unigene93931\_JingChaoZuZhi2, Unigene93936\_JingChaoZuZhi2, Unigene93955\_JingChaoZuZhi2, Unigene93998\_JingChaoZuZhi2, Unigene94005\_JingChaoZuZhi2, Unigene94011\_JingChaoZuZhi2, Unigene9402\_JingChaoZuZhi2, Unigene94057\_JingChaoZuZhi2, Unigene94058\_JingChaoZuZhi2, Unigene94069\_JingChaoZuZhi2, Unigene94103\_JingChaoZuZhi2, Unigene94107\_JingChaoZuZhi2, Unigene94149\_JingChaoZuZhi2, Unigene94204\_JingChaoZuZhi2, Unigene94214\_JingChaoZuZhi2, Unigene94266\_JingChaoZuZhi2, Unigene94305\_JingChaoZuZhi2, Unigene94311\_JingChaoZuZhi2, Unigene94313\_JingChaoZuZhi2, Unigene94320\_JingChaoZuZhi2, Unigene94364\_JingChaoZuZhi2, Unigene94370\_JingChaoZuZhi2, Unigene94376\_JingChaoZuZhi2, Unigene94384\_JingChaoZuZhi2, Unigene94386\_JingChaoZuZhi2, Unigene94445\_JingChaoZuZhi2, Unigene9445\_JingChaoZuZhi2, Unigene94476\_JingChaoZuZhi2, Unigene94484\_JingChaoZuZhi2, Unigene94488\_JingChaoZuZhi2, Unigene94496\_JingChaoZuZhi2, Unigene94509\_JingChaoZuZhi2, Unigene94518\_JingChaoZuZhi2, Unigene94526\_JingChaoZuZhi2, Unigene94540\_JingChaoZuZhi2, Unigene94578\_JingChaoZuZhi2, Unigene94586\_JingChaoZuZhi2, Unigene94616\_JingChaoZuZhi2, Unigene94664\_JingChaoZuZhi2, Unigene94674\_JingChaoZuZhi2, Unigene94685\_JingChaoZuZhi2, Unigene94690\_JingChaoZuZhi2, Unigene94721\_JingChaoZuZhi2, Unigene94725\_JingChaoZuZhi2, Unigene94792\_JingChaoZuZhi2, Unigene94810\_JingChaoZuZhi2, Unigene94841\_JingChaoZuZhi2, Unigene94842\_JingChaoZuZhi2, Unigene94851\_JingChaoZuZhi2, Unigene94872\_JingChaoZuZhi2, Unigene9490\_JingChaoZuZhi2, Unigene94974\_JingChaoZuZhi2, Unigene94975\_JingChaoZuZhi2, Unigene94989\_JingChaoZuZhi2, Unigene94999\_JingChaoZuZhi2, Unigene95020\_JingChaoZuZhi2, Unigene95035\_JingChaoZuZhi2, Unigene95089\_JingChaoZuZhi2, Unigene95130\_JingChaoZuZhi2, Unigene95140\_JingChaoZuZhi2, Unigene95178\_JingChaoZuZhi2, Unigene95181\_JingChaoZuZhi2, Unigene95283\_JingChaoZuZhi2, Unigene95290\_JingChaoZuZhi2, Unigene95336\_JingChaoZuZhi2, Unigene95390\_JingChaoZuZhi2, Unigene95391\_JingChaoZuZhi2, Unigene95415\_JingChaoZuZhi2, Unigene95427\_JingChaoZuZhi2, Unigene95483\_JingChaoZuZhi2, Unigene95485\_JingChaoZuZhi2, Unigene95489\_JingChaoZuZhi2, Unigene95510\_JingChaoZuZhi2, Unigene95526\_JingChaoZuZhi2, Unigene95538\_JingChaoZuZhi2, Unigene95633\_JingChaoZuZhi2, Unigene95647\_JingChaoZuZhi2, Unigene95654\_JingChaoZuZhi2, Unigene95661\_JingChaoZuZhi2, Unigene95677\_JingChaoZuZhi2, Unigene95690\_JingChaoZuZhi2, Unigene95699\_JingChaoZuZhi2, Unigene95701\_JingChaoZuZhi2, Unigene95710\_JingChaoZuZhi2, Unigene95758\_JingChaoZuZhi2, Unigene95761\_JingChaoZuZhi2, Unigene95762\_JingChaoZuZhi2, Unigene95770\_JingChaoZuZhi2, Unigene95782\_JingChaoZuZhi2, Unigene95799\_JingChaoZuZhi2, Unigene95807\_JingChaoZuZhi2, Unigene95809\_JingChaoZuZhi2, Unigene95821\_JingChaoZuZhi2, Unigene95828\_JingChaoZuZhi2, Unigene95849\_JingChaoZuZhi2, Unigene95851\_JingChaoZuZhi2, Unigene95853\_JingChaoZuZhi2, Unigene95860\_JingChaoZuZhi2, Unigene95864\_JingChaoZuZhi2, Unigene95880\_JingChaoZuZhi2, Unigene95897\_JingChaoZuZhi2, Unigene95910\_JingChaoZuZhi2, Unigene95916\_JingChaoZuZhi2, Unigene95948\_JingChaoZuZhi2, Unigene95973\_JingChaoZuZhi2, Unigene95977\_JingChaoZuZhi2, Unigene96025\_JingChaoZuZhi2, Unigene96030\_JingChaoZuZhi2, Unigene96042\_JingChaoZuZhi2, Unigene96057\_JingChaoZuZhi2, Unigene96063\_JingChaoZuZhi2, Unigene96069\_JingChaoZuZhi2, Unigene96102\_JingChaoZuZhi2, Unigene96104\_JingChaoZuZhi2, Unigene96115\_JingChaoZuZhi2, Unigene9616\_JingChaoZuZhi2, Unigene96171\_JingChaoZuZhi2, Unigene96182\_JingChaoZuZhi2, Unigene96204\_JingChaoZuZhi2, Unigene96219\_JingChaoZuZhi2, Unigene96228\_JingChaoZuZhi2, Unigene96237\_JingChaoZuZhi2, Unigene96242\_JingChaoZuZhi2, Unigene96245\_JingChaoZuZhi2, Unigene96246\_JingChaoZuZhi2, Unigene96262\_JingChaoZuZhi2, Unigene96294\_JingChaoZuZhi2, Unigene9636\_JingChaoZuZhi2, Unigene9654\_JingChaoZuZhi2, Unigene9674\_JingChaoZuZhi2, Unigene9675\_JingChaoZuZhi2, Unigene9811\_JingChaoZuZhi2, Unigene9867\_JingChaoZuZhi2, Unigene9871\_JingChaoZuZhi2, Unigene9892\_JingChaoZuZhi2, Unigene98\_JingChaoZuZhi2, Unigene9946\_JingChaoZuZhi2, Unigene9978\_JingChaoZuZhi2 |
| 5 | Adherens junction | Unigene10016\_JingChaoZuZhi2, Unigene1002\_JingChaoZuZhi2, Unigene10031\_JingChaoZuZhi2, Unigene10047\_JingChaoZuZhi2, Unigene10051\_JingChaoZuZhi2, Unigene10078\_JingChaoZuZhi2, Unigene10128\_JingChaoZuZhi2, Unigene10147\_JingChaoZuZhi2, Unigene10150\_JingChaoZuZhi2, Unigene1017\_JingChaoZuZhi2, Unigene10208\_JingChaoZuZhi2, Unigene10259\_JingChaoZuZhi2, Unigene10351\_JingChaoZuZhi2, Unigene10422\_JingChaoZuZhi2, Unigene10456\_JingChaoZuZhi2, Unigene10492\_JingChaoZuZhi2, Unigene10498\_JingChaoZuZhi2, Unigene10576\_JingChaoZuZhi2, Unigene10582\_JingChaoZuZhi2, Unigene1060\_JingChaoZuZhi2, Unigene10733\_JingChaoZuZhi2, Unigene10772\_JingChaoZuZhi2, Unigene10922\_JingChaoZuZhi2, Unigene10932\_JingChaoZuZhi2, Unigene10936\_JingChaoZuZhi2, Unigene10974\_JingChaoZuZhi2, Unigene10979\_JingChaoZuZhi2, Unigene10\_JingChaoZuZhi2, Unigene11000\_JingChaoZuZhi2, Unigene1121\_JingChaoZuZhi2, Unigene11232\_JingChaoZuZhi2, Unigene11240\_JingChaoZuZhi2, Unigene11258\_JingChaoZuZhi2, Unigene11370\_JingChaoZuZhi2, Unigene1153\_JingChaoZuZhi2, Unigene11569\_JingChaoZuZhi2, Unigene11581\_JingChaoZuZhi2, Unigene115\_JingChaoZuZhi2, Unigene1162\_JingChaoZuZhi2, Unigene11729\_JingChaoZuZhi2, Unigene11732\_JingChaoZuZhi2, Unigene11810\_JingChaoZuZhi2, Unigene11902\_JingChaoZuZhi2, Unigene11918\_JingChaoZuZhi2, Unigene11932\_JingChaoZuZhi2, Unigene11995\_JingChaoZuZhi2, Unigene12011\_JingChaoZuZhi2, Unigene12148\_JingChaoZuZhi2, Unigene12207\_JingChaoZuZhi2, Unigene12285\_JingChaoZuZhi2, Unigene12303\_JingChaoZuZhi2, Unigene12310\_JingChaoZuZhi2, Unigene12320\_JingChaoZuZhi2, Unigene12326\_JingChaoZuZhi2, Unigene12343\_JingChaoZuZhi2, Unigene12372\_JingChaoZuZhi2, Unigene12428\_JingChaoZuZhi2, Unigene12549\_JingChaoZuZhi2, Unigene12577\_JingChaoZuZhi2, Unigene12634\_JingChaoZuZhi2, Unigene12644\_JingChaoZuZhi2, Unigene12791\_JingChaoZuZhi2, Unigene12815\_JingChaoZuZhi2, Unigene12874\_JingChaoZuZhi2, Unigene12984\_JingChaoZuZhi2, Unigene1301\_JingChaoZuZhi2, Unigene13085\_JingChaoZuZhi2, Unigene13097\_JingChaoZuZhi2, Unigene13196\_JingChaoZuZhi2, Unigene13249\_JingChaoZuZhi2, Unigene1325\_JingChaoZuZhi2, Unigene13478\_JingChaoZuZhi2, Unigene13572\_JingChaoZuZhi2, Unigene13573\_JingChaoZuZhi2, Unigene13607\_JingChaoZuZhi2, Unigene13618\_JingChaoZuZhi2, Unigene13650\_JingChaoZuZhi2, Unigene13713\_JingChaoZuZhi2, Unigene13720\_JingChaoZuZhi2, Unigene13766\_JingChaoZuZhi2, Unigene1376\_JingChaoZuZhi2, Unigene13890\_JingChaoZuZhi2, Unigene13901\_JingChaoZuZhi2, Unigene13904\_JingChaoZuZhi2, Unigene13927\_JingChaoZuZhi2, Unigene13963\_JingChaoZuZhi2, Unigene14071\_JingChaoZuZhi2, Unigene14159\_JingChaoZuZhi2, Unigene14193\_JingChaoZuZhi2, Unigene14246\_JingChaoZuZhi2, Unigene14285\_JingChaoZuZhi2, Unigene14289\_JingChaoZuZhi2, Unigene14450\_JingChaoZuZhi2, Unigene14476\_JingChaoZuZhi2, Unigene14534\_JingChaoZuZhi2, Unigene14610\_JingChaoZuZhi2, Unigene14635\_JingChaoZuZhi2, Unigene14714\_JingChaoZuZhi2, Unigene14715\_JingChaoZuZhi2, Unigene14749\_JingChaoZuZhi2, Unigene14845\_JingChaoZuZhi2, Unigene14878\_JingChaoZuZhi2, Unigene14926\_JingChaoZuZhi2, Unigene14970\_JingChaoZuZhi2, Unigene15029\_JingChaoZuZhi2, Unigene15088\_JingChaoZuZhi2, Unigene15096\_JingChaoZuZhi2, Unigene150\_JingChaoZuZhi2, Unigene15168\_JingChaoZuZhi2, Unigene15176\_JingChaoZuZhi2, Unigene15251\_JingChaoZuZhi2, Unigene15286\_JingChaoZuZhi2, Unigene15288\_JingChaoZuZhi2, Unigene15319\_JingChaoZuZhi2, Unigene15374\_JingChaoZuZhi2, Unigene15381\_JingChaoZuZhi2, Unigene15429\_JingChaoZuZhi2, Unigene15492\_JingChaoZuZhi2, Unigene15557\_JingChaoZuZhi2, Unigene15615\_JingChaoZuZhi2, Unigene15650\_JingChaoZuZhi2, Unigene15651\_JingChaoZuZhi2, Unigene15659\_JingChaoZuZhi2, Unigene15731\_JingChaoZuZhi2, Unigene15736\_JingChaoZuZhi2, Unigene15744\_JingChaoZuZhi2, Unigene15857\_JingChaoZuZhi2, Unigene15879\_JingChaoZuZhi2, Unigene16004\_JingChaoZuZhi2, Unigene16028\_JingChaoZuZhi2, Unigene16103\_JingChaoZuZhi2, Unigene16137\_JingChaoZuZhi2, Unigene16152\_JingChaoZuZhi2, Unigene16176\_JingChaoZuZhi2, Unigene16182\_JingChaoZuZhi2, Unigene16229\_JingChaoZuZhi2, Unigene16402\_JingChaoZuZhi2, Unigene16468\_JingChaoZuZhi2, Unigene16481\_JingChaoZuZhi2, Unigene16510\_JingChaoZuZhi2, Unigene16591\_JingChaoZuZhi2, Unigene16606\_JingChaoZuZhi2, Unigene16637\_JingChaoZuZhi2, Unigene16655\_JingChaoZuZhi2, Unigene16730\_JingChaoZuZhi2, Unigene16950\_JingChaoZuZhi2, Unigene16958\_JingChaoZuZhi2, Unigene17101\_JingChaoZuZhi2, Unigene17122\_JingChaoZuZhi2, Unigene17225\_JingChaoZuZhi2, Unigene17501\_JingChaoZuZhi2, Unigene17547\_JingChaoZuZhi2, Unigene1760\_JingChaoZuZhi2, Unigene17764\_JingChaoZuZhi2, Unigene17789\_JingChaoZuZhi2, Unigene17845\_JingChaoZuZhi2, Unigene17894\_JingChaoZuZhi2, Unigene17896\_JingChaoZuZhi2, Unigene18010\_JingChaoZuZhi2, Unigene18043\_JingChaoZuZhi2, Unigene18256\_JingChaoZuZhi2, Unigene18259\_JingChaoZuZhi2, Unigene18269\_JingChaoZuZhi2, Unigene18334\_JingChaoZuZhi2, Unigene18348\_JingChaoZuZhi2, Unigene18441\_JingChaoZuZhi2, Unigene18505\_JingChaoZuZhi2, Unigene18608\_JingChaoZuZhi2, Unigene18614\_JingChaoZuZhi2, Unigene18662\_JingChaoZuZhi2, Unigene18685\_JingChaoZuZhi2, Unigene18697\_JingChaoZuZhi2, Unigene18790\_JingChaoZuZhi2, Unigene18835\_JingChaoZuZhi2, Unigene1887\_JingChaoZuZhi2, Unigene18960\_JingChaoZuZhi2, Unigene19001\_JingChaoZuZhi2, Unigene19021\_JingChaoZuZhi2, Unigene19086\_JingChaoZuZhi2, Unigene19137\_JingChaoZuZhi2, Unigene19204\_JingChaoZuZhi2, Unigene19222\_JingChaoZuZhi2, Unigene19223\_JingChaoZuZhi2, Unigene19238\_JingChaoZuZhi2, Unigene19319\_JingChaoZuZhi2, Unigene19365\_JingChaoZuZhi2, Unigene19391\_JingChaoZuZhi2, Unigene19396\_JingChaoZuZhi2, Unigene19512\_JingChaoZuZhi2, Unigene19533\_JingChaoZuZhi2, Unigene19701\_JingChaoZuZhi2, Unigene19741\_JingChaoZuZhi2, Unigene19813\_JingChaoZuZhi2, Unigene19883\_JingChaoZuZhi2, Unigene19979\_JingChaoZuZhi2, Unigene20025\_JingChaoZuZhi2, Unigene20030\_JingChaoZuZhi2, Unigene20049\_JingChaoZuZhi2, Unigene20052\_JingChaoZuZhi2, Unigene20083\_JingChaoZuZhi2, Unigene20107\_JingChaoZuZhi2, Unigene201\_JingChaoZuZhi2, Unigene20239\_JingChaoZuZhi2, Unigene2029\_JingChaoZuZhi2, Unigene20373\_JingChaoZuZhi2, Unigene20394\_JingChaoZuZhi2, Unigene20410\_JingChaoZuZhi2, Unigene20468\_JingChaoZuZhi2, Unigene20474\_JingChaoZuZhi2, Unigene204\_JingChaoZuZhi2, Unigene20557\_JingChaoZuZhi2, Unigene20698\_JingChaoZuZhi2, Unigene20720\_JingChaoZuZhi2, Unigene20727\_JingChaoZuZhi2, Unigene20837\_JingChaoZuZhi2, Unigene20943\_JingChaoZuZhi2, Unigene21057\_JingChaoZuZhi2, Unigene21100\_JingChaoZuZhi2, Unigene2115\_JingChaoZuZhi2, Unigene21306\_JingChaoZuZhi2, Unigene21311\_JingChaoZuZhi2, Unigene21411\_JingChaoZuZhi2, Unigene21543\_JingChaoZuZhi2, Unigene21594\_JingChaoZuZhi2, Unigene21605\_JingChaoZuZhi2, Unigene21636\_JingChaoZuZhi2, Unigene21646\_JingChaoZuZhi2, Unigene21801\_JingChaoZuZhi2, Unigene21818\_JingChaoZuZhi2, Unigene21860\_JingChaoZuZhi2, Unigene21898\_JingChaoZuZhi2, Unigene21932\_JingChaoZuZhi2, Unigene21933\_JingChaoZuZhi2, Unigene21963\_JingChaoZuZhi2, Unigene219\_JingChaoZuZhi2, Unigene22024\_JingChaoZuZhi2, Unigene22047\_JingChaoZuZhi2, Unigene22055\_JingChaoZuZhi2, Unigene22091\_JingChaoZuZhi2, Unigene22119\_JingChaoZuZhi2, Unigene22128\_JingChaoZuZhi2, Unigene22145\_JingChaoZuZhi2, Unigene22220\_JingChaoZuZhi2, Unigene2226\_JingChaoZuZhi2, Unigene22295\_JingChaoZuZhi2, Unigene22329\_JingChaoZuZhi2, Unigene22350\_JingChaoZuZhi2, Unigene22372\_JingChaoZuZhi2, Unigene22389\_JingChaoZuZhi2, Unigene22414\_JingChaoZuZhi2, Unigene22455\_JingChaoZuZhi2, Unigene22482\_JingChaoZuZhi2, Unigene22517\_JingChaoZuZhi2, Unigene22566\_JingChaoZuZhi2, Unigene22580\_JingChaoZuZhi2, Unigene22586\_JingChaoZuZhi2, Unigene2258\_JingChaoZuZhi2, Unigene22595\_JingChaoZuZhi2, Unigene22673\_JingChaoZuZhi2, Unigene22708\_JingChaoZuZhi2, Unigene22822\_JingChaoZuZhi2, Unigene22850\_JingChaoZuZhi2, Unigene22896\_JingChaoZuZhi2, Unigene2289\_JingChaoZuZhi2, Unigene22909\_JingChaoZuZhi2, Unigene23164\_JingChaoZuZhi2, Unigene23447\_JingChaoZuZhi2, Unigene23924\_JingChaoZuZhi2, Unigene23978\_JingChaoZuZhi2, Unigene2445\_JingChaoZuZhi2, Unigene24600\_JingChaoZuZhi2, Unigene2469\_JingChaoZuZhi2, Unigene2473\_JingChaoZuZhi2, Unigene2500\_JingChaoZuZhi2, Unigene2510\_JingChaoZuZhi2, Unigene2517\_JingChaoZuZhi2, Unigene25240\_JingChaoZuZhi2, Unigene25278\_JingChaoZuZhi2, Unigene2563\_JingChaoZuZhi2, Unigene25693\_JingChaoZuZhi2, Unigene25962\_JingChaoZuZhi2, Unigene2628\_JingChaoZuZhi2, Unigene26820\_JingChaoZuZhi2, Unigene26987\_JingChaoZuZhi2, Unigene2790\_JingChaoZuZhi2, Unigene28064\_JingChaoZuZhi2, Unigene28243\_JingChaoZuZhi2, Unigene2857\_JingChaoZuZhi2, Unigene28737\_JingChaoZuZhi2, Unigene28793\_JingChaoZuZhi2, Unigene2901\_JingChaoZuZhi2, Unigene29367\_JingChaoZuZhi2, Unigene29519\_JingChaoZuZhi2, Unigene30281\_JingChaoZuZhi2, Unigene30775\_JingChaoZuZhi2, Unigene30851\_JingChaoZuZhi2, Unigene31912\_JingChaoZuZhi2, Unigene31918\_JingChaoZuZhi2, Unigene3235\_JingChaoZuZhi2, Unigene32373\_JingChaoZuZhi2, Unigene324\_JingChaoZuZhi2, Unigene33044\_JingChaoZuZhi2, Unigene33173\_JingChaoZuZhi2, Unigene3321\_JingChaoZuZhi2, Unigene33561\_JingChaoZuZhi2, Unigene3374\_JingChaoZuZhi2, Unigene3413\_JingChaoZuZhi2, Unigene34142\_JingChaoZuZhi2, Unigene34518\_JingChaoZuZhi2, Unigene34785\_JingChaoZuZhi2, Unigene3493\_JingChaoZuZhi2, Unigene35016\_JingChaoZuZhi2, Unigene35080\_JingChaoZuZhi2, Unigene35744\_JingChaoZuZhi2, Unigene35927\_JingChaoZuZhi2, Unigene36257\_JingChaoZuZhi2, Unigene36342\_JingChaoZuZhi2, Unigene36361\_JingChaoZuZhi2, Unigene3640\_JingChaoZuZhi2, Unigene3646\_JingChaoZuZhi2, Unigene3665\_JingChaoZuZhi2, Unigene3671\_JingChaoZuZhi2, Unigene36730\_JingChaoZuZhi2, Unigene3693\_JingChaoZuZhi2, Unigene36974\_JingChaoZuZhi2, Unigene36976\_JingChaoZuZhi2, Unigene37164\_JingChaoZuZhi2, Unigene37665\_JingChaoZuZhi2, Unigene37818\_JingChaoZuZhi2, Unigene3854\_JingChaoZuZhi2, Unigene38616\_JingChaoZuZhi2, Unigene38755\_JingChaoZuZhi2, Unigene39102\_JingChaoZuZhi2, Unigene39148\_JingChaoZuZhi2, Unigene39584\_JingChaoZuZhi2, Unigene395\_JingChaoZuZhi2, Unigene39825\_JingChaoZuZhi2, Unigene39876\_JingChaoZuZhi2, Unigene39914\_JingChaoZuZhi2, Unigene40020\_JingChaoZuZhi2, Unigene4015\_JingChaoZuZhi2, Unigene40200\_JingChaoZuZhi2, Unigene40281\_JingChaoZuZhi2, Unigene4044\_JingChaoZuZhi2, Unigene40919\_JingChaoZuZhi2, Unigene41040\_JingChaoZuZhi2, Unigene41148\_JingChaoZuZhi2, Unigene411\_JingChaoZuZhi2, Unigene42146\_JingChaoZuZhi2, Unigene42248\_JingChaoZuZhi2, Unigene42461\_JingChaoZuZhi2, Unigene4254\_JingChaoZuZhi2, Unigene42836\_JingChaoZuZhi2, Unigene43041\_JingChaoZuZhi2, Unigene43042\_JingChaoZuZhi2, Unigene43147\_JingChaoZuZhi2, Unigene43169\_JingChaoZuZhi2, Unigene43202\_JingChaoZuZhi2, Unigene43249\_JingChaoZuZhi2, Unigene43260\_JingChaoZuZhi2, Unigene4337\_JingChaoZuZhi2, Unigene43512\_JingChaoZuZhi2, Unigene43574\_JingChaoZuZhi2, Unigene4390\_JingChaoZuZhi2, Unigene44190\_JingChaoZuZhi2, Unigene4426\_JingChaoZuZhi2, Unigene44454\_JingChaoZuZhi2, Unigene44516\_JingChaoZuZhi2, Unigene45100\_JingChaoZuZhi2, Unigene45134\_JingChaoZuZhi2, Unigene4529\_JingChaoZuZhi2, Unigene45367\_JingChaoZuZhi2, Unigene4547\_JingChaoZuZhi2, Unigene45593\_JingChaoZuZhi2, Unigene45622\_JingChaoZuZhi2, Unigene45901\_JingChaoZuZhi2, Unigene45940\_JingChaoZuZhi2, Unigene4599\_JingChaoZuZhi2, Unigene46240\_JingChaoZuZhi2, Unigene46407\_JingChaoZuZhi2, Unigene4713\_JingChaoZuZhi2, Unigene47578\_JingChaoZuZhi2, Unigene4758\_JingChaoZuZhi2, Unigene47949\_JingChaoZuZhi2, Unigene4829\_JingChaoZuZhi2, Unigene4853\_JingChaoZuZhi2, Unigene4873\_JingChaoZuZhi2, Unigene49277\_JingChaoZuZhi2, Unigene49387\_JingChaoZuZhi2, Unigene49761\_JingChaoZuZhi2, Unigene49798\_JingChaoZuZhi2, Unigene49973\_JingChaoZuZhi2, Unigene50540\_JingChaoZuZhi2, Unigene50593\_JingChaoZuZhi2, Unigene50668\_JingChaoZuZhi2, Unigene50713\_JingChaoZuZhi2, Unigene50744\_JingChaoZuZhi2, Unigene5108\_JingChaoZuZhi2, Unigene51090\_JingChaoZuZhi2, Unigene51115\_JingChaoZuZhi2, Unigene5125\_JingChaoZuZhi2, Unigene5126\_JingChaoZuZhi2, Unigene51272\_JingChaoZuZhi2, Unigene51467\_JingChaoZuZhi2, Unigene51843\_JingChaoZuZhi2, Unigene52100\_JingChaoZuZhi2, Unigene52125\_JingChaoZuZhi2, Unigene52456\_JingChaoZuZhi2, Unigene524\_JingChaoZuZhi2, Unigene52638\_JingChaoZuZhi2, Unigene52763\_JingChaoZuZhi2, Unigene52860\_JingChaoZuZhi2, Unigene5298\_JingChaoZuZhi2, Unigene53131\_JingChaoZuZhi2, Unigene53138\_JingChaoZuZhi2, Unigene5335\_JingChaoZuZhi2, Unigene53484\_JingChaoZuZhi2, Unigene53500\_JingChaoZuZhi2, Unigene53603\_JingChaoZuZhi2, Unigene53751\_JingChaoZuZhi2, Unigene53958\_JingChaoZuZhi2, Unigene54081\_JingChaoZuZhi2, Unigene54153\_JingChaoZuZhi2, Unigene54260\_JingChaoZuZhi2, Unigene54470\_JingChaoZuZhi2, Unigene54744\_JingChaoZuZhi2, Unigene54904\_JingChaoZuZhi2, Unigene54918\_JingChaoZuZhi2, Unigene54974\_JingChaoZuZhi2, Unigene54986\_JingChaoZuZhi2, Unigene55009\_JingChaoZuZhi2, Unigene55019\_JingChaoZuZhi2, Unigene55385\_JingChaoZuZhi2, Unigene55839\_JingChaoZuZhi2, Unigene55917\_JingChaoZuZhi2, Unigene5617\_JingChaoZuZhi2, Unigene56421\_JingChaoZuZhi2, Unigene5650\_JingChaoZuZhi2, Unigene56560\_JingChaoZuZhi2, Unigene56660\_JingChaoZuZhi2, Unigene57240\_JingChaoZuZhi2, Unigene57247\_JingChaoZuZhi2, Unigene57597\_JingChaoZuZhi2, Unigene58017\_JingChaoZuZhi2, Unigene5809\_JingChaoZuZhi2, Unigene58169\_JingChaoZuZhi2, Unigene58474\_JingChaoZuZhi2, Unigene58675\_JingChaoZuZhi2, Unigene5881\_JingChaoZuZhi2, Unigene5896\_JingChaoZuZhi2, Unigene59101\_JingChaoZuZhi2, Unigene59122\_JingChaoZuZhi2, Unigene59146\_JingChaoZuZhi2, Unigene59240\_JingChaoZuZhi2, Unigene59265\_JingChaoZuZhi2, Unigene59336\_JingChaoZuZhi2, Unigene59433\_JingChaoZuZhi2, Unigene59684\_JingChaoZuZhi2, Unigene60121\_JingChaoZuZhi2, Unigene60180\_JingChaoZuZhi2, Unigene60551\_JingChaoZuZhi2, Unigene60586\_JingChaoZuZhi2, Unigene60607\_JingChaoZuZhi2, Unigene6072\_JingChaoZuZhi2, Unigene60930\_JingChaoZuZhi2, Unigene60990\_JingChaoZuZhi2, Unigene61009\_JingChaoZuZhi2, Unigene61244\_JingChaoZuZhi2, Unigene6135\_JingChaoZuZhi2, Unigene61474\_JingChaoZuZhi2, Unigene6158\_JingChaoZuZhi2, Unigene6171\_JingChaoZuZhi2, Unigene61736\_JingChaoZuZhi2, Unigene6175\_JingChaoZuZhi2, Unigene61879\_JingChaoZuZhi2, Unigene61880\_JingChaoZuZhi2, Unigene61911\_JingChaoZuZhi2, Unigene61931\_JingChaoZuZhi2, Unigene62100\_JingChaoZuZhi2, Unigene6215\_JingChaoZuZhi2, Unigene62218\_JingChaoZuZhi2, Unigene62257\_JingChaoZuZhi2, Unigene6233\_JingChaoZuZhi2, Unigene62606\_JingChaoZuZhi2, Unigene62695\_JingChaoZuZhi2, Unigene62745\_JingChaoZuZhi2, Unigene62786\_JingChaoZuZhi2, Unigene63000\_JingChaoZuZhi2, Unigene63052\_JingChaoZuZhi2, Unigene63091\_JingChaoZuZhi2, Unigene63145\_JingChaoZuZhi2, Unigene63323\_JingChaoZuZhi2, Unigene63384\_JingChaoZuZhi2, Unigene63406\_JingChaoZuZhi2, Unigene63424\_JingChaoZuZhi2, Unigene63449\_JingChaoZuZhi2, Unigene63584\_JingChaoZuZhi2, Unigene63604\_JingChaoZuZhi2, Unigene63615\_JingChaoZuZhi2, Unigene63812\_JingChaoZuZhi2, Unigene63873\_JingChaoZuZhi2, Unigene63925\_JingChaoZuZhi2, Unigene64208\_JingChaoZuZhi2, Unigene64227\_JingChaoZuZhi2, Unigene64329\_JingChaoZuZhi2, Unigene6438\_JingChaoZuZhi2, Unigene64452\_JingChaoZuZhi2, Unigene64458\_JingChaoZuZhi2, Unigene64583\_JingChaoZuZhi2, Unigene6460\_JingChaoZuZhi2, Unigene64650\_JingChaoZuZhi2, Unigene6467\_JingChaoZuZhi2, Unigene64692\_JingChaoZuZhi2, Unigene64843\_JingChaoZuZhi2, Unigene6493\_JingChaoZuZhi2, Unigene65022\_JingChaoZuZhi2, Unigene65129\_JingChaoZuZhi2, Unigene65150\_JingChaoZuZhi2, Unigene65187\_JingChaoZuZhi2, Unigene65326\_JingChaoZuZhi2, Unigene65332\_JingChaoZuZhi2, Unigene65375\_JingChaoZuZhi2, Unigene6539\_JingChaoZuZhi2, Unigene6567\_JingChaoZuZhi2, Unigene65773\_JingChaoZuZhi2, Unigene6605\_JingChaoZuZhi2, Unigene66087\_JingChaoZuZhi2, Unigene66253\_JingChaoZuZhi2, Unigene6634\_JingChaoZuZhi2, Unigene6648\_JingChaoZuZhi2, Unigene6651\_JingChaoZuZhi2, Unigene66559\_JingChaoZuZhi2, Unigene66590\_JingChaoZuZhi2, Unigene66986\_JingChaoZuZhi2, Unigene67005\_JingChaoZuZhi2, Unigene67063\_JingChaoZuZhi2, Unigene67070\_JingChaoZuZhi2, Unigene67177\_JingChaoZuZhi2, Unigene67249\_JingChaoZuZhi2, Unigene67279\_JingChaoZuZhi2, Unigene67777\_JingChaoZuZhi2, Unigene67788\_JingChaoZuZhi2, Unigene67958\_JingChaoZuZhi2, Unigene6796\_JingChaoZuZhi2, Unigene6797\_JingChaoZuZhi2, Unigene68108\_JingChaoZuZhi2, Unigene68139\_JingChaoZuZhi2, Unigene6815\_JingChaoZuZhi2, Unigene68191\_JingChaoZuZhi2, Unigene6828\_JingChaoZuZhi2, Unigene683\_JingChaoZuZhi2, Unigene68527\_JingChaoZuZhi2, Unigene68672\_JingChaoZuZhi2, Unigene68716\_JingChaoZuZhi2, Unigene6872\_JingChaoZuZhi2, Unigene69092\_JingChaoZuZhi2, Unigene69100\_JingChaoZuZhi2, Unigene69141\_JingChaoZuZhi2, Unigene6914\_JingChaoZuZhi2, Unigene691\_JingChaoZuZhi2, Unigene69221\_JingChaoZuZhi2, Unigene69386\_JingChaoZuZhi2, Unigene69562\_JingChaoZuZhi2, Unigene69565\_JingChaoZuZhi2, Unigene69602\_JingChaoZuZhi2, Unigene69775\_JingChaoZuZhi2, Unigene69934\_JingChaoZuZhi2, Unigene70045\_JingChaoZuZhi2, Unigene70063\_JingChaoZuZhi2, Unigene70168\_JingChaoZuZhi2, Unigene7016\_JingChaoZuZhi2, Unigene70329\_JingChaoZuZhi2, Unigene7032\_JingChaoZuZhi2, Unigene70970\_JingChaoZuZhi2, Unigene71043\_JingChaoZuZhi2, Unigene71132\_JingChaoZuZhi2, Unigene7116\_JingChaoZuZhi2, Unigene7118\_JingChaoZuZhi2, Unigene71213\_JingChaoZuZhi2, Unigene71241\_JingChaoZuZhi2, Unigene71385\_JingChaoZuZhi2, Unigene7151\_JingChaoZuZhi2, Unigene71546\_JingChaoZuZhi2, Unigene71592\_JingChaoZuZhi2, Unigene71630\_JingChaoZuZhi2, Unigene71774\_JingChaoZuZhi2, Unigene71808\_JingChaoZuZhi2, Unigene7182\_JingChaoZuZhi2, Unigene71932\_JingChaoZuZhi2, Unigene72033\_JingChaoZuZhi2, Unigene7206\_JingChaoZuZhi2, Unigene72249\_JingChaoZuZhi2, Unigene7224\_JingChaoZuZhi2, Unigene72334\_JingChaoZuZhi2, Unigene72351\_JingChaoZuZhi2, Unigene72503\_JingChaoZuZhi2, Unigene72521\_JingChaoZuZhi2, Unigene7268\_JingChaoZuZhi2, Unigene72708\_JingChaoZuZhi2, Unigene72769\_JingChaoZuZhi2, Unigene72832\_JingChaoZuZhi2, Unigene72931\_JingChaoZuZhi2, Unigene73015\_JingChaoZuZhi2, Unigene73241\_JingChaoZuZhi2, Unigene73257\_JingChaoZuZhi2, Unigene7326\_JingChaoZuZhi2, Unigene73446\_JingChaoZuZhi2, Unigene73475\_JingChaoZuZhi2, Unigene73550\_JingChaoZuZhi2, Unigene73817\_JingChaoZuZhi2, Unigene73887\_JingChaoZuZhi2, Unigene74027\_JingChaoZuZhi2, Unigene74050\_JingChaoZuZhi2, Unigene74193\_JingChaoZuZhi2, Unigene741\_JingChaoZuZhi2, Unigene74212\_JingChaoZuZhi2, Unigene74221\_JingChaoZuZhi2, Unigene74259\_JingChaoZuZhi2, Unigene74298\_JingChaoZuZhi2, Unigene742\_JingChaoZuZhi2, Unigene74474\_JingChaoZuZhi2, Unigene74475\_JingChaoZuZhi2, Unigene74521\_JingChaoZuZhi2, Unigene74609\_JingChaoZuZhi2, Unigene74719\_JingChaoZuZhi2, Unigene74730\_JingChaoZuZhi2, Unigene74854\_JingChaoZuZhi2, Unigene74897\_JingChaoZuZhi2, Unigene7489\_JingChaoZuZhi2, Unigene75009\_JingChaoZuZhi2, Unigene7508\_JingChaoZuZhi2, Unigene75112\_JingChaoZuZhi2, Unigene75167\_JingChaoZuZhi2, Unigene75317\_JingChaoZuZhi2, Unigene75507\_JingChaoZuZhi2, Unigene75902\_JingChaoZuZhi2, Unigene75924\_JingChaoZuZhi2, Unigene75946\_JingChaoZuZhi2, Unigene76002\_JingChaoZuZhi2, Unigene76111\_JingChaoZuZhi2, Unigene76214\_JingChaoZuZhi2, Unigene76269\_JingChaoZuZhi2, Unigene76302\_JingChaoZuZhi2, Unigene7634\_JingChaoZuZhi2, Unigene76400\_JingChaoZuZhi2, Unigene76515\_JingChaoZuZhi2, Unigene7663\_JingChaoZuZhi2, Unigene76776\_JingChaoZuZhi2, Unigene76785\_JingChaoZuZhi2, Unigene76819\_JingChaoZuZhi2, Unigene77054\_JingChaoZuZhi2, Unigene77200\_JingChaoZuZhi2, Unigene77406\_JingChaoZuZhi2, Unigene77413\_JingChaoZuZhi2, Unigene77467\_JingChaoZuZhi2, Unigene77510\_JingChaoZuZhi2, Unigene77520\_JingChaoZuZhi2, Unigene77712\_JingChaoZuZhi2, Unigene77724\_JingChaoZuZhi2, Unigene7794\_JingChaoZuZhi2, Unigene77954\_JingChaoZuZhi2, Unigene77\_JingChaoZuZhi2, Unigene78009\_JingChaoZuZhi2, Unigene78112\_JingChaoZuZhi2, Unigene78288\_JingChaoZuZhi2, Unigene78325\_JingChaoZuZhi2, Unigene78361\_JingChaoZuZhi2, Unigene78387\_JingChaoZuZhi2, Unigene78410\_JingChaoZuZhi2, Unigene7850\_JingChaoZuZhi2, Unigene78521\_JingChaoZuZhi2, Unigene78522\_JingChaoZuZhi2, Unigene78555\_JingChaoZuZhi2, Unigene78574\_JingChaoZuZhi2, Unigene78636\_JingChaoZuZhi2, Unigene78818\_JingChaoZuZhi2, Unigene79058\_JingChaoZuZhi2, Unigene79118\_JingChaoZuZhi2, Unigene7911\_JingChaoZuZhi2, Unigene79141\_JingChaoZuZhi2, Unigene79186\_JingChaoZuZhi2, Unigene79257\_JingChaoZuZhi2, Unigene79358\_JingChaoZuZhi2, Unigene7939\_JingChaoZuZhi2, Unigene79411\_JingChaoZuZhi2, Unigene79433\_JingChaoZuZhi2, Unigene79443\_JingChaoZuZhi2, Unigene79653\_JingChaoZuZhi2, Unigene7970\_JingChaoZuZhi2, Unigene79726\_JingChaoZuZhi2, Unigene79789\_JingChaoZuZhi2, Unigene79824\_JingChaoZuZhi2, Unigene79864\_JingChaoZuZhi2, Unigene79871\_JingChaoZuZhi2, Unigene7987\_JingChaoZuZhi2, Unigene79940\_JingChaoZuZhi2, Unigene80095\_JingChaoZuZhi2, Unigene80113\_JingChaoZuZhi2, Unigene80123\_JingChaoZuZhi2, Unigene80143\_JingChaoZuZhi2, Unigene80212\_JingChaoZuZhi2, Unigene80403\_JingChaoZuZhi2, Unigene80519\_JingChaoZuZhi2, Unigene80619\_JingChaoZuZhi2, Unigene80649\_JingChaoZuZhi2, Unigene80684\_JingChaoZuZhi2, Unigene80893\_JingChaoZuZhi2, Unigene80936\_JingChaoZuZhi2, Unigene80954\_JingChaoZuZhi2, Unigene80980\_JingChaoZuZhi2, Unigene81145\_JingChaoZuZhi2, Unigene81167\_JingChaoZuZhi2, Unigene81253\_JingChaoZuZhi2, Unigene81271\_JingChaoZuZhi2, Unigene81327\_JingChaoZuZhi2, Unigene81372\_JingChaoZuZhi2, Unigene81434\_JingChaoZuZhi2, Unigene81440\_JingChaoZuZhi2, Unigene81450\_JingChaoZuZhi2, Unigene81561\_JingChaoZuZhi2, Unigene81599\_JingChaoZuZhi2, Unigene81686\_JingChaoZuZhi2, Unigene81716\_JingChaoZuZhi2, Unigene81719\_JingChaoZuZhi2, Unigene81757\_JingChaoZuZhi2, Unigene81803\_JingChaoZuZhi2, Unigene81825\_JingChaoZuZhi2, Unigene81858\_JingChaoZuZhi2, Unigene81864\_JingChaoZuZhi2, Unigene8191\_JingChaoZuZhi2, Unigene81955\_JingChaoZuZhi2, Unigene82047\_JingChaoZuZhi2, Unigene82070\_JingChaoZuZhi2, Unigene82111\_JingChaoZuZhi2, Unigene82117\_JingChaoZuZhi2, Unigene82144\_JingChaoZuZhi2, Unigene82215\_JingChaoZuZhi2, Unigene82301\_JingChaoZuZhi2, Unigene82340\_JingChaoZuZhi2, Unigene8237\_JingChaoZuZhi2, Unigene82489\_JingChaoZuZhi2, Unigene82521\_JingChaoZuZhi2, Unigene82578\_JingChaoZuZhi2, Unigene82688\_JingChaoZuZhi2, Unigene82760\_JingChaoZuZhi2, Unigene82921\_JingChaoZuZhi2, Unigene82930\_JingChaoZuZhi2, Unigene83079\_JingChaoZuZhi2, Unigene83188\_JingChaoZuZhi2, Unigene83214\_JingChaoZuZhi2, Unigene83267\_JingChaoZuZhi2, Unigene8328\_JingChaoZuZhi2, Unigene83433\_JingChaoZuZhi2, Unigene83529\_JingChaoZuZhi2, Unigene83588\_JingChaoZuZhi2, Unigene83766\_JingChaoZuZhi2, Unigene83782\_JingChaoZuZhi2, Unigene83825\_JingChaoZuZhi2, Unigene83872\_JingChaoZuZhi2, Unigene8392\_JingChaoZuZhi2, Unigene83988\_JingChaoZuZhi2, Unigene8406\_JingChaoZuZhi2, Unigene84076\_JingChaoZuZhi2, Unigene84109\_JingChaoZuZhi2, Unigene84152\_JingChaoZuZhi2, Unigene84168\_JingChaoZuZhi2, Unigene841\_JingChaoZuZhi2, Unigene84206\_JingChaoZuZhi2, Unigene84212\_JingChaoZuZhi2, Unigene84352\_JingChaoZuZhi2, Unigene8443\_JingChaoZuZhi2, Unigene84533\_JingChaoZuZhi2, Unigene84563\_JingChaoZuZhi2, Unigene84566\_JingChaoZuZhi2, Unigene84631\_JingChaoZuZhi2, Unigene84748\_JingChaoZuZhi2, Unigene84924\_JingChaoZuZhi2, Unigene84945\_JingChaoZuZhi2, Unigene84993\_JingChaoZuZhi2, Unigene85193\_JingChaoZuZhi2, Unigene851\_JingChaoZuZhi2, Unigene85306\_JingChaoZuZhi2, Unigene85314\_JingChaoZuZhi2, Unigene85472\_JingChaoZuZhi2, Unigene85770\_JingChaoZuZhi2, Unigene85902\_JingChaoZuZhi2, Unigene85923\_JingChaoZuZhi2, Unigene8600\_JingChaoZuZhi2, Unigene86020\_JingChaoZuZhi2, Unigene86091\_JingChaoZuZhi2, Unigene86119\_JingChaoZuZhi2, Unigene86222\_JingChaoZuZhi2, Unigene86259\_JingChaoZuZhi2, Unigene86349\_JingChaoZuZhi2, Unigene86504\_JingChaoZuZhi2, Unigene86506\_JingChaoZuZhi2, Unigene86546\_JingChaoZuZhi2, Unigene86579\_JingChaoZuZhi2, Unigene8658\_JingChaoZuZhi2, Unigene8661\_JingChaoZuZhi2, Unigene86669\_JingChaoZuZhi2, Unigene86741\_JingChaoZuZhi2, Unigene86807\_JingChaoZuZhi2, Unigene87082\_JingChaoZuZhi2, Unigene8709\_JingChaoZuZhi2, Unigene87191\_JingChaoZuZhi2, Unigene87203\_JingChaoZuZhi2, Unigene87256\_JingChaoZuZhi2, Unigene87362\_JingChaoZuZhi2, Unigene87365\_JingChaoZuZhi2, Unigene87459\_JingChaoZuZhi2, Unigene87478\_JingChaoZuZhi2, Unigene87488\_JingChaoZuZhi2, Unigene87532\_JingChaoZuZhi2, Unigene87533\_JingChaoZuZhi2, Unigene87731\_JingChaoZuZhi2, Unigene87774\_JingChaoZuZhi2, Unigene87853\_JingChaoZuZhi2, Unigene87946\_JingChaoZuZhi2, Unigene88059\_JingChaoZuZhi2, Unigene88307\_JingChaoZuZhi2, Unigene8833\_JingChaoZuZhi2, Unigene88342\_JingChaoZuZhi2, Unigene88400\_JingChaoZuZhi2, Unigene88406\_JingChaoZuZhi2, Unigene88442\_JingChaoZuZhi2, Unigene88470\_JingChaoZuZhi2, Unigene88544\_JingChaoZuZhi2, Unigene88608\_JingChaoZuZhi2, Unigene88683\_JingChaoZuZhi2, Unigene88738\_JingChaoZuZhi2, Unigene88741\_JingChaoZuZhi2, Unigene88776\_JingChaoZuZhi2, Unigene88778\_JingChaoZuZhi2, Unigene88779\_JingChaoZuZhi2, Unigene88781\_JingChaoZuZhi2, Unigene88797\_JingChaoZuZhi2, Unigene88830\_JingChaoZuZhi2, Unigene88837\_JingChaoZuZhi2, Unigene88945\_JingChaoZuZhi2, Unigene88979\_JingChaoZuZhi2, Unigene88988\_JingChaoZuZhi2, Unigene89013\_JingChaoZuZhi2, Unigene89045\_JingChaoZuZhi2, Unigene89060\_JingChaoZuZhi2, Unigene89093\_JingChaoZuZhi2, Unigene89121\_JingChaoZuZhi2, Unigene89161\_JingChaoZuZhi2, Unigene89178\_JingChaoZuZhi2, Unigene89194\_JingChaoZuZhi2, Unigene89323\_JingChaoZuZhi2, Unigene89337\_JingChaoZuZhi2, Unigene89352\_JingChaoZuZhi2, Unigene89367\_JingChaoZuZhi2, Unigene89541\_JingChaoZuZhi2, Unigene89596\_JingChaoZuZhi2, Unigene89714\_JingChaoZuZhi2, Unigene89741\_JingChaoZuZhi2, Unigene89746\_JingChaoZuZhi2, Unigene89751\_JingChaoZuZhi2, Unigene89769\_JingChaoZuZhi2, Unigene89834\_JingChaoZuZhi2, Unigene89867\_JingChaoZuZhi2, Unigene89901\_JingChaoZuZhi2, Unigene89942\_JingChaoZuZhi2, Unigene89956\_JingChaoZuZhi2, Unigene89986\_JingChaoZuZhi2, Unigene90027\_JingChaoZuZhi2, Unigene90038\_JingChaoZuZhi2, Unigene90066\_JingChaoZuZhi2, Unigene90071\_JingChaoZuZhi2, Unigene90209\_JingChaoZuZhi2, Unigene90220\_JingChaoZuZhi2, Unigene90341\_JingChaoZuZhi2, Unigene9050\_JingChaoZuZhi2, Unigene90530\_JingChaoZuZhi2, Unigene90564\_JingChaoZuZhi2, Unigene90650\_JingChaoZuZhi2, Unigene90714\_JingChaoZuZhi2, Unigene90779\_JingChaoZuZhi2, Unigene90832\_JingChaoZuZhi2, Unigene90953\_JingChaoZuZhi2, Unigene90995\_JingChaoZuZhi2, Unigene91099\_JingChaoZuZhi2, Unigene91119\_JingChaoZuZhi2, Unigene91148\_JingChaoZuZhi2, Unigene91163\_JingChaoZuZhi2, Unigene91179\_JingChaoZuZhi2, Unigene91233\_JingChaoZuZhi2, Unigene91382\_JingChaoZuZhi2, Unigene91390\_JingChaoZuZhi2, Unigene91456\_JingChaoZuZhi2, Unigene91475\_JingChaoZuZhi2, Unigene91487\_JingChaoZuZhi2, Unigene91529\_JingChaoZuZhi2, Unigene91553\_JingChaoZuZhi2, Unigene91589\_JingChaoZuZhi2, Unigene91703\_JingChaoZuZhi2, Unigene91837\_JingChaoZuZhi2, Unigene91848\_JingChaoZuZhi2, Unigene92016\_JingChaoZuZhi2, Unigene92019\_JingChaoZuZhi2, Unigene92090\_JingChaoZuZhi2, Unigene92346\_JingChaoZuZhi2, Unigene92466\_JingChaoZuZhi2, Unigene92510\_JingChaoZuZhi2, Unigene92580\_JingChaoZuZhi2, Unigene92681\_JingChaoZuZhi2, Unigene92700\_JingChaoZuZhi2, Unigene92715\_JingChaoZuZhi2, Unigene92780\_JingChaoZuZhi2, Unigene92832\_JingChaoZuZhi2, Unigene92838\_JingChaoZuZhi2, Unigene92846\_JingChaoZuZhi2, Unigene92951\_JingChaoZuZhi2, Unigene92962\_JingChaoZuZhi2, Unigene93073\_JingChaoZuZhi2, Unigene93125\_JingChaoZuZhi2, Unigene93195\_JingChaoZuZhi2, Unigene93246\_JingChaoZuZhi2, Unigene93270\_JingChaoZuZhi2, Unigene93303\_JingChaoZuZhi2, Unigene93326\_JingChaoZuZhi2, Unigene93439\_JingChaoZuZhi2, Unigene93552\_JingChaoZuZhi2, Unigene93562\_JingChaoZuZhi2, Unigene93573\_JingChaoZuZhi2, Unigene93590\_JingChaoZuZhi2, Unigene93592\_JingChaoZuZhi2, Unigene93610\_JingChaoZuZhi2, Unigene93702\_JingChaoZuZhi2, Unigene93742\_JingChaoZuZhi2, Unigene93780\_JingChaoZuZhi2, Unigene93782\_JingChaoZuZhi2, Unigene93793\_JingChaoZuZhi2, Unigene93853\_JingChaoZuZhi2, Unigene93912\_JingChaoZuZhi2, Unigene93979\_JingChaoZuZhi2, Unigene94083\_JingChaoZuZhi2, Unigene94110\_JingChaoZuZhi2, Unigene94120\_JingChaoZuZhi2, Unigene94129\_JingChaoZuZhi2, Unigene94134\_JingChaoZuZhi2, Unigene94242\_JingChaoZuZhi2, Unigene94272\_JingChaoZuZhi2, Unigene9428\_JingChaoZuZhi2, Unigene94358\_JingChaoZuZhi2, Unigene94370\_JingChaoZuZhi2, Unigene94404\_JingChaoZuZhi2, Unigene94471\_JingChaoZuZhi2, Unigene94520\_JingChaoZuZhi2, Unigene94584\_JingChaoZuZhi2, Unigene94687\_JingChaoZuZhi2, Unigene94711\_JingChaoZuZhi2, Unigene94747\_JingChaoZuZhi2, Unigene9490\_JingChaoZuZhi2, Unigene94950\_JingChaoZuZhi2, Unigene94968\_JingChaoZuZhi2, Unigene95020\_JingChaoZuZhi2, Unigene95088\_JingChaoZuZhi2, Unigene95098\_JingChaoZuZhi2, Unigene95119\_JingChaoZuZhi2, Unigene9517\_JingChaoZuZhi2, Unigene951\_JingChaoZuZhi2, Unigene95210\_JingChaoZuZhi2, Unigene95238\_JingChaoZuZhi2, Unigene9530\_JingChaoZuZhi2, Unigene95315\_JingChaoZuZhi2, Unigene95371\_JingChaoZuZhi2, Unigene9540\_JingChaoZuZhi2, Unigene95565\_JingChaoZuZhi2, Unigene95568\_JingChaoZuZhi2, Unigene95589\_JingChaoZuZhi2, Unigene95677\_JingChaoZuZhi2, Unigene95770\_JingChaoZuZhi2, Unigene95774\_JingChaoZuZhi2, Unigene95807\_JingChaoZuZhi2, Unigene95812\_JingChaoZuZhi2, Unigene95863\_JingChaoZuZhi2, Unigene95875\_JingChaoZuZhi2, Unigene95881\_JingChaoZuZhi2, Unigene95883\_JingChaoZuZhi2, Unigene95907\_JingChaoZuZhi2, Unigene95980\_JingChaoZuZhi2, Unigene95988\_JingChaoZuZhi2, Unigene95993\_JingChaoZuZhi2, Unigene96024\_JingChaoZuZhi2, Unigene96124\_JingChaoZuZhi2, Unigene96234\_JingChaoZuZhi2, Unigene9688\_JingChaoZuZhi2, Unigene9722\_JingChaoZuZhi2, Unigene9739\_JingChaoZuZhi2, Unigene9743\_JingChaoZuZhi2, Unigene9807\_JingChaoZuZhi2, Unigene9833\_JingChaoZuZhi2, Unigene9988\_JingChaoZuZhi2, Unigene9992\_JingChaoZuZhi2 |
| 6 | Vibrio cholerae infection | Unigene10047\_JingChaoZuZhi2, Unigene10128\_JingChaoZuZhi2, Unigene10149\_JingChaoZuZhi2, Unigene10259\_JingChaoZuZhi2, Unigene10318\_JingChaoZuZhi2, Unigene10320\_JingChaoZuZhi2, Unigene10362\_JingChaoZuZhi2, Unigene10368\_JingChaoZuZhi2, Unigene10380\_JingChaoZuZhi2, Unigene1060\_JingChaoZuZhi2, Unigene10758\_JingChaoZuZhi2, Unigene10\_JingChaoZuZhi2, Unigene11048\_JingChaoZuZhi2, Unigene11060\_JingChaoZuZhi2, Unigene11064\_JingChaoZuZhi2, Unigene11095\_JingChaoZuZhi2, Unigene11125\_JingChaoZuZhi2, Unigene11130\_JingChaoZuZhi2, Unigene1115\_JingChaoZuZhi2, Unigene11320\_JingChaoZuZhi2, Unigene11408\_JingChaoZuZhi2, Unigene11483\_JingChaoZuZhi2, Unigene11524\_JingChaoZuZhi2, Unigene1154\_JingChaoZuZhi2, Unigene11560\_JingChaoZuZhi2, Unigene1162\_JingChaoZuZhi2, Unigene11668\_JingChaoZuZhi2, Unigene11730\_JingChaoZuZhi2, Unigene11830\_JingChaoZuZhi2, Unigene11889\_JingChaoZuZhi2, Unigene11969\_JingChaoZuZhi2, Unigene11996\_JingChaoZuZhi2, Unigene12011\_JingChaoZuZhi2, Unigene12052\_JingChaoZuZhi2, Unigene12066\_JingChaoZuZhi2, Unigene12091\_JingChaoZuZhi2, Unigene12193\_JingChaoZuZhi2, Unigene12244\_JingChaoZuZhi2, Unigene12253\_JingChaoZuZhi2, Unigene12296\_JingChaoZuZhi2, Unigene12297\_JingChaoZuZhi2, Unigene12311\_JingChaoZuZhi2, Unigene12329\_JingChaoZuZhi2, Unigene12361\_JingChaoZuZhi2, Unigene12543\_JingChaoZuZhi2, Unigene1256\_JingChaoZuZhi2, Unigene12655\_JingChaoZuZhi2, Unigene12669\_JingChaoZuZhi2, Unigene12728\_JingChaoZuZhi2, Unigene12897\_JingChaoZuZhi2, Unigene12904\_JingChaoZuZhi2, Unigene12906\_JingChaoZuZhi2, Unigene12912\_JingChaoZuZhi2, Unigene12913\_JingChaoZuZhi2, Unigene12985\_JingChaoZuZhi2, Unigene13016\_JingChaoZuZhi2, Unigene13036\_JingChaoZuZhi2, Unigene13112\_JingChaoZuZhi2, Unigene13225\_JingChaoZuZhi2, Unigene13228\_JingChaoZuZhi2, Unigene13269\_JingChaoZuZhi2, Unigene13297\_JingChaoZuZhi2, Unigene13381\_JingChaoZuZhi2, Unigene13487\_JingChaoZuZhi2, Unigene13493\_JingChaoZuZhi2, Unigene13607\_JingChaoZuZhi2, Unigene13720\_JingChaoZuZhi2, Unigene13770\_JingChaoZuZhi2, Unigene13864\_JingChaoZuZhi2, Unigene13875\_JingChaoZuZhi2, Unigene13880\_JingChaoZuZhi2, Unigene13890\_JingChaoZuZhi2, Unigene13914\_JingChaoZuZhi2, Unigene13941\_JingChaoZuZhi2, Unigene13961\_JingChaoZuZhi2, Unigene13966\_JingChaoZuZhi2, Unigene13975\_JingChaoZuZhi2, Unigene13992\_JingChaoZuZhi2, Unigene14074\_JingChaoZuZhi2, Unigene14104\_JingChaoZuZhi2, Unigene14322\_JingChaoZuZhi2, Unigene14338\_JingChaoZuZhi2, Unigene1437\_JingChaoZuZhi2, Unigene14393\_JingChaoZuZhi2, Unigene14409\_JingChaoZuZhi2, Unigene14438\_JingChaoZuZhi2, Unigene14610\_JingChaoZuZhi2, Unigene14684\_JingChaoZuZhi2, Unigene14706\_JingChaoZuZhi2, Unigene14717\_JingChaoZuZhi2, Unigene14770\_JingChaoZuZhi2, Unigene14792\_JingChaoZuZhi2, Unigene147\_JingChaoZuZhi2, Unigene14878\_JingChaoZuZhi2, Unigene14903\_JingChaoZuZhi2, Unigene14914\_JingChaoZuZhi2, Unigene14928\_JingChaoZuZhi2, Unigene14963\_JingChaoZuZhi2, Unigene15010\_JingChaoZuZhi2, Unigene15088\_JingChaoZuZhi2, Unigene15129\_JingChaoZuZhi2, Unigene15146\_JingChaoZuZhi2, Unigene15160\_JingChaoZuZhi2, Unigene15172\_JingChaoZuZhi2, Unigene15229\_JingChaoZuZhi2, Unigene15276\_JingChaoZuZhi2, Unigene15304\_JingChaoZuZhi2, Unigene15331\_JingChaoZuZhi2, Unigene15475\_JingChaoZuZhi2, Unigene15536\_JingChaoZuZhi2, Unigene15626\_JingChaoZuZhi2, Unigene15651\_JingChaoZuZhi2, Unigene15677\_JingChaoZuZhi2, Unigene15763\_JingChaoZuZhi2, Unigene15803\_JingChaoZuZhi2, Unigene15840\_JingChaoZuZhi2, Unigene16004\_JingChaoZuZhi2, Unigene16064\_JingChaoZuZhi2, Unigene1606\_JingChaoZuZhi2, Unigene16266\_JingChaoZuZhi2, Unigene16283\_JingChaoZuZhi2, Unigene16300\_JingChaoZuZhi2, Unigene16312\_JingChaoZuZhi2, Unigene16410\_JingChaoZuZhi2, Unigene16578\_JingChaoZuZhi2, Unigene16591\_JingChaoZuZhi2, Unigene16596\_JingChaoZuZhi2, Unigene16780\_JingChaoZuZhi2, Unigene16785\_JingChaoZuZhi2, Unigene16791\_JingChaoZuZhi2, Unigene16833\_JingChaoZuZhi2, Unigene16844\_JingChaoZuZhi2, Unigene16855\_JingChaoZuZhi2, Unigene16903\_JingChaoZuZhi2, Unigene16938\_JingChaoZuZhi2, Unigene169\_JingChaoZuZhi2, Unigene17015\_JingChaoZuZhi2, Unigene17020\_JingChaoZuZhi2, Unigene17026\_JingChaoZuZhi2, Unigene1710\_JingChaoZuZhi2, Unigene1719\_JingChaoZuZhi2, Unigene17219\_JingChaoZuZhi2, Unigene17280\_JingChaoZuZhi2, Unigene17288\_JingChaoZuZhi2, Unigene17425\_JingChaoZuZhi2, Unigene1746\_JingChaoZuZhi2, Unigene1749\_JingChaoZuZhi2, Unigene17501\_JingChaoZuZhi2, Unigene17513\_JingChaoZuZhi2, Unigene17605\_JingChaoZuZhi2, Unigene17735\_JingChaoZuZhi2, Unigene17758\_JingChaoZuZhi2, Unigene17764\_JingChaoZuZhi2, Unigene17810\_JingChaoZuZhi2, Unigene17983\_JingChaoZuZhi2, Unigene18042\_JingChaoZuZhi2, Unigene18075\_JingChaoZuZhi2, Unigene18089\_JingChaoZuZhi2, Unigene18101\_JingChaoZuZhi2, Unigene18107\_JingChaoZuZhi2, Unigene18126\_JingChaoZuZhi2, Unigene18211\_JingChaoZuZhi2, Unigene18292\_JingChaoZuZhi2, Unigene18307\_JingChaoZuZhi2, Unigene18325\_JingChaoZuZhi2, Unigene18333\_JingChaoZuZhi2, Unigene18375\_JingChaoZuZhi2, Unigene18435\_JingChaoZuZhi2, Unigene18464\_JingChaoZuZhi2, Unigene18465\_JingChaoZuZhi2, Unigene18483\_JingChaoZuZhi2, Unigene18488\_JingChaoZuZhi2, Unigene18588\_JingChaoZuZhi2, Unigene18656\_JingChaoZuZhi2, Unigene18682\_JingChaoZuZhi2, Unigene18683\_JingChaoZuZhi2, Unigene18733\_JingChaoZuZhi2, Unigene18781\_JingChaoZuZhi2, Unigene18868\_JingChaoZuZhi2, Unigene19049\_JingChaoZuZhi2, Unigene19143\_JingChaoZuZhi2, Unigene19207\_JingChaoZuZhi2, Unigene19349\_JingChaoZuZhi2, Unigene19459\_JingChaoZuZhi2, Unigene19511\_JingChaoZuZhi2, Unigene19512\_JingChaoZuZhi2, Unigene19618\_JingChaoZuZhi2, Unigene19640\_JingChaoZuZhi2, Unigene19662\_JingChaoZuZhi2, Unigene19742\_JingChaoZuZhi2, Unigene19768\_JingChaoZuZhi2, Unigene19790\_JingChaoZuZhi2, Unigene19800\_JingChaoZuZhi2, Unigene19879\_JingChaoZuZhi2, Unigene19908\_JingChaoZuZhi2, Unigene19948\_JingChaoZuZhi2, Unigene19961\_JingChaoZuZhi2, Unigene19973\_JingChaoZuZhi2, Unigene20051\_JingChaoZuZhi2, Unigene20067\_JingChaoZuZhi2, Unigene20101\_JingChaoZuZhi2, Unigene20130\_JingChaoZuZhi2, Unigene20141\_JingChaoZuZhi2, Unigene20204\_JingChaoZuZhi2, Unigene20211\_JingChaoZuZhi2, Unigene20243\_JingChaoZuZhi2, Unigene20255\_JingChaoZuZhi2, Unigene20365\_JingChaoZuZhi2, Unigene20407\_JingChaoZuZhi2, Unigene20433\_JingChaoZuZhi2, Unigene20529\_JingChaoZuZhi2, Unigene20575\_JingChaoZuZhi2, Unigene20641\_JingChaoZuZhi2, Unigene20646\_JingChaoZuZhi2, Unigene20705\_JingChaoZuZhi2, Unigene20716\_JingChaoZuZhi2, Unigene20768\_JingChaoZuZhi2, Unigene20773\_JingChaoZuZhi2, Unigene20796\_JingChaoZuZhi2, Unigene20805\_JingChaoZuZhi2, Unigene20843\_JingChaoZuZhi2, Unigene20892\_JingChaoZuZhi2, Unigene20913\_JingChaoZuZhi2, Unigene20921\_JingChaoZuZhi2, Unigene20937\_JingChaoZuZhi2, Unigene20942\_JingChaoZuZhi2, Unigene20973\_JingChaoZuZhi2, Unigene21016\_JingChaoZuZhi2, Unigene21023\_JingChaoZuZhi2, Unigene21108\_JingChaoZuZhi2, Unigene21118\_JingChaoZuZhi2, Unigene21137\_JingChaoZuZhi2, Unigene21175\_JingChaoZuZhi2, Unigene21233\_JingChaoZuZhi2, Unigene21295\_JingChaoZuZhi2, Unigene21399\_JingChaoZuZhi2, Unigene21401\_JingChaoZuZhi2, Unigene21443\_JingChaoZuZhi2, Unigene21460\_JingChaoZuZhi2, Unigene21479\_JingChaoZuZhi2, Unigene21482\_JingChaoZuZhi2, Unigene21524\_JingChaoZuZhi2, Unigene21531\_JingChaoZuZhi2, Unigene21550\_JingChaoZuZhi2, Unigene21556\_JingChaoZuZhi2, Unigene21578\_JingChaoZuZhi2, Unigene21602\_JingChaoZuZhi2, Unigene21609\_JingChaoZuZhi2, Unigene21623\_JingChaoZuZhi2, Unigene21658\_JingChaoZuZhi2, Unigene21723\_JingChaoZuZhi2, Unigene21724\_JingChaoZuZhi2, Unigene2173\_JingChaoZuZhi2, Unigene21780\_JingChaoZuZhi2, Unigene21815\_JingChaoZuZhi2, Unigene21818\_JingChaoZuZhi2, Unigene2184\_JingChaoZuZhi2, Unigene21863\_JingChaoZuZhi2, Unigene21963\_JingChaoZuZhi2, Unigene21993\_JingChaoZuZhi2, Unigene22057\_JingChaoZuZhi2, Unigene22071\_JingChaoZuZhi2, Unigene22073\_JingChaoZuZhi2, Unigene22075\_JingChaoZuZhi2, Unigene22193\_JingChaoZuZhi2, Unigene22201\_JingChaoZuZhi2, Unigene22221\_JingChaoZuZhi2, Unigene2223\_JingChaoZuZhi2, Unigene22277\_JingChaoZuZhi2, Unigene22278\_JingChaoZuZhi2, Unigene22305\_JingChaoZuZhi2, Unigene22310\_JingChaoZuZhi2, Unigene22313\_JingChaoZuZhi2, Unigene22396\_JingChaoZuZhi2, Unigene22399\_JingChaoZuZhi2, Unigene22418\_JingChaoZuZhi2, Unigene22419\_JingChaoZuZhi2, Unigene22420\_JingChaoZuZhi2, Unigene22426\_JingChaoZuZhi2, Unigene22430\_JingChaoZuZhi2, Unigene22442\_JingChaoZuZhi2, Unigene22455\_JingChaoZuZhi2, Unigene22491\_JingChaoZuZhi2, Unigene2250\_JingChaoZuZhi2, Unigene22530\_JingChaoZuZhi2, Unigene22538\_JingChaoZuZhi2, Unigene22556\_JingChaoZuZhi2, Unigene22574\_JingChaoZuZhi2, Unigene22594\_JingChaoZuZhi2, Unigene22637\_JingChaoZuZhi2, Unigene22640\_JingChaoZuZhi2, Unigene22643\_JingChaoZuZhi2, Unigene22647\_JingChaoZuZhi2, Unigene22686\_JingChaoZuZhi2, Unigene22743\_JingChaoZuZhi2, Unigene22770\_JingChaoZuZhi2, Unigene22779\_JingChaoZuZhi2, Unigene22794\_JingChaoZuZhi2, Unigene22828\_JingChaoZuZhi2, Unigene22839\_JingChaoZuZhi2, Unigene22851\_JingChaoZuZhi2, Unigene22852\_JingChaoZuZhi2, Unigene22857\_JingChaoZuZhi2, Unigene22863\_JingChaoZuZhi2, Unigene22865\_JingChaoZuZhi2, Unigene22880\_JingChaoZuZhi2, Unigene22886\_JingChaoZuZhi2, Unigene22901\_JingChaoZuZhi2, Unigene22908\_JingChaoZuZhi2, Unigene22914\_JingChaoZuZhi2, Unigene22920\_JingChaoZuZhi2, Unigene22922\_JingChaoZuZhi2, Unigene22925\_JingChaoZuZhi2, Unigene22928\_JingChaoZuZhi2, Unigene22931\_JingChaoZuZhi2, Unigene229\_JingChaoZuZhi2, Unigene2324\_JingChaoZuZhi2, Unigene2342\_JingChaoZuZhi2, Unigene2345\_JingChaoZuZhi2, Unigene25014\_JingChaoZuZhi2, Unigene2517\_JingChaoZuZhi2, Unigene2519\_JingChaoZuZhi2, Unigene25228\_JingChaoZuZhi2, Unigene2525\_JingChaoZuZhi2, Unigene25310\_JingChaoZuZhi2, Unigene26730\_JingChaoZuZhi2, Unigene26782\_JingChaoZuZhi2, Unigene26987\_JingChaoZuZhi2, Unigene2721\_JingChaoZuZhi2, Unigene27\_JingChaoZuZhi2, Unigene2960\_JingChaoZuZhi2, Unigene31090\_JingChaoZuZhi2, Unigene3122\_JingChaoZuZhi2, Unigene3176\_JingChaoZuZhi2, Unigene31918\_JingChaoZuZhi2, Unigene324\_JingChaoZuZhi2, Unigene3286\_JingChaoZuZhi2, Unigene3300\_JingChaoZuZhi2, Unigene33026\_JingChaoZuZhi2, Unigene3324\_JingChaoZuZhi2, Unigene332\_JingChaoZuZhi2, Unigene3362\_JingChaoZuZhi2, Unigene34429\_JingChaoZuZhi2, Unigene34589\_JingChaoZuZhi2, Unigene3469\_JingChaoZuZhi2, Unigene35016\_JingChaoZuZhi2, Unigene36376\_JingChaoZuZhi2, Unigene36381\_JingChaoZuZhi2, Unigene36859\_JingChaoZuZhi2, Unigene3799\_JingChaoZuZhi2, Unigene3872\_JingChaoZuZhi2, Unigene38924\_JingChaoZuZhi2, Unigene39102\_JingChaoZuZhi2, Unigene39325\_JingChaoZuZhi2, Unigene39832\_JingChaoZuZhi2, Unigene39892\_JingChaoZuZhi2, Unigene3994\_JingChaoZuZhi2, Unigene40335\_JingChaoZuZhi2, Unigene40919\_JingChaoZuZhi2, Unigene41089\_JingChaoZuZhi2, Unigene4112\_JingChaoZuZhi2, Unigene4174\_JingChaoZuZhi2, Unigene42248\_JingChaoZuZhi2, Unigene4339\_JingChaoZuZhi2, Unigene43889\_JingChaoZuZhi2, Unigene44190\_JingChaoZuZhi2, Unigene4474\_JingChaoZuZhi2, Unigene4529\_JingChaoZuZhi2, Unigene45468\_JingChaoZuZhi2, Unigene4618\_JingChaoZuZhi2, Unigene46581\_JingChaoZuZhi2, Unigene47543\_JingChaoZuZhi2, Unigene48408\_JingChaoZuZhi2, Unigene49253\_JingChaoZuZhi2, Unigene4975\_JingChaoZuZhi2, Unigene5035\_JingChaoZuZhi2, Unigene5073\_JingChaoZuZhi2, Unigene5124\_JingChaoZuZhi2, Unigene51467\_JingChaoZuZhi2, Unigene5146\_JingChaoZuZhi2, Unigene52122\_JingChaoZuZhi2, Unigene5349\_JingChaoZuZhi2, Unigene53935\_JingChaoZuZhi2, Unigene54012\_JingChaoZuZhi2, Unigene54777\_JingChaoZuZhi2, Unigene5529\_JingChaoZuZhi2, Unigene5534\_JingChaoZuZhi2, Unigene55368\_JingChaoZuZhi2, Unigene5554\_JingChaoZuZhi2, Unigene563\_JingChaoZuZhi2, Unigene56557\_JingChaoZuZhi2, Unigene57338\_JingChaoZuZhi2, Unigene5733\_JingChaoZuZhi2, Unigene5739\_JingChaoZuZhi2, Unigene57749\_JingChaoZuZhi2, Unigene5814\_JingChaoZuZhi2, Unigene5820\_JingChaoZuZhi2, Unigene5863\_JingChaoZuZhi2, Unigene58967\_JingChaoZuZhi2, Unigene589\_JingChaoZuZhi2, Unigene5903\_JingChaoZuZhi2, Unigene59101\_JingChaoZuZhi2, Unigene59146\_JingChaoZuZhi2, Unigene59375\_JingChaoZuZhi2, Unigene5959\_JingChaoZuZhi2, Unigene59604\_JingChaoZuZhi2, Unigene60026\_JingChaoZuZhi2, Unigene60650\_JingChaoZuZhi2, Unigene6074\_JingChaoZuZhi2, Unigene60988\_JingChaoZuZhi2, Unigene61043\_JingChaoZuZhi2, Unigene610\_JingChaoZuZhi2, Unigene6121\_JingChaoZuZhi2, Unigene61244\_JingChaoZuZhi2, Unigene61390\_JingChaoZuZhi2, Unigene61406\_JingChaoZuZhi2, Unigene61657\_JingChaoZuZhi2, Unigene61675\_JingChaoZuZhi2, Unigene61702\_JingChaoZuZhi2, Unigene6262\_JingChaoZuZhi2, Unigene62835\_JingChaoZuZhi2, Unigene63589\_JingChaoZuZhi2, Unigene63\_JingChaoZuZhi2, Unigene64164\_JingChaoZuZhi2, Unigene64529\_JingChaoZuZhi2, Unigene64626\_JingChaoZuZhi2, Unigene6468\_JingChaoZuZhi2, Unigene64830\_JingChaoZuZhi2, Unigene6495\_JingChaoZuZhi2, Unigene65431\_JingChaoZuZhi2, Unigene6563\_JingChaoZuZhi2, Unigene65663\_JingChaoZuZhi2, Unigene65862\_JingChaoZuZhi2, Unigene66105\_JingChaoZuZhi2, Unigene66160\_JingChaoZuZhi2, Unigene6625\_JingChaoZuZhi2, Unigene6634\_JingChaoZuZhi2, Unigene66535\_JingChaoZuZhi2, Unigene66757\_JingChaoZuZhi2, Unigene6735\_JingChaoZuZhi2, Unigene67655\_JingChaoZuZhi2, Unigene68867\_JingChaoZuZhi2, Unigene68906\_JingChaoZuZhi2, Unigene69062\_JingChaoZuZhi2, Unigene69100\_JingChaoZuZhi2, Unigene69445\_JingChaoZuZhi2, Unigene6979\_JingChaoZuZhi2, Unigene70156\_JingChaoZuZhi2, Unigene70308\_JingChaoZuZhi2, Unigene70319\_JingChaoZuZhi2, Unigene70353\_JingChaoZuZhi2, Unigene705\_JingChaoZuZhi2, Unigene7062\_JingChaoZuZhi2, Unigene70723\_JingChaoZuZhi2, Unigene7074\_JingChaoZuZhi2, Unigene7083\_JingChaoZuZhi2, Unigene71079\_JingChaoZuZhi2, Unigene71172\_JingChaoZuZhi2, Unigene71365\_JingChaoZuZhi2, Unigene7139\_JingChaoZuZhi2, Unigene71592\_JingChaoZuZhi2, Unigene7162\_JingChaoZuZhi2, Unigene71738\_JingChaoZuZhi2, Unigene71928\_JingChaoZuZhi2, Unigene72015\_JingChaoZuZhi2, Unigene72219\_JingChaoZuZhi2, Unigene7306\_JingChaoZuZhi2, Unigene73445\_JingChaoZuZhi2, Unigene73470\_JingChaoZuZhi2, Unigene73554\_JingChaoZuZhi2, Unigene7407\_JingChaoZuZhi2, Unigene74502\_JingChaoZuZhi2, Unigene7480\_JingChaoZuZhi2, Unigene75030\_JingChaoZuZhi2, Unigene75045\_JingChaoZuZhi2, Unigene75123\_JingChaoZuZhi2, Unigene751\_JingChaoZuZhi2, Unigene75244\_JingChaoZuZhi2, Unigene75422\_JingChaoZuZhi2, Unigene754\_JingChaoZuZhi2, Unigene75793\_JingChaoZuZhi2, Unigene75821\_JingChaoZuZhi2, Unigene75857\_JingChaoZuZhi2, Unigene76863\_JingChaoZuZhi2, Unigene77072\_JingChaoZuZhi2, Unigene77539\_JingChaoZuZhi2, Unigene77543\_JingChaoZuZhi2, Unigene77596\_JingChaoZuZhi2, Unigene77644\_JingChaoZuZhi2, Unigene77816\_JingChaoZuZhi2, Unigene77871\_JingChaoZuZhi2, Unigene78235\_JingChaoZuZhi2, Unigene78429\_JingChaoZuZhi2, Unigene78518\_JingChaoZuZhi2, Unigene78715\_JingChaoZuZhi2, Unigene787\_JingChaoZuZhi2, Unigene78950\_JingChaoZuZhi2, Unigene78987\_JingChaoZuZhi2, Unigene79203\_JingChaoZuZhi2, Unigene79529\_JingChaoZuZhi2, Unigene79560\_JingChaoZuZhi2, Unigene79660\_JingChaoZuZhi2, Unigene79778\_JingChaoZuZhi2, Unigene79810\_JingChaoZuZhi2, Unigene79902\_JingChaoZuZhi2, Unigene79987\_JingChaoZuZhi2, Unigene80069\_JingChaoZuZhi2, Unigene80120\_JingChaoZuZhi2, Unigene80193\_JingChaoZuZhi2, Unigene80225\_JingChaoZuZhi2, Unigene80247\_JingChaoZuZhi2, Unigene80312\_JingChaoZuZhi2, Unigene80412\_JingChaoZuZhi2, Unigene80475\_JingChaoZuZhi2, Unigene80608\_JingChaoZuZhi2, Unigene80653\_JingChaoZuZhi2, Unigene8094\_JingChaoZuZhi2, Unigene81148\_JingChaoZuZhi2, Unigene81225\_JingChaoZuZhi2, Unigene81393\_JingChaoZuZhi2, Unigene81599\_JingChaoZuZhi2, Unigene81601\_JingChaoZuZhi2, Unigene81700\_JingChaoZuZhi2, Unigene81793\_JingChaoZuZhi2, Unigene81964\_JingChaoZuZhi2, Unigene82040\_JingChaoZuZhi2, Unigene82117\_JingChaoZuZhi2, Unigene82120\_JingChaoZuZhi2, Unigene82489\_JingChaoZuZhi2, Unigene82734\_JingChaoZuZhi2, Unigene8288\_JingChaoZuZhi2, Unigene82918\_JingChaoZuZhi2, Unigene82\_JingChaoZuZhi2, Unigene83094\_JingChaoZuZhi2, Unigene83252\_JingChaoZuZhi2, Unigene83276\_JingChaoZuZhi2, Unigene83303\_JingChaoZuZhi2, Unigene8338\_JingChaoZuZhi2, Unigene83846\_JingChaoZuZhi2, Unigene83872\_JingChaoZuZhi2, Unigene83935\_JingChaoZuZhi2, Unigene83973\_JingChaoZuZhi2, Unigene84190\_JingChaoZuZhi2, Unigene84252\_JingChaoZuZhi2, Unigene84270\_JingChaoZuZhi2, Unigene84352\_JingChaoZuZhi2, Unigene843\_JingChaoZuZhi2, Unigene84658\_JingChaoZuZhi2, Unigene84671\_JingChaoZuZhi2, Unigene84829\_JingChaoZuZhi2, Unigene84880\_JingChaoZuZhi2, Unigene84883\_JingChaoZuZhi2, Unigene84966\_JingChaoZuZhi2, Unigene85055\_JingChaoZuZhi2, Unigene85069\_JingChaoZuZhi2, Unigene85099\_JingChaoZuZhi2, Unigene8510\_JingChaoZuZhi2, Unigene85193\_JingChaoZuZhi2, Unigene8520\_JingChaoZuZhi2, Unigene85215\_JingChaoZuZhi2, Unigene85335\_JingChaoZuZhi2, Unigene85375\_JingChaoZuZhi2, Unigene85405\_JingChaoZuZhi2, Unigene85490\_JingChaoZuZhi2, Unigene85503\_JingChaoZuZhi2, Unigene85504\_JingChaoZuZhi2, Unigene85520\_JingChaoZuZhi2, Unigene85743\_JingChaoZuZhi2, Unigene85781\_JingChaoZuZhi2, Unigene85789\_JingChaoZuZhi2, Unigene8607\_JingChaoZuZhi2, Unigene86107\_JingChaoZuZhi2, Unigene86131\_JingChaoZuZhi2, Unigene86243\_JingChaoZuZhi2, Unigene86271\_JingChaoZuZhi2, Unigene86362\_JingChaoZuZhi2, Unigene86504\_JingChaoZuZhi2, Unigene86630\_JingChaoZuZhi2, Unigene86941\_JingChaoZuZhi2, Unigene86975\_JingChaoZuZhi2, Unigene8710\_JingChaoZuZhi2, Unigene87160\_JingChaoZuZhi2, Unigene87169\_JingChaoZuZhi2, Unigene87203\_JingChaoZuZhi2, Unigene87228\_JingChaoZuZhi2, Unigene87246\_JingChaoZuZhi2, Unigene87332\_JingChaoZuZhi2, Unigene87347\_JingChaoZuZhi2, Unigene87400\_JingChaoZuZhi2, Unigene87474\_JingChaoZuZhi2, Unigene87587\_JingChaoZuZhi2, Unigene87653\_JingChaoZuZhi2, Unigene87741\_JingChaoZuZhi2, Unigene87903\_JingChaoZuZhi2, Unigene87917\_JingChaoZuZhi2, Unigene87946\_JingChaoZuZhi2, Unigene87963\_JingChaoZuZhi2, Unigene88059\_JingChaoZuZhi2, Unigene88087\_JingChaoZuZhi2, Unigene88089\_JingChaoZuZhi2, Unigene88115\_JingChaoZuZhi2, Unigene88203\_JingChaoZuZhi2, Unigene88235\_JingChaoZuZhi2, Unigene88262\_JingChaoZuZhi2, Unigene88283\_JingChaoZuZhi2, Unigene88342\_JingChaoZuZhi2, Unigene8839\_JingChaoZuZhi2, Unigene88546\_JingChaoZuZhi2, Unigene88618\_JingChaoZuZhi2, Unigene88661\_JingChaoZuZhi2, Unigene88672\_JingChaoZuZhi2, Unigene88761\_JingChaoZuZhi2, Unigene88774\_JingChaoZuZhi2, Unigene88806\_JingChaoZuZhi2, Unigene88886\_JingChaoZuZhi2, Unigene89057\_JingChaoZuZhi2, Unigene89060\_JingChaoZuZhi2, Unigene89087\_JingChaoZuZhi2, Unigene89094\_JingChaoZuZhi2, Unigene89127\_JingChaoZuZhi2, Unigene89128\_JingChaoZuZhi2, Unigene89192\_JingChaoZuZhi2, Unigene89251\_JingChaoZuZhi2, Unigene89305\_JingChaoZuZhi2, Unigene89321\_JingChaoZuZhi2, Unigene89354\_JingChaoZuZhi2, Unigene89413\_JingChaoZuZhi2, Unigene89572\_JingChaoZuZhi2, Unigene89595\_JingChaoZuZhi2, Unigene89597\_JingChaoZuZhi2, Unigene89628\_JingChaoZuZhi2, Unigene896\_JingChaoZuZhi2, Unigene89741\_JingChaoZuZhi2, Unigene89946\_JingChaoZuZhi2, Unigene8996\_JingChaoZuZhi2, Unigene89973\_JingChaoZuZhi2, Unigene90040\_JingChaoZuZhi2, Unigene90082\_JingChaoZuZhi2, Unigene90084\_JingChaoZuZhi2, Unigene90173\_JingChaoZuZhi2, Unigene90284\_JingChaoZuZhi2, Unigene9028\_JingChaoZuZhi2, Unigene90442\_JingChaoZuZhi2, Unigene90494\_JingChaoZuZhi2, Unigene9052\_JingChaoZuZhi2, Unigene9053\_JingChaoZuZhi2, Unigene90600\_JingChaoZuZhi2, Unigene90609\_JingChaoZuZhi2, Unigene90724\_JingChaoZuZhi2, Unigene90742\_JingChaoZuZhi2, Unigene90828\_JingChaoZuZhi2, Unigene90830\_JingChaoZuZhi2, Unigene90832\_JingChaoZuZhi2, Unigene90889\_JingChaoZuZhi2, Unigene90892\_JingChaoZuZhi2, Unigene90933\_JingChaoZuZhi2, Unigene90955\_JingChaoZuZhi2, Unigene91138\_JingChaoZuZhi2, Unigene91163\_JingChaoZuZhi2, Unigene91252\_JingChaoZuZhi2, Unigene91307\_JingChaoZuZhi2, Unigene9132\_JingChaoZuZhi2, Unigene91350\_JingChaoZuZhi2, Unigene91359\_JingChaoZuZhi2, Unigene91446\_JingChaoZuZhi2, Unigene91490\_JingChaoZuZhi2, Unigene91527\_JingChaoZuZhi2, Unigene91529\_JingChaoZuZhi2, Unigene91534\_JingChaoZuZhi2, Unigene91584\_JingChaoZuZhi2, Unigene91592\_JingChaoZuZhi2, Unigene91685\_JingChaoZuZhi2, Unigene91686\_JingChaoZuZhi2, Unigene91710\_JingChaoZuZhi2, Unigene91733\_JingChaoZuZhi2, Unigene91952\_JingChaoZuZhi2, Unigene92015\_JingChaoZuZhi2, Unigene92046\_JingChaoZuZhi2, Unigene92084\_JingChaoZuZhi2, Unigene92101\_JingChaoZuZhi2, Unigene92139\_JingChaoZuZhi2, Unigene92170\_JingChaoZuZhi2, Unigene9220\_JingChaoZuZhi2, Unigene92227\_JingChaoZuZhi2, Unigene92245\_JingChaoZuZhi2, Unigene92271\_JingChaoZuZhi2, Unigene92379\_JingChaoZuZhi2, Unigene92417\_JingChaoZuZhi2, Unigene92452\_JingChaoZuZhi2, Unigene92529\_JingChaoZuZhi2, Unigene92536\_JingChaoZuZhi2, Unigene92548\_JingChaoZuZhi2, Unigene92566\_JingChaoZuZhi2, Unigene92596\_JingChaoZuZhi2, Unigene92607\_JingChaoZuZhi2, Unigene92627\_JingChaoZuZhi2, Unigene92631\_JingChaoZuZhi2, Unigene92749\_JingChaoZuZhi2, Unigene92901\_JingChaoZuZhi2, Unigene92905\_JingChaoZuZhi2, Unigene92912\_JingChaoZuZhi2, Unigene92920\_JingChaoZuZhi2, Unigene92962\_JingChaoZuZhi2, Unigene93016\_JingChaoZuZhi2, Unigene93025\_JingChaoZuZhi2, Unigene93032\_JingChaoZuZhi2, Unigene93047\_JingChaoZuZhi2, Unigene93070\_JingChaoZuZhi2, Unigene93081\_JingChaoZuZhi2, Unigene93093\_JingChaoZuZhi2, Unigene93111\_JingChaoZuZhi2, Unigene93115\_JingChaoZuZhi2, Unigene93229\_JingChaoZuZhi2, Unigene93238\_JingChaoZuZhi2, Unigene93270\_JingChaoZuZhi2, Unigene93333\_JingChaoZuZhi2, Unigene93343\_JingChaoZuZhi2, Unigene93362\_JingChaoZuZhi2, Unigene93412\_JingChaoZuZhi2, Unigene93444\_JingChaoZuZhi2, Unigene93470\_JingChaoZuZhi2, Unigene93492\_JingChaoZuZhi2, Unigene93516\_JingChaoZuZhi2, Unigene93520\_JingChaoZuZhi2, Unigene93583\_JingChaoZuZhi2, Unigene93594\_JingChaoZuZhi2, Unigene93646\_JingChaoZuZhi2, Unigene93717\_JingChaoZuZhi2, Unigene93734\_JingChaoZuZhi2, Unigene93746\_JingChaoZuZhi2, Unigene93765\_JingChaoZuZhi2, Unigene93785\_JingChaoZuZhi2, Unigene93798\_JingChaoZuZhi2, Unigene9380\_JingChaoZuZhi2, Unigene93837\_JingChaoZuZhi2, Unigene93874\_JingChaoZuZhi2, Unigene93902\_JingChaoZuZhi2, Unigene93930\_JingChaoZuZhi2, Unigene93931\_JingChaoZuZhi2, Unigene93936\_JingChaoZuZhi2, Unigene93955\_JingChaoZuZhi2, Unigene94005\_JingChaoZuZhi2, Unigene94011\_JingChaoZuZhi2, Unigene94057\_JingChaoZuZhi2, Unigene94058\_JingChaoZuZhi2, Unigene94069\_JingChaoZuZhi2, Unigene94103\_JingChaoZuZhi2, Unigene94107\_JingChaoZuZhi2, Unigene94110\_JingChaoZuZhi2, Unigene94149\_JingChaoZuZhi2, Unigene94214\_JingChaoZuZhi2, Unigene94266\_JingChaoZuZhi2, Unigene94305\_JingChaoZuZhi2, Unigene94311\_JingChaoZuZhi2, Unigene94313\_JingChaoZuZhi2, Unigene94320\_JingChaoZuZhi2, Unigene94364\_JingChaoZuZhi2, Unigene94376\_JingChaoZuZhi2, Unigene94384\_JingChaoZuZhi2, Unigene94386\_JingChaoZuZhi2, Unigene94445\_JingChaoZuZhi2, Unigene9445\_JingChaoZuZhi2, Unigene94484\_JingChaoZuZhi2, Unigene94488\_JingChaoZuZhi2, Unigene94509\_JingChaoZuZhi2, Unigene94518\_JingChaoZuZhi2, Unigene94526\_JingChaoZuZhi2, Unigene94540\_JingChaoZuZhi2, Unigene94552\_JingChaoZuZhi2, Unigene94578\_JingChaoZuZhi2, Unigene94586\_JingChaoZuZhi2, Unigene94616\_JingChaoZuZhi2, Unigene9461\_JingChaoZuZhi2, Unigene94664\_JingChaoZuZhi2, Unigene94674\_JingChaoZuZhi2, Unigene94690\_JingChaoZuZhi2, Unigene94721\_JingChaoZuZhi2, Unigene94725\_JingChaoZuZhi2, Unigene94775\_JingChaoZuZhi2, Unigene94792\_JingChaoZuZhi2, Unigene94810\_JingChaoZuZhi2, Unigene94841\_JingChaoZuZhi2, Unigene94842\_JingChaoZuZhi2, Unigene94851\_JingChaoZuZhi2, Unigene9490\_JingChaoZuZhi2, Unigene94975\_JingChaoZuZhi2, Unigene94989\_JingChaoZuZhi2, Unigene94999\_JingChaoZuZhi2, Unigene95035\_JingChaoZuZhi2, Unigene95089\_JingChaoZuZhi2, Unigene95130\_JingChaoZuZhi2, Unigene95140\_JingChaoZuZhi2, Unigene95178\_JingChaoZuZhi2, Unigene95181\_JingChaoZuZhi2, Unigene951\_JingChaoZuZhi2, Unigene95283\_JingChaoZuZhi2, Unigene95290\_JingChaoZuZhi2, Unigene95331\_JingChaoZuZhi2, Unigene95336\_JingChaoZuZhi2, Unigene95390\_JingChaoZuZhi2, Unigene95391\_JingChaoZuZhi2, Unigene95415\_JingChaoZuZhi2, Unigene95427\_JingChaoZuZhi2, Unigene95485\_JingChaoZuZhi2, Unigene95489\_JingChaoZuZhi2, Unigene95496\_JingChaoZuZhi2, Unigene95510\_JingChaoZuZhi2, Unigene95538\_JingChaoZuZhi2, Unigene95633\_JingChaoZuZhi2, Unigene95647\_JingChaoZuZhi2, Unigene95654\_JingChaoZuZhi2, Unigene95661\_JingChaoZuZhi2, Unigene95677\_JingChaoZuZhi2, Unigene95690\_JingChaoZuZhi2, Unigene95701\_JingChaoZuZhi2, Unigene95710\_JingChaoZuZhi2, Unigene95758\_JingChaoZuZhi2, Unigene95761\_JingChaoZuZhi2, Unigene95762\_JingChaoZuZhi2, Unigene95770\_JingChaoZuZhi2, Unigene95789\_JingChaoZuZhi2, Unigene95809\_JingChaoZuZhi2, Unigene95849\_JingChaoZuZhi2, Unigene95851\_JingChaoZuZhi2, Unigene95860\_JingChaoZuZhi2, Unigene95864\_JingChaoZuZhi2, Unigene95880\_JingChaoZuZhi2, Unigene95897\_JingChaoZuZhi2, Unigene95907\_JingChaoZuZhi2, Unigene95910\_JingChaoZuZhi2, Unigene95916\_JingChaoZuZhi2, Unigene95948\_JingChaoZuZhi2, Unigene95973\_JingChaoZuZhi2, Unigene95977\_JingChaoZuZhi2, Unigene96002\_JingChaoZuZhi2, Unigene96023\_JingChaoZuZhi2, Unigene96025\_JingChaoZuZhi2, Unigene96030\_JingChaoZuZhi2, Unigene96045\_JingChaoZuZhi2, Unigene96063\_JingChaoZuZhi2, Unigene96069\_JingChaoZuZhi2, Unigene96102\_JingChaoZuZhi2, Unigene96104\_JingChaoZuZhi2, Unigene96115\_JingChaoZuZhi2, Unigene9616\_JingChaoZuZhi2, Unigene96171\_JingChaoZuZhi2, Unigene96177\_JingChaoZuZhi2, Unigene96182\_JingChaoZuZhi2, Unigene96204\_JingChaoZuZhi2, Unigene96219\_JingChaoZuZhi2, Unigene96228\_JingChaoZuZhi2, Unigene96229\_JingChaoZuZhi2, Unigene96237\_JingChaoZuZhi2, Unigene96242\_JingChaoZuZhi2, Unigene96245\_JingChaoZuZhi2, Unigene96246\_JingChaoZuZhi2, Unigene96252\_JingChaoZuZhi2, Unigene9636\_JingChaoZuZhi2, Unigene9674\_JingChaoZuZhi2, Unigene9675\_JingChaoZuZhi2, Unigene9739\_JingChaoZuZhi2, Unigene9871\_JingChaoZuZhi2, Unigene98\_JingChaoZuZhi2, Unigene9978\_JingChaoZuZhi2 |
| 7 | Phototransduction | Unigene10017\_JingChaoZuZhi2, Unigene10084\_JingChaoZuZhi2, Unigene1008\_JingChaoZuZhi2, Unigene10147\_JingChaoZuZhi2, Unigene1022\_JingChaoZuZhi2, Unigene1033\_JingChaoZuZhi2, Unigene10347\_JingChaoZuZhi2, Unigene10558\_JingChaoZuZhi2, Unigene1065\_JingChaoZuZhi2, Unigene10747\_JingChaoZuZhi2, Unigene10843\_JingChaoZuZhi2, Unigene10884\_JingChaoZuZhi2, Unigene1089\_JingChaoZuZhi2, Unigene11001\_JingChaoZuZhi2, Unigene11002\_JingChaoZuZhi2, Unigene11135\_JingChaoZuZhi2, Unigene11138\_JingChaoZuZhi2, Unigene11338\_JingChaoZuZhi2, Unigene11394\_JingChaoZuZhi2, Unigene11499\_JingChaoZuZhi2, Unigene11660\_JingChaoZuZhi2, Unigene11745\_JingChaoZuZhi2, Unigene11758\_JingChaoZuZhi2, Unigene11762\_JingChaoZuZhi2, Unigene11766\_JingChaoZuZhi2, Unigene11869\_JingChaoZuZhi2, Unigene1196\_JingChaoZuZhi2, Unigene12053\_JingChaoZuZhi2, Unigene12068\_JingChaoZuZhi2, Unigene12115\_JingChaoZuZhi2, Unigene12370\_JingChaoZuZhi2, Unigene12381\_JingChaoZuZhi2, Unigene1239\_JingChaoZuZhi2, Unigene12430\_JingChaoZuZhi2, Unigene1247\_JingChaoZuZhi2, Unigene12495\_JingChaoZuZhi2, Unigene12519\_JingChaoZuZhi2, Unigene12622\_JingChaoZuZhi2, Unigene12694\_JingChaoZuZhi2, Unigene1273\_JingChaoZuZhi2, Unigene12757\_JingChaoZuZhi2, Unigene12787\_JingChaoZuZhi2, Unigene12898\_JingChaoZuZhi2, Unigene13224\_JingChaoZuZhi2, Unigene13241\_JingChaoZuZhi2, Unigene13294\_JingChaoZuZhi2, Unigene13379\_JingChaoZuZhi2, Unigene13529\_JingChaoZuZhi2, Unigene13561\_JingChaoZuZhi2, Unigene13573\_JingChaoZuZhi2, Unigene13684\_JingChaoZuZhi2, Unigene1368\_JingChaoZuZhi2, Unigene1373\_JingChaoZuZhi2, Unigene13798\_JingChaoZuZhi2, Unigene13815\_JingChaoZuZhi2, Unigene13859\_JingChaoZuZhi2, Unigene1385\_JingChaoZuZhi2, Unigene13919\_JingChaoZuZhi2, Unigene14053\_JingChaoZuZhi2, Unigene1417\_JingChaoZuZhi2, Unigene142\_JingChaoZuZhi2, Unigene14335\_JingChaoZuZhi2, Unigene14387\_JingChaoZuZhi2, Unigene1440\_JingChaoZuZhi2, Unigene14461\_JingChaoZuZhi2, Unigene1447\_JingChaoZuZhi2, Unigene14507\_JingChaoZuZhi2, Unigene14531\_JingChaoZuZhi2, Unigene1475\_JingChaoZuZhi2, Unigene1480\_JingChaoZuZhi2, Unigene14900\_JingChaoZuZhi2, Unigene14966\_JingChaoZuZhi2, Unigene14994\_JingChaoZuZhi2, Unigene15121\_JingChaoZuZhi2, Unigene15163\_JingChaoZuZhi2, Unigene15196\_JingChaoZuZhi2, Unigene15226\_JingChaoZuZhi2, Unigene15374\_JingChaoZuZhi2, Unigene15525\_JingChaoZuZhi2, Unigene15621\_JingChaoZuZhi2, Unigene15744\_JingChaoZuZhi2, Unigene15782\_JingChaoZuZhi2, Unigene1579\_JingChaoZuZhi2, Unigene15832\_JingChaoZuZhi2, Unigene15842\_JingChaoZuZhi2, Unigene15928\_JingChaoZuZhi2, Unigene15932\_JingChaoZuZhi2, Unigene15933\_JingChaoZuZhi2, Unigene15935\_JingChaoZuZhi2, Unigene16041\_JingChaoZuZhi2, Unigene1605\_JingChaoZuZhi2, Unigene16068\_JingChaoZuZhi2, Unigene16205\_JingChaoZuZhi2, Unigene16237\_JingChaoZuZhi2, Unigene16257\_JingChaoZuZhi2, Unigene16274\_JingChaoZuZhi2, Unigene16315\_JingChaoZuZhi2, Unigene16339\_JingChaoZuZhi2, Unigene16454\_JingChaoZuZhi2, Unigene16497\_JingChaoZuZhi2, Unigene1654\_JingChaoZuZhi2, Unigene16566\_JingChaoZuZhi2, Unigene16637\_JingChaoZuZhi2, Unigene16672\_JingChaoZuZhi2, Unigene16770\_JingChaoZuZhi2, Unigene16803\_JingChaoZuZhi2, Unigene16810\_JingChaoZuZhi2, Unigene16857\_JingChaoZuZhi2, Unigene16971\_JingChaoZuZhi2, Unigene17167\_JingChaoZuZhi2, Unigene17200\_JingChaoZuZhi2, Unigene17232\_JingChaoZuZhi2, Unigene1740\_JingChaoZuZhi2, Unigene1742\_JingChaoZuZhi2, Unigene17509\_JingChaoZuZhi2, Unigene17557\_JingChaoZuZhi2, Unigene17577\_JingChaoZuZhi2, Unigene17713\_JingChaoZuZhi2, Unigene17850\_JingChaoZuZhi2, Unigene18054\_JingChaoZuZhi2, Unigene18069\_JingChaoZuZhi2, Unigene18207\_JingChaoZuZhi2, Unigene18217\_JingChaoZuZhi2, Unigene18224\_JingChaoZuZhi2, Unigene18301\_JingChaoZuZhi2, Unigene18697\_JingChaoZuZhi2, Unigene1874\_JingChaoZuZhi2, Unigene18901\_JingChaoZuZhi2, Unigene18911\_JingChaoZuZhi2, Unigene18922\_JingChaoZuZhi2, Unigene1892\_JingChaoZuZhi2, Unigene18961\_JingChaoZuZhi2, Unigene19166\_JingChaoZuZhi2, Unigene19322\_JingChaoZuZhi2, Unigene19421\_JingChaoZuZhi2, Unigene19713\_JingChaoZuZhi2, Unigene19746\_JingChaoZuZhi2, Unigene19757\_JingChaoZuZhi2, Unigene1975\_JingChaoZuZhi2, Unigene1977\_JingChaoZuZhi2, Unigene1991\_JingChaoZuZhi2, Unigene19927\_JingChaoZuZhi2, Unigene20003\_JingChaoZuZhi2, Unigene20015\_JingChaoZuZhi2, Unigene20029\_JingChaoZuZhi2, Unigene2003\_JingChaoZuZhi2, Unigene20056\_JingChaoZuZhi2, Unigene20136\_JingChaoZuZhi2, Unigene20267\_JingChaoZuZhi2, Unigene20353\_JingChaoZuZhi2, Unigene20480\_JingChaoZuZhi2, Unigene20778\_JingChaoZuZhi2, Unigene20860\_JingChaoZuZhi2, Unigene2086\_JingChaoZuZhi2, Unigene20900\_JingChaoZuZhi2, Unigene21056\_JingChaoZuZhi2, Unigene21197\_JingChaoZuZhi2, Unigene2160\_JingChaoZuZhi2, Unigene21646\_JingChaoZuZhi2, Unigene21799\_JingChaoZuZhi2, Unigene21802\_JingChaoZuZhi2, Unigene21805\_JingChaoZuZhi2, Unigene21913\_JingChaoZuZhi2, Unigene21925\_JingChaoZuZhi2, Unigene21927\_JingChaoZuZhi2, Unigene21945\_JingChaoZuZhi2, Unigene21981\_JingChaoZuZhi2, Unigene22037\_JingChaoZuZhi2, Unigene22051\_JingChaoZuZhi2, Unigene22059\_JingChaoZuZhi2, Unigene22162\_JingChaoZuZhi2, Unigene22219\_JingChaoZuZhi2, Unigene22268\_JingChaoZuZhi2, Unigene22389\_JingChaoZuZhi2, Unigene22450\_JingChaoZuZhi2, Unigene22452\_JingChaoZuZhi2, Unigene22468\_JingChaoZuZhi2, Unigene2246\_JingChaoZuZhi2, Unigene22671\_JingChaoZuZhi2, Unigene22698\_JingChaoZuZhi2, Unigene22757\_JingChaoZuZhi2, Unigene2331\_JingChaoZuZhi2, Unigene23339\_JingChaoZuZhi2, Unigene23772\_JingChaoZuZhi2, Unigene23885\_JingChaoZuZhi2, Unigene24820\_JingChaoZuZhi2, Unigene25338\_JingChaoZuZhi2, Unigene25554\_JingChaoZuZhi2, Unigene25688\_JingChaoZuZhi2, Unigene25817\_JingChaoZuZhi2, Unigene26054\_JingChaoZuZhi2, Unigene26389\_JingChaoZuZhi2, Unigene26407\_JingChaoZuZhi2, Unigene26700\_JingChaoZuZhi2, Unigene26701\_JingChaoZuZhi2, Unigene2675\_JingChaoZuZhi2, Unigene26878\_JingChaoZuZhi2, Unigene26911\_JingChaoZuZhi2, Unigene26919\_JingChaoZuZhi2, Unigene2701\_JingChaoZuZhi2, Unigene27045\_JingChaoZuZhi2, Unigene27141\_JingChaoZuZhi2, Unigene27512\_JingChaoZuZhi2, Unigene27772\_JingChaoZuZhi2, Unigene28336\_JingChaoZuZhi2, Unigene28427\_JingChaoZuZhi2, Unigene28517\_JingChaoZuZhi2, Unigene2903\_JingChaoZuZhi2, Unigene29084\_JingChaoZuZhi2, Unigene29091\_JingChaoZuZhi2, Unigene29133\_JingChaoZuZhi2, Unigene30193\_JingChaoZuZhi2, Unigene30207\_JingChaoZuZhi2, Unigene30409\_JingChaoZuZhi2, Unigene30624\_JingChaoZuZhi2, Unigene30738\_JingChaoZuZhi2, Unigene30762\_JingChaoZuZhi2, Unigene31186\_JingChaoZuZhi2, Unigene31320\_JingChaoZuZhi2, Unigene31406\_JingChaoZuZhi2, Unigene31790\_JingChaoZuZhi2, Unigene31857\_JingChaoZuZhi2, Unigene31886\_JingChaoZuZhi2, Unigene31910\_JingChaoZuZhi2, Unigene3234\_JingChaoZuZhi2, Unigene32937\_JingChaoZuZhi2, Unigene33069\_JingChaoZuZhi2, Unigene33135\_JingChaoZuZhi2, Unigene3329\_JingChaoZuZhi2, Unigene33302\_JingChaoZuZhi2, Unigene3330\_JingChaoZuZhi2, Unigene33373\_JingChaoZuZhi2, Unigene33556\_JingChaoZuZhi2, Unigene33683\_JingChaoZuZhi2, Unigene33839\_JingChaoZuZhi2, Unigene34463\_JingChaoZuZhi2, Unigene3464\_JingChaoZuZhi2, Unigene34711\_JingChaoZuZhi2, Unigene3478\_JingChaoZuZhi2, Unigene34802\_JingChaoZuZhi2, Unigene34892\_JingChaoZuZhi2, Unigene34894\_JingChaoZuZhi2, Unigene34988\_JingChaoZuZhi2, Unigene35364\_JingChaoZuZhi2, Unigene35418\_JingChaoZuZhi2, Unigene3588\_JingChaoZuZhi2, Unigene35977\_JingChaoZuZhi2, Unigene3613\_JingChaoZuZhi2, Unigene36382\_JingChaoZuZhi2, Unigene36411\_JingChaoZuZhi2, Unigene36559\_JingChaoZuZhi2, Unigene36684\_JingChaoZuZhi2, Unigene36760\_JingChaoZuZhi2, Unigene3688\_JingChaoZuZhi2, Unigene36901\_JingChaoZuZhi2, Unigene36999\_JingChaoZuZhi2, Unigene37009\_JingChaoZuZhi2, Unigene37051\_JingChaoZuZhi2, Unigene37082\_JingChaoZuZhi2, Unigene37084\_JingChaoZuZhi2, Unigene37297\_JingChaoZuZhi2, Unigene37595\_JingChaoZuZhi2, Unigene3774\_JingChaoZuZhi2, Unigene37817\_JingChaoZuZhi2, Unigene37880\_JingChaoZuZhi2, Unigene38036\_JingChaoZuZhi2, Unigene38214\_JingChaoZuZhi2, Unigene38317\_JingChaoZuZhi2, Unigene38321\_JingChaoZuZhi2, Unigene38336\_JingChaoZuZhi2, Unigene38468\_JingChaoZuZhi2, Unigene38502\_JingChaoZuZhi2, Unigene38651\_JingChaoZuZhi2, Unigene38801\_JingChaoZuZhi2, Unigene38955\_JingChaoZuZhi2, Unigene39029\_JingChaoZuZhi2, Unigene39197\_JingChaoZuZhi2, Unigene39222\_JingChaoZuZhi2, Unigene39316\_JingChaoZuZhi2, Unigene39392\_JingChaoZuZhi2, Unigene39457\_JingChaoZuZhi2, Unigene39594\_JingChaoZuZhi2, Unigene39650\_JingChaoZuZhi2, Unigene397\_JingChaoZuZhi2, Unigene39917\_JingChaoZuZhi2, Unigene39973\_JingChaoZuZhi2, Unigene40241\_JingChaoZuZhi2, Unigene40281\_JingChaoZuZhi2, Unigene40666\_JingChaoZuZhi2, Unigene40893\_JingChaoZuZhi2, Unigene410\_JingChaoZuZhi2, Unigene41200\_JingChaoZuZhi2, Unigene41407\_JingChaoZuZhi2, Unigene41430\_JingChaoZuZhi2, Unigene4144\_JingChaoZuZhi2, Unigene41542\_JingChaoZuZhi2, Unigene41624\_JingChaoZuZhi2, Unigene416\_JingChaoZuZhi2, Unigene41723\_JingChaoZuZhi2, Unigene41781\_JingChaoZuZhi2, Unigene41868\_JingChaoZuZhi2, Unigene41880\_JingChaoZuZhi2, Unigene42168\_JingChaoZuZhi2, Unigene42272\_JingChaoZuZhi2, Unigene42422\_JingChaoZuZhi2, Unigene42447\_JingChaoZuZhi2, Unigene42522\_JingChaoZuZhi2, Unigene42566\_JingChaoZuZhi2, Unigene42682\_JingChaoZuZhi2, Unigene42858\_JingChaoZuZhi2, Unigene42863\_JingChaoZuZhi2, Unigene43060\_JingChaoZuZhi2, Unigene43131\_JingChaoZuZhi2, Unigene43227\_JingChaoZuZhi2, Unigene43298\_JingChaoZuZhi2, Unigene43846\_JingChaoZuZhi2, Unigene44192\_JingChaoZuZhi2, Unigene44224\_JingChaoZuZhi2, Unigene44286\_JingChaoZuZhi2, Unigene44364\_JingChaoZuZhi2, Unigene4453\_JingChaoZuZhi2, Unigene4459\_JingChaoZuZhi2, Unigene44713\_JingChaoZuZhi2, Unigene44870\_JingChaoZuZhi2, Unigene44944\_JingChaoZuZhi2, Unigene45105\_JingChaoZuZhi2, Unigene452\_JingChaoZuZhi2, Unigene45394\_JingChaoZuZhi2, Unigene45642\_JingChaoZuZhi2, Unigene45724\_JingChaoZuZhi2, Unigene45778\_JingChaoZuZhi2, Unigene4584\_JingChaoZuZhi2, Unigene45907\_JingChaoZuZhi2, Unigene45940\_JingChaoZuZhi2, Unigene46151\_JingChaoZuZhi2, Unigene46222\_JingChaoZuZhi2, Unigene46663\_JingChaoZuZhi2, Unigene47002\_JingChaoZuZhi2, Unigene47022\_JingChaoZuZhi2, Unigene47056\_JingChaoZuZhi2, Unigene4730\_JingChaoZuZhi2, Unigene47416\_JingChaoZuZhi2, Unigene47451\_JingChaoZuZhi2, Unigene47574\_JingChaoZuZhi2, Unigene47765\_JingChaoZuZhi2, Unigene47979\_JingChaoZuZhi2, Unigene48141\_JingChaoZuZhi2, Unigene483\_JingChaoZuZhi2, Unigene4852\_JingChaoZuZhi2, Unigene48536\_JingChaoZuZhi2, Unigene48647\_JingChaoZuZhi2, Unigene48810\_JingChaoZuZhi2, Unigene48857\_JingChaoZuZhi2, Unigene4896\_JingChaoZuZhi2, Unigene49385\_JingChaoZuZhi2, Unigene49551\_JingChaoZuZhi2, Unigene49652\_JingChaoZuZhi2, Unigene49657\_JingChaoZuZhi2, Unigene49845\_JingChaoZuZhi2, Unigene49902\_JingChaoZuZhi2, Unigene50026\_JingChaoZuZhi2, Unigene50381\_JingChaoZuZhi2, Unigene50505\_JingChaoZuZhi2, Unigene50668\_JingChaoZuZhi2, Unigene50732\_JingChaoZuZhi2, Unigene50770\_JingChaoZuZhi2, Unigene50927\_JingChaoZuZhi2, Unigene51070\_JingChaoZuZhi2, Unigene5121\_JingChaoZuZhi2, Unigene51270\_JingChaoZuZhi2, Unigene51291\_JingChaoZuZhi2, Unigene51403\_JingChaoZuZhi2, Unigene51424\_JingChaoZuZhi2, Unigene51753\_JingChaoZuZhi2, Unigene52051\_JingChaoZuZhi2, Unigene52449\_JingChaoZuZhi2, Unigene52526\_JingChaoZuZhi2, Unigene5258\_JingChaoZuZhi2, Unigene5267\_JingChaoZuZhi2, Unigene52739\_JingChaoZuZhi2, Unigene52778\_JingChaoZuZhi2, Unigene52860\_JingChaoZuZhi2, Unigene52926\_JingChaoZuZhi2, Unigene53103\_JingChaoZuZhi2, Unigene535\_JingChaoZuZhi2, Unigene54144\_JingChaoZuZhi2, Unigene54242\_JingChaoZuZhi2, Unigene54285\_JingChaoZuZhi2, Unigene54385\_JingChaoZuZhi2, Unigene54408\_JingChaoZuZhi2, Unigene54412\_JingChaoZuZhi2, Unigene54569\_JingChaoZuZhi2, Unigene54790\_JingChaoZuZhi2, Unigene54862\_JingChaoZuZhi2, Unigene54965\_JingChaoZuZhi2, Unigene55111\_JingChaoZuZhi2, Unigene55393\_JingChaoZuZhi2, Unigene55409\_JingChaoZuZhi2, Unigene55425\_JingChaoZuZhi2, Unigene55437\_JingChaoZuZhi2, Unigene55541\_JingChaoZuZhi2, Unigene55788\_JingChaoZuZhi2, Unigene55909\_JingChaoZuZhi2, Unigene55999\_JingChaoZuZhi2, Unigene56143\_JingChaoZuZhi2, Unigene56148\_JingChaoZuZhi2, Unigene56341\_JingChaoZuZhi2, Unigene56342\_JingChaoZuZhi2, Unigene56490\_JingChaoZuZhi2, Unigene56863\_JingChaoZuZhi2, Unigene56907\_JingChaoZuZhi2, Unigene57037\_JingChaoZuZhi2, Unigene57160\_JingChaoZuZhi2, Unigene57552\_JingChaoZuZhi2, Unigene57586\_JingChaoZuZhi2, Unigene57593\_JingChaoZuZhi2, Unigene57641\_JingChaoZuZhi2, Unigene58091\_JingChaoZuZhi2, Unigene5824\_JingChaoZuZhi2, Unigene58696\_JingChaoZuZhi2, Unigene586\_JingChaoZuZhi2, Unigene58914\_JingChaoZuZhi2, Unigene58945\_JingChaoZuZhi2, Unigene5895\_JingChaoZuZhi2, Unigene59083\_JingChaoZuZhi2, Unigene59118\_JingChaoZuZhi2, Unigene59166\_JingChaoZuZhi2, Unigene59465\_JingChaoZuZhi2, Unigene59626\_JingChaoZuZhi2, Unigene59909\_JingChaoZuZhi2, Unigene59921\_JingChaoZuZhi2, Unigene59964\_JingChaoZuZhi2, Unigene60028\_JingChaoZuZhi2, Unigene60151\_JingChaoZuZhi2, Unigene60187\_JingChaoZuZhi2, Unigene60359\_JingChaoZuZhi2, Unigene6042\_JingChaoZuZhi2, Unigene60543\_JingChaoZuZhi2, Unigene6056\_JingChaoZuZhi2, Unigene6072\_JingChaoZuZhi2, Unigene61107\_JingChaoZuZhi2, Unigene6120\_JingChaoZuZhi2, Unigene61736\_JingChaoZuZhi2, Unigene6179\_JingChaoZuZhi2, Unigene61845\_JingChaoZuZhi2, Unigene61855\_JingChaoZuZhi2, Unigene61887\_JingChaoZuZhi2, Unigene619\_JingChaoZuZhi2, Unigene62081\_JingChaoZuZhi2, Unigene62220\_JingChaoZuZhi2, Unigene62255\_JingChaoZuZhi2, Unigene62317\_JingChaoZuZhi2, Unigene62690\_JingChaoZuZhi2, Unigene63091\_JingChaoZuZhi2, Unigene63149\_JingChaoZuZhi2, Unigene6321\_JingChaoZuZhi2, Unigene63351\_JingChaoZuZhi2, Unigene63368\_JingChaoZuZhi2, Unigene63771\_JingChaoZuZhi2, Unigene63829\_JingChaoZuZhi2, Unigene64236\_JingChaoZuZhi2, Unigene64324\_JingChaoZuZhi2, Unigene64387\_JingChaoZuZhi2, Unigene64474\_JingChaoZuZhi2, Unigene64499\_JingChaoZuZhi2, Unigene64520\_JingChaoZuZhi2, Unigene64623\_JingChaoZuZhi2, Unigene64666\_JingChaoZuZhi2, Unigene64678\_JingChaoZuZhi2, Unigene64799\_JingChaoZuZhi2, Unigene65096\_JingChaoZuZhi2, Unigene6519\_JingChaoZuZhi2, Unigene65205\_JingChaoZuZhi2, Unigene65219\_JingChaoZuZhi2, Unigene65614\_JingChaoZuZhi2, Unigene65621\_JingChaoZuZhi2, Unigene65724\_JingChaoZuZhi2, Unigene65831\_JingChaoZuZhi2, Unigene65968\_JingChaoZuZhi2, Unigene6598\_JingChaoZuZhi2, Unigene66002\_JingChaoZuZhi2, Unigene66102\_JingChaoZuZhi2, Unigene66116\_JingChaoZuZhi2, Unigene66121\_JingChaoZuZhi2, Unigene66172\_JingChaoZuZhi2, Unigene66319\_JingChaoZuZhi2, Unigene6651\_JingChaoZuZhi2, Unigene66586\_JingChaoZuZhi2, Unigene66708\_JingChaoZuZhi2, Unigene66922\_JingChaoZuZhi2, Unigene67088\_JingChaoZuZhi2, Unigene67102\_JingChaoZuZhi2, Unigene67112\_JingChaoZuZhi2, Unigene67283\_JingChaoZuZhi2, Unigene67321\_JingChaoZuZhi2, Unigene6753\_JingChaoZuZhi2, Unigene67583\_JingChaoZuZhi2, Unigene67599\_JingChaoZuZhi2, Unigene67661\_JingChaoZuZhi2, Unigene67798\_JingChaoZuZhi2, Unigene67845\_JingChaoZuZhi2, Unigene67883\_JingChaoZuZhi2, Unigene67889\_JingChaoZuZhi2, Unigene68013\_JingChaoZuZhi2, Unigene68214\_JingChaoZuZhi2, Unigene68272\_JingChaoZuZhi2, Unigene6832\_JingChaoZuZhi2, Unigene68337\_JingChaoZuZhi2, Unigene68460\_JingChaoZuZhi2, Unigene68569\_JingChaoZuZhi2, Unigene68711\_JingChaoZuZhi2, Unigene68783\_JingChaoZuZhi2, Unigene68883\_JingChaoZuZhi2, Unigene69293\_JingChaoZuZhi2, Unigene6929\_JingChaoZuZhi2, Unigene692\_JingChaoZuZhi2, Unigene69362\_JingChaoZuZhi2, Unigene6939\_JingChaoZuZhi2, Unigene69508\_JingChaoZuZhi2, Unigene69575\_JingChaoZuZhi2, Unigene69647\_JingChaoZuZhi2, Unigene69689\_JingChaoZuZhi2, Unigene69938\_JingChaoZuZhi2, Unigene70063\_JingChaoZuZhi2, Unigene70182\_JingChaoZuZhi2, Unigene70234\_JingChaoZuZhi2, Unigene70328\_JingChaoZuZhi2, Unigene7032\_JingChaoZuZhi2, Unigene70605\_JingChaoZuZhi2, Unigene71214\_JingChaoZuZhi2, Unigene71355\_JingChaoZuZhi2, Unigene71630\_JingChaoZuZhi2, Unigene71692\_JingChaoZuZhi2, Unigene71732\_JingChaoZuZhi2, Unigene71822\_JingChaoZuZhi2, Unigene71862\_JingChaoZuZhi2, Unigene71881\_JingChaoZuZhi2, Unigene71950\_JingChaoZuZhi2, Unigene71956\_JingChaoZuZhi2, Unigene71979\_JingChaoZuZhi2, Unigene72036\_JingChaoZuZhi2, Unigene72130\_JingChaoZuZhi2, Unigene72185\_JingChaoZuZhi2, Unigene72362\_JingChaoZuZhi2, Unigene72524\_JingChaoZuZhi2, Unigene72539\_JingChaoZuZhi2, Unigene72579\_JingChaoZuZhi2, Unigene728\_JingChaoZuZhi2, Unigene72923\_JingChaoZuZhi2, Unigene72987\_JingChaoZuZhi2, Unigene72990\_JingChaoZuZhi2, Unigene73115\_JingChaoZuZhi2, Unigene73132\_JingChaoZuZhi2, Unigene73208\_JingChaoZuZhi2, Unigene73242\_JingChaoZuZhi2, Unigene73266\_JingChaoZuZhi2, Unigene73287\_JingChaoZuZhi2, Unigene73318\_JingChaoZuZhi2, Unigene73379\_JingChaoZuZhi2, Unigene73401\_JingChaoZuZhi2, Unigene73435\_JingChaoZuZhi2, Unigene736\_JingChaoZuZhi2, Unigene73926\_JingChaoZuZhi2, Unigene74163\_JingChaoZuZhi2, Unigene74166\_JingChaoZuZhi2, Unigene74288\_JingChaoZuZhi2, Unigene74402\_JingChaoZuZhi2, Unigene74421\_JingChaoZuZhi2, Unigene74441\_JingChaoZuZhi2, Unigene74606\_JingChaoZuZhi2, Unigene74607\_JingChaoZuZhi2, Unigene74701\_JingChaoZuZhi2, Unigene747\_JingChaoZuZhi2, Unigene74832\_JingChaoZuZhi2, Unigene74850\_JingChaoZuZhi2, Unigene74872\_JingChaoZuZhi2, Unigene74994\_JingChaoZuZhi2, Unigene75130\_JingChaoZuZhi2, Unigene75147\_JingChaoZuZhi2, Unigene75345\_JingChaoZuZhi2, Unigene75503\_JingChaoZuZhi2, Unigene75633\_JingChaoZuZhi2, Unigene75799\_JingChaoZuZhi2, Unigene75899\_JingChaoZuZhi2, Unigene75924\_JingChaoZuZhi2, Unigene76005\_JingChaoZuZhi2, Unigene76058\_JingChaoZuZhi2, Unigene7609\_JingChaoZuZhi2, Unigene76127\_JingChaoZuZhi2, Unigene76177\_JingChaoZuZhi2, Unigene76228\_JingChaoZuZhi2, Unigene76446\_JingChaoZuZhi2, Unigene76614\_JingChaoZuZhi2, Unigene76669\_JingChaoZuZhi2, Unigene76709\_JingChaoZuZhi2, Unigene76726\_JingChaoZuZhi2, Unigene76748\_JingChaoZuZhi2, Unigene76762\_JingChaoZuZhi2, Unigene76786\_JingChaoZuZhi2, Unigene7679\_JingChaoZuZhi2, Unigene76821\_JingChaoZuZhi2, Unigene76912\_JingChaoZuZhi2, Unigene76995\_JingChaoZuZhi2, Unigene76997\_JingChaoZuZhi2, Unigene7705\_JingChaoZuZhi2, Unigene77064\_JingChaoZuZhi2, Unigene77151\_JingChaoZuZhi2, Unigene7723\_JingChaoZuZhi2, Unigene77367\_JingChaoZuZhi2, Unigene77513\_JingChaoZuZhi2, Unigene77568\_JingChaoZuZhi2, Unigene7756\_JingChaoZuZhi2, Unigene77612\_JingChaoZuZhi2, Unigene77687\_JingChaoZuZhi2, Unigene77723\_JingChaoZuZhi2, Unigene77790\_JingChaoZuZhi2, Unigene777\_JingChaoZuZhi2, Unigene77815\_JingChaoZuZhi2, Unigene78067\_JingChaoZuZhi2, Unigene78112\_JingChaoZuZhi2, Unigene78191\_JingChaoZuZhi2, Unigene78243\_JingChaoZuZhi2, Unigene78390\_JingChaoZuZhi2, Unigene78542\_JingChaoZuZhi2, Unigene78561\_JingChaoZuZhi2, Unigene78587\_JingChaoZuZhi2, Unigene78626\_JingChaoZuZhi2, Unigene79063\_JingChaoZuZhi2, Unigene79152\_JingChaoZuZhi2, Unigene79206\_JingChaoZuZhi2, Unigene79280\_JingChaoZuZhi2, Unigene79333\_JingChaoZuZhi2, Unigene7936\_JingChaoZuZhi2, Unigene79388\_JingChaoZuZhi2, Unigene79399\_JingChaoZuZhi2, Unigene79508\_JingChaoZuZhi2, Unigene79592\_JingChaoZuZhi2, Unigene79618\_JingChaoZuZhi2, Unigene79712\_JingChaoZuZhi2, Unigene79733\_JingChaoZuZhi2, Unigene79811\_JingChaoZuZhi2, Unigene79857\_JingChaoZuZhi2, Unigene79899\_JingChaoZuZhi2, Unigene79950\_JingChaoZuZhi2, Unigene79975\_JingChaoZuZhi2, Unigene80043\_JingChaoZuZhi2, Unigene8015\_JingChaoZuZhi2, Unigene80189\_JingChaoZuZhi2, Unigene80244\_JingChaoZuZhi2, Unigene80310\_JingChaoZuZhi2, Unigene80481\_JingChaoZuZhi2, Unigene80657\_JingChaoZuZhi2, Unigene80685\_JingChaoZuZhi2, Unigene8069\_JingChaoZuZhi2, Unigene80795\_JingChaoZuZhi2, Unigene80826\_JingChaoZuZhi2, Unigene80889\_JingChaoZuZhi2, Unigene80931\_JingChaoZuZhi2, Unigene80953\_JingChaoZuZhi2, Unigene80968\_JingChaoZuZhi2, Unigene81055\_JingChaoZuZhi2, Unigene81059\_JingChaoZuZhi2, Unigene81082\_JingChaoZuZhi2, Unigene81119\_JingChaoZuZhi2, Unigene81229\_JingChaoZuZhi2, Unigene81366\_JingChaoZuZhi2, Unigene81491\_JingChaoZuZhi2, Unigene81551\_JingChaoZuZhi2, Unigene81684\_JingChaoZuZhi2, Unigene817\_JingChaoZuZhi2, Unigene8182\_JingChaoZuZhi2, Unigene81864\_JingChaoZuZhi2, Unigene81955\_JingChaoZuZhi2, Unigene82018\_JingChaoZuZhi2, Unigene82101\_JingChaoZuZhi2, Unigene82138\_JingChaoZuZhi2, Unigene82143\_JingChaoZuZhi2, Unigene8221\_JingChaoZuZhi2, Unigene8229\_JingChaoZuZhi2, Unigene82300\_JingChaoZuZhi2, Unigene82366\_JingChaoZuZhi2, Unigene82370\_JingChaoZuZhi2, Unigene82446\_JingChaoZuZhi2, Unigene82513\_JingChaoZuZhi2, Unigene82521\_JingChaoZuZhi2, Unigene82547\_JingChaoZuZhi2, Unigene82595\_JingChaoZuZhi2, Unigene82742\_JingChaoZuZhi2, Unigene82773\_JingChaoZuZhi2, Unigene82821\_JingChaoZuZhi2, Unigene82843\_JingChaoZuZhi2, Unigene82907\_JingChaoZuZhi2, Unigene82943\_JingChaoZuZhi2, Unigene82986\_JingChaoZuZhi2, Unigene83000\_JingChaoZuZhi2, Unigene83124\_JingChaoZuZhi2, Unigene83176\_JingChaoZuZhi2, Unigene83267\_JingChaoZuZhi2, Unigene83540\_JingChaoZuZhi2, Unigene83590\_JingChaoZuZhi2, Unigene83604\_JingChaoZuZhi2, Unigene83678\_JingChaoZuZhi2, Unigene83754\_JingChaoZuZhi2, Unigene83880\_JingChaoZuZhi2, Unigene839\_JingChaoZuZhi2, Unigene84017\_JingChaoZuZhi2, Unigene84032\_JingChaoZuZhi2, Unigene84111\_JingChaoZuZhi2, Unigene84116\_JingChaoZuZhi2, Unigene84180\_JingChaoZuZhi2, Unigene84216\_JingChaoZuZhi2, Unigene84409\_JingChaoZuZhi2, Unigene84431\_JingChaoZuZhi2, Unigene8460\_JingChaoZuZhi2, Unigene84610\_JingChaoZuZhi2, Unigene84638\_JingChaoZuZhi2, Unigene84673\_JingChaoZuZhi2, Unigene84731\_JingChaoZuZhi2, Unigene84806\_JingChaoZuZhi2, Unigene84931\_JingChaoZuZhi2, Unigene84960\_JingChaoZuZhi2, Unigene84979\_JingChaoZuZhi2, Unigene85458\_JingChaoZuZhi2, Unigene85541\_JingChaoZuZhi2, Unigene85632\_JingChaoZuZhi2, Unigene85774\_JingChaoZuZhi2, Unigene85775\_JingChaoZuZhi2, Unigene85804\_JingChaoZuZhi2, Unigene85823\_JingChaoZuZhi2, Unigene85844\_JingChaoZuZhi2, Unigene85849\_JingChaoZuZhi2, Unigene85909\_JingChaoZuZhi2, Unigene85979\_JingChaoZuZhi2, Unigene86028\_JingChaoZuZhi2, Unigene86050\_JingChaoZuZhi2, Unigene86294\_JingChaoZuZhi2, Unigene86440\_JingChaoZuZhi2, Unigene86519\_JingChaoZuZhi2, Unigene86748\_JingChaoZuZhi2, Unigene86749\_JingChaoZuZhi2, Unigene86808\_JingChaoZuZhi2, Unigene87014\_JingChaoZuZhi2, Unigene87053\_JingChaoZuZhi2, Unigene87118\_JingChaoZuZhi2, Unigene87180\_JingChaoZuZhi2, Unigene8732\_JingChaoZuZhi2, Unigene87331\_JingChaoZuZhi2, Unigene87357\_JingChaoZuZhi2, Unigene87378\_JingChaoZuZhi2, Unigene87447\_JingChaoZuZhi2, Unigene87543\_JingChaoZuZhi2, Unigene8777\_JingChaoZuZhi2, Unigene877\_JingChaoZuZhi2, Unigene87867\_JingChaoZuZhi2, Unigene87938\_JingChaoZuZhi2, Unigene88072\_JingChaoZuZhi2, Unigene88177\_JingChaoZuZhi2, Unigene88232\_JingChaoZuZhi2, Unigene88449\_JingChaoZuZhi2, Unigene88454\_JingChaoZuZhi2, Unigene88461\_JingChaoZuZhi2, Unigene88538\_JingChaoZuZhi2, Unigene8853\_JingChaoZuZhi2, Unigene88563\_JingChaoZuZhi2, Unigene88594\_JingChaoZuZhi2, Unigene88664\_JingChaoZuZhi2, Unigene88665\_JingChaoZuZhi2, Unigene88724\_JingChaoZuZhi2, Unigene88823\_JingChaoZuZhi2, Unigene88844\_JingChaoZuZhi2, Unigene88851\_JingChaoZuZhi2, Unigene88957\_JingChaoZuZhi2, Unigene89089\_JingChaoZuZhi2, Unigene89285\_JingChaoZuZhi2, Unigene89320\_JingChaoZuZhi2, Unigene89385\_JingChaoZuZhi2, Unigene89434\_JingChaoZuZhi2, Unigene89645\_JingChaoZuZhi2, Unigene89659\_JingChaoZuZhi2, Unigene89728\_JingChaoZuZhi2, Unigene89937\_JingChaoZuZhi2, Unigene89999\_JingChaoZuZhi2, Unigene90075\_JingChaoZuZhi2, Unigene90196\_JingChaoZuZhi2, Unigene90257\_JingChaoZuZhi2, Unigene90383\_JingChaoZuZhi2, Unigene90459\_JingChaoZuZhi2, Unigene90484\_JingChaoZuZhi2, Unigene90515\_JingChaoZuZhi2, Unigene90822\_JingChaoZuZhi2, Unigene9087\_JingChaoZuZhi2, Unigene91310\_JingChaoZuZhi2, Unigene91503\_JingChaoZuZhi2, Unigene91586\_JingChaoZuZhi2, Unigene91673\_JingChaoZuZhi2, Unigene9181\_JingChaoZuZhi2, Unigene91841\_JingChaoZuZhi2, Unigene91924\_JingChaoZuZhi2, Unigene91974\_JingChaoZuZhi2, Unigene92047\_JingChaoZuZhi2, Unigene92211\_JingChaoZuZhi2, Unigene92252\_JingChaoZuZhi2, Unigene92310\_JingChaoZuZhi2, Unigene92325\_JingChaoZuZhi2, Unigene92433\_JingChaoZuZhi2, Unigene92510\_JingChaoZuZhi2, Unigene92573\_JingChaoZuZhi2, Unigene92581\_JingChaoZuZhi2, Unigene92602\_JingChaoZuZhi2, Unigene92747\_JingChaoZuZhi2, Unigene9284\_JingChaoZuZhi2, Unigene9288\_JingChaoZuZhi2, Unigene93084\_JingChaoZuZhi2, Unigene93176\_JingChaoZuZhi2, Unigene93193\_JingChaoZuZhi2, Unigene93239\_JingChaoZuZhi2, Unigene93538\_JingChaoZuZhi2, Unigene93588\_JingChaoZuZhi2, Unigene93714\_JingChaoZuZhi2, Unigene93823\_JingChaoZuZhi2, Unigene93978\_JingChaoZuZhi2, Unigene9403\_JingChaoZuZhi2, Unigene9408\_JingChaoZuZhi2, Unigene94442\_JingChaoZuZhi2, Unigene94588\_JingChaoZuZhi2, Unigene94678\_JingChaoZuZhi2, Unigene94763\_JingChaoZuZhi2, Unigene948\_JingChaoZuZhi2, Unigene94912\_JingChaoZuZhi2, Unigene94950\_JingChaoZuZhi2, Unigene94958\_JingChaoZuZhi2, Unigene95153\_JingChaoZuZhi2, Unigene95190\_JingChaoZuZhi2, Unigene95236\_JingChaoZuZhi2, Unigene95332\_JingChaoZuZhi2, Unigene95431\_JingChaoZuZhi2, Unigene95439\_JingChaoZuZhi2, Unigene95741\_JingChaoZuZhi2, Unigene95826\_JingChaoZuZhi2, Unigene95961\_JingChaoZuZhi2, Unigene9610\_JingChaoZuZhi2, Unigene96172\_JingChaoZuZhi2, Unigene9618\_JingChaoZuZhi2, Unigene9657\_JingChaoZuZhi2, Unigene9676\_JingChaoZuZhi2, Unigene9886\_JingChaoZuZhi2, Unigene989\_JingChaoZuZhi2, Unigene992\_JingChaoZuZhi2 |
| 8 | Fc gamma R-mediated phagocytosis | Unigene10016\_JingChaoZuZhi2, Unigene10051\_JingChaoZuZhi2, Unigene10053\_JingChaoZuZhi2, Unigene10128\_JingChaoZuZhi2, Unigene1017\_JingChaoZuZhi2, Unigene10208\_JingChaoZuZhi2, Unigene10318\_JingChaoZuZhi2, Unigene10456\_JingChaoZuZhi2, Unigene10492\_JingChaoZuZhi2, Unigene10498\_JingChaoZuZhi2, Unigene10532\_JingChaoZuZhi2, Unigene10576\_JingChaoZuZhi2, Unigene10582\_JingChaoZuZhi2, Unigene10733\_JingChaoZuZhi2, Unigene10772\_JingChaoZuZhi2, Unigene10809\_JingChaoZuZhi2, Unigene10922\_JingChaoZuZhi2, Unigene10936\_JingChaoZuZhi2, Unigene10974\_JingChaoZuZhi2, Unigene10979\_JingChaoZuZhi2, Unigene11055\_JingChaoZuZhi2, Unigene1121\_JingChaoZuZhi2, Unigene11240\_JingChaoZuZhi2, Unigene11258\_JingChaoZuZhi2, Unigene11569\_JingChaoZuZhi2, Unigene115\_JingChaoZuZhi2, Unigene11729\_JingChaoZuZhi2, Unigene11788\_JingChaoZuZhi2, Unigene11810\_JingChaoZuZhi2, Unigene11834\_JingChaoZuZhi2, Unigene11851\_JingChaoZuZhi2, Unigene11902\_JingChaoZuZhi2, Unigene11918\_JingChaoZuZhi2, Unigene11932\_JingChaoZuZhi2, Unigene11961\_JingChaoZuZhi2, Unigene11995\_JingChaoZuZhi2, Unigene12011\_JingChaoZuZhi2, Unigene12148\_JingChaoZuZhi2, Unigene12260\_JingChaoZuZhi2, Unigene12285\_JingChaoZuZhi2, Unigene12310\_JingChaoZuZhi2, Unigene12326\_JingChaoZuZhi2, Unigene12343\_JingChaoZuZhi2, Unigene12372\_JingChaoZuZhi2, Unigene12549\_JingChaoZuZhi2, Unigene12644\_JingChaoZuZhi2, Unigene12815\_JingChaoZuZhi2, Unigene12899\_JingChaoZuZhi2, Unigene12937\_JingChaoZuZhi2, Unigene13085\_JingChaoZuZhi2, Unigene13097\_JingChaoZuZhi2, Unigene13149\_JingChaoZuZhi2, Unigene13180\_JingChaoZuZhi2, Unigene13196\_JingChaoZuZhi2, Unigene13249\_JingChaoZuZhi2, Unigene1325\_JingChaoZuZhi2, Unigene13285\_JingChaoZuZhi2, Unigene13342\_JingChaoZuZhi2, Unigene13420\_JingChaoZuZhi2, Unigene13478\_JingChaoZuZhi2, Unigene13572\_JingChaoZuZhi2, Unigene13607\_JingChaoZuZhi2, Unigene13616\_JingChaoZuZhi2, Unigene13618\_JingChaoZuZhi2, Unigene13650\_JingChaoZuZhi2, Unigene13720\_JingChaoZuZhi2, Unigene13766\_JingChaoZuZhi2, Unigene13869\_JingChaoZuZhi2, Unigene13890\_JingChaoZuZhi2, Unigene13901\_JingChaoZuZhi2, Unigene13904\_JingChaoZuZhi2, Unigene13927\_JingChaoZuZhi2, Unigene13946\_JingChaoZuZhi2, Unigene14068\_JingChaoZuZhi2, Unigene14071\_JingChaoZuZhi2, Unigene14159\_JingChaoZuZhi2, Unigene14193\_JingChaoZuZhi2, Unigene14246\_JingChaoZuZhi2, Unigene14289\_JingChaoZuZhi2, Unigene14357\_JingChaoZuZhi2, Unigene14450\_JingChaoZuZhi2, Unigene14476\_JingChaoZuZhi2, Unigene14534\_JingChaoZuZhi2, Unigene14610\_JingChaoZuZhi2, Unigene14714\_JingChaoZuZhi2, Unigene14715\_JingChaoZuZhi2, Unigene14845\_JingChaoZuZhi2, Unigene14912\_JingChaoZuZhi2, Unigene14970\_JingChaoZuZhi2, Unigene15088\_JingChaoZuZhi2, Unigene15095\_JingChaoZuZhi2, Unigene15096\_JingChaoZuZhi2, Unigene15176\_JingChaoZuZhi2, Unigene15216\_JingChaoZuZhi2, Unigene15228\_JingChaoZuZhi2, Unigene15251\_JingChaoZuZhi2, Unigene15286\_JingChaoZuZhi2, Unigene15288\_JingChaoZuZhi2, Unigene15319\_JingChaoZuZhi2, Unigene15374\_JingChaoZuZhi2, Unigene15380\_JingChaoZuZhi2, Unigene15381\_JingChaoZuZhi2, Unigene15425\_JingChaoZuZhi2, Unigene15492\_JingChaoZuZhi2, Unigene15615\_JingChaoZuZhi2, Unigene15650\_JingChaoZuZhi2, Unigene15651\_JingChaoZuZhi2, Unigene15659\_JingChaoZuZhi2, Unigene15731\_JingChaoZuZhi2, Unigene15736\_JingChaoZuZhi2, Unigene15794\_JingChaoZuZhi2, Unigene15857\_JingChaoZuZhi2, Unigene15879\_JingChaoZuZhi2, Unigene15942\_JingChaoZuZhi2, Unigene16071\_JingChaoZuZhi2, Unigene16103\_JingChaoZuZhi2, Unigene16176\_JingChaoZuZhi2, Unigene16182\_JingChaoZuZhi2, Unigene16318\_JingChaoZuZhi2, Unigene16402\_JingChaoZuZhi2, Unigene16481\_JingChaoZuZhi2, Unigene16591\_JingChaoZuZhi2, Unigene16606\_JingChaoZuZhi2, Unigene16655\_JingChaoZuZhi2, Unigene16709\_JingChaoZuZhi2, Unigene16730\_JingChaoZuZhi2, Unigene16950\_JingChaoZuZhi2, Unigene17101\_JingChaoZuZhi2, Unigene17122\_JingChaoZuZhi2, Unigene17247\_JingChaoZuZhi2, Unigene17501\_JingChaoZuZhi2, Unigene17506\_JingChaoZuZhi2, Unigene17547\_JingChaoZuZhi2, Unigene17764\_JingChaoZuZhi2, Unigene17789\_JingChaoZuZhi2, Unigene17829\_JingChaoZuZhi2, Unigene17839\_JingChaoZuZhi2, Unigene17845\_JingChaoZuZhi2, Unigene17888\_JingChaoZuZhi2, Unigene17894\_JingChaoZuZhi2, Unigene18010\_JingChaoZuZhi2, Unigene18256\_JingChaoZuZhi2, Unigene18259\_JingChaoZuZhi2, Unigene18260\_JingChaoZuZhi2, Unigene18269\_JingChaoZuZhi2, Unigene18334\_JingChaoZuZhi2, Unigene18348\_JingChaoZuZhi2, Unigene18464\_JingChaoZuZhi2, Unigene18505\_JingChaoZuZhi2, Unigene18662\_JingChaoZuZhi2, Unigene18672\_JingChaoZuZhi2, Unigene18685\_JingChaoZuZhi2, Unigene18790\_JingChaoZuZhi2, Unigene18835\_JingChaoZuZhi2, Unigene18868\_JingChaoZuZhi2, Unigene19001\_JingChaoZuZhi2, Unigene19021\_JingChaoZuZhi2, Unigene19086\_JingChaoZuZhi2, Unigene19137\_JingChaoZuZhi2, Unigene19204\_JingChaoZuZhi2, Unigene19223\_JingChaoZuZhi2, Unigene19319\_JingChaoZuZhi2, Unigene19391\_JingChaoZuZhi2, Unigene19512\_JingChaoZuZhi2, Unigene19533\_JingChaoZuZhi2, Unigene19545\_JingChaoZuZhi2, Unigene19579\_JingChaoZuZhi2, Unigene19679\_JingChaoZuZhi2, Unigene19701\_JingChaoZuZhi2, Unigene19883\_JingChaoZuZhi2, Unigene19904\_JingChaoZuZhi2, Unigene19979\_JingChaoZuZhi2, Unigene20025\_JingChaoZuZhi2, Unigene20030\_JingChaoZuZhi2, Unigene20049\_JingChaoZuZhi2, Unigene20083\_JingChaoZuZhi2, Unigene201\_JingChaoZuZhi2, Unigene20239\_JingChaoZuZhi2, Unigene20373\_JingChaoZuZhi2, Unigene20394\_JingChaoZuZhi2, Unigene20410\_JingChaoZuZhi2, Unigene20468\_JingChaoZuZhi2, Unigene20474\_JingChaoZuZhi2, Unigene20557\_JingChaoZuZhi2, Unigene20720\_JingChaoZuZhi2, Unigene20727\_JingChaoZuZhi2, Unigene20837\_JingChaoZuZhi2, Unigene20897\_JingChaoZuZhi2, Unigene20918\_JingChaoZuZhi2, Unigene20943\_JingChaoZuZhi2, Unigene21057\_JingChaoZuZhi2, Unigene2115\_JingChaoZuZhi2, Unigene21295\_JingChaoZuZhi2, Unigene21311\_JingChaoZuZhi2, Unigene21407\_JingChaoZuZhi2, Unigene21543\_JingChaoZuZhi2, Unigene21578\_JingChaoZuZhi2, Unigene21636\_JingChaoZuZhi2, Unigene21860\_JingChaoZuZhi2, Unigene21898\_JingChaoZuZhi2, Unigene21932\_JingChaoZuZhi2, Unigene21933\_JingChaoZuZhi2, Unigene22055\_JingChaoZuZhi2, Unigene22091\_JingChaoZuZhi2, Unigene22119\_JingChaoZuZhi2, Unigene22128\_JingChaoZuZhi2, Unigene22174\_JingChaoZuZhi2, Unigene22220\_JingChaoZuZhi2, Unigene2226\_JingChaoZuZhi2, Unigene22329\_JingChaoZuZhi2, Unigene22372\_JingChaoZuZhi2, Unigene22414\_JingChaoZuZhi2, Unigene22455\_JingChaoZuZhi2, Unigene22482\_JingChaoZuZhi2, Unigene22586\_JingChaoZuZhi2, Unigene2258\_JingChaoZuZhi2, Unigene2275\_JingChaoZuZhi2, Unigene22779\_JingChaoZuZhi2, Unigene22804\_JingChaoZuZhi2, Unigene2289\_JingChaoZuZhi2, Unigene22909\_JingChaoZuZhi2, Unigene23164\_JingChaoZuZhi2, Unigene23199\_JingChaoZuZhi2, Unigene23447\_JingChaoZuZhi2, Unigene23924\_JingChaoZuZhi2, Unigene23978\_JingChaoZuZhi2, Unigene2445\_JingChaoZuZhi2, Unigene2456\_JingChaoZuZhi2, Unigene24600\_JingChaoZuZhi2, Unigene2510\_JingChaoZuZhi2, Unigene2517\_JingChaoZuZhi2, Unigene25350\_JingChaoZuZhi2, Unigene25693\_JingChaoZuZhi2, Unigene25962\_JingChaoZuZhi2, Unigene26730\_JingChaoZuZhi2, Unigene28064\_JingChaoZuZhi2, Unigene28243\_JingChaoZuZhi2, Unigene2857\_JingChaoZuZhi2, Unigene28793\_JingChaoZuZhi2, Unigene2901\_JingChaoZuZhi2, Unigene2960\_JingChaoZuZhi2, Unigene30281\_JingChaoZuZhi2, Unigene31234\_JingChaoZuZhi2, Unigene31912\_JingChaoZuZhi2, Unigene31934\_JingChaoZuZhi2, Unigene3235\_JingChaoZuZhi2, Unigene32373\_JingChaoZuZhi2, Unigene324\_JingChaoZuZhi2, Unigene33044\_JingChaoZuZhi2, Unigene33173\_JingChaoZuZhi2, Unigene3321\_JingChaoZuZhi2, Unigene3388\_JingChaoZuZhi2, Unigene34142\_JingChaoZuZhi2, Unigene34785\_JingChaoZuZhi2, Unigene3493\_JingChaoZuZhi2, Unigene35080\_JingChaoZuZhi2, Unigene35927\_JingChaoZuZhi2, Unigene36257\_JingChaoZuZhi2, Unigene36342\_JingChaoZuZhi2, Unigene36361\_JingChaoZuZhi2, Unigene3646\_JingChaoZuZhi2, Unigene3671\_JingChaoZuZhi2, Unigene3693\_JingChaoZuZhi2, Unigene36974\_JingChaoZuZhi2, Unigene36976\_JingChaoZuZhi2, Unigene3700\_JingChaoZuZhi2, Unigene37164\_JingChaoZuZhi2, Unigene37818\_JingChaoZuZhi2, Unigene3854\_JingChaoZuZhi2, Unigene39216\_JingChaoZuZhi2, Unigene3931\_JingChaoZuZhi2, Unigene4015\_JingChaoZuZhi2, Unigene40200\_JingChaoZuZhi2, Unigene40281\_JingChaoZuZhi2, Unigene4044\_JingChaoZuZhi2, Unigene41040\_JingChaoZuZhi2, Unigene41148\_JingChaoZuZhi2, Unigene411\_JingChaoZuZhi2, Unigene41298\_JingChaoZuZhi2, Unigene4188\_JingChaoZuZhi2, Unigene42032\_JingChaoZuZhi2, Unigene4209\_JingChaoZuZhi2, Unigene42146\_JingChaoZuZhi2, Unigene42256\_JingChaoZuZhi2, Unigene42329\_JingChaoZuZhi2, Unigene4254\_JingChaoZuZhi2, Unigene43041\_JingChaoZuZhi2, Unigene43147\_JingChaoZuZhi2, Unigene43169\_JingChaoZuZhi2, Unigene43249\_JingChaoZuZhi2, Unigene43260\_JingChaoZuZhi2, Unigene4337\_JingChaoZuZhi2, Unigene4345\_JingChaoZuZhi2, Unigene43512\_JingChaoZuZhi2, Unigene43574\_JingChaoZuZhi2, Unigene43723\_JingChaoZuZhi2, Unigene4390\_JingChaoZuZhi2, Unigene44246\_JingChaoZuZhi2, Unigene44516\_JingChaoZuZhi2, Unigene45134\_JingChaoZuZhi2, Unigene45367\_JingChaoZuZhi2, Unigene4547\_JingChaoZuZhi2, Unigene45532\_JingChaoZuZhi2, Unigene45593\_JingChaoZuZhi2, Unigene45622\_JingChaoZuZhi2, Unigene45901\_JingChaoZuZhi2, Unigene45962\_JingChaoZuZhi2, Unigene46497\_JingChaoZuZhi2, Unigene4713\_JingChaoZuZhi2, Unigene47578\_JingChaoZuZhi2, Unigene4758\_JingChaoZuZhi2, Unigene4775\_JingChaoZuZhi2, Unigene47872\_JingChaoZuZhi2, Unigene47949\_JingChaoZuZhi2, Unigene4829\_JingChaoZuZhi2, Unigene4873\_JingChaoZuZhi2, Unigene48751\_JingChaoZuZhi2, Unigene49387\_JingChaoZuZhi2, Unigene49664\_JingChaoZuZhi2, Unigene49973\_JingChaoZuZhi2, Unigene50231\_JingChaoZuZhi2, Unigene50540\_JingChaoZuZhi2, Unigene50668\_JingChaoZuZhi2, Unigene50744\_JingChaoZuZhi2, Unigene5108\_JingChaoZuZhi2, Unigene51090\_JingChaoZuZhi2, Unigene51115\_JingChaoZuZhi2, Unigene5125\_JingChaoZuZhi2, Unigene5126\_JingChaoZuZhi2, Unigene5196\_JingChaoZuZhi2, Unigene52100\_JingChaoZuZhi2, Unigene52125\_JingChaoZuZhi2, Unigene52456\_JingChaoZuZhi2, Unigene524\_JingChaoZuZhi2, Unigene52554\_JingChaoZuZhi2, Unigene52638\_JingChaoZuZhi2, Unigene5298\_JingChaoZuZhi2, Unigene53131\_JingChaoZuZhi2, Unigene5335\_JingChaoZuZhi2, Unigene53484\_JingChaoZuZhi2, Unigene53500\_JingChaoZuZhi2, Unigene53751\_JingChaoZuZhi2, Unigene54081\_JingChaoZuZhi2, Unigene54918\_JingChaoZuZhi2, Unigene54974\_JingChaoZuZhi2, Unigene55009\_JingChaoZuZhi2, Unigene55019\_JingChaoZuZhi2, Unigene55239\_JingChaoZuZhi2, Unigene55326\_JingChaoZuZhi2, Unigene55385\_JingChaoZuZhi2, Unigene55839\_JingChaoZuZhi2, Unigene56089\_JingChaoZuZhi2, Unigene56421\_JingChaoZuZhi2, Unigene56560\_JingChaoZuZhi2, Unigene56660\_JingChaoZuZhi2, Unigene57240\_JingChaoZuZhi2, Unigene57488\_JingChaoZuZhi2, Unigene57597\_JingChaoZuZhi2, Unigene5809\_JingChaoZuZhi2, Unigene58169\_JingChaoZuZhi2, Unigene5823\_JingChaoZuZhi2, Unigene58474\_JingChaoZuZhi2, Unigene58675\_JingChaoZuZhi2, Unigene5896\_JingChaoZuZhi2, Unigene59101\_JingChaoZuZhi2, Unigene59265\_JingChaoZuZhi2, Unigene59336\_JingChaoZuZhi2, Unigene59433\_JingChaoZuZhi2, Unigene59480\_JingChaoZuZhi2, Unigene59684\_JingChaoZuZhi2, Unigene60121\_JingChaoZuZhi2, Unigene6028\_JingChaoZuZhi2, Unigene60551\_JingChaoZuZhi2, Unigene60586\_JingChaoZuZhi2, Unigene60607\_JingChaoZuZhi2, Unigene60654\_JingChaoZuZhi2, Unigene6072\_JingChaoZuZhi2, Unigene60930\_JingChaoZuZhi2, Unigene60990\_JingChaoZuZhi2, Unigene61342\_JingChaoZuZhi2, Unigene61474\_JingChaoZuZhi2, Unigene6158\_JingChaoZuZhi2, Unigene6171\_JingChaoZuZhi2, Unigene61879\_JingChaoZuZhi2, Unigene61931\_JingChaoZuZhi2, Unigene62100\_JingChaoZuZhi2, Unigene6215\_JingChaoZuZhi2, Unigene62257\_JingChaoZuZhi2, Unigene6233\_JingChaoZuZhi2, Unigene62606\_JingChaoZuZhi2, Unigene62695\_JingChaoZuZhi2, Unigene63052\_JingChaoZuZhi2, Unigene63145\_JingChaoZuZhi2, Unigene63384\_JingChaoZuZhi2, Unigene63424\_JingChaoZuZhi2, Unigene63584\_JingChaoZuZhi2, Unigene63604\_JingChaoZuZhi2, Unigene63615\_JingChaoZuZhi2, Unigene63799\_JingChaoZuZhi2, Unigene63812\_JingChaoZuZhi2, Unigene63873\_JingChaoZuZhi2, Unigene63925\_JingChaoZuZhi2, Unigene64164\_JingChaoZuZhi2, Unigene64208\_JingChaoZuZhi2, Unigene6438\_JingChaoZuZhi2, Unigene64452\_JingChaoZuZhi2, Unigene64458\_JingChaoZuZhi2, Unigene64650\_JingChaoZuZhi2, Unigene64692\_JingChaoZuZhi2, Unigene64830\_JingChaoZuZhi2, Unigene64843\_JingChaoZuZhi2, Unigene6493\_JingChaoZuZhi2, Unigene65022\_JingChaoZuZhi2, Unigene65129\_JingChaoZuZhi2, Unigene65150\_JingChaoZuZhi2, Unigene65187\_JingChaoZuZhi2, Unigene65326\_JingChaoZuZhi2, Unigene65375\_JingChaoZuZhi2, Unigene6539\_JingChaoZuZhi2, Unigene65591\_JingChaoZuZhi2, Unigene65773\_JingChaoZuZhi2, Unigene6605\_JingChaoZuZhi2, Unigene66087\_JingChaoZuZhi2, Unigene66253\_JingChaoZuZhi2, Unigene6651\_JingChaoZuZhi2, Unigene66559\_JingChaoZuZhi2, Unigene66575\_JingChaoZuZhi2, Unigene66590\_JingChaoZuZhi2, Unigene6676\_JingChaoZuZhi2, Unigene66813\_JingChaoZuZhi2, Unigene6688\_JingChaoZuZhi2, Unigene66986\_JingChaoZuZhi2, Unigene67005\_JingChaoZuZhi2, Unigene67063\_JingChaoZuZhi2, Unigene67070\_JingChaoZuZhi2, Unigene67249\_JingChaoZuZhi2, Unigene67279\_JingChaoZuZhi2, Unigene6728\_JingChaoZuZhi2, Unigene67328\_JingChaoZuZhi2, Unigene67367\_JingChaoZuZhi2, Unigene67572\_JingChaoZuZhi2, Unigene67655\_JingChaoZuZhi2, Unigene67777\_JingChaoZuZhi2, Unigene67958\_JingChaoZuZhi2, Unigene6796\_JingChaoZuZhi2, Unigene6797\_JingChaoZuZhi2, Unigene68108\_JingChaoZuZhi2, Unigene68139\_JingChaoZuZhi2, Unigene68151\_JingChaoZuZhi2, Unigene68159\_JingChaoZuZhi2, Unigene6815\_JingChaoZuZhi2, Unigene6828\_JingChaoZuZhi2, Unigene68527\_JingChaoZuZhi2, Unigene68672\_JingChaoZuZhi2, Unigene6872\_JingChaoZuZhi2, Unigene69049\_JingChaoZuZhi2, Unigene69092\_JingChaoZuZhi2, Unigene69141\_JingChaoZuZhi2, Unigene6914\_JingChaoZuZhi2, Unigene69221\_JingChaoZuZhi2, Unigene69386\_JingChaoZuZhi2, Unigene69402\_JingChaoZuZhi2, Unigene69562\_JingChaoZuZhi2, Unigene69775\_JingChaoZuZhi2, Unigene70045\_JingChaoZuZhi2, Unigene70063\_JingChaoZuZhi2, Unigene7016\_JingChaoZuZhi2, Unigene70329\_JingChaoZuZhi2, Unigene7032\_JingChaoZuZhi2, Unigene70549\_JingChaoZuZhi2, Unigene70723\_JingChaoZuZhi2, Unigene70970\_JingChaoZuZhi2, Unigene71043\_JingChaoZuZhi2, Unigene7116\_JingChaoZuZhi2, Unigene7118\_JingChaoZuZhi2, Unigene71213\_JingChaoZuZhi2, Unigene71241\_JingChaoZuZhi2, Unigene71385\_JingChaoZuZhi2, Unigene7151\_JingChaoZuZhi2, Unigene71546\_JingChaoZuZhi2, Unigene71774\_JingChaoZuZhi2, Unigene71808\_JingChaoZuZhi2, Unigene7206\_JingChaoZuZhi2, Unigene72098\_JingChaoZuZhi2, Unigene72136\_JingChaoZuZhi2, Unigene7219\_JingChaoZuZhi2, Unigene72249\_JingChaoZuZhi2, Unigene72334\_JingChaoZuZhi2, Unigene72351\_JingChaoZuZhi2, Unigene72503\_JingChaoZuZhi2, Unigene7268\_JingChaoZuZhi2, Unigene72708\_JingChaoZuZhi2, Unigene72832\_JingChaoZuZhi2, Unigene73257\_JingChaoZuZhi2, Unigene7326\_JingChaoZuZhi2, Unigene73446\_JingChaoZuZhi2, Unigene73475\_JingChaoZuZhi2, Unigene73550\_JingChaoZuZhi2, Unigene73606\_JingChaoZuZhi2, Unigene73620\_JingChaoZuZhi2, Unigene73817\_JingChaoZuZhi2, Unigene73887\_JingChaoZuZhi2, Unigene73983\_JingChaoZuZhi2, Unigene74050\_JingChaoZuZhi2, Unigene741\_JingChaoZuZhi2, Unigene74221\_JingChaoZuZhi2, Unigene74259\_JingChaoZuZhi2, Unigene74298\_JingChaoZuZhi2, Unigene74474\_JingChaoZuZhi2, Unigene74475\_JingChaoZuZhi2, Unigene74505\_JingChaoZuZhi2, Unigene74594\_JingChaoZuZhi2, Unigene74719\_JingChaoZuZhi2, Unigene74730\_JingChaoZuZhi2, Unigene74854\_JingChaoZuZhi2, Unigene7489\_JingChaoZuZhi2, Unigene75009\_JingChaoZuZhi2, Unigene7508\_JingChaoZuZhi2, Unigene75112\_JingChaoZuZhi2, Unigene75119\_JingChaoZuZhi2, Unigene75167\_JingChaoZuZhi2, Unigene75491\_JingChaoZuZhi2, Unigene75577\_JingChaoZuZhi2, Unigene75902\_JingChaoZuZhi2, Unigene75946\_JingChaoZuZhi2, Unigene75989\_JingChaoZuZhi2, Unigene76002\_JingChaoZuZhi2, Unigene76069\_JingChaoZuZhi2, Unigene76111\_JingChaoZuZhi2, Unigene76182\_JingChaoZuZhi2, Unigene76269\_JingChaoZuZhi2, Unigene76302\_JingChaoZuZhi2, Unigene7634\_JingChaoZuZhi2, Unigene76480\_JingChaoZuZhi2, Unigene76515\_JingChaoZuZhi2, Unigene7663\_JingChaoZuZhi2, Unigene76776\_JingChaoZuZhi2, Unigene76785\_JingChaoZuZhi2, Unigene76819\_JingChaoZuZhi2, Unigene76871\_JingChaoZuZhi2, Unigene77406\_JingChaoZuZhi2, Unigene77413\_JingChaoZuZhi2, Unigene77510\_JingChaoZuZhi2, Unigene77520\_JingChaoZuZhi2, Unigene77712\_JingChaoZuZhi2, Unigene7794\_JingChaoZuZhi2, Unigene77954\_JingChaoZuZhi2, Unigene77955\_JingChaoZuZhi2, Unigene78009\_JingChaoZuZhi2, Unigene78288\_JingChaoZuZhi2, Unigene78325\_JingChaoZuZhi2, Unigene78361\_JingChaoZuZhi2, Unigene78410\_JingChaoZuZhi2, Unigene7850\_JingChaoZuZhi2, Unigene78521\_JingChaoZuZhi2, Unigene78555\_JingChaoZuZhi2, Unigene78636\_JingChaoZuZhi2, Unigene78758\_JingChaoZuZhi2, Unigene79058\_JingChaoZuZhi2, Unigene79118\_JingChaoZuZhi2, Unigene7911\_JingChaoZuZhi2, Unigene79141\_JingChaoZuZhi2, Unigene79166\_JingChaoZuZhi2, Unigene79186\_JingChaoZuZhi2, Unigene79358\_JingChaoZuZhi2, Unigene79411\_JingChaoZuZhi2, Unigene79433\_JingChaoZuZhi2, Unigene79443\_JingChaoZuZhi2, Unigene79653\_JingChaoZuZhi2, Unigene79726\_JingChaoZuZhi2, Unigene7977\_JingChaoZuZhi2, Unigene79789\_JingChaoZuZhi2, Unigene79824\_JingChaoZuZhi2, Unigene7985\_JingChaoZuZhi2, Unigene79860\_JingChaoZuZhi2, Unigene79864\_JingChaoZuZhi2, Unigene79871\_JingChaoZuZhi2, Unigene7987\_JingChaoZuZhi2, Unigene79882\_JingChaoZuZhi2, Unigene79940\_JingChaoZuZhi2, Unigene80095\_JingChaoZuZhi2, Unigene80113\_JingChaoZuZhi2, Unigene80143\_JingChaoZuZhi2, Unigene80212\_JingChaoZuZhi2, Unigene80331\_JingChaoZuZhi2, Unigene80403\_JingChaoZuZhi2, Unigene80619\_JingChaoZuZhi2, Unigene80893\_JingChaoZuZhi2, Unigene80895\_JingChaoZuZhi2, Unigene80980\_JingChaoZuZhi2, Unigene8099\_JingChaoZuZhi2, Unigene81145\_JingChaoZuZhi2, Unigene81167\_JingChaoZuZhi2, Unigene81253\_JingChaoZuZhi2, Unigene81271\_JingChaoZuZhi2, Unigene81327\_JingChaoZuZhi2, Unigene8133\_JingChaoZuZhi2, Unigene81372\_JingChaoZuZhi2, Unigene81434\_JingChaoZuZhi2, Unigene81440\_JingChaoZuZhi2, Unigene81443\_JingChaoZuZhi2, Unigene81450\_JingChaoZuZhi2, Unigene81561\_JingChaoZuZhi2, Unigene81599\_JingChaoZuZhi2, Unigene81686\_JingChaoZuZhi2, Unigene81757\_JingChaoZuZhi2, Unigene81803\_JingChaoZuZhi2, Unigene81825\_JingChaoZuZhi2, Unigene8191\_JingChaoZuZhi2, Unigene82047\_JingChaoZuZhi2, Unigene82070\_JingChaoZuZhi2, Unigene82111\_JingChaoZuZhi2, Unigene82215\_JingChaoZuZhi2, Unigene8237\_JingChaoZuZhi2, Unigene82389\_JingChaoZuZhi2, Unigene82521\_JingChaoZuZhi2, Unigene82578\_JingChaoZuZhi2, Unigene82688\_JingChaoZuZhi2, Unigene82760\_JingChaoZuZhi2, Unigene82921\_JingChaoZuZhi2, Unigene83079\_JingChaoZuZhi2, Unigene83214\_JingChaoZuZhi2, Unigene83308\_JingChaoZuZhi2, Unigene83433\_JingChaoZuZhi2, Unigene83583\_JingChaoZuZhi2, Unigene83766\_JingChaoZuZhi2, Unigene83782\_JingChaoZuZhi2, Unigene83825\_JingChaoZuZhi2, Unigene8392\_JingChaoZuZhi2, Unigene8406\_JingChaoZuZhi2, Unigene84076\_JingChaoZuZhi2, Unigene84109\_JingChaoZuZhi2, Unigene84152\_JingChaoZuZhi2, Unigene84168\_JingChaoZuZhi2, Unigene841\_JingChaoZuZhi2, Unigene84212\_JingChaoZuZhi2, Unigene84352\_JingChaoZuZhi2, Unigene8443\_JingChaoZuZhi2, Unigene84533\_JingChaoZuZhi2, Unigene84566\_JingChaoZuZhi2, Unigene84748\_JingChaoZuZhi2, Unigene84760\_JingChaoZuZhi2, Unigene84924\_JingChaoZuZhi2, Unigene84945\_JingChaoZuZhi2, Unigene84993\_JingChaoZuZhi2, Unigene851\_JingChaoZuZhi2, Unigene85314\_JingChaoZuZhi2, Unigene85770\_JingChaoZuZhi2, Unigene85902\_JingChaoZuZhi2, Unigene86020\_JingChaoZuZhi2, Unigene86091\_JingChaoZuZhi2, Unigene86145\_JingChaoZuZhi2, Unigene86206\_JingChaoZuZhi2, Unigene8621\_JingChaoZuZhi2, Unigene86259\_JingChaoZuZhi2, Unigene86349\_JingChaoZuZhi2, Unigene86506\_JingChaoZuZhi2, Unigene86526\_JingChaoZuZhi2, Unigene86546\_JingChaoZuZhi2, Unigene86579\_JingChaoZuZhi2, Unigene8658\_JingChaoZuZhi2, Unigene8661\_JingChaoZuZhi2, Unigene86713\_JingChaoZuZhi2, Unigene86741\_JingChaoZuZhi2, Unigene86899\_JingChaoZuZhi2, Unigene87082\_JingChaoZuZhi2, Unigene8709\_JingChaoZuZhi2, Unigene87203\_JingChaoZuZhi2, Unigene87254\_JingChaoZuZhi2, Unigene87256\_JingChaoZuZhi2, Unigene87305\_JingChaoZuZhi2, Unigene87332\_JingChaoZuZhi2, Unigene87362\_JingChaoZuZhi2, Unigene87365\_JingChaoZuZhi2, Unigene87459\_JingChaoZuZhi2, Unigene87478\_JingChaoZuZhi2, Unigene87488\_JingChaoZuZhi2, Unigene87533\_JingChaoZuZhi2, Unigene87774\_JingChaoZuZhi2, Unigene87851\_JingChaoZuZhi2, Unigene87853\_JingChaoZuZhi2, Unigene87946\_JingChaoZuZhi2, Unigene88059\_JingChaoZuZhi2, Unigene88068\_JingChaoZuZhi2, Unigene88090\_JingChaoZuZhi2, Unigene88215\_JingChaoZuZhi2, Unigene88307\_JingChaoZuZhi2, Unigene8833\_JingChaoZuZhi2, Unigene88400\_JingChaoZuZhi2, Unigene88406\_JingChaoZuZhi2, Unigene88442\_JingChaoZuZhi2, Unigene88470\_JingChaoZuZhi2, Unigene88484\_JingChaoZuZhi2, Unigene88608\_JingChaoZuZhi2, Unigene88683\_JingChaoZuZhi2, Unigene88738\_JingChaoZuZhi2, Unigene88741\_JingChaoZuZhi2, Unigene88778\_JingChaoZuZhi2, Unigene88779\_JingChaoZuZhi2, Unigene88781\_JingChaoZuZhi2, Unigene88797\_JingChaoZuZhi2, Unigene88830\_JingChaoZuZhi2, Unigene88837\_JingChaoZuZhi2, Unigene88887\_JingChaoZuZhi2, Unigene88945\_JingChaoZuZhi2, Unigene88979\_JingChaoZuZhi2, Unigene88988\_JingChaoZuZhi2, Unigene89013\_JingChaoZuZhi2, Unigene8907\_JingChaoZuZhi2, Unigene89093\_JingChaoZuZhi2, Unigene89121\_JingChaoZuZhi2, Unigene89323\_JingChaoZuZhi2, Unigene89337\_JingChaoZuZhi2, Unigene89352\_JingChaoZuZhi2, Unigene89367\_JingChaoZuZhi2, Unigene89541\_JingChaoZuZhi2, Unigene89606\_JingChaoZuZhi2, Unigene89746\_JingChaoZuZhi2, Unigene89769\_JingChaoZuZhi2, Unigene89834\_JingChaoZuZhi2, Unigene89867\_JingChaoZuZhi2, Unigene89956\_JingChaoZuZhi2, Unigene90027\_JingChaoZuZhi2, Unigene90038\_JingChaoZuZhi2, Unigene90066\_JingChaoZuZhi2, Unigene90071\_JingChaoZuZhi2, Unigene90209\_JingChaoZuZhi2, Unigene90341\_JingChaoZuZhi2, Unigene9050\_JingChaoZuZhi2, Unigene90564\_JingChaoZuZhi2, Unigene90714\_JingChaoZuZhi2, Unigene90715\_JingChaoZuZhi2, Unigene90747\_JingChaoZuZhi2, Unigene90779\_JingChaoZuZhi2, Unigene90923\_JingChaoZuZhi2, Unigene90953\_JingChaoZuZhi2, Unigene90995\_JingChaoZuZhi2, Unigene91099\_JingChaoZuZhi2, Unigene91148\_JingChaoZuZhi2, Unigene91179\_JingChaoZuZhi2, Unigene91233\_JingChaoZuZhi2, Unigene91390\_JingChaoZuZhi2, Unigene91456\_JingChaoZuZhi2, Unigene91475\_JingChaoZuZhi2, Unigene91487\_JingChaoZuZhi2, Unigene91529\_JingChaoZuZhi2, Unigene91538\_JingChaoZuZhi2, Unigene91553\_JingChaoZuZhi2, Unigene91848\_JingChaoZuZhi2, Unigene91971\_JingChaoZuZhi2, Unigene92112\_JingChaoZuZhi2, Unigene92315\_JingChaoZuZhi2, Unigene92346\_JingChaoZuZhi2, Unigene92372\_JingChaoZuZhi2, Unigene92394\_JingChaoZuZhi2, Unigene92395\_JingChaoZuZhi2, Unigene92580\_JingChaoZuZhi2, Unigene92681\_JingChaoZuZhi2, Unigene92700\_JingChaoZuZhi2, Unigene92737\_JingChaoZuZhi2, Unigene92780\_JingChaoZuZhi2, Unigene92832\_JingChaoZuZhi2, Unigene92838\_JingChaoZuZhi2, Unigene92951\_JingChaoZuZhi2, Unigene93073\_JingChaoZuZhi2, Unigene93244\_JingChaoZuZhi2, Unigene93246\_JingChaoZuZhi2, Unigene93271\_JingChaoZuZhi2, Unigene93552\_JingChaoZuZhi2, Unigene93562\_JingChaoZuZhi2, Unigene93573\_JingChaoZuZhi2, Unigene93592\_JingChaoZuZhi2, Unigene93610\_JingChaoZuZhi2, Unigene93742\_JingChaoZuZhi2, Unigene93780\_JingChaoZuZhi2, Unigene93782\_JingChaoZuZhi2, Unigene93807\_JingChaoZuZhi2, Unigene93853\_JingChaoZuZhi2, Unigene9389\_JingChaoZuZhi2, Unigene93979\_JingChaoZuZhi2, Unigene94083\_JingChaoZuZhi2, Unigene94120\_JingChaoZuZhi2, Unigene94129\_JingChaoZuZhi2, Unigene94134\_JingChaoZuZhi2, Unigene94208\_JingChaoZuZhi2, Unigene94404\_JingChaoZuZhi2, Unigene94471\_JingChaoZuZhi2, Unigene94520\_JingChaoZuZhi2, Unigene94544\_JingChaoZuZhi2, Unigene94578\_JingChaoZuZhi2, Unigene94584\_JingChaoZuZhi2, Unigene9461\_JingChaoZuZhi2, Unigene94685\_JingChaoZuZhi2, Unigene94687\_JingChaoZuZhi2, Unigene94891\_JingChaoZuZhi2, Unigene9490\_JingChaoZuZhi2, Unigene94922\_JingChaoZuZhi2, Unigene94950\_JingChaoZuZhi2, Unigene94968\_JingChaoZuZhi2, Unigene95088\_JingChaoZuZhi2, Unigene95098\_JingChaoZuZhi2, Unigene95119\_JingChaoZuZhi2, Unigene95169\_JingChaoZuZhi2, Unigene95203\_JingChaoZuZhi2, Unigene95371\_JingChaoZuZhi2, Unigene9540\_JingChaoZuZhi2, Unigene95521\_JingChaoZuZhi2, Unigene95589\_JingChaoZuZhi2, Unigene95633\_JingChaoZuZhi2, Unigene95677\_JingChaoZuZhi2, Unigene95686\_JingChaoZuZhi2, Unigene95812\_JingChaoZuZhi2, Unigene95875\_JingChaoZuZhi2, Unigene96193\_JingChaoZuZhi2, Unigene96294\_JingChaoZuZhi2, Unigene9688\_JingChaoZuZhi2, Unigene9743\_JingChaoZuZhi2, Unigene9807\_JingChaoZuZhi2, Unigene9833\_JingChaoZuZhi2, Unigene9902\_JingChaoZuZhi2, Unigene9988\_JingChaoZuZhi2, Unigene9992\_JingChaoZuZhi2, Unigene9\_JingChaoZuZhi2 |
| 9 | Olfactory transduction | Unigene10017\_JingChaoZuZhi2, Unigene10084\_JingChaoZuZhi2, Unigene1008\_JingChaoZuZhi2, Unigene10147\_JingChaoZuZhi2, Unigene1022\_JingChaoZuZhi2, Unigene10347\_JingChaoZuZhi2, Unigene10414\_JingChaoZuZhi2, Unigene10558\_JingChaoZuZhi2, Unigene1065\_JingChaoZuZhi2, Unigene10747\_JingChaoZuZhi2, Unigene10843\_JingChaoZuZhi2, Unigene10884\_JingChaoZuZhi2, Unigene1089\_JingChaoZuZhi2, Unigene11001\_JingChaoZuZhi2, Unigene11135\_JingChaoZuZhi2, Unigene11138\_JingChaoZuZhi2, Unigene11338\_JingChaoZuZhi2, Unigene11394\_JingChaoZuZhi2, Unigene11499\_JingChaoZuZhi2, Unigene11605\_JingChaoZuZhi2, Unigene11660\_JingChaoZuZhi2, Unigene11745\_JingChaoZuZhi2, Unigene11758\_JingChaoZuZhi2, Unigene11762\_JingChaoZuZhi2, Unigene11766\_JingChaoZuZhi2, Unigene1196\_JingChaoZuZhi2, Unigene12053\_JingChaoZuZhi2, Unigene12068\_JingChaoZuZhi2, Unigene12115\_JingChaoZuZhi2, Unigene12370\_JingChaoZuZhi2, Unigene12381\_JingChaoZuZhi2, Unigene1239\_JingChaoZuZhi2, Unigene12430\_JingChaoZuZhi2, Unigene1247\_JingChaoZuZhi2, Unigene12495\_JingChaoZuZhi2, Unigene12519\_JingChaoZuZhi2, Unigene12622\_JingChaoZuZhi2, Unigene12694\_JingChaoZuZhi2, Unigene1273\_JingChaoZuZhi2, Unigene12757\_JingChaoZuZhi2, Unigene12787\_JingChaoZuZhi2, Unigene12898\_JingChaoZuZhi2, Unigene13224\_JingChaoZuZhi2, Unigene13241\_JingChaoZuZhi2, Unigene13294\_JingChaoZuZhi2, Unigene13379\_JingChaoZuZhi2, Unigene13529\_JingChaoZuZhi2, Unigene13561\_JingChaoZuZhi2, Unigene13573\_JingChaoZuZhi2, Unigene13684\_JingChaoZuZhi2, Unigene1368\_JingChaoZuZhi2, Unigene1373\_JingChaoZuZhi2, Unigene13771\_JingChaoZuZhi2, Unigene13798\_JingChaoZuZhi2, Unigene13815\_JingChaoZuZhi2, Unigene13859\_JingChaoZuZhi2, Unigene1385\_JingChaoZuZhi2, Unigene13919\_JingChaoZuZhi2, Unigene14053\_JingChaoZuZhi2, Unigene1417\_JingChaoZuZhi2, Unigene142\_JingChaoZuZhi2, Unigene14335\_JingChaoZuZhi2, Unigene14387\_JingChaoZuZhi2, Unigene1440\_JingChaoZuZhi2, Unigene14461\_JingChaoZuZhi2, Unigene1447\_JingChaoZuZhi2, Unigene14507\_JingChaoZuZhi2, Unigene14531\_JingChaoZuZhi2, Unigene1475\_JingChaoZuZhi2, Unigene1480\_JingChaoZuZhi2, Unigene14900\_JingChaoZuZhi2, Unigene14966\_JingChaoZuZhi2, Unigene14994\_JingChaoZuZhi2, Unigene15121\_JingChaoZuZhi2, Unigene15163\_JingChaoZuZhi2, Unigene15196\_JingChaoZuZhi2, Unigene15226\_JingChaoZuZhi2, Unigene15374\_JingChaoZuZhi2, Unigene15525\_JingChaoZuZhi2, Unigene15744\_JingChaoZuZhi2, Unigene15782\_JingChaoZuZhi2, Unigene1579\_JingChaoZuZhi2, Unigene15832\_JingChaoZuZhi2, Unigene15842\_JingChaoZuZhi2, Unigene15928\_JingChaoZuZhi2, Unigene15932\_JingChaoZuZhi2, Unigene15935\_JingChaoZuZhi2, Unigene16041\_JingChaoZuZhi2, Unigene1605\_JingChaoZuZhi2, Unigene16068\_JingChaoZuZhi2, Unigene16205\_JingChaoZuZhi2, Unigene16237\_JingChaoZuZhi2, Unigene16257\_JingChaoZuZhi2, Unigene16274\_JingChaoZuZhi2, Unigene16315\_JingChaoZuZhi2, Unigene16339\_JingChaoZuZhi2, Unigene16454\_JingChaoZuZhi2, Unigene16497\_JingChaoZuZhi2, Unigene1654\_JingChaoZuZhi2, Unigene16566\_JingChaoZuZhi2, Unigene16637\_JingChaoZuZhi2, Unigene16672\_JingChaoZuZhi2, Unigene16770\_JingChaoZuZhi2, Unigene16803\_JingChaoZuZhi2, Unigene16810\_JingChaoZuZhi2, Unigene17167\_JingChaoZuZhi2, Unigene17200\_JingChaoZuZhi2, Unigene17232\_JingChaoZuZhi2, Unigene1740\_JingChaoZuZhi2, Unigene1742\_JingChaoZuZhi2, Unigene17509\_JingChaoZuZhi2, Unigene17557\_JingChaoZuZhi2, Unigene17577\_JingChaoZuZhi2, Unigene17713\_JingChaoZuZhi2, Unigene17850\_JingChaoZuZhi2, Unigene18054\_JingChaoZuZhi2, Unigene18069\_JingChaoZuZhi2, Unigene18207\_JingChaoZuZhi2, Unigene18217\_JingChaoZuZhi2, Unigene18224\_JingChaoZuZhi2, Unigene18301\_JingChaoZuZhi2, Unigene18307\_JingChaoZuZhi2, Unigene18697\_JingChaoZuZhi2, Unigene1874\_JingChaoZuZhi2, Unigene18901\_JingChaoZuZhi2, Unigene18911\_JingChaoZuZhi2, Unigene18922\_JingChaoZuZhi2, Unigene1892\_JingChaoZuZhi2, Unigene18961\_JingChaoZuZhi2, Unigene19166\_JingChaoZuZhi2, Unigene19322\_JingChaoZuZhi2, Unigene19421\_JingChaoZuZhi2, Unigene19713\_JingChaoZuZhi2, Unigene19746\_JingChaoZuZhi2, Unigene19757\_JingChaoZuZhi2, Unigene1975\_JingChaoZuZhi2, Unigene1977\_JingChaoZuZhi2, Unigene1991\_JingChaoZuZhi2, Unigene20003\_JingChaoZuZhi2, Unigene20015\_JingChaoZuZhi2, Unigene20029\_JingChaoZuZhi2, Unigene2003\_JingChaoZuZhi2, Unigene20056\_JingChaoZuZhi2, Unigene20136\_JingChaoZuZhi2, Unigene2015\_JingChaoZuZhi2, Unigene20267\_JingChaoZuZhi2, Unigene20353\_JingChaoZuZhi2, Unigene20778\_JingChaoZuZhi2, Unigene20860\_JingChaoZuZhi2, Unigene2086\_JingChaoZuZhi2, Unigene20900\_JingChaoZuZhi2, Unigene21040\_JingChaoZuZhi2, Unigene21056\_JingChaoZuZhi2, Unigene21197\_JingChaoZuZhi2, Unigene21576\_JingChaoZuZhi2, Unigene2160\_JingChaoZuZhi2, Unigene21646\_JingChaoZuZhi2, Unigene21799\_JingChaoZuZhi2, Unigene21802\_JingChaoZuZhi2, Unigene21805\_JingChaoZuZhi2, Unigene21815\_JingChaoZuZhi2, Unigene21913\_JingChaoZuZhi2, Unigene21927\_JingChaoZuZhi2, Unigene21945\_JingChaoZuZhi2, Unigene21981\_JingChaoZuZhi2, Unigene22037\_JingChaoZuZhi2, Unigene22051\_JingChaoZuZhi2, Unigene22059\_JingChaoZuZhi2, Unigene22136\_JingChaoZuZhi2, Unigene22162\_JingChaoZuZhi2, Unigene22219\_JingChaoZuZhi2, Unigene22268\_JingChaoZuZhi2, Unigene22305\_JingChaoZuZhi2, Unigene22389\_JingChaoZuZhi2, Unigene22450\_JingChaoZuZhi2, Unigene22452\_JingChaoZuZhi2, Unigene22468\_JingChaoZuZhi2, Unigene2246\_JingChaoZuZhi2, Unigene22507\_JingChaoZuZhi2, Unigene22671\_JingChaoZuZhi2, Unigene22698\_JingChaoZuZhi2, Unigene22757\_JingChaoZuZhi2, Unigene2331\_JingChaoZuZhi2, Unigene23339\_JingChaoZuZhi2, Unigene23772\_JingChaoZuZhi2, Unigene23885\_JingChaoZuZhi2, Unigene24820\_JingChaoZuZhi2, Unigene25338\_JingChaoZuZhi2, Unigene25554\_JingChaoZuZhi2, Unigene25688\_JingChaoZuZhi2, Unigene26054\_JingChaoZuZhi2, Unigene26389\_JingChaoZuZhi2, Unigene26407\_JingChaoZuZhi2, Unigene26700\_JingChaoZuZhi2, Unigene26878\_JingChaoZuZhi2, Unigene26911\_JingChaoZuZhi2, Unigene26919\_JingChaoZuZhi2, Unigene2701\_JingChaoZuZhi2, Unigene27045\_JingChaoZuZhi2, Unigene27141\_JingChaoZuZhi2, Unigene27512\_JingChaoZuZhi2, Unigene27772\_JingChaoZuZhi2, Unigene28336\_JingChaoZuZhi2, Unigene28427\_JingChaoZuZhi2, Unigene28517\_JingChaoZuZhi2, Unigene2903\_JingChaoZuZhi2, Unigene29084\_JingChaoZuZhi2, Unigene29133\_JingChaoZuZhi2, Unigene29180\_JingChaoZuZhi2, Unigene30207\_JingChaoZuZhi2, Unigene30409\_JingChaoZuZhi2, Unigene30624\_JingChaoZuZhi2, Unigene30738\_JingChaoZuZhi2, Unigene30762\_JingChaoZuZhi2, Unigene31186\_JingChaoZuZhi2, Unigene31406\_JingChaoZuZhi2, Unigene31790\_JingChaoZuZhi2, Unigene31857\_JingChaoZuZhi2, Unigene31910\_JingChaoZuZhi2, Unigene3234\_JingChaoZuZhi2, Unigene33069\_JingChaoZuZhi2, Unigene3329\_JingChaoZuZhi2, Unigene3330\_JingChaoZuZhi2, Unigene33373\_JingChaoZuZhi2, Unigene33839\_JingChaoZuZhi2, Unigene34463\_JingChaoZuZhi2, Unigene3464\_JingChaoZuZhi2, Unigene34711\_JingChaoZuZhi2, Unigene3478\_JingChaoZuZhi2, Unigene34802\_JingChaoZuZhi2, Unigene34892\_JingChaoZuZhi2, Unigene34894\_JingChaoZuZhi2, Unigene34988\_JingChaoZuZhi2, Unigene35364\_JingChaoZuZhi2, Unigene3588\_JingChaoZuZhi2, Unigene35977\_JingChaoZuZhi2, Unigene3613\_JingChaoZuZhi2, Unigene36382\_JingChaoZuZhi2, Unigene36684\_JingChaoZuZhi2, Unigene36760\_JingChaoZuZhi2, Unigene3688\_JingChaoZuZhi2, Unigene36901\_JingChaoZuZhi2, Unigene36999\_JingChaoZuZhi2, Unigene37051\_JingChaoZuZhi2, Unigene37082\_JingChaoZuZhi2, Unigene37084\_JingChaoZuZhi2, Unigene37595\_JingChaoZuZhi2, Unigene3759\_JingChaoZuZhi2, Unigene3774\_JingChaoZuZhi2, Unigene37817\_JingChaoZuZhi2, Unigene38036\_JingChaoZuZhi2, Unigene38317\_JingChaoZuZhi2, Unigene38321\_JingChaoZuZhi2, Unigene38336\_JingChaoZuZhi2, Unigene38468\_JingChaoZuZhi2, Unigene38502\_JingChaoZuZhi2, Unigene38651\_JingChaoZuZhi2, Unigene38801\_JingChaoZuZhi2, Unigene39029\_JingChaoZuZhi2, Unigene39316\_JingChaoZuZhi2, Unigene39392\_JingChaoZuZhi2, Unigene39457\_JingChaoZuZhi2, Unigene39594\_JingChaoZuZhi2, Unigene397\_JingChaoZuZhi2, Unigene39917\_JingChaoZuZhi2, Unigene39973\_JingChaoZuZhi2, Unigene40241\_JingChaoZuZhi2, Unigene40281\_JingChaoZuZhi2, Unigene40666\_JingChaoZuZhi2, Unigene40893\_JingChaoZuZhi2, Unigene41200\_JingChaoZuZhi2, Unigene41407\_JingChaoZuZhi2, Unigene41430\_JingChaoZuZhi2, Unigene4144\_JingChaoZuZhi2, Unigene41542\_JingChaoZuZhi2, Unigene416\_JingChaoZuZhi2, Unigene41723\_JingChaoZuZhi2, Unigene41781\_JingChaoZuZhi2, Unigene41880\_JingChaoZuZhi2, Unigene42272\_JingChaoZuZhi2, Unigene42447\_JingChaoZuZhi2, Unigene42522\_JingChaoZuZhi2, Unigene42566\_JingChaoZuZhi2, Unigene42682\_JingChaoZuZhi2, Unigene42858\_JingChaoZuZhi2, Unigene42863\_JingChaoZuZhi2, Unigene43060\_JingChaoZuZhi2, Unigene43131\_JingChaoZuZhi2, Unigene43227\_JingChaoZuZhi2, Unigene43298\_JingChaoZuZhi2, Unigene43846\_JingChaoZuZhi2, Unigene44192\_JingChaoZuZhi2, Unigene44224\_JingChaoZuZhi2, Unigene44286\_JingChaoZuZhi2, Unigene44364\_JingChaoZuZhi2, Unigene4453\_JingChaoZuZhi2, Unigene4459\_JingChaoZuZhi2, Unigene44713\_JingChaoZuZhi2, Unigene44870\_JingChaoZuZhi2, Unigene44944\_JingChaoZuZhi2, Unigene452\_JingChaoZuZhi2, Unigene45394\_JingChaoZuZhi2, Unigene45642\_JingChaoZuZhi2, Unigene45724\_JingChaoZuZhi2, Unigene45778\_JingChaoZuZhi2, Unigene4584\_JingChaoZuZhi2, Unigene45940\_JingChaoZuZhi2, Unigene46151\_JingChaoZuZhi2, Unigene46663\_JingChaoZuZhi2, Unigene47002\_JingChaoZuZhi2, Unigene47022\_JingChaoZuZhi2, Unigene4730\_JingChaoZuZhi2, Unigene47416\_JingChaoZuZhi2, Unigene47451\_JingChaoZuZhi2, Unigene47574\_JingChaoZuZhi2, Unigene4760\_JingChaoZuZhi2, Unigene4762\_JingChaoZuZhi2, Unigene47979\_JingChaoZuZhi2, Unigene48141\_JingChaoZuZhi2, Unigene483\_JingChaoZuZhi2, Unigene48499\_JingChaoZuZhi2, Unigene4852\_JingChaoZuZhi2, Unigene48536\_JingChaoZuZhi2, Unigene48647\_JingChaoZuZhi2, Unigene49385\_JingChaoZuZhi2, Unigene49504\_JingChaoZuZhi2, Unigene49551\_JingChaoZuZhi2, Unigene49657\_JingChaoZuZhi2, Unigene50381\_JingChaoZuZhi2, Unigene50505\_JingChaoZuZhi2, Unigene50668\_JingChaoZuZhi2, Unigene50770\_JingChaoZuZhi2, Unigene50927\_JingChaoZuZhi2, Unigene51070\_JingChaoZuZhi2, Unigene5121\_JingChaoZuZhi2, Unigene51270\_JingChaoZuZhi2, Unigene51291\_JingChaoZuZhi2, Unigene51403\_JingChaoZuZhi2, Unigene51753\_JingChaoZuZhi2, Unigene52051\_JingChaoZuZhi2, Unigene52449\_JingChaoZuZhi2, Unigene52526\_JingChaoZuZhi2, Unigene5258\_JingChaoZuZhi2, Unigene5267\_JingChaoZuZhi2, Unigene52739\_JingChaoZuZhi2, Unigene52778\_JingChaoZuZhi2, Unigene52860\_JingChaoZuZhi2, Unigene54012\_JingChaoZuZhi2, Unigene54144\_JingChaoZuZhi2, Unigene54242\_JingChaoZuZhi2, Unigene54285\_JingChaoZuZhi2, Unigene54385\_JingChaoZuZhi2, Unigene54408\_JingChaoZuZhi2, Unigene54412\_JingChaoZuZhi2, Unigene54569\_JingChaoZuZhi2, Unigene54790\_JingChaoZuZhi2, Unigene54862\_JingChaoZuZhi2, Unigene54965\_JingChaoZuZhi2, Unigene55111\_JingChaoZuZhi2, Unigene55393\_JingChaoZuZhi2, Unigene55409\_JingChaoZuZhi2, Unigene55425\_JingChaoZuZhi2, Unigene55437\_JingChaoZuZhi2, Unigene55541\_JingChaoZuZhi2, Unigene55788\_JingChaoZuZhi2, Unigene5589\_JingChaoZuZhi2, Unigene55909\_JingChaoZuZhi2, Unigene55999\_JingChaoZuZhi2, Unigene56143\_JingChaoZuZhi2, Unigene56148\_JingChaoZuZhi2, Unigene56342\_JingChaoZuZhi2, Unigene56490\_JingChaoZuZhi2, Unigene56863\_JingChaoZuZhi2, Unigene56907\_JingChaoZuZhi2, Unigene57160\_JingChaoZuZhi2, Unigene57184\_JingChaoZuZhi2, Unigene57552\_JingChaoZuZhi2, Unigene57586\_JingChaoZuZhi2, Unigene57593\_JingChaoZuZhi2, Unigene58091\_JingChaoZuZhi2, Unigene5824\_JingChaoZuZhi2, Unigene58696\_JingChaoZuZhi2, Unigene586\_JingChaoZuZhi2, Unigene58914\_JingChaoZuZhi2, Unigene58945\_JingChaoZuZhi2, Unigene5895\_JingChaoZuZhi2, Unigene59083\_JingChaoZuZhi2, Unigene59118\_JingChaoZuZhi2, Unigene59166\_JingChaoZuZhi2, Unigene59465\_JingChaoZuZhi2, Unigene59626\_JingChaoZuZhi2, Unigene59909\_JingChaoZuZhi2, Unigene59921\_JingChaoZuZhi2, Unigene59964\_JingChaoZuZhi2, Unigene60028\_JingChaoZuZhi2, Unigene60151\_JingChaoZuZhi2, Unigene60187\_JingChaoZuZhi2, Unigene60359\_JingChaoZuZhi2, Unigene6042\_JingChaoZuZhi2, Unigene60543\_JingChaoZuZhi2, Unigene6056\_JingChaoZuZhi2, Unigene6072\_JingChaoZuZhi2, Unigene60800\_JingChaoZuZhi2, Unigene61107\_JingChaoZuZhi2, Unigene6120\_JingChaoZuZhi2, Unigene61736\_JingChaoZuZhi2, Unigene6179\_JingChaoZuZhi2, Unigene61845\_JingChaoZuZhi2, Unigene61855\_JingChaoZuZhi2, Unigene61887\_JingChaoZuZhi2, Unigene619\_JingChaoZuZhi2, Unigene62081\_JingChaoZuZhi2, Unigene62220\_JingChaoZuZhi2, Unigene62255\_JingChaoZuZhi2, Unigene62317\_JingChaoZuZhi2, Unigene62690\_JingChaoZuZhi2, Unigene63091\_JingChaoZuZhi2, Unigene63149\_JingChaoZuZhi2, Unigene63351\_JingChaoZuZhi2, Unigene63368\_JingChaoZuZhi2, Unigene63771\_JingChaoZuZhi2, Unigene63829\_JingChaoZuZhi2, Unigene64236\_JingChaoZuZhi2, Unigene64324\_JingChaoZuZhi2, Unigene64387\_JingChaoZuZhi2, Unigene64474\_JingChaoZuZhi2, Unigene64499\_JingChaoZuZhi2, Unigene64520\_JingChaoZuZhi2, Unigene64666\_JingChaoZuZhi2, Unigene64678\_JingChaoZuZhi2, Unigene64799\_JingChaoZuZhi2, Unigene6519\_JingChaoZuZhi2, Unigene65205\_JingChaoZuZhi2, Unigene65219\_JingChaoZuZhi2, Unigene65621\_JingChaoZuZhi2, Unigene65724\_JingChaoZuZhi2, Unigene65831\_JingChaoZuZhi2, Unigene65968\_JingChaoZuZhi2, Unigene6598\_JingChaoZuZhi2, Unigene66002\_JingChaoZuZhi2, Unigene66102\_JingChaoZuZhi2, Unigene66121\_JingChaoZuZhi2, Unigene66172\_JingChaoZuZhi2, Unigene66319\_JingChaoZuZhi2, Unigene6651\_JingChaoZuZhi2, Unigene66586\_JingChaoZuZhi2, Unigene66708\_JingChaoZuZhi2, Unigene66922\_JingChaoZuZhi2, Unigene67088\_JingChaoZuZhi2, Unigene67102\_JingChaoZuZhi2, Unigene67112\_JingChaoZuZhi2, Unigene67283\_JingChaoZuZhi2, Unigene67321\_JingChaoZuZhi2, Unigene6753\_JingChaoZuZhi2, Unigene67583\_JingChaoZuZhi2, Unigene67599\_JingChaoZuZhi2, Unigene67661\_JingChaoZuZhi2, Unigene6776\_JingChaoZuZhi2, Unigene67798\_JingChaoZuZhi2, Unigene67845\_JingChaoZuZhi2, Unigene67883\_JingChaoZuZhi2, Unigene67889\_JingChaoZuZhi2, Unigene68013\_JingChaoZuZhi2, Unigene6818\_JingChaoZuZhi2, Unigene68214\_JingChaoZuZhi2, Unigene68272\_JingChaoZuZhi2, Unigene6832\_JingChaoZuZhi2, Unigene68337\_JingChaoZuZhi2, Unigene68460\_JingChaoZuZhi2, Unigene68569\_JingChaoZuZhi2, Unigene68711\_JingChaoZuZhi2, Unigene68783\_JingChaoZuZhi2, Unigene68883\_JingChaoZuZhi2, Unigene69293\_JingChaoZuZhi2, Unigene6929\_JingChaoZuZhi2, Unigene692\_JingChaoZuZhi2, Unigene6933\_JingChaoZuZhi2, Unigene69362\_JingChaoZuZhi2, Unigene6939\_JingChaoZuZhi2, Unigene69508\_JingChaoZuZhi2, Unigene69575\_JingChaoZuZhi2, Unigene69689\_JingChaoZuZhi2, Unigene69910\_JingChaoZuZhi2, Unigene69938\_JingChaoZuZhi2, Unigene70063\_JingChaoZuZhi2, Unigene70182\_JingChaoZuZhi2, Unigene70234\_JingChaoZuZhi2, Unigene70328\_JingChaoZuZhi2, Unigene7032\_JingChaoZuZhi2, Unigene70605\_JingChaoZuZhi2, Unigene71214\_JingChaoZuZhi2, Unigene71355\_JingChaoZuZhi2, Unigene71365\_JingChaoZuZhi2, Unigene71630\_JingChaoZuZhi2, Unigene71692\_JingChaoZuZhi2, Unigene71732\_JingChaoZuZhi2, Unigene71822\_JingChaoZuZhi2, Unigene71862\_JingChaoZuZhi2, Unigene71881\_JingChaoZuZhi2, Unigene71950\_JingChaoZuZhi2, Unigene71979\_JingChaoZuZhi2, Unigene72036\_JingChaoZuZhi2, Unigene72130\_JingChaoZuZhi2, Unigene72362\_JingChaoZuZhi2, Unigene72524\_JingChaoZuZhi2, Unigene72539\_JingChaoZuZhi2, Unigene72579\_JingChaoZuZhi2, Unigene72802\_JingChaoZuZhi2, Unigene728\_JingChaoZuZhi2, Unigene72923\_JingChaoZuZhi2, Unigene72987\_JingChaoZuZhi2, Unigene72990\_JingChaoZuZhi2, Unigene73115\_JingChaoZuZhi2, Unigene73132\_JingChaoZuZhi2, Unigene73208\_JingChaoZuZhi2, Unigene73242\_JingChaoZuZhi2, Unigene73266\_JingChaoZuZhi2, Unigene73287\_JingChaoZuZhi2, Unigene73318\_JingChaoZuZhi2, Unigene73379\_JingChaoZuZhi2, Unigene73401\_JingChaoZuZhi2, Unigene73435\_JingChaoZuZhi2, Unigene736\_JingChaoZuZhi2, Unigene73926\_JingChaoZuZhi2, Unigene74163\_JingChaoZuZhi2, Unigene74166\_JingChaoZuZhi2, Unigene74288\_JingChaoZuZhi2, Unigene74402\_JingChaoZuZhi2, Unigene74421\_JingChaoZuZhi2, Unigene74441\_JingChaoZuZhi2, Unigene74606\_JingChaoZuZhi2, Unigene74607\_JingChaoZuZhi2, Unigene74701\_JingChaoZuZhi2, Unigene747\_JingChaoZuZhi2, Unigene74832\_JingChaoZuZhi2, Unigene74850\_JingChaoZuZhi2, Unigene74872\_JingChaoZuZhi2, Unigene74994\_JingChaoZuZhi2, Unigene75130\_JingChaoZuZhi2, Unigene75147\_JingChaoZuZhi2, Unigene75345\_JingChaoZuZhi2, Unigene75503\_JingChaoZuZhi2, Unigene75633\_JingChaoZuZhi2, Unigene75899\_JingChaoZuZhi2, Unigene75924\_JingChaoZuZhi2, Unigene76005\_JingChaoZuZhi2, Unigene76058\_JingChaoZuZhi2, Unigene7609\_JingChaoZuZhi2, Unigene76127\_JingChaoZuZhi2, Unigene76177\_JingChaoZuZhi2, Unigene76228\_JingChaoZuZhi2, Unigene76446\_JingChaoZuZhi2, Unigene76495\_JingChaoZuZhi2, Unigene76709\_JingChaoZuZhi2, Unigene76726\_JingChaoZuZhi2, Unigene76748\_JingChaoZuZhi2, Unigene76762\_JingChaoZuZhi2, Unigene76786\_JingChaoZuZhi2, Unigene7679\_JingChaoZuZhi2, Unigene76821\_JingChaoZuZhi2, Unigene76912\_JingChaoZuZhi2, Unigene76995\_JingChaoZuZhi2, Unigene76997\_JingChaoZuZhi2, Unigene7705\_JingChaoZuZhi2, Unigene77064\_JingChaoZuZhi2, Unigene7723\_JingChaoZuZhi2, Unigene77367\_JingChaoZuZhi2, Unigene77513\_JingChaoZuZhi2, Unigene77568\_JingChaoZuZhi2, Unigene7756\_JingChaoZuZhi2, Unigene77687\_JingChaoZuZhi2, Unigene77723\_JingChaoZuZhi2, Unigene77790\_JingChaoZuZhi2, Unigene777\_JingChaoZuZhi2, Unigene77815\_JingChaoZuZhi2, Unigene78067\_JingChaoZuZhi2, Unigene78112\_JingChaoZuZhi2, Unigene78191\_JingChaoZuZhi2, Unigene78235\_JingChaoZuZhi2, Unigene78243\_JingChaoZuZhi2, Unigene78542\_JingChaoZuZhi2, Unigene78587\_JingChaoZuZhi2, Unigene78626\_JingChaoZuZhi2, Unigene79063\_JingChaoZuZhi2, Unigene79152\_JingChaoZuZhi2, Unigene79206\_JingChaoZuZhi2, Unigene79280\_JingChaoZuZhi2, Unigene79333\_JingChaoZuZhi2, Unigene7936\_JingChaoZuZhi2, Unigene79388\_JingChaoZuZhi2, Unigene79399\_JingChaoZuZhi2, Unigene79508\_JingChaoZuZhi2, Unigene79592\_JingChaoZuZhi2, Unigene79618\_JingChaoZuZhi2, Unigene79712\_JingChaoZuZhi2, Unigene79733\_JingChaoZuZhi2, Unigene79811\_JingChaoZuZhi2, Unigene79857\_JingChaoZuZhi2, Unigene79899\_JingChaoZuZhi2, Unigene79950\_JingChaoZuZhi2, Unigene79975\_JingChaoZuZhi2, Unigene80043\_JingChaoZuZhi2, Unigene8015\_JingChaoZuZhi2, Unigene80189\_JingChaoZuZhi2, Unigene80244\_JingChaoZuZhi2, Unigene80310\_JingChaoZuZhi2, Unigene80451\_JingChaoZuZhi2, Unigene80481\_JingChaoZuZhi2, Unigene80657\_JingChaoZuZhi2, Unigene80685\_JingChaoZuZhi2, Unigene8069\_JingChaoZuZhi2, Unigene80826\_JingChaoZuZhi2, Unigene80889\_JingChaoZuZhi2, Unigene80931\_JingChaoZuZhi2, Unigene80953\_JingChaoZuZhi2, Unigene80968\_JingChaoZuZhi2, Unigene81059\_JingChaoZuZhi2, Unigene81082\_JingChaoZuZhi2, Unigene81119\_JingChaoZuZhi2, Unigene81183\_JingChaoZuZhi2, Unigene81229\_JingChaoZuZhi2, Unigene81317\_JingChaoZuZhi2, Unigene81366\_JingChaoZuZhi2, Unigene81491\_JingChaoZuZhi2, Unigene81551\_JingChaoZuZhi2, Unigene81684\_JingChaoZuZhi2, Unigene8169\_JingChaoZuZhi2, Unigene817\_JingChaoZuZhi2, Unigene8182\_JingChaoZuZhi2, Unigene81864\_JingChaoZuZhi2, Unigene81955\_JingChaoZuZhi2, Unigene82018\_JingChaoZuZhi2, Unigene82101\_JingChaoZuZhi2, Unigene82138\_JingChaoZuZhi2, Unigene82143\_JingChaoZuZhi2, Unigene8221\_JingChaoZuZhi2, Unigene8229\_JingChaoZuZhi2, Unigene82300\_JingChaoZuZhi2, Unigene82366\_JingChaoZuZhi2, Unigene82370\_JingChaoZuZhi2, Unigene82446\_JingChaoZuZhi2, Unigene82513\_JingChaoZuZhi2, Unigene82521\_JingChaoZuZhi2, Unigene82547\_JingChaoZuZhi2, Unigene82595\_JingChaoZuZhi2, Unigene82742\_JingChaoZuZhi2, Unigene82773\_JingChaoZuZhi2, Unigene82821\_JingChaoZuZhi2, Unigene82843\_JingChaoZuZhi2, Unigene82875\_JingChaoZuZhi2, Unigene82907\_JingChaoZuZhi2, Unigene82943\_JingChaoZuZhi2, Unigene83267\_JingChaoZuZhi2, Unigene83540\_JingChaoZuZhi2, Unigene83590\_JingChaoZuZhi2, Unigene83604\_JingChaoZuZhi2, Unigene83667\_JingChaoZuZhi2, Unigene83678\_JingChaoZuZhi2, Unigene83754\_JingChaoZuZhi2, Unigene83880\_JingChaoZuZhi2, Unigene839\_JingChaoZuZhi2, Unigene84017\_JingChaoZuZhi2, Unigene84032\_JingChaoZuZhi2, Unigene84111\_JingChaoZuZhi2, Unigene84115\_JingChaoZuZhi2, Unigene84116\_JingChaoZuZhi2, Unigene84180\_JingChaoZuZhi2, Unigene84190\_JingChaoZuZhi2, Unigene84216\_JingChaoZuZhi2, Unigene84409\_JingChaoZuZhi2, Unigene84431\_JingChaoZuZhi2, Unigene8460\_JingChaoZuZhi2, Unigene84610\_JingChaoZuZhi2, Unigene84638\_JingChaoZuZhi2, Unigene84673\_JingChaoZuZhi2, Unigene84731\_JingChaoZuZhi2, Unigene84806\_JingChaoZuZhi2, Unigene84931\_JingChaoZuZhi2, Unigene84960\_JingChaoZuZhi2, Unigene84979\_JingChaoZuZhi2, Unigene85059\_JingChaoZuZhi2, Unigene8507\_JingChaoZuZhi2, Unigene85458\_JingChaoZuZhi2, Unigene85464\_JingChaoZuZhi2, Unigene85541\_JingChaoZuZhi2, Unigene85580\_JingChaoZuZhi2, Unigene85632\_JingChaoZuZhi2, Unigene85774\_JingChaoZuZhi2, Unigene85775\_JingChaoZuZhi2, Unigene85804\_JingChaoZuZhi2, Unigene85823\_JingChaoZuZhi2, Unigene85844\_JingChaoZuZhi2, Unigene85849\_JingChaoZuZhi2, Unigene85909\_JingChaoZuZhi2, Unigene85979\_JingChaoZuZhi2, Unigene86050\_JingChaoZuZhi2, Unigene86294\_JingChaoZuZhi2, Unigene86359\_JingChaoZuZhi2, Unigene86440\_JingChaoZuZhi2, Unigene86519\_JingChaoZuZhi2, Unigene86748\_JingChaoZuZhi2, Unigene86808\_JingChaoZuZhi2, Unigene87014\_JingChaoZuZhi2, Unigene87053\_JingChaoZuZhi2, Unigene87118\_JingChaoZuZhi2, Unigene87180\_JingChaoZuZhi2, Unigene8732\_JingChaoZuZhi2, Unigene87331\_JingChaoZuZhi2, Unigene87357\_JingChaoZuZhi2, Unigene87378\_JingChaoZuZhi2, Unigene87447\_JingChaoZuZhi2, Unigene87470\_JingChaoZuZhi2, Unigene87543\_JingChaoZuZhi2, Unigene8777\_JingChaoZuZhi2, Unigene877\_JingChaoZuZhi2, Unigene87938\_JingChaoZuZhi2, Unigene88072\_JingChaoZuZhi2, Unigene88177\_JingChaoZuZhi2, Unigene88232\_JingChaoZuZhi2, Unigene88449\_JingChaoZuZhi2, Unigene88454\_JingChaoZuZhi2, Unigene88461\_JingChaoZuZhi2, Unigene88538\_JingChaoZuZhi2, Unigene8853\_JingChaoZuZhi2, Unigene88563\_JingChaoZuZhi2, Unigene88594\_JingChaoZuZhi2, Unigene88664\_JingChaoZuZhi2, Unigene88665\_JingChaoZuZhi2, Unigene88724\_JingChaoZuZhi2, Unigene88823\_JingChaoZuZhi2, Unigene88844\_JingChaoZuZhi2, Unigene88851\_JingChaoZuZhi2, Unigene88957\_JingChaoZuZhi2, Unigene89089\_JingChaoZuZhi2, Unigene89285\_JingChaoZuZhi2, Unigene89320\_JingChaoZuZhi2, Unigene89385\_JingChaoZuZhi2, Unigene89645\_JingChaoZuZhi2, Unigene89659\_JingChaoZuZhi2, Unigene89728\_JingChaoZuZhi2, Unigene89937\_JingChaoZuZhi2, Unigene89999\_JingChaoZuZhi2, Unigene90075\_JingChaoZuZhi2, Unigene90196\_JingChaoZuZhi2, Unigene90228\_JingChaoZuZhi2, Unigene90257\_JingChaoZuZhi2, Unigene90383\_JingChaoZuZhi2, Unigene90459\_JingChaoZuZhi2, Unigene90484\_JingChaoZuZhi2, Unigene90515\_JingChaoZuZhi2, Unigene90822\_JingChaoZuZhi2, Unigene9087\_JingChaoZuZhi2, Unigene90993\_JingChaoZuZhi2, Unigene91310\_JingChaoZuZhi2, Unigene91503\_JingChaoZuZhi2, Unigene91527\_JingChaoZuZhi2, Unigene91586\_JingChaoZuZhi2, Unigene91673\_JingChaoZuZhi2, Unigene9181\_JingChaoZuZhi2, Unigene91841\_JingChaoZuZhi2, Unigene91924\_JingChaoZuZhi2, Unigene92047\_JingChaoZuZhi2, Unigene92211\_JingChaoZuZhi2, Unigene92310\_JingChaoZuZhi2, Unigene92325\_JingChaoZuZhi2, Unigene92433\_JingChaoZuZhi2, Unigene92510\_JingChaoZuZhi2, Unigene92573\_JingChaoZuZhi2, Unigene92602\_JingChaoZuZhi2, Unigene92686\_JingChaoZuZhi2, Unigene92747\_JingChaoZuZhi2, Unigene92751\_JingChaoZuZhi2, Unigene9284\_JingChaoZuZhi2, Unigene9288\_JingChaoZuZhi2, Unigene92918\_JingChaoZuZhi2, Unigene93084\_JingChaoZuZhi2, Unigene93176\_JingChaoZuZhi2, Unigene93193\_JingChaoZuZhi2, Unigene93239\_JingChaoZuZhi2, Unigene93538\_JingChaoZuZhi2, Unigene93588\_JingChaoZuZhi2, Unigene93714\_JingChaoZuZhi2, Unigene93823\_JingChaoZuZhi2, Unigene93978\_JingChaoZuZhi2, Unigene9403\_JingChaoZuZhi2, Unigene9408\_JingChaoZuZhi2, Unigene94442\_JingChaoZuZhi2, Unigene94588\_JingChaoZuZhi2, Unigene94657\_JingChaoZuZhi2, Unigene94678\_JingChaoZuZhi2, Unigene94763\_JingChaoZuZhi2, Unigene948\_JingChaoZuZhi2, Unigene94912\_JingChaoZuZhi2, Unigene94950\_JingChaoZuZhi2, Unigene95153\_JingChaoZuZhi2, Unigene95190\_JingChaoZuZhi2, Unigene95236\_JingChaoZuZhi2, Unigene95332\_JingChaoZuZhi2, Unigene95431\_JingChaoZuZhi2, Unigene95439\_JingChaoZuZhi2, Unigene95826\_JingChaoZuZhi2, Unigene95961\_JingChaoZuZhi2, Unigene9610\_JingChaoZuZhi2, Unigene9618\_JingChaoZuZhi2, Unigene9657\_JingChaoZuZhi2, Unigene9676\_JingChaoZuZhi2, Unigene9886\_JingChaoZuZhi2, Unigene989\_JingChaoZuZhi2, Unigene992\_JingChaoZuZhi2 |
| 10 | Shigellosis | Unigene10016\_JingChaoZuZhi2, Unigene10047\_JingChaoZuZhi2, Unigene10051\_JingChaoZuZhi2, Unigene10128\_JingChaoZuZhi2, Unigene1017\_JingChaoZuZhi2, Unigene10208\_JingChaoZuZhi2, Unigene10259\_JingChaoZuZhi2, Unigene10456\_JingChaoZuZhi2, Unigene10492\_JingChaoZuZhi2, Unigene10498\_JingChaoZuZhi2, Unigene10576\_JingChaoZuZhi2, Unigene10582\_JingChaoZuZhi2, Unigene1060\_JingChaoZuZhi2, Unigene10733\_JingChaoZuZhi2, Unigene10772\_JingChaoZuZhi2, Unigene10922\_JingChaoZuZhi2, Unigene10926\_JingChaoZuZhi2, Unigene10936\_JingChaoZuZhi2, Unigene10974\_JingChaoZuZhi2, Unigene10979\_JingChaoZuZhi2, Unigene10\_JingChaoZuZhi2, Unigene1121\_JingChaoZuZhi2, Unigene11240\_JingChaoZuZhi2, Unigene11258\_JingChaoZuZhi2, Unigene11446\_JingChaoZuZhi2, Unigene11569\_JingChaoZuZhi2, Unigene115\_JingChaoZuZhi2, Unigene1162\_JingChaoZuZhi2, Unigene11641\_JingChaoZuZhi2, Unigene11729\_JingChaoZuZhi2, Unigene11810\_JingChaoZuZhi2, Unigene11902\_JingChaoZuZhi2, Unigene11918\_JingChaoZuZhi2, Unigene11932\_JingChaoZuZhi2, Unigene11951\_JingChaoZuZhi2, Unigene11995\_JingChaoZuZhi2, Unigene12011\_JingChaoZuZhi2, Unigene12060\_JingChaoZuZhi2, Unigene12105\_JingChaoZuZhi2, Unigene12148\_JingChaoZuZhi2, Unigene12285\_JingChaoZuZhi2, Unigene12310\_JingChaoZuZhi2, Unigene12326\_JingChaoZuZhi2, Unigene12343\_JingChaoZuZhi2, Unigene12372\_JingChaoZuZhi2, Unigene12549\_JingChaoZuZhi2, Unigene12644\_JingChaoZuZhi2, Unigene12815\_JingChaoZuZhi2, Unigene13085\_JingChaoZuZhi2, Unigene13097\_JingChaoZuZhi2, Unigene13196\_JingChaoZuZhi2, Unigene13249\_JingChaoZuZhi2, Unigene1325\_JingChaoZuZhi2, Unigene13291\_JingChaoZuZhi2, Unigene13478\_JingChaoZuZhi2, Unigene13572\_JingChaoZuZhi2, Unigene13607\_JingChaoZuZhi2, Unigene13618\_JingChaoZuZhi2, Unigene13650\_JingChaoZuZhi2, Unigene13720\_JingChaoZuZhi2, Unigene13766\_JingChaoZuZhi2, Unigene13890\_JingChaoZuZhi2, Unigene13901\_JingChaoZuZhi2, Unigene13904\_JingChaoZuZhi2, Unigene13927\_JingChaoZuZhi2, Unigene1393\_JingChaoZuZhi2, Unigene13952\_JingChaoZuZhi2, Unigene14071\_JingChaoZuZhi2, Unigene14159\_JingChaoZuZhi2, Unigene14193\_JingChaoZuZhi2, Unigene14246\_JingChaoZuZhi2, Unigene14289\_JingChaoZuZhi2, Unigene14418\_JingChaoZuZhi2, Unigene14450\_JingChaoZuZhi2, Unigene14476\_JingChaoZuZhi2, Unigene14534\_JingChaoZuZhi2, Unigene14610\_JingChaoZuZhi2, Unigene14714\_JingChaoZuZhi2, Unigene14715\_JingChaoZuZhi2, Unigene14845\_JingChaoZuZhi2, Unigene14970\_JingChaoZuZhi2, Unigene15088\_JingChaoZuZhi2, Unigene15096\_JingChaoZuZhi2, Unigene15176\_JingChaoZuZhi2, Unigene15251\_JingChaoZuZhi2, Unigene15286\_JingChaoZuZhi2, Unigene15288\_JingChaoZuZhi2, Unigene15319\_JingChaoZuZhi2, Unigene15374\_JingChaoZuZhi2, Unigene15381\_JingChaoZuZhi2, Unigene15492\_JingChaoZuZhi2, Unigene15560\_JingChaoZuZhi2, Unigene15615\_JingChaoZuZhi2, Unigene15650\_JingChaoZuZhi2, Unigene15651\_JingChaoZuZhi2, Unigene15659\_JingChaoZuZhi2, Unigene15731\_JingChaoZuZhi2, Unigene15736\_JingChaoZuZhi2, Unigene15857\_JingChaoZuZhi2, Unigene15879\_JingChaoZuZhi2, Unigene16004\_JingChaoZuZhi2, Unigene16103\_JingChaoZuZhi2, Unigene16121\_JingChaoZuZhi2, Unigene16176\_JingChaoZuZhi2, Unigene16182\_JingChaoZuZhi2, Unigene16318\_JingChaoZuZhi2, Unigene16324\_JingChaoZuZhi2, Unigene16402\_JingChaoZuZhi2, Unigene16481\_JingChaoZuZhi2, Unigene16591\_JingChaoZuZhi2, Unigene16606\_JingChaoZuZhi2, Unigene16640\_JingChaoZuZhi2, Unigene16655\_JingChaoZuZhi2, Unigene16730\_JingChaoZuZhi2, Unigene16778\_JingChaoZuZhi2, Unigene16873\_JingChaoZuZhi2, Unigene16950\_JingChaoZuZhi2, Unigene17018\_JingChaoZuZhi2, Unigene17101\_JingChaoZuZhi2, Unigene17122\_JingChaoZuZhi2, Unigene17501\_JingChaoZuZhi2, Unigene17547\_JingChaoZuZhi2, Unigene17764\_JingChaoZuZhi2, Unigene17789\_JingChaoZuZhi2, Unigene17845\_JingChaoZuZhi2, Unigene17894\_JingChaoZuZhi2, Unigene17930\_JingChaoZuZhi2, Unigene18010\_JingChaoZuZhi2, Unigene18256\_JingChaoZuZhi2, Unigene18259\_JingChaoZuZhi2, Unigene18269\_JingChaoZuZhi2, Unigene18334\_JingChaoZuZhi2, Unigene18348\_JingChaoZuZhi2, Unigene18360\_JingChaoZuZhi2, Unigene18441\_JingChaoZuZhi2, Unigene18505\_JingChaoZuZhi2, Unigene18648\_JingChaoZuZhi2, Unigene18662\_JingChaoZuZhi2, Unigene18685\_JingChaoZuZhi2, Unigene18790\_JingChaoZuZhi2, Unigene18835\_JingChaoZuZhi2, Unigene19001\_JingChaoZuZhi2, Unigene19021\_JingChaoZuZhi2, Unigene19086\_JingChaoZuZhi2, Unigene19137\_JingChaoZuZhi2, Unigene19204\_JingChaoZuZhi2, Unigene19223\_JingChaoZuZhi2, Unigene19319\_JingChaoZuZhi2, Unigene19365\_JingChaoZuZhi2, Unigene19391\_JingChaoZuZhi2, Unigene19512\_JingChaoZuZhi2, Unigene19533\_JingChaoZuZhi2, Unigene19701\_JingChaoZuZhi2, Unigene19883\_JingChaoZuZhi2, Unigene19979\_JingChaoZuZhi2, Unigene20025\_JingChaoZuZhi2, Unigene20030\_JingChaoZuZhi2, Unigene20049\_JingChaoZuZhi2, Unigene20083\_JingChaoZuZhi2, Unigene20086\_JingChaoZuZhi2, Unigene20120\_JingChaoZuZhi2, Unigene20157\_JingChaoZuZhi2, Unigene20159\_JingChaoZuZhi2, Unigene201\_JingChaoZuZhi2, Unigene20220\_JingChaoZuZhi2, Unigene20239\_JingChaoZuZhi2, Unigene20373\_JingChaoZuZhi2, Unigene20394\_JingChaoZuZhi2, Unigene20410\_JingChaoZuZhi2, Unigene20468\_JingChaoZuZhi2, Unigene20474\_JingChaoZuZhi2, Unigene20557\_JingChaoZuZhi2, Unigene20608\_JingChaoZuZhi2, Unigene20720\_JingChaoZuZhi2, Unigene20727\_JingChaoZuZhi2, Unigene20837\_JingChaoZuZhi2, Unigene20943\_JingChaoZuZhi2, Unigene20991\_JingChaoZuZhi2, Unigene21057\_JingChaoZuZhi2, Unigene2115\_JingChaoZuZhi2, Unigene21311\_JingChaoZuZhi2, Unigene21515\_JingChaoZuZhi2, Unigene21543\_JingChaoZuZhi2, Unigene2161\_JingChaoZuZhi2, Unigene21636\_JingChaoZuZhi2, Unigene21697\_JingChaoZuZhi2, Unigene21713\_JingChaoZuZhi2, Unigene21818\_JingChaoZuZhi2, Unigene21860\_JingChaoZuZhi2, Unigene21898\_JingChaoZuZhi2, Unigene21932\_JingChaoZuZhi2, Unigene21933\_JingChaoZuZhi2, Unigene22055\_JingChaoZuZhi2, Unigene22091\_JingChaoZuZhi2, Unigene22119\_JingChaoZuZhi2, Unigene22128\_JingChaoZuZhi2, Unigene22220\_JingChaoZuZhi2, Unigene2226\_JingChaoZuZhi2, Unigene22329\_JingChaoZuZhi2, Unigene22372\_JingChaoZuZhi2, Unigene22379\_JingChaoZuZhi2, Unigene22414\_JingChaoZuZhi2, Unigene22455\_JingChaoZuZhi2, Unigene22466\_JingChaoZuZhi2, Unigene22487\_JingChaoZuZhi2, Unigene22586\_JingChaoZuZhi2, Unigene2258\_JingChaoZuZhi2, Unigene22652\_JingChaoZuZhi2, Unigene22787\_JingChaoZuZhi2, Unigene22820\_JingChaoZuZhi2, Unigene2289\_JingChaoZuZhi2, Unigene22909\_JingChaoZuZhi2, Unigene23164\_JingChaoZuZhi2, Unigene2330\_JingChaoZuZhi2, Unigene23447\_JingChaoZuZhi2, Unigene23924\_JingChaoZuZhi2, Unigene23978\_JingChaoZuZhi2, Unigene2445\_JingChaoZuZhi2, Unigene24600\_JingChaoZuZhi2, Unigene2510\_JingChaoZuZhi2, Unigene2517\_JingChaoZuZhi2, Unigene25693\_JingChaoZuZhi2, Unigene25962\_JingChaoZuZhi2, Unigene26086\_JingChaoZuZhi2, Unigene26987\_JingChaoZuZhi2, Unigene2728\_JingChaoZuZhi2, Unigene28064\_JingChaoZuZhi2, Unigene28243\_JingChaoZuZhi2, Unigene2857\_JingChaoZuZhi2, Unigene28793\_JingChaoZuZhi2, Unigene2901\_JingChaoZuZhi2, Unigene30281\_JingChaoZuZhi2, Unigene30726\_JingChaoZuZhi2, Unigene31612\_JingChaoZuZhi2, Unigene31912\_JingChaoZuZhi2, Unigene31918\_JingChaoZuZhi2, Unigene3235\_JingChaoZuZhi2, Unigene32373\_JingChaoZuZhi2, Unigene324\_JingChaoZuZhi2, Unigene3292\_JingChaoZuZhi2, Unigene33044\_JingChaoZuZhi2, Unigene33173\_JingChaoZuZhi2, Unigene3321\_JingChaoZuZhi2, Unigene3388\_JingChaoZuZhi2, Unigene34142\_JingChaoZuZhi2, Unigene34785\_JingChaoZuZhi2, Unigene3493\_JingChaoZuZhi2, Unigene35016\_JingChaoZuZhi2, Unigene35080\_JingChaoZuZhi2, Unigene35243\_JingChaoZuZhi2, Unigene3560\_JingChaoZuZhi2, Unigene35927\_JingChaoZuZhi2, Unigene36257\_JingChaoZuZhi2, Unigene36342\_JingChaoZuZhi2, Unigene36361\_JingChaoZuZhi2, Unigene3646\_JingChaoZuZhi2, Unigene3671\_JingChaoZuZhi2, Unigene3693\_JingChaoZuZhi2, Unigene36974\_JingChaoZuZhi2, Unigene36976\_JingChaoZuZhi2, Unigene37164\_JingChaoZuZhi2, Unigene37176\_JingChaoZuZhi2, Unigene37818\_JingChaoZuZhi2, Unigene3854\_JingChaoZuZhi2, Unigene39102\_JingChaoZuZhi2, Unigene39216\_JingChaoZuZhi2, Unigene4015\_JingChaoZuZhi2, Unigene40200\_JingChaoZuZhi2, Unigene40281\_JingChaoZuZhi2, Unigene4044\_JingChaoZuZhi2, Unigene40919\_JingChaoZuZhi2, Unigene41040\_JingChaoZuZhi2, Unigene41148\_JingChaoZuZhi2, Unigene411\_JingChaoZuZhi2, Unigene41213\_JingChaoZuZhi2, Unigene41700\_JingChaoZuZhi2, Unigene42146\_JingChaoZuZhi2, Unigene42248\_JingChaoZuZhi2, Unigene4254\_JingChaoZuZhi2, Unigene4294\_JingChaoZuZhi2, Unigene43041\_JingChaoZuZhi2, Unigene43147\_JingChaoZuZhi2, Unigene43169\_JingChaoZuZhi2, Unigene43249\_JingChaoZuZhi2, Unigene43260\_JingChaoZuZhi2, Unigene4337\_JingChaoZuZhi2, Unigene43512\_JingChaoZuZhi2, Unigene43574\_JingChaoZuZhi2, Unigene44190\_JingChaoZuZhi2, Unigene44336\_JingChaoZuZhi2, Unigene44454\_JingChaoZuZhi2, Unigene44516\_JingChaoZuZhi2, Unigene45134\_JingChaoZuZhi2, Unigene45367\_JingChaoZuZhi2, Unigene4547\_JingChaoZuZhi2, Unigene45593\_JingChaoZuZhi2, Unigene45622\_JingChaoZuZhi2, Unigene45782\_JingChaoZuZhi2, Unigene45901\_JingChaoZuZhi2, Unigene4599\_JingChaoZuZhi2, Unigene46407\_JingChaoZuZhi2, Unigene4713\_JingChaoZuZhi2, Unigene47578\_JingChaoZuZhi2, Unigene4758\_JingChaoZuZhi2, Unigene47749\_JingChaoZuZhi2, Unigene47949\_JingChaoZuZhi2, Unigene4829\_JingChaoZuZhi2, Unigene4873\_JingChaoZuZhi2, Unigene48843\_JingChaoZuZhi2, Unigene48853\_JingChaoZuZhi2, Unigene49387\_JingChaoZuZhi2, Unigene49973\_JingChaoZuZhi2, Unigene50201\_JingChaoZuZhi2, Unigene50540\_JingChaoZuZhi2, Unigene50660\_JingChaoZuZhi2, Unigene50668\_JingChaoZuZhi2, Unigene50744\_JingChaoZuZhi2, Unigene5108\_JingChaoZuZhi2, Unigene51090\_JingChaoZuZhi2, Unigene51115\_JingChaoZuZhi2, Unigene5125\_JingChaoZuZhi2, Unigene5126\_JingChaoZuZhi2, Unigene51467\_JingChaoZuZhi2, Unigene51567\_JingChaoZuZhi2, Unigene52100\_JingChaoZuZhi2, Unigene52125\_JingChaoZuZhi2, Unigene52456\_JingChaoZuZhi2, Unigene524\_JingChaoZuZhi2, Unigene52638\_JingChaoZuZhi2, Unigene5265\_JingChaoZuZhi2, Unigene5298\_JingChaoZuZhi2, Unigene53131\_JingChaoZuZhi2, Unigene5335\_JingChaoZuZhi2, Unigene53484\_JingChaoZuZhi2, Unigene53500\_JingChaoZuZhi2, Unigene53751\_JingChaoZuZhi2, Unigene54081\_JingChaoZuZhi2, Unigene5418\_JingChaoZuZhi2, Unigene54320\_JingChaoZuZhi2, Unigene54918\_JingChaoZuZhi2, Unigene54974\_JingChaoZuZhi2, Unigene55009\_JingChaoZuZhi2, Unigene55019\_JingChaoZuZhi2, Unigene55385\_JingChaoZuZhi2, Unigene56421\_JingChaoZuZhi2, Unigene56560\_JingChaoZuZhi2, Unigene56660\_JingChaoZuZhi2, Unigene57240\_JingChaoZuZhi2, Unigene57597\_JingChaoZuZhi2, Unigene5782\_JingChaoZuZhi2, Unigene5809\_JingChaoZuZhi2, Unigene58169\_JingChaoZuZhi2, Unigene58348\_JingChaoZuZhi2, Unigene58474\_JingChaoZuZhi2, Unigene58675\_JingChaoZuZhi2, Unigene5896\_JingChaoZuZhi2, Unigene59101\_JingChaoZuZhi2, Unigene59146\_JingChaoZuZhi2, Unigene59265\_JingChaoZuZhi2, Unigene59336\_JingChaoZuZhi2, Unigene59433\_JingChaoZuZhi2, Unigene59629\_JingChaoZuZhi2, Unigene59684\_JingChaoZuZhi2, Unigene60121\_JingChaoZuZhi2, Unigene60551\_JingChaoZuZhi2, Unigene60586\_JingChaoZuZhi2, Unigene60607\_JingChaoZuZhi2, Unigene6072\_JingChaoZuZhi2, Unigene60930\_JingChaoZuZhi2, Unigene60990\_JingChaoZuZhi2, Unigene61244\_JingChaoZuZhi2, Unigene61474\_JingChaoZuZhi2, Unigene6158\_JingChaoZuZhi2, Unigene6170\_JingChaoZuZhi2, Unigene6171\_JingChaoZuZhi2, Unigene61879\_JingChaoZuZhi2, Unigene61931\_JingChaoZuZhi2, Unigene62100\_JingChaoZuZhi2, Unigene6215\_JingChaoZuZhi2, Unigene62257\_JingChaoZuZhi2, Unigene6233\_JingChaoZuZhi2, Unigene62606\_JingChaoZuZhi2, Unigene62695\_JingChaoZuZhi2, Unigene63052\_JingChaoZuZhi2, Unigene63145\_JingChaoZuZhi2, Unigene63384\_JingChaoZuZhi2, Unigene63424\_JingChaoZuZhi2, Unigene63584\_JingChaoZuZhi2, Unigene63604\_JingChaoZuZhi2, Unigene63615\_JingChaoZuZhi2, Unigene63812\_JingChaoZuZhi2, Unigene63873\_JingChaoZuZhi2, Unigene63925\_JingChaoZuZhi2, Unigene64208\_JingChaoZuZhi2, Unigene6438\_JingChaoZuZhi2, Unigene64452\_JingChaoZuZhi2, Unigene64458\_JingChaoZuZhi2, Unigene6459\_JingChaoZuZhi2, Unigene64650\_JingChaoZuZhi2, Unigene64692\_JingChaoZuZhi2, Unigene64843\_JingChaoZuZhi2, Unigene6493\_JingChaoZuZhi2, Unigene6498\_JingChaoZuZhi2, Unigene65022\_JingChaoZuZhi2, Unigene65129\_JingChaoZuZhi2, Unigene65150\_JingChaoZuZhi2, Unigene65187\_JingChaoZuZhi2, Unigene65326\_JingChaoZuZhi2, Unigene65375\_JingChaoZuZhi2, Unigene6539\_JingChaoZuZhi2, Unigene65773\_JingChaoZuZhi2, Unigene6605\_JingChaoZuZhi2, Unigene66087\_JingChaoZuZhi2, Unigene66253\_JingChaoZuZhi2, Unigene6634\_JingChaoZuZhi2, Unigene6651\_JingChaoZuZhi2, Unigene66559\_JingChaoZuZhi2, Unigene66590\_JingChaoZuZhi2, Unigene66813\_JingChaoZuZhi2, Unigene66913\_JingChaoZuZhi2, Unigene66986\_JingChaoZuZhi2, Unigene67005\_JingChaoZuZhi2, Unigene67063\_JingChaoZuZhi2, Unigene67070\_JingChaoZuZhi2, Unigene67249\_JingChaoZuZhi2, Unigene67279\_JingChaoZuZhi2, Unigene67572\_JingChaoZuZhi2, Unigene67777\_JingChaoZuZhi2, Unigene67958\_JingChaoZuZhi2, Unigene6796\_JingChaoZuZhi2, Unigene6797\_JingChaoZuZhi2, Unigene68108\_JingChaoZuZhi2, Unigene68139\_JingChaoZuZhi2, Unigene6815\_JingChaoZuZhi2, Unigene6828\_JingChaoZuZhi2, Unigene68527\_JingChaoZuZhi2, Unigene68672\_JingChaoZuZhi2, Unigene6872\_JingChaoZuZhi2, Unigene69069\_JingChaoZuZhi2, Unigene69092\_JingChaoZuZhi2, Unigene69100\_JingChaoZuZhi2, Unigene69141\_JingChaoZuZhi2, Unigene6914\_JingChaoZuZhi2, Unigene69221\_JingChaoZuZhi2, Unigene69386\_JingChaoZuZhi2, Unigene69562\_JingChaoZuZhi2, Unigene69658\_JingChaoZuZhi2, Unigene69775\_JingChaoZuZhi2, Unigene70045\_JingChaoZuZhi2, Unigene70063\_JingChaoZuZhi2, Unigene7016\_JingChaoZuZhi2, Unigene70329\_JingChaoZuZhi2, Unigene7032\_JingChaoZuZhi2, Unigene70970\_JingChaoZuZhi2, Unigene71043\_JingChaoZuZhi2, Unigene7116\_JingChaoZuZhi2, Unigene7118\_JingChaoZuZhi2, Unigene71213\_JingChaoZuZhi2, Unigene71241\_JingChaoZuZhi2, Unigene71385\_JingChaoZuZhi2, Unigene7151\_JingChaoZuZhi2, Unigene71546\_JingChaoZuZhi2, Unigene71592\_JingChaoZuZhi2, Unigene71729\_JingChaoZuZhi2, Unigene71755\_JingChaoZuZhi2, Unigene71774\_JingChaoZuZhi2, Unigene71808\_JingChaoZuZhi2, Unigene7206\_JingChaoZuZhi2, Unigene72249\_JingChaoZuZhi2, Unigene72334\_JingChaoZuZhi2, Unigene72351\_JingChaoZuZhi2, Unigene72503\_JingChaoZuZhi2, Unigene7268\_JingChaoZuZhi2, Unigene72708\_JingChaoZuZhi2, Unigene72832\_JingChaoZuZhi2, Unigene73257\_JingChaoZuZhi2, Unigene7326\_JingChaoZuZhi2, Unigene73446\_JingChaoZuZhi2, Unigene73475\_JingChaoZuZhi2, Unigene73550\_JingChaoZuZhi2, Unigene73620\_JingChaoZuZhi2, Unigene73817\_JingChaoZuZhi2, Unigene73887\_JingChaoZuZhi2, Unigene74050\_JingChaoZuZhi2, Unigene741\_JingChaoZuZhi2, Unigene74221\_JingChaoZuZhi2, Unigene74259\_JingChaoZuZhi2, Unigene74298\_JingChaoZuZhi2, Unigene74474\_JingChaoZuZhi2, Unigene74475\_JingChaoZuZhi2, Unigene74719\_JingChaoZuZhi2, Unigene74730\_JingChaoZuZhi2, Unigene74854\_JingChaoZuZhi2, Unigene7489\_JingChaoZuZhi2, Unigene75009\_JingChaoZuZhi2, Unigene7508\_JingChaoZuZhi2, Unigene75112\_JingChaoZuZhi2, Unigene75167\_JingChaoZuZhi2, Unigene75491\_JingChaoZuZhi2, Unigene75840\_JingChaoZuZhi2, Unigene75902\_JingChaoZuZhi2, Unigene75946\_JingChaoZuZhi2, Unigene76002\_JingChaoZuZhi2, Unigene76023\_JingChaoZuZhi2, Unigene76069\_JingChaoZuZhi2, Unigene76111\_JingChaoZuZhi2, Unigene76269\_JingChaoZuZhi2, Unigene76302\_JingChaoZuZhi2, Unigene7634\_JingChaoZuZhi2, Unigene76515\_JingChaoZuZhi2, Unigene7663\_JingChaoZuZhi2, Unigene76776\_JingChaoZuZhi2, Unigene76785\_JingChaoZuZhi2, Unigene76819\_JingChaoZuZhi2, Unigene77406\_JingChaoZuZhi2, Unigene77413\_JingChaoZuZhi2, Unigene77510\_JingChaoZuZhi2, Unigene77520\_JingChaoZuZhi2, Unigene77570\_JingChaoZuZhi2, Unigene77712\_JingChaoZuZhi2, Unigene7794\_JingChaoZuZhi2, Unigene77954\_JingChaoZuZhi2, Unigene77955\_JingChaoZuZhi2, Unigene78009\_JingChaoZuZhi2, Unigene78288\_JingChaoZuZhi2, Unigene78325\_JingChaoZuZhi2, Unigene78361\_JingChaoZuZhi2, Unigene78410\_JingChaoZuZhi2, Unigene7850\_JingChaoZuZhi2, Unigene78521\_JingChaoZuZhi2, Unigene78555\_JingChaoZuZhi2, Unigene78636\_JingChaoZuZhi2, Unigene79058\_JingChaoZuZhi2, Unigene79118\_JingChaoZuZhi2, Unigene7911\_JingChaoZuZhi2, Unigene79141\_JingChaoZuZhi2, Unigene79358\_JingChaoZuZhi2, Unigene79411\_JingChaoZuZhi2, Unigene79433\_JingChaoZuZhi2, Unigene79443\_JingChaoZuZhi2, Unigene79653\_JingChaoZuZhi2, Unigene79726\_JingChaoZuZhi2, Unigene79789\_JingChaoZuZhi2, Unigene79824\_JingChaoZuZhi2, Unigene79864\_JingChaoZuZhi2, Unigene79871\_JingChaoZuZhi2, Unigene7987\_JingChaoZuZhi2, Unigene79882\_JingChaoZuZhi2, Unigene79940\_JingChaoZuZhi2, Unigene80095\_JingChaoZuZhi2, Unigene80143\_JingChaoZuZhi2, Unigene80212\_JingChaoZuZhi2, Unigene80246\_JingChaoZuZhi2, Unigene80315\_JingChaoZuZhi2, Unigene80403\_JingChaoZuZhi2, Unigene80619\_JingChaoZuZhi2, Unigene80893\_JingChaoZuZhi2, Unigene80980\_JingChaoZuZhi2, Unigene81145\_JingChaoZuZhi2, Unigene81167\_JingChaoZuZhi2, Unigene81253\_JingChaoZuZhi2, Unigene81271\_JingChaoZuZhi2, Unigene81327\_JingChaoZuZhi2, Unigene81372\_JingChaoZuZhi2, Unigene81434\_JingChaoZuZhi2, Unigene81440\_JingChaoZuZhi2, Unigene81450\_JingChaoZuZhi2, Unigene81471\_JingChaoZuZhi2, Unigene81561\_JingChaoZuZhi2, Unigene81592\_JingChaoZuZhi2, Unigene81599\_JingChaoZuZhi2, Unigene81686\_JingChaoZuZhi2, Unigene81757\_JingChaoZuZhi2, Unigene81803\_JingChaoZuZhi2, Unigene81825\_JingChaoZuZhi2, Unigene8191\_JingChaoZuZhi2, Unigene82047\_JingChaoZuZhi2, Unigene82070\_JingChaoZuZhi2, Unigene82111\_JingChaoZuZhi2, Unigene82117\_JingChaoZuZhi2, Unigene82215\_JingChaoZuZhi2, Unigene8237\_JingChaoZuZhi2, Unigene82489\_JingChaoZuZhi2, Unigene8248\_JingChaoZuZhi2, Unigene82521\_JingChaoZuZhi2, Unigene82578\_JingChaoZuZhi2, Unigene82605\_JingChaoZuZhi2, Unigene82688\_JingChaoZuZhi2, Unigene82760\_JingChaoZuZhi2, Unigene82921\_JingChaoZuZhi2, Unigene83079\_JingChaoZuZhi2, Unigene83214\_JingChaoZuZhi2, Unigene83433\_JingChaoZuZhi2, Unigene83766\_JingChaoZuZhi2, Unigene83782\_JingChaoZuZhi2, Unigene83825\_JingChaoZuZhi2, Unigene83872\_JingChaoZuZhi2, Unigene8392\_JingChaoZuZhi2, Unigene8406\_JingChaoZuZhi2, Unigene84076\_JingChaoZuZhi2, Unigene84109\_JingChaoZuZhi2, Unigene84152\_JingChaoZuZhi2, Unigene84168\_JingChaoZuZhi2, Unigene841\_JingChaoZuZhi2, Unigene84212\_JingChaoZuZhi2, Unigene8443\_JingChaoZuZhi2, Unigene84519\_JingChaoZuZhi2, Unigene84533\_JingChaoZuZhi2, Unigene84566\_JingChaoZuZhi2, Unigene84748\_JingChaoZuZhi2, Unigene84760\_JingChaoZuZhi2, Unigene84924\_JingChaoZuZhi2, Unigene84945\_JingChaoZuZhi2, Unigene84993\_JingChaoZuZhi2, Unigene85193\_JingChaoZuZhi2, Unigene851\_JingChaoZuZhi2, Unigene85214\_JingChaoZuZhi2, Unigene85314\_JingChaoZuZhi2, Unigene85503\_JingChaoZuZhi2, Unigene85577\_JingChaoZuZhi2, Unigene85578\_JingChaoZuZhi2, Unigene85770\_JingChaoZuZhi2, Unigene85902\_JingChaoZuZhi2, Unigene86020\_JingChaoZuZhi2, Unigene86091\_JingChaoZuZhi2, Unigene86145\_JingChaoZuZhi2, Unigene86349\_JingChaoZuZhi2, Unigene86470\_JingChaoZuZhi2, Unigene86546\_JingChaoZuZhi2, Unigene86579\_JingChaoZuZhi2, Unigene8658\_JingChaoZuZhi2, Unigene8661\_JingChaoZuZhi2, Unigene86669\_JingChaoZuZhi2, Unigene86741\_JingChaoZuZhi2, Unigene86789\_JingChaoZuZhi2, Unigene87082\_JingChaoZuZhi2, Unigene8709\_JingChaoZuZhi2, Unigene87203\_JingChaoZuZhi2, Unigene87256\_JingChaoZuZhi2, Unigene87362\_JingChaoZuZhi2, Unigene87365\_JingChaoZuZhi2, Unigene87459\_JingChaoZuZhi2, Unigene87478\_JingChaoZuZhi2, Unigene87488\_JingChaoZuZhi2, Unigene87533\_JingChaoZuZhi2, Unigene87774\_JingChaoZuZhi2, Unigene87853\_JingChaoZuZhi2, Unigene87946\_JingChaoZuZhi2, Unigene88059\_JingChaoZuZhi2, Unigene88169\_JingChaoZuZhi2, Unigene88307\_JingChaoZuZhi2, Unigene8833\_JingChaoZuZhi2, Unigene88342\_JingChaoZuZhi2, Unigene88400\_JingChaoZuZhi2, Unigene88406\_JingChaoZuZhi2, Unigene88442\_JingChaoZuZhi2, Unigene88470\_JingChaoZuZhi2, Unigene88484\_JingChaoZuZhi2, Unigene88608\_JingChaoZuZhi2, Unigene88683\_JingChaoZuZhi2, Unigene88738\_JingChaoZuZhi2, Unigene88741\_JingChaoZuZhi2, Unigene88778\_JingChaoZuZhi2, Unigene88779\_JingChaoZuZhi2, Unigene88781\_JingChaoZuZhi2, Unigene88797\_JingChaoZuZhi2, Unigene88830\_JingChaoZuZhi2, Unigene88837\_JingChaoZuZhi2, Unigene88945\_JingChaoZuZhi2, Unigene88979\_JingChaoZuZhi2, Unigene88988\_JingChaoZuZhi2, Unigene89013\_JingChaoZuZhi2, Unigene89060\_JingChaoZuZhi2, Unigene8907\_JingChaoZuZhi2, Unigene89093\_JingChaoZuZhi2, Unigene89121\_JingChaoZuZhi2, Unigene89323\_JingChaoZuZhi2, Unigene89337\_JingChaoZuZhi2, Unigene89352\_JingChaoZuZhi2, Unigene89367\_JingChaoZuZhi2, Unigene89541\_JingChaoZuZhi2, Unigene89606\_JingChaoZuZhi2, Unigene89746\_JingChaoZuZhi2, Unigene89769\_JingChaoZuZhi2, Unigene89834\_JingChaoZuZhi2, Unigene89867\_JingChaoZuZhi2, Unigene89956\_JingChaoZuZhi2, Unigene90027\_JingChaoZuZhi2, Unigene90038\_JingChaoZuZhi2, Unigene90066\_JingChaoZuZhi2, Unigene90071\_JingChaoZuZhi2, Unigene90209\_JingChaoZuZhi2, Unigene90341\_JingChaoZuZhi2, Unigene90454\_JingChaoZuZhi2, Unigene9049\_JingChaoZuZhi2, Unigene9050\_JingChaoZuZhi2, Unigene90564\_JingChaoZuZhi2, Unigene90714\_JingChaoZuZhi2, Unigene90779\_JingChaoZuZhi2, Unigene90953\_JingChaoZuZhi2, Unigene90995\_JingChaoZuZhi2, Unigene91077\_JingChaoZuZhi2, Unigene91099\_JingChaoZuZhi2, Unigene91148\_JingChaoZuZhi2, Unigene91163\_JingChaoZuZhi2, Unigene91179\_JingChaoZuZhi2, Unigene91233\_JingChaoZuZhi2, Unigene91379\_JingChaoZuZhi2, Unigene91390\_JingChaoZuZhi2, Unigene91456\_JingChaoZuZhi2, Unigene91475\_JingChaoZuZhi2, Unigene91487\_JingChaoZuZhi2, Unigene91529\_JingChaoZuZhi2, Unigene91553\_JingChaoZuZhi2, Unigene91574\_JingChaoZuZhi2, Unigene91648\_JingChaoZuZhi2, Unigene91848\_JingChaoZuZhi2, Unigene91906\_JingChaoZuZhi2, Unigene92059\_JingChaoZuZhi2, Unigene92346\_JingChaoZuZhi2, Unigene92492\_JingChaoZuZhi2, Unigene92580\_JingChaoZuZhi2, Unigene92650\_JingChaoZuZhi2, Unigene92671\_JingChaoZuZhi2, Unigene92681\_JingChaoZuZhi2, Unigene92700\_JingChaoZuZhi2, Unigene92780\_JingChaoZuZhi2, Unigene92832\_JingChaoZuZhi2, Unigene92838\_JingChaoZuZhi2, Unigene92951\_JingChaoZuZhi2, Unigene92995\_JingChaoZuZhi2, Unigene93073\_JingChaoZuZhi2, Unigene93246\_JingChaoZuZhi2, Unigene93479\_JingChaoZuZhi2, Unigene93552\_JingChaoZuZhi2, Unigene93562\_JingChaoZuZhi2, Unigene93573\_JingChaoZuZhi2, Unigene93592\_JingChaoZuZhi2, Unigene93610\_JingChaoZuZhi2, Unigene93702\_JingChaoZuZhi2, Unigene93742\_JingChaoZuZhi2, Unigene93768\_JingChaoZuZhi2, Unigene93780\_JingChaoZuZhi2, Unigene93782\_JingChaoZuZhi2, Unigene93821\_JingChaoZuZhi2, Unigene93853\_JingChaoZuZhi2, Unigene93979\_JingChaoZuZhi2, Unigene94036\_JingChaoZuZhi2, Unigene94083\_JingChaoZuZhi2, Unigene94110\_JingChaoZuZhi2, Unigene94120\_JingChaoZuZhi2, Unigene94129\_JingChaoZuZhi2, Unigene94134\_JingChaoZuZhi2, Unigene94138\_JingChaoZuZhi2, Unigene94208\_JingChaoZuZhi2, Unigene94404\_JingChaoZuZhi2, Unigene94471\_JingChaoZuZhi2, Unigene94520\_JingChaoZuZhi2, Unigene94544\_JingChaoZuZhi2, Unigene94561\_JingChaoZuZhi2, Unigene94584\_JingChaoZuZhi2, Unigene94619\_JingChaoZuZhi2, Unigene94636\_JingChaoZuZhi2, Unigene94656\_JingChaoZuZhi2, Unigene94687\_JingChaoZuZhi2, Unigene94891\_JingChaoZuZhi2, Unigene94903\_JingChaoZuZhi2, Unigene9490\_JingChaoZuZhi2, Unigene94950\_JingChaoZuZhi2, Unigene94968\_JingChaoZuZhi2, Unigene95088\_JingChaoZuZhi2, Unigene95098\_JingChaoZuZhi2, Unigene95119\_JingChaoZuZhi2, Unigene951\_JingChaoZuZhi2, Unigene95203\_JingChaoZuZhi2, Unigene95212\_JingChaoZuZhi2, Unigene95371\_JingChaoZuZhi2, Unigene9540\_JingChaoZuZhi2, Unigene95433\_JingChaoZuZhi2, Unigene95589\_JingChaoZuZhi2, Unigene95633\_JingChaoZuZhi2, Unigene95686\_JingChaoZuZhi2, Unigene95812\_JingChaoZuZhi2, Unigene95875\_JingChaoZuZhi2, Unigene95907\_JingChaoZuZhi2, Unigene96121\_JingChaoZuZhi2, Unigene9688\_JingChaoZuZhi2, Unigene9739\_JingChaoZuZhi2, Unigene9743\_JingChaoZuZhi2, Unigene9807\_JingChaoZuZhi2, Unigene9833\_JingChaoZuZhi2, Unigene9988\_JingChaoZuZhi2, Unigene9992\_JingChaoZuZhi2 |
| 11 | Bacterial invasion of epithelial cells | Unigene10016\_JingChaoZuZhi2, Unigene10047\_JingChaoZuZhi2, Unigene10051\_JingChaoZuZhi2, Unigene10128\_JingChaoZuZhi2, Unigene1017\_JingChaoZuZhi2, Unigene10208\_JingChaoZuZhi2, Unigene10259\_JingChaoZuZhi2, Unigene10456\_JingChaoZuZhi2, Unigene10492\_JingChaoZuZhi2, Unigene10498\_JingChaoZuZhi2, Unigene10576\_JingChaoZuZhi2, Unigene10582\_JingChaoZuZhi2, Unigene1060\_JingChaoZuZhi2, Unigene10637\_JingChaoZuZhi2, Unigene10733\_JingChaoZuZhi2, Unigene10772\_JingChaoZuZhi2, Unigene10922\_JingChaoZuZhi2, Unigene10936\_JingChaoZuZhi2, Unigene10974\_JingChaoZuZhi2, Unigene10979\_JingChaoZuZhi2, Unigene10\_JingChaoZuZhi2, Unigene1121\_JingChaoZuZhi2, Unigene11240\_JingChaoZuZhi2, Unigene11258\_JingChaoZuZhi2, Unigene11446\_JingChaoZuZhi2, Unigene11569\_JingChaoZuZhi2, Unigene115\_JingChaoZuZhi2, Unigene1162\_JingChaoZuZhi2, Unigene11641\_JingChaoZuZhi2, Unigene11729\_JingChaoZuZhi2, Unigene11810\_JingChaoZuZhi2, Unigene11889\_JingChaoZuZhi2, Unigene11902\_JingChaoZuZhi2, Unigene11918\_JingChaoZuZhi2, Unigene11932\_JingChaoZuZhi2, Unigene11961\_JingChaoZuZhi2, Unigene11995\_JingChaoZuZhi2, Unigene12011\_JingChaoZuZhi2, Unigene12148\_JingChaoZuZhi2, Unigene1227\_JingChaoZuZhi2, Unigene12285\_JingChaoZuZhi2, Unigene12310\_JingChaoZuZhi2, Unigene12326\_JingChaoZuZhi2, Unigene12343\_JingChaoZuZhi2, Unigene12372\_JingChaoZuZhi2, Unigene12549\_JingChaoZuZhi2, Unigene12595\_JingChaoZuZhi2, Unigene12644\_JingChaoZuZhi2, Unigene12815\_JingChaoZuZhi2, Unigene12881\_JingChaoZuZhi2, Unigene13085\_JingChaoZuZhi2, Unigene13097\_JingChaoZuZhi2, Unigene13196\_JingChaoZuZhi2, Unigene13249\_JingChaoZuZhi2, Unigene1325\_JingChaoZuZhi2, Unigene13478\_JingChaoZuZhi2, Unigene13510\_JingChaoZuZhi2, Unigene13572\_JingChaoZuZhi2, Unigene13588\_JingChaoZuZhi2, Unigene13607\_JingChaoZuZhi2, Unigene13618\_JingChaoZuZhi2, Unigene13650\_JingChaoZuZhi2, Unigene13720\_JingChaoZuZhi2, Unigene13766\_JingChaoZuZhi2, Unigene13795\_JingChaoZuZhi2, Unigene13890\_JingChaoZuZhi2, Unigene13901\_JingChaoZuZhi2, Unigene13904\_JingChaoZuZhi2, Unigene13927\_JingChaoZuZhi2, Unigene14071\_JingChaoZuZhi2, Unigene14159\_JingChaoZuZhi2, Unigene14193\_JingChaoZuZhi2, Unigene14246\_JingChaoZuZhi2, Unigene14285\_JingChaoZuZhi2, Unigene14289\_JingChaoZuZhi2, Unigene14401\_JingChaoZuZhi2, Unigene14418\_JingChaoZuZhi2, Unigene14450\_JingChaoZuZhi2, Unigene14476\_JingChaoZuZhi2, Unigene14534\_JingChaoZuZhi2, Unigene14610\_JingChaoZuZhi2, Unigene14714\_JingChaoZuZhi2, Unigene14715\_JingChaoZuZhi2, Unigene14845\_JingChaoZuZhi2, Unigene14912\_JingChaoZuZhi2, Unigene14970\_JingChaoZuZhi2, Unigene15029\_JingChaoZuZhi2, Unigene15088\_JingChaoZuZhi2, Unigene15096\_JingChaoZuZhi2, Unigene15176\_JingChaoZuZhi2, Unigene15228\_JingChaoZuZhi2, Unigene15251\_JingChaoZuZhi2, Unigene15286\_JingChaoZuZhi2, Unigene15288\_JingChaoZuZhi2, Unigene15319\_JingChaoZuZhi2, Unigene15374\_JingChaoZuZhi2, Unigene15381\_JingChaoZuZhi2, Unigene15492\_JingChaoZuZhi2, Unigene15615\_JingChaoZuZhi2, Unigene15650\_JingChaoZuZhi2, Unigene15651\_JingChaoZuZhi2, Unigene15659\_JingChaoZuZhi2, Unigene15731\_JingChaoZuZhi2, Unigene15736\_JingChaoZuZhi2, Unigene15857\_JingChaoZuZhi2, Unigene15879\_JingChaoZuZhi2, Unigene16004\_JingChaoZuZhi2, Unigene16103\_JingChaoZuZhi2, Unigene16121\_JingChaoZuZhi2, Unigene16176\_JingChaoZuZhi2, Unigene16182\_JingChaoZuZhi2, Unigene16318\_JingChaoZuZhi2, Unigene16402\_JingChaoZuZhi2, Unigene16481\_JingChaoZuZhi2, Unigene16591\_JingChaoZuZhi2, Unigene16606\_JingChaoZuZhi2, Unigene16655\_JingChaoZuZhi2, Unigene16730\_JingChaoZuZhi2, Unigene16837\_JingChaoZuZhi2, Unigene16873\_JingChaoZuZhi2, Unigene16950\_JingChaoZuZhi2, Unigene17101\_JingChaoZuZhi2, Unigene17122\_JingChaoZuZhi2, Unigene17247\_JingChaoZuZhi2, Unigene17501\_JingChaoZuZhi2, Unigene17547\_JingChaoZuZhi2, Unigene17764\_JingChaoZuZhi2, Unigene17788\_JingChaoZuZhi2, Unigene17789\_JingChaoZuZhi2, Unigene17821\_JingChaoZuZhi2, Unigene17845\_JingChaoZuZhi2, Unigene17894\_JingChaoZuZhi2, Unigene18010\_JingChaoZuZhi2, Unigene18256\_JingChaoZuZhi2, Unigene18259\_JingChaoZuZhi2, Unigene18269\_JingChaoZuZhi2, Unigene18334\_JingChaoZuZhi2, Unigene18348\_JingChaoZuZhi2, Unigene18383\_JingChaoZuZhi2, Unigene18441\_JingChaoZuZhi2, Unigene18505\_JingChaoZuZhi2, Unigene18648\_JingChaoZuZhi2, Unigene18662\_JingChaoZuZhi2, Unigene18672\_JingChaoZuZhi2, Unigene1867\_JingChaoZuZhi2, Unigene18685\_JingChaoZuZhi2, Unigene18790\_JingChaoZuZhi2, Unigene18835\_JingChaoZuZhi2, Unigene18986\_JingChaoZuZhi2, Unigene19001\_JingChaoZuZhi2, Unigene19021\_JingChaoZuZhi2, Unigene19046\_JingChaoZuZhi2, Unigene19086\_JingChaoZuZhi2, Unigene19137\_JingChaoZuZhi2, Unigene19204\_JingChaoZuZhi2, Unigene19223\_JingChaoZuZhi2, Unigene19319\_JingChaoZuZhi2, Unigene19365\_JingChaoZuZhi2, Unigene19391\_JingChaoZuZhi2, Unigene19512\_JingChaoZuZhi2, Unigene19533\_JingChaoZuZhi2, Unigene19579\_JingChaoZuZhi2, Unigene19701\_JingChaoZuZhi2, Unigene19883\_JingChaoZuZhi2, Unigene19979\_JingChaoZuZhi2, Unigene20025\_JingChaoZuZhi2, Unigene20030\_JingChaoZuZhi2, Unigene20049\_JingChaoZuZhi2, Unigene20083\_JingChaoZuZhi2, Unigene20086\_JingChaoZuZhi2, Unigene20159\_JingChaoZuZhi2, Unigene201\_JingChaoZuZhi2, Unigene20239\_JingChaoZuZhi2, Unigene20373\_JingChaoZuZhi2, Unigene20394\_JingChaoZuZhi2, Unigene20410\_JingChaoZuZhi2, Unigene20468\_JingChaoZuZhi2, Unigene20474\_JingChaoZuZhi2, Unigene20557\_JingChaoZuZhi2, Unigene20720\_JingChaoZuZhi2, Unigene20727\_JingChaoZuZhi2, Unigene20794\_JingChaoZuZhi2, Unigene20837\_JingChaoZuZhi2, Unigene20918\_JingChaoZuZhi2, Unigene20943\_JingChaoZuZhi2, Unigene21057\_JingChaoZuZhi2, Unigene2115\_JingChaoZuZhi2, Unigene21311\_JingChaoZuZhi2, Unigene21536\_JingChaoZuZhi2, Unigene21543\_JingChaoZuZhi2, Unigene21605\_JingChaoZuZhi2, Unigene2161\_JingChaoZuZhi2, Unigene21636\_JingChaoZuZhi2, Unigene21790\_JingChaoZuZhi2, Unigene21818\_JingChaoZuZhi2, Unigene21860\_JingChaoZuZhi2, Unigene21898\_JingChaoZuZhi2, Unigene21932\_JingChaoZuZhi2, Unigene21933\_JingChaoZuZhi2, Unigene22055\_JingChaoZuZhi2, Unigene22091\_JingChaoZuZhi2, Unigene22119\_JingChaoZuZhi2, Unigene22128\_JingChaoZuZhi2, Unigene2216\_JingChaoZuZhi2, Unigene22220\_JingChaoZuZhi2, Unigene2226\_JingChaoZuZhi2, Unigene22329\_JingChaoZuZhi2, Unigene22372\_JingChaoZuZhi2, Unigene22414\_JingChaoZuZhi2, Unigene22455\_JingChaoZuZhi2, Unigene22500\_JingChaoZuZhi2, Unigene22517\_JingChaoZuZhi2, Unigene22586\_JingChaoZuZhi2, Unigene2258\_JingChaoZuZhi2, Unigene22885\_JingChaoZuZhi2, Unigene2289\_JingChaoZuZhi2, Unigene22909\_JingChaoZuZhi2, Unigene23164\_JingChaoZuZhi2, Unigene2330\_JingChaoZuZhi2, Unigene23447\_JingChaoZuZhi2, Unigene23924\_JingChaoZuZhi2, Unigene23978\_JingChaoZuZhi2, Unigene2445\_JingChaoZuZhi2, Unigene2456\_JingChaoZuZhi2, Unigene24600\_JingChaoZuZhi2, Unigene2510\_JingChaoZuZhi2, Unigene2517\_JingChaoZuZhi2, Unigene25693\_JingChaoZuZhi2, Unigene25962\_JingChaoZuZhi2, Unigene26987\_JingChaoZuZhi2, Unigene28064\_JingChaoZuZhi2, Unigene28243\_JingChaoZuZhi2, Unigene283\_JingChaoZuZhi2, Unigene2857\_JingChaoZuZhi2, Unigene28737\_JingChaoZuZhi2, Unigene28793\_JingChaoZuZhi2, Unigene2901\_JingChaoZuZhi2, Unigene30281\_JingChaoZuZhi2, Unigene31612\_JingChaoZuZhi2, Unigene31912\_JingChaoZuZhi2, Unigene31918\_JingChaoZuZhi2, Unigene3235\_JingChaoZuZhi2, Unigene32373\_JingChaoZuZhi2, Unigene324\_JingChaoZuZhi2, Unigene33044\_JingChaoZuZhi2, Unigene33173\_JingChaoZuZhi2, Unigene3321\_JingChaoZuZhi2, Unigene3388\_JingChaoZuZhi2, Unigene34142\_JingChaoZuZhi2, Unigene34785\_JingChaoZuZhi2, Unigene3493\_JingChaoZuZhi2, Unigene35016\_JingChaoZuZhi2, Unigene35080\_JingChaoZuZhi2, Unigene35243\_JingChaoZuZhi2, Unigene35927\_JingChaoZuZhi2, Unigene36257\_JingChaoZuZhi2, Unigene36342\_JingChaoZuZhi2, Unigene36361\_JingChaoZuZhi2, Unigene3646\_JingChaoZuZhi2, Unigene3665\_JingChaoZuZhi2, Unigene3671\_JingChaoZuZhi2, Unigene3693\_JingChaoZuZhi2, Unigene36974\_JingChaoZuZhi2, Unigene36976\_JingChaoZuZhi2, Unigene37164\_JingChaoZuZhi2, Unigene37818\_JingChaoZuZhi2, Unigene3854\_JingChaoZuZhi2, Unigene39102\_JingChaoZuZhi2, Unigene39216\_JingChaoZuZhi2, Unigene4015\_JingChaoZuZhi2, Unigene40200\_JingChaoZuZhi2, Unigene40281\_JingChaoZuZhi2, Unigene4044\_JingChaoZuZhi2, Unigene40919\_JingChaoZuZhi2, Unigene41040\_JingChaoZuZhi2, Unigene41148\_JingChaoZuZhi2, Unigene411\_JingChaoZuZhi2, Unigene41700\_JingChaoZuZhi2, Unigene42146\_JingChaoZuZhi2, Unigene42248\_JingChaoZuZhi2, Unigene4254\_JingChaoZuZhi2, Unigene43041\_JingChaoZuZhi2, Unigene43147\_JingChaoZuZhi2, Unigene43169\_JingChaoZuZhi2, Unigene43249\_JingChaoZuZhi2, Unigene43260\_JingChaoZuZhi2, Unigene4337\_JingChaoZuZhi2, Unigene43512\_JingChaoZuZhi2, Unigene43574\_JingChaoZuZhi2, Unigene44190\_JingChaoZuZhi2, Unigene44336\_JingChaoZuZhi2, Unigene44454\_JingChaoZuZhi2, Unigene44516\_JingChaoZuZhi2, Unigene45134\_JingChaoZuZhi2, Unigene45367\_JingChaoZuZhi2, Unigene4547\_JingChaoZuZhi2, Unigene45593\_JingChaoZuZhi2, Unigene45622\_JingChaoZuZhi2, Unigene45782\_JingChaoZuZhi2, Unigene45901\_JingChaoZuZhi2, Unigene4599\_JingChaoZuZhi2, Unigene46407\_JingChaoZuZhi2, Unigene4713\_JingChaoZuZhi2, Unigene47578\_JingChaoZuZhi2, Unigene4758\_JingChaoZuZhi2, Unigene47949\_JingChaoZuZhi2, Unigene4829\_JingChaoZuZhi2, Unigene4873\_JingChaoZuZhi2, Unigene48751\_JingChaoZuZhi2, Unigene48843\_JingChaoZuZhi2, Unigene49387\_JingChaoZuZhi2, Unigene49973\_JingChaoZuZhi2, Unigene50540\_JingChaoZuZhi2, Unigene50668\_JingChaoZuZhi2, Unigene50744\_JingChaoZuZhi2, Unigene5108\_JingChaoZuZhi2, Unigene51090\_JingChaoZuZhi2, Unigene51115\_JingChaoZuZhi2, Unigene5125\_JingChaoZuZhi2, Unigene5126\_JingChaoZuZhi2, Unigene51467\_JingChaoZuZhi2, Unigene52100\_JingChaoZuZhi2, Unigene52125\_JingChaoZuZhi2, Unigene52456\_JingChaoZuZhi2, Unigene524\_JingChaoZuZhi2, Unigene52638\_JingChaoZuZhi2, Unigene5298\_JingChaoZuZhi2, Unigene53131\_JingChaoZuZhi2, Unigene5335\_JingChaoZuZhi2, Unigene53484\_JingChaoZuZhi2, Unigene53500\_JingChaoZuZhi2, Unigene53524\_JingChaoZuZhi2, Unigene53751\_JingChaoZuZhi2, Unigene53917\_JingChaoZuZhi2, Unigene53958\_JingChaoZuZhi2, Unigene54081\_JingChaoZuZhi2, Unigene54918\_JingChaoZuZhi2, Unigene54974\_JingChaoZuZhi2, Unigene55009\_JingChaoZuZhi2, Unigene55019\_JingChaoZuZhi2, Unigene55385\_JingChaoZuZhi2, Unigene56421\_JingChaoZuZhi2, Unigene56560\_JingChaoZuZhi2, Unigene56660\_JingChaoZuZhi2, Unigene57240\_JingChaoZuZhi2, Unigene57488\_JingChaoZuZhi2, Unigene57597\_JingChaoZuZhi2, Unigene5809\_JingChaoZuZhi2, Unigene58169\_JingChaoZuZhi2, Unigene58474\_JingChaoZuZhi2, Unigene58675\_JingChaoZuZhi2, Unigene5896\_JingChaoZuZhi2, Unigene59101\_JingChaoZuZhi2, Unigene59146\_JingChaoZuZhi2, Unigene59265\_JingChaoZuZhi2, Unigene59336\_JingChaoZuZhi2, Unigene59433\_JingChaoZuZhi2, Unigene59684\_JingChaoZuZhi2, Unigene60121\_JingChaoZuZhi2, Unigene60551\_JingChaoZuZhi2, Unigene60586\_JingChaoZuZhi2, Unigene60607\_JingChaoZuZhi2, Unigene60654\_JingChaoZuZhi2, Unigene6072\_JingChaoZuZhi2, Unigene60930\_JingChaoZuZhi2, Unigene60990\_JingChaoZuZhi2, Unigene61244\_JingChaoZuZhi2, Unigene61474\_JingChaoZuZhi2, Unigene6158\_JingChaoZuZhi2, Unigene6171\_JingChaoZuZhi2, Unigene61879\_JingChaoZuZhi2, Unigene61931\_JingChaoZuZhi2, Unigene62100\_JingChaoZuZhi2, Unigene6215\_JingChaoZuZhi2, Unigene62257\_JingChaoZuZhi2, Unigene6233\_JingChaoZuZhi2, Unigene62606\_JingChaoZuZhi2, Unigene62695\_JingChaoZuZhi2, Unigene63052\_JingChaoZuZhi2, Unigene63145\_JingChaoZuZhi2, Unigene63384\_JingChaoZuZhi2, Unigene63424\_JingChaoZuZhi2, Unigene63584\_JingChaoZuZhi2, Unigene63604\_JingChaoZuZhi2, Unigene63615\_JingChaoZuZhi2, Unigene63663\_JingChaoZuZhi2, Unigene63812\_JingChaoZuZhi2, Unigene63873\_JingChaoZuZhi2, Unigene63925\_JingChaoZuZhi2, Unigene64208\_JingChaoZuZhi2, Unigene6438\_JingChaoZuZhi2, Unigene64452\_JingChaoZuZhi2, Unigene64458\_JingChaoZuZhi2, Unigene64650\_JingChaoZuZhi2, Unigene64692\_JingChaoZuZhi2, Unigene64843\_JingChaoZuZhi2, Unigene6493\_JingChaoZuZhi2, Unigene65022\_JingChaoZuZhi2, Unigene65129\_JingChaoZuZhi2, Unigene65150\_JingChaoZuZhi2, Unigene65187\_JingChaoZuZhi2, Unigene65326\_JingChaoZuZhi2, Unigene65375\_JingChaoZuZhi2, Unigene6539\_JingChaoZuZhi2, Unigene65773\_JingChaoZuZhi2, Unigene6605\_JingChaoZuZhi2, Unigene66087\_JingChaoZuZhi2, Unigene66253\_JingChaoZuZhi2, Unigene6634\_JingChaoZuZhi2, Unigene6651\_JingChaoZuZhi2, Unigene66559\_JingChaoZuZhi2, Unigene66590\_JingChaoZuZhi2, Unigene66813\_JingChaoZuZhi2, Unigene66913\_JingChaoZuZhi2, Unigene66986\_JingChaoZuZhi2, Unigene67005\_JingChaoZuZhi2, Unigene67063\_JingChaoZuZhi2, Unigene67070\_JingChaoZuZhi2, Unigene67249\_JingChaoZuZhi2, Unigene67279\_JingChaoZuZhi2, Unigene67572\_JingChaoZuZhi2, Unigene67777\_JingChaoZuZhi2, Unigene67958\_JingChaoZuZhi2, Unigene6796\_JingChaoZuZhi2, Unigene6797\_JingChaoZuZhi2, Unigene68108\_JingChaoZuZhi2, Unigene68139\_JingChaoZuZhi2, Unigene6815\_JingChaoZuZhi2, Unigene6828\_JingChaoZuZhi2, Unigene68527\_JingChaoZuZhi2, Unigene68672\_JingChaoZuZhi2, Unigene6872\_JingChaoZuZhi2, Unigene69092\_JingChaoZuZhi2, Unigene69100\_JingChaoZuZhi2, Unigene69141\_JingChaoZuZhi2, Unigene6914\_JingChaoZuZhi2, Unigene69221\_JingChaoZuZhi2, Unigene69386\_JingChaoZuZhi2, Unigene69562\_JingChaoZuZhi2, Unigene69775\_JingChaoZuZhi2, Unigene70045\_JingChaoZuZhi2, Unigene70063\_JingChaoZuZhi2, Unigene7016\_JingChaoZuZhi2, Unigene70329\_JingChaoZuZhi2, Unigene7032\_JingChaoZuZhi2, Unigene70970\_JingChaoZuZhi2, Unigene71043\_JingChaoZuZhi2, Unigene7116\_JingChaoZuZhi2, Unigene7118\_JingChaoZuZhi2, Unigene71213\_JingChaoZuZhi2, Unigene71241\_JingChaoZuZhi2, Unigene71385\_JingChaoZuZhi2, Unigene7151\_JingChaoZuZhi2, Unigene71546\_JingChaoZuZhi2, Unigene71592\_JingChaoZuZhi2, Unigene71729\_JingChaoZuZhi2, Unigene71774\_JingChaoZuZhi2, Unigene71808\_JingChaoZuZhi2, Unigene7206\_JingChaoZuZhi2, Unigene72249\_JingChaoZuZhi2, Unigene72334\_JingChaoZuZhi2, Unigene72351\_JingChaoZuZhi2, Unigene72503\_JingChaoZuZhi2, Unigene7268\_JingChaoZuZhi2, Unigene72708\_JingChaoZuZhi2, Unigene72832\_JingChaoZuZhi2, Unigene73178\_JingChaoZuZhi2, Unigene73257\_JingChaoZuZhi2, Unigene7326\_JingChaoZuZhi2, Unigene73446\_JingChaoZuZhi2, Unigene73475\_JingChaoZuZhi2, Unigene73550\_JingChaoZuZhi2, Unigene73620\_JingChaoZuZhi2, Unigene73817\_JingChaoZuZhi2, Unigene73887\_JingChaoZuZhi2, Unigene74050\_JingChaoZuZhi2, Unigene741\_JingChaoZuZhi2, Unigene74221\_JingChaoZuZhi2, Unigene74259\_JingChaoZuZhi2, Unigene74298\_JingChaoZuZhi2, Unigene74474\_JingChaoZuZhi2, Unigene74475\_JingChaoZuZhi2, Unigene74719\_JingChaoZuZhi2, Unigene74730\_JingChaoZuZhi2, Unigene74854\_JingChaoZuZhi2, Unigene7489\_JingChaoZuZhi2, Unigene75009\_JingChaoZuZhi2, Unigene7508\_JingChaoZuZhi2, Unigene75112\_JingChaoZuZhi2, Unigene75167\_JingChaoZuZhi2, Unigene75491\_JingChaoZuZhi2, Unigene75577\_JingChaoZuZhi2, Unigene75840\_JingChaoZuZhi2, Unigene75902\_JingChaoZuZhi2, Unigene75946\_JingChaoZuZhi2, Unigene76002\_JingChaoZuZhi2, Unigene76111\_JingChaoZuZhi2, Unigene76269\_JingChaoZuZhi2, Unigene76302\_JingChaoZuZhi2, Unigene7634\_JingChaoZuZhi2, Unigene76400\_JingChaoZuZhi2, Unigene76515\_JingChaoZuZhi2, Unigene7663\_JingChaoZuZhi2, Unigene76776\_JingChaoZuZhi2, Unigene76785\_JingChaoZuZhi2, Unigene76809\_JingChaoZuZhi2, Unigene76819\_JingChaoZuZhi2, Unigene76917\_JingChaoZuZhi2, Unigene77061\_JingChaoZuZhi2, Unigene77406\_JingChaoZuZhi2, Unigene77413\_JingChaoZuZhi2, Unigene77510\_JingChaoZuZhi2, Unigene77520\_JingChaoZuZhi2, Unigene77712\_JingChaoZuZhi2, Unigene7794\_JingChaoZuZhi2, Unigene77954\_JingChaoZuZhi2, Unigene78009\_JingChaoZuZhi2, Unigene78288\_JingChaoZuZhi2, Unigene78325\_JingChaoZuZhi2, Unigene78361\_JingChaoZuZhi2, Unigene78410\_JingChaoZuZhi2, Unigene7850\_JingChaoZuZhi2, Unigene78521\_JingChaoZuZhi2, Unigene78555\_JingChaoZuZhi2, Unigene78636\_JingChaoZuZhi2, Unigene78909\_JingChaoZuZhi2, Unigene79058\_JingChaoZuZhi2, Unigene79118\_JingChaoZuZhi2, Unigene7911\_JingChaoZuZhi2, Unigene79141\_JingChaoZuZhi2, Unigene79358\_JingChaoZuZhi2, Unigene7939\_JingChaoZuZhi2, Unigene79411\_JingChaoZuZhi2, Unigene79433\_JingChaoZuZhi2, Unigene79443\_JingChaoZuZhi2, Unigene79653\_JingChaoZuZhi2, Unigene79683\_JingChaoZuZhi2, Unigene79726\_JingChaoZuZhi2, Unigene79789\_JingChaoZuZhi2, Unigene79824\_JingChaoZuZhi2, Unigene79864\_JingChaoZuZhi2, Unigene79871\_JingChaoZuZhi2, Unigene7987\_JingChaoZuZhi2, Unigene79882\_JingChaoZuZhi2, Unigene79940\_JingChaoZuZhi2, Unigene80095\_JingChaoZuZhi2, Unigene80143\_JingChaoZuZhi2, Unigene80212\_JingChaoZuZhi2, Unigene80403\_JingChaoZuZhi2, Unigene80619\_JingChaoZuZhi2, Unigene80893\_JingChaoZuZhi2, Unigene80916\_JingChaoZuZhi2, Unigene80980\_JingChaoZuZhi2, Unigene81145\_JingChaoZuZhi2, Unigene81167\_JingChaoZuZhi2, Unigene81253\_JingChaoZuZhi2, Unigene81271\_JingChaoZuZhi2, Unigene81327\_JingChaoZuZhi2, Unigene8133\_JingChaoZuZhi2, Unigene81372\_JingChaoZuZhi2, Unigene81434\_JingChaoZuZhi2, Unigene81440\_JingChaoZuZhi2, Unigene81450\_JingChaoZuZhi2, Unigene81561\_JingChaoZuZhi2, Unigene81599\_JingChaoZuZhi2, Unigene81686\_JingChaoZuZhi2, Unigene81757\_JingChaoZuZhi2, Unigene81803\_JingChaoZuZhi2, Unigene81825\_JingChaoZuZhi2, Unigene81904\_JingChaoZuZhi2, Unigene82047\_JingChaoZuZhi2, Unigene82070\_JingChaoZuZhi2, Unigene82111\_JingChaoZuZhi2, Unigene82117\_JingChaoZuZhi2, Unigene82215\_JingChaoZuZhi2, Unigene8237\_JingChaoZuZhi2, Unigene82489\_JingChaoZuZhi2, Unigene82521\_JingChaoZuZhi2, Unigene82578\_JingChaoZuZhi2, Unigene82605\_JingChaoZuZhi2, Unigene82662\_JingChaoZuZhi2, Unigene82688\_JingChaoZuZhi2, Unigene82760\_JingChaoZuZhi2, Unigene82921\_JingChaoZuZhi2, Unigene83079\_JingChaoZuZhi2, Unigene83214\_JingChaoZuZhi2, Unigene83433\_JingChaoZuZhi2, Unigene83766\_JingChaoZuZhi2, Unigene83782\_JingChaoZuZhi2, Unigene83825\_JingChaoZuZhi2, Unigene83872\_JingChaoZuZhi2, Unigene8392\_JingChaoZuZhi2, Unigene8406\_JingChaoZuZhi2, Unigene84076\_JingChaoZuZhi2, Unigene84109\_JingChaoZuZhi2, Unigene84152\_JingChaoZuZhi2, Unigene84168\_JingChaoZuZhi2, Unigene841\_JingChaoZuZhi2, Unigene84212\_JingChaoZuZhi2, Unigene8443\_JingChaoZuZhi2, Unigene84519\_JingChaoZuZhi2, Unigene84533\_JingChaoZuZhi2, Unigene84566\_JingChaoZuZhi2, Unigene84748\_JingChaoZuZhi2, Unigene84760\_JingChaoZuZhi2, Unigene84924\_JingChaoZuZhi2, Unigene84945\_JingChaoZuZhi2, Unigene84993\_JingChaoZuZhi2, Unigene85193\_JingChaoZuZhi2, Unigene851\_JingChaoZuZhi2, Unigene85314\_JingChaoZuZhi2, Unigene85577\_JingChaoZuZhi2, Unigene85770\_JingChaoZuZhi2, Unigene85902\_JingChaoZuZhi2, Unigene86020\_JingChaoZuZhi2, Unigene86091\_JingChaoZuZhi2, Unigene86145\_JingChaoZuZhi2, Unigene86206\_JingChaoZuZhi2, Unigene86349\_JingChaoZuZhi2, Unigene86526\_JingChaoZuZhi2, Unigene86546\_JingChaoZuZhi2, Unigene86579\_JingChaoZuZhi2, Unigene8658\_JingChaoZuZhi2, Unigene8661\_JingChaoZuZhi2, Unigene86669\_JingChaoZuZhi2, Unigene86741\_JingChaoZuZhi2, Unigene87082\_JingChaoZuZhi2, Unigene8709\_JingChaoZuZhi2, Unigene87203\_JingChaoZuZhi2, Unigene87254\_JingChaoZuZhi2, Unigene87256\_JingChaoZuZhi2, Unigene87362\_JingChaoZuZhi2, Unigene87365\_JingChaoZuZhi2, Unigene87459\_JingChaoZuZhi2, Unigene87478\_JingChaoZuZhi2, Unigene87488\_JingChaoZuZhi2, Unigene87533\_JingChaoZuZhi2, Unigene87774\_JingChaoZuZhi2, Unigene87853\_JingChaoZuZhi2, Unigene87946\_JingChaoZuZhi2, Unigene88059\_JingChaoZuZhi2, Unigene88169\_JingChaoZuZhi2, Unigene88185\_JingChaoZuZhi2, Unigene88307\_JingChaoZuZhi2, Unigene8833\_JingChaoZuZhi2, Unigene88342\_JingChaoZuZhi2, Unigene88391\_JingChaoZuZhi2, Unigene88400\_JingChaoZuZhi2, Unigene88406\_JingChaoZuZhi2, Unigene88442\_JingChaoZuZhi2, Unigene88470\_JingChaoZuZhi2, Unigene88484\_JingChaoZuZhi2, Unigene88608\_JingChaoZuZhi2, Unigene88683\_JingChaoZuZhi2, Unigene88738\_JingChaoZuZhi2, Unigene88741\_JingChaoZuZhi2, Unigene88778\_JingChaoZuZhi2, Unigene88779\_JingChaoZuZhi2, Unigene88781\_JingChaoZuZhi2, Unigene88797\_JingChaoZuZhi2, Unigene88830\_JingChaoZuZhi2, Unigene88837\_JingChaoZuZhi2, Unigene88887\_JingChaoZuZhi2, Unigene88945\_JingChaoZuZhi2, Unigene88979\_JingChaoZuZhi2, Unigene88988\_JingChaoZuZhi2, Unigene89013\_JingChaoZuZhi2, Unigene89060\_JingChaoZuZhi2, Unigene8907\_JingChaoZuZhi2, Unigene89093\_JingChaoZuZhi2, Unigene89121\_JingChaoZuZhi2, Unigene89323\_JingChaoZuZhi2, Unigene89337\_JingChaoZuZhi2, Unigene89352\_JingChaoZuZhi2, Unigene89367\_JingChaoZuZhi2, Unigene89541\_JingChaoZuZhi2, Unigene89606\_JingChaoZuZhi2, Unigene89629\_JingChaoZuZhi2, Unigene89746\_JingChaoZuZhi2, Unigene89769\_JingChaoZuZhi2, Unigene89834\_JingChaoZuZhi2, Unigene89867\_JingChaoZuZhi2, Unigene89956\_JingChaoZuZhi2, Unigene90027\_JingChaoZuZhi2, Unigene90038\_JingChaoZuZhi2, Unigene90066\_JingChaoZuZhi2, Unigene90071\_JingChaoZuZhi2, Unigene90209\_JingChaoZuZhi2, Unigene90341\_JingChaoZuZhi2, Unigene9050\_JingChaoZuZhi2, Unigene90564\_JingChaoZuZhi2, Unigene90714\_JingChaoZuZhi2, Unigene90779\_JingChaoZuZhi2, Unigene90923\_JingChaoZuZhi2, Unigene90953\_JingChaoZuZhi2, Unigene90979\_JingChaoZuZhi2, Unigene90995\_JingChaoZuZhi2, Unigene91077\_JingChaoZuZhi2, Unigene91099\_JingChaoZuZhi2, Unigene91148\_JingChaoZuZhi2, Unigene91163\_JingChaoZuZhi2, Unigene91179\_JingChaoZuZhi2, Unigene91233\_JingChaoZuZhi2, Unigene91390\_JingChaoZuZhi2, Unigene91456\_JingChaoZuZhi2, Unigene91475\_JingChaoZuZhi2, Unigene91487\_JingChaoZuZhi2, Unigene91529\_JingChaoZuZhi2, Unigene91553\_JingChaoZuZhi2, Unigene91848\_JingChaoZuZhi2, Unigene92059\_JingChaoZuZhi2, Unigene92073\_JingChaoZuZhi2, Unigene92112\_JingChaoZuZhi2, Unigene92346\_JingChaoZuZhi2, Unigene92443\_JingChaoZuZhi2, Unigene92580\_JingChaoZuZhi2, Unigene92681\_JingChaoZuZhi2, Unigene92700\_JingChaoZuZhi2, Unigene92780\_JingChaoZuZhi2, Unigene92832\_JingChaoZuZhi2, Unigene92838\_JingChaoZuZhi2, Unigene92951\_JingChaoZuZhi2, Unigene93073\_JingChaoZuZhi2, Unigene93246\_JingChaoZuZhi2, Unigene93552\_JingChaoZuZhi2, Unigene93562\_JingChaoZuZhi2, Unigene93573\_JingChaoZuZhi2, Unigene93592\_JingChaoZuZhi2, Unigene93610\_JingChaoZuZhi2, Unigene93702\_JingChaoZuZhi2, Unigene93742\_JingChaoZuZhi2, Unigene93780\_JingChaoZuZhi2, Unigene93782\_JingChaoZuZhi2, Unigene93793\_JingChaoZuZhi2, Unigene93807\_JingChaoZuZhi2, Unigene93853\_JingChaoZuZhi2, Unigene93953\_JingChaoZuZhi2, Unigene93979\_JingChaoZuZhi2, Unigene94036\_JingChaoZuZhi2, Unigene9404\_JingChaoZuZhi2, Unigene94083\_JingChaoZuZhi2, Unigene94110\_JingChaoZuZhi2, Unigene94120\_JingChaoZuZhi2, Unigene94129\_JingChaoZuZhi2, Unigene94134\_JingChaoZuZhi2, Unigene94208\_JingChaoZuZhi2, Unigene94272\_JingChaoZuZhi2, Unigene94404\_JingChaoZuZhi2, Unigene94471\_JingChaoZuZhi2, Unigene94520\_JingChaoZuZhi2, Unigene94544\_JingChaoZuZhi2, Unigene94561\_JingChaoZuZhi2, Unigene94584\_JingChaoZuZhi2, Unigene94685\_JingChaoZuZhi2, Unigene94687\_JingChaoZuZhi2, Unigene94891\_JingChaoZuZhi2, Unigene9490\_JingChaoZuZhi2, Unigene94950\_JingChaoZuZhi2, Unigene94968\_JingChaoZuZhi2, Unigene95088\_JingChaoZuZhi2, Unigene95098\_JingChaoZuZhi2, Unigene95119\_JingChaoZuZhi2, Unigene951\_JingChaoZuZhi2, Unigene95203\_JingChaoZuZhi2, Unigene95371\_JingChaoZuZhi2, Unigene9540\_JingChaoZuZhi2, Unigene95589\_JingChaoZuZhi2, Unigene95633\_JingChaoZuZhi2, Unigene95686\_JingChaoZuZhi2, Unigene95782\_JingChaoZuZhi2, Unigene95812\_JingChaoZuZhi2, Unigene95875\_JingChaoZuZhi2, Unigene95881\_JingChaoZuZhi2, Unigene95907\_JingChaoZuZhi2, Unigene96068\_JingChaoZuZhi2, Unigene96114\_JingChaoZuZhi2, Unigene96121\_JingChaoZuZhi2, Unigene96193\_JingChaoZuZhi2, Unigene96234\_JingChaoZuZhi2, Unigene96248\_JingChaoZuZhi2, Unigene96294\_JingChaoZuZhi2, Unigene9688\_JingChaoZuZhi2, Unigene9739\_JingChaoZuZhi2, Unigene9743\_JingChaoZuZhi2, Unigene9807\_JingChaoZuZhi2, Unigene9833\_JingChaoZuZhi2, Unigene9988\_JingChaoZuZhi2, Unigene9992\_JingChaoZuZhi2 |
| 12 | Pathogenic Escherichia coli infection | Unigene10047\_JingChaoZuZhi2, Unigene10051\_JingChaoZuZhi2, Unigene10128\_JingChaoZuZhi2, Unigene10259\_JingChaoZuZhi2, Unigene10318\_JingChaoZuZhi2, Unigene10456\_JingChaoZuZhi2, Unigene10492\_JingChaoZuZhi2, Unigene10498\_JingChaoZuZhi2, Unigene10541\_JingChaoZuZhi2, Unigene10576\_JingChaoZuZhi2, Unigene10582\_JingChaoZuZhi2, Unigene1060\_JingChaoZuZhi2, Unigene10733\_JingChaoZuZhi2, Unigene10784\_JingChaoZuZhi2, Unigene10926\_JingChaoZuZhi2, Unigene10936\_JingChaoZuZhi2, Unigene10974\_JingChaoZuZhi2, Unigene10979\_JingChaoZuZhi2, Unigene10\_JingChaoZuZhi2, Unigene11240\_JingChaoZuZhi2, Unigene11446\_JingChaoZuZhi2, Unigene11569\_JingChaoZuZhi2, Unigene115\_JingChaoZuZhi2, Unigene1162\_JingChaoZuZhi2, Unigene11641\_JingChaoZuZhi2, Unigene1173\_JingChaoZuZhi2, Unigene11810\_JingChaoZuZhi2, Unigene11898\_JingChaoZuZhi2, Unigene11902\_JingChaoZuZhi2, Unigene11918\_JingChaoZuZhi2, Unigene11932\_JingChaoZuZhi2, Unigene11995\_JingChaoZuZhi2, Unigene12060\_JingChaoZuZhi2, Unigene12099\_JingChaoZuZhi2, Unigene12148\_JingChaoZuZhi2, Unigene12285\_JingChaoZuZhi2, Unigene12310\_JingChaoZuZhi2, Unigene12326\_JingChaoZuZhi2, Unigene12372\_JingChaoZuZhi2, Unigene12390\_JingChaoZuZhi2, Unigene12414\_JingChaoZuZhi2, Unigene12644\_JingChaoZuZhi2, Unigene13097\_JingChaoZuZhi2, Unigene13196\_JingChaoZuZhi2, Unigene13249\_JingChaoZuZhi2, Unigene1325\_JingChaoZuZhi2, Unigene13478\_JingChaoZuZhi2, Unigene13572\_JingChaoZuZhi2, Unigene13607\_JingChaoZuZhi2, Unigene13618\_JingChaoZuZhi2, Unigene13650\_JingChaoZuZhi2, Unigene13766\_JingChaoZuZhi2, Unigene13906\_JingChaoZuZhi2, Unigene13927\_JingChaoZuZhi2, Unigene13982\_JingChaoZuZhi2, Unigene14159\_JingChaoZuZhi2, Unigene14193\_JingChaoZuZhi2, Unigene14246\_JingChaoZuZhi2, Unigene14450\_JingChaoZuZhi2, Unigene14461\_JingChaoZuZhi2, Unigene14476\_JingChaoZuZhi2, Unigene14534\_JingChaoZuZhi2, Unigene14610\_JingChaoZuZhi2, Unigene14714\_JingChaoZuZhi2, Unigene14715\_JingChaoZuZhi2, Unigene14970\_JingChaoZuZhi2, Unigene15088\_JingChaoZuZhi2, Unigene15096\_JingChaoZuZhi2, Unigene15176\_JingChaoZuZhi2, Unigene15182\_JingChaoZuZhi2, Unigene15251\_JingChaoZuZhi2, Unigene15288\_JingChaoZuZhi2, Unigene15319\_JingChaoZuZhi2, Unigene15374\_JingChaoZuZhi2, Unigene15381\_JingChaoZuZhi2, Unigene15470\_JingChaoZuZhi2, Unigene15492\_JingChaoZuZhi2, Unigene15615\_JingChaoZuZhi2, Unigene15650\_JingChaoZuZhi2, Unigene15651\_JingChaoZuZhi2, Unigene15731\_JingChaoZuZhi2, Unigene1579\_JingChaoZuZhi2, Unigene15857\_JingChaoZuZhi2, Unigene15879\_JingChaoZuZhi2, Unigene16004\_JingChaoZuZhi2, Unigene16101\_JingChaoZuZhi2, Unigene16103\_JingChaoZuZhi2, Unigene16176\_JingChaoZuZhi2, Unigene16182\_JingChaoZuZhi2, Unigene16402\_JingChaoZuZhi2, Unigene16478\_JingChaoZuZhi2, Unigene16481\_JingChaoZuZhi2, Unigene16560\_JingChaoZuZhi2, Unigene16591\_JingChaoZuZhi2, Unigene16655\_JingChaoZuZhi2, Unigene16730\_JingChaoZuZhi2, Unigene16873\_JingChaoZuZhi2, Unigene16950\_JingChaoZuZhi2, Unigene16967\_JingChaoZuZhi2, Unigene17101\_JingChaoZuZhi2, Unigene17501\_JingChaoZuZhi2, Unigene17658\_JingChaoZuZhi2, Unigene17764\_JingChaoZuZhi2, Unigene17789\_JingChaoZuZhi2, Unigene17845\_JingChaoZuZhi2, Unigene17894\_JingChaoZuZhi2, Unigene18128\_JingChaoZuZhi2, Unigene18138\_JingChaoZuZhi2, Unigene18256\_JingChaoZuZhi2, Unigene18259\_JingChaoZuZhi2, Unigene18269\_JingChaoZuZhi2, Unigene18275\_JingChaoZuZhi2, Unigene18334\_JingChaoZuZhi2, Unigene18348\_JingChaoZuZhi2, Unigene18505\_JingChaoZuZhi2, Unigene18648\_JingChaoZuZhi2, Unigene18662\_JingChaoZuZhi2, Unigene18685\_JingChaoZuZhi2, Unigene18790\_JingChaoZuZhi2, Unigene18835\_JingChaoZuZhi2, Unigene19001\_JingChaoZuZhi2, Unigene19048\_JingChaoZuZhi2, Unigene19086\_JingChaoZuZhi2, Unigene19137\_JingChaoZuZhi2, Unigene19185\_JingChaoZuZhi2, Unigene19204\_JingChaoZuZhi2, Unigene19223\_JingChaoZuZhi2, Unigene19304\_JingChaoZuZhi2, Unigene19319\_JingChaoZuZhi2, Unigene19391\_JingChaoZuZhi2, Unigene19533\_JingChaoZuZhi2, Unigene19701\_JingChaoZuZhi2, Unigene19883\_JingChaoZuZhi2, Unigene19897\_JingChaoZuZhi2, Unigene19979\_JingChaoZuZhi2, Unigene1999\_JingChaoZuZhi2, Unigene20030\_JingChaoZuZhi2, Unigene20049\_JingChaoZuZhi2, Unigene20083\_JingChaoZuZhi2, Unigene20086\_JingChaoZuZhi2, Unigene201\_JingChaoZuZhi2, Unigene20220\_JingChaoZuZhi2, Unigene20239\_JingChaoZuZhi2, Unigene20353\_JingChaoZuZhi2, Unigene20373\_JingChaoZuZhi2, Unigene20375\_JingChaoZuZhi2, Unigene20454\_JingChaoZuZhi2, Unigene20468\_JingChaoZuZhi2, Unigene20474\_JingChaoZuZhi2, Unigene20508\_JingChaoZuZhi2, Unigene20557\_JingChaoZuZhi2, Unigene20608\_JingChaoZuZhi2, Unigene20727\_JingChaoZuZhi2, Unigene20826\_JingChaoZuZhi2, Unigene20831\_JingChaoZuZhi2, Unigene20837\_JingChaoZuZhi2, Unigene20863\_JingChaoZuZhi2, Unigene20943\_JingChaoZuZhi2, Unigene21041\_JingChaoZuZhi2, Unigene21045\_JingChaoZuZhi2, Unigene21057\_JingChaoZuZhi2, Unigene2115\_JingChaoZuZhi2, Unigene21290\_JingChaoZuZhi2, Unigene21311\_JingChaoZuZhi2, Unigene21358\_JingChaoZuZhi2, Unigene21472\_JingChaoZuZhi2, Unigene21475\_JingChaoZuZhi2, Unigene21515\_JingChaoZuZhi2, Unigene21543\_JingChaoZuZhi2, Unigene21578\_JingChaoZuZhi2, Unigene21605\_JingChaoZuZhi2, Unigene2161\_JingChaoZuZhi2, Unigene21636\_JingChaoZuZhi2, Unigene21818\_JingChaoZuZhi2, Unigene21860\_JingChaoZuZhi2, Unigene21913\_JingChaoZuZhi2, Unigene21932\_JingChaoZuZhi2, Unigene21933\_JingChaoZuZhi2, Unigene22055\_JingChaoZuZhi2, Unigene22085\_JingChaoZuZhi2, Unigene22119\_JingChaoZuZhi2, Unigene22128\_JingChaoZuZhi2, Unigene22220\_JingChaoZuZhi2, Unigene2226\_JingChaoZuZhi2, Unigene22329\_JingChaoZuZhi2, Unigene22372\_JingChaoZuZhi2, Unigene22414\_JingChaoZuZhi2, Unigene22455\_JingChaoZuZhi2, Unigene22487\_JingChaoZuZhi2, Unigene22586\_JingChaoZuZhi2, Unigene2258\_JingChaoZuZhi2, Unigene22644\_JingChaoZuZhi2, Unigene22779\_JingChaoZuZhi2, Unigene2289\_JingChaoZuZhi2, Unigene22902\_JingChaoZuZhi2, Unigene22909\_JingChaoZuZhi2, Unigene23164\_JingChaoZuZhi2, Unigene2330\_JingChaoZuZhi2, Unigene23447\_JingChaoZuZhi2, Unigene234\_JingChaoZuZhi2, Unigene23827\_JingChaoZuZhi2, Unigene23924\_JingChaoZuZhi2, Unigene23978\_JingChaoZuZhi2, Unigene2445\_JingChaoZuZhi2, Unigene24600\_JingChaoZuZhi2, Unigene2510\_JingChaoZuZhi2, Unigene25693\_JingChaoZuZhi2, Unigene25912\_JingChaoZuZhi2, Unigene25962\_JingChaoZuZhi2, Unigene2656\_JingChaoZuZhi2, Unigene26592\_JingChaoZuZhi2, Unigene26730\_JingChaoZuZhi2, Unigene26746\_JingChaoZuZhi2, Unigene26874\_JingChaoZuZhi2, Unigene26987\_JingChaoZuZhi2, Unigene28064\_JingChaoZuZhi2, Unigene28243\_JingChaoZuZhi2, Unigene2857\_JingChaoZuZhi2, Unigene28793\_JingChaoZuZhi2, Unigene2901\_JingChaoZuZhi2, Unigene29295\_JingChaoZuZhi2, Unigene2960\_JingChaoZuZhi2, Unigene30281\_JingChaoZuZhi2, Unigene3102\_JingChaoZuZhi2, Unigene31285\_JingChaoZuZhi2, Unigene31868\_JingChaoZuZhi2, Unigene31912\_JingChaoZuZhi2, Unigene31918\_JingChaoZuZhi2, Unigene32373\_JingChaoZuZhi2, Unigene324\_JingChaoZuZhi2, Unigene32941\_JingChaoZuZhi2, Unigene33044\_JingChaoZuZhi2, Unigene33173\_JingChaoZuZhi2, Unigene3321\_JingChaoZuZhi2, Unigene33692\_JingChaoZuZhi2, Unigene3388\_JingChaoZuZhi2, Unigene33963\_JingChaoZuZhi2, Unigene34142\_JingChaoZuZhi2, Unigene34785\_JingChaoZuZhi2, Unigene3493\_JingChaoZuZhi2, Unigene35016\_JingChaoZuZhi2, Unigene35080\_JingChaoZuZhi2, Unigene35243\_JingChaoZuZhi2, Unigene35927\_JingChaoZuZhi2, Unigene36257\_JingChaoZuZhi2, Unigene36342\_JingChaoZuZhi2, Unigene36354\_JingChaoZuZhi2, Unigene36361\_JingChaoZuZhi2, Unigene3646\_JingChaoZuZhi2, Unigene3665\_JingChaoZuZhi2, Unigene3671\_JingChaoZuZhi2, Unigene3693\_JingChaoZuZhi2, Unigene36974\_JingChaoZuZhi2, Unigene36976\_JingChaoZuZhi2, Unigene37164\_JingChaoZuZhi2, Unigene37252\_JingChaoZuZhi2, Unigene37639\_JingChaoZuZhi2, Unigene37818\_JingChaoZuZhi2, Unigene38458\_JingChaoZuZhi2, Unigene3854\_JingChaoZuZhi2, Unigene38665\_JingChaoZuZhi2, Unigene39102\_JingChaoZuZhi2, Unigene39216\_JingChaoZuZhi2, Unigene4015\_JingChaoZuZhi2, Unigene40281\_JingChaoZuZhi2, Unigene4028\_JingChaoZuZhi2, Unigene4044\_JingChaoZuZhi2, Unigene40674\_JingChaoZuZhi2, Unigene40919\_JingChaoZuZhi2, Unigene41148\_JingChaoZuZhi2, Unigene41171\_JingChaoZuZhi2, Unigene411\_JingChaoZuZhi2, Unigene41953\_JingChaoZuZhi2, Unigene41973\_JingChaoZuZhi2, Unigene42146\_JingChaoZuZhi2, Unigene42248\_JingChaoZuZhi2, Unigene42443\_JingChaoZuZhi2, Unigene43041\_JingChaoZuZhi2, Unigene43147\_JingChaoZuZhi2, Unigene43169\_JingChaoZuZhi2, Unigene43249\_JingChaoZuZhi2, Unigene43260\_JingChaoZuZhi2, Unigene4337\_JingChaoZuZhi2, Unigene43512\_JingChaoZuZhi2, Unigene43574\_JingChaoZuZhi2, Unigene43584\_JingChaoZuZhi2, Unigene4378\_JingChaoZuZhi2, Unigene44190\_JingChaoZuZhi2, Unigene44336\_JingChaoZuZhi2, Unigene44516\_JingChaoZuZhi2, Unigene45086\_JingChaoZuZhi2, Unigene45367\_JingChaoZuZhi2, Unigene4547\_JingChaoZuZhi2, Unigene45593\_JingChaoZuZhi2, Unigene45622\_JingChaoZuZhi2, Unigene45901\_JingChaoZuZhi2, Unigene46155\_JingChaoZuZhi2, Unigene4643\_JingChaoZuZhi2, Unigene46745\_JingChaoZuZhi2, Unigene4713\_JingChaoZuZhi2, Unigene47578\_JingChaoZuZhi2, Unigene4758\_JingChaoZuZhi2, Unigene47949\_JingChaoZuZhi2, Unigene4800\_JingChaoZuZhi2, Unigene4829\_JingChaoZuZhi2, Unigene4873\_JingChaoZuZhi2, Unigene48843\_JingChaoZuZhi2, Unigene49056\_JingChaoZuZhi2, Unigene49387\_JingChaoZuZhi2, Unigene49973\_JingChaoZuZhi2, Unigene50084\_JingChaoZuZhi2, Unigene50540\_JingChaoZuZhi2, Unigene50668\_JingChaoZuZhi2, Unigene50744\_JingChaoZuZhi2, Unigene51090\_JingChaoZuZhi2, Unigene51115\_JingChaoZuZhi2, Unigene51164\_JingChaoZuZhi2, Unigene5125\_JingChaoZuZhi2, Unigene5126\_JingChaoZuZhi2, Unigene51467\_JingChaoZuZhi2, Unigene5158\_JingChaoZuZhi2, Unigene51992\_JingChaoZuZhi2, Unigene52195\_JingChaoZuZhi2, Unigene52408\_JingChaoZuZhi2, Unigene52456\_JingChaoZuZhi2, Unigene52758\_JingChaoZuZhi2, Unigene52759\_JingChaoZuZhi2, Unigene5298\_JingChaoZuZhi2, Unigene5335\_JingChaoZuZhi2, Unigene53484\_JingChaoZuZhi2, Unigene53553\_JingChaoZuZhi2, Unigene53751\_JingChaoZuZhi2, Unigene54918\_JingChaoZuZhi2, Unigene54974\_JingChaoZuZhi2, Unigene55009\_JingChaoZuZhi2, Unigene55385\_JingChaoZuZhi2, Unigene55446\_JingChaoZuZhi2, Unigene56079\_JingChaoZuZhi2, Unigene56560\_JingChaoZuZhi2, Unigene56660\_JingChaoZuZhi2, Unigene57240\_JingChaoZuZhi2, Unigene57597\_JingChaoZuZhi2, Unigene5797\_JingChaoZuZhi2, Unigene58011\_JingChaoZuZhi2, Unigene5809\_JingChaoZuZhi2, Unigene58169\_JingChaoZuZhi2, Unigene58322\_JingChaoZuZhi2, Unigene58474\_JingChaoZuZhi2, Unigene58608\_JingChaoZuZhi2, Unigene58675\_JingChaoZuZhi2, Unigene58717\_JingChaoZuZhi2, Unigene58988\_JingChaoZuZhi2, Unigene59146\_JingChaoZuZhi2, Unigene59265\_JingChaoZuZhi2, Unigene5926\_JingChaoZuZhi2, Unigene59336\_JingChaoZuZhi2, Unigene59433\_JingChaoZuZhi2, Unigene59684\_JingChaoZuZhi2, Unigene60045\_JingChaoZuZhi2, Unigene60121\_JingChaoZuZhi2, Unigene60350\_JingChaoZuZhi2, Unigene60551\_JingChaoZuZhi2, Unigene60586\_JingChaoZuZhi2, Unigene6072\_JingChaoZuZhi2, Unigene60990\_JingChaoZuZhi2, Unigene61244\_JingChaoZuZhi2, Unigene61474\_JingChaoZuZhi2, Unigene6171\_JingChaoZuZhi2, Unigene61931\_JingChaoZuZhi2, Unigene62066\_JingChaoZuZhi2, Unigene62100\_JingChaoZuZhi2, Unigene6213\_JingChaoZuZhi2, Unigene6215\_JingChaoZuZhi2, Unigene6233\_JingChaoZuZhi2, Unigene62606\_JingChaoZuZhi2, Unigene62695\_JingChaoZuZhi2, Unigene62821\_JingChaoZuZhi2, Unigene63052\_JingChaoZuZhi2, Unigene63149\_JingChaoZuZhi2, Unigene63409\_JingChaoZuZhi2, Unigene63424\_JingChaoZuZhi2, Unigene63604\_JingChaoZuZhi2, Unigene63630\_JingChaoZuZhi2, Unigene63812\_JingChaoZuZhi2, Unigene63873\_JingChaoZuZhi2, Unigene63925\_JingChaoZuZhi2, Unigene64164\_JingChaoZuZhi2, Unigene64208\_JingChaoZuZhi2, Unigene64452\_JingChaoZuZhi2, Unigene64458\_JingChaoZuZhi2, Unigene64830\_JingChaoZuZhi2, Unigene64843\_JingChaoZuZhi2, Unigene648\_JingChaoZuZhi2, Unigene6493\_JingChaoZuZhi2, Unigene65129\_JingChaoZuZhi2, Unigene65150\_JingChaoZuZhi2, Unigene65187\_JingChaoZuZhi2, Unigene65326\_JingChaoZuZhi2, Unigene65375\_JingChaoZuZhi2, Unigene65652\_JingChaoZuZhi2, Unigene65773\_JingChaoZuZhi2, Unigene6605\_JingChaoZuZhi2, Unigene66253\_JingChaoZuZhi2, Unigene6634\_JingChaoZuZhi2, Unigene6651\_JingChaoZuZhi2, Unigene66559\_JingChaoZuZhi2, Unigene66590\_JingChaoZuZhi2, Unigene66986\_JingChaoZuZhi2, Unigene67005\_JingChaoZuZhi2, Unigene67063\_JingChaoZuZhi2, Unigene67070\_JingChaoZuZhi2, Unigene67249\_JingChaoZuZhi2, Unigene67279\_JingChaoZuZhi2, Unigene67309\_JingChaoZuZhi2, Unigene67572\_JingChaoZuZhi2, Unigene67777\_JingChaoZuZhi2, Unigene67804\_JingChaoZuZhi2, Unigene67958\_JingChaoZuZhi2, Unigene6796\_JingChaoZuZhi2, Unigene6797\_JingChaoZuZhi2, Unigene68108\_JingChaoZuZhi2, Unigene68139\_JingChaoZuZhi2, Unigene6815\_JingChaoZuZhi2, Unigene6828\_JingChaoZuZhi2, Unigene68527\_JingChaoZuZhi2, Unigene68672\_JingChaoZuZhi2, Unigene68689\_JingChaoZuZhi2, Unigene6872\_JingChaoZuZhi2, Unigene69092\_JingChaoZuZhi2, Unigene69100\_JingChaoZuZhi2, Unigene69141\_JingChaoZuZhi2, Unigene6914\_JingChaoZuZhi2, Unigene69221\_JingChaoZuZhi2, Unigene69386\_JingChaoZuZhi2, Unigene69402\_JingChaoZuZhi2, Unigene6954\_JingChaoZuZhi2, Unigene69562\_JingChaoZuZhi2, Unigene69775\_JingChaoZuZhi2, Unigene70063\_JingChaoZuZhi2, Unigene7016\_JingChaoZuZhi2, Unigene70329\_JingChaoZuZhi2, Unigene7032\_JingChaoZuZhi2, Unigene70385\_JingChaoZuZhi2, Unigene70636\_JingChaoZuZhi2, Unigene70723\_JingChaoZuZhi2, Unigene70970\_JingChaoZuZhi2, Unigene71043\_JingChaoZuZhi2, Unigene71054\_JingChaoZuZhi2, Unigene71118\_JingChaoZuZhi2, Unigene7116\_JingChaoZuZhi2, Unigene7118\_JingChaoZuZhi2, Unigene71213\_JingChaoZuZhi2, Unigene71241\_JingChaoZuZhi2, Unigene71385\_JingChaoZuZhi2, Unigene71546\_JingChaoZuZhi2, Unigene71592\_JingChaoZuZhi2, Unigene71599\_JingChaoZuZhi2, Unigene71774\_JingChaoZuZhi2, Unigene71885\_JingChaoZuZhi2, Unigene72249\_JingChaoZuZhi2, Unigene72334\_JingChaoZuZhi2, Unigene72351\_JingChaoZuZhi2, Unigene72503\_JingChaoZuZhi2, Unigene72632\_JingChaoZuZhi2, Unigene7268\_JingChaoZuZhi2, Unigene72708\_JingChaoZuZhi2, Unigene72832\_JingChaoZuZhi2, Unigene73054\_JingChaoZuZhi2, Unigene73233\_JingChaoZuZhi2, Unigene73257\_JingChaoZuZhi2, Unigene73446\_JingChaoZuZhi2, Unigene73475\_JingChaoZuZhi2, Unigene73595\_JingChaoZuZhi2, Unigene73620\_JingChaoZuZhi2, Unigene73817\_JingChaoZuZhi2, Unigene73887\_JingChaoZuZhi2, Unigene73981\_JingChaoZuZhi2, Unigene741\_JingChaoZuZhi2, Unigene74221\_JingChaoZuZhi2, Unigene74259\_JingChaoZuZhi2, Unigene74298\_JingChaoZuZhi2, Unigene74474\_JingChaoZuZhi2, Unigene74719\_JingChaoZuZhi2, Unigene74730\_JingChaoZuZhi2, Unigene74854\_JingChaoZuZhi2, Unigene7489\_JingChaoZuZhi2, Unigene74949\_JingChaoZuZhi2, Unigene75009\_JingChaoZuZhi2, Unigene7508\_JingChaoZuZhi2, Unigene75112\_JingChaoZuZhi2, Unigene75167\_JingChaoZuZhi2, Unigene75491\_JingChaoZuZhi2, Unigene75840\_JingChaoZuZhi2, Unigene75902\_JingChaoZuZhi2, Unigene75946\_JingChaoZuZhi2, Unigene76002\_JingChaoZuZhi2, Unigene76023\_JingChaoZuZhi2, Unigene76111\_JingChaoZuZhi2, Unigene76269\_JingChaoZuZhi2, Unigene76302\_JingChaoZuZhi2, Unigene7634\_JingChaoZuZhi2, Unigene76437\_JingChaoZuZhi2, Unigene76515\_JingChaoZuZhi2, Unigene7663\_JingChaoZuZhi2, Unigene76676\_JingChaoZuZhi2, Unigene76776\_JingChaoZuZhi2, Unigene76785\_JingChaoZuZhi2, Unigene76819\_JingChaoZuZhi2, Unigene77024\_JingChaoZuZhi2, Unigene77406\_JingChaoZuZhi2, Unigene77413\_JingChaoZuZhi2, Unigene77520\_JingChaoZuZhi2, Unigene7794\_JingChaoZuZhi2, Unigene78009\_JingChaoZuZhi2, Unigene78191\_JingChaoZuZhi2, Unigene78288\_JingChaoZuZhi2, Unigene78361\_JingChaoZuZhi2, Unigene78410\_JingChaoZuZhi2, Unigene78460\_JingChaoZuZhi2, Unigene7850\_JingChaoZuZhi2, Unigene78521\_JingChaoZuZhi2, Unigene78555\_JingChaoZuZhi2, Unigene78636\_JingChaoZuZhi2, Unigene78680\_JingChaoZuZhi2, Unigene79058\_JingChaoZuZhi2, Unigene79118\_JingChaoZuZhi2, Unigene7911\_JingChaoZuZhi2, Unigene79141\_JingChaoZuZhi2, Unigene79233\_JingChaoZuZhi2, Unigene79358\_JingChaoZuZhi2, Unigene7939\_JingChaoZuZhi2, Unigene79433\_JingChaoZuZhi2, Unigene79443\_JingChaoZuZhi2, Unigene79653\_JingChaoZuZhi2, Unigene79712\_JingChaoZuZhi2, Unigene79726\_JingChaoZuZhi2, Unigene79789\_JingChaoZuZhi2, Unigene79824\_JingChaoZuZhi2, Unigene79864\_JingChaoZuZhi2, Unigene7987\_JingChaoZuZhi2, Unigene79940\_JingChaoZuZhi2, Unigene80043\_JingChaoZuZhi2, Unigene80143\_JingChaoZuZhi2, Unigene80212\_JingChaoZuZhi2, Unigene80403\_JingChaoZuZhi2, Unigene80619\_JingChaoZuZhi2, Unigene80893\_JingChaoZuZhi2, Unigene80980\_JingChaoZuZhi2, Unigene81059\_JingChaoZuZhi2, Unigene81167\_JingChaoZuZhi2, Unigene81327\_JingChaoZuZhi2, Unigene81440\_JingChaoZuZhi2, Unigene81450\_JingChaoZuZhi2, Unigene81561\_JingChaoZuZhi2, Unigene81599\_JingChaoZuZhi2, Unigene81686\_JingChaoZuZhi2, Unigene81757\_JingChaoZuZhi2, Unigene81825\_JingChaoZuZhi2, Unigene82047\_JingChaoZuZhi2, Unigene82070\_JingChaoZuZhi2, Unigene82111\_JingChaoZuZhi2, Unigene82117\_JingChaoZuZhi2, Unigene82141\_JingChaoZuZhi2, Unigene82215\_JingChaoZuZhi2, Unigene82489\_JingChaoZuZhi2, Unigene82578\_JingChaoZuZhi2, Unigene82605\_JingChaoZuZhi2, Unigene82645\_JingChaoZuZhi2, Unigene82688\_JingChaoZuZhi2, Unigene82907\_JingChaoZuZhi2, Unigene83079\_JingChaoZuZhi2, Unigene83214\_JingChaoZuZhi2, Unigene83766\_JingChaoZuZhi2, Unigene83782\_JingChaoZuZhi2, Unigene83825\_JingChaoZuZhi2, Unigene83872\_JingChaoZuZhi2, Unigene8392\_JingChaoZuZhi2, Unigene8406\_JingChaoZuZhi2, Unigene84076\_JingChaoZuZhi2, Unigene84152\_JingChaoZuZhi2, Unigene84168\_JingChaoZuZhi2, Unigene841\_JingChaoZuZhi2, Unigene84212\_JingChaoZuZhi2, Unigene8443\_JingChaoZuZhi2, Unigene84519\_JingChaoZuZhi2, Unigene84533\_JingChaoZuZhi2, Unigene84566\_JingChaoZuZhi2, Unigene84591\_JingChaoZuZhi2, Unigene84655\_JingChaoZuZhi2, Unigene84748\_JingChaoZuZhi2, Unigene84760\_JingChaoZuZhi2, Unigene84945\_JingChaoZuZhi2, Unigene84993\_JingChaoZuZhi2, Unigene85193\_JingChaoZuZhi2, Unigene851\_JingChaoZuZhi2, Unigene85314\_JingChaoZuZhi2, Unigene85632\_JingChaoZuZhi2, Unigene85770\_JingChaoZuZhi2, Unigene85902\_JingChaoZuZhi2, Unigene86020\_JingChaoZuZhi2, Unigene86091\_JingChaoZuZhi2, Unigene86145\_JingChaoZuZhi2, Unigene86349\_JingChaoZuZhi2, Unigene86546\_JingChaoZuZhi2, Unigene8661\_JingChaoZuZhi2, Unigene86789\_JingChaoZuZhi2, Unigene87014\_JingChaoZuZhi2, Unigene87082\_JingChaoZuZhi2, Unigene87180\_JingChaoZuZhi2, Unigene87203\_JingChaoZuZhi2, Unigene87256\_JingChaoZuZhi2, Unigene87332\_JingChaoZuZhi2, Unigene87362\_JingChaoZuZhi2, Unigene87365\_JingChaoZuZhi2, Unigene87459\_JingChaoZuZhi2, Unigene87478\_JingChaoZuZhi2, Unigene87488\_JingChaoZuZhi2, Unigene87533\_JingChaoZuZhi2, Unigene87567\_JingChaoZuZhi2, Unigene87774\_JingChaoZuZhi2, Unigene87853\_JingChaoZuZhi2, Unigene87941\_JingChaoZuZhi2, Unigene87946\_JingChaoZuZhi2, Unigene88121\_JingChaoZuZhi2, Unigene88169\_JingChaoZuZhi2, Unigene88271\_JingChaoZuZhi2, Unigene8833\_JingChaoZuZhi2, Unigene88342\_JingChaoZuZhi2, Unigene88400\_JingChaoZuZhi2, Unigene88406\_JingChaoZuZhi2, Unigene88442\_JingChaoZuZhi2, Unigene88454\_JingChaoZuZhi2, Unigene88470\_JingChaoZuZhi2, Unigene88484\_JingChaoZuZhi2, Unigene88608\_JingChaoZuZhi2, Unigene88665\_JingChaoZuZhi2, Unigene88683\_JingChaoZuZhi2, Unigene88738\_JingChaoZuZhi2, Unigene88741\_JingChaoZuZhi2, Unigene88778\_JingChaoZuZhi2, Unigene88779\_JingChaoZuZhi2, Unigene88781\_JingChaoZuZhi2, Unigene88797\_JingChaoZuZhi2, Unigene88830\_JingChaoZuZhi2, Unigene88837\_JingChaoZuZhi2, Unigene88945\_JingChaoZuZhi2, Unigene88979\_JingChaoZuZhi2, Unigene88988\_JingChaoZuZhi2, Unigene89013\_JingChaoZuZhi2, Unigene89060\_JingChaoZuZhi2, Unigene8907\_JingChaoZuZhi2, Unigene89093\_JingChaoZuZhi2, Unigene89121\_JingChaoZuZhi2, Unigene89323\_JingChaoZuZhi2, Unigene89337\_JingChaoZuZhi2, Unigene89352\_JingChaoZuZhi2, Unigene89367\_JingChaoZuZhi2, Unigene89512\_JingChaoZuZhi2, Unigene8953\_JingChaoZuZhi2, Unigene89541\_JingChaoZuZhi2, Unigene89606\_JingChaoZuZhi2, Unigene89645\_JingChaoZuZhi2, Unigene89722\_JingChaoZuZhi2, Unigene89798\_JingChaoZuZhi2, Unigene89834\_JingChaoZuZhi2, Unigene89867\_JingChaoZuZhi2, Unigene89942\_JingChaoZuZhi2, Unigene90038\_JingChaoZuZhi2, Unigene90066\_JingChaoZuZhi2, Unigene90071\_JingChaoZuZhi2, Unigene90209\_JingChaoZuZhi2, Unigene90239\_JingChaoZuZhi2, Unigene90307\_JingChaoZuZhi2, Unigene90341\_JingChaoZuZhi2, Unigene90508\_JingChaoZuZhi2, Unigene9050\_JingChaoZuZhi2, Unigene90564\_JingChaoZuZhi2, Unigene90714\_JingChaoZuZhi2, Unigene90768\_JingChaoZuZhi2, Unigene90779\_JingChaoZuZhi2, Unigene90953\_JingChaoZuZhi2, Unigene90995\_JingChaoZuZhi2, Unigene91025\_JingChaoZuZhi2, Unigene91163\_JingChaoZuZhi2, Unigene91298\_JingChaoZuZhi2, Unigene91363\_JingChaoZuZhi2, Unigene91390\_JingChaoZuZhi2, Unigene91405\_JingChaoZuZhi2, Unigene91456\_JingChaoZuZhi2, Unigene91487\_JingChaoZuZhi2, Unigene91529\_JingChaoZuZhi2, Unigene91553\_JingChaoZuZhi2, Unigene91741\_JingChaoZuZhi2, Unigene91794\_JingChaoZuZhi2, Unigene91839\_JingChaoZuZhi2, Unigene91848\_JingChaoZuZhi2, Unigene92047\_JingChaoZuZhi2, Unigene92325\_JingChaoZuZhi2, Unigene92346\_JingChaoZuZhi2, Unigene92367\_JingChaoZuZhi2, Unigene92580\_JingChaoZuZhi2, Unigene92681\_JingChaoZuZhi2, Unigene92700\_JingChaoZuZhi2, Unigene92780\_JingChaoZuZhi2, Unigene92798\_JingChaoZuZhi2, Unigene92838\_JingChaoZuZhi2, Unigene92880\_JingChaoZuZhi2, Unigene92951\_JingChaoZuZhi2, Unigene93176\_JingChaoZuZhi2, Unigene93246\_JingChaoZuZhi2, Unigene93487\_JingChaoZuZhi2, Unigene93552\_JingChaoZuZhi2, Unigene93573\_JingChaoZuZhi2, Unigene93592\_JingChaoZuZhi2, Unigene93610\_JingChaoZuZhi2, Unigene9364\_JingChaoZuZhi2, Unigene93742\_JingChaoZuZhi2, Unigene93782\_JingChaoZuZhi2, Unigene93793\_JingChaoZuZhi2, Unigene93821\_JingChaoZuZhi2, Unigene93957\_JingChaoZuZhi2, Unigene93979\_JingChaoZuZhi2, Unigene94020\_JingChaoZuZhi2, Unigene94036\_JingChaoZuZhi2, Unigene94049\_JingChaoZuZhi2, Unigene94070\_JingChaoZuZhi2, Unigene94083\_JingChaoZuZhi2, Unigene94110\_JingChaoZuZhi2, Unigene94120\_JingChaoZuZhi2, Unigene94129\_JingChaoZuZhi2, Unigene94134\_JingChaoZuZhi2, Unigene94138\_JingChaoZuZhi2, Unigene94272\_JingChaoZuZhi2, Unigene94319\_JingChaoZuZhi2, Unigene94395\_JingChaoZuZhi2, Unigene94404\_JingChaoZuZhi2, Unigene9444\_JingChaoZuZhi2, Unigene94471\_JingChaoZuZhi2, Unigene94520\_JingChaoZuZhi2, Unigene94561\_JingChaoZuZhi2, Unigene94570\_JingChaoZuZhi2, Unigene94584\_JingChaoZuZhi2, Unigene94891\_JingChaoZuZhi2, Unigene94968\_JingChaoZuZhi2, Unigene94980\_JingChaoZuZhi2, Unigene95086\_JingChaoZuZhi2, Unigene95088\_JingChaoZuZhi2, Unigene95119\_JingChaoZuZhi2, Unigene95190\_JingChaoZuZhi2, Unigene951\_JingChaoZuZhi2, Unigene95203\_JingChaoZuZhi2, Unigene95207\_JingChaoZuZhi2, Unigene95236\_JingChaoZuZhi2, Unigene95248\_JingChaoZuZhi2, Unigene95371\_JingChaoZuZhi2, Unigene9540\_JingChaoZuZhi2, Unigene95581\_JingChaoZuZhi2, Unigene95589\_JingChaoZuZhi2, Unigene95633\_JingChaoZuZhi2, Unigene95677\_JingChaoZuZhi2, Unigene95686\_JingChaoZuZhi2, Unigene95702\_JingChaoZuZhi2, Unigene95738\_JingChaoZuZhi2, Unigene95784\_JingChaoZuZhi2, Unigene95794\_JingChaoZuZhi2, Unigene95812\_JingChaoZuZhi2, Unigene95881\_JingChaoZuZhi2, Unigene95907\_JingChaoZuZhi2, Unigene95983\_JingChaoZuZhi2, Unigene96121\_JingChaoZuZhi2, Unigene96172\_JingChaoZuZhi2, Unigene96200\_JingChaoZuZhi2, Unigene96234\_JingChaoZuZhi2, Unigene9688\_JingChaoZuZhi2, Unigene9739\_JingChaoZuZhi2, Unigene9807\_JingChaoZuZhi2, Unigene9833\_JingChaoZuZhi2, Unigene9992\_JingChaoZuZhi2 |
| 13 | Pathways in cancer | Unigene10031\_JingChaoZuZhi2, Unigene10108\_JingChaoZuZhi2, Unigene1026\_JingChaoZuZhi2, Unigene1030\_JingChaoZuZhi2, Unigene10318\_JingChaoZuZhi2, Unigene1041\_JingChaoZuZhi2, Unigene10422\_JingChaoZuZhi2, Unigene10463\_JingChaoZuZhi2, Unigene10597\_JingChaoZuZhi2, Unigene10610\_JingChaoZuZhi2, Unigene10679\_JingChaoZuZhi2, Unigene1072\_JingChaoZuZhi2, Unigene10755\_JingChaoZuZhi2, Unigene10770\_JingChaoZuZhi2, Unigene10876\_JingChaoZuZhi2, Unigene10926\_JingChaoZuZhi2, Unigene10932\_JingChaoZuZhi2, Unigene10986\_JingChaoZuZhi2, Unigene11000\_JingChaoZuZhi2, Unigene1111\_JingChaoZuZhi2, Unigene11190\_JingChaoZuZhi2, Unigene11301\_JingChaoZuZhi2, Unigene1136\_JingChaoZuZhi2, Unigene1143\_JingChaoZuZhi2, Unigene11446\_JingChaoZuZhi2, Unigene11473\_JingChaoZuZhi2, Unigene11543\_JingChaoZuZhi2, Unigene11564\_JingChaoZuZhi2, Unigene11571\_JingChaoZuZhi2, Unigene11757\_JingChaoZuZhi2, Unigene11758\_JingChaoZuZhi2, Unigene11802\_JingChaoZuZhi2, Unigene11803\_JingChaoZuZhi2, Unigene11951\_JingChaoZuZhi2, Unigene11961\_JingChaoZuZhi2, Unigene12202\_JingChaoZuZhi2, Unigene12271\_JingChaoZuZhi2, Unigene12292\_JingChaoZuZhi2, Unigene12370\_JingChaoZuZhi2, Unigene12376\_JingChaoZuZhi2, Unigene12420\_JingChaoZuZhi2, Unigene12455\_JingChaoZuZhi2, Unigene12577\_JingChaoZuZhi2, Unigene12595\_JingChaoZuZhi2, Unigene12633\_JingChaoZuZhi2, Unigene12634\_JingChaoZuZhi2, Unigene12722\_JingChaoZuZhi2, Unigene12823\_JingChaoZuZhi2, Unigene12836\_JingChaoZuZhi2, Unigene12972\_JingChaoZuZhi2, Unigene12994\_JingChaoZuZhi2, Unigene1303\_JingChaoZuZhi2, Unigene13077\_JingChaoZuZhi2, Unigene13157\_JingChaoZuZhi2, Unigene13176\_JingChaoZuZhi2, Unigene13230\_JingChaoZuZhi2, Unigene1324\_JingChaoZuZhi2, Unigene13337\_JingChaoZuZhi2, Unigene13354\_JingChaoZuZhi2, Unigene13389\_JingChaoZuZhi2, Unigene13390\_JingChaoZuZhi2, Unigene1352\_JingChaoZuZhi2, Unigene13615\_JingChaoZuZhi2, Unigene13716\_JingChaoZuZhi2, Unigene1371\_JingChaoZuZhi2, Unigene13760\_JingChaoZuZhi2, Unigene13869\_JingChaoZuZhi2, Unigene13925\_JingChaoZuZhi2, Unigene13946\_JingChaoZuZhi2, Unigene14054\_JingChaoZuZhi2, Unigene14151\_JingChaoZuZhi2, Unigene14231\_JingChaoZuZhi2, Unigene14285\_JingChaoZuZhi2, Unigene14339\_JingChaoZuZhi2, Unigene14368\_JingChaoZuZhi2, Unigene14390\_JingChaoZuZhi2, Unigene14433\_JingChaoZuZhi2, Unigene14534\_JingChaoZuZhi2, Unigene1472\_JingChaoZuZhi2, Unigene1473\_JingChaoZuZhi2, Unigene14842\_JingChaoZuZhi2, Unigene14855\_JingChaoZuZhi2, Unigene14912\_JingChaoZuZhi2, Unigene15029\_JingChaoZuZhi2, Unigene1515\_JingChaoZuZhi2, Unigene15216\_JingChaoZuZhi2, Unigene15228\_JingChaoZuZhi2, Unigene1527\_JingChaoZuZhi2, Unigene1540\_JingChaoZuZhi2, Unigene1554\_JingChaoZuZhi2, Unigene15814\_JingChaoZuZhi2, Unigene15909\_JingChaoZuZhi2, Unigene16072\_JingChaoZuZhi2, Unigene16116\_JingChaoZuZhi2, Unigene16121\_JingChaoZuZhi2, Unigene16147\_JingChaoZuZhi2, Unigene16229\_JingChaoZuZhi2, Unigene16468\_JingChaoZuZhi2, Unigene16516\_JingChaoZuZhi2, Unigene16551\_JingChaoZuZhi2, Unigene16820\_JingChaoZuZhi2, Unigene16873\_JingChaoZuZhi2, Unigene16958\_JingChaoZuZhi2, Unigene17128\_JingChaoZuZhi2, Unigene17243\_JingChaoZuZhi2, Unigene17247\_JingChaoZuZhi2, Unigene17467\_JingChaoZuZhi2, Unigene17675\_JingChaoZuZhi2, Unigene17764\_JingChaoZuZhi2, Unigene17800\_JingChaoZuZhi2, Unigene1808\_JingChaoZuZhi2, Unigene18092\_JingChaoZuZhi2, Unigene18095\_JingChaoZuZhi2, Unigene18177\_JingChaoZuZhi2, Unigene18209\_JingChaoZuZhi2, Unigene18217\_JingChaoZuZhi2, Unigene18438\_JingChaoZuZhi2, Unigene18464\_JingChaoZuZhi2, Unigene18493\_JingChaoZuZhi2, Unigene18542\_JingChaoZuZhi2, Unigene18609\_JingChaoZuZhi2, Unigene18672\_JingChaoZuZhi2, Unigene18715\_JingChaoZuZhi2, Unigene18728\_JingChaoZuZhi2, Unigene18760\_JingChaoZuZhi2, Unigene18780\_JingChaoZuZhi2, Unigene18817\_JingChaoZuZhi2, Unigene18868\_JingChaoZuZhi2, Unigene18932\_JingChaoZuZhi2, Unigene18986\_JingChaoZuZhi2, Unigene19021\_JingChaoZuZhi2, Unigene19150\_JingChaoZuZhi2, Unigene19230\_JingChaoZuZhi2, Unigene19238\_JingChaoZuZhi2, Unigene19273\_JingChaoZuZhi2, Unigene19307\_JingChaoZuZhi2, Unigene19463\_JingChaoZuZhi2, Unigene19465\_JingChaoZuZhi2, Unigene19551\_JingChaoZuZhi2, Unigene19789\_JingChaoZuZhi2, Unigene19813\_JingChaoZuZhi2, Unigene19826\_JingChaoZuZhi2, Unigene19836\_JingChaoZuZhi2, Unigene19852\_JingChaoZuZhi2, Unigene20009\_JingChaoZuZhi2, Unigene20013\_JingChaoZuZhi2, Unigene20052\_JingChaoZuZhi2, Unigene20157\_JingChaoZuZhi2, Unigene20411\_JingChaoZuZhi2, Unigene20780\_JingChaoZuZhi2, Unigene20794\_JingChaoZuZhi2, Unigene20803\_JingChaoZuZhi2, Unigene20840\_JingChaoZuZhi2, Unigene20875\_JingChaoZuZhi2, Unigene20991\_JingChaoZuZhi2, Unigene21010\_JingChaoZuZhi2, Unigene21166\_JingChaoZuZhi2, Unigene2118\_JingChaoZuZhi2, Unigene21253\_JingChaoZuZhi2, Unigene21295\_JingChaoZuZhi2, Unigene2149\_JingChaoZuZhi2, Unigene21513\_JingChaoZuZhi2, Unigene21515\_JingChaoZuZhi2, Unigene21575\_JingChaoZuZhi2, Unigene21578\_JingChaoZuZhi2, Unigene21697\_JingChaoZuZhi2, Unigene21756\_JingChaoZuZhi2, Unigene21772\_JingChaoZuZhi2, Unigene21956\_JingChaoZuZhi2, Unigene22043\_JingChaoZuZhi2, Unigene22054\_JingChaoZuZhi2, Unigene22245\_JingChaoZuZhi2, Unigene2231\_JingChaoZuZhi2, Unigene22402\_JingChaoZuZhi2, Unigene22407\_JingChaoZuZhi2, Unigene22445\_JingChaoZuZhi2, Unigene2245\_JingChaoZuZhi2, Unigene22464\_JingChaoZuZhi2, Unigene22466\_JingChaoZuZhi2, Unigene22500\_JingChaoZuZhi2, Unigene22506\_JingChaoZuZhi2, Unigene22517\_JingChaoZuZhi2, Unigene22522\_JingChaoZuZhi2, Unigene22572\_JingChaoZuZhi2, Unigene22580\_JingChaoZuZhi2, Unigene22585\_JingChaoZuZhi2, Unigene22638\_JingChaoZuZhi2, Unigene22673\_JingChaoZuZhi2, Unigene22708\_JingChaoZuZhi2, Unigene2275\_JingChaoZuZhi2, Unigene22779\_JingChaoZuZhi2, Unigene22810\_JingChaoZuZhi2, Unigene22936\_JingChaoZuZhi2, Unigene23122\_JingChaoZuZhi2, Unigene2379\_JingChaoZuZhi2, Unigene23885\_JingChaoZuZhi2, Unigene24037\_JingChaoZuZhi2, Unigene24196\_JingChaoZuZhi2, Unigene2453\_JingChaoZuZhi2, Unigene2456\_JingChaoZuZhi2, Unigene2473\_JingChaoZuZhi2, Unigene24772\_JingChaoZuZhi2, Unigene2488\_JingChaoZuZhi2, Unigene25435\_JingChaoZuZhi2, Unigene25675\_JingChaoZuZhi2, Unigene26438\_JingChaoZuZhi2, Unigene26603\_JingChaoZuZhi2, Unigene26730\_JingChaoZuZhi2, Unigene27196\_JingChaoZuZhi2, Unigene27217\_JingChaoZuZhi2, Unigene27478\_JingChaoZuZhi2, Unigene27651\_JingChaoZuZhi2, Unigene27868\_JingChaoZuZhi2, Unigene2790\_JingChaoZuZhi2, Unigene27933\_JingChaoZuZhi2, Unigene28208\_JingChaoZuZhi2, Unigene28357\_JingChaoZuZhi2, Unigene28421\_JingChaoZuZhi2, Unigene28467\_JingChaoZuZhi2, Unigene28737\_JingChaoZuZhi2, Unigene28752\_JingChaoZuZhi2, Unigene28978\_JingChaoZuZhi2, Unigene29281\_JingChaoZuZhi2, Unigene29488\_JingChaoZuZhi2, Unigene2960\_JingChaoZuZhi2, Unigene30386\_JingChaoZuZhi2, Unigene30419\_JingChaoZuZhi2, Unigene30787\_JingChaoZuZhi2, Unigene3299\_JingChaoZuZhi2, Unigene33155\_JingChaoZuZhi2, Unigene33393\_JingChaoZuZhi2, Unigene33536\_JingChaoZuZhi2, Unigene33683\_JingChaoZuZhi2, Unigene34055\_JingChaoZuZhi2, Unigene34365\_JingChaoZuZhi2, Unigene34463\_JingChaoZuZhi2, Unigene3452\_JingChaoZuZhi2, Unigene34574\_JingChaoZuZhi2, Unigene34723\_JingChaoZuZhi2, Unigene34807\_JingChaoZuZhi2, Unigene35236\_JingChaoZuZhi2, Unigene35243\_JingChaoZuZhi2, Unigene35624\_JingChaoZuZhi2, Unigene35730\_JingChaoZuZhi2, Unigene35755\_JingChaoZuZhi2, Unigene35791\_JingChaoZuZhi2, Unigene3585\_JingChaoZuZhi2, Unigene3640\_JingChaoZuZhi2, Unigene36479\_JingChaoZuZhi2, Unigene3665\_JingChaoZuZhi2, Unigene36751\_JingChaoZuZhi2, Unigene3700\_JingChaoZuZhi2, Unigene37070\_JingChaoZuZhi2, Unigene3762\_JingChaoZuZhi2, Unigene3763\_JingChaoZuZhi2, Unigene37739\_JingChaoZuZhi2, Unigene38121\_JingChaoZuZhi2, Unigene38191\_JingChaoZuZhi2, Unigene38311\_JingChaoZuZhi2, Unigene38371\_JingChaoZuZhi2, Unigene38741\_JingChaoZuZhi2, Unigene38765\_JingChaoZuZhi2, Unigene38983\_JingChaoZuZhi2, Unigene39197\_JingChaoZuZhi2, Unigene3931\_JingChaoZuZhi2, Unigene3936\_JingChaoZuZhi2, Unigene39577\_JingChaoZuZhi2, Unigene3957\_JingChaoZuZhi2, Unigene39584\_JingChaoZuZhi2, Unigene395\_JingChaoZuZhi2, Unigene39637\_JingChaoZuZhi2, Unigene39674\_JingChaoZuZhi2, Unigene39825\_JingChaoZuZhi2, Unigene39876\_JingChaoZuZhi2, Unigene40238\_JingChaoZuZhi2, Unigene4024\_JingChaoZuZhi2, Unigene40410\_JingChaoZuZhi2, Unigene41120\_JingChaoZuZhi2, Unigene41367\_JingChaoZuZhi2, Unigene4155\_JingChaoZuZhi2, Unigene4186\_JingChaoZuZhi2, Unigene42070\_JingChaoZuZhi2, Unigene42422\_JingChaoZuZhi2, Unigene42755\_JingChaoZuZhi2, Unigene4294\_JingChaoZuZhi2, Unigene42969\_JingChaoZuZhi2, Unigene42981\_JingChaoZuZhi2, Unigene43120\_JingChaoZuZhi2, Unigene4377\_JingChaoZuZhi2, Unigene44061\_JingChaoZuZhi2, Unigene44191\_JingChaoZuZhi2, Unigene44228\_JingChaoZuZhi2, Unigene44927\_JingChaoZuZhi2, Unigene45166\_JingChaoZuZhi2, Unigene45194\_JingChaoZuZhi2, Unigene451\_JingChaoZuZhi2, Unigene45309\_JingChaoZuZhi2, Unigene45352\_JingChaoZuZhi2, Unigene45662\_JingChaoZuZhi2, Unigene45739\_JingChaoZuZhi2, Unigene46021\_JingChaoZuZhi2, Unigene46160\_JingChaoZuZhi2, Unigene46175\_JingChaoZuZhi2, Unigene46219\_JingChaoZuZhi2, Unigene46240\_JingChaoZuZhi2, Unigene46463\_JingChaoZuZhi2, Unigene4678\_JingChaoZuZhi2, Unigene47217\_JingChaoZuZhi2, Unigene47450\_JingChaoZuZhi2, Unigene4803\_JingChaoZuZhi2, Unigene48223\_JingChaoZuZhi2, Unigene48228\_JingChaoZuZhi2, Unigene48357\_JingChaoZuZhi2, Unigene4838\_JingChaoZuZhi2, Unigene4841\_JingChaoZuZhi2, Unigene48673\_JingChaoZuZhi2, Unigene48751\_JingChaoZuZhi2, Unigene48816\_JingChaoZuZhi2, Unigene48843\_JingChaoZuZhi2, Unigene4886\_JingChaoZuZhi2, Unigene49664\_JingChaoZuZhi2, Unigene49726\_JingChaoZuZhi2, Unigene49798\_JingChaoZuZhi2, Unigene499\_JingChaoZuZhi2, Unigene50572\_JingChaoZuZhi2, Unigene50611\_JingChaoZuZhi2, Unigene50645\_JingChaoZuZhi2, Unigene5069\_JingChaoZuZhi2, Unigene50713\_JingChaoZuZhi2, Unigene5099\_JingChaoZuZhi2, Unigene51285\_JingChaoZuZhi2, Unigene5165\_JingChaoZuZhi2, Unigene51679\_JingChaoZuZhi2, Unigene51703\_JingChaoZuZhi2, Unigene51705\_JingChaoZuZhi2, Unigene5226\_JingChaoZuZhi2, Unigene5252\_JingChaoZuZhi2, Unigene53103\_JingChaoZuZhi2, Unigene5314\_JingChaoZuZhi2, Unigene53421\_JingChaoZuZhi2, Unigene53503\_JingChaoZuZhi2, Unigene5364\_JingChaoZuZhi2, Unigene53917\_JingChaoZuZhi2, Unigene54320\_JingChaoZuZhi2, Unigene5437\_JingChaoZuZhi2, Unigene54470\_JingChaoZuZhi2, Unigene54679\_JingChaoZuZhi2, Unigene55392\_JingChaoZuZhi2, Unigene55677\_JingChaoZuZhi2, Unigene5578\_JingChaoZuZhi2, Unigene55804\_JingChaoZuZhi2, Unigene55810\_JingChaoZuZhi2, Unigene5621\_JingChaoZuZhi2, Unigene56227\_JingChaoZuZhi2, Unigene56569\_JingChaoZuZhi2, Unigene56642\_JingChaoZuZhi2, Unigene56652\_JingChaoZuZhi2, Unigene56709\_JingChaoZuZhi2, Unigene56772\_JingChaoZuZhi2, Unigene56922\_JingChaoZuZhi2, Unigene57174\_JingChaoZuZhi2, Unigene57488\_JingChaoZuZhi2, Unigene57579\_JingChaoZuZhi2, Unigene5758\_JingChaoZuZhi2, Unigene57694\_JingChaoZuZhi2, Unigene57752\_JingChaoZuZhi2, Unigene5798\_JingChaoZuZhi2, Unigene58005\_JingChaoZuZhi2, Unigene58136\_JingChaoZuZhi2, Unigene58474\_JingChaoZuZhi2, Unigene58582\_JingChaoZuZhi2, Unigene5863\_JingChaoZuZhi2, Unigene58670\_JingChaoZuZhi2, Unigene5875\_JingChaoZuZhi2, Unigene58762\_JingChaoZuZhi2, Unigene58806\_JingChaoZuZhi2, Unigene59011\_JingChaoZuZhi2, Unigene59250\_JingChaoZuZhi2, Unigene59263\_JingChaoZuZhi2, Unigene59480\_JingChaoZuZhi2, Unigene59794\_JingChaoZuZhi2, Unigene59797\_JingChaoZuZhi2, Unigene59849\_JingChaoZuZhi2, Unigene6028\_JingChaoZuZhi2, Unigene60326\_JingChaoZuZhi2, Unigene60453\_JingChaoZuZhi2, Unigene60707\_JingChaoZuZhi2, Unigene60762\_JingChaoZuZhi2, Unigene60777\_JingChaoZuZhi2, Unigene60891\_JingChaoZuZhi2, Unigene61043\_JingChaoZuZhi2, Unigene61457\_JingChaoZuZhi2, Unigene61486\_JingChaoZuZhi2, Unigene6159\_JingChaoZuZhi2, Unigene616\_JingChaoZuZhi2, Unigene61760\_JingChaoZuZhi2, Unigene61911\_JingChaoZuZhi2, Unigene62341\_JingChaoZuZhi2, Unigene62403\_JingChaoZuZhi2, Unigene62419\_JingChaoZuZhi2, Unigene62495\_JingChaoZuZhi2, Unigene62568\_JingChaoZuZhi2, Unigene62786\_JingChaoZuZhi2, Unigene62851\_JingChaoZuZhi2, Unigene62919\_JingChaoZuZhi2, Unigene63226\_JingChaoZuZhi2, Unigene63237\_JingChaoZuZhi2, Unigene63323\_JingChaoZuZhi2, Unigene63406\_JingChaoZuZhi2, Unigene63449\_JingChaoZuZhi2, Unigene63547\_JingChaoZuZhi2, Unigene63819\_JingChaoZuZhi2, Unigene63933\_JingChaoZuZhi2, Unigene64033\_JingChaoZuZhi2, Unigene64126\_JingChaoZuZhi2, Unigene64164\_JingChaoZuZhi2, Unigene64227\_JingChaoZuZhi2, Unigene6433\_JingChaoZuZhi2, Unigene64367\_JingChaoZuZhi2, Unigene64426\_JingChaoZuZhi2, Unigene64583\_JingChaoZuZhi2, Unigene64678\_JingChaoZuZhi2, Unigene6467\_JingChaoZuZhi2, Unigene64690\_JingChaoZuZhi2, Unigene64750\_JingChaoZuZhi2, Unigene64830\_JingChaoZuZhi2, Unigene6493\_JingChaoZuZhi2, Unigene65294\_JingChaoZuZhi2, Unigene65464\_JingChaoZuZhi2, Unigene65649\_JingChaoZuZhi2, Unigene65875\_JingChaoZuZhi2, Unigene65879\_JingChaoZuZhi2, Unigene66060\_JingChaoZuZhi2, Unigene66362\_JingChaoZuZhi2, Unigene6675\_JingChaoZuZhi2, Unigene66813\_JingChaoZuZhi2, Unigene66865\_JingChaoZuZhi2, Unigene66886\_JingChaoZuZhi2, Unigene66933\_JingChaoZuZhi2, Unigene6699\_JingChaoZuZhi2, Unigene67177\_JingChaoZuZhi2, Unigene67542\_JingChaoZuZhi2, Unigene67655\_JingChaoZuZhi2, Unigene67793\_JingChaoZuZhi2, Unigene6785\_JingChaoZuZhi2, Unigene68056\_JingChaoZuZhi2, Unigene68108\_JingChaoZuZhi2, Unigene68200\_JingChaoZuZhi2, Unigene68495\_JingChaoZuZhi2, Unigene6857\_JingChaoZuZhi2, Unigene68848\_JingChaoZuZhi2, Unigene69517\_JingChaoZuZhi2, Unigene69565\_JingChaoZuZhi2, Unigene69733\_JingChaoZuZhi2, Unigene70100\_JingChaoZuZhi2, Unigene70155\_JingChaoZuZhi2, Unigene70505\_JingChaoZuZhi2, Unigene70549\_JingChaoZuZhi2, Unigene70696\_JingChaoZuZhi2, Unigene70723\_JingChaoZuZhi2, Unigene70921\_JingChaoZuZhi2, Unigene70929\_JingChaoZuZhi2, Unigene71051\_JingChaoZuZhi2, Unigene71144\_JingChaoZuZhi2, Unigene7124\_JingChaoZuZhi2, Unigene7135\_JingChaoZuZhi2, Unigene71412\_JingChaoZuZhi2, Unigene71424\_JingChaoZuZhi2, Unigene71561\_JingChaoZuZhi2, Unigene71893\_JingChaoZuZhi2, Unigene7195\_JingChaoZuZhi2, Unigene71972\_JingChaoZuZhi2, Unigene7204\_JingChaoZuZhi2, Unigene72136\_JingChaoZuZhi2, Unigene72223\_JingChaoZuZhi2, Unigene7238\_JingChaoZuZhi2, Unigene72521\_JingChaoZuZhi2, Unigene72769\_JingChaoZuZhi2, Unigene72809\_JingChaoZuZhi2, Unigene72887\_JingChaoZuZhi2, Unigene72906\_JingChaoZuZhi2, Unigene72925\_JingChaoZuZhi2, Unigene73162\_JingChaoZuZhi2, Unigene73203\_JingChaoZuZhi2, Unigene73870\_JingChaoZuZhi2, Unigene73873\_JingChaoZuZhi2, Unigene74040\_JingChaoZuZhi2, Unigene74210\_JingChaoZuZhi2, Unigene74335\_JingChaoZuZhi2, Unigene74356\_JingChaoZuZhi2, Unigene74377\_JingChaoZuZhi2, Unigene7452\_JingChaoZuZhi2, Unigene74609\_JingChaoZuZhi2, Unigene74771\_JingChaoZuZhi2, Unigene74795\_JingChaoZuZhi2, Unigene74832\_JingChaoZuZhi2, Unigene75228\_JingChaoZuZhi2, Unigene75317\_JingChaoZuZhi2, Unigene75462\_JingChaoZuZhi2, Unigene75839\_JingChaoZuZhi2, Unigene75840\_JingChaoZuZhi2, Unigene75948\_JingChaoZuZhi2, Unigene76023\_JingChaoZuZhi2, Unigene76099\_JingChaoZuZhi2, Unigene76145\_JingChaoZuZhi2, Unigene76392\_JingChaoZuZhi2, Unigene76400\_JingChaoZuZhi2, Unigene76592\_JingChaoZuZhi2, Unigene76614\_JingChaoZuZhi2, Unigene76639\_JingChaoZuZhi2, Unigene76747\_JingChaoZuZhi2, Unigene76805\_JingChaoZuZhi2, Unigene76809\_JingChaoZuZhi2, Unigene76917\_JingChaoZuZhi2, Unigene77016\_JingChaoZuZhi2, Unigene77061\_JingChaoZuZhi2, Unigene77209\_JingChaoZuZhi2, Unigene77369\_JingChaoZuZhi2, Unigene77549\_JingChaoZuZhi2, Unigene77570\_JingChaoZuZhi2, Unigene77587\_JingChaoZuZhi2, Unigene78168\_JingChaoZuZhi2, Unigene78217\_JingChaoZuZhi2, Unigene78241\_JingChaoZuZhi2, Unigene7827\_JingChaoZuZhi2, Unigene78390\_JingChaoZuZhi2, Unigene78508\_JingChaoZuZhi2, Unigene78522\_JingChaoZuZhi2, Unigene78574\_JingChaoZuZhi2, Unigene7864\_JingChaoZuZhi2, Unigene78909\_JingChaoZuZhi2, Unigene78957\_JingChaoZuZhi2, Unigene79077\_JingChaoZuZhi2, Unigene79109\_JingChaoZuZhi2, Unigene79118\_JingChaoZuZhi2, Unigene79152\_JingChaoZuZhi2, Unigene79166\_JingChaoZuZhi2, Unigene79307\_JingChaoZuZhi2, Unigene79534\_JingChaoZuZhi2, Unigene79597\_JingChaoZuZhi2, Unigene79683\_JingChaoZuZhi2, Unigene79882\_JingChaoZuZhi2, Unigene79912\_JingChaoZuZhi2, Unigene80067\_JingChaoZuZhi2, Unigene80123\_JingChaoZuZhi2, Unigene80143\_JingChaoZuZhi2, Unigene80376\_JingChaoZuZhi2, Unigene8054\_JingChaoZuZhi2, Unigene80762\_JingChaoZuZhi2, Unigene80793\_JingChaoZuZhi2, Unigene80818\_JingChaoZuZhi2, Unigene81163\_JingChaoZuZhi2, Unigene8133\_JingChaoZuZhi2, Unigene81480\_JingChaoZuZhi2, Unigene81716\_JingChaoZuZhi2, Unigene8191\_JingChaoZuZhi2, Unigene8202\_JingChaoZuZhi2, Unigene82359\_JingChaoZuZhi2, Unigene82375\_JingChaoZuZhi2, Unigene8248\_JingChaoZuZhi2, Unigene82525\_JingChaoZuZhi2, Unigene82606\_JingChaoZuZhi2, Unigene82675\_JingChaoZuZhi2, Unigene82761\_JingChaoZuZhi2, Unigene83188\_JingChaoZuZhi2, Unigene83207\_JingChaoZuZhi2, Unigene83507\_JingChaoZuZhi2, Unigene83978\_JingChaoZuZhi2, Unigene84032\_JingChaoZuZhi2, Unigene84220\_JingChaoZuZhi2, Unigene84257\_JingChaoZuZhi2, Unigene84519\_JingChaoZuZhi2, Unigene84531\_JingChaoZuZhi2, Unigene84576\_JingChaoZuZhi2, Unigene84930\_JingChaoZuZhi2, Unigene85038\_JingChaoZuZhi2, Unigene85172\_JingChaoZuZhi2, Unigene8523\_JingChaoZuZhi2, Unigene85574\_JingChaoZuZhi2, Unigene8563\_JingChaoZuZhi2, Unigene8566\_JingChaoZuZhi2, Unigene85821\_JingChaoZuZhi2, Unigene85863\_JingChaoZuZhi2, Unigene8600\_JingChaoZuZhi2, Unigene8609\_JingChaoZuZhi2, Unigene86104\_JingChaoZuZhi2, Unigene86114\_JingChaoZuZhi2, Unigene86115\_JingChaoZuZhi2, Unigene86119\_JingChaoZuZhi2, Unigene86206\_JingChaoZuZhi2, Unigene86296\_JingChaoZuZhi2, Unigene86458\_JingChaoZuZhi2, Unigene8649\_JingChaoZuZhi2, Unigene86504\_JingChaoZuZhi2, Unigene86526\_JingChaoZuZhi2, Unigene86589\_JingChaoZuZhi2, Unigene86806\_JingChaoZuZhi2, Unigene86817\_JingChaoZuZhi2, Unigene87122\_JingChaoZuZhi2, Unigene87251\_JingChaoZuZhi2, Unigene87261\_JingChaoZuZhi2, Unigene87321\_JingChaoZuZhi2, Unigene87332\_JingChaoZuZhi2, Unigene87448\_JingChaoZuZhi2, Unigene87654\_JingChaoZuZhi2, Unigene876\_JingChaoZuZhi2, Unigene87730\_JingChaoZuZhi2, Unigene87952\_JingChaoZuZhi2, Unigene8799\_JingChaoZuZhi2, Unigene88040\_JingChaoZuZhi2, Unigene88053\_JingChaoZuZhi2, Unigene88082\_JingChaoZuZhi2, Unigene88167\_JingChaoZuZhi2, Unigene88185\_JingChaoZuZhi2, Unigene88343\_JingChaoZuZhi2, Unigene88391\_JingChaoZuZhi2, Unigene88470\_JingChaoZuZhi2, Unigene8851\_JingChaoZuZhi2, Unigene88544\_JingChaoZuZhi2, Unigene88550\_JingChaoZuZhi2, Unigene88719\_JingChaoZuZhi2, Unigene88748\_JingChaoZuZhi2, Unigene88925\_JingChaoZuZhi2, Unigene88942\_JingChaoZuZhi2, Unigene89093\_JingChaoZuZhi2, Unigene89161\_JingChaoZuZhi2, Unigene89191\_JingChaoZuZhi2, Unigene89310\_JingChaoZuZhi2, Unigene89315\_JingChaoZuZhi2, Unigene89329\_JingChaoZuZhi2, Unigene89347\_JingChaoZuZhi2, Unigene89612\_JingChaoZuZhi2, Unigene89774\_JingChaoZuZhi2, Unigene89790\_JingChaoZuZhi2, Unigene8982\_JingChaoZuZhi2, Unigene90003\_JingChaoZuZhi2, Unigene90073\_JingChaoZuZhi2, Unigene90078\_JingChaoZuZhi2, Unigene90201\_JingChaoZuZhi2, Unigene90261\_JingChaoZuZhi2, Unigene9029\_JingChaoZuZhi2, Unigene90353\_JingChaoZuZhi2, Unigene90495\_JingChaoZuZhi2, Unigene90761\_JingChaoZuZhi2, Unigene90927\_JingChaoZuZhi2, Unigene90979\_JingChaoZuZhi2, Unigene91038\_JingChaoZuZhi2, Unigene91100\_JingChaoZuZhi2, Unigene91119\_JingChaoZuZhi2, Unigene91179\_JingChaoZuZhi2, Unigene91201\_JingChaoZuZhi2, Unigene91275\_JingChaoZuZhi2, Unigene91284\_JingChaoZuZhi2, Unigene91423\_JingChaoZuZhi2, Unigene91580\_JingChaoZuZhi2, Unigene91612\_JingChaoZuZhi2, Unigene91689\_JingChaoZuZhi2, Unigene91958\_JingChaoZuZhi2, Unigene92019\_JingChaoZuZhi2, Unigene92104\_JingChaoZuZhi2, Unigene92112\_JingChaoZuZhi2, Unigene92309\_JingChaoZuZhi2, Unigene92394\_JingChaoZuZhi2, Unigene92651\_JingChaoZuZhi2, Unigene92715\_JingChaoZuZhi2, Unigene92738\_JingChaoZuZhi2, Unigene92794\_JingChaoZuZhi2, Unigene92831\_JingChaoZuZhi2, Unigene92839\_JingChaoZuZhi2, Unigene92983\_JingChaoZuZhi2, Unigene93067\_JingChaoZuZhi2, Unigene93133\_JingChaoZuZhi2, Unigene93195\_JingChaoZuZhi2, Unigene93199\_JingChaoZuZhi2, Unigene93257\_JingChaoZuZhi2, Unigene93409\_JingChaoZuZhi2, Unigene93502\_JingChaoZuZhi2, Unigene93782\_JingChaoZuZhi2, Unigene93807\_JingChaoZuZhi2, Unigene93953\_JingChaoZuZhi2, Unigene9404\_JingChaoZuZhi2, Unigene94138\_JingChaoZuZhi2, Unigene94149\_JingChaoZuZhi2, Unigene94204\_JingChaoZuZhi2, Unigene94208\_JingChaoZuZhi2, Unigene94242\_JingChaoZuZhi2, Unigene94272\_JingChaoZuZhi2, Unigene9428\_JingChaoZuZhi2, Unigene94377\_JingChaoZuZhi2, Unigene94456\_JingChaoZuZhi2, Unigene94474\_JingChaoZuZhi2, Unigene94544\_JingChaoZuZhi2, Unigene94561\_JingChaoZuZhi2, Unigene9461\_JingChaoZuZhi2, Unigene94656\_JingChaoZuZhi2, Unigene94685\_JingChaoZuZhi2, Unigene94711\_JingChaoZuZhi2, Unigene94795\_JingChaoZuZhi2, Unigene94819\_JingChaoZuZhi2, Unigene94829\_JingChaoZuZhi2, Unigene94894\_JingChaoZuZhi2, Unigene94956\_JingChaoZuZhi2, Unigene95014\_JingChaoZuZhi2, Unigene95016\_JingChaoZuZhi2, Unigene95084\_JingChaoZuZhi2, Unigene95098\_JingChaoZuZhi2, Unigene95295\_JingChaoZuZhi2, Unigene95297\_JingChaoZuZhi2, Unigene95315\_JingChaoZuZhi2, Unigene95378\_JingChaoZuZhi2, Unigene95441\_JingChaoZuZhi2, Unigene95456\_JingChaoZuZhi2, Unigene95463\_JingChaoZuZhi2, Unigene95485\_JingChaoZuZhi2, Unigene95505\_JingChaoZuZhi2, Unigene95568\_JingChaoZuZhi2, Unigene95674\_JingChaoZuZhi2, Unigene95677\_JingChaoZuZhi2, Unigene95723\_JingChaoZuZhi2, Unigene95751\_JingChaoZuZhi2, Unigene95782\_JingChaoZuZhi2, Unigene95791\_JingChaoZuZhi2, Unigene95799\_JingChaoZuZhi2, Unigene95875\_JingChaoZuZhi2, Unigene95881\_JingChaoZuZhi2, Unigene95883\_JingChaoZuZhi2, Unigene95933\_JingChaoZuZhi2, Unigene96018\_JingChaoZuZhi2, Unigene96044\_JingChaoZuZhi2, Unigene96057\_JingChaoZuZhi2, Unigene96121\_JingChaoZuZhi2, Unigene96124\_JingChaoZuZhi2, Unigene96234\_JingChaoZuZhi2, Unigene96281\_JingChaoZuZhi2, Unigene96294\_JingChaoZuZhi2, Unigene9630\_JingChaoZuZhi2, Unigene9643\_JingChaoZuZhi2, Unigene9756\_JingChaoZuZhi2, Unigene9804\_JingChaoZuZhi2, Unigene9902\_JingChaoZuZhi2, Unigene9948\_JingChaoZuZhi2 |
| 14 | Chemokine signaling pathway | Unigene10051\_JingChaoZuZhi2, Unigene10053\_JingChaoZuZhi2, Unigene10128\_JingChaoZuZhi2, Unigene10218\_JingChaoZuZhi2, Unigene10318\_JingChaoZuZhi2, Unigene10456\_JingChaoZuZhi2, Unigene10492\_JingChaoZuZhi2, Unigene10498\_JingChaoZuZhi2, Unigene10576\_JingChaoZuZhi2, Unigene10582\_JingChaoZuZhi2, Unigene10637\_JingChaoZuZhi2, Unigene10733\_JingChaoZuZhi2, Unigene10932\_JingChaoZuZhi2, Unigene10936\_JingChaoZuZhi2, Unigene10974\_JingChaoZuZhi2, Unigene10979\_JingChaoZuZhi2, Unigene11240\_JingChaoZuZhi2, Unigene11569\_JingChaoZuZhi2, Unigene115\_JingChaoZuZhi2, Unigene11621\_JingChaoZuZhi2, Unigene11726\_JingChaoZuZhi2, Unigene11810\_JingChaoZuZhi2, Unigene11889\_JingChaoZuZhi2, Unigene11902\_JingChaoZuZhi2, Unigene11918\_JingChaoZuZhi2, Unigene11932\_JingChaoZuZhi2, Unigene11961\_JingChaoZuZhi2, Unigene11995\_JingChaoZuZhi2, Unigene12060\_JingChaoZuZhi2, Unigene12148\_JingChaoZuZhi2, Unigene12221\_JingChaoZuZhi2, Unigene12285\_JingChaoZuZhi2, Unigene12310\_JingChaoZuZhi2, Unigene12326\_JingChaoZuZhi2, Unigene12372\_JingChaoZuZhi2, Unigene12375\_JingChaoZuZhi2, Unigene12420\_JingChaoZuZhi2, Unigene12595\_JingChaoZuZhi2, Unigene12644\_JingChaoZuZhi2, Unigene12791\_JingChaoZuZhi2, Unigene12899\_JingChaoZuZhi2, Unigene12938\_JingChaoZuZhi2, Unigene13097\_JingChaoZuZhi2, Unigene13180\_JingChaoZuZhi2, Unigene13196\_JingChaoZuZhi2, Unigene13249\_JingChaoZuZhi2, Unigene1325\_JingChaoZuZhi2, Unigene13478\_JingChaoZuZhi2, Unigene13510\_JingChaoZuZhi2, Unigene13572\_JingChaoZuZhi2, Unigene13588\_JingChaoZuZhi2, Unigene13607\_JingChaoZuZhi2, Unigene13618\_JingChaoZuZhi2, Unigene13650\_JingChaoZuZhi2, Unigene13766\_JingChaoZuZhi2, Unigene13919\_JingChaoZuZhi2, Unigene13927\_JingChaoZuZhi2, Unigene14159\_JingChaoZuZhi2, Unigene14193\_JingChaoZuZhi2, Unigene14246\_JingChaoZuZhi2, Unigene14360\_JingChaoZuZhi2, Unigene14401\_JingChaoZuZhi2, Unigene14418\_JingChaoZuZhi2, Unigene14450\_JingChaoZuZhi2, Unigene14476\_JingChaoZuZhi2, Unigene14534\_JingChaoZuZhi2, Unigene14567\_JingChaoZuZhi2, Unigene14610\_JingChaoZuZhi2, Unigene14714\_JingChaoZuZhi2, Unigene14715\_JingChaoZuZhi2, Unigene14912\_JingChaoZuZhi2, Unigene14970\_JingChaoZuZhi2, Unigene15088\_JingChaoZuZhi2, Unigene15095\_JingChaoZuZhi2, Unigene15096\_JingChaoZuZhi2, Unigene15151\_JingChaoZuZhi2, Unigene15176\_JingChaoZuZhi2, Unigene15228\_JingChaoZuZhi2, Unigene15251\_JingChaoZuZhi2, Unigene15288\_JingChaoZuZhi2, Unigene15319\_JingChaoZuZhi2, Unigene15374\_JingChaoZuZhi2, Unigene15381\_JingChaoZuZhi2, Unigene15492\_JingChaoZuZhi2, Unigene15615\_JingChaoZuZhi2, Unigene15650\_JingChaoZuZhi2, Unigene15651\_JingChaoZuZhi2, Unigene15731\_JingChaoZuZhi2, Unigene15857\_JingChaoZuZhi2, Unigene15879\_JingChaoZuZhi2, Unigene16028\_JingChaoZuZhi2, Unigene16103\_JingChaoZuZhi2, Unigene16176\_JingChaoZuZhi2, Unigene16182\_JingChaoZuZhi2, Unigene16318\_JingChaoZuZhi2, Unigene16339\_JingChaoZuZhi2, Unigene16402\_JingChaoZuZhi2, Unigene16481\_JingChaoZuZhi2, Unigene16591\_JingChaoZuZhi2, Unigene16655\_JingChaoZuZhi2, Unigene16730\_JingChaoZuZhi2, Unigene167\_JingChaoZuZhi2, Unigene16820\_JingChaoZuZhi2, Unigene16837\_JingChaoZuZhi2, Unigene16950\_JingChaoZuZhi2, Unigene16991\_JingChaoZuZhi2, Unigene17101\_JingChaoZuZhi2, Unigene17247\_JingChaoZuZhi2, Unigene17501\_JingChaoZuZhi2, Unigene17764\_JingChaoZuZhi2, Unigene17789\_JingChaoZuZhi2, Unigene17821\_JingChaoZuZhi2, Unigene17829\_JingChaoZuZhi2, Unigene17845\_JingChaoZuZhi2, Unigene17888\_JingChaoZuZhi2, Unigene17894\_JingChaoZuZhi2, Unigene18256\_JingChaoZuZhi2, Unigene18259\_JingChaoZuZhi2, Unigene18269\_JingChaoZuZhi2, Unigene18307\_JingChaoZuZhi2, Unigene18334\_JingChaoZuZhi2, Unigene18348\_JingChaoZuZhi2, Unigene18505\_JingChaoZuZhi2, Unigene18662\_JingChaoZuZhi2, Unigene18672\_JingChaoZuZhi2, Unigene18685\_JingChaoZuZhi2, Unigene18790\_JingChaoZuZhi2, Unigene18835\_JingChaoZuZhi2, Unigene1887\_JingChaoZuZhi2, Unigene19001\_JingChaoZuZhi2, Unigene19021\_JingChaoZuZhi2, Unigene19046\_JingChaoZuZhi2, Unigene19086\_JingChaoZuZhi2, Unigene19137\_JingChaoZuZhi2, Unigene19204\_JingChaoZuZhi2, Unigene19223\_JingChaoZuZhi2, Unigene19235\_JingChaoZuZhi2, Unigene19319\_JingChaoZuZhi2, Unigene19391\_JingChaoZuZhi2, Unigene19533\_JingChaoZuZhi2, Unigene19551\_JingChaoZuZhi2, Unigene19691\_JingChaoZuZhi2, Unigene19701\_JingChaoZuZhi2, Unigene19883\_JingChaoZuZhi2, Unigene19979\_JingChaoZuZhi2, Unigene20030\_JingChaoZuZhi2, Unigene20049\_JingChaoZuZhi2, Unigene20083\_JingChaoZuZhi2, Unigene20107\_JingChaoZuZhi2, Unigene20157\_JingChaoZuZhi2, Unigene201\_JingChaoZuZhi2, Unigene20220\_JingChaoZuZhi2, Unigene20239\_JingChaoZuZhi2, Unigene20373\_JingChaoZuZhi2, Unigene20468\_JingChaoZuZhi2, Unigene20474\_JingChaoZuZhi2, Unigene20480\_JingChaoZuZhi2, Unigene204\_JingChaoZuZhi2, Unigene20557\_JingChaoZuZhi2, Unigene20608\_JingChaoZuZhi2, Unigene20727\_JingChaoZuZhi2, Unigene20794\_JingChaoZuZhi2, Unigene20837\_JingChaoZuZhi2, Unigene20943\_JingChaoZuZhi2, Unigene20991\_JingChaoZuZhi2, Unigene21010\_JingChaoZuZhi2, Unigene21057\_JingChaoZuZhi2, Unigene21100\_JingChaoZuZhi2, Unigene2115\_JingChaoZuZhi2, Unigene21311\_JingChaoZuZhi2, Unigene21451\_JingChaoZuZhi2, Unigene21513\_JingChaoZuZhi2, Unigene21536\_JingChaoZuZhi2, Unigene21543\_JingChaoZuZhi2, Unigene21578\_JingChaoZuZhi2, Unigene21636\_JingChaoZuZhi2, Unigene21697\_JingChaoZuZhi2, Unigene21815\_JingChaoZuZhi2, Unigene21860\_JingChaoZuZhi2, Unigene21932\_JingChaoZuZhi2, Unigene21933\_JingChaoZuZhi2, Unigene22055\_JingChaoZuZhi2, Unigene22119\_JingChaoZuZhi2, Unigene22128\_JingChaoZuZhi2, Unigene22174\_JingChaoZuZhi2, Unigene22220\_JingChaoZuZhi2, Unigene2226\_JingChaoZuZhi2, Unigene22329\_JingChaoZuZhi2, Unigene22372\_JingChaoZuZhi2, Unigene22414\_JingChaoZuZhi2, Unigene22455\_JingChaoZuZhi2, Unigene22466\_JingChaoZuZhi2, Unigene22482\_JingChaoZuZhi2, Unigene22487\_JingChaoZuZhi2, Unigene22586\_JingChaoZuZhi2, Unigene2258\_JingChaoZuZhi2, Unigene22779\_JingChaoZuZhi2, Unigene22795\_JingChaoZuZhi2, Unigene22804\_JingChaoZuZhi2, Unigene2289\_JingChaoZuZhi2, Unigene22909\_JingChaoZuZhi2, Unigene23164\_JingChaoZuZhi2, Unigene23447\_JingChaoZuZhi2, Unigene23924\_JingChaoZuZhi2, Unigene23978\_JingChaoZuZhi2, Unigene2445\_JingChaoZuZhi2, Unigene2456\_JingChaoZuZhi2, Unigene24600\_JingChaoZuZhi2, Unigene2510\_JingChaoZuZhi2, Unigene25693\_JingChaoZuZhi2, Unigene25962\_JingChaoZuZhi2, Unigene26730\_JingChaoZuZhi2, Unigene2686\_JingChaoZuZhi2, Unigene28064\_JingChaoZuZhi2, Unigene28243\_JingChaoZuZhi2, Unigene28263\_JingChaoZuZhi2, Unigene2857\_JingChaoZuZhi2, Unigene28793\_JingChaoZuZhi2, Unigene2901\_JingChaoZuZhi2, Unigene2960\_JingChaoZuZhi2, Unigene30281\_JingChaoZuZhi2, Unigene3122\_JingChaoZuZhi2, Unigene31912\_JingChaoZuZhi2, Unigene32373\_JingChaoZuZhi2, Unigene324\_JingChaoZuZhi2, Unigene33044\_JingChaoZuZhi2, Unigene33173\_JingChaoZuZhi2, Unigene3321\_JingChaoZuZhi2, Unigene34142\_JingChaoZuZhi2, Unigene34785\_JingChaoZuZhi2, Unigene3493\_JingChaoZuZhi2, Unigene35080\_JingChaoZuZhi2, Unigene35927\_JingChaoZuZhi2, Unigene36257\_JingChaoZuZhi2, Unigene36342\_JingChaoZuZhi2, Unigene36361\_JingChaoZuZhi2, Unigene3646\_JingChaoZuZhi2, Unigene3671\_JingChaoZuZhi2, Unigene3693\_JingChaoZuZhi2, Unigene36974\_JingChaoZuZhi2, Unigene36976\_JingChaoZuZhi2, Unigene37164\_JingChaoZuZhi2, Unigene37741\_JingChaoZuZhi2, Unigene37803\_JingChaoZuZhi2, Unigene37818\_JingChaoZuZhi2, Unigene3854\_JingChaoZuZhi2, Unigene3931\_JingChaoZuZhi2, Unigene4015\_JingChaoZuZhi2, Unigene40281\_JingChaoZuZhi2, Unigene40335\_JingChaoZuZhi2, Unigene4044\_JingChaoZuZhi2, Unigene41148\_JingChaoZuZhi2, Unigene411\_JingChaoZuZhi2, Unigene41333\_JingChaoZuZhi2, Unigene4186\_JingChaoZuZhi2, Unigene42146\_JingChaoZuZhi2, Unigene43041\_JingChaoZuZhi2, Unigene43147\_JingChaoZuZhi2, Unigene43169\_JingChaoZuZhi2, Unigene43249\_JingChaoZuZhi2, Unigene43260\_JingChaoZuZhi2, Unigene4337\_JingChaoZuZhi2, Unigene43512\_JingChaoZuZhi2, Unigene43574\_JingChaoZuZhi2, Unigene44516\_JingChaoZuZhi2, Unigene45367\_JingChaoZuZhi2, Unigene4547\_JingChaoZuZhi2, Unigene45593\_JingChaoZuZhi2, Unigene45622\_JingChaoZuZhi2, Unigene45901\_JingChaoZuZhi2, Unigene45962\_JingChaoZuZhi2, Unigene46413\_JingChaoZuZhi2, Unigene4713\_JingChaoZuZhi2, Unigene47578\_JingChaoZuZhi2, Unigene4758\_JingChaoZuZhi2, Unigene47949\_JingChaoZuZhi2, Unigene4829\_JingChaoZuZhi2, Unigene4838\_JingChaoZuZhi2, Unigene4873\_JingChaoZuZhi2, Unigene48751\_JingChaoZuZhi2, Unigene49203\_JingChaoZuZhi2, Unigene49387\_JingChaoZuZhi2, Unigene49973\_JingChaoZuZhi2, Unigene50540\_JingChaoZuZhi2, Unigene50668\_JingChaoZuZhi2, Unigene50744\_JingChaoZuZhi2, Unigene51090\_JingChaoZuZhi2, Unigene51115\_JingChaoZuZhi2, Unigene5125\_JingChaoZuZhi2, Unigene5126\_JingChaoZuZhi2, Unigene5196\_JingChaoZuZhi2, Unigene51991\_JingChaoZuZhi2, Unigene52456\_JingChaoZuZhi2, Unigene52554\_JingChaoZuZhi2, Unigene5298\_JingChaoZuZhi2, Unigene53078\_JingChaoZuZhi2, Unigene5335\_JingChaoZuZhi2, Unigene53417\_JingChaoZuZhi2, Unigene53484\_JingChaoZuZhi2, Unigene53524\_JingChaoZuZhi2, Unigene53568\_JingChaoZuZhi2, Unigene53751\_JingChaoZuZhi2, Unigene53917\_JingChaoZuZhi2, Unigene53935\_JingChaoZuZhi2, Unigene53953\_JingChaoZuZhi2, Unigene54012\_JingChaoZuZhi2, Unigene54320\_JingChaoZuZhi2, Unigene54367\_JingChaoZuZhi2, Unigene54918\_JingChaoZuZhi2, Unigene54974\_JingChaoZuZhi2, Unigene55009\_JingChaoZuZhi2, Unigene55385\_JingChaoZuZhi2, Unigene56560\_JingChaoZuZhi2, Unigene56660\_JingChaoZuZhi2, Unigene57185\_JingChaoZuZhi2, Unigene57240\_JingChaoZuZhi2, Unigene57337\_JingChaoZuZhi2, Unigene57488\_JingChaoZuZhi2, Unigene57597\_JingChaoZuZhi2, Unigene5809\_JingChaoZuZhi2, Unigene58169\_JingChaoZuZhi2, Unigene5823\_JingChaoZuZhi2, Unigene58474\_JingChaoZuZhi2, Unigene58675\_JingChaoZuZhi2, Unigene59102\_JingChaoZuZhi2, Unigene59265\_JingChaoZuZhi2, Unigene59336\_JingChaoZuZhi2, Unigene59433\_JingChaoZuZhi2, Unigene59580\_JingChaoZuZhi2, Unigene59684\_JingChaoZuZhi2, Unigene60121\_JingChaoZuZhi2, Unigene6028\_JingChaoZuZhi2, Unigene60551\_JingChaoZuZhi2, Unigene60586\_JingChaoZuZhi2, Unigene6072\_JingChaoZuZhi2, Unigene60990\_JingChaoZuZhi2, Unigene61001\_JingChaoZuZhi2, Unigene61474\_JingChaoZuZhi2, Unigene6159\_JingChaoZuZhi2, Unigene6171\_JingChaoZuZhi2, Unigene61931\_JingChaoZuZhi2, Unigene62100\_JingChaoZuZhi2, Unigene6215\_JingChaoZuZhi2, Unigene6233\_JingChaoZuZhi2, Unigene62606\_JingChaoZuZhi2, Unigene62695\_JingChaoZuZhi2, Unigene63052\_JingChaoZuZhi2, Unigene63424\_JingChaoZuZhi2, Unigene63604\_JingChaoZuZhi2, Unigene63812\_JingChaoZuZhi2, Unigene6382\_JingChaoZuZhi2, Unigene63873\_JingChaoZuZhi2, Unigene63925\_JingChaoZuZhi2, Unigene6393\_JingChaoZuZhi2, Unigene64164\_JingChaoZuZhi2, Unigene64208\_JingChaoZuZhi2, Unigene64452\_JingChaoZuZhi2, Unigene64458\_JingChaoZuZhi2, Unigene64830\_JingChaoZuZhi2, Unigene64843\_JingChaoZuZhi2, Unigene6493\_JingChaoZuZhi2, Unigene65129\_JingChaoZuZhi2, Unigene65150\_JingChaoZuZhi2, Unigene65187\_JingChaoZuZhi2, Unigene65326\_JingChaoZuZhi2, Unigene65337\_JingChaoZuZhi2, Unigene65375\_JingChaoZuZhi2, Unigene65445\_JingChaoZuZhi2, Unigene65773\_JingChaoZuZhi2, Unigene6605\_JingChaoZuZhi2, Unigene66073\_JingChaoZuZhi2, Unigene66077\_JingChaoZuZhi2, Unigene66253\_JingChaoZuZhi2, Unigene6651\_JingChaoZuZhi2, Unigene66540\_JingChaoZuZhi2, Unigene66559\_JingChaoZuZhi2, Unigene66590\_JingChaoZuZhi2, Unigene6676\_JingChaoZuZhi2, Unigene66813\_JingChaoZuZhi2, Unigene66986\_JingChaoZuZhi2, Unigene67005\_JingChaoZuZhi2, Unigene67063\_JingChaoZuZhi2, Unigene67070\_JingChaoZuZhi2, Unigene67249\_JingChaoZuZhi2, Unigene67279\_JingChaoZuZhi2, Unigene67777\_JingChaoZuZhi2, Unigene67793\_JingChaoZuZhi2, Unigene67958\_JingChaoZuZhi2, Unigene6796\_JingChaoZuZhi2, Unigene6797\_JingChaoZuZhi2, Unigene68108\_JingChaoZuZhi2, Unigene68139\_JingChaoZuZhi2, Unigene6815\_JingChaoZuZhi2, Unigene6828\_JingChaoZuZhi2, Unigene68527\_JingChaoZuZhi2, Unigene68672\_JingChaoZuZhi2, Unigene6872\_JingChaoZuZhi2, Unigene69092\_JingChaoZuZhi2, Unigene69141\_JingChaoZuZhi2, Unigene6914\_JingChaoZuZhi2, Unigene69203\_JingChaoZuZhi2, Unigene69221\_JingChaoZuZhi2, Unigene6933\_JingChaoZuZhi2, Unigene69386\_JingChaoZuZhi2, Unigene69562\_JingChaoZuZhi2, Unigene69775\_JingChaoZuZhi2, Unigene70063\_JingChaoZuZhi2, Unigene7016\_JingChaoZuZhi2, Unigene70329\_JingChaoZuZhi2, Unigene7032\_JingChaoZuZhi2, Unigene70549\_JingChaoZuZhi2, Unigene70723\_JingChaoZuZhi2, Unigene70970\_JingChaoZuZhi2, Unigene71043\_JingChaoZuZhi2, Unigene7116\_JingChaoZuZhi2, Unigene7118\_JingChaoZuZhi2, Unigene71213\_JingChaoZuZhi2, Unigene71241\_JingChaoZuZhi2, Unigene71365\_JingChaoZuZhi2, Unigene71385\_JingChaoZuZhi2, Unigene71546\_JingChaoZuZhi2, Unigene71774\_JingChaoZuZhi2, Unigene72249\_JingChaoZuZhi2, Unigene72334\_JingChaoZuZhi2, Unigene72351\_JingChaoZuZhi2, Unigene72503\_JingChaoZuZhi2, Unigene7268\_JingChaoZuZhi2, Unigene72708\_JingChaoZuZhi2, Unigene72832\_JingChaoZuZhi2, Unigene73257\_JingChaoZuZhi2, Unigene73446\_JingChaoZuZhi2, Unigene73475\_JingChaoZuZhi2, Unigene73817\_JingChaoZuZhi2, Unigene73887\_JingChaoZuZhi2, Unigene741\_JingChaoZuZhi2, Unigene74221\_JingChaoZuZhi2, Unigene74259\_JingChaoZuZhi2, Unigene74298\_JingChaoZuZhi2, Unigene74474\_JingChaoZuZhi2, Unigene7456\_JingChaoZuZhi2, Unigene74719\_JingChaoZuZhi2, Unigene74730\_JingChaoZuZhi2, Unigene74854\_JingChaoZuZhi2, Unigene7489\_JingChaoZuZhi2, Unigene75009\_JingChaoZuZhi2, Unigene7508\_JingChaoZuZhi2, Unigene75112\_JingChaoZuZhi2, Unigene75167\_JingChaoZuZhi2, Unigene75235\_JingChaoZuZhi2, Unigene75899\_JingChaoZuZhi2, Unigene75902\_JingChaoZuZhi2, Unigene75946\_JingChaoZuZhi2, Unigene76002\_JingChaoZuZhi2, Unigene76111\_JingChaoZuZhi2, Unigene76269\_JingChaoZuZhi2, Unigene76302\_JingChaoZuZhi2, Unigene7634\_JingChaoZuZhi2, Unigene76515\_JingChaoZuZhi2, Unigene7663\_JingChaoZuZhi2, Unigene76776\_JingChaoZuZhi2, Unigene76785\_JingChaoZuZhi2, Unigene76819\_JingChaoZuZhi2, Unigene76917\_JingChaoZuZhi2, Unigene77061\_JingChaoZuZhi2, Unigene77149\_JingChaoZuZhi2, Unigene77406\_JingChaoZuZhi2, Unigene77413\_JingChaoZuZhi2, Unigene77520\_JingChaoZuZhi2, Unigene77570\_JingChaoZuZhi2, Unigene7794\_JingChaoZuZhi2, Unigene78009\_JingChaoZuZhi2, Unigene78078\_JingChaoZuZhi2, Unigene78235\_JingChaoZuZhi2, Unigene78288\_JingChaoZuZhi2, Unigene78361\_JingChaoZuZhi2, Unigene78410\_JingChaoZuZhi2, Unigene7850\_JingChaoZuZhi2, Unigene78521\_JingChaoZuZhi2, Unigene78555\_JingChaoZuZhi2, Unigene78636\_JingChaoZuZhi2, Unigene78909\_JingChaoZuZhi2, Unigene79058\_JingChaoZuZhi2, Unigene79118\_JingChaoZuZhi2, Unigene7911\_JingChaoZuZhi2, Unigene79141\_JingChaoZuZhi2, Unigene79358\_JingChaoZuZhi2, Unigene79433\_JingChaoZuZhi2, Unigene79443\_JingChaoZuZhi2, Unigene79653\_JingChaoZuZhi2, Unigene79726\_JingChaoZuZhi2, Unigene79789\_JingChaoZuZhi2, Unigene79824\_JingChaoZuZhi2, Unigene79831\_JingChaoZuZhi2, Unigene79860\_JingChaoZuZhi2, Unigene79864\_JingChaoZuZhi2, Unigene7987\_JingChaoZuZhi2, Unigene79882\_JingChaoZuZhi2, Unigene79911\_JingChaoZuZhi2, Unigene79940\_JingChaoZuZhi2, Unigene80143\_JingChaoZuZhi2, Unigene80212\_JingChaoZuZhi2, Unigene80331\_JingChaoZuZhi2, Unigene80403\_JingChaoZuZhi2, Unigene80619\_JingChaoZuZhi2, Unigene80893\_JingChaoZuZhi2, Unigene80980\_JingChaoZuZhi2, Unigene81167\_JingChaoZuZhi2, Unigene81327\_JingChaoZuZhi2, Unigene81440\_JingChaoZuZhi2, Unigene81450\_JingChaoZuZhi2, Unigene81561\_JingChaoZuZhi2, Unigene81599\_JingChaoZuZhi2, Unigene81686\_JingChaoZuZhi2, Unigene81757\_JingChaoZuZhi2, Unigene81825\_JingChaoZuZhi2, Unigene81904\_JingChaoZuZhi2, Unigene8191\_JingChaoZuZhi2, Unigene82047\_JingChaoZuZhi2, Unigene82070\_JingChaoZuZhi2, Unigene82111\_JingChaoZuZhi2, Unigene82215\_JingChaoZuZhi2, Unigene82578\_JingChaoZuZhi2, Unigene82662\_JingChaoZuZhi2, Unigene82688\_JingChaoZuZhi2, Unigene83017\_JingChaoZuZhi2, Unigene83079\_JingChaoZuZhi2, Unigene83214\_JingChaoZuZhi2, Unigene83667\_JingChaoZuZhi2, Unigene83766\_JingChaoZuZhi2, Unigene83782\_JingChaoZuZhi2, Unigene83825\_JingChaoZuZhi2, Unigene8392\_JingChaoZuZhi2, Unigene83980\_JingChaoZuZhi2, Unigene8406\_JingChaoZuZhi2, Unigene84076\_JingChaoZuZhi2, Unigene84115\_JingChaoZuZhi2, Unigene84152\_JingChaoZuZhi2, Unigene84168\_JingChaoZuZhi2, Unigene841\_JingChaoZuZhi2, Unigene84212\_JingChaoZuZhi2, Unigene8443\_JingChaoZuZhi2, Unigene84533\_JingChaoZuZhi2, Unigene84566\_JingChaoZuZhi2, Unigene84748\_JingChaoZuZhi2, Unigene84830\_JingChaoZuZhi2, Unigene84945\_JingChaoZuZhi2, Unigene84993\_JingChaoZuZhi2, Unigene851\_JingChaoZuZhi2, Unigene85314\_JingChaoZuZhi2, Unigene85464\_JingChaoZuZhi2, Unigene85574\_JingChaoZuZhi2, Unigene85770\_JingChaoZuZhi2, Unigene85902\_JingChaoZuZhi2, Unigene86020\_JingChaoZuZhi2, Unigene86091\_JingChaoZuZhi2, Unigene86206\_JingChaoZuZhi2, Unigene8621\_JingChaoZuZhi2, Unigene86349\_JingChaoZuZhi2, Unigene86526\_JingChaoZuZhi2, Unigene86546\_JingChaoZuZhi2, Unigene8661\_JingChaoZuZhi2, Unigene86713\_JingChaoZuZhi2, Unigene86789\_JingChaoZuZhi2, Unigene86899\_JingChaoZuZhi2, Unigene87082\_JingChaoZuZhi2, Unigene87203\_JingChaoZuZhi2, Unigene87256\_JingChaoZuZhi2, Unigene87305\_JingChaoZuZhi2, Unigene87332\_JingChaoZuZhi2, Unigene87362\_JingChaoZuZhi2, Unigene87365\_JingChaoZuZhi2, Unigene87459\_JingChaoZuZhi2, Unigene87478\_JingChaoZuZhi2, Unigene87488\_JingChaoZuZhi2, Unigene87533\_JingChaoZuZhi2, Unigene87607\_JingChaoZuZhi2, Unigene876\_JingChaoZuZhi2, Unigene87774\_JingChaoZuZhi2, Unigene87780\_JingChaoZuZhi2, Unigene87851\_JingChaoZuZhi2, Unigene87853\_JingChaoZuZhi2, Unigene87946\_JingChaoZuZhi2, Unigene88185\_JingChaoZuZhi2, Unigene8833\_JingChaoZuZhi2, Unigene88385\_JingChaoZuZhi2, Unigene88391\_JingChaoZuZhi2, Unigene88400\_JingChaoZuZhi2, Unigene88406\_JingChaoZuZhi2, Unigene88442\_JingChaoZuZhi2, Unigene88470\_JingChaoZuZhi2, Unigene88608\_JingChaoZuZhi2, Unigene8860\_JingChaoZuZhi2, Unigene88683\_JingChaoZuZhi2, Unigene88719\_JingChaoZuZhi2, Unigene88738\_JingChaoZuZhi2, Unigene88741\_JingChaoZuZhi2, Unigene88773\_JingChaoZuZhi2, Unigene88778\_JingChaoZuZhi2, Unigene88779\_JingChaoZuZhi2, Unigene88781\_JingChaoZuZhi2, Unigene88797\_JingChaoZuZhi2, Unigene88830\_JingChaoZuZhi2, Unigene88837\_JingChaoZuZhi2, Unigene88945\_JingChaoZuZhi2, Unigene88979\_JingChaoZuZhi2, Unigene88988\_JingChaoZuZhi2, Unigene89011\_JingChaoZuZhi2, Unigene89013\_JingChaoZuZhi2, Unigene89093\_JingChaoZuZhi2, Unigene89121\_JingChaoZuZhi2, Unigene89310\_JingChaoZuZhi2, Unigene89323\_JingChaoZuZhi2, Unigene89337\_JingChaoZuZhi2, Unigene89352\_JingChaoZuZhi2, Unigene89367\_JingChaoZuZhi2, Unigene89541\_JingChaoZuZhi2, Unigene89603\_JingChaoZuZhi2, Unigene89807\_JingChaoZuZhi2, Unigene89834\_JingChaoZuZhi2, Unigene89867\_JingChaoZuZhi2, Unigene90038\_JingChaoZuZhi2, Unigene90066\_JingChaoZuZhi2, Unigene90071\_JingChaoZuZhi2, Unigene90209\_JingChaoZuZhi2, Unigene90341\_JingChaoZuZhi2, Unigene9050\_JingChaoZuZhi2, Unigene90530\_JingChaoZuZhi2, Unigene90564\_JingChaoZuZhi2, Unigene90714\_JingChaoZuZhi2, Unigene90779\_JingChaoZuZhi2, Unigene90953\_JingChaoZuZhi2, Unigene90995\_JingChaoZuZhi2, Unigene91179\_JingChaoZuZhi2, Unigene91221\_JingChaoZuZhi2, Unigene91379\_JingChaoZuZhi2, Unigene91390\_JingChaoZuZhi2, Unigene91456\_JingChaoZuZhi2, Unigene91487\_JingChaoZuZhi2, Unigene91527\_JingChaoZuZhi2, Unigene91529\_JingChaoZuZhi2, Unigene91553\_JingChaoZuZhi2, Unigene91612\_JingChaoZuZhi2, Unigene91703\_JingChaoZuZhi2, Unigene91739\_JingChaoZuZhi2, Unigene91848\_JingChaoZuZhi2, Unigene91958\_JingChaoZuZhi2, Unigene91978\_JingChaoZuZhi2, Unigene92059\_JingChaoZuZhi2, Unigene92112\_JingChaoZuZhi2, Unigene92119\_JingChaoZuZhi2, Unigene92346\_JingChaoZuZhi2, Unigene92372\_JingChaoZuZhi2, Unigene92580\_JingChaoZuZhi2, Unigene92681\_JingChaoZuZhi2, Unigene92700\_JingChaoZuZhi2, Unigene92780\_JingChaoZuZhi2, Unigene92838\_JingChaoZuZhi2, Unigene92951\_JingChaoZuZhi2, Unigene93246\_JingChaoZuZhi2, Unigene93439\_JingChaoZuZhi2, Unigene93552\_JingChaoZuZhi2, Unigene93573\_JingChaoZuZhi2, Unigene93592\_JingChaoZuZhi2, Unigene93610\_JingChaoZuZhi2, Unigene93742\_JingChaoZuZhi2, Unigene93782\_JingChaoZuZhi2, Unigene93793\_JingChaoZuZhi2, Unigene93807\_JingChaoZuZhi2, Unigene93821\_JingChaoZuZhi2, Unigene9389\_JingChaoZuZhi2, Unigene93979\_JingChaoZuZhi2, Unigene94083\_JingChaoZuZhi2, Unigene94120\_JingChaoZuZhi2, Unigene94129\_JingChaoZuZhi2, Unigene94134\_JingChaoZuZhi2, Unigene94149\_JingChaoZuZhi2, Unigene94208\_JingChaoZuZhi2, Unigene94404\_JingChaoZuZhi2, Unigene94456\_JingChaoZuZhi2, Unigene94471\_JingChaoZuZhi2, Unigene94520\_JingChaoZuZhi2, Unigene94544\_JingChaoZuZhi2, Unigene94584\_JingChaoZuZhi2, Unigene94685\_JingChaoZuZhi2, Unigene94903\_JingChaoZuZhi2, Unigene94968\_JingChaoZuZhi2, Unigene95088\_JingChaoZuZhi2, Unigene95098\_JingChaoZuZhi2, Unigene95119\_JingChaoZuZhi2, Unigene95295\_JingChaoZuZhi2, Unigene95371\_JingChaoZuZhi2, Unigene9540\_JingChaoZuZhi2, Unigene95456\_JingChaoZuZhi2, Unigene95589\_JingChaoZuZhi2, Unigene95677\_JingChaoZuZhi2, Unigene95741\_JingChaoZuZhi2, Unigene95812\_JingChaoZuZhi2, Unigene95875\_JingChaoZuZhi2, Unigene95881\_JingChaoZuZhi2, Unigene96294\_JingChaoZuZhi2, Unigene9688\_JingChaoZuZhi2, Unigene9807\_JingChaoZuZhi2, Unigene9833\_JingChaoZuZhi2, Unigene9902\_JingChaoZuZhi2, Unigene9992\_JingChaoZuZhi2 |
| 15 | Focal adhesion | Unigene10012\_JingChaoZuZhi2, Unigene10047\_JingChaoZuZhi2, Unigene10053\_JingChaoZuZhi2, Unigene10073\_JingChaoZuZhi2, Unigene10078\_JingChaoZuZhi2, Unigene10218\_JingChaoZuZhi2, Unigene10259\_JingChaoZuZhi2, Unigene10286\_JingChaoZuZhi2, Unigene10318\_JingChaoZuZhi2, Unigene10362\_JingChaoZuZhi2, Unigene10422\_JingChaoZuZhi2, Unigene10597\_JingChaoZuZhi2, Unigene1060\_JingChaoZuZhi2, Unigene10630\_JingChaoZuZhi2, Unigene10637\_JingChaoZuZhi2, Unigene10679\_JingChaoZuZhi2, Unigene10755\_JingChaoZuZhi2, Unigene10760\_JingChaoZuZhi2, Unigene10809\_JingChaoZuZhi2, Unigene10922\_JingChaoZuZhi2, Unigene10999\_JingChaoZuZhi2, Unigene10\_JingChaoZuZhi2, Unigene11000\_JingChaoZuZhi2, Unigene1111\_JingChaoZuZhi2, Unigene11251\_JingChaoZuZhi2, Unigene11446\_JingChaoZuZhi2, Unigene11464\_JingChaoZuZhi2, Unigene1162\_JingChaoZuZhi2, Unigene11747\_JingChaoZuZhi2, Unigene11834\_JingChaoZuZhi2, Unigene11851\_JingChaoZuZhi2, Unigene11889\_JingChaoZuZhi2, Unigene11919\_JingChaoZuZhi2, Unigene11951\_JingChaoZuZhi2, Unigene11961\_JingChaoZuZhi2, Unigene11982\_JingChaoZuZhi2, Unigene12060\_JingChaoZuZhi2, Unigene12105\_JingChaoZuZhi2, Unigene1227\_JingChaoZuZhi2, Unigene12376\_JingChaoZuZhi2, Unigene12420\_JingChaoZuZhi2, Unigene12577\_JingChaoZuZhi2, Unigene12595\_JingChaoZuZhi2, Unigene12652\_JingChaoZuZhi2, Unigene12665\_JingChaoZuZhi2, Unigene12850\_JingChaoZuZhi2, Unigene12937\_JingChaoZuZhi2, Unigene12972\_JingChaoZuZhi2, Unigene13078\_JingChaoZuZhi2, Unigene13097\_JingChaoZuZhi2, Unigene13132\_JingChaoZuZhi2, Unigene13149\_JingChaoZuZhi2, Unigene13176\_JingChaoZuZhi2, Unigene13180\_JingChaoZuZhi2, Unigene13285\_JingChaoZuZhi2, Unigene13342\_JingChaoZuZhi2, Unigene13389\_JingChaoZuZhi2, Unigene13390\_JingChaoZuZhi2, Unigene13464\_JingChaoZuZhi2, Unigene13509\_JingChaoZuZhi2, Unigene13510\_JingChaoZuZhi2, Unigene13588\_JingChaoZuZhi2, Unigene13866\_JingChaoZuZhi2, Unigene13894\_JingChaoZuZhi2, Unigene13925\_JingChaoZuZhi2, Unigene13927\_JingChaoZuZhi2, Unigene13952\_JingChaoZuZhi2, Unigene13978\_JingChaoZuZhi2, Unigene14056\_JingChaoZuZhi2, Unigene14068\_JingChaoZuZhi2, Unigene1413\_JingChaoZuZhi2, Unigene14225\_JingChaoZuZhi2, Unigene14252\_JingChaoZuZhi2, Unigene14401\_JingChaoZuZhi2, Unigene14476\_JingChaoZuZhi2, Unigene14492\_JingChaoZuZhi2, Unigene14912\_JingChaoZuZhi2, Unigene14948\_JingChaoZuZhi2, Unigene15095\_JingChaoZuZhi2, Unigene15228\_JingChaoZuZhi2, Unigene15470\_JingChaoZuZhi2, Unigene15535\_JingChaoZuZhi2, Unigene15560\_JingChaoZuZhi2, Unigene15777\_JingChaoZuZhi2, Unigene15794\_JingChaoZuZhi2, Unigene15814\_JingChaoZuZhi2, Unigene15879\_JingChaoZuZhi2, Unigene15883\_JingChaoZuZhi2, Unigene16004\_JingChaoZuZhi2, Unigene16008\_JingChaoZuZhi2, Unigene16072\_JingChaoZuZhi2, Unigene16121\_JingChaoZuZhi2, Unigene1616\_JingChaoZuZhi2, Unigene16240\_JingChaoZuZhi2, Unigene16318\_JingChaoZuZhi2, Unigene16324\_JingChaoZuZhi2, Unigene16467\_JingChaoZuZhi2, Unigene1651\_JingChaoZuZhi2, Unigene16709\_JingChaoZuZhi2, Unigene16778\_JingChaoZuZhi2, Unigene16820\_JingChaoZuZhi2, Unigene16837\_JingChaoZuZhi2, Unigene16873\_JingChaoZuZhi2, Unigene16970\_JingChaoZuZhi2, Unigene17128\_JingChaoZuZhi2, Unigene17190\_JingChaoZuZhi2, Unigene17226\_JingChaoZuZhi2, Unigene17243\_JingChaoZuZhi2, Unigene17247\_JingChaoZuZhi2, Unigene17382\_JingChaoZuZhi2, Unigene17506\_JingChaoZuZhi2, Unigene17537\_JingChaoZuZhi2, Unigene17675\_JingChaoZuZhi2, Unigene17754\_JingChaoZuZhi2, Unigene17810\_JingChaoZuZhi2, Unigene17821\_JingChaoZuZhi2, Unigene17829\_JingChaoZuZhi2, Unigene17839\_JingChaoZuZhi2, Unigene17946\_JingChaoZuZhi2, Unigene17977\_JingChaoZuZhi2, Unigene18309\_JingChaoZuZhi2, Unigene18360\_JingChaoZuZhi2, Unigene18361\_JingChaoZuZhi2, Unigene18426\_JingChaoZuZhi2, Unigene18441\_JingChaoZuZhi2, Unigene18462\_JingChaoZuZhi2, Unigene18608\_JingChaoZuZhi2, Unigene18672\_JingChaoZuZhi2, Unigene18715\_JingChaoZuZhi2, Unigene18730\_JingChaoZuZhi2, Unigene18780\_JingChaoZuZhi2, Unigene18815\_JingChaoZuZhi2, Unigene19021\_JingChaoZuZhi2, Unigene19046\_JingChaoZuZhi2, Unigene19339\_JingChaoZuZhi2, Unigene19365\_JingChaoZuZhi2, Unigene19404\_JingChaoZuZhi2, Unigene19466\_JingChaoZuZhi2, Unigene19512\_JingChaoZuZhi2, Unigene19545\_JingChaoZuZhi2, Unigene19679\_JingChaoZuZhi2, Unigene19826\_JingChaoZuZhi2, Unigene20159\_JingChaoZuZhi2, Unigene20220\_JingChaoZuZhi2, Unigene20223\_JingChaoZuZhi2, Unigene20297\_JingChaoZuZhi2, Unigene20298\_JingChaoZuZhi2, Unigene20305\_JingChaoZuZhi2, Unigene20367\_JingChaoZuZhi2, Unigene20394\_JingChaoZuZhi2, Unigene20608\_JingChaoZuZhi2, Unigene20611\_JingChaoZuZhi2, Unigene20629\_JingChaoZuZhi2, Unigene20697\_JingChaoZuZhi2, Unigene20748\_JingChaoZuZhi2, Unigene20794\_JingChaoZuZhi2, Unigene20893\_JingChaoZuZhi2, Unigene20897\_JingChaoZuZhi2, Unigene21028\_JingChaoZuZhi2, Unigene21167\_JingChaoZuZhi2, Unigene21253\_JingChaoZuZhi2, Unigene21490\_JingChaoZuZhi2, Unigene21513\_JingChaoZuZhi2, Unigene21536\_JingChaoZuZhi2, Unigene21571\_JingChaoZuZhi2, Unigene21575\_JingChaoZuZhi2, Unigene21578\_JingChaoZuZhi2, Unigene21605\_JingChaoZuZhi2, Unigene21713\_JingChaoZuZhi2, Unigene21804\_JingChaoZuZhi2, Unigene21807\_JingChaoZuZhi2, Unigene21809\_JingChaoZuZhi2, Unigene21818\_JingChaoZuZhi2, Unigene21888\_JingChaoZuZhi2, Unigene21973\_JingChaoZuZhi2, Unigene22024\_JingChaoZuZhi2, Unigene22174\_JingChaoZuZhi2, Unigene22245\_JingChaoZuZhi2, Unigene22251\_JingChaoZuZhi2, Unigene22379\_JingChaoZuZhi2, Unigene22445\_JingChaoZuZhi2, Unigene22464\_JingChaoZuZhi2, Unigene22482\_JingChaoZuZhi2, Unigene22487\_JingChaoZuZhi2, Unigene22517\_JingChaoZuZhi2, Unigene22572\_JingChaoZuZhi2, Unigene22624\_JingChaoZuZhi2, Unigene22638\_JingChaoZuZhi2, Unigene22652\_JingChaoZuZhi2, Unigene22737\_JingChaoZuZhi2, Unigene22779\_JingChaoZuZhi2, Unigene22788\_JingChaoZuZhi2, Unigene22804\_JingChaoZuZhi2, Unigene22917\_JingChaoZuZhi2, Unigene2379\_JingChaoZuZhi2, Unigene23978\_JingChaoZuZhi2, Unigene2456\_JingChaoZuZhi2, Unigene2465\_JingChaoZuZhi2, Unigene24772\_JingChaoZuZhi2, Unigene2503\_JingChaoZuZhi2, Unigene26299\_JingChaoZuZhi2, Unigene26438\_JingChaoZuZhi2, Unigene26730\_JingChaoZuZhi2, Unigene26987\_JingChaoZuZhi2, Unigene2728\_JingChaoZuZhi2, Unigene2790\_JingChaoZuZhi2, Unigene27933\_JingChaoZuZhi2, Unigene28208\_JingChaoZuZhi2, Unigene28467\_JingChaoZuZhi2, Unigene29312\_JingChaoZuZhi2, Unigene29488\_JingChaoZuZhi2, Unigene2960\_JingChaoZuZhi2, Unigene30593\_JingChaoZuZhi2, Unigene30726\_JingChaoZuZhi2, Unigene3131\_JingChaoZuZhi2, Unigene31612\_JingChaoZuZhi2, Unigene31918\_JingChaoZuZhi2, Unigene31924\_JingChaoZuZhi2, Unigene31934\_JingChaoZuZhi2, Unigene3206\_JingChaoZuZhi2, Unigene328\_JingChaoZuZhi2, Unigene3292\_JingChaoZuZhi2, Unigene33930\_JingChaoZuZhi2, Unigene34091\_JingChaoZuZhi2, Unigene3422\_JingChaoZuZhi2, Unigene34807\_JingChaoZuZhi2, Unigene35016\_JingChaoZuZhi2, Unigene35102\_JingChaoZuZhi2, Unigene35243\_JingChaoZuZhi2, Unigene3550\_JingChaoZuZhi2, Unigene35624\_JingChaoZuZhi2, Unigene3585\_JingChaoZuZhi2, Unigene35872\_JingChaoZuZhi2, Unigene36479\_JingChaoZuZhi2, Unigene3693\_JingChaoZuZhi2, Unigene37495\_JingChaoZuZhi2, Unigene3763\_JingChaoZuZhi2, Unigene39003\_JingChaoZuZhi2, Unigene39102\_JingChaoZuZhi2, Unigene3931\_JingChaoZuZhi2, Unigene39674\_JingChaoZuZhi2, Unigene39865\_JingChaoZuZhi2, Unigene40020\_JingChaoZuZhi2, Unigene40528\_JingChaoZuZhi2, Unigene40763\_JingChaoZuZhi2, Unigene40919\_JingChaoZuZhi2, Unigene41164\_JingChaoZuZhi2, Unigene41209\_JingChaoZuZhi2, Unigene41213\_JingChaoZuZhi2, Unigene41298\_JingChaoZuZhi2, Unigene41571\_JingChaoZuZhi2, Unigene41700\_JingChaoZuZhi2, Unigene4186\_JingChaoZuZhi2, Unigene4188\_JingChaoZuZhi2, Unigene42248\_JingChaoZuZhi2, Unigene42256\_JingChaoZuZhi2, Unigene4294\_JingChaoZuZhi2, Unigene4345\_JingChaoZuZhi2, Unigene4377\_JingChaoZuZhi2, Unigene43871\_JingChaoZuZhi2, Unigene44190\_JingChaoZuZhi2, Unigene44191\_JingChaoZuZhi2, Unigene44454\_JingChaoZuZhi2, Unigene451\_JingChaoZuZhi2, Unigene45739\_JingChaoZuZhi2, Unigene45782\_JingChaoZuZhi2, Unigene45901\_JingChaoZuZhi2, Unigene4592\_JingChaoZuZhi2, Unigene4599\_JingChaoZuZhi2, Unigene46407\_JingChaoZuZhi2, Unigene46628\_JingChaoZuZhi2, Unigene47126\_JingChaoZuZhi2, Unigene47461\_JingChaoZuZhi2, Unigene47566\_JingChaoZuZhi2, Unigene4775\_JingChaoZuZhi2, Unigene4781\_JingChaoZuZhi2, Unigene47872\_JingChaoZuZhi2, Unigene48280\_JingChaoZuZhi2, Unigene4838\_JingChaoZuZhi2, Unigene4841\_JingChaoZuZhi2, Unigene48673\_JingChaoZuZhi2, Unigene48751\_JingChaoZuZhi2, Unigene48843\_JingChaoZuZhi2, Unigene48882\_JingChaoZuZhi2, Unigene4935\_JingChaoZuZhi2, Unigene49798\_JingChaoZuZhi2, Unigene49913\_JingChaoZuZhi2, Unigene499\_JingChaoZuZhi2, Unigene50231\_JingChaoZuZhi2, Unigene5069\_JingChaoZuZhi2, Unigene50744\_JingChaoZuZhi2, Unigene51056\_JingChaoZuZhi2, Unigene51231\_JingChaoZuZhi2, Unigene51467\_JingChaoZuZhi2, Unigene51496\_JingChaoZuZhi2, Unigene51512\_JingChaoZuZhi2, Unigene51567\_JingChaoZuZhi2, Unigene51966\_JingChaoZuZhi2, Unigene5196\_JingChaoZuZhi2, Unigene52004\_JingChaoZuZhi2, Unigene5204\_JingChaoZuZhi2, Unigene52292\_JingChaoZuZhi2, Unigene5265\_JingChaoZuZhi2, Unigene52727\_JingChaoZuZhi2, Unigene5312\_JingChaoZuZhi2, Unigene53503\_JingChaoZuZhi2, Unigene53524\_JingChaoZuZhi2, Unigene53885\_JingChaoZuZhi2, Unigene53917\_JingChaoZuZhi2, Unigene53958\_JingChaoZuZhi2, Unigene5418\_JingChaoZuZhi2, Unigene54470\_JingChaoZuZhi2, Unigene54679\_JingChaoZuZhi2, Unigene54744\_JingChaoZuZhi2, Unigene54986\_JingChaoZuZhi2, Unigene55239\_JingChaoZuZhi2, Unigene55693\_JingChaoZuZhi2, Unigene55839\_JingChaoZuZhi2, Unigene55913\_JingChaoZuZhi2, Unigene56456\_JingChaoZuZhi2, Unigene57488\_JingChaoZuZhi2, Unigene5796\_JingChaoZuZhi2, Unigene58405\_JingChaoZuZhi2, Unigene58670\_JingChaoZuZhi2, Unigene59146\_JingChaoZuZhi2, Unigene5914\_JingChaoZuZhi2, Unigene59510\_JingChaoZuZhi2, Unigene59797\_JingChaoZuZhi2, Unigene6028\_JingChaoZuZhi2, Unigene61244\_JingChaoZuZhi2, Unigene6170\_JingChaoZuZhi2, Unigene62251\_JingChaoZuZhi2, Unigene62431\_JingChaoZuZhi2, Unigene62590\_JingChaoZuZhi2, Unigene62786\_JingChaoZuZhi2, Unigene62919\_JingChaoZuZhi2, Unigene63547\_JingChaoZuZhi2, Unigene63799\_JingChaoZuZhi2, Unigene63812\_JingChaoZuZhi2, Unigene6392\_JingChaoZuZhi2, Unigene64164\_JingChaoZuZhi2, Unigene6433\_JingChaoZuZhi2, Unigene64583\_JingChaoZuZhi2, Unigene64750\_JingChaoZuZhi2, Unigene64830\_JingChaoZuZhi2, Unigene64843\_JingChaoZuZhi2, Unigene6493\_JingChaoZuZhi2, Unigene6498\_JingChaoZuZhi2, Unigene65022\_JingChaoZuZhi2, Unigene65879\_JingChaoZuZhi2, Unigene66013\_JingChaoZuZhi2, Unigene66253\_JingChaoZuZhi2, Unigene6634\_JingChaoZuZhi2, Unigene66575\_JingChaoZuZhi2, Unigene66700\_JingChaoZuZhi2, Unigene6676\_JingChaoZuZhi2, Unigene66813\_JingChaoZuZhi2, Unigene6688\_JingChaoZuZhi2, Unigene66913\_JingChaoZuZhi2, Unigene6699\_JingChaoZuZhi2, Unigene67052\_JingChaoZuZhi2, Unigene67081\_JingChaoZuZhi2, Unigene67117\_JingChaoZuZhi2, Unigene67365\_JingChaoZuZhi2, Unigene67367\_JingChaoZuZhi2, Unigene67542\_JingChaoZuZhi2, Unigene67793\_JingChaoZuZhi2, Unigene67811\_JingChaoZuZhi2, Unigene67983\_JingChaoZuZhi2, Unigene68159\_JingChaoZuZhi2, Unigene68262\_JingChaoZuZhi2, Unigene68283\_JingChaoZuZhi2, Unigene6828\_JingChaoZuZhi2, Unigene68495\_JingChaoZuZhi2, Unigene69100\_JingChaoZuZhi2, Unigene69402\_JingChaoZuZhi2, Unigene69658\_JingChaoZuZhi2, Unigene70155\_JingChaoZuZhi2, Unigene70549\_JingChaoZuZhi2, Unigene7068\_JingChaoZuZhi2, Unigene70696\_JingChaoZuZhi2, Unigene70723\_JingChaoZuZhi2, Unigene71144\_JingChaoZuZhi2, Unigene71592\_JingChaoZuZhi2, Unigene71662\_JingChaoZuZhi2, Unigene71755\_JingChaoZuZhi2, Unigene7186\_JingChaoZuZhi2, Unigene7219\_JingChaoZuZhi2, Unigene72521\_JingChaoZuZhi2, Unigene72550\_JingChaoZuZhi2, Unigene7326\_JingChaoZuZhi2, Unigene73446\_JingChaoZuZhi2, Unigene73826\_JingChaoZuZhi2, Unigene74199\_JingChaoZuZhi2, Unigene74795\_JingChaoZuZhi2, Unigene75341\_JingChaoZuZhi2, Unigene75840\_JingChaoZuZhi2, Unigene75859\_JingChaoZuZhi2, Unigene76069\_JingChaoZuZhi2, Unigene76111\_JingChaoZuZhi2, Unigene76400\_JingChaoZuZhi2, Unigene76485\_JingChaoZuZhi2, Unigene76592\_JingChaoZuZhi2, Unigene7670\_JingChaoZuZhi2, Unigene76763\_JingChaoZuZhi2, Unigene76805\_JingChaoZuZhi2, Unigene76917\_JingChaoZuZhi2, Unigene77061\_JingChaoZuZhi2, Unigene77406\_JingChaoZuZhi2, Unigene77549\_JingChaoZuZhi2, Unigene7793\_JingChaoZuZhi2, Unigene77955\_JingChaoZuZhi2, Unigene78225\_JingChaoZuZhi2, Unigene78574\_JingChaoZuZhi2, Unigene78909\_JingChaoZuZhi2, Unigene79077\_JingChaoZuZhi2, Unigene79109\_JingChaoZuZhi2, Unigene79187\_JingChaoZuZhi2, Unigene79274\_JingChaoZuZhi2, Unigene7939\_JingChaoZuZhi2, Unigene79411\_JingChaoZuZhi2, Unigene79681\_JingChaoZuZhi2, Unigene7977\_JingChaoZuZhi2, Unigene79828\_JingChaoZuZhi2, Unigene7985\_JingChaoZuZhi2, Unigene79871\_JingChaoZuZhi2, Unigene79882\_JingChaoZuZhi2, Unigene80038\_JingChaoZuZhi2, Unigene8006\_JingChaoZuZhi2, Unigene80246\_JingChaoZuZhi2, Unigene80251\_JingChaoZuZhi2, Unigene80331\_JingChaoZuZhi2, Unigene80818\_JingChaoZuZhi2, Unigene8133\_JingChaoZuZhi2, Unigene81431\_JingChaoZuZhi2, Unigene81471\_JingChaoZuZhi2, Unigene81592\_JingChaoZuZhi2, Unigene81598\_JingChaoZuZhi2, Unigene81670\_JingChaoZuZhi2, Unigene81780\_JingChaoZuZhi2, Unigene81903\_JingChaoZuZhi2, Unigene81904\_JingChaoZuZhi2, Unigene8191\_JingChaoZuZhi2, Unigene8192\_JingChaoZuZhi2, Unigene81964\_JingChaoZuZhi2, Unigene81\_JingChaoZuZhi2, Unigene82117\_JingChaoZuZhi2, Unigene82215\_JingChaoZuZhi2, Unigene82389\_JingChaoZuZhi2, Unigene82489\_JingChaoZuZhi2, Unigene8248\_JingChaoZuZhi2, Unigene82662\_JingChaoZuZhi2, Unigene82761\_JingChaoZuZhi2, Unigene828\_JingChaoZuZhi2, Unigene83345\_JingChaoZuZhi2, Unigene83396\_JingChaoZuZhi2, Unigene83507\_JingChaoZuZhi2, Unigene83583\_JingChaoZuZhi2, Unigene83663\_JingChaoZuZhi2, Unigene83744\_JingChaoZuZhi2, Unigene83872\_JingChaoZuZhi2, Unigene84257\_JingChaoZuZhi2, Unigene84381\_JingChaoZuZhi2, Unigene84519\_JingChaoZuZhi2, Unigene84944\_JingChaoZuZhi2, Unigene85066\_JingChaoZuZhi2, Unigene85132\_JingChaoZuZhi2, Unigene85193\_JingChaoZuZhi2, Unigene85198\_JingChaoZuZhi2, Unigene85214\_JingChaoZuZhi2, Unigene85503\_JingChaoZuZhi2, Unigene85574\_JingChaoZuZhi2, Unigene85577\_JingChaoZuZhi2, Unigene85578\_JingChaoZuZhi2, Unigene85695\_JingChaoZuZhi2, Unigene85921\_JingChaoZuZhi2, Unigene85923\_JingChaoZuZhi2, Unigene86036\_JingChaoZuZhi2, Unigene86091\_JingChaoZuZhi2, Unigene86186\_JingChaoZuZhi2, Unigene86206\_JingChaoZuZhi2, Unigene8621\_JingChaoZuZhi2, Unigene86278\_JingChaoZuZhi2, Unigene86296\_JingChaoZuZhi2, Unigene86349\_JingChaoZuZhi2, Unigene86456\_JingChaoZuZhi2, Unigene86459\_JingChaoZuZhi2, Unigene86470\_JingChaoZuZhi2, Unigene86526\_JingChaoZuZhi2, Unigene86579\_JingChaoZuZhi2, Unigene86640\_JingChaoZuZhi2, Unigene86661\_JingChaoZuZhi2, Unigene86669\_JingChaoZuZhi2, Unigene86713\_JingChaoZuZhi2, Unigene86789\_JingChaoZuZhi2, Unigene86812\_JingChaoZuZhi2, Unigene87082\_JingChaoZuZhi2, Unigene87180\_JingChaoZuZhi2, Unigene87191\_JingChaoZuZhi2, Unigene87332\_JingChaoZuZhi2, Unigene87448\_JingChaoZuZhi2, Unigene87818\_JingChaoZuZhi2, Unigene87851\_JingChaoZuZhi2, Unigene87952\_JingChaoZuZhi2, Unigene88090\_JingChaoZuZhi2, Unigene88185\_JingChaoZuZhi2, Unigene88342\_JingChaoZuZhi2, Unigene88391\_JingChaoZuZhi2, Unigene88522\_JingChaoZuZhi2, Unigene88719\_JingChaoZuZhi2, Unigene88748\_JingChaoZuZhi2, Unigene88798\_JingChaoZuZhi2, Unigene88799\_JingChaoZuZhi2, Unigene8891\_JingChaoZuZhi2, Unigene88925\_JingChaoZuZhi2, Unigene88988\_JingChaoZuZhi2, Unigene88990\_JingChaoZuZhi2, Unigene89045\_JingChaoZuZhi2, Unigene89060\_JingChaoZuZhi2, Unigene89310\_JingChaoZuZhi2, Unigene89329\_JingChaoZuZhi2, Unigene89710\_JingChaoZuZhi2, Unigene90003\_JingChaoZuZhi2, Unigene90078\_JingChaoZuZhi2, Unigene90201\_JingChaoZuZhi2, Unigene90261\_JingChaoZuZhi2, Unigene9038\_JingChaoZuZhi2, Unigene90439\_JingChaoZuZhi2, Unigene90454\_JingChaoZuZhi2, Unigene9049\_JingChaoZuZhi2, Unigene90521\_JingChaoZuZhi2, Unigene90715\_JingChaoZuZhi2, Unigene90747\_JingChaoZuZhi2, Unigene90953\_JingChaoZuZhi2, Unigene9102\_JingChaoZuZhi2, Unigene91038\_JingChaoZuZhi2, Unigene91163\_JingChaoZuZhi2, Unigene91179\_JingChaoZuZhi2, Unigene91289\_JingChaoZuZhi2, Unigene91553\_JingChaoZuZhi2, Unigene91574\_JingChaoZuZhi2, Unigene91612\_JingChaoZuZhi2, Unigene91613\_JingChaoZuZhi2, Unigene91751\_JingChaoZuZhi2, Unigene91848\_JingChaoZuZhi2, Unigene91906\_JingChaoZuZhi2, Unigene91958\_JingChaoZuZhi2, Unigene91971\_JingChaoZuZhi2, Unigene91983\_JingChaoZuZhi2, Unigene92104\_JingChaoZuZhi2, Unigene92112\_JingChaoZuZhi2, Unigene92119\_JingChaoZuZhi2, Unigene92295\_JingChaoZuZhi2, Unigene92315\_JingChaoZuZhi2, Unigene92372\_JingChaoZuZhi2, Unigene92395\_JingChaoZuZhi2, Unigene92443\_JingChaoZuZhi2, Unigene92492\_JingChaoZuZhi2, Unigene92607\_JingChaoZuZhi2, Unigene9260\_JingChaoZuZhi2, Unigene92671\_JingChaoZuZhi2, Unigene92786\_JingChaoZuZhi2, Unigene92831\_JingChaoZuZhi2, Unigene92862\_JingChaoZuZhi2, Unigene92929\_JingChaoZuZhi2, Unigene92995\_JingChaoZuZhi2, Unigene93195\_JingChaoZuZhi2, Unigene93271\_JingChaoZuZhi2, Unigene93374\_JingChaoZuZhi2, Unigene93501\_JingChaoZuZhi2, Unigene93630\_JingChaoZuZhi2, Unigene93702\_JingChaoZuZhi2, Unigene93760\_JingChaoZuZhi2, Unigene93768\_JingChaoZuZhi2, Unigene93782\_JingChaoZuZhi2, Unigene93793\_JingChaoZuZhi2, Unigene93807\_JingChaoZuZhi2, Unigene93820\_JingChaoZuZhi2, Unigene93821\_JingChaoZuZhi2, Unigene93857\_JingChaoZuZhi2, Unigene93990\_JingChaoZuZhi2, Unigene93998\_JingChaoZuZhi2, Unigene9402\_JingChaoZuZhi2, Unigene94110\_JingChaoZuZhi2, Unigene94149\_JingChaoZuZhi2, Unigene94204\_JingChaoZuZhi2, Unigene94208\_JingChaoZuZhi2, Unigene94272\_JingChaoZuZhi2, Unigene94370\_JingChaoZuZhi2, Unigene94434\_JingChaoZuZhi2, Unigene94456\_JingChaoZuZhi2, Unigene94476\_JingChaoZuZhi2, Unigene94544\_JingChaoZuZhi2, Unigene94561\_JingChaoZuZhi2, Unigene94656\_JingChaoZuZhi2, Unigene94685\_JingChaoZuZhi2, Unigene94872\_JingChaoZuZhi2, Unigene94974\_JingChaoZuZhi2, Unigene95020\_JingChaoZuZhi2, Unigene95065\_JingChaoZuZhi2, Unigene95098\_JingChaoZuZhi2, Unigene951\_JingChaoZuZhi2, Unigene95212\_JingChaoZuZhi2, Unigene95295\_JingChaoZuZhi2, Unigene95359\_JingChaoZuZhi2, Unigene95421\_JingChaoZuZhi2, Unigene95456\_JingChaoZuZhi2, Unigene95607\_JingChaoZuZhi2, Unigene95674\_JingChaoZuZhi2, Unigene95677\_JingChaoZuZhi2, Unigene95699\_JingChaoZuZhi2, Unigene95782\_JingChaoZuZhi2, Unigene95791\_JingChaoZuZhi2, Unigene95799\_JingChaoZuZhi2, Unigene95807\_JingChaoZuZhi2, Unigene95821\_JingChaoZuZhi2, Unigene95822\_JingChaoZuZhi2, Unigene95828\_JingChaoZuZhi2, Unigene95853\_JingChaoZuZhi2, Unigene95875\_JingChaoZuZhi2, Unigene95881\_JingChaoZuZhi2, Unigene95907\_JingChaoZuZhi2, Unigene96033\_JingChaoZuZhi2, Unigene96042\_JingChaoZuZhi2, Unigene96057\_JingChaoZuZhi2, Unigene96084\_JingChaoZuZhi2, Unigene96121\_JingChaoZuZhi2, Unigene96138\_JingChaoZuZhi2, Unigene96202\_JingChaoZuZhi2, Unigene96234\_JingChaoZuZhi2, Unigene96258\_JingChaoZuZhi2, Unigene96269\_JingChaoZuZhi2, Unigene96271\_JingChaoZuZhi2, Unigene96286\_JingChaoZuZhi2, Unigene96294\_JingChaoZuZhi2, Unigene96300\_JingChaoZuZhi2, Unigene9739\_JingChaoZuZhi2, Unigene9767\_JingChaoZuZhi2, Unigene9811\_JingChaoZuZhi2, Unigene9867\_JingChaoZuZhi2, Unigene9892\_JingChaoZuZhi2, Unigene9902\_JingChaoZuZhi2, Unigene9946\_JingChaoZuZhi2 |
| 16 | Huntington's disease | Unigene10005\_JingChaoZuZhi2, Unigene10031\_JingChaoZuZhi2, Unigene10129\_JingChaoZuZhi2, Unigene10362\_JingChaoZuZhi2, Unigene10392\_JingChaoZuZhi2, Unigene103\_JingChaoZuZhi2, Unigene10496\_JingChaoZuZhi2, Unigene1082\_JingChaoZuZhi2, Unigene10835\_JingChaoZuZhi2, Unigene10883\_JingChaoZuZhi2, Unigene10888\_JingChaoZuZhi2, Unigene10939\_JingChaoZuZhi2, Unigene10976\_JingChaoZuZhi2, Unigene11134\_JingChaoZuZhi2, Unigene11230\_JingChaoZuZhi2, Unigene11372\_JingChaoZuZhi2, Unigene1154\_JingChaoZuZhi2, Unigene1164\_JingChaoZuZhi2, Unigene11664\_JingChaoZuZhi2, Unigene11700\_JingChaoZuZhi2, Unigene11963\_JingChaoZuZhi2, Unigene12073\_JingChaoZuZhi2, Unigene12189\_JingChaoZuZhi2, Unigene12244\_JingChaoZuZhi2, Unigene1229\_JingChaoZuZhi2, Unigene12634\_JingChaoZuZhi2, Unigene12706\_JingChaoZuZhi2, Unigene12872\_JingChaoZuZhi2, Unigene12881\_JingChaoZuZhi2, Unigene1313\_JingChaoZuZhi2, Unigene1323\_JingChaoZuZhi2, Unigene13457\_JingChaoZuZhi2, Unigene13583\_JingChaoZuZhi2, Unigene13584\_JingChaoZuZhi2, Unigene13650\_JingChaoZuZhi2, Unigene13715\_JingChaoZuZhi2, Unigene13795\_JingChaoZuZhi2, Unigene13803\_JingChaoZuZhi2, Unigene13878\_JingChaoZuZhi2, Unigene13988\_JingChaoZuZhi2, Unigene14135\_JingChaoZuZhi2, Unigene14151\_JingChaoZuZhi2, Unigene14164\_JingChaoZuZhi2, Unigene14203\_JingChaoZuZhi2, Unigene14298\_JingChaoZuZhi2, Unigene14360\_JingChaoZuZhi2, Unigene14522\_JingChaoZuZhi2, Unigene14551\_JingChaoZuZhi2, Unigene14631\_JingChaoZuZhi2, Unigene14634\_JingChaoZuZhi2, Unigene1468\_JingChaoZuZhi2, Unigene14746\_JingChaoZuZhi2, Unigene14865\_JingChaoZuZhi2, Unigene14871\_JingChaoZuZhi2, Unigene15288\_JingChaoZuZhi2, Unigene15465\_JingChaoZuZhi2, Unigene15539\_JingChaoZuZhi2, Unigene15627\_JingChaoZuZhi2, Unigene15648\_JingChaoZuZhi2, Unigene15681\_JingChaoZuZhi2, Unigene15867\_JingChaoZuZhi2, Unigene15893\_JingChaoZuZhi2, Unigene16232\_JingChaoZuZhi2, Unigene16295\_JingChaoZuZhi2, Unigene16302\_JingChaoZuZhi2, Unigene16317\_JingChaoZuZhi2, Unigene16337\_JingChaoZuZhi2, Unigene16468\_JingChaoZuZhi2, Unigene16524\_JingChaoZuZhi2, Unigene16589\_JingChaoZuZhi2, Unigene16752\_JingChaoZuZhi2, Unigene16786\_JingChaoZuZhi2, Unigene16877\_JingChaoZuZhi2, Unigene16958\_JingChaoZuZhi2, Unigene16991\_JingChaoZuZhi2, Unigene16996\_JingChaoZuZhi2, Unigene17015\_JingChaoZuZhi2, Unigene17035\_JingChaoZuZhi2, Unigene17065\_JingChaoZuZhi2, Unigene17099\_JingChaoZuZhi2, Unigene17151\_JingChaoZuZhi2, Unigene17190\_JingChaoZuZhi2, Unigene17195\_JingChaoZuZhi2, Unigene17423\_JingChaoZuZhi2, Unigene1744\_JingChaoZuZhi2, Unigene17466\_JingChaoZuZhi2, Unigene17562\_JingChaoZuZhi2, Unigene17636\_JingChaoZuZhi2, Unigene17696\_JingChaoZuZhi2, Unigene1778\_JingChaoZuZhi2, Unigene18000\_JingChaoZuZhi2, Unigene18010\_JingChaoZuZhi2, Unigene18099\_JingChaoZuZhi2, Unigene18260\_JingChaoZuZhi2, Unigene18475\_JingChaoZuZhi2, Unigene18507\_JingChaoZuZhi2, Unigene18874\_JingChaoZuZhi2, Unigene19006\_JingChaoZuZhi2, Unigene19075\_JingChaoZuZhi2, Unigene19126\_JingChaoZuZhi2, Unigene19238\_JingChaoZuZhi2, Unigene19256\_JingChaoZuZhi2, Unigene19283\_JingChaoZuZhi2, Unigene19475\_JingChaoZuZhi2, Unigene19476\_JingChaoZuZhi2, Unigene19510\_JingChaoZuZhi2, Unigene19647\_JingChaoZuZhi2, Unigene19710\_JingChaoZuZhi2, Unigene19838\_JingChaoZuZhi2, Unigene19979\_JingChaoZuZhi2, Unigene20050\_JingChaoZuZhi2, Unigene20052\_JingChaoZuZhi2, Unigene20087\_JingChaoZuZhi2, Unigene2012\_JingChaoZuZhi2, Unigene20150\_JingChaoZuZhi2, Unigene20172\_JingChaoZuZhi2, Unigene20209\_JingChaoZuZhi2, Unigene20219\_JingChaoZuZhi2, Unigene20350\_JingChaoZuZhi2, Unigene20441\_JingChaoZuZhi2, Unigene2046\_JingChaoZuZhi2, Unigene20553\_JingChaoZuZhi2, Unigene20711\_JingChaoZuZhi2, Unigene20814\_JingChaoZuZhi2, Unigene20826\_JingChaoZuZhi2, Unigene20962\_JingChaoZuZhi2, Unigene20992\_JingChaoZuZhi2, Unigene21054\_JingChaoZuZhi2, Unigene21321\_JingChaoZuZhi2, Unigene2132\_JingChaoZuZhi2, Unigene21537\_JingChaoZuZhi2, Unigene21556\_JingChaoZuZhi2, Unigene2155\_JingChaoZuZhi2, Unigene21587\_JingChaoZuZhi2, Unigene21648\_JingChaoZuZhi2, Unigene2186\_JingChaoZuZhi2, Unigene21917\_JingChaoZuZhi2, Unigene21938\_JingChaoZuZhi2, Unigene22008\_JingChaoZuZhi2, Unigene22034\_JingChaoZuZhi2, Unigene22196\_JingChaoZuZhi2, Unigene22254\_JingChaoZuZhi2, Unigene22263\_JingChaoZuZhi2, Unigene22310\_JingChaoZuZhi2, Unigene22334\_JingChaoZuZhi2, Unigene22451\_JingChaoZuZhi2, Unigene22545\_JingChaoZuZhi2, Unigene2254\_JingChaoZuZhi2, Unigene22580\_JingChaoZuZhi2, Unigene22673\_JingChaoZuZhi2, Unigene22708\_JingChaoZuZhi2, Unigene22842\_JingChaoZuZhi2, Unigene22864\_JingChaoZuZhi2, Unigene22870\_JingChaoZuZhi2, Unigene22892\_JingChaoZuZhi2, Unigene22908\_JingChaoZuZhi2, Unigene22911\_JingChaoZuZhi2, Unigene22923\_JingChaoZuZhi2, Unigene23504\_JingChaoZuZhi2, Unigene23946\_JingChaoZuZhi2, Unigene24040\_JingChaoZuZhi2, Unigene246\_JingChaoZuZhi2, Unigene2473\_JingChaoZuZhi2, Unigene24861\_JingChaoZuZhi2, Unigene25749\_JingChaoZuZhi2, Unigene26108\_JingChaoZuZhi2, Unigene2707\_JingChaoZuZhi2, Unigene2804\_JingChaoZuZhi2, Unigene28474\_JingChaoZuZhi2, Unigene28539\_JingChaoZuZhi2, Unigene2899\_JingChaoZuZhi2, Unigene29151\_JingChaoZuZhi2, Unigene2916\_JingChaoZuZhi2, Unigene29250\_JingChaoZuZhi2, Unigene29273\_JingChaoZuZhi2, Unigene3071\_JingChaoZuZhi2, Unigene31068\_JingChaoZuZhi2, Unigene31166\_JingChaoZuZhi2, Unigene31327\_JingChaoZuZhi2, Unigene31355\_JingChaoZuZhi2, Unigene31511\_JingChaoZuZhi2, Unigene31768\_JingChaoZuZhi2, Unigene322\_JingChaoZuZhi2, Unigene32827\_JingChaoZuZhi2, Unigene3291\_JingChaoZuZhi2, Unigene33470\_JingChaoZuZhi2, Unigene33523\_JingChaoZuZhi2, Unigene33784\_JingChaoZuZhi2, Unigene34397\_JingChaoZuZhi2, Unigene344\_JingChaoZuZhi2, Unigene34929\_JingChaoZuZhi2, Unigene35168\_JingChaoZuZhi2, Unigene35366\_JingChaoZuZhi2, Unigene35457\_JingChaoZuZhi2, Unigene3591\_JingChaoZuZhi2, Unigene3592\_JingChaoZuZhi2, Unigene36230\_JingChaoZuZhi2, Unigene3632\_JingChaoZuZhi2, Unigene3682\_JingChaoZuZhi2, Unigene37028\_JingChaoZuZhi2, Unigene37191\_JingChaoZuZhi2, Unigene37224\_JingChaoZuZhi2, Unigene37457\_JingChaoZuZhi2, Unigene37495\_JingChaoZuZhi2, Unigene37504\_JingChaoZuZhi2, Unigene37516\_JingChaoZuZhi2, Unigene37928\_JingChaoZuZhi2, Unigene38162\_JingChaoZuZhi2, Unigene385\_JingChaoZuZhi2, Unigene38886\_JingChaoZuZhi2, Unigene38940\_JingChaoZuZhi2, Unigene38980\_JingChaoZuZhi2, Unigene39374\_JingChaoZuZhi2, Unigene39389\_JingChaoZuZhi2, Unigene3938\_JingChaoZuZhi2, Unigene395\_JingChaoZuZhi2, Unigene39624\_JingChaoZuZhi2, Unigene39825\_JingChaoZuZhi2, Unigene40865\_JingChaoZuZhi2, Unigene4111\_JingChaoZuZhi2, Unigene41148\_JingChaoZuZhi2, Unigene41155\_JingChaoZuZhi2, Unigene41227\_JingChaoZuZhi2, Unigene41333\_JingChaoZuZhi2, Unigene414\_JingChaoZuZhi2, Unigene41683\_JingChaoZuZhi2, Unigene41686\_JingChaoZuZhi2, Unigene41982\_JingChaoZuZhi2, Unigene42646\_JingChaoZuZhi2, Unigene42975\_JingChaoZuZhi2, Unigene43059\_JingChaoZuZhi2, Unigene43072\_JingChaoZuZhi2, Unigene43410\_JingChaoZuZhi2, Unigene4346\_JingChaoZuZhi2, Unigene4375\_JingChaoZuZhi2, Unigene44078\_JingChaoZuZhi2, Unigene4413\_JingChaoZuZhi2, Unigene44252\_JingChaoZuZhi2, Unigene4426\_JingChaoZuZhi2, Unigene4531\_JingChaoZuZhi2, Unigene4539\_JingChaoZuZhi2, Unigene45673\_JingChaoZuZhi2, Unigene46190\_JingChaoZuZhi2, Unigene4620\_JingChaoZuZhi2, Unigene46240\_JingChaoZuZhi2, Unigene47145\_JingChaoZuZhi2, Unigene47187\_JingChaoZuZhi2, Unigene47202\_JingChaoZuZhi2, Unigene47794\_JingChaoZuZhi2, Unigene48095\_JingChaoZuZhi2, Unigene4816\_JingChaoZuZhi2, Unigene4829\_JingChaoZuZhi2, Unigene48393\_JingChaoZuZhi2, Unigene486\_JingChaoZuZhi2, Unigene4871\_JingChaoZuZhi2, Unigene49345\_JingChaoZuZhi2, Unigene49378\_JingChaoZuZhi2, Unigene49985\_JingChaoZuZhi2, Unigene49987\_JingChaoZuZhi2, Unigene50307\_JingChaoZuZhi2, Unigene51115\_JingChaoZuZhi2, Unigene51540\_JingChaoZuZhi2, Unigene51835\_JingChaoZuZhi2, Unigene5188\_JingChaoZuZhi2, Unigene5200\_JingChaoZuZhi2, Unigene52036\_JingChaoZuZhi2, Unigene52383\_JingChaoZuZhi2, Unigene52474\_JingChaoZuZhi2, Unigene5318\_JingChaoZuZhi2, Unigene53310\_JingChaoZuZhi2, Unigene53709\_JingChaoZuZhi2, Unigene53836\_JingChaoZuZhi2, Unigene53843\_JingChaoZuZhi2, Unigene54031\_JingChaoZuZhi2, Unigene54171\_JingChaoZuZhi2, Unigene54236\_JingChaoZuZhi2, Unigene542\_JingChaoZuZhi2, Unigene54367\_JingChaoZuZhi2, Unigene5484\_JingChaoZuZhi2, Unigene55303\_JingChaoZuZhi2, Unigene55529\_JingChaoZuZhi2, Unigene5552\_JingChaoZuZhi2, Unigene55604\_JingChaoZuZhi2, Unigene55617\_JingChaoZuZhi2, Unigene55644\_JingChaoZuZhi2, Unigene55677\_JingChaoZuZhi2, Unigene55700\_JingChaoZuZhi2, Unigene55745\_JingChaoZuZhi2, Unigene56386\_JingChaoZuZhi2, Unigene56567\_JingChaoZuZhi2, Unigene56843\_JingChaoZuZhi2, Unigene56889\_JingChaoZuZhi2, Unigene5742\_JingChaoZuZhi2, Unigene57878\_JingChaoZuZhi2, Unigene58428\_JingChaoZuZhi2, Unigene58474\_JingChaoZuZhi2, Unigene58502\_JingChaoZuZhi2, Unigene5897\_JingChaoZuZhi2, Unigene59197\_JingChaoZuZhi2, Unigene59270\_JingChaoZuZhi2, Unigene5976\_JingChaoZuZhi2, Unigene59958\_JingChaoZuZhi2, Unigene6011\_JingChaoZuZhi2, Unigene6077\_JingChaoZuZhi2, Unigene60921\_JingChaoZuZhi2, Unigene6121\_JingChaoZuZhi2, Unigene6134\_JingChaoZuZhi2, Unigene6135\_JingChaoZuZhi2, Unigene61651\_JingChaoZuZhi2, Unigene6166\_JingChaoZuZhi2, Unigene61\_JingChaoZuZhi2, Unigene6219\_JingChaoZuZhi2, Unigene62528\_JingChaoZuZhi2, Unigene630\_JingChaoZuZhi2, Unigene63112\_JingChaoZuZhi2, Unigene63170\_JingChaoZuZhi2, Unigene63175\_JingChaoZuZhi2, Unigene63323\_JingChaoZuZhi2, Unigene63341\_JingChaoZuZhi2, Unigene63406\_JingChaoZuZhi2, Unigene63663\_JingChaoZuZhi2, Unigene63786\_JingChaoZuZhi2, Unigene63935\_JingChaoZuZhi2, Unigene63990\_JingChaoZuZhi2, Unigene63994\_JingChaoZuZhi2, Unigene64058\_JingChaoZuZhi2, Unigene6409\_JingChaoZuZhi2, Unigene64497\_JingChaoZuZhi2, Unigene6467\_JingChaoZuZhi2, Unigene64903\_JingChaoZuZhi2, Unigene64960\_JingChaoZuZhi2, Unigene65063\_JingChaoZuZhi2, Unigene65424\_JingChaoZuZhi2, Unigene65445\_JingChaoZuZhi2, Unigene65475\_JingChaoZuZhi2, Unigene65515\_JingChaoZuZhi2, Unigene65775\_JingChaoZuZhi2, Unigene65800\_JingChaoZuZhi2, Unigene65807\_JingChaoZuZhi2, Unigene65808\_JingChaoZuZhi2, Unigene65866\_JingChaoZuZhi2, Unigene65951\_JingChaoZuZhi2, Unigene66540\_JingChaoZuZhi2, Unigene66627\_JingChaoZuZhi2, Unigene66954\_JingChaoZuZhi2, Unigene67337\_JingChaoZuZhi2, Unigene67367\_JingChaoZuZhi2, Unigene67430\_JingChaoZuZhi2, Unigene6790\_JingChaoZuZhi2, Unigene67964\_JingChaoZuZhi2, Unigene68129\_JingChaoZuZhi2, Unigene68229\_JingChaoZuZhi2, Unigene68298\_JingChaoZuZhi2, Unigene685\_JingChaoZuZhi2, Unigene68698\_JingChaoZuZhi2, Unigene68832\_JingChaoZuZhi2, Unigene69342\_JingChaoZuZhi2, Unigene69875\_JingChaoZuZhi2, Unigene70493\_JingChaoZuZhi2, Unigene70647\_JingChaoZuZhi2, Unigene7082\_JingChaoZuZhi2, Unigene71153\_JingChaoZuZhi2, Unigene71228\_JingChaoZuZhi2, Unigene7176\_JingChaoZuZhi2, Unigene7178\_JingChaoZuZhi2, Unigene7185\_JingChaoZuZhi2, Unigene71965\_JingChaoZuZhi2, Unigene72188\_JingChaoZuZhi2, Unigene7219\_JingChaoZuZhi2, Unigene7238\_JingChaoZuZhi2, Unigene72461\_JingChaoZuZhi2, Unigene72643\_JingChaoZuZhi2, Unigene72813\_JingChaoZuZhi2, Unigene72860\_JingChaoZuZhi2, Unigene72886\_JingChaoZuZhi2, Unigene73543\_JingChaoZuZhi2, Unigene73674\_JingChaoZuZhi2, Unigene73890\_JingChaoZuZhi2, Unigene74410\_JingChaoZuZhi2, Unigene74553\_JingChaoZuZhi2, Unigene74609\_JingChaoZuZhi2, Unigene75033\_JingChaoZuZhi2, Unigene75400\_JingChaoZuZhi2, Unigene76109\_JingChaoZuZhi2, Unigene76269\_JingChaoZuZhi2, Unigene76331\_JingChaoZuZhi2, Unigene76647\_JingChaoZuZhi2, Unigene76868\_JingChaoZuZhi2, Unigene76896\_JingChaoZuZhi2, Unigene77071\_JingChaoZuZhi2, Unigene7712\_JingChaoZuZhi2, Unigene77149\_JingChaoZuZhi2, Unigene77259\_JingChaoZuZhi2, Unigene77449\_JingChaoZuZhi2, Unigene77559\_JingChaoZuZhi2, Unigene77631\_JingChaoZuZhi2, Unigene77844\_JingChaoZuZhi2, Unigene78071\_JingChaoZuZhi2, Unigene78078\_JingChaoZuZhi2, Unigene78268\_JingChaoZuZhi2, Unigene78415\_JingChaoZuZhi2, Unigene78435\_JingChaoZuZhi2, Unigene7850\_JingChaoZuZhi2, Unigene78522\_JingChaoZuZhi2, Unigene78548\_JingChaoZuZhi2, Unigene78619\_JingChaoZuZhi2, Unigene78904\_JingChaoZuZhi2, Unigene78998\_JingChaoZuZhi2, Unigene79118\_JingChaoZuZhi2, Unigene79149\_JingChaoZuZhi2, Unigene79210\_JingChaoZuZhi2, Unigene79211\_JingChaoZuZhi2, Unigene7948\_JingChaoZuZhi2, Unigene79523\_JingChaoZuZhi2, Unigene79687\_JingChaoZuZhi2, Unigene79892\_JingChaoZuZhi2, Unigene7990\_JingChaoZuZhi2, Unigene79999\_JingChaoZuZhi2, Unigene80037\_JingChaoZuZhi2, Unigene80123\_JingChaoZuZhi2, Unigene80681\_JingChaoZuZhi2, Unigene81043\_JingChaoZuZhi2, Unigene81236\_JingChaoZuZhi2, Unigene81273\_JingChaoZuZhi2, Unigene81297\_JingChaoZuZhi2, Unigene8151\_JingChaoZuZhi2, Unigene81674\_JingChaoZuZhi2, Unigene81706\_JingChaoZuZhi2, Unigene81716\_JingChaoZuZhi2, Unigene81879\_JingChaoZuZhi2, Unigene81959\_JingChaoZuZhi2, Unigene82155\_JingChaoZuZhi2, Unigene82186\_JingChaoZuZhi2, Unigene8223\_JingChaoZuZhi2, Unigene8225\_JingChaoZuZhi2, Unigene82321\_JingChaoZuZhi2, Unigene82734\_JingChaoZuZhi2, Unigene82835\_JingChaoZuZhi2, Unigene828\_JingChaoZuZhi2, Unigene82924\_JingChaoZuZhi2, Unigene83101\_JingChaoZuZhi2, Unigene8310\_JingChaoZuZhi2, Unigene83161\_JingChaoZuZhi2, Unigene83188\_JingChaoZuZhi2, Unigene83206\_JingChaoZuZhi2, Unigene83219\_JingChaoZuZhi2, Unigene83409\_JingChaoZuZhi2, Unigene83448\_JingChaoZuZhi2, Unigene8391\_JingChaoZuZhi2, Unigene84050\_JingChaoZuZhi2, Unigene84077\_JingChaoZuZhi2, Unigene84181\_JingChaoZuZhi2, Unigene84203\_JingChaoZuZhi2, Unigene84247\_JingChaoZuZhi2, Unigene84436\_JingChaoZuZhi2, Unigene84592\_JingChaoZuZhi2, Unigene84623\_JingChaoZuZhi2, Unigene84930\_JingChaoZuZhi2, Unigene85035\_JingChaoZuZhi2, Unigene8506\_JingChaoZuZhi2, Unigene8508\_JingChaoZuZhi2, Unigene8517\_JingChaoZuZhi2, Unigene85391\_JingChaoZuZhi2, Unigene8552\_JingChaoZuZhi2, Unigene85930\_JingChaoZuZhi2, Unigene8609\_JingChaoZuZhi2, Unigene86119\_JingChaoZuZhi2, Unigene86157\_JingChaoZuZhi2, Unigene86367\_JingChaoZuZhi2, Unigene86504\_JingChaoZuZhi2, Unigene86528\_JingChaoZuZhi2, Unigene86582\_JingChaoZuZhi2, Unigene86821\_JingChaoZuZhi2, Unigene86874\_JingChaoZuZhi2, Unigene86945\_JingChaoZuZhi2, Unigene86958\_JingChaoZuZhi2, Unigene86967\_JingChaoZuZhi2, Unigene87015\_JingChaoZuZhi2, Unigene87349\_JingChaoZuZhi2, Unigene87389\_JingChaoZuZhi2, Unigene87416\_JingChaoZuZhi2, Unigene87433\_JingChaoZuZhi2, Unigene8755\_JingChaoZuZhi2, Unigene87607\_JingChaoZuZhi2, Unigene87740\_JingChaoZuZhi2, Unigene87741\_JingChaoZuZhi2, Unigene87796\_JingChaoZuZhi2, Unigene87855\_JingChaoZuZhi2, Unigene87948\_JingChaoZuZhi2, Unigene87997\_JingChaoZuZhi2, Unigene87\_JingChaoZuZhi2, Unigene88016\_JingChaoZuZhi2, Unigene88135\_JingChaoZuZhi2, Unigene88179\_JingChaoZuZhi2, Unigene88213\_JingChaoZuZhi2, Unigene88270\_JingChaoZuZhi2, Unigene88283\_JingChaoZuZhi2, Unigene88312\_JingChaoZuZhi2, Unigene8837\_JingChaoZuZhi2, Unigene88385\_JingChaoZuZhi2, Unigene88393\_JingChaoZuZhi2, Unigene88773\_JingChaoZuZhi2, Unigene88933\_JingChaoZuZhi2, Unigene88937\_JingChaoZuZhi2, Unigene8897\_JingChaoZuZhi2, Unigene89093\_JingChaoZuZhi2, Unigene89147\_JingChaoZuZhi2, Unigene89161\_JingChaoZuZhi2, Unigene89210\_JingChaoZuZhi2, Unigene89245\_JingChaoZuZhi2, Unigene89265\_JingChaoZuZhi2, Unigene89557\_JingChaoZuZhi2, Unigene89629\_JingChaoZuZhi2, Unigene89653\_JingChaoZuZhi2, Unigene89667\_JingChaoZuZhi2, Unigene89670\_JingChaoZuZhi2, Unigene89753\_JingChaoZuZhi2, Unigene89769\_JingChaoZuZhi2, Unigene90077\_JingChaoZuZhi2, Unigene90180\_JingChaoZuZhi2, Unigene9020\_JingChaoZuZhi2, Unigene90348\_JingChaoZuZhi2, Unigene90358\_JingChaoZuZhi2, Unigene90535\_JingChaoZuZhi2, Unigene90611\_JingChaoZuZhi2, Unigene90612\_JingChaoZuZhi2, Unigene90666\_JingChaoZuZhi2, Unigene90919\_JingChaoZuZhi2, Unigene91020\_JingChaoZuZhi2, Unigene91051\_JingChaoZuZhi2, Unigene91081\_JingChaoZuZhi2, Unigene91119\_JingChaoZuZhi2, Unigene91122\_JingChaoZuZhi2, Unigene91187\_JingChaoZuZhi2, Unigene91194\_JingChaoZuZhi2, Unigene91530\_JingChaoZuZhi2, Unigene91580\_JingChaoZuZhi2, Unigene91702\_JingChaoZuZhi2, Unigene91739\_JingChaoZuZhi2, Unigene91777\_JingChaoZuZhi2, Unigene91855\_JingChaoZuZhi2, Unigene91921\_JingChaoZuZhi2, Unigene91944\_JingChaoZuZhi2, Unigene91973\_JingChaoZuZhi2, Unigene92115\_JingChaoZuZhi2, Unigene92187\_JingChaoZuZhi2, Unigene92224\_JingChaoZuZhi2, Unigene92240\_JingChaoZuZhi2, Unigene92294\_JingChaoZuZhi2, Unigene92312\_JingChaoZuZhi2, Unigene92321\_JingChaoZuZhi2, Unigene92368\_JingChaoZuZhi2, Unigene92429\_JingChaoZuZhi2, Unigene92539\_JingChaoZuZhi2, Unigene9256\_JingChaoZuZhi2, Unigene92590\_JingChaoZuZhi2, Unigene92607\_JingChaoZuZhi2, Unigene92651\_JingChaoZuZhi2, Unigene92715\_JingChaoZuZhi2, Unigene92742\_JingChaoZuZhi2, Unigene92910\_JingChaoZuZhi2, Unigene92989\_JingChaoZuZhi2, Unigene93012\_JingChaoZuZhi2, Unigene93072\_JingChaoZuZhi2, Unigene93095\_JingChaoZuZhi2, Unigene93186\_JingChaoZuZhi2, Unigene93195\_JingChaoZuZhi2, Unigene93265\_JingChaoZuZhi2, Unigene93296\_JingChaoZuZhi2, Unigene93406\_JingChaoZuZhi2, Unigene93445\_JingChaoZuZhi2, Unigene93541\_JingChaoZuZhi2, Unigene93604\_JingChaoZuZhi2, Unigene93701\_JingChaoZuZhi2, Unigene93904\_JingChaoZuZhi2, Unigene93993\_JingChaoZuZhi2, Unigene94011\_JingChaoZuZhi2, Unigene94029\_JingChaoZuZhi2, Unigene94128\_JingChaoZuZhi2, Unigene94201\_JingChaoZuZhi2, Unigene94242\_JingChaoZuZhi2, Unigene94276\_JingChaoZuZhi2, Unigene9428\_JingChaoZuZhi2, Unigene94364\_JingChaoZuZhi2, Unigene94457\_JingChaoZuZhi2, Unigene94568\_JingChaoZuZhi2, Unigene94630\_JingChaoZuZhi2, Unigene94656\_JingChaoZuZhi2, Unigene94711\_JingChaoZuZhi2, Unigene94728\_JingChaoZuZhi2, Unigene94831\_JingChaoZuZhi2, Unigene94890\_JingChaoZuZhi2, Unigene94910\_JingChaoZuZhi2, Unigene94946\_JingChaoZuZhi2, Unigene95002\_JingChaoZuZhi2, Unigene95041\_JingChaoZuZhi2, Unigene95069\_JingChaoZuZhi2, Unigene95159\_JingChaoZuZhi2, Unigene95212\_JingChaoZuZhi2, Unigene9529\_JingChaoZuZhi2, Unigene95308\_JingChaoZuZhi2, Unigene9550\_JingChaoZuZhi2, Unigene95568\_JingChaoZuZhi2, Unigene95677\_JingChaoZuZhi2, Unigene95689\_JingChaoZuZhi2, Unigene9569\_JingChaoZuZhi2, Unigene95742\_JingChaoZuZhi2, Unigene95855\_JingChaoZuZhi2, Unigene95858\_JingChaoZuZhi2, Unigene95987\_JingChaoZuZhi2, Unigene95994\_JingChaoZuZhi2, Unigene96068\_JingChaoZuZhi2, Unigene96114\_JingChaoZuZhi2, Unigene96124\_JingChaoZuZhi2, Unigene96171\_JingChaoZuZhi2, Unigene96240\_JingChaoZuZhi2, Unigene96248\_JingChaoZuZhi2, Unigene96262\_JingChaoZuZhi2, Unigene96275\_JingChaoZuZhi2, Unigene963\_JingChaoZuZhi2, Unigene9645\_JingChaoZuZhi2, Unigene9654\_JingChaoZuZhi2, Unigene9812\_JingChaoZuZhi2, Unigene9925\_JingChaoZuZhi2 |
| 17 | MAPK signaling pathway | Unigene10012\_JingChaoZuZhi2, Unigene10016\_JingChaoZuZhi2, Unigene1001\_JingChaoZuZhi2, Unigene10053\_JingChaoZuZhi2, Unigene10218\_JingChaoZuZhi2, Unigene10290\_JingChaoZuZhi2, Unigene10294\_JingChaoZuZhi2, Unigene10318\_JingChaoZuZhi2, Unigene10339\_JingChaoZuZhi2, Unigene10350\_JingChaoZuZhi2, Unigene10363\_JingChaoZuZhi2, Unigene10380\_JingChaoZuZhi2, Unigene10422\_JingChaoZuZhi2, Unigene10555\_JingChaoZuZhi2, Unigene1075\_JingChaoZuZhi2, Unigene10857\_JingChaoZuZhi2, Unigene11055\_JingChaoZuZhi2, Unigene1109\_JingChaoZuZhi2, Unigene11251\_JingChaoZuZhi2, Unigene11287\_JingChaoZuZhi2, Unigene11545\_JingChaoZuZhi2, Unigene1160\_JingChaoZuZhi2, Unigene11636\_JingChaoZuZhi2, Unigene11732\_JingChaoZuZhi2, Unigene11951\_JingChaoZuZhi2, Unigene12171\_JingChaoZuZhi2, Unigene12214\_JingChaoZuZhi2, Unigene12219\_JingChaoZuZhi2, Unigene12260\_JingChaoZuZhi2, Unigene12329\_JingChaoZuZhi2, Unigene12420\_JingChaoZuZhi2, Unigene12570\_JingChaoZuZhi2, Unigene12836\_JingChaoZuZhi2, Unigene12912\_JingChaoZuZhi2, Unigene1301\_JingChaoZuZhi2, Unigene13077\_JingChaoZuZhi2, Unigene13115\_JingChaoZuZhi2, Unigene13332\_JingChaoZuZhi2, Unigene13354\_JingChaoZuZhi2, Unigene13395\_JingChaoZuZhi2, Unigene13420\_JingChaoZuZhi2, Unigene13854\_JingChaoZuZhi2, Unigene13869\_JingChaoZuZhi2, Unigene13919\_JingChaoZuZhi2, Unigene13996\_JingChaoZuZhi2, Unigene14054\_JingChaoZuZhi2, Unigene14234\_JingChaoZuZhi2, Unigene14250\_JingChaoZuZhi2, Unigene14317\_JingChaoZuZhi2, Unigene14357\_JingChaoZuZhi2, Unigene14370\_JingChaoZuZhi2, Unigene14409\_JingChaoZuZhi2, Unigene14488\_JingChaoZuZhi2, Unigene14635\_JingChaoZuZhi2, Unigene14688\_JingChaoZuZhi2, Unigene14707\_JingChaoZuZhi2, Unigene14749\_JingChaoZuZhi2, Unigene14817\_JingChaoZuZhi2, Unigene14842\_JingChaoZuZhi2, Unigene14973\_JingChaoZuZhi2, Unigene14990\_JingChaoZuZhi2, Unigene1504\_JingChaoZuZhi2, Unigene15095\_JingChaoZuZhi2, Unigene15104\_JingChaoZuZhi2, Unigene15425\_JingChaoZuZhi2, Unigene15557\_JingChaoZuZhi2, Unigene15692\_JingChaoZuZhi2, Unigene15737\_JingChaoZuZhi2, Unigene15765\_JingChaoZuZhi2, Unigene15777\_JingChaoZuZhi2, Unigene15926\_JingChaoZuZhi2, Unigene15942\_JingChaoZuZhi2, Unigene16039\_JingChaoZuZhi2, Unigene1609\_JingChaoZuZhi2, Unigene16116\_JingChaoZuZhi2, Unigene16154\_JingChaoZuZhi2, Unigene16339\_JingChaoZuZhi2, Unigene16417\_JingChaoZuZhi2, Unigene16480\_JingChaoZuZhi2, Unigene1648\_JingChaoZuZhi2, Unigene16521\_JingChaoZuZhi2, Unigene16589\_JingChaoZuZhi2, Unigene16933\_JingChaoZuZhi2, Unigene17132\_JingChaoZuZhi2, Unigene17136\_JingChaoZuZhi2, Unigene17168\_JingChaoZuZhi2, Unigene17397\_JingChaoZuZhi2, Unigene17506\_JingChaoZuZhi2, Unigene17666\_JingChaoZuZhi2, Unigene17710\_JingChaoZuZhi2, Unigene17727\_JingChaoZuZhi2, Unigene17829\_JingChaoZuZhi2, Unigene17933\_JingChaoZuZhi2, Unigene18184\_JingChaoZuZhi2, Unigene18307\_JingChaoZuZhi2, Unigene18571\_JingChaoZuZhi2, Unigene18577\_JingChaoZuZhi2, Unigene18715\_JingChaoZuZhi2, Unigene18815\_JingChaoZuZhi2, Unigene18885\_JingChaoZuZhi2, Unigene18904\_JingChaoZuZhi2, Unigene18944\_JingChaoZuZhi2, Unigene18956\_JingChaoZuZhi2, Unigene19021\_JingChaoZuZhi2, Unigene19036\_JingChaoZuZhi2, Unigene19131\_JingChaoZuZhi2, Unigene1942\_JingChaoZuZhi2, Unigene19531\_JingChaoZuZhi2, Unigene19624\_JingChaoZuZhi2, Unigene19730\_JingChaoZuZhi2, Unigene19813\_JingChaoZuZhi2, Unigene19854\_JingChaoZuZhi2, Unigene19904\_JingChaoZuZhi2, Unigene1993\_JingChaoZuZhi2, Unigene20009\_JingChaoZuZhi2, Unigene20120\_JingChaoZuZhi2, Unigene20157\_JingChaoZuZhi2, Unigene20236\_JingChaoZuZhi2, Unigene20256\_JingChaoZuZhi2, Unigene20649\_JingChaoZuZhi2, Unigene20875\_JingChaoZuZhi2, Unigene20991\_JingChaoZuZhi2, Unigene21103\_JingChaoZuZhi2, Unigene21144\_JingChaoZuZhi2, Unigene21167\_JingChaoZuZhi2, Unigene21246\_JingChaoZuZhi2, Unigene21348\_JingChaoZuZhi2, Unigene21407\_JingChaoZuZhi2, Unigene21513\_JingChaoZuZhi2, Unigene21518\_JingChaoZuZhi2, Unigene21552\_JingChaoZuZhi2, Unigene21578\_JingChaoZuZhi2, Unigene21594\_JingChaoZuZhi2, Unigene21691\_JingChaoZuZhi2, Unigene21697\_JingChaoZuZhi2, Unigene21737\_JingChaoZuZhi2, Unigene21815\_JingChaoZuZhi2, Unigene21868\_JingChaoZuZhi2, Unigene21973\_JingChaoZuZhi2, Unigene22049\_JingChaoZuZhi2, Unigene22102\_JingChaoZuZhi2, Unigene22174\_JingChaoZuZhi2, Unigene22185\_JingChaoZuZhi2, Unigene22273\_JingChaoZuZhi2, Unigene22386\_JingChaoZuZhi2, Unigene22426\_JingChaoZuZhi2, Unigene22466\_JingChaoZuZhi2, Unigene22482\_JingChaoZuZhi2, Unigene22509\_JingChaoZuZhi2, Unigene22595\_JingChaoZuZhi2, Unigene22604\_JingChaoZuZhi2, Unigene22648\_JingChaoZuZhi2, Unigene22729\_JingChaoZuZhi2, Unigene22766\_JingChaoZuZhi2, Unigene22779\_JingChaoZuZhi2, Unigene22804\_JingChaoZuZhi2, Unigene22874\_JingChaoZuZhi2, Unigene22934\_JingChaoZuZhi2, Unigene2305\_JingChaoZuZhi2, Unigene2308\_JingChaoZuZhi2, Unigene23260\_JingChaoZuZhi2, Unigene24841\_JingChaoZuZhi2, Unigene24881\_JingChaoZuZhi2, Unigene24980\_JingChaoZuZhi2, Unigene25437\_JingChaoZuZhi2, Unigene25966\_JingChaoZuZhi2, Unigene260\_JingChaoZuZhi2, Unigene26183\_JingChaoZuZhi2, Unigene2628\_JingChaoZuZhi2, Unigene26299\_JingChaoZuZhi2, Unigene26679\_JingChaoZuZhi2, Unigene26730\_JingChaoZuZhi2, Unigene2687\_JingChaoZuZhi2, Unigene27090\_JingChaoZuZhi2, Unigene277\_JingChaoZuZhi2, Unigene27859\_JingChaoZuZhi2, Unigene2790\_JingChaoZuZhi2, Unigene28978\_JingChaoZuZhi2, Unigene2899\_JingChaoZuZhi2, Unigene29124\_JingChaoZuZhi2, Unigene29573\_JingChaoZuZhi2, Unigene2960\_JingChaoZuZhi2, Unigene29706\_JingChaoZuZhi2, Unigene2977\_JingChaoZuZhi2, Unigene29968\_JingChaoZuZhi2, Unigene3016\_JingChaoZuZhi2, Unigene30641\_JingChaoZuZhi2, Unigene31255\_JingChaoZuZhi2, Unigene3185\_JingChaoZuZhi2, Unigene31919\_JingChaoZuZhi2, Unigene31982\_JingChaoZuZhi2, Unigene32314\_JingChaoZuZhi2, Unigene34091\_JingChaoZuZhi2, Unigene35196\_JingChaoZuZhi2, Unigene353\_JingChaoZuZhi2, Unigene35706\_JingChaoZuZhi2, Unigene35744\_JingChaoZuZhi2, Unigene35872\_JingChaoZuZhi2, Unigene35952\_JingChaoZuZhi2, Unigene35990\_JingChaoZuZhi2, Unigene36110\_JingChaoZuZhi2, Unigene3640\_JingChaoZuZhi2, Unigene36730\_JingChaoZuZhi2, Unigene36758\_JingChaoZuZhi2, Unigene36999\_JingChaoZuZhi2, Unigene37034\_JingChaoZuZhi2, Unigene38371\_JingChaoZuZhi2, Unigene38493\_JingChaoZuZhi2, Unigene3931\_JingChaoZuZhi2, Unigene39539\_JingChaoZuZhi2, Unigene39577\_JingChaoZuZhi2, Unigene39876\_JingChaoZuZhi2, Unigene40020\_JingChaoZuZhi2, Unigene40143\_JingChaoZuZhi2, Unigene40528\_JingChaoZuZhi2, Unigene40825\_JingChaoZuZhi2, Unigene4166\_JingChaoZuZhi2, Unigene41686\_JingChaoZuZhi2, Unigene4186\_JingChaoZuZhi2, Unigene42329\_JingChaoZuZhi2, Unigene42332\_JingChaoZuZhi2, Unigene42755\_JingChaoZuZhi2, Unigene4294\_JingChaoZuZhi2, Unigene43723\_JingChaoZuZhi2, Unigene43871\_JingChaoZuZhi2, Unigene44228\_JingChaoZuZhi2, Unigene44492\_JingChaoZuZhi2, Unigene44637\_JingChaoZuZhi2, Unigene45532\_JingChaoZuZhi2, Unigene45784\_JingChaoZuZhi2, Unigene45\_JingChaoZuZhi2, Unigene46219\_JingChaoZuZhi2, Unigene46413\_JingChaoZuZhi2, Unigene47199\_JingChaoZuZhi2, Unigene473\_JingChaoZuZhi2, Unigene475\_JingChaoZuZhi2, Unigene48368\_JingChaoZuZhi2, Unigene4838\_JingChaoZuZhi2, Unigene4853\_JingChaoZuZhi2, Unigene48607\_JingChaoZuZhi2, Unigene48962\_JingChaoZuZhi2, Unigene48\_JingChaoZuZhi2, Unigene49526\_JingChaoZuZhi2, Unigene49798\_JingChaoZuZhi2, Unigene50055\_JingChaoZuZhi2, Unigene50713\_JingChaoZuZhi2, Unigene5099\_JingChaoZuZhi2, Unigene51261\_JingChaoZuZhi2, Unigene51705\_JingChaoZuZhi2, Unigene51820\_JingChaoZuZhi2, Unigene51925\_JingChaoZuZhi2, Unigene52281\_JingChaoZuZhi2, Unigene52283\_JingChaoZuZhi2, Unigene52292\_JingChaoZuZhi2, Unigene5253\_JingChaoZuZhi2, Unigene5314\_JingChaoZuZhi2, Unigene53160\_JingChaoZuZhi2, Unigene5347\_JingChaoZuZhi2, Unigene53967\_JingChaoZuZhi2, Unigene54320\_JingChaoZuZhi2, Unigene54363\_JingChaoZuZhi2, Unigene5443\_JingChaoZuZhi2, Unigene54470\_JingChaoZuZhi2, Unigene54904\_JingChaoZuZhi2, Unigene55100\_JingChaoZuZhi2, Unigene56022\_JingChaoZuZhi2, Unigene5621\_JingChaoZuZhi2, Unigene56227\_JingChaoZuZhi2, Unigene56541\_JingChaoZuZhi2, Unigene56559\_JingChaoZuZhi2, Unigene56709\_JingChaoZuZhi2, Unigene57094\_JingChaoZuZhi2, Unigene57869\_JingChaoZuZhi2, Unigene58137\_JingChaoZuZhi2, Unigene58461\_JingChaoZuZhi2, Unigene58790\_JingChaoZuZhi2, Unigene59152\_JingChaoZuZhi2, Unigene59471\_JingChaoZuZhi2, Unigene59845\_JingChaoZuZhi2, Unigene59885\_JingChaoZuZhi2, Unigene601\_JingChaoZuZhi2, Unigene60281\_JingChaoZuZhi2, Unigene6028\_JingChaoZuZhi2, Unigene60762\_JingChaoZuZhi2, Unigene60927\_JingChaoZuZhi2, Unigene61044\_JingChaoZuZhi2, Unigene61108\_JingChaoZuZhi2, Unigene61406\_JingChaoZuZhi2, Unigene61801\_JingChaoZuZhi2, Unigene61925\_JingChaoZuZhi2, Unigene62136\_JingChaoZuZhi2, Unigene62218\_JingChaoZuZhi2, Unigene62786\_JingChaoZuZhi2, Unigene62841\_JingChaoZuZhi2, Unigene63104\_JingChaoZuZhi2, Unigene63226\_JingChaoZuZhi2, Unigene64164\_JingChaoZuZhi2, Unigene6459\_JingChaoZuZhi2, Unigene64640\_JingChaoZuZhi2, Unigene64651\_JingChaoZuZhi2, Unigene64751\_JingChaoZuZhi2, Unigene64830\_JingChaoZuZhi2, Unigene6493\_JingChaoZuZhi2, Unigene65294\_JingChaoZuZhi2, Unigene65488\_JingChaoZuZhi2, Unigene66362\_JingChaoZuZhi2, Unigene6676\_JingChaoZuZhi2, Unigene66802\_JingChaoZuZhi2, Unigene66813\_JingChaoZuZhi2, Unigene67008\_JingChaoZuZhi2, Unigene67320\_JingChaoZuZhi2, Unigene67328\_JingChaoZuZhi2, Unigene6747\_JingChaoZuZhi2, Unigene6752\_JingChaoZuZhi2, Unigene67537\_JingChaoZuZhi2, Unigene67788\_JingChaoZuZhi2, Unigene67793\_JingChaoZuZhi2, Unigene6785\_JingChaoZuZhi2, Unigene68108\_JingChaoZuZhi2, Unigene68151\_JingChaoZuZhi2, Unigene68200\_JingChaoZuZhi2, Unigene68300\_JingChaoZuZhi2, Unigene68553\_JingChaoZuZhi2, Unigene69494\_JingChaoZuZhi2, Unigene69565\_JingChaoZuZhi2, Unigene69757\_JingChaoZuZhi2, Unigene70063\_JingChaoZuZhi2, Unigene70064\_JingChaoZuZhi2, Unigene70087\_JingChaoZuZhi2, Unigene70100\_JingChaoZuZhi2, Unigene70193\_JingChaoZuZhi2, Unigene70505\_JingChaoZuZhi2, Unigene70549\_JingChaoZuZhi2, Unigene70723\_JingChaoZuZhi2, Unigene70957\_JingChaoZuZhi2, Unigene71053\_JingChaoZuZhi2, Unigene71414\_JingChaoZuZhi2, Unigene72003\_JingChaoZuZhi2, Unigene72033\_JingChaoZuZhi2, Unigene72098\_JingChaoZuZhi2, Unigene722\_JingChaoZuZhi2, Unigene72397\_JingChaoZuZhi2, Unigene72521\_JingChaoZuZhi2, Unigene7267\_JingChaoZuZhi2, Unigene73031\_JingChaoZuZhi2, Unigene73203\_JingChaoZuZhi2, Unigene73554\_JingChaoZuZhi2, Unigene73761\_JingChaoZuZhi2, Unigene7417\_JingChaoZuZhi2, Unigene74356\_JingChaoZuZhi2, Unigene74525\_JingChaoZuZhi2, Unigene74594\_JingChaoZuZhi2, Unigene75129\_JingChaoZuZhi2, Unigene75234\_JingChaoZuZhi2, Unigene75317\_JingChaoZuZhi2, Unigene75415\_JingChaoZuZhi2, Unigene75888\_JingChaoZuZhi2, Unigene75899\_JingChaoZuZhi2, Unigene75940\_JingChaoZuZhi2, Unigene76041\_JingChaoZuZhi2, Unigene76099\_JingChaoZuZhi2, Unigene76182\_JingChaoZuZhi2, Unigene76575\_JingChaoZuZhi2, Unigene76763\_JingChaoZuZhi2, Unigene76803\_JingChaoZuZhi2, Unigene77071\_JingChaoZuZhi2, Unigene77433\_JingChaoZuZhi2, Unigene77551\_JingChaoZuZhi2, Unigene77570\_JingChaoZuZhi2, Unigene77817\_JingChaoZuZhi2, Unigene77965\_JingChaoZuZhi2, Unigene78168\_JingChaoZuZhi2, Unigene78235\_JingChaoZuZhi2, Unigene78400\_JingChaoZuZhi2, Unigene78574\_JingChaoZuZhi2, Unigene78705\_JingChaoZuZhi2, Unigene78709\_JingChaoZuZhi2, Unigene7877\_JingChaoZuZhi2, Unigene78957\_JingChaoZuZhi2, Unigene78964\_JingChaoZuZhi2, Unigene79882\_JingChaoZuZhi2, Unigene79900\_JingChaoZuZhi2, Unigene8003\_JingChaoZuZhi2, Unigene80131\_JingChaoZuZhi2, Unigene80143\_JingChaoZuZhi2, Unigene80688\_JingChaoZuZhi2, Unigene80759\_JingChaoZuZhi2, Unigene80863\_JingChaoZuZhi2, Unigene81086\_JingChaoZuZhi2, Unigene81154\_JingChaoZuZhi2, Unigene81431\_JingChaoZuZhi2, Unigene81687\_JingChaoZuZhi2, Unigene81764\_JingChaoZuZhi2, Unigene81901\_JingChaoZuZhi2, Unigene8191\_JingChaoZuZhi2, Unigene81948\_JingChaoZuZhi2, Unigene8196\_JingChaoZuZhi2, Unigene8202\_JingChaoZuZhi2, Unigene82058\_JingChaoZuZhi2, Unigene82073\_JingChaoZuZhi2, Unigene82219\_JingChaoZuZhi2, Unigene82340\_JingChaoZuZhi2, Unigene82367\_JingChaoZuZhi2, Unigene82368\_JingChaoZuZhi2, Unigene8248\_JingChaoZuZhi2, Unigene83166\_JingChaoZuZhi2, Unigene83255\_JingChaoZuZhi2, Unigene83396\_JingChaoZuZhi2, Unigene83400\_JingChaoZuZhi2, Unigene83524\_JingChaoZuZhi2, Unigene83700\_JingChaoZuZhi2, Unigene83983\_JingChaoZuZhi2, Unigene84903\_JingChaoZuZhi2, Unigene84920\_JingChaoZuZhi2, Unigene84930\_JingChaoZuZhi2, Unigene84969\_JingChaoZuZhi2, Unigene85448\_JingChaoZuZhi2, Unigene85510\_JingChaoZuZhi2, Unigene85574\_JingChaoZuZhi2, Unigene8563\_JingChaoZuZhi2, Unigene85658\_JingChaoZuZhi2, Unigene85863\_JingChaoZuZhi2, Unigene8600\_JingChaoZuZhi2, Unigene86051\_JingChaoZuZhi2, Unigene86104\_JingChaoZuZhi2, Unigene86274\_JingChaoZuZhi2, Unigene86312\_JingChaoZuZhi2, Unigene86518\_JingChaoZuZhi2, Unigene86616\_JingChaoZuZhi2, Unigene86760\_JingChaoZuZhi2, Unigene8711\_JingChaoZuZhi2, Unigene87269\_JingChaoZuZhi2, Unigene87302\_JingChaoZuZhi2, Unigene87332\_JingChaoZuZhi2, Unigene876\_JingChaoZuZhi2, Unigene87730\_JingChaoZuZhi2, Unigene87825\_JingChaoZuZhi2, Unigene87851\_JingChaoZuZhi2, Unigene88068\_JingChaoZuZhi2, Unigene88586\_JingChaoZuZhi2, Unigene8860\_JingChaoZuZhi2, Unigene88798\_JingChaoZuZhi2, Unigene88850\_JingChaoZuZhi2, Unigene88942\_JingChaoZuZhi2, Unigene89299\_JingChaoZuZhi2, Unigene8949\_JingChaoZuZhi2, Unigene89544\_JingChaoZuZhi2, Unigene89714\_JingChaoZuZhi2, Unigene89720\_JingChaoZuZhi2, Unigene90051\_JingChaoZuZhi2, Unigene9005\_JingChaoZuZhi2, Unigene90163\_JingChaoZuZhi2, Unigene90439\_JingChaoZuZhi2, Unigene90588\_JingChaoZuZhi2, Unigene90650\_JingChaoZuZhi2, Unigene90762\_JingChaoZuZhi2, Unigene90883\_JingChaoZuZhi2, Unigene91179\_JingChaoZuZhi2, Unigene91380\_JingChaoZuZhi2, Unigene9141\_JingChaoZuZhi2, Unigene91427\_JingChaoZuZhi2, Unigene91527\_JingChaoZuZhi2, Unigene91612\_JingChaoZuZhi2, Unigene91628\_JingChaoZuZhi2, Unigene91631\_JingChaoZuZhi2, Unigene91751\_JingChaoZuZhi2, Unigene91944\_JingChaoZuZhi2, Unigene91958\_JingChaoZuZhi2, Unigene92119\_JingChaoZuZhi2, Unigene92295\_JingChaoZuZhi2, Unigene92386\_JingChaoZuZhi2, Unigene92515\_JingChaoZuZhi2, Unigene92608\_JingChaoZuZhi2, Unigene92676\_JingChaoZuZhi2, Unigene92737\_JingChaoZuZhi2, Unigene92800\_JingChaoZuZhi2, Unigene92862\_JingChaoZuZhi2, Unigene93066\_JingChaoZuZhi2, Unigene93284\_JingChaoZuZhi2, Unigene93597\_JingChaoZuZhi2, Unigene93630\_JingChaoZuZhi2, Unigene93703\_JingChaoZuZhi2, Unigene93782\_JingChaoZuZhi2, Unigene93820\_JingChaoZuZhi2, Unigene93912\_JingChaoZuZhi2, Unigene94149\_JingChaoZuZhi2, Unigene94156\_JingChaoZuZhi2, Unigene94167\_JingChaoZuZhi2, Unigene94208\_JingChaoZuZhi2, Unigene94236\_JingChaoZuZhi2, Unigene94362\_JingChaoZuZhi2, Unigene94432\_JingChaoZuZhi2, Unigene94434\_JingChaoZuZhi2, Unigene94456\_JingChaoZuZhi2, Unigene9445\_JingChaoZuZhi2, Unigene94533\_JingChaoZuZhi2, Unigene94544\_JingChaoZuZhi2, Unigene94546\_JingChaoZuZhi2, Unigene94619\_JingChaoZuZhi2, Unigene94650\_JingChaoZuZhi2, Unigene94721\_JingChaoZuZhi2, Unigene94749\_JingChaoZuZhi2, Unigene9488\_JingChaoZuZhi2, Unigene94940\_JingChaoZuZhi2, Unigene95147\_JingChaoZuZhi2, Unigene9517\_JingChaoZuZhi2, Unigene95213\_JingChaoZuZhi2, Unigene95427\_JingChaoZuZhi2, Unigene95456\_JingChaoZuZhi2, Unigene95483\_JingChaoZuZhi2, Unigene95607\_JingChaoZuZhi2, Unigene95677\_JingChaoZuZhi2, Unigene95700\_JingChaoZuZhi2, Unigene95723\_JingChaoZuZhi2, Unigene95875\_JingChaoZuZhi2, Unigene95897\_JingChaoZuZhi2, Unigene95969\_JingChaoZuZhi2, Unigene95984\_JingChaoZuZhi2, Unigene96000\_JingChaoZuZhi2, Unigene96138\_JingChaoZuZhi2, Unigene96180\_JingChaoZuZhi2, Unigene96202\_JingChaoZuZhi2, Unigene96286\_JingChaoZuZhi2, Unigene9645\_JingChaoZuZhi2, Unigene9711\_JingChaoZuZhi2, Unigene9902\_JingChaoZuZhi2, Unigene9\_JingChaoZuZhi2 |
| 18 | Purine metabolism | Unigene10005\_JingChaoZuZhi2, Unigene10045\_JingChaoZuZhi2, Unigene10129\_JingChaoZuZhi2, Unigene10340\_JingChaoZuZhi2, Unigene1037\_JingChaoZuZhi2, Unigene103\_JingChaoZuZhi2, Unigene10612\_JingChaoZuZhi2, Unigene10769\_JingChaoZuZhi2, Unigene10802\_JingChaoZuZhi2, Unigene10883\_JingChaoZuZhi2, Unigene10888\_JingChaoZuZhi2, Unigene10939\_JingChaoZuZhi2, Unigene10976\_JingChaoZuZhi2, Unigene10979\_JingChaoZuZhi2, Unigene11134\_JingChaoZuZhi2, Unigene11381\_JingChaoZuZhi2, Unigene11496\_JingChaoZuZhi2, Unigene11536\_JingChaoZuZhi2, Unigene1154\_JingChaoZuZhi2, Unigene11564\_JingChaoZuZhi2, Unigene11580\_JingChaoZuZhi2, Unigene1160\_JingChaoZuZhi2, Unigene1164\_JingChaoZuZhi2, Unigene11700\_JingChaoZuZhi2, Unigene11726\_JingChaoZuZhi2, Unigene12098\_JingChaoZuZhi2, Unigene12166\_JingChaoZuZhi2, Unigene1229\_JingChaoZuZhi2, Unigene12617\_JingChaoZuZhi2, Unigene12675\_JingChaoZuZhi2, Unigene12758\_JingChaoZuZhi2, Unigene12931\_JingChaoZuZhi2, Unigene12938\_JingChaoZuZhi2, Unigene12966\_JingChaoZuZhi2, Unigene1313\_JingChaoZuZhi2, Unigene1316\_JingChaoZuZhi2, Unigene13176\_JingChaoZuZhi2, Unigene13181\_JingChaoZuZhi2, Unigene1323\_JingChaoZuZhi2, Unigene13298\_JingChaoZuZhi2, Unigene13419\_JingChaoZuZhi2, Unigene13566\_JingChaoZuZhi2, Unigene13795\_JingChaoZuZhi2, Unigene13837\_JingChaoZuZhi2, Unigene13878\_JingChaoZuZhi2, Unigene13988\_JingChaoZuZhi2, Unigene14135\_JingChaoZuZhi2, Unigene14203\_JingChaoZuZhi2, Unigene14446\_JingChaoZuZhi2, Unigene14551\_JingChaoZuZhi2, Unigene14567\_JingChaoZuZhi2, Unigene14631\_JingChaoZuZhi2, Unigene1468\_JingChaoZuZhi2, Unigene14878\_JingChaoZuZhi2, Unigene15002\_JingChaoZuZhi2, Unigene15276\_JingChaoZuZhi2, Unigene15288\_JingChaoZuZhi2, Unigene15290\_JingChaoZuZhi2, Unigene15390\_JingChaoZuZhi2, Unigene15406\_JingChaoZuZhi2, Unigene15418\_JingChaoZuZhi2, Unigene15539\_JingChaoZuZhi2, Unigene1561\_JingChaoZuZhi2, Unigene15770\_JingChaoZuZhi2, Unigene15927\_JingChaoZuZhi2, Unigene16232\_JingChaoZuZhi2, Unigene16233\_JingChaoZuZhi2, Unigene1628\_JingChaoZuZhi2, Unigene16295\_JingChaoZuZhi2, Unigene16603\_JingChaoZuZhi2, Unigene16786\_JingChaoZuZhi2, Unigene16996\_JingChaoZuZhi2, Unigene17035\_JingChaoZuZhi2, Unigene17065\_JingChaoZuZhi2, Unigene17221\_JingChaoZuZhi2, Unigene1738\_JingChaoZuZhi2, Unigene1744\_JingChaoZuZhi2, Unigene17636\_JingChaoZuZhi2, Unigene17780\_JingChaoZuZhi2, Unigene17861\_JingChaoZuZhi2, Unigene18000\_JingChaoZuZhi2, Unigene18017\_JingChaoZuZhi2, Unigene18099\_JingChaoZuZhi2, Unigene18351\_JingChaoZuZhi2, Unigene18461\_JingChaoZuZhi2, Unigene18508\_JingChaoZuZhi2, Unigene18537\_JingChaoZuZhi2, Unigene18874\_JingChaoZuZhi2, Unigene19029\_JingChaoZuZhi2, Unigene19126\_JingChaoZuZhi2, Unigene19181\_JingChaoZuZhi2, Unigene19280\_JingChaoZuZhi2, Unigene19475\_JingChaoZuZhi2, Unigene19476\_JingChaoZuZhi2, Unigene19497\_JingChaoZuZhi2, Unigene19510\_JingChaoZuZhi2, Unigene19643\_JingChaoZuZhi2, Unigene19645\_JingChaoZuZhi2, Unigene19647\_JingChaoZuZhi2, Unigene19667\_JingChaoZuZhi2, Unigene19838\_JingChaoZuZhi2, Unigene19893\_JingChaoZuZhi2, Unigene19975\_JingChaoZuZhi2, Unigene19978\_JingChaoZuZhi2, Unigene19979\_JingChaoZuZhi2, Unigene20050\_JingChaoZuZhi2, Unigene20087\_JingChaoZuZhi2, Unigene20117\_JingChaoZuZhi2, Unigene2012\_JingChaoZuZhi2, Unigene20150\_JingChaoZuZhi2, Unigene20172\_JingChaoZuZhi2, Unigene20209\_JingChaoZuZhi2, Unigene20219\_JingChaoZuZhi2, Unigene20223\_JingChaoZuZhi2, Unigene20314\_JingChaoZuZhi2, Unigene20441\_JingChaoZuZhi2, Unigene20452\_JingChaoZuZhi2, Unigene20461\_JingChaoZuZhi2, Unigene2046\_JingChaoZuZhi2, Unigene20597\_JingChaoZuZhi2, Unigene20612\_JingChaoZuZhi2, Unigene20711\_JingChaoZuZhi2, Unigene20814\_JingChaoZuZhi2, Unigene20849\_JingChaoZuZhi2, Unigene21005\_JingChaoZuZhi2, Unigene21054\_JingChaoZuZhi2, Unigene21122\_JingChaoZuZhi2, Unigene21282\_JingChaoZuZhi2, Unigene2155\_JingChaoZuZhi2, Unigene21628\_JingChaoZuZhi2, Unigene21648\_JingChaoZuZhi2, Unigene22065\_JingChaoZuZhi2, Unigene22086\_JingChaoZuZhi2, Unigene22184\_JingChaoZuZhi2, Unigene22310\_JingChaoZuZhi2, Unigene22334\_JingChaoZuZhi2, Unigene22339\_JingChaoZuZhi2, Unigene22360\_JingChaoZuZhi2, Unigene22386\_JingChaoZuZhi2, Unigene22392\_JingChaoZuZhi2, Unigene22451\_JingChaoZuZhi2, Unigene22545\_JingChaoZuZhi2, Unigene2254\_JingChaoZuZhi2, Unigene22824\_JingChaoZuZhi2, Unigene22842\_JingChaoZuZhi2, Unigene22870\_JingChaoZuZhi2, Unigene22908\_JingChaoZuZhi2, Unigene22923\_JingChaoZuZhi2, Unigene2324\_JingChaoZuZhi2, Unigene23371\_JingChaoZuZhi2, Unigene23504\_JingChaoZuZhi2, Unigene2354\_JingChaoZuZhi2, Unigene24951\_JingChaoZuZhi2, Unigene25123\_JingChaoZuZhi2, Unigene25421\_JingChaoZuZhi2, Unigene26274\_JingChaoZuZhi2, Unigene26703\_JingChaoZuZhi2, Unigene2707\_JingChaoZuZhi2, Unigene27995\_JingChaoZuZhi2, Unigene29151\_JingChaoZuZhi2, Unigene2958\_JingChaoZuZhi2, Unigene30402\_JingChaoZuZhi2, Unigene30849\_JingChaoZuZhi2, Unigene31166\_JingChaoZuZhi2, Unigene3122\_JingChaoZuZhi2, Unigene31327\_JingChaoZuZhi2, Unigene31355\_JingChaoZuZhi2, Unigene31402\_JingChaoZuZhi2, Unigene3187\_JingChaoZuZhi2, Unigene322\_JingChaoZuZhi2, Unigene33182\_JingChaoZuZhi2, Unigene33470\_JingChaoZuZhi2, Unigene33523\_JingChaoZuZhi2, Unigene34256\_JingChaoZuZhi2, Unigene34338\_JingChaoZuZhi2, Unigene34397\_JingChaoZuZhi2, Unigene35605\_JingChaoZuZhi2, Unigene35687\_JingChaoZuZhi2, Unigene3591\_JingChaoZuZhi2, Unigene3592\_JingChaoZuZhi2, Unigene3632\_JingChaoZuZhi2, Unigene36522\_JingChaoZuZhi2, Unigene36706\_JingChaoZuZhi2, Unigene3682\_JingChaoZuZhi2, Unigene37224\_JingChaoZuZhi2, Unigene37457\_JingChaoZuZhi2, Unigene37504\_JingChaoZuZhi2, Unigene37516\_JingChaoZuZhi2, Unigene38162\_JingChaoZuZhi2, Unigene38468\_JingChaoZuZhi2, Unigene385\_JingChaoZuZhi2, Unigene3871\_JingChaoZuZhi2, Unigene38886\_JingChaoZuZhi2, Unigene38940\_JingChaoZuZhi2, Unigene3938\_JingChaoZuZhi2, Unigene39624\_JingChaoZuZhi2, Unigene3974\_JingChaoZuZhi2, Unigene40335\_JingChaoZuZhi2, Unigene4066\_JingChaoZuZhi2, Unigene41155\_JingChaoZuZhi2, Unigene41227\_JingChaoZuZhi2, Unigene414\_JingChaoZuZhi2, Unigene42364\_JingChaoZuZhi2, Unigene42414\_JingChaoZuZhi2, Unigene43072\_JingChaoZuZhi2, Unigene43410\_JingChaoZuZhi2, Unigene4346\_JingChaoZuZhi2, Unigene43658\_JingChaoZuZhi2, Unigene4375\_JingChaoZuZhi2, Unigene4377\_JingChaoZuZhi2, Unigene44252\_JingChaoZuZhi2, Unigene4426\_JingChaoZuZhi2, Unigene4449\_JingChaoZuZhi2, Unigene4461\_JingChaoZuZhi2, Unigene44745\_JingChaoZuZhi2, Unigene44797\_JingChaoZuZhi2, Unigene45198\_JingChaoZuZhi2, Unigene460\_JingChaoZuZhi2, Unigene46190\_JingChaoZuZhi2, Unigene46315\_JingChaoZuZhi2, Unigene46319\_JingChaoZuZhi2, Unigene46374\_JingChaoZuZhi2, Unigene46843\_JingChaoZuZhi2, Unigene47202\_JingChaoZuZhi2, Unigene4767\_JingChaoZuZhi2, Unigene47794\_JingChaoZuZhi2, Unigene47824\_JingChaoZuZhi2, Unigene4803\_JingChaoZuZhi2, Unigene4816\_JingChaoZuZhi2, Unigene4818\_JingChaoZuZhi2, Unigene48339\_JingChaoZuZhi2, Unigene48499\_JingChaoZuZhi2, Unigene486\_JingChaoZuZhi2, Unigene48\_JingChaoZuZhi2, Unigene4900\_JingChaoZuZhi2, Unigene49060\_JingChaoZuZhi2, Unigene49203\_JingChaoZuZhi2, Unigene49770\_JingChaoZuZhi2, Unigene49985\_JingChaoZuZhi2, Unigene50799\_JingChaoZuZhi2, Unigene50927\_JingChaoZuZhi2, Unigene51125\_JingChaoZuZhi2, Unigene51540\_JingChaoZuZhi2, Unigene51835\_JingChaoZuZhi2, Unigene51991\_JingChaoZuZhi2, Unigene5200\_JingChaoZuZhi2, Unigene5254\_JingChaoZuZhi2, Unigene52751\_JingChaoZuZhi2, Unigene53510\_JingChaoZuZhi2, Unigene53809\_JingChaoZuZhi2, Unigene53935\_JingChaoZuZhi2, Unigene54012\_JingChaoZuZhi2, Unigene542\_JingChaoZuZhi2, Unigene5449\_JingChaoZuZhi2, Unigene5457\_JingChaoZuZhi2, Unigene54768\_JingChaoZuZhi2, Unigene5484\_JingChaoZuZhi2, Unigene54875\_JingChaoZuZhi2, Unigene5552\_JingChaoZuZhi2, Unigene5653\_JingChaoZuZhi2, Unigene56889\_JingChaoZuZhi2, Unigene57185\_JingChaoZuZhi2, Unigene57726\_JingChaoZuZhi2, Unigene57931\_JingChaoZuZhi2, Unigene5897\_JingChaoZuZhi2, Unigene5900\_JingChaoZuZhi2, Unigene59102\_JingChaoZuZhi2, Unigene59414\_JingChaoZuZhi2, Unigene59502\_JingChaoZuZhi2, Unigene60108\_JingChaoZuZhi2, Unigene6011\_JingChaoZuZhi2, Unigene60457\_JingChaoZuZhi2, Unigene60927\_JingChaoZuZhi2, Unigene6134\_JingChaoZuZhi2, Unigene61651\_JingChaoZuZhi2, Unigene61659\_JingChaoZuZhi2, Unigene61679\_JingChaoZuZhi2, Unigene61\_JingChaoZuZhi2, Unigene6219\_JingChaoZuZhi2, Unigene6233\_JingChaoZuZhi2, Unigene62546\_JingChaoZuZhi2, Unigene62552\_JingChaoZuZhi2, Unigene630\_JingChaoZuZhi2, Unigene63112\_JingChaoZuZhi2, Unigene63170\_JingChaoZuZhi2, Unigene6331\_JingChaoZuZhi2, Unigene63374\_JingChaoZuZhi2, Unigene63375\_JingChaoZuZhi2, Unigene63458\_JingChaoZuZhi2, Unigene63485\_JingChaoZuZhi2, Unigene6393\_JingChaoZuZhi2, Unigene64090\_JingChaoZuZhi2, Unigene64315\_JingChaoZuZhi2, Unigene64903\_JingChaoZuZhi2, Unigene65360\_JingChaoZuZhi2, Unigene65405\_JingChaoZuZhi2, Unigene65475\_JingChaoZuZhi2, Unigene65515\_JingChaoZuZhi2, Unigene65800\_JingChaoZuZhi2, Unigene65835\_JingChaoZuZhi2, Unigene66073\_JingChaoZuZhi2, Unigene66146\_JingChaoZuZhi2, Unigene66368\_JingChaoZuZhi2, Unigene66475\_JingChaoZuZhi2, Unigene66656\_JingChaoZuZhi2, Unigene6685\_JingChaoZuZhi2, Unigene66954\_JingChaoZuZhi2, Unigene67077\_JingChaoZuZhi2, Unigene67318\_JingChaoZuZhi2, Unigene6790\_JingChaoZuZhi2, Unigene67964\_JingChaoZuZhi2, Unigene68129\_JingChaoZuZhi2, Unigene685\_JingChaoZuZhi2, Unigene6955\_JingChaoZuZhi2, Unigene69875\_JingChaoZuZhi2, Unigene69922\_JingChaoZuZhi2, Unigene70244\_JingChaoZuZhi2, Unigene70342\_JingChaoZuZhi2, Unigene70647\_JingChaoZuZhi2, Unigene71153\_JingChaoZuZhi2, Unigene71365\_JingChaoZuZhi2, Unigene71482\_JingChaoZuZhi2, Unigene7178\_JingChaoZuZhi2, Unigene7185\_JingChaoZuZhi2, Unigene72461\_JingChaoZuZhi2, Unigene72474\_JingChaoZuZhi2, Unigene72674\_JingChaoZuZhi2, Unigene72921\_JingChaoZuZhi2, Unigene73283\_JingChaoZuZhi2, Unigene73451\_JingChaoZuZhi2, Unigene74062\_JingChaoZuZhi2, Unigene7456\_JingChaoZuZhi2, Unigene74590\_JingChaoZuZhi2, Unigene74922\_JingChaoZuZhi2, Unigene75121\_JingChaoZuZhi2, Unigene75235\_JingChaoZuZhi2, Unigene75690\_JingChaoZuZhi2, Unigene76109\_JingChaoZuZhi2, Unigene76261\_JingChaoZuZhi2, Unigene76331\_JingChaoZuZhi2, Unigene76369\_JingChaoZuZhi2, Unigene76495\_JingChaoZuZhi2, Unigene76868\_JingChaoZuZhi2, Unigene76908\_JingChaoZuZhi2, Unigene77249\_JingChaoZuZhi2, Unigene77449\_JingChaoZuZhi2, Unigene77559\_JingChaoZuZhi2, Unigene77749\_JingChaoZuZhi2, Unigene77798\_JingChaoZuZhi2, Unigene77844\_JingChaoZuZhi2, Unigene78007\_JingChaoZuZhi2, Unigene78071\_JingChaoZuZhi2, Unigene78198\_JingChaoZuZhi2, Unigene78224\_JingChaoZuZhi2, Unigene78268\_JingChaoZuZhi2, Unigene78549\_JingChaoZuZhi2, Unigene78619\_JingChaoZuZhi2, Unigene78757\_JingChaoZuZhi2, Unigene78784\_JingChaoZuZhi2, Unigene78807\_JingChaoZuZhi2, Unigene78813\_JingChaoZuZhi2, Unigene79036\_JingChaoZuZhi2, Unigene79149\_JingChaoZuZhi2, Unigene79211\_JingChaoZuZhi2, Unigene79258\_JingChaoZuZhi2, Unigene7948\_JingChaoZuZhi2, Unigene7990\_JingChaoZuZhi2, Unigene79999\_JingChaoZuZhi2, Unigene80334\_JingChaoZuZhi2, Unigene81086\_JingChaoZuZhi2, Unigene81297\_JingChaoZuZhi2, Unigene81646\_JingChaoZuZhi2, Unigene81674\_JingChaoZuZhi2, Unigene82023\_JingChaoZuZhi2, Unigene8223\_JingChaoZuZhi2, Unigene8225\_JingChaoZuZhi2, Unigene8249\_JingChaoZuZhi2, Unigene82645\_JingChaoZuZhi2, Unigene8278\_JingChaoZuZhi2, Unigene82835\_JingChaoZuZhi2, Unigene83132\_JingChaoZuZhi2, Unigene83297\_JingChaoZuZhi2, Unigene83626\_JingChaoZuZhi2, Unigene84050\_JingChaoZuZhi2, Unigene84203\_JingChaoZuZhi2, Unigene84436\_JingChaoZuZhi2, Unigene84535\_JingChaoZuZhi2, Unigene84894\_JingChaoZuZhi2, Unigene8506\_JingChaoZuZhi2, Unigene8517\_JingChaoZuZhi2, Unigene85304\_JingChaoZuZhi2, Unigene85366\_JingChaoZuZhi2, Unigene85391\_JingChaoZuZhi2, Unigene85574\_JingChaoZuZhi2, Unigene85996\_JingChaoZuZhi2, Unigene86120\_JingChaoZuZhi2, Unigene86157\_JingChaoZuZhi2, Unigene86210\_JingChaoZuZhi2, Unigene86295\_JingChaoZuZhi2, Unigene86414\_JingChaoZuZhi2, Unigene8641\_JingChaoZuZhi2, Unigene8643\_JingChaoZuZhi2, Unigene86607\_JingChaoZuZhi2, Unigene86708\_JingChaoZuZhi2, Unigene86967\_JingChaoZuZhi2, Unigene86987\_JingChaoZuZhi2, Unigene87018\_JingChaoZuZhi2, Unigene87051\_JingChaoZuZhi2, Unigene87089\_JingChaoZuZhi2, Unigene87092\_JingChaoZuZhi2, Unigene87230\_JingChaoZuZhi2, Unigene87422\_JingChaoZuZhi2, Unigene87480\_JingChaoZuZhi2, Unigene8755\_JingChaoZuZhi2, Unigene87599\_JingChaoZuZhi2, Unigene8776\_JingChaoZuZhi2, Unigene87820\_JingChaoZuZhi2, Unigene87855\_JingChaoZuZhi2, Unigene87\_JingChaoZuZhi2, Unigene88016\_JingChaoZuZhi2, Unigene88157\_JingChaoZuZhi2, Unigene88179\_JingChaoZuZhi2, Unigene88283\_JingChaoZuZhi2, Unigene89279\_JingChaoZuZhi2, Unigene89310\_JingChaoZuZhi2, Unigene89375\_JingChaoZuZhi2, Unigene89464\_JingChaoZuZhi2, Unigene89502\_JingChaoZuZhi2, Unigene89603\_JingChaoZuZhi2, Unigene89662\_JingChaoZuZhi2, Unigene8973\_JingChaoZuZhi2, Unigene89753\_JingChaoZuZhi2, Unigene89769\_JingChaoZuZhi2, Unigene89807\_JingChaoZuZhi2, Unigene89976\_JingChaoZuZhi2, Unigene90008\_JingChaoZuZhi2, Unigene90104\_JingChaoZuZhi2, Unigene90180\_JingChaoZuZhi2, Unigene90228\_JingChaoZuZhi2, Unigene90612\_JingChaoZuZhi2, Unigene90730\_JingChaoZuZhi2, Unigene90832\_JingChaoZuZhi2, Unigene90862\_JingChaoZuZhi2, Unigene90919\_JingChaoZuZhi2, Unigene91020\_JingChaoZuZhi2, Unigene91050\_JingChaoZuZhi2, Unigene91087\_JingChaoZuZhi2, Unigene91122\_JingChaoZuZhi2, Unigene91126\_JingChaoZuZhi2, Unigene91187\_JingChaoZuZhi2, Unigene91194\_JingChaoZuZhi2, Unigene91329\_JingChaoZuZhi2, Unigene91469\_JingChaoZuZhi2, Unigene91513\_JingChaoZuZhi2, Unigene91530\_JingChaoZuZhi2, Unigene91701\_JingChaoZuZhi2, Unigene91777\_JingChaoZuZhi2, Unigene91921\_JingChaoZuZhi2, Unigene91943\_JingChaoZuZhi2, Unigene92159\_JingChaoZuZhi2, Unigene92196\_JingChaoZuZhi2, Unigene92224\_JingChaoZuZhi2, Unigene92240\_JingChaoZuZhi2, Unigene92368\_JingChaoZuZhi2, Unigene92515\_JingChaoZuZhi2, Unigene92607\_JingChaoZuZhi2, Unigene92742\_JingChaoZuZhi2, Unigene92803\_JingChaoZuZhi2, Unigene92815\_JingChaoZuZhi2, Unigene92908\_JingChaoZuZhi2, Unigene92910\_JingChaoZuZhi2, Unigene92989\_JingChaoZuZhi2, Unigene93016\_JingChaoZuZhi2, Unigene93270\_JingChaoZuZhi2, Unigene93428\_JingChaoZuZhi2, Unigene93445\_JingChaoZuZhi2, Unigene93591\_JingChaoZuZhi2, Unigene93904\_JingChaoZuZhi2, Unigene93928\_JingChaoZuZhi2, Unigene93993\_JingChaoZuZhi2, Unigene94118\_JingChaoZuZhi2, Unigene94128\_JingChaoZuZhi2, Unigene94131\_JingChaoZuZhi2, Unigene94364\_JingChaoZuZhi2, Unigene94457\_JingChaoZuZhi2, Unigene94536\_JingChaoZuZhi2, Unigene94568\_JingChaoZuZhi2, Unigene94585\_JingChaoZuZhi2, Unigene94630\_JingChaoZuZhi2, Unigene9466\_JingChaoZuZhi2, Unigene94831\_JingChaoZuZhi2, Unigene94910\_JingChaoZuZhi2, Unigene94946\_JingChaoZuZhi2, Unigene95002\_JingChaoZuZhi2, Unigene95132\_JingChaoZuZhi2, Unigene95179\_JingChaoZuZhi2, Unigene95237\_JingChaoZuZhi2, Unigene95296\_JingChaoZuZhi2, Unigene95309\_JingChaoZuZhi2, Unigene95378\_JingChaoZuZhi2, Unigene95476\_JingChaoZuZhi2, Unigene95509\_JingChaoZuZhi2, Unigene9550\_JingChaoZuZhi2, Unigene95591\_JingChaoZuZhi2, Unigene95618\_JingChaoZuZhi2, Unigene95677\_JingChaoZuZhi2, Unigene9569\_JingChaoZuZhi2, Unigene95845\_JingChaoZuZhi2, Unigene95855\_JingChaoZuZhi2, Unigene95901\_JingChaoZuZhi2, Unigene95929\_JingChaoZuZhi2, Unigene96060\_JingChaoZuZhi2, Unigene96165\_JingChaoZuZhi2, Unigene96171\_JingChaoZuZhi2, Unigene96222\_JingChaoZuZhi2, Unigene96240\_JingChaoZuZhi2, Unigene963\_JingChaoZuZhi2, Unigene9984\_JingChaoZuZhi2 |
| 19 | Protein processing in endoplasmic reticulum | Unigene10361\_JingChaoZuZhi2, Unigene10380\_JingChaoZuZhi2, Unigene10678\_JingChaoZuZhi2, Unigene10734\_JingChaoZuZhi2, Unigene10747\_JingChaoZuZhi2, Unigene10835\_JingChaoZuZhi2, Unigene10928\_JingChaoZuZhi2, Unigene1094\_JingChaoZuZhi2, Unigene11070\_JingChaoZuZhi2, Unigene11097\_JingChaoZuZhi2, Unigene11592\_JingChaoZuZhi2, Unigene11951\_JingChaoZuZhi2, Unigene12003\_JingChaoZuZhi2, Unigene12102\_JingChaoZuZhi2, Unigene12263\_JingChaoZuZhi2, Unigene12509\_JingChaoZuZhi2, Unigene12633\_JingChaoZuZhi2, Unigene12634\_JingChaoZuZhi2, Unigene12787\_JingChaoZuZhi2, Unigene12834\_JingChaoZuZhi2, Unigene13098\_JingChaoZuZhi2, Unigene13103\_JingChaoZuZhi2, Unigene13115\_JingChaoZuZhi2, Unigene13134\_JingChaoZuZhi2, Unigene13435\_JingChaoZuZhi2, Unigene13866\_JingChaoZuZhi2, Unigene13920\_JingChaoZuZhi2, Unigene13970\_JingChaoZuZhi2, Unigene14140\_JingChaoZuZhi2, Unigene14250\_JingChaoZuZhi2, Unigene14336\_JingChaoZuZhi2, Unigene14409\_JingChaoZuZhi2, Unigene14505\_JingChaoZuZhi2, Unigene145\_JingChaoZuZhi2, Unigene14610\_JingChaoZuZhi2, Unigene14910\_JingChaoZuZhi2, Unigene15171\_JingChaoZuZhi2, Unigene15289\_JingChaoZuZhi2, Unigene15563\_JingChaoZuZhi2, Unigene15690\_JingChaoZuZhi2, Unigene1576\_JingChaoZuZhi2, Unigene15840\_JingChaoZuZhi2, Unigene15908\_JingChaoZuZhi2, Unigene16236\_JingChaoZuZhi2, Unigene16261\_JingChaoZuZhi2, Unigene16285\_JingChaoZuZhi2, Unigene16432\_JingChaoZuZhi2, Unigene16460\_JingChaoZuZhi2, Unigene16533\_JingChaoZuZhi2, Unigene16709\_JingChaoZuZhi2, Unigene16834\_JingChaoZuZhi2, Unigene16987\_JingChaoZuZhi2, Unigene17020\_JingChaoZuZhi2, Unigene17214\_JingChaoZuZhi2, Unigene17506\_JingChaoZuZhi2, Unigene17556\_JingChaoZuZhi2, Unigene17615\_JingChaoZuZhi2, Unigene17733\_JingChaoZuZhi2, Unigene17798\_JingChaoZuZhi2, Unigene17930\_JingChaoZuZhi2, Unigene18325\_JingChaoZuZhi2, Unigene18433\_JingChaoZuZhi2, Unigene18577\_JingChaoZuZhi2, Unigene18717\_JingChaoZuZhi2, Unigene18734\_JingChaoZuZhi2, Unigene18935\_JingChaoZuZhi2, Unigene19131\_JingChaoZuZhi2, Unigene1919\_JingChaoZuZhi2, Unigene19213\_JingChaoZuZhi2, Unigene19235\_JingChaoZuZhi2, Unigene19479\_JingChaoZuZhi2, Unigene19549\_JingChaoZuZhi2, Unigene19582\_JingChaoZuZhi2, Unigene1964\_JingChaoZuZhi2, Unigene19821\_JingChaoZuZhi2, Unigene20078\_JingChaoZuZhi2, Unigene20217\_JingChaoZuZhi2, Unigene20225\_JingChaoZuZhi2, Unigene20233\_JingChaoZuZhi2, Unigene20275\_JingChaoZuZhi2, Unigene20464\_JingChaoZuZhi2, Unigene20489\_JingChaoZuZhi2, Unigene20553\_JingChaoZuZhi2, Unigene20611\_JingChaoZuZhi2, Unigene2063\_JingChaoZuZhi2, Unigene20980\_JingChaoZuZhi2, Unigene21028\_JingChaoZuZhi2, Unigene21103\_JingChaoZuZhi2, Unigene21105\_JingChaoZuZhi2, Unigene21226\_JingChaoZuZhi2, Unigene21392\_JingChaoZuZhi2, Unigene21466\_JingChaoZuZhi2, Unigene21552\_JingChaoZuZhi2, Unigene21706\_JingChaoZuZhi2, Unigene21868\_JingChaoZuZhi2, Unigene21940\_JingChaoZuZhi2, Unigene22673\_JingChaoZuZhi2, Unigene22686\_JingChaoZuZhi2, Unigene22777\_JingChaoZuZhi2, Unigene22820\_JingChaoZuZhi2, Unigene22837\_JingChaoZuZhi2, Unigene22842\_JingChaoZuZhi2, Unigene22918\_JingChaoZuZhi2, Unigene2424\_JingChaoZuZhi2, Unigene24264\_JingChaoZuZhi2, Unigene2444\_JingChaoZuZhi2, Unigene24668\_JingChaoZuZhi2, Unigene24905\_JingChaoZuZhi2, Unigene26077\_JingChaoZuZhi2, Unigene2687\_JingChaoZuZhi2, Unigene27530\_JingChaoZuZhi2, Unigene27651\_JingChaoZuZhi2, Unigene28427\_JingChaoZuZhi2, Unigene28571\_JingChaoZuZhi2, Unigene3025\_JingChaoZuZhi2, Unigene3099\_JingChaoZuZhi2, Unigene31068\_JingChaoZuZhi2, Unigene33480\_JingChaoZuZhi2, Unigene33893\_JingChaoZuZhi2, Unigene34365\_JingChaoZuZhi2, Unigene3452\_JingChaoZuZhi2, Unigene3469\_JingChaoZuZhi2, Unigene3481\_JingChaoZuZhi2, Unigene34912\_JingChaoZuZhi2, Unigene35335\_JingChaoZuZhi2, Unigene35730\_JingChaoZuZhi2, Unigene35990\_JingChaoZuZhi2, Unigene3635\_JingChaoZuZhi2, Unigene36751\_JingChaoZuZhi2, Unigene36859\_JingChaoZuZhi2, Unigene37034\_JingChaoZuZhi2, Unigene37070\_JingChaoZuZhi2, Unigene37667\_JingChaoZuZhi2, Unigene37954\_JingChaoZuZhi2, Unigene3813\_JingChaoZuZhi2, Unigene38741\_JingChaoZuZhi2, Unigene38753\_JingChaoZuZhi2, Unigene38873\_JingChaoZuZhi2, Unigene39259\_JingChaoZuZhi2, Unigene39457\_JingChaoZuZhi2, Unigene4001\_JingChaoZuZhi2, Unigene41786\_JingChaoZuZhi2, Unigene41991\_JingChaoZuZhi2, Unigene42128\_JingChaoZuZhi2, Unigene42322\_JingChaoZuZhi2, Unigene42638\_JingChaoZuZhi2, Unigene4294\_JingChaoZuZhi2, Unigene4418\_JingChaoZuZhi2, Unigene44224\_JingChaoZuZhi2, Unigene4426\_JingChaoZuZhi2, Unigene44354\_JingChaoZuZhi2, Unigene44925\_JingChaoZuZhi2, Unigene45702\_JingChaoZuZhi2, Unigene45948\_JingChaoZuZhi2, Unigene46559\_JingChaoZuZhi2, Unigene46567\_JingChaoZuZhi2, Unigene46849\_JingChaoZuZhi2, Unigene47450\_JingChaoZuZhi2, Unigene4747\_JingChaoZuZhi2, Unigene48223\_JingChaoZuZhi2, Unigene48263\_JingChaoZuZhi2, Unigene48347\_JingChaoZuZhi2, Unigene49275\_JingChaoZuZhi2, Unigene50611\_JingChaoZuZhi2, Unigene50625\_JingChaoZuZhi2, Unigene51344\_JingChaoZuZhi2, Unigene51473\_JingChaoZuZhi2, Unigene51627\_JingChaoZuZhi2, Unigene51820\_JingChaoZuZhi2, Unigene52526\_JingChaoZuZhi2, Unigene5255\_JingChaoZuZhi2, Unigene52628\_JingChaoZuZhi2, Unigene52740\_JingChaoZuZhi2, Unigene52778\_JingChaoZuZhi2, Unigene53101\_JingChaoZuZhi2, Unigene53141\_JingChaoZuZhi2, Unigene53834\_JingChaoZuZhi2, Unigene54468\_JingChaoZuZhi2, Unigene54569\_JingChaoZuZhi2, Unigene55100\_JingChaoZuZhi2, Unigene55305\_JingChaoZuZhi2, Unigene55541\_JingChaoZuZhi2, Unigene55725\_JingChaoZuZhi2, Unigene55889\_JingChaoZuZhi2, Unigene56170\_JingChaoZuZhi2, Unigene56662\_JingChaoZuZhi2, Unigene56752\_JingChaoZuZhi2, Unigene56775\_JingChaoZuZhi2, Unigene57035\_JingChaoZuZhi2, Unigene57300\_JingChaoZuZhi2, Unigene57579\_JingChaoZuZhi2, Unigene57719\_JingChaoZuZhi2, Unigene57802\_JingChaoZuZhi2, Unigene57827\_JingChaoZuZhi2, Unigene58073\_JingChaoZuZhi2, Unigene58094\_JingChaoZuZhi2, Unigene58348\_JingChaoZuZhi2, Unigene58433\_JingChaoZuZhi2, Unigene58582\_JingChaoZuZhi2, Unigene5869\_JingChaoZuZhi2, Unigene58742\_JingChaoZuZhi2, Unigene58963\_JingChaoZuZhi2, Unigene59068\_JingChaoZuZhi2, Unigene59246\_JingChaoZuZhi2, Unigene59450\_JingChaoZuZhi2, Unigene59849\_JingChaoZuZhi2, Unigene60260\_JingChaoZuZhi2, Unigene60551\_JingChaoZuZhi2, Unigene60962\_JingChaoZuZhi2, Unigene61181\_JingChaoZuZhi2, Unigene61708\_JingChaoZuZhi2, Unigene61736\_JingChaoZuZhi2, Unigene62272\_JingChaoZuZhi2, Unigene62843\_JingChaoZuZhi2, Unigene63247\_JingChaoZuZhi2, Unigene63273\_JingChaoZuZhi2, Unigene63586\_JingChaoZuZhi2, Unigene6394\_JingChaoZuZhi2, Unigene64016\_JingChaoZuZhi2, Unigene64112\_JingChaoZuZhi2, Unigene64126\_JingChaoZuZhi2, Unigene64431\_JingChaoZuZhi2, Unigene64670\_JingChaoZuZhi2, Unigene64842\_JingChaoZuZhi2, Unigene65911\_JingChaoZuZhi2, Unigene66253\_JingChaoZuZhi2, Unigene66575\_JingChaoZuZhi2, Unigene67177\_JingChaoZuZhi2, Unigene67412\_JingChaoZuZhi2, Unigene67676\_JingChaoZuZhi2, Unigene67\_JingChaoZuZhi2, Unigene68173\_JingChaoZuZhi2, Unigene6828\_JingChaoZuZhi2, Unigene68748\_JingChaoZuZhi2, Unigene69210\_JingChaoZuZhi2, Unigene70020\_JingChaoZuZhi2, Unigene700\_JingChaoZuZhi2, Unigene7015\_JingChaoZuZhi2, Unigene7018\_JingChaoZuZhi2, Unigene70543\_JingChaoZuZhi2, Unigene70622\_JingChaoZuZhi2, Unigene70701\_JingChaoZuZhi2, Unigene70734\_JingChaoZuZhi2, Unigene71152\_JingChaoZuZhi2, Unigene71419\_JingChaoZuZhi2, Unigene71821\_JingChaoZuZhi2, Unigene72036\_JingChaoZuZhi2, Unigene72041\_JingChaoZuZhi2, Unigene72397\_JingChaoZuZhi2, Unigene7253\_JingChaoZuZhi2, Unigene7292\_JingChaoZuZhi2, Unigene73038\_JingChaoZuZhi2, Unigene73068\_JingChaoZuZhi2, Unigene73162\_JingChaoZuZhi2, Unigene73396\_JingChaoZuZhi2, Unigene7362\_JingChaoZuZhi2, Unigene73730\_JingChaoZuZhi2, Unigene73920\_JingChaoZuZhi2, Unigene74028\_JingChaoZuZhi2, Unigene74389\_JingChaoZuZhi2, Unigene74661\_JingChaoZuZhi2, Unigene74919\_JingChaoZuZhi2, Unigene74963\_JingChaoZuZhi2, Unigene75159\_JingChaoZuZhi2, Unigene75353\_JingChaoZuZhi2, Unigene754\_JingChaoZuZhi2, Unigene75724\_JingChaoZuZhi2, Unigene75975\_JingChaoZuZhi2, Unigene76207\_JingChaoZuZhi2, Unigene76392\_JingChaoZuZhi2, Unigene76399\_JingChaoZuZhi2, Unigene76540\_JingChaoZuZhi2, Unigene76639\_JingChaoZuZhi2, Unigene77209\_JingChaoZuZhi2, Unigene77491\_JingChaoZuZhi2, Unigene78004\_JingChaoZuZhi2, Unigene78400\_JingChaoZuZhi2, Unigene78866\_JingChaoZuZhi2, Unigene79493\_JingChaoZuZhi2, Unigene79911\_JingChaoZuZhi2, Unigene80315\_JingChaoZuZhi2, Unigene80347\_JingChaoZuZhi2, Unigene80688\_JingChaoZuZhi2, Unigene80776\_JingChaoZuZhi2, Unigene80809\_JingChaoZuZhi2, Unigene80919\_JingChaoZuZhi2, Unigene80960\_JingChaoZuZhi2, Unigene80984\_JingChaoZuZhi2, Unigene81108\_JingChaoZuZhi2, Unigene81154\_JingChaoZuZhi2, Unigene81192\_JingChaoZuZhi2, Unigene81480\_JingChaoZuZhi2, Unigene81506\_JingChaoZuZhi2, Unigene81610\_JingChaoZuZhi2, Unigene81631\_JingChaoZuZhi2, Unigene81948\_JingChaoZuZhi2, Unigene82058\_JingChaoZuZhi2, Unigene82073\_JingChaoZuZhi2, Unigene82367\_JingChaoZuZhi2, Unigene82393\_JingChaoZuZhi2, Unigene824\_JingChaoZuZhi2, Unigene82629\_JingChaoZuZhi2, Unigene82698\_JingChaoZuZhi2, Unigene82742\_JingChaoZuZhi2, Unigene82904\_JingChaoZuZhi2, Unigene83255\_JingChaoZuZhi2, Unigene83524\_JingChaoZuZhi2, Unigene83788\_JingChaoZuZhi2, Unigene83930\_JingChaoZuZhi2, Unigene83932\_JingChaoZuZhi2, Unigene83933\_JingChaoZuZhi2, Unigene83978\_JingChaoZuZhi2, Unigene8415\_JingChaoZuZhi2, Unigene84227\_JingChaoZuZhi2, Unigene84807\_JingChaoZuZhi2, Unigene84986\_JingChaoZuZhi2, Unigene85038\_JingChaoZuZhi2, Unigene85122\_JingChaoZuZhi2, Unigene85136\_JingChaoZuZhi2, Unigene85154\_JingChaoZuZhi2, Unigene85198\_JingChaoZuZhi2, Unigene85236\_JingChaoZuZhi2, Unigene85323\_JingChaoZuZhi2, Unigene85380\_JingChaoZuZhi2, Unigene85423\_JingChaoZuZhi2, Unigene8563\_JingChaoZuZhi2, Unigene85743\_JingChaoZuZhi2, Unigene85976\_JingChaoZuZhi2, Unigene86101\_JingChaoZuZhi2, Unigene86317\_JingChaoZuZhi2, Unigene86501\_JingChaoZuZhi2, Unigene86734\_JingChaoZuZhi2, Unigene86741\_JingChaoZuZhi2, Unigene86826\_JingChaoZuZhi2, Unigene86975\_JingChaoZuZhi2, Unigene87036\_JingChaoZuZhi2, Unigene87174\_JingChaoZuZhi2, Unigene87222\_JingChaoZuZhi2, Unigene87261\_JingChaoZuZhi2, Unigene87283\_JingChaoZuZhi2, Unigene87995\_JingChaoZuZhi2, Unigene88009\_JingChaoZuZhi2, Unigene88184\_JingChaoZuZhi2, Unigene88412\_JingChaoZuZhi2, Unigene88519\_JingChaoZuZhi2, Unigene8853\_JingChaoZuZhi2, Unigene88703\_JingChaoZuZhi2, Unigene88720\_JingChaoZuZhi2, Unigene88927\_JingChaoZuZhi2, Unigene89014\_JingChaoZuZhi2, Unigene89137\_JingChaoZuZhi2, Unigene89226\_JingChaoZuZhi2, Unigene89229\_JingChaoZuZhi2, Unigene89354\_JingChaoZuZhi2, Unigene89600\_JingChaoZuZhi2, Unigene89665\_JingChaoZuZhi2, Unigene89774\_JingChaoZuZhi2, Unigene89779\_JingChaoZuZhi2, Unigene89781\_JingChaoZuZhi2, Unigene89839\_JingChaoZuZhi2, Unigene8996\_JingChaoZuZhi2, Unigene90040\_JingChaoZuZhi2, Unigene90184\_JingChaoZuZhi2, Unigene90261\_JingChaoZuZhi2, Unigene90347\_JingChaoZuZhi2, Unigene90429\_JingChaoZuZhi2, Unigene90599\_JingChaoZuZhi2, Unigene90645\_JingChaoZuZhi2, Unigene90727\_JingChaoZuZhi2, Unigene91201\_JingChaoZuZhi2, Unigene91418\_JingChaoZuZhi2, Unigene91427\_JingChaoZuZhi2, Unigene91499\_JingChaoZuZhi2, Unigene91584\_JingChaoZuZhi2, Unigene91628\_JingChaoZuZhi2, Unigene91798\_JingChaoZuZhi2, Unigene91823\_JingChaoZuZhi2, Unigene91944\_JingChaoZuZhi2, Unigene92063\_JingChaoZuZhi2, Unigene92068\_JingChaoZuZhi2, Unigene92131\_JingChaoZuZhi2, Unigene92137\_JingChaoZuZhi2, Unigene92464\_JingChaoZuZhi2, Unigene92511\_JingChaoZuZhi2, Unigene92631\_JingChaoZuZhi2, Unigene92634\_JingChaoZuZhi2, Unigene9266\_JingChaoZuZhi2, Unigene92779\_JingChaoZuZhi2, Unigene92954\_JingChaoZuZhi2, Unigene93086\_JingChaoZuZhi2, Unigene93120\_JingChaoZuZhi2, Unigene93217\_JingChaoZuZhi2, Unigene93241\_JingChaoZuZhi2, Unigene93288\_JingChaoZuZhi2, Unigene93305\_JingChaoZuZhi2, Unigene93416\_JingChaoZuZhi2, Unigene93451\_JingChaoZuZhi2, Unigene93597\_JingChaoZuZhi2, Unigene93605\_JingChaoZuZhi2, Unigene93619\_JingChaoZuZhi2, Unigene9362\_JingChaoZuZhi2, Unigene93692\_JingChaoZuZhi2, Unigene93715\_JingChaoZuZhi2, Unigene93728\_JingChaoZuZhi2, Unigene93838\_JingChaoZuZhi2, Unigene94023\_JingChaoZuZhi2, Unigene94039\_JingChaoZuZhi2, Unigene94239\_JingChaoZuZhi2, Unigene94307\_JingChaoZuZhi2, Unigene94362\_JingChaoZuZhi2, Unigene94416\_JingChaoZuZhi2, Unigene94450\_JingChaoZuZhi2, Unigene94512\_JingChaoZuZhi2, Unigene94533\_JingChaoZuZhi2, Unigene94570\_JingChaoZuZhi2, Unigene94601\_JingChaoZuZhi2, Unigene94610\_JingChaoZuZhi2, Unigene94619\_JingChaoZuZhi2, Unigene94636\_JingChaoZuZhi2, Unigene94721\_JingChaoZuZhi2, Unigene94738\_JingChaoZuZhi2, Unigene94761\_JingChaoZuZhi2, Unigene94764\_JingChaoZuZhi2, Unigene94817\_JingChaoZuZhi2, Unigene94857\_JingChaoZuZhi2, Unigene94879\_JingChaoZuZhi2, Unigene94889\_JingChaoZuZhi2, Unigene9488\_JingChaoZuZhi2, Unigene95001\_JingChaoZuZhi2, Unigene95045\_JingChaoZuZhi2, Unigene95052\_JingChaoZuZhi2, Unigene95056\_JingChaoZuZhi2, Unigene95109\_JingChaoZuZhi2, Unigene95112\_JingChaoZuZhi2, Unigene95185\_JingChaoZuZhi2, Unigene95256\_JingChaoZuZhi2, Unigene95385\_JingChaoZuZhi2, Unigene95398\_JingChaoZuZhi2, Unigene95427\_JingChaoZuZhi2, Unigene95433\_JingChaoZuZhi2, Unigene95449\_JingChaoZuZhi2, Unigene95455\_JingChaoZuZhi2, Unigene95469\_JingChaoZuZhi2, Unigene95483\_JingChaoZuZhi2, Unigene95496\_JingChaoZuZhi2, Unigene95502\_JingChaoZuZhi2, Unigene95608\_JingChaoZuZhi2, Unigene95679\_JingChaoZuZhi2, Unigene95700\_JingChaoZuZhi2, Unigene95705\_JingChaoZuZhi2, Unigene95760\_JingChaoZuZhi2, Unigene95783\_JingChaoZuZhi2, Unigene95806\_JingChaoZuZhi2, Unigene95815\_JingChaoZuZhi2, Unigene95859\_JingChaoZuZhi2, Unigene95897\_JingChaoZuZhi2, Unigene95902\_JingChaoZuZhi2, Unigene95913\_JingChaoZuZhi2, Unigene95972\_JingChaoZuZhi2, Unigene95984\_JingChaoZuZhi2, Unigene95996\_JingChaoZuZhi2, Unigene96004\_JingChaoZuZhi2, Unigene96011\_JingChaoZuZhi2, Unigene96013\_JingChaoZuZhi2, Unigene96014\_JingChaoZuZhi2, Unigene96029\_JingChaoZuZhi2, Unigene96032\_JingChaoZuZhi2, Unigene96048\_JingChaoZuZhi2, Unigene96067\_JingChaoZuZhi2, Unigene96083\_JingChaoZuZhi2, Unigene96130\_JingChaoZuZhi2, Unigene96178\_JingChaoZuZhi2, Unigene96179\_JingChaoZuZhi2, Unigene96180\_JingChaoZuZhi2, Unigene96186\_JingChaoZuZhi2, Unigene96192\_JingChaoZuZhi2, Unigene96252\_JingChaoZuZhi2, Unigene96264\_JingChaoZuZhi2, Unigene96295\_JingChaoZuZhi2, Unigene9665\_JingChaoZuZhi2, Unigene9676\_JingChaoZuZhi2, Unigene9988\_JingChaoZuZhi2 |
| 20 | Endocytosis | Unigene10380\_JingChaoZuZhi2, Unigene10422\_JingChaoZuZhi2, Unigene10932\_JingChaoZuZhi2, Unigene11000\_JingChaoZuZhi2, Unigene11111\_JingChaoZuZhi2, Unigene11420\_JingChaoZuZhi2, Unigene11557\_JingChaoZuZhi2, Unigene12075\_JingChaoZuZhi2, Unigene12133\_JingChaoZuZhi2, Unigene12221\_JingChaoZuZhi2, Unigene12253\_JingChaoZuZhi2, Unigene12375\_JingChaoZuZhi2, Unigene12425\_JingChaoZuZhi2, Unigene12577\_JingChaoZuZhi2, Unigene12728\_JingChaoZuZhi2, Unigene12791\_JingChaoZuZhi2, Unigene1281\_JingChaoZuZhi2, Unigene12832\_JingChaoZuZhi2, Unigene12935\_JingChaoZuZhi2, Unigene12954\_JingChaoZuZhi2, Unigene12958\_JingChaoZuZhi2, Unigene13115\_JingChaoZuZhi2, Unigene13149\_JingChaoZuZhi2, Unigene13454\_JingChaoZuZhi2, Unigene13509\_JingChaoZuZhi2, Unigene13919\_JingChaoZuZhi2, Unigene13946\_JingChaoZuZhi2, Unigene14068\_JingChaoZuZhi2, Unigene14074\_JingChaoZuZhi2, Unigene14225\_JingChaoZuZhi2, Unigene14250\_JingChaoZuZhi2, Unigene14361\_JingChaoZuZhi2, Unigene14409\_JingChaoZuZhi2, Unigene14413\_JingChaoZuZhi2, Unigene14523\_JingChaoZuZhi2, Unigene14534\_JingChaoZuZhi2, Unigene14635\_JingChaoZuZhi2, Unigene14839\_JingChaoZuZhi2, Unigene14882\_JingChaoZuZhi2, Unigene14998\_JingChaoZuZhi2, Unigene15151\_JingChaoZuZhi2, Unigene15216\_JingChaoZuZhi2, Unigene15240\_JingChaoZuZhi2, Unigene15336\_JingChaoZuZhi2, Unigene15346\_JingChaoZuZhi2, Unigene15413\_JingChaoZuZhi2, Unigene15751\_JingChaoZuZhi2, Unigene15823\_JingChaoZuZhi2, Unigene1600\_JingChaoZuZhi2, Unigene16027\_JingChaoZuZhi2, Unigene16028\_JingChaoZuZhi2, Unigene16071\_JingChaoZuZhi2, Unigene16134\_JingChaoZuZhi2, Unigene16339\_JingChaoZuZhi2, Unigene16427\_JingChaoZuZhi2, Unigene16530\_JingChaoZuZhi2, Unigene16597\_JingChaoZuZhi2, Unigene167\_JingChaoZuZhi2, Unigene16885\_JingChaoZuZhi2, Unigene16898\_JingChaoZuZhi2, Unigene17087\_JingChaoZuZhi2, Unigene17166\_JingChaoZuZhi2, Unigene17474\_JingChaoZuZhi2, Unigene17545\_JingChaoZuZhi2, Unigene17594\_JingChaoZuZhi2, Unigene17764\_JingChaoZuZhi2, Unigene17888\_JingChaoZuZhi2, Unigene1813\_JingChaoZuZhi2, Unigene18353\_JingChaoZuZhi2, Unigene1838\_JingChaoZuZhi2, Unigene18458\_JingChaoZuZhi2, Unigene18462\_JingChaoZuZhi2, Unigene18577\_JingChaoZuZhi2, Unigene18608\_JingChaoZuZhi2, Unigene18642\_JingChaoZuZhi2, Unigene1887\_JingChaoZuZhi2, Unigene18894\_JingChaoZuZhi2, Unigene18986\_JingChaoZuZhi2, Unigene19131\_JingChaoZuZhi2, Unigene19405\_JingChaoZuZhi2, Unigene19579\_JingChaoZuZhi2, Unigene19664\_JingChaoZuZhi2, Unigene19691\_JingChaoZuZhi2, Unigene19701\_JingChaoZuZhi2, Unigene19813\_JingChaoZuZhi2, Unigene20029\_JingChaoZuZhi2, Unigene20030\_JingChaoZuZhi2, Unigene20305\_JingChaoZuZhi2, Unigene20382\_JingChaoZuZhi2, Unigene20420\_JingChaoZuZhi2, Unigene20480\_JingChaoZuZhi2, Unigene204\_JingChaoZuZhi2, Unigene20697\_JingChaoZuZhi2, Unigene20755\_JingChaoZuZhi2, Unigene20776\_JingChaoZuZhi2, Unigene20846\_JingChaoZuZhi2, Unigene2084\_JingChaoZuZhi2, Unigene20918\_JingChaoZuZhi2, Unigene2092\_JingChaoZuZhi2, Unigene21095\_JingChaoZuZhi2, Unigene21100\_JingChaoZuZhi2, Unigene21103\_JingChaoZuZhi2, Unigene21135\_JingChaoZuZhi2, Unigene21215\_JingChaoZuZhi2, Unigene21355\_JingChaoZuZhi2, Unigene21385\_JingChaoZuZhi2, Unigene21419\_JingChaoZuZhi2, Unigene21451\_JingChaoZuZhi2, Unigene21484\_JingChaoZuZhi2, Unigene21489\_JingChaoZuZhi2, Unigene21552\_JingChaoZuZhi2, Unigene21605\_JingChaoZuZhi2, Unigene21629\_JingChaoZuZhi2, Unigene21664\_JingChaoZuZhi2, Unigene21868\_JingChaoZuZhi2, Unigene21906\_JingChaoZuZhi2, Unigene21911\_JingChaoZuZhi2, Unigene21925\_JingChaoZuZhi2, Unigene21992\_JingChaoZuZhi2, Unigene22034\_JingChaoZuZhi2, Unigene22236\_JingChaoZuZhi2, Unigene22400\_JingChaoZuZhi2, Unigene22488\_JingChaoZuZhi2, Unigene22500\_JingChaoZuZhi2, Unigene22536\_JingChaoZuZhi2, Unigene22542\_JingChaoZuZhi2, Unigene22639\_JingChaoZuZhi2, Unigene22640\_JingChaoZuZhi2, Unigene22669\_JingChaoZuZhi2, Unigene22676\_JingChaoZuZhi2, Unigene22740\_JingChaoZuZhi2, Unigene2275\_JingChaoZuZhi2, Unigene22782\_JingChaoZuZhi2, Unigene22783\_JingChaoZuZhi2, Unigene22911\_JingChaoZuZhi2, Unigene22916\_JingChaoZuZhi2, Unigene2360\_JingChaoZuZhi2, Unigene23744\_JingChaoZuZhi2, Unigene24117\_JingChaoZuZhi2, Unigene24650\_JingChaoZuZhi2, Unigene25116\_JingChaoZuZhi2, Unigene2531\_JingChaoZuZhi2, Unigene25\_JingChaoZuZhi2, Unigene2686\_JingChaoZuZhi2, Unigene27145\_JingChaoZuZhi2, Unigene2790\_JingChaoZuZhi2, Unigene28263\_JingChaoZuZhi2, Unigene2932\_JingChaoZuZhi2, Unigene29777\_JingChaoZuZhi2, Unigene30128\_JingChaoZuZhi2, Unigene31924\_JingChaoZuZhi2, Unigene32666\_JingChaoZuZhi2, Unigene3391\_JingChaoZuZhi2, Unigene35027\_JingChaoZuZhi2, Unigene35102\_JingChaoZuZhi2, Unigene35990\_JingChaoZuZhi2, Unigene36224\_JingChaoZuZhi2, Unigene36259\_JingChaoZuZhi2, Unigene3640\_JingChaoZuZhi2, Unigene3700\_JingChaoZuZhi2, Unigene37014\_JingChaoZuZhi2, Unigene37741\_JingChaoZuZhi2, Unigene37803\_JingChaoZuZhi2, Unigene39003\_JingChaoZuZhi2, Unigene39785\_JingChaoZuZhi2, Unigene39865\_JingChaoZuZhi2, Unigene39876\_JingChaoZuZhi2, Unigene40355\_JingChaoZuZhi2, Unigene4209\_JingChaoZuZhi2, Unigene4299\_JingChaoZuZhi2, Unigene43944\_JingChaoZuZhi2, Unigene44366\_JingChaoZuZhi2, Unigene4540\_JingChaoZuZhi2, Unigene46171\_JingChaoZuZhi2, Unigene46497\_JingChaoZuZhi2, Unigene46750\_JingChaoZuZhi2, Unigene49018\_JingChaoZuZhi2, Unigene49664\_JingChaoZuZhi2, Unigene49798\_JingChaoZuZhi2, Unigene50479\_JingChaoZuZhi2, Unigene50573\_JingChaoZuZhi2, Unigene50713\_JingChaoZuZhi2, Unigene5072\_JingChaoZuZhi2, Unigene5099\_JingChaoZuZhi2, Unigene5148\_JingChaoZuZhi2, Unigene51820\_JingChaoZuZhi2, Unigene52208\_JingChaoZuZhi2, Unigene52295\_JingChaoZuZhi2, Unigene52743\_JingChaoZuZhi2, Unigene5289\_JingChaoZuZhi2, Unigene53013\_JingChaoZuZhi2, Unigene5312\_JingChaoZuZhi2, Unigene53417\_JingChaoZuZhi2, Unigene53568\_JingChaoZuZhi2, Unigene53885\_JingChaoZuZhi2, Unigene53953\_JingChaoZuZhi2, Unigene53958\_JingChaoZuZhi2, Unigene5410\_JingChaoZuZhi2, Unigene54470\_JingChaoZuZhi2, Unigene54738\_JingChaoZuZhi2, Unigene55100\_JingChaoZuZhi2, Unigene55832\_JingChaoZuZhi2, Unigene58134\_JingChaoZuZhi2, Unigene58386\_JingChaoZuZhi2, Unigene59480\_JingChaoZuZhi2, Unigene59577\_JingChaoZuZhi2, Unigene59580\_JingChaoZuZhi2, Unigene60654\_JingChaoZuZhi2, Unigene60788\_JingChaoZuZhi2, Unigene6082\_JingChaoZuZhi2, Unigene61342\_JingChaoZuZhi2, Unigene62511\_JingChaoZuZhi2, Unigene6262\_JingChaoZuZhi2, Unigene62786\_JingChaoZuZhi2, Unigene63663\_JingChaoZuZhi2, Unigene6381\_JingChaoZuZhi2, Unigene64583\_JingChaoZuZhi2, Unigene64702\_JingChaoZuZhi2, Unigene65337\_JingChaoZuZhi2, Unigene65524\_JingChaoZuZhi2, Unigene65591\_JingChaoZuZhi2, Unigene65711\_JingChaoZuZhi2, Unigene65775\_JingChaoZuZhi2, Unigene65856\_JingChaoZuZhi2, Unigene66918\_JingChaoZuZhi2, Unigene66983\_JingChaoZuZhi2, Unigene67117\_JingChaoZuZhi2, Unigene67389\_JingChaoZuZhi2, Unigene68159\_JingChaoZuZhi2, Unigene69025\_JingChaoZuZhi2, Unigene69049\_JingChaoZuZhi2, Unigene69203\_JingChaoZuZhi2, Unigene6933\_JingChaoZuZhi2, Unigene69733\_JingChaoZuZhi2, Unigene69786\_JingChaoZuZhi2, Unigene70100\_JingChaoZuZhi2, Unigene70123\_JingChaoZuZhi2, Unigene71079\_JingChaoZuZhi2, Unigene71161\_JingChaoZuZhi2, Unigene71224\_JingChaoZuZhi2, Unigene71734\_JingChaoZuZhi2, Unigene72136\_JingChaoZuZhi2, Unigene72269\_JingChaoZuZhi2, Unigene72507\_JingChaoZuZhi2, Unigene72521\_JingChaoZuZhi2, Unigene73482\_JingChaoZuZhi2, Unigene73826\_JingChaoZuZhi2, Unigene74601\_JingChaoZuZhi2, Unigene74\_JingChaoZuZhi2, Unigene75415\_JingChaoZuZhi2, Unigene75577\_JingChaoZuZhi2, Unigene75781\_JingChaoZuZhi2, Unigene75899\_JingChaoZuZhi2, Unigene76400\_JingChaoZuZhi2, Unigene76524\_JingChaoZuZhi2, Unigene76809\_JingChaoZuZhi2, Unigene76863\_JingChaoZuZhi2, Unigene76904\_JingChaoZuZhi2, Unigene77003\_JingChaoZuZhi2, Unigene77262\_JingChaoZuZhi2, Unigene77618\_JingChaoZuZhi2, Unigene77748\_JingChaoZuZhi2, Unigene78168\_JingChaoZuZhi2, Unigene78574\_JingChaoZuZhi2, Unigene78632\_JingChaoZuZhi2, Unigene78758\_JingChaoZuZhi2, Unigene78888\_JingChaoZuZhi2, Unigene78895\_JingChaoZuZhi2, Unigene79081\_JingChaoZuZhi2, Unigene79166\_JingChaoZuZhi2, Unigene79193\_JingChaoZuZhi2, Unigene7939\_JingChaoZuZhi2, Unigene79486\_JingChaoZuZhi2, Unigene79606\_JingChaoZuZhi2, Unigene79608\_JingChaoZuZhi2, Unigene79683\_JingChaoZuZhi2, Unigene79768\_JingChaoZuZhi2, Unigene79805\_JingChaoZuZhi2, Unigene7985\_JingChaoZuZhi2, Unigene80204\_JingChaoZuZhi2, Unigene80282\_JingChaoZuZhi2, Unigene80893\_JingChaoZuZhi2, Unigene81151\_JingChaoZuZhi2, Unigene8133\_JingChaoZuZhi2, Unigene81440\_JingChaoZuZhi2, Unigene81670\_JingChaoZuZhi2, Unigene81725\_JingChaoZuZhi2, Unigene81789\_JingChaoZuZhi2, Unigene8193\_JingChaoZuZhi2, Unigene82058\_JingChaoZuZhi2, Unigene82367\_JingChaoZuZhi2, Unigene82389\_JingChaoZuZhi2, Unigene82530\_JingChaoZuZhi2, Unigene82567\_JingChaoZuZhi2, Unigene82656\_JingChaoZuZhi2, Unigene83393\_JingChaoZuZhi2, Unigene8341\_JingChaoZuZhi2, Unigene83524\_JingChaoZuZhi2, Unigene83528\_JingChaoZuZhi2, Unigene83542\_JingChaoZuZhi2, Unigene83667\_JingChaoZuZhi2, Unigene83843\_JingChaoZuZhi2, Unigene83944\_JingChaoZuZhi2, Unigene84095\_JingChaoZuZhi2, Unigene84115\_JingChaoZuZhi2, Unigene84178\_JingChaoZuZhi2, Unigene84219\_JingChaoZuZhi2, Unigene84599\_JingChaoZuZhi2, Unigene84830\_JingChaoZuZhi2, Unigene84955\_JingChaoZuZhi2, Unigene85464\_JingChaoZuZhi2, Unigene85635\_JingChaoZuZhi2, Unigene85689\_JingChaoZuZhi2, Unigene85762\_JingChaoZuZhi2, Unigene85980\_JingChaoZuZhi2, Unigene8600\_JingChaoZuZhi2, Unigene86417\_JingChaoZuZhi2, Unigene86640\_JingChaoZuZhi2, Unigene86648\_JingChaoZuZhi2, Unigene86781\_JingChaoZuZhi2, Unigene86996\_JingChaoZuZhi2, Unigene87254\_JingChaoZuZhi2, Unigene87457\_JingChaoZuZhi2, Unigene87585\_JingChaoZuZhi2, Unigene87597\_JingChaoZuZhi2, Unigene8764\_JingChaoZuZhi2, Unigene87780\_JingChaoZuZhi2, Unigene88009\_JingChaoZuZhi2, Unigene8806\_JingChaoZuZhi2, Unigene88090\_JingChaoZuZhi2, Unigene88131\_JingChaoZuZhi2, Unigene88213\_JingChaoZuZhi2, Unigene88522\_JingChaoZuZhi2, Unigene88690\_JingChaoZuZhi2, Unigene88887\_JingChaoZuZhi2, Unigene88942\_JingChaoZuZhi2, Unigene89011\_JingChaoZuZhi2, Unigene89322\_JingChaoZuZhi2, Unigene89339\_JingChaoZuZhi2, Unigene89398\_JingChaoZuZhi2, Unigene89409\_JingChaoZuZhi2, Unigene8945\_JingChaoZuZhi2, Unigene89629\_JingChaoZuZhi2, Unigene89755\_JingChaoZuZhi2, Unigene89969\_JingChaoZuZhi2, Unigene90110\_JingChaoZuZhi2, Unigene90116\_JingChaoZuZhi2, Unigene90154\_JingChaoZuZhi2, Unigene90213\_JingChaoZuZhi2, Unigene90222\_JingChaoZuZhi2, Unigene90353\_JingChaoZuZhi2, Unigene90427\_JingChaoZuZhi2, Unigene90527\_JingChaoZuZhi2, Unigene90530\_JingChaoZuZhi2, Unigene90767\_JingChaoZuZhi2, Unigene90923\_JingChaoZuZhi2, Unigene90979\_JingChaoZuZhi2, Unigene91204\_JingChaoZuZhi2, Unigene91328\_JingChaoZuZhi2, Unigene91427\_JingChaoZuZhi2, Unigene91621\_JingChaoZuZhi2, Unigene91628\_JingChaoZuZhi2, Unigene91703\_JingChaoZuZhi2, Unigene91717\_JingChaoZuZhi2, Unigene91911\_JingChaoZuZhi2, Unigene91944\_JingChaoZuZhi2, Unigene91978\_JingChaoZuZhi2, Unigene92019\_JingChaoZuZhi2, Unigene92141\_JingChaoZuZhi2, Unigene92247\_JingChaoZuZhi2, Unigene92319\_JingChaoZuZhi2, Unigene92341\_JingChaoZuZhi2, Unigene92394\_JingChaoZuZhi2, Unigene92395\_JingChaoZuZhi2, Unigene92438\_JingChaoZuZhi2, Unigene92624\_JingChaoZuZhi2, Unigene92756\_JingChaoZuZhi2, Unigene92785\_JingChaoZuZhi2, Unigene92795\_JingChaoZuZhi2, Unigene92937\_JingChaoZuZhi2, Unigene92941\_JingChaoZuZhi2, Unigene93010\_JingChaoZuZhi2, Unigene93103\_JingChaoZuZhi2, Unigene93112\_JingChaoZuZhi2, Unigene93186\_JingChaoZuZhi2, Unigene93207\_JingChaoZuZhi2, Unigene93257\_JingChaoZuZhi2, Unigene932\_JingChaoZuZhi2, Unigene93320\_JingChaoZuZhi2, Unigene93325\_JingChaoZuZhi2, Unigene93327\_JingChaoZuZhi2, Unigene93390\_JingChaoZuZhi2, Unigene93439\_JingChaoZuZhi2, Unigene93693\_JingChaoZuZhi2, Unigene93782\_JingChaoZuZhi2, Unigene93793\_JingChaoZuZhi2, Unigene9404\_JingChaoZuZhi2, Unigene94280\_JingChaoZuZhi2, Unigene94333\_JingChaoZuZhi2, Unigene94347\_JingChaoZuZhi2, Unigene94362\_JingChaoZuZhi2, Unigene9438\_JingChaoZuZhi2, Unigene94533\_JingChaoZuZhi2, Unigene94721\_JingChaoZuZhi2, Unigene94890\_JingChaoZuZhi2, Unigene94977\_JingChaoZuZhi2, Unigene95065\_JingChaoZuZhi2, Unigene95280\_JingChaoZuZhi2, Unigene95427\_JingChaoZuZhi2, Unigene95463\_JingChaoZuZhi2, Unigene95526\_JingChaoZuZhi2, Unigene95722\_JingChaoZuZhi2, Unigene95881\_JingChaoZuZhi2, Unigene95883\_JingChaoZuZhi2, Unigene95897\_JingChaoZuZhi2, Unigene95951\_JingChaoZuZhi2, Unigene95984\_JingChaoZuZhi2, Unigene95987\_JingChaoZuZhi2, Unigene95994\_JingChaoZuZhi2, Unigene96068\_JingChaoZuZhi2, Unigene96114\_JingChaoZuZhi2, Unigene96180\_JingChaoZuZhi2, Unigene96193\_JingChaoZuZhi2, Unigene96248\_JingChaoZuZhi2, Unigene96265\_JingChaoZuZhi2, Unigene96269\_JingChaoZuZhi2, Unigene96281\_JingChaoZuZhi2, Unigene96303\_JingChaoZuZhi2 |
| 21 | Ubiquitin mediated proteolysis | Unigene10125\_JingChaoZuZhi2, Unigene10134\_JingChaoZuZhi2, Unigene10300\_JingChaoZuZhi2, Unigene10309\_JingChaoZuZhi2, Unigene10364\_JingChaoZuZhi2, Unigene10413\_JingChaoZuZhi2, Unigene10599\_JingChaoZuZhi2, Unigene10679\_JingChaoZuZhi2, Unigene10755\_JingChaoZuZhi2, Unigene10770\_JingChaoZuZhi2, Unigene10847\_JingChaoZuZhi2, Unigene10858\_JingChaoZuZhi2, Unigene10878\_JingChaoZuZhi2, Unigene11293\_JingChaoZuZhi2, Unigene11389\_JingChaoZuZhi2, Unigene11402\_JingChaoZuZhi2, Unigene11545\_JingChaoZuZhi2, Unigene11558\_JingChaoZuZhi2, Unigene1157\_JingChaoZuZhi2, Unigene11712\_JingChaoZuZhi2, Unigene11718\_JingChaoZuZhi2, Unigene11906\_JingChaoZuZhi2, Unigene11932\_JingChaoZuZhi2, Unigene11996\_JingChaoZuZhi2, Unigene12021\_JingChaoZuZhi2, Unigene12058\_JingChaoZuZhi2, Unigene12097\_JingChaoZuZhi2, Unigene12120\_JingChaoZuZhi2, Unigene12558\_JingChaoZuZhi2, Unigene12564\_JingChaoZuZhi2, Unigene12648\_JingChaoZuZhi2, Unigene12677\_JingChaoZuZhi2, Unigene12809\_JingChaoZuZhi2, Unigene1329\_JingChaoZuZhi2, Unigene13354\_JingChaoZuZhi2, Unigene13615\_JingChaoZuZhi2, Unigene13857\_JingChaoZuZhi2, Unigene13955\_JingChaoZuZhi2, Unigene14167\_JingChaoZuZhi2, Unigene14215\_JingChaoZuZhi2, Unigene14238\_JingChaoZuZhi2, Unigene14243\_JingChaoZuZhi2, Unigene14361\_JingChaoZuZhi2, Unigene15151\_JingChaoZuZhi2, Unigene15229\_JingChaoZuZhi2, Unigene15285\_JingChaoZuZhi2, Unigene15346\_JingChaoZuZhi2, Unigene15404\_JingChaoZuZhi2, Unigene15421\_JingChaoZuZhi2, Unigene15441\_JingChaoZuZhi2, Unigene15446\_JingChaoZuZhi2, Unigene15555\_JingChaoZuZhi2, Unigene15781\_JingChaoZuZhi2, Unigene15943\_JingChaoZuZhi2, Unigene15993\_JingChaoZuZhi2, Unigene16285\_JingChaoZuZhi2, Unigene16590\_JingChaoZuZhi2, Unigene167\_JingChaoZuZhi2, Unigene16834\_JingChaoZuZhi2, Unigene1703\_JingChaoZuZhi2, Unigene17115\_JingChaoZuZhi2, Unigene17478\_JingChaoZuZhi2, Unigene17533\_JingChaoZuZhi2, Unigene17734\_JingChaoZuZhi2, Unigene17764\_JingChaoZuZhi2, Unigene1790\_JingChaoZuZhi2, Unigene17930\_JingChaoZuZhi2, Unigene18008\_JingChaoZuZhi2, Unigene18139\_JingChaoZuZhi2, Unigene18209\_JingChaoZuZhi2, Unigene18238\_JingChaoZuZhi2, Unigene1828\_JingChaoZuZhi2, Unigene18377\_JingChaoZuZhi2, Unigene18459\_JingChaoZuZhi2, Unigene1847\_JingChaoZuZhi2, Unigene18493\_JingChaoZuZhi2, Unigene18777\_JingChaoZuZhi2, Unigene18986\_JingChaoZuZhi2, Unigene19013\_JingChaoZuZhi2, Unigene19242\_JingChaoZuZhi2, Unigene19361\_JingChaoZuZhi2, Unigene19470\_JingChaoZuZhi2, Unigene19517\_JingChaoZuZhi2, Unigene19617\_JingChaoZuZhi2, Unigene19660\_JingChaoZuZhi2, Unigene19691\_JingChaoZuZhi2, Unigene19798\_JingChaoZuZhi2, Unigene19826\_JingChaoZuZhi2, Unigene19829\_JingChaoZuZhi2, Unigene19832\_JingChaoZuZhi2, Unigene20102\_JingChaoZuZhi2, Unigene20253\_JingChaoZuZhi2, Unigene20305\_JingChaoZuZhi2, Unigene20415\_JingChaoZuZhi2, Unigene20428\_JingChaoZuZhi2, Unigene20432\_JingChaoZuZhi2, Unigene20489\_JingChaoZuZhi2, Unigene20547\_JingChaoZuZhi2, Unigene20755\_JingChaoZuZhi2, Unigene20868\_JingChaoZuZhi2, Unigene20898\_JingChaoZuZhi2, Unigene20902\_JingChaoZuZhi2, Unigene20907\_JingChaoZuZhi2, Unigene21197\_JingChaoZuZhi2, Unigene21385\_JingChaoZuZhi2, Unigene21706\_JingChaoZuZhi2, Unigene21848\_JingChaoZuZhi2, Unigene21891\_JingChaoZuZhi2, Unigene21911\_JingChaoZuZhi2, Unigene21992\_JingChaoZuZhi2, Unigene22043\_JingChaoZuZhi2, Unigene22090\_JingChaoZuZhi2, Unigene22290\_JingChaoZuZhi2, Unigene22299\_JingChaoZuZhi2, Unigene22370\_JingChaoZuZhi2, Unigene22407\_JingChaoZuZhi2, Unigene22456\_JingChaoZuZhi2, Unigene22500\_JingChaoZuZhi2, Unigene22571\_JingChaoZuZhi2, Unigene22622\_JingChaoZuZhi2, Unigene22669\_JingChaoZuZhi2, Unigene22676\_JingChaoZuZhi2, Unigene22716\_JingChaoZuZhi2, Unigene22753\_JingChaoZuZhi2, Unigene22787\_JingChaoZuZhi2, Unigene22820\_JingChaoZuZhi2, Unigene24037\_JingChaoZuZhi2, Unigene2438\_JingChaoZuZhi2, Unigene2444\_JingChaoZuZhi2, Unigene26435\_JingChaoZuZhi2, Unigene2691\_JingChaoZuZhi2, Unigene2692\_JingChaoZuZhi2, Unigene28939\_JingChaoZuZhi2, Unigene2943\_JingChaoZuZhi2, Unigene30386\_JingChaoZuZhi2, Unigene30634\_JingChaoZuZhi2, Unigene30967\_JingChaoZuZhi2, Unigene31934\_JingChaoZuZhi2, Unigene33783\_JingChaoZuZhi2, Unigene34912\_JingChaoZuZhi2, Unigene35730\_JingChaoZuZhi2, Unigene3585\_JingChaoZuZhi2, Unigene36424\_JingChaoZuZhi2, Unigene36737\_JingChaoZuZhi2, Unigene36930\_JingChaoZuZhi2, Unigene37472\_JingChaoZuZhi2, Unigene37741\_JingChaoZuZhi2, Unigene37803\_JingChaoZuZhi2, Unigene39189\_JingChaoZuZhi2, Unigene40670\_JingChaoZuZhi2, Unigene4136\_JingChaoZuZhi2, Unigene42137\_JingChaoZuZhi2, Unigene4222\_JingChaoZuZhi2, Unigene42969\_JingChaoZuZhi2, Unigene43322\_JingChaoZuZhi2, Unigene4345\_JingChaoZuZhi2, Unigene44191\_JingChaoZuZhi2, Unigene45731\_JingChaoZuZhi2, Unigene45739\_JingChaoZuZhi2, Unigene46003\_JingChaoZuZhi2, Unigene46171\_JingChaoZuZhi2, Unigene46508\_JingChaoZuZhi2, Unigene46937\_JingChaoZuZhi2, Unigene473\_JingChaoZuZhi2, Unigene50479\_JingChaoZuZhi2, Unigene5099\_JingChaoZuZhi2, Unigene51238\_JingChaoZuZhi2, Unigene51415\_JingChaoZuZhi2, Unigene51568\_JingChaoZuZhi2, Unigene5163\_JingChaoZuZhi2, Unigene5165\_JingChaoZuZhi2, Unigene51875\_JingChaoZuZhi2, Unigene52452\_JingChaoZuZhi2, Unigene53027\_JingChaoZuZhi2, Unigene5312\_JingChaoZuZhi2, Unigene53741\_JingChaoZuZhi2, Unigene53953\_JingChaoZuZhi2, Unigene54030\_JingChaoZuZhi2, Unigene54101\_JingChaoZuZhi2, Unigene54166\_JingChaoZuZhi2, Unigene54292\_JingChaoZuZhi2, Unigene54781\_JingChaoZuZhi2, Unigene5608\_JingChaoZuZhi2, Unigene56772\_JingChaoZuZhi2, Unigene57074\_JingChaoZuZhi2, Unigene57781\_JingChaoZuZhi2, Unigene57989\_JingChaoZuZhi2, Unigene58110\_JingChaoZuZhi2, Unigene58348\_JingChaoZuZhi2, Unigene58386\_JingChaoZuZhi2, Unigene58529\_JingChaoZuZhi2, Unigene58767\_JingChaoZuZhi2, Unigene59068\_JingChaoZuZhi2, Unigene59421\_JingChaoZuZhi2, Unigene59471\_JingChaoZuZhi2, Unigene59580\_JingChaoZuZhi2, Unigene59885\_JingChaoZuZhi2, Unigene60647\_JingChaoZuZhi2, Unigene6082\_JingChaoZuZhi2, Unigene61044\_JingChaoZuZhi2, Unigene61178\_JingChaoZuZhi2, Unigene61381\_JingChaoZuZhi2, Unigene61590\_JingChaoZuZhi2, Unigene6381\_JingChaoZuZhi2, Unigene63966\_JingChaoZuZhi2, Unigene6439\_JingChaoZuZhi2, Unigene64842\_JingChaoZuZhi2, Unigene64888\_JingChaoZuZhi2, Unigene64890\_JingChaoZuZhi2, Unigene65241\_JingChaoZuZhi2, Unigene65337\_JingChaoZuZhi2, Unigene65536\_JingChaoZuZhi2, Unigene65879\_JingChaoZuZhi2, Unigene66231\_JingChaoZuZhi2, Unigene66253\_JingChaoZuZhi2, Unigene66299\_JingChaoZuZhi2, Unigene66479\_JingChaoZuZhi2, Unigene66498\_JingChaoZuZhi2, Unigene66743\_JingChaoZuZhi2, Unigene6700\_JingChaoZuZhi2, Unigene6710\_JingChaoZuZhi2, Unigene67308\_JingChaoZuZhi2, Unigene6732\_JingChaoZuZhi2, Unigene6772\_JingChaoZuZhi2, Unigene6786\_JingChaoZuZhi2, Unigene67909\_JingChaoZuZhi2, Unigene68053\_JingChaoZuZhi2, Unigene68312\_JingChaoZuZhi2, Unigene68762\_JingChaoZuZhi2, Unigene69195\_JingChaoZuZhi2, Unigene69203\_JingChaoZuZhi2, Unigene69935\_JingChaoZuZhi2, Unigene70006\_JingChaoZuZhi2, Unigene7002\_JingChaoZuZhi2, Unigene70063\_JingChaoZuZhi2, Unigene7018\_JingChaoZuZhi2, Unigene70701\_JingChaoZuZhi2, Unigene71161\_JingChaoZuZhi2, Unigene7139\_JingChaoZuZhi2, Unigene72561\_JingChaoZuZhi2, Unigene72925\_JingChaoZuZhi2, Unigene72926\_JingChaoZuZhi2, Unigene7315\_JingChaoZuZhi2, Unigene73220\_JingChaoZuZhi2, Unigene7345\_JingChaoZuZhi2, Unigene73510\_JingChaoZuZhi2, Unigene73685\_JingChaoZuZhi2, Unigene73688\_JingChaoZuZhi2, Unigene73826\_JingChaoZuZhi2, Unigene73920\_JingChaoZuZhi2, Unigene74040\_JingChaoZuZhi2, Unigene74892\_JingChaoZuZhi2, Unigene74943\_JingChaoZuZhi2, Unigene7511\_JingChaoZuZhi2, Unigene75283\_JingChaoZuZhi2, Unigene75293\_JingChaoZuZhi2, Unigene7557\_JingChaoZuZhi2, Unigene7621\_JingChaoZuZhi2, Unigene76639\_JingChaoZuZhi2, Unigene76809\_JingChaoZuZhi2, Unigene77262\_JingChaoZuZhi2, Unigene77534\_JingChaoZuZhi2, Unigene77742\_JingChaoZuZhi2, Unigene78004\_JingChaoZuZhi2, Unigene78024\_JingChaoZuZhi2, Unigene7813\_JingChaoZuZhi2, Unigene78168\_JingChaoZuZhi2, Unigene78198\_JingChaoZuZhi2, Unigene78441\_JingChaoZuZhi2, Unigene78722\_JingChaoZuZhi2, Unigene78866\_JingChaoZuZhi2, Unigene79001\_JingChaoZuZhi2, Unigene79048\_JingChaoZuZhi2, Unigene79715\_JingChaoZuZhi2, Unigene79776\_JingChaoZuZhi2, Unigene79826\_JingChaoZuZhi2, Unigene80315\_JingChaoZuZhi2, Unigene8061\_JingChaoZuZhi2, Unigene80863\_JingChaoZuZhi2, Unigene80960\_JingChaoZuZhi2, Unigene81000\_JingChaoZuZhi2, Unigene81186\_JingChaoZuZhi2, Unigene8142\_JingChaoZuZhi2, Unigene81980\_JingChaoZuZhi2, Unigene82094\_JingChaoZuZhi2, Unigene82526\_JingChaoZuZhi2, Unigene82567\_JingChaoZuZhi2, Unigene82675\_JingChaoZuZhi2, Unigene83651\_JingChaoZuZhi2, Unigene83698\_JingChaoZuZhi2, Unigene84337\_JingChaoZuZhi2, Unigene84498\_JingChaoZuZhi2, Unigene84830\_JingChaoZuZhi2, Unigene84934\_JingChaoZuZhi2, Unigene84986\_JingChaoZuZhi2, Unigene85041\_JingChaoZuZhi2, Unigene85140\_JingChaoZuZhi2, Unigene85359\_JingChaoZuZhi2, Unigene86498\_JingChaoZuZhi2, Unigene86617\_JingChaoZuZhi2, Unigene86754\_JingChaoZuZhi2, Unigene86908\_JingChaoZuZhi2, Unigene86927\_JingChaoZuZhi2, Unigene8711\_JingChaoZuZhi2, Unigene87155\_JingChaoZuZhi2, Unigene87200\_JingChaoZuZhi2, Unigene87345\_JingChaoZuZhi2, Unigene87375\_JingChaoZuZhi2, Unigene87745\_JingChaoZuZhi2, Unigene87780\_JingChaoZuZhi2, Unigene88363\_JingChaoZuZhi2, Unigene88522\_JingChaoZuZhi2, Unigene88635\_JingChaoZuZhi2, Unigene88730\_JingChaoZuZhi2, Unigene88837\_JingChaoZuZhi2, Unigene88926\_JingChaoZuZhi2, Unigene88942\_JingChaoZuZhi2, Unigene89011\_JingChaoZuZhi2, Unigene89092\_JingChaoZuZhi2, Unigene89157\_JingChaoZuZhi2, Unigene89310\_JingChaoZuZhi2, Unigene89335\_JingChaoZuZhi2, Unigene89398\_JingChaoZuZhi2, Unigene89554\_JingChaoZuZhi2, Unigene89782\_JingChaoZuZhi2, Unigene89794\_JingChaoZuZhi2, Unigene89893\_JingChaoZuZhi2, Unigene9003\_JingChaoZuZhi2, Unigene90163\_JingChaoZuZhi2, Unigene90353\_JingChaoZuZhi2, Unigene90500\_JingChaoZuZhi2, Unigene90567\_JingChaoZuZhi2, Unigene90653\_JingChaoZuZhi2, Unigene90671\_JingChaoZuZhi2, Unigene90823\_JingChaoZuZhi2, Unigene90921\_JingChaoZuZhi2, Unigene90979\_JingChaoZuZhi2, Unigene9128\_JingChaoZuZhi2, Unigene91493\_JingChaoZuZhi2, Unigene91540\_JingChaoZuZhi2, Unigene91617\_JingChaoZuZhi2, Unigene91731\_JingChaoZuZhi2, Unigene91791\_JingChaoZuZhi2, Unigene91965\_JingChaoZuZhi2, Unigene91978\_JingChaoZuZhi2, Unigene9210\_JingChaoZuZhi2, Unigene92201\_JingChaoZuZhi2, Unigene92438\_JingChaoZuZhi2, Unigene92547\_JingChaoZuZhi2, Unigene92581\_JingChaoZuZhi2, Unigene92587\_JingChaoZuZhi2, Unigene93018\_JingChaoZuZhi2, Unigene93103\_JingChaoZuZhi2, Unigene93288\_JingChaoZuZhi2, Unigene932\_JingChaoZuZhi2, Unigene93454\_JingChaoZuZhi2, Unigene93458\_JingChaoZuZhi2, Unigene93479\_JingChaoZuZhi2, Unigene93502\_JingChaoZuZhi2, Unigene93557\_JingChaoZuZhi2, Unigene93794\_JingChaoZuZhi2, Unigene93802\_JingChaoZuZhi2, Unigene93823\_JingChaoZuZhi2, Unigene9404\_JingChaoZuZhi2, Unigene94116\_JingChaoZuZhi2, Unigene94232\_JingChaoZuZhi2, Unigene94239\_JingChaoZuZhi2, Unigene94332\_JingChaoZuZhi2, Unigene94337\_JingChaoZuZhi2, Unigene94563\_JingChaoZuZhi2, Unigene94576\_JingChaoZuZhi2, Unigene94636\_JingChaoZuZhi2, Unigene94738\_JingChaoZuZhi2, Unigene94805\_JingChaoZuZhi2, Unigene94835\_JingChaoZuZhi2, Unigene95065\_JingChaoZuZhi2, Unigene95096\_JingChaoZuZhi2, Unigene95109\_JingChaoZuZhi2, Unigene95276\_JingChaoZuZhi2, Unigene95376\_JingChaoZuZhi2, Unigene95433\_JingChaoZuZhi2, Unigene95436\_JingChaoZuZhi2, Unigene95500\_JingChaoZuZhi2, Unigene95552\_JingChaoZuZhi2, Unigene95665\_JingChaoZuZhi2, Unigene95674\_JingChaoZuZhi2, Unigene95855\_JingChaoZuZhi2, Unigene95866\_JingChaoZuZhi2, Unigene9595\_JingChaoZuZhi2, Unigene95970\_JingChaoZuZhi2, Unigene96058\_JingChaoZuZhi2, Unigene96103\_JingChaoZuZhi2, Unigene96215\_JingChaoZuZhi2, Unigene96269\_JingChaoZuZhi2, Unigene96281\_JingChaoZuZhi2, Unigene96302\_JingChaoZuZhi2, Unigene9630\_JingChaoZuZhi2, Unigene9688\_JingChaoZuZhi2, Unigene9711\_JingChaoZuZhi2, Unigene9789\_JingChaoZuZhi2 |
| 22 | Pyrimidine metabolism | Unigene10005\_JingChaoZuZhi2, Unigene10045\_JingChaoZuZhi2, Unigene10129\_JingChaoZuZhi2, Unigene10340\_JingChaoZuZhi2, Unigene1037\_JingChaoZuZhi2, Unigene103\_JingChaoZuZhi2, Unigene10689\_JingChaoZuZhi2, Unigene10769\_JingChaoZuZhi2, Unigene10802\_JingChaoZuZhi2, Unigene10883\_JingChaoZuZhi2, Unigene10888\_JingChaoZuZhi2, Unigene10939\_JingChaoZuZhi2, Unigene10976\_JingChaoZuZhi2, Unigene10979\_JingChaoZuZhi2, Unigene11134\_JingChaoZuZhi2, Unigene1154\_JingChaoZuZhi2, Unigene11564\_JingChaoZuZhi2, Unigene1164\_JingChaoZuZhi2, Unigene11700\_JingChaoZuZhi2, Unigene12166\_JingChaoZuZhi2, Unigene1229\_JingChaoZuZhi2, Unigene12404\_JingChaoZuZhi2, Unigene12617\_JingChaoZuZhi2, Unigene12768\_JingChaoZuZhi2, Unigene12966\_JingChaoZuZhi2, Unigene1313\_JingChaoZuZhi2, Unigene13144\_JingChaoZuZhi2, Unigene13176\_JingChaoZuZhi2, Unigene1323\_JingChaoZuZhi2, Unigene13648\_JingChaoZuZhi2, Unigene13795\_JingChaoZuZhi2, Unigene13878\_JingChaoZuZhi2, Unigene13936\_JingChaoZuZhi2, Unigene13988\_JingChaoZuZhi2, Unigene14135\_JingChaoZuZhi2, Unigene14203\_JingChaoZuZhi2, Unigene14446\_JingChaoZuZhi2, Unigene14551\_JingChaoZuZhi2, Unigene14631\_JingChaoZuZhi2, Unigene1468\_JingChaoZuZhi2, Unigene15206\_JingChaoZuZhi2, Unigene15288\_JingChaoZuZhi2, Unigene15290\_JingChaoZuZhi2, Unigene15539\_JingChaoZuZhi2, Unigene16232\_JingChaoZuZhi2, Unigene1628\_JingChaoZuZhi2, Unigene16295\_JingChaoZuZhi2, Unigene16786\_JingChaoZuZhi2, Unigene16996\_JingChaoZuZhi2, Unigene16\_JingChaoZuZhi2, Unigene17035\_JingChaoZuZhi2, Unigene17065\_JingChaoZuZhi2, Unigene17221\_JingChaoZuZhi2, Unigene1738\_JingChaoZuZhi2, Unigene1744\_JingChaoZuZhi2, Unigene17636\_JingChaoZuZhi2, Unigene17861\_JingChaoZuZhi2, Unigene17\_JingChaoZuZhi2, Unigene18000\_JingChaoZuZhi2, Unigene18099\_JingChaoZuZhi2, Unigene18188\_JingChaoZuZhi2, Unigene18508\_JingChaoZuZhi2, Unigene18874\_JingChaoZuZhi2, Unigene19126\_JingChaoZuZhi2, Unigene19280\_JingChaoZuZhi2, Unigene19475\_JingChaoZuZhi2, Unigene19476\_JingChaoZuZhi2, Unigene19510\_JingChaoZuZhi2, Unigene19647\_JingChaoZuZhi2, Unigene19667\_JingChaoZuZhi2, Unigene19728\_JingChaoZuZhi2, Unigene19838\_JingChaoZuZhi2, Unigene19978\_JingChaoZuZhi2, Unigene19979\_JingChaoZuZhi2, Unigene20050\_JingChaoZuZhi2, Unigene20087\_JingChaoZuZhi2, Unigene2012\_JingChaoZuZhi2, Unigene20150\_JingChaoZuZhi2, Unigene20172\_JingChaoZuZhi2, Unigene20209\_JingChaoZuZhi2, Unigene20219\_JingChaoZuZhi2, Unigene20270\_JingChaoZuZhi2, Unigene20314\_JingChaoZuZhi2, Unigene20441\_JingChaoZuZhi2, Unigene20452\_JingChaoZuZhi2, Unigene2046\_JingChaoZuZhi2, Unigene20559\_JingChaoZuZhi2, Unigene20612\_JingChaoZuZhi2, Unigene20711\_JingChaoZuZhi2, Unigene20814\_JingChaoZuZhi2, Unigene20986\_JingChaoZuZhi2, Unigene21054\_JingChaoZuZhi2, Unigene21282\_JingChaoZuZhi2, Unigene21510\_JingChaoZuZhi2, Unigene2155\_JingChaoZuZhi2, Unigene21648\_JingChaoZuZhi2, Unigene21767\_JingChaoZuZhi2, Unigene22065\_JingChaoZuZhi2, Unigene22086\_JingChaoZuZhi2, Unigene22198\_JingChaoZuZhi2, Unigene22310\_JingChaoZuZhi2, Unigene22334\_JingChaoZuZhi2, Unigene22339\_JingChaoZuZhi2, Unigene22360\_JingChaoZuZhi2, Unigene22416\_JingChaoZuZhi2, Unigene22451\_JingChaoZuZhi2, Unigene22516\_JingChaoZuZhi2, Unigene22545\_JingChaoZuZhi2, Unigene2254\_JingChaoZuZhi2, Unigene22842\_JingChaoZuZhi2, Unigene22870\_JingChaoZuZhi2, Unigene22908\_JingChaoZuZhi2, Unigene22923\_JingChaoZuZhi2, Unigene23172\_JingChaoZuZhi2, Unigene23178\_JingChaoZuZhi2, Unigene23504\_JingChaoZuZhi2, Unigene2354\_JingChaoZuZhi2, Unigene25123\_JingChaoZuZhi2, Unigene2553\_JingChaoZuZhi2, Unigene26084\_JingChaoZuZhi2, Unigene26274\_JingChaoZuZhi2, Unigene27011\_JingChaoZuZhi2, Unigene2707\_JingChaoZuZhi2, Unigene29151\_JingChaoZuZhi2, Unigene2958\_JingChaoZuZhi2, Unigene30402\_JingChaoZuZhi2, Unigene30849\_JingChaoZuZhi2, Unigene31166\_JingChaoZuZhi2, Unigene31327\_JingChaoZuZhi2, Unigene31355\_JingChaoZuZhi2, Unigene3187\_JingChaoZuZhi2, Unigene322\_JingChaoZuZhi2, Unigene32589\_JingChaoZuZhi2, Unigene32848\_JingChaoZuZhi2, Unigene33470\_JingChaoZuZhi2, Unigene33523\_JingChaoZuZhi2, Unigene34338\_JingChaoZuZhi2, Unigene34397\_JingChaoZuZhi2, Unigene35605\_JingChaoZuZhi2, Unigene35687\_JingChaoZuZhi2, Unigene3592\_JingChaoZuZhi2, Unigene3632\_JingChaoZuZhi2, Unigene36386\_JingChaoZuZhi2, Unigene3682\_JingChaoZuZhi2, Unigene37224\_JingChaoZuZhi2, Unigene37317\_JingChaoZuZhi2, Unigene37457\_JingChaoZuZhi2, Unigene37504\_JingChaoZuZhi2, Unigene37516\_JingChaoZuZhi2, Unigene38162\_JingChaoZuZhi2, Unigene385\_JingChaoZuZhi2, Unigene38886\_JingChaoZuZhi2, Unigene38940\_JingChaoZuZhi2, Unigene3938\_JingChaoZuZhi2, Unigene39624\_JingChaoZuZhi2, Unigene3974\_JingChaoZuZhi2, Unigene4066\_JingChaoZuZhi2, Unigene41155\_JingChaoZuZhi2, Unigene41227\_JingChaoZuZhi2, Unigene414\_JingChaoZuZhi2, Unigene42364\_JingChaoZuZhi2, Unigene42890\_JingChaoZuZhi2, Unigene43072\_JingChaoZuZhi2, Unigene43410\_JingChaoZuZhi2, Unigene4346\_JingChaoZuZhi2, Unigene4375\_JingChaoZuZhi2, Unigene4377\_JingChaoZuZhi2, Unigene43926\_JingChaoZuZhi2, Unigene44252\_JingChaoZuZhi2, Unigene4426\_JingChaoZuZhi2, Unigene4461\_JingChaoZuZhi2, Unigene44745\_JingChaoZuZhi2, Unigene460\_JingChaoZuZhi2, Unigene46190\_JingChaoZuZhi2, Unigene47202\_JingChaoZuZhi2, Unigene4767\_JingChaoZuZhi2, Unigene47794\_JingChaoZuZhi2, Unigene47824\_JingChaoZuZhi2, Unigene4816\_JingChaoZuZhi2, Unigene486\_JingChaoZuZhi2, Unigene4900\_JingChaoZuZhi2, Unigene4977\_JingChaoZuZhi2, Unigene49985\_JingChaoZuZhi2, Unigene50422\_JingChaoZuZhi2, Unigene50927\_JingChaoZuZhi2, Unigene51540\_JingChaoZuZhi2, Unigene51835\_JingChaoZuZhi2, Unigene5200\_JingChaoZuZhi2, Unigene52751\_JingChaoZuZhi2, Unigene53362\_JingChaoZuZhi2, Unigene53510\_JingChaoZuZhi2, Unigene53809\_JingChaoZuZhi2, Unigene542\_JingChaoZuZhi2, Unigene5457\_JingChaoZuZhi2, Unigene5484\_JingChaoZuZhi2, Unigene5552\_JingChaoZuZhi2, Unigene56889\_JingChaoZuZhi2, Unigene57726\_JingChaoZuZhi2, Unigene57931\_JingChaoZuZhi2, Unigene5897\_JingChaoZuZhi2, Unigene5900\_JingChaoZuZhi2, Unigene59414\_JingChaoZuZhi2, Unigene59502\_JingChaoZuZhi2, Unigene59836\_JingChaoZuZhi2, Unigene6134\_JingChaoZuZhi2, Unigene61651\_JingChaoZuZhi2, Unigene61659\_JingChaoZuZhi2, Unigene61\_JingChaoZuZhi2, Unigene6219\_JingChaoZuZhi2, Unigene6233\_JingChaoZuZhi2, Unigene62546\_JingChaoZuZhi2, Unigene630\_JingChaoZuZhi2, Unigene63112\_JingChaoZuZhi2, Unigene63170\_JingChaoZuZhi2, Unigene64315\_JingChaoZuZhi2, Unigene64903\_JingChaoZuZhi2, Unigene65405\_JingChaoZuZhi2, Unigene65475\_JingChaoZuZhi2, Unigene65515\_JingChaoZuZhi2, Unigene65800\_JingChaoZuZhi2, Unigene65835\_JingChaoZuZhi2, Unigene662\_JingChaoZuZhi2, Unigene66656\_JingChaoZuZhi2, Unigene6685\_JingChaoZuZhi2, Unigene66954\_JingChaoZuZhi2, Unigene67077\_JingChaoZuZhi2, Unigene67318\_JingChaoZuZhi2, Unigene6790\_JingChaoZuZhi2, Unigene67964\_JingChaoZuZhi2, Unigene68129\_JingChaoZuZhi2, Unigene685\_JingChaoZuZhi2, Unigene69209\_JingChaoZuZhi2, Unigene6955\_JingChaoZuZhi2, Unigene69875\_JingChaoZuZhi2, Unigene69922\_JingChaoZuZhi2, Unigene70244\_JingChaoZuZhi2, Unigene70647\_JingChaoZuZhi2, Unigene71153\_JingChaoZuZhi2, Unigene71235\_JingChaoZuZhi2, Unigene71482\_JingChaoZuZhi2, Unigene7178\_JingChaoZuZhi2, Unigene7185\_JingChaoZuZhi2, Unigene72461\_JingChaoZuZhi2, Unigene72474\_JingChaoZuZhi2, Unigene73283\_JingChaoZuZhi2, Unigene73451\_JingChaoZuZhi2, Unigene74590\_JingChaoZuZhi2, Unigene75469\_JingChaoZuZhi2, Unigene75690\_JingChaoZuZhi2, Unigene76109\_JingChaoZuZhi2, Unigene76331\_JingChaoZuZhi2, Unigene76651\_JingChaoZuZhi2, Unigene76868\_JingChaoZuZhi2, Unigene76908\_JingChaoZuZhi2, Unigene77449\_JingChaoZuZhi2, Unigene77559\_JingChaoZuZhi2, Unigene77749\_JingChaoZuZhi2, Unigene77844\_JingChaoZuZhi2, Unigene78071\_JingChaoZuZhi2, Unigene78224\_JingChaoZuZhi2, Unigene78268\_JingChaoZuZhi2, Unigene7842\_JingChaoZuZhi2, Unigene78549\_JingChaoZuZhi2, Unigene78619\_JingChaoZuZhi2, Unigene78757\_JingChaoZuZhi2, Unigene78813\_JingChaoZuZhi2, Unigene79036\_JingChaoZuZhi2, Unigene79149\_JingChaoZuZhi2, Unigene79196\_JingChaoZuZhi2, Unigene79211\_JingChaoZuZhi2, Unigene7948\_JingChaoZuZhi2, Unigene79590\_JingChaoZuZhi2, Unigene79718\_JingChaoZuZhi2, Unigene7990\_JingChaoZuZhi2, Unigene79999\_JingChaoZuZhi2, Unigene80305\_JingChaoZuZhi2, Unigene81297\_JingChaoZuZhi2, Unigene81674\_JingChaoZuZhi2, Unigene8223\_JingChaoZuZhi2, Unigene8225\_JingChaoZuZhi2, Unigene82519\_JingChaoZuZhi2, Unigene8278\_JingChaoZuZhi2, Unigene82835\_JingChaoZuZhi2, Unigene83132\_JingChaoZuZhi2, Unigene83626\_JingChaoZuZhi2, Unigene83854\_JingChaoZuZhi2, Unigene84050\_JingChaoZuZhi2, Unigene84203\_JingChaoZuZhi2, Unigene84436\_JingChaoZuZhi2, Unigene84535\_JingChaoZuZhi2, Unigene84831\_JingChaoZuZhi2, Unigene84894\_JingChaoZuZhi2, Unigene8506\_JingChaoZuZhi2, Unigene8517\_JingChaoZuZhi2, Unigene85304\_JingChaoZuZhi2, Unigene85366\_JingChaoZuZhi2, Unigene85391\_JingChaoZuZhi2, Unigene85640\_JingChaoZuZhi2, Unigene85949\_JingChaoZuZhi2, Unigene86157\_JingChaoZuZhi2, Unigene86414\_JingChaoZuZhi2, Unigene8641\_JingChaoZuZhi2, Unigene8643\_JingChaoZuZhi2, Unigene86708\_JingChaoZuZhi2, Unigene86967\_JingChaoZuZhi2, Unigene87089\_JingChaoZuZhi2, Unigene87092\_JingChaoZuZhi2, Unigene87422\_JingChaoZuZhi2, Unigene8755\_JingChaoZuZhi2, Unigene87820\_JingChaoZuZhi2, Unigene87855\_JingChaoZuZhi2, Unigene87\_JingChaoZuZhi2, Unigene88016\_JingChaoZuZhi2, Unigene88179\_JingChaoZuZhi2, Unigene88283\_JingChaoZuZhi2, Unigene88958\_JingChaoZuZhi2, Unigene89375\_JingChaoZuZhi2, Unigene89504\_JingChaoZuZhi2, Unigene89639\_JingChaoZuZhi2, Unigene89662\_JingChaoZuZhi2, Unigene8973\_JingChaoZuZhi2, Unigene89753\_JingChaoZuZhi2, Unigene89769\_JingChaoZuZhi2, Unigene90008\_JingChaoZuZhi2, Unigene90104\_JingChaoZuZhi2, Unigene90180\_JingChaoZuZhi2, Unigene90612\_JingChaoZuZhi2, Unigene90793\_JingChaoZuZhi2, Unigene90919\_JingChaoZuZhi2, Unigene90999\_JingChaoZuZhi2, Unigene91020\_JingChaoZuZhi2, Unigene91122\_JingChaoZuZhi2, Unigene91187\_JingChaoZuZhi2, Unigene91194\_JingChaoZuZhi2, Unigene91270\_JingChaoZuZhi2, Unigene91350\_JingChaoZuZhi2, Unigene91513\_JingChaoZuZhi2, Unigene91530\_JingChaoZuZhi2, Unigene91541\_JingChaoZuZhi2, Unigene91701\_JingChaoZuZhi2, Unigene91777\_JingChaoZuZhi2, Unigene91914\_JingChaoZuZhi2, Unigene91921\_JingChaoZuZhi2, Unigene91943\_JingChaoZuZhi2, Unigene92088\_JingChaoZuZhi2, Unigene92196\_JingChaoZuZhi2, Unigene92224\_JingChaoZuZhi2, Unigene92240\_JingChaoZuZhi2, Unigene92368\_JingChaoZuZhi2, Unigene92607\_JingChaoZuZhi2, Unigene92742\_JingChaoZuZhi2, Unigene92803\_JingChaoZuZhi2, Unigene92908\_JingChaoZuZhi2, Unigene92910\_JingChaoZuZhi2, Unigene92976\_JingChaoZuZhi2, Unigene92989\_JingChaoZuZhi2, Unigene93107\_JingChaoZuZhi2, Unigene93445\_JingChaoZuZhi2, Unigene93519\_JingChaoZuZhi2, Unigene93589\_JingChaoZuZhi2, Unigene93591\_JingChaoZuZhi2, Unigene93666\_JingChaoZuZhi2, Unigene93904\_JingChaoZuZhi2, Unigene93993\_JingChaoZuZhi2, Unigene94118\_JingChaoZuZhi2, Unigene94128\_JingChaoZuZhi2, Unigene94364\_JingChaoZuZhi2, Unigene94457\_JingChaoZuZhi2, Unigene94568\_JingChaoZuZhi2, Unigene94585\_JingChaoZuZhi2, Unigene94630\_JingChaoZuZhi2, Unigene94831\_JingChaoZuZhi2, Unigene94910\_JingChaoZuZhi2, Unigene94946\_JingChaoZuZhi2, Unigene95002\_JingChaoZuZhi2, Unigene95155\_JingChaoZuZhi2, Unigene95179\_JingChaoZuZhi2, Unigene95237\_JingChaoZuZhi2, Unigene95309\_JingChaoZuZhi2, Unigene95325\_JingChaoZuZhi2, Unigene95398\_JingChaoZuZhi2, Unigene95476\_JingChaoZuZhi2, Unigene9550\_JingChaoZuZhi2, Unigene95541\_JingChaoZuZhi2, Unigene95677\_JingChaoZuZhi2, Unigene9569\_JingChaoZuZhi2, Unigene95855\_JingChaoZuZhi2, Unigene95901\_JingChaoZuZhi2, Unigene96171\_JingChaoZuZhi2, Unigene96240\_JingChaoZuZhi2, Unigene963\_JingChaoZuZhi2 |
| 23 | Dorso-ventral axis formation | Unigene10008\_JingChaoZuZhi2, Unigene1023\_JingChaoZuZhi2, Unigene10275\_JingChaoZuZhi2, Unigene10332\_JingChaoZuZhi2, Unigene10416\_JingChaoZuZhi2, Unigene10422\_JingChaoZuZhi2, Unigene10762\_JingChaoZuZhi2, Unigene10780\_JingChaoZuZhi2, Unigene10891\_JingChaoZuZhi2, Unigene10971\_JingChaoZuZhi2, Unigene11280\_JingChaoZuZhi2, Unigene11420\_JingChaoZuZhi2, Unigene11587\_JingChaoZuZhi2, Unigene11620\_JingChaoZuZhi2, Unigene11668\_JingChaoZuZhi2, Unigene11902\_JingChaoZuZhi2, Unigene12133\_JingChaoZuZhi2, Unigene12420\_JingChaoZuZhi2, Unigene12508\_JingChaoZuZhi2, Unigene12647\_JingChaoZuZhi2, Unigene12729\_JingChaoZuZhi2, Unigene12730\_JingChaoZuZhi2, Unigene12815\_JingChaoZuZhi2, Unigene12838\_JingChaoZuZhi2, Unigene1287\_JingChaoZuZhi2, Unigene1298\_JingChaoZuZhi2, Unigene13045\_JingChaoZuZhi2, Unigene1304\_JingChaoZuZhi2, Unigene13225\_JingChaoZuZhi2, Unigene13238\_JingChaoZuZhi2, Unigene1325\_JingChaoZuZhi2, Unigene13354\_JingChaoZuZhi2, Unigene13518\_JingChaoZuZhi2, Unigene13736\_JingChaoZuZhi2, Unigene13798\_JingChaoZuZhi2, Unigene14180\_JingChaoZuZhi2, Unigene14196\_JingChaoZuZhi2, Unigene14251\_JingChaoZuZhi2, Unigene14269\_JingChaoZuZhi2, Unigene14476\_JingChaoZuZhi2, Unigene14666\_JingChaoZuZhi2, Unigene14851\_JingChaoZuZhi2, Unigene15055\_JingChaoZuZhi2, Unigene15157\_JingChaoZuZhi2, Unigene15367\_JingChaoZuZhi2, Unigene15469\_JingChaoZuZhi2, Unigene15548\_JingChaoZuZhi2, Unigene1554\_JingChaoZuZhi2, Unigene15772\_JingChaoZuZhi2, Unigene15850\_JingChaoZuZhi2, Unigene16182\_JingChaoZuZhi2, Unigene16271\_JingChaoZuZhi2, Unigene16294\_JingChaoZuZhi2, Unigene16321\_JingChaoZuZhi2, Unigene16386\_JingChaoZuZhi2, Unigene16433\_JingChaoZuZhi2, Unigene16521\_JingChaoZuZhi2, Unigene16606\_JingChaoZuZhi2, Unigene16655\_JingChaoZuZhi2, Unigene16933\_JingChaoZuZhi2, Unigene16953\_JingChaoZuZhi2, Unigene1702\_JingChaoZuZhi2, Unigene17211\_JingChaoZuZhi2, Unigene17455\_JingChaoZuZhi2, Unigene18064\_JingChaoZuZhi2, Unigene18211\_JingChaoZuZhi2, Unigene18237\_JingChaoZuZhi2, Unigene18287\_JingChaoZuZhi2, Unigene18359\_JingChaoZuZhi2, Unigene1835\_JingChaoZuZhi2, Unigene18405\_JingChaoZuZhi2, Unigene18409\_JingChaoZuZhi2, Unigene18415\_JingChaoZuZhi2, Unigene18490\_JingChaoZuZhi2, Unigene18609\_JingChaoZuZhi2, Unigene1873\_JingChaoZuZhi2, Unigene18968\_JingChaoZuZhi2, Unigene19122\_JingChaoZuZhi2, Unigene19194\_JingChaoZuZhi2, Unigene19223\_JingChaoZuZhi2, Unigene19324\_JingChaoZuZhi2, Unigene19451\_JingChaoZuZhi2, Unigene1951\_JingChaoZuZhi2, Unigene19540\_JingChaoZuZhi2, Unigene19656\_JingChaoZuZhi2, Unigene19986\_JingChaoZuZhi2, Unigene20009\_JingChaoZuZhi2, Unigene20127\_JingChaoZuZhi2, Unigene20239\_JingChaoZuZhi2, Unigene2026\_JingChaoZuZhi2, Unigene20394\_JingChaoZuZhi2, Unigene20488\_JingChaoZuZhi2, Unigene20544\_JingChaoZuZhi2, Unigene21098\_JingChaoZuZhi2, Unigene21162\_JingChaoZuZhi2, Unigene21243\_JingChaoZuZhi2, Unigene21309\_JingChaoZuZhi2, Unigene21402\_JingChaoZuZhi2, Unigene21513\_JingChaoZuZhi2, Unigene21579\_JingChaoZuZhi2, Unigene21637\_JingChaoZuZhi2, Unigene21780\_JingChaoZuZhi2, Unigene21838\_JingChaoZuZhi2, Unigene21860\_JingChaoZuZhi2, Unigene2193\_JingChaoZuZhi2, Unigene21940\_JingChaoZuZhi2, Unigene22101\_JingChaoZuZhi2, Unigene2215\_JingChaoZuZhi2, Unigene22200\_JingChaoZuZhi2, Unigene22262\_JingChaoZuZhi2, Unigene22266\_JingChaoZuZhi2, Unigene22284\_JingChaoZuZhi2, Unigene22378\_JingChaoZuZhi2, Unigene22618\_JingChaoZuZhi2, Unigene22657\_JingChaoZuZhi2, Unigene2382\_JingChaoZuZhi2, Unigene2445\_JingChaoZuZhi2, Unigene252\_JingChaoZuZhi2, Unigene26360\_JingChaoZuZhi2, Unigene27513\_JingChaoZuZhi2, Unigene2757\_JingChaoZuZhi2, Unigene27679\_JingChaoZuZhi2, Unigene2790\_JingChaoZuZhi2, Unigene28179\_JingChaoZuZhi2, Unigene291\_JingChaoZuZhi2, Unigene29724\_JingChaoZuZhi2, Unigene32443\_JingChaoZuZhi2, Unigene33141\_JingChaoZuZhi2, Unigene3321\_JingChaoZuZhi2, Unigene34049\_JingChaoZuZhi2, Unigene34660\_JingChaoZuZhi2, Unigene346\_JingChaoZuZhi2, Unigene34723\_JingChaoZuZhi2, Unigene34853\_JingChaoZuZhi2, Unigene3671\_JingChaoZuZhi2, Unigene36916\_JingChaoZuZhi2, Unigene3693\_JingChaoZuZhi2, Unigene37958\_JingChaoZuZhi2, Unigene3814\_JingChaoZuZhi2, Unigene38543\_JingChaoZuZhi2, Unigene3981\_JingChaoZuZhi2, Unigene40410\_JingChaoZuZhi2, Unigene4119\_JingChaoZuZhi2, Unigene4186\_JingChaoZuZhi2, Unigene4295\_JingChaoZuZhi2, Unigene43128\_JingChaoZuZhi2, Unigene43145\_JingChaoZuZhi2, Unigene43277\_JingChaoZuZhi2, Unigene43694\_JingChaoZuZhi2, Unigene43830\_JingChaoZuZhi2, Unigene44141\_JingChaoZuZhi2, Unigene44918\_JingChaoZuZhi2, Unigene44927\_JingChaoZuZhi2, Unigene4531\_JingChaoZuZhi2, Unigene47578\_JingChaoZuZhi2, Unigene47688\_JingChaoZuZhi2, Unigene48236\_JingChaoZuZhi2, Unigene48357\_JingChaoZuZhi2, Unigene4838\_JingChaoZuZhi2, Unigene48558\_JingChaoZuZhi2, Unigene48674\_JingChaoZuZhi2, Unigene48927\_JingChaoZuZhi2, Unigene4909\_JingChaoZuZhi2, Unigene49798\_JingChaoZuZhi2, Unigene50668\_JingChaoZuZhi2, Unigene51612\_JingChaoZuZhi2, Unigene516\_JingChaoZuZhi2, Unigene5176\_JingChaoZuZhi2, Unigene5234\_JingChaoZuZhi2, Unigene52381\_JingChaoZuZhi2, Unigene52534\_JingChaoZuZhi2, Unigene5290\_JingChaoZuZhi2, Unigene53020\_JingChaoZuZhi2, Unigene53029\_JingChaoZuZhi2, Unigene54470\_JingChaoZuZhi2, Unigene54967\_JingChaoZuZhi2, Unigene5559\_JingChaoZuZhi2, Unigene5607\_JingChaoZuZhi2, Unigene56660\_JingChaoZuZhi2, Unigene56897\_JingChaoZuZhi2, Unigene5771\_JingChaoZuZhi2, Unigene57853\_JingChaoZuZhi2, Unigene59336\_JingChaoZuZhi2, Unigene59374\_JingChaoZuZhi2, Unigene59849\_JingChaoZuZhi2, Unigene60082\_JingChaoZuZhi2, Unigene6028\_JingChaoZuZhi2, Unigene60586\_JingChaoZuZhi2, Unigene60664\_JingChaoZuZhi2, Unigene60762\_JingChaoZuZhi2, Unigene61660\_JingChaoZuZhi2, Unigene61876\_JingChaoZuZhi2, Unigene62647\_JingChaoZuZhi2, Unigene62786\_JingChaoZuZhi2, Unigene62851\_JingChaoZuZhi2, Unigene63079\_JingChaoZuZhi2, Unigene6391\_JingChaoZuZhi2, Unigene6424\_JingChaoZuZhi2, Unigene64671\_JingChaoZuZhi2, Unigene6493\_JingChaoZuZhi2, Unigene64989\_JingChaoZuZhi2, Unigene65086\_JingChaoZuZhi2, Unigene65375\_JingChaoZuZhi2, Unigene6539\_JingChaoZuZhi2, Unigene65910\_JingChaoZuZhi2, Unigene66271\_JingChaoZuZhi2, Unigene66888\_JingChaoZuZhi2, Unigene67047\_JingChaoZuZhi2, Unigene67063\_JingChaoZuZhi2, Unigene67177\_JingChaoZuZhi2, Unigene67364\_JingChaoZuZhi2, Unigene67393\_JingChaoZuZhi2, Unigene67523\_JingChaoZuZhi2, Unigene67793\_JingChaoZuZhi2, Unigene68814\_JingChaoZuZhi2, Unigene6914\_JingChaoZuZhi2, Unigene69657\_JingChaoZuZhi2, Unigene69678\_JingChaoZuZhi2, Unigene70069\_JingChaoZuZhi2, Unigene70270\_JingChaoZuZhi2, Unigene70549\_JingChaoZuZhi2, Unigene70740\_JingChaoZuZhi2, Unigene71130\_JingChaoZuZhi2, Unigene71213\_JingChaoZuZhi2, Unigene72040\_JingChaoZuZhi2, Unigene72521\_JingChaoZuZhi2, Unigene72860\_JingChaoZuZhi2, Unigene73063\_JingChaoZuZhi2, Unigene73117\_JingChaoZuZhi2, Unigene73445\_JingChaoZuZhi2, Unigene74050\_JingChaoZuZhi2, Unigene7412\_JingChaoZuZhi2, Unigene741\_JingChaoZuZhi2, Unigene74474\_JingChaoZuZhi2, Unigene74559\_JingChaoZuZhi2, Unigene74592\_JingChaoZuZhi2, Unigene75668\_JingChaoZuZhi2, Unigene7567\_JingChaoZuZhi2, Unigene75830\_JingChaoZuZhi2, Unigene75902\_JingChaoZuZhi2, Unigene75920\_JingChaoZuZhi2, Unigene75975\_JingChaoZuZhi2, Unigene76069\_JingChaoZuZhi2, Unigene76179\_JingChaoZuZhi2, Unigene7624\_JingChaoZuZhi2, Unigene77000\_JingChaoZuZhi2, Unigene77424\_JingChaoZuZhi2, Unigene77803\_JingChaoZuZhi2, Unigene77871\_JingChaoZuZhi2, Unigene78084\_JingChaoZuZhi2, Unigene78136\_JingChaoZuZhi2, Unigene78203\_JingChaoZuZhi2, Unigene7824\_JingChaoZuZhi2, Unigene78252\_JingChaoZuZhi2, Unigene78574\_JingChaoZuZhi2, Unigene7930\_JingChaoZuZhi2, Unigene80234\_JingChaoZuZhi2, Unigene80385\_JingChaoZuZhi2, Unigene80702\_JingChaoZuZhi2, Unigene80772\_JingChaoZuZhi2, Unigene8113\_JingChaoZuZhi2, Unigene81253\_JingChaoZuZhi2, Unigene81553\_JingChaoZuZhi2, Unigene81687\_JingChaoZuZhi2, Unigene81712\_JingChaoZuZhi2, Unigene81716\_JingChaoZuZhi2, Unigene8185\_JingChaoZuZhi2, Unigene8191\_JingChaoZuZhi2, Unigene82112\_JingChaoZuZhi2, Unigene8230\_JingChaoZuZhi2, Unigene82318\_JingChaoZuZhi2, Unigene82517\_JingChaoZuZhi2, Unigene82606\_JingChaoZuZhi2, Unigene83303\_JingChaoZuZhi2, Unigene8350\_JingChaoZuZhi2, Unigene83960\_JingChaoZuZhi2, Unigene83996\_JingChaoZuZhi2, Unigene841\_JingChaoZuZhi2, Unigene84220\_JingChaoZuZhi2, Unigene84399\_JingChaoZuZhi2, Unigene84475\_JingChaoZuZhi2, Unigene84564\_JingChaoZuZhi2, Unigene84608\_JingChaoZuZhi2, Unigene84660\_JingChaoZuZhi2, Unigene84820\_JingChaoZuZhi2, Unigene84988\_JingChaoZuZhi2, Unigene85222\_JingChaoZuZhi2, Unigene85574\_JingChaoZuZhi2, Unigene85578\_JingChaoZuZhi2, Unigene85976\_JingChaoZuZhi2, Unigene86115\_JingChaoZuZhi2, Unigene86579\_JingChaoZuZhi2, Unigene8709\_JingChaoZuZhi2, Unigene8711\_JingChaoZuZhi2, Unigene87121\_JingChaoZuZhi2, Unigene87168\_JingChaoZuZhi2, Unigene87213\_JingChaoZuZhi2, Unigene87239\_JingChaoZuZhi2, Unigene8726\_JingChaoZuZhi2, Unigene87640\_JingChaoZuZhi2, Unigene876\_JingChaoZuZhi2, Unigene87853\_JingChaoZuZhi2, Unigene87963\_JingChaoZuZhi2, Unigene88092\_JingChaoZuZhi2, Unigene88231\_JingChaoZuZhi2, Unigene88382\_JingChaoZuZhi2, Unigene88584\_JingChaoZuZhi2, Unigene88686\_JingChaoZuZhi2, Unigene88731\_JingChaoZuZhi2, Unigene88908\_JingChaoZuZhi2, Unigene88930\_JingChaoZuZhi2, Unigene88945\_JingChaoZuZhi2, Unigene89013\_JingChaoZuZhi2, Unigene89120\_JingChaoZuZhi2, Unigene89337\_JingChaoZuZhi2, Unigene89829\_JingChaoZuZhi2, Unigene89942\_JingChaoZuZhi2, Unigene90124\_JingChaoZuZhi2, Unigene90209\_JingChaoZuZhi2, Unigene90226\_JingChaoZuZhi2, Unigene90265\_JingChaoZuZhi2, Unigene90389\_JingChaoZuZhi2, Unigene9049\_JingChaoZuZhi2, Unigene90666\_JingChaoZuZhi2, Unigene90716\_JingChaoZuZhi2, Unigene91028\_JingChaoZuZhi2, Unigene91284\_JingChaoZuZhi2, Unigene91442\_JingChaoZuZhi2, Unigene91606\_JingChaoZuZhi2, Unigene91612\_JingChaoZuZhi2, Unigene91715\_JingChaoZuZhi2, Unigene91958\_JingChaoZuZhi2, Unigene92091\_JingChaoZuZhi2, Unigene92203\_JingChaoZuZhi2, Unigene9220\_JingChaoZuZhi2, Unigene92492\_JingChaoZuZhi2, Unigene92502\_JingChaoZuZhi2, Unigene92671\_JingChaoZuZhi2, Unigene92832\_JingChaoZuZhi2, Unigene92948\_JingChaoZuZhi2, Unigene93014\_JingChaoZuZhi2, Unigene93056\_JingChaoZuZhi2, Unigene93073\_JingChaoZuZhi2, Unigene93271\_JingChaoZuZhi2, Unigene93312\_JingChaoZuZhi2, Unigene93341\_JingChaoZuZhi2, Unigene93419\_JingChaoZuZhi2, Unigene93653\_JingChaoZuZhi2, Unigene93679\_JingChaoZuZhi2, Unigene93768\_JingChaoZuZhi2, Unigene93780\_JingChaoZuZhi2, Unigene93921\_JingChaoZuZhi2, Unigene93956\_JingChaoZuZhi2, Unigene93982\_JingChaoZuZhi2, Unigene93987\_JingChaoZuZhi2, Unigene9412\_JingChaoZuZhi2, Unigene94149\_JingChaoZuZhi2, Unigene94333\_JingChaoZuZhi2, Unigene94456\_JingChaoZuZhi2, Unigene94751\_JingChaoZuZhi2, Unigene94855\_JingChaoZuZhi2, Unigene94878\_JingChaoZuZhi2, Unigene94939\_JingChaoZuZhi2, Unigene95101\_JingChaoZuZhi2, Unigene9511\_JingChaoZuZhi2, Unigene95281\_JingChaoZuZhi2, Unigene95299\_JingChaoZuZhi2, Unigene95310\_JingChaoZuZhi2, Unigene95378\_JingChaoZuZhi2, Unigene95456\_JingChaoZuZhi2, Unigene95518\_JingChaoZuZhi2, Unigene95602\_JingChaoZuZhi2, Unigene95621\_JingChaoZuZhi2, Unigene95750\_JingChaoZuZhi2, Unigene95895\_JingChaoZuZhi2, Unigene95923\_JingChaoZuZhi2, Unigene96044\_JingChaoZuZhi2, Unigene96125\_JingChaoZuZhi2, Unigene9637\_JingChaoZuZhi2, Unigene9675\_JingChaoZuZhi2, Unigene9688\_JingChaoZuZhi2, Unigene9879\_JingChaoZuZhi2, Unigene9902\_JingChaoZuZhi2, Unigene9924\_JingChaoZuZhi2 |
| 24 | Dilated cardiomyopathy | Unigene10027\_JingChaoZuZhi2, Unigene10034\_JingChaoZuZhi2, Unigene10047\_JingChaoZuZhi2, Unigene10259\_JingChaoZuZhi2, Unigene10294\_JingChaoZuZhi2, Unigene10350\_JingChaoZuZhi2, Unigene1060\_JingChaoZuZhi2, Unigene10628\_JingChaoZuZhi2, Unigene10765\_JingChaoZuZhi2, Unigene10994\_JingChaoZuZhi2, Unigene10\_JingChaoZuZhi2, Unigene11112\_JingChaoZuZhi2, Unigene1111\_JingChaoZuZhi2, Unigene11446\_JingChaoZuZhi2, Unigene11475\_JingChaoZuZhi2, Unigene1162\_JingChaoZuZhi2, Unigene11726\_JingChaoZuZhi2, Unigene12091\_JingChaoZuZhi2, Unigene12183\_JingChaoZuZhi2, Unigene12316\_JingChaoZuZhi2, Unigene12329\_JingChaoZuZhi2, Unigene12889\_JingChaoZuZhi2, Unigene12938\_JingChaoZuZhi2, Unigene13395\_JingChaoZuZhi2, Unigene13607\_JingChaoZuZhi2, Unigene13770\_JingChaoZuZhi2, Unigene13854\_JingChaoZuZhi2, Unigene14136\_JingChaoZuZhi2, Unigene14234\_JingChaoZuZhi2, Unigene14567\_JingChaoZuZhi2, Unigene14707\_JingChaoZuZhi2, Unigene15030\_JingChaoZuZhi2, Unigene15054\_JingChaoZuZhi2, Unigene15333\_JingChaoZuZhi2, Unigene15345\_JingChaoZuZhi2, Unigene15649\_JingChaoZuZhi2, Unigene15651\_JingChaoZuZhi2, Unigene15937\_JingChaoZuZhi2, Unigene15986\_JingChaoZuZhi2, Unigene16004\_JingChaoZuZhi2, Unigene16121\_JingChaoZuZhi2, Unigene16427\_JingChaoZuZhi2, Unigene16480\_JingChaoZuZhi2, Unigene16597\_JingChaoZuZhi2, Unigene16873\_JingChaoZuZhi2, Unigene17128\_JingChaoZuZhi2, Unigene17132\_JingChaoZuZhi2, Unigene17443\_JingChaoZuZhi2, Unigene17592\_JingChaoZuZhi2, Unigene18051\_JingChaoZuZhi2, Unigene18184\_JingChaoZuZhi2, Unigene18260\_JingChaoZuZhi2, Unigene18307\_JingChaoZuZhi2, Unigene18438\_JingChaoZuZhi2, Unigene18462\_JingChaoZuZhi2, Unigene18491\_JingChaoZuZhi2, Unigene18545\_JingChaoZuZhi2, Unigene18643\_JingChaoZuZhi2, Unigene18699\_JingChaoZuZhi2, Unigene18779\_JingChaoZuZhi2, Unigene18790\_JingChaoZuZhi2, Unigene187\_JingChaoZuZhi2, Unigene18808\_JingChaoZuZhi2, Unigene18865\_JingChaoZuZhi2, Unigene18892\_JingChaoZuZhi2, Unigene19404\_JingChaoZuZhi2, Unigene19559\_JingChaoZuZhi2, Unigene20000\_JingChaoZuZhi2, Unigene20159\_JingChaoZuZhi2, Unigene20223\_JingChaoZuZhi2, Unigene20255\_JingChaoZuZhi2, Unigene20289\_JingChaoZuZhi2, Unigene20349\_JingChaoZuZhi2, Unigene20464\_JingChaoZuZhi2, Unigene20479\_JingChaoZuZhi2, Unigene20580\_JingChaoZuZhi2, Unigene20629\_JingChaoZuZhi2, Unigene20890\_JingChaoZuZhi2, Unigene20892\_JingChaoZuZhi2, Unigene21225\_JingChaoZuZhi2, Unigene21354\_JingChaoZuZhi2, Unigene21398\_JingChaoZuZhi2, Unigene21487\_JingChaoZuZhi2, Unigene21815\_JingChaoZuZhi2, Unigene21818\_JingChaoZuZhi2, Unigene21905\_JingChaoZuZhi2, Unigene21956\_JingChaoZuZhi2, Unigene22178\_JingChaoZuZhi2, Unigene22183\_JingChaoZuZhi2, Unigene22189\_JingChaoZuZhi2, Unigene22216\_JingChaoZuZhi2, Unigene22243\_JingChaoZuZhi2, Unigene22301\_JingChaoZuZhi2, Unigene22323\_JingChaoZuZhi2, Unigene22440\_JingChaoZuZhi2, Unigene22445\_JingChaoZuZhi2, Unigene22487\_JingChaoZuZhi2, Unigene22551\_JingChaoZuZhi2, Unigene22891\_JingChaoZuZhi2, Unigene22895\_JingChaoZuZhi2, Unigene2305\_JingChaoZuZhi2, Unigene24645\_JingChaoZuZhi2, Unigene24754\_JingChaoZuZhi2, Unigene24772\_JingChaoZuZhi2, Unigene25164\_JingChaoZuZhi2, Unigene25759\_JingChaoZuZhi2, Unigene26679\_JingChaoZuZhi2, Unigene26987\_JingChaoZuZhi2, Unigene2770\_JingChaoZuZhi2, Unigene27962\_JingChaoZuZhi2, Unigene28208\_JingChaoZuZhi2, Unigene2823\_JingChaoZuZhi2, Unigene28467\_JingChaoZuZhi2, Unigene29312\_JingChaoZuZhi2, Unigene2968\_JingChaoZuZhi2, Unigene30852\_JingChaoZuZhi2, Unigene3085\_JingChaoZuZhi2, Unigene3122\_JingChaoZuZhi2, Unigene31918\_JingChaoZuZhi2, Unigene3191\_JingChaoZuZhi2, Unigene31982\_JingChaoZuZhi2, Unigene32314\_JingChaoZuZhi2, Unigene35016\_JingChaoZuZhi2, Unigene35243\_JingChaoZuZhi2, Unigene35304\_JingChaoZuZhi2, Unigene35624\_JingChaoZuZhi2, Unigene35952\_JingChaoZuZhi2, Unigene36404\_JingChaoZuZhi2, Unigene37675\_JingChaoZuZhi2, Unigene37739\_JingChaoZuZhi2, Unigene39049\_JingChaoZuZhi2, Unigene39102\_JingChaoZuZhi2, Unigene3912\_JingChaoZuZhi2, Unigene39316\_JingChaoZuZhi2, Unigene39674\_JingChaoZuZhi2, Unigene39832\_JingChaoZuZhi2, Unigene40335\_JingChaoZuZhi2, Unigene40919\_JingChaoZuZhi2, Unigene41842\_JingChaoZuZhi2, Unigene42248\_JingChaoZuZhi2, Unigene4224\_JingChaoZuZhi2, Unigene4316\_JingChaoZuZhi2, Unigene44066\_JingChaoZuZhi2, Unigene44190\_JingChaoZuZhi2, Unigene44731\_JingChaoZuZhi2, Unigene4522\_JingChaoZuZhi2, Unigene45399\_JingChaoZuZhi2, Unigene45468\_JingChaoZuZhi2, Unigene45782\_JingChaoZuZhi2, Unigene46160\_JingChaoZuZhi2, Unigene46256\_JingChaoZuZhi2, Unigene47199\_JingChaoZuZhi2, Unigene4782\_JingChaoZuZhi2, Unigene47926\_JingChaoZuZhi2, Unigene48309\_JingChaoZuZhi2, Unigene48576\_JingChaoZuZhi2, Unigene4878\_JingChaoZuZhi2, Unigene48843\_JingChaoZuZhi2, Unigene49203\_JingChaoZuZhi2, Unigene49526\_JingChaoZuZhi2, Unigene49821\_JingChaoZuZhi2, Unigene499\_JingChaoZuZhi2, Unigene50563\_JingChaoZuZhi2, Unigene50645\_JingChaoZuZhi2, Unigene50673\_JingChaoZuZhi2, Unigene5069\_JingChaoZuZhi2, Unigene5072\_JingChaoZuZhi2, Unigene51228\_JingChaoZuZhi2, Unigene51467\_JingChaoZuZhi2, Unigene5173\_JingChaoZuZhi2, Unigene51991\_JingChaoZuZhi2, Unigene52073\_JingChaoZuZhi2, Unigene52167\_JingChaoZuZhi2, Unigene53160\_JingChaoZuZhi2, Unigene53935\_JingChaoZuZhi2, Unigene54012\_JingChaoZuZhi2, Unigene54980\_JingChaoZuZhi2, Unigene55684\_JingChaoZuZhi2, Unigene56114\_JingChaoZuZhi2, Unigene56788\_JingChaoZuZhi2, Unigene57185\_JingChaoZuZhi2, Unigene57200\_JingChaoZuZhi2, Unigene57751\_JingChaoZuZhi2, Unigene5796\_JingChaoZuZhi2, Unigene58029\_JingChaoZuZhi2, Unigene59102\_JingChaoZuZhi2, Unigene59146\_JingChaoZuZhi2, Unigene5989\_JingChaoZuZhi2, Unigene60401\_JingChaoZuZhi2, Unigene61244\_JingChaoZuZhi2, Unigene61526\_JingChaoZuZhi2, Unigene6174\_JingChaoZuZhi2, Unigene61801\_JingChaoZuZhi2, Unigene61925\_JingChaoZuZhi2, Unigene6239\_JingChaoZuZhi2, Unigene62511\_JingChaoZuZhi2, Unigene62776\_JingChaoZuZhi2, Unigene63040\_JingChaoZuZhi2, Unigene6361\_JingChaoZuZhi2, Unigene63938\_JingChaoZuZhi2, Unigene6393\_JingChaoZuZhi2, Unigene64750\_JingChaoZuZhi2, Unigene6483\_JingChaoZuZhi2, Unigene65002\_JingChaoZuZhi2, Unigene65648\_JingChaoZuZhi2, Unigene66073\_JingChaoZuZhi2, Unigene66082\_JingChaoZuZhi2, Unigene6634\_JingChaoZuZhi2, Unigene6699\_JingChaoZuZhi2, Unigene67542\_JingChaoZuZhi2, Unigene6769\_JingChaoZuZhi2, Unigene68302\_JingChaoZuZhi2, Unigene68368\_JingChaoZuZhi2, Unigene6864\_JingChaoZuZhi2, Unigene69005\_JingChaoZuZhi2, Unigene69100\_JingChaoZuZhi2, Unigene69673\_JingChaoZuZhi2, Unigene70373\_JingChaoZuZhi2, Unigene71017\_JingChaoZuZhi2, Unigene71047\_JingChaoZuZhi2, Unigene71247\_JingChaoZuZhi2, Unigene71365\_JingChaoZuZhi2, Unigene71414\_JingChaoZuZhi2, Unigene71592\_JingChaoZuZhi2, Unigene71640\_JingChaoZuZhi2, Unigene72249\_JingChaoZuZhi2, Unigene72826\_JingChaoZuZhi2, Unigene73554\_JingChaoZuZhi2, Unigene73571\_JingChaoZuZhi2, Unigene73579\_JingChaoZuZhi2, Unigene7364\_JingChaoZuZhi2, Unigene7456\_JingChaoZuZhi2, Unigene75045\_JingChaoZuZhi2, Unigene75235\_JingChaoZuZhi2, Unigene75641\_JingChaoZuZhi2, Unigene75710\_JingChaoZuZhi2, Unigene75840\_JingChaoZuZhi2, Unigene75969\_JingChaoZuZhi2, Unigene76303\_JingChaoZuZhi2, Unigene76803\_JingChaoZuZhi2, Unigene77071\_JingChaoZuZhi2, Unigene77074\_JingChaoZuZhi2, Unigene77406\_JingChaoZuZhi2, Unigene77549\_JingChaoZuZhi2, Unigene77738\_JingChaoZuZhi2, Unigene77965\_JingChaoZuZhi2, Unigene78235\_JingChaoZuZhi2, Unigene78249\_JingChaoZuZhi2, Unigene78916\_JingChaoZuZhi2, Unigene79109\_JingChaoZuZhi2, Unigene79274\_JingChaoZuZhi2, Unigene79568\_JingChaoZuZhi2, Unigene80099\_JingChaoZuZhi2, Unigene8014\_JingChaoZuZhi2, Unigene80215\_JingChaoZuZhi2, Unigene80494\_JingChaoZuZhi2, Unigene80605\_JingChaoZuZhi2, Unigene80863\_JingChaoZuZhi2, Unigene81395\_JingChaoZuZhi2, Unigene81598\_JingChaoZuZhi2, Unigene82117\_JingChaoZuZhi2, Unigene82489\_JingChaoZuZhi2, Unigene8261\_JingChaoZuZhi2, Unigene82842\_JingChaoZuZhi2, Unigene83400\_JingChaoZuZhi2, Unigene83487\_JingChaoZuZhi2, Unigene83606\_JingChaoZuZhi2, Unigene83744\_JingChaoZuZhi2, Unigene83872\_JingChaoZuZhi2, Unigene84359\_JingChaoZuZhi2, Unigene84510\_JingChaoZuZhi2, Unigene84519\_JingChaoZuZhi2, Unigene85122\_JingChaoZuZhi2, Unigene85132\_JingChaoZuZhi2, Unigene85193\_JingChaoZuZhi2, Unigene85387\_JingChaoZuZhi2, Unigene85658\_JingChaoZuZhi2, Unigene86051\_JingChaoZuZhi2, Unigene86312\_JingChaoZuZhi2, Unigene86519\_JingChaoZuZhi2, Unigene86616\_JingChaoZuZhi2, Unigene866\_JingChaoZuZhi2, Unigene86759\_JingChaoZuZhi2, Unigene8679\_JingChaoZuZhi2, Unigene87122\_JingChaoZuZhi2, Unigene87360\_JingChaoZuZhi2, Unigene87677\_JingChaoZuZhi2, Unigene87825\_JingChaoZuZhi2, Unigene87958\_JingChaoZuZhi2, Unigene88040\_JingChaoZuZhi2, Unigene88342\_JingChaoZuZhi2, Unigene88547\_JingChaoZuZhi2, Unigene88925\_JingChaoZuZhi2, Unigene89060\_JingChaoZuZhi2, Unigene89090\_JingChaoZuZhi2, Unigene89175\_JingChaoZuZhi2, Unigene89329\_JingChaoZuZhi2, Unigene89409\_JingChaoZuZhi2, Unigene89536\_JingChaoZuZhi2, Unigene89603\_JingChaoZuZhi2, Unigene89807\_JingChaoZuZhi2, Unigene89959\_JingChaoZuZhi2, Unigene90179\_JingChaoZuZhi2, Unigene90401\_JingChaoZuZhi2, Unigene90551\_JingChaoZuZhi2, Unigene90588\_JingChaoZuZhi2, Unigene90626\_JingChaoZuZhi2, Unigene90767\_JingChaoZuZhi2, Unigene91030\_JingChaoZuZhi2, Unigene91043\_JingChaoZuZhi2, Unigene91163\_JingChaoZuZhi2, Unigene91358\_JingChaoZuZhi2, Unigene91362\_JingChaoZuZhi2, Unigene91527\_JingChaoZuZhi2, Unigene91631\_JingChaoZuZhi2, Unigene91991\_JingChaoZuZhi2, Unigene92057\_JingChaoZuZhi2, Unigene92104\_JingChaoZuZhi2, Unigene92141\_JingChaoZuZhi2, Unigene92329\_JingChaoZuZhi2, Unigene92448\_JingChaoZuZhi2, Unigene92471\_JingChaoZuZhi2, Unigene92577\_JingChaoZuZhi2, Unigene92671\_JingChaoZuZhi2, Unigene92673\_JingChaoZuZhi2, Unigene92697\_JingChaoZuZhi2, Unigene92769\_JingChaoZuZhi2, Unigene93010\_JingChaoZuZhi2, Unigene93279\_JingChaoZuZhi2, Unigene93374\_JingChaoZuZhi2, Unigene93703\_JingChaoZuZhi2, Unigene93865\_JingChaoZuZhi2, Unigene93990\_JingChaoZuZhi2, Unigene94110\_JingChaoZuZhi2, Unigene94113\_JingChaoZuZhi2, Unigene94156\_JingChaoZuZhi2, Unigene94214\_JingChaoZuZhi2, Unigene94337\_JingChaoZuZhi2, Unigene944\_JingChaoZuZhi2, Unigene94561\_JingChaoZuZhi2, Unigene94641\_JingChaoZuZhi2, Unigene94723\_JingChaoZuZhi2, Unigene94823\_JingChaoZuZhi2, Unigene95031\_JingChaoZuZhi2, Unigene95059\_JingChaoZuZhi2, Unigene951\_JingChaoZuZhi2, Unigene95213\_JingChaoZuZhi2, Unigene95243\_JingChaoZuZhi2, Unigene95254\_JingChaoZuZhi2, Unigene95385\_JingChaoZuZhi2, Unigene95438\_JingChaoZuZhi2, Unigene95486\_JingChaoZuZhi2, Unigene95580\_JingChaoZuZhi2, Unigene95799\_JingChaoZuZhi2, Unigene95879\_JingChaoZuZhi2, Unigene95882\_JingChaoZuZhi2, Unigene95889\_JingChaoZuZhi2, Unigene95907\_JingChaoZuZhi2, Unigene95916\_JingChaoZuZhi2, Unigene95954\_JingChaoZuZhi2, Unigene95972\_JingChaoZuZhi2, Unigene95982\_JingChaoZuZhi2, Unigene95987\_JingChaoZuZhi2, Unigene95994\_JingChaoZuZhi2, Unigene96012\_JingChaoZuZhi2, Unigene96033\_JingChaoZuZhi2, Unigene96061\_JingChaoZuZhi2, Unigene96083\_JingChaoZuZhi2, Unigene96091\_JingChaoZuZhi2, Unigene96097\_JingChaoZuZhi2, Unigene96121\_JingChaoZuZhi2, Unigene96217\_JingChaoZuZhi2, Unigene96243\_JingChaoZuZhi2, Unigene96247\_JingChaoZuZhi2, Unigene96288\_JingChaoZuZhi2, Unigene96292\_JingChaoZuZhi2, Unigene96303\_JingChaoZuZhi2, Unigene96308\_JingChaoZuZhi2, Unigene9739\_JingChaoZuZhi2 |
| 25 | Hypertrophic cardiomyopathy (HCM) | Unigene10027\_JingChaoZuZhi2, Unigene10034\_JingChaoZuZhi2, Unigene10047\_JingChaoZuZhi2, Unigene10259\_JingChaoZuZhi2, Unigene10294\_JingChaoZuZhi2, Unigene10350\_JingChaoZuZhi2, Unigene1060\_JingChaoZuZhi2, Unigene10628\_JingChaoZuZhi2, Unigene10765\_JingChaoZuZhi2, Unigene10994\_JingChaoZuZhi2, Unigene10\_JingChaoZuZhi2, Unigene11112\_JingChaoZuZhi2, Unigene1111\_JingChaoZuZhi2, Unigene11446\_JingChaoZuZhi2, Unigene11475\_JingChaoZuZhi2, Unigene1162\_JingChaoZuZhi2, Unigene12091\_JingChaoZuZhi2, Unigene12183\_JingChaoZuZhi2, Unigene12316\_JingChaoZuZhi2, Unigene12329\_JingChaoZuZhi2, Unigene12889\_JingChaoZuZhi2, Unigene13008\_JingChaoZuZhi2, Unigene13395\_JingChaoZuZhi2, Unigene13607\_JingChaoZuZhi2, Unigene13854\_JingChaoZuZhi2, Unigene14136\_JingChaoZuZhi2, Unigene14234\_JingChaoZuZhi2, Unigene14707\_JingChaoZuZhi2, Unigene14877\_JingChaoZuZhi2, Unigene15030\_JingChaoZuZhi2, Unigene15054\_JingChaoZuZhi2, Unigene15333\_JingChaoZuZhi2, Unigene15345\_JingChaoZuZhi2, Unigene15649\_JingChaoZuZhi2, Unigene15937\_JingChaoZuZhi2, Unigene15986\_JingChaoZuZhi2, Unigene16004\_JingChaoZuZhi2, Unigene16121\_JingChaoZuZhi2, Unigene16427\_JingChaoZuZhi2, Unigene16480\_JingChaoZuZhi2, Unigene16597\_JingChaoZuZhi2, Unigene16764\_JingChaoZuZhi2, Unigene16873\_JingChaoZuZhi2, Unigene17128\_JingChaoZuZhi2, Unigene17132\_JingChaoZuZhi2, Unigene17443\_JingChaoZuZhi2, Unigene17592\_JingChaoZuZhi2, Unigene18051\_JingChaoZuZhi2, Unigene18184\_JingChaoZuZhi2, Unigene18260\_JingChaoZuZhi2, Unigene18438\_JingChaoZuZhi2, Unigene18462\_JingChaoZuZhi2, Unigene18491\_JingChaoZuZhi2, Unigene18545\_JingChaoZuZhi2, Unigene18643\_JingChaoZuZhi2, Unigene18699\_JingChaoZuZhi2, Unigene18729\_JingChaoZuZhi2, Unigene18779\_JingChaoZuZhi2, Unigene18790\_JingChaoZuZhi2, Unigene187\_JingChaoZuZhi2, Unigene18808\_JingChaoZuZhi2, Unigene18865\_JingChaoZuZhi2, Unigene18892\_JingChaoZuZhi2, Unigene19404\_JingChaoZuZhi2, Unigene19559\_JingChaoZuZhi2, Unigene20000\_JingChaoZuZhi2, Unigene20159\_JingChaoZuZhi2, Unigene20223\_JingChaoZuZhi2, Unigene20255\_JingChaoZuZhi2, Unigene20289\_JingChaoZuZhi2, Unigene20349\_JingChaoZuZhi2, Unigene20464\_JingChaoZuZhi2, Unigene20479\_JingChaoZuZhi2, Unigene20580\_JingChaoZuZhi2, Unigene20629\_JingChaoZuZhi2, Unigene20890\_JingChaoZuZhi2, Unigene20892\_JingChaoZuZhi2, Unigene21132\_JingChaoZuZhi2, Unigene21225\_JingChaoZuZhi2, Unigene21354\_JingChaoZuZhi2, Unigene21398\_JingChaoZuZhi2, Unigene21487\_JingChaoZuZhi2, Unigene21818\_JingChaoZuZhi2, Unigene21905\_JingChaoZuZhi2, Unigene21956\_JingChaoZuZhi2, Unigene22178\_JingChaoZuZhi2, Unigene22183\_JingChaoZuZhi2, Unigene22189\_JingChaoZuZhi2, Unigene22216\_JingChaoZuZhi2, Unigene22243\_JingChaoZuZhi2, Unigene22293\_JingChaoZuZhi2, Unigene22301\_JingChaoZuZhi2, Unigene22323\_JingChaoZuZhi2, Unigene22440\_JingChaoZuZhi2, Unigene22445\_JingChaoZuZhi2, Unigene22487\_JingChaoZuZhi2, Unigene22551\_JingChaoZuZhi2, Unigene22891\_JingChaoZuZhi2, Unigene22895\_JingChaoZuZhi2, Unigene2305\_JingChaoZuZhi2, Unigene24645\_JingChaoZuZhi2, Unigene24754\_JingChaoZuZhi2, Unigene24772\_JingChaoZuZhi2, Unigene25164\_JingChaoZuZhi2, Unigene25759\_JingChaoZuZhi2, Unigene26679\_JingChaoZuZhi2, Unigene26987\_JingChaoZuZhi2, Unigene2770\_JingChaoZuZhi2, Unigene27962\_JingChaoZuZhi2, Unigene28208\_JingChaoZuZhi2, Unigene2823\_JingChaoZuZhi2, Unigene28467\_JingChaoZuZhi2, Unigene29179\_JingChaoZuZhi2, Unigene29312\_JingChaoZuZhi2, Unigene2968\_JingChaoZuZhi2, Unigene30852\_JingChaoZuZhi2, Unigene3085\_JingChaoZuZhi2, Unigene31918\_JingChaoZuZhi2, Unigene3191\_JingChaoZuZhi2, Unigene31982\_JingChaoZuZhi2, Unigene32314\_JingChaoZuZhi2, Unigene35016\_JingChaoZuZhi2, Unigene35243\_JingChaoZuZhi2, Unigene35304\_JingChaoZuZhi2, Unigene35574\_JingChaoZuZhi2, Unigene35624\_JingChaoZuZhi2, Unigene35952\_JingChaoZuZhi2, Unigene36404\_JingChaoZuZhi2, Unigene37675\_JingChaoZuZhi2, Unigene37739\_JingChaoZuZhi2, Unigene39049\_JingChaoZuZhi2, Unigene39102\_JingChaoZuZhi2, Unigene3912\_JingChaoZuZhi2, Unigene39316\_JingChaoZuZhi2, Unigene39674\_JingChaoZuZhi2, Unigene40919\_JingChaoZuZhi2, Unigene41842\_JingChaoZuZhi2, Unigene42248\_JingChaoZuZhi2, Unigene4224\_JingChaoZuZhi2, Unigene4316\_JingChaoZuZhi2, Unigene44066\_JingChaoZuZhi2, Unigene44190\_JingChaoZuZhi2, Unigene44731\_JingChaoZuZhi2, Unigene4522\_JingChaoZuZhi2, Unigene45399\_JingChaoZuZhi2, Unigene45468\_JingChaoZuZhi2, Unigene45782\_JingChaoZuZhi2, Unigene46160\_JingChaoZuZhi2, Unigene46256\_JingChaoZuZhi2, Unigene47199\_JingChaoZuZhi2, Unigene4782\_JingChaoZuZhi2, Unigene47926\_JingChaoZuZhi2, Unigene48309\_JingChaoZuZhi2, Unigene48576\_JingChaoZuZhi2, Unigene4878\_JingChaoZuZhi2, Unigene48843\_JingChaoZuZhi2, Unigene49526\_JingChaoZuZhi2, Unigene49821\_JingChaoZuZhi2, Unigene499\_JingChaoZuZhi2, Unigene50563\_JingChaoZuZhi2, Unigene50645\_JingChaoZuZhi2, Unigene50673\_JingChaoZuZhi2, Unigene5069\_JingChaoZuZhi2, Unigene5072\_JingChaoZuZhi2, Unigene51228\_JingChaoZuZhi2, Unigene51467\_JingChaoZuZhi2, Unigene5173\_JingChaoZuZhi2, Unigene52073\_JingChaoZuZhi2, Unigene52167\_JingChaoZuZhi2, Unigene52793\_JingChaoZuZhi2, Unigene53160\_JingChaoZuZhi2, Unigene54066\_JingChaoZuZhi2, Unigene54980\_JingChaoZuZhi2, Unigene55684\_JingChaoZuZhi2, Unigene56114\_JingChaoZuZhi2, Unigene56788\_JingChaoZuZhi2, Unigene57200\_JingChaoZuZhi2, Unigene57751\_JingChaoZuZhi2, Unigene5796\_JingChaoZuZhi2, Unigene58029\_JingChaoZuZhi2, Unigene59146\_JingChaoZuZhi2, Unigene5989\_JingChaoZuZhi2, Unigene60401\_JingChaoZuZhi2, Unigene61244\_JingChaoZuZhi2, Unigene61526\_JingChaoZuZhi2, Unigene6174\_JingChaoZuZhi2, Unigene61801\_JingChaoZuZhi2, Unigene61925\_JingChaoZuZhi2, Unigene6239\_JingChaoZuZhi2, Unigene62511\_JingChaoZuZhi2, Unigene62776\_JingChaoZuZhi2, Unigene63040\_JingChaoZuZhi2, Unigene6361\_JingChaoZuZhi2, Unigene63938\_JingChaoZuZhi2, Unigene64750\_JingChaoZuZhi2, Unigene6483\_JingChaoZuZhi2, Unigene65002\_JingChaoZuZhi2, Unigene65447\_JingChaoZuZhi2, Unigene65648\_JingChaoZuZhi2, Unigene66082\_JingChaoZuZhi2, Unigene6634\_JingChaoZuZhi2, Unigene66772\_JingChaoZuZhi2, Unigene6699\_JingChaoZuZhi2, Unigene67542\_JingChaoZuZhi2, Unigene6769\_JingChaoZuZhi2, Unigene68302\_JingChaoZuZhi2, Unigene68368\_JingChaoZuZhi2, Unigene6864\_JingChaoZuZhi2, Unigene69005\_JingChaoZuZhi2, Unigene69100\_JingChaoZuZhi2, Unigene69673\_JingChaoZuZhi2, Unigene70373\_JingChaoZuZhi2, Unigene71017\_JingChaoZuZhi2, Unigene71047\_JingChaoZuZhi2, Unigene71247\_JingChaoZuZhi2, Unigene71414\_JingChaoZuZhi2, Unigene71592\_JingChaoZuZhi2, Unigene71640\_JingChaoZuZhi2, Unigene72249\_JingChaoZuZhi2, Unigene72826\_JingChaoZuZhi2, Unigene73554\_JingChaoZuZhi2, Unigene73571\_JingChaoZuZhi2, Unigene73579\_JingChaoZuZhi2, Unigene7364\_JingChaoZuZhi2, Unigene73882\_JingChaoZuZhi2, Unigene75045\_JingChaoZuZhi2, Unigene75641\_JingChaoZuZhi2, Unigene75710\_JingChaoZuZhi2, Unigene75840\_JingChaoZuZhi2, Unigene75969\_JingChaoZuZhi2, Unigene76303\_JingChaoZuZhi2, Unigene76803\_JingChaoZuZhi2, Unigene77071\_JingChaoZuZhi2, Unigene77074\_JingChaoZuZhi2, Unigene77207\_JingChaoZuZhi2, Unigene77406\_JingChaoZuZhi2, Unigene77549\_JingChaoZuZhi2, Unigene77738\_JingChaoZuZhi2, Unigene77965\_JingChaoZuZhi2, Unigene78249\_JingChaoZuZhi2, Unigene78367\_JingChaoZuZhi2, Unigene78916\_JingChaoZuZhi2, Unigene79109\_JingChaoZuZhi2, Unigene79274\_JingChaoZuZhi2, Unigene79568\_JingChaoZuZhi2, Unigene80099\_JingChaoZuZhi2, Unigene8014\_JingChaoZuZhi2, Unigene80215\_JingChaoZuZhi2, Unigene80494\_JingChaoZuZhi2, Unigene80605\_JingChaoZuZhi2, Unigene80863\_JingChaoZuZhi2, Unigene81395\_JingChaoZuZhi2, Unigene81598\_JingChaoZuZhi2, Unigene82117\_JingChaoZuZhi2, Unigene82489\_JingChaoZuZhi2, Unigene8261\_JingChaoZuZhi2, Unigene82842\_JingChaoZuZhi2, Unigene83400\_JingChaoZuZhi2, Unigene83487\_JingChaoZuZhi2, Unigene83606\_JingChaoZuZhi2, Unigene83744\_JingChaoZuZhi2, Unigene83872\_JingChaoZuZhi2, Unigene84024\_JingChaoZuZhi2, Unigene84359\_JingChaoZuZhi2, Unigene84510\_JingChaoZuZhi2, Unigene84519\_JingChaoZuZhi2, Unigene85122\_JingChaoZuZhi2, Unigene85132\_JingChaoZuZhi2, Unigene85193\_JingChaoZuZhi2, Unigene85387\_JingChaoZuZhi2, Unigene85658\_JingChaoZuZhi2, Unigene86051\_JingChaoZuZhi2, Unigene86312\_JingChaoZuZhi2, Unigene86519\_JingChaoZuZhi2, Unigene86616\_JingChaoZuZhi2, Unigene866\_JingChaoZuZhi2, Unigene86759\_JingChaoZuZhi2, Unigene8679\_JingChaoZuZhi2, Unigene87122\_JingChaoZuZhi2, Unigene87360\_JingChaoZuZhi2, Unigene87677\_JingChaoZuZhi2, Unigene87825\_JingChaoZuZhi2, Unigene8784\_JingChaoZuZhi2, Unigene87958\_JingChaoZuZhi2, Unigene88040\_JingChaoZuZhi2, Unigene88342\_JingChaoZuZhi2, Unigene88547\_JingChaoZuZhi2, Unigene88925\_JingChaoZuZhi2, Unigene89060\_JingChaoZuZhi2, Unigene89090\_JingChaoZuZhi2, Unigene89175\_JingChaoZuZhi2, Unigene89329\_JingChaoZuZhi2, Unigene89409\_JingChaoZuZhi2, Unigene89536\_JingChaoZuZhi2, Unigene89959\_JingChaoZuZhi2, Unigene90179\_JingChaoZuZhi2, Unigene90401\_JingChaoZuZhi2, Unigene90551\_JingChaoZuZhi2, Unigene90588\_JingChaoZuZhi2, Unigene90626\_JingChaoZuZhi2, Unigene90767\_JingChaoZuZhi2, Unigene91030\_JingChaoZuZhi2, Unigene91043\_JingChaoZuZhi2, Unigene91163\_JingChaoZuZhi2, Unigene91358\_JingChaoZuZhi2, Unigene91362\_JingChaoZuZhi2, Unigene91631\_JingChaoZuZhi2, Unigene91816\_JingChaoZuZhi2, Unigene91895\_JingChaoZuZhi2, Unigene91991\_JingChaoZuZhi2, Unigene92057\_JingChaoZuZhi2, Unigene92104\_JingChaoZuZhi2, Unigene92141\_JingChaoZuZhi2, Unigene92329\_JingChaoZuZhi2, Unigene92448\_JingChaoZuZhi2, Unigene92471\_JingChaoZuZhi2, Unigene92577\_JingChaoZuZhi2, Unigene92671\_JingChaoZuZhi2, Unigene92673\_JingChaoZuZhi2, Unigene92697\_JingChaoZuZhi2, Unigene92769\_JingChaoZuZhi2, Unigene93010\_JingChaoZuZhi2, Unigene93279\_JingChaoZuZhi2, Unigene93374\_JingChaoZuZhi2, Unigene93703\_JingChaoZuZhi2, Unigene93774\_JingChaoZuZhi2, Unigene93865\_JingChaoZuZhi2, Unigene93990\_JingChaoZuZhi2, Unigene94110\_JingChaoZuZhi2, Unigene94113\_JingChaoZuZhi2, Unigene94156\_JingChaoZuZhi2, Unigene94178\_JingChaoZuZhi2, Unigene94214\_JingChaoZuZhi2, Unigene94337\_JingChaoZuZhi2, Unigene944\_JingChaoZuZhi2, Unigene94561\_JingChaoZuZhi2, Unigene94641\_JingChaoZuZhi2, Unigene94723\_JingChaoZuZhi2, Unigene94823\_JingChaoZuZhi2, Unigene95031\_JingChaoZuZhi2, Unigene95059\_JingChaoZuZhi2, Unigene951\_JingChaoZuZhi2, Unigene95213\_JingChaoZuZhi2, Unigene95243\_JingChaoZuZhi2, Unigene95254\_JingChaoZuZhi2, Unigene95385\_JingChaoZuZhi2, Unigene95438\_JingChaoZuZhi2, Unigene95486\_JingChaoZuZhi2, Unigene95580\_JingChaoZuZhi2, Unigene95799\_JingChaoZuZhi2, Unigene95879\_JingChaoZuZhi2, Unigene95882\_JingChaoZuZhi2, Unigene95889\_JingChaoZuZhi2, Unigene95907\_JingChaoZuZhi2, Unigene95954\_JingChaoZuZhi2, Unigene95972\_JingChaoZuZhi2, Unigene95982\_JingChaoZuZhi2, Unigene95987\_JingChaoZuZhi2, Unigene95994\_JingChaoZuZhi2, Unigene96012\_JingChaoZuZhi2, Unigene96033\_JingChaoZuZhi2, Unigene96061\_JingChaoZuZhi2, Unigene96083\_JingChaoZuZhi2, Unigene96091\_JingChaoZuZhi2, Unigene96097\_JingChaoZuZhi2, Unigene96121\_JingChaoZuZhi2, Unigene96217\_JingChaoZuZhi2, Unigene96243\_JingChaoZuZhi2, Unigene96247\_JingChaoZuZhi2, Unigene96288\_JingChaoZuZhi2, Unigene96292\_JingChaoZuZhi2, Unigene96303\_JingChaoZuZhi2, Unigene96308\_JingChaoZuZhi2, Unigene9739\_JingChaoZuZhi2 |
| 26 | Tight junction | Unigene10027\_JingChaoZuZhi2, Unigene1002\_JingChaoZuZhi2, Unigene10047\_JingChaoZuZhi2, Unigene10078\_JingChaoZuZhi2, Unigene10218\_JingChaoZuZhi2, Unigene10259\_JingChaoZuZhi2, Unigene10318\_JingChaoZuZhi2, Unigene10541\_JingChaoZuZhi2, Unigene10601\_JingChaoZuZhi2, Unigene1060\_JingChaoZuZhi2, Unigene10628\_JingChaoZuZhi2, Unigene10765\_JingChaoZuZhi2, Unigene10932\_JingChaoZuZhi2, Unigene10\_JingChaoZuZhi2, Unigene11112\_JingChaoZuZhi2, Unigene11188\_JingChaoZuZhi2, Unigene11475\_JingChaoZuZhi2, Unigene11621\_JingChaoZuZhi2, Unigene1162\_JingChaoZuZhi2, Unigene11641\_JingChaoZuZhi2, Unigene12161\_JingChaoZuZhi2, Unigene12198\_JingChaoZuZhi2, Unigene12221\_JingChaoZuZhi2, Unigene12308\_JingChaoZuZhi2, Unigene12337\_JingChaoZuZhi2, Unigene12425\_JingChaoZuZhi2, Unigene12791\_JingChaoZuZhi2, Unigene12899\_JingChaoZuZhi2, Unigene13176\_JingChaoZuZhi2, Unigene13298\_JingChaoZuZhi2, Unigene13335\_JingChaoZuZhi2, Unigene13717\_JingChaoZuZhi2, Unigene13766\_JingChaoZuZhi2, Unigene1376\_JingChaoZuZhi2, Unigene14285\_JingChaoZuZhi2, Unigene14534\_JingChaoZuZhi2, Unigene14872\_JingChaoZuZhi2, Unigene14878\_JingChaoZuZhi2, Unigene15029\_JingChaoZuZhi2, Unigene15030\_JingChaoZuZhi2, Unigene15333\_JingChaoZuZhi2, Unigene15345\_JingChaoZuZhi2, Unigene15649\_JingChaoZuZhi2, Unigene15876\_JingChaoZuZhi2, Unigene15986\_JingChaoZuZhi2, Unigene16004\_JingChaoZuZhi2, Unigene16028\_JingChaoZuZhi2, Unigene16422\_JingChaoZuZhi2, Unigene16427\_JingChaoZuZhi2, Unigene16597\_JingChaoZuZhi2, Unigene16746\_JingChaoZuZhi2, Unigene16797\_JingChaoZuZhi2, Unigene16969\_JingChaoZuZhi2, Unigene17087\_JingChaoZuZhi2, Unigene1760\_JingChaoZuZhi2, Unigene17732\_JingChaoZuZhi2, Unigene17888\_JingChaoZuZhi2, Unigene17957\_JingChaoZuZhi2, Unigene18017\_JingChaoZuZhi2, Unigene18051\_JingChaoZuZhi2, Unigene18215\_JingChaoZuZhi2, Unigene18260\_JingChaoZuZhi2, Unigene18438\_JingChaoZuZhi2, Unigene18491\_JingChaoZuZhi2, Unigene18545\_JingChaoZuZhi2, Unigene18614\_JingChaoZuZhi2, Unigene18648\_JingChaoZuZhi2, Unigene18699\_JingChaoZuZhi2, Unigene187\_JingChaoZuZhi2, Unigene1887\_JingChaoZuZhi2, Unigene18892\_JingChaoZuZhi2, Unigene18894\_JingChaoZuZhi2, Unigene18957\_JingChaoZuZhi2, Unigene19222\_JingChaoZuZhi2, Unigene19396\_JingChaoZuZhi2, Unigene19466\_JingChaoZuZhi2, Unigene19559\_JingChaoZuZhi2, Unigene19974\_JingChaoZuZhi2, Unigene20086\_JingChaoZuZhi2, Unigene20137\_JingChaoZuZhi2, Unigene20307\_JingChaoZuZhi2, Unigene20312\_JingChaoZuZhi2, Unigene20464\_JingChaoZuZhi2, Unigene20479\_JingChaoZuZhi2, Unigene204\_JingChaoZuZhi2, Unigene20580\_JingChaoZuZhi2, Unigene20608\_JingChaoZuZhi2, Unigene20630\_JingChaoZuZhi2, Unigene20846\_JingChaoZuZhi2, Unigene21100\_JingChaoZuZhi2, Unigene21225\_JingChaoZuZhi2, Unigene21487\_JingChaoZuZhi2, Unigene21578\_JingChaoZuZhi2, Unigene21605\_JingChaoZuZhi2, Unigene2161\_JingChaoZuZhi2, Unigene21625\_JingChaoZuZhi2, Unigene21801\_JingChaoZuZhi2, Unigene21818\_JingChaoZuZhi2, Unigene22024\_JingChaoZuZhi2, Unigene22183\_JingChaoZuZhi2, Unigene22189\_JingChaoZuZhi2, Unigene22216\_JingChaoZuZhi2, Unigene22243\_JingChaoZuZhi2, Unigene22487\_JingChaoZuZhi2, Unigene22551\_JingChaoZuZhi2, Unigene22595\_JingChaoZuZhi2, Unigene22779\_JingChaoZuZhi2, Unigene22850\_JingChaoZuZhi2, Unigene22895\_JingChaoZuZhi2, Unigene2330\_JingChaoZuZhi2, Unigene25164\_JingChaoZuZhi2, Unigene26730\_JingChaoZuZhi2, Unigene26987\_JingChaoZuZhi2, Unigene28263\_JingChaoZuZhi2, Unigene28737\_JingChaoZuZhi2, Unigene2960\_JingChaoZuZhi2, Unigene2968\_JingChaoZuZhi2, Unigene30852\_JingChaoZuZhi2, Unigene31918\_JingChaoZuZhi2, Unigene3191\_JingChaoZuZhi2, Unigene3258\_JingChaoZuZhi2, Unigene33561\_JingChaoZuZhi2, Unigene3413\_JingChaoZuZhi2, Unigene35016\_JingChaoZuZhi2, Unigene3519\_JingChaoZuZhi2, Unigene35549\_JingChaoZuZhi2, Unigene39102\_JingChaoZuZhi2, Unigene3912\_JingChaoZuZhi2, Unigene3931\_JingChaoZuZhi2, Unigene39726\_JingChaoZuZhi2, Unigene40919\_JingChaoZuZhi2, Unigene41688\_JingChaoZuZhi2, Unigene42248\_JingChaoZuZhi2, Unigene4377\_JingChaoZuZhi2, Unigene44190\_JingChaoZuZhi2, Unigene44336\_JingChaoZuZhi2, Unigene44441\_JingChaoZuZhi2, Unigene44731\_JingChaoZuZhi2, Unigene46256\_JingChaoZuZhi2, Unigene46460\_JingChaoZuZhi2, Unigene475\_JingChaoZuZhi2, Unigene4778\_JingChaoZuZhi2, Unigene48309\_JingChaoZuZhi2, Unigene4878\_JingChaoZuZhi2, Unigene49761\_JingChaoZuZhi2, Unigene50563\_JingChaoZuZhi2, Unigene50673\_JingChaoZuZhi2, Unigene5072\_JingChaoZuZhi2, Unigene51285\_JingChaoZuZhi2, Unigene51467\_JingChaoZuZhi2, Unigene52073\_JingChaoZuZhi2, Unigene5211\_JingChaoZuZhi2, Unigene52554\_JingChaoZuZhi2, Unigene53568\_JingChaoZuZhi2, Unigene53958\_JingChaoZuZhi2, Unigene54744\_JingChaoZuZhi2, Unigene54758\_JingChaoZuZhi2, Unigene54980\_JingChaoZuZhi2, Unigene54986\_JingChaoZuZhi2, Unigene55839\_JingChaoZuZhi2, Unigene559\_JingChaoZuZhi2, Unigene56456\_JingChaoZuZhi2, Unigene56642\_JingChaoZuZhi2, Unigene57200\_JingChaoZuZhi2, Unigene57349\_JingChaoZuZhi2, Unigene57751\_JingChaoZuZhi2, Unigene5823\_JingChaoZuZhi2, Unigene58685\_JingChaoZuZhi2, Unigene59146\_JingChaoZuZhi2, Unigene61244\_JingChaoZuZhi2, Unigene61526\_JingChaoZuZhi2, Unigene6174\_JingChaoZuZhi2, Unigene62167\_JingChaoZuZhi2, Unigene6239\_JingChaoZuZhi2, Unigene6361\_JingChaoZuZhi2, Unigene64164\_JingChaoZuZhi2, Unigene64189\_JingChaoZuZhi2, Unigene64743\_JingChaoZuZhi2, Unigene64830\_JingChaoZuZhi2, Unigene65002\_JingChaoZuZhi2, Unigene65332\_JingChaoZuZhi2, Unigene65648\_JingChaoZuZhi2, Unigene65657\_JingChaoZuZhi2, Unigene6634\_JingChaoZuZhi2, Unigene67081\_JingChaoZuZhi2, Unigene6769\_JingChaoZuZhi2, Unigene683\_JingChaoZuZhi2, Unigene6864\_JingChaoZuZhi2, Unigene69005\_JingChaoZuZhi2, Unigene69100\_JingChaoZuZhi2, Unigene691\_JingChaoZuZhi2, Unigene69368\_JingChaoZuZhi2, Unigene69934\_JingChaoZuZhi2, Unigene70373\_JingChaoZuZhi2, Unigene70414\_JingChaoZuZhi2, Unigene70569\_JingChaoZuZhi2, Unigene70723\_JingChaoZuZhi2, Unigene71247\_JingChaoZuZhi2, Unigene71592\_JingChaoZuZhi2, Unigene72249\_JingChaoZuZhi2, Unigene75641\_JingChaoZuZhi2, Unigene76303\_JingChaoZuZhi2, Unigene77310\_JingChaoZuZhi2, Unigene77406\_JingChaoZuZhi2, Unigene7931\_JingChaoZuZhi2, Unigene7939\_JingChaoZuZhi2, Unigene79568\_JingChaoZuZhi2, Unigene79882\_JingChaoZuZhi2, Unigene80215\_JingChaoZuZhi2, Unigene80252\_JingChaoZuZhi2, Unigene80731\_JingChaoZuZhi2, Unigene81798\_JingChaoZuZhi2, Unigene82117\_JingChaoZuZhi2, Unigene82140\_JingChaoZuZhi2, Unigene82144\_JingChaoZuZhi2, Unigene82489\_JingChaoZuZhi2, Unigene82508\_JingChaoZuZhi2, Unigene82605\_JingChaoZuZhi2, Unigene82607\_JingChaoZuZhi2, Unigene8261\_JingChaoZuZhi2, Unigene82842\_JingChaoZuZhi2, Unigene83017\_JingChaoZuZhi2, Unigene83487\_JingChaoZuZhi2, Unigene83872\_JingChaoZuZhi2, Unigene84510\_JingChaoZuZhi2, Unigene84563\_JingChaoZuZhi2, Unigene85122\_JingChaoZuZhi2, Unigene85132\_JingChaoZuZhi2, Unigene85193\_JingChaoZuZhi2, Unigene85306\_JingChaoZuZhi2, Unigene85387\_JingChaoZuZhi2, Unigene85721\_JingChaoZuZhi2, Unigene85923\_JingChaoZuZhi2, Unigene866\_JingChaoZuZhi2, Unigene86759\_JingChaoZuZhi2, Unigene86899\_JingChaoZuZhi2, Unigene87183\_JingChaoZuZhi2, Unigene87191\_JingChaoZuZhi2, Unigene87290\_JingChaoZuZhi2, Unigene87305\_JingChaoZuZhi2, Unigene87332\_JingChaoZuZhi2, Unigene87677\_JingChaoZuZhi2, Unigene876\_JingChaoZuZhi2, Unigene87825\_JingChaoZuZhi2, Unigene87958\_JingChaoZuZhi2, Unigene87974\_JingChaoZuZhi2, Unigene88106\_JingChaoZuZhi2, Unigene88169\_JingChaoZuZhi2, Unigene88342\_JingChaoZuZhi2, Unigene88748\_JingChaoZuZhi2, Unigene8877\_JingChaoZuZhi2, Unigene89045\_JingChaoZuZhi2, Unigene89060\_JingChaoZuZhi2, Unigene89194\_JingChaoZuZhi2, Unigene89360\_JingChaoZuZhi2, Unigene89409\_JingChaoZuZhi2, Unigene89741\_JingChaoZuZhi2, Unigene89811\_JingChaoZuZhi2, Unigene8981\_JingChaoZuZhi2, Unigene89901\_JingChaoZuZhi2, Unigene89959\_JingChaoZuZhi2, Unigene9005\_JingChaoZuZhi2, Unigene90401\_JingChaoZuZhi2, Unigene90530\_JingChaoZuZhi2, Unigene90626\_JingChaoZuZhi2, Unigene90767\_JingChaoZuZhi2, Unigene90832\_JingChaoZuZhi2, Unigene91030\_JingChaoZuZhi2, Unigene91163\_JingChaoZuZhi2, Unigene91221\_JingChaoZuZhi2, Unigene91228\_JingChaoZuZhi2, Unigene91631\_JingChaoZuZhi2, Unigene91703\_JingChaoZuZhi2, Unigene91991\_JingChaoZuZhi2, Unigene92141\_JingChaoZuZhi2, Unigene92158\_JingChaoZuZhi2, Unigene92323\_JingChaoZuZhi2, Unigene92329\_JingChaoZuZhi2, Unigene92407\_JingChaoZuZhi2, Unigene92448\_JingChaoZuZhi2, Unigene92471\_JingChaoZuZhi2, Unigene92577\_JingChaoZuZhi2, Unigene92673\_JingChaoZuZhi2, Unigene92769\_JingChaoZuZhi2, Unigene92782\_JingChaoZuZhi2, Unigene92962\_JingChaoZuZhi2, Unigene93010\_JingChaoZuZhi2, Unigene93114\_JingChaoZuZhi2, Unigene93270\_JingChaoZuZhi2, Unigene93279\_JingChaoZuZhi2, Unigene93294\_JingChaoZuZhi2, Unigene932\_JingChaoZuZhi2, Unigene93374\_JingChaoZuZhi2, Unigene93384\_JingChaoZuZhi2, Unigene93439\_JingChaoZuZhi2, Unigene93754\_JingChaoZuZhi2, Unigene93782\_JingChaoZuZhi2, Unigene93793\_JingChaoZuZhi2, Unigene93865\_JingChaoZuZhi2, Unigene93875\_JingChaoZuZhi2, Unigene9389\_JingChaoZuZhi2, Unigene93989\_JingChaoZuZhi2, Unigene94036\_JingChaoZuZhi2, Unigene94110\_JingChaoZuZhi2, Unigene94124\_JingChaoZuZhi2, Unigene94272\_JingChaoZuZhi2, Unigene94370\_JingChaoZuZhi2, Unigene94388\_JingChaoZuZhi2, Unigene94634\_JingChaoZuZhi2, Unigene94672\_JingChaoZuZhi2, Unigene94723\_JingChaoZuZhi2, Unigene94823\_JingChaoZuZhi2, Unigene9483\_JingChaoZuZhi2, Unigene95020\_JingChaoZuZhi2, Unigene95031\_JingChaoZuZhi2, Unigene95043\_JingChaoZuZhi2, Unigene95059\_JingChaoZuZhi2, Unigene95062\_JingChaoZuZhi2, Unigene95100\_JingChaoZuZhi2, Unigene95143\_JingChaoZuZhi2, Unigene95163\_JingChaoZuZhi2, Unigene951\_JingChaoZuZhi2, Unigene95224\_JingChaoZuZhi2, Unigene95238\_JingChaoZuZhi2, Unigene95243\_JingChaoZuZhi2, Unigene9532\_JingChaoZuZhi2, Unigene95486\_JingChaoZuZhi2, Unigene95677\_JingChaoZuZhi2, Unigene95727\_JingChaoZuZhi2, Unigene95774\_JingChaoZuZhi2, Unigene95791\_JingChaoZuZhi2, Unigene95807\_JingChaoZuZhi2, Unigene95811\_JingChaoZuZhi2, Unigene95863\_JingChaoZuZhi2, Unigene95879\_JingChaoZuZhi2, Unigene95881\_JingChaoZuZhi2, Unigene95882\_JingChaoZuZhi2, Unigene95907\_JingChaoZuZhi2, Unigene95934\_JingChaoZuZhi2, Unigene95954\_JingChaoZuZhi2, Unigene95972\_JingChaoZuZhi2, Unigene95982\_JingChaoZuZhi2, Unigene95987\_JingChaoZuZhi2, Unigene95994\_JingChaoZuZhi2, Unigene96012\_JingChaoZuZhi2, Unigene96061\_JingChaoZuZhi2, Unigene96083\_JingChaoZuZhi2, Unigene96097\_JingChaoZuZhi2, Unigene96144\_JingChaoZuZhi2, Unigene96169\_JingChaoZuZhi2, Unigene96217\_JingChaoZuZhi2, Unigene96234\_JingChaoZuZhi2, Unigene96243\_JingChaoZuZhi2, Unigene96244\_JingChaoZuZhi2, Unigene96247\_JingChaoZuZhi2, Unigene96288\_JingChaoZuZhi2, Unigene96292\_JingChaoZuZhi2, Unigene96303\_JingChaoZuZhi2, Unigene96308\_JingChaoZuZhi2, Unigene96309\_JingChaoZuZhi2, Unigene9739\_JingChaoZuZhi2 |
| 27 | Phagosome | Unigene10047\_JingChaoZuZhi2, Unigene10189\_JingChaoZuZhi2, Unigene10259\_JingChaoZuZhi2, Unigene10286\_JingChaoZuZhi2, Unigene10443\_JingChaoZuZhi2, Unigene1060\_JingChaoZuZhi2, Unigene10747\_JingChaoZuZhi2, Unigene10784\_JingChaoZuZhi2, Unigene10\_JingChaoZuZhi2, Unigene11146\_JingChaoZuZhi2, Unigene11446\_JingChaoZuZhi2, Unigene11592\_JingChaoZuZhi2, Unigene1162\_JingChaoZuZhi2, Unigene11730\_JingChaoZuZhi2, Unigene11732\_JingChaoZuZhi2, Unigene12414\_JingChaoZuZhi2, Unigene12729\_JingChaoZuZhi2, Unigene12787\_JingChaoZuZhi2, Unigene12819\_JingChaoZuZhi2, Unigene1288\_JingChaoZuZhi2, Unigene12935\_JingChaoZuZhi2, Unigene13026\_JingChaoZuZhi2, Unigene14104\_JingChaoZuZhi2, Unigene14109\_JingChaoZuZhi2, Unigene14125\_JingChaoZuZhi2, Unigene1422\_JingChaoZuZhi2, Unigene14856\_JingChaoZuZhi2, Unigene14882\_JingChaoZuZhi2, Unigene15172\_JingChaoZuZhi2, Unigene15182\_JingChaoZuZhi2, Unigene16004\_JingChaoZuZhi2, Unigene16121\_JingChaoZuZhi2, Unigene16152\_JingChaoZuZhi2, Unigene16289\_JingChaoZuZhi2, Unigene16427\_JingChaoZuZhi2, Unigene16597\_JingChaoZuZhi2, Unigene16873\_JingChaoZuZhi2, Unigene16884\_JingChaoZuZhi2, Unigene16938\_JingChaoZuZhi2, Unigene17062\_JingChaoZuZhi2, Unigene17087\_JingChaoZuZhi2, Unigene18059\_JingChaoZuZhi2, Unigene18128\_JingChaoZuZhi2, Unigene18176\_JingChaoZuZhi2, Unigene18309\_JingChaoZuZhi2, Unigene18353\_JingChaoZuZhi2, Unigene18515\_JingChaoZuZhi2, Unigene1878\_JingChaoZuZhi2, Unigene19001\_JingChaoZuZhi2, Unigene19021\_JingChaoZuZhi2, Unigene19108\_JingChaoZuZhi2, Unigene19304\_JingChaoZuZhi2, Unigene19951\_JingChaoZuZhi2, Unigene20159\_JingChaoZuZhi2, Unigene20297\_JingChaoZuZhi2, Unigene20995\_JingChaoZuZhi2, Unigene20998\_JingChaoZuZhi2, Unigene21020\_JingChaoZuZhi2, Unigene21147\_JingChaoZuZhi2, Unigene21215\_JingChaoZuZhi2, Unigene21472\_JingChaoZuZhi2, Unigene21533\_JingChaoZuZhi2, Unigene21555\_JingChaoZuZhi2, Unigene21624\_JingChaoZuZhi2, Unigene21741\_JingChaoZuZhi2, Unigene21759\_JingChaoZuZhi2, Unigene21804\_JingChaoZuZhi2, Unigene21818\_JingChaoZuZhi2, Unigene21993\_JingChaoZuZhi2, Unigene22488\_JingChaoZuZhi2, Unigene22520\_JingChaoZuZhi2, Unigene22583\_JingChaoZuZhi2, Unigene22586\_JingChaoZuZhi2, Unigene2273\_JingChaoZuZhi2, Unigene22914\_JingChaoZuZhi2, Unigene22916\_JingChaoZuZhi2, Unigene22963\_JingChaoZuZhi2, Unigene23482\_JingChaoZuZhi2, Unigene234\_JingChaoZuZhi2, Unigene23827\_JingChaoZuZhi2, Unigene25228\_JingChaoZuZhi2, Unigene25912\_JingChaoZuZhi2, Unigene2656\_JingChaoZuZhi2, Unigene26592\_JingChaoZuZhi2, Unigene26746\_JingChaoZuZhi2, Unigene26874\_JingChaoZuZhi2, Unigene26987\_JingChaoZuZhi2, Unigene27057\_JingChaoZuZhi2, Unigene28427\_JingChaoZuZhi2, Unigene29201\_JingChaoZuZhi2, Unigene29295\_JingChaoZuZhi2, Unigene2932\_JingChaoZuZhi2, Unigene31285\_JingChaoZuZhi2, Unigene31416\_JingChaoZuZhi2, Unigene31868\_JingChaoZuZhi2, Unigene31918\_JingChaoZuZhi2, Unigene3245\_JingChaoZuZhi2, Unigene32941\_JingChaoZuZhi2, Unigene33026\_JingChaoZuZhi2, Unigene33604\_JingChaoZuZhi2, Unigene33692\_JingChaoZuZhi2, Unigene35016\_JingChaoZuZhi2, Unigene35243\_JingChaoZuZhi2, Unigene36354\_JingChaoZuZhi2, Unigene3665\_JingChaoZuZhi2, Unigene36859\_JingChaoZuZhi2, Unigene37252\_JingChaoZuZhi2, Unigene37639\_JingChaoZuZhi2, Unigene3782\_JingChaoZuZhi2, Unigene38458\_JingChaoZuZhi2, Unigene38924\_JingChaoZuZhi2, Unigene39102\_JingChaoZuZhi2, Unigene39259\_JingChaoZuZhi2, Unigene39457\_JingChaoZuZhi2, Unigene4028\_JingChaoZuZhi2, Unigene4032\_JingChaoZuZhi2, Unigene40559\_JingChaoZuZhi2, Unigene40674\_JingChaoZuZhi2, Unigene40848\_JingChaoZuZhi2, Unigene40919\_JingChaoZuZhi2, Unigene4094\_JingChaoZuZhi2, Unigene41953\_JingChaoZuZhi2, Unigene41973\_JingChaoZuZhi2, Unigene42248\_JingChaoZuZhi2, Unigene42346\_JingChaoZuZhi2, Unigene42610\_JingChaoZuZhi2, Unigene43322\_JingChaoZuZhi2, Unigene43537\_JingChaoZuZhi2, Unigene43584\_JingChaoZuZhi2, Unigene44190\_JingChaoZuZhi2, Unigene44224\_JingChaoZuZhi2, Unigene45044\_JingChaoZuZhi2, Unigene45086\_JingChaoZuZhi2, Unigene45331\_JingChaoZuZhi2, Unigene45782\_JingChaoZuZhi2, Unigene45948\_JingChaoZuZhi2, Unigene46155\_JingChaoZuZhi2, Unigene47374\_JingChaoZuZhi2, Unigene47701\_JingChaoZuZhi2, Unigene47840\_JingChaoZuZhi2, Unigene48843\_JingChaoZuZhi2, Unigene48984\_JingChaoZuZhi2, Unigene49056\_JingChaoZuZhi2, Unigene50084\_JingChaoZuZhi2, Unigene5060\_JingChaoZuZhi2, Unigene5072\_JingChaoZuZhi2, Unigene51467\_JingChaoZuZhi2, Unigene51895\_JingChaoZuZhi2, Unigene52002\_JingChaoZuZhi2, Unigene52195\_JingChaoZuZhi2, Unigene52526\_JingChaoZuZhi2, Unigene52758\_JingChaoZuZhi2, Unigene52759\_JingChaoZuZhi2, Unigene52821\_JingChaoZuZhi2, Unigene53553\_JingChaoZuZhi2, Unigene53871\_JingChaoZuZhi2, Unigene54569\_JingChaoZuZhi2, Unigene55368\_JingChaoZuZhi2, Unigene56079\_JingChaoZuZhi2, Unigene56557\_JingChaoZuZhi2, Unigene56775\_JingChaoZuZhi2, Unigene58011\_JingChaoZuZhi2, Unigene58608\_JingChaoZuZhi2, Unigene58988\_JingChaoZuZhi2, Unigene59146\_JingChaoZuZhi2, Unigene59426\_JingChaoZuZhi2, Unigene59896\_JingChaoZuZhi2, Unigene60139\_JingChaoZuZhi2, Unigene60221\_JingChaoZuZhi2, Unigene60242\_JingChaoZuZhi2, Unigene60512\_JingChaoZuZhi2, Unigene60580\_JingChaoZuZhi2, Unigene61033\_JingChaoZuZhi2, Unigene61189\_JingChaoZuZhi2, Unigene61244\_JingChaoZuZhi2, Unigene61736\_JingChaoZuZhi2, Unigene61846\_JingChaoZuZhi2, Unigene62041\_JingChaoZuZhi2, Unigene62272\_JingChaoZuZhi2, Unigene6245\_JingChaoZuZhi2, Unigene62821\_JingChaoZuZhi2, Unigene63409\_JingChaoZuZhi2, Unigene64251\_JingChaoZuZhi2, Unigene65652\_JingChaoZuZhi2, Unigene6634\_JingChaoZuZhi2, Unigene66535\_JingChaoZuZhi2, Unigene66590\_JingChaoZuZhi2, Unigene66806\_JingChaoZuZhi2, Unigene66983\_JingChaoZuZhi2, Unigene67542\_JingChaoZuZhi2, Unigene67676\_JingChaoZuZhi2, Unigene67804\_JingChaoZuZhi2, Unigene67\_JingChaoZuZhi2, Unigene68009\_JingChaoZuZhi2, Unigene68097\_JingChaoZuZhi2, Unigene68111\_JingChaoZuZhi2, Unigene68477\_JingChaoZuZhi2, Unigene6863\_JingChaoZuZhi2, Unigene68689\_JingChaoZuZhi2, Unigene68845\_JingChaoZuZhi2, Unigene69071\_JingChaoZuZhi2, Unigene69100\_JingChaoZuZhi2, Unigene69708\_JingChaoZuZhi2, Unigene70308\_JingChaoZuZhi2, Unigene70385\_JingChaoZuZhi2, Unigene7068\_JingChaoZuZhi2, Unigene70808\_JingChaoZuZhi2, Unigene71118\_JingChaoZuZhi2, Unigene71592\_JingChaoZuZhi2, Unigene71599\_JingChaoZuZhi2, Unigene71885\_JingChaoZuZhi2, Unigene71939\_JingChaoZuZhi2, Unigene72036\_JingChaoZuZhi2, Unigene7286\_JingChaoZuZhi2, Unigene73052\_JingChaoZuZhi2, Unigene7344\_JingChaoZuZhi2, Unigene73595\_JingChaoZuZhi2, Unigene74599\_JingChaoZuZhi2, Unigene74948\_JingChaoZuZhi2, Unigene74979\_JingChaoZuZhi2, Unigene75821\_JingChaoZuZhi2, Unigene75840\_JingChaoZuZhi2, Unigene76439\_JingChaoZuZhi2, Unigene7672\_JingChaoZuZhi2, Unigene77024\_JingChaoZuZhi2, Unigene77211\_JingChaoZuZhi2, Unigene77528\_JingChaoZuZhi2, Unigene77543\_JingChaoZuZhi2, Unigene78460\_JingChaoZuZhi2, Unigene78518\_JingChaoZuZhi2, Unigene79077\_JingChaoZuZhi2, Unigene79268\_JingChaoZuZhi2, Unigene79577\_JingChaoZuZhi2, Unigene79925\_JingChaoZuZhi2, Unigene80489\_JingChaoZuZhi2, Unigene80609\_JingChaoZuZhi2, Unigene81700\_JingChaoZuZhi2, Unigene82040\_JingChaoZuZhi2, Unigene82061\_JingChaoZuZhi2, Unigene82117\_JingChaoZuZhi2, Unigene82489\_JingChaoZuZhi2, Unigene82742\_JingChaoZuZhi2, Unigene82811\_JingChaoZuZhi2, Unigene82\_JingChaoZuZhi2, Unigene83846\_JingChaoZuZhi2, Unigene83872\_JingChaoZuZhi2, Unigene83933\_JingChaoZuZhi2, Unigene84046\_JingChaoZuZhi2, Unigene84406\_JingChaoZuZhi2, Unigene84512\_JingChaoZuZhi2, Unigene84519\_JingChaoZuZhi2, Unigene8455\_JingChaoZuZhi2, Unigene84655\_JingChaoZuZhi2, Unigene8505\_JingChaoZuZhi2, Unigene85193\_JingChaoZuZhi2, Unigene85789\_JingChaoZuZhi2, Unigene8596\_JingChaoZuZhi2, Unigene86417\_JingChaoZuZhi2, Unigene86975\_JingChaoZuZhi2, Unigene86995\_JingChaoZuZhi2, Unigene87020\_JingChaoZuZhi2, Unigene87241\_JingChaoZuZhi2, Unigene87261\_JingChaoZuZhi2, Unigene87347\_JingChaoZuZhi2, Unigene87567\_JingChaoZuZhi2, Unigene87941\_JingChaoZuZhi2, Unigene87946\_JingChaoZuZhi2, Unigene88009\_JingChaoZuZhi2, Unigene88041\_JingChaoZuZhi2, Unigene88095\_JingChaoZuZhi2, Unigene88121\_JingChaoZuZhi2, Unigene88262\_JingChaoZuZhi2, Unigene88342\_JingChaoZuZhi2, Unigene8853\_JingChaoZuZhi2, Unigene88703\_JingChaoZuZhi2, Unigene88774\_JingChaoZuZhi2, Unigene88909\_JingChaoZuZhi2, Unigene89057\_JingChaoZuZhi2, Unigene89060\_JingChaoZuZhi2, Unigene89128\_JingChaoZuZhi2, Unigene89354\_JingChaoZuZhi2, Unigene89409\_JingChaoZuZhi2, Unigene89410\_JingChaoZuZhi2, Unigene89595\_JingChaoZuZhi2, Unigene89755\_JingChaoZuZhi2, Unigene8986\_JingChaoZuZhi2, Unigene90239\_JingChaoZuZhi2, Unigene90486\_JingChaoZuZhi2, Unigene90645\_JingChaoZuZhi2, Unigene90767\_JingChaoZuZhi2, Unigene91163\_JingChaoZuZhi2, Unigene91174\_JingChaoZuZhi2, Unigene91179\_JingChaoZuZhi2, Unigene91223\_JingChaoZuZhi2, Unigene91490\_JingChaoZuZhi2, Unigene91591\_JingChaoZuZhi2, Unigene91611\_JingChaoZuZhi2, Unigene91670\_JingChaoZuZhi2, Unigene91730\_JingChaoZuZhi2, Unigene91994\_JingChaoZuZhi2, Unigene92135\_JingChaoZuZhi2, Unigene92141\_JingChaoZuZhi2, Unigene92367\_JingChaoZuZhi2, Unigene92410\_JingChaoZuZhi2, Unigene92452\_JingChaoZuZhi2, Unigene92631\_JingChaoZuZhi2, Unigene92734\_JingChaoZuZhi2, Unigene92795\_JingChaoZuZhi2, Unigene92942\_JingChaoZuZhi2, Unigene92949\_JingChaoZuZhi2, Unigene93010\_JingChaoZuZhi2, Unigene93207\_JingChaoZuZhi2, Unigene93785\_JingChaoZuZhi2, Unigene93840\_JingChaoZuZhi2, Unigene94049\_JingChaoZuZhi2, Unigene94110\_JingChaoZuZhi2, Unigene94150\_JingChaoZuZhi2, Unigene94165\_JingChaoZuZhi2, Unigene94214\_JingChaoZuZhi2, Unigene94337\_JingChaoZuZhi2, Unigene94552\_JingChaoZuZhi2, Unigene94561\_JingChaoZuZhi2, Unigene94706\_JingChaoZuZhi2, Unigene94775\_JingChaoZuZhi2, Unigene94898\_JingChaoZuZhi2, Unigene94980\_JingChaoZuZhi2, Unigene94997\_JingChaoZuZhi2, Unigene95042\_JingChaoZuZhi2, Unigene951\_JingChaoZuZhi2, Unigene95200\_JingChaoZuZhi2, Unigene95331\_JingChaoZuZhi2, Unigene95395\_JingChaoZuZhi2, Unigene95496\_JingChaoZuZhi2, Unigene95526\_JingChaoZuZhi2, Unigene95753\_JingChaoZuZhi2, Unigene95789\_JingChaoZuZhi2, Unigene95875\_JingChaoZuZhi2, Unigene95907\_JingChaoZuZhi2, Unigene95964\_JingChaoZuZhi2, Unigene95987\_JingChaoZuZhi2, Unigene95994\_JingChaoZuZhi2, Unigene96002\_JingChaoZuZhi2, Unigene96023\_JingChaoZuZhi2, Unigene96032\_JingChaoZuZhi2, Unigene96084\_JingChaoZuZhi2, Unigene96121\_JingChaoZuZhi2, Unigene96151\_JingChaoZuZhi2, Unigene96229\_JingChaoZuZhi2, Unigene96279\_JingChaoZuZhi2, Unigene96303\_JingChaoZuZhi2, Unigene9676\_JingChaoZuZhi2, Unigene9739\_JingChaoZuZhi2, Unigene990\_JingChaoZuZhi2 |
| 28 | Lysosome | Unigene1016\_JingChaoZuZhi2, Unigene10279\_JingChaoZuZhi2, Unigene10571\_JingChaoZuZhi2, Unigene10605\_JingChaoZuZhi2, Unigene10695\_JingChaoZuZhi2, Unigene11462\_JingChaoZuZhi2, Unigene11468\_JingChaoZuZhi2, Unigene11592\_JingChaoZuZhi2, Unigene11681\_JingChaoZuZhi2, Unigene11808\_JingChaoZuZhi2, Unigene12083\_JingChaoZuZhi2, Unigene12099\_JingChaoZuZhi2, Unigene12154\_JingChaoZuZhi2, Unigene12257\_JingChaoZuZhi2, Unigene12412\_JingChaoZuZhi2, Unigene12520\_JingChaoZuZhi2, Unigene12560\_JingChaoZuZhi2, Unigene12576\_JingChaoZuZhi2, Unigene12821\_JingChaoZuZhi2, Unigene13280\_JingChaoZuZhi2, Unigene13403\_JingChaoZuZhi2, Unigene13565\_JingChaoZuZhi2, Unigene13699\_JingChaoZuZhi2, Unigene13712\_JingChaoZuZhi2, Unigene14018\_JingChaoZuZhi2, Unigene14046\_JingChaoZuZhi2, Unigene14104\_JingChaoZuZhi2, Unigene14329\_JingChaoZuZhi2, Unigene14363\_JingChaoZuZhi2, Unigene144\_JingChaoZuZhi2, Unigene14545\_JingChaoZuZhi2, Unigene14673\_JingChaoZuZhi2, Unigene14856\_JingChaoZuZhi2, Unigene15172\_JingChaoZuZhi2, Unigene15633\_JingChaoZuZhi2, Unigene15837\_JingChaoZuZhi2, Unigene16152\_JingChaoZuZhi2, Unigene16679\_JingChaoZuZhi2, Unigene16795\_JingChaoZuZhi2, Unigene16938\_JingChaoZuZhi2, Unigene16955\_JingChaoZuZhi2, Unigene1724\_JingChaoZuZhi2, Unigene17816\_JingChaoZuZhi2, Unigene17905\_JingChaoZuZhi2, Unigene18328\_JingChaoZuZhi2, Unigene18401\_JingChaoZuZhi2, Unigene18479\_JingChaoZuZhi2, Unigene18856\_JingChaoZuZhi2, Unigene18905\_JingChaoZuZhi2, Unigene18991\_JingChaoZuZhi2, Unigene19001\_JingChaoZuZhi2, Unigene19140\_JingChaoZuZhi2, Unigene19251\_JingChaoZuZhi2, Unigene19296\_JingChaoZuZhi2, Unigene19312\_JingChaoZuZhi2, Unigene19428\_JingChaoZuZhi2, Unigene19445\_JingChaoZuZhi2, Unigene19575\_JingChaoZuZhi2, Unigene19609\_JingChaoZuZhi2, Unigene19620\_JingChaoZuZhi2, Unigene19670\_JingChaoZuZhi2, Unigene19754\_JingChaoZuZhi2, Unigene20186\_JingChaoZuZhi2, Unigene20393\_JingChaoZuZhi2, Unigene20434\_JingChaoZuZhi2, Unigene20557\_JingChaoZuZhi2, Unigene20722\_JingChaoZuZhi2, Unigene20831\_JingChaoZuZhi2, Unigene20950\_JingChaoZuZhi2, Unigene21067\_JingChaoZuZhi2, Unigene2131\_JingChaoZuZhi2, Unigene21326\_JingChaoZuZhi2, Unigene21358\_JingChaoZuZhi2, Unigene21475\_JingChaoZuZhi2, Unigene21913\_JingChaoZuZhi2, Unigene21993\_JingChaoZuZhi2, Unigene219\_JingChaoZuZhi2, Unigene22514\_JingChaoZuZhi2, Unigene22520\_JingChaoZuZhi2, Unigene22683\_JingChaoZuZhi2, Unigene22764\_JingChaoZuZhi2, Unigene22838\_JingChaoZuZhi2, Unigene23731\_JingChaoZuZhi2, Unigene23882\_JingChaoZuZhi2, Unigene24793\_JingChaoZuZhi2, Unigene25032\_JingChaoZuZhi2, Unigene25310\_JingChaoZuZhi2, Unigene2535\_JingChaoZuZhi2, Unigene2558\_JingChaoZuZhi2, Unigene25723\_JingChaoZuZhi2, Unigene2643\_JingChaoZuZhi2, Unigene26442\_JingChaoZuZhi2, Unigene26787\_JingChaoZuZhi2, Unigene27057\_JingChaoZuZhi2, Unigene27220\_JingChaoZuZhi2, Unigene2865\_JingChaoZuZhi2, Unigene2881\_JingChaoZuZhi2, Unigene30126\_JingChaoZuZhi2, Unigene30207\_JingChaoZuZhi2, Unigene30881\_JingChaoZuZhi2, Unigene31320\_JingChaoZuZhi2, Unigene33026\_JingChaoZuZhi2, Unigene33441\_JingChaoZuZhi2, Unigene33604\_JingChaoZuZhi2, Unigene34946\_JingChaoZuZhi2, Unigene35048\_JingChaoZuZhi2, Unigene35247\_JingChaoZuZhi2, Unigene36714\_JingChaoZuZhi2, Unigene3685\_JingChaoZuZhi2, Unigene36874\_JingChaoZuZhi2, Unigene36907\_JingChaoZuZhi2, Unigene37067\_JingChaoZuZhi2, Unigene37453\_JingChaoZuZhi2, Unigene37531\_JingChaoZuZhi2, Unigene37607\_JingChaoZuZhi2, Unigene37747\_JingChaoZuZhi2, Unigene38013\_JingChaoZuZhi2, Unigene38556\_JingChaoZuZhi2, Unigene39913\_JingChaoZuZhi2, Unigene40140\_JingChaoZuZhi2, Unigene40354\_JingChaoZuZhi2, Unigene42020\_JingChaoZuZhi2, Unigene42089\_JingChaoZuZhi2, Unigene42520\_JingChaoZuZhi2, Unigene42\_JingChaoZuZhi2, Unigene43293\_JingChaoZuZhi2, Unigene43959\_JingChaoZuZhi2, Unigene44000\_JingChaoZuZhi2, Unigene4431\_JingChaoZuZhi2, Unigene4438\_JingChaoZuZhi2, Unigene45202\_JingChaoZuZhi2, Unigene45407\_JingChaoZuZhi2, Unigene46156\_JingChaoZuZhi2, Unigene46314\_JingChaoZuZhi2, Unigene47051\_JingChaoZuZhi2, Unigene47300\_JingChaoZuZhi2, Unigene4797\_JingChaoZuZhi2, Unigene4805\_JingChaoZuZhi2, Unigene48506\_JingChaoZuZhi2, Unigene49373\_JingChaoZuZhi2, Unigene49877\_JingChaoZuZhi2, Unigene49884\_JingChaoZuZhi2, Unigene51033\_JingChaoZuZhi2, Unigene51085\_JingChaoZuZhi2, Unigene51610\_JingChaoZuZhi2, Unigene52142\_JingChaoZuZhi2, Unigene52205\_JingChaoZuZhi2, Unigene523\_JingChaoZuZhi2, Unigene52753\_JingChaoZuZhi2, Unigene5425\_JingChaoZuZhi2, Unigene54578\_JingChaoZuZhi2, Unigene54904\_JingChaoZuZhi2, Unigene55040\_JingChaoZuZhi2, Unigene55368\_JingChaoZuZhi2, Unigene55909\_JingChaoZuZhi2, Unigene56073\_JingChaoZuZhi2, Unigene56481\_JingChaoZuZhi2, Unigene57540\_JingChaoZuZhi2, Unigene57807\_JingChaoZuZhi2, Unigene57970\_JingChaoZuZhi2, Unigene58876\_JingChaoZuZhi2, Unigene59000\_JingChaoZuZhi2, Unigene59210\_JingChaoZuZhi2, Unigene5957\_JingChaoZuZhi2, Unigene59913\_JingChaoZuZhi2, Unigene60512\_JingChaoZuZhi2, Unigene60650\_JingChaoZuZhi2, Unigene60856\_JingChaoZuZhi2, Unigene60942\_JingChaoZuZhi2, Unigene61262\_JingChaoZuZhi2, Unigene61390\_JingChaoZuZhi2, Unigene61737\_JingChaoZuZhi2, Unigene61827\_JingChaoZuZhi2, Unigene62146\_JingChaoZuZhi2, Unigene6245\_JingChaoZuZhi2, Unigene63663\_JingChaoZuZhi2, Unigene63747\_JingChaoZuZhi2, Unigene64104\_JingChaoZuZhi2, Unigene64434\_JingChaoZuZhi2, Unigene64563\_JingChaoZuZhi2, Unigene64839\_JingChaoZuZhi2, Unigene64890\_JingChaoZuZhi2, Unigene65084\_JingChaoZuZhi2, Unigene65663\_JingChaoZuZhi2, Unigene6573\_JingChaoZuZhi2, Unigene6608\_JingChaoZuZhi2, Unigene66217\_JingChaoZuZhi2, Unigene67055\_JingChaoZuZhi2, Unigene67150\_JingChaoZuZhi2, Unigene67946\_JingChaoZuZhi2, Unigene68331\_JingChaoZuZhi2, Unigene6856\_JingChaoZuZhi2, Unigene68814\_JingChaoZuZhi2, Unigene69178\_JingChaoZuZhi2, Unigene6931\_JingChaoZuZhi2, Unigene70217\_JingChaoZuZhi2, Unigene7059\_JingChaoZuZhi2, Unigene70995\_JingChaoZuZhi2, Unigene71230\_JingChaoZuZhi2, Unigene71248\_JingChaoZuZhi2, Unigene72266\_JingChaoZuZhi2, Unigene72924\_JingChaoZuZhi2, Unigene73054\_JingChaoZuZhi2, Unigene735\_JingChaoZuZhi2, Unigene74009\_JingChaoZuZhi2, Unigene74269\_JingChaoZuZhi2, Unigene74479\_JingChaoZuZhi2, Unigene7457\_JingChaoZuZhi2, Unigene75050\_JingChaoZuZhi2, Unigene75238\_JingChaoZuZhi2, Unigene75580\_JingChaoZuZhi2, Unigene757\_JingChaoZuZhi2, Unigene76437\_JingChaoZuZhi2, Unigene76927\_JingChaoZuZhi2, Unigene77192\_JingChaoZuZhi2, Unigene77543\_JingChaoZuZhi2, Unigene77799\_JingChaoZuZhi2, Unigene77903\_JingChaoZuZhi2, Unigene78602\_JingChaoZuZhi2, Unigene78720\_JingChaoZuZhi2, Unigene78818\_JingChaoZuZhi2, Unigene79284\_JingChaoZuZhi2, Unigene79288\_JingChaoZuZhi2, Unigene79315\_JingChaoZuZhi2, Unigene79613\_JingChaoZuZhi2, Unigene80149\_JingChaoZuZhi2, Unigene80358\_JingChaoZuZhi2, Unigene80848\_JingChaoZuZhi2, Unigene81253\_JingChaoZuZhi2, Unigene81418\_JingChaoZuZhi2, Unigene814\_JingChaoZuZhi2, Unigene81654\_JingChaoZuZhi2, Unigene81661\_JingChaoZuZhi2, Unigene81754\_JingChaoZuZhi2, Unigene81902\_JingChaoZuZhi2, Unigene81908\_JingChaoZuZhi2, Unigene82055\_JingChaoZuZhi2, Unigene82258\_JingChaoZuZhi2, Unigene82339\_JingChaoZuZhi2, Unigene83023\_JingChaoZuZhi2, Unigene83138\_JingChaoZuZhi2, Unigene83373\_JingChaoZuZhi2, Unigene83523\_JingChaoZuZhi2, Unigene83572\_JingChaoZuZhi2, Unigene83760\_JingChaoZuZhi2, Unigene83852\_JingChaoZuZhi2, Unigene84154\_JingChaoZuZhi2, Unigene84323\_JingChaoZuZhi2, Unigene8505\_JingChaoZuZhi2, Unigene85353\_JingChaoZuZhi2, Unigene85560\_JingChaoZuZhi2, Unigene85841\_JingChaoZuZhi2, Unigene85892\_JingChaoZuZhi2, Unigene86041\_JingChaoZuZhi2, Unigene8607\_JingChaoZuZhi2, Unigene86148\_JingChaoZuZhi2, Unigene86368\_JingChaoZuZhi2, Unigene86427\_JingChaoZuZhi2, Unigene86909\_JingChaoZuZhi2, Unigene87007\_JingChaoZuZhi2, Unigene87046\_JingChaoZuZhi2, Unigene87062\_JingChaoZuZhi2, Unigene87341\_JingChaoZuZhi2, Unigene87347\_JingChaoZuZhi2, Unigene87474\_JingChaoZuZhi2, Unigene88047\_JingChaoZuZhi2, Unigene88158\_JingChaoZuZhi2, Unigene88229\_JingChaoZuZhi2, Unigene88528\_JingChaoZuZhi2, Unigene88586\_JingChaoZuZhi2, Unigene88876\_JingChaoZuZhi2, Unigene88891\_JingChaoZuZhi2, Unigene88909\_JingChaoZuZhi2, Unigene89128\_JingChaoZuZhi2, Unigene89232\_JingChaoZuZhi2, Unigene89255\_JingChaoZuZhi2, Unigene89414\_JingChaoZuZhi2, Unigene89454\_JingChaoZuZhi2, Unigene89629\_JingChaoZuZhi2, Unigene89751\_JingChaoZuZhi2, Unigene89882\_JingChaoZuZhi2, Unigene90093\_JingChaoZuZhi2, Unigene90531\_JingChaoZuZhi2, Unigene90812\_JingChaoZuZhi2, Unigene9095\_JingChaoZuZhi2, Unigene91205\_JingChaoZuZhi2, Unigene91227\_JingChaoZuZhi2, Unigene91303\_JingChaoZuZhi2, Unigene91324\_JingChaoZuZhi2, Unigene91441\_JingChaoZuZhi2, Unigene91460\_JingChaoZuZhi2, Unigene91508\_JingChaoZuZhi2, Unigene91666\_JingChaoZuZhi2, Unigene91720\_JingChaoZuZhi2, Unigene91723\_JingChaoZuZhi2, Unigene91925\_JingChaoZuZhi2, Unigene92103\_JingChaoZuZhi2, Unigene92149\_JingChaoZuZhi2, Unigene92183\_JingChaoZuZhi2, Unigene92363\_JingChaoZuZhi2, Unigene92418\_JingChaoZuZhi2, Unigene92452\_JingChaoZuZhi2, Unigene92470\_JingChaoZuZhi2, Unigene92579\_JingChaoZuZhi2, Unigene9279\_JingChaoZuZhi2, Unigene92949\_JingChaoZuZhi2, Unigene92981\_JingChaoZuZhi2, Unigene93025\_JingChaoZuZhi2, Unigene93136\_JingChaoZuZhi2, Unigene93154\_JingChaoZuZhi2, Unigene93182\_JingChaoZuZhi2, Unigene93338\_JingChaoZuZhi2, Unigene93425\_JingChaoZuZhi2, Unigene93453\_JingChaoZuZhi2, Unigene93611\_JingChaoZuZhi2, Unigene93785\_JingChaoZuZhi2, Unigene93795\_JingChaoZuZhi2, Unigene93837\_JingChaoZuZhi2, Unigene93843\_JingChaoZuZhi2, Unigene93936\_JingChaoZuZhi2, Unigene94040\_JingChaoZuZhi2, Unigene94215\_JingChaoZuZhi2, Unigene9422\_JingChaoZuZhi2, Unigene94286\_JingChaoZuZhi2, Unigene94337\_JingChaoZuZhi2, Unigene94398\_JingChaoZuZhi2, Unigene94428\_JingChaoZuZhi2, Unigene94552\_JingChaoZuZhi2, Unigene94775\_JingChaoZuZhi2, Unigene94997\_JingChaoZuZhi2, Unigene95197\_JingChaoZuZhi2, Unigene95275\_JingChaoZuZhi2, Unigene95331\_JingChaoZuZhi2, Unigene95395\_JingChaoZuZhi2, Unigene95404\_JingChaoZuZhi2, Unigene95477\_JingChaoZuZhi2, Unigene95550\_JingChaoZuZhi2, Unigene95599\_JingChaoZuZhi2, Unigene95664\_JingChaoZuZhi2, Unigene95688\_JingChaoZuZhi2, Unigene95745\_JingChaoZuZhi2, Unigene95780\_JingChaoZuZhi2, Unigene95789\_JingChaoZuZhi2, Unigene95911\_JingChaoZuZhi2, Unigene96023\_JingChaoZuZhi2, Unigene96039\_JingChaoZuZhi2, Unigene96068\_JingChaoZuZhi2, Unigene96114\_JingChaoZuZhi2, Unigene96163\_JingChaoZuZhi2, Unigene96223\_JingChaoZuZhi2, Unigene96248\_JingChaoZuZhi2, Unigene96278\_JingChaoZuZhi2 |
| 29 | Wnt signaling pathway | Unigene1001\_JingChaoZuZhi2, Unigene10031\_JingChaoZuZhi2, Unigene10073\_JingChaoZuZhi2, Unigene10290\_JingChaoZuZhi2, Unigene10318\_JingChaoZuZhi2, Unigene10922\_JingChaoZuZhi2, Unigene1109\_JingChaoZuZhi2, Unigene11240\_JingChaoZuZhi2, Unigene11287\_JingChaoZuZhi2, Unigene11575\_JingChaoZuZhi2, Unigene11732\_JingChaoZuZhi2, Unigene11951\_JingChaoZuZhi2, Unigene12060\_JingChaoZuZhi2, Unigene12161\_JingChaoZuZhi2, Unigene12171\_JingChaoZuZhi2, Unigene12214\_JingChaoZuZhi2, Unigene12455\_JingChaoZuZhi2, Unigene12584\_JingChaoZuZhi2, Unigene12634\_JingChaoZuZhi2, Unigene12809\_JingChaoZuZhi2, Unigene1301\_JingChaoZuZhi2, Unigene13869\_JingChaoZuZhi2, Unigene14181\_JingChaoZuZhi2, Unigene14317\_JingChaoZuZhi2, Unigene14360\_JingChaoZuZhi2, Unigene14370\_JingChaoZuZhi2, Unigene14488\_JingChaoZuZhi2, Unigene14635\_JingChaoZuZhi2, Unigene14874\_JingChaoZuZhi2, Unigene14973\_JingChaoZuZhi2, Unigene14990\_JingChaoZuZhi2, Unigene15104\_JingChaoZuZhi2, Unigene15525\_JingChaoZuZhi2, Unigene16039\_JingChaoZuZhi2, Unigene1609\_JingChaoZuZhi2, Unigene16116\_JingChaoZuZhi2, Unigene16154\_JingChaoZuZhi2, Unigene16229\_JingChaoZuZhi2, Unigene16468\_JingChaoZuZhi2, Unigene16521\_JingChaoZuZhi2, Unigene16589\_JingChaoZuZhi2, Unigene16820\_JingChaoZuZhi2, Unigene16933\_JingChaoZuZhi2, Unigene16958\_JingChaoZuZhi2, Unigene16991\_JingChaoZuZhi2, Unigene17136\_JingChaoZuZhi2, Unigene17168\_JingChaoZuZhi2, Unigene17710\_JingChaoZuZhi2, Unigene17800\_JingChaoZuZhi2, Unigene18095\_JingChaoZuZhi2, Unigene18167\_JingChaoZuZhi2, Unigene18177\_JingChaoZuZhi2, Unigene18203\_JingChaoZuZhi2, Unigene18307\_JingChaoZuZhi2, Unigene18715\_JingChaoZuZhi2, Unigene1872\_JingChaoZuZhi2, Unigene18817\_JingChaoZuZhi2, Unigene18904\_JingChaoZuZhi2, Unigene18932\_JingChaoZuZhi2, Unigene19021\_JingChaoZuZhi2, Unigene19036\_JingChaoZuZhi2, Unigene19238\_JingChaoZuZhi2, Unigene19403\_JingChaoZuZhi2, Unigene1942\_JingChaoZuZhi2, Unigene19465\_JingChaoZuZhi2, Unigene19481\_JingChaoZuZhi2, Unigene19531\_JingChaoZuZhi2, Unigene1993\_JingChaoZuZhi2, Unigene20005\_JingChaoZuZhi2, Unigene20052\_JingChaoZuZhi2, Unigene20220\_JingChaoZuZhi2, Unigene20256\_JingChaoZuZhi2, Unigene20489\_JingChaoZuZhi2, Unigene20608\_JingChaoZuZhi2, Unigene20649\_JingChaoZuZhi2, Unigene20674\_JingChaoZuZhi2, Unigene20812\_JingChaoZuZhi2, Unigene21144\_JingChaoZuZhi2, Unigene21246\_JingChaoZuZhi2, Unigene21348\_JingChaoZuZhi2, Unigene21578\_JingChaoZuZhi2, Unigene21737\_JingChaoZuZhi2, Unigene21815\_JingChaoZuZhi2, Unigene21973\_JingChaoZuZhi2, Unigene22102\_JingChaoZuZhi2, Unigene22107\_JingChaoZuZhi2, Unigene22110\_JingChaoZuZhi2, Unigene22262\_JingChaoZuZhi2, Unigene22386\_JingChaoZuZhi2, Unigene22482\_JingChaoZuZhi2, Unigene22487\_JingChaoZuZhi2, Unigene22506\_JingChaoZuZhi2, Unigene22580\_JingChaoZuZhi2, Unigene22648\_JingChaoZuZhi2, Unigene22673\_JingChaoZuZhi2, Unigene22708\_JingChaoZuZhi2, Unigene22779\_JingChaoZuZhi2, Unigene22787\_JingChaoZuZhi2, Unigene22934\_JingChaoZuZhi2, Unigene23122\_JingChaoZuZhi2, Unigene2473\_JingChaoZuZhi2, Unigene24841\_JingChaoZuZhi2, Unigene24881\_JingChaoZuZhi2, Unigene25825\_JingChaoZuZhi2, Unigene25966\_JingChaoZuZhi2, Unigene26183\_JingChaoZuZhi2, Unigene2628\_JingChaoZuZhi2, Unigene26730\_JingChaoZuZhi2, Unigene277\_JingChaoZuZhi2, Unigene2960\_JingChaoZuZhi2, Unigene3016\_JingChaoZuZhi2, Unigene30787\_JingChaoZuZhi2, Unigene3185\_JingChaoZuZhi2, Unigene33683\_JingChaoZuZhi2, Unigene34463\_JingChaoZuZhi2, Unigene34574\_JingChaoZuZhi2, Unigene34594\_JingChaoZuZhi2, Unigene3466\_JingChaoZuZhi2, Unigene34723\_JingChaoZuZhi2, Unigene35288\_JingChaoZuZhi2, Unigene35457\_JingChaoZuZhi2, Unigene35730\_JingChaoZuZhi2, Unigene35744\_JingChaoZuZhi2, Unigene36144\_JingChaoZuZhi2, Unigene3671\_JingChaoZuZhi2, Unigene36730\_JingChaoZuZhi2, Unigene37739\_JingChaoZuZhi2, Unigene38191\_JingChaoZuZhi2, Unigene38493\_JingChaoZuZhi2, Unigene39072\_JingChaoZuZhi2, Unigene39147\_JingChaoZuZhi2, Unigene39539\_JingChaoZuZhi2, Unigene39584\_JingChaoZuZhi2, Unigene395\_JingChaoZuZhi2, Unigene39825\_JingChaoZuZhi2, Unigene40020\_JingChaoZuZhi2, Unigene40191\_JingChaoZuZhi2, Unigene40727\_JingChaoZuZhi2, Unigene40825\_JingChaoZuZhi2, Unigene41333\_JingChaoZuZhi2, Unigene41550\_JingChaoZuZhi2, Unigene41809\_JingChaoZuZhi2, Unigene42332\_JingChaoZuZhi2, Unigene4294\_JingChaoZuZhi2, Unigene4299\_JingChaoZuZhi2, Unigene43249\_JingChaoZuZhi2, Unigene44008\_JingChaoZuZhi2, Unigene44228\_JingChaoZuZhi2, Unigene44314\_JingChaoZuZhi2, Unigene45166\_JingChaoZuZhi2, Unigene45\_JingChaoZuZhi2, Unigene46046\_JingChaoZuZhi2, Unigene46240\_JingChaoZuZhi2, Unigene46470\_JingChaoZuZhi2, Unigene46682\_JingChaoZuZhi2, Unigene47546\_JingChaoZuZhi2, Unigene4803\_JingChaoZuZhi2, Unigene48368\_JingChaoZuZhi2, Unigene4853\_JingChaoZuZhi2, Unigene48607\_JingChaoZuZhi2, Unigene4875\_JingChaoZuZhi2, Unigene48850\_JingChaoZuZhi2, Unigene4886\_JingChaoZuZhi2, Unigene49922\_JingChaoZuZhi2, Unigene50235\_JingChaoZuZhi2, Unigene50408\_JingChaoZuZhi2, Unigene51098\_JingChaoZuZhi2, Unigene51619\_JingChaoZuZhi2, Unigene51632\_JingChaoZuZhi2, Unigene5226\_JingChaoZuZhi2, Unigene5314\_JingChaoZuZhi2, Unigene53421\_JingChaoZuZhi2, Unigene54367\_JingChaoZuZhi2, Unigene54698\_JingChaoZuZhi2, Unigene54904\_JingChaoZuZhi2, Unigene55118\_JingChaoZuZhi2, Unigene55392\_JingChaoZuZhi2, Unigene55739\_JingChaoZuZhi2, Unigene56709\_JingChaoZuZhi2, Unigene56922\_JingChaoZuZhi2, Unigene58474\_JingChaoZuZhi2, Unigene58806\_JingChaoZuZhi2, Unigene59068\_JingChaoZuZhi2, Unigene59122\_JingChaoZuZhi2, Unigene601\_JingChaoZuZhi2, Unigene60453\_JingChaoZuZhi2, Unigene60777\_JingChaoZuZhi2, Unigene61043\_JingChaoZuZhi2, Unigene61108\_JingChaoZuZhi2, Unigene61406\_JingChaoZuZhi2, Unigene61911\_JingChaoZuZhi2, Unigene62218\_JingChaoZuZhi2, Unigene62340\_JingChaoZuZhi2, Unigene62870\_JingChaoZuZhi2, Unigene63323\_JingChaoZuZhi2, Unigene63406\_JingChaoZuZhi2, Unigene63449\_JingChaoZuZhi2, Unigene64164\_JingChaoZuZhi2, Unigene64227\_JingChaoZuZhi2, Unigene64678\_JingChaoZuZhi2, Unigene6467\_JingChaoZuZhi2, Unigene64830\_JingChaoZuZhi2, Unigene65445\_JingChaoZuZhi2, Unigene65785\_JingChaoZuZhi2, Unigene66540\_JingChaoZuZhi2, Unigene66865\_JingChaoZuZhi2, Unigene67177\_JingChaoZuZhi2, Unigene6752\_JingChaoZuZhi2, Unigene67541\_JingChaoZuZhi2, Unigene67788\_JingChaoZuZhi2, Unigene67838\_JingChaoZuZhi2, Unigene6785\_JingChaoZuZhi2, Unigene6798\_JingChaoZuZhi2, Unigene68056\_JingChaoZuZhi2, Unigene68298\_JingChaoZuZhi2, Unigene69817\_JingChaoZuZhi2, Unigene70087\_JingChaoZuZhi2, Unigene70723\_JingChaoZuZhi2, Unigene72003\_JingChaoZuZhi2, Unigene72033\_JingChaoZuZhi2, Unigene722\_JingChaoZuZhi2, Unigene72769\_JingChaoZuZhi2, Unigene72809\_JingChaoZuZhi2, Unigene73203\_JingChaoZuZhi2, Unigene73814\_JingChaoZuZhi2, Unigene73873\_JingChaoZuZhi2, Unigene7398\_JingChaoZuZhi2, Unigene7417\_JingChaoZuZhi2, Unigene74609\_JingChaoZuZhi2, Unigene7464\_JingChaoZuZhi2, Unigene75228\_JingChaoZuZhi2, Unigene75347\_JingChaoZuZhi2, Unigene76575\_JingChaoZuZhi2, Unigene76614\_JingChaoZuZhi2, Unigene76639\_JingChaoZuZhi2, Unigene77149\_JingChaoZuZhi2, Unigene77587\_JingChaoZuZhi2, Unigene77748\_JingChaoZuZhi2, Unigene78078\_JingChaoZuZhi2, Unigene78168\_JingChaoZuZhi2, Unigene78235\_JingChaoZuZhi2, Unigene78241\_JingChaoZuZhi2, Unigene78390\_JingChaoZuZhi2, Unigene78522\_JingChaoZuZhi2, Unigene79118\_JingChaoZuZhi2, Unigene8003\_JingChaoZuZhi2, Unigene80068\_JingChaoZuZhi2, Unigene80123\_JingChaoZuZhi2, Unigene8054\_JingChaoZuZhi2, Unigene80699\_JingChaoZuZhi2, Unigene80927\_JingChaoZuZhi2, Unigene81086\_JingChaoZuZhi2, Unigene81687\_JingChaoZuZhi2, Unigene81716\_JingChaoZuZhi2, Unigene82144\_JingChaoZuZhi2, Unigene82219\_JingChaoZuZhi2, Unigene82340\_JingChaoZuZhi2, Unigene82359\_JingChaoZuZhi2, Unigene82525\_JingChaoZuZhi2, Unigene82675\_JingChaoZuZhi2, Unigene83188\_JingChaoZuZhi2, Unigene83532\_JingChaoZuZhi2, Unigene83537\_JingChaoZuZhi2, Unigene84080\_JingChaoZuZhi2, Unigene84930\_JingChaoZuZhi2, Unigene8507\_JingChaoZuZhi2, Unigene8523\_JingChaoZuZhi2, Unigene86119\_JingChaoZuZhi2, Unigene86234\_JingChaoZuZhi2, Unigene86504\_JingChaoZuZhi2, Unigene86789\_JingChaoZuZhi2, Unigene87050\_JingChaoZuZhi2, Unigene87332\_JingChaoZuZhi2, Unigene87607\_JingChaoZuZhi2, Unigene87654\_JingChaoZuZhi2, Unigene8799\_JingChaoZuZhi2, Unigene88167\_JingChaoZuZhi2, Unigene88385\_JingChaoZuZhi2, Unigene88544\_JingChaoZuZhi2, Unigene88719\_JingChaoZuZhi2, Unigene88773\_JingChaoZuZhi2, Unigene88797\_JingChaoZuZhi2, Unigene89093\_JingChaoZuZhi2, Unigene89161\_JingChaoZuZhi2, Unigene89194\_JingChaoZuZhi2, Unigene89215\_JingChaoZuZhi2, Unigene89310\_JingChaoZuZhi2, Unigene8949\_JingChaoZuZhi2, Unigene89544\_JingChaoZuZhi2, Unigene89970\_JingChaoZuZhi2, Unigene90183\_JingChaoZuZhi2, Unigene90283\_JingChaoZuZhi2, Unigene90454\_JingChaoZuZhi2, Unigene90761\_JingChaoZuZhi2, Unigene90762\_JingChaoZuZhi2, Unigene90941\_JingChaoZuZhi2, Unigene91021\_JingChaoZuZhi2, Unigene91100\_JingChaoZuZhi2, Unigene91119\_JingChaoZuZhi2, Unigene91179\_JingChaoZuZhi2, Unigene91527\_JingChaoZuZhi2, Unigene91739\_JingChaoZuZhi2, Unigene92019\_JingChaoZuZhi2, Unigene92309\_JingChaoZuZhi2, Unigene92576\_JingChaoZuZhi2, Unigene92651\_JingChaoZuZhi2, Unigene92715\_JingChaoZuZhi2, Unigene92800\_JingChaoZuZhi2, Unigene93133\_JingChaoZuZhi2, Unigene93195\_JingChaoZuZhi2, Unigene93245\_JingChaoZuZhi2, Unigene93288\_JingChaoZuZhi2, Unigene93479\_JingChaoZuZhi2, Unigene93754\_JingChaoZuZhi2, Unigene93821\_JingChaoZuZhi2, Unigene93912\_JingChaoZuZhi2, Unigene94045\_JingChaoZuZhi2, Unigene94242\_JingChaoZuZhi2, Unigene94272\_JingChaoZuZhi2, Unigene9428\_JingChaoZuZhi2, Unigene94388\_JingChaoZuZhi2, Unigene9445\_JingChaoZuZhi2, Unigene94668\_JingChaoZuZhi2, Unigene9470\_JingChaoZuZhi2, Unigene94711\_JingChaoZuZhi2, Unigene94749\_JingChaoZuZhi2, Unigene94795\_JingChaoZuZhi2, Unigene94819\_JingChaoZuZhi2, Unigene95014\_JingChaoZuZhi2, Unigene95058\_JingChaoZuZhi2, Unigene95098\_JingChaoZuZhi2, Unigene95147\_JingChaoZuZhi2, Unigene9517\_JingChaoZuZhi2, Unigene95204\_JingChaoZuZhi2, Unigene95295\_JingChaoZuZhi2, Unigene95315\_JingChaoZuZhi2, Unigene95373\_JingChaoZuZhi2, Unigene95393\_JingChaoZuZhi2, Unigene95549\_JingChaoZuZhi2, Unigene95568\_JingChaoZuZhi2, Unigene95677\_JingChaoZuZhi2, Unigene95751\_JingChaoZuZhi2, Unigene95835\_JingChaoZuZhi2, Unigene95863\_JingChaoZuZhi2, Unigene95866\_JingChaoZuZhi2, Unigene95875\_JingChaoZuZhi2, Unigene95881\_JingChaoZuZhi2, Unigene95883\_JingChaoZuZhi2, Unigene96124\_JingChaoZuZhi2, Unigene96234\_JingChaoZuZhi2, Unigene9645\_JingChaoZuZhi2 |
| 30 | Salivary secretion | Unigene10129\_JingChaoZuZhi2, Unigene10208\_JingChaoZuZhi2, Unigene10318\_JingChaoZuZhi2, Unigene10503\_JingChaoZuZhi2, Unigene10538\_JingChaoZuZhi2, Unigene10562\_JingChaoZuZhi2, Unigene10574\_JingChaoZuZhi2, Unigene10689\_JingChaoZuZhi2, Unigene10905\_JingChaoZuZhi2, Unigene10931\_JingChaoZuZhi2, Unigene1154\_JingChaoZuZhi2, Unigene11605\_JingChaoZuZhi2, Unigene11726\_JingChaoZuZhi2, Unigene11825\_JingChaoZuZhi2, Unigene11918\_JingChaoZuZhi2, Unigene12073\_JingChaoZuZhi2, Unigene12098\_JingChaoZuZhi2, Unigene12140\_JingChaoZuZhi2, Unigene12148\_JingChaoZuZhi2, Unigene12311\_JingChaoZuZhi2, Unigene12326\_JingChaoZuZhi2, Unigene12338\_JingChaoZuZhi2, Unigene1233\_JingChaoZuZhi2, Unigene12530\_JingChaoZuZhi2, Unigene12584\_JingChaoZuZhi2, Unigene12784\_JingChaoZuZhi2, Unigene12850\_JingChaoZuZhi2, Unigene12938\_JingChaoZuZhi2, Unigene13085\_JingChaoZuZhi2, Unigene13417\_JingChaoZuZhi2, Unigene13430\_JingChaoZuZhi2, Unigene13485\_JingChaoZuZhi2, Unigene13770\_JingChaoZuZhi2, Unigene13806\_JingChaoZuZhi2, Unigene14138\_JingChaoZuZhi2, Unigene14144\_JingChaoZuZhi2, Unigene14164\_JingChaoZuZhi2, Unigene14252\_JingChaoZuZhi2, Unigene14289\_JingChaoZuZhi2, Unigene14360\_JingChaoZuZhi2, Unigene14475\_JingChaoZuZhi2, Unigene14567\_JingChaoZuZhi2, Unigene14845\_JingChaoZuZhi2, Unigene14914\_JingChaoZuZhi2, Unigene15146\_JingChaoZuZhi2, Unigene15279\_JingChaoZuZhi2, Unigene15406\_JingChaoZuZhi2, Unigene15490\_JingChaoZuZhi2, Unigene15626\_JingChaoZuZhi2, Unigene15651\_JingChaoZuZhi2, Unigene16210\_JingChaoZuZhi2, Unigene16388\_JingChaoZuZhi2, Unigene16514\_JingChaoZuZhi2, Unigene16566\_JingChaoZuZhi2, Unigene16651\_JingChaoZuZhi2, Unigene16729\_JingChaoZuZhi2, Unigene16759\_JingChaoZuZhi2, Unigene167\_JingChaoZuZhi2, Unigene16991\_JingChaoZuZhi2, Unigene17065\_JingChaoZuZhi2, Unigene17462\_JingChaoZuZhi2, Unigene17513\_JingChaoZuZhi2, Unigene17518\_JingChaoZuZhi2, Unigene17605\_JingChaoZuZhi2, Unigene17735\_JingChaoZuZhi2, Unigene17744\_JingChaoZuZhi2, Unigene17809\_JingChaoZuZhi2, Unigene17813\_JingChaoZuZhi2, Unigene18006\_JingChaoZuZhi2, Unigene18081\_JingChaoZuZhi2, Unigene18101\_JingChaoZuZhi2, Unigene18307\_JingChaoZuZhi2, Unigene18334\_JingChaoZuZhi2, Unigene18604\_JingChaoZuZhi2, Unigene18704\_JingChaoZuZhi2, Unigene18713\_JingChaoZuZhi2, Unigene18897\_JingChaoZuZhi2, Unigene19238\_JingChaoZuZhi2, Unigene19663\_JingChaoZuZhi2, Unigene19679\_JingChaoZuZhi2, Unigene19812\_JingChaoZuZhi2, Unigene19862\_JingChaoZuZhi2, Unigene20048\_JingChaoZuZhi2, Unigene20060\_JingChaoZuZhi2, Unigene20223\_JingChaoZuZhi2, Unigene20243\_JingChaoZuZhi2, Unigene20356\_JingChaoZuZhi2, Unigene20474\_JingChaoZuZhi2, Unigene20805\_JingChaoZuZhi2, Unigene20873\_JingChaoZuZhi2, Unigene21026\_JingChaoZuZhi2, Unigene21028\_JingChaoZuZhi2, Unigene21137\_JingChaoZuZhi2, Unigene21460\_JingChaoZuZhi2, Unigene21578\_JingChaoZuZhi2, Unigene21703\_JingChaoZuZhi2, Unigene21713\_JingChaoZuZhi2, Unigene21755\_JingChaoZuZhi2, Unigene21792\_JingChaoZuZhi2, Unigene21815\_JingChaoZuZhi2, Unigene21866\_JingChaoZuZhi2, Unigene21938\_JingChaoZuZhi2, Unigene21940\_JingChaoZuZhi2, Unigene21946\_JingChaoZuZhi2, Unigene22061\_JingChaoZuZhi2, Unigene22120\_JingChaoZuZhi2, Unigene22228\_JingChaoZuZhi2, Unigene22377\_JingChaoZuZhi2, Unigene22401\_JingChaoZuZhi2, Unigene22510\_JingChaoZuZhi2, Unigene22530\_JingChaoZuZhi2, Unigene22779\_JingChaoZuZhi2, Unigene22852\_JingChaoZuZhi2, Unigene22885\_JingChaoZuZhi2, Unigene24425\_JingChaoZuZhi2, Unigene24506\_JingChaoZuZhi2, Unigene2519\_JingChaoZuZhi2, Unigene2544\_JingChaoZuZhi2, Unigene26730\_JingChaoZuZhi2, Unigene28522\_JingChaoZuZhi2, Unigene2960\_JingChaoZuZhi2, Unigene2965\_JingChaoZuZhi2, Unigene3022\_JingChaoZuZhi2, Unigene3122\_JingChaoZuZhi2, Unigene31660\_JingChaoZuZhi2, Unigene32126\_JingChaoZuZhi2, Unigene32827\_JingChaoZuZhi2, Unigene3292\_JingChaoZuZhi2, Unigene32999\_JingChaoZuZhi2, Unigene33142\_JingChaoZuZhi2, Unigene33441\_JingChaoZuZhi2, Unigene3345\_JingChaoZuZhi2, Unigene34426\_JingChaoZuZhi2, Unigene34429\_JingChaoZuZhi2, Unigene34929\_JingChaoZuZhi2, Unigene3557\_JingChaoZuZhi2, Unigene38950\_JingChaoZuZhi2, Unigene39742\_JingChaoZuZhi2, Unigene39832\_JingChaoZuZhi2, Unigene40335\_JingChaoZuZhi2, Unigene41333\_JingChaoZuZhi2, Unigene41447\_JingChaoZuZhi2, Unigene42033\_JingChaoZuZhi2, Unigene43059\_JingChaoZuZhi2, Unigene4449\_JingChaoZuZhi2, Unigene44625\_JingChaoZuZhi2, Unigene47543\_JingChaoZuZhi2, Unigene49203\_JingChaoZuZhi2, Unigene51024\_JingChaoZuZhi2, Unigene51991\_JingChaoZuZhi2, Unigene5229\_JingChaoZuZhi2, Unigene53284\_JingChaoZuZhi2, Unigene53774\_JingChaoZuZhi2, Unigene53935\_JingChaoZuZhi2, Unigene54012\_JingChaoZuZhi2, Unigene5423\_JingChaoZuZhi2, Unigene54367\_JingChaoZuZhi2, Unigene54449\_JingChaoZuZhi2, Unigene55\_JingChaoZuZhi2, Unigene56368\_JingChaoZuZhi2, Unigene56513\_JingChaoZuZhi2, Unigene5674\_JingChaoZuZhi2, Unigene57184\_JingChaoZuZhi2, Unigene57185\_JingChaoZuZhi2, Unigene5756\_JingChaoZuZhi2, Unigene5782\_JingChaoZuZhi2, Unigene5799\_JingChaoZuZhi2, Unigene59102\_JingChaoZuZhi2, Unigene5986\_JingChaoZuZhi2, Unigene60121\_JingChaoZuZhi2, Unigene60582\_JingChaoZuZhi2, Unigene60800\_JingChaoZuZhi2, Unigene6088\_JingChaoZuZhi2, Unigene61189\_JingChaoZuZhi2, Unigene61421\_JingChaoZuZhi2, Unigene62059\_JingChaoZuZhi2, Unigene62376\_JingChaoZuZhi2, Unigene62590\_JingChaoZuZhi2, Unigene6262\_JingChaoZuZhi2, Unigene6305\_JingChaoZuZhi2, Unigene6393\_JingChaoZuZhi2, Unigene63994\_JingChaoZuZhi2, Unigene64164\_JingChaoZuZhi2, Unigene64830\_JingChaoZuZhi2, Unigene65445\_JingChaoZuZhi2, Unigene66073\_JingChaoZuZhi2, Unigene66540\_JingChaoZuZhi2, Unigene66806\_JingChaoZuZhi2, Unigene6735\_JingChaoZuZhi2, Unigene68097\_JingChaoZuZhi2, Unigene68356\_JingChaoZuZhi2, Unigene68510\_JingChaoZuZhi2, Unigene6865\_JingChaoZuZhi2, Unigene69551\_JingChaoZuZhi2, Unigene69910\_JingChaoZuZhi2, Unigene70368\_JingChaoZuZhi2, Unigene70493\_JingChaoZuZhi2, Unigene70723\_JingChaoZuZhi2, Unigene71043\_JingChaoZuZhi2, Unigene7116\_JingChaoZuZhi2, Unigene71365\_JingChaoZuZhi2, Unigene71385\_JingChaoZuZhi2, Unigene71408\_JingChaoZuZhi2, Unigene71558\_JingChaoZuZhi2, Unigene7186\_JingChaoZuZhi2, Unigene72015\_JingChaoZuZhi2, Unigene72643\_JingChaoZuZhi2, Unigene72978\_JingChaoZuZhi2, Unigene73632\_JingChaoZuZhi2, Unigene7398\_JingChaoZuZhi2, Unigene7412\_JingChaoZuZhi2, Unigene74199\_JingChaoZuZhi2, Unigene7456\_JingChaoZuZhi2, Unigene75235\_JingChaoZuZhi2, Unigene75316\_JingChaoZuZhi2, Unigene75634\_JingChaoZuZhi2, Unigene76546\_JingChaoZuZhi2, Unigene76602\_JingChaoZuZhi2, Unigene7663\_JingChaoZuZhi2, Unigene76694\_JingChaoZuZhi2, Unigene76800\_JingChaoZuZhi2, Unigene77149\_JingChaoZuZhi2, Unigene77255\_JingChaoZuZhi2, Unigene7731\_JingChaoZuZhi2, Unigene77520\_JingChaoZuZhi2, Unigene7762\_JingChaoZuZhi2, Unigene78007\_JingChaoZuZhi2, Unigene78078\_JingChaoZuZhi2, Unigene78235\_JingChaoZuZhi2, Unigene78398\_JingChaoZuZhi2, Unigene78490\_JingChaoZuZhi2, Unigene78784\_JingChaoZuZhi2, Unigene78942\_JingChaoZuZhi2, Unigene79595\_JingChaoZuZhi2, Unigene79649\_JingChaoZuZhi2, Unigene79769\_JingChaoZuZhi2, Unigene80070\_JingChaoZuZhi2, Unigene80412\_JingChaoZuZhi2, Unigene81101\_JingChaoZuZhi2, Unigene81437\_JingChaoZuZhi2, Unigene81692\_JingChaoZuZhi2, Unigene82364\_JingChaoZuZhi2, Unigene82565\_JingChaoZuZhi2, Unigene82597\_JingChaoZuZhi2, Unigene82629\_JingChaoZuZhi2, Unigene82932\_JingChaoZuZhi2, Unigene83154\_JingChaoZuZhi2, Unigene83526\_JingChaoZuZhi2, Unigene83602\_JingChaoZuZhi2, Unigene83898\_JingChaoZuZhi2, Unigene83899\_JingChaoZuZhi2, Unigene84161\_JingChaoZuZhi2, Unigene84232\_JingChaoZuZhi2, Unigene84247\_JingChaoZuZhi2, Unigene84671\_JingChaoZuZhi2, Unigene84829\_JingChaoZuZhi2, Unigene85059\_JingChaoZuZhi2, Unigene85076\_JingChaoZuZhi2, Unigene85463\_JingChaoZuZhi2, Unigene85503\_JingChaoZuZhi2, Unigene85692\_JingChaoZuZhi2, Unigene85781\_JingChaoZuZhi2, Unigene8598\_JingChaoZuZhi2, Unigene86243\_JingChaoZuZhi2, Unigene86359\_JingChaoZuZhi2, Unigene86552\_JingChaoZuZhi2, Unigene86848\_JingChaoZuZhi2, Unigene87332\_JingChaoZuZhi2, Unigene87485\_JingChaoZuZhi2, Unigene87607\_JingChaoZuZhi2, Unigene87738\_JingChaoZuZhi2, Unigene87818\_JingChaoZuZhi2, Unigene87968\_JingChaoZuZhi2, Unigene87992\_JingChaoZuZhi2, Unigene88006\_JingChaoZuZhi2, Unigene88283\_JingChaoZuZhi2, Unigene88385\_JingChaoZuZhi2, Unigene88439\_JingChaoZuZhi2, Unigene88594\_JingChaoZuZhi2, Unigene88724\_JingChaoZuZhi2, Unigene88773\_JingChaoZuZhi2, Unigene89147\_JingChaoZuZhi2, Unigene89309\_JingChaoZuZhi2, Unigene89352\_JingChaoZuZhi2, Unigene89432\_JingChaoZuZhi2, Unigene89603\_JingChaoZuZhi2, Unigene89807\_JingChaoZuZhi2, Unigene89819\_JingChaoZuZhi2, Unigene8996\_JingChaoZuZhi2, Unigene90470\_JingChaoZuZhi2, Unigene90529\_JingChaoZuZhi2, Unigene90682\_JingChaoZuZhi2, Unigene90749\_JingChaoZuZhi2, Unigene909\_JingChaoZuZhi2, Unigene91350\_JingChaoZuZhi2, Unigene91456\_JingChaoZuZhi2, Unigene91527\_JingChaoZuZhi2, Unigene91592\_JingChaoZuZhi2, Unigene91686\_JingChaoZuZhi2, Unigene91739\_JingChaoZuZhi2, Unigene91848\_JingChaoZuZhi2, Unigene91924\_JingChaoZuZhi2, Unigene91953\_JingChaoZuZhi2, Unigene92245\_JingChaoZuZhi2, Unigene92397\_JingChaoZuZhi2, Unigene92529\_JingChaoZuZhi2, Unigene92918\_JingChaoZuZhi2, Unigene92942\_JingChaoZuZhi2, Unigene93012\_JingChaoZuZhi2, Unigene93296\_JingChaoZuZhi2, Unigene93343\_JingChaoZuZhi2, Unigene93588\_JingChaoZuZhi2, Unigene93713\_JingChaoZuZhi2, Unigene93714\_JingChaoZuZhi2, Unigene93742\_JingChaoZuZhi2, Unigene93838\_JingChaoZuZhi2, Unigene93874\_JingChaoZuZhi2, Unigene93923\_JingChaoZuZhi2, Unigene93949\_JingChaoZuZhi2, Unigene94168\_JingChaoZuZhi2, Unigene94222\_JingChaoZuZhi2, Unigene94266\_JingChaoZuZhi2, Unigene94279\_JingChaoZuZhi2, Unigene94939\_JingChaoZuZhi2, Unigene95008\_JingChaoZuZhi2, Unigene95035\_JingChaoZuZhi2, Unigene95205\_JingChaoZuZhi2, Unigene95367\_JingChaoZuZhi2, Unigene95510\_JingChaoZuZhi2, Unigene95613\_JingChaoZuZhi2, Unigene95673\_JingChaoZuZhi2, Unigene95677\_JingChaoZuZhi2, Unigene95776\_JingChaoZuZhi2, Unigene95821\_JingChaoZuZhi2, Unigene95899\_JingChaoZuZhi2, Unigene95914\_JingChaoZuZhi2, Unigene95916\_JingChaoZuZhi2, Unigene95944\_JingChaoZuZhi2, Unigene95995\_JingChaoZuZhi2, Unigene96038\_JingChaoZuZhi2, Unigene96045\_JingChaoZuZhi2, Unigene96056\_JingChaoZuZhi2, Unigene96153\_JingChaoZuZhi2, Unigene96206\_JingChaoZuZhi2, Unigene96262\_JingChaoZuZhi2, Unigene96278\_JingChaoZuZhi2, Unigene9633\_JingChaoZuZhi2, Unigene9654\_JingChaoZuZhi2, Unigene9992\_JingChaoZuZhi2 |
| 31 | Amyotrophic lateral sclerosis (ALS) | Unigene10042\_JingChaoZuZhi2, Unigene10392\_JingChaoZuZhi2, Unigene10475\_JingChaoZuZhi2, Unigene10616\_JingChaoZuZhi2, Unigene10747\_JingChaoZuZhi2, Unigene11723\_JingChaoZuZhi2, Unigene12236\_JingChaoZuZhi2, Unigene12264\_JingChaoZuZhi2, Unigene1244\_JingChaoZuZhi2, Unigene1247\_JingChaoZuZhi2, Unigene12617\_JingChaoZuZhi2, Unigene12748\_JingChaoZuZhi2, Unigene1295\_JingChaoZuZhi2, Unigene12\_JingChaoZuZhi2, Unigene13178\_JingChaoZuZhi2, Unigene13241\_JingChaoZuZhi2, Unigene13379\_JingChaoZuZhi2, Unigene1356\_JingChaoZuZhi2, Unigene13584\_JingChaoZuZhi2, Unigene13717\_JingChaoZuZhi2, Unigene13733\_JingChaoZuZhi2, Unigene14172\_JingChaoZuZhi2, Unigene14367\_JingChaoZuZhi2, Unigene14370\_JingChaoZuZhi2, Unigene14458\_JingChaoZuZhi2, Unigene1459\_JingChaoZuZhi2, Unigene14672\_JingChaoZuZhi2, Unigene15196\_JingChaoZuZhi2, Unigene1522\_JingChaoZuZhi2, Unigene15648\_JingChaoZuZhi2, Unigene15658\_JingChaoZuZhi2, Unigene1565\_JingChaoZuZhi2, Unigene15681\_JingChaoZuZhi2, Unigene15808\_JingChaoZuZhi2, Unigene15893\_JingChaoZuZhi2, Unigene15933\_JingChaoZuZhi2, Unigene16041\_JingChaoZuZhi2, Unigene16101\_JingChaoZuZhi2, Unigene16102\_JingChaoZuZhi2, Unigene16118\_JingChaoZuZhi2, Unigene16169\_JingChaoZuZhi2, Unigene16236\_JingChaoZuZhi2, Unigene16257\_JingChaoZuZhi2, Unigene16299\_JingChaoZuZhi2, Unigene1637\_JingChaoZuZhi2, Unigene16478\_JingChaoZuZhi2, Unigene16524\_JingChaoZuZhi2, Unigene16821\_JingChaoZuZhi2, Unigene17058\_JingChaoZuZhi2, Unigene17231\_JingChaoZuZhi2, Unigene17232\_JingChaoZuZhi2, Unigene17310\_JingChaoZuZhi2, Unigene1742\_JingChaoZuZhi2, Unigene17795\_JingChaoZuZhi2, Unigene18489\_JingChaoZuZhi2, Unigene18551\_JingChaoZuZhi2, Unigene18567\_JingChaoZuZhi2, Unigene1856\_JingChaoZuZhi2, Unigene18618\_JingChaoZuZhi2, Unigene18647\_JingChaoZuZhi2, Unigene18944\_JingChaoZuZhi2, Unigene19021\_JingChaoZuZhi2, Unigene1902\_JingChaoZuZhi2, Unigene19249\_JingChaoZuZhi2, Unigene19426\_JingChaoZuZhi2, Unigene19508\_JingChaoZuZhi2, Unigene19528\_JingChaoZuZhi2, Unigene19545\_JingChaoZuZhi2, Unigene19770\_JingChaoZuZhi2, Unigene19986\_JingChaoZuZhi2, Unigene20056\_JingChaoZuZhi2, Unigene20091\_JingChaoZuZhi2, Unigene20120\_JingChaoZuZhi2, Unigene20282\_JingChaoZuZhi2, Unigene20312\_JingChaoZuZhi2, Unigene20349\_JingChaoZuZhi2, Unigene20707\_JingChaoZuZhi2, Unigene20740\_JingChaoZuZhi2, Unigene20900\_JingChaoZuZhi2, Unigene21107\_JingChaoZuZhi2, Unigene21166\_JingChaoZuZhi2, Unigene21246\_JingChaoZuZhi2, Unigene21305\_JingChaoZuZhi2, Unigene21341\_JingChaoZuZhi2, Unigene21366\_JingChaoZuZhi2, Unigene21498\_JingChaoZuZhi2, Unigene21516\_JingChaoZuZhi2, Unigene21528\_JingChaoZuZhi2, Unigene21542\_JingChaoZuZhi2, Unigene21651\_JingChaoZuZhi2, Unigene21737\_JingChaoZuZhi2, Unigene21794\_JingChaoZuZhi2, Unigene217\_JingChaoZuZhi2, Unigene2186\_JingChaoZuZhi2, Unigene21905\_JingChaoZuZhi2, Unigene21919\_JingChaoZuZhi2, Unigene21931\_JingChaoZuZhi2, Unigene22097\_JingChaoZuZhi2, Unigene22273\_JingChaoZuZhi2, Unigene22350\_JingChaoZuZhi2, Unigene22387\_JingChaoZuZhi2, Unigene22429\_JingChaoZuZhi2, Unigene22469\_JingChaoZuZhi2, Unigene22475\_JingChaoZuZhi2, Unigene22698\_JingChaoZuZhi2, Unigene22720\_JingChaoZuZhi2, Unigene22757\_JingChaoZuZhi2, Unigene22791\_JingChaoZuZhi2, Unigene22807\_JingChaoZuZhi2, Unigene22857\_JingChaoZuZhi2, Unigene22902\_JingChaoZuZhi2, Unigene22910\_JingChaoZuZhi2, Unigene22927\_JingChaoZuZhi2, Unigene23874\_JingChaoZuZhi2, Unigene2701\_JingChaoZuZhi2, Unigene2720\_JingChaoZuZhi2, Unigene278\_JingChaoZuZhi2, Unigene28398\_JingChaoZuZhi2, Unigene2916\_JingChaoZuZhi2, Unigene3055\_JingChaoZuZhi2, Unigene31790\_JingChaoZuZhi2, Unigene31857\_JingChaoZuZhi2, Unigene31886\_JingChaoZuZhi2, Unigene32139\_JingChaoZuZhi2, Unigene32505\_JingChaoZuZhi2, Unigene3333\_JingChaoZuZhi2, Unigene33536\_JingChaoZuZhi2, Unigene33784\_JingChaoZuZhi2, Unigene33839\_JingChaoZuZhi2, Unigene339\_JingChaoZuZhi2, Unigene34768\_JingChaoZuZhi2, Unigene34989\_JingChaoZuZhi2, Unigene35551\_JingChaoZuZhi2, Unigene35755\_JingChaoZuZhi2, Unigene35977\_JingChaoZuZhi2, Unigene36999\_JingChaoZuZhi2, Unigene37009\_JingChaoZuZhi2, Unigene37034\_JingChaoZuZhi2, Unigene37143\_JingChaoZuZhi2, Unigene37623\_JingChaoZuZhi2, Unigene37739\_JingChaoZuZhi2, Unigene38900\_JingChaoZuZhi2, Unigene39259\_JingChaoZuZhi2, Unigene39658\_JingChaoZuZhi2, Unigene40292\_JingChaoZuZhi2, Unigene416\_JingChaoZuZhi2, Unigene4226\_JingChaoZuZhi2, Unigene42566\_JingChaoZuZhi2, Unigene42575\_JingChaoZuZhi2, Unigene43582\_JingChaoZuZhi2, Unigene43699\_JingChaoZuZhi2, Unigene4584\_JingChaoZuZhi2, Unigene45\_JingChaoZuZhi2, Unigene4643\_JingChaoZuZhi2, Unigene48647\_JingChaoZuZhi2, Unigene48857\_JingChaoZuZhi2, Unigene49294\_JingChaoZuZhi2, Unigene4957\_JingChaoZuZhi2, Unigene50026\_JingChaoZuZhi2, Unigene50069\_JingChaoZuZhi2, Unigene50503\_JingChaoZuZhi2, Unigene50505\_JingChaoZuZhi2, Unigene51527\_JingChaoZuZhi2, Unigene51663\_JingChaoZuZhi2, Unigene51753\_JingChaoZuZhi2, Unigene52694\_JingChaoZuZhi2, Unigene53103\_JingChaoZuZhi2, Unigene53310\_JingChaoZuZhi2, Unigene535\_JingChaoZuZhi2, Unigene54569\_JingChaoZuZhi2, Unigene545\_JingChaoZuZhi2, Unigene54994\_JingChaoZuZhi2, Unigene56189\_JingChaoZuZhi2, Unigene5619\_JingChaoZuZhi2, Unigene57037\_JingChaoZuZhi2, Unigene57641\_JingChaoZuZhi2, Unigene58091\_JingChaoZuZhi2, Unigene58275\_JingChaoZuZhi2, Unigene58527\_JingChaoZuZhi2, Unigene59373\_JingChaoZuZhi2, Unigene6001\_JingChaoZuZhi2, Unigene60081\_JingChaoZuZhi2, Unigene60350\_JingChaoZuZhi2, Unigene607\_JingChaoZuZhi2, Unigene61742\_JingChaoZuZhi2, Unigene6179\_JingChaoZuZhi2, Unigene62148\_JingChaoZuZhi2, Unigene63091\_JingChaoZuZhi2, Unigene63145\_JingChaoZuZhi2, Unigene6321\_JingChaoZuZhi2, Unigene6331\_JingChaoZuZhi2, Unigene63829\_JingChaoZuZhi2, Unigene63990\_JingChaoZuZhi2, Unigene64236\_JingChaoZuZhi2, Unigene64497\_JingChaoZuZhi2, Unigene6459\_JingChaoZuZhi2, Unigene64839\_JingChaoZuZhi2, Unigene648\_JingChaoZuZhi2, Unigene65096\_JingChaoZuZhi2, Unigene65178\_JingChaoZuZhi2, Unigene654\_JingChaoZuZhi2, Unigene6567\_JingChaoZuZhi2, Unigene66116\_JingChaoZuZhi2, Unigene67112\_JingChaoZuZhi2, Unigene67488\_JingChaoZuZhi2, Unigene68097\_JingChaoZuZhi2, Unigene68298\_JingChaoZuZhi2, Unigene6908\_JingChaoZuZhi2, Unigene70590\_JingChaoZuZhi2, Unigene7192\_JingChaoZuZhi2, Unigene728\_JingChaoZuZhi2, Unigene73401\_JingChaoZuZhi2, Unigene73435\_JingChaoZuZhi2, Unigene74142\_JingChaoZuZhi2, Unigene74166\_JingChaoZuZhi2, Unigene74597\_JingChaoZuZhi2, Unigene76058\_JingChaoZuZhi2, Unigene77433\_JingChaoZuZhi2, Unigene78045\_JingChaoZuZhi2, Unigene78191\_JingChaoZuZhi2, Unigene78243\_JingChaoZuZhi2, Unigene78400\_JingChaoZuZhi2, Unigene78587\_JingChaoZuZhi2, Unigene79152\_JingChaoZuZhi2, Unigene7932\_JingChaoZuZhi2, Unigene79388\_JingChaoZuZhi2, Unigene7972\_JingChaoZuZhi2, Unigene80688\_JingChaoZuZhi2, Unigene80765\_JingChaoZuZhi2, Unigene80914\_JingChaoZuZhi2, Unigene80919\_JingChaoZuZhi2, Unigene81055\_JingChaoZuZhi2, Unigene81154\_JingChaoZuZhi2, Unigene81166\_JingChaoZuZhi2, Unigene81236\_JingChaoZuZhi2, Unigene81959\_JingChaoZuZhi2, Unigene81961\_JingChaoZuZhi2, Unigene82101\_JingChaoZuZhi2, Unigene82219\_JingChaoZuZhi2, Unigene82515\_JingChaoZuZhi2, Unigene82742\_JingChaoZuZhi2, Unigene82773\_JingChaoZuZhi2, Unigene83255\_JingChaoZuZhi2, Unigene84077\_JingChaoZuZhi2, Unigene84343\_JingChaoZuZhi2, Unigene84806\_JingChaoZuZhi2, Unigene84930\_JingChaoZuZhi2, Unigene85033\_JingChaoZuZhi2, Unigene85035\_JingChaoZuZhi2, Unigene85541\_JingChaoZuZhi2, Unigene85909\_JingChaoZuZhi2, Unigene86028\_JingChaoZuZhi2, Unigene86124\_JingChaoZuZhi2, Unigene86368\_JingChaoZuZhi2, Unigene86536\_JingChaoZuZhi2, Unigene86613\_JingChaoZuZhi2, Unigene86741\_JingChaoZuZhi2, Unigene86808\_JingChaoZuZhi2, Unigene87416\_JingChaoZuZhi2, Unigene87453\_JingChaoZuZhi2, Unigene87488\_JingChaoZuZhi2, Unigene87541\_JingChaoZuZhi2, Unigene8777\_JingChaoZuZhi2, Unigene87935\_JingChaoZuZhi2, Unigene88232\_JingChaoZuZhi2, Unigene88327\_JingChaoZuZhi2, Unigene88586\_JingChaoZuZhi2, Unigene88602\_JingChaoZuZhi2, Unigene88664\_JingChaoZuZhi2, Unigene88753\_JingChaoZuZhi2, Unigene88844\_JingChaoZuZhi2, Unigene89089\_JingChaoZuZhi2, Unigene89137\_JingChaoZuZhi2, Unigene89299\_JingChaoZuZhi2, Unigene89385\_JingChaoZuZhi2, Unigene89399\_JingChaoZuZhi2, Unigene89495\_JingChaoZuZhi2, Unigene89659\_JingChaoZuZhi2, Unigene89937\_JingChaoZuZhi2, Unigene90083\_JingChaoZuZhi2, Unigene90263\_JingChaoZuZhi2, Unigene90307\_JingChaoZuZhi2, Unigene90526\_JingChaoZuZhi2, Unigene90535\_JingChaoZuZhi2, Unigene90645\_JingChaoZuZhi2, Unigene90835\_JingChaoZuZhi2, Unigene9102\_JingChaoZuZhi2, Unigene91065\_JingChaoZuZhi2, Unigene91179\_JingChaoZuZhi2, Unigene91418\_JingChaoZuZhi2, Unigene91580\_JingChaoZuZhi2, Unigene91624\_JingChaoZuZhi2, Unigene91673\_JingChaoZuZhi2, Unigene91794\_JingChaoZuZhi2, Unigene92294\_JingChaoZuZhi2, Unigene92598\_JingChaoZuZhi2, Unigene92699\_JingChaoZuZhi2, Unigene92709\_JingChaoZuZhi2, Unigene93239\_JingChaoZuZhi2, Unigene93385\_JingChaoZuZhi2, Unigene93415\_JingChaoZuZhi2, Unigene93555\_JingChaoZuZhi2, Unigene93653\_JingChaoZuZhi2, Unigene93748\_JingChaoZuZhi2, Unigene93823\_JingChaoZuZhi2, Unigene93899\_JingChaoZuZhi2, Unigene94020\_JingChaoZuZhi2, Unigene94036\_JingChaoZuZhi2, Unigene9403\_JingChaoZuZhi2, Unigene94113\_JingChaoZuZhi2, Unigene94154\_JingChaoZuZhi2, Unigene94276\_JingChaoZuZhi2, Unigene94442\_JingChaoZuZhi2, Unigene94545\_JingChaoZuZhi2, Unigene94619\_JingChaoZuZhi2, Unigene94726\_JingChaoZuZhi2, Unigene94749\_JingChaoZuZhi2, Unigene94912\_JingChaoZuZhi2, Unigene94979\_JingChaoZuZhi2, Unigene94985\_JingChaoZuZhi2, Unigene95105\_JingChaoZuZhi2, Unigene95172\_JingChaoZuZhi2, Unigene95190\_JingChaoZuZhi2, Unigene95191\_JingChaoZuZhi2, Unigene95207\_JingChaoZuZhi2, Unigene95251\_JingChaoZuZhi2, Unigene95334\_JingChaoZuZhi2, Unigene95385\_JingChaoZuZhi2, Unigene95430\_JingChaoZuZhi2, Unigene95438\_JingChaoZuZhi2, Unigene95581\_JingChaoZuZhi2, Unigene95700\_JingChaoZuZhi2, Unigene95740\_JingChaoZuZhi2, Unigene95766\_JingChaoZuZhi2, Unigene95819\_JingChaoZuZhi2, Unigene95826\_JingChaoZuZhi2, Unigene95830\_JingChaoZuZhi2, Unigene95854\_JingChaoZuZhi2, Unigene95875\_JingChaoZuZhi2, Unigene95890\_JingChaoZuZhi2, Unigene958\_JingChaoZuZhi2, Unigene95994\_JingChaoZuZhi2, Unigene96003\_JingChaoZuZhi2, Unigene96018\_JingChaoZuZhi2, Unigene96133\_JingChaoZuZhi2, Unigene96272\_JingChaoZuZhi2, Unigene96285\_JingChaoZuZhi2, Unigene9883\_JingChaoZuZhi2 |
| 32 | Alzheimer's disease | Unigene10392\_JingChaoZuZhi2, Unigene10716\_JingChaoZuZhi2, Unigene1082\_JingChaoZuZhi2, Unigene1135\_JingChaoZuZhi2, Unigene11372\_JingChaoZuZhi2, Unigene11469\_JingChaoZuZhi2, Unigene11963\_JingChaoZuZhi2, Unigene12073\_JingChaoZuZhi2, Unigene1247\_JingChaoZuZhi2, Unigene12941\_JingChaoZuZhi2, Unigene1333\_JingChaoZuZhi2, Unigene13531\_JingChaoZuZhi2, Unigene13584\_JingChaoZuZhi2, Unigene13803\_JingChaoZuZhi2, Unigene1393\_JingChaoZuZhi2, Unigene14261\_JingChaoZuZhi2, Unigene14360\_JingChaoZuZhi2, Unigene14370\_JingChaoZuZhi2, Unigene14458\_JingChaoZuZhi2, Unigene14522\_JingChaoZuZhi2, Unigene14620\_JingChaoZuZhi2, Unigene14707\_JingChaoZuZhi2, Unigene14729\_JingChaoZuZhi2, Unigene14871\_JingChaoZuZhi2, Unigene15173\_JingChaoZuZhi2, Unigene15236\_JingChaoZuZhi2, Unigene15278\_JingChaoZuZhi2, Unigene1535\_JingChaoZuZhi2, Unigene15867\_JingChaoZuZhi2, Unigene16169\_JingChaoZuZhi2, Unigene16261\_JingChaoZuZhi2, Unigene16317\_JingChaoZuZhi2, Unigene16566\_JingChaoZuZhi2, Unigene16820\_JingChaoZuZhi2, Unigene16991\_JingChaoZuZhi2, Unigene17015\_JingChaoZuZhi2, Unigene17099\_JingChaoZuZhi2, Unigene17151\_JingChaoZuZhi2, Unigene18387\_JingChaoZuZhi2, Unigene18433\_JingChaoZuZhi2, Unigene18475\_JingChaoZuZhi2, Unigene18652\_JingChaoZuZhi2, Unigene19006\_JingChaoZuZhi2, Unigene19213\_JingChaoZuZhi2, Unigene19256\_JingChaoZuZhi2, Unigene19425\_JingChaoZuZhi2, Unigene19597\_JingChaoZuZhi2, Unigene19612\_JingChaoZuZhi2, Unigene19664\_JingChaoZuZhi2, Unigene19670\_JingChaoZuZhi2, Unigene19847\_JingChaoZuZhi2, Unigene20000\_JingChaoZuZhi2, Unigene20343\_JingChaoZuZhi2, Unigene20611\_JingChaoZuZhi2, Unigene20622\_JingChaoZuZhi2, Unigene20760\_JingChaoZuZhi2, Unigene20962\_JingChaoZuZhi2, Unigene21166\_JingChaoZuZhi2, Unigene21222\_JingChaoZuZhi2, Unigene21246\_JingChaoZuZhi2, Unigene2132\_JingChaoZuZhi2, Unigene21354\_JingChaoZuZhi2, Unigene21651\_JingChaoZuZhi2, Unigene21737\_JingChaoZuZhi2, Unigene2178\_JingChaoZuZhi2, Unigene21917\_JingChaoZuZhi2, Unigene21952\_JingChaoZuZhi2, Unigene22763\_JingChaoZuZhi2, Unigene23874\_JingChaoZuZhi2, Unigene24905\_JingChaoZuZhi2, Unigene2720\_JingChaoZuZhi2, Unigene27479\_JingChaoZuZhi2, Unigene28398\_JingChaoZuZhi2, Unigene31886\_JingChaoZuZhi2, Unigene32139\_JingChaoZuZhi2, Unigene32827\_JingChaoZuZhi2, Unigene33536\_JingChaoZuZhi2, Unigene34768\_JingChaoZuZhi2, Unigene34929\_JingChaoZuZhi2, Unigene34989\_JingChaoZuZhi2, Unigene35366\_JingChaoZuZhi2, Unigene35755\_JingChaoZuZhi2, Unigene35952\_JingChaoZuZhi2, Unigene36089\_JingChaoZuZhi2, Unigene36230\_JingChaoZuZhi2, Unigene3632\_JingChaoZuZhi2, Unigene36404\_JingChaoZuZhi2, Unigene37009\_JingChaoZuZhi2, Unigene37143\_JingChaoZuZhi2, Unigene37739\_JingChaoZuZhi2, Unigene38900\_JingChaoZuZhi2, Unigene3903\_JingChaoZuZhi2, Unigene39259\_JingChaoZuZhi2, Unigene39658\_JingChaoZuZhi2, Unigene4001\_JingChaoZuZhi2, Unigene40292\_JingChaoZuZhi2, Unigene40865\_JingChaoZuZhi2, Unigene40890\_JingChaoZuZhi2, Unigene4111\_JingChaoZuZhi2, Unigene41333\_JingChaoZuZhi2, Unigene41683\_JingChaoZuZhi2, Unigene416\_JingChaoZuZhi2, Unigene41982\_JingChaoZuZhi2, Unigene42087\_JingChaoZuZhi2, Unigene42566\_JingChaoZuZhi2, Unigene42575\_JingChaoZuZhi2, Unigene43059\_JingChaoZuZhi2, Unigene43582\_JingChaoZuZhi2, Unigene43699\_JingChaoZuZhi2, Unigene44078\_JingChaoZuZhi2, Unigene4413\_JingChaoZuZhi2, Unigene4418\_JingChaoZuZhi2, Unigene44195\_JingChaoZuZhi2, Unigene45069\_JingChaoZuZhi2, Unigene45216\_JingChaoZuZhi2, Unigene4522\_JingChaoZuZhi2, Unigene45\_JingChaoZuZhi2, Unigene46526\_JingChaoZuZhi2, Unigene4738\_JingChaoZuZhi2, Unigene4782\_JingChaoZuZhi2, Unigene48095\_JingChaoZuZhi2, Unigene48263\_JingChaoZuZhi2, Unigene48576\_JingChaoZuZhi2, Unigene48647\_JingChaoZuZhi2, Unigene48714\_JingChaoZuZhi2, Unigene49294\_JingChaoZuZhi2, Unigene49345\_JingChaoZuZhi2, Unigene4957\_JingChaoZuZhi2, Unigene49977\_JingChaoZuZhi2, Unigene49987\_JingChaoZuZhi2, Unigene50026\_JingChaoZuZhi2, Unigene50405\_JingChaoZuZhi2, Unigene50503\_JingChaoZuZhi2, Unigene51663\_JingChaoZuZhi2, Unigene51994\_JingChaoZuZhi2, Unigene52036\_JingChaoZuZhi2, Unigene52167\_JingChaoZuZhi2, Unigene52383\_JingChaoZuZhi2, Unigene52694\_JingChaoZuZhi2, Unigene53103\_JingChaoZuZhi2, Unigene53295\_JingChaoZuZhi2, Unigene53310\_JingChaoZuZhi2, Unigene54236\_JingChaoZuZhi2, Unigene54367\_JingChaoZuZhi2, Unigene54449\_JingChaoZuZhi2, Unigene54468\_JingChaoZuZhi2, Unigene54994\_JingChaoZuZhi2, Unigene55617\_JingChaoZuZhi2, Unigene55700\_JingChaoZuZhi2, Unigene55738\_JingChaoZuZhi2, Unigene55745\_JingChaoZuZhi2, Unigene5673\_JingChaoZuZhi2, Unigene57037\_JingChaoZuZhi2, Unigene57238\_JingChaoZuZhi2, Unigene57878\_JingChaoZuZhi2, Unigene57909\_JingChaoZuZhi2, Unigene58275\_JingChaoZuZhi2, Unigene58502\_JingChaoZuZhi2, Unigene58527\_JingChaoZuZhi2, Unigene59373\_JingChaoZuZhi2, Unigene59731\_JingChaoZuZhi2, Unigene60081\_JingChaoZuZhi2, Unigene60117\_JingChaoZuZhi2, Unigene60776\_JingChaoZuZhi2, Unigene607\_JingChaoZuZhi2, Unigene60975\_JingChaoZuZhi2, Unigene6166\_JingChaoZuZhi2, Unigene61742\_JingChaoZuZhi2, Unigene62656\_JingChaoZuZhi2, Unigene62979\_JingChaoZuZhi2, Unigene63091\_JingChaoZuZhi2, Unigene63175\_JingChaoZuZhi2, Unigene63341\_JingChaoZuZhi2, Unigene63935\_JingChaoZuZhi2, Unigene63990\_JingChaoZuZhi2, Unigene63994\_JingChaoZuZhi2, Unigene64058\_JingChaoZuZhi2, Unigene64497\_JingChaoZuZhi2, Unigene65081\_JingChaoZuZhi2, Unigene65096\_JingChaoZuZhi2, Unigene65445\_JingChaoZuZhi2, Unigene65807\_JingChaoZuZhi2, Unigene66116\_JingChaoZuZhi2, Unigene66540\_JingChaoZuZhi2, Unigene67304\_JingChaoZuZhi2, Unigene67337\_JingChaoZuZhi2, Unigene6755\_JingChaoZuZhi2, Unigene68097\_JingChaoZuZhi2, Unigene68113\_JingChaoZuZhi2, Unigene68298\_JingChaoZuZhi2, Unigene68698\_JingChaoZuZhi2, Unigene68832\_JingChaoZuZhi2, Unigene69427\_JingChaoZuZhi2, Unigene69817\_JingChaoZuZhi2, Unigene70493\_JingChaoZuZhi2, Unigene7082\_JingChaoZuZhi2, Unigene70920\_JingChaoZuZhi2, Unigene7176\_JingChaoZuZhi2, Unigene7192\_JingChaoZuZhi2, Unigene72170\_JingChaoZuZhi2, Unigene72613\_JingChaoZuZhi2, Unigene72643\_JingChaoZuZhi2, Unigene72813\_JingChaoZuZhi2, Unigene72826\_JingChaoZuZhi2, Unigene73543\_JingChaoZuZhi2, Unigene74597\_JingChaoZuZhi2, Unigene74661\_JingChaoZuZhi2, Unigene76458\_JingChaoZuZhi2, Unigene76567\_JingChaoZuZhi2, Unigene76582\_JingChaoZuZhi2, Unigene76647\_JingChaoZuZhi2, Unigene76896\_JingChaoZuZhi2, Unigene77149\_JingChaoZuZhi2, Unigene77259\_JingChaoZuZhi2, Unigene77631\_JingChaoZuZhi2, Unigene77904\_JingChaoZuZhi2, Unigene78078\_JingChaoZuZhi2, Unigene78435\_JingChaoZuZhi2, Unigene78587\_JingChaoZuZhi2, Unigene78998\_JingChaoZuZhi2, Unigene79210\_JingChaoZuZhi2, Unigene79307\_JingChaoZuZhi2, Unigene79388\_JingChaoZuZhi2, Unigene79523\_JingChaoZuZhi2, Unigene79892\_JingChaoZuZhi2, Unigene80163\_JingChaoZuZhi2, Unigene80347\_JingChaoZuZhi2, Unigene80494\_JingChaoZuZhi2, Unigene80984\_JingChaoZuZhi2, Unigene81395\_JingChaoZuZhi2, Unigene81706\_JingChaoZuZhi2, Unigene8191\_JingChaoZuZhi2, Unigene82186\_JingChaoZuZhi2, Unigene82219\_JingChaoZuZhi2, Unigene82309\_JingChaoZuZhi2, Unigene83101\_JingChaoZuZhi2, Unigene8310\_JingChaoZuZhi2, Unigene83110\_JingChaoZuZhi2, Unigene83327\_JingChaoZuZhi2, Unigene83398\_JingChaoZuZhi2, Unigene83409\_JingChaoZuZhi2, Unigene83448\_JingChaoZuZhi2, Unigene84070\_JingChaoZuZhi2, Unigene84080\_JingChaoZuZhi2, Unigene8415\_JingChaoZuZhi2, Unigene84181\_JingChaoZuZhi2, Unigene84247\_JingChaoZuZhi2, Unigene84343\_JingChaoZuZhi2, Unigene84377\_JingChaoZuZhi2, Unigene84689\_JingChaoZuZhi2, Unigene84790\_JingChaoZuZhi2, Unigene85510\_JingChaoZuZhi2, Unigene86528\_JingChaoZuZhi2, Unigene86536\_JingChaoZuZhi2, Unigene86582\_JingChaoZuZhi2, Unigene86821\_JingChaoZuZhi2, Unigene87015\_JingChaoZuZhi2, Unigene87026\_JingChaoZuZhi2, Unigene87283\_JingChaoZuZhi2, Unigene8728\_JingChaoZuZhi2, Unigene87349\_JingChaoZuZhi2, Unigene87389\_JingChaoZuZhi2, Unigene87433\_JingChaoZuZhi2, Unigene87607\_JingChaoZuZhi2, Unigene87796\_JingChaoZuZhi2, Unigene87948\_JingChaoZuZhi2, Unigene88135\_JingChaoZuZhi2, Unigene8819\_JingChaoZuZhi2, Unigene88312\_JingChaoZuZhi2, Unigene8837\_JingChaoZuZhi2, Unigene88385\_JingChaoZuZhi2, Unigene88393\_JingChaoZuZhi2, Unigene88428\_JingChaoZuZhi2, Unigene88594\_JingChaoZuZhi2, Unigene88719\_JingChaoZuZhi2, Unigene88724\_JingChaoZuZhi2, Unigene88773\_JingChaoZuZhi2, Unigene88933\_JingChaoZuZhi2, Unigene89147\_JingChaoZuZhi2, Unigene89175\_JingChaoZuZhi2, Unigene89210\_JingChaoZuZhi2, Unigene89245\_JingChaoZuZhi2, Unigene89265\_JingChaoZuZhi2, Unigene89310\_JingChaoZuZhi2, Unigene89385\_JingChaoZuZhi2, Unigene89406\_JingChaoZuZhi2, Unigene89462\_JingChaoZuZhi2, Unigene89653\_JingChaoZuZhi2, Unigene89665\_JingChaoZuZhi2, Unigene89670\_JingChaoZuZhi2, Unigene90077\_JingChaoZuZhi2, Unigene90179\_JingChaoZuZhi2, Unigene90348\_JingChaoZuZhi2, Unigene90358\_JingChaoZuZhi2, Unigene90535\_JingChaoZuZhi2, Unigene91051\_JingChaoZuZhi2, Unigene91081\_JingChaoZuZhi2, Unigene91580\_JingChaoZuZhi2, Unigene91739\_JingChaoZuZhi2, Unigene91855\_JingChaoZuZhi2, Unigene91924\_JingChaoZuZhi2, Unigene91973\_JingChaoZuZhi2, Unigene92076\_JingChaoZuZhi2, Unigene92131\_JingChaoZuZhi2, Unigene92137\_JingChaoZuZhi2, Unigene92187\_JingChaoZuZhi2, Unigene92312\_JingChaoZuZhi2, Unigene92321\_JingChaoZuZhi2, Unigene92429\_JingChaoZuZhi2, Unigene92511\_JingChaoZuZhi2, Unigene92539\_JingChaoZuZhi2, Unigene9256\_JingChaoZuZhi2, Unigene92954\_JingChaoZuZhi2, Unigene93012\_JingChaoZuZhi2, Unigene9301\_JingChaoZuZhi2, Unigene93095\_JingChaoZuZhi2, Unigene93178\_JingChaoZuZhi2, Unigene93265\_JingChaoZuZhi2, Unigene93406\_JingChaoZuZhi2, Unigene93581\_JingChaoZuZhi2, Unigene93588\_JingChaoZuZhi2, Unigene93604\_JingChaoZuZhi2, Unigene93701\_JingChaoZuZhi2, Unigene93714\_JingChaoZuZhi2, Unigene94029\_JingChaoZuZhi2, Unigene94276\_JingChaoZuZhi2, Unigene94511\_JingChaoZuZhi2, Unigene94619\_JingChaoZuZhi2, Unigene94749\_JingChaoZuZhi2, Unigene95041\_JingChaoZuZhi2, Unigene95069\_JingChaoZuZhi2, Unigene95098\_JingChaoZuZhi2, Unigene95159\_JingChaoZuZhi2, Unigene95213\_JingChaoZuZhi2, Unigene95295\_JingChaoZuZhi2, Unigene95668\_JingChaoZuZhi2, Unigene9569\_JingChaoZuZhi2, Unigene95742\_JingChaoZuZhi2, Unigene95994\_JingChaoZuZhi2, Unigene96164\_JingChaoZuZhi2, Unigene96262\_JingChaoZuZhi2, Unigene96275\_JingChaoZuZhi2, Unigene9654\_JingChaoZuZhi2, Unigene9973\_JingChaoZuZhi2 |
| 33 | Vascular smooth muscle contraction | Unigene10027\_JingChaoZuZhi2, Unigene10318\_JingChaoZuZhi2, Unigene10628\_JingChaoZuZhi2, Unigene10765\_JingChaoZuZhi2, Unigene11055\_JingChaoZuZhi2, Unigene11112\_JingChaoZuZhi2, Unigene11381\_JingChaoZuZhi2, Unigene11475\_JingChaoZuZhi2, Unigene11605\_JingChaoZuZhi2, Unigene11726\_JingChaoZuZhi2, Unigene12060\_JingChaoZuZhi2, Unigene12073\_JingChaoZuZhi2, Unigene12098\_JingChaoZuZhi2, Unigene12260\_JingChaoZuZhi2, Unigene12899\_JingChaoZuZhi2, Unigene12938\_JingChaoZuZhi2, Unigene1316\_JingChaoZuZhi2, Unigene13241\_JingChaoZuZhi2, Unigene13420\_JingChaoZuZhi2, Unigene13770\_JingChaoZuZhi2, Unigene13869\_JingChaoZuZhi2, Unigene13894\_JingChaoZuZhi2, Unigene14282\_JingChaoZuZhi2, Unigene14357\_JingChaoZuZhi2, Unigene14360\_JingChaoZuZhi2, Unigene14567\_JingChaoZuZhi2, Unigene14682\_JingChaoZuZhi2, Unigene14707\_JingChaoZuZhi2, Unigene14835\_JingChaoZuZhi2, Unigene15030\_JingChaoZuZhi2, Unigene15276\_JingChaoZuZhi2, Unigene15333\_JingChaoZuZhi2, Unigene15345\_JingChaoZuZhi2, Unigene15374\_JingChaoZuZhi2, Unigene15406\_JingChaoZuZhi2, Unigene15425\_JingChaoZuZhi2, Unigene15649\_JingChaoZuZhi2, Unigene15651\_JingChaoZuZhi2, Unigene15942\_JingChaoZuZhi2, Unigene15986\_JingChaoZuZhi2, Unigene16236\_JingChaoZuZhi2, Unigene16427\_JingChaoZuZhi2, Unigene16467\_JingChaoZuZhi2, Unigene1651\_JingChaoZuZhi2, Unigene16566\_JingChaoZuZhi2, Unigene16597\_JingChaoZuZhi2, Unigene16991\_JingChaoZuZhi2, Unigene17809\_JingChaoZuZhi2, Unigene17888\_JingChaoZuZhi2, Unigene18051\_JingChaoZuZhi2, Unigene18260\_JingChaoZuZhi2, Unigene18307\_JingChaoZuZhi2, Unigene18426\_JingChaoZuZhi2, Unigene18438\_JingChaoZuZhi2, Unigene18491\_JingChaoZuZhi2, Unigene18545\_JingChaoZuZhi2, Unigene18699\_JingChaoZuZhi2, Unigene187\_JingChaoZuZhi2, Unigene18892\_JingChaoZuZhi2, Unigene19466\_JingChaoZuZhi2, Unigene19559\_JingChaoZuZhi2, Unigene19904\_JingChaoZuZhi2, Unigene20086\_JingChaoZuZhi2, Unigene20220\_JingChaoZuZhi2, Unigene20461\_JingChaoZuZhi2, Unigene20464\_JingChaoZuZhi2, Unigene20479\_JingChaoZuZhi2, Unigene20580\_JingChaoZuZhi2, Unigene20608\_JingChaoZuZhi2, Unigene20740\_JingChaoZuZhi2, Unigene21056\_JingChaoZuZhi2, Unigene21225\_JingChaoZuZhi2, Unigene21407\_JingChaoZuZhi2, Unigene21487\_JingChaoZuZhi2, Unigene21513\_JingChaoZuZhi2, Unigene21578\_JingChaoZuZhi2, Unigene21809\_JingChaoZuZhi2, Unigene21815\_JingChaoZuZhi2, Unigene22183\_JingChaoZuZhi2, Unigene22184\_JingChaoZuZhi2, Unigene22189\_JingChaoZuZhi2, Unigene22216\_JingChaoZuZhi2, Unigene22243\_JingChaoZuZhi2, Unigene22251\_JingChaoZuZhi2, Unigene22487\_JingChaoZuZhi2, Unigene22551\_JingChaoZuZhi2, Unigene22779\_JingChaoZuZhi2, Unigene22895\_JingChaoZuZhi2, Unigene22896\_JingChaoZuZhi2, Unigene23874\_JingChaoZuZhi2, Unigene2465\_JingChaoZuZhi2, Unigene24951\_JingChaoZuZhi2, Unigene25164\_JingChaoZuZhi2, Unigene26730\_JingChaoZuZhi2, Unigene2904\_JingChaoZuZhi2, Unigene293\_JingChaoZuZhi2, Unigene2960\_JingChaoZuZhi2, Unigene2968\_JingChaoZuZhi2, Unigene30593\_JingChaoZuZhi2, Unigene30852\_JingChaoZuZhi2, Unigene3122\_JingChaoZuZhi2, Unigene3131\_JingChaoZuZhi2, Unigene31790\_JingChaoZuZhi2, Unigene3191\_JingChaoZuZhi2, Unigene3259\_JingChaoZuZhi2, Unigene32827\_JingChaoZuZhi2, Unigene34426\_JingChaoZuZhi2, Unigene34929\_JingChaoZuZhi2, Unigene3550\_JingChaoZuZhi2, Unigene35952\_JingChaoZuZhi2, Unigene38468\_JingChaoZuZhi2, Unigene3912\_JingChaoZuZhi2, Unigene39832\_JingChaoZuZhi2, Unigene40335\_JingChaoZuZhi2, Unigene41333\_JingChaoZuZhi2, Unigene41571\_JingChaoZuZhi2, Unigene42329\_JingChaoZuZhi2, Unigene42798\_JingChaoZuZhi2, Unigene43059\_JingChaoZuZhi2, Unigene43723\_JingChaoZuZhi2, Unigene4449\_JingChaoZuZhi2, Unigene44731\_JingChaoZuZhi2, Unigene45532\_JingChaoZuZhi2, Unigene457\_JingChaoZuZhi2, Unigene46256\_JingChaoZuZhi2, Unigene48280\_JingChaoZuZhi2, Unigene48309\_JingChaoZuZhi2, Unigene4878\_JingChaoZuZhi2, Unigene48962\_JingChaoZuZhi2, Unigene49203\_JingChaoZuZhi2, Unigene49385\_JingChaoZuZhi2, Unigene50563\_JingChaoZuZhi2, Unigene50673\_JingChaoZuZhi2, Unigene5072\_JingChaoZuZhi2, Unigene51024\_JingChaoZuZhi2, Unigene51496\_JingChaoZuZhi2, Unigene51991\_JingChaoZuZhi2, Unigene5204\_JingChaoZuZhi2, Unigene52073\_JingChaoZuZhi2, Unigene52262\_JingChaoZuZhi2, Unigene52554\_JingChaoZuZhi2, Unigene52727\_JingChaoZuZhi2, Unigene53284\_JingChaoZuZhi2, Unigene53935\_JingChaoZuZhi2, Unigene54012\_JingChaoZuZhi2, Unigene54367\_JingChaoZuZhi2, Unigene54449\_JingChaoZuZhi2, Unigene54875\_JingChaoZuZhi2, Unigene54980\_JingChaoZuZhi2, Unigene56456\_JingChaoZuZhi2, Unigene57184\_JingChaoZuZhi2, Unigene57185\_JingChaoZuZhi2, Unigene57200\_JingChaoZuZhi2, Unigene5756\_JingChaoZuZhi2, Unigene57751\_JingChaoZuZhi2, Unigene5823\_JingChaoZuZhi2, Unigene5881\_JingChaoZuZhi2, Unigene59102\_JingChaoZuZhi2, Unigene6028\_JingChaoZuZhi2, Unigene60800\_JingChaoZuZhi2, Unigene61526\_JingChaoZuZhi2, Unigene6174\_JingChaoZuZhi2, Unigene6239\_JingChaoZuZhi2, Unigene62431\_JingChaoZuZhi2, Unigene62552\_JingChaoZuZhi2, Unigene63091\_JingChaoZuZhi2, Unigene6361\_JingChaoZuZhi2, Unigene6382\_JingChaoZuZhi2, Unigene6393\_JingChaoZuZhi2, Unigene63994\_JingChaoZuZhi2, Unigene64164\_JingChaoZuZhi2, Unigene64830\_JingChaoZuZhi2, Unigene65002\_JingChaoZuZhi2, Unigene65445\_JingChaoZuZhi2, Unigene65648\_JingChaoZuZhi2, Unigene66073\_JingChaoZuZhi2, Unigene66540\_JingChaoZuZhi2, Unigene67328\_JingChaoZuZhi2, Unigene6769\_JingChaoZuZhi2, Unigene68151\_JingChaoZuZhi2, Unigene6864\_JingChaoZuZhi2, Unigene69005\_JingChaoZuZhi2, Unigene69910\_JingChaoZuZhi2, Unigene70373\_JingChaoZuZhi2, Unigene70493\_JingChaoZuZhi2, Unigene70549\_JingChaoZuZhi2, Unigene70723\_JingChaoZuZhi2, Unigene71043\_JingChaoZuZhi2, Unigene71247\_JingChaoZuZhi2, Unigene71365\_JingChaoZuZhi2, Unigene71558\_JingChaoZuZhi2, Unigene71662\_JingChaoZuZhi2, Unigene72098\_JingChaoZuZhi2, Unigene72249\_JingChaoZuZhi2, Unigene72643\_JingChaoZuZhi2, Unigene73044\_JingChaoZuZhi2, Unigene7330\_JingChaoZuZhi2, Unigene7456\_JingChaoZuZhi2, Unigene74594\_JingChaoZuZhi2, Unigene74922\_JingChaoZuZhi2, Unigene75235\_JingChaoZuZhi2, Unigene75641\_JingChaoZuZhi2, Unigene76182\_JingChaoZuZhi2, Unigene76303\_JingChaoZuZhi2, Unigene77149\_JingChaoZuZhi2, Unigene77406\_JingChaoZuZhi2, Unigene78007\_JingChaoZuZhi2, Unigene78078\_JingChaoZuZhi2, Unigene78225\_JingChaoZuZhi2, Unigene78235\_JingChaoZuZhi2, Unigene78398\_JingChaoZuZhi2, Unigene78784\_JingChaoZuZhi2, Unigene79274\_JingChaoZuZhi2, Unigene79568\_JingChaoZuZhi2, Unigene79681\_JingChaoZuZhi2, Unigene79829\_JingChaoZuZhi2, Unigene8006\_JingChaoZuZhi2, Unigene80215\_JingChaoZuZhi2, Unigene80251\_JingChaoZuZhi2, Unigene81016\_JingChaoZuZhi2, Unigene81086\_JingChaoZuZhi2, Unigene81101\_JingChaoZuZhi2, Unigene81780\_JingChaoZuZhi2, Unigene81903\_JingChaoZuZhi2, Unigene8191\_JingChaoZuZhi2, Unigene8192\_JingChaoZuZhi2, Unigene82023\_JingChaoZuZhi2, Unigene8261\_JingChaoZuZhi2, Unigene82842\_JingChaoZuZhi2, Unigene83487\_JingChaoZuZhi2, Unigene83663\_JingChaoZuZhi2, Unigene84247\_JingChaoZuZhi2, Unigene84510\_JingChaoZuZhi2, Unigene85059\_JingChaoZuZhi2, Unigene85122\_JingChaoZuZhi2, Unigene85126\_JingChaoZuZhi2, Unigene852\_JingChaoZuZhi2, Unigene85387\_JingChaoZuZhi2, Unigene8598\_JingChaoZuZhi2, Unigene86359\_JingChaoZuZhi2, Unigene86661\_JingChaoZuZhi2, Unigene866\_JingChaoZuZhi2, Unigene86713\_JingChaoZuZhi2, Unigene86759\_JingChaoZuZhi2, Unigene86789\_JingChaoZuZhi2, Unigene86899\_JingChaoZuZhi2, Unigene87305\_JingChaoZuZhi2, Unigene87332\_JingChaoZuZhi2, Unigene87607\_JingChaoZuZhi2, Unigene87677\_JingChaoZuZhi2, Unigene87825\_JingChaoZuZhi2, Unigene87958\_JingChaoZuZhi2, Unigene88068\_JingChaoZuZhi2, Unigene88385\_JingChaoZuZhi2, Unigene88594\_JingChaoZuZhi2, Unigene88724\_JingChaoZuZhi2, Unigene88773\_JingChaoZuZhi2, Unigene88974\_JingChaoZuZhi2, Unigene89147\_JingChaoZuZhi2, Unigene89409\_JingChaoZuZhi2, Unigene89603\_JingChaoZuZhi2, Unigene89807\_JingChaoZuZhi2, Unigene89959\_JingChaoZuZhi2, Unigene9038\_JingChaoZuZhi2, Unigene90401\_JingChaoZuZhi2, Unigene90626\_JingChaoZuZhi2, Unigene90749\_JingChaoZuZhi2, Unigene90767\_JingChaoZuZhi2, Unigene91030\_JingChaoZuZhi2, Unigene91289\_JingChaoZuZhi2, Unigene91527\_JingChaoZuZhi2, Unigene91631\_JingChaoZuZhi2, Unigene91739\_JingChaoZuZhi2, Unigene91794\_JingChaoZuZhi2, Unigene91924\_JingChaoZuZhi2, Unigene91991\_JingChaoZuZhi2, Unigene92141\_JingChaoZuZhi2, Unigene92211\_JingChaoZuZhi2, Unigene92246\_JingChaoZuZhi2, Unigene92329\_JingChaoZuZhi2, Unigene92448\_JingChaoZuZhi2, Unigene92471\_JingChaoZuZhi2, Unigene92577\_JingChaoZuZhi2, Unigene9260\_JingChaoZuZhi2, Unigene92673\_JingChaoZuZhi2, Unigene92737\_JingChaoZuZhi2, Unigene92769\_JingChaoZuZhi2, Unigene92918\_JingChaoZuZhi2, Unigene92929\_JingChaoZuZhi2, Unigene93010\_JingChaoZuZhi2, Unigene93012\_JingChaoZuZhi2, Unigene93279\_JingChaoZuZhi2, Unigene93588\_JingChaoZuZhi2, Unigene93714\_JingChaoZuZhi2, Unigene93821\_JingChaoZuZhi2, Unigene93865\_JingChaoZuZhi2, Unigene9389\_JingChaoZuZhi2, Unigene93990\_JingChaoZuZhi2, Unigene94723\_JingChaoZuZhi2, Unigene94823\_JingChaoZuZhi2, Unigene95031\_JingChaoZuZhi2, Unigene95059\_JingChaoZuZhi2, Unigene95105\_JingChaoZuZhi2, Unigene95205\_JingChaoZuZhi2, Unigene95213\_JingChaoZuZhi2, Unigene95243\_JingChaoZuZhi2, Unigene95359\_JingChaoZuZhi2, Unigene95486\_JingChaoZuZhi2, Unigene95581\_JingChaoZuZhi2, Unigene95613\_JingChaoZuZhi2, Unigene95677\_JingChaoZuZhi2, Unigene95822\_JingChaoZuZhi2, Unigene95879\_JingChaoZuZhi2, Unigene95881\_JingChaoZuZhi2, Unigene95882\_JingChaoZuZhi2, Unigene95914\_JingChaoZuZhi2, Unigene95916\_JingChaoZuZhi2, Unigene95954\_JingChaoZuZhi2, Unigene95972\_JingChaoZuZhi2, Unigene95982\_JingChaoZuZhi2, Unigene95987\_JingChaoZuZhi2, Unigene95994\_JingChaoZuZhi2, Unigene96012\_JingChaoZuZhi2, Unigene96061\_JingChaoZuZhi2, Unigene96083\_JingChaoZuZhi2, Unigene96097\_JingChaoZuZhi2, Unigene96217\_JingChaoZuZhi2, Unigene96243\_JingChaoZuZhi2, Unigene96247\_JingChaoZuZhi2, Unigene96262\_JingChaoZuZhi2, Unigene96288\_JingChaoZuZhi2, Unigene96292\_JingChaoZuZhi2, Unigene96293\_JingChaoZuZhi2, Unigene96303\_JingChaoZuZhi2, Unigene96308\_JingChaoZuZhi2, Unigene9654\_JingChaoZuZhi2, Unigene9902\_JingChaoZuZhi2, Unigene9\_JingChaoZuZhi2 |
| 34 | ECM-receptor interaction | Unigene10058\_JingChaoZuZhi2, Unigene10286\_JingChaoZuZhi2, Unigene10320\_JingChaoZuZhi2, Unigene10362\_JingChaoZuZhi2, Unigene10527\_JingChaoZuZhi2, Unigene10597\_JingChaoZuZhi2, Unigene10630\_JingChaoZuZhi2, Unigene1085\_JingChaoZuZhi2, Unigene10999\_JingChaoZuZhi2, Unigene1111\_JingChaoZuZhi2, Unigene11209\_JingChaoZuZhi2, Unigene11308\_JingChaoZuZhi2, Unigene11446\_JingChaoZuZhi2, Unigene11464\_JingChaoZuZhi2, Unigene11747\_JingChaoZuZhi2, Unigene11919\_JingChaoZuZhi2, Unigene11982\_JingChaoZuZhi2, Unigene12091\_JingChaoZuZhi2, Unigene12376\_JingChaoZuZhi2, Unigene12652\_JingChaoZuZhi2, Unigene12665\_JingChaoZuZhi2, Unigene12850\_JingChaoZuZhi2, Unigene12889\_JingChaoZuZhi2, Unigene12972\_JingChaoZuZhi2, Unigene13078\_JingChaoZuZhi2, Unigene13132\_JingChaoZuZhi2, Unigene13389\_JingChaoZuZhi2, Unigene13390\_JingChaoZuZhi2, Unigene13464\_JingChaoZuZhi2, Unigene1351\_JingChaoZuZhi2, Unigene13607\_JingChaoZuZhi2, Unigene13843\_JingChaoZuZhi2, Unigene13866\_JingChaoZuZhi2, Unigene13925\_JingChaoZuZhi2, Unigene13978\_JingChaoZuZhi2, Unigene14056\_JingChaoZuZhi2, Unigene1413\_JingChaoZuZhi2, Unigene14252\_JingChaoZuZhi2, Unigene14492\_JingChaoZuZhi2, Unigene14948\_JingChaoZuZhi2, Unigene1541\_JingChaoZuZhi2, Unigene15470\_JingChaoZuZhi2, Unigene15649\_JingChaoZuZhi2, Unigene15814\_JingChaoZuZhi2, Unigene15879\_JingChaoZuZhi2, Unigene15883\_JingChaoZuZhi2, Unigene16008\_JingChaoZuZhi2, Unigene16048\_JingChaoZuZhi2, Unigene16072\_JingChaoZuZhi2, Unigene16121\_JingChaoZuZhi2, Unigene1616\_JingChaoZuZhi2, Unigene16240\_JingChaoZuZhi2, Unigene16873\_JingChaoZuZhi2, Unigene16970\_JingChaoZuZhi2, Unigene17064\_JingChaoZuZhi2, Unigene17128\_JingChaoZuZhi2, Unigene17190\_JingChaoZuZhi2, Unigene17226\_JingChaoZuZhi2, Unigene17243\_JingChaoZuZhi2, Unigene17382\_JingChaoZuZhi2, Unigene17505\_JingChaoZuZhi2, Unigene17537\_JingChaoZuZhi2, Unigene17675\_JingChaoZuZhi2, Unigene17754\_JingChaoZuZhi2, Unigene17810\_JingChaoZuZhi2, Unigene17977\_JingChaoZuZhi2, Unigene18107\_JingChaoZuZhi2, Unigene18309\_JingChaoZuZhi2, Unigene18361\_JingChaoZuZhi2, Unigene1859\_JingChaoZuZhi2, Unigene18730\_JingChaoZuZhi2, Unigene18780\_JingChaoZuZhi2, Unigene18808\_JingChaoZuZhi2, Unigene19339\_JingChaoZuZhi2, Unigene19404\_JingChaoZuZhi2, Unigene19562\_JingChaoZuZhi2, Unigene20159\_JingChaoZuZhi2, Unigene20223\_JingChaoZuZhi2, Unigene20255\_JingChaoZuZhi2, Unigene20297\_JingChaoZuZhi2, Unigene20298\_JingChaoZuZhi2, Unigene20367\_JingChaoZuZhi2, Unigene20629\_JingChaoZuZhi2, Unigene20748\_JingChaoZuZhi2, Unigene20824\_JingChaoZuZhi2, Unigene20892\_JingChaoZuZhi2, Unigene20893\_JingChaoZuZhi2, Unigene20914\_JingChaoZuZhi2, Unigene20990\_JingChaoZuZhi2, Unigene21028\_JingChaoZuZhi2, Unigene21253\_JingChaoZuZhi2, Unigene21439\_JingChaoZuZhi2, Unigene21490\_JingChaoZuZhi2, Unigene21571\_JingChaoZuZhi2, Unigene21575\_JingChaoZuZhi2, Unigene21804\_JingChaoZuZhi2, Unigene21888\_JingChaoZuZhi2, Unigene21973\_JingChaoZuZhi2, Unigene22017\_JingChaoZuZhi2, Unigene22245\_JingChaoZuZhi2, Unigene22440\_JingChaoZuZhi2, Unigene22445\_JingChaoZuZhi2, Unigene22464\_JingChaoZuZhi2, Unigene22517\_JingChaoZuZhi2, Unigene22572\_JingChaoZuZhi2, Unigene22624\_JingChaoZuZhi2, Unigene22638\_JingChaoZuZhi2, Unigene22697\_JingChaoZuZhi2, Unigene22700\_JingChaoZuZhi2, Unigene22769\_JingChaoZuZhi2, Unigene22788\_JingChaoZuZhi2, Unigene22794\_JingChaoZuZhi2, Unigene22895\_JingChaoZuZhi2, Unigene22897\_JingChaoZuZhi2, Unigene22917\_JingChaoZuZhi2, Unigene2379\_JingChaoZuZhi2, Unigene24645\_JingChaoZuZhi2, Unigene24772\_JingChaoZuZhi2, Unigene26438\_JingChaoZuZhi2, Unigene27933\_JingChaoZuZhi2, Unigene28208\_JingChaoZuZhi2, Unigene28467\_JingChaoZuZhi2, Unigene29312\_JingChaoZuZhi2, Unigene29488\_JingChaoZuZhi2, Unigene3206\_JingChaoZuZhi2, Unigene328\_JingChaoZuZhi2, Unigene33930\_JingChaoZuZhi2, Unigene3422\_JingChaoZuZhi2, Unigene34807\_JingChaoZuZhi2, Unigene35243\_JingChaoZuZhi2, Unigene35304\_JingChaoZuZhi2, Unigene35624\_JingChaoZuZhi2, Unigene36479\_JingChaoZuZhi2, Unigene3763\_JingChaoZuZhi2, Unigene38238\_JingChaoZuZhi2, Unigene38617\_JingChaoZuZhi2, Unigene39487\_JingChaoZuZhi2, Unigene39674\_JingChaoZuZhi2, Unigene39806\_JingChaoZuZhi2, Unigene40551\_JingChaoZuZhi2, Unigene40763\_JingChaoZuZhi2, Unigene41077\_JingChaoZuZhi2, Unigene41164\_JingChaoZuZhi2, Unigene41209\_JingChaoZuZhi2, Unigene41842\_JingChaoZuZhi2, Unigene42289\_JingChaoZuZhi2, Unigene44141\_JingChaoZuZhi2, Unigene451\_JingChaoZuZhi2, Unigene45399\_JingChaoZuZhi2, Unigene45468\_JingChaoZuZhi2, Unigene45782\_JingChaoZuZhi2, Unigene46628\_JingChaoZuZhi2, Unigene46740\_JingChaoZuZhi2, Unigene47126\_JingChaoZuZhi2, Unigene47566\_JingChaoZuZhi2, Unigene4781\_JingChaoZuZhi2, Unigene4841\_JingChaoZuZhi2, Unigene48673\_JingChaoZuZhi2, Unigene48843\_JingChaoZuZhi2, Unigene48882\_JingChaoZuZhi2, Unigene49253\_JingChaoZuZhi2, Unigene499\_JingChaoZuZhi2, Unigene50282\_JingChaoZuZhi2, Unigene5069\_JingChaoZuZhi2, Unigene51056\_JingChaoZuZhi2, Unigene51231\_JingChaoZuZhi2, Unigene51431\_JingChaoZuZhi2, Unigene51512\_JingChaoZuZhi2, Unigene51994\_JingChaoZuZhi2, Unigene52004\_JingChaoZuZhi2, Unigene5306\_JingChaoZuZhi2, Unigene53503\_JingChaoZuZhi2, Unigene54679\_JingChaoZuZhi2, Unigene55684\_JingChaoZuZhi2, Unigene55693\_JingChaoZuZhi2, Unigene55913\_JingChaoZuZhi2, Unigene56534\_JingChaoZuZhi2, Unigene5695\_JingChaoZuZhi2, Unigene57538\_JingChaoZuZhi2, Unigene5796\_JingChaoZuZhi2, Unigene58405\_JingChaoZuZhi2, Unigene58670\_JingChaoZuZhi2, Unigene59510\_JingChaoZuZhi2, Unigene59797\_JingChaoZuZhi2, Unigene60002\_JingChaoZuZhi2, Unigene60401\_JingChaoZuZhi2, Unigene61033\_JingChaoZuZhi2, Unigene62251\_JingChaoZuZhi2, Unigene62590\_JingChaoZuZhi2, Unigene62878\_JingChaoZuZhi2, Unigene62919\_JingChaoZuZhi2, Unigene63547\_JingChaoZuZhi2, Unigene6392\_JingChaoZuZhi2, Unigene63938\_JingChaoZuZhi2, Unigene64309\_JingChaoZuZhi2, Unigene6433\_JingChaoZuZhi2, Unigene64750\_JingChaoZuZhi2, Unigene64949\_JingChaoZuZhi2, Unigene66700\_JingChaoZuZhi2, Unigene6681\_JingChaoZuZhi2, Unigene6699\_JingChaoZuZhi2, Unigene67052\_JingChaoZuZhi2, Unigene67365\_JingChaoZuZhi2, Unigene67542\_JingChaoZuZhi2, Unigene67811\_JingChaoZuZhi2, Unigene68262\_JingChaoZuZhi2, Unigene68495\_JingChaoZuZhi2, Unigene69445\_JingChaoZuZhi2, Unigene70155\_JingChaoZuZhi2, Unigene7068\_JingChaoZuZhi2, Unigene70696\_JingChaoZuZhi2, Unigene71017\_JingChaoZuZhi2, Unigene71144\_JingChaoZuZhi2, Unigene7186\_JingChaoZuZhi2, Unigene72500\_JingChaoZuZhi2, Unigene72550\_JingChaoZuZhi2, Unigene73571\_JingChaoZuZhi2, Unigene73579\_JingChaoZuZhi2, Unigene7364\_JingChaoZuZhi2, Unigene74199\_JingChaoZuZhi2, Unigene74795\_JingChaoZuZhi2, Unigene75009\_JingChaoZuZhi2, Unigene75341\_JingChaoZuZhi2, Unigene75710\_JingChaoZuZhi2, Unigene75840\_JingChaoZuZhi2, Unigene76415\_JingChaoZuZhi2, Unigene76485\_JingChaoZuZhi2, Unigene76592\_JingChaoZuZhi2, Unigene7670\_JingChaoZuZhi2, Unigene76805\_JingChaoZuZhi2, Unigene77549\_JingChaoZuZhi2, Unigene7793\_JingChaoZuZhi2, Unigene78715\_JingChaoZuZhi2, Unigene79077\_JingChaoZuZhi2, Unigene79109\_JingChaoZuZhi2, Unigene79187\_JingChaoZuZhi2, Unigene79828\_JingChaoZuZhi2, Unigene80038\_JingChaoZuZhi2, Unigene80818\_JingChaoZuZhi2, Unigene8133\_JingChaoZuZhi2, Unigene81483\_JingChaoZuZhi2, Unigene81598\_JingChaoZuZhi2, Unigene81\_JingChaoZuZhi2, Unigene82637\_JingChaoZuZhi2, Unigene82761\_JingChaoZuZhi2, Unigene82872\_JingChaoZuZhi2, Unigene828\_JingChaoZuZhi2, Unigene83345\_JingChaoZuZhi2, Unigene83507\_JingChaoZuZhi2, Unigene83606\_JingChaoZuZhi2, Unigene83744\_JingChaoZuZhi2, Unigene83777\_JingChaoZuZhi2, Unigene84054\_JingChaoZuZhi2, Unigene84257\_JingChaoZuZhi2, Unigene84381\_JingChaoZuZhi2, Unigene84519\_JingChaoZuZhi2, Unigene85066\_JingChaoZuZhi2, Unigene85198\_JingChaoZuZhi2, Unigene85441\_JingChaoZuZhi2, Unigene85503\_JingChaoZuZhi2, Unigene85559\_JingChaoZuZhi2, Unigene85695\_JingChaoZuZhi2, Unigene86186\_JingChaoZuZhi2, Unigene86296\_JingChaoZuZhi2, Unigene87022\_JingChaoZuZhi2, Unigene87180\_JingChaoZuZhi2, Unigene87448\_JingChaoZuZhi2, Unigene87818\_JingChaoZuZhi2, Unigene87850\_JingChaoZuZhi2, Unigene87952\_JingChaoZuZhi2, Unigene88040\_JingChaoZuZhi2, Unigene88050\_JingChaoZuZhi2, Unigene88581\_JingChaoZuZhi2, Unigene88799\_JingChaoZuZhi2, Unigene8891\_JingChaoZuZhi2, Unigene88925\_JingChaoZuZhi2, Unigene88990\_JingChaoZuZhi2, Unigene89090\_JingChaoZuZhi2, Unigene89329\_JingChaoZuZhi2, Unigene89492\_JingChaoZuZhi2, Unigene89710\_JingChaoZuZhi2, Unigene89762\_JingChaoZuZhi2, Unigene90003\_JingChaoZuZhi2, Unigene90078\_JingChaoZuZhi2, Unigene90201\_JingChaoZuZhi2, Unigene90213\_JingChaoZuZhi2, Unigene90261\_JingChaoZuZhi2, Unigene90596\_JingChaoZuZhi2, Unigene90953\_JingChaoZuZhi2, Unigene91043\_JingChaoZuZhi2, Unigene91613\_JingChaoZuZhi2, Unigene91848\_JingChaoZuZhi2, Unigene91983\_JingChaoZuZhi2, Unigene92104\_JingChaoZuZhi2, Unigene92371\_JingChaoZuZhi2, Unigene92671\_JingChaoZuZhi2, Unigene92786\_JingChaoZuZhi2, Unigene92831\_JingChaoZuZhi2, Unigene93022\_JingChaoZuZhi2, Unigene93195\_JingChaoZuZhi2, Unigene93402\_JingChaoZuZhi2, Unigene93501\_JingChaoZuZhi2, Unigene93760\_JingChaoZuZhi2, Unigene93769\_JingChaoZuZhi2, Unigene93857\_JingChaoZuZhi2, Unigene93998\_JingChaoZuZhi2, Unigene9402\_JingChaoZuZhi2, Unigene94204\_JingChaoZuZhi2, Unigene94214\_JingChaoZuZhi2, Unigene94337\_JingChaoZuZhi2, Unigene94446\_JingChaoZuZhi2, Unigene94476\_JingChaoZuZhi2, Unigene94540\_JingChaoZuZhi2, Unigene94561\_JingChaoZuZhi2, Unigene94872\_JingChaoZuZhi2, Unigene94974\_JingChaoZuZhi2, Unigene9520\_JingChaoZuZhi2, Unigene95254\_JingChaoZuZhi2, Unigene95571\_JingChaoZuZhi2, Unigene95699\_JingChaoZuZhi2, Unigene95782\_JingChaoZuZhi2, Unigene95799\_JingChaoZuZhi2, Unigene95821\_JingChaoZuZhi2, Unigene95828\_JingChaoZuZhi2, Unigene95853\_JingChaoZuZhi2, Unigene95889\_JingChaoZuZhi2, Unigene96033\_JingChaoZuZhi2, Unigene96042\_JingChaoZuZhi2, Unigene96057\_JingChaoZuZhi2, Unigene96084\_JingChaoZuZhi2, Unigene96121\_JingChaoZuZhi2, Unigene96271\_JingChaoZuZhi2, Unigene9811\_JingChaoZuZhi2, Unigene9867\_JingChaoZuZhi2, Unigene9892\_JingChaoZuZhi2, Unigene9946\_JingChaoZuZhi2 |
| 35 | Calcium signaling pathway | Unigene10102\_JingChaoZuZhi2, Unigene10284\_JingChaoZuZhi2, Unigene10318\_JingChaoZuZhi2, Unigene10392\_JingChaoZuZhi2, Unigene10422\_JingChaoZuZhi2, Unigene10850\_JingChaoZuZhi2, Unigene11049\_JingChaoZuZhi2, Unigene11074\_JingChaoZuZhi2, Unigene11116\_JingChaoZuZhi2, Unigene11281\_JingChaoZuZhi2, Unigene11964\_JingChaoZuZhi2, Unigene12005\_JingChaoZuZhi2, Unigene12073\_JingChaoZuZhi2, Unigene1247\_JingChaoZuZhi2, Unigene12700\_JingChaoZuZhi2, Unigene13452\_JingChaoZuZhi2, Unigene13584\_JingChaoZuZhi2, Unigene13770\_JingChaoZuZhi2, Unigene13996\_JingChaoZuZhi2, Unigene14360\_JingChaoZuZhi2, Unigene14370\_JingChaoZuZhi2, Unigene14458\_JingChaoZuZhi2, Unigene14600\_JingChaoZuZhi2, Unigene14707\_JingChaoZuZhi2, Unigene15009\_JingChaoZuZhi2, Unigene15181\_JingChaoZuZhi2, Unigene15651\_JingChaoZuZhi2, Unigene16169\_JingChaoZuZhi2, Unigene16266\_JingChaoZuZhi2, Unigene16467\_JingChaoZuZhi2, Unigene16566\_JingChaoZuZhi2, Unigene16646\_JingChaoZuZhi2, Unigene16752\_JingChaoZuZhi2, Unigene16991\_JingChaoZuZhi2, Unigene17462\_JingChaoZuZhi2, Unigene17555\_JingChaoZuZhi2, Unigene17809\_JingChaoZuZhi2, Unigene18205\_JingChaoZuZhi2, Unigene18307\_JingChaoZuZhi2, Unigene18426\_JingChaoZuZhi2, Unigene18464\_JingChaoZuZhi2, Unigene18857\_JingChaoZuZhi2, Unigene18868\_JingChaoZuZhi2, Unigene19458\_JingChaoZuZhi2, Unigene19710\_JingChaoZuZhi2, Unigene19730\_JingChaoZuZhi2, Unigene20000\_JingChaoZuZhi2, Unigene20379\_JingChaoZuZhi2, Unigene20959\_JingChaoZuZhi2, Unigene20992\_JingChaoZuZhi2, Unigene21142\_JingChaoZuZhi2, Unigene21166\_JingChaoZuZhi2, Unigene21246\_JingChaoZuZhi2, Unigene21295\_JingChaoZuZhi2, Unigene21354\_JingChaoZuZhi2, Unigene21578\_JingChaoZuZhi2, Unigene21651\_JingChaoZuZhi2, Unigene21737\_JingChaoZuZhi2, Unigene21815\_JingChaoZuZhi2, Unigene21946\_JingChaoZuZhi2, Unigene22426\_JingChaoZuZhi2, Unigene22779\_JingChaoZuZhi2, Unigene22848\_JingChaoZuZhi2, Unigene2370\_JingChaoZuZhi2, Unigene23874\_JingChaoZuZhi2, Unigene24506\_JingChaoZuZhi2, Unigene2544\_JingChaoZuZhi2, Unigene26730\_JingChaoZuZhi2, Unigene2720\_JingChaoZuZhi2, Unigene2790\_JingChaoZuZhi2, Unigene28398\_JingChaoZuZhi2, Unigene2899\_JingChaoZuZhi2, Unigene2960\_JingChaoZuZhi2, Unigene3122\_JingChaoZuZhi2, Unigene31255\_JingChaoZuZhi2, Unigene31886\_JingChaoZuZhi2, Unigene32139\_JingChaoZuZhi2, Unigene32827\_JingChaoZuZhi2, Unigene33536\_JingChaoZuZhi2, Unigene34212\_JingChaoZuZhi2, Unigene34426\_JingChaoZuZhi2, Unigene34724\_JingChaoZuZhi2, Unigene34768\_JingChaoZuZhi2, Unigene34929\_JingChaoZuZhi2, Unigene34989\_JingChaoZuZhi2, Unigene3550\_JingChaoZuZhi2, Unigene35755\_JingChaoZuZhi2, Unigene35952\_JingChaoZuZhi2, Unigene36404\_JingChaoZuZhi2, Unigene36619\_JingChaoZuZhi2, Unigene37009\_JingChaoZuZhi2, Unigene37143\_JingChaoZuZhi2, Unigene37675\_JingChaoZuZhi2, Unigene37739\_JingChaoZuZhi2, Unigene3807\_JingChaoZuZhi2, Unigene38900\_JingChaoZuZhi2, Unigene38950\_JingChaoZuZhi2, Unigene39002\_JingChaoZuZhi2, Unigene39259\_JingChaoZuZhi2, Unigene39405\_JingChaoZuZhi2, Unigene39658\_JingChaoZuZhi2, Unigene39742\_JingChaoZuZhi2, Unigene39832\_JingChaoZuZhi2, Unigene40143\_JingChaoZuZhi2, Unigene40292\_JingChaoZuZhi2, Unigene40335\_JingChaoZuZhi2, Unigene41333\_JingChaoZuZhi2, Unigene41571\_JingChaoZuZhi2, Unigene416\_JingChaoZuZhi2, Unigene42566\_JingChaoZuZhi2, Unigene42575\_JingChaoZuZhi2, Unigene43059\_JingChaoZuZhi2, Unigene43328\_JingChaoZuZhi2, Unigene43582\_JingChaoZuZhi2, Unigene43699\_JingChaoZuZhi2, Unigene4522\_JingChaoZuZhi2, Unigene4539\_JingChaoZuZhi2, Unigene45\_JingChaoZuZhi2, Unigene47810\_JingChaoZuZhi2, Unigene4782\_JingChaoZuZhi2, Unigene47926\_JingChaoZuZhi2, Unigene48280\_JingChaoZuZhi2, Unigene48499\_JingChaoZuZhi2, Unigene48576\_JingChaoZuZhi2, Unigene48647\_JingChaoZuZhi2, Unigene49203\_JingChaoZuZhi2, Unigene49294\_JingChaoZuZhi2, Unigene49472\_JingChaoZuZhi2, Unigene4957\_JingChaoZuZhi2, Unigene49798\_JingChaoZuZhi2, Unigene49963\_JingChaoZuZhi2, Unigene50026\_JingChaoZuZhi2, Unigene50503\_JingChaoZuZhi2, Unigene51024\_JingChaoZuZhi2, Unigene5113\_JingChaoZuZhi2, Unigene51496\_JingChaoZuZhi2, Unigene51663\_JingChaoZuZhi2, Unigene5173\_JingChaoZuZhi2, Unigene51991\_JingChaoZuZhi2, Unigene5204\_JingChaoZuZhi2, Unigene52167\_JingChaoZuZhi2, Unigene52262\_JingChaoZuZhi2, Unigene52353\_JingChaoZuZhi2, Unigene52694\_JingChaoZuZhi2, Unigene52727\_JingChaoZuZhi2, Unigene53031\_JingChaoZuZhi2, Unigene53078\_JingChaoZuZhi2, Unigene53103\_JingChaoZuZhi2, Unigene53310\_JingChaoZuZhi2, Unigene53935\_JingChaoZuZhi2, Unigene54012\_JingChaoZuZhi2, Unigene54367\_JingChaoZuZhi2, Unigene54449\_JingChaoZuZhi2, Unigene54470\_JingChaoZuZhi2, Unigene54994\_JingChaoZuZhi2, Unigene55303\_JingChaoZuZhi2, Unigene56089\_JingChaoZuZhi2, Unigene56567\_JingChaoZuZhi2, Unigene56709\_JingChaoZuZhi2, Unigene57037\_JingChaoZuZhi2, Unigene57112\_JingChaoZuZhi2, Unigene57127\_JingChaoZuZhi2, Unigene57185\_JingChaoZuZhi2, Unigene57337\_JingChaoZuZhi2, Unigene5756\_JingChaoZuZhi2, Unigene57848\_JingChaoZuZhi2, Unigene58029\_JingChaoZuZhi2, Unigene58275\_JingChaoZuZhi2, Unigene58527\_JingChaoZuZhi2, Unigene59270\_JingChaoZuZhi2, Unigene59373\_JingChaoZuZhi2, Unigene59973\_JingChaoZuZhi2, Unigene60081\_JingChaoZuZhi2, Unigene607\_JingChaoZuZhi2, Unigene6151\_JingChaoZuZhi2, Unigene61742\_JingChaoZuZhi2, Unigene62059\_JingChaoZuZhi2, Unigene62246\_JingChaoZuZhi2, Unigene62431\_JingChaoZuZhi2, Unigene62702\_JingChaoZuZhi2, Unigene62776\_JingChaoZuZhi2, Unigene62786\_JingChaoZuZhi2, Unigene63091\_JingChaoZuZhi2, Unigene63982\_JingChaoZuZhi2, Unigene63990\_JingChaoZuZhi2, Unigene63994\_JingChaoZuZhi2, Unigene64164\_JingChaoZuZhi2, Unigene64497\_JingChaoZuZhi2, Unigene64615\_JingChaoZuZhi2, Unigene64830\_JingChaoZuZhi2, Unigene65096\_JingChaoZuZhi2, Unigene65445\_JingChaoZuZhi2, Unigene65951\_JingChaoZuZhi2, Unigene66116\_JingChaoZuZhi2, Unigene66346\_JingChaoZuZhi2, Unigene66540\_JingChaoZuZhi2, Unigene6669\_JingChaoZuZhi2, Unigene66904\_JingChaoZuZhi2, Unigene67655\_JingChaoZuZhi2, Unigene68097\_JingChaoZuZhi2, Unigene68223\_JingChaoZuZhi2, Unigene68300\_JingChaoZuZhi2, Unigene69608\_JingChaoZuZhi2, Unigene70087\_JingChaoZuZhi2, Unigene7010\_JingChaoZuZhi2, Unigene70493\_JingChaoZuZhi2, Unigene70723\_JingChaoZuZhi2, Unigene71043\_JingChaoZuZhi2, Unigene71046\_JingChaoZuZhi2, Unigene71297\_JingChaoZuZhi2, Unigene71365\_JingChaoZuZhi2, Unigene71640\_JingChaoZuZhi2, Unigene71662\_JingChaoZuZhi2, Unigene7192\_JingChaoZuZhi2, Unigene72466\_JingChaoZuZhi2, Unigene72521\_JingChaoZuZhi2, Unigene72643\_JingChaoZuZhi2, Unigene72826\_JingChaoZuZhi2, Unigene72978\_JingChaoZuZhi2, Unigene73674\_JingChaoZuZhi2, Unigene74505\_JingChaoZuZhi2, Unigene7456\_JingChaoZuZhi2, Unigene74597\_JingChaoZuZhi2, Unigene7480\_JingChaoZuZhi2, Unigene75235\_JingChaoZuZhi2, Unigene76495\_JingChaoZuZhi2, Unigene77149\_JingChaoZuZhi2, Unigene77471\_JingChaoZuZhi2, Unigene78078\_JingChaoZuZhi2, Unigene78189\_JingChaoZuZhi2, Unigene78225\_JingChaoZuZhi2, Unigene78235\_JingChaoZuZhi2, Unigene78490\_JingChaoZuZhi2, Unigene78574\_JingChaoZuZhi2, Unigene78587\_JingChaoZuZhi2, Unigene78756\_JingChaoZuZhi2, Unigene79274\_JingChaoZuZhi2, Unigene79388\_JingChaoZuZhi2, Unigene79567\_JingChaoZuZhi2, Unigene8006\_JingChaoZuZhi2, Unigene80494\_JingChaoZuZhi2, Unigene80605\_JingChaoZuZhi2, Unigene8081\_JingChaoZuZhi2, Unigene81016\_JingChaoZuZhi2, Unigene81043\_JingChaoZuZhi2, Unigene81395\_JingChaoZuZhi2, Unigene81901\_JingChaoZuZhi2, Unigene81903\_JingChaoZuZhi2, Unigene8192\_JingChaoZuZhi2, Unigene82219\_JingChaoZuZhi2, Unigene82259\_JingChaoZuZhi2, Unigene82329\_JingChaoZuZhi2, Unigene82368\_JingChaoZuZhi2, Unigene82695\_JingChaoZuZhi2, Unigene83166\_JingChaoZuZhi2, Unigene83308\_JingChaoZuZhi2, Unigene83526\_JingChaoZuZhi2, Unigene83832\_JingChaoZuZhi2, Unigene84161\_JingChaoZuZhi2, Unigene84247\_JingChaoZuZhi2, Unigene84550\_JingChaoZuZhi2, Unigene84592\_JingChaoZuZhi2, Unigene84903\_JingChaoZuZhi2, Unigene8507\_JingChaoZuZhi2, Unigene85126\_JingChaoZuZhi2, Unigene86007\_JingChaoZuZhi2, Unigene86074\_JingChaoZuZhi2, Unigene8632\_JingChaoZuZhi2, Unigene86367\_JingChaoZuZhi2, Unigene86536\_JingChaoZuZhi2, Unigene86801\_JingChaoZuZhi2, Unigene87302\_JingChaoZuZhi2, Unigene87332\_JingChaoZuZhi2, Unigene87360\_JingChaoZuZhi2, Unigene87607\_JingChaoZuZhi2, Unigene87968\_JingChaoZuZhi2, Unigene88385\_JingChaoZuZhi2, Unigene88594\_JingChaoZuZhi2, Unigene88724\_JingChaoZuZhi2, Unigene88773\_JingChaoZuZhi2, Unigene89147\_JingChaoZuZhi2, Unigene89175\_JingChaoZuZhi2, Unigene89309\_JingChaoZuZhi2, Unigene89385\_JingChaoZuZhi2, Unigene89807\_JingChaoZuZhi2, Unigene90179\_JingChaoZuZhi2, Unigene90228\_JingChaoZuZhi2, Unigene9038\_JingChaoZuZhi2, Unigene91289\_JingChaoZuZhi2, Unigene91527\_JingChaoZuZhi2, Unigene91537\_JingChaoZuZhi2, Unigene91739\_JingChaoZuZhi2, Unigene91924\_JingChaoZuZhi2, Unigene92115\_JingChaoZuZhi2, Unigene92123\_JingChaoZuZhi2, Unigene92154\_JingChaoZuZhi2, Unigene9260\_JingChaoZuZhi2, Unigene92929\_JingChaoZuZhi2, Unigene9295\_JingChaoZuZhi2, Unigene93012\_JingChaoZuZhi2, Unigene93072\_JingChaoZuZhi2, Unigene93588\_JingChaoZuZhi2, Unigene93714\_JingChaoZuZhi2, Unigene93949\_JingChaoZuZhi2, Unigene93990\_JingChaoZuZhi2, Unigene94076\_JingChaoZuZhi2, Unigene9461\_JingChaoZuZhi2, Unigene94749\_JingChaoZuZhi2, Unigene95213\_JingChaoZuZhi2, Unigene9522\_JingChaoZuZhi2, Unigene95580\_JingChaoZuZhi2, Unigene95613\_JingChaoZuZhi2, Unigene95677\_JingChaoZuZhi2, Unigene95822\_JingChaoZuZhi2, Unigene95916\_JingChaoZuZhi2, Unigene95951\_JingChaoZuZhi2, Unigene95992\_JingChaoZuZhi2, Unigene96262\_JingChaoZuZhi2, Unigene9654\_JingChaoZuZhi2, Unigene9812\_JingChaoZuZhi2 |
| 36 | Neuroactive ligand-receptor interaction | Unigene10392\_JingChaoZuZhi2, Unigene10526\_JingChaoZuZhi2, Unigene10673\_JingChaoZuZhi2, Unigene10850\_JingChaoZuZhi2, Unigene10932\_JingChaoZuZhi2, Unigene10984\_JingChaoZuZhi2, Unigene11049\_JingChaoZuZhi2, Unigene11116\_JingChaoZuZhi2, Unigene11281\_JingChaoZuZhi2, Unigene11351\_JingChaoZuZhi2, Unigene1157\_JingChaoZuZhi2, Unigene11600\_JingChaoZuZhi2, Unigene11718\_JingChaoZuZhi2, Unigene11904\_JingChaoZuZhi2, Unigene1247\_JingChaoZuZhi2, Unigene12791\_JingChaoZuZhi2, Unigene13566\_JingChaoZuZhi2, Unigene13584\_JingChaoZuZhi2, Unigene13854\_JingChaoZuZhi2, Unigene13857\_JingChaoZuZhi2, Unigene13881\_JingChaoZuZhi2, Unigene14195\_JingChaoZuZhi2, Unigene14458\_JingChaoZuZhi2, Unigene14534\_JingChaoZuZhi2, Unigene14591\_JingChaoZuZhi2, Unigene14601\_JingChaoZuZhi2, Unigene14836\_JingChaoZuZhi2, Unigene14933\_JingChaoZuZhi2, Unigene15009\_JingChaoZuZhi2, Unigene15023\_JingChaoZuZhi2, Unigene15151\_JingChaoZuZhi2, Unigene15181\_JingChaoZuZhi2, Unigene1599\_JingChaoZuZhi2, Unigene16028\_JingChaoZuZhi2, Unigene16169\_JingChaoZuZhi2, Unigene16266\_JingChaoZuZhi2, Unigene16366\_JingChaoZuZhi2, Unigene16486\_JingChaoZuZhi2, Unigene16553\_JingChaoZuZhi2, Unigene16645\_JingChaoZuZhi2, Unigene167\_JingChaoZuZhi2, Unigene16939\_JingChaoZuZhi2, Unigene17496\_JingChaoZuZhi2, Unigene17572\_JingChaoZuZhi2, Unigene17628\_JingChaoZuZhi2, Unigene17795\_JingChaoZuZhi2, Unigene17806\_JingChaoZuZhi2, Unigene17809\_JingChaoZuZhi2, Unigene18739\_JingChaoZuZhi2, Unigene1887\_JingChaoZuZhi2, Unigene19196\_JingChaoZuZhi2, Unigene19286\_JingChaoZuZhi2, Unigene19406\_JingChaoZuZhi2, Unigene19691\_JingChaoZuZhi2, Unigene20214\_JingChaoZuZhi2, Unigene20305\_JingChaoZuZhi2, Unigene204\_JingChaoZuZhi2, Unigene20502\_JingChaoZuZhi2, Unigene21100\_JingChaoZuZhi2, Unigene21166\_JingChaoZuZhi2, Unigene21368\_JingChaoZuZhi2, Unigene21651\_JingChaoZuZhi2, Unigene21663\_JingChaoZuZhi2, Unigene21768\_JingChaoZuZhi2, Unigene22002\_JingChaoZuZhi2, Unigene2201\_JingChaoZuZhi2, Unigene22080\_JingChaoZuZhi2, Unigene2211\_JingChaoZuZhi2, Unigene22651\_JingChaoZuZhi2, Unigene22691\_JingChaoZuZhi2, Unigene22829\_JingChaoZuZhi2, Unigene23488\_JingChaoZuZhi2, Unigene23874\_JingChaoZuZhi2, Unigene24453\_JingChaoZuZhi2, Unigene2544\_JingChaoZuZhi2, Unigene2610\_JingChaoZuZhi2, Unigene2720\_JingChaoZuZhi2, Unigene27884\_JingChaoZuZhi2, Unigene28398\_JingChaoZuZhi2, Unigene2918\_JingChaoZuZhi2, Unigene29734\_JingChaoZuZhi2, Unigene30891\_JingChaoZuZhi2, Unigene31308\_JingChaoZuZhi2, Unigene31446\_JingChaoZuZhi2, Unigene31704\_JingChaoZuZhi2, Unigene31886\_JingChaoZuZhi2, Unigene3204\_JingChaoZuZhi2, Unigene32056\_JingChaoZuZhi2, Unigene32139\_JingChaoZuZhi2, Unigene32417\_JingChaoZuZhi2, Unigene3298\_JingChaoZuZhi2, Unigene33207\_JingChaoZuZhi2, Unigene33536\_JingChaoZuZhi2, Unigene3377\_JingChaoZuZhi2, Unigene34426\_JingChaoZuZhi2, Unigene34724\_JingChaoZuZhi2, Unigene34764\_JingChaoZuZhi2, Unigene34768\_JingChaoZuZhi2, Unigene347\_JingChaoZuZhi2, Unigene34931\_JingChaoZuZhi2, Unigene34989\_JingChaoZuZhi2, Unigene35493\_JingChaoZuZhi2, Unigene35744\_JingChaoZuZhi2, Unigene35755\_JingChaoZuZhi2, Unigene36619\_JingChaoZuZhi2, Unigene37009\_JingChaoZuZhi2, Unigene37143\_JingChaoZuZhi2, Unigene37739\_JingChaoZuZhi2, Unigene37741\_JingChaoZuZhi2, Unigene37803\_JingChaoZuZhi2, Unigene38900\_JingChaoZuZhi2, Unigene39259\_JingChaoZuZhi2, Unigene39658\_JingChaoZuZhi2, Unigene40292\_JingChaoZuZhi2, Unigene40336\_JingChaoZuZhi2, Unigene41339\_JingChaoZuZhi2, Unigene416\_JingChaoZuZhi2, Unigene42008\_JingChaoZuZhi2, Unigene42369\_JingChaoZuZhi2, Unigene42385\_JingChaoZuZhi2, Unigene42432\_JingChaoZuZhi2, Unigene42566\_JingChaoZuZhi2, Unigene42575\_JingChaoZuZhi2, Unigene42723\_JingChaoZuZhi2, Unigene43255\_JingChaoZuZhi2, Unigene43582\_JingChaoZuZhi2, Unigene43699\_JingChaoZuZhi2, Unigene45043\_JingChaoZuZhi2, Unigene45596\_JingChaoZuZhi2, Unigene46019\_JingChaoZuZhi2, Unigene48647\_JingChaoZuZhi2, Unigene49277\_JingChaoZuZhi2, Unigene49294\_JingChaoZuZhi2, Unigene49472\_JingChaoZuZhi2, Unigene49554\_JingChaoZuZhi2, Unigene4957\_JingChaoZuZhi2, Unigene49981\_JingChaoZuZhi2, Unigene50026\_JingChaoZuZhi2, Unigene50503\_JingChaoZuZhi2, Unigene51024\_JingChaoZuZhi2, Unigene51663\_JingChaoZuZhi2, Unigene5180\_JingChaoZuZhi2, Unigene52262\_JingChaoZuZhi2, Unigene52694\_JingChaoZuZhi2, Unigene53103\_JingChaoZuZhi2, Unigene5312\_JingChaoZuZhi2, Unigene53310\_JingChaoZuZhi2, Unigene53889\_JingChaoZuZhi2, Unigene53953\_JingChaoZuZhi2, Unigene53982\_JingChaoZuZhi2, Unigene53983\_JingChaoZuZhi2, Unigene5401\_JingChaoZuZhi2, Unigene54122\_JingChaoZuZhi2, Unigene54994\_JingChaoZuZhi2, Unigene55498\_JingChaoZuZhi2, Unigene55838\_JingChaoZuZhi2, Unigene56567\_JingChaoZuZhi2, Unigene57037\_JingChaoZuZhi2, Unigene57226\_JingChaoZuZhi2, Unigene57358\_JingChaoZuZhi2, Unigene5735\_JingChaoZuZhi2, Unigene5756\_JingChaoZuZhi2, Unigene57607\_JingChaoZuZhi2, Unigene58275\_JingChaoZuZhi2, Unigene58527\_JingChaoZuZhi2, Unigene5905\_JingChaoZuZhi2, Unigene59373\_JingChaoZuZhi2, Unigene59580\_JingChaoZuZhi2, Unigene60081\_JingChaoZuZhi2, Unigene602\_JingChaoZuZhi2, Unigene60481\_JingChaoZuZhi2, Unigene60768\_JingChaoZuZhi2, Unigene607\_JingChaoZuZhi2, Unigene61354\_JingChaoZuZhi2, Unigene61742\_JingChaoZuZhi2, Unigene62702\_JingChaoZuZhi2, Unigene62752\_JingChaoZuZhi2, Unigene6303\_JingChaoZuZhi2, Unigene63091\_JingChaoZuZhi2, Unigene63365\_JingChaoZuZhi2, Unigene63679\_JingChaoZuZhi2, Unigene63990\_JingChaoZuZhi2, Unigene64497\_JingChaoZuZhi2, Unigene64760\_JingChaoZuZhi2, Unigene65013\_JingChaoZuZhi2, Unigene65096\_JingChaoZuZhi2, Unigene65337\_JingChaoZuZhi2, Unigene66077\_JingChaoZuZhi2, Unigene66116\_JingChaoZuZhi2, Unigene66346\_JingChaoZuZhi2, Unigene66649\_JingChaoZuZhi2, Unigene6669\_JingChaoZuZhi2, Unigene66904\_JingChaoZuZhi2, Unigene67320\_JingChaoZuZhi2, Unigene67986\_JingChaoZuZhi2, Unigene68074\_JingChaoZuZhi2, Unigene68275\_JingChaoZuZhi2, Unigene68516\_JingChaoZuZhi2, Unigene68722\_JingChaoZuZhi2, Unigene69203\_JingChaoZuZhi2, Unigene7037\_JingChaoZuZhi2, Unigene70632\_JingChaoZuZhi2, Unigene70853\_JingChaoZuZhi2, Unigene71043\_JingChaoZuZhi2, Unigene7119\_JingChaoZuZhi2, Unigene71297\_JingChaoZuZhi2, Unigene7139\_JingChaoZuZhi2, Unigene71693\_JingChaoZuZhi2, Unigene7169\_JingChaoZuZhi2, Unigene7184\_JingChaoZuZhi2, Unigene7192\_JingChaoZuZhi2, Unigene71987\_JingChaoZuZhi2, Unigene73224\_JingChaoZuZhi2, Unigene73826\_JingChaoZuZhi2, Unigene73951\_JingChaoZuZhi2, Unigene74597\_JingChaoZuZhi2, Unigene7480\_JingChaoZuZhi2, Unigene74913\_JingChaoZuZhi2, Unigene75381\_JingChaoZuZhi2, Unigene75727\_JingChaoZuZhi2, Unigene75941\_JingChaoZuZhi2, Unigene76756\_JingChaoZuZhi2, Unigene77238\_JingChaoZuZhi2, Unigene77278\_JingChaoZuZhi2, Unigene77358\_JingChaoZuZhi2, Unigene77558\_JingChaoZuZhi2, Unigene78029\_JingChaoZuZhi2, Unigene78047\_JingChaoZuZhi2, Unigene78353\_JingChaoZuZhi2, Unigene78490\_JingChaoZuZhi2, Unigene78587\_JingChaoZuZhi2, Unigene7859\_JingChaoZuZhi2, Unigene78756\_JingChaoZuZhi2, Unigene79388\_JingChaoZuZhi2, Unigene79407\_JingChaoZuZhi2, Unigene79889\_JingChaoZuZhi2, Unigene80095\_JingChaoZuZhi2, Unigene8024\_JingChaoZuZhi2, Unigene80353\_JingChaoZuZhi2, Unigene80700\_JingChaoZuZhi2, Unigene8081\_JingChaoZuZhi2, Unigene8100\_JingChaoZuZhi2, Unigene81016\_JingChaoZuZhi2, Unigene81496\_JingChaoZuZhi2, Unigene81619\_JingChaoZuZhi2, Unigene82259\_JingChaoZuZhi2, Unigene82695\_JingChaoZuZhi2, Unigene82974\_JingChaoZuZhi2, Unigene8332\_JingChaoZuZhi2, Unigene83727\_JingChaoZuZhi2, Unigene8372\_JingChaoZuZhi2, Unigene83832\_JingChaoZuZhi2, Unigene84075\_JingChaoZuZhi2, Unigene84530\_JingChaoZuZhi2, Unigene84569\_JingChaoZuZhi2, Unigene84830\_JingChaoZuZhi2, Unigene8483\_JingChaoZuZhi2, Unigene84920\_JingChaoZuZhi2, Unigene84984\_JingChaoZuZhi2, Unigene85126\_JingChaoZuZhi2, Unigene8532\_JingChaoZuZhi2, Unigene86536\_JingChaoZuZhi2, Unigene86544\_JingChaoZuZhi2, Unigene86640\_JingChaoZuZhi2, Unigene86801\_JingChaoZuZhi2, Unigene869\_JingChaoZuZhi2, Unigene87780\_JingChaoZuZhi2, Unigene87926\_JingChaoZuZhi2, Unigene87968\_JingChaoZuZhi2, Unigene88201\_JingChaoZuZhi2, Unigene88338\_JingChaoZuZhi2, Unigene88501\_JingChaoZuZhi2, Unigene88522\_JingChaoZuZhi2, Unigene88706\_JingChaoZuZhi2, Unigene88966\_JingChaoZuZhi2, Unigene88974\_JingChaoZuZhi2, Unigene88977\_JingChaoZuZhi2, Unigene89011\_JingChaoZuZhi2, Unigene89120\_JingChaoZuZhi2, Unigene89385\_JingChaoZuZhi2, Unigene89674\_JingChaoZuZhi2, Unigene89978\_JingChaoZuZhi2, Unigene90246\_JingChaoZuZhi2, Unigene90530\_JingChaoZuZhi2, Unigene90839\_JingChaoZuZhi2, Unigene90865\_JingChaoZuZhi2, Unigene91537\_JingChaoZuZhi2, Unigene91703\_JingChaoZuZhi2, Unigene91791\_JingChaoZuZhi2, Unigene91978\_JingChaoZuZhi2, Unigene92154\_JingChaoZuZhi2, Unigene92378\_JingChaoZuZhi2, Unigene92547\_JingChaoZuZhi2, Unigene92842\_JingChaoZuZhi2, Unigene93394\_JingChaoZuZhi2, Unigene93439\_JingChaoZuZhi2, Unigene93781\_JingChaoZuZhi2, Unigene93894\_JingChaoZuZhi2, Unigene94076\_JingChaoZuZhi2, Unigene94117\_JingChaoZuZhi2, Unigene94426\_JingChaoZuZhi2, Unigene94828\_JingChaoZuZhi2, Unigene94969\_JingChaoZuZhi2, Unigene95065\_JingChaoZuZhi2, Unigene95261\_JingChaoZuZhi2, Unigene95323\_JingChaoZuZhi2, Unigene95437\_JingChaoZuZhi2, Unigene95560\_JingChaoZuZhi2, Unigene95580\_JingChaoZuZhi2, Unigene95613\_JingChaoZuZhi2, Unigene95729\_JingChaoZuZhi2, Unigene95855\_JingChaoZuZhi2, Unigene96214\_JingChaoZuZhi2, Unigene96269\_JingChaoZuZhi2, Unigene9812\_JingChaoZuZhi2, Unigene9999\_JingChaoZuZhi2 |
| 37 | Lysine degradation | Unigene10112\_JingChaoZuZhi2, Unigene10208\_JingChaoZuZhi2, Unigene10381\_JingChaoZuZhi2, Unigene10687\_JingChaoZuZhi2, Unigene11005\_JingChaoZuZhi2, Unigene1104\_JingChaoZuZhi2, Unigene11196\_JingChaoZuZhi2, Unigene11312\_JingChaoZuZhi2, Unigene11491\_JingChaoZuZhi2, Unigene11684\_JingChaoZuZhi2, Unigene12087\_JingChaoZuZhi2, Unigene12088\_JingChaoZuZhi2, Unigene12099\_JingChaoZuZhi2, Unigene12136\_JingChaoZuZhi2, Unigene12198\_JingChaoZuZhi2, Unigene12290\_JingChaoZuZhi2, Unigene12584\_JingChaoZuZhi2, Unigene12727\_JingChaoZuZhi2, Unigene12820\_JingChaoZuZhi2, Unigene12978\_JingChaoZuZhi2, Unigene13025\_JingChaoZuZhi2, Unigene1314\_JingChaoZuZhi2, Unigene13429\_JingChaoZuZhi2, Unigene13461\_JingChaoZuZhi2, Unigene13559\_JingChaoZuZhi2, Unigene1356\_JingChaoZuZhi2, Unigene13714\_JingChaoZuZhi2, Unigene13717\_JingChaoZuZhi2, Unigene14020\_JingChaoZuZhi2, Unigene14138\_JingChaoZuZhi2, Unigene14152\_JingChaoZuZhi2, Unigene14251\_JingChaoZuZhi2, Unigene14945\_JingChaoZuZhi2, Unigene14947\_JingChaoZuZhi2, Unigene14983\_JingChaoZuZhi2, Unigene15039\_JingChaoZuZhi2, Unigene15285\_JingChaoZuZhi2, Unigene15659\_JingChaoZuZhi2, Unigene16113\_JingChaoZuZhi2, Unigene16176\_JingChaoZuZhi2, Unigene16254\_JingChaoZuZhi2, Unigene1631\_JingChaoZuZhi2, Unigene16352\_JingChaoZuZhi2, Unigene16512\_JingChaoZuZhi2, Unigene1652\_JingChaoZuZhi2, Unigene16606\_JingChaoZuZhi2, Unigene16738\_JingChaoZuZhi2, Unigene16892\_JingChaoZuZhi2, Unigene17068\_JingChaoZuZhi2, Unigene17248\_JingChaoZuZhi2, Unigene17516\_JingChaoZuZhi2, Unigene17957\_JingChaoZuZhi2, Unigene17995\_JingChaoZuZhi2, Unigene18051\_JingChaoZuZhi2, Unigene18117\_JingChaoZuZhi2, Unigene18179\_JingChaoZuZhi2, Unigene18212\_JingChaoZuZhi2, Unigene18215\_JingChaoZuZhi2, Unigene18348\_JingChaoZuZhi2, Unigene18358\_JingChaoZuZhi2, Unigene18409\_JingChaoZuZhi2, Unigene18545\_JingChaoZuZhi2, Unigene18553\_JingChaoZuZhi2, Unigene19319\_JingChaoZuZhi2, Unigene19336\_JingChaoZuZhi2, Unigene19531\_JingChaoZuZhi2, Unigene19926\_JingChaoZuZhi2, Unigene20049\_JingChaoZuZhi2, Unigene20086\_JingChaoZuZhi2, Unigene20312\_JingChaoZuZhi2, Unigene20321\_JingChaoZuZhi2, Unigene20347\_JingChaoZuZhi2, Unigene20375\_JingChaoZuZhi2, Unigene20404\_JingChaoZuZhi2, Unigene20560\_JingChaoZuZhi2, Unigene20722\_JingChaoZuZhi2, Unigene21028\_JingChaoZuZhi2, Unigene21049\_JingChaoZuZhi2, Unigene21074\_JingChaoZuZhi2, Unigene21099\_JingChaoZuZhi2, Unigene21153\_JingChaoZuZhi2, Unigene21176\_JingChaoZuZhi2, Unigene21278\_JingChaoZuZhi2, Unigene215\_JingChaoZuZhi2, Unigene21627\_JingChaoZuZhi2, Unigene21662\_JingChaoZuZhi2, Unigene21794\_JingChaoZuZhi2, Unigene21821\_JingChaoZuZhi2, Unigene2191\_JingChaoZuZhi2, Unigene22091\_JingChaoZuZhi2, Unigene2235\_JingChaoZuZhi2, Unigene22616\_JingChaoZuZhi2, Unigene22664\_JingChaoZuZhi2, Unigene22680\_JingChaoZuZhi2, Unigene22715\_JingChaoZuZhi2, Unigene22732\_JingChaoZuZhi2, Unigene22793\_JingChaoZuZhi2, Unigene22892\_JingChaoZuZhi2, Unigene22908\_JingChaoZuZhi2, Unigene23164\_JingChaoZuZhi2, Unigene24440\_JingChaoZuZhi2, Unigene26062\_JingChaoZuZhi2, Unigene27033\_JingChaoZuZhi2, Unigene2711\_JingChaoZuZhi2, Unigene27219\_JingChaoZuZhi2, Unigene27407\_JingChaoZuZhi2, Unigene27548\_JingChaoZuZhi2, Unigene28711\_JingChaoZuZhi2, Unigene2926\_JingChaoZuZhi2, Unigene29676\_JingChaoZuZhi2, Unigene3014\_JingChaoZuZhi2, Unigene30608\_JingChaoZuZhi2, Unigene30726\_JingChaoZuZhi2, Unigene3235\_JingChaoZuZhi2, Unigene32980\_JingChaoZuZhi2, Unigene3481\_JingChaoZuZhi2, Unigene35136\_JingChaoZuZhi2, Unigene36758\_JingChaoZuZhi2, Unigene36916\_JingChaoZuZhi2, Unigene37321\_JingChaoZuZhi2, Unigene37848\_JingChaoZuZhi2, Unigene38104\_JingChaoZuZhi2, Unigene39374\_JingChaoZuZhi2, Unigene40384\_JingChaoZuZhi2, Unigene42137\_JingChaoZuZhi2, Unigene42806\_JingChaoZuZhi2, Unigene43444\_JingChaoZuZhi2, Unigene4371\_JingChaoZuZhi2, Unigene44424\_JingChaoZuZhi2, Unigene45198\_JingChaoZuZhi2, Unigene46460\_JingChaoZuZhi2, Unigene47145\_JingChaoZuZhi2, Unigene47527\_JingChaoZuZhi2, Unigene47954\_JingChaoZuZhi2, Unigene48266\_JingChaoZuZhi2, Unigene4955\_JingChaoZuZhi2, Unigene50371\_JingChaoZuZhi2, Unigene5068\_JingChaoZuZhi2, Unigene51044\_JingChaoZuZhi2, Unigene51532\_JingChaoZuZhi2, Unigene51567\_JingChaoZuZhi2, Unigene52538\_JingChaoZuZhi2, Unigene52598\_JingChaoZuZhi2, Unigene53467\_JingChaoZuZhi2, Unigene5347\_JingChaoZuZhi2, Unigene53484\_JingChaoZuZhi2, Unigene53812\_JingChaoZuZhi2, Unigene53834\_JingChaoZuZhi2, Unigene54698\_JingChaoZuZhi2, Unigene55019\_JingChaoZuZhi2, Unigene55446\_JingChaoZuZhi2, Unigene55983\_JingChaoZuZhi2, Unigene56284\_JingChaoZuZhi2, Unigene56774\_JingChaoZuZhi2, Unigene57857\_JingChaoZuZhi2, Unigene57909\_JingChaoZuZhi2, Unigene5797\_JingChaoZuZhi2, Unigene5863\_JingChaoZuZhi2, Unigene58763\_JingChaoZuZhi2, Unigene5904\_JingChaoZuZhi2, Unigene59127\_JingChaoZuZhi2, Unigene59627\_JingChaoZuZhi2, Unigene59684\_JingChaoZuZhi2, Unigene61294\_JingChaoZuZhi2, Unigene61464\_JingChaoZuZhi2, Unigene61664\_JingChaoZuZhi2, Unigene61955\_JingChaoZuZhi2, Unigene62545\_JingChaoZuZhi2, Unigene62851\_JingChaoZuZhi2, Unigene63584\_JingChaoZuZhi2, Unigene63649\_JingChaoZuZhi2, Unigene63715\_JingChaoZuZhi2, Unigene6396\_JingChaoZuZhi2, Unigene64300\_JingChaoZuZhi2, Unigene64843\_JingChaoZuZhi2, Unigene64903\_JingChaoZuZhi2, Unigene649\_JingChaoZuZhi2, Unigene65187\_JingChaoZuZhi2, Unigene65823\_JingChaoZuZhi2, Unigene66337\_JingChaoZuZhi2, Unigene66341\_JingChaoZuZhi2, Unigene66456\_JingChaoZuZhi2, Unigene66476\_JingChaoZuZhi2, Unigene67475\_JingChaoZuZhi2, Unigene67613\_JingChaoZuZhi2, Unigene67704\_JingChaoZuZhi2, Unigene6783\_JingChaoZuZhi2, Unigene6797\_JingChaoZuZhi2, Unigene68300\_JingChaoZuZhi2, Unigene69368\_JingChaoZuZhi2, Unigene70045\_JingChaoZuZhi2, Unigene70087\_JingChaoZuZhi2, Unigene70869\_JingChaoZuZhi2, Unigene7102\_JingChaoZuZhi2, Unigene71479\_JingChaoZuZhi2, Unigene72476\_JingChaoZuZhi2, Unigene72629\_JingChaoZuZhi2, Unigene72921\_JingChaoZuZhi2, Unigene73912\_JingChaoZuZhi2, Unigene74030\_JingChaoZuZhi2, Unigene7495\_JingChaoZuZhi2, Unigene74970\_JingChaoZuZhi2, Unigene75281\_JingChaoZuZhi2, Unigene7583\_JingChaoZuZhi2, Unigene7586\_JingChaoZuZhi2, Unigene76441\_JingChaoZuZhi2, Unigene77009\_JingChaoZuZhi2, Unigene77305\_JingChaoZuZhi2, Unigene77748\_JingChaoZuZhi2, Unigene78125\_JingChaoZuZhi2, Unigene78348\_JingChaoZuZhi2, Unigene78889\_JingChaoZuZhi2, Unigene78\_JingChaoZuZhi2, Unigene79494\_JingChaoZuZhi2, Unigene7972\_JingChaoZuZhi2, Unigene7985\_JingChaoZuZhi2, Unigene80636\_JingChaoZuZhi2, Unigene80881\_JingChaoZuZhi2, Unigene80995\_JingChaoZuZhi2, Unigene81640\_JingChaoZuZhi2, Unigene81764\_JingChaoZuZhi2, Unigene81798\_JingChaoZuZhi2, Unigene82129\_JingChaoZuZhi2, Unigene8258\_JingChaoZuZhi2, Unigene82674\_JingChaoZuZhi2, Unigene82793\_JingChaoZuZhi2, Unigene8290\_JingChaoZuZhi2, Unigene83124\_JingChaoZuZhi2, Unigene83134\_JingChaoZuZhi2, Unigene83561\_JingChaoZuZhi2, Unigene83885\_JingChaoZuZhi2, Unigene84063\_JingChaoZuZhi2, Unigene84328\_JingChaoZuZhi2, Unigene84343\_JingChaoZuZhi2, Unigene84475\_JingChaoZuZhi2, Unigene8454\_JingChaoZuZhi2, Unigene84851\_JingChaoZuZhi2, Unigene85224\_JingChaoZuZhi2, Unigene85976\_JingChaoZuZhi2, Unigene86352\_JingChaoZuZhi2, Unigene8647\_JingChaoZuZhi2, Unigene86616\_JingChaoZuZhi2, Unigene8661\_JingChaoZuZhi2, Unigene87044\_JingChaoZuZhi2, Unigene87261\_JingChaoZuZhi2, Unigene87485\_JingChaoZuZhi2, Unigene874\_JingChaoZuZhi2, Unigene87619\_JingChaoZuZhi2, Unigene87639\_JingChaoZuZhi2, Unigene87844\_JingChaoZuZhi2, Unigene87994\_JingChaoZuZhi2, Unigene88675\_JingChaoZuZhi2, Unigene89018\_JingChaoZuZhi2, Unigene89746\_JingChaoZuZhi2, Unigene89798\_JingChaoZuZhi2, Unigene8981\_JingChaoZuZhi2, Unigene89829\_JingChaoZuZhi2, Unigene90127\_JingChaoZuZhi2, Unigene90250\_JingChaoZuZhi2, Unigene90838\_JingChaoZuZhi2, Unigene90933\_JingChaoZuZhi2, Unigene90935\_JingChaoZuZhi2, Unigene91025\_JingChaoZuZhi2, Unigene91574\_JingChaoZuZhi2, Unigene91673\_JingChaoZuZhi2, Unigene91700\_JingChaoZuZhi2, Unigene91721\_JingChaoZuZhi2, Unigene91741\_JingChaoZuZhi2, Unigene91905\_JingChaoZuZhi2, Unigene92107\_JingChaoZuZhi2, Unigene9233\_JingChaoZuZhi2, Unigene92346\_JingChaoZuZhi2, Unigene92426\_JingChaoZuZhi2, Unigene92517\_JingChaoZuZhi2, Unigene92674\_JingChaoZuZhi2, Unigene92687\_JingChaoZuZhi2, Unigene93054\_JingChaoZuZhi2, Unigene93334\_JingChaoZuZhi2, Unigene93558\_JingChaoZuZhi2, Unigene93585\_JingChaoZuZhi2, Unigene93681\_JingChaoZuZhi2, Unigene93953\_JingChaoZuZhi2, Unigene93997\_JingChaoZuZhi2, Unigene9411\_JingChaoZuZhi2, Unigene94156\_JingChaoZuZhi2, Unigene94373\_JingChaoZuZhi2, Unigene94746\_JingChaoZuZhi2, Unigene94826\_JingChaoZuZhi2, Unigene94837\_JingChaoZuZhi2, Unigene9483\_JingChaoZuZhi2, Unigene94902\_JingChaoZuZhi2, Unigene94958\_JingChaoZuZhi2, Unigene94968\_JingChaoZuZhi2, Unigene94998\_JingChaoZuZhi2, Unigene9532\_JingChaoZuZhi2, Unigene95330\_JingChaoZuZhi2, Unigene95534\_JingChaoZuZhi2, Unigene95702\_JingChaoZuZhi2, Unigene95771\_JingChaoZuZhi2, Unigene9580\_JingChaoZuZhi2, Unigene95846\_JingChaoZuZhi2, Unigene95941\_JingChaoZuZhi2, Unigene95959\_JingChaoZuZhi2, Unigene95985\_JingChaoZuZhi2, Unigene96031\_JingChaoZuZhi2 |
| 38 | Cell cycle | Unigene10031\_JingChaoZuZhi2, Unigene10073\_JingChaoZuZhi2, Unigene10125\_JingChaoZuZhi2, Unigene10300\_JingChaoZuZhi2, Unigene10309\_JingChaoZuZhi2, Unigene10599\_JingChaoZuZhi2, Unigene10847\_JingChaoZuZhi2, Unigene10878\_JingChaoZuZhi2, Unigene10926\_JingChaoZuZhi2, Unigene11043\_JingChaoZuZhi2, Unigene11105\_JingChaoZuZhi2, Unigene1157\_JingChaoZuZhi2, Unigene11690\_JingChaoZuZhi2, Unigene11718\_JingChaoZuZhi2, Unigene11898\_JingChaoZuZhi2, Unigene11906\_JingChaoZuZhi2, Unigene11937\_JingChaoZuZhi2, Unigene11996\_JingChaoZuZhi2, Unigene12214\_JingChaoZuZhi2, Unigene12634\_JingChaoZuZhi2, Unigene12754\_JingChaoZuZhi2, Unigene13117\_JingChaoZuZhi2, Unigene13359\_JingChaoZuZhi2, Unigene13623\_JingChaoZuZhi2, Unigene13857\_JingChaoZuZhi2, Unigene14051\_JingChaoZuZhi2, Unigene14250\_JingChaoZuZhi2, Unigene14263\_JingChaoZuZhi2, Unigene14390\_JingChaoZuZhi2, Unigene14462\_JingChaoZuZhi2, Unigene14776\_JingChaoZuZhi2, Unigene15118\_JingChaoZuZhi2, Unigene15128\_JingChaoZuZhi2, Unigene15151\_JingChaoZuZhi2, Unigene15446\_JingChaoZuZhi2, Unigene15501\_JingChaoZuZhi2, Unigene15827\_JingChaoZuZhi2, Unigene15993\_JingChaoZuZhi2, Unigene15998\_JingChaoZuZhi2, Unigene16468\_JingChaoZuZhi2, Unigene167\_JingChaoZuZhi2, Unigene16820\_JingChaoZuZhi2, Unigene16958\_JingChaoZuZhi2, Unigene17010\_JingChaoZuZhi2, Unigene17115\_JingChaoZuZhi2, Unigene17187\_JingChaoZuZhi2, Unigene17467\_JingChaoZuZhi2, Unigene18008\_JingChaoZuZhi2, Unigene18139\_JingChaoZuZhi2, Unigene18209\_JingChaoZuZhi2, Unigene18236\_JingChaoZuZhi2, Unigene1831\_JingChaoZuZhi2, Unigene18390\_JingChaoZuZhi2, Unigene19013\_JingChaoZuZhi2, Unigene19238\_JingChaoZuZhi2, Unigene19361\_JingChaoZuZhi2, Unigene19453\_JingChaoZuZhi2, Unigene19691\_JingChaoZuZhi2, Unigene19980\_JingChaoZuZhi2, Unigene20017\_JingChaoZuZhi2, Unigene20052\_JingChaoZuZhi2, Unigene20102\_JingChaoZuZhi2, Unigene20261\_JingChaoZuZhi2, Unigene20303\_JingChaoZuZhi2, Unigene20305\_JingChaoZuZhi2, Unigene20489\_JingChaoZuZhi2, Unigene20635\_JingChaoZuZhi2, Unigene21515\_JingChaoZuZhi2, Unigene21805\_JingChaoZuZhi2, Unigene21891\_JingChaoZuZhi2, Unigene22022\_JingChaoZuZhi2, Unigene22290\_JingChaoZuZhi2, Unigene22299\_JingChaoZuZhi2, Unigene22375\_JingChaoZuZhi2, Unigene22402\_JingChaoZuZhi2, Unigene22435\_JingChaoZuZhi2, Unigene22459\_JingChaoZuZhi2, Unigene22580\_JingChaoZuZhi2, Unigene22622\_JingChaoZuZhi2, Unigene22673\_JingChaoZuZhi2, Unigene22680\_JingChaoZuZhi2, Unigene22708\_JingChaoZuZhi2, Unigene22736\_JingChaoZuZhi2, Unigene22836\_JingChaoZuZhi2, Unigene2473\_JingChaoZuZhi2, Unigene2691\_JingChaoZuZhi2, Unigene2692\_JingChaoZuZhi2, Unigene29918\_JingChaoZuZhi2, Unigene32208\_JingChaoZuZhi2, Unigene32677\_JingChaoZuZhi2, Unigene3290\_JingChaoZuZhi2, Unigene33333\_JingChaoZuZhi2, Unigene35730\_JingChaoZuZhi2, Unigene36126\_JingChaoZuZhi2, Unigene36424\_JingChaoZuZhi2, Unigene36737\_JingChaoZuZhi2, Unigene37741\_JingChaoZuZhi2, Unigene37803\_JingChaoZuZhi2, Unigene38124\_JingChaoZuZhi2, Unigene38493\_JingChaoZuZhi2, Unigene39584\_JingChaoZuZhi2, Unigene395\_JingChaoZuZhi2, Unigene39825\_JingChaoZuZhi2, Unigene40239\_JingChaoZuZhi2, Unigene42902\_JingChaoZuZhi2, Unigene42969\_JingChaoZuZhi2, Unigene42981\_JingChaoZuZhi2, Unigene43322\_JingChaoZuZhi2, Unigene44039\_JingChaoZuZhi2, Unigene46240\_JingChaoZuZhi2, Unigene46937\_JingChaoZuZhi2, Unigene5003\_JingChaoZuZhi2, Unigene51164\_JingChaoZuZhi2, Unigene51285\_JingChaoZuZhi2, Unigene51992\_JingChaoZuZhi2, Unigene5312\_JingChaoZuZhi2, Unigene53953\_JingChaoZuZhi2, Unigene554\_JingChaoZuZhi2, Unigene55677\_JingChaoZuZhi2, Unigene55878\_JingChaoZuZhi2, Unigene5608\_JingChaoZuZhi2, Unigene56642\_JingChaoZuZhi2, Unigene56772\_JingChaoZuZhi2, Unigene5758\_JingChaoZuZhi2, Unigene58089\_JingChaoZuZhi2, Unigene58101\_JingChaoZuZhi2, Unigene58474\_JingChaoZuZhi2, Unigene5871\_JingChaoZuZhi2, Unigene58767\_JingChaoZuZhi2, Unigene59068\_JingChaoZuZhi2, Unigene59580\_JingChaoZuZhi2, Unigene60045\_JingChaoZuZhi2, Unigene61015\_JingChaoZuZhi2, Unigene61053\_JingChaoZuZhi2, Unigene61590\_JingChaoZuZhi2, Unigene62180\_JingChaoZuZhi2, Unigene63320\_JingChaoZuZhi2, Unigene63323\_JingChaoZuZhi2, Unigene63406\_JingChaoZuZhi2, Unigene6355\_JingChaoZuZhi2, Unigene63966\_JingChaoZuZhi2, Unigene6439\_JingChaoZuZhi2, Unigene6467\_JingChaoZuZhi2, Unigene6477\_JingChaoZuZhi2, Unigene65337\_JingChaoZuZhi2, Unigene66036\_JingChaoZuZhi2, Unigene66445\_JingChaoZuZhi2, Unigene66737\_JingChaoZuZhi2, Unigene66743\_JingChaoZuZhi2, Unigene6700\_JingChaoZuZhi2, Unigene67177\_JingChaoZuZhi2, Unigene6772\_JingChaoZuZhi2, Unigene6785\_JingChaoZuZhi2, Unigene67881\_JingChaoZuZhi2, Unigene67909\_JingChaoZuZhi2, Unigene68448\_JingChaoZuZhi2, Unigene69203\_JingChaoZuZhi2, Unigene70006\_JingChaoZuZhi2, Unigene7002\_JingChaoZuZhi2, Unigene70146\_JingChaoZuZhi2, Unigene70584\_JingChaoZuZhi2, Unigene70752\_JingChaoZuZhi2, Unigene7139\_JingChaoZuZhi2, Unigene71542\_JingChaoZuZhi2, Unigene71729\_JingChaoZuZhi2, Unigene7238\_JingChaoZuZhi2, Unigene7245\_JingChaoZuZhi2, Unigene72632\_JingChaoZuZhi2, Unigene72663\_JingChaoZuZhi2, Unigene72769\_JingChaoZuZhi2, Unigene72926\_JingChaoZuZhi2, Unigene73233\_JingChaoZuZhi2, Unigene73796\_JingChaoZuZhi2, Unigene73826\_JingChaoZuZhi2, Unigene73864\_JingChaoZuZhi2, Unigene7390\_JingChaoZuZhi2, Unigene74609\_JingChaoZuZhi2, Unigene7557\_JingChaoZuZhi2, Unigene76023\_JingChaoZuZhi2, Unigene76147\_JingChaoZuZhi2, Unigene76639\_JingChaoZuZhi2, Unigene77113\_JingChaoZuZhi2, Unigene77688\_JingChaoZuZhi2, Unigene78192\_JingChaoZuZhi2, Unigene78441\_JingChaoZuZhi2, Unigene78522\_JingChaoZuZhi2, Unigene79100\_JingChaoZuZhi2, Unigene79233\_JingChaoZuZhi2, Unigene80123\_JingChaoZuZhi2, Unigene81071\_JingChaoZuZhi2, Unigene81086\_JingChaoZuZhi2, Unigene8142\_JingChaoZuZhi2, Unigene81716\_JingChaoZuZhi2, Unigene81865\_JingChaoZuZhi2, Unigene82093\_JingChaoZuZhi2, Unigene82454\_JingChaoZuZhi2, Unigene8245\_JingChaoZuZhi2, Unigene82580\_JingChaoZuZhi2, Unigene83188\_JingChaoZuZhi2, Unigene84007\_JingChaoZuZhi2, Unigene84706\_JingChaoZuZhi2, Unigene84830\_JingChaoZuZhi2, Unigene84844\_JingChaoZuZhi2, Unigene84930\_JingChaoZuZhi2, Unigene84934\_JingChaoZuZhi2, Unigene85030\_JingChaoZuZhi2, Unigene85771\_JingChaoZuZhi2, Unigene86060\_JingChaoZuZhi2, Unigene8609\_JingChaoZuZhi2, Unigene86119\_JingChaoZuZhi2, Unigene86239\_JingChaoZuZhi2, Unigene86504\_JingChaoZuZhi2, Unigene86633\_JingChaoZuZhi2, Unigene86959\_JingChaoZuZhi2, Unigene87133\_JingChaoZuZhi2, Unigene87780\_JingChaoZuZhi2, Unigene87923\_JingChaoZuZhi2, Unigene88082\_JingChaoZuZhi2, Unigene88455\_JingChaoZuZhi2, Unigene88522\_JingChaoZuZhi2, Unigene88719\_JingChaoZuZhi2, Unigene88855\_JingChaoZuZhi2, Unigene89011\_JingChaoZuZhi2, Unigene89064\_JingChaoZuZhi2, Unigene89093\_JingChaoZuZhi2, Unigene89161\_JingChaoZuZhi2, Unigene89310\_JingChaoZuZhi2, Unigene9003\_JingChaoZuZhi2, Unigene90415\_JingChaoZuZhi2, Unigene90500\_JingChaoZuZhi2, Unigene90508\_JingChaoZuZhi2, Unigene90541\_JingChaoZuZhi2, Unigene90567\_JingChaoZuZhi2, Unigene91008\_JingChaoZuZhi2, Unigene91077\_JingChaoZuZhi2, Unigene91119\_JingChaoZuZhi2, Unigene91200\_JingChaoZuZhi2, Unigene91594\_JingChaoZuZhi2, Unigene91678\_JingChaoZuZhi2, Unigene91689\_JingChaoZuZhi2, Unigene91705\_JingChaoZuZhi2, Unigene91791\_JingChaoZuZhi2, Unigene91978\_JingChaoZuZhi2, Unigene92019\_JingChaoZuZhi2, Unigene92078\_JingChaoZuZhi2, Unigene92547\_JingChaoZuZhi2, Unigene92715\_JingChaoZuZhi2, Unigene93195\_JingChaoZuZhi2, Unigene93288\_JingChaoZuZhi2, Unigene93458\_JingChaoZuZhi2, Unigene93522\_JingChaoZuZhi2, Unigene93823\_JingChaoZuZhi2, Unigene94116\_JingChaoZuZhi2, Unigene94138\_JingChaoZuZhi2, Unigene94242\_JingChaoZuZhi2, Unigene9428\_JingChaoZuZhi2, Unigene94335\_JingChaoZuZhi2, Unigene94377\_JingChaoZuZhi2, Unigene94391\_JingChaoZuZhi2, Unigene9444\_JingChaoZuZhi2, Unigene945\_JingChaoZuZhi2, Unigene94602\_JingChaoZuZhi2, Unigene94604\_JingChaoZuZhi2, Unigene94711\_JingChaoZuZhi2, Unigene94829\_JingChaoZuZhi2, Unigene94846\_JingChaoZuZhi2, Unigene95065\_JingChaoZuZhi2, Unigene95098\_JingChaoZuZhi2, Unigene95295\_JingChaoZuZhi2, Unigene95315\_JingChaoZuZhi2, Unigene95389\_JingChaoZuZhi2, Unigene95503\_JingChaoZuZhi2, Unigene95568\_JingChaoZuZhi2, Unigene95596\_JingChaoZuZhi2, Unigene95632\_JingChaoZuZhi2, Unigene95665\_JingChaoZuZhi2, Unigene95768\_JingChaoZuZhi2, Unigene95793\_JingChaoZuZhi2, Unigene95855\_JingChaoZuZhi2, Unigene95857\_JingChaoZuZhi2, Unigene95883\_JingChaoZuZhi2, Unigene95898\_JingChaoZuZhi2, Unigene96043\_JingChaoZuZhi2, Unigene96058\_JingChaoZuZhi2, Unigene96124\_JingChaoZuZhi2, Unigene96143\_JingChaoZuZhi2, Unigene96157\_JingChaoZuZhi2, Unigene96215\_JingChaoZuZhi2, Unigene96269\_JingChaoZuZhi2, Unigene96281\_JingChaoZuZhi2, Unigene96298\_JingChaoZuZhi2, Unigene9787\_JingChaoZuZhi2, Unigene9856\_JingChaoZuZhi2 |
| 39 | Axon guidance | Unigene10001\_JingChaoZuZhi2, Unigene1001\_JingChaoZuZhi2, Unigene10053\_JingChaoZuZhi2, Unigene10290\_JingChaoZuZhi2, Unigene10555\_JingChaoZuZhi2, Unigene10926\_JingChaoZuZhi2, Unigene10995\_JingChaoZuZhi2, Unigene1109\_JingChaoZuZhi2, Unigene11287\_JingChaoZuZhi2, Unigene11446\_JingChaoZuZhi2, Unigene11621\_JingChaoZuZhi2, Unigene12060\_JingChaoZuZhi2, Unigene12171\_JingChaoZuZhi2, Unigene12214\_JingChaoZuZhi2, Unigene12369\_JingChaoZuZhi2, Unigene12595\_JingChaoZuZhi2, Unigene12627\_JingChaoZuZhi2, Unigene1301\_JingChaoZuZhi2, Unigene13444\_JingChaoZuZhi2, Unigene13543\_JingChaoZuZhi2, Unigene13680\_JingChaoZuZhi2, Unigene13927\_JingChaoZuZhi2, Unigene14317\_JingChaoZuZhi2, Unigene14370\_JingChaoZuZhi2, Unigene14488\_JingChaoZuZhi2, Unigene14682\_JingChaoZuZhi2, Unigene14688\_JingChaoZuZhi2, Unigene14959\_JingChaoZuZhi2, Unigene14973\_JingChaoZuZhi2, Unigene14990\_JingChaoZuZhi2, Unigene15095\_JingChaoZuZhi2, Unigene15104\_JingChaoZuZhi2, Unigene15470\_JingChaoZuZhi2, Unigene15884\_JingChaoZuZhi2, Unigene16039\_JingChaoZuZhi2, Unigene1609\_JingChaoZuZhi2, Unigene16116\_JingChaoZuZhi2, Unigene16154\_JingChaoZuZhi2, Unigene16308\_JingChaoZuZhi2, Unigene16320\_JingChaoZuZhi2, Unigene16521\_JingChaoZuZhi2, Unigene16589\_JingChaoZuZhi2, Unigene16820\_JingChaoZuZhi2, Unigene16873\_JingChaoZuZhi2, Unigene16933\_JingChaoZuZhi2, Unigene17136\_JingChaoZuZhi2, Unigene17157\_JingChaoZuZhi2, Unigene17168\_JingChaoZuZhi2, Unigene17208\_JingChaoZuZhi2, Unigene17566\_JingChaoZuZhi2, Unigene17710\_JingChaoZuZhi2, Unigene17829\_JingChaoZuZhi2, Unigene1867\_JingChaoZuZhi2, Unigene18691\_JingChaoZuZhi2, Unigene18904\_JingChaoZuZhi2, Unigene19021\_JingChaoZuZhi2, Unigene19036\_JingChaoZuZhi2, Unigene1942\_JingChaoZuZhi2, Unigene19531\_JingChaoZuZhi2, Unigene19749\_JingChaoZuZhi2, Unigene19808\_JingChaoZuZhi2, Unigene19908\_JingChaoZuZhi2, Unigene1993\_JingChaoZuZhi2, Unigene20022\_JingChaoZuZhi2, Unigene20220\_JingChaoZuZhi2, Unigene20256\_JingChaoZuZhi2, Unigene20608\_JingChaoZuZhi2, Unigene20613\_JingChaoZuZhi2, Unigene20649\_JingChaoZuZhi2, Unigene20667\_JingChaoZuZhi2, Unigene20794\_JingChaoZuZhi2, Unigene21045\_JingChaoZuZhi2, Unigene21144\_JingChaoZuZhi2, Unigene21246\_JingChaoZuZhi2, Unigene21348\_JingChaoZuZhi2, Unigene21447\_JingChaoZuZhi2, Unigene21515\_JingChaoZuZhi2, Unigene21605\_JingChaoZuZhi2, Unigene21737\_JingChaoZuZhi2, Unigene21973\_JingChaoZuZhi2, Unigene22102\_JingChaoZuZhi2, Unigene22150\_JingChaoZuZhi2, Unigene22174\_JingChaoZuZhi2, Unigene22386\_JingChaoZuZhi2, Unigene22482\_JingChaoZuZhi2, Unigene22487\_JingChaoZuZhi2, Unigene22648\_JingChaoZuZhi2, Unigene22737\_JingChaoZuZhi2, Unigene22804\_JingChaoZuZhi2, Unigene22934\_JingChaoZuZhi2, Unigene24841\_JingChaoZuZhi2, Unigene24881\_JingChaoZuZhi2, Unigene24980\_JingChaoZuZhi2, Unigene25350\_JingChaoZuZhi2, Unigene25966\_JingChaoZuZhi2, Unigene26183\_JingChaoZuZhi2, Unigene27478\_JingChaoZuZhi2, Unigene27775\_JingChaoZuZhi2, Unigene277\_JingChaoZuZhi2, Unigene28421\_JingChaoZuZhi2, Unigene30021\_JingChaoZuZhi2, Unigene3016\_JingChaoZuZhi2, Unigene3185\_JingChaoZuZhi2, Unigene3303\_JingChaoZuZhi2, Unigene33871\_JingChaoZuZhi2, Unigene34853\_JingChaoZuZhi2, Unigene35052\_JingChaoZuZhi2, Unigene35243\_JingChaoZuZhi2, Unigene3527\_JingChaoZuZhi2, Unigene35744\_JingChaoZuZhi2, Unigene38493\_JingChaoZuZhi2, Unigene39539\_JingChaoZuZhi2, Unigene39982\_JingChaoZuZhi2, Unigene40017\_JingChaoZuZhi2, Unigene40020\_JingChaoZuZhi2, Unigene40825\_JingChaoZuZhi2, Unigene42332\_JingChaoZuZhi2, Unigene4240\_JingChaoZuZhi2, Unigene4278\_JingChaoZuZhi2, Unigene4301\_JingChaoZuZhi2, Unigene44995\_JingChaoZuZhi2, Unigene45\_JingChaoZuZhi2, Unigene46265\_JingChaoZuZhi2, Unigene46406\_JingChaoZuZhi2, Unigene46973\_JingChaoZuZhi2, Unigene4789\_JingChaoZuZhi2, Unigene4801\_JingChaoZuZhi2, Unigene480\_JingChaoZuZhi2, Unigene48368\_JingChaoZuZhi2, Unigene48607\_JingChaoZuZhi2, Unigene48843\_JingChaoZuZhi2, Unigene4915\_JingChaoZuZhi2, Unigene50282\_JingChaoZuZhi2, Unigene50470\_JingChaoZuZhi2, Unigene51966\_JingChaoZuZhi2, Unigene53659\_JingChaoZuZhi2, Unigene53722\_JingChaoZuZhi2, Unigene53917\_JingChaoZuZhi2, Unigene55326\_JingChaoZuZhi2, Unigene55362\_JingChaoZuZhi2, Unigene55555\_JingChaoZuZhi2, Unigene58136\_JingChaoZuZhi2, Unigene59152\_JingChaoZuZhi2, Unigene601\_JingChaoZuZhi2, Unigene60352\_JingChaoZuZhi2, Unigene60454\_JingChaoZuZhi2, Unigene61108\_JingChaoZuZhi2, Unigene61406\_JingChaoZuZhi2, Unigene62066\_JingChaoZuZhi2, Unigene6382\_JingChaoZuZhi2, Unigene64101\_JingChaoZuZhi2, Unigene64309\_JingChaoZuZhi2, Unigene64556\_JingChaoZuZhi2, Unigene66013\_JingChaoZuZhi2, Unigene66402\_JingChaoZuZhi2, Unigene6676\_JingChaoZuZhi2, Unigene6680\_JingChaoZuZhi2, Unigene67299\_JingChaoZuZhi2, Unigene6752\_JingChaoZuZhi2, Unigene67751\_JingChaoZuZhi2, Unigene67863\_JingChaoZuZhi2, Unigene67913\_JingChaoZuZhi2, Unigene68130\_JingChaoZuZhi2, Unigene69970\_JingChaoZuZhi2, Unigene70636\_JingChaoZuZhi2, Unigene71402\_JingChaoZuZhi2, Unigene71603\_JingChaoZuZhi2, Unigene7182\_JingChaoZuZhi2, Unigene71891\_JingChaoZuZhi2, Unigene7204\_JingChaoZuZhi2, Unigene72075\_JingChaoZuZhi2, Unigene722\_JingChaoZuZhi2, Unigene73178\_JingChaoZuZhi2, Unigene7351\_JingChaoZuZhi2, Unigene7357\_JingChaoZuZhi2, Unigene73606\_JingChaoZuZhi2, Unigene74021\_JingChaoZuZhi2, Unigene7411\_JingChaoZuZhi2, Unigene74125\_JingChaoZuZhi2, Unigene7417\_JingChaoZuZhi2, Unigene74935\_JingChaoZuZhi2, Unigene75446\_JingChaoZuZhi2, Unigene756\_JingChaoZuZhi2, Unigene75840\_JingChaoZuZhi2, Unigene75859\_JingChaoZuZhi2, Unigene75940\_JingChaoZuZhi2, Unigene76023\_JingChaoZuZhi2, Unigene76400\_JingChaoZuZhi2, Unigene76575\_JingChaoZuZhi2, Unigene76871\_JingChaoZuZhi2, Unigene76917\_JingChaoZuZhi2, Unigene77061\_JingChaoZuZhi2, Unigene77666\_JingChaoZuZhi2, Unigene77973\_JingChaoZuZhi2, Unigene78909\_JingChaoZuZhi2, Unigene79076\_JingChaoZuZhi2, Unigene79274\_JingChaoZuZhi2, Unigene7939\_JingChaoZuZhi2, Unigene79528\_JingChaoZuZhi2, Unigene79912\_JingChaoZuZhi2, Unigene8003\_JingChaoZuZhi2, Unigene80131\_JingChaoZuZhi2, Unigene81006\_JingChaoZuZhi2, Unigene81086\_JingChaoZuZhi2, Unigene81443\_JingChaoZuZhi2, Unigene8157\_JingChaoZuZhi2, Unigene81687\_JingChaoZuZhi2, Unigene8191\_JingChaoZuZhi2, Unigene82219\_JingChaoZuZhi2, Unigene83017\_JingChaoZuZhi2, Unigene83327\_JingChaoZuZhi2, Unigene8375\_JingChaoZuZhi2, Unigene84519\_JingChaoZuZhi2, Unigene84944\_JingChaoZuZhi2, Unigene85640\_JingChaoZuZhi2, Unigene8566\_JingChaoZuZhi2, Unigene85972\_JingChaoZuZhi2, Unigene86628\_JingChaoZuZhi2, Unigene86637\_JingChaoZuZhi2, Unigene86789\_JingChaoZuZhi2, Unigene86964\_JingChaoZuZhi2, Unigene87369\_JingChaoZuZhi2, Unigene8756\_JingChaoZuZhi2, Unigene876\_JingChaoZuZhi2, Unigene87851\_JingChaoZuZhi2, Unigene87867\_JingChaoZuZhi2, Unigene88132\_JingChaoZuZhi2, Unigene88185\_JingChaoZuZhi2, Unigene88215\_JingChaoZuZhi2, Unigene88391\_JingChaoZuZhi2, Unigene8851\_JingChaoZuZhi2, Unigene88666\_JingChaoZuZhi2, Unigene88719\_JingChaoZuZhi2, Unigene89310\_JingChaoZuZhi2, Unigene89462\_JingChaoZuZhi2, Unigene89492\_JingChaoZuZhi2, Unigene8949\_JingChaoZuZhi2, Unigene89625\_JingChaoZuZhi2, Unigene89722\_JingChaoZuZhi2, Unigene90762\_JingChaoZuZhi2, Unigene90908\_JingChaoZuZhi2, Unigene91156\_JingChaoZuZhi2, Unigene91179\_JingChaoZuZhi2, Unigene91221\_JingChaoZuZhi2, Unigene91314\_JingChaoZuZhi2, Unigene92371\_JingChaoZuZhi2, Unigene92581\_JingChaoZuZhi2, Unigene92800\_JingChaoZuZhi2, Unigene93782\_JingChaoZuZhi2, Unigene93793\_JingChaoZuZhi2, Unigene93821\_JingChaoZuZhi2, Unigene93932\_JingChaoZuZhi2, Unigene94077\_JingChaoZuZhi2, Unigene94138\_JingChaoZuZhi2, Unigene94164\_JingChaoZuZhi2, Unigene9445\_JingChaoZuZhi2, Unigene94561\_JingChaoZuZhi2, Unigene94749\_JingChaoZuZhi2, Unigene94919\_JingChaoZuZhi2, Unigene94922\_JingChaoZuZhi2, Unigene94928\_JingChaoZuZhi2, Unigene95098\_JingChaoZuZhi2, Unigene95147\_JingChaoZuZhi2, Unigene95295\_JingChaoZuZhi2, Unigene95506\_JingChaoZuZhi2, Unigene95875\_JingChaoZuZhi2, Unigene95881\_JingChaoZuZhi2, Unigene9589\_JingChaoZuZhi2, Unigene96121\_JingChaoZuZhi2, Unigene9645\_JingChaoZuZhi2 |
| 40 | Viral myocarditis | Unigene10027\_JingChaoZuZhi2, Unigene10034\_JingChaoZuZhi2, Unigene10047\_JingChaoZuZhi2, Unigene10259\_JingChaoZuZhi2, Unigene1060\_JingChaoZuZhi2, Unigene10628\_JingChaoZuZhi2, Unigene10765\_JingChaoZuZhi2, Unigene10926\_JingChaoZuZhi2, Unigene10\_JingChaoZuZhi2, Unigene11112\_JingChaoZuZhi2, Unigene11475\_JingChaoZuZhi2, Unigene11497\_JingChaoZuZhi2, Unigene1162\_JingChaoZuZhi2, Unigene12091\_JingChaoZuZhi2, Unigene12889\_JingChaoZuZhi2, Unigene13607\_JingChaoZuZhi2, Unigene15030\_JingChaoZuZhi2, Unigene15333\_JingChaoZuZhi2, Unigene15345\_JingChaoZuZhi2, Unigene15649\_JingChaoZuZhi2, Unigene15861\_JingChaoZuZhi2, Unigene15937\_JingChaoZuZhi2, Unigene15986\_JingChaoZuZhi2, Unigene16004\_JingChaoZuZhi2, Unigene1614\_JingChaoZuZhi2, Unigene16427\_JingChaoZuZhi2, Unigene16597\_JingChaoZuZhi2, Unigene17394\_JingChaoZuZhi2, Unigene17467\_JingChaoZuZhi2, Unigene17813\_JingChaoZuZhi2, Unigene18051\_JingChaoZuZhi2, Unigene18081\_JingChaoZuZhi2, Unigene18260\_JingChaoZuZhi2, Unigene18299\_JingChaoZuZhi2, Unigene18438\_JingChaoZuZhi2, Unigene18491\_JingChaoZuZhi2, Unigene18545\_JingChaoZuZhi2, Unigene18643\_JingChaoZuZhi2, Unigene18699\_JingChaoZuZhi2, Unigene18790\_JingChaoZuZhi2, Unigene187\_JingChaoZuZhi2, Unigene18808\_JingChaoZuZhi2, Unigene18892\_JingChaoZuZhi2, Unigene19021\_JingChaoZuZhi2, Unigene19559\_JingChaoZuZhi2, Unigene19691\_JingChaoZuZhi2, Unigene20127\_JingChaoZuZhi2, Unigene20150\_JingChaoZuZhi2, Unigene20172\_JingChaoZuZhi2, Unigene20255\_JingChaoZuZhi2, Unigene2033\_JingChaoZuZhi2, Unigene20464\_JingChaoZuZhi2, Unigene20479\_JingChaoZuZhi2, Unigene2048\_JingChaoZuZhi2, Unigene20580\_JingChaoZuZhi2, Unigene20892\_JingChaoZuZhi2, Unigene21225\_JingChaoZuZhi2, Unigene21310\_JingChaoZuZhi2, Unigene21487\_JingChaoZuZhi2, Unigene21515\_JingChaoZuZhi2, Unigene21605\_JingChaoZuZhi2, Unigene21818\_JingChaoZuZhi2, Unigene21932\_JingChaoZuZhi2, Unigene21956\_JingChaoZuZhi2, Unigene22128\_JingChaoZuZhi2, Unigene22183\_JingChaoZuZhi2, Unigene22189\_JingChaoZuZhi2, Unigene22216\_JingChaoZuZhi2, Unigene22243\_JingChaoZuZhi2, Unigene22323\_JingChaoZuZhi2, Unigene22440\_JingChaoZuZhi2, Unigene22445\_JingChaoZuZhi2, Unigene22487\_JingChaoZuZhi2, Unigene22551\_JingChaoZuZhi2, Unigene22652\_JingChaoZuZhi2, Unigene22796\_JingChaoZuZhi2, Unigene22807\_JingChaoZuZhi2, Unigene22895\_JingChaoZuZhi2, Unigene22909\_JingChaoZuZhi2, Unigene24645\_JingChaoZuZhi2, Unigene24772\_JingChaoZuZhi2, Unigene25164\_JingChaoZuZhi2, Unigene26987\_JingChaoZuZhi2, Unigene28208\_JingChaoZuZhi2, Unigene28467\_JingChaoZuZhi2, Unigene29201\_JingChaoZuZhi2, Unigene2968\_JingChaoZuZhi2, Unigene30852\_JingChaoZuZhi2, Unigene3085\_JingChaoZuZhi2, Unigene31918\_JingChaoZuZhi2, Unigene3191\_JingChaoZuZhi2, Unigene32812\_JingChaoZuZhi2, Unigene32999\_JingChaoZuZhi2, Unigene35016\_JingChaoZuZhi2, Unigene35304\_JingChaoZuZhi2, Unigene35624\_JingChaoZuZhi2, Unigene39102\_JingChaoZuZhi2, Unigene3912\_JingChaoZuZhi2, Unigene39674\_JingChaoZuZhi2, Unigene40919\_JingChaoZuZhi2, Unigene41842\_JingChaoZuZhi2, Unigene42202\_JingChaoZuZhi2, Unigene42248\_JingChaoZuZhi2, Unigene4224\_JingChaoZuZhi2, Unigene44066\_JingChaoZuZhi2, Unigene44190\_JingChaoZuZhi2, Unigene4470\_JingChaoZuZhi2, Unigene44731\_JingChaoZuZhi2, Unigene45399\_JingChaoZuZhi2, Unigene45468\_JingChaoZuZhi2, Unigene46256\_JingChaoZuZhi2, Unigene48309\_JingChaoZuZhi2, Unigene4878\_JingChaoZuZhi2, Unigene48843\_JingChaoZuZhi2, Unigene499\_JingChaoZuZhi2, Unigene50563\_JingChaoZuZhi2, Unigene50673\_JingChaoZuZhi2, Unigene5072\_JingChaoZuZhi2, Unigene51467\_JingChaoZuZhi2, Unigene52073\_JingChaoZuZhi2, Unigene54980\_JingChaoZuZhi2, Unigene55684\_JingChaoZuZhi2, Unigene57200\_JingChaoZuZhi2, Unigene57751\_JingChaoZuZhi2, Unigene58291\_JingChaoZuZhi2, Unigene58450\_JingChaoZuZhi2, Unigene59146\_JingChaoZuZhi2, Unigene5989\_JingChaoZuZhi2, Unigene60401\_JingChaoZuZhi2, Unigene60756\_JingChaoZuZhi2, Unigene61244\_JingChaoZuZhi2, Unigene61377\_JingChaoZuZhi2, Unigene61526\_JingChaoZuZhi2, Unigene6174\_JingChaoZuZhi2, Unigene6239\_JingChaoZuZhi2, Unigene63040\_JingChaoZuZhi2, Unigene6361\_JingChaoZuZhi2, Unigene64750\_JingChaoZuZhi2, Unigene65002\_JingChaoZuZhi2, Unigene65648\_JingChaoZuZhi2, Unigene6634\_JingChaoZuZhi2, Unigene6699\_JingChaoZuZhi2, Unigene6735\_JingChaoZuZhi2, Unigene6769\_JingChaoZuZhi2, Unigene6864\_JingChaoZuZhi2, Unigene69005\_JingChaoZuZhi2, Unigene69100\_JingChaoZuZhi2, Unigene70373\_JingChaoZuZhi2, Unigene71017\_JingChaoZuZhi2, Unigene71247\_JingChaoZuZhi2, Unigene71592\_JingChaoZuZhi2, Unigene72249\_JingChaoZuZhi2, Unigene73571\_JingChaoZuZhi2, Unigene73579\_JingChaoZuZhi2, Unigene7364\_JingChaoZuZhi2, Unigene75641\_JingChaoZuZhi2, Unigene76023\_JingChaoZuZhi2, Unigene76303\_JingChaoZuZhi2, Unigene76604\_JingChaoZuZhi2, Unigene77406\_JingChaoZuZhi2, Unigene77549\_JingChaoZuZhi2, Unigene79109\_JingChaoZuZhi2, Unigene7939\_JingChaoZuZhi2, Unigene79568\_JingChaoZuZhi2, Unigene80215\_JingChaoZuZhi2, Unigene82117\_JingChaoZuZhi2, Unigene82489\_JingChaoZuZhi2, Unigene8261\_JingChaoZuZhi2, Unigene82842\_JingChaoZuZhi2, Unigene8288\_JingChaoZuZhi2, Unigene83487\_JingChaoZuZhi2, Unigene83606\_JingChaoZuZhi2, Unigene83872\_JingChaoZuZhi2, Unigene84257\_JingChaoZuZhi2, Unigene84510\_JingChaoZuZhi2, Unigene85122\_JingChaoZuZhi2, Unigene85193\_JingChaoZuZhi2, Unigene85387\_JingChaoZuZhi2, Unigene86552\_JingChaoZuZhi2, Unigene866\_JingChaoZuZhi2, Unigene86759\_JingChaoZuZhi2, Unigene87677\_JingChaoZuZhi2, Unigene87825\_JingChaoZuZhi2, Unigene87958\_JingChaoZuZhi2, Unigene88040\_JingChaoZuZhi2, Unigene88342\_JingChaoZuZhi2, Unigene89060\_JingChaoZuZhi2, Unigene89090\_JingChaoZuZhi2, Unigene89409\_JingChaoZuZhi2, Unigene89959\_JingChaoZuZhi2, Unigene90401\_JingChaoZuZhi2, Unigene90626\_JingChaoZuZhi2, Unigene90767\_JingChaoZuZhi2, Unigene91030\_JingChaoZuZhi2, Unigene91043\_JingChaoZuZhi2, Unigene91163\_JingChaoZuZhi2, Unigene91179\_JingChaoZuZhi2, Unigene91580\_JingChaoZuZhi2, Unigene91631\_JingChaoZuZhi2, Unigene91991\_JingChaoZuZhi2, Unigene92141\_JingChaoZuZhi2, Unigene92329\_JingChaoZuZhi2, Unigene92448\_JingChaoZuZhi2, Unigene92471\_JingChaoZuZhi2, Unigene92577\_JingChaoZuZhi2, Unigene92673\_JingChaoZuZhi2, Unigene92681\_JingChaoZuZhi2, Unigene92769\_JingChaoZuZhi2, Unigene93010\_JingChaoZuZhi2, Unigene93279\_JingChaoZuZhi2, Unigene93516\_JingChaoZuZhi2, Unigene93793\_JingChaoZuZhi2, Unigene93865\_JingChaoZuZhi2, Unigene94110\_JingChaoZuZhi2, Unigene94138\_JingChaoZuZhi2, Unigene94214\_JingChaoZuZhi2, Unigene94337\_JingChaoZuZhi2, Unigene94510\_JingChaoZuZhi2, Unigene94723\_JingChaoZuZhi2, Unigene94823\_JingChaoZuZhi2, Unigene95031\_JingChaoZuZhi2, Unigene95059\_JingChaoZuZhi2, Unigene95110\_JingChaoZuZhi2, Unigene951\_JingChaoZuZhi2, Unigene95243\_JingChaoZuZhi2, Unigene95254\_JingChaoZuZhi2, Unigene95486\_JingChaoZuZhi2, Unigene95646\_JingChaoZuZhi2, Unigene95799\_JingChaoZuZhi2, Unigene95875\_JingChaoZuZhi2, Unigene95876\_JingChaoZuZhi2, Unigene95879\_JingChaoZuZhi2, Unigene95882\_JingChaoZuZhi2, Unigene95889\_JingChaoZuZhi2, Unigene95897\_JingChaoZuZhi2, Unigene95907\_JingChaoZuZhi2, Unigene95954\_JingChaoZuZhi2, Unigene95972\_JingChaoZuZhi2, Unigene95982\_JingChaoZuZhi2, Unigene95987\_JingChaoZuZhi2, Unigene95994\_JingChaoZuZhi2, Unigene96012\_JingChaoZuZhi2, Unigene96028\_JingChaoZuZhi2, Unigene96061\_JingChaoZuZhi2, Unigene96083\_JingChaoZuZhi2, Unigene96091\_JingChaoZuZhi2, Unigene96097\_JingChaoZuZhi2, Unigene96141\_JingChaoZuZhi2, Unigene96167\_JingChaoZuZhi2, Unigene96217\_JingChaoZuZhi2, Unigene96243\_JingChaoZuZhi2, Unigene96247\_JingChaoZuZhi2, Unigene96288\_JingChaoZuZhi2, Unigene96292\_JingChaoZuZhi2, Unigene96303\_JingChaoZuZhi2, Unigene96308\_JingChaoZuZhi2, Unigene9739\_JingChaoZuZhi2 |
| 41 | Leukocyte transendothelial migration | Unigene1002\_JingChaoZuZhi2, Unigene10047\_JingChaoZuZhi2, Unigene10078\_JingChaoZuZhi2, Unigene10218\_JingChaoZuZhi2, Unigene10259\_JingChaoZuZhi2, Unigene10318\_JingChaoZuZhi2, Unigene10541\_JingChaoZuZhi2, Unigene1060\_JingChaoZuZhi2, Unigene10637\_JingChaoZuZhi2, Unigene10809\_JingChaoZuZhi2, Unigene10922\_JingChaoZuZhi2, Unigene10\_JingChaoZuZhi2, Unigene11446\_JingChaoZuZhi2, Unigene11621\_JingChaoZuZhi2, Unigene1162\_JingChaoZuZhi2, Unigene11834\_JingChaoZuZhi2, Unigene11851\_JingChaoZuZhi2, Unigene11889\_JingChaoZuZhi2, Unigene11961\_JingChaoZuZhi2, Unigene12060\_JingChaoZuZhi2, Unigene12271\_JingChaoZuZhi2, Unigene12595\_JingChaoZuZhi2, Unigene12813\_JingChaoZuZhi2, Unigene12937\_JingChaoZuZhi2, Unigene13180\_JingChaoZuZhi2, Unigene13204\_JingChaoZuZhi2, Unigene13285\_JingChaoZuZhi2, Unigene13342\_JingChaoZuZhi2, Unigene13588\_JingChaoZuZhi2, Unigene1376\_JingChaoZuZhi2, Unigene14151\_JingChaoZuZhi2, Unigene14285\_JingChaoZuZhi2, Unigene14401\_JingChaoZuZhi2, Unigene14476\_JingChaoZuZhi2, Unigene14738\_JingChaoZuZhi2, Unigene14912\_JingChaoZuZhi2, Unigene15029\_JingChaoZuZhi2, Unigene150\_JingChaoZuZhi2, Unigene15168\_JingChaoZuZhi2, Unigene15228\_JingChaoZuZhi2, Unigene15794\_JingChaoZuZhi2, Unigene15879\_JingChaoZuZhi2, Unigene16004\_JingChaoZuZhi2, Unigene16709\_JingChaoZuZhi2, Unigene16837\_JingChaoZuZhi2, Unigene16873\_JingChaoZuZhi2, Unigene17247\_JingChaoZuZhi2, Unigene17506\_JingChaoZuZhi2, Unigene1760\_JingChaoZuZhi2, Unigene17821\_JingChaoZuZhi2, Unigene17839\_JingChaoZuZhi2, Unigene18441\_JingChaoZuZhi2, Unigene18464\_JingChaoZuZhi2, Unigene18614\_JingChaoZuZhi2, Unigene18672\_JingChaoZuZhi2, Unigene18868\_JingChaoZuZhi2, Unigene19021\_JingChaoZuZhi2, Unigene19222\_JingChaoZuZhi2, Unigene19365\_JingChaoZuZhi2, Unigene19396\_JingChaoZuZhi2, Unigene19404\_JingChaoZuZhi2, Unigene19466\_JingChaoZuZhi2, Unigene19545\_JingChaoZuZhi2, Unigene19679\_JingChaoZuZhi2, Unigene20107\_JingChaoZuZhi2, Unigene20120\_JingChaoZuZhi2, Unigene20220\_JingChaoZuZhi2, Unigene20608\_JingChaoZuZhi2, Unigene20794\_JingChaoZuZhi2, Unigene20897\_JingChaoZuZhi2, Unigene21295\_JingChaoZuZhi2, Unigene21505\_JingChaoZuZhi2, Unigene21536\_JingChaoZuZhi2, Unigene21578\_JingChaoZuZhi2, Unigene21801\_JingChaoZuZhi2, Unigene21807\_JingChaoZuZhi2, Unigene21818\_JingChaoZuZhi2, Unigene22024\_JingChaoZuZhi2, Unigene22487\_JingChaoZuZhi2, Unigene22595\_JingChaoZuZhi2, Unigene22644\_JingChaoZuZhi2, Unigene22779\_JingChaoZuZhi2, Unigene22850\_JingChaoZuZhi2, Unigene23978\_JingChaoZuZhi2, Unigene2456\_JingChaoZuZhi2, Unigene2503\_JingChaoZuZhi2, Unigene26730\_JingChaoZuZhi2, Unigene26987\_JingChaoZuZhi2, Unigene28737\_JingChaoZuZhi2, Unigene29201\_JingChaoZuZhi2, Unigene2960\_JingChaoZuZhi2, Unigene31918\_JingChaoZuZhi2, Unigene31934\_JingChaoZuZhi2, Unigene33561\_JingChaoZuZhi2, Unigene3413\_JingChaoZuZhi2, Unigene35016\_JingChaoZuZhi2, Unigene35243\_JingChaoZuZhi2, Unigene3693\_JingChaoZuZhi2, Unigene37053\_JingChaoZuZhi2, Unigene38616\_JingChaoZuZhi2, Unigene39102\_JingChaoZuZhi2, Unigene4003\_JingChaoZuZhi2, Unigene40919\_JingChaoZuZhi2, Unigene41298\_JingChaoZuZhi2, Unigene4188\_JingChaoZuZhi2, Unigene4223\_JingChaoZuZhi2, Unigene42248\_JingChaoZuZhi2, Unigene42256\_JingChaoZuZhi2, Unigene43427\_JingChaoZuZhi2, Unigene4345\_JingChaoZuZhi2, Unigene44190\_JingChaoZuZhi2, Unigene44454\_JingChaoZuZhi2, Unigene4599\_JingChaoZuZhi2, Unigene46407\_JingChaoZuZhi2, Unigene4775\_JingChaoZuZhi2, Unigene47872\_JingChaoZuZhi2, Unigene48751\_JingChaoZuZhi2, Unigene48843\_JingChaoZuZhi2, Unigene4935\_JingChaoZuZhi2, Unigene49761\_JingChaoZuZhi2, Unigene50231\_JingChaoZuZhi2, Unigene50744\_JingChaoZuZhi2, Unigene51467\_JingChaoZuZhi2, Unigene51525\_JingChaoZuZhi2, Unigene5196\_JingChaoZuZhi2, Unigene53078\_JingChaoZuZhi2, Unigene53524\_JingChaoZuZhi2, Unigene5364\_JingChaoZuZhi2, Unigene53917\_JingChaoZuZhi2, Unigene54616\_JingChaoZuZhi2, Unigene54744\_JingChaoZuZhi2, Unigene54986\_JingChaoZuZhi2, Unigene55239\_JingChaoZuZhi2, Unigene55839\_JingChaoZuZhi2, Unigene5638\_JingChaoZuZhi2, Unigene56456\_JingChaoZuZhi2, Unigene57337\_JingChaoZuZhi2, Unigene57488\_JingChaoZuZhi2, Unigene58322\_JingChaoZuZhi2, Unigene59146\_JingChaoZuZhi2, Unigene5914\_JingChaoZuZhi2, Unigene61244\_JingChaoZuZhi2, Unigene62041\_JingChaoZuZhi2, Unigene63799\_JingChaoZuZhi2, Unigene64164\_JingChaoZuZhi2, Unigene6459\_JingChaoZuZhi2, Unigene64830\_JingChaoZuZhi2, Unigene64843\_JingChaoZuZhi2, Unigene65332\_JingChaoZuZhi2, Unigene66253\_JingChaoZuZhi2, Unigene6634\_JingChaoZuZhi2, Unigene66575\_JingChaoZuZhi2, Unigene6688\_JingChaoZuZhi2, Unigene67081\_JingChaoZuZhi2, Unigene67367\_JingChaoZuZhi2, Unigene67655\_JingChaoZuZhi2, Unigene6828\_JingChaoZuZhi2, Unigene683\_JingChaoZuZhi2, Unigene69100\_JingChaoZuZhi2, Unigene691\_JingChaoZuZhi2, Unigene69402\_JingChaoZuZhi2, Unigene69934\_JingChaoZuZhi2, Unigene70723\_JingChaoZuZhi2, Unigene71592\_JingChaoZuZhi2, Unigene7219\_JingChaoZuZhi2, Unigene72728\_JingChaoZuZhi2, Unigene73446\_JingChaoZuZhi2, Unigene75840\_JingChaoZuZhi2, Unigene76069\_JingChaoZuZhi2, Unigene76111\_JingChaoZuZhi2, Unigene76917\_JingChaoZuZhi2, Unigene77061\_JingChaoZuZhi2, Unigene77406\_JingChaoZuZhi2, Unigene77955\_JingChaoZuZhi2, Unigene78909\_JingChaoZuZhi2, Unigene79411\_JingChaoZuZhi2, Unigene7977\_JingChaoZuZhi2, Unigene7985\_JingChaoZuZhi2, Unigene80331\_JingChaoZuZhi2, Unigene81904\_JingChaoZuZhi2, Unigene82117\_JingChaoZuZhi2, Unigene82489\_JingChaoZuZhi2, Unigene82662\_JingChaoZuZhi2, Unigene83017\_JingChaoZuZhi2, Unigene83583\_JingChaoZuZhi2, Unigene83872\_JingChaoZuZhi2, Unigene84519\_JingChaoZuZhi2, Unigene84563\_JingChaoZuZhi2, Unigene84862\_JingChaoZuZhi2, Unigene85132\_JingChaoZuZhi2, Unigene85193\_JingChaoZuZhi2, Unigene85306\_JingChaoZuZhi2, Unigene85923\_JingChaoZuZhi2, Unigene86206\_JingChaoZuZhi2, Unigene8621\_JingChaoZuZhi2, Unigene86349\_JingChaoZuZhi2, Unigene86526\_JingChaoZuZhi2, Unigene86669\_JingChaoZuZhi2, Unigene86713\_JingChaoZuZhi2, Unigene86789\_JingChaoZuZhi2, Unigene87082\_JingChaoZuZhi2, Unigene87191\_JingChaoZuZhi2, Unigene87332\_JingChaoZuZhi2, Unigene87984\_JingChaoZuZhi2, Unigene88185\_JingChaoZuZhi2, Unigene88342\_JingChaoZuZhi2, Unigene88391\_JingChaoZuZhi2, Unigene88988\_JingChaoZuZhi2, Unigene89045\_JingChaoZuZhi2, Unigene89060\_JingChaoZuZhi2, Unigene89728\_JingChaoZuZhi2, Unigene89901\_JingChaoZuZhi2, Unigene90255\_JingChaoZuZhi2, Unigene90715\_JingChaoZuZhi2, Unigene90747\_JingChaoZuZhi2, Unigene91163\_JingChaoZuZhi2, Unigene91179\_JingChaoZuZhi2, Unigene91221\_JingChaoZuZhi2, Unigene91480\_JingChaoZuZhi2, Unigene91839\_JingChaoZuZhi2, Unigene91971\_JingChaoZuZhi2, Unigene92112\_JingChaoZuZhi2, Unigene92119\_JingChaoZuZhi2, Unigene92186\_JingChaoZuZhi2, Unigene92315\_JingChaoZuZhi2, Unigene92372\_JingChaoZuZhi2, Unigene93271\_JingChaoZuZhi2, Unigene93374\_JingChaoZuZhi2, Unigene93702\_JingChaoZuZhi2, Unigene93782\_JingChaoZuZhi2, Unigene93807\_JingChaoZuZhi2, Unigene93821\_JingChaoZuZhi2, Unigene94110\_JingChaoZuZhi2, Unigene94272\_JingChaoZuZhi2, Unigene94370\_JingChaoZuZhi2, Unigene94561\_JingChaoZuZhi2, Unigene94619\_JingChaoZuZhi2, Unigene9461\_JingChaoZuZhi2, Unigene94685\_JingChaoZuZhi2, Unigene95020\_JingChaoZuZhi2, Unigene95098\_JingChaoZuZhi2, Unigene951\_JingChaoZuZhi2, Unigene95238\_JingChaoZuZhi2, Unigene95677\_JingChaoZuZhi2, Unigene95774\_JingChaoZuZhi2, Unigene95807\_JingChaoZuZhi2, Unigene95875\_JingChaoZuZhi2, Unigene95881\_JingChaoZuZhi2, Unigene95907\_JingChaoZuZhi2, Unigene96121\_JingChaoZuZhi2, Unigene96234\_JingChaoZuZhi2, Unigene96294\_JingChaoZuZhi2, Unigene9739\_JingChaoZuZhi2 |
| 42 | Cardiac muscle contraction | Unigene10027\_JingChaoZuZhi2, Unigene10294\_JingChaoZuZhi2, Unigene10350\_JingChaoZuZhi2, Unigene10628\_JingChaoZuZhi2, Unigene10765\_JingChaoZuZhi2, Unigene11112\_JingChaoZuZhi2, Unigene11475\_JingChaoZuZhi2, Unigene11963\_JingChaoZuZhi2, Unigene12329\_JingChaoZuZhi2, Unigene13395\_JingChaoZuZhi2, Unigene13485\_JingChaoZuZhi2, Unigene13854\_JingChaoZuZhi2, Unigene14234\_JingChaoZuZhi2, Unigene14522\_JingChaoZuZhi2, Unigene14707\_JingChaoZuZhi2, Unigene15030\_JingChaoZuZhi2, Unigene15333\_JingChaoZuZhi2, Unigene15345\_JingChaoZuZhi2, Unigene15649\_JingChaoZuZhi2, Unigene15986\_JingChaoZuZhi2, Unigene16210\_JingChaoZuZhi2, Unigene16427\_JingChaoZuZhi2, Unigene16480\_JingChaoZuZhi2, Unigene16597\_JingChaoZuZhi2, Unigene17132\_JingChaoZuZhi2, Unigene18051\_JingChaoZuZhi2, Unigene18184\_JingChaoZuZhi2, Unigene18260\_JingChaoZuZhi2, Unigene18438\_JingChaoZuZhi2, Unigene18491\_JingChaoZuZhi2, Unigene18545\_JingChaoZuZhi2, Unigene18699\_JingChaoZuZhi2, Unigene187\_JingChaoZuZhi2, Unigene18892\_JingChaoZuZhi2, Unigene19559\_JingChaoZuZhi2, Unigene20000\_JingChaoZuZhi2, Unigene20356\_JingChaoZuZhi2, Unigene20464\_JingChaoZuZhi2, Unigene20479\_JingChaoZuZhi2, Unigene20580\_JingChaoZuZhi2, Unigene20962\_JingChaoZuZhi2, Unigene21225\_JingChaoZuZhi2, Unigene21354\_JingChaoZuZhi2, Unigene21487\_JingChaoZuZhi2, Unigene21792\_JingChaoZuZhi2, Unigene21866\_JingChaoZuZhi2, Unigene22183\_JingChaoZuZhi2, Unigene22189\_JingChaoZuZhi2, Unigene22216\_JingChaoZuZhi2, Unigene22243\_JingChaoZuZhi2, Unigene22487\_JingChaoZuZhi2, Unigene22551\_JingChaoZuZhi2, Unigene22895\_JingChaoZuZhi2, Unigene2305\_JingChaoZuZhi2, Unigene24754\_JingChaoZuZhi2, Unigene25164\_JingChaoZuZhi2, Unigene26679\_JingChaoZuZhi2, Unigene28522\_JingChaoZuZhi2, Unigene2968\_JingChaoZuZhi2, Unigene30852\_JingChaoZuZhi2, Unigene31660\_JingChaoZuZhi2, Unigene3191\_JingChaoZuZhi2, Unigene31982\_JingChaoZuZhi2, Unigene32314\_JingChaoZuZhi2, Unigene35366\_JingChaoZuZhi2, Unigene35952\_JingChaoZuZhi2, Unigene36230\_JingChaoZuZhi2, Unigene36404\_JingChaoZuZhi2, Unigene37675\_JingChaoZuZhi2, Unigene3912\_JingChaoZuZhi2, Unigene41683\_JingChaoZuZhi2, Unigene42033\_JingChaoZuZhi2, Unigene44078\_JingChaoZuZhi2, Unigene44731\_JingChaoZuZhi2, Unigene4522\_JingChaoZuZhi2, Unigene46160\_JingChaoZuZhi2, Unigene46256\_JingChaoZuZhi2, Unigene47199\_JingChaoZuZhi2, Unigene4782\_JingChaoZuZhi2, Unigene47926\_JingChaoZuZhi2, Unigene48095\_JingChaoZuZhi2, Unigene48309\_JingChaoZuZhi2, Unigene48576\_JingChaoZuZhi2, Unigene4878\_JingChaoZuZhi2, Unigene49526\_JingChaoZuZhi2, Unigene50563\_JingChaoZuZhi2, Unigene50645\_JingChaoZuZhi2, Unigene50673\_JingChaoZuZhi2, Unigene5072\_JingChaoZuZhi2, Unigene51467\_JingChaoZuZhi2, Unigene5173\_JingChaoZuZhi2, Unigene52036\_JingChaoZuZhi2, Unigene52073\_JingChaoZuZhi2, Unigene52167\_JingChaoZuZhi2, Unigene52383\_JingChaoZuZhi2, Unigene5276\_JingChaoZuZhi2, Unigene53160\_JingChaoZuZhi2, Unigene54262\_JingChaoZuZhi2, Unigene54980\_JingChaoZuZhi2, Unigene55617\_JingChaoZuZhi2, Unigene55745\_JingChaoZuZhi2, Unigene56513\_JingChaoZuZhi2, Unigene56788\_JingChaoZuZhi2, Unigene57200\_JingChaoZuZhi2, Unigene57751\_JingChaoZuZhi2, Unigene5799\_JingChaoZuZhi2, Unigene58029\_JingChaoZuZhi2, Unigene58502\_JingChaoZuZhi2, Unigene61526\_JingChaoZuZhi2, Unigene6166\_JingChaoZuZhi2, Unigene6174\_JingChaoZuZhi2, Unigene61801\_JingChaoZuZhi2, Unigene61925\_JingChaoZuZhi2, Unigene6239\_JingChaoZuZhi2, Unigene6271\_JingChaoZuZhi2, Unigene62776\_JingChaoZuZhi2, Unigene63175\_JingChaoZuZhi2, Unigene6361\_JingChaoZuZhi2, Unigene65002\_JingChaoZuZhi2, Unigene65648\_JingChaoZuZhi2, Unigene65807\_JingChaoZuZhi2, Unigene6769\_JingChaoZuZhi2, Unigene68368\_JingChaoZuZhi2, Unigene6864\_JingChaoZuZhi2, Unigene68832\_JingChaoZuZhi2, Unigene69005\_JingChaoZuZhi2, Unigene70373\_JingChaoZuZhi2, Unigene71247\_JingChaoZuZhi2, Unigene71414\_JingChaoZuZhi2, Unigene71640\_JingChaoZuZhi2, Unigene7176\_JingChaoZuZhi2, Unigene72249\_JingChaoZuZhi2, Unigene72826\_JingChaoZuZhi2, Unigene73554\_JingChaoZuZhi2, Unigene75641\_JingChaoZuZhi2, Unigene76303\_JingChaoZuZhi2, Unigene76647\_JingChaoZuZhi2, Unigene76803\_JingChaoZuZhi2, Unigene77071\_JingChaoZuZhi2, Unigene77255\_JingChaoZuZhi2, Unigene77406\_JingChaoZuZhi2, Unigene77631\_JingChaoZuZhi2, Unigene77965\_JingChaoZuZhi2, Unigene78998\_JingChaoZuZhi2, Unigene79568\_JingChaoZuZhi2, Unigene79892\_JingChaoZuZhi2, Unigene80215\_JingChaoZuZhi2, Unigene80494\_JingChaoZuZhi2, Unigene80605\_JingChaoZuZhi2, Unigene80612\_JingChaoZuZhi2, Unigene80863\_JingChaoZuZhi2, Unigene81395\_JingChaoZuZhi2, Unigene81692\_JingChaoZuZhi2, Unigene8261\_JingChaoZuZhi2, Unigene82842\_JingChaoZuZhi2, Unigene8310\_JingChaoZuZhi2, Unigene83400\_JingChaoZuZhi2, Unigene83448\_JingChaoZuZhi2, Unigene83487\_JingChaoZuZhi2, Unigene83602\_JingChaoZuZhi2, Unigene84232\_JingChaoZuZhi2, Unigene84510\_JingChaoZuZhi2, Unigene85122\_JingChaoZuZhi2, Unigene85132\_JingChaoZuZhi2, Unigene85387\_JingChaoZuZhi2, Unigene85658\_JingChaoZuZhi2, Unigene86051\_JingChaoZuZhi2, Unigene86312\_JingChaoZuZhi2, Unigene86616\_JingChaoZuZhi2, Unigene866\_JingChaoZuZhi2, Unigene86759\_JingChaoZuZhi2, Unigene8679\_JingChaoZuZhi2, Unigene86821\_JingChaoZuZhi2, Unigene87122\_JingChaoZuZhi2, Unigene87360\_JingChaoZuZhi2, Unigene87389\_JingChaoZuZhi2, Unigene87677\_JingChaoZuZhi2, Unigene87796\_JingChaoZuZhi2, Unigene87825\_JingChaoZuZhi2, Unigene87958\_JingChaoZuZhi2, Unigene88393\_JingChaoZuZhi2, Unigene88547\_JingChaoZuZhi2, Unigene89060\_JingChaoZuZhi2, Unigene89175\_JingChaoZuZhi2, Unigene89409\_JingChaoZuZhi2, Unigene89653\_JingChaoZuZhi2, Unigene89959\_JingChaoZuZhi2, Unigene90077\_JingChaoZuZhi2, Unigene90179\_JingChaoZuZhi2, Unigene90401\_JingChaoZuZhi2, Unigene90588\_JingChaoZuZhi2, Unigene90626\_JingChaoZuZhi2, Unigene90767\_JingChaoZuZhi2, Unigene91030\_JingChaoZuZhi2, Unigene91163\_JingChaoZuZhi2, Unigene91358\_JingChaoZuZhi2, Unigene91631\_JingChaoZuZhi2, Unigene91973\_JingChaoZuZhi2, Unigene91991\_JingChaoZuZhi2, Unigene92141\_JingChaoZuZhi2, Unigene92187\_JingChaoZuZhi2, Unigene92329\_JingChaoZuZhi2, Unigene92448\_JingChaoZuZhi2, Unigene92471\_JingChaoZuZhi2, Unigene92539\_JingChaoZuZhi2, Unigene92577\_JingChaoZuZhi2, Unigene92673\_JingChaoZuZhi2, Unigene92769\_JingChaoZuZhi2, Unigene93010\_JingChaoZuZhi2, Unigene93279\_JingChaoZuZhi2, Unigene93374\_JingChaoZuZhi2, Unigene93406\_JingChaoZuZhi2, Unigene93604\_JingChaoZuZhi2, Unigene93701\_JingChaoZuZhi2, Unigene93703\_JingChaoZuZhi2, Unigene93865\_JingChaoZuZhi2, Unigene94110\_JingChaoZuZhi2, Unigene94156\_JingChaoZuZhi2, Unigene94279\_JingChaoZuZhi2, Unigene944\_JingChaoZuZhi2, Unigene94723\_JingChaoZuZhi2, Unigene94823\_JingChaoZuZhi2, Unigene95031\_JingChaoZuZhi2, Unigene95041\_JingChaoZuZhi2, Unigene95059\_JingChaoZuZhi2, Unigene95213\_JingChaoZuZhi2, Unigene95243\_JingChaoZuZhi2, Unigene95486\_JingChaoZuZhi2, Unigene95776\_JingChaoZuZhi2, Unigene95879\_JingChaoZuZhi2, Unigene95882\_JingChaoZuZhi2, Unigene95954\_JingChaoZuZhi2, Unigene95972\_JingChaoZuZhi2, Unigene95982\_JingChaoZuZhi2, Unigene95987\_JingChaoZuZhi2, Unigene95994\_JingChaoZuZhi2, Unigene96012\_JingChaoZuZhi2, Unigene96061\_JingChaoZuZhi2, Unigene96083\_JingChaoZuZhi2, Unigene96097\_JingChaoZuZhi2, Unigene96206\_JingChaoZuZhi2, Unigene96217\_JingChaoZuZhi2, Unigene96243\_JingChaoZuZhi2, Unigene96247\_JingChaoZuZhi2, Unigene96288\_JingChaoZuZhi2, Unigene96292\_JingChaoZuZhi2, Unigene96303\_JingChaoZuZhi2, Unigene96308\_JingChaoZuZhi2, Unigene9739\_JingChaoZuZhi2, Unigene973\_JingChaoZuZhi2 |
| 43 | RNA polymerase | Unigene10005\_JingChaoZuZhi2, Unigene10129\_JingChaoZuZhi2, Unigene10340\_JingChaoZuZhi2, Unigene103\_JingChaoZuZhi2, Unigene10883\_JingChaoZuZhi2, Unigene10888\_JingChaoZuZhi2, Unigene10939\_JingChaoZuZhi2, Unigene10976\_JingChaoZuZhi2, Unigene11134\_JingChaoZuZhi2, Unigene1154\_JingChaoZuZhi2, Unigene11564\_JingChaoZuZhi2, Unigene1164\_JingChaoZuZhi2, Unigene11700\_JingChaoZuZhi2, Unigene1229\_JingChaoZuZhi2, Unigene1313\_JingChaoZuZhi2, Unigene13176\_JingChaoZuZhi2, Unigene1323\_JingChaoZuZhi2, Unigene13795\_JingChaoZuZhi2, Unigene13878\_JingChaoZuZhi2, Unigene13988\_JingChaoZuZhi2, Unigene14135\_JingChaoZuZhi2, Unigene14203\_JingChaoZuZhi2, Unigene14446\_JingChaoZuZhi2, Unigene14551\_JingChaoZuZhi2, Unigene14631\_JingChaoZuZhi2, Unigene1468\_JingChaoZuZhi2, Unigene15288\_JingChaoZuZhi2, Unigene15290\_JingChaoZuZhi2, Unigene15539\_JingChaoZuZhi2, Unigene16232\_JingChaoZuZhi2, Unigene1628\_JingChaoZuZhi2, Unigene16295\_JingChaoZuZhi2, Unigene16786\_JingChaoZuZhi2, Unigene16996\_JingChaoZuZhi2, Unigene17035\_JingChaoZuZhi2, Unigene17065\_JingChaoZuZhi2, Unigene17221\_JingChaoZuZhi2, Unigene1744\_JingChaoZuZhi2, Unigene17636\_JingChaoZuZhi2, Unigene18000\_JingChaoZuZhi2, Unigene18099\_JingChaoZuZhi2, Unigene18508\_JingChaoZuZhi2, Unigene18874\_JingChaoZuZhi2, Unigene19126\_JingChaoZuZhi2, Unigene19280\_JingChaoZuZhi2, Unigene19475\_JingChaoZuZhi2, Unigene19476\_JingChaoZuZhi2, Unigene19510\_JingChaoZuZhi2, Unigene19647\_JingChaoZuZhi2, Unigene19667\_JingChaoZuZhi2, Unigene19838\_JingChaoZuZhi2, Unigene19979\_JingChaoZuZhi2, Unigene20050\_JingChaoZuZhi2, Unigene20087\_JingChaoZuZhi2, Unigene2012\_JingChaoZuZhi2, Unigene20150\_JingChaoZuZhi2, Unigene20172\_JingChaoZuZhi2, Unigene20209\_JingChaoZuZhi2, Unigene20219\_JingChaoZuZhi2, Unigene20441\_JingChaoZuZhi2, Unigene2046\_JingChaoZuZhi2, Unigene20711\_JingChaoZuZhi2, Unigene20814\_JingChaoZuZhi2, Unigene21054\_JingChaoZuZhi2, Unigene2155\_JingChaoZuZhi2, Unigene21648\_JingChaoZuZhi2, Unigene22086\_JingChaoZuZhi2, Unigene22310\_JingChaoZuZhi2, Unigene22334\_JingChaoZuZhi2, Unigene22339\_JingChaoZuZhi2, Unigene22451\_JingChaoZuZhi2, Unigene22545\_JingChaoZuZhi2, Unigene2254\_JingChaoZuZhi2, Unigene22842\_JingChaoZuZhi2, Unigene22870\_JingChaoZuZhi2, Unigene22908\_JingChaoZuZhi2, Unigene22923\_JingChaoZuZhi2, Unigene23504\_JingChaoZuZhi2, Unigene25123\_JingChaoZuZhi2, Unigene26274\_JingChaoZuZhi2, Unigene2707\_JingChaoZuZhi2, Unigene29151\_JingChaoZuZhi2, Unigene2958\_JingChaoZuZhi2, Unigene30402\_JingChaoZuZhi2, Unigene30849\_JingChaoZuZhi2, Unigene31166\_JingChaoZuZhi2, Unigene31327\_JingChaoZuZhi2, Unigene31355\_JingChaoZuZhi2, Unigene3187\_JingChaoZuZhi2, Unigene322\_JingChaoZuZhi2, Unigene33470\_JingChaoZuZhi2, Unigene33523\_JingChaoZuZhi2, Unigene34338\_JingChaoZuZhi2, Unigene34397\_JingChaoZuZhi2, Unigene3592\_JingChaoZuZhi2, Unigene3632\_JingChaoZuZhi2, Unigene3682\_JingChaoZuZhi2, Unigene37224\_JingChaoZuZhi2, Unigene37457\_JingChaoZuZhi2, Unigene37504\_JingChaoZuZhi2, Unigene37516\_JingChaoZuZhi2, Unigene38162\_JingChaoZuZhi2, Unigene385\_JingChaoZuZhi2, Unigene38886\_JingChaoZuZhi2, Unigene38940\_JingChaoZuZhi2, Unigene3938\_JingChaoZuZhi2, Unigene39624\_JingChaoZuZhi2, Unigene41155\_JingChaoZuZhi2, Unigene41227\_JingChaoZuZhi2, Unigene414\_JingChaoZuZhi2, Unigene42364\_JingChaoZuZhi2, Unigene43072\_JingChaoZuZhi2, Unigene43410\_JingChaoZuZhi2, Unigene4346\_JingChaoZuZhi2, Unigene4375\_JingChaoZuZhi2, Unigene4377\_JingChaoZuZhi2, Unigene44252\_JingChaoZuZhi2, Unigene4426\_JingChaoZuZhi2, Unigene4461\_JingChaoZuZhi2, Unigene44745\_JingChaoZuZhi2, Unigene46190\_JingChaoZuZhi2, Unigene47202\_JingChaoZuZhi2, Unigene47794\_JingChaoZuZhi2, Unigene47824\_JingChaoZuZhi2, Unigene4816\_JingChaoZuZhi2, Unigene486\_JingChaoZuZhi2, Unigene49985\_JingChaoZuZhi2, Unigene50927\_JingChaoZuZhi2, Unigene51540\_JingChaoZuZhi2, Unigene51835\_JingChaoZuZhi2, Unigene5200\_JingChaoZuZhi2, Unigene52751\_JingChaoZuZhi2, Unigene53809\_JingChaoZuZhi2, Unigene542\_JingChaoZuZhi2, Unigene5484\_JingChaoZuZhi2, Unigene5552\_JingChaoZuZhi2, Unigene56889\_JingChaoZuZhi2, Unigene5897\_JingChaoZuZhi2, Unigene6134\_JingChaoZuZhi2, Unigene61651\_JingChaoZuZhi2, Unigene61659\_JingChaoZuZhi2, Unigene61\_JingChaoZuZhi2, Unigene6219\_JingChaoZuZhi2, Unigene630\_JingChaoZuZhi2, Unigene63112\_JingChaoZuZhi2, Unigene63170\_JingChaoZuZhi2, Unigene64903\_JingChaoZuZhi2, Unigene65405\_JingChaoZuZhi2, Unigene65475\_JingChaoZuZhi2, Unigene65515\_JingChaoZuZhi2, Unigene65800\_JingChaoZuZhi2, Unigene66954\_JingChaoZuZhi2, Unigene6790\_JingChaoZuZhi2, Unigene67964\_JingChaoZuZhi2, Unigene68129\_JingChaoZuZhi2, Unigene685\_JingChaoZuZhi2, Unigene6955\_JingChaoZuZhi2, Unigene69875\_JingChaoZuZhi2, Unigene70647\_JingChaoZuZhi2, Unigene71153\_JingChaoZuZhi2, Unigene7178\_JingChaoZuZhi2, Unigene7185\_JingChaoZuZhi2, Unigene72461\_JingChaoZuZhi2, Unigene73283\_JingChaoZuZhi2, Unigene73451\_JingChaoZuZhi2, Unigene74590\_JingChaoZuZhi2, Unigene76109\_JingChaoZuZhi2, Unigene76331\_JingChaoZuZhi2, Unigene76868\_JingChaoZuZhi2, Unigene77449\_JingChaoZuZhi2, Unigene77559\_JingChaoZuZhi2, Unigene77844\_JingChaoZuZhi2, Unigene78071\_JingChaoZuZhi2, Unigene78224\_JingChaoZuZhi2, Unigene78268\_JingChaoZuZhi2, Unigene78619\_JingChaoZuZhi2, Unigene79149\_JingChaoZuZhi2, Unigene79211\_JingChaoZuZhi2, Unigene7948\_JingChaoZuZhi2, Unigene7990\_JingChaoZuZhi2, Unigene79999\_JingChaoZuZhi2, Unigene81297\_JingChaoZuZhi2, Unigene81674\_JingChaoZuZhi2, Unigene8223\_JingChaoZuZhi2, Unigene8225\_JingChaoZuZhi2, Unigene82835\_JingChaoZuZhi2, Unigene83132\_JingChaoZuZhi2, Unigene84050\_JingChaoZuZhi2, Unigene84203\_JingChaoZuZhi2, Unigene84436\_JingChaoZuZhi2, Unigene8506\_JingChaoZuZhi2, Unigene8517\_JingChaoZuZhi2, Unigene85391\_JingChaoZuZhi2, Unigene86157\_JingChaoZuZhi2, Unigene8641\_JingChaoZuZhi2, Unigene86967\_JingChaoZuZhi2, Unigene87092\_JingChaoZuZhi2, Unigene8755\_JingChaoZuZhi2, Unigene87820\_JingChaoZuZhi2, Unigene87855\_JingChaoZuZhi2, Unigene87\_JingChaoZuZhi2, Unigene88016\_JingChaoZuZhi2, Unigene88179\_JingChaoZuZhi2, Unigene88283\_JingChaoZuZhi2, Unigene89375\_JingChaoZuZhi2, Unigene8973\_JingChaoZuZhi2, Unigene89753\_JingChaoZuZhi2, Unigene89769\_JingChaoZuZhi2, Unigene90180\_JingChaoZuZhi2, Unigene90612\_JingChaoZuZhi2, Unigene90919\_JingChaoZuZhi2, Unigene91020\_JingChaoZuZhi2, Unigene91122\_JingChaoZuZhi2, Unigene91187\_JingChaoZuZhi2, Unigene91194\_JingChaoZuZhi2, Unigene91513\_JingChaoZuZhi2, Unigene91530\_JingChaoZuZhi2, Unigene91777\_JingChaoZuZhi2, Unigene91921\_JingChaoZuZhi2, Unigene92224\_JingChaoZuZhi2, Unigene92240\_JingChaoZuZhi2, Unigene92368\_JingChaoZuZhi2, Unigene92607\_JingChaoZuZhi2, Unigene92742\_JingChaoZuZhi2, Unigene92908\_JingChaoZuZhi2, Unigene92910\_JingChaoZuZhi2, Unigene92989\_JingChaoZuZhi2, Unigene93445\_JingChaoZuZhi2, Unigene93904\_JingChaoZuZhi2, Unigene93993\_JingChaoZuZhi2, Unigene94118\_JingChaoZuZhi2, Unigene94128\_JingChaoZuZhi2, Unigene94364\_JingChaoZuZhi2, Unigene94457\_JingChaoZuZhi2, Unigene94568\_JingChaoZuZhi2, Unigene94630\_JingChaoZuZhi2, Unigene94831\_JingChaoZuZhi2, Unigene94910\_JingChaoZuZhi2, Unigene94946\_JingChaoZuZhi2, Unigene95002\_JingChaoZuZhi2, Unigene95237\_JingChaoZuZhi2, Unigene95476\_JingChaoZuZhi2, Unigene9550\_JingChaoZuZhi2, Unigene95677\_JingChaoZuZhi2, Unigene9569\_JingChaoZuZhi2, Unigene95855\_JingChaoZuZhi2, Unigene96171\_JingChaoZuZhi2, Unigene96240\_JingChaoZuZhi2, Unigene963\_JingChaoZuZhi2 |
| 44 | Insulin signaling pathway | Unigene10102\_JingChaoZuZhi2, Unigene1045\_JingChaoZuZhi2, Unigene10532\_JingChaoZuZhi2, Unigene11251\_JingChaoZuZhi2, Unigene11431\_JingChaoZuZhi2, Unigene11719\_JingChaoZuZhi2, Unigene11951\_JingChaoZuZhi2, Unigene11961\_JingChaoZuZhi2, Unigene12005\_JingChaoZuZhi2, Unigene12204\_JingChaoZuZhi2, Unigene12221\_JingChaoZuZhi2, Unigene12243\_JingChaoZuZhi2, Unigene12420\_JingChaoZuZhi2, Unigene12577\_JingChaoZuZhi2, Unigene12823\_JingChaoZuZhi2, Unigene12892\_JingChaoZuZhi2, Unigene12979\_JingChaoZuZhi2, Unigene13008\_JingChaoZuZhi2, Unigene13510\_JingChaoZuZhi2, Unigene13564\_JingChaoZuZhi2, Unigene13894\_JingChaoZuZhi2, Unigene14289\_JingChaoZuZhi2, Unigene14603\_JingChaoZuZhi2, Unigene14877\_JingChaoZuZhi2, Unigene14912\_JingChaoZuZhi2, Unigene14977\_JingChaoZuZhi2, Unigene15228\_JingChaoZuZhi2, Unigene16012\_JingChaoZuZhi2, Unigene16145\_JingChaoZuZhi2, Unigene16566\_JingChaoZuZhi2, Unigene16762\_JingChaoZuZhi2, Unigene16768\_JingChaoZuZhi2, Unigene16820\_JingChaoZuZhi2, Unigene17160\_JingChaoZuZhi2, Unigene17247\_JingChaoZuZhi2, Unigene17555\_JingChaoZuZhi2, Unigene17691\_JingChaoZuZhi2, Unigene17764\_JingChaoZuZhi2, Unigene17839\_JingChaoZuZhi2, Unigene17946\_JingChaoZuZhi2, Unigene18307\_JingChaoZuZhi2, Unigene18672\_JingChaoZuZhi2, Unigene18721\_JingChaoZuZhi2, Unigene18729\_JingChaoZuZhi2, Unigene18821\_JingChaoZuZhi2, Unigene18986\_JingChaoZuZhi2, Unigene19046\_JingChaoZuZhi2, Unigene19086\_JingChaoZuZhi2, Unigene19307\_JingChaoZuZhi2, Unigene20107\_JingChaoZuZhi2, Unigene20129\_JingChaoZuZhi2, Unigene20157\_JingChaoZuZhi2, Unigene20379\_JingChaoZuZhi2, Unigene20840\_JingChaoZuZhi2, Unigene20991\_JingChaoZuZhi2, Unigene21132\_JingChaoZuZhi2, Unigene21142\_JingChaoZuZhi2, Unigene21383\_JingChaoZuZhi2, Unigene21513\_JingChaoZuZhi2, Unigene21815\_JingChaoZuZhi2, Unigene22079\_JingChaoZuZhi2, Unigene22145\_JingChaoZuZhi2, Unigene22176\_JingChaoZuZhi2, Unigene22218\_JingChaoZuZhi2, Unigene22295\_JingChaoZuZhi2, Unigene22500\_JingChaoZuZhi2, Unigene22517\_JingChaoZuZhi2, Unigene22579\_JingChaoZuZhi2, Unigene22823\_JingChaoZuZhi2, Unigene22848\_JingChaoZuZhi2, Unigene2342\_JingChaoZuZhi2, Unigene2343\_JingChaoZuZhi2, Unigene2456\_JingChaoZuZhi2, Unigene25147\_JingChaoZuZhi2, Unigene26820\_JingChaoZuZhi2, Unigene28263\_JingChaoZuZhi2, Unigene28801\_JingChaoZuZhi2, Unigene31336\_JingChaoZuZhi2, Unigene32031\_JingChaoZuZhi2, Unigene37495\_JingChaoZuZhi2, Unigene3769\_JingChaoZuZhi2, Unigene3931\_JingChaoZuZhi2, Unigene40020\_JingChaoZuZhi2, Unigene4186\_JingChaoZuZhi2, Unigene42836\_JingChaoZuZhi2, Unigene4294\_JingChaoZuZhi2, Unigene4592\_JingChaoZuZhi2, Unigene47888\_JingChaoZuZhi2, Unigene4838\_JingChaoZuZhi2, Unigene48751\_JingChaoZuZhi2, Unigene48948\_JingChaoZuZhi2, Unigene49913\_JingChaoZuZhi2, Unigene50485\_JingChaoZuZhi2, Unigene51478\_JingChaoZuZhi2, Unigene51777\_JingChaoZuZhi2, Unigene51819\_JingChaoZuZhi2, Unigene52040\_JingChaoZuZhi2, Unigene52538\_JingChaoZuZhi2, Unigene52793\_JingChaoZuZhi2, Unigene53568\_JingChaoZuZhi2, Unigene54320\_JingChaoZuZhi2, Unigene5437\_JingChaoZuZhi2, Unigene55687\_JingChaoZuZhi2, Unigene56237\_JingChaoZuZhi2, Unigene5674\_JingChaoZuZhi2, Unigene57015\_JingChaoZuZhi2, Unigene57112\_JingChaoZuZhi2, Unigene57127\_JingChaoZuZhi2, Unigene57247\_JingChaoZuZhi2, Unigene5742\_JingChaoZuZhi2, Unigene57488\_JingChaoZuZhi2, Unigene58566\_JingChaoZuZhi2, Unigene60180\_JingChaoZuZhi2, Unigene6028\_JingChaoZuZhi2, Unigene60703\_JingChaoZuZhi2, Unigene6075\_JingChaoZuZhi2, Unigene60906\_JingChaoZuZhi2, Unigene61304\_JingChaoZuZhi2, Unigene6151\_JingChaoZuZhi2, Unigene62360\_JingChaoZuZhi2, Unigene64583\_JingChaoZuZhi2, Unigene64650\_JingChaoZuZhi2, Unigene6493\_JingChaoZuZhi2, Unigene66060\_JingChaoZuZhi2, Unigene66813\_JingChaoZuZhi2, Unigene67673\_JingChaoZuZhi2, Unigene67793\_JingChaoZuZhi2, Unigene67983\_JingChaoZuZhi2, Unigene68223\_JingChaoZuZhi2, Unigene68283\_JingChaoZuZhi2, Unigene6862\_JingChaoZuZhi2, Unigene68736\_JingChaoZuZhi2, Unigene70140\_JingChaoZuZhi2, Unigene70549\_JingChaoZuZhi2, Unigene7182\_JingChaoZuZhi2, Unigene71974\_JingChaoZuZhi2, Unigene7224\_JingChaoZuZhi2, Unigene72972\_JingChaoZuZhi2, Unigene75617\_JingChaoZuZhi2, Unigene76400\_JingChaoZuZhi2, Unigene76809\_JingChaoZuZhi2, Unigene77114\_JingChaoZuZhi2, Unigene77207\_JingChaoZuZhi2, Unigene77374\_JingChaoZuZhi2, Unigene77570\_JingChaoZuZhi2, Unigene77649\_JingChaoZuZhi2, Unigene78235\_JingChaoZuZhi2, Unigene79539\_JingChaoZuZhi2, Unigene79681\_JingChaoZuZhi2, Unigene79882\_JingChaoZuZhi2, Unigene80251\_JingChaoZuZhi2, Unigene80504\_JingChaoZuZhi2, Unigene80971\_JingChaoZuZhi2, Unigene81780\_JingChaoZuZhi2, Unigene8191\_JingChaoZuZhi2, Unigene83318\_JingChaoZuZhi2, Unigene83440\_JingChaoZuZhi2, Unigene83531\_JingChaoZuZhi2, Unigene83588\_JingChaoZuZhi2, Unigene83663\_JingChaoZuZhi2, Unigene84024\_JingChaoZuZhi2, Unigene84222\_JingChaoZuZhi2, Unigene84340\_JingChaoZuZhi2, Unigene84418\_JingChaoZuZhi2, Unigene8464\_JingChaoZuZhi2, Unigene84839\_JingChaoZuZhi2, Unigene85172\_JingChaoZuZhi2, Unigene85574\_JingChaoZuZhi2, Unigene85576\_JingChaoZuZhi2, Unigene85769\_JingChaoZuZhi2, Unigene86036\_JingChaoZuZhi2, Unigene86206\_JingChaoZuZhi2, Unigene86278\_JingChaoZuZhi2, Unigene86456\_JingChaoZuZhi2, Unigene86526\_JingChaoZuZhi2, Unigene8716\_JingChaoZuZhi2, Unigene87251\_JingChaoZuZhi2, Unigene87524\_JingChaoZuZhi2, Unigene87565\_JingChaoZuZhi2, Unigene876\_JingChaoZuZhi2, Unigene88101\_JingChaoZuZhi2, Unigene88594\_JingChaoZuZhi2, Unigene88719\_JingChaoZuZhi2, Unigene88724\_JingChaoZuZhi2, Unigene89310\_JingChaoZuZhi2, Unigene89444\_JingChaoZuZhi2, Unigene90122\_JingChaoZuZhi2, Unigene90209\_JingChaoZuZhi2, Unigene90979\_JingChaoZuZhi2, Unigene91175\_JingChaoZuZhi2, Unigene91382\_JingChaoZuZhi2, Unigene91527\_JingChaoZuZhi2, Unigene91538\_JingChaoZuZhi2, Unigene91605\_JingChaoZuZhi2, Unigene91612\_JingChaoZuZhi2, Unigene91816\_JingChaoZuZhi2, Unigene91837\_JingChaoZuZhi2, Unigene91895\_JingChaoZuZhi2, Unigene91910\_JingChaoZuZhi2, Unigene91924\_JingChaoZuZhi2, Unigene91958\_JingChaoZuZhi2, Unigene92112\_JingChaoZuZhi2, Unigene92251\_JingChaoZuZhi2, Unigene92580\_JingChaoZuZhi2, Unigene92957\_JingChaoZuZhi2, Unigene9295\_JingChaoZuZhi2, Unigene92978\_JingChaoZuZhi2, Unigene93409\_JingChaoZuZhi2, Unigene93588\_JingChaoZuZhi2, Unigene93714\_JingChaoZuZhi2, Unigene93774\_JingChaoZuZhi2, Unigene93807\_JingChaoZuZhi2, Unigene9404\_JingChaoZuZhi2, Unigene94149\_JingChaoZuZhi2, Unigene94178\_JingChaoZuZhi2, Unigene94208\_JingChaoZuZhi2, Unigene94287\_JingChaoZuZhi2, Unigene94456\_JingChaoZuZhi2, Unigene94544\_JingChaoZuZhi2, Unigene94685\_JingChaoZuZhi2, Unigene95098\_JingChaoZuZhi2, Unigene95295\_JingChaoZuZhi2, Unigene9530\_JingChaoZuZhi2, Unigene95350\_JingChaoZuZhi2, Unigene95359\_JingChaoZuZhi2, Unigene95443\_JingChaoZuZhi2, Unigene95456\_JingChaoZuZhi2, Unigene95773\_JingChaoZuZhi2, Unigene95921\_JingChaoZuZhi2, Unigene95947\_JingChaoZuZhi2, Unigene95969\_JingChaoZuZhi2, Unigene95993\_JingChaoZuZhi2, Unigene96064\_JingChaoZuZhi2, Unigene96263\_JingChaoZuZhi2, Unigene96294\_JingChaoZuZhi2, Unigene9767\_JingChaoZuZhi2, Unigene9902\_JingChaoZuZhi2 |
| 45 | Hedgehog signaling pathway | Unigene10108\_JingChaoZuZhi2, Unigene1026\_JingChaoZuZhi2, Unigene1030\_JingChaoZuZhi2, Unigene10326\_JingChaoZuZhi2, Unigene1041\_JingChaoZuZhi2, Unigene1072\_JingChaoZuZhi2, Unigene11301\_JingChaoZuZhi2, Unigene1136\_JingChaoZuZhi2, Unigene1143\_JingChaoZuZhi2, Unigene11543\_JingChaoZuZhi2, Unigene11571\_JingChaoZuZhi2, Unigene11757\_JingChaoZuZhi2, Unigene11758\_JingChaoZuZhi2, Unigene11802\_JingChaoZuZhi2, Unigene12722\_JingChaoZuZhi2, Unigene12864\_JingChaoZuZhi2, Unigene1303\_JingChaoZuZhi2, Unigene13157\_JingChaoZuZhi2, Unigene13230\_JingChaoZuZhi2, Unigene1324\_JingChaoZuZhi2, Unigene1352\_JingChaoZuZhi2, Unigene1371\_JingChaoZuZhi2, Unigene13760\_JingChaoZuZhi2, Unigene14170\_JingChaoZuZhi2, Unigene14368\_JingChaoZuZhi2, Unigene1472\_JingChaoZuZhi2, Unigene1473\_JingChaoZuZhi2, Unigene14855\_JingChaoZuZhi2, Unigene14874\_JingChaoZuZhi2, Unigene1515\_JingChaoZuZhi2, Unigene1527\_JingChaoZuZhi2, Unigene1540\_JingChaoZuZhi2, Unigene15909\_JingChaoZuZhi2, Unigene16116\_JingChaoZuZhi2, Unigene16147\_JingChaoZuZhi2, Unigene16820\_JingChaoZuZhi2, Unigene1808\_JingChaoZuZhi2, Unigene18217\_JingChaoZuZhi2, Unigene18307\_JingChaoZuZhi2, Unigene18609\_JingChaoZuZhi2, Unigene18652\_JingChaoZuZhi2, Unigene19230\_JingChaoZuZhi2, Unigene19425\_JingChaoZuZhi2, Unigene19789\_JingChaoZuZhi2, Unigene19852\_JingChaoZuZhi2, Unigene21166\_JingChaoZuZhi2, Unigene2118\_JingChaoZuZhi2, Unigene21222\_JingChaoZuZhi2, Unigene21423\_JingChaoZuZhi2, Unigene21453\_JingChaoZuZhi2, Unigene2149\_JingChaoZuZhi2, Unigene21539\_JingChaoZuZhi2, Unigene21598\_JingChaoZuZhi2, Unigene21772\_JingChaoZuZhi2, Unigene21815\_JingChaoZuZhi2, Unigene2231\_JingChaoZuZhi2, Unigene22502\_JingChaoZuZhi2, Unigene22763\_JingChaoZuZhi2, Unigene22787\_JingChaoZuZhi2, Unigene22936\_JingChaoZuZhi2, Unigene23122\_JingChaoZuZhi2, Unigene23885\_JingChaoZuZhi2, Unigene2453\_JingChaoZuZhi2, Unigene2488\_JingChaoZuZhi2, Unigene25435\_JingChaoZuZhi2, Unigene25675\_JingChaoZuZhi2, Unigene26603\_JingChaoZuZhi2, Unigene27196\_JingChaoZuZhi2, Unigene27217\_JingChaoZuZhi2, Unigene27868\_JingChaoZuZhi2, Unigene28357\_JingChaoZuZhi2, Unigene28752\_JingChaoZuZhi2, Unigene28996\_JingChaoZuZhi2, Unigene29281\_JingChaoZuZhi2, Unigene30419\_JingChaoZuZhi2, Unigene32081\_JingChaoZuZhi2, Unigene33155\_JingChaoZuZhi2, Unigene33393\_JingChaoZuZhi2, Unigene33536\_JingChaoZuZhi2, Unigene34055\_JingChaoZuZhi2, Unigene35755\_JingChaoZuZhi2, Unigene35791\_JingChaoZuZhi2, Unigene37387\_JingChaoZuZhi2, Unigene3762\_JingChaoZuZhi2, Unigene38121\_JingChaoZuZhi2, Unigene38311\_JingChaoZuZhi2, Unigene38765\_JingChaoZuZhi2, Unigene38983\_JingChaoZuZhi2, Unigene39197\_JingChaoZuZhi2, Unigene39806\_JingChaoZuZhi2, Unigene40238\_JingChaoZuZhi2, Unigene41120\_JingChaoZuZhi2, Unigene41367\_JingChaoZuZhi2, Unigene42422\_JingChaoZuZhi2, Unigene45194\_JingChaoZuZhi2, Unigene45309\_JingChaoZuZhi2, Unigene45352\_JingChaoZuZhi2, Unigene45662\_JingChaoZuZhi2, Unigene46021\_JingChaoZuZhi2, Unigene4614\_JingChaoZuZhi2, Unigene46175\_JingChaoZuZhi2, Unigene46526\_JingChaoZuZhi2, Unigene4678\_JingChaoZuZhi2, Unigene47389\_JingChaoZuZhi2, Unigene47663\_JingChaoZuZhi2, Unigene4783\_JingChaoZuZhi2, Unigene48228\_JingChaoZuZhi2, Unigene48789\_JingChaoZuZhi2, Unigene48816\_JingChaoZuZhi2, Unigene4918\_JingChaoZuZhi2, Unigene49694\_JingChaoZuZhi2, Unigene50572\_JingChaoZuZhi2, Unigene51679\_JingChaoZuZhi2, Unigene51703\_JingChaoZuZhi2, Unigene51994\_JingChaoZuZhi2, Unigene53103\_JingChaoZuZhi2, Unigene53165\_JingChaoZuZhi2, Unigene55804\_JingChaoZuZhi2, Unigene56569\_JingChaoZuZhi2, Unigene56652\_JingChaoZuZhi2, Unigene56748\_JingChaoZuZhi2, Unigene57174\_JingChaoZuZhi2, Unigene57694\_JingChaoZuZhi2, Unigene57752\_JingChaoZuZhi2, Unigene5863\_JingChaoZuZhi2, Unigene58762\_JingChaoZuZhi2, Unigene59250\_JingChaoZuZhi2, Unigene59263\_JingChaoZuZhi2, Unigene59794\_JingChaoZuZhi2, Unigene60707\_JingChaoZuZhi2, Unigene60891\_JingChaoZuZhi2, Unigene60975\_JingChaoZuZhi2, Unigene61457\_JingChaoZuZhi2, Unigene61486\_JingChaoZuZhi2, Unigene616\_JingChaoZuZhi2, Unigene62341\_JingChaoZuZhi2, Unigene62403\_JingChaoZuZhi2, Unigene62419\_JingChaoZuZhi2, Unigene62495\_JingChaoZuZhi2, Unigene63006\_JingChaoZuZhi2, Unigene63124\_JingChaoZuZhi2, Unigene63819\_JingChaoZuZhi2, Unigene63933\_JingChaoZuZhi2, Unigene64033\_JingChaoZuZhi2, Unigene64367\_JingChaoZuZhi2, Unigene64426\_JingChaoZuZhi2, Unigene64690\_JingChaoZuZhi2, Unigene65464\_JingChaoZuZhi2, Unigene65649\_JingChaoZuZhi2, Unigene65875\_JingChaoZuZhi2, Unigene6675\_JingChaoZuZhi2, Unigene66886\_JingChaoZuZhi2, Unigene68113\_JingChaoZuZhi2, Unigene68841\_JingChaoZuZhi2, Unigene69585\_JingChaoZuZhi2, Unigene69733\_JingChaoZuZhi2, Unigene70929\_JingChaoZuZhi2, Unigene71051\_JingChaoZuZhi2, Unigene7124\_JingChaoZuZhi2, Unigene71561\_JingChaoZuZhi2, Unigene71597\_JingChaoZuZhi2, Unigene7195\_JingChaoZuZhi2, Unigene71972\_JingChaoZuZhi2, Unigene72223\_JingChaoZuZhi2, Unigene72337\_JingChaoZuZhi2, Unigene72782\_JingChaoZuZhi2, Unigene72809\_JingChaoZuZhi2, Unigene72887\_JingChaoZuZhi2, Unigene72906\_JingChaoZuZhi2, Unigene74030\_JingChaoZuZhi2, Unigene74210\_JingChaoZuZhi2, Unigene74832\_JingChaoZuZhi2, Unigene75462\_JingChaoZuZhi2, Unigene75839\_JingChaoZuZhi2, Unigene76010\_JingChaoZuZhi2, Unigene76145\_JingChaoZuZhi2, Unigene76747\_JingChaoZuZhi2, Unigene77035\_JingChaoZuZhi2, Unigene77319\_JingChaoZuZhi2, Unigene77369\_JingChaoZuZhi2, Unigene77618\_JingChaoZuZhi2, Unigene78235\_JingChaoZuZhi2, Unigene78341\_JingChaoZuZhi2, Unigene7864\_JingChaoZuZhi2, Unigene79152\_JingChaoZuZhi2, Unigene79482\_JingChaoZuZhi2, Unigene79534\_JingChaoZuZhi2, Unigene79597\_JingChaoZuZhi2, Unigene8035\_JingChaoZuZhi2, Unigene80376\_JingChaoZuZhi2, Unigene80762\_JingChaoZuZhi2, Unigene80793\_JingChaoZuZhi2, Unigene80927\_JingChaoZuZhi2, Unigene82359\_JingChaoZuZhi2, Unigene83207\_JingChaoZuZhi2, Unigene84032\_JingChaoZuZhi2, Unigene84080\_JingChaoZuZhi2, Unigene84576\_JingChaoZuZhi2, Unigene84693\_JingChaoZuZhi2, Unigene86114\_JingChaoZuZhi2, Unigene86589\_JingChaoZuZhi2, Unigene86996\_JingChaoZuZhi2, Unigene87474\_JingChaoZuZhi2, Unigene8764\_JingChaoZuZhi2, Unigene88053\_JingChaoZuZhi2, Unigene88054\_JingChaoZuZhi2, Unigene88517\_JingChaoZuZhi2, Unigene88719\_JingChaoZuZhi2, Unigene89191\_JingChaoZuZhi2, Unigene89310\_JingChaoZuZhi2, Unigene89790\_JingChaoZuZhi2, Unigene9029\_JingChaoZuZhi2, Unigene90527\_JingChaoZuZhi2, Unigene91423\_JingChaoZuZhi2, Unigene91527\_JingChaoZuZhi2, Unigene91774\_JingChaoZuZhi2, Unigene92033\_JingChaoZuZhi2, Unigene93178\_JingChaoZuZhi2, Unigene93479\_JingChaoZuZhi2, Unigene93752\_JingChaoZuZhi2, Unigene94819\_JingChaoZuZhi2, Unigene94956\_JingChaoZuZhi2, Unigene94983\_JingChaoZuZhi2, Unigene95058\_JingChaoZuZhi2, Unigene95098\_JingChaoZuZhi2, Unigene95295\_JingChaoZuZhi2, Unigene95297\_JingChaoZuZhi2, Unigene95441\_JingChaoZuZhi2, Unigene95485\_JingChaoZuZhi2, Unigene9587\_JingChaoZuZhi2, Unigene96127\_JingChaoZuZhi2, Unigene96249\_JingChaoZuZhi2, Unigene9756\_JingChaoZuZhi2, Unigene9804\_JingChaoZuZhi2, Unigene9948\_JingChaoZuZhi2, Unigene9961\_JingChaoZuZhi2 |
| 46 | Basal cell carcinoma | Unigene10108\_JingChaoZuZhi2, Unigene1026\_JingChaoZuZhi2, Unigene1030\_JingChaoZuZhi2, Unigene1041\_JingChaoZuZhi2, Unigene1072\_JingChaoZuZhi2, Unigene11301\_JingChaoZuZhi2, Unigene1136\_JingChaoZuZhi2, Unigene1143\_JingChaoZuZhi2, Unigene11543\_JingChaoZuZhi2, Unigene11571\_JingChaoZuZhi2, Unigene11757\_JingChaoZuZhi2, Unigene11758\_JingChaoZuZhi2, Unigene11802\_JingChaoZuZhi2, Unigene12455\_JingChaoZuZhi2, Unigene12722\_JingChaoZuZhi2, Unigene1303\_JingChaoZuZhi2, Unigene13157\_JingChaoZuZhi2, Unigene13230\_JingChaoZuZhi2, Unigene1324\_JingChaoZuZhi2, Unigene1352\_JingChaoZuZhi2, Unigene1371\_JingChaoZuZhi2, Unigene13760\_JingChaoZuZhi2, Unigene13869\_JingChaoZuZhi2, Unigene14368\_JingChaoZuZhi2, Unigene1472\_JingChaoZuZhi2, Unigene1473\_JingChaoZuZhi2, Unigene14855\_JingChaoZuZhi2, Unigene1515\_JingChaoZuZhi2, Unigene1527\_JingChaoZuZhi2, Unigene1540\_JingChaoZuZhi2, Unigene15909\_JingChaoZuZhi2, Unigene16116\_JingChaoZuZhi2, Unigene16147\_JingChaoZuZhi2, Unigene16229\_JingChaoZuZhi2, Unigene16820\_JingChaoZuZhi2, Unigene17800\_JingChaoZuZhi2, Unigene1808\_JingChaoZuZhi2, Unigene18095\_JingChaoZuZhi2, Unigene18177\_JingChaoZuZhi2, Unigene18217\_JingChaoZuZhi2, Unigene18609\_JingChaoZuZhi2, Unigene18817\_JingChaoZuZhi2, Unigene18932\_JingChaoZuZhi2, Unigene19230\_JingChaoZuZhi2, Unigene19465\_JingChaoZuZhi2, Unigene19789\_JingChaoZuZhi2, Unigene19852\_JingChaoZuZhi2, Unigene21166\_JingChaoZuZhi2, Unigene2118\_JingChaoZuZhi2, Unigene2149\_JingChaoZuZhi2, Unigene21772\_JingChaoZuZhi2, Unigene2231\_JingChaoZuZhi2, Unigene22506\_JingChaoZuZhi2, Unigene22936\_JingChaoZuZhi2, Unigene23122\_JingChaoZuZhi2, Unigene23885\_JingChaoZuZhi2, Unigene2453\_JingChaoZuZhi2, Unigene2488\_JingChaoZuZhi2, Unigene25435\_JingChaoZuZhi2, Unigene25675\_JingChaoZuZhi2, Unigene26603\_JingChaoZuZhi2, Unigene27196\_JingChaoZuZhi2, Unigene27217\_JingChaoZuZhi2, Unigene27868\_JingChaoZuZhi2, Unigene28357\_JingChaoZuZhi2, Unigene28752\_JingChaoZuZhi2, Unigene29281\_JingChaoZuZhi2, Unigene30419\_JingChaoZuZhi2, Unigene30787\_JingChaoZuZhi2, Unigene33155\_JingChaoZuZhi2, Unigene33393\_JingChaoZuZhi2, Unigene33536\_JingChaoZuZhi2, Unigene33683\_JingChaoZuZhi2, Unigene34055\_JingChaoZuZhi2, Unigene34463\_JingChaoZuZhi2, Unigene34574\_JingChaoZuZhi2, Unigene34723\_JingChaoZuZhi2, Unigene35755\_JingChaoZuZhi2, Unigene35791\_JingChaoZuZhi2, Unigene3762\_JingChaoZuZhi2, Unigene37739\_JingChaoZuZhi2, Unigene38121\_JingChaoZuZhi2, Unigene38191\_JingChaoZuZhi2, Unigene38311\_JingChaoZuZhi2, Unigene38765\_JingChaoZuZhi2, Unigene38983\_JingChaoZuZhi2, Unigene39197\_JingChaoZuZhi2, Unigene40238\_JingChaoZuZhi2, Unigene41120\_JingChaoZuZhi2, Unigene41367\_JingChaoZuZhi2, Unigene42422\_JingChaoZuZhi2, Unigene44228\_JingChaoZuZhi2, Unigene45166\_JingChaoZuZhi2, Unigene45194\_JingChaoZuZhi2, Unigene45309\_JingChaoZuZhi2, Unigene45352\_JingChaoZuZhi2, Unigene45662\_JingChaoZuZhi2, Unigene46021\_JingChaoZuZhi2, Unigene46175\_JingChaoZuZhi2, Unigene4678\_JingChaoZuZhi2, Unigene4803\_JingChaoZuZhi2, Unigene48228\_JingChaoZuZhi2, Unigene48816\_JingChaoZuZhi2, Unigene4886\_JingChaoZuZhi2, Unigene50572\_JingChaoZuZhi2, Unigene51679\_JingChaoZuZhi2, Unigene51703\_JingChaoZuZhi2, Unigene5226\_JingChaoZuZhi2, Unigene53103\_JingChaoZuZhi2, Unigene53421\_JingChaoZuZhi2, Unigene55392\_JingChaoZuZhi2, Unigene55804\_JingChaoZuZhi2, Unigene56569\_JingChaoZuZhi2, Unigene56652\_JingChaoZuZhi2, Unigene56709\_JingChaoZuZhi2, Unigene56922\_JingChaoZuZhi2, Unigene57174\_JingChaoZuZhi2, Unigene57694\_JingChaoZuZhi2, Unigene57752\_JingChaoZuZhi2, Unigene5863\_JingChaoZuZhi2, Unigene58762\_JingChaoZuZhi2, Unigene58806\_JingChaoZuZhi2, Unigene59250\_JingChaoZuZhi2, Unigene59263\_JingChaoZuZhi2, Unigene59794\_JingChaoZuZhi2, Unigene60707\_JingChaoZuZhi2, Unigene60777\_JingChaoZuZhi2, Unigene60891\_JingChaoZuZhi2, Unigene61043\_JingChaoZuZhi2, Unigene61457\_JingChaoZuZhi2, Unigene61486\_JingChaoZuZhi2, Unigene616\_JingChaoZuZhi2, Unigene61911\_JingChaoZuZhi2, Unigene62341\_JingChaoZuZhi2, Unigene62403\_JingChaoZuZhi2, Unigene62419\_JingChaoZuZhi2, Unigene62495\_JingChaoZuZhi2, Unigene63449\_JingChaoZuZhi2, Unigene63819\_JingChaoZuZhi2, Unigene63933\_JingChaoZuZhi2, Unigene64033\_JingChaoZuZhi2, Unigene64227\_JingChaoZuZhi2, Unigene64367\_JingChaoZuZhi2, Unigene64426\_JingChaoZuZhi2, Unigene64678\_JingChaoZuZhi2, Unigene64690\_JingChaoZuZhi2, Unigene65464\_JingChaoZuZhi2, Unigene65649\_JingChaoZuZhi2, Unigene65875\_JingChaoZuZhi2, Unigene6675\_JingChaoZuZhi2, Unigene66865\_JingChaoZuZhi2, Unigene66886\_JingChaoZuZhi2, Unigene68056\_JingChaoZuZhi2, Unigene69733\_JingChaoZuZhi2, Unigene70929\_JingChaoZuZhi2, Unigene71051\_JingChaoZuZhi2, Unigene7124\_JingChaoZuZhi2, Unigene71561\_JingChaoZuZhi2, Unigene7195\_JingChaoZuZhi2, Unigene71972\_JingChaoZuZhi2, Unigene72223\_JingChaoZuZhi2, Unigene72809\_JingChaoZuZhi2, Unigene72887\_JingChaoZuZhi2, Unigene72906\_JingChaoZuZhi2, Unigene73203\_JingChaoZuZhi2, Unigene73873\_JingChaoZuZhi2, Unigene74210\_JingChaoZuZhi2, Unigene74832\_JingChaoZuZhi2, Unigene75228\_JingChaoZuZhi2, Unigene75462\_JingChaoZuZhi2, Unigene75839\_JingChaoZuZhi2, Unigene76145\_JingChaoZuZhi2, Unigene76614\_JingChaoZuZhi2, Unigene76747\_JingChaoZuZhi2, Unigene77369\_JingChaoZuZhi2, Unigene77587\_JingChaoZuZhi2, Unigene78168\_JingChaoZuZhi2, Unigene78241\_JingChaoZuZhi2, Unigene78390\_JingChaoZuZhi2, Unigene7864\_JingChaoZuZhi2, Unigene79118\_JingChaoZuZhi2, Unigene79152\_JingChaoZuZhi2, Unigene79534\_JingChaoZuZhi2, Unigene79597\_JingChaoZuZhi2, Unigene80376\_JingChaoZuZhi2, Unigene8054\_JingChaoZuZhi2, Unigene80762\_JingChaoZuZhi2, Unigene80793\_JingChaoZuZhi2, Unigene82359\_JingChaoZuZhi2, Unigene82525\_JingChaoZuZhi2, Unigene83207\_JingChaoZuZhi2, Unigene84032\_JingChaoZuZhi2, Unigene84576\_JingChaoZuZhi2, Unigene84930\_JingChaoZuZhi2, Unigene8523\_JingChaoZuZhi2, Unigene86114\_JingChaoZuZhi2, Unigene86589\_JingChaoZuZhi2, Unigene8799\_JingChaoZuZhi2, Unigene88053\_JingChaoZuZhi2, Unigene88167\_JingChaoZuZhi2, Unigene88544\_JingChaoZuZhi2, Unigene88719\_JingChaoZuZhi2, Unigene89191\_JingChaoZuZhi2, Unigene89310\_JingChaoZuZhi2, Unigene89790\_JingChaoZuZhi2, Unigene9029\_JingChaoZuZhi2, Unigene90761\_JingChaoZuZhi2, Unigene91423\_JingChaoZuZhi2, Unigene92309\_JingChaoZuZhi2, Unigene92651\_JingChaoZuZhi2, Unigene93133\_JingChaoZuZhi2, Unigene94272\_JingChaoZuZhi2, Unigene94795\_JingChaoZuZhi2, Unigene94819\_JingChaoZuZhi2, Unigene94956\_JingChaoZuZhi2, Unigene95014\_JingChaoZuZhi2, Unigene95098\_JingChaoZuZhi2, Unigene95295\_JingChaoZuZhi2, Unigene95297\_JingChaoZuZhi2, Unigene95441\_JingChaoZuZhi2, Unigene95485\_JingChaoZuZhi2, Unigene95751\_JingChaoZuZhi2, Unigene96234\_JingChaoZuZhi2, Unigene9756\_JingChaoZuZhi2, Unigene9804\_JingChaoZuZhi2, Unigene9948\_JingChaoZuZhi2 |
| 47 | Pancreatic secretion | Unigene10218\_JingChaoZuZhi2, Unigene10268\_JingChaoZuZhi2, Unigene10318\_JingChaoZuZhi2, Unigene10414\_JingChaoZuZhi2, Unigene11055\_JingChaoZuZhi2, Unigene11726\_JingChaoZuZhi2, Unigene12073\_JingChaoZuZhi2, Unigene12260\_JingChaoZuZhi2, Unigene12938\_JingChaoZuZhi2, Unigene13276\_JingChaoZuZhi2, Unigene13393\_JingChaoZuZhi2, Unigene13420\_JingChaoZuZhi2, Unigene13485\_JingChaoZuZhi2, Unigene13538\_JingChaoZuZhi2, Unigene13770\_JingChaoZuZhi2, Unigene13771\_JingChaoZuZhi2, Unigene13869\_JingChaoZuZhi2, Unigene14095\_JingChaoZuZhi2, Unigene14357\_JingChaoZuZhi2, Unigene14360\_JingChaoZuZhi2, Unigene14567\_JingChaoZuZhi2, Unigene14906\_JingChaoZuZhi2, Unigene15425\_JingChaoZuZhi2, Unigene15577\_JingChaoZuZhi2, Unigene15626\_JingChaoZuZhi2, Unigene15651\_JingChaoZuZhi2, Unigene15942\_JingChaoZuZhi2, Unigene16210\_JingChaoZuZhi2, Unigene16991\_JingChaoZuZhi2, Unigene17462\_JingChaoZuZhi2, Unigene18977\_JingChaoZuZhi2, Unigene19021\_JingChaoZuZhi2, Unigene19196\_JingChaoZuZhi2, Unigene19904\_JingChaoZuZhi2, Unigene20000\_JingChaoZuZhi2, Unigene2015\_JingChaoZuZhi2, Unigene20356\_JingChaoZuZhi2, Unigene20427\_JingChaoZuZhi2, Unigene20631\_JingChaoZuZhi2, Unigene20651\_JingChaoZuZhi2, Unigene20693\_JingChaoZuZhi2, Unigene21354\_JingChaoZuZhi2, Unigene21407\_JingChaoZuZhi2, Unigene21578\_JingChaoZuZhi2, Unigene21663\_JingChaoZuZhi2, Unigene21792\_JingChaoZuZhi2, Unigene21866\_JingChaoZuZhi2, Unigene21946\_JingChaoZuZhi2, Unigene22002\_JingChaoZuZhi2, Unigene22136\_JingChaoZuZhi2, Unigene22362\_JingChaoZuZhi2, Unigene22507\_JingChaoZuZhi2, Unigene22779\_JingChaoZuZhi2, Unigene24506\_JingChaoZuZhi2, Unigene2519\_JingChaoZuZhi2, Unigene2544\_JingChaoZuZhi2, Unigene26730\_JingChaoZuZhi2, Unigene28522\_JingChaoZuZhi2, Unigene29180\_JingChaoZuZhi2, Unigene2960\_JingChaoZuZhi2, Unigene3018\_JingChaoZuZhi2, Unigene3122\_JingChaoZuZhi2, Unigene31660\_JingChaoZuZhi2, Unigene32417\_JingChaoZuZhi2, Unigene32827\_JingChaoZuZhi2, Unigene33704\_JingChaoZuZhi2, Unigene34429\_JingChaoZuZhi2, Unigene34929\_JingChaoZuZhi2, Unigene36404\_JingChaoZuZhi2, Unigene36790\_JingChaoZuZhi2, Unigene38950\_JingChaoZuZhi2, Unigene39299\_JingChaoZuZhi2, Unigene39742\_JingChaoZuZhi2, Unigene39832\_JingChaoZuZhi2, Unigene39892\_JingChaoZuZhi2, Unigene40335\_JingChaoZuZhi2, Unigene41333\_JingChaoZuZhi2, Unigene42033\_JingChaoZuZhi2, Unigene42329\_JingChaoZuZhi2, Unigene43059\_JingChaoZuZhi2, Unigene43723\_JingChaoZuZhi2, Unigene4522\_JingChaoZuZhi2, Unigene45532\_JingChaoZuZhi2, Unigene45644\_JingChaoZuZhi2, Unigene47543\_JingChaoZuZhi2, Unigene4760\_JingChaoZuZhi2, Unigene4762\_JingChaoZuZhi2, Unigene4782\_JingChaoZuZhi2, Unigene48576\_JingChaoZuZhi2, Unigene49203\_JingChaoZuZhi2, Unigene49504\_JingChaoZuZhi2, Unigene49554\_JingChaoZuZhi2, Unigene49757\_JingChaoZuZhi2, Unigene51991\_JingChaoZuZhi2, Unigene52167\_JingChaoZuZhi2, Unigene53284\_JingChaoZuZhi2, Unigene53935\_JingChaoZuZhi2, Unigene54012\_JingChaoZuZhi2, Unigene5401\_JingChaoZuZhi2, Unigene5423\_JingChaoZuZhi2, Unigene54367\_JingChaoZuZhi2, Unigene54449\_JingChaoZuZhi2, Unigene5545\_JingChaoZuZhi2, Unigene55865\_JingChaoZuZhi2, Unigene5589\_JingChaoZuZhi2, Unigene56513\_JingChaoZuZhi2, Unigene57185\_JingChaoZuZhi2, Unigene57226\_JingChaoZuZhi2, Unigene5799\_JingChaoZuZhi2, Unigene59102\_JingChaoZuZhi2, Unigene60117\_JingChaoZuZhi2, Unigene60768\_JingChaoZuZhi2, Unigene61354\_JingChaoZuZhi2, Unigene62059\_JingChaoZuZhi2, Unigene62724\_JingChaoZuZhi2, Unigene63391\_JingChaoZuZhi2, Unigene6393\_JingChaoZuZhi2, Unigene63994\_JingChaoZuZhi2, Unigene64164\_JingChaoZuZhi2, Unigene64830\_JingChaoZuZhi2, Unigene65445\_JingChaoZuZhi2, Unigene66073\_JingChaoZuZhi2, Unigene66540\_JingChaoZuZhi2, Unigene67011\_JingChaoZuZhi2, Unigene67328\_JingChaoZuZhi2, Unigene6776\_JingChaoZuZhi2, Unigene68151\_JingChaoZuZhi2, Unigene6818\_JingChaoZuZhi2, Unigene68275\_JingChaoZuZhi2, Unigene68356\_JingChaoZuZhi2, Unigene69551\_JingChaoZuZhi2, Unigene70493\_JingChaoZuZhi2, Unigene70723\_JingChaoZuZhi2, Unigene71365\_JingChaoZuZhi2, Unigene71558\_JingChaoZuZhi2, Unigene71693\_JingChaoZuZhi2, Unigene71958\_JingChaoZuZhi2, Unigene72098\_JingChaoZuZhi2, Unigene72269\_JingChaoZuZhi2, Unigene72643\_JingChaoZuZhi2, Unigene72802\_JingChaoZuZhi2, Unigene72826\_JingChaoZuZhi2, Unigene72978\_JingChaoZuZhi2, Unigene73632\_JingChaoZuZhi2, Unigene73896\_JingChaoZuZhi2, Unigene7456\_JingChaoZuZhi2, Unigene74594\_JingChaoZuZhi2, Unigene75235\_JingChaoZuZhi2, Unigene76182\_JingChaoZuZhi2, Unigene76756\_JingChaoZuZhi2, Unigene77149\_JingChaoZuZhi2, Unigene77255\_JingChaoZuZhi2, Unigene77558\_JingChaoZuZhi2, Unigene78078\_JingChaoZuZhi2, Unigene78398\_JingChaoZuZhi2, Unigene78490\_JingChaoZuZhi2, Unigene7859\_JingChaoZuZhi2, Unigene78950\_JingChaoZuZhi2, Unigene80275\_JingChaoZuZhi2, Unigene80451\_JingChaoZuZhi2, Unigene80494\_JingChaoZuZhi2, Unigene81101\_JingChaoZuZhi2, Unigene81183\_JingChaoZuZhi2, Unigene81317\_JingChaoZuZhi2, Unigene81395\_JingChaoZuZhi2, Unigene81692\_JingChaoZuZhi2, Unigene8169\_JingChaoZuZhi2, Unigene82875\_JingChaoZuZhi2, Unigene83526\_JingChaoZuZhi2, Unigene83602\_JingChaoZuZhi2, Unigene83832\_JingChaoZuZhi2, Unigene84161\_JingChaoZuZhi2, Unigene84190\_JingChaoZuZhi2, Unigene84232\_JingChaoZuZhi2, Unigene84247\_JingChaoZuZhi2, Unigene8598\_JingChaoZuZhi2, Unigene87332\_JingChaoZuZhi2, Unigene87470\_JingChaoZuZhi2, Unigene87607\_JingChaoZuZhi2, Unigene87738\_JingChaoZuZhi2, Unigene88068\_JingChaoZuZhi2, Unigene88319\_JingChaoZuZhi2, Unigene88385\_JingChaoZuZhi2, Unigene88773\_JingChaoZuZhi2, Unigene89120\_JingChaoZuZhi2, Unigene89147\_JingChaoZuZhi2, Unigene89175\_JingChaoZuZhi2, Unigene89309\_JingChaoZuZhi2, Unigene89603\_JingChaoZuZhi2, Unigene89807\_JingChaoZuZhi2, Unigene90179\_JingChaoZuZhi2, Unigene90190\_JingChaoZuZhi2, Unigene90749\_JingChaoZuZhi2, Unigene90865\_JingChaoZuZhi2, Unigene91179\_JingChaoZuZhi2, Unigene91344\_JingChaoZuZhi2, Unigene91739\_JingChaoZuZhi2, Unigene92119\_JingChaoZuZhi2, Unigene92378\_JingChaoZuZhi2, Unigene92737\_JingChaoZuZhi2, Unigene92751\_JingChaoZuZhi2, Unigene93012\_JingChaoZuZhi2, Unigene93693\_JingChaoZuZhi2, Unigene93949\_JingChaoZuZhi2, Unigene9415\_JingChaoZuZhi2, Unigene94279\_JingChaoZuZhi2, Unigene9446\_JingChaoZuZhi2, Unigene94828\_JingChaoZuZhi2, Unigene95063\_JingChaoZuZhi2, Unigene95205\_JingChaoZuZhi2, Unigene95394\_JingChaoZuZhi2, Unigene95677\_JingChaoZuZhi2, Unigene95729\_JingChaoZuZhi2, Unigene95776\_JingChaoZuZhi2, Unigene95875\_JingChaoZuZhi2, Unigene95881\_JingChaoZuZhi2, Unigene95914\_JingChaoZuZhi2, Unigene95916\_JingChaoZuZhi2, Unigene96045\_JingChaoZuZhi2, Unigene96206\_JingChaoZuZhi2, Unigene96262\_JingChaoZuZhi2, Unigene9654\_JingChaoZuZhi2, Unigene9\_JingChaoZuZhi2 |
| 48 | Peroxisome | Unigene10165\_JingChaoZuZhi2, Unigene10779\_JingChaoZuZhi2, Unigene10977\_JingChaoZuZhi2, Unigene11260\_JingChaoZuZhi2, Unigene11285\_JingChaoZuZhi2, Unigene11308\_JingChaoZuZhi2, Unigene12190\_JingChaoZuZhi2, Unigene12672\_JingChaoZuZhi2, Unigene12675\_JingChaoZuZhi2, Unigene12758\_JingChaoZuZhi2, Unigene13176\_JingChaoZuZhi2, Unigene13307\_JingChaoZuZhi2, Unigene13436\_JingChaoZuZhi2, Unigene13931\_JingChaoZuZhi2, Unigene14389\_JingChaoZuZhi2, Unigene14456\_JingChaoZuZhi2, Unigene14505\_JingChaoZuZhi2, Unigene15617\_JingChaoZuZhi2, Unigene15648\_JingChaoZuZhi2, Unigene15681\_JingChaoZuZhi2, Unigene15886\_JingChaoZuZhi2, Unigene16656\_JingChaoZuZhi2, Unigene16663\_JingChaoZuZhi2, Unigene16792\_JingChaoZuZhi2, Unigene16892\_JingChaoZuZhi2, Unigene16899\_JingChaoZuZhi2, Unigene17330\_JingChaoZuZhi2, Unigene17461\_JingChaoZuZhi2, Unigene17884\_JingChaoZuZhi2, Unigene18152\_JingChaoZuZhi2, Unigene18187\_JingChaoZuZhi2, Unigene18351\_JingChaoZuZhi2, Unigene18470\_JingChaoZuZhi2, Unigene18846\_JingChaoZuZhi2, Unigene18851\_JingChaoZuZhi2, Unigene19050\_JingChaoZuZhi2, Unigene19643\_JingChaoZuZhi2, Unigene19778\_JingChaoZuZhi2, Unigene19858\_JingChaoZuZhi2, Unigene20058\_JingChaoZuZhi2, Unigene20176\_JingChaoZuZhi2, Unigene20501\_JingChaoZuZhi2, Unigene2054\_JingChaoZuZhi2, Unigene20555\_JingChaoZuZhi2, Unigene20706\_JingChaoZuZhi2, Unigene21340\_JingChaoZuZhi2, Unigene21806\_JingChaoZuZhi2, Unigene2186\_JingChaoZuZhi2, Unigene22072\_JingChaoZuZhi2, Unigene22352\_JingChaoZuZhi2, Unigene22603\_JingChaoZuZhi2, Unigene22775\_JingChaoZuZhi2, Unigene22778\_JingChaoZuZhi2, Unigene22830\_JingChaoZuZhi2, Unigene22833\_JingChaoZuZhi2, Unigene23634\_JingChaoZuZhi2, Unigene26104\_JingChaoZuZhi2, Unigene2761\_JingChaoZuZhi2, Unigene30651\_JingChaoZuZhi2, Unigene30662\_JingChaoZuZhi2, Unigene3068\_JingChaoZuZhi2, Unigene31040\_JingChaoZuZhi2, Unigene31678\_JingChaoZuZhi2, Unigene3207\_JingChaoZuZhi2, Unigene33784\_JingChaoZuZhi2, Unigene35187\_JingChaoZuZhi2, Unigene35436\_JingChaoZuZhi2, Unigene36227\_JingChaoZuZhi2, Unigene36522\_JingChaoZuZhi2, Unigene37477\_JingChaoZuZhi2, Unigene388\_JingChaoZuZhi2, Unigene39406\_JingChaoZuZhi2, Unigene39876\_JingChaoZuZhi2, Unigene4132\_JingChaoZuZhi2, Unigene42298\_JingChaoZuZhi2, Unigene4231\_JingChaoZuZhi2, Unigene44828\_JingChaoZuZhi2, Unigene45176\_JingChaoZuZhi2, Unigene45324\_JingChaoZuZhi2, Unigene47407\_JingChaoZuZhi2, Unigene48019\_JingChaoZuZhi2, Unigene48335\_JingChaoZuZhi2, Unigene48393\_JingChaoZuZhi2, Unigene49819\_JingChaoZuZhi2, Unigene51682\_JingChaoZuZhi2, Unigene51765\_JingChaoZuZhi2, Unigene52317\_JingChaoZuZhi2, Unigene52773\_JingChaoZuZhi2, Unigene54127\_JingChaoZuZhi2, Unigene54350\_JingChaoZuZhi2, Unigene5449\_JingChaoZuZhi2, Unigene54532\_JingChaoZuZhi2, Unigene54768\_JingChaoZuZhi2, Unigene56662\_JingChaoZuZhi2, Unigene57105\_JingChaoZuZhi2, Unigene57750\_JingChaoZuZhi2, Unigene57866\_JingChaoZuZhi2, Unigene58530\_JingChaoZuZhi2, Unigene59548\_JingChaoZuZhi2, Unigene6109\_JingChaoZuZhi2, Unigene61162\_JingChaoZuZhi2, Unigene6247\_JingChaoZuZhi2, Unigene626\_JingChaoZuZhi2, Unigene62962\_JingChaoZuZhi2, Unigene63375\_JingChaoZuZhi2, Unigene63485\_JingChaoZuZhi2, Unigene64092\_JingChaoZuZhi2, Unigene64673\_JingChaoZuZhi2, Unigene65441\_JingChaoZuZhi2, Unigene6555\_JingChaoZuZhi2, Unigene66174\_JingChaoZuZhi2, Unigene66416\_JingChaoZuZhi2, Unigene66433\_JingChaoZuZhi2, Unigene6684\_JingChaoZuZhi2, Unigene67419\_JingChaoZuZhi2, Unigene6783\_JingChaoZuZhi2, Unigene68509\_JingChaoZuZhi2, Unigene7053\_JingChaoZuZhi2, Unigene70703\_JingChaoZuZhi2, Unigene7146\_JingChaoZuZhi2, Unigene71936\_JingChaoZuZhi2, Unigene7206\_JingChaoZuZhi2, Unigene72264\_JingChaoZuZhi2, Unigene72886\_JingChaoZuZhi2, Unigene72947\_JingChaoZuZhi2, Unigene7305\_JingChaoZuZhi2, Unigene73451\_JingChaoZuZhi2, Unigene73472\_JingChaoZuZhi2, Unigene74590\_JingChaoZuZhi2, Unigene75010\_JingChaoZuZhi2, Unigene75174\_JingChaoZuZhi2, Unigene75743\_JingChaoZuZhi2, Unigene75770\_JingChaoZuZhi2, Unigene76294\_JingChaoZuZhi2, Unigene77103\_JingChaoZuZhi2, Unigene78083\_JingChaoZuZhi2, Unigene78854\_JingChaoZuZhi2, Unigene79066\_JingChaoZuZhi2, Unigene79135\_JingChaoZuZhi2, Unigene79907\_JingChaoZuZhi2, Unigene80446\_JingChaoZuZhi2, Unigene80629\_JingChaoZuZhi2, Unigene8075\_JingChaoZuZhi2, Unigene80798\_JingChaoZuZhi2, Unigene81114\_JingChaoZuZhi2, Unigene81214\_JingChaoZuZhi2, Unigene81708\_JingChaoZuZhi2, Unigene81774\_JingChaoZuZhi2, Unigene82095\_JingChaoZuZhi2, Unigene8213\_JingChaoZuZhi2, Unigene82433\_JingChaoZuZhi2, Unigene82435\_JingChaoZuZhi2, Unigene83277\_JingChaoZuZhi2, Unigene83411\_JingChaoZuZhi2, Unigene84077\_JingChaoZuZhi2, Unigene84124\_JingChaoZuZhi2, Unigene84420\_JingChaoZuZhi2, Unigene84590\_JingChaoZuZhi2, Unigene85356\_JingChaoZuZhi2, Unigene85989\_JingChaoZuZhi2, Unigene86047\_JingChaoZuZhi2, Unigene86141\_JingChaoZuZhi2, Unigene86415\_JingChaoZuZhi2, Unigene86715\_JingChaoZuZhi2, Unigene87018\_JingChaoZuZhi2, Unigene87475\_JingChaoZuZhi2, Unigene87833\_JingChaoZuZhi2, Unigene88045\_JingChaoZuZhi2, Unigene88377\_JingChaoZuZhi2, Unigene89115\_JingChaoZuZhi2, Unigene89233\_JingChaoZuZhi2, Unigene89280\_JingChaoZuZhi2, Unigene89311\_JingChaoZuZhi2, Unigene89461\_JingChaoZuZhi2, Unigene90034\_JingChaoZuZhi2, Unigene90758\_JingChaoZuZhi2, Unigene90859\_JingChaoZuZhi2, Unigene9097\_JingChaoZuZhi2, Unigene91059\_JingChaoZuZhi2, Unigene91702\_JingChaoZuZhi2, Unigene92294\_JingChaoZuZhi2, Unigene92376\_JingChaoZuZhi2, Unigene92681\_JingChaoZuZhi2, Unigene92702\_JingChaoZuZhi2, Unigene93033\_JingChaoZuZhi2, Unigene93101\_JingChaoZuZhi2, Unigene93275\_JingChaoZuZhi2, Unigene93537\_JingChaoZuZhi2, Unigene93598\_JingChaoZuZhi2, Unigene93635\_JingChaoZuZhi2, Unigene93924\_JingChaoZuZhi2, Unigene93972\_JingChaoZuZhi2, Unigene94009\_JingChaoZuZhi2, Unigene94201\_JingChaoZuZhi2, Unigene942\_JingChaoZuZhi2, Unigene94726\_JingChaoZuZhi2, Unigene9484\_JingChaoZuZhi2, Unigene95141\_JingChaoZuZhi2, Unigene95191\_JingChaoZuZhi2, Unigene95326\_JingChaoZuZhi2, Unigene95397\_JingChaoZuZhi2, Unigene95535\_JingChaoZuZhi2, Unigene95653\_JingChaoZuZhi2, Unigene96033\_JingChaoZuZhi2, Unigene96266\_JingChaoZuZhi2, Unigene98\_JingChaoZuZhi2 |
| 49 | RNA degradation | Unigene10022\_JingChaoZuZhi2, Unigene11148\_JingChaoZuZhi2, Unigene11468\_JingChaoZuZhi2, Unigene11720\_JingChaoZuZhi2, Unigene11921\_JingChaoZuZhi2, Unigene12166\_JingChaoZuZhi2, Unigene12611\_JingChaoZuZhi2, Unigene13210\_JingChaoZuZhi2, Unigene13906\_JingChaoZuZhi2, Unigene14160\_JingChaoZuZhi2, Unigene14656\_JingChaoZuZhi2, Unigene15223\_JingChaoZuZhi2, Unigene15834\_JingChaoZuZhi2, Unigene15908\_JingChaoZuZhi2, Unigene16118\_JingChaoZuZhi2, Unigene16362\_JingChaoZuZhi2, Unigene16377\_JingChaoZuZhi2, Unigene16610\_JingChaoZuZhi2, Unigene16703\_JingChaoZuZhi2, Unigene1675\_JingChaoZuZhi2, Unigene16875\_JingChaoZuZhi2, Unigene17430\_JingChaoZuZhi2, Unigene18103\_JingChaoZuZhi2, Unigene18160\_JingChaoZuZhi2, Unigene18286\_JingChaoZuZhi2, Unigene18395\_JingChaoZuZhi2, Unigene18434\_JingChaoZuZhi2, Unigene18492\_JingChaoZuZhi2, Unigene18522\_JingChaoZuZhi2, Unigene18573\_JingChaoZuZhi2, Unigene18636\_JingChaoZuZhi2, Unigene19117\_JingChaoZuZhi2, Unigene19190\_JingChaoZuZhi2, Unigene19431\_JingChaoZuZhi2, Unigene19438\_JingChaoZuZhi2, Unigene19598\_JingChaoZuZhi2, Unigene19829\_JingChaoZuZhi2, Unigene19996\_JingChaoZuZhi2, Unigene2013\_JingChaoZuZhi2, Unigene20246\_JingChaoZuZhi2, Unigene20344\_JingChaoZuZhi2, Unigene20554\_JingChaoZuZhi2, Unigene2085\_JingChaoZuZhi2, Unigene20955\_JingChaoZuZhi2, Unigene20960\_JingChaoZuZhi2, Unigene21536\_JingChaoZuZhi2, Unigene21550\_JingChaoZuZhi2, Unigene21700\_JingChaoZuZhi2, Unigene21930\_JingChaoZuZhi2, Unigene22246\_JingChaoZuZhi2, Unigene22312\_JingChaoZuZhi2, Unigene22388\_JingChaoZuZhi2, Unigene22395\_JingChaoZuZhi2, Unigene22412\_JingChaoZuZhi2, Unigene22422\_JingChaoZuZhi2, Unigene22695\_JingChaoZuZhi2, Unigene22752\_JingChaoZuZhi2, Unigene22845\_JingChaoZuZhi2, Unigene2387\_JingChaoZuZhi2, Unigene24093\_JingChaoZuZhi2, Unigene270\_JingChaoZuZhi2, Unigene29355\_JingChaoZuZhi2, Unigene3232\_JingChaoZuZhi2, Unigene33915\_JingChaoZuZhi2, Unigene3414\_JingChaoZuZhi2, Unigene37093\_JingChaoZuZhi2, Unigene3867\_JingChaoZuZhi2, Unigene43120\_JingChaoZuZhi2, Unigene43158\_JingChaoZuZhi2, Unigene4366\_JingChaoZuZhi2, Unigene4401\_JingChaoZuZhi2, Unigene4449\_JingChaoZuZhi2, Unigene44638\_JingChaoZuZhi2, Unigene4578\_JingChaoZuZhi2, Unigene473\_JingChaoZuZhi2, Unigene4749\_JingChaoZuZhi2, Unigene48772\_JingChaoZuZhi2, Unigene495\_JingChaoZuZhi2, Unigene4978\_JingChaoZuZhi2, Unigene5029\_JingChaoZuZhi2, Unigene5064\_JingChaoZuZhi2, Unigene5104\_JingChaoZuZhi2, Unigene5263\_JingChaoZuZhi2, Unigene52746\_JingChaoZuZhi2, Unigene5288\_JingChaoZuZhi2, Unigene5317\_JingChaoZuZhi2, Unigene56005\_JingChaoZuZhi2, Unigene58704\_JingChaoZuZhi2, Unigene59414\_JingChaoZuZhi2, Unigene59990\_JingChaoZuZhi2, Unigene6086\_JingChaoZuZhi2, Unigene63365\_JingChaoZuZhi2, Unigene64521\_JingChaoZuZhi2, Unigene66160\_JingChaoZuZhi2, Unigene67077\_JingChaoZuZhi2, Unigene6768\_JingChaoZuZhi2, Unigene68024\_JingChaoZuZhi2, Unigene6828\_JingChaoZuZhi2, Unigene68615\_JingChaoZuZhi2, Unigene68777\_JingChaoZuZhi2, Unigene70201\_JingChaoZuZhi2, Unigene71791\_JingChaoZuZhi2, Unigene71838\_JingChaoZuZhi2, Unigene72697\_JingChaoZuZhi2, Unigene74056\_JingChaoZuZhi2, Unigene74063\_JingChaoZuZhi2, Unigene74512\_JingChaoZuZhi2, Unigene74758\_JingChaoZuZhi2, Unigene74892\_JingChaoZuZhi2, Unigene76056\_JingChaoZuZhi2, Unigene76747\_JingChaoZuZhi2, Unigene76793\_JingChaoZuZhi2, Unigene7685\_JingChaoZuZhi2, Unigene76957\_JingChaoZuZhi2, Unigene77205\_JingChaoZuZhi2, Unigene77982\_JingChaoZuZhi2, Unigene78136\_JingChaoZuZhi2, Unigene78522\_JingChaoZuZhi2, Unigene78549\_JingChaoZuZhi2, Unigene78815\_JingChaoZuZhi2, Unigene7977\_JingChaoZuZhi2, Unigene79831\_JingChaoZuZhi2, Unigene79985\_JingChaoZuZhi2, Unigene80203\_JingChaoZuZhi2, Unigene80221\_JingChaoZuZhi2, Unigene80333\_JingChaoZuZhi2, Unigene8089\_JingChaoZuZhi2, Unigene81261\_JingChaoZuZhi2, Unigene81366\_JingChaoZuZhi2, Unigene81512\_JingChaoZuZhi2, Unigene81651\_JingChaoZuZhi2, Unigene82075\_JingChaoZuZhi2, Unigene8207\_JingChaoZuZhi2, Unigene82219\_JingChaoZuZhi2, Unigene82351\_JingChaoZuZhi2, Unigene82591\_JingChaoZuZhi2, Unigene82615\_JingChaoZuZhi2, Unigene83176\_JingChaoZuZhi2, Unigene83221\_JingChaoZuZhi2, Unigene83777\_JingChaoZuZhi2, Unigene83910\_JingChaoZuZhi2, Unigene84085\_JingChaoZuZhi2, Unigene8449\_JingChaoZuZhi2, Unigene85294\_JingChaoZuZhi2, Unigene85496\_JingChaoZuZhi2, Unigene85755\_JingChaoZuZhi2, Unigene85983\_JingChaoZuZhi2, Unigene86908\_JingChaoZuZhi2, Unigene87037\_JingChaoZuZhi2, Unigene87089\_JingChaoZuZhi2, Unigene87238\_JingChaoZuZhi2, Unigene87275\_JingChaoZuZhi2, Unigene87365\_JingChaoZuZhi2, Unigene87825\_JingChaoZuZhi2, Unigene88021\_JingChaoZuZhi2, Unigene88370\_JingChaoZuZhi2, Unigene88725\_JingChaoZuZhi2, Unigene88897\_JingChaoZuZhi2, Unigene89021\_JingChaoZuZhi2, Unigene89037\_JingChaoZuZhi2, Unigene89247\_JingChaoZuZhi2, Unigene89282\_JingChaoZuZhi2, Unigene89593\_JingChaoZuZhi2, Unigene89688\_JingChaoZuZhi2, Unigene89788\_JingChaoZuZhi2, Unigene90244\_JingChaoZuZhi2, Unigene90780\_JingChaoZuZhi2, Unigene91037\_JingChaoZuZhi2, Unigene91296\_JingChaoZuZhi2, Unigene91333\_JingChaoZuZhi2, Unigene91703\_JingChaoZuZhi2, Unigene92283\_JingChaoZuZhi2, Unigene92304\_JingChaoZuZhi2, Unigene92410\_JingChaoZuZhi2, Unigene92427\_JingChaoZuZhi2, Unigene92865\_JingChaoZuZhi2, Unigene92975\_JingChaoZuZhi2, Unigene93662\_JingChaoZuZhi2, Unigene93728\_JingChaoZuZhi2, Unigene93802\_JingChaoZuZhi2, Unigene94065\_JingChaoZuZhi2, Unigene94121\_JingChaoZuZhi2, Unigene94172\_JingChaoZuZhi2, Unigene94553\_JingChaoZuZhi2, Unigene94632\_JingChaoZuZhi2, Unigene94790\_JingChaoZuZhi2, Unigene94801\_JingChaoZuZhi2, Unigene95042\_JingChaoZuZhi2, Unigene95183\_JingChaoZuZhi2, Unigene95402\_JingChaoZuZhi2, Unigene95411\_JingChaoZuZhi2, Unigene95445\_JingChaoZuZhi2, Unigene95457\_JingChaoZuZhi2, Unigene95462\_JingChaoZuZhi2, Unigene95786\_JingChaoZuZhi2, Unigene95968\_JingChaoZuZhi2, Unigene96187\_JingChaoZuZhi2, Unigene96260\_JingChaoZuZhi2, Unigene9695\_JingChaoZuZhi2, Unigene9697\_JingChaoZuZhi2, Unigene9791\_JingChaoZuZhi2, Unigene9982\_JingChaoZuZhi2 |
| 50 | Neurotrophin signaling pathway | Unigene10016\_JingChaoZuZhi2, Unigene10218\_JingChaoZuZhi2, Unigene10339\_JingChaoZuZhi2, Unigene10926\_JingChaoZuZhi2, Unigene11078\_JingChaoZuZhi2, Unigene11951\_JingChaoZuZhi2, Unigene11961\_JingChaoZuZhi2, Unigene12257\_JingChaoZuZhi2, Unigene12420\_JingChaoZuZhi2, Unigene12813\_JingChaoZuZhi2, Unigene12899\_JingChaoZuZhi2, Unigene12912\_JingChaoZuZhi2, Unigene13510\_JingChaoZuZhi2, Unigene13823\_JingChaoZuZhi2, Unigene13991\_JingChaoZuZhi2, Unigene14738\_JingChaoZuZhi2, Unigene14842\_JingChaoZuZhi2, Unigene14912\_JingChaoZuZhi2, Unigene15228\_JingChaoZuZhi2, Unigene16566\_JingChaoZuZhi2, Unigene16768\_JingChaoZuZhi2, Unigene16820\_JingChaoZuZhi2, Unigene17178\_JingChaoZuZhi2, Unigene17247\_JingChaoZuZhi2, Unigene17666\_JingChaoZuZhi2, Unigene17839\_JingChaoZuZhi2, Unigene17888\_JingChaoZuZhi2, Unigene17946\_JingChaoZuZhi2, Unigene18464\_JingChaoZuZhi2, Unigene18672\_JingChaoZuZhi2, Unigene18715\_JingChaoZuZhi2, Unigene18821\_JingChaoZuZhi2, Unigene18868\_JingChaoZuZhi2, Unigene19021\_JingChaoZuZhi2, Unigene19046\_JingChaoZuZhi2, Unigene19086\_JingChaoZuZhi2, Unigene19624\_JingChaoZuZhi2, Unigene20120\_JingChaoZuZhi2, Unigene20157\_JingChaoZuZhi2, Unigene20977\_JingChaoZuZhi2, Unigene20991\_JingChaoZuZhi2, Unigene21295\_JingChaoZuZhi2, Unigene21513\_JingChaoZuZhi2, Unigene21515\_JingChaoZuZhi2, Unigene21578\_JingChaoZuZhi2, Unigene21697\_JingChaoZuZhi2, Unigene21862\_JingChaoZuZhi2, Unigene22509\_JingChaoZuZhi2, Unigene2456\_JingChaoZuZhi2, Unigene25870\_JingChaoZuZhi2, Unigene2687\_JingChaoZuZhi2, Unigene32272\_JingChaoZuZhi2, Unigene37034\_JingChaoZuZhi2, Unigene3918\_JingChaoZuZhi2, Unigene3931\_JingChaoZuZhi2, Unigene39577\_JingChaoZuZhi2, Unigene4186\_JingChaoZuZhi2, Unigene42146\_JingChaoZuZhi2, Unigene4223\_JingChaoZuZhi2, Unigene4294\_JingChaoZuZhi2, Unigene45695\_JingChaoZuZhi2, Unigene4592\_JingChaoZuZhi2, Unigene46219\_JingChaoZuZhi2, Unigene47155\_JingChaoZuZhi2, Unigene473\_JingChaoZuZhi2, Unigene4838\_JingChaoZuZhi2, Unigene48751\_JingChaoZuZhi2, Unigene49913\_JingChaoZuZhi2, Unigene5099\_JingChaoZuZhi2, Unigene51164\_JingChaoZuZhi2, Unigene51705\_JingChaoZuZhi2, Unigene51992\_JingChaoZuZhi2, Unigene5253\_JingChaoZuZhi2, Unigene52542\_JingChaoZuZhi2, Unigene52554\_JingChaoZuZhi2, Unigene54320\_JingChaoZuZhi2, Unigene55687\_JingChaoZuZhi2, Unigene5638\_JingChaoZuZhi2, Unigene5674\_JingChaoZuZhi2, Unigene57488\_JingChaoZuZhi2, Unigene5823\_JingChaoZuZhi2, Unigene58550\_JingChaoZuZhi2, Unigene59012\_JingChaoZuZhi2, Unigene59471\_JingChaoZuZhi2, Unigene59885\_JingChaoZuZhi2, Unigene60045\_JingChaoZuZhi2, Unigene6028\_JingChaoZuZhi2, Unigene61044\_JingChaoZuZhi2, Unigene62846\_JingChaoZuZhi2, Unigene63226\_JingChaoZuZhi2, Unigene6459\_JingChaoZuZhi2, Unigene6493\_JingChaoZuZhi2, Unigene65294\_JingChaoZuZhi2, Unigene66813\_JingChaoZuZhi2, Unigene67655\_JingChaoZuZhi2, Unigene67793\_JingChaoZuZhi2, Unigene68043\_JingChaoZuZhi2, Unigene68108\_JingChaoZuZhi2, Unigene68200\_JingChaoZuZhi2, Unigene70063\_JingChaoZuZhi2, Unigene70505\_JingChaoZuZhi2, Unigene70549\_JingChaoZuZhi2, Unigene72632\_JingChaoZuZhi2, Unigene7267\_JingChaoZuZhi2, Unigene72803\_JingChaoZuZhi2, Unigene73733\_JingChaoZuZhi2, Unigene76023\_JingChaoZuZhi2, Unigene77471\_JingChaoZuZhi2, Unigene77570\_JingChaoZuZhi2, Unigene78400\_JingChaoZuZhi2, Unigene7877\_JingChaoZuZhi2, Unigene78957\_JingChaoZuZhi2, Unigene79233\_JingChaoZuZhi2, Unigene79882\_JingChaoZuZhi2, Unigene80688\_JingChaoZuZhi2, Unigene81154\_JingChaoZuZhi2, Unigene81501\_JingChaoZuZhi2, Unigene8191\_JingChaoZuZhi2, Unigene81948\_JingChaoZuZhi2, Unigene8202\_JingChaoZuZhi2, Unigene82073\_JingChaoZuZhi2, Unigene8248\_JingChaoZuZhi2, Unigene83255\_JingChaoZuZhi2, Unigene83561\_JingChaoZuZhi2, Unigene83700\_JingChaoZuZhi2, Unigene84340\_JingChaoZuZhi2, Unigene8434\_JingChaoZuZhi2, Unigene8464\_JingChaoZuZhi2, Unigene84862\_JingChaoZuZhi2, Unigene84930\_JingChaoZuZhi2, Unigene8507\_JingChaoZuZhi2, Unigene85574\_JingChaoZuZhi2, Unigene85615\_JingChaoZuZhi2, Unigene86036\_JingChaoZuZhi2, Unigene86206\_JingChaoZuZhi2, Unigene86526\_JingChaoZuZhi2, Unigene86899\_JingChaoZuZhi2, Unigene87305\_JingChaoZuZhi2, Unigene876\_JingChaoZuZhi2, Unigene87730\_JingChaoZuZhi2, Unigene88594\_JingChaoZuZhi2, Unigene88719\_JingChaoZuZhi2, Unigene88724\_JingChaoZuZhi2, Unigene88752\_JingChaoZuZhi2, Unigene88942\_JingChaoZuZhi2, Unigene89310\_JingChaoZuZhi2, Unigene89379\_JingChaoZuZhi2, Unigene90209\_JingChaoZuZhi2, Unigene90508\_JingChaoZuZhi2, Unigene90879\_JingChaoZuZhi2, Unigene91179\_JingChaoZuZhi2, Unigene91379\_JingChaoZuZhi2, Unigene91612\_JingChaoZuZhi2, Unigene91631\_JingChaoZuZhi2, Unigene91661\_JingChaoZuZhi2, Unigene91825\_JingChaoZuZhi2, Unigene91924\_JingChaoZuZhi2, Unigene91925\_JingChaoZuZhi2, Unigene91958\_JingChaoZuZhi2, Unigene92112\_JingChaoZuZhi2, Unigene92119\_JingChaoZuZhi2, Unigene92580\_JingChaoZuZhi2, Unigene93284\_JingChaoZuZhi2, Unigene93357\_JingChaoZuZhi2, Unigene93588\_JingChaoZuZhi2, Unigene93597\_JingChaoZuZhi2, Unigene93714\_JingChaoZuZhi2, Unigene93782\_JingChaoZuZhi2, Unigene93807\_JingChaoZuZhi2, Unigene9389\_JingChaoZuZhi2, Unigene94047\_JingChaoZuZhi2, Unigene94138\_JingChaoZuZhi2, Unigene94149\_JingChaoZuZhi2, Unigene94208\_JingChaoZuZhi2, Unigene9444\_JingChaoZuZhi2, Unigene94456\_JingChaoZuZhi2, Unigene94544\_JingChaoZuZhi2, Unigene94619\_JingChaoZuZhi2, Unigene9461\_JingChaoZuZhi2, Unigene94680\_JingChaoZuZhi2, Unigene94685\_JingChaoZuZhi2, Unigene9488\_JingChaoZuZhi2, Unigene94903\_JingChaoZuZhi2, Unigene95044\_JingChaoZuZhi2, Unigene95098\_JingChaoZuZhi2, Unigene95295\_JingChaoZuZhi2, Unigene95456\_JingChaoZuZhi2, Unigene95665\_JingChaoZuZhi2, Unigene95700\_JingChaoZuZhi2, Unigene95875\_JingChaoZuZhi2, Unigene95881\_JingChaoZuZhi2, Unigene96294\_JingChaoZuZhi2, Unigene9703\_JingChaoZuZhi2, Unigene9711\_JingChaoZuZhi2, Unigene9767\_JingChaoZuZhi2, Unigene9902\_JingChaoZuZhi2 |
| 51 | Phosphatidylinositol signaling system | Unigene10122\_JingChaoZuZhi2, Unigene10318\_JingChaoZuZhi2, Unigene10443\_JingChaoZuZhi2, Unigene10782\_JingChaoZuZhi2, Unigene10806\_JingChaoZuZhi2, Unigene10860\_JingChaoZuZhi2, Unigene11074\_JingChaoZuZhi2, Unigene11961\_JingChaoZuZhi2, Unigene11995\_JingChaoZuZhi2, Unigene12073\_JingChaoZuZhi2, Unigene12700\_JingChaoZuZhi2, Unigene12979\_JingChaoZuZhi2, Unigene13149\_JingChaoZuZhi2, Unigene13176\_JingChaoZuZhi2, Unigene13429\_JingChaoZuZhi2, Unigene13452\_JingChaoZuZhi2, Unigene13972\_JingChaoZuZhi2, Unigene14068\_JingChaoZuZhi2, Unigene14360\_JingChaoZuZhi2, Unigene14912\_JingChaoZuZhi2, Unigene14977\_JingChaoZuZhi2, Unigene15228\_JingChaoZuZhi2, Unigene16149\_JingChaoZuZhi2, Unigene16293\_JingChaoZuZhi2, Unigene16566\_JingChaoZuZhi2, Unigene16594\_JingChaoZuZhi2, Unigene16646\_JingChaoZuZhi2, Unigene16991\_JingChaoZuZhi2, Unigene17247\_JingChaoZuZhi2, Unigene18176\_JingChaoZuZhi2, Unigene18464\_JingChaoZuZhi2, Unigene18672\_JingChaoZuZhi2, Unigene18721\_JingChaoZuZhi2, Unigene1878\_JingChaoZuZhi2, Unigene18868\_JingChaoZuZhi2, Unigene19186\_JingChaoZuZhi2, Unigene19458\_JingChaoZuZhi2, Unigene19565\_JingChaoZuZhi2, Unigene19862\_JingChaoZuZhi2, Unigene20857\_JingChaoZuZhi2, Unigene20959\_JingChaoZuZhi2, Unigene21020\_JingChaoZuZhi2, Unigene21295\_JingChaoZuZhi2, Unigene21578\_JingChaoZuZhi2, Unigene22211\_JingChaoZuZhi2, Unigene22309\_JingChaoZuZhi2, Unigene22387\_JingChaoZuZhi2, Unigene22579\_JingChaoZuZhi2, Unigene22779\_JingChaoZuZhi2, Unigene2370\_JingChaoZuZhi2, Unigene2456\_JingChaoZuZhi2, Unigene25108\_JingChaoZuZhi2, Unigene2514\_JingChaoZuZhi2, Unigene26730\_JingChaoZuZhi2, Unigene26869\_JingChaoZuZhi2, Unigene2960\_JingChaoZuZhi2, Unigene30799\_JingChaoZuZhi2, Unigene3116\_JingChaoZuZhi2, Unigene3457\_JingChaoZuZhi2, Unigene37471\_JingChaoZuZhi2, Unigene37689\_JingChaoZuZhi2, Unigene3807\_JingChaoZuZhi2, Unigene3850\_JingChaoZuZhi2, Unigene38514\_JingChaoZuZhi2, Unigene39577\_JingChaoZuZhi2, Unigene4050\_JingChaoZuZhi2, Unigene41333\_JingChaoZuZhi2, Unigene4160\_JingChaoZuZhi2, Unigene43322\_JingChaoZuZhi2, Unigene4377\_JingChaoZuZhi2, Unigene44152\_JingChaoZuZhi2, Unigene45669\_JingChaoZuZhi2, Unigene45682\_JingChaoZuZhi2, Unigene4713\_JingChaoZuZhi2, Unigene48751\_JingChaoZuZhi2, Unigene49963\_JingChaoZuZhi2, Unigene50173\_JingChaoZuZhi2, Unigene50908\_JingChaoZuZhi2, Unigene5113\_JingChaoZuZhi2, Unigene51678\_JingChaoZuZhi2, Unigene51895\_JingChaoZuZhi2, Unigene54367\_JingChaoZuZhi2, Unigene54449\_JingChaoZuZhi2, Unigene57488\_JingChaoZuZhi2, Unigene5782\_JingChaoZuZhi2, Unigene57848\_JingChaoZuZhi2, Unigene59493\_JingChaoZuZhi2, Unigene6009\_JingChaoZuZhi2, Unigene62246\_JingChaoZuZhi2, Unigene6250\_JingChaoZuZhi2, Unigene62887\_JingChaoZuZhi2, Unigene63807\_JingChaoZuZhi2, Unigene63982\_JingChaoZuZhi2, Unigene63994\_JingChaoZuZhi2, Unigene64164\_JingChaoZuZhi2, Unigene64393\_JingChaoZuZhi2, Unigene64489\_JingChaoZuZhi2, Unigene64615\_JingChaoZuZhi2, Unigene64830\_JingChaoZuZhi2, Unigene65445\_JingChaoZuZhi2, Unigene66540\_JingChaoZuZhi2, Unigene67655\_JingChaoZuZhi2, Unigene68024\_JingChaoZuZhi2, Unigene68159\_JingChaoZuZhi2, Unigene68845\_JingChaoZuZhi2, Unigene69249\_JingChaoZuZhi2, Unigene7010\_JingChaoZuZhi2, Unigene70723\_JingChaoZuZhi2, Unigene71246\_JingChaoZuZhi2, Unigene71418\_JingChaoZuZhi2, Unigene71939\_JingChaoZuZhi2, Unigene72466\_JingChaoZuZhi2, Unigene74599\_JingChaoZuZhi2, Unigene7493\_JingChaoZuZhi2, Unigene7508\_JingChaoZuZhi2, Unigene75749\_JingChaoZuZhi2, Unigene76000\_JingChaoZuZhi2, Unigene77149\_JingChaoZuZhi2, Unigene77344\_JingChaoZuZhi2, Unigene77528\_JingChaoZuZhi2, Unigene7799\_JingChaoZuZhi2, Unigene78078\_JingChaoZuZhi2, Unigene7817\_JingChaoZuZhi2, Unigene78189\_JingChaoZuZhi2, Unigene78992\_JingChaoZuZhi2, Unigene79239\_JingChaoZuZhi2, Unigene79268\_JingChaoZuZhi2, Unigene79895\_JingChaoZuZhi2, Unigene8011\_JingChaoZuZhi2, Unigene81951\_JingChaoZuZhi2, Unigene82061\_JingChaoZuZhi2, Unigene82389\_JingChaoZuZhi2, Unigene82414\_JingChaoZuZhi2, Unigene83218\_JingChaoZuZhi2, Unigene83268\_JingChaoZuZhi2, Unigene83570\_JingChaoZuZhi2, Unigene83898\_JingChaoZuZhi2, Unigene84076\_JingChaoZuZhi2, Unigene84222\_JingChaoZuZhi2, Unigene84247\_JingChaoZuZhi2, Unigene8455\_JingChaoZuZhi2, Unigene84839\_JingChaoZuZhi2, Unigene86007\_JingChaoZuZhi2, Unigene86034\_JingChaoZuZhi2, Unigene8606\_JingChaoZuZhi2, Unigene86206\_JingChaoZuZhi2, Unigene86526\_JingChaoZuZhi2, Unigene86545\_JingChaoZuZhi2, Unigene86995\_JingChaoZuZhi2, Unigene87020\_JingChaoZuZhi2, Unigene87332\_JingChaoZuZhi2, Unigene8737\_JingChaoZuZhi2, Unigene87478\_JingChaoZuZhi2, Unigene87524\_JingChaoZuZhi2, Unigene87607\_JingChaoZuZhi2, Unigene87893\_JingChaoZuZhi2, Unigene87960\_JingChaoZuZhi2, Unigene88090\_JingChaoZuZhi2, Unigene88385\_JingChaoZuZhi2, Unigene88594\_JingChaoZuZhi2, Unigene88724\_JingChaoZuZhi2, Unigene88748\_JingChaoZuZhi2, Unigene88773\_JingChaoZuZhi2, Unigene88801\_JingChaoZuZhi2, Unigene89147\_JingChaoZuZhi2, Unigene90131\_JingChaoZuZhi2, Unigene91039\_JingChaoZuZhi2, Unigene91687\_JingChaoZuZhi2, Unigene91739\_JingChaoZuZhi2, Unigene91751\_JingChaoZuZhi2, Unigene91924\_JingChaoZuZhi2, Unigene92112\_JingChaoZuZhi2, Unigene92263\_JingChaoZuZhi2, Unigene92395\_JingChaoZuZhi2, Unigene92978\_JingChaoZuZhi2, Unigene92989\_JingChaoZuZhi2, Unigene93588\_JingChaoZuZhi2, Unigene93714\_JingChaoZuZhi2, Unigene93731\_JingChaoZuZhi2, Unigene93807\_JingChaoZuZhi2, Unigene94406\_JingChaoZuZhi2, Unigene9461\_JingChaoZuZhi2, Unigene94647\_JingChaoZuZhi2, Unigene94685\_JingChaoZuZhi2, Unigene94777\_JingChaoZuZhi2, Unigene94898\_JingChaoZuZhi2, Unigene94962\_JingChaoZuZhi2, Unigene9522\_JingChaoZuZhi2, Unigene95622\_JingChaoZuZhi2, Unigene95677\_JingChaoZuZhi2, Unigene95791\_JingChaoZuZhi2, Unigene95992\_JingChaoZuZhi2, Unigene96076\_JingChaoZuZhi2, Unigene96294\_JingChaoZuZhi2 |
| 52 | T cell receptor signaling pathway | Unigene1001\_JingChaoZuZhi2, Unigene10053\_JingChaoZuZhi2, Unigene10290\_JingChaoZuZhi2, Unigene1109\_JingChaoZuZhi2, Unigene11287\_JingChaoZuZhi2, Unigene11732\_JingChaoZuZhi2, Unigene11951\_JingChaoZuZhi2, Unigene11961\_JingChaoZuZhi2, Unigene12171\_JingChaoZuZhi2, Unigene12214\_JingChaoZuZhi2, Unigene12420\_JingChaoZuZhi2, Unigene12899\_JingChaoZuZhi2, Unigene1301\_JingChaoZuZhi2, Unigene13180\_JingChaoZuZhi2, Unigene13927\_JingChaoZuZhi2, Unigene14317\_JingChaoZuZhi2, Unigene14370\_JingChaoZuZhi2, Unigene14488\_JingChaoZuZhi2, Unigene14635\_JingChaoZuZhi2, Unigene14912\_JingChaoZuZhi2, Unigene14973\_JingChaoZuZhi2, Unigene14990\_JingChaoZuZhi2, Unigene15095\_JingChaoZuZhi2, Unigene15104\_JingChaoZuZhi2, Unigene15228\_JingChaoZuZhi2, Unigene16039\_JingChaoZuZhi2, Unigene1609\_JingChaoZuZhi2, Unigene16116\_JingChaoZuZhi2, Unigene16154\_JingChaoZuZhi2, Unigene16521\_JingChaoZuZhi2, Unigene16589\_JingChaoZuZhi2, Unigene16820\_JingChaoZuZhi2, Unigene16933\_JingChaoZuZhi2, Unigene17136\_JingChaoZuZhi2, Unigene17168\_JingChaoZuZhi2, Unigene17247\_JingChaoZuZhi2, Unigene17710\_JingChaoZuZhi2, Unigene17764\_JingChaoZuZhi2, Unigene17829\_JingChaoZuZhi2, Unigene17888\_JingChaoZuZhi2, Unigene18464\_JingChaoZuZhi2, Unigene18672\_JingChaoZuZhi2, Unigene18715\_JingChaoZuZhi2, Unigene18868\_JingChaoZuZhi2, Unigene18904\_JingChaoZuZhi2, Unigene18986\_JingChaoZuZhi2, Unigene19036\_JingChaoZuZhi2, Unigene1942\_JingChaoZuZhi2, Unigene19531\_JingChaoZuZhi2, Unigene1993\_JingChaoZuZhi2, Unigene20107\_JingChaoZuZhi2, Unigene20120\_JingChaoZuZhi2, Unigene20157\_JingChaoZuZhi2, Unigene20256\_JingChaoZuZhi2, Unigene20649\_JingChaoZuZhi2, Unigene20991\_JingChaoZuZhi2, Unigene21045\_JingChaoZuZhi2, Unigene21144\_JingChaoZuZhi2, Unigene21246\_JingChaoZuZhi2, Unigene21295\_JingChaoZuZhi2, Unigene21348\_JingChaoZuZhi2, Unigene21578\_JingChaoZuZhi2, Unigene21605\_JingChaoZuZhi2, Unigene21697\_JingChaoZuZhi2, Unigene21737\_JingChaoZuZhi2, Unigene21849\_JingChaoZuZhi2, Unigene21973\_JingChaoZuZhi2, Unigene22102\_JingChaoZuZhi2, Unigene22174\_JingChaoZuZhi2, Unigene22386\_JingChaoZuZhi2, Unigene22466\_JingChaoZuZhi2, Unigene22482\_JingChaoZuZhi2, Unigene22500\_JingChaoZuZhi2, Unigene22648\_JingChaoZuZhi2, Unigene22737\_JingChaoZuZhi2, Unigene22804\_JingChaoZuZhi2, Unigene22934\_JingChaoZuZhi2, Unigene2456\_JingChaoZuZhi2, Unigene24841\_JingChaoZuZhi2, Unigene24881\_JingChaoZuZhi2, Unigene25966\_JingChaoZuZhi2, Unigene26183\_JingChaoZuZhi2, Unigene2628\_JingChaoZuZhi2, Unigene2687\_JingChaoZuZhi2, Unigene277\_JingChaoZuZhi2, Unigene3016\_JingChaoZuZhi2, Unigene3185\_JingChaoZuZhi2, Unigene35744\_JingChaoZuZhi2, Unigene38493\_JingChaoZuZhi2, Unigene3931\_JingChaoZuZhi2, Unigene39539\_JingChaoZuZhi2, Unigene40020\_JingChaoZuZhi2, Unigene40825\_JingChaoZuZhi2, Unigene4151\_JingChaoZuZhi2, Unigene4186\_JingChaoZuZhi2, Unigene42332\_JingChaoZuZhi2, Unigene4294\_JingChaoZuZhi2, Unigene45962\_JingChaoZuZhi2, Unigene45\_JingChaoZuZhi2, Unigene48368\_JingChaoZuZhi2, Unigene4838\_JingChaoZuZhi2, Unigene4853\_JingChaoZuZhi2, Unigene48607\_JingChaoZuZhi2, Unigene48751\_JingChaoZuZhi2, Unigene51285\_JingChaoZuZhi2, Unigene51966\_JingChaoZuZhi2, Unigene5196\_JingChaoZuZhi2, Unigene52554\_JingChaoZuZhi2, Unigene5314\_JingChaoZuZhi2, Unigene54320\_JingChaoZuZhi2, Unigene56642\_JingChaoZuZhi2, Unigene57488\_JingChaoZuZhi2, Unigene5823\_JingChaoZuZhi2, Unigene601\_JingChaoZuZhi2, Unigene6028\_JingChaoZuZhi2, Unigene61108\_JingChaoZuZhi2, Unigene61406\_JingChaoZuZhi2, Unigene62066\_JingChaoZuZhi2, Unigene6211\_JingChaoZuZhi2, Unigene6459\_JingChaoZuZhi2, Unigene6493\_JingChaoZuZhi2, Unigene66013\_JingChaoZuZhi2, Unigene6676\_JingChaoZuZhi2, Unigene6752\_JingChaoZuZhi2, Unigene67655\_JingChaoZuZhi2, Unigene67793\_JingChaoZuZhi2, Unigene69915\_JingChaoZuZhi2, Unigene70549\_JingChaoZuZhi2, Unigene71566\_JingChaoZuZhi2, Unigene72033\_JingChaoZuZhi2, Unigene722\_JingChaoZuZhi2, Unigene7417\_JingChaoZuZhi2, Unigene75859\_JingChaoZuZhi2, Unigene76575\_JingChaoZuZhi2, Unigene76809\_JingChaoZuZhi2, Unigene77570\_JingChaoZuZhi2, Unigene7939\_JingChaoZuZhi2, Unigene8003\_JingChaoZuZhi2, Unigene80331\_JingChaoZuZhi2, Unigene81086\_JingChaoZuZhi2, Unigene81687\_JingChaoZuZhi2, Unigene8191\_JingChaoZuZhi2, Unigene81948\_JingChaoZuZhi2, Unigene82073\_JingChaoZuZhi2, Unigene82219\_JingChaoZuZhi2, Unigene84944\_JingChaoZuZhi2, Unigene85574\_JingChaoZuZhi2, Unigene86206\_JingChaoZuZhi2, Unigene8621\_JingChaoZuZhi2, Unigene86526\_JingChaoZuZhi2, Unigene86713\_JingChaoZuZhi2, Unigene86899\_JingChaoZuZhi2, Unigene87004\_JingChaoZuZhi2, Unigene87305\_JingChaoZuZhi2, Unigene876\_JingChaoZuZhi2, Unigene87851\_JingChaoZuZhi2, Unigene88719\_JingChaoZuZhi2, Unigene89310\_JingChaoZuZhi2, Unigene8949\_JingChaoZuZhi2, Unigene89722\_JingChaoZuZhi2, Unigene90762\_JingChaoZuZhi2, Unigene90879\_JingChaoZuZhi2, Unigene90979\_JingChaoZuZhi2, Unigene91612\_JingChaoZuZhi2, Unigene91958\_JingChaoZuZhi2, Unigene92112\_JingChaoZuZhi2, Unigene92372\_JingChaoZuZhi2, Unigene92800\_JingChaoZuZhi2, Unigene93782\_JingChaoZuZhi2, Unigene93793\_JingChaoZuZhi2, Unigene93807\_JingChaoZuZhi2, Unigene9389\_JingChaoZuZhi2, Unigene93912\_JingChaoZuZhi2, Unigene9404\_JingChaoZuZhi2, Unigene94149\_JingChaoZuZhi2, Unigene94225\_JingChaoZuZhi2, Unigene94456\_JingChaoZuZhi2, Unigene9445\_JingChaoZuZhi2, Unigene94619\_JingChaoZuZhi2, Unigene9461\_JingChaoZuZhi2, Unigene94685\_JingChaoZuZhi2, Unigene94749\_JingChaoZuZhi2, Unigene9488\_JingChaoZuZhi2, Unigene95098\_JingChaoZuZhi2, Unigene95147\_JingChaoZuZhi2, Unigene9517\_JingChaoZuZhi2, Unigene95295\_JingChaoZuZhi2, Unigene95456\_JingChaoZuZhi2, Unigene95881\_JingChaoZuZhi2, Unigene96294\_JingChaoZuZhi2, Unigene9645\_JingChaoZuZhi2, Unigene9902\_JingChaoZuZhi2 |
| 53 | Arrhythmogenic right ventricular cardiomyopathy (ARVC) | Unigene10034\_JingChaoZuZhi2, Unigene10047\_JingChaoZuZhi2, Unigene10078\_JingChaoZuZhi2, Unigene10259\_JingChaoZuZhi2, Unigene10294\_JingChaoZuZhi2, Unigene10350\_JingChaoZuZhi2, Unigene1060\_JingChaoZuZhi2, Unigene10\_JingChaoZuZhi2, Unigene1111\_JingChaoZuZhi2, Unigene11446\_JingChaoZuZhi2, Unigene1162\_JingChaoZuZhi2, Unigene12091\_JingChaoZuZhi2, Unigene12329\_JingChaoZuZhi2, Unigene12889\_JingChaoZuZhi2, Unigene13026\_JingChaoZuZhi2, Unigene13395\_JingChaoZuZhi2, Unigene13607\_JingChaoZuZhi2, Unigene13854\_JingChaoZuZhi2, Unigene14234\_JingChaoZuZhi2, Unigene14285\_JingChaoZuZhi2, Unigene14707\_JingChaoZuZhi2, Unigene15029\_JingChaoZuZhi2, Unigene15937\_JingChaoZuZhi2, Unigene16004\_JingChaoZuZhi2, Unigene16121\_JingChaoZuZhi2, Unigene16229\_JingChaoZuZhi2, Unigene16480\_JingChaoZuZhi2, Unigene16873\_JingChaoZuZhi2, Unigene17128\_JingChaoZuZhi2, Unigene17132\_JingChaoZuZhi2, Unigene18184\_JingChaoZuZhi2, Unigene18643\_JingChaoZuZhi2, Unigene18790\_JingChaoZuZhi2, Unigene18808\_JingChaoZuZhi2, Unigene19404\_JingChaoZuZhi2, Unigene19957\_JingChaoZuZhi2, Unigene20000\_JingChaoZuZhi2, Unigene20159\_JingChaoZuZhi2, Unigene20223\_JingChaoZuZhi2, Unigene20255\_JingChaoZuZhi2, Unigene20629\_JingChaoZuZhi2, Unigene20892\_JingChaoZuZhi2, Unigene21011\_JingChaoZuZhi2, Unigene21354\_JingChaoZuZhi2, Unigene21818\_JingChaoZuZhi2, Unigene21956\_JingChaoZuZhi2, Unigene22024\_JingChaoZuZhi2, Unigene22323\_JingChaoZuZhi2, Unigene22440\_JingChaoZuZhi2, Unigene22445\_JingChaoZuZhi2, Unigene2305\_JingChaoZuZhi2, Unigene24645\_JingChaoZuZhi2, Unigene24772\_JingChaoZuZhi2, Unigene26679\_JingChaoZuZhi2, Unigene26987\_JingChaoZuZhi2, Unigene28208\_JingChaoZuZhi2, Unigene28467\_JingChaoZuZhi2, Unigene28737\_JingChaoZuZhi2, Unigene29312\_JingChaoZuZhi2, Unigene3085\_JingChaoZuZhi2, Unigene31918\_JingChaoZuZhi2, Unigene31982\_JingChaoZuZhi2, Unigene32314\_JingChaoZuZhi2, Unigene35016\_JingChaoZuZhi2, Unigene35243\_JingChaoZuZhi2, Unigene35304\_JingChaoZuZhi2, Unigene35624\_JingChaoZuZhi2, Unigene35952\_JingChaoZuZhi2, Unigene36404\_JingChaoZuZhi2, Unigene37675\_JingChaoZuZhi2, Unigene39102\_JingChaoZuZhi2, Unigene39674\_JingChaoZuZhi2, Unigene40919\_JingChaoZuZhi2, Unigene41842\_JingChaoZuZhi2, Unigene42248\_JingChaoZuZhi2, Unigene4224\_JingChaoZuZhi2, Unigene44066\_JingChaoZuZhi2, Unigene44190\_JingChaoZuZhi2, Unigene4522\_JingChaoZuZhi2, Unigene45399\_JingChaoZuZhi2, Unigene45468\_JingChaoZuZhi2, Unigene45782\_JingChaoZuZhi2, Unigene47199\_JingChaoZuZhi2, Unigene4782\_JingChaoZuZhi2, Unigene47926\_JingChaoZuZhi2, Unigene48576\_JingChaoZuZhi2, Unigene48843\_JingChaoZuZhi2, Unigene49526\_JingChaoZuZhi2, Unigene499\_JingChaoZuZhi2, Unigene5069\_JingChaoZuZhi2, Unigene51467\_JingChaoZuZhi2, Unigene5173\_JingChaoZuZhi2, Unigene52167\_JingChaoZuZhi2, Unigene53160\_JingChaoZuZhi2, Unigene54744\_JingChaoZuZhi2, Unigene54986\_JingChaoZuZhi2, Unigene55684\_JingChaoZuZhi2, Unigene5796\_JingChaoZuZhi2, Unigene58029\_JingChaoZuZhi2, Unigene59146\_JingChaoZuZhi2, Unigene5989\_JingChaoZuZhi2, Unigene60401\_JingChaoZuZhi2, Unigene61244\_JingChaoZuZhi2, Unigene61801\_JingChaoZuZhi2, Unigene61911\_JingChaoZuZhi2, Unigene61925\_JingChaoZuZhi2, Unigene62776\_JingChaoZuZhi2, Unigene63040\_JingChaoZuZhi2, Unigene63449\_JingChaoZuZhi2, Unigene6361\_JingChaoZuZhi2, Unigene64227\_JingChaoZuZhi2, Unigene64750\_JingChaoZuZhi2, Unigene6634\_JingChaoZuZhi2, Unigene6699\_JingChaoZuZhi2, Unigene67542\_JingChaoZuZhi2, Unigene69100\_JingChaoZuZhi2, Unigene71017\_JingChaoZuZhi2, Unigene71414\_JingChaoZuZhi2, Unigene71592\_JingChaoZuZhi2, Unigene71640\_JingChaoZuZhi2, Unigene72826\_JingChaoZuZhi2, Unigene73554\_JingChaoZuZhi2, Unigene73571\_JingChaoZuZhi2, Unigene73579\_JingChaoZuZhi2, Unigene7364\_JingChaoZuZhi2, Unigene75840\_JingChaoZuZhi2, Unigene76803\_JingChaoZuZhi2, Unigene77071\_JingChaoZuZhi2, Unigene77549\_JingChaoZuZhi2, Unigene77965\_JingChaoZuZhi2, Unigene79109\_JingChaoZuZhi2, Unigene80494\_JingChaoZuZhi2, Unigene80605\_JingChaoZuZhi2, Unigene80863\_JingChaoZuZhi2, Unigene81395\_JingChaoZuZhi2, Unigene81598\_JingChaoZuZhi2, Unigene82117\_JingChaoZuZhi2, Unigene82489\_JingChaoZuZhi2, Unigene82848\_JingChaoZuZhi2, Unigene83400\_JingChaoZuZhi2, Unigene83606\_JingChaoZuZhi2, Unigene83744\_JingChaoZuZhi2, Unigene83872\_JingChaoZuZhi2, Unigene84519\_JingChaoZuZhi2, Unigene85193\_JingChaoZuZhi2, Unigene85658\_JingChaoZuZhi2, Unigene85923\_JingChaoZuZhi2, Unigene86051\_JingChaoZuZhi2, Unigene86312\_JingChaoZuZhi2, Unigene86616\_JingChaoZuZhi2, Unigene87191\_JingChaoZuZhi2, Unigene87360\_JingChaoZuZhi2, Unigene88040\_JingChaoZuZhi2, Unigene88342\_JingChaoZuZhi2, Unigene88544\_JingChaoZuZhi2, Unigene88925\_JingChaoZuZhi2, Unigene89045\_JingChaoZuZhi2, Unigene89060\_JingChaoZuZhi2, Unigene89090\_JingChaoZuZhi2, Unigene89175\_JingChaoZuZhi2, Unigene89329\_JingChaoZuZhi2, Unigene90179\_JingChaoZuZhi2, Unigene90588\_JingChaoZuZhi2, Unigene91043\_JingChaoZuZhi2, Unigene91163\_JingChaoZuZhi2, Unigene92104\_JingChaoZuZhi2, Unigene92671\_JingChaoZuZhi2, Unigene93703\_JingChaoZuZhi2, Unigene94110\_JingChaoZuZhi2, Unigene94156\_JingChaoZuZhi2, Unigene94214\_JingChaoZuZhi2, Unigene94272\_JingChaoZuZhi2, Unigene94337\_JingChaoZuZhi2, Unigene94370\_JingChaoZuZhi2, Unigene94561\_JingChaoZuZhi2, Unigene95020\_JingChaoZuZhi2, Unigene951\_JingChaoZuZhi2, Unigene95213\_JingChaoZuZhi2, Unigene95254\_JingChaoZuZhi2, Unigene95799\_JingChaoZuZhi2, Unigene95807\_JingChaoZuZhi2, Unigene95889\_JingChaoZuZhi2, Unigene95907\_JingChaoZuZhi2, Unigene96033\_JingChaoZuZhi2, Unigene96091\_JingChaoZuZhi2, Unigene96121\_JingChaoZuZhi2, Unigene96234\_JingChaoZuZhi2, Unigene9739\_JingChaoZuZhi2 |
| 54 | Melanogenesis | Unigene10031\_JingChaoZuZhi2, Unigene10318\_JingChaoZuZhi2, Unigene11621\_JingChaoZuZhi2, Unigene11726\_JingChaoZuZhi2, Unigene12455\_JingChaoZuZhi2, Unigene12634\_JingChaoZuZhi2, Unigene12938\_JingChaoZuZhi2, Unigene13650\_JingChaoZuZhi2, Unigene13770\_JingChaoZuZhi2, Unigene13869\_JingChaoZuZhi2, Unigene14360\_JingChaoZuZhi2, Unigene14567\_JingChaoZuZhi2, Unigene15651\_JingChaoZuZhi2, Unigene16229\_JingChaoZuZhi2, Unigene16468\_JingChaoZuZhi2, Unigene16566\_JingChaoZuZhi2, Unigene16820\_JingChaoZuZhi2, Unigene16958\_JingChaoZuZhi2, Unigene16991\_JingChaoZuZhi2, Unigene1778\_JingChaoZuZhi2, Unigene18177\_JingChaoZuZhi2, Unigene18307\_JingChaoZuZhi2, Unigene19238\_JingChaoZuZhi2, Unigene19273\_JingChaoZuZhi2, Unigene20013\_JingChaoZuZhi2, Unigene20052\_JingChaoZuZhi2, Unigene21578\_JingChaoZuZhi2, Unigene21678\_JingChaoZuZhi2, Unigene21815\_JingChaoZuZhi2, Unigene22506\_JingChaoZuZhi2, Unigene22580\_JingChaoZuZhi2, Unigene22673\_JingChaoZuZhi2, Unigene22708\_JingChaoZuZhi2, Unigene22779\_JingChaoZuZhi2, Unigene23122\_JingChaoZuZhi2, Unigene24425\_JingChaoZuZhi2, Unigene2473\_JingChaoZuZhi2, Unigene26730\_JingChaoZuZhi2, Unigene2960\_JingChaoZuZhi2, Unigene3122\_JingChaoZuZhi2, Unigene32827\_JingChaoZuZhi2, Unigene32999\_JingChaoZuZhi2, Unigene34574\_JingChaoZuZhi2, Unigene34723\_JingChaoZuZhi2, Unigene34929\_JingChaoZuZhi2, Unigene36819\_JingChaoZuZhi2, Unigene38191\_JingChaoZuZhi2, Unigene38324\_JingChaoZuZhi2, Unigene3910\_JingChaoZuZhi2, Unigene395\_JingChaoZuZhi2, Unigene39825\_JingChaoZuZhi2, Unigene39832\_JingChaoZuZhi2, Unigene40335\_JingChaoZuZhi2, Unigene41333\_JingChaoZuZhi2, Unigene43059\_JingChaoZuZhi2, Unigene44228\_JingChaoZuZhi2, Unigene45166\_JingChaoZuZhi2, Unigene46240\_JingChaoZuZhi2, Unigene47872\_JingChaoZuZhi2, Unigene4803\_JingChaoZuZhi2, Unigene49203\_JingChaoZuZhi2, Unigene51991\_JingChaoZuZhi2, Unigene5226\_JingChaoZuZhi2, Unigene53935\_JingChaoZuZhi2, Unigene54012\_JingChaoZuZhi2, Unigene54367\_JingChaoZuZhi2, Unigene55392\_JingChaoZuZhi2, Unigene56922\_JingChaoZuZhi2, Unigene57185\_JingChaoZuZhi2, Unigene58474\_JingChaoZuZhi2, Unigene59102\_JingChaoZuZhi2, Unigene59750\_JingChaoZuZhi2, Unigene6028\_JingChaoZuZhi2, Unigene60777\_JingChaoZuZhi2, Unigene61043\_JingChaoZuZhi2, Unigene61702\_JingChaoZuZhi2, Unigene61911\_JingChaoZuZhi2, Unigene63323\_JingChaoZuZhi2, Unigene63406\_JingChaoZuZhi2, Unigene63449\_JingChaoZuZhi2, Unigene6393\_JingChaoZuZhi2, Unigene64164\_JingChaoZuZhi2, Unigene64227\_JingChaoZuZhi2, Unigene6467\_JingChaoZuZhi2, Unigene64830\_JingChaoZuZhi2, Unigene65294\_JingChaoZuZhi2, Unigene65445\_JingChaoZuZhi2, Unigene66073\_JingChaoZuZhi2, Unigene66540\_JingChaoZuZhi2, Unigene66865\_JingChaoZuZhi2, Unigene70225\_JingChaoZuZhi2, Unigene70493\_JingChaoZuZhi2, Unigene70549\_JingChaoZuZhi2, Unigene70723\_JingChaoZuZhi2, Unigene71365\_JingChaoZuZhi2, Unigene72445\_JingChaoZuZhi2, Unigene72643\_JingChaoZuZhi2, Unigene72809\_JingChaoZuZhi2, Unigene73203\_JingChaoZuZhi2, Unigene7373\_JingChaoZuZhi2, Unigene73765\_JingChaoZuZhi2, Unigene73873\_JingChaoZuZhi2, Unigene7407\_JingChaoZuZhi2, Unigene74569\_JingChaoZuZhi2, Unigene7456\_JingChaoZuZhi2, Unigene74587\_JingChaoZuZhi2, Unigene74609\_JingChaoZuZhi2, Unigene75228\_JingChaoZuZhi2, Unigene75235\_JingChaoZuZhi2, Unigene75919\_JingChaoZuZhi2, Unigene76614\_JingChaoZuZhi2, Unigene77149\_JingChaoZuZhi2, Unigene7747\_JingChaoZuZhi2, Unigene77587\_JingChaoZuZhi2, Unigene78078\_JingChaoZuZhi2, Unigene78168\_JingChaoZuZhi2, Unigene78235\_JingChaoZuZhi2, Unigene78241\_JingChaoZuZhi2, Unigene78255\_JingChaoZuZhi2, Unigene78522\_JingChaoZuZhi2, Unigene7881\_JingChaoZuZhi2, Unigene79118\_JingChaoZuZhi2, Unigene80123\_JingChaoZuZhi2, Unigene8054\_JingChaoZuZhi2, Unigene81716\_JingChaoZuZhi2, Unigene8191\_JingChaoZuZhi2, Unigene82359\_JingChaoZuZhi2, Unigene82525\_JingChaoZuZhi2, Unigene83017\_JingChaoZuZhi2, Unigene83188\_JingChaoZuZhi2, Unigene83196\_JingChaoZuZhi2, Unigene8507\_JingChaoZuZhi2, Unigene8523\_JingChaoZuZhi2, Unigene86091\_JingChaoZuZhi2, Unigene86119\_JingChaoZuZhi2, Unigene86504\_JingChaoZuZhi2, Unigene87082\_JingChaoZuZhi2, Unigene87332\_JingChaoZuZhi2, Unigene87607\_JingChaoZuZhi2, Unigene876\_JingChaoZuZhi2, Unigene88167\_JingChaoZuZhi2, Unigene88385\_JingChaoZuZhi2, Unigene88544\_JingChaoZuZhi2, Unigene88594\_JingChaoZuZhi2, Unigene88620\_JingChaoZuZhi2, Unigene88708\_JingChaoZuZhi2, Unigene88719\_JingChaoZuZhi2, Unigene88724\_JingChaoZuZhi2, Unigene88773\_JingChaoZuZhi2, Unigene89093\_JingChaoZuZhi2, Unigene89161\_JingChaoZuZhi2, Unigene89310\_JingChaoZuZhi2, Unigene89323\_JingChaoZuZhi2, Unigene89603\_JingChaoZuZhi2, Unigene89752\_JingChaoZuZhi2, Unigene89807\_JingChaoZuZhi2, Unigene90761\_JingChaoZuZhi2, Unigene91119\_JingChaoZuZhi2, Unigene91221\_JingChaoZuZhi2, Unigene91254\_JingChaoZuZhi2, Unigene91458\_JingChaoZuZhi2, Unigene91527\_JingChaoZuZhi2, Unigene91739\_JingChaoZuZhi2, Unigene91924\_JingChaoZuZhi2, Unigene92651\_JingChaoZuZhi2, Unigene92715\_JingChaoZuZhi2, Unigene93012\_JingChaoZuZhi2, Unigene93195\_JingChaoZuZhi2, Unigene93541\_JingChaoZuZhi2, Unigene93588\_JingChaoZuZhi2, Unigene93714\_JingChaoZuZhi2, Unigene94242\_JingChaoZuZhi2, Unigene94272\_JingChaoZuZhi2, Unigene9428\_JingChaoZuZhi2, Unigene94711\_JingChaoZuZhi2, Unigene94819\_JingChaoZuZhi2, Unigene95098\_JingChaoZuZhi2, Unigene95295\_JingChaoZuZhi2, Unigene95568\_JingChaoZuZhi2, Unigene95677\_JingChaoZuZhi2, Unigene95751\_JingChaoZuZhi2, Unigene95916\_JingChaoZuZhi2, Unigene96124\_JingChaoZuZhi2, Unigene96234\_JingChaoZuZhi2, Unigene96262\_JingChaoZuZhi2, Unigene9654\_JingChaoZuZhi2, Unigene9902\_JingChaoZuZhi2 |
| 55 | Vasopressin-regulated water reabsorption | Unigene10189\_JingChaoZuZhi2, Unigene10503\_JingChaoZuZhi2, Unigene10526\_JingChaoZuZhi2, Unigene10835\_JingChaoZuZhi2, Unigene11146\_JingChaoZuZhi2, Unigene1157\_JingChaoZuZhi2, Unigene11600\_JingChaoZuZhi2, Unigene11718\_JingChaoZuZhi2, Unigene12706\_JingChaoZuZhi2, Unigene12729\_JingChaoZuZhi2, Unigene12938\_JingChaoZuZhi2, Unigene13026\_JingChaoZuZhi2, Unigene13650\_JingChaoZuZhi2, Unigene13770\_JingChaoZuZhi2, Unigene13857\_JingChaoZuZhi2, Unigene14125\_JingChaoZuZhi2, Unigene14144\_JingChaoZuZhi2, Unigene14298\_JingChaoZuZhi2, Unigene15151\_JingChaoZuZhi2, Unigene15651\_JingChaoZuZhi2, Unigene1599\_JingChaoZuZhi2, Unigene16337\_JingChaoZuZhi2, Unigene16388\_JingChaoZuZhi2, Unigene167\_JingChaoZuZhi2, Unigene16939\_JingChaoZuZhi2, Unigene1778\_JingChaoZuZhi2, Unigene17813\_JingChaoZuZhi2, Unigene18059\_JingChaoZuZhi2, Unigene18307\_JingChaoZuZhi2, Unigene19108\_JingChaoZuZhi2, Unigene19691\_JingChaoZuZhi2, Unigene20305\_JingChaoZuZhi2, Unigene20770\_JingChaoZuZhi2, Unigene21063\_JingChaoZuZhi2, Unigene21147\_JingChaoZuZhi2, Unigene21533\_JingChaoZuZhi2, Unigene21713\_JingChaoZuZhi2, Unigene21741\_JingChaoZuZhi2, Unigene21815\_JingChaoZuZhi2, Unigene22263\_JingChaoZuZhi2, Unigene2273\_JingChaoZuZhi2, Unigene22892\_JingChaoZuZhi2, Unigene22963\_JingChaoZuZhi2, Unigene23482\_JingChaoZuZhi2, Unigene24252\_JingChaoZuZhi2, Unigene24425\_JingChaoZuZhi2, Unigene27884\_JingChaoZuZhi2, Unigene3022\_JingChaoZuZhi2, Unigene3122\_JingChaoZuZhi2, Unigene3298\_JingChaoZuZhi2, Unigene32999\_JingChaoZuZhi2, Unigene3345\_JingChaoZuZhi2, Unigene3377\_JingChaoZuZhi2, Unigene347\_JingChaoZuZhi2, Unigene3665\_JingChaoZuZhi2, Unigene37741\_JingChaoZuZhi2, Unigene37803\_JingChaoZuZhi2, Unigene3782\_JingChaoZuZhi2, Unigene37928\_JingChaoZuZhi2, Unigene39832\_JingChaoZuZhi2, Unigene4032\_JingChaoZuZhi2, Unigene40335\_JingChaoZuZhi2, Unigene40848\_JingChaoZuZhi2, Unigene41339\_JingChaoZuZhi2, Unigene41686\_JingChaoZuZhi2, Unigene42369\_JingChaoZuZhi2, Unigene43255\_JingChaoZuZhi2, Unigene43537\_JingChaoZuZhi2, Unigene44625\_JingChaoZuZhi2, Unigene45043\_JingChaoZuZhi2, Unigene45044\_JingChaoZuZhi2, Unigene47374\_JingChaoZuZhi2, Unigene47701\_JingChaoZuZhi2, Unigene48984\_JingChaoZuZhi2, Unigene49981\_JingChaoZuZhi2, Unigene50307\_JingChaoZuZhi2, Unigene52002\_JingChaoZuZhi2, Unigene52821\_JingChaoZuZhi2, Unigene5312\_JingChaoZuZhi2, Unigene53871\_JingChaoZuZhi2, Unigene53935\_JingChaoZuZhi2, Unigene53953\_JingChaoZuZhi2, Unigene54012\_JingChaoZuZhi2, Unigene55529\_JingChaoZuZhi2, Unigene57607\_JingChaoZuZhi2, Unigene5953\_JingChaoZuZhi2, Unigene59580\_JingChaoZuZhi2, Unigene59896\_JingChaoZuZhi2, Unigene60221\_JingChaoZuZhi2, Unigene60242\_JingChaoZuZhi2, Unigene60481\_JingChaoZuZhi2, Unigene60580\_JingChaoZuZhi2, Unigene6121\_JingChaoZuZhi2, Unigene61421\_JingChaoZuZhi2, Unigene62752\_JingChaoZuZhi2, Unigene63679\_JingChaoZuZhi2, Unigene64251\_JingChaoZuZhi2, Unigene64760\_JingChaoZuZhi2, Unigene65013\_JingChaoZuZhi2, Unigene65337\_JingChaoZuZhi2, Unigene65775\_JingChaoZuZhi2, Unigene66590\_JingChaoZuZhi2, Unigene67676\_JingChaoZuZhi2, Unigene67986\_JingChaoZuZhi2, Unigene68009\_JingChaoZuZhi2, Unigene68111\_JingChaoZuZhi2, Unigene68477\_JingChaoZuZhi2, Unigene69203\_JingChaoZuZhi2, Unigene69708\_JingChaoZuZhi2, Unigene70314\_JingChaoZuZhi2, Unigene70632\_JingChaoZuZhi2, Unigene70808\_JingChaoZuZhi2, Unigene70924\_JingChaoZuZhi2, Unigene71365\_JingChaoZuZhi2, Unigene7139\_JingChaoZuZhi2, Unigene72269\_JingChaoZuZhi2, Unigene73052\_JingChaoZuZhi2, Unigene7344\_JingChaoZuZhi2, Unigene73826\_JingChaoZuZhi2, Unigene7398\_JingChaoZuZhi2, Unigene74948\_JingChaoZuZhi2, Unigene74979\_JingChaoZuZhi2, Unigene75316\_JingChaoZuZhi2, Unigene76439\_JingChaoZuZhi2, Unigene7672\_JingChaoZuZhi2, Unigene76855\_JingChaoZuZhi2, Unigene77238\_JingChaoZuZhi2, Unigene77358\_JingChaoZuZhi2, Unigene78235\_JingChaoZuZhi2, Unigene79595\_JingChaoZuZhi2, Unigene79649\_JingChaoZuZhi2, Unigene79856\_JingChaoZuZhi2, Unigene79925\_JingChaoZuZhi2, Unigene80070\_JingChaoZuZhi2, Unigene80489\_JingChaoZuZhi2, Unigene83219\_JingChaoZuZhi2, Unigene8372\_JingChaoZuZhi2, Unigene83931\_JingChaoZuZhi2, Unigene84046\_JingChaoZuZhi2, Unigene84512\_JingChaoZuZhi2, Unigene84569\_JingChaoZuZhi2, Unigene84830\_JingChaoZuZhi2, Unigene84984\_JingChaoZuZhi2, Unigene86544\_JingChaoZuZhi2, Unigene86640\_JingChaoZuZhi2, Unigene86945\_JingChaoZuZhi2, Unigene87780\_JingChaoZuZhi2, Unigene88041\_JingChaoZuZhi2, Unigene88095\_JingChaoZuZhi2, Unigene88284\_JingChaoZuZhi2, Unigene88522\_JingChaoZuZhi2, Unigene89011\_JingChaoZuZhi2, Unigene89410\_JingChaoZuZhi2, Unigene90486\_JingChaoZuZhi2, Unigene91391\_JingChaoZuZhi2, Unigene91527\_JingChaoZuZhi2, Unigene91591\_JingChaoZuZhi2, Unigene91611\_JingChaoZuZhi2, Unigene91670\_JingChaoZuZhi2, Unigene91730\_JingChaoZuZhi2, Unigene91791\_JingChaoZuZhi2, Unigene91953\_JingChaoZuZhi2, Unigene91978\_JingChaoZuZhi2, Unigene92547\_JingChaoZuZhi2, Unigene92590\_JingChaoZuZhi2, Unigene92651\_JingChaoZuZhi2, Unigene92863\_JingChaoZuZhi2, Unigene93207\_JingChaoZuZhi2, Unigene93541\_JingChaoZuZhi2, Unigene93693\_JingChaoZuZhi2, Unigene93781\_JingChaoZuZhi2, Unigene94165\_JingChaoZuZhi2, Unigene94656\_JingChaoZuZhi2, Unigene94680\_JingChaoZuZhi2, Unigene94706\_JingChaoZuZhi2, Unigene95065\_JingChaoZuZhi2, Unigene95308\_JingChaoZuZhi2, Unigene95488\_JingChaoZuZhi2, Unigene95689\_JingChaoZuZhi2, Unigene95855\_JingChaoZuZhi2, Unigene95916\_JingChaoZuZhi2, Unigene95964\_JingChaoZuZhi2, Unigene95987\_JingChaoZuZhi2, Unigene96151\_JingChaoZuZhi2, Unigene96269\_JingChaoZuZhi2, Unigene9645\_JingChaoZuZhi2 |
| 56 | Gap junction | Unigene10016\_JingChaoZuZhi2, Unigene10318\_JingChaoZuZhi2, Unigene10422\_JingChaoZuZhi2, Unigene10784\_JingChaoZuZhi2, Unigene11281\_JingChaoZuZhi2, Unigene11605\_JingChaoZuZhi2, Unigene11621\_JingChaoZuZhi2, Unigene11726\_JingChaoZuZhi2, Unigene12073\_JingChaoZuZhi2, Unigene12098\_JingChaoZuZhi2, Unigene12414\_JingChaoZuZhi2, Unigene12420\_JingChaoZuZhi2, Unigene12912\_JingChaoZuZhi2, Unigene12938\_JingChaoZuZhi2, Unigene13770\_JingChaoZuZhi2, Unigene14170\_JingChaoZuZhi2, Unigene14360\_JingChaoZuZhi2, Unigene14567\_JingChaoZuZhi2, Unigene14878\_JingChaoZuZhi2, Unigene15009\_JingChaoZuZhi2, Unigene15182\_JingChaoZuZhi2, Unigene15406\_JingChaoZuZhi2, Unigene15651\_JingChaoZuZhi2, Unigene16266\_JingChaoZuZhi2, Unigene16991\_JingChaoZuZhi2, Unigene17666\_JingChaoZuZhi2, Unigene18128\_JingChaoZuZhi2, Unigene18307\_JingChaoZuZhi2, Unigene19304\_JingChaoZuZhi2, Unigene21578\_JingChaoZuZhi2, Unigene21605\_JingChaoZuZhi2, Unigene21815\_JingChaoZuZhi2, Unigene22779\_JingChaoZuZhi2, Unigene234\_JingChaoZuZhi2, Unigene23827\_JingChaoZuZhi2, Unigene25912\_JingChaoZuZhi2, Unigene2656\_JingChaoZuZhi2, Unigene26592\_JingChaoZuZhi2, Unigene26730\_JingChaoZuZhi2, Unigene26746\_JingChaoZuZhi2, Unigene26874\_JingChaoZuZhi2, Unigene2790\_JingChaoZuZhi2, Unigene29295\_JingChaoZuZhi2, Unigene2960\_JingChaoZuZhi2, Unigene3122\_JingChaoZuZhi2, Unigene31285\_JingChaoZuZhi2, Unigene31868\_JingChaoZuZhi2, Unigene32827\_JingChaoZuZhi2, Unigene32941\_JingChaoZuZhi2, Unigene33692\_JingChaoZuZhi2, Unigene34929\_JingChaoZuZhi2, Unigene36354\_JingChaoZuZhi2, Unigene37252\_JingChaoZuZhi2, Unigene37639\_JingChaoZuZhi2, Unigene38458\_JingChaoZuZhi2, Unigene39832\_JingChaoZuZhi2, Unigene4028\_JingChaoZuZhi2, Unigene40335\_JingChaoZuZhi2, Unigene40674\_JingChaoZuZhi2, Unigene41333\_JingChaoZuZhi2, Unigene4186\_JingChaoZuZhi2, Unigene41953\_JingChaoZuZhi2, Unigene41973\_JingChaoZuZhi2, Unigene43059\_JingChaoZuZhi2, Unigene43584\_JingChaoZuZhi2, Unigene4449\_JingChaoZuZhi2, Unigene45086\_JingChaoZuZhi2, Unigene46155\_JingChaoZuZhi2, Unigene4838\_JingChaoZuZhi2, Unigene49056\_JingChaoZuZhi2, Unigene49203\_JingChaoZuZhi2, Unigene49472\_JingChaoZuZhi2, Unigene49798\_JingChaoZuZhi2, Unigene50084\_JingChaoZuZhi2, Unigene51991\_JingChaoZuZhi2, Unigene52195\_JingChaoZuZhi2, Unigene52758\_JingChaoZuZhi2, Unigene52759\_JingChaoZuZhi2, Unigene53553\_JingChaoZuZhi2, Unigene53935\_JingChaoZuZhi2, Unigene53958\_JingChaoZuZhi2, Unigene54012\_JingChaoZuZhi2, Unigene54367\_JingChaoZuZhi2, Unigene54449\_JingChaoZuZhi2, Unigene54470\_JingChaoZuZhi2, Unigene56079\_JingChaoZuZhi2, Unigene56567\_JingChaoZuZhi2, Unigene57184\_JingChaoZuZhi2, Unigene57185\_JingChaoZuZhi2, Unigene58011\_JingChaoZuZhi2, Unigene58608\_JingChaoZuZhi2, Unigene58988\_JingChaoZuZhi2, Unigene59102\_JingChaoZuZhi2, Unigene6028\_JingChaoZuZhi2, Unigene60800\_JingChaoZuZhi2, Unigene62786\_JingChaoZuZhi2, Unigene62821\_JingChaoZuZhi2, Unigene63409\_JingChaoZuZhi2, Unigene6393\_JingChaoZuZhi2, Unigene63994\_JingChaoZuZhi2, Unigene64164\_JingChaoZuZhi2, Unigene64830\_JingChaoZuZhi2, Unigene6493\_JingChaoZuZhi2, Unigene65445\_JingChaoZuZhi2, Unigene65652\_JingChaoZuZhi2, Unigene66073\_JingChaoZuZhi2, Unigene66540\_JingChaoZuZhi2, Unigene66904\_JingChaoZuZhi2, Unigene67793\_JingChaoZuZhi2, Unigene67804\_JingChaoZuZhi2, Unigene68689\_JingChaoZuZhi2, Unigene69910\_JingChaoZuZhi2, Unigene70385\_JingChaoZuZhi2, Unigene70493\_JingChaoZuZhi2, Unigene70549\_JingChaoZuZhi2, Unigene70723\_JingChaoZuZhi2, Unigene71118\_JingChaoZuZhi2, Unigene71365\_JingChaoZuZhi2, Unigene71599\_JingChaoZuZhi2, Unigene71885\_JingChaoZuZhi2, Unigene72521\_JingChaoZuZhi2, Unigene72643\_JingChaoZuZhi2, Unigene73595\_JingChaoZuZhi2, Unigene7456\_JingChaoZuZhi2, Unigene75235\_JingChaoZuZhi2, Unigene77024\_JingChaoZuZhi2, Unigene77149\_JingChaoZuZhi2, Unigene78007\_JingChaoZuZhi2, Unigene78078\_JingChaoZuZhi2, Unigene78235\_JingChaoZuZhi2, Unigene78460\_JingChaoZuZhi2, Unigene78490\_JingChaoZuZhi2, Unigene78574\_JingChaoZuZhi2, Unigene7877\_JingChaoZuZhi2, Unigene78784\_JingChaoZuZhi2, Unigene7939\_JingChaoZuZhi2, Unigene80927\_JingChaoZuZhi2, Unigene8191\_JingChaoZuZhi2, Unigene83017\_JingChaoZuZhi2, Unigene84247\_JingChaoZuZhi2, Unigene84655\_JingChaoZuZhi2, Unigene84844\_JingChaoZuZhi2, Unigene85059\_JingChaoZuZhi2, Unigene85574\_JingChaoZuZhi2, Unigene86359\_JingChaoZuZhi2, Unigene87332\_JingChaoZuZhi2, Unigene87567\_JingChaoZuZhi2, Unigene87607\_JingChaoZuZhi2, Unigene876\_JingChaoZuZhi2, Unigene87941\_JingChaoZuZhi2, Unigene88121\_JingChaoZuZhi2, Unigene88385\_JingChaoZuZhi2, Unigene88773\_JingChaoZuZhi2, Unigene89147\_JingChaoZuZhi2, Unigene89603\_JingChaoZuZhi2, Unigene89741\_JingChaoZuZhi2, Unigene89807\_JingChaoZuZhi2, Unigene90239\_JingChaoZuZhi2, Unigene90832\_JingChaoZuZhi2, Unigene91221\_JingChaoZuZhi2, Unigene91527\_JingChaoZuZhi2, Unigene91612\_JingChaoZuZhi2, Unigene91739\_JingChaoZuZhi2, Unigene91958\_JingChaoZuZhi2, Unigene92367\_JingChaoZuZhi2, Unigene92918\_JingChaoZuZhi2, Unigene92962\_JingChaoZuZhi2, Unigene93012\_JingChaoZuZhi2, Unigene93270\_JingChaoZuZhi2, Unigene93522\_JingChaoZuZhi2, Unigene93793\_JingChaoZuZhi2, Unigene94049\_JingChaoZuZhi2, Unigene94076\_JingChaoZuZhi2, Unigene94149\_JingChaoZuZhi2, Unigene94456\_JingChaoZuZhi2, Unigene94980\_JingChaoZuZhi2, Unigene95456\_JingChaoZuZhi2, Unigene95677\_JingChaoZuZhi2, Unigene95916\_JingChaoZuZhi2, Unigene96262\_JingChaoZuZhi2, Unigene9654\_JingChaoZuZhi2, Unigene9812\_JingChaoZuZhi2, Unigene9902\_JingChaoZuZhi2 |
| 57 | Prostate cancer | Unigene10031\_JingChaoZuZhi2, Unigene10422\_JingChaoZuZhi2, Unigene10932\_JingChaoZuZhi2, Unigene11000\_JingChaoZuZhi2, Unigene11803\_JingChaoZuZhi2, Unigene11961\_JingChaoZuZhi2, Unigene12292\_JingChaoZuZhi2, Unigene12420\_JingChaoZuZhi2, Unigene12577\_JingChaoZuZhi2, Unigene12633\_JingChaoZuZhi2, Unigene12634\_JingChaoZuZhi2, Unigene12823\_JingChaoZuZhi2, Unigene13176\_JingChaoZuZhi2, Unigene13337\_JingChaoZuZhi2, Unigene13650\_JingChaoZuZhi2, Unigene13716\_JingChaoZuZhi2, Unigene14339\_JingChaoZuZhi2, Unigene14390\_JingChaoZuZhi2, Unigene14912\_JingChaoZuZhi2, Unigene15228\_JingChaoZuZhi2, Unigene16229\_JingChaoZuZhi2, Unigene16468\_JingChaoZuZhi2, Unigene16820\_JingChaoZuZhi2, Unigene16958\_JingChaoZuZhi2, Unigene17247\_JingChaoZuZhi2, Unigene17467\_JingChaoZuZhi2, Unigene1778\_JingChaoZuZhi2, Unigene18672\_JingChaoZuZhi2, Unigene19238\_JingChaoZuZhi2, Unigene19307\_JingChaoZuZhi2, Unigene20052\_JingChaoZuZhi2, Unigene20157\_JingChaoZuZhi2, Unigene20840\_JingChaoZuZhi2, Unigene20991\_JingChaoZuZhi2, Unigene21513\_JingChaoZuZhi2, Unigene21697\_JingChaoZuZhi2, Unigene22466\_JingChaoZuZhi2, Unigene22580\_JingChaoZuZhi2, Unigene22673\_JingChaoZuZhi2, Unigene22708\_JingChaoZuZhi2, Unigene22892\_JingChaoZuZhi2, Unigene2456\_JingChaoZuZhi2, Unigene2473\_JingChaoZuZhi2, Unigene27651\_JingChaoZuZhi2, Unigene2790\_JingChaoZuZhi2, Unigene34365\_JingChaoZuZhi2, Unigene36751\_JingChaoZuZhi2, Unigene37070\_JingChaoZuZhi2, Unigene37495\_JingChaoZuZhi2, Unigene37928\_JingChaoZuZhi2, Unigene38741\_JingChaoZuZhi2, Unigene3931\_JingChaoZuZhi2, Unigene3936\_JingChaoZuZhi2, Unigene395\_JingChaoZuZhi2, Unigene39825\_JingChaoZuZhi2, Unigene39876\_JingChaoZuZhi2, Unigene40020\_JingChaoZuZhi2, Unigene41686\_JingChaoZuZhi2, Unigene4186\_JingChaoZuZhi2, Unigene4377\_JingChaoZuZhi2, Unigene46240\_JingChaoZuZhi2, Unigene46463\_JingChaoZuZhi2, Unigene47450\_JingChaoZuZhi2, Unigene48223\_JingChaoZuZhi2, Unigene4838\_JingChaoZuZhi2, Unigene48751\_JingChaoZuZhi2, Unigene49798\_JingChaoZuZhi2, Unigene50611\_JingChaoZuZhi2, Unigene50713\_JingChaoZuZhi2, Unigene54320\_JingChaoZuZhi2, Unigene54470\_JingChaoZuZhi2, Unigene55810\_JingChaoZuZhi2, Unigene57488\_JingChaoZuZhi2, Unigene57579\_JingChaoZuZhi2, Unigene5758\_JingChaoZuZhi2, Unigene58474\_JingChaoZuZhi2, Unigene58582\_JingChaoZuZhi2, Unigene5875\_JingChaoZuZhi2, Unigene60180\_JingChaoZuZhi2, Unigene6028\_JingChaoZuZhi2, Unigene60326\_JingChaoZuZhi2, Unigene6121\_JingChaoZuZhi2, Unigene61911\_JingChaoZuZhi2, Unigene62568\_JingChaoZuZhi2, Unigene62786\_JingChaoZuZhi2, Unigene63237\_JingChaoZuZhi2, Unigene63323\_JingChaoZuZhi2, Unigene63406\_JingChaoZuZhi2, Unigene63449\_JingChaoZuZhi2, Unigene64126\_JingChaoZuZhi2, Unigene64227\_JingChaoZuZhi2, Unigene64583\_JingChaoZuZhi2, Unigene6467\_JingChaoZuZhi2, Unigene6493\_JingChaoZuZhi2, Unigene65775\_JingChaoZuZhi2, Unigene66060\_JingChaoZuZhi2, Unigene67793\_JingChaoZuZhi2, Unigene67983\_JingChaoZuZhi2, Unigene68283\_JingChaoZuZhi2, Unigene69565\_JingChaoZuZhi2, Unigene70549\_JingChaoZuZhi2, Unigene70921\_JingChaoZuZhi2, Unigene72521\_JingChaoZuZhi2, Unigene73162\_JingChaoZuZhi2, Unigene74609\_JingChaoZuZhi2, Unigene74771\_JingChaoZuZhi2, Unigene75317\_JingChaoZuZhi2, Unigene75948\_JingChaoZuZhi2, Unigene76392\_JingChaoZuZhi2, Unigene77016\_JingChaoZuZhi2, Unigene77209\_JingChaoZuZhi2, Unigene77570\_JingChaoZuZhi2, Unigene78522\_JingChaoZuZhi2, Unigene78574\_JingChaoZuZhi2, Unigene80123\_JingChaoZuZhi2, Unigene81480\_JingChaoZuZhi2, Unigene81716\_JingChaoZuZhi2, Unigene8191\_JingChaoZuZhi2, Unigene83188\_JingChaoZuZhi2, Unigene83588\_JingChaoZuZhi2, Unigene83978\_JingChaoZuZhi2, Unigene84531\_JingChaoZuZhi2, Unigene84930\_JingChaoZuZhi2, Unigene85038\_JingChaoZuZhi2, Unigene85172\_JingChaoZuZhi2, Unigene85574\_JingChaoZuZhi2, Unigene86119\_JingChaoZuZhi2, Unigene86206\_JingChaoZuZhi2, Unigene86278\_JingChaoZuZhi2, Unigene86456\_JingChaoZuZhi2, Unigene86504\_JingChaoZuZhi2, Unigene86526\_JingChaoZuZhi2, Unigene86806\_JingChaoZuZhi2, Unigene86945\_JingChaoZuZhi2, Unigene876\_JingChaoZuZhi2, Unigene88544\_JingChaoZuZhi2, Unigene88719\_JingChaoZuZhi2, Unigene88748\_JingChaoZuZhi2, Unigene89093\_JingChaoZuZhi2, Unigene89161\_JingChaoZuZhi2, Unigene89310\_JingChaoZuZhi2, Unigene89347\_JingChaoZuZhi2, Unigene89774\_JingChaoZuZhi2, Unigene91119\_JingChaoZuZhi2, Unigene91201\_JingChaoZuZhi2, Unigene91612\_JingChaoZuZhi2, Unigene91689\_JingChaoZuZhi2, Unigene91958\_JingChaoZuZhi2, Unigene92112\_JingChaoZuZhi2, Unigene92651\_JingChaoZuZhi2, Unigene92715\_JingChaoZuZhi2, Unigene92983\_JingChaoZuZhi2, Unigene93067\_JingChaoZuZhi2, Unigene93195\_JingChaoZuZhi2, Unigene93409\_JingChaoZuZhi2, Unigene93541\_JingChaoZuZhi2, Unigene93597\_JingChaoZuZhi2, Unigene93807\_JingChaoZuZhi2, Unigene94149\_JingChaoZuZhi2, Unigene94242\_JingChaoZuZhi2, Unigene94272\_JingChaoZuZhi2, Unigene9428\_JingChaoZuZhi2, Unigene94456\_JingChaoZuZhi2, Unigene94685\_JingChaoZuZhi2, Unigene94711\_JingChaoZuZhi2, Unigene94829\_JingChaoZuZhi2, Unigene94894\_JingChaoZuZhi2, Unigene95016\_JingChaoZuZhi2, Unigene95098\_JingChaoZuZhi2, Unigene95295\_JingChaoZuZhi2, Unigene95456\_JingChaoZuZhi2, Unigene95568\_JingChaoZuZhi2, Unigene95791\_JingChaoZuZhi2, Unigene96124\_JingChaoZuZhi2, Unigene96234\_JingChaoZuZhi2, Unigene96281\_JingChaoZuZhi2, Unigene96294\_JingChaoZuZhi2, Unigene9645\_JingChaoZuZhi2, Unigene9902\_JingChaoZuZhi2 |
| 58 | VEGF signaling pathway | Unigene1001\_JingChaoZuZhi2, Unigene10290\_JingChaoZuZhi2, Unigene10318\_JingChaoZuZhi2, Unigene10637\_JingChaoZuZhi2, Unigene11055\_JingChaoZuZhi2, Unigene1109\_JingChaoZuZhi2, Unigene11287\_JingChaoZuZhi2, Unigene11961\_JingChaoZuZhi2, Unigene12171\_JingChaoZuZhi2, Unigene12214\_JingChaoZuZhi2, Unigene12260\_JingChaoZuZhi2, Unigene12595\_JingChaoZuZhi2, Unigene1301\_JingChaoZuZhi2, Unigene13420\_JingChaoZuZhi2, Unigene13510\_JingChaoZuZhi2, Unigene13588\_JingChaoZuZhi2, Unigene13869\_JingChaoZuZhi2, Unigene14317\_JingChaoZuZhi2, Unigene14357\_JingChaoZuZhi2, Unigene14370\_JingChaoZuZhi2, Unigene14401\_JingChaoZuZhi2, Unigene14476\_JingChaoZuZhi2, Unigene14488\_JingChaoZuZhi2, Unigene14912\_JingChaoZuZhi2, Unigene14973\_JingChaoZuZhi2, Unigene14990\_JingChaoZuZhi2, Unigene15104\_JingChaoZuZhi2, Unigene15228\_JingChaoZuZhi2, Unigene15425\_JingChaoZuZhi2, Unigene15942\_JingChaoZuZhi2, Unigene16039\_JingChaoZuZhi2, Unigene1609\_JingChaoZuZhi2, Unigene16116\_JingChaoZuZhi2, Unigene16154\_JingChaoZuZhi2, Unigene16521\_JingChaoZuZhi2, Unigene16589\_JingChaoZuZhi2, Unigene16933\_JingChaoZuZhi2, Unigene17136\_JingChaoZuZhi2, Unigene17168\_JingChaoZuZhi2, Unigene17247\_JingChaoZuZhi2, Unigene17506\_JingChaoZuZhi2, Unigene17710\_JingChaoZuZhi2, Unigene17821\_JingChaoZuZhi2, Unigene18464\_JingChaoZuZhi2, Unigene18672\_JingChaoZuZhi2, Unigene18868\_JingChaoZuZhi2, Unigene18904\_JingChaoZuZhi2, Unigene19021\_JingChaoZuZhi2, Unigene19036\_JingChaoZuZhi2, Unigene19046\_JingChaoZuZhi2, Unigene1942\_JingChaoZuZhi2, Unigene19531\_JingChaoZuZhi2, Unigene19904\_JingChaoZuZhi2, Unigene1993\_JingChaoZuZhi2, Unigene20120\_JingChaoZuZhi2, Unigene20256\_JingChaoZuZhi2, Unigene20305\_JingChaoZuZhi2, Unigene20411\_JingChaoZuZhi2, Unigene20649\_JingChaoZuZhi2, Unigene20794\_JingChaoZuZhi2, Unigene21144\_JingChaoZuZhi2, Unigene21246\_JingChaoZuZhi2, Unigene21295\_JingChaoZuZhi2, Unigene21348\_JingChaoZuZhi2, Unigene21407\_JingChaoZuZhi2, Unigene21536\_JingChaoZuZhi2, Unigene21578\_JingChaoZuZhi2, Unigene21605\_JingChaoZuZhi2, Unigene21737\_JingChaoZuZhi2, Unigene21973\_JingChaoZuZhi2, Unigene22102\_JingChaoZuZhi2, Unigene22386\_JingChaoZuZhi2, Unigene22648\_JingChaoZuZhi2, Unigene22779\_JingChaoZuZhi2, Unigene22934\_JingChaoZuZhi2, Unigene2456\_JingChaoZuZhi2, Unigene24841\_JingChaoZuZhi2, Unigene24881\_JingChaoZuZhi2, Unigene25966\_JingChaoZuZhi2, Unigene26183\_JingChaoZuZhi2, Unigene26730\_JingChaoZuZhi2, Unigene277\_JingChaoZuZhi2, Unigene2960\_JingChaoZuZhi2, Unigene3016\_JingChaoZuZhi2, Unigene3185\_JingChaoZuZhi2, Unigene31924\_JingChaoZuZhi2, Unigene35744\_JingChaoZuZhi2, Unigene38493\_JingChaoZuZhi2, Unigene3931\_JingChaoZuZhi2, Unigene39539\_JingChaoZuZhi2, Unigene39865\_JingChaoZuZhi2, Unigene40020\_JingChaoZuZhi2, Unigene40825\_JingChaoZuZhi2, Unigene42329\_JingChaoZuZhi2, Unigene42332\_JingChaoZuZhi2, Unigene43723\_JingChaoZuZhi2, Unigene45532\_JingChaoZuZhi2, Unigene45\_JingChaoZuZhi2, Unigene48368\_JingChaoZuZhi2, Unigene48607\_JingChaoZuZhi2, Unigene48751\_JingChaoZuZhi2, Unigene5312\_JingChaoZuZhi2, Unigene53524\_JingChaoZuZhi2, Unigene53917\_JingChaoZuZhi2, Unigene53958\_JingChaoZuZhi2, Unigene56089\_JingChaoZuZhi2, Unigene57488\_JingChaoZuZhi2, Unigene601\_JingChaoZuZhi2, Unigene6028\_JingChaoZuZhi2, Unigene61108\_JingChaoZuZhi2, Unigene61406\_JingChaoZuZhi2, Unigene64164\_JingChaoZuZhi2, Unigene6459\_JingChaoZuZhi2, Unigene64830\_JingChaoZuZhi2, Unigene67117\_JingChaoZuZhi2, Unigene67328\_JingChaoZuZhi2, Unigene6752\_JingChaoZuZhi2, Unigene67655\_JingChaoZuZhi2, Unigene68151\_JingChaoZuZhi2, Unigene70549\_JingChaoZuZhi2, Unigene70723\_JingChaoZuZhi2, Unigene72098\_JingChaoZuZhi2, Unigene722\_JingChaoZuZhi2, Unigene72397\_JingChaoZuZhi2, Unigene7267\_JingChaoZuZhi2, Unigene73826\_JingChaoZuZhi2, Unigene7417\_JingChaoZuZhi2, Unigene74505\_JingChaoZuZhi2, Unigene74594\_JingChaoZuZhi2, Unigene76182\_JingChaoZuZhi2, Unigene76575\_JingChaoZuZhi2, Unigene76917\_JingChaoZuZhi2, Unigene77061\_JingChaoZuZhi2, Unigene78508\_JingChaoZuZhi2, Unigene78909\_JingChaoZuZhi2, Unigene7939\_JingChaoZuZhi2, Unigene8003\_JingChaoZuZhi2, Unigene81086\_JingChaoZuZhi2, Unigene81687\_JingChaoZuZhi2, Unigene81904\_JingChaoZuZhi2, Unigene8191\_JingChaoZuZhi2, Unigene82219\_JingChaoZuZhi2, Unigene82662\_JingChaoZuZhi2, Unigene83308\_JingChaoZuZhi2, Unigene86206\_JingChaoZuZhi2, Unigene86526\_JingChaoZuZhi2, Unigene86640\_JingChaoZuZhi2, Unigene87332\_JingChaoZuZhi2, Unigene876\_JingChaoZuZhi2, Unigene88068\_JingChaoZuZhi2, Unigene88185\_JingChaoZuZhi2, Unigene88391\_JingChaoZuZhi2, Unigene88522\_JingChaoZuZhi2, Unigene8949\_JingChaoZuZhi2, Unigene90762\_JingChaoZuZhi2, Unigene91179\_JingChaoZuZhi2, Unigene92112\_JingChaoZuZhi2, Unigene92737\_JingChaoZuZhi2, Unigene92800\_JingChaoZuZhi2, Unigene93782\_JingChaoZuZhi2, Unigene93793\_JingChaoZuZhi2, Unigene93807\_JingChaoZuZhi2, Unigene9445\_JingChaoZuZhi2, Unigene94619\_JingChaoZuZhi2, Unigene9461\_JingChaoZuZhi2, Unigene94685\_JingChaoZuZhi2, Unigene94749\_JingChaoZuZhi2, Unigene95065\_JingChaoZuZhi2, Unigene95098\_JingChaoZuZhi2, Unigene95147\_JingChaoZuZhi2, Unigene95483\_JingChaoZuZhi2, Unigene95677\_JingChaoZuZhi2, Unigene95875\_JingChaoZuZhi2, Unigene96269\_JingChaoZuZhi2, Unigene96294\_JingChaoZuZhi2, Unigene9645\_JingChaoZuZhi2, Unigene9902\_JingChaoZuZhi2, Unigene9\_JingChaoZuZhi2 |
| 59 | Long-term potentiation | Unigene10031\_JingChaoZuZhi2, Unigene10218\_JingChaoZuZhi2, Unigene10318\_JingChaoZuZhi2, Unigene10392\_JingChaoZuZhi2, Unigene12073\_JingChaoZuZhi2, Unigene1247\_JingChaoZuZhi2, Unigene12634\_JingChaoZuZhi2, Unigene13584\_JingChaoZuZhi2, Unigene13894\_JingChaoZuZhi2, Unigene14360\_JingChaoZuZhi2, Unigene14370\_JingChaoZuZhi2, Unigene14458\_JingChaoZuZhi2, Unigene14707\_JingChaoZuZhi2, Unigene16169\_JingChaoZuZhi2, Unigene16468\_JingChaoZuZhi2, Unigene1651\_JingChaoZuZhi2, Unigene16566\_JingChaoZuZhi2, Unigene16958\_JingChaoZuZhi2, Unigene16991\_JingChaoZuZhi2, Unigene18307\_JingChaoZuZhi2, Unigene19238\_JingChaoZuZhi2, Unigene19624\_JingChaoZuZhi2, Unigene20052\_JingChaoZuZhi2, Unigene21166\_JingChaoZuZhi2, Unigene21246\_JingChaoZuZhi2, Unigene21513\_JingChaoZuZhi2, Unigene21578\_JingChaoZuZhi2, Unigene21651\_JingChaoZuZhi2, Unigene21737\_JingChaoZuZhi2, Unigene21809\_JingChaoZuZhi2, Unigene21815\_JingChaoZuZhi2, Unigene22251\_JingChaoZuZhi2, Unigene22509\_JingChaoZuZhi2, Unigene22580\_JingChaoZuZhi2, Unigene22673\_JingChaoZuZhi2, Unigene22708\_JingChaoZuZhi2, Unigene22779\_JingChaoZuZhi2, Unigene23874\_JingChaoZuZhi2, Unigene2465\_JingChaoZuZhi2, Unigene2473\_JingChaoZuZhi2, Unigene26730\_JingChaoZuZhi2, Unigene2720\_JingChaoZuZhi2, Unigene28398\_JingChaoZuZhi2, Unigene2960\_JingChaoZuZhi2, Unigene30593\_JingChaoZuZhi2, Unigene3131\_JingChaoZuZhi2, Unigene31886\_JingChaoZuZhi2, Unigene32139\_JingChaoZuZhi2, Unigene32827\_JingChaoZuZhi2, Unigene33536\_JingChaoZuZhi2, Unigene34768\_JingChaoZuZhi2, Unigene34929\_JingChaoZuZhi2, Unigene34989\_JingChaoZuZhi2, Unigene35755\_JingChaoZuZhi2, Unigene37009\_JingChaoZuZhi2, Unigene37143\_JingChaoZuZhi2, Unigene37739\_JingChaoZuZhi2, Unigene38900\_JingChaoZuZhi2, Unigene39259\_JingChaoZuZhi2, Unigene395\_JingChaoZuZhi2, Unigene39658\_JingChaoZuZhi2, Unigene39825\_JingChaoZuZhi2, Unigene4003\_JingChaoZuZhi2, Unigene40292\_JingChaoZuZhi2, Unigene41333\_JingChaoZuZhi2, Unigene416\_JingChaoZuZhi2, Unigene42566\_JingChaoZuZhi2, Unigene42575\_JingChaoZuZhi2, Unigene43059\_JingChaoZuZhi2, Unigene43582\_JingChaoZuZhi2, Unigene43699\_JingChaoZuZhi2, Unigene45\_JingChaoZuZhi2, Unigene46240\_JingChaoZuZhi2, Unigene473\_JingChaoZuZhi2, Unigene48647\_JingChaoZuZhi2, Unigene49294\_JingChaoZuZhi2, Unigene4957\_JingChaoZuZhi2, Unigene50026\_JingChaoZuZhi2, Unigene50503\_JingChaoZuZhi2, Unigene51663\_JingChaoZuZhi2, Unigene51991\_JingChaoZuZhi2, Unigene5253\_JingChaoZuZhi2, Unigene52694\_JingChaoZuZhi2, Unigene53103\_JingChaoZuZhi2, Unigene53310\_JingChaoZuZhi2, Unigene54367\_JingChaoZuZhi2, Unigene54449\_JingChaoZuZhi2, Unigene54994\_JingChaoZuZhi2, Unigene56567\_JingChaoZuZhi2, Unigene57037\_JingChaoZuZhi2, Unigene58275\_JingChaoZuZhi2, Unigene58474\_JingChaoZuZhi2, Unigene58527\_JingChaoZuZhi2, Unigene59373\_JingChaoZuZhi2, Unigene60081\_JingChaoZuZhi2, Unigene6028\_JingChaoZuZhi2, Unigene607\_JingChaoZuZhi2, Unigene61742\_JingChaoZuZhi2, Unigene63091\_JingChaoZuZhi2, Unigene63323\_JingChaoZuZhi2, Unigene63406\_JingChaoZuZhi2, Unigene63990\_JingChaoZuZhi2, Unigene63994\_JingChaoZuZhi2, Unigene64164\_JingChaoZuZhi2, Unigene64497\_JingChaoZuZhi2, Unigene6467\_JingChaoZuZhi2, Unigene64830\_JingChaoZuZhi2, Unigene65096\_JingChaoZuZhi2, Unigene65445\_JingChaoZuZhi2, Unigene66116\_JingChaoZuZhi2, Unigene66540\_JingChaoZuZhi2, Unigene70493\_JingChaoZuZhi2, Unigene70549\_JingChaoZuZhi2, Unigene70723\_JingChaoZuZhi2, Unigene7192\_JingChaoZuZhi2, Unigene72643\_JingChaoZuZhi2, Unigene7456\_JingChaoZuZhi2, Unigene74597\_JingChaoZuZhi2, Unigene74609\_JingChaoZuZhi2, Unigene75235\_JingChaoZuZhi2, Unigene77149\_JingChaoZuZhi2, Unigene77471\_JingChaoZuZhi2, Unigene78078\_JingChaoZuZhi2, Unigene78235\_JingChaoZuZhi2, Unigene78522\_JingChaoZuZhi2, Unigene78587\_JingChaoZuZhi2, Unigene79388\_JingChaoZuZhi2, Unigene79681\_JingChaoZuZhi2, Unigene80123\_JingChaoZuZhi2, Unigene80251\_JingChaoZuZhi2, Unigene81716\_JingChaoZuZhi2, Unigene81780\_JingChaoZuZhi2, Unigene8191\_JingChaoZuZhi2, Unigene82219\_JingChaoZuZhi2, Unigene83188\_JingChaoZuZhi2, Unigene83663\_JingChaoZuZhi2, Unigene84247\_JingChaoZuZhi2, Unigene8507\_JingChaoZuZhi2, Unigene86119\_JingChaoZuZhi2, Unigene86504\_JingChaoZuZhi2, Unigene86536\_JingChaoZuZhi2, Unigene86661\_JingChaoZuZhi2, Unigene87332\_JingChaoZuZhi2, Unigene87607\_JingChaoZuZhi2, Unigene876\_JingChaoZuZhi2, Unigene88385\_JingChaoZuZhi2, Unigene88594\_JingChaoZuZhi2, Unigene88724\_JingChaoZuZhi2, Unigene88773\_JingChaoZuZhi2, Unigene89093\_JingChaoZuZhi2, Unigene89147\_JingChaoZuZhi2, Unigene89161\_JingChaoZuZhi2, Unigene89385\_JingChaoZuZhi2, Unigene89807\_JingChaoZuZhi2, Unigene91119\_JingChaoZuZhi2, Unigene91527\_JingChaoZuZhi2, Unigene91631\_JingChaoZuZhi2, Unigene91739\_JingChaoZuZhi2, Unigene91924\_JingChaoZuZhi2, Unigene92119\_JingChaoZuZhi2, Unigene92715\_JingChaoZuZhi2, Unigene93012\_JingChaoZuZhi2, Unigene93195\_JingChaoZuZhi2, Unigene93588\_JingChaoZuZhi2, Unigene93597\_JingChaoZuZhi2, Unigene93714\_JingChaoZuZhi2, Unigene94242\_JingChaoZuZhi2, Unigene9428\_JingChaoZuZhi2, Unigene94711\_JingChaoZuZhi2, Unigene94749\_JingChaoZuZhi2, Unigene95359\_JingChaoZuZhi2, Unigene95568\_JingChaoZuZhi2, Unigene95677\_JingChaoZuZhi2, Unigene96124\_JingChaoZuZhi2, Unigene96262\_JingChaoZuZhi2, Unigene9654\_JingChaoZuZhi2, Unigene9812\_JingChaoZuZhi2, Unigene9902\_JingChaoZuZhi2 |
| 60 | Parkinson's disease | Unigene10000\_JingChaoZuZhi2, Unigene1082\_JingChaoZuZhi2, Unigene11372\_JingChaoZuZhi2, Unigene11963\_JingChaoZuZhi2, Unigene12648\_JingChaoZuZhi2, Unigene13346\_JingChaoZuZhi2, Unigene13803\_JingChaoZuZhi2, Unigene14522\_JingChaoZuZhi2, Unigene14871\_JingChaoZuZhi2, Unigene15555\_JingChaoZuZhi2, Unigene15867\_JingChaoZuZhi2, Unigene16188\_JingChaoZuZhi2, Unigene16317\_JingChaoZuZhi2, Unigene16420\_JingChaoZuZhi2, Unigene16752\_JingChaoZuZhi2, Unigene16834\_JingChaoZuZhi2, Unigene17015\_JingChaoZuZhi2, Unigene17099\_JingChaoZuZhi2, Unigene17151\_JingChaoZuZhi2, Unigene18326\_JingChaoZuZhi2, Unigene18475\_JingChaoZuZhi2, Unigene19006\_JingChaoZuZhi2, Unigene19256\_JingChaoZuZhi2, Unigene19634\_JingChaoZuZhi2, Unigene19710\_JingChaoZuZhi2, Unigene20510\_JingChaoZuZhi2, Unigene20962\_JingChaoZuZhi2, Unigene20992\_JingChaoZuZhi2, Unigene21024\_JingChaoZuZhi2, Unigene2132\_JingChaoZuZhi2, Unigene21917\_JingChaoZuZhi2, Unigene22190\_JingChaoZuZhi2, Unigene22820\_JingChaoZuZhi2, Unigene26596\_JingChaoZuZhi2, Unigene2899\_JingChaoZuZhi2, Unigene34912\_JingChaoZuZhi2, Unigene35298\_JingChaoZuZhi2, Unigene35366\_JingChaoZuZhi2, Unigene36230\_JingChaoZuZhi2, Unigene36634\_JingChaoZuZhi2, Unigene39907\_JingChaoZuZhi2, Unigene40865\_JingChaoZuZhi2, Unigene4111\_JingChaoZuZhi2, Unigene41683\_JingChaoZuZhi2, Unigene41982\_JingChaoZuZhi2, Unigene44074\_JingChaoZuZhi2, Unigene44078\_JingChaoZuZhi2, Unigene4413\_JingChaoZuZhi2, Unigene4539\_JingChaoZuZhi2, Unigene46297\_JingChaoZuZhi2, Unigene48095\_JingChaoZuZhi2, Unigene49345\_JingChaoZuZhi2, Unigene49987\_JingChaoZuZhi2, Unigene51415\_JingChaoZuZhi2, Unigene52036\_JingChaoZuZhi2, Unigene52383\_JingChaoZuZhi2, Unigene54236\_JingChaoZuZhi2, Unigene54333\_JingChaoZuZhi2, Unigene55303\_JingChaoZuZhi2, Unigene55617\_JingChaoZuZhi2, Unigene55700\_JingChaoZuZhi2, Unigene55745\_JingChaoZuZhi2, Unigene56858\_JingChaoZuZhi2, Unigene57878\_JingChaoZuZhi2, Unigene58502\_JingChaoZuZhi2, Unigene59270\_JingChaoZuZhi2, Unigene6166\_JingChaoZuZhi2, Unigene63175\_JingChaoZuZhi2, Unigene63341\_JingChaoZuZhi2, Unigene63935\_JingChaoZuZhi2, Unigene64058\_JingChaoZuZhi2, Unigene65721\_JingChaoZuZhi2, Unigene65807\_JingChaoZuZhi2, Unigene65951\_JingChaoZuZhi2, Unigene67337\_JingChaoZuZhi2, Unigene68298\_JingChaoZuZhi2, Unigene68599\_JingChaoZuZhi2, Unigene68698\_JingChaoZuZhi2, Unigene68832\_JingChaoZuZhi2, Unigene68962\_JingChaoZuZhi2, Unigene69047\_JingChaoZuZhi2, Unigene70701\_JingChaoZuZhi2, Unigene7082\_JingChaoZuZhi2, Unigene7176\_JingChaoZuZhi2, Unigene72813\_JingChaoZuZhi2, Unigene73543\_JingChaoZuZhi2, Unigene73674\_JingChaoZuZhi2, Unigene74274\_JingChaoZuZhi2, Unigene76057\_JingChaoZuZhi2, Unigene76647\_JingChaoZuZhi2, Unigene76848\_JingChaoZuZhi2, Unigene76896\_JingChaoZuZhi2, Unigene77259\_JingChaoZuZhi2, Unigene77631\_JingChaoZuZhi2, Unigene78004\_JingChaoZuZhi2, Unigene78435\_JingChaoZuZhi2, Unigene78866\_JingChaoZuZhi2, Unigene78998\_JingChaoZuZhi2, Unigene79210\_JingChaoZuZhi2, Unigene7947\_JingChaoZuZhi2, Unigene79523\_JingChaoZuZhi2, Unigene79892\_JingChaoZuZhi2, Unigene80960\_JingChaoZuZhi2, Unigene81043\_JingChaoZuZhi2, Unigene81068\_JingChaoZuZhi2, Unigene81706\_JingChaoZuZhi2, Unigene81996\_JingChaoZuZhi2, Unigene82186\_JingChaoZuZhi2, Unigene83101\_JingChaoZuZhi2, Unigene8310\_JingChaoZuZhi2, Unigene83409\_JingChaoZuZhi2, Unigene83448\_JingChaoZuZhi2, Unigene84181\_JingChaoZuZhi2, Unigene84592\_JingChaoZuZhi2, Unigene86035\_JingChaoZuZhi2, Unigene86367\_JingChaoZuZhi2, Unigene86528\_JingChaoZuZhi2, Unigene86582\_JingChaoZuZhi2, Unigene86821\_JingChaoZuZhi2, Unigene87015\_JingChaoZuZhi2, Unigene87349\_JingChaoZuZhi2, Unigene87389\_JingChaoZuZhi2, Unigene87433\_JingChaoZuZhi2, Unigene87796\_JingChaoZuZhi2, Unigene87948\_JingChaoZuZhi2, Unigene88135\_JingChaoZuZhi2, Unigene88312\_JingChaoZuZhi2, Unigene8837\_JingChaoZuZhi2, Unigene88393\_JingChaoZuZhi2, Unigene88933\_JingChaoZuZhi2, Unigene89210\_JingChaoZuZhi2, Unigene89245\_JingChaoZuZhi2, Unigene89265\_JingChaoZuZhi2, Unigene89653\_JingChaoZuZhi2, Unigene89670\_JingChaoZuZhi2, Unigene90077\_JingChaoZuZhi2, Unigene90348\_JingChaoZuZhi2, Unigene90358\_JingChaoZuZhi2, Unigene90535\_JingChaoZuZhi2, Unigene90671\_JingChaoZuZhi2, Unigene91051\_JingChaoZuZhi2, Unigene91081\_JingChaoZuZhi2, Unigene91580\_JingChaoZuZhi2, Unigene91855\_JingChaoZuZhi2, Unigene91973\_JingChaoZuZhi2, Unigene92115\_JingChaoZuZhi2, Unigene92187\_JingChaoZuZhi2, Unigene92312\_JingChaoZuZhi2, Unigene92321\_JingChaoZuZhi2, Unigene92429\_JingChaoZuZhi2, Unigene92539\_JingChaoZuZhi2, Unigene9256\_JingChaoZuZhi2, Unigene92659\_JingChaoZuZhi2, Unigene92778\_JingChaoZuZhi2, Unigene93072\_JingChaoZuZhi2, Unigene93095\_JingChaoZuZhi2, Unigene93265\_JingChaoZuZhi2, Unigene93406\_JingChaoZuZhi2, Unigene93604\_JingChaoZuZhi2, Unigene93701\_JingChaoZuZhi2, Unigene93940\_JingChaoZuZhi2, Unigene94021\_JingChaoZuZhi2, Unigene94029\_JingChaoZuZhi2, Unigene94276\_JingChaoZuZhi2, Unigene94738\_JingChaoZuZhi2, Unigene94892\_JingChaoZuZhi2, Unigene95041\_JingChaoZuZhi2, Unigene95069\_JingChaoZuZhi2, Unigene95109\_JingChaoZuZhi2, Unigene95159\_JingChaoZuZhi2, Unigene95531\_JingChaoZuZhi2, Unigene95742\_JingChaoZuZhi2, Unigene95994\_JingChaoZuZhi2, Unigene96190\_JingChaoZuZhi2, Unigene96275\_JingChaoZuZhi2, Unigene96302\_JingChaoZuZhi2 |
| 61 | Progesterone-mediated oocyte maturation | Unigene10125\_JingChaoZuZhi2, Unigene10300\_JingChaoZuZhi2, Unigene10309\_JingChaoZuZhi2, Unigene10599\_JingChaoZuZhi2, Unigene10847\_JingChaoZuZhi2, Unigene10878\_JingChaoZuZhi2, Unigene11000\_JingChaoZuZhi2, Unigene1157\_JingChaoZuZhi2, Unigene11621\_JingChaoZuZhi2, Unigene11718\_JingChaoZuZhi2, Unigene11726\_JingChaoZuZhi2, Unigene11906\_JingChaoZuZhi2, Unigene11951\_JingChaoZuZhi2, Unigene11961\_JingChaoZuZhi2, Unigene11996\_JingChaoZuZhi2, Unigene12577\_JingChaoZuZhi2, Unigene12633\_JingChaoZuZhi2, Unigene12938\_JingChaoZuZhi2, Unigene13857\_JingChaoZuZhi2, Unigene14425\_JingChaoZuZhi2, Unigene14567\_JingChaoZuZhi2, Unigene14912\_JingChaoZuZhi2, Unigene15118\_JingChaoZuZhi2, Unigene15151\_JingChaoZuZhi2, Unigene15228\_JingChaoZuZhi2, Unigene15446\_JingChaoZuZhi2, Unigene15469\_JingChaoZuZhi2, Unigene15993\_JingChaoZuZhi2, Unigene1648\_JingChaoZuZhi2, Unigene167\_JingChaoZuZhi2, Unigene17115\_JingChaoZuZhi2, Unigene17247\_JingChaoZuZhi2, Unigene18008\_JingChaoZuZhi2, Unigene18307\_JingChaoZuZhi2, Unigene18672\_JingChaoZuZhi2, Unigene19013\_JingChaoZuZhi2, Unigene19361\_JingChaoZuZhi2, Unigene19453\_JingChaoZuZhi2, Unigene19624\_JingChaoZuZhi2, Unigene19691\_JingChaoZuZhi2, Unigene20102\_JingChaoZuZhi2, Unigene20120\_JingChaoZuZhi2, Unigene20305\_JingChaoZuZhi2, Unigene20455\_JingChaoZuZhi2, Unigene21513\_JingChaoZuZhi2, Unigene21815\_JingChaoZuZhi2, Unigene21891\_JingChaoZuZhi2, Unigene22290\_JingChaoZuZhi2, Unigene22299\_JingChaoZuZhi2, Unigene22402\_JingChaoZuZhi2, Unigene22435\_JingChaoZuZhi2, Unigene22509\_JingChaoZuZhi2, Unigene22622\_JingChaoZuZhi2, Unigene22736\_JingChaoZuZhi2, Unigene2456\_JingChaoZuZhi2, Unigene2691\_JingChaoZuZhi2, Unigene2692\_JingChaoZuZhi2, Unigene3122\_JingChaoZuZhi2, Unigene32208\_JingChaoZuZhi2, Unigene36424\_JingChaoZuZhi2, Unigene36737\_JingChaoZuZhi2, Unigene36751\_JingChaoZuZhi2, Unigene37741\_JingChaoZuZhi2, Unigene37803\_JingChaoZuZhi2, Unigene38741\_JingChaoZuZhi2, Unigene3931\_JingChaoZuZhi2, Unigene40335\_JingChaoZuZhi2, Unigene4294\_JingChaoZuZhi2, Unigene43322\_JingChaoZuZhi2, Unigene46937\_JingChaoZuZhi2, Unigene473\_JingChaoZuZhi2, Unigene47450\_JingChaoZuZhi2, Unigene48223\_JingChaoZuZhi2, Unigene48751\_JingChaoZuZhi2, Unigene49203\_JingChaoZuZhi2, Unigene5003\_JingChaoZuZhi2, Unigene50611\_JingChaoZuZhi2, Unigene51612\_JingChaoZuZhi2, Unigene51991\_JingChaoZuZhi2, Unigene5253\_JingChaoZuZhi2, Unigene5312\_JingChaoZuZhi2, Unigene53935\_JingChaoZuZhi2, Unigene53953\_JingChaoZuZhi2, Unigene54012\_JingChaoZuZhi2, Unigene5608\_JingChaoZuZhi2, Unigene57185\_JingChaoZuZhi2, Unigene57488\_JingChaoZuZhi2, Unigene58582\_JingChaoZuZhi2, Unigene58767\_JingChaoZuZhi2, Unigene59102\_JingChaoZuZhi2, Unigene59580\_JingChaoZuZhi2, Unigene6028\_JingChaoZuZhi2, Unigene61590\_JingChaoZuZhi2, Unigene6393\_JingChaoZuZhi2, Unigene63966\_JingChaoZuZhi2, Unigene6439\_JingChaoZuZhi2, Unigene64583\_JingChaoZuZhi2, Unigene6459\_JingChaoZuZhi2, Unigene64671\_JingChaoZuZhi2, Unigene65337\_JingChaoZuZhi2, Unigene66073\_JingChaoZuZhi2, Unigene66743\_JingChaoZuZhi2, Unigene6700\_JingChaoZuZhi2, Unigene6772\_JingChaoZuZhi2, Unigene67909\_JingChaoZuZhi2, Unigene69203\_JingChaoZuZhi2, Unigene70006\_JingChaoZuZhi2, Unigene7002\_JingChaoZuZhi2, Unigene70549\_JingChaoZuZhi2, Unigene71365\_JingChaoZuZhi2, Unigene7139\_JingChaoZuZhi2, Unigene71729\_JingChaoZuZhi2, Unigene72926\_JingChaoZuZhi2, Unigene73233\_JingChaoZuZhi2, Unigene73826\_JingChaoZuZhi2, Unigene7390\_JingChaoZuZhi2, Unigene7456\_JingChaoZuZhi2, Unigene75235\_JingChaoZuZhi2, Unigene7557\_JingChaoZuZhi2, Unigene76392\_JingChaoZuZhi2, Unigene77209\_JingChaoZuZhi2, Unigene78235\_JingChaoZuZhi2, Unigene78441\_JingChaoZuZhi2, Unigene80385\_JingChaoZuZhi2, Unigene8142\_JingChaoZuZhi2, Unigene8191\_JingChaoZuZhi2, Unigene81952\_JingChaoZuZhi2, Unigene83017\_JingChaoZuZhi2, Unigene84830\_JingChaoZuZhi2, Unigene84844\_JingChaoZuZhi2, Unigene84934\_JingChaoZuZhi2, Unigene85030\_JingChaoZuZhi2, Unigene85038\_JingChaoZuZhi2, Unigene86206\_JingChaoZuZhi2, Unigene86526\_JingChaoZuZhi2, Unigene86633\_JingChaoZuZhi2, Unigene876\_JingChaoZuZhi2, Unigene87780\_JingChaoZuZhi2, Unigene88082\_JingChaoZuZhi2, Unigene88522\_JingChaoZuZhi2, Unigene89011\_JingChaoZuZhi2, Unigene89603\_JingChaoZuZhi2, Unigene89774\_JingChaoZuZhi2, Unigene89807\_JingChaoZuZhi2, Unigene90415\_JingChaoZuZhi2, Unigene90500\_JingChaoZuZhi2, Unigene90567\_JingChaoZuZhi2, Unigene91077\_JingChaoZuZhi2, Unigene91221\_JingChaoZuZhi2, Unigene91527\_JingChaoZuZhi2, Unigene91631\_JingChaoZuZhi2, Unigene91791\_JingChaoZuZhi2, Unigene91978\_JingChaoZuZhi2, Unigene92112\_JingChaoZuZhi2, Unigene92547\_JingChaoZuZhi2, Unigene93253\_JingChaoZuZhi2, Unigene93458\_JingChaoZuZhi2, Unigene93522\_JingChaoZuZhi2, Unigene93807\_JingChaoZuZhi2, Unigene93823\_JingChaoZuZhi2, Unigene94116\_JingChaoZuZhi2, Unigene94377\_JingChaoZuZhi2, Unigene94619\_JingChaoZuZhi2, Unigene94685\_JingChaoZuZhi2, Unigene94829\_JingChaoZuZhi2, Unigene95065\_JingChaoZuZhi2, Unigene95665\_JingChaoZuZhi2, Unigene95703\_JingChaoZuZhi2, Unigene95855\_JingChaoZuZhi2, Unigene96058\_JingChaoZuZhi2, Unigene96143\_JingChaoZuZhi2, Unigene96215\_JingChaoZuZhi2, Unigene96269\_JingChaoZuZhi2, Unigene96294\_JingChaoZuZhi2, Unigene96298\_JingChaoZuZhi2, Unigene9902\_JingChaoZuZhi2 |
| 62 | Gastric acid secretion | Unigene10047\_JingChaoZuZhi2, Unigene10259\_JingChaoZuZhi2, Unigene10318\_JingChaoZuZhi2, Unigene1060\_JingChaoZuZhi2, Unigene10\_JingChaoZuZhi2, Unigene11621\_JingChaoZuZhi2, Unigene1162\_JingChaoZuZhi2, Unigene11726\_JingChaoZuZhi2, Unigene12073\_JingChaoZuZhi2, Unigene12938\_JingChaoZuZhi2, Unigene13485\_JingChaoZuZhi2, Unigene13770\_JingChaoZuZhi2, Unigene14360\_JingChaoZuZhi2, Unigene14567\_JingChaoZuZhi2, Unigene15651\_JingChaoZuZhi2, Unigene16004\_JingChaoZuZhi2, Unigene16210\_JingChaoZuZhi2, Unigene16467\_JingChaoZuZhi2, Unigene16566\_JingChaoZuZhi2, Unigene16991\_JingChaoZuZhi2, Unigene17572\_JingChaoZuZhi2, Unigene18307\_JingChaoZuZhi2, Unigene18426\_JingChaoZuZhi2, Unigene18739\_JingChaoZuZhi2, Unigene18977\_JingChaoZuZhi2, Unigene20356\_JingChaoZuZhi2, Unigene20651\_JingChaoZuZhi2, Unigene21578\_JingChaoZuZhi2, Unigene21792\_JingChaoZuZhi2, Unigene21815\_JingChaoZuZhi2, Unigene21818\_JingChaoZuZhi2, Unigene21866\_JingChaoZuZhi2, Unigene22644\_JingChaoZuZhi2, Unigene22779\_JingChaoZuZhi2, Unigene24949\_JingChaoZuZhi2, Unigene2544\_JingChaoZuZhi2, Unigene26730\_JingChaoZuZhi2, Unigene26987\_JingChaoZuZhi2, Unigene28522\_JingChaoZuZhi2, Unigene2960\_JingChaoZuZhi2, Unigene3122\_JingChaoZuZhi2, Unigene31660\_JingChaoZuZhi2, Unigene31918\_JingChaoZuZhi2, Unigene32827\_JingChaoZuZhi2, Unigene34929\_JingChaoZuZhi2, Unigene35016\_JingChaoZuZhi2, Unigene3550\_JingChaoZuZhi2, Unigene39102\_JingChaoZuZhi2, Unigene39299\_JingChaoZuZhi2, Unigene39832\_JingChaoZuZhi2, Unigene39892\_JingChaoZuZhi2, Unigene40335\_JingChaoZuZhi2, Unigene40919\_JingChaoZuZhi2, Unigene41333\_JingChaoZuZhi2, Unigene41571\_JingChaoZuZhi2, Unigene42033\_JingChaoZuZhi2, Unigene42248\_JingChaoZuZhi2, Unigene43059\_JingChaoZuZhi2, Unigene44190\_JingChaoZuZhi2, Unigene48280\_JingChaoZuZhi2, Unigene49203\_JingChaoZuZhi2, Unigene49757\_JingChaoZuZhi2, Unigene51467\_JingChaoZuZhi2, Unigene51496\_JingChaoZuZhi2, Unigene51991\_JingChaoZuZhi2, Unigene5204\_JingChaoZuZhi2, Unigene52727\_JingChaoZuZhi2, Unigene5276\_JingChaoZuZhi2, Unigene53935\_JingChaoZuZhi2, Unigene54012\_JingChaoZuZhi2, Unigene54367\_JingChaoZuZhi2, Unigene54449\_JingChaoZuZhi2, Unigene5545\_JingChaoZuZhi2, Unigene55865\_JingChaoZuZhi2, Unigene56513\_JingChaoZuZhi2, Unigene57185\_JingChaoZuZhi2, Unigene5799\_JingChaoZuZhi2, Unigene58322\_JingChaoZuZhi2, Unigene59102\_JingChaoZuZhi2, Unigene59146\_JingChaoZuZhi2, Unigene61244\_JingChaoZuZhi2, Unigene61860\_JingChaoZuZhi2, Unigene62431\_JingChaoZuZhi2, Unigene63391\_JingChaoZuZhi2, Unigene6393\_JingChaoZuZhi2, Unigene63994\_JingChaoZuZhi2, Unigene64164\_JingChaoZuZhi2, Unigene64830\_JingChaoZuZhi2, Unigene65371\_JingChaoZuZhi2, Unigene65445\_JingChaoZuZhi2, Unigene66073\_JingChaoZuZhi2, Unigene6634\_JingChaoZuZhi2, Unigene66540\_JingChaoZuZhi2, Unigene67011\_JingChaoZuZhi2, Unigene68074\_JingChaoZuZhi2, Unigene69100\_JingChaoZuZhi2, Unigene69551\_JingChaoZuZhi2, Unigene6991\_JingChaoZuZhi2, Unigene70493\_JingChaoZuZhi2, Unigene70723\_JingChaoZuZhi2, Unigene71365\_JingChaoZuZhi2, Unigene71592\_JingChaoZuZhi2, Unigene71662\_JingChaoZuZhi2, Unigene72643\_JingChaoZuZhi2, Unigene73896\_JingChaoZuZhi2, Unigene7456\_JingChaoZuZhi2, Unigene75235\_JingChaoZuZhi2, Unigene77149\_JingChaoZuZhi2, Unigene77255\_JingChaoZuZhi2, Unigene78078\_JingChaoZuZhi2, Unigene78225\_JingChaoZuZhi2, Unigene78235\_JingChaoZuZhi2, Unigene78490\_JingChaoZuZhi2, Unigene78950\_JingChaoZuZhi2, Unigene79274\_JingChaoZuZhi2, Unigene8006\_JingChaoZuZhi2, Unigene80275\_JingChaoZuZhi2, Unigene81692\_JingChaoZuZhi2, Unigene81903\_JingChaoZuZhi2, Unigene8192\_JingChaoZuZhi2, Unigene82117\_JingChaoZuZhi2, Unigene82259\_JingChaoZuZhi2, Unigene82489\_JingChaoZuZhi2, Unigene83017\_JingChaoZuZhi2, Unigene83602\_JingChaoZuZhi2, Unigene83872\_JingChaoZuZhi2, Unigene84232\_JingChaoZuZhi2, Unigene84247\_JingChaoZuZhi2, Unigene8507\_JingChaoZuZhi2, Unigene85193\_JingChaoZuZhi2, Unigene87332\_JingChaoZuZhi2, Unigene87607\_JingChaoZuZhi2, Unigene87738\_JingChaoZuZhi2, Unigene88342\_JingChaoZuZhi2, Unigene88385\_JingChaoZuZhi2, Unigene88594\_JingChaoZuZhi2, Unigene88724\_JingChaoZuZhi2, Unigene88773\_JingChaoZuZhi2, Unigene89060\_JingChaoZuZhi2, Unigene89147\_JingChaoZuZhi2, Unigene89603\_JingChaoZuZhi2, Unigene89807\_JingChaoZuZhi2, Unigene9038\_JingChaoZuZhi2, Unigene91163\_JingChaoZuZhi2, Unigene91221\_JingChaoZuZhi2, Unigene91289\_JingChaoZuZhi2, Unigene91344\_JingChaoZuZhi2, Unigene91527\_JingChaoZuZhi2, Unigene91739\_JingChaoZuZhi2, Unigene91839\_JingChaoZuZhi2, Unigene91924\_JingChaoZuZhi2, Unigene92231\_JingChaoZuZhi2, Unigene9260\_JingChaoZuZhi2, Unigene92929\_JingChaoZuZhi2, Unigene93012\_JingChaoZuZhi2, Unigene93588\_JingChaoZuZhi2, Unigene93714\_JingChaoZuZhi2, Unigene93990\_JingChaoZuZhi2, Unigene94110\_JingChaoZuZhi2, Unigene94279\_JingChaoZuZhi2, Unigene9446\_JingChaoZuZhi2, Unigene95063\_JingChaoZuZhi2, Unigene951\_JingChaoZuZhi2, Unigene95677\_JingChaoZuZhi2, Unigene95776\_JingChaoZuZhi2, Unigene95822\_JingChaoZuZhi2, Unigene95907\_JingChaoZuZhi2, Unigene95916\_JingChaoZuZhi2, Unigene96206\_JingChaoZuZhi2, Unigene96262\_JingChaoZuZhi2, Unigene9654\_JingChaoZuZhi2, Unigene9739\_JingChaoZuZhi2 |
| 63 | Notch signaling pathway | Unigene10031\_JingChaoZuZhi2, Unigene10275\_JingChaoZuZhi2, Unigene10979\_JingChaoZuZhi2, Unigene11478\_JingChaoZuZhi2, Unigene11620\_JingChaoZuZhi2, Unigene12455\_JingChaoZuZhi2, Unigene12469\_JingChaoZuZhi2, Unigene12634\_JingChaoZuZhi2, Unigene12647\_JingChaoZuZhi2, Unigene12693\_JingChaoZuZhi2, Unigene12729\_JingChaoZuZhi2, Unigene12950\_JingChaoZuZhi2, Unigene13531\_JingChaoZuZhi2, Unigene14729\_JingChaoZuZhi2, Unigene15166\_JingChaoZuZhi2, Unigene15489\_JingChaoZuZhi2, Unigene15643\_JingChaoZuZhi2, Unigene15935\_JingChaoZuZhi2, Unigene16314\_JingChaoZuZhi2, Unigene16468\_JingChaoZuZhi2, Unigene16538\_JingChaoZuZhi2, Unigene16695\_JingChaoZuZhi2, Unigene16958\_JingChaoZuZhi2, Unigene17350\_JingChaoZuZhi2, Unigene18184\_JingChaoZuZhi2, Unigene18203\_JingChaoZuZhi2, Unigene18387\_JingChaoZuZhi2, Unigene18490\_JingChaoZuZhi2, Unigene18607\_JingChaoZuZhi2, Unigene19068\_JingChaoZuZhi2, Unigene19170\_JingChaoZuZhi2, Unigene19238\_JingChaoZuZhi2, Unigene19438\_JingChaoZuZhi2, Unigene19481\_JingChaoZuZhi2, Unigene19943\_JingChaoZuZhi2, Unigene20052\_JingChaoZuZhi2, Unigene20054\_JingChaoZuZhi2, Unigene20343\_JingChaoZuZhi2, Unigene21488\_JingChaoZuZhi2, Unigene21637\_JingChaoZuZhi2, Unigene21926\_JingChaoZuZhi2, Unigene22124\_JingChaoZuZhi2, Unigene22234\_JingChaoZuZhi2, Unigene22284\_JingChaoZuZhi2, Unigene22386\_JingChaoZuZhi2, Unigene22506\_JingChaoZuZhi2, Unigene22578\_JingChaoZuZhi2, Unigene22580\_JingChaoZuZhi2, Unigene22646\_JingChaoZuZhi2, Unigene22673\_JingChaoZuZhi2, Unigene22708\_JingChaoZuZhi2, Unigene2338\_JingChaoZuZhi2, Unigene2473\_JingChaoZuZhi2, Unigene252\_JingChaoZuZhi2, Unigene28870\_JingChaoZuZhi2, Unigene29734\_JingChaoZuZhi2, Unigene3308\_JingChaoZuZhi2, Unigene3414\_JingChaoZuZhi2, Unigene34853\_JingChaoZuZhi2, Unigene36411\_JingChaoZuZhi2, Unigene38409\_JingChaoZuZhi2, Unigene395\_JingChaoZuZhi2, Unigene39825\_JingChaoZuZhi2, Unigene42087\_JingChaoZuZhi2, Unigene43145\_JingChaoZuZhi2, Unigene432\_JingChaoZuZhi2, Unigene44141\_JingChaoZuZhi2, Unigene44435\_JingChaoZuZhi2, Unigene44516\_JingChaoZuZhi2, Unigene44927\_JingChaoZuZhi2, Unigene45443\_JingChaoZuZhi2, Unigene46240\_JingChaoZuZhi2, Unigene48769\_JingChaoZuZhi2, Unigene5021\_JingChaoZuZhi2, Unigene50573\_JingChaoZuZhi2, Unigene50934\_JingChaoZuZhi2, Unigene52166\_JingChaoZuZhi2, Unigene52452\_JingChaoZuZhi2, Unigene53020\_JingChaoZuZhi2, Unigene53836\_JingChaoZuZhi2, Unigene54428\_JingChaoZuZhi2, Unigene55677\_JingChaoZuZhi2, Unigene58474\_JingChaoZuZhi2, Unigene59604\_JingChaoZuZhi2, Unigene601\_JingChaoZuZhi2, Unigene60453\_JingChaoZuZhi2, Unigene60827\_JingChaoZuZhi2, Unigene61624\_JingChaoZuZhi2, Unigene62100\_JingChaoZuZhi2, Unigene62454\_JingChaoZuZhi2, Unigene63261\_JingChaoZuZhi2, Unigene63323\_JingChaoZuZhi2, Unigene63406\_JingChaoZuZhi2, Unigene6391\_JingChaoZuZhi2, Unigene6424\_JingChaoZuZhi2, Unigene6467\_JingChaoZuZhi2, Unigene64989\_JingChaoZuZhi2, Unigene65081\_JingChaoZuZhi2, Unigene67177\_JingChaoZuZhi2, Unigene67249\_JingChaoZuZhi2, Unigene67364\_JingChaoZuZhi2, Unigene67523\_JingChaoZuZhi2, Unigene67724\_JingChaoZuZhi2, Unigene68143\_JingChaoZuZhi2, Unigene69678\_JingChaoZuZhi2, Unigene7238\_JingChaoZuZhi2, Unigene73814\_JingChaoZuZhi2, Unigene74609\_JingChaoZuZhi2, Unigene7567\_JingChaoZuZhi2, Unigene76312\_JingChaoZuZhi2, Unigene77798\_JingChaoZuZhi2, Unigene77992\_JingChaoZuZhi2, Unigene78136\_JingChaoZuZhi2, Unigene78522\_JingChaoZuZhi2, Unigene78786\_JingChaoZuZhi2, Unigene78\_JingChaoZuZhi2, Unigene79443\_JingChaoZuZhi2, Unigene79581\_JingChaoZuZhi2, Unigene79704\_JingChaoZuZhi2, Unigene79835\_JingChaoZuZhi2, Unigene80123\_JingChaoZuZhi2, Unigene80385\_JingChaoZuZhi2, Unigene80465\_JingChaoZuZhi2, Unigene80772\_JingChaoZuZhi2, Unigene80776\_JingChaoZuZhi2, Unigene81553\_JingChaoZuZhi2, Unigene81716\_JingChaoZuZhi2, Unigene82769\_JingChaoZuZhi2, Unigene83188\_JingChaoZuZhi2, Unigene84070\_JingChaoZuZhi2, Unigene84129\_JingChaoZuZhi2, Unigene84377\_JingChaoZuZhi2, Unigene84608\_JingChaoZuZhi2, Unigene8523\_JingChaoZuZhi2, Unigene8609\_JingChaoZuZhi2, Unigene86119\_JingChaoZuZhi2, Unigene86234\_JingChaoZuZhi2, Unigene86504\_JingChaoZuZhi2, Unigene86837\_JingChaoZuZhi2, Unigene87488\_JingChaoZuZhi2, Unigene87540\_JingChaoZuZhi2, Unigene87640\_JingChaoZuZhi2, Unigene87654\_JingChaoZuZhi2, Unigene88050\_JingChaoZuZhi2, Unigene89093\_JingChaoZuZhi2, Unigene89161\_JingChaoZuZhi2, Unigene89755\_JingChaoZuZhi2, Unigene90664\_JingChaoZuZhi2, Unigene91028\_JingChaoZuZhi2, Unigene91100\_JingChaoZuZhi2, Unigene91119\_JingChaoZuZhi2, Unigene92076\_JingChaoZuZhi2, Unigene92402\_JingChaoZuZhi2, Unigene92714\_JingChaoZuZhi2, Unigene92715\_JingChaoZuZhi2, Unigene92977\_JingChaoZuZhi2, Unigene93056\_JingChaoZuZhi2, Unigene93195\_JingChaoZuZhi2, Unigene93307\_JingChaoZuZhi2, Unigene93679\_JingChaoZuZhi2, Unigene93852\_JingChaoZuZhi2, Unigene93916\_JingChaoZuZhi2, Unigene93921\_JingChaoZuZhi2, Unigene94242\_JingChaoZuZhi2, Unigene9428\_JingChaoZuZhi2, Unigene94507\_JingChaoZuZhi2, Unigene94546\_JingChaoZuZhi2, Unigene94711\_JingChaoZuZhi2, Unigene9511\_JingChaoZuZhi2, Unigene95431\_JingChaoZuZhi2, Unigene95568\_JingChaoZuZhi2, Unigene96124\_JingChaoZuZhi2 |
| 64 | Jak-STAT signaling pathway | Unigene10031\_JingChaoZuZhi2, Unigene10073\_JingChaoZuZhi2, Unigene10612\_JingChaoZuZhi2, Unigene11961\_JingChaoZuZhi2, Unigene12420\_JingChaoZuZhi2, Unigene12607\_JingChaoZuZhi2, Unigene12634\_JingChaoZuZhi2, Unigene12658\_JingChaoZuZhi2, Unigene12813\_JingChaoZuZhi2, Unigene1298\_JingChaoZuZhi2, Unigene13320\_JingChaoZuZhi2, Unigene13615\_JingChaoZuZhi2, Unigene14738\_JingChaoZuZhi2, Unigene14912\_JingChaoZuZhi2, Unigene15228\_JingChaoZuZhi2, Unigene15780\_JingChaoZuZhi2, Unigene16468\_JingChaoZuZhi2, Unigene16480\_JingChaoZuZhi2, Unigene16958\_JingChaoZuZhi2, Unigene17247\_JingChaoZuZhi2, Unigene17764\_JingChaoZuZhi2, Unigene18672\_JingChaoZuZhi2, Unigene18802\_JingChaoZuZhi2, Unigene18921\_JingChaoZuZhi2, Unigene18975\_JingChaoZuZhi2, Unigene18986\_JingChaoZuZhi2, Unigene19235\_JingChaoZuZhi2, Unigene19238\_JingChaoZuZhi2, Unigene19551\_JingChaoZuZhi2, Unigene20052\_JingChaoZuZhi2, Unigene21010\_JingChaoZuZhi2, Unigene21759\_JingChaoZuZhi2, Unigene22349\_JingChaoZuZhi2, Unigene22384\_JingChaoZuZhi2, Unigene22400\_JingChaoZuZhi2, Unigene22407\_JingChaoZuZhi2, Unigene22500\_JingChaoZuZhi2, Unigene22546\_JingChaoZuZhi2, Unigene22580\_JingChaoZuZhi2, Unigene22673\_JingChaoZuZhi2, Unigene22708\_JingChaoZuZhi2, Unigene22892\_JingChaoZuZhi2, Unigene24440\_JingChaoZuZhi2, Unigene2456\_JingChaoZuZhi2, Unigene2473\_JingChaoZuZhi2, Unigene27436\_JingChaoZuZhi2, Unigene27532\_JingChaoZuZhi2, Unigene27637\_JingChaoZuZhi2, Unigene28228\_JingChaoZuZhi2, Unigene28238\_JingChaoZuZhi2, Unigene28889\_JingChaoZuZhi2, Unigene29719\_JingChaoZuZhi2, Unigene30411\_JingChaoZuZhi2, Unigene31255\_JingChaoZuZhi2, Unigene32002\_JingChaoZuZhi2, Unigene37116\_JingChaoZuZhi2, Unigene37748\_JingChaoZuZhi2, Unigene3931\_JingChaoZuZhi2, Unigene395\_JingChaoZuZhi2, Unigene39825\_JingChaoZuZhi2, Unigene39829\_JingChaoZuZhi2, Unigene40032\_JingChaoZuZhi2, Unigene41454\_JingChaoZuZhi2, Unigene4186\_JingChaoZuZhi2, Unigene4223\_JingChaoZuZhi2, Unigene42346\_JingChaoZuZhi2, Unigene42610\_JingChaoZuZhi2, Unigene44228\_JingChaoZuZhi2, Unigene46114\_JingChaoZuZhi2, Unigene46240\_JingChaoZuZhi2, Unigene47213\_JingChaoZuZhi2, Unigene47548\_JingChaoZuZhi2, Unigene4783\_JingChaoZuZhi2, Unigene4838\_JingChaoZuZhi2, Unigene48751\_JingChaoZuZhi2, Unigene50055\_JingChaoZuZhi2, Unigene51012\_JingChaoZuZhi2, Unigene5165\_JingChaoZuZhi2, Unigene5240\_JingChaoZuZhi2, Unigene5306\_JingChaoZuZhi2, Unigene53232\_JingChaoZuZhi2, Unigene54363\_JingChaoZuZhi2, Unigene56284\_JingChaoZuZhi2, Unigene5638\_JingChaoZuZhi2, Unigene57488\_JingChaoZuZhi2, Unigene58354\_JingChaoZuZhi2, Unigene58474\_JingChaoZuZhi2, Unigene59086\_JingChaoZuZhi2, Unigene59535\_JingChaoZuZhi2, Unigene60729\_JingChaoZuZhi2, Unigene60734\_JingChaoZuZhi2, Unigene61189\_JingChaoZuZhi2, Unigene6159\_JingChaoZuZhi2, Unigene61955\_JingChaoZuZhi2, Unigene6262\_JingChaoZuZhi2, Unigene63323\_JingChaoZuZhi2, Unigene63406\_JingChaoZuZhi2, Unigene6467\_JingChaoZuZhi2, Unigene6493\_JingChaoZuZhi2, Unigene652\_JingChaoZuZhi2, Unigene65933\_JingChaoZuZhi2, Unigene66146\_JingChaoZuZhi2, Unigene66696\_JingChaoZuZhi2, Unigene67320\_JingChaoZuZhi2, Unigene67589\_JingChaoZuZhi2, Unigene67793\_JingChaoZuZhi2, Unigene6785\_JingChaoZuZhi2, Unigene70087\_JingChaoZuZhi2, Unigene72194\_JingChaoZuZhi2, Unigene72629\_JingChaoZuZhi2, Unigene74609\_JingChaoZuZhi2, Unigene75129\_JingChaoZuZhi2, Unigene7549\_JingChaoZuZhi2, Unigene76809\_JingChaoZuZhi2, Unigene77114\_JingChaoZuZhi2, Unigene78522\_JingChaoZuZhi2, Unigene79081\_JingChaoZuZhi2, Unigene79577\_JingChaoZuZhi2, Unigene79860\_JingChaoZuZhi2, Unigene79911\_JingChaoZuZhi2, Unigene80123\_JingChaoZuZhi2, Unigene80583\_JingChaoZuZhi2, Unigene80608\_JingChaoZuZhi2, Unigene81716\_JingChaoZuZhi2, Unigene81725\_JingChaoZuZhi2, Unigene8193\_JingChaoZuZhi2, Unigene83188\_JingChaoZuZhi2, Unigene83300\_JingChaoZuZhi2, Unigene83458\_JingChaoZuZhi2, Unigene84458\_JingChaoZuZhi2, Unigene84599\_JingChaoZuZhi2, Unigene84862\_JingChaoZuZhi2, Unigene85574\_JingChaoZuZhi2, Unigene86110\_JingChaoZuZhi2, Unigene86119\_JingChaoZuZhi2, Unigene86206\_JingChaoZuZhi2, Unigene86504\_JingChaoZuZhi2, Unigene86526\_JingChaoZuZhi2, Unigene89093\_JingChaoZuZhi2, Unigene89161\_JingChaoZuZhi2, Unigene89621\_JingChaoZuZhi2, Unigene90005\_JingChaoZuZhi2, Unigene90588\_JingChaoZuZhi2, Unigene90979\_JingChaoZuZhi2, Unigene91119\_JingChaoZuZhi2, Unigene91328\_JingChaoZuZhi2, Unigene91567\_JingChaoZuZhi2, Unigene91612\_JingChaoZuZhi2, Unigene91958\_JingChaoZuZhi2, Unigene9204\_JingChaoZuZhi2, Unigene92112\_JingChaoZuZhi2, Unigene92135\_JingChaoZuZhi2, Unigene92465\_JingChaoZuZhi2, Unigene92715\_JingChaoZuZhi2, Unigene9293\_JingChaoZuZhi2, Unigene92942\_JingChaoZuZhi2, Unigene93195\_JingChaoZuZhi2, Unigene93807\_JingChaoZuZhi2, Unigene9404\_JingChaoZuZhi2, Unigene94149\_JingChaoZuZhi2, Unigene94242\_JingChaoZuZhi2, Unigene9428\_JingChaoZuZhi2, Unigene94456\_JingChaoZuZhi2, Unigene94685\_JingChaoZuZhi2, Unigene94711\_JingChaoZuZhi2, Unigene95456\_JingChaoZuZhi2, Unigene95568\_JingChaoZuZhi2, Unigene96018\_JingChaoZuZhi2, Unigene96124\_JingChaoZuZhi2, Unigene96294\_JingChaoZuZhi2, Unigene9630\_JingChaoZuZhi2 |
| 65 | Oocyte meiosis | Unigene10125\_JingChaoZuZhi2, Unigene10300\_JingChaoZuZhi2, Unigene10309\_JingChaoZuZhi2, Unigene10599\_JingChaoZuZhi2, Unigene10878\_JingChaoZuZhi2, Unigene10932\_JingChaoZuZhi2, Unigene11000\_JingChaoZuZhi2, Unigene11726\_JingChaoZuZhi2, Unigene11906\_JingChaoZuZhi2, Unigene11996\_JingChaoZuZhi2, Unigene12073\_JingChaoZuZhi2, Unigene12161\_JingChaoZuZhi2, Unigene12577\_JingChaoZuZhi2, Unigene12938\_JingChaoZuZhi2, Unigene13716\_JingChaoZuZhi2, Unigene13894\_JingChaoZuZhi2, Unigene14370\_JingChaoZuZhi2, Unigene14390\_JingChaoZuZhi2, Unigene14425\_JingChaoZuZhi2, Unigene14567\_JingChaoZuZhi2, Unigene15118\_JingChaoZuZhi2, Unigene15469\_JingChaoZuZhi2, Unigene15501\_JingChaoZuZhi2, Unigene15993\_JingChaoZuZhi2, Unigene1648\_JingChaoZuZhi2, Unigene16566\_JingChaoZuZhi2, Unigene17115\_JingChaoZuZhi2, Unigene18008\_JingChaoZuZhi2, Unigene18139\_JingChaoZuZhi2, Unigene18307\_JingChaoZuZhi2, Unigene19092\_JingChaoZuZhi2, Unigene19361\_JingChaoZuZhi2, Unigene19453\_JingChaoZuZhi2, Unigene19624\_JingChaoZuZhi2, Unigene20120\_JingChaoZuZhi2, Unigene20455\_JingChaoZuZhi2, Unigene20489\_JingChaoZuZhi2, Unigene21246\_JingChaoZuZhi2, Unigene21737\_JingChaoZuZhi2, Unigene21815\_JingChaoZuZhi2, Unigene21891\_JingChaoZuZhi2, Unigene22290\_JingChaoZuZhi2, Unigene22299\_JingChaoZuZhi2, Unigene22375\_JingChaoZuZhi2, Unigene22435\_JingChaoZuZhi2, Unigene22509\_JingChaoZuZhi2, Unigene22622\_JingChaoZuZhi2, Unigene22661\_JingChaoZuZhi2, Unigene22680\_JingChaoZuZhi2, Unigene22736\_JingChaoZuZhi2, Unigene22787\_JingChaoZuZhi2, Unigene22889\_JingChaoZuZhi2, Unigene2691\_JingChaoZuZhi2, Unigene2692\_JingChaoZuZhi2, Unigene3122\_JingChaoZuZhi2, Unigene32208\_JingChaoZuZhi2, Unigene35730\_JingChaoZuZhi2, Unigene39072\_JingChaoZuZhi2, Unigene40335\_JingChaoZuZhi2, Unigene43322\_JingChaoZuZhi2, Unigene45\_JingChaoZuZhi2, Unigene46682\_JingChaoZuZhi2, Unigene473\_JingChaoZuZhi2, Unigene49203\_JingChaoZuZhi2, Unigene5003\_JingChaoZuZhi2, Unigene51164\_JingChaoZuZhi2, Unigene51612\_JingChaoZuZhi2, Unigene51991\_JingChaoZuZhi2, Unigene51992\_JingChaoZuZhi2, Unigene5253\_JingChaoZuZhi2, Unigene53935\_JingChaoZuZhi2, Unigene54012\_JingChaoZuZhi2, Unigene54449\_JingChaoZuZhi2, Unigene5608\_JingChaoZuZhi2, Unigene57185\_JingChaoZuZhi2, Unigene58767\_JingChaoZuZhi2, Unigene59068\_JingChaoZuZhi2, Unigene59102\_JingChaoZuZhi2, Unigene60045\_JingChaoZuZhi2, Unigene6028\_JingChaoZuZhi2, Unigene63237\_JingChaoZuZhi2, Unigene6393\_JingChaoZuZhi2, Unigene63966\_JingChaoZuZhi2, Unigene63994\_JingChaoZuZhi2, Unigene6439\_JingChaoZuZhi2, Unigene64583\_JingChaoZuZhi2, Unigene6459\_JingChaoZuZhi2, Unigene64671\_JingChaoZuZhi2, Unigene66073\_JingChaoZuZhi2, Unigene66743\_JingChaoZuZhi2, Unigene6700\_JingChaoZuZhi2, Unigene6772\_JingChaoZuZhi2, Unigene67909\_JingChaoZuZhi2, Unigene70006\_JingChaoZuZhi2, Unigene7002\_JingChaoZuZhi2, Unigene70549\_JingChaoZuZhi2, Unigene71365\_JingChaoZuZhi2, Unigene71729\_JingChaoZuZhi2, Unigene72632\_JingChaoZuZhi2, Unigene72926\_JingChaoZuZhi2, Unigene73233\_JingChaoZuZhi2, Unigene7390\_JingChaoZuZhi2, Unigene7456\_JingChaoZuZhi2, Unigene75235\_JingChaoZuZhi2, Unigene7557\_JingChaoZuZhi2, Unigene76639\_JingChaoZuZhi2, Unigene78235\_JingChaoZuZhi2, Unigene78441\_JingChaoZuZhi2, Unigene79233\_JingChaoZuZhi2, Unigene79681\_JingChaoZuZhi2, Unigene80251\_JingChaoZuZhi2, Unigene80385\_JingChaoZuZhi2, Unigene8142\_JingChaoZuZhi2, Unigene81780\_JingChaoZuZhi2, Unigene8191\_JingChaoZuZhi2, Unigene81952\_JingChaoZuZhi2, Unigene82219\_JingChaoZuZhi2, Unigene82454\_JingChaoZuZhi2, Unigene83663\_JingChaoZuZhi2, Unigene84247\_JingChaoZuZhi2, Unigene84844\_JingChaoZuZhi2, Unigene84934\_JingChaoZuZhi2, Unigene8507\_JingChaoZuZhi2, Unigene86239\_JingChaoZuZhi2, Unigene86633\_JingChaoZuZhi2, Unigene88594\_JingChaoZuZhi2, Unigene88724\_JingChaoZuZhi2, Unigene89147\_JingChaoZuZhi2, Unigene89603\_JingChaoZuZhi2, Unigene89807\_JingChaoZuZhi2, Unigene9003\_JingChaoZuZhi2, Unigene90415\_JingChaoZuZhi2, Unigene90508\_JingChaoZuZhi2, Unigene90900\_JingChaoZuZhi2, Unigene91008\_JingChaoZuZhi2, Unigene91077\_JingChaoZuZhi2, Unigene91527\_JingChaoZuZhi2, Unigene91631\_JingChaoZuZhi2, Unigene91678\_JingChaoZuZhi2, Unigene91924\_JingChaoZuZhi2, Unigene93245\_JingChaoZuZhi2, Unigene93253\_JingChaoZuZhi2, Unigene93288\_JingChaoZuZhi2, Unigene93458\_JingChaoZuZhi2, Unigene93479\_JingChaoZuZhi2, Unigene93522\_JingChaoZuZhi2, Unigene93588\_JingChaoZuZhi2, Unigene93714\_JingChaoZuZhi2, Unigene93754\_JingChaoZuZhi2, Unigene93823\_JingChaoZuZhi2, Unigene94116\_JingChaoZuZhi2, Unigene94377\_JingChaoZuZhi2, Unigene94388\_JingChaoZuZhi2, Unigene94391\_JingChaoZuZhi2, Unigene9444\_JingChaoZuZhi2, Unigene945\_JingChaoZuZhi2, Unigene94619\_JingChaoZuZhi2, Unigene94749\_JingChaoZuZhi2, Unigene94829\_JingChaoZuZhi2, Unigene94846\_JingChaoZuZhi2, Unigene95204\_JingChaoZuZhi2, Unigene95359\_JingChaoZuZhi2, Unigene95373\_JingChaoZuZhi2, Unigene95596\_JingChaoZuZhi2, Unigene95703\_JingChaoZuZhi2, Unigene95768\_JingChaoZuZhi2, Unigene95793\_JingChaoZuZhi2, Unigene95898\_JingChaoZuZhi2, Unigene96043\_JingChaoZuZhi2, Unigene96082\_JingChaoZuZhi2, Unigene96298\_JingChaoZuZhi2 |
| 66 | Oxidative phosphorylation | Unigene1082\_JingChaoZuZhi2, Unigene11372\_JingChaoZuZhi2, Unigene11730\_JingChaoZuZhi2, Unigene11963\_JingChaoZuZhi2, Unigene13803\_JingChaoZuZhi2, Unigene14104\_JingChaoZuZhi2, Unigene14522\_JingChaoZuZhi2, Unigene14871\_JingChaoZuZhi2, Unigene15172\_JingChaoZuZhi2, Unigene15867\_JingChaoZuZhi2, Unigene16317\_JingChaoZuZhi2, Unigene16938\_JingChaoZuZhi2, Unigene17015\_JingChaoZuZhi2, Unigene17099\_JingChaoZuZhi2, Unigene17151\_JingChaoZuZhi2, Unigene18326\_JingChaoZuZhi2, Unigene18475\_JingChaoZuZhi2, Unigene19006\_JingChaoZuZhi2, Unigene19256\_JingChaoZuZhi2, Unigene19634\_JingChaoZuZhi2, Unigene20357\_JingChaoZuZhi2, Unigene20962\_JingChaoZuZhi2, Unigene21024\_JingChaoZuZhi2, Unigene2132\_JingChaoZuZhi2, Unigene21917\_JingChaoZuZhi2, Unigene21993\_JingChaoZuZhi2, Unigene22914\_JingChaoZuZhi2, Unigene25228\_JingChaoZuZhi2, Unigene33026\_JingChaoZuZhi2, Unigene35298\_JingChaoZuZhi2, Unigene35366\_JingChaoZuZhi2, Unigene36230\_JingChaoZuZhi2, Unigene37183\_JingChaoZuZhi2, Unigene38924\_JingChaoZuZhi2, Unigene40865\_JingChaoZuZhi2, Unigene4111\_JingChaoZuZhi2, Unigene41683\_JingChaoZuZhi2, Unigene41982\_JingChaoZuZhi2, Unigene42033\_JingChaoZuZhi2, Unigene44078\_JingChaoZuZhi2, Unigene4413\_JingChaoZuZhi2, Unigene46297\_JingChaoZuZhi2, Unigene47901\_JingChaoZuZhi2, Unigene48095\_JingChaoZuZhi2, Unigene49345\_JingChaoZuZhi2, Unigene49987\_JingChaoZuZhi2, Unigene52036\_JingChaoZuZhi2, Unigene52383\_JingChaoZuZhi2, Unigene52981\_JingChaoZuZhi2, Unigene54236\_JingChaoZuZhi2, Unigene55368\_JingChaoZuZhi2, Unigene55617\_JingChaoZuZhi2, Unigene55700\_JingChaoZuZhi2, Unigene55745\_JingChaoZuZhi2, Unigene56557\_JingChaoZuZhi2, Unigene57878\_JingChaoZuZhi2, Unigene58502\_JingChaoZuZhi2, Unigene6166\_JingChaoZuZhi2, Unigene63175\_JingChaoZuZhi2, Unigene63341\_JingChaoZuZhi2, Unigene63935\_JingChaoZuZhi2, Unigene64058\_JingChaoZuZhi2, Unigene65721\_JingChaoZuZhi2, Unigene65807\_JingChaoZuZhi2, Unigene66535\_JingChaoZuZhi2, Unigene67337\_JingChaoZuZhi2, Unigene68599\_JingChaoZuZhi2, Unigene68698\_JingChaoZuZhi2, Unigene68832\_JingChaoZuZhi2, Unigene68962\_JingChaoZuZhi2, Unigene70308\_JingChaoZuZhi2, Unigene7082\_JingChaoZuZhi2, Unigene7176\_JingChaoZuZhi2, Unigene72813\_JingChaoZuZhi2, Unigene73543\_JingChaoZuZhi2, Unigene74274\_JingChaoZuZhi2, Unigene76057\_JingChaoZuZhi2, Unigene76647\_JingChaoZuZhi2, Unigene76896\_JingChaoZuZhi2, Unigene77259\_JingChaoZuZhi2, Unigene77631\_JingChaoZuZhi2, Unigene78435\_JingChaoZuZhi2, Unigene78518\_JingChaoZuZhi2, Unigene78998\_JingChaoZuZhi2, Unigene79210\_JingChaoZuZhi2, Unigene7947\_JingChaoZuZhi2, Unigene79523\_JingChaoZuZhi2, Unigene79892\_JingChaoZuZhi2, Unigene81692\_JingChaoZuZhi2, Unigene81706\_JingChaoZuZhi2, Unigene82186\_JingChaoZuZhi2, Unigene83101\_JingChaoZuZhi2, Unigene8310\_JingChaoZuZhi2, Unigene83409\_JingChaoZuZhi2, Unigene83448\_JingChaoZuZhi2, Unigene83564\_JingChaoZuZhi2, Unigene83846\_JingChaoZuZhi2, Unigene84181\_JingChaoZuZhi2, Unigene84292\_JingChaoZuZhi2, Unigene85355\_JingChaoZuZhi2, Unigene85789\_JingChaoZuZhi2, Unigene86035\_JingChaoZuZhi2, Unigene86528\_JingChaoZuZhi2, Unigene86582\_JingChaoZuZhi2, Unigene86821\_JingChaoZuZhi2, Unigene87015\_JingChaoZuZhi2, Unigene87347\_JingChaoZuZhi2, Unigene87349\_JingChaoZuZhi2, Unigene87389\_JingChaoZuZhi2, Unigene87433\_JingChaoZuZhi2, Unigene87796\_JingChaoZuZhi2, Unigene87948\_JingChaoZuZhi2, Unigene88102\_JingChaoZuZhi2, Unigene88135\_JingChaoZuZhi2, Unigene88262\_JingChaoZuZhi2, Unigene88312\_JingChaoZuZhi2, Unigene8837\_JingChaoZuZhi2, Unigene88393\_JingChaoZuZhi2, Unigene88475\_JingChaoZuZhi2, Unigene88774\_JingChaoZuZhi2, Unigene88933\_JingChaoZuZhi2, Unigene89128\_JingChaoZuZhi2, Unigene89210\_JingChaoZuZhi2, Unigene89245\_JingChaoZuZhi2, Unigene89265\_JingChaoZuZhi2, Unigene89595\_JingChaoZuZhi2, Unigene89653\_JingChaoZuZhi2, Unigene89670\_JingChaoZuZhi2, Unigene90062\_JingChaoZuZhi2, Unigene90077\_JingChaoZuZhi2, Unigene90348\_JingChaoZuZhi2, Unigene90358\_JingChaoZuZhi2, Unigene90938\_JingChaoZuZhi2, Unigene91051\_JingChaoZuZhi2, Unigene91080\_JingChaoZuZhi2, Unigene91081\_JingChaoZuZhi2, Unigene91490\_JingChaoZuZhi2, Unigene91855\_JingChaoZuZhi2, Unigene91973\_JingChaoZuZhi2, Unigene92187\_JingChaoZuZhi2, Unigene92312\_JingChaoZuZhi2, Unigene92321\_JingChaoZuZhi2, Unigene92429\_JingChaoZuZhi2, Unigene92452\_JingChaoZuZhi2, Unigene92539\_JingChaoZuZhi2, Unigene9256\_JingChaoZuZhi2, Unigene92659\_JingChaoZuZhi2, Unigene92778\_JingChaoZuZhi2, Unigene93095\_JingChaoZuZhi2, Unigene93265\_JingChaoZuZhi2, Unigene93406\_JingChaoZuZhi2, Unigene93604\_JingChaoZuZhi2, Unigene93701\_JingChaoZuZhi2, Unigene93785\_JingChaoZuZhi2, Unigene93940\_JingChaoZuZhi2, Unigene94021\_JingChaoZuZhi2, Unigene94029\_JingChaoZuZhi2, Unigene94552\_JingChaoZuZhi2, Unigene94775\_JingChaoZuZhi2, Unigene94892\_JingChaoZuZhi2, Unigene95041\_JingChaoZuZhi2, Unigene95069\_JingChaoZuZhi2, Unigene95159\_JingChaoZuZhi2, Unigene95331\_JingChaoZuZhi2, Unigene95742\_JingChaoZuZhi2, Unigene95789\_JingChaoZuZhi2, Unigene96002\_JingChaoZuZhi2, Unigene96023\_JingChaoZuZhi2, Unigene96229\_JingChaoZuZhi2, Unigene96275\_JingChaoZuZhi2 |
| 67 | Natural killer cell mediated cytotoxicity | Unigene1001\_JingChaoZuZhi2, Unigene10053\_JingChaoZuZhi2, Unigene10290\_JingChaoZuZhi2, Unigene10318\_JingChaoZuZhi2, Unigene1109\_JingChaoZuZhi2, Unigene11287\_JingChaoZuZhi2, Unigene11961\_JingChaoZuZhi2, Unigene12171\_JingChaoZuZhi2, Unigene12214\_JingChaoZuZhi2, Unigene12420\_JingChaoZuZhi2, Unigene12813\_JingChaoZuZhi2, Unigene1298\_JingChaoZuZhi2, Unigene1301\_JingChaoZuZhi2, Unigene13180\_JingChaoZuZhi2, Unigene13510\_JingChaoZuZhi2, Unigene14317\_JingChaoZuZhi2, Unigene14370\_JingChaoZuZhi2, Unigene14488\_JingChaoZuZhi2, Unigene14738\_JingChaoZuZhi2, Unigene14842\_JingChaoZuZhi2, Unigene14912\_JingChaoZuZhi2, Unigene14973\_JingChaoZuZhi2, Unigene14990\_JingChaoZuZhi2, Unigene15095\_JingChaoZuZhi2, Unigene15104\_JingChaoZuZhi2, Unigene15228\_JingChaoZuZhi2, Unigene16039\_JingChaoZuZhi2, Unigene1609\_JingChaoZuZhi2, Unigene16116\_JingChaoZuZhi2, Unigene16154\_JingChaoZuZhi2, Unigene16521\_JingChaoZuZhi2, Unigene16589\_JingChaoZuZhi2, Unigene16933\_JingChaoZuZhi2, Unigene17136\_JingChaoZuZhi2, Unigene17168\_JingChaoZuZhi2, Unigene17247\_JingChaoZuZhi2, Unigene17710\_JingChaoZuZhi2, Unigene17829\_JingChaoZuZhi2, Unigene18464\_JingChaoZuZhi2, Unigene18672\_JingChaoZuZhi2, Unigene18868\_JingChaoZuZhi2, Unigene18904\_JingChaoZuZhi2, Unigene19021\_JingChaoZuZhi2, Unigene19036\_JingChaoZuZhi2, Unigene19046\_JingChaoZuZhi2, Unigene1942\_JingChaoZuZhi2, Unigene19531\_JingChaoZuZhi2, Unigene1993\_JingChaoZuZhi2, Unigene20256\_JingChaoZuZhi2, Unigene20649\_JingChaoZuZhi2, Unigene21144\_JingChaoZuZhi2, Unigene21246\_JingChaoZuZhi2, Unigene21295\_JingChaoZuZhi2, Unigene21348\_JingChaoZuZhi2, Unigene21513\_JingChaoZuZhi2, Unigene21578\_JingChaoZuZhi2, Unigene21605\_JingChaoZuZhi2, Unigene21737\_JingChaoZuZhi2, Unigene21973\_JingChaoZuZhi2, Unigene22102\_JingChaoZuZhi2, Unigene22174\_JingChaoZuZhi2, Unigene22386\_JingChaoZuZhi2, Unigene22482\_JingChaoZuZhi2, Unigene22648\_JingChaoZuZhi2, Unigene22779\_JingChaoZuZhi2, Unigene22804\_JingChaoZuZhi2, Unigene22934\_JingChaoZuZhi2, Unigene2456\_JingChaoZuZhi2, Unigene24841\_JingChaoZuZhi2, Unigene24881\_JingChaoZuZhi2, Unigene25966\_JingChaoZuZhi2, Unigene26183\_JingChaoZuZhi2, Unigene26730\_JingChaoZuZhi2, Unigene277\_JingChaoZuZhi2, Unigene29201\_JingChaoZuZhi2, Unigene2960\_JingChaoZuZhi2, Unigene3016\_JingChaoZuZhi2, Unigene3185\_JingChaoZuZhi2, Unigene35744\_JingChaoZuZhi2, Unigene38493\_JingChaoZuZhi2, Unigene39539\_JingChaoZuZhi2, Unigene39577\_JingChaoZuZhi2, Unigene40020\_JingChaoZuZhi2, Unigene40825\_JingChaoZuZhi2, Unigene4186\_JingChaoZuZhi2, Unigene4223\_JingChaoZuZhi2, Unigene42332\_JingChaoZuZhi2, Unigene45962\_JingChaoZuZhi2, Unigene45\_JingChaoZuZhi2, Unigene46219\_JingChaoZuZhi2, Unigene48368\_JingChaoZuZhi2, Unigene4838\_JingChaoZuZhi2, Unigene48607\_JingChaoZuZhi2, Unigene48751\_JingChaoZuZhi2, Unigene48843\_JingChaoZuZhi2, Unigene51705\_JingChaoZuZhi2, Unigene5196\_JingChaoZuZhi2, Unigene53078\_JingChaoZuZhi2, Unigene5638\_JingChaoZuZhi2, Unigene57337\_JingChaoZuZhi2, Unigene57488\_JingChaoZuZhi2, Unigene601\_JingChaoZuZhi2, Unigene6028\_JingChaoZuZhi2, Unigene61108\_JingChaoZuZhi2, Unigene61406\_JingChaoZuZhi2, Unigene6211\_JingChaoZuZhi2, Unigene63226\_JingChaoZuZhi2, Unigene64164\_JingChaoZuZhi2, Unigene64830\_JingChaoZuZhi2, Unigene6493\_JingChaoZuZhi2, Unigene65294\_JingChaoZuZhi2, Unigene6676\_JingChaoZuZhi2, Unigene6752\_JingChaoZuZhi2, Unigene67655\_JingChaoZuZhi2, Unigene67793\_JingChaoZuZhi2, Unigene68108\_JingChaoZuZhi2, Unigene68200\_JingChaoZuZhi2, Unigene70505\_JingChaoZuZhi2, Unigene70549\_JingChaoZuZhi2, Unigene70723\_JingChaoZuZhi2, Unigene722\_JingChaoZuZhi2, Unigene7417\_JingChaoZuZhi2, Unigene76575\_JingChaoZuZhi2, Unigene78957\_JingChaoZuZhi2, Unigene7939\_JingChaoZuZhi2, Unigene79860\_JingChaoZuZhi2, Unigene8003\_JingChaoZuZhi2, Unigene80331\_JingChaoZuZhi2, Unigene81086\_JingChaoZuZhi2, Unigene81687\_JingChaoZuZhi2, Unigene8191\_JingChaoZuZhi2, Unigene8202\_JingChaoZuZhi2, Unigene82219\_JingChaoZuZhi2, Unigene8248\_JingChaoZuZhi2, Unigene84862\_JingChaoZuZhi2, Unigene85574\_JingChaoZuZhi2, Unigene86206\_JingChaoZuZhi2, Unigene8621\_JingChaoZuZhi2, Unigene86526\_JingChaoZuZhi2, Unigene86713\_JingChaoZuZhi2, Unigene87332\_JingChaoZuZhi2, Unigene876\_JingChaoZuZhi2, Unigene87851\_JingChaoZuZhi2, Unigene8949\_JingChaoZuZhi2, Unigene90762\_JingChaoZuZhi2, Unigene91179\_JingChaoZuZhi2, Unigene91612\_JingChaoZuZhi2, Unigene91958\_JingChaoZuZhi2, Unigene92112\_JingChaoZuZhi2, Unigene92372\_JingChaoZuZhi2, Unigene92800\_JingChaoZuZhi2, Unigene93793\_JingChaoZuZhi2, Unigene93807\_JingChaoZuZhi2, Unigene94149\_JingChaoZuZhi2, Unigene94225\_JingChaoZuZhi2, Unigene94456\_JingChaoZuZhi2, Unigene9445\_JingChaoZuZhi2, Unigene9461\_JingChaoZuZhi2, Unigene94685\_JingChaoZuZhi2, Unigene94749\_JingChaoZuZhi2, Unigene95147\_JingChaoZuZhi2, Unigene95456\_JingChaoZuZhi2, Unigene95677\_JingChaoZuZhi2, Unigene95875\_JingChaoZuZhi2, Unigene96294\_JingChaoZuZhi2, Unigene9645\_JingChaoZuZhi2, Unigene9902\_JingChaoZuZhi2 |
| 68 | GnRH signaling pathway | Unigene10016\_JingChaoZuZhi2, Unigene10318\_JingChaoZuZhi2, Unigene10422\_JingChaoZuZhi2, Unigene11055\_JingChaoZuZhi2, Unigene11251\_JingChaoZuZhi2, Unigene11726\_JingChaoZuZhi2, Unigene11951\_JingChaoZuZhi2, Unigene12073\_JingChaoZuZhi2, Unigene12260\_JingChaoZuZhi2, Unigene12420\_JingChaoZuZhi2, Unigene12899\_JingChaoZuZhi2, Unigene12912\_JingChaoZuZhi2, Unigene12938\_JingChaoZuZhi2, Unigene13420\_JingChaoZuZhi2, Unigene13770\_JingChaoZuZhi2, Unigene13869\_JingChaoZuZhi2, Unigene13946\_JingChaoZuZhi2, Unigene14357\_JingChaoZuZhi2, Unigene14360\_JingChaoZuZhi2, Unigene14567\_JingChaoZuZhi2, Unigene14707\_JingChaoZuZhi2, Unigene15216\_JingChaoZuZhi2, Unigene15425\_JingChaoZuZhi2, Unigene15651\_JingChaoZuZhi2, Unigene15942\_JingChaoZuZhi2, Unigene16566\_JingChaoZuZhi2, Unigene16991\_JingChaoZuZhi2, Unigene17666\_JingChaoZuZhi2, Unigene17888\_JingChaoZuZhi2, Unigene18307\_JingChaoZuZhi2, Unigene18715\_JingChaoZuZhi2, Unigene18944\_JingChaoZuZhi2, Unigene19904\_JingChaoZuZhi2, Unigene20120\_JingChaoZuZhi2, Unigene21407\_JingChaoZuZhi2, Unigene21578\_JingChaoZuZhi2, Unigene21605\_JingChaoZuZhi2, Unigene21691\_JingChaoZuZhi2, Unigene21815\_JingChaoZuZhi2, Unigene22226\_JingChaoZuZhi2, Unigene2275\_JingChaoZuZhi2, Unigene22779\_JingChaoZuZhi2, Unigene26730\_JingChaoZuZhi2, Unigene2687\_JingChaoZuZhi2, Unigene2790\_JingChaoZuZhi2, Unigene2960\_JingChaoZuZhi2, Unigene3122\_JingChaoZuZhi2, Unigene32827\_JingChaoZuZhi2, Unigene34929\_JingChaoZuZhi2, Unigene35952\_JingChaoZuZhi2, Unigene3700\_JingChaoZuZhi2, Unigene39832\_JingChaoZuZhi2, Unigene40335\_JingChaoZuZhi2, Unigene41333\_JingChaoZuZhi2, Unigene4186\_JingChaoZuZhi2, Unigene42329\_JingChaoZuZhi2, Unigene4294\_JingChaoZuZhi2, Unigene43059\_JingChaoZuZhi2, Unigene43723\_JingChaoZuZhi2, Unigene45532\_JingChaoZuZhi2, Unigene4838\_JingChaoZuZhi2, Unigene49203\_JingChaoZuZhi2, Unigene49664\_JingChaoZuZhi2, Unigene49798\_JingChaoZuZhi2, Unigene51991\_JingChaoZuZhi2, Unigene52554\_JingChaoZuZhi2, Unigene53078\_JingChaoZuZhi2, Unigene53935\_JingChaoZuZhi2, Unigene53958\_JingChaoZuZhi2, Unigene54012\_JingChaoZuZhi2, Unigene54367\_JingChaoZuZhi2, Unigene54449\_JingChaoZuZhi2, Unigene54470\_JingChaoZuZhi2, Unigene57185\_JingChaoZuZhi2, Unigene57337\_JingChaoZuZhi2, Unigene5823\_JingChaoZuZhi2, Unigene59102\_JingChaoZuZhi2, Unigene59471\_JingChaoZuZhi2, Unigene59480\_JingChaoZuZhi2, Unigene59885\_JingChaoZuZhi2, Unigene6028\_JingChaoZuZhi2, Unigene61044\_JingChaoZuZhi2, Unigene62786\_JingChaoZuZhi2, Unigene6393\_JingChaoZuZhi2, Unigene63994\_JingChaoZuZhi2, Unigene64164\_JingChaoZuZhi2, Unigene6459\_JingChaoZuZhi2, Unigene64830\_JingChaoZuZhi2, Unigene6493\_JingChaoZuZhi2, Unigene65445\_JingChaoZuZhi2, Unigene66073\_JingChaoZuZhi2, Unigene66540\_JingChaoZuZhi2, Unigene67095\_JingChaoZuZhi2, Unigene67328\_JingChaoZuZhi2, Unigene67793\_JingChaoZuZhi2, Unigene68151\_JingChaoZuZhi2, Unigene70063\_JingChaoZuZhi2, Unigene70193\_JingChaoZuZhi2, Unigene70493\_JingChaoZuZhi2, Unigene70549\_JingChaoZuZhi2, Unigene70723\_JingChaoZuZhi2, Unigene71365\_JingChaoZuZhi2, Unigene72098\_JingChaoZuZhi2, Unigene72136\_JingChaoZuZhi2, Unigene72521\_JingChaoZuZhi2, Unigene72643\_JingChaoZuZhi2, Unigene7456\_JingChaoZuZhi2, Unigene74594\_JingChaoZuZhi2, Unigene75235\_JingChaoZuZhi2, Unigene76182\_JingChaoZuZhi2, Unigene77149\_JingChaoZuZhi2, Unigene78078\_JingChaoZuZhi2, Unigene78235\_JingChaoZuZhi2, Unigene78574\_JingChaoZuZhi2, Unigene7877\_JingChaoZuZhi2, Unigene79166\_JingChaoZuZhi2, Unigene7939\_JingChaoZuZhi2, Unigene8191\_JingChaoZuZhi2, Unigene81948\_JingChaoZuZhi2, Unigene82073\_JingChaoZuZhi2, Unigene84247\_JingChaoZuZhi2, Unigene84969\_JingChaoZuZhi2, Unigene8507\_JingChaoZuZhi2, Unigene85574\_JingChaoZuZhi2, Unigene86899\_JingChaoZuZhi2, Unigene87305\_JingChaoZuZhi2, Unigene87332\_JingChaoZuZhi2, Unigene87607\_JingChaoZuZhi2, Unigene876\_JingChaoZuZhi2, Unigene88068\_JingChaoZuZhi2, Unigene88385\_JingChaoZuZhi2, Unigene88594\_JingChaoZuZhi2, Unigene88724\_JingChaoZuZhi2, Unigene88773\_JingChaoZuZhi2, Unigene89147\_JingChaoZuZhi2, Unigene89603\_JingChaoZuZhi2, Unigene89807\_JingChaoZuZhi2, Unigene91527\_JingChaoZuZhi2, Unigene91612\_JingChaoZuZhi2, Unigene91739\_JingChaoZuZhi2, Unigene91924\_JingChaoZuZhi2, Unigene91958\_JingChaoZuZhi2, Unigene92394\_JingChaoZuZhi2, Unigene92737\_JingChaoZuZhi2, Unigene93012\_JingChaoZuZhi2, Unigene93588\_JingChaoZuZhi2, Unigene93597\_JingChaoZuZhi2, Unigene93714\_JingChaoZuZhi2, Unigene93782\_JingChaoZuZhi2, Unigene93793\_JingChaoZuZhi2, Unigene9389\_JingChaoZuZhi2, Unigene94149\_JingChaoZuZhi2, Unigene94456\_JingChaoZuZhi2, Unigene94619\_JingChaoZuZhi2, Unigene9488\_JingChaoZuZhi2, Unigene95213\_JingChaoZuZhi2, Unigene95456\_JingChaoZuZhi2, Unigene95677\_JingChaoZuZhi2, Unigene95916\_JingChaoZuZhi2, Unigene96262\_JingChaoZuZhi2, Unigene9654\_JingChaoZuZhi2, Unigene9711\_JingChaoZuZhi2, Unigene9902\_JingChaoZuZhi2, Unigene9\_JingChaoZuZhi2 |
| 69 | Small cell lung cancer | Unigene10597\_JingChaoZuZhi2, Unigene10679\_JingChaoZuZhi2, Unigene10755\_JingChaoZuZhi2, Unigene1111\_JingChaoZuZhi2, Unigene11446\_JingChaoZuZhi2, Unigene11961\_JingChaoZuZhi2, Unigene12202\_JingChaoZuZhi2, Unigene12376\_JingChaoZuZhi2, Unigene12595\_JingChaoZuZhi2, Unigene12972\_JingChaoZuZhi2, Unigene13176\_JingChaoZuZhi2, Unigene13389\_JingChaoZuZhi2, Unigene13390\_JingChaoZuZhi2, Unigene13615\_JingChaoZuZhi2, Unigene13925\_JingChaoZuZhi2, Unigene14390\_JingChaoZuZhi2, Unigene14534\_JingChaoZuZhi2, Unigene14912\_JingChaoZuZhi2, Unigene15228\_JingChaoZuZhi2, Unigene15814\_JingChaoZuZhi2, Unigene16072\_JingChaoZuZhi2, Unigene16121\_JingChaoZuZhi2, Unigene16873\_JingChaoZuZhi2, Unigene17128\_JingChaoZuZhi2, Unigene17243\_JingChaoZuZhi2, Unigene17247\_JingChaoZuZhi2, Unigene17467\_JingChaoZuZhi2, Unigene17675\_JingChaoZuZhi2, Unigene18209\_JingChaoZuZhi2, Unigene18672\_JingChaoZuZhi2, Unigene18780\_JingChaoZuZhi2, Unigene19826\_JingChaoZuZhi2, Unigene20157\_JingChaoZuZhi2, Unigene20411\_JingChaoZuZhi2, Unigene20794\_JingChaoZuZhi2, Unigene20991\_JingChaoZuZhi2, Unigene21253\_JingChaoZuZhi2, Unigene21575\_JingChaoZuZhi2, Unigene21697\_JingChaoZuZhi2, Unigene22245\_JingChaoZuZhi2, Unigene22407\_JingChaoZuZhi2, Unigene22445\_JingChaoZuZhi2, Unigene22464\_JingChaoZuZhi2, Unigene22466\_JingChaoZuZhi2, Unigene22517\_JingChaoZuZhi2, Unigene22572\_JingChaoZuZhi2, Unigene22638\_JingChaoZuZhi2, Unigene2379\_JingChaoZuZhi2, Unigene2456\_JingChaoZuZhi2, Unigene24772\_JingChaoZuZhi2, Unigene26438\_JingChaoZuZhi2, Unigene27933\_JingChaoZuZhi2, Unigene28208\_JingChaoZuZhi2, Unigene28467\_JingChaoZuZhi2, Unigene29488\_JingChaoZuZhi2, Unigene33182\_JingChaoZuZhi2, Unigene34807\_JingChaoZuZhi2, Unigene35243\_JingChaoZuZhi2, Unigene35624\_JingChaoZuZhi2, Unigene3585\_JingChaoZuZhi2, Unigene36479\_JingChaoZuZhi2, Unigene3763\_JingChaoZuZhi2, Unigene3931\_JingChaoZuZhi2, Unigene39674\_JingChaoZuZhi2, Unigene42070\_JingChaoZuZhi2, Unigene42969\_JingChaoZuZhi2, Unigene42981\_JingChaoZuZhi2, Unigene4377\_JingChaoZuZhi2, Unigene44191\_JingChaoZuZhi2, Unigene451\_JingChaoZuZhi2, Unigene45739\_JingChaoZuZhi2, Unigene4841\_JingChaoZuZhi2, Unigene48673\_JingChaoZuZhi2, Unigene48751\_JingChaoZuZhi2, Unigene48843\_JingChaoZuZhi2, Unigene499\_JingChaoZuZhi2, Unigene5069\_JingChaoZuZhi2, Unigene5099\_JingChaoZuZhi2, Unigene51285\_JingChaoZuZhi2, Unigene5165\_JingChaoZuZhi2, Unigene53503\_JingChaoZuZhi2, Unigene53917\_JingChaoZuZhi2, Unigene54320\_JingChaoZuZhi2, Unigene54679\_JingChaoZuZhi2, Unigene56642\_JingChaoZuZhi2, Unigene56772\_JingChaoZuZhi2, Unigene57488\_JingChaoZuZhi2, Unigene5758\_JingChaoZuZhi2, Unigene58670\_JingChaoZuZhi2, Unigene59797\_JingChaoZuZhi2, Unigene62919\_JingChaoZuZhi2, Unigene63547\_JingChaoZuZhi2, Unigene6433\_JingChaoZuZhi2, Unigene64750\_JingChaoZuZhi2, Unigene65879\_JingChaoZuZhi2, Unigene66362\_JingChaoZuZhi2, Unigene6699\_JingChaoZuZhi2, Unigene67542\_JingChaoZuZhi2, Unigene6785\_JingChaoZuZhi2, Unigene68298\_JingChaoZuZhi2, Unigene68495\_JingChaoZuZhi2, Unigene70155\_JingChaoZuZhi2, Unigene70696\_JingChaoZuZhi2, Unigene71144\_JingChaoZuZhi2, Unigene74335\_JingChaoZuZhi2, Unigene74795\_JingChaoZuZhi2, Unigene75840\_JingChaoZuZhi2, Unigene76592\_JingChaoZuZhi2, Unigene76805\_JingChaoZuZhi2, Unigene76917\_JingChaoZuZhi2, Unigene77061\_JingChaoZuZhi2, Unigene77549\_JingChaoZuZhi2, Unigene77570\_JingChaoZuZhi2, Unigene78508\_JingChaoZuZhi2, Unigene78909\_JingChaoZuZhi2, Unigene79077\_JingChaoZuZhi2, Unigene79109\_JingChaoZuZhi2, Unigene80818\_JingChaoZuZhi2, Unigene8133\_JingChaoZuZhi2, Unigene82761\_JingChaoZuZhi2, Unigene83507\_JingChaoZuZhi2, Unigene84257\_JingChaoZuZhi2, Unigene84519\_JingChaoZuZhi2, Unigene84930\_JingChaoZuZhi2, Unigene86206\_JingChaoZuZhi2, Unigene86296\_JingChaoZuZhi2, Unigene86526\_JingChaoZuZhi2, Unigene87448\_JingChaoZuZhi2, Unigene87952\_JingChaoZuZhi2, Unigene88185\_JingChaoZuZhi2, Unigene88391\_JingChaoZuZhi2, Unigene88748\_JingChaoZuZhi2, Unigene88925\_JingChaoZuZhi2, Unigene88942\_JingChaoZuZhi2, Unigene89329\_JingChaoZuZhi2, Unigene89502\_JingChaoZuZhi2, Unigene89612\_JingChaoZuZhi2, Unigene90003\_JingChaoZuZhi2, Unigene90078\_JingChaoZuZhi2, Unigene90201\_JingChaoZuZhi2, Unigene90261\_JingChaoZuZhi2, Unigene90535\_JingChaoZuZhi2, Unigene91580\_JingChaoZuZhi2, Unigene91689\_JingChaoZuZhi2, Unigene92104\_JingChaoZuZhi2, Unigene92112\_JingChaoZuZhi2, Unigene92831\_JingChaoZuZhi2, Unigene93195\_JingChaoZuZhi2, Unigene93807\_JingChaoZuZhi2, Unigene94204\_JingChaoZuZhi2, Unigene94276\_JingChaoZuZhi2, Unigene94561\_JingChaoZuZhi2, Unigene94685\_JingChaoZuZhi2, Unigene94829\_JingChaoZuZhi2, Unigene95674\_JingChaoZuZhi2, Unigene95782\_JingChaoZuZhi2, Unigene95791\_JingChaoZuZhi2, Unigene95799\_JingChaoZuZhi2, Unigene95994\_JingChaoZuZhi2, Unigene96018\_JingChaoZuZhi2, Unigene96057\_JingChaoZuZhi2, Unigene96121\_JingChaoZuZhi2, Unigene96294\_JingChaoZuZhi2, Unigene9630\_JingChaoZuZhi2 |
| 70 | Inositol phosphate metabolism | Unigene10122\_JingChaoZuZhi2, Unigene10443\_JingChaoZuZhi2, Unigene10806\_JingChaoZuZhi2, Unigene10860\_JingChaoZuZhi2, Unigene11074\_JingChaoZuZhi2, Unigene11961\_JingChaoZuZhi2, Unigene11995\_JingChaoZuZhi2, Unigene12700\_JingChaoZuZhi2, Unigene12979\_JingChaoZuZhi2, Unigene13149\_JingChaoZuZhi2, Unigene13176\_JingChaoZuZhi2, Unigene13429\_JingChaoZuZhi2, Unigene13452\_JingChaoZuZhi2, Unigene13972\_JingChaoZuZhi2, Unigene14068\_JingChaoZuZhi2, Unigene14360\_JingChaoZuZhi2, Unigene14977\_JingChaoZuZhi2, Unigene16149\_JingChaoZuZhi2, Unigene16293\_JingChaoZuZhi2, Unigene16646\_JingChaoZuZhi2, Unigene16991\_JingChaoZuZhi2, Unigene17247\_JingChaoZuZhi2, Unigene18176\_JingChaoZuZhi2, Unigene18464\_JingChaoZuZhi2, Unigene18672\_JingChaoZuZhi2, Unigene18721\_JingChaoZuZhi2, Unigene1878\_JingChaoZuZhi2, Unigene18868\_JingChaoZuZhi2, Unigene1922\_JingChaoZuZhi2, Unigene19458\_JingChaoZuZhi2, Unigene19565\_JingChaoZuZhi2, Unigene19862\_JingChaoZuZhi2, Unigene20857\_JingChaoZuZhi2, Unigene20959\_JingChaoZuZhi2, Unigene21020\_JingChaoZuZhi2, Unigene21295\_JingChaoZuZhi2, Unigene22309\_JingChaoZuZhi2, Unigene22387\_JingChaoZuZhi2, Unigene22579\_JingChaoZuZhi2, Unigene2456\_JingChaoZuZhi2, Unigene2485\_JingChaoZuZhi2, Unigene26869\_JingChaoZuZhi2, Unigene30799\_JingChaoZuZhi2, Unigene3116\_JingChaoZuZhi2, Unigene3457\_JingChaoZuZhi2, Unigene37471\_JingChaoZuZhi2, Unigene3850\_JingChaoZuZhi2, Unigene38514\_JingChaoZuZhi2, Unigene39577\_JingChaoZuZhi2, Unigene4050\_JingChaoZuZhi2, Unigene41333\_JingChaoZuZhi2, Unigene42887\_JingChaoZuZhi2, Unigene43322\_JingChaoZuZhi2, Unigene4377\_JingChaoZuZhi2, Unigene44152\_JingChaoZuZhi2, Unigene44961\_JingChaoZuZhi2, Unigene45682\_JingChaoZuZhi2, Unigene46223\_JingChaoZuZhi2, Unigene46908\_JingChaoZuZhi2, Unigene4713\_JingChaoZuZhi2, Unigene48751\_JingChaoZuZhi2, Unigene49963\_JingChaoZuZhi2, Unigene50173\_JingChaoZuZhi2, Unigene50908\_JingChaoZuZhi2, Unigene5113\_JingChaoZuZhi2, Unigene51895\_JingChaoZuZhi2, Unigene53240\_JingChaoZuZhi2, Unigene54367\_JingChaoZuZhi2, Unigene57488\_JingChaoZuZhi2, Unigene5782\_JingChaoZuZhi2, Unigene59493\_JingChaoZuZhi2, Unigene6009\_JingChaoZuZhi2, Unigene62246\_JingChaoZuZhi2, Unigene6250\_JingChaoZuZhi2, Unigene62804\_JingChaoZuZhi2, Unigene62887\_JingChaoZuZhi2, Unigene63807\_JingChaoZuZhi2, Unigene64393\_JingChaoZuZhi2, Unigene64615\_JingChaoZuZhi2, Unigene65445\_JingChaoZuZhi2, Unigene66540\_JingChaoZuZhi2, Unigene67655\_JingChaoZuZhi2, Unigene68024\_JingChaoZuZhi2, Unigene68159\_JingChaoZuZhi2, Unigene68452\_JingChaoZuZhi2, Unigene68845\_JingChaoZuZhi2, Unigene71246\_JingChaoZuZhi2, Unigene71939\_JingChaoZuZhi2, Unigene72466\_JingChaoZuZhi2, Unigene74599\_JingChaoZuZhi2, Unigene7508\_JingChaoZuZhi2, Unigene75749\_JingChaoZuZhi2, Unigene76000\_JingChaoZuZhi2, Unigene77149\_JingChaoZuZhi2, Unigene77344\_JingChaoZuZhi2, Unigene77528\_JingChaoZuZhi2, Unigene7799\_JingChaoZuZhi2, Unigene78078\_JingChaoZuZhi2, Unigene7817\_JingChaoZuZhi2, Unigene78189\_JingChaoZuZhi2, Unigene79239\_JingChaoZuZhi2, Unigene79268\_JingChaoZuZhi2, Unigene79895\_JingChaoZuZhi2, Unigene81951\_JingChaoZuZhi2, Unigene82061\_JingChaoZuZhi2, Unigene82389\_JingChaoZuZhi2, Unigene82414\_JingChaoZuZhi2, Unigene83268\_JingChaoZuZhi2, Unigene83898\_JingChaoZuZhi2, Unigene84076\_JingChaoZuZhi2, Unigene84222\_JingChaoZuZhi2, Unigene84455\_JingChaoZuZhi2, Unigene8455\_JingChaoZuZhi2, Unigene84839\_JingChaoZuZhi2, Unigene86007\_JingChaoZuZhi2, Unigene8606\_JingChaoZuZhi2, Unigene86206\_JingChaoZuZhi2, Unigene86526\_JingChaoZuZhi2, Unigene86545\_JingChaoZuZhi2, Unigene86995\_JingChaoZuZhi2, Unigene87020\_JingChaoZuZhi2, Unigene87332\_JingChaoZuZhi2, Unigene8737\_JingChaoZuZhi2, Unigene87478\_JingChaoZuZhi2, Unigene87524\_JingChaoZuZhi2, Unigene87607\_JingChaoZuZhi2, Unigene87776\_JingChaoZuZhi2, Unigene87847\_JingChaoZuZhi2, Unigene87960\_JingChaoZuZhi2, Unigene88090\_JingChaoZuZhi2, Unigene881\_JingChaoZuZhi2, Unigene88385\_JingChaoZuZhi2, Unigene88748\_JingChaoZuZhi2, Unigene88773\_JingChaoZuZhi2, Unigene88801\_JingChaoZuZhi2, Unigene91687\_JingChaoZuZhi2, Unigene91739\_JingChaoZuZhi2, Unigene91751\_JingChaoZuZhi2, Unigene92112\_JingChaoZuZhi2, Unigene92395\_JingChaoZuZhi2, Unigene92978\_JingChaoZuZhi2, Unigene92989\_JingChaoZuZhi2, Unigene93731\_JingChaoZuZhi2, Unigene93807\_JingChaoZuZhi2, Unigene94406\_JingChaoZuZhi2, Unigene94565\_JingChaoZuZhi2, Unigene9461\_JingChaoZuZhi2, Unigene94647\_JingChaoZuZhi2, Unigene94685\_JingChaoZuZhi2, Unigene94777\_JingChaoZuZhi2, Unigene94898\_JingChaoZuZhi2, Unigene94962\_JingChaoZuZhi2, Unigene95523\_JingChaoZuZhi2, Unigene95622\_JingChaoZuZhi2, Unigene95791\_JingChaoZuZhi2, Unigene95992\_JingChaoZuZhi2, Unigene96076\_JingChaoZuZhi2, Unigene96119\_JingChaoZuZhi2 |
| 71 | B cell receptor signaling pathway | Unigene1001\_JingChaoZuZhi2, Unigene10290\_JingChaoZuZhi2, Unigene10318\_JingChaoZuZhi2, Unigene1109\_JingChaoZuZhi2, Unigene11287\_JingChaoZuZhi2, Unigene11961\_JingChaoZuZhi2, Unigene12171\_JingChaoZuZhi2, Unigene12214\_JingChaoZuZhi2, Unigene12420\_JingChaoZuZhi2, Unigene1301\_JingChaoZuZhi2, Unigene13180\_JingChaoZuZhi2, Unigene14317\_JingChaoZuZhi2, Unigene14370\_JingChaoZuZhi2, Unigene14488\_JingChaoZuZhi2, Unigene14912\_JingChaoZuZhi2, Unigene14973\_JingChaoZuZhi2, Unigene14990\_JingChaoZuZhi2, Unigene15104\_JingChaoZuZhi2, Unigene15228\_JingChaoZuZhi2, Unigene16039\_JingChaoZuZhi2, Unigene1609\_JingChaoZuZhi2, Unigene16116\_JingChaoZuZhi2, Unigene16154\_JingChaoZuZhi2, Unigene16521\_JingChaoZuZhi2, Unigene16589\_JingChaoZuZhi2, Unigene16820\_JingChaoZuZhi2, Unigene16933\_JingChaoZuZhi2, Unigene17136\_JingChaoZuZhi2, Unigene17168\_JingChaoZuZhi2, Unigene17247\_JingChaoZuZhi2, Unigene17710\_JingChaoZuZhi2, Unigene18464\_JingChaoZuZhi2, Unigene18672\_JingChaoZuZhi2, Unigene18715\_JingChaoZuZhi2, Unigene18868\_JingChaoZuZhi2, Unigene18904\_JingChaoZuZhi2, Unigene18966\_JingChaoZuZhi2, Unigene19021\_JingChaoZuZhi2, Unigene19036\_JingChaoZuZhi2, Unigene1942\_JingChaoZuZhi2, Unigene19531\_JingChaoZuZhi2, Unigene1993\_JingChaoZuZhi2, Unigene20157\_JingChaoZuZhi2, Unigene20256\_JingChaoZuZhi2, Unigene20649\_JingChaoZuZhi2, Unigene20991\_JingChaoZuZhi2, Unigene21144\_JingChaoZuZhi2, Unigene21246\_JingChaoZuZhi2, Unigene21295\_JingChaoZuZhi2, Unigene21348\_JingChaoZuZhi2, Unigene21578\_JingChaoZuZhi2, Unigene21697\_JingChaoZuZhi2, Unigene21737\_JingChaoZuZhi2, Unigene21973\_JingChaoZuZhi2, Unigene22102\_JingChaoZuZhi2, Unigene22386\_JingChaoZuZhi2, Unigene22466\_JingChaoZuZhi2, Unigene22648\_JingChaoZuZhi2, Unigene22779\_JingChaoZuZhi2, Unigene22934\_JingChaoZuZhi2, Unigene2456\_JingChaoZuZhi2, Unigene24841\_JingChaoZuZhi2, Unigene24881\_JingChaoZuZhi2, Unigene25966\_JingChaoZuZhi2, Unigene26183\_JingChaoZuZhi2, Unigene26730\_JingChaoZuZhi2, Unigene277\_JingChaoZuZhi2, Unigene2960\_JingChaoZuZhi2, Unigene3016\_JingChaoZuZhi2, Unigene3185\_JingChaoZuZhi2, Unigene35744\_JingChaoZuZhi2, Unigene38493\_JingChaoZuZhi2, Unigene3931\_JingChaoZuZhi2, Unigene39539\_JingChaoZuZhi2, Unigene40020\_JingChaoZuZhi2, Unigene40825\_JingChaoZuZhi2, Unigene4186\_JingChaoZuZhi2, Unigene42332\_JingChaoZuZhi2, Unigene4470\_JingChaoZuZhi2, Unigene45\_JingChaoZuZhi2, Unigene48368\_JingChaoZuZhi2, Unigene4838\_JingChaoZuZhi2, Unigene48607\_JingChaoZuZhi2, Unigene48751\_JingChaoZuZhi2, Unigene5196\_JingChaoZuZhi2, Unigene5314\_JingChaoZuZhi2, Unigene54320\_JingChaoZuZhi2, Unigene57488\_JingChaoZuZhi2, Unigene58450\_JingChaoZuZhi2, Unigene601\_JingChaoZuZhi2, Unigene6028\_JingChaoZuZhi2, Unigene61108\_JingChaoZuZhi2, Unigene61406\_JingChaoZuZhi2, Unigene6211\_JingChaoZuZhi2, Unigene62824\_JingChaoZuZhi2, Unigene64164\_JingChaoZuZhi2, Unigene64830\_JingChaoZuZhi2, Unigene6493\_JingChaoZuZhi2, Unigene6752\_JingChaoZuZhi2, Unigene67655\_JingChaoZuZhi2, Unigene67793\_JingChaoZuZhi2, Unigene69915\_JingChaoZuZhi2, Unigene70549\_JingChaoZuZhi2, Unigene70723\_JingChaoZuZhi2, Unigene722\_JingChaoZuZhi2, Unigene7417\_JingChaoZuZhi2, Unigene76375\_JingChaoZuZhi2, Unigene76575\_JingChaoZuZhi2, Unigene77570\_JingChaoZuZhi2, Unigene79860\_JingChaoZuZhi2, Unigene8003\_JingChaoZuZhi2, Unigene80331\_JingChaoZuZhi2, Unigene81086\_JingChaoZuZhi2, Unigene81687\_JingChaoZuZhi2, Unigene8191\_JingChaoZuZhi2, Unigene82219\_JingChaoZuZhi2, Unigene85529\_JingChaoZuZhi2, Unigene85562\_JingChaoZuZhi2, Unigene85574\_JingChaoZuZhi2, Unigene86206\_JingChaoZuZhi2, Unigene8621\_JingChaoZuZhi2, Unigene86526\_JingChaoZuZhi2, Unigene86713\_JingChaoZuZhi2, Unigene87332\_JingChaoZuZhi2, Unigene876\_JingChaoZuZhi2, Unigene88719\_JingChaoZuZhi2, Unigene89310\_JingChaoZuZhi2, Unigene8949\_JingChaoZuZhi2, Unigene90762\_JingChaoZuZhi2, Unigene90879\_JingChaoZuZhi2, Unigene91179\_JingChaoZuZhi2, Unigene91612\_JingChaoZuZhi2, Unigene91958\_JingChaoZuZhi2, Unigene92112\_JingChaoZuZhi2, Unigene92372\_JingChaoZuZhi2, Unigene92800\_JingChaoZuZhi2, Unigene93807\_JingChaoZuZhi2, Unigene94149\_JingChaoZuZhi2, Unigene94456\_JingChaoZuZhi2, Unigene9445\_JingChaoZuZhi2, Unigene9461\_JingChaoZuZhi2, Unigene94685\_JingChaoZuZhi2, Unigene94749\_JingChaoZuZhi2, Unigene95098\_JingChaoZuZhi2, Unigene95147\_JingChaoZuZhi2, Unigene95293\_JingChaoZuZhi2, Unigene95295\_JingChaoZuZhi2, Unigene95456\_JingChaoZuZhi2, Unigene95677\_JingChaoZuZhi2, Unigene95875\_JingChaoZuZhi2, Unigene96294\_JingChaoZuZhi2, Unigene9645\_JingChaoZuZhi2, Unigene9902\_JingChaoZuZhi2 |
| 72 | Glycolysis / Gluconeogenesis | Unigene10082\_JingChaoZuZhi2, Unigene1045\_JingChaoZuZhi2, Unigene10514\_JingChaoZuZhi2, Unigene12419\_JingChaoZuZhi2, Unigene12745\_JingChaoZuZhi2, Unigene13096\_JingChaoZuZhi2, Unigene13587\_JingChaoZuZhi2, Unigene13837\_JingChaoZuZhi2, Unigene1393\_JingChaoZuZhi2, Unigene15755\_JingChaoZuZhi2, Unigene15856\_JingChaoZuZhi2, Unigene16107\_JingChaoZuZhi2, Unigene16281\_JingChaoZuZhi2, Unigene16711\_JingChaoZuZhi2, Unigene16762\_JingChaoZuZhi2, Unigene17467\_JingChaoZuZhi2, Unigene18132\_JingChaoZuZhi2, Unigene18358\_JingChaoZuZhi2, Unigene18495\_JingChaoZuZhi2, Unigene18525\_JingChaoZuZhi2, Unigene18776\_JingChaoZuZhi2, Unigene19050\_JingChaoZuZhi2, Unigene1922\_JingChaoZuZhi2, Unigene19750\_JingChaoZuZhi2, Unigene20936\_JingChaoZuZhi2, Unigene21049\_JingChaoZuZhi2, Unigene21122\_JingChaoZuZhi2, Unigene21176\_JingChaoZuZhi2, Unigene22075\_JingChaoZuZhi2, Unigene22143\_JingChaoZuZhi2, Unigene2235\_JingChaoZuZhi2, Unigene23447\_JingChaoZuZhi2, Unigene23700\_JingChaoZuZhi2, Unigene24204\_JingChaoZuZhi2, Unigene2705\_JingChaoZuZhi2, Unigene28123\_JingChaoZuZhi2, Unigene31068\_JingChaoZuZhi2, Unigene32031\_JingChaoZuZhi2, Unigene32472\_JingChaoZuZhi2, Unigene32999\_JingChaoZuZhi2, Unigene37548\_JingChaoZuZhi2, Unigene39661\_JingChaoZuZhi2, Unigene40890\_JingChaoZuZhi2, Unigene40914\_JingChaoZuZhi2, Unigene42887\_JingChaoZuZhi2, Unigene43\_JingChaoZuZhi2, Unigene44195\_JingChaoZuZhi2, Unigene44590\_JingChaoZuZhi2, Unigene46223\_JingChaoZuZhi2, Unigene46382\_JingChaoZuZhi2, Unigene46843\_JingChaoZuZhi2, Unigene4738\_JingChaoZuZhi2, Unigene48004\_JingChaoZuZhi2, Unigene48948\_JingChaoZuZhi2, Unigene490\_JingChaoZuZhi2, Unigene49331\_JingChaoZuZhi2, Unigene50456\_JingChaoZuZhi2, Unigene50485\_JingChaoZuZhi2, Unigene5057\_JingChaoZuZhi2, Unigene51478\_JingChaoZuZhi2, Unigene51848\_JingChaoZuZhi2, Unigene52974\_JingChaoZuZhi2, Unigene53030\_JingChaoZuZhi2, Unigene53240\_JingChaoZuZhi2, Unigene53627\_JingChaoZuZhi2, Unigene54974\_JingChaoZuZhi2, Unigene55009\_JingChaoZuZhi2, Unigene55371\_JingChaoZuZhi2, Unigene56660\_JingChaoZuZhi2, Unigene57635\_JingChaoZuZhi2, Unigene5852\_JingChaoZuZhi2, Unigene59449\_JingChaoZuZhi2, Unigene59731\_JingChaoZuZhi2, Unigene63384\_JingChaoZuZhi2, Unigene63649\_JingChaoZuZhi2, Unigene65498\_JingChaoZuZhi2, Unigene66456\_JingChaoZuZhi2, Unigene67191\_JingChaoZuZhi2, Unigene67613\_JingChaoZuZhi2, Unigene68200\_JingChaoZuZhi2, Unigene68497\_JingChaoZuZhi2, Unigene69092\_JingChaoZuZhi2, Unigene70140\_JingChaoZuZhi2, Unigene72476\_JingChaoZuZhi2, Unigene72674\_JingChaoZuZhi2, Unigene7326\_JingChaoZuZhi2, Unigene75288\_JingChaoZuZhi2, Unigene75529\_JingChaoZuZhi2, Unigene76337\_JingChaoZuZhi2, Unigene76385\_JingChaoZuZhi2, Unigene7641\_JingChaoZuZhi2, Unigene76903\_JingChaoZuZhi2, Unigene77374\_JingChaoZuZhi2, Unigene78168\_JingChaoZuZhi2, Unigene78415\_JingChaoZuZhi2, Unigene79539\_JingChaoZuZhi2, Unigene80504\_JingChaoZuZhi2, Unigene80881\_JingChaoZuZhi2, Unigene81511\_JingChaoZuZhi2, Unigene81825\_JingChaoZuZhi2, Unigene82129\_JingChaoZuZhi2, Unigene83318\_JingChaoZuZhi2, Unigene83440\_JingChaoZuZhi2, Unigene83531\_JingChaoZuZhi2, Unigene84063\_JingChaoZuZhi2, Unigene84701\_JingChaoZuZhi2, Unigene84785\_JingChaoZuZhi2, Unigene84851\_JingChaoZuZhi2, Unigene8508\_JingChaoZuZhi2, Unigene85131\_JingChaoZuZhi2, Unigene85365\_JingChaoZuZhi2, Unigene85769\_JingChaoZuZhi2, Unigene86120\_JingChaoZuZhi2, Unigene86998\_JingChaoZuZhi2, Unigene87044\_JingChaoZuZhi2, Unigene87583\_JingChaoZuZhi2, Unigene87776\_JingChaoZuZhi2, Unigene87853\_JingChaoZuZhi2, Unigene881\_JingChaoZuZhi2, Unigene889\_JingChaoZuZhi2, Unigene89279\_JingChaoZuZhi2, Unigene89788\_JingChaoZuZhi2, Unigene90034\_JingChaoZuZhi2, Unigene9006\_JingChaoZuZhi2, Unigene90127\_JingChaoZuZhi2, Unigene90875\_JingChaoZuZhi2, Unigene91175\_JingChaoZuZhi2, Unigene91700\_JingChaoZuZhi2, Unigene91948\_JingChaoZuZhi2, Unigene92815\_JingChaoZuZhi2, Unigene93212\_JingChaoZuZhi2, Unigene93334\_JingChaoZuZhi2, Unigene93422\_JingChaoZuZhi2, Unigene93710\_JingChaoZuZhi2, Unigene93928\_JingChaoZuZhi2, Unigene94353\_JingChaoZuZhi2, Unigene94565\_JingChaoZuZhi2, Unigene94808\_JingChaoZuZhi2, Unigene94837\_JingChaoZuZhi2, Unigene95296\_JingChaoZuZhi2, Unigene95350\_JingChaoZuZhi2, Unigene95445\_JingChaoZuZhi2, Unigene95561\_JingChaoZuZhi2, Unigene95616\_JingChaoZuZhi2, Unigene95668\_JingChaoZuZhi2, Unigene95744\_JingChaoZuZhi2, Unigene95959\_JingChaoZuZhi2, Unigene95978\_JingChaoZuZhi2, Unigene95985\_JingChaoZuZhi2, Unigene96031\_JingChaoZuZhi2, Unigene96220\_JingChaoZuZhi2 |
| 73 | Renal cell carcinoma | Unigene10031\_JingChaoZuZhi2, Unigene10053\_JingChaoZuZhi2, Unigene10218\_JingChaoZuZhi2, Unigene10770\_JingChaoZuZhi2, Unigene11564\_JingChaoZuZhi2, Unigene11961\_JingChaoZuZhi2, Unigene12420\_JingChaoZuZhi2, Unigene12634\_JingChaoZuZhi2, Unigene12813\_JingChaoZuZhi2, Unigene13354\_JingChaoZuZhi2, Unigene13927\_JingChaoZuZhi2, Unigene14738\_JingChaoZuZhi2, Unigene14912\_JingChaoZuZhi2, Unigene15095\_JingChaoZuZhi2, Unigene15228\_JingChaoZuZhi2, Unigene1554\_JingChaoZuZhi2, Unigene16468\_JingChaoZuZhi2, Unigene16958\_JingChaoZuZhi2, Unigene17247\_JingChaoZuZhi2, Unigene17829\_JingChaoZuZhi2, Unigene17946\_JingChaoZuZhi2, Unigene18493\_JingChaoZuZhi2, Unigene18609\_JingChaoZuZhi2, Unigene18672\_JingChaoZuZhi2, Unigene18715\_JingChaoZuZhi2, Unigene19021\_JingChaoZuZhi2, Unigene19238\_JingChaoZuZhi2, Unigene20052\_JingChaoZuZhi2, Unigene21513\_JingChaoZuZhi2, Unigene21756\_JingChaoZuZhi2, Unigene22043\_JingChaoZuZhi2, Unigene22174\_JingChaoZuZhi2, Unigene22482\_JingChaoZuZhi2, Unigene22580\_JingChaoZuZhi2, Unigene22585\_JingChaoZuZhi2, Unigene22673\_JingChaoZuZhi2, Unigene22708\_JingChaoZuZhi2, Unigene22737\_JingChaoZuZhi2, Unigene22804\_JingChaoZuZhi2, Unigene24037\_JingChaoZuZhi2, Unigene2456\_JingChaoZuZhi2, Unigene2473\_JingChaoZuZhi2, Unigene30386\_JingChaoZuZhi2, Unigene35730\_JingChaoZuZhi2, Unigene3873\_JingChaoZuZhi2, Unigene3931\_JingChaoZuZhi2, Unigene395\_JingChaoZuZhi2, Unigene39825\_JingChaoZuZhi2, Unigene40410\_JingChaoZuZhi2, Unigene4186\_JingChaoZuZhi2, Unigene4223\_JingChaoZuZhi2, Unigene44927\_JingChaoZuZhi2, Unigene4592\_JingChaoZuZhi2, Unigene46240\_JingChaoZuZhi2, Unigene48357\_JingChaoZuZhi2, Unigene4838\_JingChaoZuZhi2, Unigene48751\_JingChaoZuZhi2, Unigene49913\_JingChaoZuZhi2, Unigene51966\_JingChaoZuZhi2, Unigene5437\_JingChaoZuZhi2, Unigene5638\_JingChaoZuZhi2, Unigene57488\_JingChaoZuZhi2, Unigene58005\_JingChaoZuZhi2, Unigene58474\_JingChaoZuZhi2, Unigene59849\_JingChaoZuZhi2, Unigene6028\_JingChaoZuZhi2, Unigene60762\_JingChaoZuZhi2, Unigene62851\_JingChaoZuZhi2, Unigene63323\_JingChaoZuZhi2, Unigene63406\_JingChaoZuZhi2, Unigene6467\_JingChaoZuZhi2, Unigene6493\_JingChaoZuZhi2, Unigene66013\_JingChaoZuZhi2, Unigene6676\_JingChaoZuZhi2, Unigene66813\_JingChaoZuZhi2, Unigene67793\_JingChaoZuZhi2, Unigene70549\_JingChaoZuZhi2, Unigene7135\_JingChaoZuZhi2, Unigene72925\_JingChaoZuZhi2, Unigene74040\_JingChaoZuZhi2, Unigene74609\_JingChaoZuZhi2, Unigene75859\_JingChaoZuZhi2, Unigene76400\_JingChaoZuZhi2, Unigene76639\_JingChaoZuZhi2, Unigene78217\_JingChaoZuZhi2, Unigene78522\_JingChaoZuZhi2, Unigene79307\_JingChaoZuZhi2, Unigene79882\_JingChaoZuZhi2, Unigene80123\_JingChaoZuZhi2, Unigene81716\_JingChaoZuZhi2, Unigene8191\_JingChaoZuZhi2, Unigene82375\_JingChaoZuZhi2, Unigene82606\_JingChaoZuZhi2, Unigene82675\_JingChaoZuZhi2, Unigene83188\_JingChaoZuZhi2, Unigene84220\_JingChaoZuZhi2, Unigene84862\_JingChaoZuZhi2, Unigene84944\_JingChaoZuZhi2, Unigene85574\_JingChaoZuZhi2, Unigene85821\_JingChaoZuZhi2, Unigene86036\_JingChaoZuZhi2, Unigene86115\_JingChaoZuZhi2, Unigene86119\_JingChaoZuZhi2, Unigene86206\_JingChaoZuZhi2, Unigene86504\_JingChaoZuZhi2, Unigene86526\_JingChaoZuZhi2, Unigene87251\_JingChaoZuZhi2, Unigene87321\_JingChaoZuZhi2, Unigene876\_JingChaoZuZhi2, Unigene87851\_JingChaoZuZhi2, Unigene89093\_JingChaoZuZhi2, Unigene89161\_JingChaoZuZhi2, Unigene90073\_JingChaoZuZhi2, Unigene91038\_JingChaoZuZhi2, Unigene91119\_JingChaoZuZhi2, Unigene91179\_JingChaoZuZhi2, Unigene91284\_JingChaoZuZhi2, Unigene91612\_JingChaoZuZhi2, Unigene91958\_JingChaoZuZhi2, Unigene92112\_JingChaoZuZhi2, Unigene92119\_JingChaoZuZhi2, Unigene92715\_JingChaoZuZhi2, Unigene93195\_JingChaoZuZhi2, Unigene93502\_JingChaoZuZhi2, Unigene93782\_JingChaoZuZhi2, Unigene93807\_JingChaoZuZhi2, Unigene94149\_JingChaoZuZhi2, Unigene94208\_JingChaoZuZhi2, Unigene94242\_JingChaoZuZhi2, Unigene9428\_JingChaoZuZhi2, Unigene94456\_JingChaoZuZhi2, Unigene94474\_JingChaoZuZhi2, Unigene94544\_JingChaoZuZhi2, Unigene94685\_JingChaoZuZhi2, Unigene94711\_JingChaoZuZhi2, Unigene95378\_JingChaoZuZhi2, Unigene95456\_JingChaoZuZhi2, Unigene95505\_JingChaoZuZhi2, Unigene95568\_JingChaoZuZhi2, Unigene95875\_JingChaoZuZhi2, Unigene95933\_JingChaoZuZhi2, Unigene96044\_JingChaoZuZhi2, Unigene96124\_JingChaoZuZhi2, Unigene96294\_JingChaoZuZhi2, Unigene9767\_JingChaoZuZhi2, Unigene9902\_JingChaoZuZhi2 |
| 74 | ErbB signaling pathway | Unigene10053\_JingChaoZuZhi2, Unigene10318\_JingChaoZuZhi2, Unigene10422\_JingChaoZuZhi2, Unigene10532\_JingChaoZuZhi2, Unigene10926\_JingChaoZuZhi2, Unigene11251\_JingChaoZuZhi2, Unigene11951\_JingChaoZuZhi2, Unigene11961\_JingChaoZuZhi2, Unigene12420\_JingChaoZuZhi2, Unigene12595\_JingChaoZuZhi2, Unigene12823\_JingChaoZuZhi2, Unigene13510\_JingChaoZuZhi2, Unigene13927\_JingChaoZuZhi2, Unigene14289\_JingChaoZuZhi2, Unigene14912\_JingChaoZuZhi2, Unigene15095\_JingChaoZuZhi2, Unigene15228\_JingChaoZuZhi2, Unigene16820\_JingChaoZuZhi2, Unigene17247\_JingChaoZuZhi2, Unigene17764\_JingChaoZuZhi2, Unigene17829\_JingChaoZuZhi2, Unigene18464\_JingChaoZuZhi2, Unigene18672\_JingChaoZuZhi2, Unigene18715\_JingChaoZuZhi2, Unigene18868\_JingChaoZuZhi2, Unigene18986\_JingChaoZuZhi2, Unigene19046\_JingChaoZuZhi2, Unigene19307\_JingChaoZuZhi2, Unigene19551\_JingChaoZuZhi2, Unigene20794\_JingChaoZuZhi2, Unigene20840\_JingChaoZuZhi2, Unigene21010\_JingChaoZuZhi2, Unigene21045\_JingChaoZuZhi2, Unigene21295\_JingChaoZuZhi2, Unigene21513\_JingChaoZuZhi2, Unigene21515\_JingChaoZuZhi2, Unigene21578\_JingChaoZuZhi2, Unigene21605\_JingChaoZuZhi2, Unigene21691\_JingChaoZuZhi2, Unigene22174\_JingChaoZuZhi2, Unigene22482\_JingChaoZuZhi2, Unigene22500\_JingChaoZuZhi2, Unigene22737\_JingChaoZuZhi2, Unigene22779\_JingChaoZuZhi2, Unigene22804\_JingChaoZuZhi2, Unigene2456\_JingChaoZuZhi2, Unigene26730\_JingChaoZuZhi2, Unigene2687\_JingChaoZuZhi2, Unigene2790\_JingChaoZuZhi2, Unigene2960\_JingChaoZuZhi2, Unigene3931\_JingChaoZuZhi2, Unigene4186\_JingChaoZuZhi2, Unigene4294\_JingChaoZuZhi2, Unigene4838\_JingChaoZuZhi2, Unigene48751\_JingChaoZuZhi2, Unigene49798\_JingChaoZuZhi2, Unigene51966\_JingChaoZuZhi2, Unigene53917\_JingChaoZuZhi2, Unigene53958\_JingChaoZuZhi2, Unigene54470\_JingChaoZuZhi2, Unigene57488\_JingChaoZuZhi2, Unigene6028\_JingChaoZuZhi2, Unigene61562\_JingChaoZuZhi2, Unigene6159\_JingChaoZuZhi2, Unigene62066\_JingChaoZuZhi2, Unigene62786\_JingChaoZuZhi2, Unigene64164\_JingChaoZuZhi2, Unigene64650\_JingChaoZuZhi2, Unigene64830\_JingChaoZuZhi2, Unigene6493\_JingChaoZuZhi2, Unigene66013\_JingChaoZuZhi2, Unigene66060\_JingChaoZuZhi2, Unigene6676\_JingChaoZuZhi2, Unigene66813\_JingChaoZuZhi2, Unigene67655\_JingChaoZuZhi2, Unigene67793\_JingChaoZuZhi2, Unigene6785\_JingChaoZuZhi2, Unigene70549\_JingChaoZuZhi2, Unigene70723\_JingChaoZuZhi2, Unigene72521\_JingChaoZuZhi2, Unigene75859\_JingChaoZuZhi2, Unigene76023\_JingChaoZuZhi2, Unigene76809\_JingChaoZuZhi2, Unigene76917\_JingChaoZuZhi2, Unigene77061\_JingChaoZuZhi2, Unigene78574\_JingChaoZuZhi2, Unigene78909\_JingChaoZuZhi2, Unigene7939\_JingChaoZuZhi2, Unigene79529\_JingChaoZuZhi2, Unigene79882\_JingChaoZuZhi2, Unigene8191\_JingChaoZuZhi2, Unigene81948\_JingChaoZuZhi2, Unigene82073\_JingChaoZuZhi2, Unigene84944\_JingChaoZuZhi2, Unigene8507\_JingChaoZuZhi2, Unigene85172\_JingChaoZuZhi2, Unigene85574\_JingChaoZuZhi2, Unigene86206\_JingChaoZuZhi2, Unigene86526\_JingChaoZuZhi2, Unigene87332\_JingChaoZuZhi2, Unigene876\_JingChaoZuZhi2, Unigene87851\_JingChaoZuZhi2, Unigene88185\_JingChaoZuZhi2, Unigene88391\_JingChaoZuZhi2, Unigene88719\_JingChaoZuZhi2, Unigene89310\_JingChaoZuZhi2, Unigene89722\_JingChaoZuZhi2, Unigene90979\_JingChaoZuZhi2, Unigene91538\_JingChaoZuZhi2, Unigene91612\_JingChaoZuZhi2, Unigene91689\_JingChaoZuZhi2, Unigene91958\_JingChaoZuZhi2, Unigene92112\_JingChaoZuZhi2, Unigene93409\_JingChaoZuZhi2, Unigene93793\_JingChaoZuZhi2, Unigene93807\_JingChaoZuZhi2, Unigene9404\_JingChaoZuZhi2, Unigene94138\_JingChaoZuZhi2, Unigene94149\_JingChaoZuZhi2, Unigene94208\_JingChaoZuZhi2, Unigene94456\_JingChaoZuZhi2, Unigene94544\_JingChaoZuZhi2, Unigene9461\_JingChaoZuZhi2, Unigene94685\_JingChaoZuZhi2, Unigene9488\_JingChaoZuZhi2, Unigene95098\_JingChaoZuZhi2, Unigene95295\_JingChaoZuZhi2, Unigene95456\_JingChaoZuZhi2, Unigene95677\_JingChaoZuZhi2, Unigene95921\_JingChaoZuZhi2, Unigene95951\_JingChaoZuZhi2, Unigene96294\_JingChaoZuZhi2, Unigene9902\_JingChaoZuZhi2 |
| 75 | Chagas disease | Unigene11592\_JingChaoZuZhi2, Unigene11621\_JingChaoZuZhi2, Unigene11951\_JingChaoZuZhi2, Unigene11961\_JingChaoZuZhi2, Unigene12161\_JingChaoZuZhi2, Unigene12337\_JingChaoZuZhi2, Unigene1298\_JingChaoZuZhi2, Unigene13650\_JingChaoZuZhi2, Unigene13770\_JingChaoZuZhi2, Unigene14109\_JingChaoZuZhi2, Unigene14360\_JingChaoZuZhi2, Unigene14842\_JingChaoZuZhi2, Unigene14912\_JingChaoZuZhi2, Unigene15228\_JingChaoZuZhi2, Unigene15651\_JingChaoZuZhi2, Unigene16764\_JingChaoZuZhi2, Unigene16991\_JingChaoZuZhi2, Unigene17062\_JingChaoZuZhi2, Unigene17178\_JingChaoZuZhi2, Unigene17247\_JingChaoZuZhi2, Unigene1829\_JingChaoZuZhi2, Unigene18515\_JingChaoZuZhi2, Unigene18672\_JingChaoZuZhi2, Unigene18715\_JingChaoZuZhi2, Unigene19813\_JingChaoZuZhi2, Unigene20120\_JingChaoZuZhi2, Unigene20157\_JingChaoZuZhi2, Unigene20991\_JingChaoZuZhi2, Unigene21472\_JingChaoZuZhi2, Unigene21691\_JingChaoZuZhi2, Unigene21697\_JingChaoZuZhi2, Unigene22293\_JingChaoZuZhi2, Unigene22466\_JingChaoZuZhi2, Unigene24425\_JingChaoZuZhi2, Unigene2456\_JingChaoZuZhi2, Unigene25870\_JingChaoZuZhi2, Unigene28427\_JingChaoZuZhi2, Unigene29179\_JingChaoZuZhi2, Unigene32827\_JingChaoZuZhi2, Unigene32999\_JingChaoZuZhi2, Unigene34929\_JingChaoZuZhi2, Unigene35574\_JingChaoZuZhi2, Unigene3640\_JingChaoZuZhi2, Unigene3931\_JingChaoZuZhi2, Unigene39577\_JingChaoZuZhi2, Unigene39832\_JingChaoZuZhi2, Unigene41333\_JingChaoZuZhi2, Unigene4294\_JingChaoZuZhi2, Unigene43059\_JingChaoZuZhi2, Unigene44224\_JingChaoZuZhi2, Unigene45948\_JingChaoZuZhi2, Unigene46219\_JingChaoZuZhi2, Unigene47155\_JingChaoZuZhi2, Unigene47872\_JingChaoZuZhi2, Unigene48751\_JingChaoZuZhi2, Unigene5099\_JingChaoZuZhi2, Unigene51705\_JingChaoZuZhi2, Unigene52526\_JingChaoZuZhi2, Unigene5314\_JingChaoZuZhi2, Unigene54066\_JingChaoZuZhi2, Unigene54320\_JingChaoZuZhi2, Unigene54367\_JingChaoZuZhi2, Unigene54569\_JingChaoZuZhi2, Unigene56775\_JingChaoZuZhi2, Unigene57488\_JingChaoZuZhi2, Unigene58550\_JingChaoZuZhi2, Unigene59750\_JingChaoZuZhi2, Unigene63226\_JingChaoZuZhi2, Unigene6459\_JingChaoZuZhi2, Unigene65294\_JingChaoZuZhi2, Unigene65445\_JingChaoZuZhi2, Unigene65447\_JingChaoZuZhi2, Unigene66540\_JingChaoZuZhi2, Unigene66772\_JingChaoZuZhi2, Unigene67676\_JingChaoZuZhi2, Unigene68108\_JingChaoZuZhi2, Unigene68200\_JingChaoZuZhi2, Unigene69071\_JingChaoZuZhi2, Unigene70493\_JingChaoZuZhi2, Unigene70505\_JingChaoZuZhi2, Unigene72036\_JingChaoZuZhi2, Unigene72445\_JingChaoZuZhi2, Unigene72643\_JingChaoZuZhi2, Unigene7373\_JingChaoZuZhi2, Unigene73882\_JingChaoZuZhi2, Unigene74569\_JingChaoZuZhi2, Unigene75235\_JingChaoZuZhi2, Unigene77149\_JingChaoZuZhi2, Unigene7747\_JingChaoZuZhi2, Unigene77570\_JingChaoZuZhi2, Unigene78078\_JingChaoZuZhi2, Unigene78255\_JingChaoZuZhi2, Unigene78367\_JingChaoZuZhi2, Unigene78957\_JingChaoZuZhi2, Unigene8191\_JingChaoZuZhi2, Unigene8202\_JingChaoZuZhi2, Unigene8248\_JingChaoZuZhi2, Unigene83017\_JingChaoZuZhi2, Unigene83196\_JingChaoZuZhi2, Unigene85615\_JingChaoZuZhi2, Unigene85880\_JingChaoZuZhi2, Unigene8600\_JingChaoZuZhi2, Unigene86091\_JingChaoZuZhi2, Unigene86206\_JingChaoZuZhi2, Unigene86526\_JingChaoZuZhi2, Unigene87082\_JingChaoZuZhi2, Unigene87261\_JingChaoZuZhi2, Unigene87607\_JingChaoZuZhi2, Unigene8784\_JingChaoZuZhi2, Unigene88385\_JingChaoZuZhi2, Unigene88708\_JingChaoZuZhi2, Unigene88773\_JingChaoZuZhi2, Unigene88942\_JingChaoZuZhi2, Unigene89323\_JingChaoZuZhi2, Unigene91221\_JingChaoZuZhi2, Unigene91661\_JingChaoZuZhi2, Unigene91739\_JingChaoZuZhi2, Unigene92019\_JingChaoZuZhi2, Unigene92112\_JingChaoZuZhi2, Unigene93012\_JingChaoZuZhi2, Unigene93754\_JingChaoZuZhi2, Unigene93807\_JingChaoZuZhi2, Unigene94225\_JingChaoZuZhi2, Unigene94388\_JingChaoZuZhi2, Unigene94619\_JingChaoZuZhi2, Unigene94685\_JingChaoZuZhi2, Unigene95163\_JingChaoZuZhi2, Unigene95883\_JingChaoZuZhi2, Unigene95916\_JingChaoZuZhi2, Unigene96262\_JingChaoZuZhi2, Unigene96294\_JingChaoZuZhi2, Unigene9654\_JingChaoZuZhi2 |
| 76 | Cell adhesion molecules (CAMs) | Unigene10001\_JingChaoZuZhi2, Unigene10541\_JingChaoZuZhi2, Unigene11072\_JingChaoZuZhi2, Unigene1111\_JingChaoZuZhi2, Unigene1126\_JingChaoZuZhi2, Unigene11333\_JingChaoZuZhi2, Unigene11371\_JingChaoZuZhi2, Unigene11446\_JingChaoZuZhi2, Unigene11579\_JingChaoZuZhi2, Unigene11866\_JingChaoZuZhi2, Unigene118\_JingChaoZuZhi2, Unigene12174\_JingChaoZuZhi2, Unigene1228\_JingChaoZuZhi2, Unigene12428\_JingChaoZuZhi2, Unigene12889\_JingChaoZuZhi2, Unigene13026\_JingChaoZuZhi2, Unigene13417\_JingChaoZuZhi2, Unigene13541\_JingChaoZuZhi2, Unigene13642\_JingChaoZuZhi2, Unigene13680\_JingChaoZuZhi2, Unigene1372\_JingChaoZuZhi2, Unigene14208\_JingChaoZuZhi2, Unigene14417\_JingChaoZuZhi2, Unigene15113\_JingChaoZuZhi2, Unigene16048\_JingChaoZuZhi2, Unigene16121\_JingChaoZuZhi2, Unigene16152\_JingChaoZuZhi2, Unigene16873\_JingChaoZuZhi2, Unigene17157\_JingChaoZuZhi2, Unigene17508\_JingChaoZuZhi2, Unigene17934\_JingChaoZuZhi2, Unigene18107\_JingChaoZuZhi2, Unigene18153\_JingChaoZuZhi2, Unigene18608\_JingChaoZuZhi2, Unigene18706\_JingChaoZuZhi2, Unigene18768\_JingChaoZuZhi2, Unigene18808\_JingChaoZuZhi2, Unigene19041\_JingChaoZuZhi2, Unigene19404\_JingChaoZuZhi2, Unigene19423\_JingChaoZuZhi2, Unigene19678\_JingChaoZuZhi2, Unigene19917\_JingChaoZuZhi2, Unigene20022\_JingChaoZuZhi2, Unigene2028\_JingChaoZuZhi2, Unigene21156\_JingChaoZuZhi2, Unigene21505\_JingChaoZuZhi2, Unigene22145\_JingChaoZuZhi2, Unigene22517\_JingChaoZuZhi2, Unigene22794\_JingChaoZuZhi2, Unigene26820\_JingChaoZuZhi2, Unigene29201\_JingChaoZuZhi2, Unigene30120\_JingChaoZuZhi2, Unigene32805\_JingChaoZuZhi2, Unigene33187\_JingChaoZuZhi2, Unigene33245\_JingChaoZuZhi2, Unigene3356\_JingChaoZuZhi2, Unigene35243\_JingChaoZuZhi2, Unigene36170\_JingChaoZuZhi2, Unigene3665\_JingChaoZuZhi2, Unigene38931\_JingChaoZuZhi2, Unigene3908\_JingChaoZuZhi2, Unigene42836\_JingChaoZuZhi2, Unigene43427\_JingChaoZuZhi2, Unigene44535\_JingChaoZuZhi2, Unigene4479\_JingChaoZuZhi2, Unigene45468\_JingChaoZuZhi2, Unigene45782\_JingChaoZuZhi2, Unigene48466\_JingChaoZuZhi2, Unigene48506\_JingChaoZuZhi2, Unigene48843\_JingChaoZuZhi2, Unigene5069\_JingChaoZuZhi2, Unigene5445\_JingChaoZuZhi2, Unigene55684\_JingChaoZuZhi2, Unigene56158\_JingChaoZuZhi2, Unigene57247\_JingChaoZuZhi2, Unigene57566\_JingChaoZuZhi2, Unigene58136\_JingChaoZuZhi2, Unigene58386\_JingChaoZuZhi2, Unigene60370\_JingChaoZuZhi2, Unigene60401\_JingChaoZuZhi2, Unigene60650\_JingChaoZuZhi2, Unigene6183\_JingChaoZuZhi2, Unigene62781\_JingChaoZuZhi2, Unigene64060\_JingChaoZuZhi2, Unigene67542\_JingChaoZuZhi2, Unigene69708\_JingChaoZuZhi2, Unigene71055\_JingChaoZuZhi2, Unigene71891\_JingChaoZuZhi2, Unigene71941\_JingChaoZuZhi2, Unigene7204\_JingChaoZuZhi2, Unigene73475\_JingChaoZuZhi2, Unigene73579\_JingChaoZuZhi2, Unigene7364\_JingChaoZuZhi2, Unigene74084\_JingChaoZuZhi2, Unigene75840\_JingChaoZuZhi2, Unigene76044\_JingChaoZuZhi2, Unigene76375\_JingChaoZuZhi2, Unigene76415\_JingChaoZuZhi2, Unigene77067\_JingChaoZuZhi2, Unigene77738\_JingChaoZuZhi2, Unigene79071\_JingChaoZuZhi2, Unigene799\_JingChaoZuZhi2, Unigene80507\_JingChaoZuZhi2, Unigene81558\_JingChaoZuZhi2, Unigene81598\_JingChaoZuZhi2, Unigene81615\_JingChaoZuZhi2, Unigene84519\_JingChaoZuZhi2, Unigene85175\_JingChaoZuZhi2, Unigene8568\_JingChaoZuZhi2, Unigene85944\_JingChaoZuZhi2, Unigene86786\_JingChaoZuZhi2, Unigene87670\_JingChaoZuZhi2, Unigene87717\_JingChaoZuZhi2, Unigene87912\_JingChaoZuZhi2, Unigene8851\_JingChaoZuZhi2, Unigene88551\_JingChaoZuZhi2, Unigene8855\_JingChaoZuZhi2, Unigene89759\_JingChaoZuZhi2, Unigene91043\_JingChaoZuZhi2, Unigene92104\_JingChaoZuZhi2, Unigene92170\_JingChaoZuZhi2, Unigene92235\_JingChaoZuZhi2, Unigene92406\_JingChaoZuZhi2, Unigene93729\_JingChaoZuZhi2, Unigene93779\_JingChaoZuZhi2, Unigene94130\_JingChaoZuZhi2, Unigene94561\_JingChaoZuZhi2, Unigene9530\_JingChaoZuZhi2, Unigene95317\_JingChaoZuZhi2, Unigene96016\_JingChaoZuZhi2, Unigene96121\_JingChaoZuZhi2, Unigene96304\_JingChaoZuZhi2 |
| 77 | Basal transcription factors | Unigene10362\_JingChaoZuZhi2, Unigene10558\_JingChaoZuZhi2, Unigene10585\_JingChaoZuZhi2, Unigene12244\_JingChaoZuZhi2, Unigene12881\_JingChaoZuZhi2, Unigene13945\_JingChaoZuZhi2, Unigene14151\_JingChaoZuZhi2, Unigene14333\_JingChaoZuZhi2, Unigene1433\_JingChaoZuZhi2, Unigene14634\_JingChaoZuZhi2, Unigene14865\_JingChaoZuZhi2, Unigene15075\_JingChaoZuZhi2, Unigene15627\_JingChaoZuZhi2, Unigene1625\_JingChaoZuZhi2, Unigene17190\_JingChaoZuZhi2, Unigene17269\_JingChaoZuZhi2, Unigene17357\_JingChaoZuZhi2, Unigene17423\_JingChaoZuZhi2, Unigene17890\_JingChaoZuZhi2, Unigene18140\_JingChaoZuZhi2, Unigene18577\_JingChaoZuZhi2, Unigene18785\_JingChaoZuZhi2, Unigene18909\_JingChaoZuZhi2, Unigene19283\_JingChaoZuZhi2, Unigene19371\_JingChaoZuZhi2, Unigene20553\_JingChaoZuZhi2, Unigene20558\_JingChaoZuZhi2, Unigene20731\_JingChaoZuZhi2, Unigene20896\_JingChaoZuZhi2, Unigene21321\_JingChaoZuZhi2, Unigene21537\_JingChaoZuZhi2, Unigene21938\_JingChaoZuZhi2, Unigene22186\_JingChaoZuZhi2, Unigene22196\_JingChaoZuZhi2, Unigene22383\_JingChaoZuZhi2, Unigene2239\_JingChaoZuZhi2, Unigene22725\_JingChaoZuZhi2, Unigene22887\_JingChaoZuZhi2, Unigene23468\_JingChaoZuZhi2, Unigene246\_JingChaoZuZhi2, Unigene26108\_JingChaoZuZhi2, Unigene2891\_JingChaoZuZhi2, Unigene29734\_JingChaoZuZhi2, Unigene3054\_JingChaoZuZhi2, Unigene30634\_JingChaoZuZhi2, Unigene34964\_JingChaoZuZhi2, Unigene35457\_JingChaoZuZhi2, Unigene35680\_JingChaoZuZhi2, Unigene3632\_JingChaoZuZhi2, Unigene3650\_JingChaoZuZhi2, Unigene3895\_JingChaoZuZhi2, Unigene3896\_JingChaoZuZhi2, Unigene4064\_JingChaoZuZhi2, Unigene4242\_JingChaoZuZhi2, Unigene43821\_JingChaoZuZhi2, Unigene46282\_JingChaoZuZhi2, Unigene4829\_JingChaoZuZhi2, Unigene49204\_JingChaoZuZhi2, Unigene5064\_JingChaoZuZhi2, Unigene5188\_JingChaoZuZhi2, Unigene5321\_JingChaoZuZhi2, Unigene54171\_JingChaoZuZhi2, Unigene56709\_JingChaoZuZhi2, Unigene59197\_JingChaoZuZhi2, Unigene6135\_JingChaoZuZhi2, Unigene6179\_JingChaoZuZhi2, Unigene62011\_JingChaoZuZhi2, Unigene63949\_JingChaoZuZhi2, Unigene65063\_JingChaoZuZhi2, Unigene6623\_JingChaoZuZhi2, Unigene66795\_JingChaoZuZhi2, Unigene67367\_JingChaoZuZhi2, Unigene67492\_JingChaoZuZhi2, Unigene68298\_JingChaoZuZhi2, Unigene6838\_JingChaoZuZhi2, Unigene68411\_JingChaoZuZhi2, Unigene69161\_JingChaoZuZhi2, Unigene7219\_JingChaoZuZhi2, Unigene72771\_JingChaoZuZhi2, Unigene73890\_JingChaoZuZhi2, Unigene74684\_JingChaoZuZhi2, Unigene74892\_JingChaoZuZhi2, Unigene74943\_JingChaoZuZhi2, Unigene75289\_JingChaoZuZhi2, Unigene75400\_JingChaoZuZhi2, Unigene7850\_JingChaoZuZhi2, Unigene78673\_JingChaoZuZhi2, Unigene80880\_JingChaoZuZhi2, Unigene80902\_JingChaoZuZhi2, Unigene828\_JingChaoZuZhi2, Unigene84241\_JingChaoZuZhi2, Unigene84346\_JingChaoZuZhi2, Unigene84399\_JingChaoZuZhi2, Unigene85734\_JingChaoZuZhi2, Unigene86219\_JingChaoZuZhi2, Unigene86228\_JingChaoZuZhi2, Unigene86749\_JingChaoZuZhi2, Unigene86908\_JingChaoZuZhi2, Unigene86938\_JingChaoZuZhi2, Unigene87231\_JingChaoZuZhi2, Unigene87740\_JingChaoZuZhi2, Unigene87741\_JingChaoZuZhi2, Unigene87792\_JingChaoZuZhi2, Unigene87997\_JingChaoZuZhi2, Unigene88370\_JingChaoZuZhi2, Unigene91157\_JingChaoZuZhi2, Unigene91431\_JingChaoZuZhi2, Unigene91702\_JingChaoZuZhi2, Unigene91944\_JingChaoZuZhi2, Unigene92668\_JingChaoZuZhi2, Unigene92846\_JingChaoZuZhi2, Unigene93176\_JingChaoZuZhi2, Unigene93296\_JingChaoZuZhi2, Unigene93498\_JingChaoZuZhi2, Unigene93533\_JingChaoZuZhi2, Unigene93703\_JingChaoZuZhi2, Unigene93802\_JingChaoZuZhi2, Unigene93812\_JingChaoZuZhi2, Unigene94114\_JingChaoZuZhi2, Unigene94190\_JingChaoZuZhi2, Unigene94790\_JingChaoZuZhi2, Unigene94985\_JingChaoZuZhi2, Unigene95105\_JingChaoZuZhi2, Unigene95212\_JingChaoZuZhi2, Unigene95411\_JingChaoZuZhi2, Unigene95431\_JingChaoZuZhi2, Unigene95669\_JingChaoZuZhi2, Unigene95858\_JingChaoZuZhi2 |
| 78 | Glycerophospholipid metabolism | Unigene10782\_JingChaoZuZhi2, Unigene11055\_JingChaoZuZhi2, Unigene11721\_JingChaoZuZhi2, Unigene12083\_JingChaoZuZhi2, Unigene12154\_JingChaoZuZhi2, Unigene12260\_JingChaoZuZhi2, Unigene12639\_JingChaoZuZhi2, Unigene12860\_JingChaoZuZhi2, Unigene13420\_JingChaoZuZhi2, Unigene13869\_JingChaoZuZhi2, Unigene13877\_JingChaoZuZhi2, Unigene13946\_JingChaoZuZhi2, Unigene14041\_JingChaoZuZhi2, Unigene14357\_JingChaoZuZhi2, Unigene15216\_JingChaoZuZhi2, Unigene15425\_JingChaoZuZhi2, Unigene15942\_JingChaoZuZhi2, Unigene16106\_JingChaoZuZhi2, Unigene16594\_JingChaoZuZhi2, Unigene19186\_JingChaoZuZhi2, Unigene19296\_JingChaoZuZhi2, Unigene19539\_JingChaoZuZhi2, Unigene19904\_JingChaoZuZhi2, Unigene19938\_JingChaoZuZhi2, Unigene20071\_JingChaoZuZhi2, Unigene20192\_JingChaoZuZhi2, Unigene2058\_JingChaoZuZhi2, Unigene20760\_JingChaoZuZhi2, Unigene20979\_JingChaoZuZhi2, Unigene21407\_JingChaoZuZhi2, Unigene22211\_JingChaoZuZhi2, Unigene22257\_JingChaoZuZhi2, Unigene22498\_JingChaoZuZhi2, Unigene2275\_JingChaoZuZhi2, Unigene25108\_JingChaoZuZhi2, Unigene2514\_JingChaoZuZhi2, Unigene27353\_JingChaoZuZhi2, Unigene28252\_JingChaoZuZhi2, Unigene3700\_JingChaoZuZhi2, Unigene37689\_JingChaoZuZhi2, Unigene4160\_JingChaoZuZhi2, Unigene42056\_JingChaoZuZhi2, Unigene42329\_JingChaoZuZhi2, Unigene43723\_JingChaoZuZhi2, Unigene44246\_JingChaoZuZhi2, Unigene45532\_JingChaoZuZhi2, Unigene45669\_JingChaoZuZhi2, Unigene46680\_JingChaoZuZhi2, Unigene48098\_JingChaoZuZhi2, Unigene49664\_JingChaoZuZhi2, Unigene5027\_JingChaoZuZhi2, Unigene51678\_JingChaoZuZhi2, Unigene5182\_JingChaoZuZhi2, Unigene57970\_JingChaoZuZhi2, Unigene59480\_JingChaoZuZhi2, Unigene59837\_JingChaoZuZhi2, Unigene60117\_JingChaoZuZhi2, Unigene6117\_JingChaoZuZhi2, Unigene62892\_JingChaoZuZhi2, Unigene63968\_JingChaoZuZhi2, Unigene64489\_JingChaoZuZhi2, Unigene66246\_JingChaoZuZhi2, Unigene66533\_JingChaoZuZhi2, Unigene6684\_JingChaoZuZhi2, Unigene67328\_JingChaoZuZhi2, Unigene68151\_JingChaoZuZhi2, Unigene69249\_JingChaoZuZhi2, Unigene70620\_JingChaoZuZhi2, Unigene71078\_JingChaoZuZhi2, Unigene71103\_JingChaoZuZhi2, Unigene71418\_JingChaoZuZhi2, Unigene72016\_JingChaoZuZhi2, Unigene72098\_JingChaoZuZhi2, Unigene72136\_JingChaoZuZhi2, Unigene7242\_JingChaoZuZhi2, Unigene72565\_JingChaoZuZhi2, Unigene7283\_JingChaoZuZhi2, Unigene72974\_JingChaoZuZhi2, Unigene74594\_JingChaoZuZhi2, Unigene74643\_JingChaoZuZhi2, Unigene7493\_JingChaoZuZhi2, Unigene75535\_JingChaoZuZhi2, Unigene76182\_JingChaoZuZhi2, Unigene76480\_JingChaoZuZhi2, Unigene76609\_JingChaoZuZhi2, Unigene78156\_JingChaoZuZhi2, Unigene78226\_JingChaoZuZhi2, Unigene78992\_JingChaoZuZhi2, Unigene79166\_JingChaoZuZhi2, Unigene79362\_JingChaoZuZhi2, Unigene8011\_JingChaoZuZhi2, Unigene80528\_JingChaoZuZhi2, Unigene80629\_JingChaoZuZhi2, Unigene80895\_JingChaoZuZhi2, Unigene81477\_JingChaoZuZhi2, Unigene81575\_JingChaoZuZhi2, Unigene83131\_JingChaoZuZhi2, Unigene83182\_JingChaoZuZhi2, Unigene83218\_JingChaoZuZhi2, Unigene83231\_JingChaoZuZhi2, Unigene83570\_JingChaoZuZhi2, Unigene84021\_JingChaoZuZhi2, Unigene86034\_JingChaoZuZhi2, Unigene86629\_JingChaoZuZhi2, Unigene87893\_JingChaoZuZhi2, Unigene88068\_JingChaoZuZhi2, Unigene89232\_JingChaoZuZhi2, Unigene90131\_JingChaoZuZhi2, Unigene90531\_JingChaoZuZhi2, Unigene90616\_JingChaoZuZhi2, Unigene90914\_JingChaoZuZhi2, Unigene91039\_JingChaoZuZhi2, Unigene91369\_JingChaoZuZhi2, Unigene91798\_JingChaoZuZhi2, Unigene92087\_JingChaoZuZhi2, Unigene92263\_JingChaoZuZhi2, Unigene92394\_JingChaoZuZhi2, Unigene92737\_JingChaoZuZhi2, Unigene93106\_JingChaoZuZhi2, Unigene9319\_JingChaoZuZhi2, Unigene93600\_JingChaoZuZhi2, Unigene93910\_JingChaoZuZhi2, Unigene94274\_JingChaoZuZhi2, Unigene94907\_JingChaoZuZhi2, Unigene95739\_JingChaoZuZhi2, Unigene95974\_JingChaoZuZhi2, Unigene9\_JingChaoZuZhi2 |
| 79 | TGF-beta signaling pathway | Unigene10031\_JingChaoZuZhi2, Unigene10286\_JingChaoZuZhi2, Unigene10532\_JingChaoZuZhi2, Unigene10910\_JingChaoZuZhi2, Unigene11557\_JingChaoZuZhi2, Unigene11985\_JingChaoZuZhi2, Unigene12060\_JingChaoZuZhi2, Unigene12161\_JingChaoZuZhi2, Unigene12634\_JingChaoZuZhi2, Unigene14289\_JingChaoZuZhi2, Unigene14361\_JingChaoZuZhi2, Unigene16157\_JingChaoZuZhi2, Unigene16468\_JingChaoZuZhi2, Unigene16700\_JingChaoZuZhi2, Unigene16728\_JingChaoZuZhi2, Unigene16735\_JingChaoZuZhi2, Unigene16958\_JingChaoZuZhi2, Unigene17474\_JingChaoZuZhi2, Unigene1813\_JingChaoZuZhi2, Unigene18309\_JingChaoZuZhi2, Unigene1838\_JingChaoZuZhi2, Unigene19238\_JingChaoZuZhi2, Unigene19422\_JingChaoZuZhi2, Unigene19813\_JingChaoZuZhi2, Unigene20029\_JingChaoZuZhi2, Unigene20052\_JingChaoZuZhi2, Unigene20220\_JingChaoZuZhi2, Unigene20297\_JingChaoZuZhi2, Unigene20489\_JingChaoZuZhi2, Unigene20608\_JingChaoZuZhi2, Unigene20755\_JingChaoZuZhi2, Unigene21088\_JingChaoZuZhi2, Unigene21385\_JingChaoZuZhi2, Unigene21629\_JingChaoZuZhi2, Unigene21992\_JingChaoZuZhi2, Unigene22413\_JingChaoZuZhi2, Unigene22487\_JingChaoZuZhi2, Unigene22504\_JingChaoZuZhi2, Unigene22580\_JingChaoZuZhi2, Unigene22669\_JingChaoZuZhi2, Unigene22673\_JingChaoZuZhi2, Unigene22676\_JingChaoZuZhi2, Unigene22708\_JingChaoZuZhi2, Unigene2473\_JingChaoZuZhi2, Unigene3239\_JingChaoZuZhi2, Unigene35730\_JingChaoZuZhi2, Unigene3640\_JingChaoZuZhi2, Unigene36929\_JingChaoZuZhi2, Unigene3950\_JingChaoZuZhi2, Unigene39584\_JingChaoZuZhi2, Unigene395\_JingChaoZuZhi2, Unigene39825\_JingChaoZuZhi2, Unigene4550\_JingChaoZuZhi2, Unigene46171\_JingChaoZuZhi2, Unigene46240\_JingChaoZuZhi2, Unigene50014\_JingChaoZuZhi2, Unigene50479\_JingChaoZuZhi2, Unigene50615\_JingChaoZuZhi2, Unigene554\_JingChaoZuZhi2, Unigene58134\_JingChaoZuZhi2, Unigene58386\_JingChaoZuZhi2, Unigene58428\_JingChaoZuZhi2, Unigene58474\_JingChaoZuZhi2, Unigene59068\_JingChaoZuZhi2, Unigene62970\_JingChaoZuZhi2, Unigene63323\_JingChaoZuZhi2, Unigene63355\_JingChaoZuZhi2, Unigene63406\_JingChaoZuZhi2, Unigene64650\_JingChaoZuZhi2, Unigene6467\_JingChaoZuZhi2, Unigene64820\_JingChaoZuZhi2, Unigene67177\_JingChaoZuZhi2, Unigene6785\_JingChaoZuZhi2, Unigene7068\_JingChaoZuZhi2, Unigene71161\_JingChaoZuZhi2, Unigene71550\_JingChaoZuZhi2, Unigene72769\_JingChaoZuZhi2, Unigene74227\_JingChaoZuZhi2, Unigene74609\_JingChaoZuZhi2, Unigene75652\_JingChaoZuZhi2, Unigene75739\_JingChaoZuZhi2, Unigene76639\_JingChaoZuZhi2, Unigene78522\_JingChaoZuZhi2, Unigene788\_JingChaoZuZhi2, Unigene79482\_JingChaoZuZhi2, Unigene80123\_JingChaoZuZhi2, Unigene8151\_JingChaoZuZhi2, Unigene81716\_JingChaoZuZhi2, Unigene8191\_JingChaoZuZhi2, Unigene83188\_JingChaoZuZhi2, Unigene83195\_JingChaoZuZhi2, Unigene84178\_JingChaoZuZhi2, Unigene851\_JingChaoZuZhi2, Unigene8600\_JingChaoZuZhi2, Unigene86119\_JingChaoZuZhi2, Unigene86504\_JingChaoZuZhi2, Unigene86789\_JingChaoZuZhi2, Unigene87474\_JingChaoZuZhi2, Unigene8773\_JingChaoZuZhi2, Unigene88685\_JingChaoZuZhi2, Unigene89093\_JingChaoZuZhi2, Unigene89161\_JingChaoZuZhi2, Unigene91119\_JingChaoZuZhi2, Unigene91538\_JingChaoZuZhi2, Unigene92019\_JingChaoZuZhi2, Unigene92116\_JingChaoZuZhi2, Unigene92715\_JingChaoZuZhi2, Unigene93170\_JingChaoZuZhi2, Unigene93195\_JingChaoZuZhi2, Unigene93288\_JingChaoZuZhi2, Unigene932\_JingChaoZuZhi2, Unigene93534\_JingChaoZuZhi2, Unigene93754\_JingChaoZuZhi2, Unigene93821\_JingChaoZuZhi2, Unigene94242\_JingChaoZuZhi2, Unigene9428\_JingChaoZuZhi2, Unigene94388\_JingChaoZuZhi2, Unigene94604\_JingChaoZuZhi2, Unigene94711\_JingChaoZuZhi2, Unigene95315\_JingChaoZuZhi2, Unigene95566\_JingChaoZuZhi2, Unigene95568\_JingChaoZuZhi2, Unigene95881\_JingChaoZuZhi2, Unigene95883\_JingChaoZuZhi2, Unigene96084\_JingChaoZuZhi2, Unigene96124\_JingChaoZuZhi2 |
| 80 | Valine, leucine and isoleucine degradation | Unigene11312\_JingChaoZuZhi2, Unigene11466\_JingChaoZuZhi2, Unigene12758\_JingChaoZuZhi2, Unigene15929\_JingChaoZuZhi2, Unigene16352\_JingChaoZuZhi2, Unigene16663\_JingChaoZuZhi2, Unigene16788\_JingChaoZuZhi2, Unigene16863\_JingChaoZuZhi2, Unigene16899\_JingChaoZuZhi2, Unigene17162\_JingChaoZuZhi2, Unigene17217\_JingChaoZuZhi2, Unigene17401\_JingChaoZuZhi2, Unigene17889\_JingChaoZuZhi2, Unigene17995\_JingChaoZuZhi2, Unigene18179\_JingChaoZuZhi2, Unigene18358\_JingChaoZuZhi2, Unigene18409\_JingChaoZuZhi2, Unigene18553\_JingChaoZuZhi2, Unigene18846\_JingChaoZuZhi2, Unigene19907\_JingChaoZuZhi2, Unigene20010\_JingChaoZuZhi2, Unigene21049\_JingChaoZuZhi2, Unigene21176\_JingChaoZuZhi2, Unigene21820\_JingChaoZuZhi2, Unigene21821\_JingChaoZuZhi2, Unigene22123\_JingChaoZuZhi2, Unigene22143\_JingChaoZuZhi2, Unigene22352\_JingChaoZuZhi2, Unigene2235\_JingChaoZuZhi2, Unigene22684\_JingChaoZuZhi2, Unigene22747\_JingChaoZuZhi2, Unigene22793\_JingChaoZuZhi2, Unigene240\_JingChaoZuZhi2, Unigene25361\_JingChaoZuZhi2, Unigene27219\_JingChaoZuZhi2, Unigene28844\_JingChaoZuZhi2, Unigene3162\_JingChaoZuZhi2, Unigene32951\_JingChaoZuZhi2, Unigene35187\_JingChaoZuZhi2, Unigene36089\_JingChaoZuZhi2, Unigene38622\_JingChaoZuZhi2, Unigene41503\_JingChaoZuZhi2, Unigene43048\_JingChaoZuZhi2, Unigene4423\_JingChaoZuZhi2, Unigene44292\_JingChaoZuZhi2, Unigene45176\_JingChaoZuZhi2, Unigene49819\_JingChaoZuZhi2, Unigene50371\_JingChaoZuZhi2, Unigene5068\_JingChaoZuZhi2, Unigene51532\_JingChaoZuZhi2, Unigene5449\_JingChaoZuZhi2, Unigene5663\_JingChaoZuZhi2, Unigene57841\_JingChaoZuZhi2, Unigene58621\_JingChaoZuZhi2, Unigene58763\_JingChaoZuZhi2, Unigene59548\_JingChaoZuZhi2, Unigene61162\_JingChaoZuZhi2, Unigene61664\_JingChaoZuZhi2, Unigene62337\_JingChaoZuZhi2, Unigene62804\_JingChaoZuZhi2, Unigene62962\_JingChaoZuZhi2, Unigene63649\_JingChaoZuZhi2, Unigene63715\_JingChaoZuZhi2, Unigene64627\_JingChaoZuZhi2, Unigene64673\_JingChaoZuZhi2, Unigene64862\_JingChaoZuZhi2, Unigene64903\_JingChaoZuZhi2, Unigene65441\_JingChaoZuZhi2, Unigene66337\_JingChaoZuZhi2, Unigene67613\_JingChaoZuZhi2, Unigene68001\_JingChaoZuZhi2, Unigene68716\_JingChaoZuZhi2, Unigene70703\_JingChaoZuZhi2, Unigene71639\_JingChaoZuZhi2, Unigene72476\_JingChaoZuZhi2, Unigene73150\_JingChaoZuZhi2, Unigene76068\_JingChaoZuZhi2, Unigene76458\_JingChaoZuZhi2, Unigene78625\_JingChaoZuZhi2, Unigene78854\_JingChaoZuZhi2, Unigene79135\_JingChaoZuZhi2, Unigene80375\_JingChaoZuZhi2, Unigene80397\_JingChaoZuZhi2, Unigene80446\_JingChaoZuZhi2, Unigene80881\_JingChaoZuZhi2, Unigene81722\_JingChaoZuZhi2, Unigene82129\_JingChaoZuZhi2, Unigene8213\_JingChaoZuZhi2, Unigene82435\_JingChaoZuZhi2, Unigene83981\_JingChaoZuZhi2, Unigene84063\_JingChaoZuZhi2, Unigene84124\_JingChaoZuZhi2, Unigene84149\_JingChaoZuZhi2, Unigene84343\_JingChaoZuZhi2, Unigene8442\_JingChaoZuZhi2, Unigene8454\_JingChaoZuZhi2, Unigene84851\_JingChaoZuZhi2, Unigene85224\_JingChaoZuZhi2, Unigene85989\_JingChaoZuZhi2, Unigene86463\_JingChaoZuZhi2, Unigene87044\_JingChaoZuZhi2, Unigene87485\_JingChaoZuZhi2, Unigene87847\_JingChaoZuZhi2, Unigene87856\_JingChaoZuZhi2, Unigene88377\_JingChaoZuZhi2, Unigene89289\_JingChaoZuZhi2, Unigene90127\_JingChaoZuZhi2, Unigene90838\_JingChaoZuZhi2, Unigene91180\_JingChaoZuZhi2, Unigene91700\_JingChaoZuZhi2, Unigene92256\_JingChaoZuZhi2, Unigene92534\_JingChaoZuZhi2, Unigene93143\_JingChaoZuZhi2, Unigene93334\_JingChaoZuZhi2, Unigene93681\_JingChaoZuZhi2, Unigene94651\_JingChaoZuZhi2, Unigene94837\_JingChaoZuZhi2, Unigene95523\_JingChaoZuZhi2, Unigene9580\_JingChaoZuZhi2, Unigene95953\_JingChaoZuZhi2, Unigene95959\_JingChaoZuZhi2, Unigene95985\_JingChaoZuZhi2, Unigene95986\_JingChaoZuZhi2, Unigene96031\_JingChaoZuZhi2, Unigene96087\_JingChaoZuZhi2 |
| 81 | Riboflavin metabolism | Unigene10351\_JingChaoZuZhi2, Unigene11581\_JingChaoZuZhi2, Unigene13455\_JingChaoZuZhi2, Unigene14071\_JingChaoZuZhi2, Unigene14926\_JingChaoZuZhi2, Unigene15429\_JingChaoZuZhi2, Unigene15557\_JingChaoZuZhi2, Unigene15770\_JingChaoZuZhi2, Unigene16137\_JingChaoZuZhi2, Unigene16152\_JingChaoZuZhi2, Unigene16510\_JingChaoZuZhi2, Unigene17896\_JingChaoZuZhi2, Unigene18043\_JingChaoZuZhi2, Unigene18608\_JingChaoZuZhi2, Unigene19751\_JingChaoZuZhi2, Unigene20698\_JingChaoZuZhi2, Unigene20862\_JingChaoZuZhi2, Unigene21306\_JingChaoZuZhi2, Unigene21678\_JingChaoZuZhi2, Unigene21963\_JingChaoZuZhi2, Unigene219\_JingChaoZuZhi2, Unigene22047\_JingChaoZuZhi2, Unigene22145\_JingChaoZuZhi2, Unigene22392\_JingChaoZuZhi2, Unigene22517\_JingChaoZuZhi2, Unigene22566\_JingChaoZuZhi2, Unigene22822\_JingChaoZuZhi2, Unigene2469\_JingChaoZuZhi2, Unigene2480\_JingChaoZuZhi2, Unigene25240\_JingChaoZuZhi2, Unigene2563\_JingChaoZuZhi2, Unigene29367\_JingChaoZuZhi2, Unigene29519\_JingChaoZuZhi2, Unigene30775\_JingChaoZuZhi2, Unigene3374\_JingChaoZuZhi2, Unigene35744\_JingChaoZuZhi2, Unigene36819\_JingChaoZuZhi2, Unigene37665\_JingChaoZuZhi2, Unigene38324\_JingChaoZuZhi2, Unigene38755\_JingChaoZuZhi2, Unigene3910\_JingChaoZuZhi2, Unigene39914\_JingChaoZuZhi2, Unigene40020\_JingChaoZuZhi2, Unigene41513\_JingChaoZuZhi2, Unigene42461\_JingChaoZuZhi2, Unigene42836\_JingChaoZuZhi2, Unigene4426\_JingChaoZuZhi2, Unigene46240\_JingChaoZuZhi2, Unigene49277\_JingChaoZuZhi2, Unigene51191\_JingChaoZuZhi2, Unigene51272\_JingChaoZuZhi2, Unigene52763\_JingChaoZuZhi2, Unigene54153\_JingChaoZuZhi2, Unigene57247\_JingChaoZuZhi2, Unigene59122\_JingChaoZuZhi2, Unigene59240\_JingChaoZuZhi2, Unigene6135\_JingChaoZuZhi2, Unigene61702\_JingChaoZuZhi2, Unigene6175\_JingChaoZuZhi2, Unigene62745\_JingChaoZuZhi2, Unigene63000\_JingChaoZuZhi2, Unigene6460\_JingChaoZuZhi2, Unigene6567\_JingChaoZuZhi2, Unigene6648\_JingChaoZuZhi2, Unigene68716\_JingChaoZuZhi2, Unigene69255\_JingChaoZuZhi2, Unigene69602\_JingChaoZuZhi2, Unigene70225\_JingChaoZuZhi2, Unigene71132\_JingChaoZuZhi2, Unigene73037\_JingChaoZuZhi2, Unigene73241\_JingChaoZuZhi2, Unigene73765\_JingChaoZuZhi2, Unigene74027\_JingChaoZuZhi2, Unigene7407\_JingChaoZuZhi2, Unigene74193\_JingChaoZuZhi2, Unigene74521\_JingChaoZuZhi2, Unigene74587\_JingChaoZuZhi2, Unigene74897\_JingChaoZuZhi2, Unigene75125\_JingChaoZuZhi2, Unigene75507\_JingChaoZuZhi2, Unigene75919\_JingChaoZuZhi2, Unigene76214\_JingChaoZuZhi2, Unigene77467\_JingChaoZuZhi2, Unigene77528\_JingChaoZuZhi2, Unigene78522\_JingChaoZuZhi2, Unigene78818\_JingChaoZuZhi2, Unigene7881\_JingChaoZuZhi2, Unigene79257\_JingChaoZuZhi2, Unigene7970\_JingChaoZuZhi2, Unigene80649\_JingChaoZuZhi2, Unigene80684\_JingChaoZuZhi2, Unigene80936\_JingChaoZuZhi2, Unigene81858\_JingChaoZuZhi2, Unigene82301\_JingChaoZuZhi2, Unigene8328\_JingChaoZuZhi2, Unigene83988\_JingChaoZuZhi2, Unigene84345\_JingChaoZuZhi2, Unigene86222\_JingChaoZuZhi2, Unigene86295\_JingChaoZuZhi2, Unigene86566\_JingChaoZuZhi2, Unigene88620\_JingChaoZuZhi2, Unigene88776\_JingChaoZuZhi2, Unigene89178\_JingChaoZuZhi2, Unigene89714\_JingChaoZuZhi2, Unigene89751\_JingChaoZuZhi2, Unigene89752\_JingChaoZuZhi2, Unigene89942\_JingChaoZuZhi2, Unigene89986\_JingChaoZuZhi2, Unigene90557\_JingChaoZuZhi2, Unigene91254\_JingChaoZuZhi2, Unigene91458\_JingChaoZuZhi2, Unigene91837\_JingChaoZuZhi2, Unigene92016\_JingChaoZuZhi2, Unigene92503\_JingChaoZuZhi2, Unigene93125\_JingChaoZuZhi2, Unigene93326\_JingChaoZuZhi2, Unigene93590\_JingChaoZuZhi2, Unigene94747\_JingChaoZuZhi2, Unigene95210\_JingChaoZuZhi2, Unigene9530\_JingChaoZuZhi2, Unigene95677\_JingChaoZuZhi2, Unigene95988\_JingChaoZuZhi2, Unigene96024\_JingChaoZuZhi2 |
| 82 | Prion diseases | Unigene10275\_JingChaoZuZhi2, Unigene10380\_JingChaoZuZhi2, Unigene10800\_JingChaoZuZhi2, Unigene11251\_JingChaoZuZhi2, Unigene11333\_JingChaoZuZhi2, Unigene11371\_JingChaoZuZhi2, Unigene11620\_JingChaoZuZhi2, Unigene12647\_JingChaoZuZhi2, Unigene12729\_JingChaoZuZhi2, Unigene12852\_JingChaoZuZhi2, Unigene13115\_JingChaoZuZhi2, Unigene13389\_JingChaoZuZhi2, Unigene13680\_JingChaoZuZhi2, Unigene14250\_JingChaoZuZhi2, Unigene14409\_JingChaoZuZhi2, Unigene14417\_JingChaoZuZhi2, Unigene15648\_JingChaoZuZhi2, Unigene15681\_JingChaoZuZhi2, Unigene15765\_JingChaoZuZhi2, Unigene17756\_JingChaoZuZhi2, Unigene18307\_JingChaoZuZhi2, Unigene18490\_JingChaoZuZhi2, Unigene18577\_JingChaoZuZhi2, Unigene18768\_JingChaoZuZhi2, Unigene18780\_JingChaoZuZhi2, Unigene19131\_JingChaoZuZhi2, Unigene19887\_JingChaoZuZhi2, Unigene20218\_JingChaoZuZhi2, Unigene21103\_JingChaoZuZhi2, Unigene21552\_JingChaoZuZhi2, Unigene21605\_JingChaoZuZhi2, Unigene21637\_JingChaoZuZhi2, Unigene21815\_JingChaoZuZhi2, Unigene21868\_JingChaoZuZhi2, Unigene2186\_JingChaoZuZhi2, Unigene21989\_JingChaoZuZhi2, Unigene22284\_JingChaoZuZhi2, Unigene252\_JingChaoZuZhi2, Unigene2899\_JingChaoZuZhi2, Unigene33784\_JingChaoZuZhi2, Unigene34853\_JingChaoZuZhi2, Unigene35624\_JingChaoZuZhi2, Unigene35990\_JingChaoZuZhi2, Unigene37667\_JingChaoZuZhi2, Unigene43145\_JingChaoZuZhi2, Unigene44141\_JingChaoZuZhi2, Unigene44435\_JingChaoZuZhi2, Unigene51820\_JingChaoZuZhi2, Unigene53020\_JingChaoZuZhi2, Unigene53503\_JingChaoZuZhi2, Unigene55100\_JingChaoZuZhi2, Unigene55864\_JingChaoZuZhi2, Unigene56752\_JingChaoZuZhi2, Unigene57566\_JingChaoZuZhi2, Unigene58670\_JingChaoZuZhi2, Unigene6028\_JingChaoZuZhi2, Unigene6222\_JingChaoZuZhi2, Unigene63875\_JingChaoZuZhi2, Unigene6391\_JingChaoZuZhi2, Unigene6424\_JingChaoZuZhi2, Unigene64989\_JingChaoZuZhi2, Unigene67177\_JingChaoZuZhi2, Unigene67364\_JingChaoZuZhi2, Unigene67523\_JingChaoZuZhi2, Unigene69678\_JingChaoZuZhi2, Unigene70549\_JingChaoZuZhi2, Unigene7068\_JingChaoZuZhi2, Unigene71144\_JingChaoZuZhi2, Unigene74963\_JingChaoZuZhi2, Unigene7567\_JingChaoZuZhi2, Unigene7586\_JingChaoZuZhi2, Unigene77067\_JingChaoZuZhi2, Unigene77738\_JingChaoZuZhi2, Unigene78136\_JingChaoZuZhi2, Unigene78235\_JingChaoZuZhi2, Unigene7939\_JingChaoZuZhi2, Unigene80385\_JingChaoZuZhi2, Unigene80507\_JingChaoZuZhi2, Unigene80772\_JingChaoZuZhi2, Unigene81553\_JingChaoZuZhi2, Unigene81716\_JingChaoZuZhi2, Unigene8191\_JingChaoZuZhi2, Unigene82058\_JingChaoZuZhi2, Unigene82367\_JingChaoZuZhi2, Unigene83524\_JingChaoZuZhi2, Unigene84077\_JingChaoZuZhi2, Unigene84608\_JingChaoZuZhi2, Unigene86221\_JingChaoZuZhi2, Unigene86296\_JingChaoZuZhi2, Unigene87640\_JingChaoZuZhi2, Unigene87670\_JingChaoZuZhi2, Unigene87912\_JingChaoZuZhi2, Unigene88767\_JingChaoZuZhi2, Unigene89759\_JingChaoZuZhi2, Unigene91028\_JingChaoZuZhi2, Unigene91427\_JingChaoZuZhi2, Unigene91511\_JingChaoZuZhi2, Unigene91527\_JingChaoZuZhi2, Unigene91628\_JingChaoZuZhi2, Unigene91944\_JingChaoZuZhi2, Unigene92235\_JingChaoZuZhi2, Unigene92294\_JingChaoZuZhi2, Unigene92831\_JingChaoZuZhi2, Unigene93056\_JingChaoZuZhi2, Unigene93563\_JingChaoZuZhi2, Unigene93679\_JingChaoZuZhi2, Unigene93729\_JingChaoZuZhi2, Unigene93779\_JingChaoZuZhi2, Unigene93793\_JingChaoZuZhi2, Unigene93921\_JingChaoZuZhi2, Unigene94362\_JingChaoZuZhi2, Unigene94533\_JingChaoZuZhi2, Unigene94721\_JingChaoZuZhi2, Unigene94825\_JingChaoZuZhi2, Unigene94897\_JingChaoZuZhi2, Unigene9511\_JingChaoZuZhi2, Unigene95185\_JingChaoZuZhi2, Unigene95427\_JingChaoZuZhi2, Unigene95897\_JingChaoZuZhi2, Unigene95984\_JingChaoZuZhi2, Unigene96016\_JingChaoZuZhi2, Unigene96180\_JingChaoZuZhi2, Unigene96221\_JingChaoZuZhi2 |
| 83 | Amino sugar and nucleotide sugar metabolism | Unigene10315\_JingChaoZuZhi2, Unigene1045\_JingChaoZuZhi2, Unigene12186\_JingChaoZuZhi2, Unigene12503\_JingChaoZuZhi2, Unigene13103\_JingChaoZuZhi2, Unigene13176\_JingChaoZuZhi2, Unigene13508\_JingChaoZuZhi2, Unigene13837\_JingChaoZuZhi2, Unigene14999\_JingChaoZuZhi2, Unigene15503\_JingChaoZuZhi2, Unigene15830\_JingChaoZuZhi2, Unigene15939\_JingChaoZuZhi2, Unigene16281\_JingChaoZuZhi2, Unigene16456\_JingChaoZuZhi2, Unigene16762\_JingChaoZuZhi2, Unigene17591\_JingChaoZuZhi2, Unigene19303\_JingChaoZuZhi2, Unigene20658\_JingChaoZuZhi2, Unigene21122\_JingChaoZuZhi2, Unigene21949\_JingChaoZuZhi2, Unigene22103\_JingChaoZuZhi2, Unigene22832\_JingChaoZuZhi2, Unigene24568\_JingChaoZuZhi2, Unigene25992\_JingChaoZuZhi2, Unigene26937\_JingChaoZuZhi2, Unigene30179\_JingChaoZuZhi2, Unigene35321\_JingChaoZuZhi2, Unigene36996\_JingChaoZuZhi2, Unigene41663\_JingChaoZuZhi2, Unigene4377\_JingChaoZuZhi2, Unigene44891\_JingChaoZuZhi2, Unigene45887\_JingChaoZuZhi2, Unigene46843\_JingChaoZuZhi2, Unigene48948\_JingChaoZuZhi2, Unigene49865\_JingChaoZuZhi2, Unigene50259\_JingChaoZuZhi2, Unigene5055\_JingChaoZuZhi2, Unigene50599\_JingChaoZuZhi2, Unigene50833\_JingChaoZuZhi2, Unigene51610\_JingChaoZuZhi2, Unigene51906\_JingChaoZuZhi2, Unigene53809\_JingChaoZuZhi2, Unigene55751\_JingChaoZuZhi2, Unigene57152\_JingChaoZuZhi2, Unigene58141\_JingChaoZuZhi2, Unigene59180\_JingChaoZuZhi2, Unigene59764\_JingChaoZuZhi2, Unigene60210\_JingChaoZuZhi2, Unigene61836\_JingChaoZuZhi2, Unigene65555\_JingChaoZuZhi2, Unigene65632\_JingChaoZuZhi2, Unigene67218\_JingChaoZuZhi2, Unigene67946\_JingChaoZuZhi2, Unigene68475\_JingChaoZuZhi2, Unigene69968\_JingChaoZuZhi2, Unigene70140\_JingChaoZuZhi2, Unigene7059\_JingChaoZuZhi2, Unigene71248\_JingChaoZuZhi2, Unigene73451\_JingChaoZuZhi2, Unigene74590\_JingChaoZuZhi2, Unigene74725\_JingChaoZuZhi2, Unigene75880\_JingChaoZuZhi2, Unigene76385\_JingChaoZuZhi2, Unigene76824\_JingChaoZuZhi2, Unigene76840\_JingChaoZuZhi2, Unigene77374\_JingChaoZuZhi2, Unigene78250\_JingChaoZuZhi2, Unigene78413\_JingChaoZuZhi2, Unigene78578\_JingChaoZuZhi2, Unigene78901\_JingChaoZuZhi2, Unigene79539\_JingChaoZuZhi2, Unigene79827\_JingChaoZuZhi2, Unigene80848\_JingChaoZuZhi2, Unigene81427\_JingChaoZuZhi2, Unigene81505\_JingChaoZuZhi2, Unigene81511\_JingChaoZuZhi2, Unigene81642\_JingChaoZuZhi2, Unigene81654\_JingChaoZuZhi2, Unigene82270\_JingChaoZuZhi2, Unigene82744\_JingChaoZuZhi2, Unigene82868\_JingChaoZuZhi2, Unigene83023\_JingChaoZuZhi2, Unigene83440\_JingChaoZuZhi2, Unigene84559\_JingChaoZuZhi2, Unigene85353\_JingChaoZuZhi2, Unigene86120\_JingChaoZuZhi2, Unigene88238\_JingChaoZuZhi2, Unigene88528\_JingChaoZuZhi2, Unigene89279\_JingChaoZuZhi2, Unigene89292\_JingChaoZuZhi2, Unigene89607\_JingChaoZuZhi2, Unigene90638\_JingChaoZuZhi2, Unigene90834\_JingChaoZuZhi2, Unigene9127\_JingChaoZuZhi2, Unigene91360\_JingChaoZuZhi2, Unigene91845\_JingChaoZuZhi2, Unigene91969\_JingChaoZuZhi2, Unigene92035\_JingChaoZuZhi2, Unigene92238\_JingChaoZuZhi2, Unigene9225\_JingChaoZuZhi2, Unigene92575\_JingChaoZuZhi2, Unigene93427\_JingChaoZuZhi2, Unigene93877\_JingChaoZuZhi2, Unigene93925\_JingChaoZuZhi2, Unigene94262\_JingChaoZuZhi2, Unigene94393\_JingChaoZuZhi2, Unigene94595\_JingChaoZuZhi2, Unigene95051\_JingChaoZuZhi2, Unigene95057\_JingChaoZuZhi2, Unigene95070\_JingChaoZuZhi2, Unigene95296\_JingChaoZuZhi2, Unigene95464\_JingChaoZuZhi2, Unigene95495\_JingChaoZuZhi2, Unigene95593\_JingChaoZuZhi2, Unigene95867\_JingChaoZuZhi2, Unigene95868\_JingChaoZuZhi2, Unigene95888\_JingChaoZuZhi2, Unigene95998\_JingChaoZuZhi2, Unigene96039\_JingChaoZuZhi2 |
| 84 | Long-term depression | Unigene10318\_JingChaoZuZhi2, Unigene11000\_JingChaoZuZhi2, Unigene11055\_JingChaoZuZhi2, Unigene11605\_JingChaoZuZhi2, Unigene11621\_JingChaoZuZhi2, Unigene12073\_JingChaoZuZhi2, Unigene12098\_JingChaoZuZhi2, Unigene12161\_JingChaoZuZhi2, Unigene12260\_JingChaoZuZhi2, Unigene12577\_JingChaoZuZhi2, Unigene13420\_JingChaoZuZhi2, Unigene13650\_JingChaoZuZhi2, Unigene13770\_JingChaoZuZhi2, Unigene13869\_JingChaoZuZhi2, Unigene13996\_JingChaoZuZhi2, Unigene14357\_JingChaoZuZhi2, Unigene14360\_JingChaoZuZhi2, Unigene15406\_JingChaoZuZhi2, Unigene15425\_JingChaoZuZhi2, Unigene15651\_JingChaoZuZhi2, Unigene15942\_JingChaoZuZhi2, Unigene16366\_JingChaoZuZhi2, Unigene16991\_JingChaoZuZhi2, Unigene19904\_JingChaoZuZhi2, Unigene21407\_JingChaoZuZhi2, Unigene21513\_JingChaoZuZhi2, Unigene21578\_JingChaoZuZhi2, Unigene22426\_JingChaoZuZhi2, Unigene22779\_JingChaoZuZhi2, Unigene24425\_JingChaoZuZhi2, Unigene26730\_JingChaoZuZhi2, Unigene2960\_JingChaoZuZhi2, Unigene32827\_JingChaoZuZhi2, Unigene32999\_JingChaoZuZhi2, Unigene34929\_JingChaoZuZhi2, Unigene35952\_JingChaoZuZhi2, Unigene39832\_JingChaoZuZhi2, Unigene41333\_JingChaoZuZhi2, Unigene42329\_JingChaoZuZhi2, Unigene43059\_JingChaoZuZhi2, Unigene43723\_JingChaoZuZhi2, Unigene4449\_JingChaoZuZhi2, Unigene45532\_JingChaoZuZhi2, Unigene47872\_JingChaoZuZhi2, Unigene48962\_JingChaoZuZhi2, Unigene54367\_JingChaoZuZhi2, Unigene54449\_JingChaoZuZhi2, Unigene56567\_JingChaoZuZhi2, Unigene56709\_JingChaoZuZhi2, Unigene57184\_JingChaoZuZhi2, Unigene59750\_JingChaoZuZhi2, Unigene6028\_JingChaoZuZhi2, Unigene60800\_JingChaoZuZhi2, Unigene63994\_JingChaoZuZhi2, Unigene64164\_JingChaoZuZhi2, Unigene64583\_JingChaoZuZhi2, Unigene64830\_JingChaoZuZhi2, Unigene65294\_JingChaoZuZhi2, Unigene65445\_JingChaoZuZhi2, Unigene66540\_JingChaoZuZhi2, Unigene67328\_JingChaoZuZhi2, Unigene68097\_JingChaoZuZhi2, Unigene68151\_JingChaoZuZhi2, Unigene68300\_JingChaoZuZhi2, Unigene68516\_JingChaoZuZhi2, Unigene69910\_JingChaoZuZhi2, Unigene70087\_JingChaoZuZhi2, Unigene70493\_JingChaoZuZhi2, Unigene70549\_JingChaoZuZhi2, Unigene70723\_JingChaoZuZhi2, Unigene72098\_JingChaoZuZhi2, Unigene72445\_JingChaoZuZhi2, Unigene72643\_JingChaoZuZhi2, Unigene7373\_JingChaoZuZhi2, Unigene74569\_JingChaoZuZhi2, Unigene74594\_JingChaoZuZhi2, Unigene76182\_JingChaoZuZhi2, Unigene77149\_JingChaoZuZhi2, Unigene7747\_JingChaoZuZhi2, Unigene78007\_JingChaoZuZhi2, Unigene78078\_JingChaoZuZhi2, Unigene78255\_JingChaoZuZhi2, Unigene78784\_JingChaoZuZhi2, Unigene8191\_JingChaoZuZhi2, Unigene82329\_JingChaoZuZhi2, Unigene82368\_JingChaoZuZhi2, Unigene83017\_JingChaoZuZhi2, Unigene83166\_JingChaoZuZhi2, Unigene83196\_JingChaoZuZhi2, Unigene84247\_JingChaoZuZhi2, Unigene85059\_JingChaoZuZhi2, Unigene86091\_JingChaoZuZhi2, Unigene86359\_JingChaoZuZhi2, Unigene87082\_JingChaoZuZhi2, Unigene87245\_JingChaoZuZhi2, Unigene87332\_JingChaoZuZhi2, Unigene87607\_JingChaoZuZhi2, Unigene876\_JingChaoZuZhi2, Unigene88068\_JingChaoZuZhi2, Unigene88385\_JingChaoZuZhi2, Unigene88708\_JingChaoZuZhi2, Unigene88773\_JingChaoZuZhi2, Unigene89147\_JingChaoZuZhi2, Unigene89323\_JingChaoZuZhi2, Unigene91221\_JingChaoZuZhi2, Unigene91739\_JingChaoZuZhi2, Unigene92737\_JingChaoZuZhi2, Unigene92918\_JingChaoZuZhi2, Unigene93012\_JingChaoZuZhi2, Unigene93754\_JingChaoZuZhi2, Unigene94388\_JingChaoZuZhi2, Unigene95677\_JingChaoZuZhi2, Unigene95916\_JingChaoZuZhi2, Unigene96262\_JingChaoZuZhi2, Unigene9654\_JingChaoZuZhi2, Unigene9812\_JingChaoZuZhi2, Unigene9902\_JingChaoZuZhi2, Unigene9\_JingChaoZuZhi2 |
| 85 | Phototransduction - fly | Unigene10047\_JingChaoZuZhi2, Unigene10259\_JingChaoZuZhi2, Unigene10318\_JingChaoZuZhi2, Unigene1060\_JingChaoZuZhi2, Unigene10666\_JingChaoZuZhi2, Unigene10\_JingChaoZuZhi2, Unigene1162\_JingChaoZuZhi2, Unigene11772\_JingChaoZuZhi2, Unigene12073\_JingChaoZuZhi2, Unigene14360\_JingChaoZuZhi2, Unigene16004\_JingChaoZuZhi2, Unigene16566\_JingChaoZuZhi2, Unigene16991\_JingChaoZuZhi2, Unigene17282\_JingChaoZuZhi2, Unigene18354\_JingChaoZuZhi2, Unigene18466\_JingChaoZuZhi2, Unigene21578\_JingChaoZuZhi2, Unigene21818\_JingChaoZuZhi2, Unigene22336\_JingChaoZuZhi2, Unigene22779\_JingChaoZuZhi2, Unigene2370\_JingChaoZuZhi2, Unigene2446\_JingChaoZuZhi2, Unigene25495\_JingChaoZuZhi2, Unigene26730\_JingChaoZuZhi2, Unigene26987\_JingChaoZuZhi2, Unigene2960\_JingChaoZuZhi2, Unigene3150\_JingChaoZuZhi2, Unigene31918\_JingChaoZuZhi2, Unigene32827\_JingChaoZuZhi2, Unigene3313\_JingChaoZuZhi2, Unigene33440\_JingChaoZuZhi2, Unigene33866\_JingChaoZuZhi2, Unigene3400\_JingChaoZuZhi2, Unigene34929\_JingChaoZuZhi2, Unigene35016\_JingChaoZuZhi2, Unigene3807\_JingChaoZuZhi2, Unigene39102\_JingChaoZuZhi2, Unigene39726\_JingChaoZuZhi2, Unigene40581\_JingChaoZuZhi2, Unigene40919\_JingChaoZuZhi2, Unigene41333\_JingChaoZuZhi2, Unigene42248\_JingChaoZuZhi2, Unigene43059\_JingChaoZuZhi2, Unigene43910\_JingChaoZuZhi2, Unigene44190\_JingChaoZuZhi2, Unigene46044\_JingChaoZuZhi2, Unigene51467\_JingChaoZuZhi2, Unigene53933\_JingChaoZuZhi2, Unigene54367\_JingChaoZuZhi2, Unigene54449\_JingChaoZuZhi2, Unigene57450\_JingChaoZuZhi2, Unigene57848\_JingChaoZuZhi2, Unigene59146\_JingChaoZuZhi2, Unigene61163\_JingChaoZuZhi2, Unigene61244\_JingChaoZuZhi2, Unigene63588\_JingChaoZuZhi2, Unigene63982\_JingChaoZuZhi2, Unigene63994\_JingChaoZuZhi2, Unigene64164\_JingChaoZuZhi2, Unigene64830\_JingChaoZuZhi2, Unigene65118\_JingChaoZuZhi2, Unigene65445\_JingChaoZuZhi2, Unigene6634\_JingChaoZuZhi2, Unigene66540\_JingChaoZuZhi2, Unigene69100\_JingChaoZuZhi2, Unigene6933\_JingChaoZuZhi2, Unigene6956\_JingChaoZuZhi2, Unigene7010\_JingChaoZuZhi2, Unigene70493\_JingChaoZuZhi2, Unigene7055\_JingChaoZuZhi2, Unigene70723\_JingChaoZuZhi2, Unigene71323\_JingChaoZuZhi2, Unigene71420\_JingChaoZuZhi2, Unigene71592\_JingChaoZuZhi2, Unigene72643\_JingChaoZuZhi2, Unigene75396\_JingChaoZuZhi2, Unigene77149\_JingChaoZuZhi2, Unigene78078\_JingChaoZuZhi2, Unigene82117\_JingChaoZuZhi2, Unigene82489\_JingChaoZuZhi2, Unigene83667\_JingChaoZuZhi2, Unigene83872\_JingChaoZuZhi2, Unigene84115\_JingChaoZuZhi2, Unigene84247\_JingChaoZuZhi2, Unigene8507\_JingChaoZuZhi2, Unigene85193\_JingChaoZuZhi2, Unigene85464\_JingChaoZuZhi2, Unigene862\_JingChaoZuZhi2, Unigene87332\_JingChaoZuZhi2, Unigene87607\_JingChaoZuZhi2, Unigene88342\_JingChaoZuZhi2, Unigene88385\_JingChaoZuZhi2, Unigene88594\_JingChaoZuZhi2, Unigene88724\_JingChaoZuZhi2, Unigene88773\_JingChaoZuZhi2, Unigene89060\_JingChaoZuZhi2, Unigene89147\_JingChaoZuZhi2, Unigene89843\_JingChaoZuZhi2, Unigene90524\_JingChaoZuZhi2, Unigene9086\_JingChaoZuZhi2, Unigene91163\_JingChaoZuZhi2, Unigene91289\_JingChaoZuZhi2, Unigene91739\_JingChaoZuZhi2, Unigene91761\_JingChaoZuZhi2, Unigene91924\_JingChaoZuZhi2, Unigene93012\_JingChaoZuZhi2, Unigene93545\_JingChaoZuZhi2, Unigene93588\_JingChaoZuZhi2, Unigene93714\_JingChaoZuZhi2, Unigene94110\_JingChaoZuZhi2, Unigene951\_JingChaoZuZhi2, Unigene9522\_JingChaoZuZhi2, Unigene95677\_JingChaoZuZhi2, Unigene95907\_JingChaoZuZhi2, Unigene96262\_JingChaoZuZhi2, Unigene9654\_JingChaoZuZhi2, Unigene9739\_JingChaoZuZhi2, Unigene9902\_JingChaoZuZhi2 |
| 86 | Systemic lupus erythematosus | Unigene10078\_JingChaoZuZhi2, Unigene11389\_JingChaoZuZhi2, Unigene11732\_JingChaoZuZhi2, Unigene12624\_JingChaoZuZhi2, Unigene1288\_JingChaoZuZhi2, Unigene13379\_JingChaoZuZhi2, Unigene14239\_JingChaoZuZhi2, Unigene15223\_JingChaoZuZhi2, Unigene16289\_JingChaoZuZhi2, Unigene16446\_JingChaoZuZhi2, Unigene16803\_JingChaoZuZhi2, Unigene16884\_JingChaoZuZhi2, Unigene17062\_JingChaoZuZhi2, Unigene1728\_JingChaoZuZhi2, Unigene17756\_JingChaoZuZhi2, Unigene19292\_JingChaoZuZhi2, Unigene20995\_JingChaoZuZhi2, Unigene21280\_JingChaoZuZhi2, Unigene21512\_JingChaoZuZhi2, Unigene22024\_JingChaoZuZhi2, Unigene22405\_JingChaoZuZhi2, Unigene25338\_JingChaoZuZhi2, Unigene27772\_JingChaoZuZhi2, Unigene30738\_JingChaoZuZhi2, Unigene31644\_JingChaoZuZhi2, Unigene31886\_JingChaoZuZhi2, Unigene32024\_JingChaoZuZhi2, Unigene32305\_JingChaoZuZhi2, Unigene3264\_JingChaoZuZhi2, Unigene33386\_JingChaoZuZhi2, Unigene34477\_JingChaoZuZhi2, Unigene34892\_JingChaoZuZhi2, Unigene34894\_JingChaoZuZhi2, Unigene36372\_JingChaoZuZhi2, Unigene38321\_JingChaoZuZhi2, Unigene38928\_JingChaoZuZhi2, Unigene39217\_JingChaoZuZhi2, Unigene39917\_JingChaoZuZhi2, Unigene40559\_JingChaoZuZhi2, Unigene40585\_JingChaoZuZhi2, Unigene43726\_JingChaoZuZhi2, Unigene45105\_JingChaoZuZhi2, Unigene47375\_JingChaoZuZhi2, Unigene4926\_JingChaoZuZhi2, Unigene51080\_JingChaoZuZhi2, Unigene5258\_JingChaoZuZhi2, Unigene53228\_JingChaoZuZhi2, Unigene53402\_JingChaoZuZhi2, Unigene54569\_JingChaoZuZhi2, Unigene54744\_JingChaoZuZhi2, Unigene54986\_JingChaoZuZhi2, Unigene57974\_JingChaoZuZhi2, Unigene58121\_JingChaoZuZhi2, Unigene58945\_JingChaoZuZhi2, Unigene59465\_JingChaoZuZhi2, Unigene59921\_JingChaoZuZhi2, Unigene60139\_JingChaoZuZhi2, Unigene60253\_JingChaoZuZhi2, Unigene61388\_JingChaoZuZhi2, Unigene62103\_JingChaoZuZhi2, Unigene63042\_JingChaoZuZhi2, Unigene63182\_JingChaoZuZhi2, Unigene63875\_JingChaoZuZhi2, Unigene66158\_JingChaoZuZhi2, Unigene66765\_JingChaoZuZhi2, Unigene67283\_JingChaoZuZhi2, Unigene67378\_JingChaoZuZhi2, Unigene67944\_JingChaoZuZhi2, Unigene7068\_JingChaoZuZhi2, Unigene7286\_JingChaoZuZhi2, Unigene73266\_JingChaoZuZhi2, Unigene75821\_JingChaoZuZhi2, Unigene76912\_JingChaoZuZhi2, Unigene76969\_JingChaoZuZhi2, Unigene77639\_JingChaoZuZhi2, Unigene77864\_JingChaoZuZhi2, Unigene79077\_JingChaoZuZhi2, Unigene80152\_JingChaoZuZhi2, Unigene81700\_JingChaoZuZhi2, Unigene82040\_JingChaoZuZhi2, Unigene82199\_JingChaoZuZhi2, Unigene82370\_JingChaoZuZhi2, Unigene82811\_JingChaoZuZhi2, Unigene82\_JingChaoZuZhi2, Unigene83003\_JingChaoZuZhi2, Unigene83808\_JingChaoZuZhi2, Unigene84044\_JingChaoZuZhi2, Unigene84909\_JingChaoZuZhi2, Unigene84939\_JingChaoZuZhi2, Unigene84993\_JingChaoZuZhi2, Unigene85221\_JingChaoZuZhi2, Unigene85431\_JingChaoZuZhi2, Unigene85923\_JingChaoZuZhi2, Unigene8596\_JingChaoZuZhi2, Unigene86309\_JingChaoZuZhi2, Unigene87191\_JingChaoZuZhi2, Unigene87543\_JingChaoZuZhi2, Unigene87946\_JingChaoZuZhi2, Unigene88264\_JingChaoZuZhi2, Unigene88422\_JingChaoZuZhi2, Unigene89045\_JingChaoZuZhi2, Unigene89057\_JingChaoZuZhi2, Unigene90003\_JingChaoZuZhi2, Unigene90307\_JingChaoZuZhi2, Unigene9070\_JingChaoZuZhi2, Unigene91174\_JingChaoZuZhi2, Unigene91994\_JingChaoZuZhi2, Unigene92175\_JingChaoZuZhi2, Unigene93834\_JingChaoZuZhi2, Unigene94214\_JingChaoZuZhi2, Unigene94370\_JingChaoZuZhi2, Unigene94825\_JingChaoZuZhi2, Unigene95020\_JingChaoZuZhi2, Unigene95042\_JingChaoZuZhi2, Unigene95501\_JingChaoZuZhi2, Unigene95807\_JingChaoZuZhi2, Unigene990\_JingChaoZuZhi2 |
| 87 | Fatty acid metabolism | Unigene11260\_JingChaoZuZhi2, Unigene11312\_JingChaoZuZhi2, Unigene12759\_JingChaoZuZhi2, Unigene13334\_JingChaoZuZhi2, Unigene14158\_JingChaoZuZhi2, Unigene16352\_JingChaoZuZhi2, Unigene16663\_JingChaoZuZhi2, Unigene16863\_JingChaoZuZhi2, Unigene16899\_JingChaoZuZhi2, Unigene17330\_JingChaoZuZhi2, Unigene17461\_JingChaoZuZhi2, Unigene17995\_JingChaoZuZhi2, Unigene18179\_JingChaoZuZhi2, Unigene18187\_JingChaoZuZhi2, Unigene18358\_JingChaoZuZhi2, Unigene18409\_JingChaoZuZhi2, Unigene18470\_JingChaoZuZhi2, Unigene18553\_JingChaoZuZhi2, Unigene18846\_JingChaoZuZhi2, Unigene19398\_JingChaoZuZhi2, Unigene21049\_JingChaoZuZhi2, Unigene21176\_JingChaoZuZhi2, Unigene21806\_JingChaoZuZhi2, Unigene21821\_JingChaoZuZhi2, Unigene22352\_JingChaoZuZhi2, Unigene2235\_JingChaoZuZhi2, Unigene22603\_JingChaoZuZhi2, Unigene22747\_JingChaoZuZhi2, Unigene22793\_JingChaoZuZhi2, Unigene22830\_JingChaoZuZhi2, Unigene22833\_JingChaoZuZhi2, Unigene240\_JingChaoZuZhi2, Unigene25361\_JingChaoZuZhi2, Unigene27219\_JingChaoZuZhi2, Unigene2761\_JingChaoZuZhi2, Unigene35187\_JingChaoZuZhi2, Unigene45176\_JingChaoZuZhi2, Unigene49819\_JingChaoZuZhi2, Unigene50371\_JingChaoZuZhi2, Unigene5068\_JingChaoZuZhi2, Unigene51532\_JingChaoZuZhi2, Unigene52317\_JingChaoZuZhi2, Unigene5518\_JingChaoZuZhi2, Unigene58763\_JingChaoZuZhi2, Unigene59548\_JingChaoZuZhi2, Unigene60610\_JingChaoZuZhi2, Unigene61162\_JingChaoZuZhi2, Unigene61664\_JingChaoZuZhi2, Unigene62962\_JingChaoZuZhi2, Unigene63649\_JingChaoZuZhi2, Unigene63715\_JingChaoZuZhi2, Unigene64903\_JingChaoZuZhi2, Unigene65441\_JingChaoZuZhi2, Unigene66337\_JingChaoZuZhi2, Unigene66815\_JingChaoZuZhi2, Unigene67613\_JingChaoZuZhi2, Unigene7053\_JingChaoZuZhi2, Unigene71252\_JingChaoZuZhi2, Unigene71936\_JingChaoZuZhi2, Unigene72476\_JingChaoZuZhi2, Unigene73332\_JingChaoZuZhi2, Unigene76323\_JingChaoZuZhi2, Unigene7821\_JingChaoZuZhi2, Unigene78854\_JingChaoZuZhi2, Unigene79066\_JingChaoZuZhi2, Unigene79135\_JingChaoZuZhi2, Unigene80446\_JingChaoZuZhi2, Unigene8075\_JingChaoZuZhi2, Unigene80881\_JingChaoZuZhi2, Unigene81214\_JingChaoZuZhi2, Unigene82129\_JingChaoZuZhi2, Unigene8213\_JingChaoZuZhi2, Unigene82435\_JingChaoZuZhi2, Unigene83066\_JingChaoZuZhi2, Unigene83411\_JingChaoZuZhi2, Unigene83441\_JingChaoZuZhi2, Unigene83670\_JingChaoZuZhi2, Unigene83795\_JingChaoZuZhi2, Unigene84063\_JingChaoZuZhi2, Unigene84124\_JingChaoZuZhi2, Unigene84343\_JingChaoZuZhi2, Unigene8454\_JingChaoZuZhi2, Unigene84851\_JingChaoZuZhi2, Unigene85224\_JingChaoZuZhi2, Unigene85989\_JingChaoZuZhi2, Unigene86141\_JingChaoZuZhi2, Unigene86715\_JingChaoZuZhi2, Unigene87044\_JingChaoZuZhi2, Unigene87485\_JingChaoZuZhi2, Unigene87856\_JingChaoZuZhi2, Unigene88045\_JingChaoZuZhi2, Unigene88377\_JingChaoZuZhi2, Unigene88858\_JingChaoZuZhi2, Unigene88914\_JingChaoZuZhi2, Unigene89280\_JingChaoZuZhi2, Unigene89465\_JingChaoZuZhi2, Unigene90127\_JingChaoZuZhi2, Unigene90838\_JingChaoZuZhi2, Unigene9097\_JingChaoZuZhi2, Unigene91700\_JingChaoZuZhi2, Unigene92256\_JingChaoZuZhi2, Unigene92517\_JingChaoZuZhi2, Unigene93033\_JingChaoZuZhi2, Unigene93143\_JingChaoZuZhi2, Unigene93334\_JingChaoZuZhi2, Unigene93635\_JingChaoZuZhi2, Unigene93681\_JingChaoZuZhi2, Unigene94837\_JingChaoZuZhi2, Unigene94902\_JingChaoZuZhi2, Unigene95397\_JingChaoZuZhi2, Unigene95535\_JingChaoZuZhi2, Unigene95561\_JingChaoZuZhi2, Unigene9580\_JingChaoZuZhi2, Unigene95959\_JingChaoZuZhi2, Unigene95985\_JingChaoZuZhi2, Unigene95986\_JingChaoZuZhi2, Unigene96031\_JingChaoZuZhi2 |
| 88 | ABC transporters | Unigene10779\_JingChaoZuZhi2, Unigene11711\_JingChaoZuZhi2, Unigene12147\_JingChaoZuZhi2, Unigene13722\_JingChaoZuZhi2, Unigene14329\_JingChaoZuZhi2, Unigene15081\_JingChaoZuZhi2, Unigene15684\_JingChaoZuZhi2, Unigene16325\_JingChaoZuZhi2, Unigene16792\_JingChaoZuZhi2, Unigene17046\_JingChaoZuZhi2, Unigene17398\_JingChaoZuZhi2, Unigene18000\_JingChaoZuZhi2, Unigene18044\_JingChaoZuZhi2, Unigene18844\_JingChaoZuZhi2, Unigene19427\_JingChaoZuZhi2, Unigene20058\_JingChaoZuZhi2, Unigene20175\_JingChaoZuZhi2, Unigene20628\_JingChaoZuZhi2, Unigene21545\_JingChaoZuZhi2, Unigene22108\_JingChaoZuZhi2, Unigene22416\_JingChaoZuZhi2, Unigene22798\_JingChaoZuZhi2, Unigene22921\_JingChaoZuZhi2, Unigene24800\_JingChaoZuZhi2, Unigene2553\_JingChaoZuZhi2, Unigene29655\_JingChaoZuZhi2, Unigene2997\_JingChaoZuZhi2, Unigene35436\_JingChaoZuZhi2, Unigene3564\_JingChaoZuZhi2, Unigene3757\_JingChaoZuZhi2, Unigene42889\_JingChaoZuZhi2, Unigene45177\_JingChaoZuZhi2, Unigene45202\_JingChaoZuZhi2, Unigene45533\_JingChaoZuZhi2, Unigene48335\_JingChaoZuZhi2, Unigene5066\_JingChaoZuZhi2, Unigene5335\_JingChaoZuZhi2, Unigene55075\_JingChaoZuZhi2, Unigene55485\_JingChaoZuZhi2, Unigene56894\_JingChaoZuZhi2, Unigene56904\_JingChaoZuZhi2, Unigene57866\_JingChaoZuZhi2, Unigene57\_JingChaoZuZhi2, Unigene58028\_JingChaoZuZhi2, Unigene58546\_JingChaoZuZhi2, Unigene59836\_JingChaoZuZhi2, Unigene61005\_JingChaoZuZhi2, Unigene6339\_JingChaoZuZhi2, Unigene64662\_JingChaoZuZhi2, Unigene6494\_JingChaoZuZhi2, Unigene66174\_JingChaoZuZhi2, Unigene66433\_JingChaoZuZhi2, Unigene66446\_JingChaoZuZhi2, Unigene6748\_JingChaoZuZhi2, Unigene6756\_JingChaoZuZhi2, Unigene69785\_JingChaoZuZhi2, Unigene70322\_JingChaoZuZhi2, Unigene70340\_JingChaoZuZhi2, Unigene70606\_JingChaoZuZhi2, Unigene71509\_JingChaoZuZhi2, Unigene71763\_JingChaoZuZhi2, Unigene72862\_JingChaoZuZhi2, Unigene72947\_JingChaoZuZhi2, Unigene73804\_JingChaoZuZhi2, Unigene7424\_JingChaoZuZhi2, Unigene75174\_JingChaoZuZhi2, Unigene75815\_JingChaoZuZhi2, Unigene76061\_JingChaoZuZhi2, Unigene76237\_JingChaoZuZhi2, Unigene77324\_JingChaoZuZhi2, Unigene7856\_JingChaoZuZhi2, Unigene7934\_JingChaoZuZhi2, Unigene80206\_JingChaoZuZhi2, Unigene80322\_JingChaoZuZhi2, Unigene81114\_JingChaoZuZhi2, Unigene82174\_JingChaoZuZhi2, Unigene82263\_JingChaoZuZhi2, Unigene82686\_JingChaoZuZhi2, Unigene83364\_JingChaoZuZhi2, Unigene83496\_JingChaoZuZhi2, Unigene83850\_JingChaoZuZhi2, Unigene83971\_JingChaoZuZhi2, Unigene84031\_JingChaoZuZhi2, Unigene84141\_JingChaoZuZhi2, Unigene84245\_JingChaoZuZhi2, Unigene84693\_JingChaoZuZhi2, Unigene85212\_JingChaoZuZhi2, Unigene85932\_JingChaoZuZhi2, Unigene87799\_JingChaoZuZhi2, Unigene87944\_JingChaoZuZhi2, Unigene88263\_JingChaoZuZhi2, Unigene88452\_JingChaoZuZhi2, Unigene88575\_JingChaoZuZhi2, Unigene8862\_JingChaoZuZhi2, Unigene89256\_JingChaoZuZhi2, Unigene89805\_JingChaoZuZhi2, Unigene89856\_JingChaoZuZhi2, Unigene89962\_JingChaoZuZhi2, Unigene9030\_JingChaoZuZhi2, Unigene90758\_JingChaoZuZhi2, Unigene90999\_JingChaoZuZhi2, Unigene91245\_JingChaoZuZhi2, Unigene91355\_JingChaoZuZhi2, Unigene91914\_JingChaoZuZhi2, Unigene92691\_JingChaoZuZhi2, Unigene9306\_JingChaoZuZhi2, Unigene93471\_JingChaoZuZhi2, Unigene93598\_JingChaoZuZhi2, Unigene93666\_JingChaoZuZhi2, Unigene936\_JingChaoZuZhi2, Unigene93886\_JingChaoZuZhi2, Unigene94644\_JingChaoZuZhi2, Unigene94788\_JingChaoZuZhi2, Unigene94871\_JingChaoZuZhi2, Unigene95048\_JingChaoZuZhi2, Unigene95155\_JingChaoZuZhi2 |
| 89 | Nucleotide excision repair | Unigene10802\_JingChaoZuZhi2, Unigene11389\_JingChaoZuZhi2, Unigene11402\_JingChaoZuZhi2, Unigene11558\_JingChaoZuZhi2, Unigene11894\_JingChaoZuZhi2, Unigene12436\_JingChaoZuZhi2, Unigene12617\_JingChaoZuZhi2, Unigene13861\_JingChaoZuZhi2, Unigene1433\_JingChaoZuZhi2, Unigene14463\_JingChaoZuZhi2, Unigene14592\_JingChaoZuZhi2, Unigene1520\_JingChaoZuZhi2, Unigene16653\_JingChaoZuZhi2, Unigene1695\_JingChaoZuZhi2, Unigene170\_JingChaoZuZhi2, Unigene1738\_JingChaoZuZhi2, Unigene17491\_JingChaoZuZhi2, Unigene17673\_JingChaoZuZhi2, Unigene17734\_JingChaoZuZhi2, Unigene17890\_JingChaoZuZhi2, Unigene18862\_JingChaoZuZhi2, Unigene1888\_JingChaoZuZhi2, Unigene1902\_JingChaoZuZhi2, Unigene20525\_JingChaoZuZhi2, Unigene20612\_JingChaoZuZhi2, Unigene21282\_JingChaoZuZhi2, Unigene21305\_JingChaoZuZhi2, Unigene21781\_JingChaoZuZhi2, Unigene22259\_JingChaoZuZhi2, Unigene22304\_JingChaoZuZhi2, Unigene22578\_JingChaoZuZhi2, Unigene22817\_JingChaoZuZhi2, Unigene23468\_JingChaoZuZhi2, Unigene2354\_JingChaoZuZhi2, Unigene25056\_JingChaoZuZhi2, Unigene27296\_JingChaoZuZhi2, Unigene35605\_JingChaoZuZhi2, Unigene35680\_JingChaoZuZhi2, Unigene35687\_JingChaoZuZhi2, Unigene35730\_JingChaoZuZhi2, Unigene3974\_JingChaoZuZhi2, Unigene41651\_JingChaoZuZhi2, Unigene41885\_JingChaoZuZhi2, Unigene460\_JingChaoZuZhi2, Unigene49368\_JingChaoZuZhi2, Unigene49812\_JingChaoZuZhi2, Unigene51032\_JingChaoZuZhi2, Unigene55490\_JingChaoZuZhi2, Unigene57163\_JingChaoZuZhi2, Unigene57726\_JingChaoZuZhi2, Unigene57931\_JingChaoZuZhi2, Unigene58443\_JingChaoZuZhi2, Unigene5882\_JingChaoZuZhi2, Unigene59182\_JingChaoZuZhi2, Unigene5948\_JingChaoZuZhi2, Unigene6073\_JingChaoZuZhi2, Unigene6233\_JingChaoZuZhi2, Unigene63198\_JingChaoZuZhi2, Unigene6370\_JingChaoZuZhi2, Unigene64315\_JingChaoZuZhi2, Unigene6461\_JingChaoZuZhi2, Unigene64812\_JingChaoZuZhi2, Unigene6623\_JingChaoZuZhi2, Unigene67564\_JingChaoZuZhi2, Unigene6913\_JingChaoZuZhi2, Unigene6944\_JingChaoZuZhi2, Unigene71482\_JingChaoZuZhi2, Unigene72771\_JingChaoZuZhi2, Unigene74540\_JingChaoZuZhi2, Unigene75555\_JingChaoZuZhi2, Unigene75981\_JingChaoZuZhi2, Unigene76639\_JingChaoZuZhi2, Unigene78612\_JingChaoZuZhi2, Unigene78673\_JingChaoZuZhi2, Unigene78813\_JingChaoZuZhi2, Unigene79005\_JingChaoZuZhi2, Unigene79036\_JingChaoZuZhi2, Unigene81603\_JingChaoZuZhi2, Unigene8245\_JingChaoZuZhi2, Unigene82698\_JingChaoZuZhi2, Unigene83287\_JingChaoZuZhi2, Unigene84227\_JingChaoZuZhi2, Unigene86037\_JingChaoZuZhi2, Unigene86708\_JingChaoZuZhi2, Unigene86938\_JingChaoZuZhi2, Unigene87989\_JingChaoZuZhi2, Unigene88770\_JingChaoZuZhi2, Unigene89645\_JingChaoZuZhi2, Unigene90104\_JingChaoZuZhi2, Unigene90653\_JingChaoZuZhi2, Unigene90781\_JingChaoZuZhi2, Unigene91096\_JingChaoZuZhi2, Unigene92389\_JingChaoZuZhi2, Unigene92803\_JingChaoZuZhi2, Unigene92825\_JingChaoZuZhi2, Unigene93191\_JingChaoZuZhi2, Unigene93305\_JingChaoZuZhi2, Unigene93454\_JingChaoZuZhi2, Unigene93619\_JingChaoZuZhi2, Unigene93812\_JingChaoZuZhi2, Unigene93971\_JingChaoZuZhi2, Unigene94055\_JingChaoZuZhi2, Unigene94187\_JingChaoZuZhi2, Unigene94583\_JingChaoZuZhi2, Unigene94602\_JingChaoZuZhi2, Unigene94875\_JingChaoZuZhi2, Unigene95055\_JingChaoZuZhi2, Unigene95153\_JingChaoZuZhi2, Unigene95309\_JingChaoZuZhi2, Unigene95352\_JingChaoZuZhi2, Unigene95381\_JingChaoZuZhi2, Unigene95389\_JingChaoZuZhi2, Unigene95444\_JingChaoZuZhi2, Unigene95632\_JingChaoZuZhi2, Unigene95819\_JingChaoZuZhi2 |
| 90 | Colorectal cancer | Unigene10986\_JingChaoZuZhi2, Unigene11190\_JingChaoZuZhi2, Unigene11951\_JingChaoZuZhi2, Unigene11961\_JingChaoZuZhi2, Unigene14912\_JingChaoZuZhi2, Unigene15228\_JingChaoZuZhi2, Unigene16229\_JingChaoZuZhi2, Unigene16820\_JingChaoZuZhi2, Unigene17247\_JingChaoZuZhi2, Unigene17800\_JingChaoZuZhi2, Unigene18092\_JingChaoZuZhi2, Unigene18095\_JingChaoZuZhi2, Unigene18542\_JingChaoZuZhi2, Unigene18672\_JingChaoZuZhi2, Unigene18715\_JingChaoZuZhi2, Unigene18728\_JingChaoZuZhi2, Unigene18817\_JingChaoZuZhi2, Unigene18932\_JingChaoZuZhi2, Unigene19021\_JingChaoZuZhi2, Unigene19465\_JingChaoZuZhi2, Unigene19813\_JingChaoZuZhi2, Unigene20803\_JingChaoZuZhi2, Unigene21513\_JingChaoZuZhi2, Unigene2245\_JingChaoZuZhi2, Unigene22522\_JingChaoZuZhi2, Unigene22810\_JingChaoZuZhi2, Unigene2456\_JingChaoZuZhi2, Unigene27478\_JingChaoZuZhi2, Unigene28421\_JingChaoZuZhi2, Unigene30787\_JingChaoZuZhi2, Unigene33683\_JingChaoZuZhi2, Unigene34463\_JingChaoZuZhi2, Unigene3640\_JingChaoZuZhi2, Unigene37739\_JingChaoZuZhi2, Unigene3931\_JingChaoZuZhi2, Unigene39584\_JingChaoZuZhi2, Unigene4155\_JingChaoZuZhi2, Unigene4294\_JingChaoZuZhi2, Unigene43120\_JingChaoZuZhi2, Unigene44061\_JingChaoZuZhi2, Unigene46240\_JingChaoZuZhi2, Unigene48751\_JingChaoZuZhi2, Unigene4886\_JingChaoZuZhi2, Unigene5252\_JingChaoZuZhi2, Unigene5314\_JingChaoZuZhi2, Unigene53421\_JingChaoZuZhi2, Unigene5578\_JingChaoZuZhi2, Unigene56709\_JingChaoZuZhi2, Unigene57488\_JingChaoZuZhi2, Unigene58136\_JingChaoZuZhi2, Unigene58806\_JingChaoZuZhi2, Unigene59011\_JingChaoZuZhi2, Unigene6028\_JingChaoZuZhi2, Unigene61760\_JingChaoZuZhi2, Unigene61911\_JingChaoZuZhi2, Unigene63449\_JingChaoZuZhi2, Unigene64227\_JingChaoZuZhi2, Unigene64678\_JingChaoZuZhi2, Unigene66933\_JingChaoZuZhi2, Unigene67177\_JingChaoZuZhi2, Unigene6785\_JingChaoZuZhi2, Unigene68056\_JingChaoZuZhi2, Unigene68848\_JingChaoZuZhi2, Unigene69517\_JingChaoZuZhi2, Unigene70549\_JingChaoZuZhi2, Unigene71412\_JingChaoZuZhi2, Unigene71424\_JingChaoZuZhi2, Unigene71893\_JingChaoZuZhi2, Unigene7204\_JingChaoZuZhi2, Unigene72769\_JingChaoZuZhi2, Unigene73870\_JingChaoZuZhi2, Unigene74377\_JingChaoZuZhi2, Unigene78390\_JingChaoZuZhi2, Unigene79912\_JingChaoZuZhi2, Unigene80067\_JingChaoZuZhi2, Unigene8191\_JingChaoZuZhi2, Unigene84930\_JingChaoZuZhi2, Unigene8566\_JingChaoZuZhi2, Unigene8600\_JingChaoZuZhi2, Unigene86206\_JingChaoZuZhi2, Unigene86526\_JingChaoZuZhi2, Unigene86817\_JingChaoZuZhi2, Unigene876\_JingChaoZuZhi2, Unigene8799\_JingChaoZuZhi2, Unigene8851\_JingChaoZuZhi2, Unigene88544\_JingChaoZuZhi2, Unigene88719\_JingChaoZuZhi2, Unigene89310\_JingChaoZuZhi2, Unigene89315\_JingChaoZuZhi2, Unigene91179\_JingChaoZuZhi2, Unigene91275\_JingChaoZuZhi2, Unigene91580\_JingChaoZuZhi2, Unigene92019\_JingChaoZuZhi2, Unigene92112\_JingChaoZuZhi2, Unigene92309\_JingChaoZuZhi2, Unigene92738\_JingChaoZuZhi2, Unigene93133\_JingChaoZuZhi2, Unigene93807\_JingChaoZuZhi2, Unigene93953\_JingChaoZuZhi2, Unigene94272\_JingChaoZuZhi2, Unigene94685\_JingChaoZuZhi2, Unigene94795\_JingChaoZuZhi2, Unigene95014\_JingChaoZuZhi2, Unigene95098\_JingChaoZuZhi2, Unigene95295\_JingChaoZuZhi2, Unigene95315\_JingChaoZuZhi2, Unigene95875\_JingChaoZuZhi2, Unigene95881\_JingChaoZuZhi2, Unigene95883\_JingChaoZuZhi2, Unigene96234\_JingChaoZuZhi2, Unigene96294\_JingChaoZuZhi2, Unigene9902\_JingChaoZuZhi2 |
| 91 | Adipocytokine signaling pathway | Unigene10856\_JingChaoZuZhi2, Unigene11260\_JingChaoZuZhi2, Unigene11719\_JingChaoZuZhi2, Unigene11951\_JingChaoZuZhi2, Unigene12202\_JingChaoZuZhi2, Unigene12716\_JingChaoZuZhi2, Unigene12759\_JingChaoZuZhi2, Unigene12813\_JingChaoZuZhi2, Unigene12823\_JingChaoZuZhi2, Unigene12899\_JingChaoZuZhi2, Unigene13008\_JingChaoZuZhi2, Unigene14534\_JingChaoZuZhi2, Unigene14738\_JingChaoZuZhi2, Unigene14877\_JingChaoZuZhi2, Unigene16768\_JingChaoZuZhi2, Unigene17330\_JingChaoZuZhi2, Unigene17839\_JingChaoZuZhi2, Unigene17888\_JingChaoZuZhi2, Unigene18187\_JingChaoZuZhi2, Unigene18729\_JingChaoZuZhi2, Unigene18821\_JingChaoZuZhi2, Unigene19086\_JingChaoZuZhi2, Unigene19235\_JingChaoZuZhi2, Unigene19307\_JingChaoZuZhi2, Unigene19998\_JingChaoZuZhi2, Unigene20129\_JingChaoZuZhi2, Unigene20157\_JingChaoZuZhi2, Unigene20840\_JingChaoZuZhi2, Unigene20991\_JingChaoZuZhi2, Unigene21132\_JingChaoZuZhi2, Unigene21578\_JingChaoZuZhi2, Unigene21697\_JingChaoZuZhi2, Unigene21806\_JingChaoZuZhi2, Unigene22466\_JingChaoZuZhi2, Unigene22585\_JingChaoZuZhi2, Unigene22830\_JingChaoZuZhi2, Unigene22833\_JingChaoZuZhi2, Unigene229\_JingChaoZuZhi2, Unigene2761\_JingChaoZuZhi2, Unigene32031\_JingChaoZuZhi2, Unigene3769\_JingChaoZuZhi2, Unigene3931\_JingChaoZuZhi2, Unigene4223\_JingChaoZuZhi2, Unigene4294\_JingChaoZuZhi2, Unigene51478\_JingChaoZuZhi2, Unigene52040\_JingChaoZuZhi2, Unigene52317\_JingChaoZuZhi2, Unigene52554\_JingChaoZuZhi2, Unigene52793\_JingChaoZuZhi2, Unigene54320\_JingChaoZuZhi2, Unigene5437\_JingChaoZuZhi2, Unigene55687\_JingChaoZuZhi2, Unigene5638\_JingChaoZuZhi2, Unigene5674\_JingChaoZuZhi2, Unigene5742\_JingChaoZuZhi2, Unigene5823\_JingChaoZuZhi2, Unigene60610\_JingChaoZuZhi2, Unigene60906\_JingChaoZuZhi2, Unigene61033\_JingChaoZuZhi2, Unigene66060\_JingChaoZuZhi2, Unigene68322\_JingChaoZuZhi2, Unigene71936\_JingChaoZuZhi2, Unigene75364\_JingChaoZuZhi2, Unigene76323\_JingChaoZuZhi2, Unigene77207\_JingChaoZuZhi2, Unigene77570\_JingChaoZuZhi2, Unigene7821\_JingChaoZuZhi2, Unigene79066\_JingChaoZuZhi2, Unigene79860\_JingChaoZuZhi2, Unigene79911\_JingChaoZuZhi2, Unigene80504\_JingChaoZuZhi2, Unigene8075\_JingChaoZuZhi2, Unigene80971\_JingChaoZuZhi2, Unigene83318\_JingChaoZuZhi2, Unigene83411\_JingChaoZuZhi2, Unigene83531\_JingChaoZuZhi2, Unigene83670\_JingChaoZuZhi2, Unigene84024\_JingChaoZuZhi2, Unigene84340\_JingChaoZuZhi2, Unigene8464\_JingChaoZuZhi2, Unigene84862\_JingChaoZuZhi2, Unigene85172\_JingChaoZuZhi2, Unigene85731\_JingChaoZuZhi2, Unigene85769\_JingChaoZuZhi2, Unigene86141\_JingChaoZuZhi2, Unigene86715\_JingChaoZuZhi2, Unigene86899\_JingChaoZuZhi2, Unigene87251\_JingChaoZuZhi2, Unigene87278\_JingChaoZuZhi2, Unigene87305\_JingChaoZuZhi2, Unigene88045\_JingChaoZuZhi2, Unigene88858\_JingChaoZuZhi2, Unigene89280\_JingChaoZuZhi2, Unigene89465\_JingChaoZuZhi2, Unigene90209\_JingChaoZuZhi2, Unigene90879\_JingChaoZuZhi2, Unigene9097\_JingChaoZuZhi2, Unigene91175\_JingChaoZuZhi2, Unigene91816\_JingChaoZuZhi2, Unigene91895\_JingChaoZuZhi2, Unigene91919\_JingChaoZuZhi2, Unigene92580\_JingChaoZuZhi2, Unigene93409\_JingChaoZuZhi2, Unigene93774\_JingChaoZuZhi2, Unigene9389\_JingChaoZuZhi2, Unigene94178\_JingChaoZuZhi2, Unigene94474\_JingChaoZuZhi2, Unigene95454\_JingChaoZuZhi2, Unigene95535\_JingChaoZuZhi2 |
| 92 | PPAR signaling pathway | Unigene11260\_JingChaoZuZhi2, Unigene12202\_JingChaoZuZhi2, Unigene1227\_JingChaoZuZhi2, Unigene12492\_JingChaoZuZhi2, Unigene12759\_JingChaoZuZhi2, Unigene13107\_JingChaoZuZhi2, Unigene13334\_JingChaoZuZhi2, Unigene14158\_JingChaoZuZhi2, Unigene14534\_JingChaoZuZhi2, Unigene16663\_JingChaoZuZhi2, Unigene16899\_JingChaoZuZhi2, Unigene17028\_JingChaoZuZhi2, Unigene17217\_JingChaoZuZhi2, Unigene17330\_JingChaoZuZhi2, Unigene18187\_JingChaoZuZhi2, Unigene18846\_JingChaoZuZhi2, Unigene20760\_JingChaoZuZhi2, Unigene21718\_JingChaoZuZhi2, Unigene21806\_JingChaoZuZhi2, Unigene22295\_JingChaoZuZhi2, Unigene22352\_JingChaoZuZhi2, Unigene22830\_JingChaoZuZhi2, Unigene22833\_JingChaoZuZhi2, Unigene24512\_JingChaoZuZhi2, Unigene2761\_JingChaoZuZhi2, Unigene32031\_JingChaoZuZhi2, Unigene3467\_JingChaoZuZhi2, Unigene35187\_JingChaoZuZhi2, Unigene37495\_JingChaoZuZhi2, Unigene40020\_JingChaoZuZhi2, Unigene4233\_JingChaoZuZhi2, Unigene45176\_JingChaoZuZhi2, Unigene46661\_JingChaoZuZhi2, Unigene47032\_JingChaoZuZhi2, Unigene48660\_JingChaoZuZhi2, Unigene49819\_JingChaoZuZhi2, Unigene50937\_JingChaoZuZhi2, Unigene51478\_JingChaoZuZhi2, Unigene51741\_JingChaoZuZhi2, Unigene52259\_JingChaoZuZhi2, Unigene52317\_JingChaoZuZhi2, Unigene52\_JingChaoZuZhi2, Unigene54890\_JingChaoZuZhi2, Unigene5663\_JingChaoZuZhi2, Unigene59548\_JingChaoZuZhi2, Unigene60117\_JingChaoZuZhi2, Unigene60610\_JingChaoZuZhi2, Unigene61033\_JingChaoZuZhi2, Unigene61162\_JingChaoZuZhi2, Unigene62962\_JingChaoZuZhi2, Unigene65441\_JingChaoZuZhi2, Unigene67983\_JingChaoZuZhi2, Unigene68283\_JingChaoZuZhi2, Unigene68474\_JingChaoZuZhi2, Unigene71252\_JingChaoZuZhi2, Unigene71936\_JingChaoZuZhi2, Unigene73332\_JingChaoZuZhi2, Unigene73451\_JingChaoZuZhi2, Unigene74590\_JingChaoZuZhi2, Unigene76323\_JingChaoZuZhi2, Unigene77454\_JingChaoZuZhi2, Unigene78083\_JingChaoZuZhi2, Unigene7821\_JingChaoZuZhi2, Unigene78631\_JingChaoZuZhi2, Unigene78854\_JingChaoZuZhi2, Unigene79066\_JingChaoZuZhi2, Unigene79135\_JingChaoZuZhi2, Unigene79414\_JingChaoZuZhi2, Unigene80312\_JingChaoZuZhi2, Unigene80446\_JingChaoZuZhi2, Unigene80504\_JingChaoZuZhi2, Unigene8075\_JingChaoZuZhi2, Unigene8213\_JingChaoZuZhi2, Unigene82435\_JingChaoZuZhi2, Unigene83066\_JingChaoZuZhi2, Unigene83411\_JingChaoZuZhi2, Unigene83441\_JingChaoZuZhi2, Unigene83531\_JingChaoZuZhi2, Unigene83670\_JingChaoZuZhi2, Unigene84124\_JingChaoZuZhi2, Unigene8442\_JingChaoZuZhi2, Unigene85989\_JingChaoZuZhi2, Unigene86141\_JingChaoZuZhi2, Unigene86278\_JingChaoZuZhi2, Unigene86456\_JingChaoZuZhi2, Unigene86715\_JingChaoZuZhi2, Unigene88045\_JingChaoZuZhi2, Unigene88233\_JingChaoZuZhi2, Unigene88377\_JingChaoZuZhi2, Unigene88858\_JingChaoZuZhi2, Unigene89280\_JingChaoZuZhi2, Unigene89465\_JingChaoZuZhi2, Unigene89497\_JingChaoZuZhi2, Unigene9020\_JingChaoZuZhi2, Unigene90859\_JingChaoZuZhi2, Unigene9097\_JingChaoZuZhi2, Unigene91175\_JingChaoZuZhi2, Unigene92443\_JingChaoZuZhi2, Unigene92489\_JingChaoZuZhi2, Unigene93275\_JingChaoZuZhi2, Unigene93437\_JingChaoZuZhi2, Unigene93810\_JingChaoZuZhi2, Unigene93924\_JingChaoZuZhi2, Unigene94759\_JingChaoZuZhi2, Unigene95397\_JingChaoZuZhi2, Unigene95535\_JingChaoZuZhi2, Unigene96129\_JingChaoZuZhi2 |
| 93 | Ribosome | Unigene10639\_JingChaoZuZhi2, Unigene13528\_JingChaoZuZhi2, Unigene20115\_JingChaoZuZhi2, Unigene24218\_JingChaoZuZhi2, Unigene24797\_JingChaoZuZhi2, Unigene26325\_JingChaoZuZhi2, Unigene27466\_JingChaoZuZhi2, Unigene28090\_JingChaoZuZhi2, Unigene29643\_JingChaoZuZhi2, Unigene30251\_JingChaoZuZhi2, Unigene33703\_JingChaoZuZhi2, Unigene36041\_JingChaoZuZhi2, Unigene36466\_JingChaoZuZhi2, Unigene36481\_JingChaoZuZhi2, Unigene37126\_JingChaoZuZhi2, Unigene38037\_JingChaoZuZhi2, Unigene38172\_JingChaoZuZhi2, Unigene38626\_JingChaoZuZhi2, Unigene38629\_JingChaoZuZhi2, Unigene41268\_JingChaoZuZhi2, Unigene42704\_JingChaoZuZhi2, Unigene42777\_JingChaoZuZhi2, Unigene42952\_JingChaoZuZhi2, Unigene42992\_JingChaoZuZhi2, Unigene43465\_JingChaoZuZhi2, Unigene43547\_JingChaoZuZhi2, Unigene43685\_JingChaoZuZhi2, Unigene43713\_JingChaoZuZhi2, Unigene43976\_JingChaoZuZhi2, Unigene46689\_JingChaoZuZhi2, Unigene46977\_JingChaoZuZhi2, Unigene47048\_JingChaoZuZhi2, Unigene47432\_JingChaoZuZhi2, Unigene48561\_JingChaoZuZhi2, Unigene49096\_JingChaoZuZhi2, Unigene50426\_JingChaoZuZhi2, Unigene51797\_JingChaoZuZhi2, Unigene51930\_JingChaoZuZhi2, Unigene52310\_JingChaoZuZhi2, Unigene52675\_JingChaoZuZhi2, Unigene53065\_JingChaoZuZhi2, Unigene53134\_JingChaoZuZhi2, Unigene53593\_JingChaoZuZhi2, Unigene53873\_JingChaoZuZhi2, Unigene53874\_JingChaoZuZhi2, Unigene54015\_JingChaoZuZhi2, Unigene54938\_JingChaoZuZhi2, Unigene55335\_JingChaoZuZhi2, Unigene55491\_JingChaoZuZhi2, Unigene56264\_JingChaoZuZhi2, Unigene56730\_JingChaoZuZhi2, Unigene57177\_JingChaoZuZhi2, Unigene57391\_JingChaoZuZhi2, Unigene57512\_JingChaoZuZhi2, Unigene58159\_JingChaoZuZhi2, Unigene58722\_JingChaoZuZhi2, Unigene58891\_JingChaoZuZhi2, Unigene59789\_JingChaoZuZhi2, Unigene59966\_JingChaoZuZhi2, Unigene61063\_JingChaoZuZhi2, Unigene62360\_JingChaoZuZhi2, Unigene63414\_JingChaoZuZhi2, Unigene64128\_JingChaoZuZhi2, Unigene64632\_JingChaoZuZhi2, Unigene64740\_JingChaoZuZhi2, Unigene65011\_JingChaoZuZhi2, Unigene65147\_JingChaoZuZhi2, Unigene65293\_JingChaoZuZhi2, Unigene67643\_JingChaoZuZhi2, Unigene68347\_JingChaoZuZhi2, Unigene68869\_JingChaoZuZhi2, Unigene69502\_JingChaoZuZhi2, Unigene69533\_JingChaoZuZhi2, Unigene69769\_JingChaoZuZhi2, Unigene69847\_JingChaoZuZhi2, Unigene70238\_JingChaoZuZhi2, Unigene72263\_JingChaoZuZhi2, Unigene72884\_JingChaoZuZhi2, Unigene73069\_JingChaoZuZhi2, Unigene74775\_JingChaoZuZhi2, Unigene75069\_JingChaoZuZhi2, Unigene75767\_JingChaoZuZhi2, Unigene78919\_JingChaoZuZhi2, Unigene80445\_JingChaoZuZhi2, Unigene80944\_JingChaoZuZhi2, Unigene81205\_JingChaoZuZhi2, Unigene82613\_JingChaoZuZhi2, Unigene82690\_JingChaoZuZhi2, Unigene82914\_JingChaoZuZhi2, Unigene84000\_JingChaoZuZhi2, Unigene84653\_JingChaoZuZhi2, Unigene84976\_JingChaoZuZhi2, Unigene84984\_JingChaoZuZhi2, Unigene86523\_JingChaoZuZhi2, Unigene86571\_JingChaoZuZhi2, Unigene87273\_JingChaoZuZhi2, Unigene87732\_JingChaoZuZhi2, Unigene90496\_JingChaoZuZhi2, Unigene91293\_JingChaoZuZhi2, Unigene91376\_JingChaoZuZhi2, Unigene91561\_JingChaoZuZhi2, Unigene91989\_JingChaoZuZhi2, Unigene9237\_JingChaoZuZhi2, Unigene92725\_JingChaoZuZhi2, Unigene93530\_JingChaoZuZhi2, Unigene94490\_JingChaoZuZhi2, Unigene95855\_JingChaoZuZhi2 |
| 94 | Chronic myeloid leukemia | Unigene10463\_JingChaoZuZhi2, Unigene10926\_JingChaoZuZhi2, Unigene11961\_JingChaoZuZhi2, Unigene12420\_JingChaoZuZhi2, Unigene12813\_JingChaoZuZhi2, Unigene12836\_JingChaoZuZhi2, Unigene12994\_JingChaoZuZhi2, Unigene13077\_JingChaoZuZhi2, Unigene13510\_JingChaoZuZhi2, Unigene14738\_JingChaoZuZhi2, Unigene14912\_JingChaoZuZhi2, Unigene15228\_JingChaoZuZhi2, Unigene17247\_JingChaoZuZhi2, Unigene17467\_JingChaoZuZhi2, Unigene17764\_JingChaoZuZhi2, Unigene18672\_JingChaoZuZhi2, Unigene18986\_JingChaoZuZhi2, Unigene19046\_JingChaoZuZhi2, Unigene19551\_JingChaoZuZhi2, Unigene19813\_JingChaoZuZhi2, Unigene20009\_JingChaoZuZhi2, Unigene20157\_JingChaoZuZhi2, Unigene20875\_JingChaoZuZhi2, Unigene20991\_JingChaoZuZhi2, Unigene21010\_JingChaoZuZhi2, Unigene21513\_JingChaoZuZhi2, Unigene21515\_JingChaoZuZhi2, Unigene21697\_JingChaoZuZhi2, Unigene22466\_JingChaoZuZhi2, Unigene22500\_JingChaoZuZhi2, Unigene2456\_JingChaoZuZhi2, Unigene3640\_JingChaoZuZhi2, Unigene38371\_JingChaoZuZhi2, Unigene3931\_JingChaoZuZhi2, Unigene39584\_JingChaoZuZhi2, Unigene4186\_JingChaoZuZhi2, Unigene4223\_JingChaoZuZhi2, Unigene42755\_JingChaoZuZhi2, Unigene42981\_JingChaoZuZhi2, Unigene46240\_JingChaoZuZhi2, Unigene4838\_JingChaoZuZhi2, Unigene48751\_JingChaoZuZhi2, Unigene51285\_JingChaoZuZhi2, Unigene54320\_JingChaoZuZhi2, Unigene55677\_JingChaoZuZhi2, Unigene5621\_JingChaoZuZhi2, Unigene56227\_JingChaoZuZhi2, Unigene5638\_JingChaoZuZhi2, Unigene56642\_JingChaoZuZhi2, Unigene57488\_JingChaoZuZhi2, Unigene5758\_JingChaoZuZhi2, Unigene6028\_JingChaoZuZhi2, Unigene60453\_JingChaoZuZhi2, Unigene6159\_JingChaoZuZhi2, Unigene6493\_JingChaoZuZhi2, Unigene66813\_JingChaoZuZhi2, Unigene67177\_JingChaoZuZhi2, Unigene67793\_JingChaoZuZhi2, Unigene6785\_JingChaoZuZhi2, Unigene70549\_JingChaoZuZhi2, Unigene7238\_JingChaoZuZhi2, Unigene72769\_JingChaoZuZhi2, Unigene74356\_JingChaoZuZhi2, Unigene7452\_JingChaoZuZhi2, Unigene76023\_JingChaoZuZhi2, Unigene76099\_JingChaoZuZhi2, Unigene76809\_JingChaoZuZhi2, Unigene77570\_JingChaoZuZhi2, Unigene79683\_JingChaoZuZhi2, Unigene79882\_JingChaoZuZhi2, Unigene8191\_JingChaoZuZhi2, Unigene84862\_JingChaoZuZhi2, Unigene84930\_JingChaoZuZhi2, Unigene85574\_JingChaoZuZhi2, Unigene8563\_JingChaoZuZhi2, Unigene85863\_JingChaoZuZhi2, Unigene8600\_JingChaoZuZhi2, Unigene8609\_JingChaoZuZhi2, Unigene86206\_JingChaoZuZhi2, Unigene86526\_JingChaoZuZhi2, Unigene87654\_JingChaoZuZhi2, Unigene876\_JingChaoZuZhi2, Unigene87730\_JingChaoZuZhi2, Unigene8982\_JingChaoZuZhi2, Unigene90979\_JingChaoZuZhi2, Unigene91100\_JingChaoZuZhi2, Unigene91612\_JingChaoZuZhi2, Unigene91689\_JingChaoZuZhi2, Unigene91958\_JingChaoZuZhi2, Unigene92019\_JingChaoZuZhi2, Unigene92112\_JingChaoZuZhi2, Unigene93807\_JingChaoZuZhi2, Unigene9404\_JingChaoZuZhi2, Unigene94138\_JingChaoZuZhi2, Unigene94149\_JingChaoZuZhi2, Unigene94208\_JingChaoZuZhi2, Unigene94456\_JingChaoZuZhi2, Unigene94544\_JingChaoZuZhi2, Unigene94685\_JingChaoZuZhi2, Unigene95315\_JingChaoZuZhi2, Unigene95456\_JingChaoZuZhi2, Unigene95723\_JingChaoZuZhi2, Unigene95883\_JingChaoZuZhi2, Unigene96018\_JingChaoZuZhi2, Unigene96281\_JingChaoZuZhi2, Unigene96294\_JingChaoZuZhi2, Unigene9902\_JingChaoZuZhi2 |
| 95 | Aminoacyl-tRNA biosynthesis | Unigene10712\_JingChaoZuZhi2, Unigene10831\_JingChaoZuZhi2, Unigene1118\_JingChaoZuZhi2, Unigene11229\_JingChaoZuZhi2, Unigene11349\_JingChaoZuZhi2, Unigene11821\_JingChaoZuZhi2, Unigene13212\_JingChaoZuZhi2, Unigene13399\_JingChaoZuZhi2, Unigene13844\_JingChaoZuZhi2, Unigene14487\_JingChaoZuZhi2, Unigene14996\_JingChaoZuZhi2, Unigene15018\_JingChaoZuZhi2, Unigene15046\_JingChaoZuZhi2, Unigene15097\_JingChaoZuZhi2, Unigene15198\_JingChaoZuZhi2, Unigene15547\_JingChaoZuZhi2, Unigene16069\_JingChaoZuZhi2, Unigene16081\_JingChaoZuZhi2, Unigene16864\_JingChaoZuZhi2, Unigene17052\_JingChaoZuZhi2, Unigene17125\_JingChaoZuZhi2, Unigene17430\_JingChaoZuZhi2, Unigene17972\_JingChaoZuZhi2, Unigene1900\_JingChaoZuZhi2, Unigene19801\_JingChaoZuZhi2, Unigene19811\_JingChaoZuZhi2, Unigene20112\_JingChaoZuZhi2, Unigene20327\_JingChaoZuZhi2, Unigene20676\_JingChaoZuZhi2, Unigene20701\_JingChaoZuZhi2, Unigene21367\_JingChaoZuZhi2, Unigene21393\_JingChaoZuZhi2, Unigene21480\_JingChaoZuZhi2, Unigene22227\_JingChaoZuZhi2, Unigene2321\_JingChaoZuZhi2, Unigene23389\_JingChaoZuZhi2, Unigene2369\_JingChaoZuZhi2, Unigene29776\_JingChaoZuZhi2, Unigene30393\_JingChaoZuZhi2, Unigene30574\_JingChaoZuZhi2, Unigene3476\_JingChaoZuZhi2, Unigene4190\_JingChaoZuZhi2, Unigene43256\_JingChaoZuZhi2, Unigene4498\_JingChaoZuZhi2, Unigene45733\_JingChaoZuZhi2, Unigene46524\_JingChaoZuZhi2, Unigene4718\_JingChaoZuZhi2, Unigene49350\_JingChaoZuZhi2, Unigene52097\_JingChaoZuZhi2, Unigene552\_JingChaoZuZhi2, Unigene56572\_JingChaoZuZhi2, Unigene62056\_JingChaoZuZhi2, Unigene62948\_JingChaoZuZhi2, Unigene65415\_JingChaoZuZhi2, Unigene65838\_JingChaoZuZhi2, Unigene65915\_JingChaoZuZhi2, Unigene67664\_JingChaoZuZhi2, Unigene6846\_JingChaoZuZhi2, Unigene684\_JingChaoZuZhi2, Unigene69185\_JingChaoZuZhi2, Unigene69404\_JingChaoZuZhi2, Unigene71741\_JingChaoZuZhi2, Unigene72032\_JingChaoZuZhi2, Unigene72734\_JingChaoZuZhi2, Unigene73547\_JingChaoZuZhi2, Unigene73857\_JingChaoZuZhi2, Unigene73946\_JingChaoZuZhi2, Unigene74244\_JingChaoZuZhi2, Unigene74650\_JingChaoZuZhi2, Unigene76052\_JingChaoZuZhi2, Unigene77212\_JingChaoZuZhi2, Unigene77756\_JingChaoZuZhi2, Unigene78771\_JingChaoZuZhi2, Unigene79003\_JingChaoZuZhi2, Unigene79622\_JingChaoZuZhi2, Unigene8020\_JingChaoZuZhi2, Unigene80820\_JingChaoZuZhi2, Unigene81321\_JingChaoZuZhi2, Unigene8159\_JingChaoZuZhi2, Unigene83418\_JingChaoZuZhi2, Unigene84698\_JingChaoZuZhi2, Unigene87153\_JingChaoZuZhi2, Unigene87178\_JingChaoZuZhi2, Unigene87874\_JingChaoZuZhi2, Unigene88310\_JingChaoZuZhi2, Unigene89214\_JingChaoZuZhi2, Unigene89466\_JingChaoZuZhi2, Unigene89887\_JingChaoZuZhi2, Unigene90605\_JingChaoZuZhi2, Unigene91543\_JingChaoZuZhi2, Unigene91997\_JingChaoZuZhi2, Unigene92318\_JingChaoZuZhi2, Unigene92799\_JingChaoZuZhi2, Unigene92958\_JingChaoZuZhi2, Unigene93355\_JingChaoZuZhi2, Unigene93579\_JingChaoZuZhi2, Unigene93786\_JingChaoZuZhi2, Unigene94628\_JingChaoZuZhi2, Unigene94707\_JingChaoZuZhi2, Unigene94833\_JingChaoZuZhi2, Unigene95175\_JingChaoZuZhi2, Unigene95470\_JingChaoZuZhi2, Unigene95517\_JingChaoZuZhi2, Unigene95936\_JingChaoZuZhi2, Unigene95937\_JingChaoZuZhi2, Unigene96113\_JingChaoZuZhi2 |
| 96 | Starch and sucrose metabolism | Unigene10451\_JingChaoZuZhi2, Unigene1045\_JingChaoZuZhi2, Unigene12118\_JingChaoZuZhi2, Unigene12560\_JingChaoZuZhi2, Unigene12892\_JingChaoZuZhi2, Unigene13176\_JingChaoZuZhi2, Unigene13280\_JingChaoZuZhi2, Unigene13837\_JingChaoZuZhi2, Unigene15256\_JingChaoZuZhi2, Unigene15770\_JingChaoZuZhi2, Unigene16281\_JingChaoZuZhi2, Unigene16460\_JingChaoZuZhi2, Unigene16762\_JingChaoZuZhi2, Unigene17235\_JingChaoZuZhi2, Unigene18270\_JingChaoZuZhi2, Unigene18991\_JingChaoZuZhi2, Unigene19140\_JingChaoZuZhi2, Unigene19709\_JingChaoZuZhi2, Unigene20579\_JingChaoZuZhi2, Unigene20950\_JingChaoZuZhi2, Unigene21122\_JingChaoZuZhi2, Unigene21383\_JingChaoZuZhi2, Unigene21490\_JingChaoZuZhi2, Unigene22366\_JingChaoZuZhi2, Unigene22392\_JingChaoZuZhi2, Unigene23882\_JingChaoZuZhi2, Unigene28256\_JingChaoZuZhi2, Unigene28801\_JingChaoZuZhi2, Unigene32746\_JingChaoZuZhi2, Unigene34300\_JingChaoZuZhi2, Unigene35848\_JingChaoZuZhi2, Unigene36714\_JingChaoZuZhi2, Unigene42637\_JingChaoZuZhi2, Unigene42809\_JingChaoZuZhi2, Unigene43293\_JingChaoZuZhi2, Unigene4377\_JingChaoZuZhi2, Unigene45887\_JingChaoZuZhi2, Unigene4673\_JingChaoZuZhi2, Unigene46843\_JingChaoZuZhi2, Unigene48948\_JingChaoZuZhi2, Unigene52057\_JingChaoZuZhi2, Unigene53809\_JingChaoZuZhi2, Unigene5423\_JingChaoZuZhi2, Unigene56237\_JingChaoZuZhi2, Unigene57061\_JingChaoZuZhi2, Unigene57130\_JingChaoZuZhi2, Unigene5957\_JingChaoZuZhi2, Unigene64434\_JingChaoZuZhi2, Unigene64643\_JingChaoZuZhi2, Unigene66850\_JingChaoZuZhi2, Unigene68356\_JingChaoZuZhi2, Unigene68502\_JingChaoZuZhi2, Unigene70140\_JingChaoZuZhi2, Unigene70806\_JingChaoZuZhi2, Unigene71152\_JingChaoZuZhi2, Unigene71196\_JingChaoZuZhi2, Unigene71976\_JingChaoZuZhi2, Unigene73451\_JingChaoZuZhi2, Unigene73632\_JingChaoZuZhi2, Unigene73757\_JingChaoZuZhi2, Unigene74263\_JingChaoZuZhi2, Unigene74590\_JingChaoZuZhi2, Unigene75050\_JingChaoZuZhi2, Unigene75543\_JingChaoZuZhi2, Unigene76128\_JingChaoZuZhi2, Unigene76385\_JingChaoZuZhi2, Unigene77041\_JingChaoZuZhi2, Unigene77192\_JingChaoZuZhi2, Unigene77374\_JingChaoZuZhi2, Unigene79198\_JingChaoZuZhi2, Unigene79539\_JingChaoZuZhi2, Unigene79613\_JingChaoZuZhi2, Unigene79906\_JingChaoZuZhi2, Unigene80249\_JingChaoZuZhi2, Unigene80285\_JingChaoZuZhi2, Unigene814\_JingChaoZuZhi2, Unigene81511\_JingChaoZuZhi2, Unigene82270\_JingChaoZuZhi2, Unigene82818\_JingChaoZuZhi2, Unigene83318\_JingChaoZuZhi2, Unigene83440\_JingChaoZuZhi2, Unigene84154\_JingChaoZuZhi2, Unigene84385\_JingChaoZuZhi2, Unigene85769\_JingChaoZuZhi2, Unigene86120\_JingChaoZuZhi2, Unigene86295\_JingChaoZuZhi2, Unigene86332\_JingChaoZuZhi2, Unigene87046\_JingChaoZuZhi2, Unigene88238\_JingChaoZuZhi2, Unigene88876\_JingChaoZuZhi2, Unigene89026\_JingChaoZuZhi2, Unigene89279\_JingChaoZuZhi2, Unigene89766\_JingChaoZuZhi2, Unigene89833\_JingChaoZuZhi2, Unigene90122\_JingChaoZuZhi2, Unigene90683\_JingChaoZuZhi2, Unigene91430\_JingChaoZuZhi2, Unigene91720\_JingChaoZuZhi2, Unigene91969\_JingChaoZuZhi2, Unigene92418\_JingChaoZuZhi2, Unigene93877\_JingChaoZuZhi2, Unigene95057\_JingChaoZuZhi2, Unigene95296\_JingChaoZuZhi2, Unigene95998\_JingChaoZuZhi2, Unigene96263\_JingChaoZuZhi2 |
| 97 | Epithelial cell signaling in Helicobacter pylori infection | Unigene10053\_JingChaoZuZhi2, Unigene10422\_JingChaoZuZhi2, Unigene11469\_JingChaoZuZhi2, Unigene11730\_JingChaoZuZhi2, Unigene11951\_JingChaoZuZhi2, Unigene12813\_JingChaoZuZhi2, Unigene13291\_JingChaoZuZhi2, Unigene14104\_JingChaoZuZhi2, Unigene14729\_JingChaoZuZhi2, Unigene14738\_JingChaoZuZhi2, Unigene14878\_JingChaoZuZhi2, Unigene15095\_JingChaoZuZhi2, Unigene15172\_JingChaoZuZhi2, Unigene16938\_JingChaoZuZhi2, Unigene17829\_JingChaoZuZhi2, Unigene18464\_JingChaoZuZhi2, Unigene18715\_JingChaoZuZhi2, Unigene18868\_JingChaoZuZhi2, Unigene19021\_JingChaoZuZhi2, Unigene20120\_JingChaoZuZhi2, Unigene20157\_JingChaoZuZhi2, Unigene2092\_JingChaoZuZhi2, Unigene20991\_JingChaoZuZhi2, Unigene21295\_JingChaoZuZhi2, Unigene21605\_JingChaoZuZhi2, Unigene21691\_JingChaoZuZhi2, Unigene21697\_JingChaoZuZhi2, Unigene21952\_JingChaoZuZhi2, Unigene21993\_JingChaoZuZhi2, Unigene22174\_JingChaoZuZhi2, Unigene22466\_JingChaoZuZhi2, Unigene22482\_JingChaoZuZhi2, Unigene22804\_JingChaoZuZhi2, Unigene22914\_JingChaoZuZhi2, Unigene25228\_JingChaoZuZhi2, Unigene2790\_JingChaoZuZhi2, Unigene33026\_JingChaoZuZhi2, Unigene3560\_JingChaoZuZhi2, Unigene38924\_JingChaoZuZhi2, Unigene4223\_JingChaoZuZhi2, Unigene4294\_JingChaoZuZhi2, Unigene49798\_JingChaoZuZhi2, Unigene53958\_JingChaoZuZhi2, Unigene54320\_JingChaoZuZhi2, Unigene54470\_JingChaoZuZhi2, Unigene55368\_JingChaoZuZhi2, Unigene5638\_JingChaoZuZhi2, Unigene56557\_JingChaoZuZhi2, Unigene62656\_JingChaoZuZhi2, Unigene62786\_JingChaoZuZhi2, Unigene63391\_JingChaoZuZhi2, Unigene6459\_JingChaoZuZhi2, Unigene65081\_JingChaoZuZhi2, Unigene66535\_JingChaoZuZhi2, Unigene6676\_JingChaoZuZhi2, Unigene67655\_JingChaoZuZhi2, Unigene69786\_JingChaoZuZhi2, Unigene70308\_JingChaoZuZhi2, Unigene72521\_JingChaoZuZhi2, Unigene75781\_JingChaoZuZhi2, Unigene76400\_JingChaoZuZhi2, Unigene76567\_JingChaoZuZhi2, Unigene77570\_JingChaoZuZhi2, Unigene78518\_JingChaoZuZhi2, Unigene78574\_JingChaoZuZhi2, Unigene79307\_JingChaoZuZhi2, Unigene7939\_JingChaoZuZhi2, Unigene80204\_JingChaoZuZhi2, Unigene83846\_JingChaoZuZhi2, Unigene84070\_JingChaoZuZhi2, Unigene84689\_JingChaoZuZhi2, Unigene84862\_JingChaoZuZhi2, Unigene85789\_JingChaoZuZhi2, Unigene87347\_JingChaoZuZhi2, Unigene87851\_JingChaoZuZhi2, Unigene88262\_JingChaoZuZhi2, Unigene88774\_JingChaoZuZhi2, Unigene89128\_JingChaoZuZhi2, Unigene89595\_JingChaoZuZhi2, Unigene89741\_JingChaoZuZhi2, Unigene90832\_JingChaoZuZhi2, Unigene91179\_JingChaoZuZhi2, Unigene91379\_JingChaoZuZhi2, Unigene91490\_JingChaoZuZhi2, Unigene91717\_JingChaoZuZhi2, Unigene91944\_JingChaoZuZhi2, Unigene92452\_JingChaoZuZhi2, Unigene92962\_JingChaoZuZhi2, Unigene93270\_JingChaoZuZhi2, Unigene93782\_JingChaoZuZhi2, Unigene93785\_JingChaoZuZhi2, Unigene93793\_JingChaoZuZhi2, Unigene94552\_JingChaoZuZhi2, Unigene94619\_JingChaoZuZhi2, Unigene9461\_JingChaoZuZhi2, Unigene94775\_JingChaoZuZhi2, Unigene94903\_JingChaoZuZhi2, Unigene95331\_JingChaoZuZhi2, Unigene95789\_JingChaoZuZhi2, Unigene95875\_JingChaoZuZhi2, Unigene96002\_JingChaoZuZhi2, Unigene96023\_JingChaoZuZhi2, Unigene96229\_JingChaoZuZhi2 |
| 98 | DNA replication | Unigene1037\_JingChaoZuZhi2, Unigene10802\_JingChaoZuZhi2, Unigene11043\_JingChaoZuZhi2, Unigene12617\_JingChaoZuZhi2, Unigene12754\_JingChaoZuZhi2, Unigene13861\_JingChaoZuZhi2, Unigene14051\_JingChaoZuZhi2, Unigene14592\_JingChaoZuZhi2, Unigene15128\_JingChaoZuZhi2, Unigene1520\_JingChaoZuZhi2, Unigene16653\_JingChaoZuZhi2, Unigene1679\_JingChaoZuZhi2, Unigene1695\_JingChaoZuZhi2, Unigene170\_JingChaoZuZhi2, Unigene1738\_JingChaoZuZhi2, Unigene17861\_JingChaoZuZhi2, Unigene1833\_JingChaoZuZhi2, Unigene18390\_JingChaoZuZhi2, Unigene18862\_JingChaoZuZhi2, Unigene1888\_JingChaoZuZhi2, Unigene1902\_JingChaoZuZhi2, Unigene20261\_JingChaoZuZhi2, Unigene20303\_JingChaoZuZhi2, Unigene20612\_JingChaoZuZhi2, Unigene20751\_JingChaoZuZhi2, Unigene21282\_JingChaoZuZhi2, Unigene21305\_JingChaoZuZhi2, Unigene21673\_JingChaoZuZhi2, Unigene21781\_JingChaoZuZhi2, Unigene22022\_JingChaoZuZhi2, Unigene2210\_JingChaoZuZhi2, Unigene22259\_JingChaoZuZhi2, Unigene22304\_JingChaoZuZhi2, Unigene22578\_JingChaoZuZhi2, Unigene2354\_JingChaoZuZhi2, Unigene29918\_JingChaoZuZhi2, Unigene30160\_JingChaoZuZhi2, Unigene32677\_JingChaoZuZhi2, Unigene3290\_JingChaoZuZhi2, Unigene35605\_JingChaoZuZhi2, Unigene35687\_JingChaoZuZhi2, Unigene3974\_JingChaoZuZhi2, Unigene40239\_JingChaoZuZhi2, Unigene41885\_JingChaoZuZhi2, Unigene460\_JingChaoZuZhi2, Unigene4900\_JingChaoZuZhi2, Unigene53510\_JingChaoZuZhi2, Unigene5457\_JingChaoZuZhi2, Unigene57726\_JingChaoZuZhi2, Unigene57931\_JingChaoZuZhi2, Unigene5882\_JingChaoZuZhi2, Unigene5948\_JingChaoZuZhi2, Unigene6073\_JingChaoZuZhi2, Unigene6233\_JingChaoZuZhi2, Unigene63198\_JingChaoZuZhi2, Unigene63504\_JingChaoZuZhi2, Unigene64315\_JingChaoZuZhi2, Unigene64812\_JingChaoZuZhi2, Unigene66445\_JingChaoZuZhi2, Unigene6685\_JingChaoZuZhi2, Unigene67318\_JingChaoZuZhi2, Unigene6944\_JingChaoZuZhi2, Unigene69922\_JingChaoZuZhi2, Unigene70146\_JingChaoZuZhi2, Unigene70244\_JingChaoZuZhi2, Unigene71482\_JingChaoZuZhi2, Unigene72474\_JingChaoZuZhi2, Unigene72663\_JingChaoZuZhi2, Unigene73796\_JingChaoZuZhi2, Unigene74846\_JingChaoZuZhi2, Unigene76908\_JingChaoZuZhi2, Unigene77113\_JingChaoZuZhi2, Unigene78192\_JingChaoZuZhi2, Unigene78813\_JingChaoZuZhi2, Unigene79036\_JingChaoZuZhi2, Unigene79100\_JingChaoZuZhi2, Unigene80083\_JingChaoZuZhi2, Unigene84007\_JingChaoZuZhi2, Unigene84751\_JingChaoZuZhi2, Unigene85304\_JingChaoZuZhi2, Unigene85366\_JingChaoZuZhi2, Unigene86060\_JingChaoZuZhi2, Unigene86708\_JingChaoZuZhi2, Unigene87422\_JingChaoZuZhi2, Unigene87989\_JingChaoZuZhi2, Unigene88455\_JingChaoZuZhi2, Unigene88770\_JingChaoZuZhi2, Unigene89645\_JingChaoZuZhi2, Unigene90104\_JingChaoZuZhi2, Unigene91096\_JingChaoZuZhi2, Unigene92389\_JingChaoZuZhi2, Unigene92803\_JingChaoZuZhi2, Unigene94583\_JingChaoZuZhi2, Unigene94585\_JingChaoZuZhi2, Unigene95055\_JingChaoZuZhi2, Unigene95153\_JingChaoZuZhi2, Unigene95179\_JingChaoZuZhi2, Unigene95309\_JingChaoZuZhi2, Unigene95352\_JingChaoZuZhi2, Unigene95381\_JingChaoZuZhi2, Unigene95389\_JingChaoZuZhi2, Unigene95819\_JingChaoZuZhi2, Unigene96157\_JingChaoZuZhi2 |
| 99 | Base excision repair | Unigene10200\_JingChaoZuZhi2, Unigene10802\_JingChaoZuZhi2, Unigene11592\_JingChaoZuZhi2, Unigene11789\_JingChaoZuZhi2, Unigene12617\_JingChaoZuZhi2, Unigene13756\_JingChaoZuZhi2, Unigene13861\_JingChaoZuZhi2, Unigene14592\_JingChaoZuZhi2, Unigene1520\_JingChaoZuZhi2, Unigene16653\_JingChaoZuZhi2, Unigene1695\_JingChaoZuZhi2, Unigene17353\_JingChaoZuZhi2, Unigene1738\_JingChaoZuZhi2, Unigene17962\_JingChaoZuZhi2, Unigene18211\_JingChaoZuZhi2, Unigene1833\_JingChaoZuZhi2, Unigene18583\_JingChaoZuZhi2, Unigene18662\_JingChaoZuZhi2, Unigene18862\_JingChaoZuZhi2, Unigene1888\_JingChaoZuZhi2, Unigene1902\_JingChaoZuZhi2, Unigene20426\_JingChaoZuZhi2, Unigene20612\_JingChaoZuZhi2, Unigene21282\_JingChaoZuZhi2, Unigene21305\_JingChaoZuZhi2, Unigene21373\_JingChaoZuZhi2, Unigene21440\_JingChaoZuZhi2, Unigene21481\_JingChaoZuZhi2, Unigene21745\_JingChaoZuZhi2, Unigene21776\_JingChaoZuZhi2, Unigene21781\_JingChaoZuZhi2, Unigene22259\_JingChaoZuZhi2, Unigene22578\_JingChaoZuZhi2, Unigene2354\_JingChaoZuZhi2, Unigene27343\_JingChaoZuZhi2, Unigene32455\_JingChaoZuZhi2, Unigene35605\_JingChaoZuZhi2, Unigene35687\_JingChaoZuZhi2, Unigene3974\_JingChaoZuZhi2, Unigene41823\_JingChaoZuZhi2, Unigene4208\_JingChaoZuZhi2, Unigene43937\_JingChaoZuZhi2, Unigene44373\_JingChaoZuZhi2, Unigene45593\_JingChaoZuZhi2, Unigene45900\_JingChaoZuZhi2, Unigene460\_JingChaoZuZhi2, Unigene48432\_JingChaoZuZhi2, Unigene55839\_JingChaoZuZhi2, Unigene57726\_JingChaoZuZhi2, Unigene57931\_JingChaoZuZhi2, Unigene5882\_JingChaoZuZhi2, Unigene6073\_JingChaoZuZhi2, Unigene6233\_JingChaoZuZhi2, Unigene62423\_JingChaoZuZhi2, Unigene62822\_JingChaoZuZhi2, Unigene63110\_JingChaoZuZhi2, Unigene64315\_JingChaoZuZhi2, Unigene6944\_JingChaoZuZhi2, Unigene71267\_JingChaoZuZhi2, Unigene71482\_JingChaoZuZhi2, Unigene73233\_JingChaoZuZhi2, Unigene73741\_JingChaoZuZhi2, Unigene75150\_JingChaoZuZhi2, Unigene76053\_JingChaoZuZhi2, Unigene77601\_JingChaoZuZhi2, Unigene78813\_JingChaoZuZhi2, Unigene79036\_JingChaoZuZhi2, Unigene79229\_JingChaoZuZhi2, Unigene79600\_JingChaoZuZhi2, Unigene79990\_JingChaoZuZhi2, Unigene81924\_JingChaoZuZhi2, Unigene85796\_JingChaoZuZhi2, Unigene8619\_JingChaoZuZhi2, Unigene86282\_JingChaoZuZhi2, Unigene86708\_JingChaoZuZhi2, Unigene87249\_JingChaoZuZhi2, Unigene87385\_JingChaoZuZhi2, Unigene87698\_JingChaoZuZhi2, Unigene88507\_JingChaoZuZhi2, Unigene89645\_JingChaoZuZhi2, Unigene90104\_JingChaoZuZhi2, Unigene91898\_JingChaoZuZhi2, Unigene92645\_JingChaoZuZhi2, Unigene92662\_JingChaoZuZhi2, Unigene92803\_JingChaoZuZhi2, Unigene93413\_JingChaoZuZhi2, Unigene93529\_JingChaoZuZhi2, Unigene94383\_JingChaoZuZhi2, Unigene94915\_JingChaoZuZhi2, Unigene95005\_JingChaoZuZhi2, Unigene95055\_JingChaoZuZhi2, Unigene95078\_JingChaoZuZhi2, Unigene95153\_JingChaoZuZhi2, Unigene95309\_JingChaoZuZhi2, Unigene95352\_JingChaoZuZhi2, Unigene95389\_JingChaoZuZhi2, Unigene9546\_JingChaoZuZhi2, Unigene95525\_JingChaoZuZhi2, Unigene95819\_JingChaoZuZhi2, Unigene95841\_JingChaoZuZhi2, Unigene95924\_JingChaoZuZhi2 |
| 100 | Drug metabolism - other enzymes | Unigene10268\_JingChaoZuZhi2, Unigene11471\_JingChaoZuZhi2, Unigene12675\_JingChaoZuZhi2, Unigene12758\_JingChaoZuZhi2, Unigene12966\_JingChaoZuZhi2, Unigene13068\_JingChaoZuZhi2, Unigene13144\_JingChaoZuZhi2, Unigene13160\_JingChaoZuZhi2, Unigene14448\_JingChaoZuZhi2, Unigene18000\_JingChaoZuZhi2, Unigene18188\_JingChaoZuZhi2, Unigene18351\_JingChaoZuZhi2, Unigene18461\_JingChaoZuZhi2, Unigene18666\_JingChaoZuZhi2, Unigene18991\_JingChaoZuZhi2, Unigene19140\_JingChaoZuZhi2, Unigene19643\_JingChaoZuZhi2, Unigene20264\_JingChaoZuZhi2, Unigene20504\_JingChaoZuZhi2, Unigene20631\_JingChaoZuZhi2, Unigene21510\_JingChaoZuZhi2, Unigene21767\_JingChaoZuZhi2, Unigene22109\_JingChaoZuZhi2, Unigene22198\_JingChaoZuZhi2, Unigene22416\_JingChaoZuZhi2, Unigene22516\_JingChaoZuZhi2, Unigene23172\_JingChaoZuZhi2, Unigene2553\_JingChaoZuZhi2, Unigene27011\_JingChaoZuZhi2, Unigene32848\_JingChaoZuZhi2, Unigene36386\_JingChaoZuZhi2, Unigene36522\_JingChaoZuZhi2, Unigene37317\_JingChaoZuZhi2, Unigene42890\_JingChaoZuZhi2, Unigene43926\_JingChaoZuZhi2, Unigene45706\_JingChaoZuZhi2, Unigene47722\_JingChaoZuZhi2, Unigene48142\_JingChaoZuZhi2, Unigene48343\_JingChaoZuZhi2, Unigene50305\_JingChaoZuZhi2, Unigene50422\_JingChaoZuZhi2, Unigene5182\_JingChaoZuZhi2, Unigene52712\_JingChaoZuZhi2, Unigene53362\_JingChaoZuZhi2, Unigene53447\_JingChaoZuZhi2, Unigene53634\_JingChaoZuZhi2, Unigene5449\_JingChaoZuZhi2, Unigene54768\_JingChaoZuZhi2, Unigene56370\_JingChaoZuZhi2, Unigene57061\_JingChaoZuZhi2, Unigene57130\_JingChaoZuZhi2, Unigene59427\_JingChaoZuZhi2, Unigene59502\_JingChaoZuZhi2, Unigene5957\_JingChaoZuZhi2, Unigene59836\_JingChaoZuZhi2, Unigene63375\_JingChaoZuZhi2, Unigene63485\_JingChaoZuZhi2, Unigene662\_JingChaoZuZhi2, Unigene69209\_JingChaoZuZhi2, Unigene70620\_JingChaoZuZhi2, Unigene71235\_JingChaoZuZhi2, Unigene71976\_JingChaoZuZhi2, Unigene72767\_JingChaoZuZhi2, Unigene75469\_JingChaoZuZhi2, Unigene77994\_JingChaoZuZhi2, Unigene78226\_JingChaoZuZhi2, Unigene78351\_JingChaoZuZhi2, Unigene79196\_JingChaoZuZhi2, Unigene79198\_JingChaoZuZhi2, Unigene79590\_JingChaoZuZhi2, Unigene80285\_JingChaoZuZhi2, Unigene80305\_JingChaoZuZhi2, Unigene81615\_JingChaoZuZhi2, Unigene81630\_JingChaoZuZhi2, Unigene81878\_JingChaoZuZhi2, Unigene82818\_JingChaoZuZhi2, Unigene83273\_JingChaoZuZhi2, Unigene84385\_JingChaoZuZhi2, Unigene84831\_JingChaoZuZhi2, Unigene85539\_JingChaoZuZhi2, Unigene85640\_JingChaoZuZhi2, Unigene87018\_JingChaoZuZhi2, Unigene87046\_JingChaoZuZhi2, Unigene87491\_JingChaoZuZhi2, Unigene88383\_JingChaoZuZhi2, Unigene90190\_JingChaoZuZhi2, Unigene90793\_JingChaoZuZhi2, Unigene90999\_JingChaoZuZhi2, Unigene91087\_JingChaoZuZhi2, Unigene91541\_JingChaoZuZhi2, Unigene91914\_JingChaoZuZhi2, Unigene92088\_JingChaoZuZhi2, Unigene92281\_JingChaoZuZhi2, Unigene93255\_JingChaoZuZhi2, Unigene93589\_JingChaoZuZhi2, Unigene93666\_JingChaoZuZhi2, Unigene94536\_JingChaoZuZhi2, Unigene95132\_JingChaoZuZhi2, Unigene95155\_JingChaoZuZhi2, Unigene95618\_JingChaoZuZhi2 |
| 101 | Glutathione metabolism | Unigene10072\_JingChaoZuZhi2, Unigene10660\_JingChaoZuZhi2, Unigene11803\_JingChaoZuZhi2, Unigene12292\_JingChaoZuZhi2, Unigene13337\_JingChaoZuZhi2, Unigene14339\_JingChaoZuZhi2, Unigene15893\_JingChaoZuZhi2, Unigene16073\_JingChaoZuZhi2, Unigene16524\_JingChaoZuZhi2, Unigene16549\_JingChaoZuZhi2, Unigene16788\_JingChaoZuZhi2, Unigene17082\_JingChaoZuZhi2, Unigene18123\_JingChaoZuZhi2, Unigene18214\_JingChaoZuZhi2, Unigene18335\_JingChaoZuZhi2, Unigene20314\_JingChaoZuZhi2, Unigene21281\_JingChaoZuZhi2, Unigene22123\_JingChaoZuZhi2, Unigene22360\_JingChaoZuZhi2, Unigene22904\_JingChaoZuZhi2, Unigene2666\_JingChaoZuZhi2, Unigene26903\_JingChaoZuZhi2, Unigene2750\_JingChaoZuZhi2, Unigene28379\_JingChaoZuZhi2, Unigene28844\_JingChaoZuZhi2, Unigene2916\_JingChaoZuZhi2, Unigene3061\_JingChaoZuZhi2, Unigene39201\_JingChaoZuZhi2, Unigene3936\_JingChaoZuZhi2, Unigene42718\_JingChaoZuZhi2, Unigene44595\_JingChaoZuZhi2, Unigene4542\_JingChaoZuZhi2, Unigene45815\_JingChaoZuZhi2, Unigene46342\_JingChaoZuZhi2, Unigene46463\_JingChaoZuZhi2, Unigene4767\_JingChaoZuZhi2, Unigene49647\_JingChaoZuZhi2, Unigene52898\_JingChaoZuZhi2, Unigene5345\_JingChaoZuZhi2, Unigene55482\_JingChaoZuZhi2, Unigene55810\_JingChaoZuZhi2, Unigene5714\_JingChaoZuZhi2, Unigene57596\_JingChaoZuZhi2, Unigene5875\_JingChaoZuZhi2, Unigene60326\_JingChaoZuZhi2, Unigene60905\_JingChaoZuZhi2, Unigene6096\_JingChaoZuZhi2, Unigene62191\_JingChaoZuZhi2, Unigene62568\_JingChaoZuZhi2, Unigene64846\_JingChaoZuZhi2, Unigene65006\_JingChaoZuZhi2, Unigene65960\_JingChaoZuZhi2, Unigene66403\_JingChaoZuZhi2, Unigene67767\_JingChaoZuZhi2, Unigene68509\_JingChaoZuZhi2, Unigene70921\_JingChaoZuZhi2, Unigene74736\_JingChaoZuZhi2, Unigene74771\_JingChaoZuZhi2, Unigene75912\_JingChaoZuZhi2, Unigene75948\_JingChaoZuZhi2, Unigene76514\_JingChaoZuZhi2, Unigene76706\_JingChaoZuZhi2, Unigene76826\_JingChaoZuZhi2, Unigene76920\_JingChaoZuZhi2, Unigene77016\_JingChaoZuZhi2, Unigene78966\_JingChaoZuZhi2, Unigene81236\_JingChaoZuZhi2, Unigene81935\_JingChaoZuZhi2, Unigene81947\_JingChaoZuZhi2, Unigene81959\_JingChaoZuZhi2, Unigene82234\_JingChaoZuZhi2, Unigene8278\_JingChaoZuZhi2, Unigene84420\_JingChaoZuZhi2, Unigene84531\_JingChaoZuZhi2, Unigene85035\_JingChaoZuZhi2, Unigene86047\_JingChaoZuZhi2, Unigene86806\_JingChaoZuZhi2, Unigene87416\_JingChaoZuZhi2, Unigene89115\_JingChaoZuZhi2, Unigene89233\_JingChaoZuZhi2, Unigene89289\_JingChaoZuZhi2, Unigene89347\_JingChaoZuZhi2, Unigene89725\_JingChaoZuZhi2, Unigene90393\_JingChaoZuZhi2, Unigene92983\_JingChaoZuZhi2, Unigene93067\_JingChaoZuZhi2, Unigene94607\_JingChaoZuZhi2, Unigene94614\_JingChaoZuZhi2, Unigene94803\_JingChaoZuZhi2, Unigene94894\_JingChaoZuZhi2, Unigene95016\_JingChaoZuZhi2, Unigene95563\_JingChaoZuZhi2, Unigene95656\_JingChaoZuZhi2, Unigene95737\_JingChaoZuZhi2, Unigene95901\_JingChaoZuZhi2, Unigene95953\_JingChaoZuZhi2, Unigene96266\_JingChaoZuZhi2, Unigene96284\_JingChaoZuZhi2, Unigene9631\_JingChaoZuZhi2 |
| 102 | Fc epsilon RI signaling pathway | Unigene10318\_JingChaoZuZhi2, Unigene11055\_JingChaoZuZhi2, Unigene11951\_JingChaoZuZhi2, Unigene11961\_JingChaoZuZhi2, Unigene12260\_JingChaoZuZhi2, Unigene12420\_JingChaoZuZhi2, Unigene12899\_JingChaoZuZhi2, Unigene13180\_JingChaoZuZhi2, Unigene13420\_JingChaoZuZhi2, Unigene13869\_JingChaoZuZhi2, Unigene14357\_JingChaoZuZhi2, Unigene14912\_JingChaoZuZhi2, Unigene15228\_JingChaoZuZhi2, Unigene15425\_JingChaoZuZhi2, Unigene15942\_JingChaoZuZhi2, Unigene17247\_JingChaoZuZhi2, Unigene17888\_JingChaoZuZhi2, Unigene18464\_JingChaoZuZhi2, Unigene18672\_JingChaoZuZhi2, Unigene18868\_JingChaoZuZhi2, Unigene18944\_JingChaoZuZhi2, Unigene19021\_JingChaoZuZhi2, Unigene19904\_JingChaoZuZhi2, Unigene20120\_JingChaoZuZhi2, Unigene21295\_JingChaoZuZhi2, Unigene21407\_JingChaoZuZhi2, Unigene21578\_JingChaoZuZhi2, Unigene21605\_JingChaoZuZhi2, Unigene21691\_JingChaoZuZhi2, Unigene22779\_JingChaoZuZhi2, Unigene2456\_JingChaoZuZhi2, Unigene26730\_JingChaoZuZhi2, Unigene2687\_JingChaoZuZhi2, Unigene2960\_JingChaoZuZhi2, Unigene3931\_JingChaoZuZhi2, Unigene4186\_JingChaoZuZhi2, Unigene42329\_JingChaoZuZhi2, Unigene4294\_JingChaoZuZhi2, Unigene43723\_JingChaoZuZhi2, Unigene45532\_JingChaoZuZhi2, Unigene4838\_JingChaoZuZhi2, Unigene48751\_JingChaoZuZhi2, Unigene5196\_JingChaoZuZhi2, Unigene52554\_JingChaoZuZhi2, Unigene57488\_JingChaoZuZhi2, Unigene5823\_JingChaoZuZhi2, Unigene6028\_JingChaoZuZhi2, Unigene6211\_JingChaoZuZhi2, Unigene64164\_JingChaoZuZhi2, Unigene6459\_JingChaoZuZhi2, Unigene64830\_JingChaoZuZhi2, Unigene6493\_JingChaoZuZhi2, Unigene67328\_JingChaoZuZhi2, Unigene67655\_JingChaoZuZhi2, Unigene67793\_JingChaoZuZhi2, Unigene68151\_JingChaoZuZhi2, Unigene70549\_JingChaoZuZhi2, Unigene70723\_JingChaoZuZhi2, Unigene72098\_JingChaoZuZhi2, Unigene74594\_JingChaoZuZhi2, Unigene76182\_JingChaoZuZhi2, Unigene7939\_JingChaoZuZhi2, Unigene79860\_JingChaoZuZhi2, Unigene80331\_JingChaoZuZhi2, Unigene8191\_JingChaoZuZhi2, Unigene81948\_JingChaoZuZhi2, Unigene82073\_JingChaoZuZhi2, Unigene85574\_JingChaoZuZhi2, Unigene86206\_JingChaoZuZhi2, Unigene8621\_JingChaoZuZhi2, Unigene86526\_JingChaoZuZhi2, Unigene86713\_JingChaoZuZhi2, Unigene86899\_JingChaoZuZhi2, Unigene87305\_JingChaoZuZhi2, Unigene87332\_JingChaoZuZhi2, Unigene876\_JingChaoZuZhi2, Unigene88068\_JingChaoZuZhi2, Unigene91179\_JingChaoZuZhi2, Unigene91612\_JingChaoZuZhi2, Unigene91958\_JingChaoZuZhi2, Unigene92112\_JingChaoZuZhi2, Unigene92372\_JingChaoZuZhi2, Unigene92737\_JingChaoZuZhi2, Unigene93793\_JingChaoZuZhi2, Unigene93807\_JingChaoZuZhi2, Unigene9389\_JingChaoZuZhi2, Unigene94149\_JingChaoZuZhi2, Unigene94456\_JingChaoZuZhi2, Unigene94619\_JingChaoZuZhi2, Unigene9461\_JingChaoZuZhi2, Unigene94685\_JingChaoZuZhi2, Unigene9488\_JingChaoZuZhi2, Unigene95456\_JingChaoZuZhi2, Unigene95677\_JingChaoZuZhi2, Unigene95875\_JingChaoZuZhi2, Unigene96294\_JingChaoZuZhi2, Unigene9902\_JingChaoZuZhi2, Unigene9\_JingChaoZuZhi2 |
| 103 | Glycerolipid metabolism | Unigene10268\_JingChaoZuZhi2, Unigene12083\_JingChaoZuZhi2, Unigene12639\_JingChaoZuZhi2, Unigene13538\_JingChaoZuZhi2, Unigene15363\_JingChaoZuZhi2, Unigene16594\_JingChaoZuZhi2, Unigene17028\_JingChaoZuZhi2, Unigene18308\_JingChaoZuZhi2, Unigene18358\_JingChaoZuZhi2, Unigene19186\_JingChaoZuZhi2, Unigene19539\_JingChaoZuZhi2, Unigene20071\_JingChaoZuZhi2, Unigene2058\_JingChaoZuZhi2, Unigene20631\_JingChaoZuZhi2, Unigene20760\_JingChaoZuZhi2, Unigene20802\_JingChaoZuZhi2, Unigene21049\_JingChaoZuZhi2, Unigene21176\_JingChaoZuZhi2, Unigene21718\_JingChaoZuZhi2, Unigene22211\_JingChaoZuZhi2, Unigene2235\_JingChaoZuZhi2, Unigene22498\_JingChaoZuZhi2, Unigene23842\_JingChaoZuZhi2, Unigene24146\_JingChaoZuZhi2, Unigene2474\_JingChaoZuZhi2, Unigene25108\_JingChaoZuZhi2, Unigene2514\_JingChaoZuZhi2, Unigene33875\_JingChaoZuZhi2, Unigene36790\_JingChaoZuZhi2, Unigene37689\_JingChaoZuZhi2, Unigene4160\_JingChaoZuZhi2, Unigene41739\_JingChaoZuZhi2, Unigene42056\_JingChaoZuZhi2, Unigene44246\_JingChaoZuZhi2, Unigene45669\_JingChaoZuZhi2, Unigene46661\_JingChaoZuZhi2, Unigene47032\_JingChaoZuZhi2, Unigene50937\_JingChaoZuZhi2, Unigene51678\_JingChaoZuZhi2, Unigene51741\_JingChaoZuZhi2, Unigene52889\_JingChaoZuZhi2, Unigene54783\_JingChaoZuZhi2, Unigene57970\_JingChaoZuZhi2, Unigene59735\_JingChaoZuZhi2, Unigene59979\_JingChaoZuZhi2, Unigene60117\_JingChaoZuZhi2, Unigene6117\_JingChaoZuZhi2, Unigene62892\_JingChaoZuZhi2, Unigene63649\_JingChaoZuZhi2, Unigene64489\_JingChaoZuZhi2, Unigene65222\_JingChaoZuZhi2, Unigene67613\_JingChaoZuZhi2, Unigene69249\_JingChaoZuZhi2, Unigene7242\_JingChaoZuZhi2, Unigene72476\_JingChaoZuZhi2, Unigene72974\_JingChaoZuZhi2, Unigene74643\_JingChaoZuZhi2, Unigene7493\_JingChaoZuZhi2, Unigene75535\_JingChaoZuZhi2, Unigene76480\_JingChaoZuZhi2, Unigene78885\_JingChaoZuZhi2, Unigene78992\_JingChaoZuZhi2, Unigene8011\_JingChaoZuZhi2, Unigene80141\_JingChaoZuZhi2, Unigene80881\_JingChaoZuZhi2, Unigene80895\_JingChaoZuZhi2, Unigene82129\_JingChaoZuZhi2, Unigene83218\_JingChaoZuZhi2, Unigene83570\_JingChaoZuZhi2, Unigene84021\_JingChaoZuZhi2, Unigene84063\_JingChaoZuZhi2, Unigene84851\_JingChaoZuZhi2, Unigene86034\_JingChaoZuZhi2, Unigene87044\_JingChaoZuZhi2, Unigene87893\_JingChaoZuZhi2, Unigene89232\_JingChaoZuZhi2, Unigene89783\_JingChaoZuZhi2, Unigene90127\_JingChaoZuZhi2, Unigene90131\_JingChaoZuZhi2, Unigene90190\_JingChaoZuZhi2, Unigene90268\_JingChaoZuZhi2, Unigene90531\_JingChaoZuZhi2, Unigene90616\_JingChaoZuZhi2, Unigene90914\_JingChaoZuZhi2, Unigene91700\_JingChaoZuZhi2, Unigene91798\_JingChaoZuZhi2, Unigene92263\_JingChaoZuZhi2, Unigene93334\_JingChaoZuZhi2, Unigene94001\_JingChaoZuZhi2, Unigene9415\_JingChaoZuZhi2, Unigene94274\_JingChaoZuZhi2, Unigene94837\_JingChaoZuZhi2, Unigene95394\_JingChaoZuZhi2, Unigene95616\_JingChaoZuZhi2, Unigene95818\_JingChaoZuZhi2, Unigene95959\_JingChaoZuZhi2, Unigene95985\_JingChaoZuZhi2, Unigene96031\_JingChaoZuZhi2 |
| 104 | Antigen processing and presentation | Unigene10380\_JingChaoZuZhi2, Unigene10747\_JingChaoZuZhi2, Unigene11592\_JingChaoZuZhi2, Unigene12633\_JingChaoZuZhi2, Unigene12787\_JingChaoZuZhi2, Unigene13115\_JingChaoZuZhi2, Unigene14250\_JingChaoZuZhi2, Unigene14409\_JingChaoZuZhi2, Unigene17905\_JingChaoZuZhi2, Unigene18577\_JingChaoZuZhi2, Unigene19131\_JingChaoZuZhi2, Unigene19575\_JingChaoZuZhi2, Unigene21103\_JingChaoZuZhi2, Unigene2131\_JingChaoZuZhi2, Unigene21552\_JingChaoZuZhi2, Unigene21868\_JingChaoZuZhi2, Unigene22686\_JingChaoZuZhi2, Unigene28427\_JingChaoZuZhi2, Unigene33604\_JingChaoZuZhi2, Unigene35990\_JingChaoZuZhi2, Unigene36617\_JingChaoZuZhi2, Unigene36751\_JingChaoZuZhi2, Unigene37667\_JingChaoZuZhi2, Unigene38133\_JingChaoZuZhi2, Unigene38741\_JingChaoZuZhi2, Unigene39259\_JingChaoZuZhi2, Unigene39457\_JingChaoZuZhi2, Unigene44158\_JingChaoZuZhi2, Unigene44224\_JingChaoZuZhi2, Unigene45948\_JingChaoZuZhi2, Unigene47450\_JingChaoZuZhi2, Unigene48223\_JingChaoZuZhi2, Unigene50611\_JingChaoZuZhi2, Unigene51820\_JingChaoZuZhi2, Unigene52526\_JingChaoZuZhi2, Unigene53034\_JingChaoZuZhi2, Unigene54569\_JingChaoZuZhi2, Unigene55100\_JingChaoZuZhi2, Unigene56775\_JingChaoZuZhi2, Unigene58582\_JingChaoZuZhi2, Unigene58742\_JingChaoZuZhi2, Unigene61736\_JingChaoZuZhi2, Unigene62272\_JingChaoZuZhi2, Unigene63452\_JingChaoZuZhi2, Unigene64104\_JingChaoZuZhi2, Unigene64957\_JingChaoZuZhi2, Unigene67676\_JingChaoZuZhi2, Unigene67\_JingChaoZuZhi2, Unigene72036\_JingChaoZuZhi2, Unigene74269\_JingChaoZuZhi2, Unigene74963\_JingChaoZuZhi2, Unigene76131\_JingChaoZuZhi2, Unigene76392\_JingChaoZuZhi2, Unigene77209\_JingChaoZuZhi2, Unigene7850\_JingChaoZuZhi2, Unigene78904\_JingChaoZuZhi2, Unigene82058\_JingChaoZuZhi2, Unigene82367\_JingChaoZuZhi2, Unigene82742\_JingChaoZuZhi2, Unigene83523\_JingChaoZuZhi2, Unigene83524\_JingChaoZuZhi2, Unigene83933\_JingChaoZuZhi2, Unigene85038\_JingChaoZuZhi2, Unigene85639\_JingChaoZuZhi2, Unigene86501\_JingChaoZuZhi2, Unigene87261\_JingChaoZuZhi2, Unigene8853\_JingChaoZuZhi2, Unigene89774\_JingChaoZuZhi2, Unigene90312\_JingChaoZuZhi2, Unigene90347\_JingChaoZuZhi2, Unigene90645\_JingChaoZuZhi2, Unigene9095\_JingChaoZuZhi2, Unigene91303\_JingChaoZuZhi2, Unigene91427\_JingChaoZuZhi2, Unigene91548\_JingChaoZuZhi2, Unigene91628\_JingChaoZuZhi2, Unigene91944\_JingChaoZuZhi2, Unigene92508\_JingChaoZuZhi2, Unigene92949\_JingChaoZuZhi2, Unigene93099\_JingChaoZuZhi2, Unigene93154\_JingChaoZuZhi2, Unigene93433\_JingChaoZuZhi2, Unigene93536\_JingChaoZuZhi2, Unigene94362\_JingChaoZuZhi2, Unigene94512\_JingChaoZuZhi2, Unigene94524\_JingChaoZuZhi2, Unigene94533\_JingChaoZuZhi2, Unigene94721\_JingChaoZuZhi2, Unigene94762\_JingChaoZuZhi2, Unigene95185\_JingChaoZuZhi2, Unigene95395\_JingChaoZuZhi2, Unigene95427\_JingChaoZuZhi2, Unigene95897\_JingChaoZuZhi2, Unigene95984\_JingChaoZuZhi2, Unigene96029\_JingChaoZuZhi2, Unigene96032\_JingChaoZuZhi2, Unigene96180\_JingChaoZuZhi2, Unigene9676\_JingChaoZuZhi2 |
| 105 | Hematopoietic cell lineage | Unigene10320\_JingChaoZuZhi2, Unigene1111\_JingChaoZuZhi2, Unigene11209\_JingChaoZuZhi2, Unigene15780\_JingChaoZuZhi2, Unigene16073\_JingChaoZuZhi2, Unigene16121\_JingChaoZuZhi2, Unigene16549\_JingChaoZuZhi2, Unigene17128\_JingChaoZuZhi2, Unigene18921\_JingChaoZuZhi2, Unigene18966\_JingChaoZuZhi2, Unigene19404\_JingChaoZuZhi2, Unigene19562\_JingChaoZuZhi2, Unigene20159\_JingChaoZuZhi2, Unigene20990\_JingChaoZuZhi2, Unigene21281\_JingChaoZuZhi2, Unigene21759\_JingChaoZuZhi2, Unigene21996\_JingChaoZuZhi2, Unigene22546\_JingChaoZuZhi2, Unigene22700\_JingChaoZuZhi2, Unigene26903\_JingChaoZuZhi2, Unigene28889\_JingChaoZuZhi2, Unigene29312\_JingChaoZuZhi2, Unigene3061\_JingChaoZuZhi2, Unigene38238\_JingChaoZuZhi2, Unigene38617\_JingChaoZuZhi2, Unigene39201\_JingChaoZuZhi2, Unigene39829\_JingChaoZuZhi2, Unigene40551\_JingChaoZuZhi2, Unigene41077\_JingChaoZuZhi2, Unigene42346\_JingChaoZuZhi2, Unigene42610\_JingChaoZuZhi2, Unigene42718\_JingChaoZuZhi2, Unigene4470\_JingChaoZuZhi2, Unigene45216\_JingChaoZuZhi2, Unigene45331\_JingChaoZuZhi2, Unigene4542\_JingChaoZuZhi2, Unigene45782\_JingChaoZuZhi2, Unigene46740\_JingChaoZuZhi2, Unigene49253\_JingChaoZuZhi2, Unigene49977\_JingChaoZuZhi2, Unigene5069\_JingChaoZuZhi2, Unigene51012\_JingChaoZuZhi2, Unigene5240\_JingChaoZuZhi2, Unigene5306\_JingChaoZuZhi2, Unigene55482\_JingChaoZuZhi2, Unigene5714\_JingChaoZuZhi2, Unigene57538\_JingChaoZuZhi2, Unigene57596\_JingChaoZuZhi2, Unigene57799\_JingChaoZuZhi2, Unigene58354\_JingChaoZuZhi2, Unigene58450\_JingChaoZuZhi2, Unigene60905\_JingChaoZuZhi2, Unigene61033\_JingChaoZuZhi2, Unigene61189\_JingChaoZuZhi2, Unigene62191\_JingChaoZuZhi2, Unigene62824\_JingChaoZuZhi2, Unigene64309\_JingChaoZuZhi2, Unigene64846\_JingChaoZuZhi2, Unigene65960\_JingChaoZuZhi2, Unigene67589\_JingChaoZuZhi2, Unigene69427\_JingChaoZuZhi2, Unigene69445\_JingChaoZuZhi2, Unigene72500\_JingChaoZuZhi2, Unigene75009\_JingChaoZuZhi2, Unigene75710\_JingChaoZuZhi2, Unigene76375\_JingChaoZuZhi2, Unigene76514\_JingChaoZuZhi2, Unigene76604\_JingChaoZuZhi2, Unigene78715\_JingChaoZuZhi2, Unigene79577\_JingChaoZuZhi2, Unigene81483\_JingChaoZuZhi2, Unigene81935\_JingChaoZuZhi2, Unigene81947\_JingChaoZuZhi2, Unigene82234\_JingChaoZuZhi2, Unigene83777\_JingChaoZuZhi2, Unigene84054\_JingChaoZuZhi2, Unigene84257\_JingChaoZuZhi2, Unigene85503\_JingChaoZuZhi2, Unigene85529\_JingChaoZuZhi2, Unigene88050\_JingChaoZuZhi2, Unigene88581\_JingChaoZuZhi2, Unigene88925\_JingChaoZuZhi2, Unigene89329\_JingChaoZuZhi2, Unigene89492\_JingChaoZuZhi2, Unigene89762\_JingChaoZuZhi2, Unigene90596\_JingChaoZuZhi2, Unigene9104\_JingChaoZuZhi2, Unigene91668\_JingChaoZuZhi2, Unigene92104\_JingChaoZuZhi2, Unigene92135\_JingChaoZuZhi2, Unigene92942\_JingChaoZuZhi2, Unigene93022\_JingChaoZuZhi2, Unigene94540\_JingChaoZuZhi2, Unigene9520\_JingChaoZuZhi2, Unigene95571\_JingChaoZuZhi2, Unigene96284\_JingChaoZuZhi2 |
| 106 | Endometrial cancer | Unigene10422\_JingChaoZuZhi2, Unigene11251\_JingChaoZuZhi2, Unigene11961\_JingChaoZuZhi2, Unigene1227\_JingChaoZuZhi2, Unigene12420\_JingChaoZuZhi2, Unigene13176\_JingChaoZuZhi2, Unigene14285\_JingChaoZuZhi2, Unigene14912\_JingChaoZuZhi2, Unigene15029\_JingChaoZuZhi2, Unigene15228\_JingChaoZuZhi2, Unigene16229\_JingChaoZuZhi2, Unigene16820\_JingChaoZuZhi2, Unigene17247\_JingChaoZuZhi2, Unigene17800\_JingChaoZuZhi2, Unigene18095\_JingChaoZuZhi2, Unigene18672\_JingChaoZuZhi2, Unigene18817\_JingChaoZuZhi2, Unigene18932\_JingChaoZuZhi2, Unigene19465\_JingChaoZuZhi2, Unigene21513\_JingChaoZuZhi2, Unigene22522\_JingChaoZuZhi2, Unigene2456\_JingChaoZuZhi2, Unigene2790\_JingChaoZuZhi2, Unigene28737\_JingChaoZuZhi2, Unigene30787\_JingChaoZuZhi2, Unigene33683\_JingChaoZuZhi2, Unigene34463\_JingChaoZuZhi2, Unigene3665\_JingChaoZuZhi2, Unigene37495\_JingChaoZuZhi2, Unigene37739\_JingChaoZuZhi2, Unigene3931\_JingChaoZuZhi2, Unigene40020\_JingChaoZuZhi2, Unigene4186\_JingChaoZuZhi2, Unigene4377\_JingChaoZuZhi2, Unigene4838\_JingChaoZuZhi2, Unigene48751\_JingChaoZuZhi2, Unigene4886\_JingChaoZuZhi2, Unigene49798\_JingChaoZuZhi2, Unigene53421\_JingChaoZuZhi2, Unigene54470\_JingChaoZuZhi2, Unigene56709\_JingChaoZuZhi2, Unigene57488\_JingChaoZuZhi2, Unigene58806\_JingChaoZuZhi2, Unigene6028\_JingChaoZuZhi2, Unigene61911\_JingChaoZuZhi2, Unigene62786\_JingChaoZuZhi2, Unigene63449\_JingChaoZuZhi2, Unigene64227\_JingChaoZuZhi2, Unigene64678\_JingChaoZuZhi2, Unigene6493\_JingChaoZuZhi2, Unigene67793\_JingChaoZuZhi2, Unigene6785\_JingChaoZuZhi2, Unigene67983\_JingChaoZuZhi2, Unigene68056\_JingChaoZuZhi2, Unigene68283\_JingChaoZuZhi2, Unigene69517\_JingChaoZuZhi2, Unigene70549\_JingChaoZuZhi2, Unigene72521\_JingChaoZuZhi2, Unigene78390\_JingChaoZuZhi2, Unigene78574\_JingChaoZuZhi2, Unigene8191\_JingChaoZuZhi2, Unigene84930\_JingChaoZuZhi2, Unigene85574\_JingChaoZuZhi2, Unigene86206\_JingChaoZuZhi2, Unigene86278\_JingChaoZuZhi2, Unigene86456\_JingChaoZuZhi2, Unigene86526\_JingChaoZuZhi2, Unigene876\_JingChaoZuZhi2, Unigene8799\_JingChaoZuZhi2, Unigene88544\_JingChaoZuZhi2, Unigene88719\_JingChaoZuZhi2, Unigene88748\_JingChaoZuZhi2, Unigene89310\_JingChaoZuZhi2, Unigene89315\_JingChaoZuZhi2, Unigene91612\_JingChaoZuZhi2, Unigene91958\_JingChaoZuZhi2, Unigene92112\_JingChaoZuZhi2, Unigene92309\_JingChaoZuZhi2, Unigene92443\_JingChaoZuZhi2, Unigene93133\_JingChaoZuZhi2, Unigene93807\_JingChaoZuZhi2, Unigene94149\_JingChaoZuZhi2, Unigene94272\_JingChaoZuZhi2, Unigene94456\_JingChaoZuZhi2, Unigene94685\_JingChaoZuZhi2, Unigene94795\_JingChaoZuZhi2, Unigene95014\_JingChaoZuZhi2, Unigene95098\_JingChaoZuZhi2, Unigene95295\_JingChaoZuZhi2, Unigene95456\_JingChaoZuZhi2, Unigene95791\_JingChaoZuZhi2, Unigene96234\_JingChaoZuZhi2, Unigene96294\_JingChaoZuZhi2, Unigene9902\_JingChaoZuZhi2 |
| 107 | Tyrosine metabolism | Unigene10192\_JingChaoZuZhi2, Unigene11153\_JingChaoZuZhi2, Unigene11803\_JingChaoZuZhi2, Unigene12292\_JingChaoZuZhi2, Unigene12758\_JingChaoZuZhi2, Unigene13337\_JingChaoZuZhi2, Unigene14339\_JingChaoZuZhi2, Unigene15780\_JingChaoZuZhi2, Unigene16501\_JingChaoZuZhi2, Unigene18788\_JingChaoZuZhi2, Unigene18921\_JingChaoZuZhi2, Unigene19048\_JingChaoZuZhi2, Unigene19552\_JingChaoZuZhi2, Unigene20192\_JingChaoZuZhi2, Unigene21678\_JingChaoZuZhi2, Unigene21759\_JingChaoZuZhi2, Unigene22003\_JingChaoZuZhi2, Unigene2230\_JingChaoZuZhi2, Unigene22546\_JingChaoZuZhi2, Unigene28889\_JingChaoZuZhi2, Unigene36819\_JingChaoZuZhi2, Unigene38324\_JingChaoZuZhi2, Unigene3910\_JingChaoZuZhi2, Unigene3936\_JingChaoZuZhi2, Unigene39829\_JingChaoZuZhi2, Unigene42346\_JingChaoZuZhi2, Unigene42610\_JingChaoZuZhi2, Unigene46463\_JingChaoZuZhi2, Unigene48046\_JingChaoZuZhi2, Unigene50289\_JingChaoZuZhi2, Unigene51012\_JingChaoZuZhi2, Unigene5240\_JingChaoZuZhi2, Unigene5449\_JingChaoZuZhi2, Unigene55810\_JingChaoZuZhi2, Unigene58354\_JingChaoZuZhi2, Unigene5875\_JingChaoZuZhi2, Unigene58985\_JingChaoZuZhi2, Unigene60326\_JingChaoZuZhi2, Unigene61189\_JingChaoZuZhi2, Unigene61702\_JingChaoZuZhi2, Unigene62568\_JingChaoZuZhi2, Unigene64994\_JingChaoZuZhi2, Unigene66496\_JingChaoZuZhi2, Unigene67589\_JingChaoZuZhi2, Unigene69787\_JingChaoZuZhi2, Unigene70225\_JingChaoZuZhi2, Unigene70659\_JingChaoZuZhi2, Unigene70921\_JingChaoZuZhi2, Unigene72476\_JingChaoZuZhi2, Unigene72901\_JingChaoZuZhi2, Unigene73765\_JingChaoZuZhi2, Unigene7407\_JingChaoZuZhi2, Unigene7436\_JingChaoZuZhi2, Unigene74587\_JingChaoZuZhi2, Unigene74771\_JingChaoZuZhi2, Unigene75919\_JingChaoZuZhi2, Unigene75948\_JingChaoZuZhi2, Unigene76685\_JingChaoZuZhi2, Unigene77016\_JingChaoZuZhi2, Unigene7755\_JingChaoZuZhi2, Unigene7881\_JingChaoZuZhi2, Unigene79234\_JingChaoZuZhi2, Unigene79246\_JingChaoZuZhi2, Unigene79577\_JingChaoZuZhi2, Unigene8173\_JingChaoZuZhi2, Unigene81795\_JingChaoZuZhi2, Unigene82161\_JingChaoZuZhi2, Unigene84531\_JingChaoZuZhi2, Unigene86806\_JingChaoZuZhi2, Unigene8689\_JingChaoZuZhi2, Unigene88620\_JingChaoZuZhi2, Unigene89347\_JingChaoZuZhi2, Unigene89752\_JingChaoZuZhi2, Unigene91254\_JingChaoZuZhi2, Unigene91458\_JingChaoZuZhi2, Unigene92037\_JingChaoZuZhi2, Unigene92135\_JingChaoZuZhi2, Unigene92569\_JingChaoZuZhi2, Unigene92942\_JingChaoZuZhi2, Unigene92983\_JingChaoZuZhi2, Unigene93067\_JingChaoZuZhi2, Unigene93088\_JingChaoZuZhi2, Unigene93334\_JingChaoZuZhi2, Unigene93600\_JingChaoZuZhi2, Unigene93910\_JingChaoZuZhi2, Unigene94622\_JingChaoZuZhi2, Unigene94894\_JingChaoZuZhi2, Unigene95016\_JingChaoZuZhi2, Unigene95343\_JingChaoZuZhi2, Unigene95561\_JingChaoZuZhi2, Unigene95658\_JingChaoZuZhi2, Unigene95673\_JingChaoZuZhi2, Unigene9631\_JingChaoZuZhi2 |
| 108 | Arginine and proline metabolism | Unigene11303\_JingChaoZuZhi2, Unigene1252\_JingChaoZuZhi2, Unigene13702\_JingChaoZuZhi2, Unigene13966\_JingChaoZuZhi2, Unigene17078\_JingChaoZuZhi2, Unigene17082\_JingChaoZuZhi2, Unigene17395\_JingChaoZuZhi2, Unigene18335\_JingChaoZuZhi2, Unigene18358\_JingChaoZuZhi2, Unigene18613\_JingChaoZuZhi2, Unigene18788\_JingChaoZuZhi2, Unigene19226\_JingChaoZuZhi2, Unigene20099\_JingChaoZuZhi2, Unigene20114\_JingChaoZuZhi2, Unigene20163\_JingChaoZuZhi2, Unigene20268\_JingChaoZuZhi2, Unigene21049\_JingChaoZuZhi2, Unigene21176\_JingChaoZuZhi2, Unigene21503\_JingChaoZuZhi2, Unigene21986\_JingChaoZuZhi2, Unigene2235\_JingChaoZuZhi2, Unigene24261\_JingChaoZuZhi2, Unigene26104\_JingChaoZuZhi2, Unigene30780\_JingChaoZuZhi2, Unigene3098\_JingChaoZuZhi2, Unigene32752\_JingChaoZuZhi2, Unigene3292\_JingChaoZuZhi2, Unigene3852\_JingChaoZuZhi2, Unigene39505\_JingChaoZuZhi2, Unigene42036\_JingChaoZuZhi2, Unigene46280\_JingChaoZuZhi2, Unigene47262\_JingChaoZuZhi2, Unigene47407\_JingChaoZuZhi2, Unigene47798\_JingChaoZuZhi2, Unigene50147\_JingChaoZuZhi2, Unigene52898\_JingChaoZuZhi2, Unigene52911\_JingChaoZuZhi2, Unigene5944\_JingChaoZuZhi2, Unigene63261\_JingChaoZuZhi2, Unigene63649\_JingChaoZuZhi2, Unigene64169\_JingChaoZuZhi2, Unigene65006\_JingChaoZuZhi2, Unigene67588\_JingChaoZuZhi2, Unigene67613\_JingChaoZuZhi2, Unigene68097\_JingChaoZuZhi2, Unigene69271\_JingChaoZuZhi2, Unigene69536\_JingChaoZuZhi2, Unigene70659\_JingChaoZuZhi2, Unigene72476\_JingChaoZuZhi2, Unigene73853\_JingChaoZuZhi2, Unigene76438\_JingChaoZuZhi2, Unigene76706\_JingChaoZuZhi2, Unigene77197\_JingChaoZuZhi2, Unigene78939\_JingChaoZuZhi2, Unigene79203\_JingChaoZuZhi2, Unigene80032\_JingChaoZuZhi2, Unigene80205\_JingChaoZuZhi2, Unigene80881\_JingChaoZuZhi2, Unigene8153\_JingChaoZuZhi2, Unigene81597\_JingChaoZuZhi2, Unigene82129\_JingChaoZuZhi2, Unigene83634\_JingChaoZuZhi2, Unigene83716\_JingChaoZuZhi2, Unigene84063\_JingChaoZuZhi2, Unigene84546\_JingChaoZuZhi2, Unigene84851\_JingChaoZuZhi2, Unigene86271\_JingChaoZuZhi2, Unigene87044\_JingChaoZuZhi2, Unigene87210\_JingChaoZuZhi2, Unigene87997\_JingChaoZuZhi2, Unigene88819\_JingChaoZuZhi2, Unigene89496\_JingChaoZuZhi2, Unigene89813\_JingChaoZuZhi2, Unigene90127\_JingChaoZuZhi2, Unigene90147\_JingChaoZuZhi2, Unigene90961\_JingChaoZuZhi2, Unigene91663\_JingChaoZuZhi2, Unigene91700\_JingChaoZuZhi2, Unigene9207\_JingChaoZuZhi2, Unigene92868\_JingChaoZuZhi2, Unigene93334\_JingChaoZuZhi2, Unigene94607\_JingChaoZuZhi2, Unigene94837\_JingChaoZuZhi2, Unigene95658\_JingChaoZuZhi2, Unigene95673\_JingChaoZuZhi2, Unigene95695\_JingChaoZuZhi2, Unigene95737\_JingChaoZuZhi2, Unigene95958\_JingChaoZuZhi2, Unigene95959\_JingChaoZuZhi2, Unigene95985\_JingChaoZuZhi2, Unigene96031\_JingChaoZuZhi2, Unigene96100\_JingChaoZuZhi2, Unigene96124\_JingChaoZuZhi2 |
| 109 | Propanoate metabolism | Unigene11312\_JingChaoZuZhi2, Unigene11719\_JingChaoZuZhi2, Unigene16352\_JingChaoZuZhi2, Unigene16663\_JingChaoZuZhi2, Unigene16899\_JingChaoZuZhi2, Unigene17162\_JingChaoZuZhi2, Unigene17401\_JingChaoZuZhi2, Unigene17995\_JingChaoZuZhi2, Unigene18358\_JingChaoZuZhi2, Unigene18409\_JingChaoZuZhi2, Unigene18553\_JingChaoZuZhi2, Unigene18846\_JingChaoZuZhi2, Unigene20010\_JingChaoZuZhi2, Unigene20129\_JingChaoZuZhi2, Unigene20684\_JingChaoZuZhi2, Unigene21049\_JingChaoZuZhi2, Unigene21176\_JingChaoZuZhi2, Unigene21821\_JingChaoZuZhi2, Unigene22352\_JingChaoZuZhi2, Unigene2235\_JingChaoZuZhi2, Unigene22684\_JingChaoZuZhi2, Unigene22793\_JingChaoZuZhi2, Unigene27219\_JingChaoZuZhi2, Unigene32951\_JingChaoZuZhi2, Unigene35187\_JingChaoZuZhi2, Unigene3769\_JingChaoZuZhi2, Unigene41503\_JingChaoZuZhi2, Unigene43097\_JingChaoZuZhi2, Unigene4423\_JingChaoZuZhi2, Unigene45176\_JingChaoZuZhi2, Unigene49331\_JingChaoZuZhi2, Unigene49819\_JingChaoZuZhi2, Unigene5068\_JingChaoZuZhi2, Unigene51532\_JingChaoZuZhi2, Unigene52040\_JingChaoZuZhi2, Unigene52974\_JingChaoZuZhi2, Unigene58763\_JingChaoZuZhi2, Unigene59548\_JingChaoZuZhi2, Unigene60906\_JingChaoZuZhi2, Unigene61162\_JingChaoZuZhi2, Unigene61664\_JingChaoZuZhi2, Unigene62804\_JingChaoZuZhi2, Unigene62962\_JingChaoZuZhi2, Unigene63649\_JingChaoZuZhi2, Unigene63715\_JingChaoZuZhi2, Unigene64862\_JingChaoZuZhi2, Unigene64903\_JingChaoZuZhi2, Unigene65441\_JingChaoZuZhi2, Unigene66337\_JingChaoZuZhi2, Unigene67613\_JingChaoZuZhi2, Unigene72476\_JingChaoZuZhi2, Unigene76068\_JingChaoZuZhi2, Unigene78625\_JingChaoZuZhi2, Unigene78854\_JingChaoZuZhi2, Unigene79135\_JingChaoZuZhi2, Unigene80446\_JingChaoZuZhi2, Unigene80881\_JingChaoZuZhi2, Unigene80971\_JingChaoZuZhi2, Unigene82129\_JingChaoZuZhi2, Unigene8213\_JingChaoZuZhi2, Unigene82435\_JingChaoZuZhi2, Unigene83981\_JingChaoZuZhi2, Unigene84063\_JingChaoZuZhi2, Unigene84124\_JingChaoZuZhi2, Unigene84149\_JingChaoZuZhi2, Unigene84343\_JingChaoZuZhi2, Unigene8454\_JingChaoZuZhi2, Unigene84851\_JingChaoZuZhi2, Unigene85224\_JingChaoZuZhi2, Unigene85365\_JingChaoZuZhi2, Unigene85989\_JingChaoZuZhi2, Unigene86463\_JingChaoZuZhi2, Unigene86998\_JingChaoZuZhi2, Unigene87044\_JingChaoZuZhi2, Unigene87485\_JingChaoZuZhi2, Unigene87847\_JingChaoZuZhi2, Unigene8809\_JingChaoZuZhi2, Unigene88377\_JingChaoZuZhi2, Unigene9006\_JingChaoZuZhi2, Unigene90127\_JingChaoZuZhi2, Unigene91700\_JingChaoZuZhi2, Unigene93212\_JingChaoZuZhi2, Unigene93334\_JingChaoZuZhi2, Unigene93681\_JingChaoZuZhi2, Unigene94353\_JingChaoZuZhi2, Unigene94837\_JingChaoZuZhi2, Unigene95141\_JingChaoZuZhi2, Unigene95160\_JingChaoZuZhi2, Unigene95523\_JingChaoZuZhi2, Unigene95959\_JingChaoZuZhi2, Unigene95985\_JingChaoZuZhi2, Unigene96031\_JingChaoZuZhi2 |
| 110 | Type II diabetes mellitus | Unigene1045\_JingChaoZuZhi2, Unigene11951\_JingChaoZuZhi2, Unigene11961\_JingChaoZuZhi2, Unigene12221\_JingChaoZuZhi2, Unigene12577\_JingChaoZuZhi2, Unigene12823\_JingChaoZuZhi2, Unigene12899\_JingChaoZuZhi2, Unigene13996\_JingChaoZuZhi2, Unigene14707\_JingChaoZuZhi2, Unigene14912\_JingChaoZuZhi2, Unigene15228\_JingChaoZuZhi2, Unigene16762\_JingChaoZuZhi2, Unigene16768\_JingChaoZuZhi2, Unigene17247\_JingChaoZuZhi2, Unigene17839\_JingChaoZuZhi2, Unigene17888\_JingChaoZuZhi2, Unigene18672\_JingChaoZuZhi2, Unigene18821\_JingChaoZuZhi2, Unigene19086\_JingChaoZuZhi2, Unigene19307\_JingChaoZuZhi2, Unigene19730\_JingChaoZuZhi2, Unigene20107\_JingChaoZuZhi2, Unigene20157\_JingChaoZuZhi2, Unigene20840\_JingChaoZuZhi2, Unigene20991\_JingChaoZuZhi2, Unigene21578\_JingChaoZuZhi2, Unigene22426\_JingChaoZuZhi2, Unigene2456\_JingChaoZuZhi2, Unigene27995\_JingChaoZuZhi2, Unigene28263\_JingChaoZuZhi2, Unigene35952\_JingChaoZuZhi2, Unigene3776\_JingChaoZuZhi2, Unigene40143\_JingChaoZuZhi2, Unigene4294\_JingChaoZuZhi2, Unigene48751\_JingChaoZuZhi2, Unigene48948\_JingChaoZuZhi2, Unigene48978\_JingChaoZuZhi2, Unigene52554\_JingChaoZuZhi2, Unigene53568\_JingChaoZuZhi2, Unigene54320\_JingChaoZuZhi2, Unigene5437\_JingChaoZuZhi2, Unigene55687\_JingChaoZuZhi2, Unigene56709\_JingChaoZuZhi2, Unigene5674\_JingChaoZuZhi2, Unigene57488\_JingChaoZuZhi2, Unigene58209\_JingChaoZuZhi2, Unigene5823\_JingChaoZuZhi2, Unigene60180\_JingChaoZuZhi2, Unigene64529\_JingChaoZuZhi2, Unigene64583\_JingChaoZuZhi2, Unigene66060\_JingChaoZuZhi2, Unigene68300\_JingChaoZuZhi2, Unigene70087\_JingChaoZuZhi2, Unigene70140\_JingChaoZuZhi2, Unigene7182\_JingChaoZuZhi2, Unigene7224\_JingChaoZuZhi2, Unigene72674\_JingChaoZuZhi2, Unigene73344\_JingChaoZuZhi2, Unigene73475\_JingChaoZuZhi2, Unigene75121\_JingChaoZuZhi2, Unigene75\_JingChaoZuZhi2, Unigene76400\_JingChaoZuZhi2, Unigene77114\_JingChaoZuZhi2, Unigene77374\_JingChaoZuZhi2, Unigene77570\_JingChaoZuZhi2, Unigene79539\_JingChaoZuZhi2, Unigene81901\_JingChaoZuZhi2, Unigene8191\_JingChaoZuZhi2, Unigene82368\_JingChaoZuZhi2, Unigene83166\_JingChaoZuZhi2, Unigene83440\_JingChaoZuZhi2, Unigene83588\_JingChaoZuZhi2, Unigene84340\_JingChaoZuZhi2, Unigene8464\_JingChaoZuZhi2, Unigene85172\_JingChaoZuZhi2, Unigene86206\_JingChaoZuZhi2, Unigene86526\_JingChaoZuZhi2, Unigene86899\_JingChaoZuZhi2, Unigene87251\_JingChaoZuZhi2, Unigene87305\_JingChaoZuZhi2, Unigene90209\_JingChaoZuZhi2, Unigene90903\_JingChaoZuZhi2, Unigene92112\_JingChaoZuZhi2, Unigene92580\_JingChaoZuZhi2, Unigene92815\_JingChaoZuZhi2, Unigene93409\_JingChaoZuZhi2, Unigene93807\_JingChaoZuZhi2, Unigene9389\_JingChaoZuZhi2, Unigene93928\_JingChaoZuZhi2, Unigene94685\_JingChaoZuZhi2, Unigene95993\_JingChaoZuZhi2, Unigene96294\_JingChaoZuZhi2 |
| 111 | mTOR signaling pathway | Unigene10532\_JingChaoZuZhi2, Unigene10856\_JingChaoZuZhi2, Unigene11431\_JingChaoZuZhi2, Unigene11495\_JingChaoZuZhi2, Unigene11742\_JingChaoZuZhi2, Unigene11961\_JingChaoZuZhi2, Unigene12204\_JingChaoZuZhi2, Unigene12243\_JingChaoZuZhi2, Unigene12823\_JingChaoZuZhi2, Unigene13564\_JingChaoZuZhi2, Unigene14289\_JingChaoZuZhi2, Unigene14912\_JingChaoZuZhi2, Unigene15228\_JingChaoZuZhi2, Unigene15384\_JingChaoZuZhi2, Unigene16012\_JingChaoZuZhi2, Unigene17247\_JingChaoZuZhi2, Unigene18672\_JingChaoZuZhi2, Unigene18729\_JingChaoZuZhi2, Unigene19307\_JingChaoZuZhi2, Unigene19615\_JingChaoZuZhi2, Unigene19624\_JingChaoZuZhi2, Unigene19909\_JingChaoZuZhi2, Unigene20840\_JingChaoZuZhi2, Unigene21132\_JingChaoZuZhi2, Unigene21513\_JingChaoZuZhi2, Unigene21766\_JingChaoZuZhi2, Unigene22079\_JingChaoZuZhi2, Unigene22509\_JingChaoZuZhi2, Unigene22823\_JingChaoZuZhi2, Unigene2456\_JingChaoZuZhi2, Unigene2512\_JingChaoZuZhi2, Unigene33138\_JingChaoZuZhi2, Unigene36555\_JingChaoZuZhi2, Unigene37495\_JingChaoZuZhi2, Unigene3931\_JingChaoZuZhi2, Unigene40020\_JingChaoZuZhi2, Unigene473\_JingChaoZuZhi2, Unigene48751\_JingChaoZuZhi2, Unigene50661\_JingChaoZuZhi2, Unigene51777\_JingChaoZuZhi2, Unigene5253\_JingChaoZuZhi2, Unigene57488\_JingChaoZuZhi2, Unigene61559\_JingChaoZuZhi2, Unigene62360\_JingChaoZuZhi2, Unigene64650\_JingChaoZuZhi2, Unigene66060\_JingChaoZuZhi2, Unigene67983\_JingChaoZuZhi2, Unigene68283\_JingChaoZuZhi2, Unigene69666\_JingChaoZuZhi2, Unigene71974\_JingChaoZuZhi2, Unigene72466\_JingChaoZuZhi2, Unigene72972\_JingChaoZuZhi2, Unigene74169\_JingChaoZuZhi2, Unigene75410\_JingChaoZuZhi2, Unigene78217\_JingChaoZuZhi2, Unigene79307\_JingChaoZuZhi2, Unigene8191\_JingChaoZuZhi2, Unigene82728\_JingChaoZuZhi2, Unigene82913\_JingChaoZuZhi2, Unigene84418\_JingChaoZuZhi2, Unigene85172\_JingChaoZuZhi2, Unigene85576\_JingChaoZuZhi2, Unigene85731\_JingChaoZuZhi2, Unigene85821\_JingChaoZuZhi2, Unigene86206\_JingChaoZuZhi2, Unigene86278\_JingChaoZuZhi2, Unigene86344\_JingChaoZuZhi2, Unigene86456\_JingChaoZuZhi2, Unigene86526\_JingChaoZuZhi2, Unigene86596\_JingChaoZuZhi2, Unigene87565\_JingChaoZuZhi2, Unigene89979\_JingChaoZuZhi2, Unigene91038\_JingChaoZuZhi2, Unigene91538\_JingChaoZuZhi2, Unigene91631\_JingChaoZuZhi2, Unigene91910\_JingChaoZuZhi2, Unigene91919\_JingChaoZuZhi2, Unigene92112\_JingChaoZuZhi2, Unigene93259\_JingChaoZuZhi2, Unigene93409\_JingChaoZuZhi2, Unigene93623\_JingChaoZuZhi2, Unigene93807\_JingChaoZuZhi2, Unigene94230\_JingChaoZuZhi2, Unigene94394\_JingChaoZuZhi2, Unigene94685\_JingChaoZuZhi2, Unigene95443\_JingChaoZuZhi2, Unigene95921\_JingChaoZuZhi2, Unigene95933\_JingChaoZuZhi2, Unigene95947\_JingChaoZuZhi2, Unigene96294\_JingChaoZuZhi2, Unigene9995\_JingChaoZuZhi2 |
| 112 | N-Glycan biosynthesis | Unigene1071\_JingChaoZuZhi2, Unigene10781\_JingChaoZuZhi2, Unigene12263\_JingChaoZuZhi2, Unigene12885\_JingChaoZuZhi2, Unigene13227\_JingChaoZuZhi2, Unigene13435\_JingChaoZuZhi2, Unigene14388\_JingChaoZuZhi2, Unigene14533\_JingChaoZuZhi2, Unigene15542\_JingChaoZuZhi2, Unigene15591\_JingChaoZuZhi2, Unigene1576\_JingChaoZuZhi2, Unigene16460\_JingChaoZuZhi2, Unigene17084\_JingChaoZuZhi2, Unigene17278\_JingChaoZuZhi2, Unigene17423\_JingChaoZuZhi2, Unigene17452\_JingChaoZuZhi2, Unigene18268\_JingChaoZuZhi2, Unigene18935\_JingChaoZuZhi2, Unigene19015\_JingChaoZuZhi2, Unigene19582\_JingChaoZuZhi2, Unigene19619\_JingChaoZuZhi2, Unigene20453\_JingChaoZuZhi2, Unigene21392\_JingChaoZuZhi2, Unigene21754\_JingChaoZuZhi2, Unigene22156\_JingChaoZuZhi2, Unigene22376\_JingChaoZuZhi2, Unigene22462\_JingChaoZuZhi2, Unigene22474\_JingChaoZuZhi2, Unigene2424\_JingChaoZuZhi2, Unigene24264\_JingChaoZuZhi2, Unigene2506\_JingChaoZuZhi2, Unigene30682\_JingChaoZuZhi2, Unigene33161\_JingChaoZuZhi2, Unigene3384\_JingChaoZuZhi2, Unigene38753\_JingChaoZuZhi2, Unigene4384\_JingChaoZuZhi2, Unigene46849\_JingChaoZuZhi2, Unigene47095\_JingChaoZuZhi2, Unigene49337\_JingChaoZuZhi2, Unigene50269\_JingChaoZuZhi2, Unigene55725\_JingChaoZuZhi2, Unigene5640\_JingChaoZuZhi2, Unigene58214\_JingChaoZuZhi2, Unigene58963\_JingChaoZuZhi2, Unigene60260\_JingChaoZuZhi2, Unigene60970\_JingChaoZuZhi2, Unigene61708\_JingChaoZuZhi2, Unigene6207\_JingChaoZuZhi2, Unigene65330\_JingChaoZuZhi2, Unigene67412\_JingChaoZuZhi2, Unigene67968\_JingChaoZuZhi2, Unigene6868\_JingChaoZuZhi2, Unigene7225\_JingChaoZuZhi2, Unigene75828\_JingChaoZuZhi2, Unigene75945\_JingChaoZuZhi2, Unigene76769\_JingChaoZuZhi2, Unigene7810\_JingChaoZuZhi2, Unigene78726\_JingChaoZuZhi2, Unigene78781\_JingChaoZuZhi2, Unigene79033\_JingChaoZuZhi2, Unigene80736\_JingChaoZuZhi2, Unigene80809\_JingChaoZuZhi2, Unigene81192\_JingChaoZuZhi2, Unigene81770\_JingChaoZuZhi2, Unigene82393\_JingChaoZuZhi2, Unigene84663\_JingChaoZuZhi2, Unigene86058\_JingChaoZuZhi2, Unigene86734\_JingChaoZuZhi2, Unigene86826\_JingChaoZuZhi2, Unigene86965\_JingChaoZuZhi2, Unigene87222\_JingChaoZuZhi2, Unigene87559\_JingChaoZuZhi2, Unigene89042\_JingChaoZuZhi2, Unigene89199\_JingChaoZuZhi2, Unigene89238\_JingChaoZuZhi2, Unigene89779\_JingChaoZuZhi2, Unigene90215\_JingChaoZuZhi2, Unigene9167\_JingChaoZuZhi2, Unigene92063\_JingChaoZuZhi2, Unigene93416\_JingChaoZuZhi2, Unigene94416\_JingChaoZuZhi2, Unigene94957\_JingChaoZuZhi2, Unigene95469\_JingChaoZuZhi2, Unigene95473\_JingChaoZuZhi2, Unigene95746\_JingChaoZuZhi2, Unigene95806\_JingChaoZuZhi2, Unigene96178\_JingChaoZuZhi2, Unigene96179\_JingChaoZuZhi2, Unigene9836\_JingChaoZuZhi2 |
| 113 | Citrate cycle (TCA cycle) | Unigene10133\_JingChaoZuZhi2, Unigene11119\_JingChaoZuZhi2, Unigene11491\_JingChaoZuZhi2, Unigene11892\_JingChaoZuZhi2, Unigene14871\_JingChaoZuZhi2, Unigene15659\_JingChaoZuZhi2, Unigene15755\_JingChaoZuZhi2, Unigene15856\_JingChaoZuZhi2, Unigene15867\_JingChaoZuZhi2, Unigene16176\_JingChaoZuZhi2, Unigene16711\_JingChaoZuZhi2, Unigene16738\_JingChaoZuZhi2, Unigene17099\_JingChaoZuZhi2, Unigene17997\_JingChaoZuZhi2, Unigene18132\_JingChaoZuZhi2, Unigene18348\_JingChaoZuZhi2, Unigene18525\_JingChaoZuZhi2, Unigene18776\_JingChaoZuZhi2, Unigene20049\_JingChaoZuZhi2, Unigene20404\_JingChaoZuZhi2, Unigene20684\_JingChaoZuZhi2, Unigene2132\_JingChaoZuZhi2, Unigene2162\_JingChaoZuZhi2, Unigene21756\_JingChaoZuZhi2, Unigene22143\_JingChaoZuZhi2, Unigene22325\_JingChaoZuZhi2, Unigene22582\_JingChaoZuZhi2, Unigene22680\_JingChaoZuZhi2, Unigene2306\_JingChaoZuZhi2, Unigene2487\_JingChaoZuZhi2, Unigene2711\_JingChaoZuZhi2, Unigene32031\_JingChaoZuZhi2, Unigene35136\_JingChaoZuZhi2, Unigene41982\_JingChaoZuZhi2, Unigene42806\_JingChaoZuZhi2, Unigene43097\_JingChaoZuZhi2, Unigene490\_JingChaoZuZhi2, Unigene5057\_JingChaoZuZhi2, Unigene51478\_JingChaoZuZhi2, Unigene51567\_JingChaoZuZhi2, Unigene52598\_JingChaoZuZhi2, Unigene53484\_JingChaoZuZhi2, Unigene53812\_JingChaoZuZhi2, Unigene57635\_JingChaoZuZhi2, Unigene59449\_JingChaoZuZhi2, Unigene59684\_JingChaoZuZhi2, Unigene62545\_JingChaoZuZhi2, Unigene65187\_JingChaoZuZhi2, Unigene6797\_JingChaoZuZhi2, Unigene68509\_JingChaoZuZhi2, Unigene75288\_JingChaoZuZhi2, Unigene75529\_JingChaoZuZhi2, Unigene7608\_JingChaoZuZhi2, Unigene76337\_JingChaoZuZhi2, Unigene7985\_JingChaoZuZhi2, Unigene80504\_JingChaoZuZhi2, Unigene83134\_JingChaoZuZhi2, Unigene83531\_JingChaoZuZhi2, Unigene83623\_JingChaoZuZhi2, Unigene84181\_JingChaoZuZhi2, Unigene84420\_JingChaoZuZhi2, Unigene85584\_JingChaoZuZhi2, Unigene8809\_JingChaoZuZhi2, Unigene89115\_JingChaoZuZhi2, Unigene89233\_JingChaoZuZhi2, Unigene89265\_JingChaoZuZhi2, Unigene89746\_JingChaoZuZhi2, Unigene90935\_JingChaoZuZhi2, Unigene91175\_JingChaoZuZhi2, Unigene91662\_JingChaoZuZhi2, Unigene91854\_JingChaoZuZhi2, Unigene92429\_JingChaoZuZhi2, Unigene9256\_JingChaoZuZhi2, Unigene92681\_JingChaoZuZhi2, Unigene92990\_JingChaoZuZhi2, Unigene93422\_JingChaoZuZhi2, Unigene93828\_JingChaoZuZhi2, Unigene94125\_JingChaoZuZhi2, Unigene94157\_JingChaoZuZhi2, Unigene94464\_JingChaoZuZhi2, Unigene94745\_JingChaoZuZhi2, Unigene94826\_JingChaoZuZhi2, Unigene95160\_JingChaoZuZhi2, Unigene95384\_JingChaoZuZhi2, Unigene95505\_JingChaoZuZhi2, Unigene95648\_JingChaoZuZhi2, Unigene95771\_JingChaoZuZhi2, Unigene96266\_JingChaoZuZhi2, Unigene9748\_JingChaoZuZhi2 |
| 114 | Cytokine-cytokine receptor interaction | Unigene10422\_JingChaoZuZhi2, Unigene10910\_JingChaoZuZhi2, Unigene1298\_JingChaoZuZhi2, Unigene13509\_JingChaoZuZhi2, Unigene14225\_JingChaoZuZhi2, Unigene14842\_JingChaoZuZhi2, Unigene15151\_JingChaoZuZhi2, Unigene15780\_JingChaoZuZhi2, Unigene16735\_JingChaoZuZhi2, Unigene167\_JingChaoZuZhi2, Unigene18462\_JingChaoZuZhi2, Unigene18608\_JingChaoZuZhi2, Unigene18921\_JingChaoZuZhi2, Unigene19422\_JingChaoZuZhi2, Unigene19691\_JingChaoZuZhi2, Unigene19813\_JingChaoZuZhi2, Unigene20305\_JingChaoZuZhi2, Unigene20697\_JingChaoZuZhi2, Unigene21759\_JingChaoZuZhi2, Unigene22504\_JingChaoZuZhi2, Unigene22546\_JingChaoZuZhi2, Unigene22795\_JingChaoZuZhi2, Unigene2790\_JingChaoZuZhi2, Unigene28889\_JingChaoZuZhi2, Unigene31426\_JingChaoZuZhi2, Unigene31924\_JingChaoZuZhi2, Unigene35102\_JingChaoZuZhi2, Unigene3640\_JingChaoZuZhi2, Unigene37741\_JingChaoZuZhi2, Unigene37803\_JingChaoZuZhi2, Unigene39003\_JingChaoZuZhi2, Unigene39577\_JingChaoZuZhi2, Unigene39829\_JingChaoZuZhi2, Unigene39865\_JingChaoZuZhi2, Unigene42346\_JingChaoZuZhi2, Unigene42610\_JingChaoZuZhi2, Unigene46219\_JingChaoZuZhi2, Unigene49798\_JingChaoZuZhi2, Unigene51012\_JingChaoZuZhi2, Unigene51705\_JingChaoZuZhi2, Unigene5240\_JingChaoZuZhi2, Unigene5312\_JingChaoZuZhi2, Unigene53885\_JingChaoZuZhi2, Unigene53953\_JingChaoZuZhi2, Unigene54470\_JingChaoZuZhi2, Unigene58354\_JingChaoZuZhi2, Unigene59510\_JingChaoZuZhi2, Unigene59580\_JingChaoZuZhi2, Unigene61189\_JingChaoZuZhi2, Unigene62786\_JingChaoZuZhi2, Unigene63226\_JingChaoZuZhi2, Unigene63355\_JingChaoZuZhi2, Unigene65294\_JingChaoZuZhi2, Unigene65337\_JingChaoZuZhi2, Unigene67117\_JingChaoZuZhi2, Unigene67589\_JingChaoZuZhi2, Unigene68108\_JingChaoZuZhi2, Unigene68200\_JingChaoZuZhi2, Unigene69203\_JingChaoZuZhi2, Unigene70505\_JingChaoZuZhi2, Unigene72521\_JingChaoZuZhi2, Unigene73826\_JingChaoZuZhi2, Unigene76400\_JingChaoZuZhi2, Unigene78574\_JingChaoZuZhi2, Unigene78957\_JingChaoZuZhi2, Unigene79482\_JingChaoZuZhi2, Unigene79577\_JingChaoZuZhi2, Unigene80583\_JingChaoZuZhi2, Unigene81670\_JingChaoZuZhi2, Unigene8202\_JingChaoZuZhi2, Unigene8248\_JingChaoZuZhi2, Unigene83195\_JingChaoZuZhi2, Unigene84830\_JingChaoZuZhi2, Unigene85072\_JingChaoZuZhi2, Unigene8600\_JingChaoZuZhi2, Unigene86640\_JingChaoZuZhi2, Unigene87474\_JingChaoZuZhi2, Unigene8773\_JingChaoZuZhi2, Unigene87780\_JingChaoZuZhi2, Unigene88522\_JingChaoZuZhi2, Unigene89011\_JingChaoZuZhi2, Unigene90768\_JingChaoZuZhi2, Unigene91038\_JingChaoZuZhi2, Unigene91978\_JingChaoZuZhi2, Unigene92135\_JingChaoZuZhi2, Unigene92942\_JingChaoZuZhi2, Unigene93534\_JingChaoZuZhi2, Unigene95065\_JingChaoZuZhi2, Unigene96269\_JingChaoZuZhi2 |
| 115 | Pyruvate metabolism | Unigene10384\_JingChaoZuZhi2, Unigene11150\_JingChaoZuZhi2, Unigene11312\_JingChaoZuZhi2, Unigene11719\_JingChaoZuZhi2, Unigene14801\_JingChaoZuZhi2, Unigene15755\_JingChaoZuZhi2, Unigene15856\_JingChaoZuZhi2, Unigene16711\_JingChaoZuZhi2, Unigene17995\_JingChaoZuZhi2, Unigene18132\_JingChaoZuZhi2, Unigene18358\_JingChaoZuZhi2, Unigene18525\_JingChaoZuZhi2, Unigene18776\_JingChaoZuZhi2, Unigene19650\_JingChaoZuZhi2, Unigene20129\_JingChaoZuZhi2, Unigene21049\_JingChaoZuZhi2, Unigene21176\_JingChaoZuZhi2, Unigene21320\_JingChaoZuZhi2, Unigene2162\_JingChaoZuZhi2, Unigene22143\_JingChaoZuZhi2, Unigene2235\_JingChaoZuZhi2, Unigene2474\_JingChaoZuZhi2, Unigene27219\_JingChaoZuZhi2, Unigene32031\_JingChaoZuZhi2, Unigene3769\_JingChaoZuZhi2, Unigene48660\_JingChaoZuZhi2, Unigene490\_JingChaoZuZhi2, Unigene49331\_JingChaoZuZhi2, Unigene5057\_JingChaoZuZhi2, Unigene51478\_JingChaoZuZhi2, Unigene52040\_JingChaoZuZhi2, Unigene52974\_JingChaoZuZhi2, Unigene53419\_JingChaoZuZhi2, Unigene57635\_JingChaoZuZhi2, Unigene58763\_JingChaoZuZhi2, Unigene59449\_JingChaoZuZhi2, Unigene59735\_JingChaoZuZhi2, Unigene59979\_JingChaoZuZhi2, Unigene60906\_JingChaoZuZhi2, Unigene63649\_JingChaoZuZhi2, Unigene6714\_JingChaoZuZhi2, Unigene67613\_JingChaoZuZhi2, Unigene70893\_JingChaoZuZhi2, Unigene72476\_JingChaoZuZhi2, Unigene72674\_JingChaoZuZhi2, Unigene75288\_JingChaoZuZhi2, Unigene75529\_JingChaoZuZhi2, Unigene7591\_JingChaoZuZhi2, Unigene7608\_JingChaoZuZhi2, Unigene76337\_JingChaoZuZhi2, Unigene77454\_JingChaoZuZhi2, Unigene80141\_JingChaoZuZhi2, Unigene80504\_JingChaoZuZhi2, Unigene80881\_JingChaoZuZhi2, Unigene80971\_JingChaoZuZhi2, Unigene82129\_JingChaoZuZhi2, Unigene83531\_JingChaoZuZhi2, Unigene83623\_JingChaoZuZhi2, Unigene84063\_JingChaoZuZhi2, Unigene84417\_JingChaoZuZhi2, Unigene84851\_JingChaoZuZhi2, Unigene85224\_JingChaoZuZhi2, Unigene85365\_JingChaoZuZhi2, Unigene86998\_JingChaoZuZhi2, Unigene87044\_JingChaoZuZhi2, Unigene87054\_JingChaoZuZhi2, Unigene9006\_JingChaoZuZhi2, Unigene90127\_JingChaoZuZhi2, Unigene90268\_JingChaoZuZhi2, Unigene91175\_JingChaoZuZhi2, Unigene91662\_JingChaoZuZhi2, Unigene91700\_JingChaoZuZhi2, Unigene92489\_JingChaoZuZhi2, Unigene92815\_JingChaoZuZhi2, Unigene93212\_JingChaoZuZhi2, Unigene93334\_JingChaoZuZhi2, Unigene93422\_JingChaoZuZhi2, Unigene93928\_JingChaoZuZhi2, Unigene94353\_JingChaoZuZhi2, Unigene94745\_JingChaoZuZhi2, Unigene94759\_JingChaoZuZhi2, Unigene94837\_JingChaoZuZhi2, Unigene95493\_JingChaoZuZhi2, Unigene95648\_JingChaoZuZhi2, Unigene95818\_JingChaoZuZhi2, Unigene95959\_JingChaoZuZhi2, Unigene95985\_JingChaoZuZhi2, Unigene96031\_JingChaoZuZhi2 |
| 116 | NOD-like receptor signaling pathway | Unigene10679\_JingChaoZuZhi2, Unigene10755\_JingChaoZuZhi2, Unigene11723\_JingChaoZuZhi2, Unigene11732\_JingChaoZuZhi2, Unigene11951\_JingChaoZuZhi2, Unigene12633\_JingChaoZuZhi2, Unigene1301\_JingChaoZuZhi2, Unigene13181\_JingChaoZuZhi2, Unigene13291\_JingChaoZuZhi2, Unigene13332\_JingChaoZuZhi2, Unigene14635\_JingChaoZuZhi2, Unigene15675\_JingChaoZuZhi2, Unigene16263\_JingChaoZuZhi2, Unigene17018\_JingChaoZuZhi2, Unigene19826\_JingChaoZuZhi2, Unigene20024\_JingChaoZuZhi2, Unigene20120\_JingChaoZuZhi2, Unigene20157\_JingChaoZuZhi2, Unigene20553\_JingChaoZuZhi2, Unigene20991\_JingChaoZuZhi2, Unigene21697\_JingChaoZuZhi2, Unigene2214\_JingChaoZuZhi2, Unigene22203\_JingChaoZuZhi2, Unigene22466\_JingChaoZuZhi2, Unigene22482\_JingChaoZuZhi2, Unigene22604\_JingChaoZuZhi2, Unigene22672\_JingChaoZuZhi2, Unigene260\_JingChaoZuZhi2, Unigene2628\_JingChaoZuZhi2, Unigene27072\_JingChaoZuZhi2, Unigene27651\_JingChaoZuZhi2, Unigene34365\_JingChaoZuZhi2, Unigene3560\_JingChaoZuZhi2, Unigene3585\_JingChaoZuZhi2, Unigene36751\_JingChaoZuZhi2, Unigene37070\_JingChaoZuZhi2, Unigene38741\_JingChaoZuZhi2, Unigene41982\_JingChaoZuZhi2, Unigene4294\_JingChaoZuZhi2, Unigene44191\_JingChaoZuZhi2, Unigene45739\_JingChaoZuZhi2, Unigene47083\_JingChaoZuZhi2, Unigene47450\_JingChaoZuZhi2, Unigene48223\_JingChaoZuZhi2, Unigene4853\_JingChaoZuZhi2, Unigene50611\_JingChaoZuZhi2, Unigene5099\_JingChaoZuZhi2, Unigene54320\_JingChaoZuZhi2, Unigene566\_JingChaoZuZhi2, Unigene57579\_JingChaoZuZhi2, Unigene58582\_JingChaoZuZhi2, Unigene62841\_JingChaoZuZhi2, Unigene64126\_JingChaoZuZhi2, Unigene6459\_JingChaoZuZhi2, Unigene65879\_JingChaoZuZhi2, Unigene6815\_JingChaoZuZhi2, Unigene71665\_JingChaoZuZhi2, Unigene72033\_JingChaoZuZhi2, Unigene73162\_JingChaoZuZhi2, Unigene76392\_JingChaoZuZhi2, Unigene77209\_JingChaoZuZhi2, Unigene77570\_JingChaoZuZhi2, Unigene79016\_JingChaoZuZhi2, Unigene81480\_JingChaoZuZhi2, Unigene8191\_JingChaoZuZhi2, Unigene83777\_JingChaoZuZhi2, Unigene83978\_JingChaoZuZhi2, Unigene85038\_JingChaoZuZhi2, Unigene88299\_JingChaoZuZhi2, Unigene88686\_JingChaoZuZhi2, Unigene88942\_JingChaoZuZhi2, Unigene89774\_JingChaoZuZhi2, Unigene90006\_JingChaoZuZhi2, Unigene90071\_JingChaoZuZhi2, Unigene90468\_JingChaoZuZhi2, Unigene91201\_JingChaoZuZhi2, Unigene91574\_JingChaoZuZhi2, Unigene93677\_JingChaoZuZhi2, Unigene93912\_JingChaoZuZhi2, Unigene94236\_JingChaoZuZhi2, Unigene94619\_JingChaoZuZhi2, Unigene94858\_JingChaoZuZhi2, Unigene9517\_JingChaoZuZhi2, Unigene95344\_JingChaoZuZhi2, Unigene95446\_JingChaoZuZhi2, Unigene95674\_JingChaoZuZhi2 |
| 117 | Complement and coagulation cascades | Unigene11464\_JingChaoZuZhi2, Unigene11732\_JingChaoZuZhi2, Unigene1288\_JingChaoZuZhi2, Unigene13132\_JingChaoZuZhi2, Unigene13891\_JingChaoZuZhi2, Unigene14792\_JingChaoZuZhi2, Unigene15883\_JingChaoZuZhi2, Unigene16289\_JingChaoZuZhi2, Unigene16884\_JingChaoZuZhi2, Unigene17062\_JingChaoZuZhi2, Unigene17756\_JingChaoZuZhi2, Unigene17977\_JingChaoZuZhi2, Unigene18966\_JingChaoZuZhi2, Unigene19209\_JingChaoZuZhi2, Unigene19406\_JingChaoZuZhi2, Unigene19748\_JingChaoZuZhi2, Unigene20046\_JingChaoZuZhi2, Unigene20995\_JingChaoZuZhi2, Unigene21996\_JingChaoZuZhi2, Unigene22405\_JingChaoZuZhi2, Unigene22611\_JingChaoZuZhi2, Unigene22819\_JingChaoZuZhi2, Unigene2918\_JingChaoZuZhi2, Unigene29413\_JingChaoZuZhi2, Unigene32417\_JingChaoZuZhi2, Unigene328\_JingChaoZuZhi2, Unigene33930\_JingChaoZuZhi2, Unigene35493\_JingChaoZuZhi2, Unigene375\_JingChaoZuZhi2, Unigene40559\_JingChaoZuZhi2, Unigene4425\_JingChaoZuZhi2, Unigene4470\_JingChaoZuZhi2, Unigene49754\_JingChaoZuZhi2, Unigene51133\_JingChaoZuZhi2, Unigene5318\_JingChaoZuZhi2, Unigene55693\_JingChaoZuZhi2, Unigene58450\_JingChaoZuZhi2, Unigene59160\_JingChaoZuZhi2, Unigene59608\_JingChaoZuZhi2, Unigene59747\_JingChaoZuZhi2, Unigene59846\_JingChaoZuZhi2, Unigene60139\_JingChaoZuZhi2, Unigene61354\_JingChaoZuZhi2, Unigene61626\_JingChaoZuZhi2, Unigene62511\_JingChaoZuZhi2, Unigene62590\_JingChaoZuZhi2, Unigene62824\_JingChaoZuZhi2, Unigene63875\_JingChaoZuZhi2, Unigene66093\_JingChaoZuZhi2, Unigene67045\_JingChaoZuZhi2, Unigene69094\_JingChaoZuZhi2, Unigene7068\_JingChaoZuZhi2, Unigene72522\_JingChaoZuZhi2, Unigene72563\_JingChaoZuZhi2, Unigene7286\_JingChaoZuZhi2, Unigene75793\_JingChaoZuZhi2, Unigene75821\_JingChaoZuZhi2, Unigene76604\_JingChaoZuZhi2, Unigene76756\_JingChaoZuZhi2, Unigene77558\_JingChaoZuZhi2, Unigene78700\_JingChaoZuZhi2, Unigene79077\_JingChaoZuZhi2, Unigene81700\_JingChaoZuZhi2, Unigene81899\_JingChaoZuZhi2, Unigene82040\_JingChaoZuZhi2, Unigene82811\_JingChaoZuZhi2, Unigene82\_JingChaoZuZhi2, Unigene84381\_JingChaoZuZhi2, Unigene8596\_JingChaoZuZhi2, Unigene87946\_JingChaoZuZhi2, Unigene88422\_JingChaoZuZhi2, Unigene88706\_JingChaoZuZhi2, Unigene89057\_JingChaoZuZhi2, Unigene90881\_JingChaoZuZhi2, Unigene91174\_JingChaoZuZhi2, Unigene91302\_JingChaoZuZhi2, Unigene91994\_JingChaoZuZhi2, Unigene92048\_JingChaoZuZhi2, Unigene92378\_JingChaoZuZhi2, Unigene94214\_JingChaoZuZhi2, Unigene94825\_JingChaoZuZhi2, Unigene94828\_JingChaoZuZhi2, Unigene95042\_JingChaoZuZhi2, Unigene95626\_JingChaoZuZhi2, Unigene96086\_JingChaoZuZhi2, Unigene990\_JingChaoZuZhi2 |
| 118 | Pancreatic cancer | Unigene10422\_JingChaoZuZhi2, Unigene10610\_JingChaoZuZhi2, Unigene11473\_JingChaoZuZhi2, Unigene11951\_JingChaoZuZhi2, Unigene11961\_JingChaoZuZhi2, Unigene12370\_JingChaoZuZhi2, Unigene13946\_JingChaoZuZhi2, Unigene14231\_JingChaoZuZhi2, Unigene14912\_JingChaoZuZhi2, Unigene15216\_JingChaoZuZhi2, Unigene15228\_JingChaoZuZhi2, Unigene17247\_JingChaoZuZhi2, Unigene17467\_JingChaoZuZhi2, Unigene18672\_JingChaoZuZhi2, Unigene19021\_JingChaoZuZhi2, Unigene19463\_JingChaoZuZhi2, Unigene19813\_JingChaoZuZhi2, Unigene20157\_JingChaoZuZhi2, Unigene20780\_JingChaoZuZhi2, Unigene20991\_JingChaoZuZhi2, Unigene21513\_JingChaoZuZhi2, Unigene21697\_JingChaoZuZhi2, Unigene22466\_JingChaoZuZhi2, Unigene2275\_JingChaoZuZhi2, Unigene22810\_JingChaoZuZhi2, Unigene2456\_JingChaoZuZhi2, Unigene2790\_JingChaoZuZhi2, Unigene3299\_JingChaoZuZhi2, Unigene3640\_JingChaoZuZhi2, Unigene3700\_JingChaoZuZhi2, Unigene3931\_JingChaoZuZhi2, Unigene39584\_JingChaoZuZhi2, Unigene4294\_JingChaoZuZhi2, Unigene42981\_JingChaoZuZhi2, Unigene43120\_JingChaoZuZhi2, Unigene46240\_JingChaoZuZhi2, Unigene47217\_JingChaoZuZhi2, Unigene48751\_JingChaoZuZhi2, Unigene49664\_JingChaoZuZhi2, Unigene49798\_JingChaoZuZhi2, Unigene51285\_JingChaoZuZhi2, Unigene54320\_JingChaoZuZhi2, Unigene54470\_JingChaoZuZhi2, Unigene56642\_JingChaoZuZhi2, Unigene57488\_JingChaoZuZhi2, Unigene5758\_JingChaoZuZhi2, Unigene59480\_JingChaoZuZhi2, Unigene6028\_JingChaoZuZhi2, Unigene61760\_JingChaoZuZhi2, Unigene62786\_JingChaoZuZhi2, Unigene64697\_JingChaoZuZhi2, Unigene67177\_JingChaoZuZhi2, Unigene70549\_JingChaoZuZhi2, Unigene72136\_JingChaoZuZhi2, Unigene72521\_JingChaoZuZhi2, Unigene72769\_JingChaoZuZhi2, Unigene77570\_JingChaoZuZhi2, Unigene7827\_JingChaoZuZhi2, Unigene78574\_JingChaoZuZhi2, Unigene79166\_JingChaoZuZhi2, Unigene79683\_JingChaoZuZhi2, Unigene81163\_JingChaoZuZhi2, Unigene8191\_JingChaoZuZhi2, Unigene84930\_JingChaoZuZhi2, Unigene8600\_JingChaoZuZhi2, Unigene86206\_JingChaoZuZhi2, Unigene86458\_JingChaoZuZhi2, Unigene86526\_JingChaoZuZhi2, Unigene876\_JingChaoZuZhi2, Unigene91038\_JingChaoZuZhi2, Unigene91179\_JingChaoZuZhi2, Unigene92019\_JingChaoZuZhi2, Unigene92112\_JingChaoZuZhi2, Unigene92394\_JingChaoZuZhi2, Unigene92738\_JingChaoZuZhi2, Unigene93782\_JingChaoZuZhi2, Unigene93807\_JingChaoZuZhi2, Unigene93953\_JingChaoZuZhi2, Unigene94685\_JingChaoZuZhi2, Unigene95084\_JingChaoZuZhi2, Unigene95315\_JingChaoZuZhi2, Unigene95875\_JingChaoZuZhi2, Unigene95883\_JingChaoZuZhi2, Unigene96018\_JingChaoZuZhi2, Unigene96294\_JingChaoZuZhi2, Unigene9902\_JingChaoZuZhi2 |
| 119 | beta-Alanine metabolism | Unigene16352\_JingChaoZuZhi2, Unigene16663\_JingChaoZuZhi2, Unigene16899\_JingChaoZuZhi2, Unigene17082\_JingChaoZuZhi2, Unigene18000\_JingChaoZuZhi2, Unigene18335\_JingChaoZuZhi2, Unigene18358\_JingChaoZuZhi2, Unigene18409\_JingChaoZuZhi2, Unigene18553\_JingChaoZuZhi2, Unigene18846\_JingChaoZuZhi2, Unigene21049\_JingChaoZuZhi2, Unigene21176\_JingChaoZuZhi2, Unigene21821\_JingChaoZuZhi2, Unigene22352\_JingChaoZuZhi2, Unigene2235\_JingChaoZuZhi2, Unigene22416\_JingChaoZuZhi2, Unigene22793\_JingChaoZuZhi2, Unigene2553\_JingChaoZuZhi2, Unigene35187\_JingChaoZuZhi2, Unigene35450\_JingChaoZuZhi2, Unigene36386\_JingChaoZuZhi2, Unigene37317\_JingChaoZuZhi2, Unigene42890\_JingChaoZuZhi2, Unigene43926\_JingChaoZuZhi2, Unigene45176\_JingChaoZuZhi2, Unigene47206\_JingChaoZuZhi2, Unigene49819\_JingChaoZuZhi2, Unigene5068\_JingChaoZuZhi2, Unigene51532\_JingChaoZuZhi2, Unigene52898\_JingChaoZuZhi2, Unigene53362\_JingChaoZuZhi2, Unigene55906\_JingChaoZuZhi2, Unigene59548\_JingChaoZuZhi2, Unigene59836\_JingChaoZuZhi2, Unigene61162\_JingChaoZuZhi2, Unigene61664\_JingChaoZuZhi2, Unigene62962\_JingChaoZuZhi2, Unigene63649\_JingChaoZuZhi2, Unigene63715\_JingChaoZuZhi2, Unigene64903\_JingChaoZuZhi2, Unigene65441\_JingChaoZuZhi2, Unigene662\_JingChaoZuZhi2, Unigene66337\_JingChaoZuZhi2, Unigene67613\_JingChaoZuZhi2, Unigene69209\_JingChaoZuZhi2, Unigene71235\_JingChaoZuZhi2, Unigene72476\_JingChaoZuZhi2, Unigene72849\_JingChaoZuZhi2, Unigene76068\_JingChaoZuZhi2, Unigene78854\_JingChaoZuZhi2, Unigene79135\_JingChaoZuZhi2, Unigene79590\_JingChaoZuZhi2, Unigene80446\_JingChaoZuZhi2, Unigene80881\_JingChaoZuZhi2, Unigene82129\_JingChaoZuZhi2, Unigene8213\_JingChaoZuZhi2, Unigene82435\_JingChaoZuZhi2, Unigene82594\_JingChaoZuZhi2, Unigene84063\_JingChaoZuZhi2, Unigene84124\_JingChaoZuZhi2, Unigene84149\_JingChaoZuZhi2, Unigene84343\_JingChaoZuZhi2, Unigene8454\_JingChaoZuZhi2, Unigene84851\_JingChaoZuZhi2, Unigene85640\_JingChaoZuZhi2, Unigene85989\_JingChaoZuZhi2, Unigene87044\_JingChaoZuZhi2, Unigene87485\_JingChaoZuZhi2, Unigene88377\_JingChaoZuZhi2, Unigene90127\_JingChaoZuZhi2, Unigene90999\_JingChaoZuZhi2, Unigene91700\_JingChaoZuZhi2, Unigene91914\_JingChaoZuZhi2, Unigene93334\_JingChaoZuZhi2, Unigene93589\_JingChaoZuZhi2, Unigene93666\_JingChaoZuZhi2, Unigene93681\_JingChaoZuZhi2, Unigene94837\_JingChaoZuZhi2, Unigene95141\_JingChaoZuZhi2, Unigene95155\_JingChaoZuZhi2, Unigene95959\_JingChaoZuZhi2, Unigene95985\_JingChaoZuZhi2, Unigene96031\_JingChaoZuZhi2 |
| 120 | Glioma | Unigene10318\_JingChaoZuZhi2, Unigene10422\_JingChaoZuZhi2, Unigene11000\_JingChaoZuZhi2, Unigene11961\_JingChaoZuZhi2, Unigene12420\_JingChaoZuZhi2, Unigene12577\_JingChaoZuZhi2, Unigene12823\_JingChaoZuZhi2, Unigene13176\_JingChaoZuZhi2, Unigene13510\_JingChaoZuZhi2, Unigene14912\_JingChaoZuZhi2, Unigene15228\_JingChaoZuZhi2, Unigene16566\_JingChaoZuZhi2, Unigene17247\_JingChaoZuZhi2, Unigene17467\_JingChaoZuZhi2, Unigene18464\_JingChaoZuZhi2, Unigene18672\_JingChaoZuZhi2, Unigene18868\_JingChaoZuZhi2, Unigene19046\_JingChaoZuZhi2, Unigene19307\_JingChaoZuZhi2, Unigene20840\_JingChaoZuZhi2, Unigene21295\_JingChaoZuZhi2, Unigene21513\_JingChaoZuZhi2, Unigene21578\_JingChaoZuZhi2, Unigene22779\_JingChaoZuZhi2, Unigene2456\_JingChaoZuZhi2, Unigene26730\_JingChaoZuZhi2, Unigene2790\_JingChaoZuZhi2, Unigene2960\_JingChaoZuZhi2, Unigene3931\_JingChaoZuZhi2, Unigene4186\_JingChaoZuZhi2, Unigene42981\_JingChaoZuZhi2, Unigene4377\_JingChaoZuZhi2, Unigene4838\_JingChaoZuZhi2, Unigene48751\_JingChaoZuZhi2, Unigene49798\_JingChaoZuZhi2, Unigene51285\_JingChaoZuZhi2, Unigene54470\_JingChaoZuZhi2, Unigene56642\_JingChaoZuZhi2, Unigene57488\_JingChaoZuZhi2, Unigene5758\_JingChaoZuZhi2, Unigene6028\_JingChaoZuZhi2, Unigene62786\_JingChaoZuZhi2, Unigene64164\_JingChaoZuZhi2, Unigene64583\_JingChaoZuZhi2, Unigene64830\_JingChaoZuZhi2, Unigene6493\_JingChaoZuZhi2, Unigene66060\_JingChaoZuZhi2, Unigene67655\_JingChaoZuZhi2, Unigene67793\_JingChaoZuZhi2, Unigene70549\_JingChaoZuZhi2, Unigene70723\_JingChaoZuZhi2, Unigene72521\_JingChaoZuZhi2, Unigene78574\_JingChaoZuZhi2, Unigene8191\_JingChaoZuZhi2, Unigene84930\_JingChaoZuZhi2, Unigene8507\_JingChaoZuZhi2, Unigene85172\_JingChaoZuZhi2, Unigene85574\_JingChaoZuZhi2, Unigene86206\_JingChaoZuZhi2, Unigene86526\_JingChaoZuZhi2, Unigene87332\_JingChaoZuZhi2, Unigene876\_JingChaoZuZhi2, Unigene88594\_JingChaoZuZhi2, Unigene88724\_JingChaoZuZhi2, Unigene88748\_JingChaoZuZhi2, Unigene91612\_JingChaoZuZhi2, Unigene91924\_JingChaoZuZhi2, Unigene91958\_JingChaoZuZhi2, Unigene92112\_JingChaoZuZhi2, Unigene93409\_JingChaoZuZhi2, Unigene93588\_JingChaoZuZhi2, Unigene93714\_JingChaoZuZhi2, Unigene93807\_JingChaoZuZhi2, Unigene94149\_JingChaoZuZhi2, Unigene94456\_JingChaoZuZhi2, Unigene9461\_JingChaoZuZhi2, Unigene94685\_JingChaoZuZhi2, Unigene95456\_JingChaoZuZhi2, Unigene95677\_JingChaoZuZhi2, Unigene95791\_JingChaoZuZhi2, Unigene96281\_JingChaoZuZhi2, Unigene96294\_JingChaoZuZhi2, Unigene9902\_JingChaoZuZhi2 |
| 121 | Toll-like receptor signaling pathway | Unigene11732\_JingChaoZuZhi2, Unigene11951\_JingChaoZuZhi2, Unigene11961\_JingChaoZuZhi2, Unigene1301\_JingChaoZuZhi2, Unigene13332\_JingChaoZuZhi2, Unigene14109\_JingChaoZuZhi2, Unigene14635\_JingChaoZuZhi2, Unigene14912\_JingChaoZuZhi2, Unigene15228\_JingChaoZuZhi2, Unigene15470\_JingChaoZuZhi2, Unigene17178\_JingChaoZuZhi2, Unigene17247\_JingChaoZuZhi2, Unigene1829\_JingChaoZuZhi2, Unigene18515\_JingChaoZuZhi2, Unigene18672\_JingChaoZuZhi2, Unigene18715\_JingChaoZuZhi2, Unigene18944\_JingChaoZuZhi2, Unigene19021\_JingChaoZuZhi2, Unigene20120\_JingChaoZuZhi2, Unigene20157\_JingChaoZuZhi2, Unigene20450\_JingChaoZuZhi2, Unigene20991\_JingChaoZuZhi2, Unigene21472\_JingChaoZuZhi2, Unigene21691\_JingChaoZuZhi2, Unigene21697\_JingChaoZuZhi2, Unigene22126\_JingChaoZuZhi2, Unigene22466\_JingChaoZuZhi2, Unigene22482\_JingChaoZuZhi2, Unigene22604\_JingChaoZuZhi2, Unigene2456\_JingChaoZuZhi2, Unigene25870\_JingChaoZuZhi2, Unigene260\_JingChaoZuZhi2, Unigene2628\_JingChaoZuZhi2, Unigene2687\_JingChaoZuZhi2, Unigene3931\_JingChaoZuZhi2, Unigene4294\_JingChaoZuZhi2, Unigene47155\_JingChaoZuZhi2, Unigene4853\_JingChaoZuZhi2, Unigene48751\_JingChaoZuZhi2, Unigene5099\_JingChaoZuZhi2, Unigene5314\_JingChaoZuZhi2, Unigene54320\_JingChaoZuZhi2, Unigene57488\_JingChaoZuZhi2, Unigene58550\_JingChaoZuZhi2, Unigene6028\_JingChaoZuZhi2, Unigene62841\_JingChaoZuZhi2, Unigene6459\_JingChaoZuZhi2, Unigene69071\_JingChaoZuZhi2, Unigene70549\_JingChaoZuZhi2, Unigene70636\_JingChaoZuZhi2, Unigene71643\_JingChaoZuZhi2, Unigene72033\_JingChaoZuZhi2, Unigene7459\_JingChaoZuZhi2, Unigene77570\_JingChaoZuZhi2, Unigene8191\_JingChaoZuZhi2, Unigene81948\_JingChaoZuZhi2, Unigene82073\_JingChaoZuZhi2, Unigene85615\_JingChaoZuZhi2, Unigene85880\_JingChaoZuZhi2, Unigene85958\_JingChaoZuZhi2, Unigene86206\_JingChaoZuZhi2, Unigene86526\_JingChaoZuZhi2, Unigene88451\_JingChaoZuZhi2, Unigene88542\_JingChaoZuZhi2, Unigene88942\_JingChaoZuZhi2, Unigene89492\_JingChaoZuZhi2, Unigene91179\_JingChaoZuZhi2, Unigene91661\_JingChaoZuZhi2, Unigene92112\_JingChaoZuZhi2, Unigene93807\_JingChaoZuZhi2, Unigene93859\_JingChaoZuZhi2, Unigene93912\_JingChaoZuZhi2, Unigene94236\_JingChaoZuZhi2, Unigene94619\_JingChaoZuZhi2, Unigene94685\_JingChaoZuZhi2, Unigene94844\_JingChaoZuZhi2, Unigene9488\_JingChaoZuZhi2, Unigene94918\_JingChaoZuZhi2, Unigene9517\_JingChaoZuZhi2, Unigene9520\_JingChaoZuZhi2, Unigene95875\_JingChaoZuZhi2, Unigene96294\_JingChaoZuZhi2 |
| 122 | p53 signaling pathway | Unigene10073\_JingChaoZuZhi2, Unigene10286\_JingChaoZuZhi2, Unigene11431\_JingChaoZuZhi2, Unigene12243\_JingChaoZuZhi2, Unigene12809\_JingChaoZuZhi2, Unigene13176\_JingChaoZuZhi2, Unigene13254\_JingChaoZuZhi2, Unigene13564\_JingChaoZuZhi2, Unigene14141\_JingChaoZuZhi2, Unigene14390\_JingChaoZuZhi2, Unigene15375\_JingChaoZuZhi2, Unigene15998\_JingChaoZuZhi2, Unigene16200\_JingChaoZuZhi2, Unigene1714\_JingChaoZuZhi2, Unigene173\_JingChaoZuZhi2, Unigene17879\_JingChaoZuZhi2, Unigene18309\_JingChaoZuZhi2, Unigene18925\_JingChaoZuZhi2, Unigene19013\_JingChaoZuZhi2, Unigene19453\_JingChaoZuZhi2, Unigene20297\_JingChaoZuZhi2, Unigene20314\_JingChaoZuZhi2, Unigene21379\_JingChaoZuZhi2, Unigene21848\_JingChaoZuZhi2, Unigene22079\_JingChaoZuZhi2, Unigene22255\_JingChaoZuZhi2, Unigene22459\_JingChaoZuZhi2, Unigene25346\_JingChaoZuZhi2, Unigene3022\_JingChaoZuZhi2, Unigene38124\_JingChaoZuZhi2, Unigene38708\_JingChaoZuZhi2, Unigene42981\_JingChaoZuZhi2, Unigene4377\_JingChaoZuZhi2, Unigene5003\_JingChaoZuZhi2, Unigene51285\_JingChaoZuZhi2, Unigene56642\_JingChaoZuZhi2, Unigene5871\_JingChaoZuZhi2, Unigene5882\_JingChaoZuZhi2, Unigene6710\_JingChaoZuZhi2, Unigene68298\_JingChaoZuZhi2, Unigene70584\_JingChaoZuZhi2, Unigene7068\_JingChaoZuZhi2, Unigene7245\_JingChaoZuZhi2, Unigene73510\_JingChaoZuZhi2, Unigene73864\_JingChaoZuZhi2, Unigene76147\_JingChaoZuZhi2, Unigene76511\_JingChaoZuZhi2, Unigene77181\_JingChaoZuZhi2, Unigene79940\_JingChaoZuZhi2, Unigene81000\_JingChaoZuZhi2, Unigene81490\_JingChaoZuZhi2, Unigene84418\_JingChaoZuZhi2, Unigene84844\_JingChaoZuZhi2, Unigene84930\_JingChaoZuZhi2, Unigene84996\_JingChaoZuZhi2, Unigene85771\_JingChaoZuZhi2, Unigene86091\_JingChaoZuZhi2, Unigene86959\_JingChaoZuZhi2, Unigene87565\_JingChaoZuZhi2, Unigene88318\_JingChaoZuZhi2, Unigene88400\_JingChaoZuZhi2, Unigene88748\_JingChaoZuZhi2, Unigene90535\_JingChaoZuZhi2, Unigene90541\_JingChaoZuZhi2, Unigene91147\_JingChaoZuZhi2, Unigene91580\_JingChaoZuZhi2, Unigene91668\_JingChaoZuZhi2, Unigene91705\_JingChaoZuZhi2, Unigene93522\_JingChaoZuZhi2, Unigene94276\_JingChaoZuZhi2, Unigene94377\_JingChaoZuZhi2, Unigene94829\_JingChaoZuZhi2, Unigene95573\_JingChaoZuZhi2, Unigene95791\_JingChaoZuZhi2, Unigene95866\_JingChaoZuZhi2, Unigene95901\_JingChaoZuZhi2, Unigene95981\_JingChaoZuZhi2, Unigene95994\_JingChaoZuZhi2, Unigene96084\_JingChaoZuZhi2, Unigene96281\_JingChaoZuZhi2, Unigene9787\_JingChaoZuZhi2 |
| 123 | Apoptosis | Unigene10679\_JingChaoZuZhi2, Unigene10755\_JingChaoZuZhi2, Unigene11961\_JingChaoZuZhi2, Unigene14370\_JingChaoZuZhi2, Unigene14842\_JingChaoZuZhi2, Unigene14912\_JingChaoZuZhi2, Unigene15228\_JingChaoZuZhi2, Unigene15998\_JingChaoZuZhi2, Unigene17178\_JingChaoZuZhi2, Unigene17247\_JingChaoZuZhi2, Unigene17268\_JingChaoZuZhi2, Unigene18307\_JingChaoZuZhi2, Unigene18672\_JingChaoZuZhi2, Unigene19826\_JingChaoZuZhi2, Unigene20157\_JingChaoZuZhi2, Unigene20611\_JingChaoZuZhi2, Unigene20991\_JingChaoZuZhi2, Unigene21246\_JingChaoZuZhi2, Unigene21697\_JingChaoZuZhi2, Unigene21737\_JingChaoZuZhi2, Unigene21815\_JingChaoZuZhi2, Unigene22466\_JingChaoZuZhi2, Unigene2343\_JingChaoZuZhi2, Unigene2456\_JingChaoZuZhi2, Unigene25147\_JingChaoZuZhi2, Unigene25870\_JingChaoZuZhi2, Unigene3585\_JingChaoZuZhi2, Unigene3931\_JingChaoZuZhi2, Unigene39577\_JingChaoZuZhi2, Unigene44191\_JingChaoZuZhi2, Unigene45739\_JingChaoZuZhi2, Unigene45\_JingChaoZuZhi2, Unigene46219\_JingChaoZuZhi2, Unigene47155\_JingChaoZuZhi2, Unigene48751\_JingChaoZuZhi2, Unigene51705\_JingChaoZuZhi2, Unigene51819\_JingChaoZuZhi2, Unigene54320\_JingChaoZuZhi2, Unigene57488\_JingChaoZuZhi2, Unigene58426\_JingChaoZuZhi2, Unigene58550\_JingChaoZuZhi2, Unigene5932\_JingChaoZuZhi2, Unigene63226\_JingChaoZuZhi2, Unigene65294\_JingChaoZuZhi2, Unigene65879\_JingChaoZuZhi2, Unigene68108\_JingChaoZuZhi2, Unigene68200\_JingChaoZuZhi2, Unigene68298\_JingChaoZuZhi2, Unigene70505\_JingChaoZuZhi2, Unigene70584\_JingChaoZuZhi2, Unigene75165\_JingChaoZuZhi2, Unigene76008\_JingChaoZuZhi2, Unigene77570\_JingChaoZuZhi2, Unigene78235\_JingChaoZuZhi2, Unigene78957\_JingChaoZuZhi2, Unigene8202\_JingChaoZuZhi2, Unigene82153\_JingChaoZuZhi2, Unigene82219\_JingChaoZuZhi2, Unigene8248\_JingChaoZuZhi2, Unigene84930\_JingChaoZuZhi2, Unigene85615\_JingChaoZuZhi2, Unigene85880\_JingChaoZuZhi2, Unigene86206\_JingChaoZuZhi2, Unigene86252\_JingChaoZuZhi2, Unigene86526\_JingChaoZuZhi2, Unigene90535\_JingChaoZuZhi2, Unigene91527\_JingChaoZuZhi2, Unigene91580\_JingChaoZuZhi2, Unigene91661\_JingChaoZuZhi2, Unigene91705\_JingChaoZuZhi2, Unigene92112\_JingChaoZuZhi2, Unigene93807\_JingChaoZuZhi2, Unigene94276\_JingChaoZuZhi2, Unigene94287\_JingChaoZuZhi2, Unigene94685\_JingChaoZuZhi2, Unigene94749\_JingChaoZuZhi2, Unigene94876\_JingChaoZuZhi2, Unigene95674\_JingChaoZuZhi2, Unigene95994\_JingChaoZuZhi2, Unigene96018\_JingChaoZuZhi2, Unigene96294\_JingChaoZuZhi2 |
| 124 | Galactose metabolism | Unigene10315\_JingChaoZuZhi2, Unigene10451\_JingChaoZuZhi2, Unigene1045\_JingChaoZuZhi2, Unigene11681\_JingChaoZuZhi2, Unigene12419\_JingChaoZuZhi2, Unigene12560\_JingChaoZuZhi2, Unigene12745\_JingChaoZuZhi2, Unigene13280\_JingChaoZuZhi2, Unigene13837\_JingChaoZuZhi2, Unigene15503\_JingChaoZuZhi2, Unigene16107\_JingChaoZuZhi2, Unigene16460\_JingChaoZuZhi2, Unigene16762\_JingChaoZuZhi2, Unigene17235\_JingChaoZuZhi2, Unigene19251\_JingChaoZuZhi2, Unigene19303\_JingChaoZuZhi2, Unigene20658\_JingChaoZuZhi2, Unigene20936\_JingChaoZuZhi2, Unigene20950\_JingChaoZuZhi2, Unigene21122\_JingChaoZuZhi2, Unigene21490\_JingChaoZuZhi2, Unigene21754\_JingChaoZuZhi2, Unigene22474\_JingChaoZuZhi2, Unigene23882\_JingChaoZuZhi2, Unigene2474\_JingChaoZuZhi2, Unigene28256\_JingChaoZuZhi2, Unigene34300\_JingChaoZuZhi2, Unigene35848\_JingChaoZuZhi2, Unigene36714\_JingChaoZuZhi2, Unigene36996\_JingChaoZuZhi2, Unigene42637\_JingChaoZuZhi2, Unigene43293\_JingChaoZuZhi2, Unigene46843\_JingChaoZuZhi2, Unigene48948\_JingChaoZuZhi2, Unigene52057\_JingChaoZuZhi2, Unigene55751\_JingChaoZuZhi2, Unigene59180\_JingChaoZuZhi2, Unigene59735\_JingChaoZuZhi2, Unigene59979\_JingChaoZuZhi2, Unigene60210\_JingChaoZuZhi2, Unigene64434\_JingChaoZuZhi2, Unigene64643\_JingChaoZuZhi2, Unigene65555\_JingChaoZuZhi2, Unigene65632\_JingChaoZuZhi2, Unigene66850\_JingChaoZuZhi2, Unigene68502\_JingChaoZuZhi2, Unigene70140\_JingChaoZuZhi2, Unigene71152\_JingChaoZuZhi2, Unigene71196\_JingChaoZuZhi2, Unigene72924\_JingChaoZuZhi2, Unigene74725\_JingChaoZuZhi2, Unigene75050\_JingChaoZuZhi2, Unigene76128\_JingChaoZuZhi2, Unigene77041\_JingChaoZuZhi2, Unigene77192\_JingChaoZuZhi2, Unigene77374\_JingChaoZuZhi2, Unigene78578\_JingChaoZuZhi2, Unigene79539\_JingChaoZuZhi2, Unigene79613\_JingChaoZuZhi2, Unigene79906\_JingChaoZuZhi2, Unigene80141\_JingChaoZuZhi2, Unigene814\_JingChaoZuZhi2, Unigene83318\_JingChaoZuZhi2, Unigene83440\_JingChaoZuZhi2, Unigene84154\_JingChaoZuZhi2, Unigene85769\_JingChaoZuZhi2, Unigene86120\_JingChaoZuZhi2, Unigene86332\_JingChaoZuZhi2, Unigene88876\_JingChaoZuZhi2, Unigene89026\_JingChaoZuZhi2, Unigene89279\_JingChaoZuZhi2, Unigene90268\_JingChaoZuZhi2, Unigene91430\_JingChaoZuZhi2, Unigene91720\_JingChaoZuZhi2, Unigene91969\_JingChaoZuZhi2, Unigene92418\_JingChaoZuZhi2, Unigene95057\_JingChaoZuZhi2, Unigene95296\_JingChaoZuZhi2, Unigene95593\_JingChaoZuZhi2, Unigene95818\_JingChaoZuZhi2 |
| 125 | Glycine, serine and threonine metabolism | Unigene10384\_JingChaoZuZhi2, Unigene10394\_JingChaoZuZhi2, Unigene11433\_JingChaoZuZhi2, Unigene12055\_JingChaoZuZhi2, Unigene12741\_JingChaoZuZhi2, Unigene14816\_JingChaoZuZhi2, Unigene16542\_JingChaoZuZhi2, Unigene16892\_JingChaoZuZhi2, Unigene17655\_JingChaoZuZhi2, Unigene19858\_JingChaoZuZhi2, Unigene20033\_JingChaoZuZhi2, Unigene20162\_JingChaoZuZhi2, Unigene20742\_JingChaoZuZhi2, Unigene2190\_JingChaoZuZhi2, Unigene22143\_JingChaoZuZhi2, Unigene22601\_JingChaoZuZhi2, Unigene2530\_JingChaoZuZhi2, Unigene26104\_JingChaoZuZhi2, Unigene3273\_JingChaoZuZhi2, Unigene36183\_JingChaoZuZhi2, Unigene3643\_JingChaoZuZhi2, Unigene38105\_JingChaoZuZhi2, Unigene44196\_JingChaoZuZhi2, Unigene45512\_JingChaoZuZhi2, Unigene45770\_JingChaoZuZhi2, Unigene45888\_JingChaoZuZhi2, Unigene47407\_JingChaoZuZhi2, Unigene48070\_JingChaoZuZhi2, Unigene4889\_JingChaoZuZhi2, Unigene49766\_JingChaoZuZhi2, Unigene49963\_JingChaoZuZhi2, Unigene5028\_JingChaoZuZhi2, Unigene55063\_JingChaoZuZhi2, Unigene57967\_JingChaoZuZhi2, Unigene59697\_JingChaoZuZhi2, Unigene63447\_JingChaoZuZhi2, Unigene6783\_JingChaoZuZhi2, Unigene69154\_JingChaoZuZhi2, Unigene69398\_JingChaoZuZhi2, Unigene69978\_JingChaoZuZhi2, Unigene70355\_JingChaoZuZhi2, Unigene70893\_JingChaoZuZhi2, Unigene73913\_JingChaoZuZhi2, Unigene73953\_JingChaoZuZhi2, Unigene75743\_JingChaoZuZhi2, Unigene76424\_JingChaoZuZhi2, Unigene77256\_JingChaoZuZhi2, Unigene77273\_JingChaoZuZhi2, Unigene78752\_JingChaoZuZhi2, Unigene79646\_JingChaoZuZhi2, Unigene80265\_JingChaoZuZhi2, Unigene80622\_JingChaoZuZhi2, Unigene80745\_JingChaoZuZhi2, Unigene81293\_JingChaoZuZhi2, Unigene81346\_JingChaoZuZhi2, Unigene81481\_JingChaoZuZhi2, Unigene82671\_JingChaoZuZhi2, Unigene83277\_JingChaoZuZhi2, Unigene83939\_JingChaoZuZhi2, Unigene843\_JingChaoZuZhi2, Unigene84417\_JingChaoZuZhi2, Unigene84539\_JingChaoZuZhi2, Unigene84910\_JingChaoZuZhi2, Unigene85477\_JingChaoZuZhi2, Unigene87054\_JingChaoZuZhi2, Unigene88890\_JingChaoZuZhi2, Unigene88932\_JingChaoZuZhi2, Unigene88980\_JingChaoZuZhi2, Unigene89186\_JingChaoZuZhi2, Unigene90392\_JingChaoZuZhi2, Unigene92024\_JingChaoZuZhi2, Unigene92027\_JingChaoZuZhi2, Unigene92442\_JingChaoZuZhi2, Unigene92462\_JingChaoZuZhi2, Unigene94013\_JingChaoZuZhi2, Unigene9521\_JingChaoZuZhi2, Unigene95480\_JingChaoZuZhi2, Unigene95481\_JingChaoZuZhi2, Unigene96021\_JingChaoZuZhi2 |
| 126 | Fructose and mannose metabolism | Unigene1045\_JingChaoZuZhi2, Unigene12419\_JingChaoZuZhi2, Unigene12745\_JingChaoZuZhi2, Unigene13103\_JingChaoZuZhi2, Unigene13743\_JingChaoZuZhi2, Unigene14132\_JingChaoZuZhi2, Unigene16107\_JingChaoZuZhi2, Unigene16762\_JingChaoZuZhi2, Unigene18114\_JingChaoZuZhi2, Unigene18510\_JingChaoZuZhi2, Unigene1922\_JingChaoZuZhi2, Unigene19751\_JingChaoZuZhi2, Unigene20066\_JingChaoZuZhi2, Unigene20862\_JingChaoZuZhi2, Unigene20936\_JingChaoZuZhi2, Unigene21949\_JingChaoZuZhi2, Unigene22492\_JingChaoZuZhi2, Unigene2474\_JingChaoZuZhi2, Unigene2480\_JingChaoZuZhi2, Unigene41513\_JingChaoZuZhi2, Unigene41663\_JingChaoZuZhi2, Unigene42670\_JingChaoZuZhi2, Unigene42887\_JingChaoZuZhi2, Unigene44590\_JingChaoZuZhi2, Unigene46223\_JingChaoZuZhi2, Unigene46843\_JingChaoZuZhi2, Unigene48736\_JingChaoZuZhi2, Unigene48948\_JingChaoZuZhi2, Unigene50456\_JingChaoZuZhi2, Unigene50485\_JingChaoZuZhi2, Unigene51906\_JingChaoZuZhi2, Unigene53240\_JingChaoZuZhi2, Unigene57410\_JingChaoZuZhi2, Unigene58141\_JingChaoZuZhi2, Unigene59735\_JingChaoZuZhi2, Unigene59764\_JingChaoZuZhi2, Unigene59979\_JingChaoZuZhi2, Unigene61836\_JingChaoZuZhi2, Unigene65498\_JingChaoZuZhi2, Unigene69255\_JingChaoZuZhi2, Unigene69571\_JingChaoZuZhi2, Unigene70140\_JingChaoZuZhi2, Unigene70488\_JingChaoZuZhi2, Unigene73037\_JingChaoZuZhi2, Unigene75125\_JingChaoZuZhi2, Unigene75891\_JingChaoZuZhi2, Unigene76824\_JingChaoZuZhi2, Unigene77374\_JingChaoZuZhi2, Unigene77528\_JingChaoZuZhi2, Unigene79539\_JingChaoZuZhi2, Unigene80141\_JingChaoZuZhi2, Unigene80364\_JingChaoZuZhi2, Unigene82744\_JingChaoZuZhi2, Unigene83440\_JingChaoZuZhi2, Unigene84345\_JingChaoZuZhi2, Unigene85322\_JingChaoZuZhi2, Unigene86585\_JingChaoZuZhi2, Unigene87776\_JingChaoZuZhi2, Unigene881\_JingChaoZuZhi2, Unigene89607\_JingChaoZuZhi2, Unigene89723\_JingChaoZuZhi2, Unigene90268\_JingChaoZuZhi2, Unigene90317\_JingChaoZuZhi2, Unigene90376\_JingChaoZuZhi2, Unigene90474\_JingChaoZuZhi2, Unigene90557\_JingChaoZuZhi2, Unigene90638\_JingChaoZuZhi2, Unigene90875\_JingChaoZuZhi2, Unigene9127\_JingChaoZuZhi2, Unigene91845\_JingChaoZuZhi2, Unigene92035\_JingChaoZuZhi2, Unigene92757\_JingChaoZuZhi2, Unigene94565\_JingChaoZuZhi2, Unigene94595\_JingChaoZuZhi2, Unigene95350\_JingChaoZuZhi2, Unigene95818\_JingChaoZuZhi2, Unigene95868\_JingChaoZuZhi2, Unigene95888\_JingChaoZuZhi2, Unigene96220\_JingChaoZuZhi2 |
| 127 | Alanine, aspartate and glutamate metabolism | Unigene11496\_JingChaoZuZhi2, Unigene12055\_JingChaoZuZhi2, Unigene12380\_JingChaoZuZhi2, Unigene12404\_JingChaoZuZhi2, Unigene14630\_JingChaoZuZhi2, Unigene15992\_JingChaoZuZhi2, Unigene18788\_JingChaoZuZhi2, Unigene19858\_JingChaoZuZhi2, Unigene20033\_JingChaoZuZhi2, Unigene20163\_JingChaoZuZhi2, Unigene20270\_JingChaoZuZhi2, Unigene20559\_JingChaoZuZhi2, Unigene20597\_JingChaoZuZhi2, Unigene20986\_JingChaoZuZhi2, Unigene21428\_JingChaoZuZhi2, Unigene22252\_JingChaoZuZhi2, Unigene22780\_JingChaoZuZhi2, Unigene25903\_JingChaoZuZhi2, Unigene26104\_JingChaoZuZhi2, Unigene30780\_JingChaoZuZhi2, Unigene3098\_JingChaoZuZhi2, Unigene31402\_JingChaoZuZhi2, Unigene31696\_JingChaoZuZhi2, Unigene35450\_JingChaoZuZhi2, Unigene3852\_JingChaoZuZhi2, Unigene39505\_JingChaoZuZhi2, Unigene42036\_JingChaoZuZhi2, Unigene45770\_JingChaoZuZhi2, Unigene46280\_JingChaoZuZhi2, Unigene47206\_JingChaoZuZhi2, Unigene47407\_JingChaoZuZhi2, Unigene49770\_JingChaoZuZhi2, Unigene50259\_JingChaoZuZhi2, Unigene52339\_JingChaoZuZhi2, Unigene52911\_JingChaoZuZhi2, Unigene54075\_JingChaoZuZhi2, Unigene55906\_JingChaoZuZhi2, Unigene5944\_JingChaoZuZhi2, Unigene69271\_JingChaoZuZhi2, Unigene69536\_JingChaoZuZhi2, Unigene70659\_JingChaoZuZhi2, Unigene70672\_JingChaoZuZhi2, Unigene7110\_JingChaoZuZhi2, Unigene71728\_JingChaoZuZhi2, Unigene72849\_JingChaoZuZhi2, Unigene73740\_JingChaoZuZhi2, Unigene74062\_JingChaoZuZhi2, Unigene75743\_JingChaoZuZhi2, Unigene76438\_JingChaoZuZhi2, Unigene76651\_JingChaoZuZhi2, Unigene80745\_JingChaoZuZhi2, Unigene8235\_JingChaoZuZhi2, Unigene82594\_JingChaoZuZhi2, Unigene82868\_JingChaoZuZhi2, Unigene83277\_JingChaoZuZhi2, Unigene83716\_JingChaoZuZhi2, Unigene84416\_JingChaoZuZhi2, Unigene84851\_JingChaoZuZhi2, Unigene85107\_JingChaoZuZhi2, Unigene85428\_JingChaoZuZhi2, Unigene88157\_JingChaoZuZhi2, Unigene88890\_JingChaoZuZhi2, Unigene89496\_JingChaoZuZhi2, Unigene89813\_JingChaoZuZhi2, Unigene90147\_JingChaoZuZhi2, Unigene90961\_JingChaoZuZhi2, Unigene91329\_JingChaoZuZhi2, Unigene92024\_JingChaoZuZhi2, Unigene93427\_JingChaoZuZhi2, Unigene93486\_JingChaoZuZhi2, Unigene93519\_JingChaoZuZhi2, Unigene94013\_JingChaoZuZhi2, Unigene94393\_JingChaoZuZhi2, Unigene95541\_JingChaoZuZhi2, Unigene95658\_JingChaoZuZhi2, Unigene95845\_JingChaoZuZhi2, Unigene95958\_JingChaoZuZhi2 |
| 128 | Tryptophan metabolism | Unigene11206\_JingChaoZuZhi2, Unigene11312\_JingChaoZuZhi2, Unigene11491\_JingChaoZuZhi2, Unigene12354\_JingChaoZuZhi2, Unigene12758\_JingChaoZuZhi2, Unigene13212\_JingChaoZuZhi2, Unigene16352\_JingChaoZuZhi2, Unigene16864\_JingChaoZuZhi2, Unigene17995\_JingChaoZuZhi2, Unigene18179\_JingChaoZuZhi2, Unigene18358\_JingChaoZuZhi2, Unigene18409\_JingChaoZuZhi2, Unigene18553\_JingChaoZuZhi2, Unigene21049\_JingChaoZuZhi2, Unigene21176\_JingChaoZuZhi2, Unigene21821\_JingChaoZuZhi2, Unigene2235\_JingChaoZuZhi2, Unigene22793\_JingChaoZuZhi2, Unigene23105\_JingChaoZuZhi2, Unigene23475\_JingChaoZuZhi2, Unigene2369\_JingChaoZuZhi2, Unigene27219\_JingChaoZuZhi2, Unigene35136\_JingChaoZuZhi2, Unigene37825\_JingChaoZuZhi2, Unigene42806\_JingChaoZuZhi2, Unigene47722\_JingChaoZuZhi2, Unigene48173\_JingChaoZuZhi2, Unigene50371\_JingChaoZuZhi2, Unigene5068\_JingChaoZuZhi2, Unigene51532\_JingChaoZuZhi2, Unigene52598\_JingChaoZuZhi2, Unigene5449\_JingChaoZuZhi2, Unigene55983\_JingChaoZuZhi2, Unigene58763\_JingChaoZuZhi2, Unigene61664\_JingChaoZuZhi2, Unigene63649\_JingChaoZuZhi2, Unigene63715\_JingChaoZuZhi2, Unigene64903\_JingChaoZuZhi2, Unigene66337\_JingChaoZuZhi2, Unigene67613\_JingChaoZuZhi2, Unigene72476\_JingChaoZuZhi2, Unigene72686\_JingChaoZuZhi2, Unigene75416\_JingChaoZuZhi2, Unigene7964\_JingChaoZuZhi2, Unigene80881\_JingChaoZuZhi2, Unigene82129\_JingChaoZuZhi2, Unigene83634\_JingChaoZuZhi2, Unigene84063\_JingChaoZuZhi2, Unigene84343\_JingChaoZuZhi2, Unigene8454\_JingChaoZuZhi2, Unigene84851\_JingChaoZuZhi2, Unigene85224\_JingChaoZuZhi2, Unigene87044\_JingChaoZuZhi2, Unigene87060\_JingChaoZuZhi2, Unigene87485\_JingChaoZuZhi2, Unigene89144\_JingChaoZuZhi2, Unigene90127\_JingChaoZuZhi2, Unigene90838\_JingChaoZuZhi2, Unigene90935\_JingChaoZuZhi2, Unigene91700\_JingChaoZuZhi2, Unigene92517\_JingChaoZuZhi2, Unigene93334\_JingChaoZuZhi2, Unigene93681\_JingChaoZuZhi2, Unigene94726\_JingChaoZuZhi2, Unigene94826\_JingChaoZuZhi2, Unigene94837\_JingChaoZuZhi2, Unigene94902\_JingChaoZuZhi2, Unigene95191\_JingChaoZuZhi2, Unigene95552\_JingChaoZuZhi2, Unigene95695\_JingChaoZuZhi2, Unigene9580\_JingChaoZuZhi2, Unigene95846\_JingChaoZuZhi2, Unigene95941\_JingChaoZuZhi2, Unigene95959\_JingChaoZuZhi2, Unigene95985\_JingChaoZuZhi2, Unigene96031\_JingChaoZuZhi2 |
| 129 | Proteasome | Unigene1007\_JingChaoZuZhi2, Unigene1148\_JingChaoZuZhi2, Unigene1299\_JingChaoZuZhi2, Unigene15045\_JingChaoZuZhi2, Unigene17351\_JingChaoZuZhi2, Unigene28190\_JingChaoZuZhi2, Unigene33097\_JingChaoZuZhi2, Unigene34211\_JingChaoZuZhi2, Unigene34848\_JingChaoZuZhi2, Unigene36640\_JingChaoZuZhi2, Unigene45755\_JingChaoZuZhi2, Unigene46356\_JingChaoZuZhi2, Unigene46986\_JingChaoZuZhi2, Unigene51663\_JingChaoZuZhi2, Unigene51918\_JingChaoZuZhi2, Unigene54328\_JingChaoZuZhi2, Unigene55541\_JingChaoZuZhi2, Unigene57042\_JingChaoZuZhi2, Unigene69101\_JingChaoZuZhi2, Unigene69994\_JingChaoZuZhi2, Unigene7136\_JingChaoZuZhi2, Unigene74147\_JingChaoZuZhi2, Unigene74641\_JingChaoZuZhi2, Unigene75084\_JingChaoZuZhi2, Unigene77626\_JingChaoZuZhi2, Unigene77687\_JingChaoZuZhi2, Unigene78626\_JingChaoZuZhi2, Unigene78698\_JingChaoZuZhi2, Unigene79905\_JingChaoZuZhi2, Unigene81639\_JingChaoZuZhi2, Unigene82362\_JingChaoZuZhi2, Unigene82501\_JingChaoZuZhi2, Unigene83046\_JingChaoZuZhi2, Unigene83361\_JingChaoZuZhi2, Unigene85624\_JingChaoZuZhi2, Unigene88444\_JingChaoZuZhi2, Unigene88539\_JingChaoZuZhi2, Unigene88940\_JingChaoZuZhi2, Unigene88952\_JingChaoZuZhi2, Unigene89078\_JingChaoZuZhi2, Unigene90646\_JingChaoZuZhi2, Unigene90948\_JingChaoZuZhi2, Unigene91197\_JingChaoZuZhi2, Unigene91655\_JingChaoZuZhi2, Unigene91967\_JingChaoZuZhi2, Unigene91972\_JingChaoZuZhi2, Unigene92213\_JingChaoZuZhi2, Unigene92237\_JingChaoZuZhi2, Unigene92244\_JingChaoZuZhi2, Unigene92342\_JingChaoZuZhi2, Unigene92479\_JingChaoZuZhi2, Unigene92549\_JingChaoZuZhi2, Unigene92592\_JingChaoZuZhi2, Unigene92705\_JingChaoZuZhi2, Unigene93301\_JingChaoZuZhi2, Unigene93567\_JingChaoZuZhi2, Unigene93872\_JingChaoZuZhi2, Unigene94072\_JingChaoZuZhi2, Unigene94143\_JingChaoZuZhi2, Unigene94221\_JingChaoZuZhi2, Unigene94375\_JingChaoZuZhi2, Unigene94415\_JingChaoZuZhi2, Unigene94477\_JingChaoZuZhi2, Unigene94493\_JingChaoZuZhi2, Unigene94524\_JingChaoZuZhi2, Unigene94818\_JingChaoZuZhi2, Unigene95138\_JingChaoZuZhi2, Unigene95206\_JingChaoZuZhi2, Unigene95273\_JingChaoZuZhi2, Unigene95452\_JingChaoZuZhi2, Unigene95548\_JingChaoZuZhi2, Unigene95827\_JingChaoZuZhi2, Unigene95832\_JingChaoZuZhi2, Unigene95839\_JingChaoZuZhi2, Unigene96224\_JingChaoZuZhi2, Unigene96291\_JingChaoZuZhi2 |
| 130 | Non-small cell lung cancer | Unigene10318\_JingChaoZuZhi2, Unigene10422\_JingChaoZuZhi2, Unigene11961\_JingChaoZuZhi2, Unigene12202\_JingChaoZuZhi2, Unigene12271\_JingChaoZuZhi2, Unigene12420\_JingChaoZuZhi2, Unigene14054\_JingChaoZuZhi2, Unigene14534\_JingChaoZuZhi2, Unigene14912\_JingChaoZuZhi2, Unigene15228\_JingChaoZuZhi2, Unigene17247\_JingChaoZuZhi2, Unigene17467\_JingChaoZuZhi2, Unigene18464\_JingChaoZuZhi2, Unigene18672\_JingChaoZuZhi2, Unigene18868\_JingChaoZuZhi2, Unigene21295\_JingChaoZuZhi2, Unigene21513\_JingChaoZuZhi2, Unigene21578\_JingChaoZuZhi2, Unigene22779\_JingChaoZuZhi2, Unigene2456\_JingChaoZuZhi2, Unigene26730\_JingChaoZuZhi2, Unigene2790\_JingChaoZuZhi2, Unigene28978\_JingChaoZuZhi2, Unigene2960\_JingChaoZuZhi2, Unigene33182\_JingChaoZuZhi2, Unigene37495\_JingChaoZuZhi2, Unigene3931\_JingChaoZuZhi2, Unigene40020\_JingChaoZuZhi2, Unigene4186\_JingChaoZuZhi2, Unigene42981\_JingChaoZuZhi2, Unigene4838\_JingChaoZuZhi2, Unigene48751\_JingChaoZuZhi2, Unigene49798\_JingChaoZuZhi2, Unigene51285\_JingChaoZuZhi2, Unigene5364\_JingChaoZuZhi2, Unigene54470\_JingChaoZuZhi2, Unigene56642\_JingChaoZuZhi2, Unigene57488\_JingChaoZuZhi2, Unigene5758\_JingChaoZuZhi2, Unigene6028\_JingChaoZuZhi2, Unigene62786\_JingChaoZuZhi2, Unigene64164\_JingChaoZuZhi2, Unigene64830\_JingChaoZuZhi2, Unigene6493\_JingChaoZuZhi2, Unigene67655\_JingChaoZuZhi2, Unigene67793\_JingChaoZuZhi2, Unigene67983\_JingChaoZuZhi2, Unigene68283\_JingChaoZuZhi2, Unigene70549\_JingChaoZuZhi2, Unigene70723\_JingChaoZuZhi2, Unigene72521\_JingChaoZuZhi2, Unigene78574\_JingChaoZuZhi2, Unigene8191\_JingChaoZuZhi2, Unigene84930\_JingChaoZuZhi2, Unigene85574\_JingChaoZuZhi2, Unigene86206\_JingChaoZuZhi2, Unigene86278\_JingChaoZuZhi2, Unigene86456\_JingChaoZuZhi2, Unigene86526\_JingChaoZuZhi2, Unigene87332\_JingChaoZuZhi2, Unigene876\_JingChaoZuZhi2, Unigene88343\_JingChaoZuZhi2, Unigene89502\_JingChaoZuZhi2, Unigene91612\_JingChaoZuZhi2, Unigene91958\_JingChaoZuZhi2, Unigene92112\_JingChaoZuZhi2, Unigene93807\_JingChaoZuZhi2, Unigene94149\_JingChaoZuZhi2, Unigene94456\_JingChaoZuZhi2, Unigene9461\_JingChaoZuZhi2, Unigene94685\_JingChaoZuZhi2, Unigene95456\_JingChaoZuZhi2, Unigene95677\_JingChaoZuZhi2, Unigene96294\_JingChaoZuZhi2, Unigene9902\_JingChaoZuZhi2 |
| 131 | Cysteine and methionine metabolism | Unigene10042\_JingChaoZuZhi2, Unigene10578\_JingChaoZuZhi2, Unigene10895\_JingChaoZuZhi2, Unigene12264\_JingChaoZuZhi2, Unigene1244\_JingChaoZuZhi2, Unigene12\_JingChaoZuZhi2, Unigene14324\_JingChaoZuZhi2, Unigene14757\_JingChaoZuZhi2, Unigene14786\_JingChaoZuZhi2, Unigene15083\_JingChaoZuZhi2, Unigene16478\_JingChaoZuZhi2, Unigene16660\_JingChaoZuZhi2, Unigene16868\_JingChaoZuZhi2, Unigene17082\_JingChaoZuZhi2, Unigene1728\_JingChaoZuZhi2, Unigene18335\_JingChaoZuZhi2, Unigene18489\_JingChaoZuZhi2, Unigene18740\_JingChaoZuZhi2, Unigene18788\_JingChaoZuZhi2, Unigene18862\_JingChaoZuZhi2, Unigene19226\_JingChaoZuZhi2, Unigene19644\_JingChaoZuZhi2, Unigene20201\_JingChaoZuZhi2, Unigene21092\_JingChaoZuZhi2, Unigene21094\_JingChaoZuZhi2, Unigene21197\_JingChaoZuZhi2, Unigene21318\_JingChaoZuZhi2, Unigene21805\_JingChaoZuZhi2, Unigene22317\_JingChaoZuZhi2, Unigene22601\_JingChaoZuZhi2, Unigene22706\_JingChaoZuZhi2, Unigene2530\_JingChaoZuZhi2, Unigene289\_JingChaoZuZhi2, Unigene3106\_JingChaoZuZhi2, Unigene339\_JingChaoZuZhi2, Unigene38105\_JingChaoZuZhi2, Unigene4428\_JingChaoZuZhi2, Unigene46569\_JingChaoZuZhi2, Unigene4795\_JingChaoZuZhi2, Unigene4889\_JingChaoZuZhi2, Unigene49331\_JingChaoZuZhi2, Unigene52898\_JingChaoZuZhi2, Unigene535\_JingChaoZuZhi2, Unigene57967\_JingChaoZuZhi2, Unigene5938\_JingChaoZuZhi2, Unigene60177\_JingChaoZuZhi2, Unigene6073\_JingChaoZuZhi2, Unigene63447\_JingChaoZuZhi2, Unigene648\_JingChaoZuZhi2, Unigene6908\_JingChaoZuZhi2, Unigene6944\_JingChaoZuZhi2, Unigene6945\_JingChaoZuZhi2, Unigene70659\_JingChaoZuZhi2, Unigene73953\_JingChaoZuZhi2, Unigene76502\_JingChaoZuZhi2, Unigene77256\_JingChaoZuZhi2, Unigene80205\_JingChaoZuZhi2, Unigene84279\_JingChaoZuZhi2, Unigene86213\_JingChaoZuZhi2, Unigene8689\_JingChaoZuZhi2, Unigene86998\_JingChaoZuZhi2, Unigene87966\_JingChaoZuZhi2, Unigene8826\_JingChaoZuZhi2, Unigene89879\_JingChaoZuZhi2, Unigene90556\_JingChaoZuZhi2, Unigene90667\_JingChaoZuZhi2, Unigene93212\_JingChaoZuZhi2, Unigene95481\_JingChaoZuZhi2, Unigene95658\_JingChaoZuZhi2, Unigene96071\_JingChaoZuZhi2, Unigene96133\_JingChaoZuZhi2, Unigene96214\_JingChaoZuZhi2, Unigene96274\_JingChaoZuZhi2 |
| 132 | Malaria | Unigene10286\_JingChaoZuZhi2, Unigene12941\_JingChaoZuZhi2, Unigene1372\_JingChaoZuZhi2, Unigene14109\_JingChaoZuZhi2, Unigene14261\_JingChaoZuZhi2, Unigene14620\_JingChaoZuZhi2, Unigene15236\_JingChaoZuZhi2, Unigene15278\_JingChaoZuZhi2, Unigene16048\_JingChaoZuZhi2, Unigene18107\_JingChaoZuZhi2, Unigene18309\_JingChaoZuZhi2, Unigene18515\_JingChaoZuZhi2, Unigene18652\_JingChaoZuZhi2, Unigene19423\_JingChaoZuZhi2, Unigene19425\_JingChaoZuZhi2, Unigene19597\_JingChaoZuZhi2, Unigene19664\_JingChaoZuZhi2, Unigene19678\_JingChaoZuZhi2, Unigene19847\_JingChaoZuZhi2, Unigene20297\_JingChaoZuZhi2, Unigene20622\_JingChaoZuZhi2, Unigene21222\_JingChaoZuZhi2, Unigene21472\_JingChaoZuZhi2, Unigene21996\_JingChaoZuZhi2, Unigene22763\_JingChaoZuZhi2, Unigene22794\_JingChaoZuZhi2, Unigene27479\_JingChaoZuZhi2, Unigene29201\_JingChaoZuZhi2, Unigene3903\_JingChaoZuZhi2, Unigene43427\_JingChaoZuZhi2, Unigene45069\_JingChaoZuZhi2, Unigene46526\_JingChaoZuZhi2, Unigene48714\_JingChaoZuZhi2, Unigene48843\_JingChaoZuZhi2, Unigene50405\_JingChaoZuZhi2, Unigene51994\_JingChaoZuZhi2, Unigene55738\_JingChaoZuZhi2, Unigene5673\_JingChaoZuZhi2, Unigene57238\_JingChaoZuZhi2, Unigene60370\_JingChaoZuZhi2, Unigene60776\_JingChaoZuZhi2, Unigene60975\_JingChaoZuZhi2, Unigene61033\_JingChaoZuZhi2, Unigene62979\_JingChaoZuZhi2, Unigene67304\_JingChaoZuZhi2, Unigene6755\_JingChaoZuZhi2, Unigene68113\_JingChaoZuZhi2, Unigene69071\_JingChaoZuZhi2, Unigene69817\_JingChaoZuZhi2, Unigene7068\_JingChaoZuZhi2, Unigene70920\_JingChaoZuZhi2, Unigene72170\_JingChaoZuZhi2, Unigene72613\_JingChaoZuZhi2, Unigene76400\_JingChaoZuZhi2, Unigene76415\_JingChaoZuZhi2, Unigene77904\_JingChaoZuZhi2, Unigene82309\_JingChaoZuZhi2, Unigene83398\_JingChaoZuZhi2, Unigene84080\_JingChaoZuZhi2, Unigene84343\_JingChaoZuZhi2, Unigene85175\_JingChaoZuZhi2, Unigene85529\_JingChaoZuZhi2, Unigene85880\_JingChaoZuZhi2, Unigene87026\_JingChaoZuZhi2, Unigene8819\_JingChaoZuZhi2, Unigene88428\_JingChaoZuZhi2, Unigene89406\_JingChaoZuZhi2, Unigene91043\_JingChaoZuZhi2, Unigene9301\_JingChaoZuZhi2, Unigene93178\_JingChaoZuZhi2, Unigene95293\_JingChaoZuZhi2, Unigene96084\_JingChaoZuZhi2 |
| 133 | MAPK signaling pathway - fly | Unigene10422\_JingChaoZuZhi2, Unigene12420\_JingChaoZuZhi2, Unigene12813\_JingChaoZuZhi2, Unigene12840\_JingChaoZuZhi2, Unigene13354\_JingChaoZuZhi2, Unigene13794\_JingChaoZuZhi2, Unigene14145\_JingChaoZuZhi2, Unigene14738\_JingChaoZuZhi2, Unigene15429\_JingChaoZuZhi2, Unigene1554\_JingChaoZuZhi2, Unigene18405\_JingChaoZuZhi2, Unigene18609\_JingChaoZuZhi2, Unigene21513\_JingChaoZuZhi2, Unigene2215\_JingChaoZuZhi2, Unigene2308\_JingChaoZuZhi2, Unigene26604\_JingChaoZuZhi2, Unigene2790\_JingChaoZuZhi2, Unigene35809\_JingChaoZuZhi2, Unigene40410\_JingChaoZuZhi2, Unigene4186\_JingChaoZuZhi2, Unigene4223\_JingChaoZuZhi2, Unigene44927\_JingChaoZuZhi2, Unigene46723\_JingChaoZuZhi2, Unigene48357\_JingChaoZuZhi2, Unigene4838\_JingChaoZuZhi2, Unigene49798\_JingChaoZuZhi2, Unigene49823\_JingChaoZuZhi2, Unigene54470\_JingChaoZuZhi2, Unigene54475\_JingChaoZuZhi2, Unigene5638\_JingChaoZuZhi2, Unigene59849\_JingChaoZuZhi2, Unigene6028\_JingChaoZuZhi2, Unigene60762\_JingChaoZuZhi2, Unigene62786\_JingChaoZuZhi2, Unigene62851\_JingChaoZuZhi2, Unigene6493\_JingChaoZuZhi2, Unigene65524\_JingChaoZuZhi2, Unigene66888\_JingChaoZuZhi2, Unigene67523\_JingChaoZuZhi2, Unigene67793\_JingChaoZuZhi2, Unigene70100\_JingChaoZuZhi2, Unigene70549\_JingChaoZuZhi2, Unigene72521\_JingChaoZuZhi2, Unigene75317\_JingChaoZuZhi2, Unigene76400\_JingChaoZuZhi2, Unigene78574\_JingChaoZuZhi2, Unigene79830\_JingChaoZuZhi2, Unigene80980\_JingChaoZuZhi2, Unigene8191\_JingChaoZuZhi2, Unigene82606\_JingChaoZuZhi2, Unigene8388\_JingChaoZuZhi2, Unigene84220\_JingChaoZuZhi2, Unigene84862\_JingChaoZuZhi2, Unigene84911\_JingChaoZuZhi2, Unigene85574\_JingChaoZuZhi2, Unigene86115\_JingChaoZuZhi2, Unigene87213\_JingChaoZuZhi2, Unigene876\_JingChaoZuZhi2, Unigene89709\_JingChaoZuZhi2, Unigene91284\_JingChaoZuZhi2, Unigene91612\_JingChaoZuZhi2, Unigene91958\_JingChaoZuZhi2, Unigene92203\_JingChaoZuZhi2, Unigene94149\_JingChaoZuZhi2, Unigene94456\_JingChaoZuZhi2, Unigene95378\_JingChaoZuZhi2, Unigene95456\_JingChaoZuZhi2, Unigene95620\_JingChaoZuZhi2, Unigene95677\_JingChaoZuZhi2, Unigene96044\_JingChaoZuZhi2, Unigene9713\_JingChaoZuZhi2, Unigene9902\_JingChaoZuZhi2 |
| 134 | Circadian rhythm - mammal | Unigene13167\_JingChaoZuZhi2, Unigene13172\_JingChaoZuZhi2, Unigene14170\_JingChaoZuZhi2, Unigene16289\_JingChaoZuZhi2, Unigene16407\_JingChaoZuZhi2, Unigene17868\_JingChaoZuZhi2, Unigene1790\_JingChaoZuZhi2, Unigene18027\_JingChaoZuZhi2, Unigene18090\_JingChaoZuZhi2, Unigene18812\_JingChaoZuZhi2, Unigene19137\_JingChaoZuZhi2, Unigene20239\_JingChaoZuZhi2, Unigene20487\_JingChaoZuZhi2, Unigene20489\_JingChaoZuZhi2, Unigene21036\_JingChaoZuZhi2, Unigene22008\_JingChaoZuZhi2, Unigene22708\_JingChaoZuZhi2, Unigene22787\_JingChaoZuZhi2, Unigene24668\_JingChaoZuZhi2, Unigene27057\_JingChaoZuZhi2, Unigene28711\_JingChaoZuZhi2, Unigene32049\_JingChaoZuZhi2, Unigene3307\_JingChaoZuZhi2, Unigene33281\_JingChaoZuZhi2, Unigene34185\_JingChaoZuZhi2, Unigene34624\_JingChaoZuZhi2, Unigene35730\_JingChaoZuZhi2, Unigene41670\_JingChaoZuZhi2, Unigene42310\_JingChaoZuZhi2, Unigene4625\_JingChaoZuZhi2, Unigene47548\_JingChaoZuZhi2, Unigene48813\_JingChaoZuZhi2, Unigene48986\_JingChaoZuZhi2, Unigene5102\_JingChaoZuZhi2, Unigene51034\_JingChaoZuZhi2, Unigene53160\_JingChaoZuZhi2, Unigene5671\_JingChaoZuZhi2, Unigene5815\_JingChaoZuZhi2, Unigene59068\_JingChaoZuZhi2, Unigene59127\_JingChaoZuZhi2, Unigene61372\_JingChaoZuZhi2, Unigene63247\_JingChaoZuZhi2, Unigene63604\_JingChaoZuZhi2, Unigene63885\_JingChaoZuZhi2, Unigene64880\_JingChaoZuZhi2, Unigene6546\_JingChaoZuZhi2, Unigene67959\_JingChaoZuZhi2, Unigene68206\_JingChaoZuZhi2, Unigene70125\_JingChaoZuZhi2, Unigene72534\_JingChaoZuZhi2, Unigene74056\_JingChaoZuZhi2, Unigene74498\_JingChaoZuZhi2, Unigene74609\_JingChaoZuZhi2, Unigene75700\_JingChaoZuZhi2, Unigene76614\_JingChaoZuZhi2, Unigene76639\_JingChaoZuZhi2, Unigene77305\_JingChaoZuZhi2, Unigene77359\_JingChaoZuZhi2, Unigene80385\_JingChaoZuZhi2, Unigene80927\_JingChaoZuZhi2, Unigene82688\_JingChaoZuZhi2, Unigene83736\_JingChaoZuZhi2, Unigene85510\_JingChaoZuZhi2, Unigene88755\_JingChaoZuZhi2, Unigene91904\_JingChaoZuZhi2, Unigene93288\_JingChaoZuZhi2, Unigene93479\_JingChaoZuZhi2, Unigene94071\_JingChaoZuZhi2, Unigene94827\_JingChaoZuZhi2 |
| 135 | Butanoate metabolism | Unigene11312\_JingChaoZuZhi2, Unigene14132\_JingChaoZuZhi2, Unigene15755\_JingChaoZuZhi2, Unigene15856\_JingChaoZuZhi2, Unigene16352\_JingChaoZuZhi2, Unigene17217\_JingChaoZuZhi2, Unigene17995\_JingChaoZuZhi2, Unigene18114\_JingChaoZuZhi2, Unigene18179\_JingChaoZuZhi2, Unigene18251\_JingChaoZuZhi2, Unigene18409\_JingChaoZuZhi2, Unigene18510\_JingChaoZuZhi2, Unigene18553\_JingChaoZuZhi2, Unigene21099\_JingChaoZuZhi2, Unigene21821\_JingChaoZuZhi2, Unigene22492\_JingChaoZuZhi2, Unigene22780\_JingChaoZuZhi2, Unigene22793\_JingChaoZuZhi2, Unigene25361\_JingChaoZuZhi2, Unigene27219\_JingChaoZuZhi2, Unigene29455\_JingChaoZuZhi2, Unigene35450\_JingChaoZuZhi2, Unigene38871\_JingChaoZuZhi2, Unigene44775\_JingChaoZuZhi2, Unigene4686\_JingChaoZuZhi2, Unigene47206\_JingChaoZuZhi2, Unigene490\_JingChaoZuZhi2, Unigene50371\_JingChaoZuZhi2, Unigene5057\_JingChaoZuZhi2, Unigene5068\_JingChaoZuZhi2, Unigene51532\_JingChaoZuZhi2, Unigene55906\_JingChaoZuZhi2, Unigene5663\_JingChaoZuZhi2, Unigene57635\_JingChaoZuZhi2, Unigene58763\_JingChaoZuZhi2, Unigene59449\_JingChaoZuZhi2, Unigene61664\_JingChaoZuZhi2, Unigene63715\_JingChaoZuZhi2, Unigene64673\_JingChaoZuZhi2, Unigene64903\_JingChaoZuZhi2, Unigene66337\_JingChaoZuZhi2, Unigene70488\_JingChaoZuZhi2, Unigene70703\_JingChaoZuZhi2, Unigene72849\_JingChaoZuZhi2, Unigene72880\_JingChaoZuZhi2, Unigene75891\_JingChaoZuZhi2, Unigene7911\_JingChaoZuZhi2, Unigene79637\_JingChaoZuZhi2, Unigene82594\_JingChaoZuZhi2, Unigene84343\_JingChaoZuZhi2, Unigene8442\_JingChaoZuZhi2, Unigene8454\_JingChaoZuZhi2, Unigene84851\_JingChaoZuZhi2, Unigene85224\_JingChaoZuZhi2, Unigene87485\_JingChaoZuZhi2, Unigene87611\_JingChaoZuZhi2, Unigene87856\_JingChaoZuZhi2, Unigene89941\_JingChaoZuZhi2, Unigene90317\_JingChaoZuZhi2, Unigene90376\_JingChaoZuZhi2, Unigene90817\_JingChaoZuZhi2, Unigene90838\_JingChaoZuZhi2, Unigene92256\_JingChaoZuZhi2, Unigene92757\_JingChaoZuZhi2, Unigene93422\_JingChaoZuZhi2, Unigene93486\_JingChaoZuZhi2, Unigene93681\_JingChaoZuZhi2, Unigene9580\_JingChaoZuZhi2, Unigene96087\_JingChaoZuZhi2 |
| 136 | Acute myeloid leukemia | Unigene10532\_JingChaoZuZhi2, Unigene11961\_JingChaoZuZhi2, Unigene12420\_JingChaoZuZhi2, Unigene12658\_JingChaoZuZhi2, Unigene12823\_JingChaoZuZhi2, Unigene14289\_JingChaoZuZhi2, Unigene14912\_JingChaoZuZhi2, Unigene15228\_JingChaoZuZhi2, Unigene16229\_JingChaoZuZhi2, Unigene17247\_JingChaoZuZhi2, Unigene18672\_JingChaoZuZhi2, Unigene19307\_JingChaoZuZhi2, Unigene19551\_JingChaoZuZhi2, Unigene20157\_JingChaoZuZhi2, Unigene20840\_JingChaoZuZhi2, Unigene20991\_JingChaoZuZhi2, Unigene21010\_JingChaoZuZhi2, Unigene21513\_JingChaoZuZhi2, Unigene21697\_JingChaoZuZhi2, Unigene22402\_JingChaoZuZhi2, Unigene22466\_JingChaoZuZhi2, Unigene2456\_JingChaoZuZhi2, Unigene3931\_JingChaoZuZhi2, Unigene4186\_JingChaoZuZhi2, Unigene4838\_JingChaoZuZhi2, Unigene48751\_JingChaoZuZhi2, Unigene49726\_JingChaoZuZhi2, Unigene54320\_JingChaoZuZhi2, Unigene57488\_JingChaoZuZhi2, Unigene6028\_JingChaoZuZhi2, Unigene6159\_JingChaoZuZhi2, Unigene61911\_JingChaoZuZhi2, Unigene63449\_JingChaoZuZhi2, Unigene64227\_JingChaoZuZhi2, Unigene64650\_JingChaoZuZhi2, Unigene6493\_JingChaoZuZhi2, Unigene66060\_JingChaoZuZhi2, Unigene67793\_JingChaoZuZhi2, Unigene6785\_JingChaoZuZhi2, Unigene70549\_JingChaoZuZhi2, Unigene7549\_JingChaoZuZhi2, Unigene77570\_JingChaoZuZhi2, Unigene8191\_JingChaoZuZhi2, Unigene85172\_JingChaoZuZhi2, Unigene85574\_JingChaoZuZhi2, Unigene86206\_JingChaoZuZhi2, Unigene86526\_JingChaoZuZhi2, Unigene876\_JingChaoZuZhi2, Unigene88082\_JingChaoZuZhi2, Unigene88544\_JingChaoZuZhi2, Unigene89621\_JingChaoZuZhi2, Unigene90353\_JingChaoZuZhi2, Unigene91538\_JingChaoZuZhi2, Unigene91612\_JingChaoZuZhi2, Unigene91958\_JingChaoZuZhi2, Unigene9204\_JingChaoZuZhi2, Unigene92112\_JingChaoZuZhi2, Unigene93409\_JingChaoZuZhi2, Unigene93807\_JingChaoZuZhi2, Unigene94149\_JingChaoZuZhi2, Unigene94377\_JingChaoZuZhi2, Unigene94456\_JingChaoZuZhi2, Unigene94685\_JingChaoZuZhi2, Unigene95456\_JingChaoZuZhi2, Unigene95921\_JingChaoZuZhi2, Unigene96294\_JingChaoZuZhi2, Unigene9902\_JingChaoZuZhi2 |
| 137 | RIG-I-like receptor signaling pathway | Unigene11732\_JingChaoZuZhi2, Unigene11951\_JingChaoZuZhi2, Unigene1301\_JingChaoZuZhi2, Unigene13210\_JingChaoZuZhi2, Unigene14635\_JingChaoZuZhi2, Unigene14854\_JingChaoZuZhi2, Unigene17251\_JingChaoZuZhi2, Unigene20120\_JingChaoZuZhi2, Unigene20157\_JingChaoZuZhi2, Unigene20991\_JingChaoZuZhi2, Unigene21636\_JingChaoZuZhi2, Unigene21697\_JingChaoZuZhi2, Unigene22126\_JingChaoZuZhi2, Unigene22220\_JingChaoZuZhi2, Unigene22466\_JingChaoZuZhi2, Unigene22482\_JingChaoZuZhi2, Unigene2628\_JingChaoZuZhi2, Unigene4294\_JingChaoZuZhi2, Unigene45626\_JingChaoZuZhi2, Unigene4853\_JingChaoZuZhi2, Unigene50201\_JingChaoZuZhi2, Unigene5099\_JingChaoZuZhi2, Unigene54320\_JingChaoZuZhi2, Unigene59471\_JingChaoZuZhi2, Unigene59885\_JingChaoZuZhi2, Unigene60715\_JingChaoZuZhi2, Unigene61044\_JingChaoZuZhi2, Unigene6459\_JingChaoZuZhi2, Unigene65117\_JingChaoZuZhi2, Unigene69069\_JingChaoZuZhi2, Unigene70063\_JingChaoZuZhi2, Unigene72033\_JingChaoZuZhi2, Unigene72460\_JingChaoZuZhi2, Unigene72708\_JingChaoZuZhi2, Unigene7459\_JingChaoZuZhi2, Unigene77172\_JingChaoZuZhi2, Unigene77570\_JingChaoZuZhi2, Unigene77592\_JingChaoZuZhi2, Unigene78885\_JingChaoZuZhi2, Unigene80583\_JingChaoZuZhi2, Unigene80762\_JingChaoZuZhi2, Unigene81156\_JingChaoZuZhi2, Unigene81592\_JingChaoZuZhi2, Unigene82646\_JingChaoZuZhi2, Unigene82971\_JingChaoZuZhi2, Unigene85019\_JingChaoZuZhi2, Unigene85140\_JingChaoZuZhi2, Unigene85958\_JingChaoZuZhi2, Unigene86252\_JingChaoZuZhi2, Unigene86485\_JingChaoZuZhi2, Unigene87771\_JingChaoZuZhi2, Unigene87774\_JingChaoZuZhi2, Unigene88272\_JingChaoZuZhi2, Unigene88451\_JingChaoZuZhi2, Unigene88942\_JingChaoZuZhi2, Unigene89535\_JingChaoZuZhi2, Unigene91648\_JingChaoZuZhi2, Unigene92138\_JingChaoZuZhi2, Unigene93911\_JingChaoZuZhi2, Unigene93912\_JingChaoZuZhi2, Unigene94032\_JingChaoZuZhi2, Unigene94619\_JingChaoZuZhi2, Unigene94689\_JingChaoZuZhi2, Unigene9517\_JingChaoZuZhi2, Unigene9599\_JingChaoZuZhi2, Unigene9711\_JingChaoZuZhi2, Unigene9998\_JingChaoZuZhi2 |
| 138 | Aldosterone-regulated sodium reabsorption | Unigene10318\_JingChaoZuZhi2, Unigene11961\_JingChaoZuZhi2, Unigene12577\_JingChaoZuZhi2, Unigene14912\_JingChaoZuZhi2, Unigene15228\_JingChaoZuZhi2, Unigene16768\_JingChaoZuZhi2, Unigene17247\_JingChaoZuZhi2, Unigene17839\_JingChaoZuZhi2, Unigene18222\_JingChaoZuZhi2, Unigene18672\_JingChaoZuZhi2, Unigene18821\_JingChaoZuZhi2, Unigene19086\_JingChaoZuZhi2, Unigene20107\_JingChaoZuZhi2, Unigene21578\_JingChaoZuZhi2, Unigene21792\_JingChaoZuZhi2, Unigene21866\_JingChaoZuZhi2, Unigene22779\_JingChaoZuZhi2, Unigene2456\_JingChaoZuZhi2, Unigene26730\_JingChaoZuZhi2, Unigene28522\_JingChaoZuZhi2, Unigene2960\_JingChaoZuZhi2, Unigene37495\_JingChaoZuZhi2, Unigene40020\_JingChaoZuZhi2, Unigene42033\_JingChaoZuZhi2, Unigene48751\_JingChaoZuZhi2, Unigene55687\_JingChaoZuZhi2, Unigene56513\_JingChaoZuZhi2, Unigene5674\_JingChaoZuZhi2, Unigene57488\_JingChaoZuZhi2, Unigene5799\_JingChaoZuZhi2, Unigene60180\_JingChaoZuZhi2, Unigene63489\_JingChaoZuZhi2, Unigene6381\_JingChaoZuZhi2, Unigene64164\_JingChaoZuZhi2, Unigene64583\_JingChaoZuZhi2, Unigene64830\_JingChaoZuZhi2, Unigene67983\_JingChaoZuZhi2, Unigene68283\_JingChaoZuZhi2, Unigene70723\_JingChaoZuZhi2, Unigene7182\_JingChaoZuZhi2, Unigene7224\_JingChaoZuZhi2, Unigene76400\_JingChaoZuZhi2, Unigene77255\_JingChaoZuZhi2, Unigene81692\_JingChaoZuZhi2, Unigene8191\_JingChaoZuZhi2, Unigene83588\_JingChaoZuZhi2, Unigene83602\_JingChaoZuZhi2, Unigene84232\_JingChaoZuZhi2, Unigene84340\_JingChaoZuZhi2, Unigene8464\_JingChaoZuZhi2, Unigene86206\_JingChaoZuZhi2, Unigene86278\_JingChaoZuZhi2, Unigene86456\_JingChaoZuZhi2, Unigene86526\_JingChaoZuZhi2, Unigene87332\_JingChaoZuZhi2, Unigene876\_JingChaoZuZhi2, Unigene90209\_JingChaoZuZhi2, Unigene92112\_JingChaoZuZhi2, Unigene92580\_JingChaoZuZhi2, Unigene93807\_JingChaoZuZhi2, Unigene94279\_JingChaoZuZhi2, Unigene94685\_JingChaoZuZhi2, Unigene95677\_JingChaoZuZhi2, Unigene95776\_JingChaoZuZhi2, Unigene95993\_JingChaoZuZhi2, Unigene96206\_JingChaoZuZhi2, Unigene96294\_JingChaoZuZhi2 |
| 139 | Mismatch repair | Unigene10802\_JingChaoZuZhi2, Unigene10986\_JingChaoZuZhi2, Unigene11190\_JingChaoZuZhi2, Unigene12617\_JingChaoZuZhi2, Unigene13861\_JingChaoZuZhi2, Unigene14592\_JingChaoZuZhi2, Unigene1520\_JingChaoZuZhi2, Unigene16653\_JingChaoZuZhi2, Unigene1695\_JingChaoZuZhi2, Unigene170\_JingChaoZuZhi2, Unigene1738\_JingChaoZuZhi2, Unigene18542\_JingChaoZuZhi2, Unigene18728\_JingChaoZuZhi2, Unigene18862\_JingChaoZuZhi2, Unigene1888\_JingChaoZuZhi2, Unigene1902\_JingChaoZuZhi2, Unigene20803\_JingChaoZuZhi2, Unigene21305\_JingChaoZuZhi2, Unigene21604\_JingChaoZuZhi2, Unigene21673\_JingChaoZuZhi2, Unigene21781\_JingChaoZuZhi2, Unigene22259\_JingChaoZuZhi2, Unigene22304\_JingChaoZuZhi2, Unigene2245\_JingChaoZuZhi2, Unigene22522\_JingChaoZuZhi2, Unigene22578\_JingChaoZuZhi2, Unigene2354\_JingChaoZuZhi2, Unigene36525\_JingChaoZuZhi2, Unigene4155\_JingChaoZuZhi2, Unigene41885\_JingChaoZuZhi2, Unigene460\_JingChaoZuZhi2, Unigene5578\_JingChaoZuZhi2, Unigene5882\_JingChaoZuZhi2, Unigene59011\_JingChaoZuZhi2, Unigene59112\_JingChaoZuZhi2, Unigene5948\_JingChaoZuZhi2, Unigene6073\_JingChaoZuZhi2, Unigene63198\_JingChaoZuZhi2, Unigene64812\_JingChaoZuZhi2, Unigene66933\_JingChaoZuZhi2, Unigene68136\_JingChaoZuZhi2, Unigene6944\_JingChaoZuZhi2, Unigene69517\_JingChaoZuZhi2, Unigene7073\_JingChaoZuZhi2, Unigene71412\_JingChaoZuZhi2, Unigene71424\_JingChaoZuZhi2, Unigene71482\_JingChaoZuZhi2, Unigene71893\_JingChaoZuZhi2, Unigene73870\_JingChaoZuZhi2, Unigene74294\_JingChaoZuZhi2, Unigene74377\_JingChaoZuZhi2, Unigene75930\_JingChaoZuZhi2, Unigene85704\_JingChaoZuZhi2, Unigene87989\_JingChaoZuZhi2, Unigene88770\_JingChaoZuZhi2, Unigene89315\_JingChaoZuZhi2, Unigene89645\_JingChaoZuZhi2, Unigene91096\_JingChaoZuZhi2, Unigene92389\_JingChaoZuZhi2, Unigene92803\_JingChaoZuZhi2, Unigene94583\_JingChaoZuZhi2, Unigene95055\_JingChaoZuZhi2, Unigene95153\_JingChaoZuZhi2, Unigene95352\_JingChaoZuZhi2, Unigene95381\_JingChaoZuZhi2, Unigene95389\_JingChaoZuZhi2, Unigene95819\_JingChaoZuZhi2 |
| 140 | Retinol metabolism | Unigene12217\_JingChaoZuZhi2, Unigene12454\_JingChaoZuZhi2, Unigene13068\_JingChaoZuZhi2, Unigene13160\_JingChaoZuZhi2, Unigene13307\_JingChaoZuZhi2, Unigene14448\_JingChaoZuZhi2, Unigene15363\_JingChaoZuZhi2, Unigene18510\_JingChaoZuZhi2, Unigene18569\_JingChaoZuZhi2, Unigene18666\_JingChaoZuZhi2, Unigene19410\_JingChaoZuZhi2, Unigene19778\_JingChaoZuZhi2, Unigene20264\_JingChaoZuZhi2, Unigene20504\_JingChaoZuZhi2, Unigene21682\_JingChaoZuZhi2, Unigene21977\_JingChaoZuZhi2, Unigene26954\_JingChaoZuZhi2, Unigene29995\_JingChaoZuZhi2, Unigene3714\_JingChaoZuZhi2, Unigene37828\_JingChaoZuZhi2, Unigene41739\_JingChaoZuZhi2, Unigene47722\_JingChaoZuZhi2, Unigene48173\_JingChaoZuZhi2, Unigene48343\_JingChaoZuZhi2, Unigene50305\_JingChaoZuZhi2, Unigene52889\_JingChaoZuZhi2, Unigene53447\_JingChaoZuZhi2, Unigene54783\_JingChaoZuZhi2, Unigene55355\_JingChaoZuZhi2, Unigene56370\_JingChaoZuZhi2, Unigene57061\_JingChaoZuZhi2, Unigene57130\_JingChaoZuZhi2, Unigene59427\_JingChaoZuZhi2, Unigene62924\_JingChaoZuZhi2, Unigene62972\_JingChaoZuZhi2, Unigene67078\_JingChaoZuZhi2, Unigene6739\_JingChaoZuZhi2, Unigene70488\_JingChaoZuZhi2, Unigene71976\_JingChaoZuZhi2, Unigene7528\_JingChaoZuZhi2, Unigene79198\_JingChaoZuZhi2, Unigene80285\_JingChaoZuZhi2, Unigene81630\_JingChaoZuZhi2, Unigene81708\_JingChaoZuZhi2, Unigene81878\_JingChaoZuZhi2, Unigene82818\_JingChaoZuZhi2, Unigene83273\_JingChaoZuZhi2, Unigene83913\_JingChaoZuZhi2, Unigene84371\_JingChaoZuZhi2, Unigene84385\_JingChaoZuZhi2, Unigene85539\_JingChaoZuZhi2, Unigene85937\_JingChaoZuZhi2, Unigene88383\_JingChaoZuZhi2, Unigene883\_JingChaoZuZhi2, Unigene89783\_JingChaoZuZhi2, Unigene90376\_JingChaoZuZhi2, Unigene90773\_JingChaoZuZhi2, Unigene92172\_JingChaoZuZhi2, Unigene92281\_JingChaoZuZhi2, Unigene92757\_JingChaoZuZhi2, Unigene93255\_JingChaoZuZhi2, Unigene94001\_JingChaoZuZhi2, Unigene94009\_JingChaoZuZhi2, Unigene94838\_JingChaoZuZhi2, Unigene95458\_JingChaoZuZhi2, Unigene95561\_JingChaoZuZhi2 |
| 141 | Metabolism of xenobiotics by cytochrome P450 | Unigene11803\_JingChaoZuZhi2, Unigene12292\_JingChaoZuZhi2, Unigene13068\_JingChaoZuZhi2, Unigene13160\_JingChaoZuZhi2, Unigene13337\_JingChaoZuZhi2, Unigene14339\_JingChaoZuZhi2, Unigene14448\_JingChaoZuZhi2, Unigene18029\_JingChaoZuZhi2, Unigene18666\_JingChaoZuZhi2, Unigene19646\_JingChaoZuZhi2, Unigene20264\_JingChaoZuZhi2, Unigene20504\_JingChaoZuZhi2, Unigene26954\_JingChaoZuZhi2, Unigene29995\_JingChaoZuZhi2, Unigene3936\_JingChaoZuZhi2, Unigene3955\_JingChaoZuZhi2, Unigene46463\_JingChaoZuZhi2, Unigene47722\_JingChaoZuZhi2, Unigene48173\_JingChaoZuZhi2, Unigene48343\_JingChaoZuZhi2, Unigene49290\_JingChaoZuZhi2, Unigene50305\_JingChaoZuZhi2, Unigene53447\_JingChaoZuZhi2, Unigene55355\_JingChaoZuZhi2, Unigene55810\_JingChaoZuZhi2, Unigene56370\_JingChaoZuZhi2, Unigene57061\_JingChaoZuZhi2, Unigene57130\_JingChaoZuZhi2, Unigene5875\_JingChaoZuZhi2, Unigene59427\_JingChaoZuZhi2, Unigene60326\_JingChaoZuZhi2, Unigene62568\_JingChaoZuZhi2, Unigene62924\_JingChaoZuZhi2, Unigene70921\_JingChaoZuZhi2, Unigene71976\_JingChaoZuZhi2, Unigene7200\_JingChaoZuZhi2, Unigene72476\_JingChaoZuZhi2, Unigene74771\_JingChaoZuZhi2, Unigene75948\_JingChaoZuZhi2, Unigene77016\_JingChaoZuZhi2, Unigene79198\_JingChaoZuZhi2, Unigene80285\_JingChaoZuZhi2, Unigene81630\_JingChaoZuZhi2, Unigene81878\_JingChaoZuZhi2, Unigene82818\_JingChaoZuZhi2, Unigene83273\_JingChaoZuZhi2, Unigene83913\_JingChaoZuZhi2, Unigene84385\_JingChaoZuZhi2, Unigene84531\_JingChaoZuZhi2, Unigene85539\_JingChaoZuZhi2, Unigene85937\_JingChaoZuZhi2, Unigene86047\_JingChaoZuZhi2, Unigene86806\_JingChaoZuZhi2, Unigene87676\_JingChaoZuZhi2, Unigene88383\_JingChaoZuZhi2, Unigene89347\_JingChaoZuZhi2, Unigene92281\_JingChaoZuZhi2, Unigene92983\_JingChaoZuZhi2, Unigene93067\_JingChaoZuZhi2, Unigene93255\_JingChaoZuZhi2, Unigene93334\_JingChaoZuZhi2, Unigene94894\_JingChaoZuZhi2, Unigene95016\_JingChaoZuZhi2, Unigene95561\_JingChaoZuZhi2, Unigene9631\_JingChaoZuZhi2 |
| 142 | Drug metabolism - cytochrome P450 | Unigene11803\_JingChaoZuZhi2, Unigene12292\_JingChaoZuZhi2, Unigene12758\_JingChaoZuZhi2, Unigene13068\_JingChaoZuZhi2, Unigene13160\_JingChaoZuZhi2, Unigene13337\_JingChaoZuZhi2, Unigene14339\_JingChaoZuZhi2, Unigene14448\_JingChaoZuZhi2, Unigene18029\_JingChaoZuZhi2, Unigene18666\_JingChaoZuZhi2, Unigene20264\_JingChaoZuZhi2, Unigene20504\_JingChaoZuZhi2, Unigene21025\_JingChaoZuZhi2, Unigene22846\_JingChaoZuZhi2, Unigene26954\_JingChaoZuZhi2, Unigene29995\_JingChaoZuZhi2, Unigene3936\_JingChaoZuZhi2, Unigene46463\_JingChaoZuZhi2, Unigene47722\_JingChaoZuZhi2, Unigene48173\_JingChaoZuZhi2, Unigene48343\_JingChaoZuZhi2, Unigene50305\_JingChaoZuZhi2, Unigene53447\_JingChaoZuZhi2, Unigene5449\_JingChaoZuZhi2, Unigene55355\_JingChaoZuZhi2, Unigene55810\_JingChaoZuZhi2, Unigene56370\_JingChaoZuZhi2, Unigene57061\_JingChaoZuZhi2, Unigene57130\_JingChaoZuZhi2, Unigene5875\_JingChaoZuZhi2, Unigene59427\_JingChaoZuZhi2, Unigene60326\_JingChaoZuZhi2, Unigene62568\_JingChaoZuZhi2, Unigene62924\_JingChaoZuZhi2, Unigene67985\_JingChaoZuZhi2, Unigene70921\_JingChaoZuZhi2, Unigene71976\_JingChaoZuZhi2, Unigene72476\_JingChaoZuZhi2, Unigene74771\_JingChaoZuZhi2, Unigene75948\_JingChaoZuZhi2, Unigene77016\_JingChaoZuZhi2, Unigene79198\_JingChaoZuZhi2, Unigene80285\_JingChaoZuZhi2, Unigene81630\_JingChaoZuZhi2, Unigene81878\_JingChaoZuZhi2, Unigene82818\_JingChaoZuZhi2, Unigene83273\_JingChaoZuZhi2, Unigene83913\_JingChaoZuZhi2, Unigene84385\_JingChaoZuZhi2, Unigene84531\_JingChaoZuZhi2, Unigene85539\_JingChaoZuZhi2, Unigene85937\_JingChaoZuZhi2, Unigene86047\_JingChaoZuZhi2, Unigene86806\_JingChaoZuZhi2, Unigene88383\_JingChaoZuZhi2, Unigene89347\_JingChaoZuZhi2, Unigene92281\_JingChaoZuZhi2, Unigene92983\_JingChaoZuZhi2, Unigene93067\_JingChaoZuZhi2, Unigene93255\_JingChaoZuZhi2, Unigene93334\_JingChaoZuZhi2, Unigene94894\_JingChaoZuZhi2, Unigene95016\_JingChaoZuZhi2, Unigene95561\_JingChaoZuZhi2, Unigene9631\_JingChaoZuZhi2 |
| 143 | Circadian rhythm - fly | Unigene11875\_JingChaoZuZhi2, Unigene12452\_JingChaoZuZhi2, Unigene13167\_JingChaoZuZhi2, Unigene16289\_JingChaoZuZhi2, Unigene16407\_JingChaoZuZhi2, Unigene16820\_JingChaoZuZhi2, Unigene17868\_JingChaoZuZhi2, Unigene1790\_JingChaoZuZhi2, Unigene18027\_JingChaoZuZhi2, Unigene18812\_JingChaoZuZhi2, Unigene20239\_JingChaoZuZhi2, Unigene21036\_JingChaoZuZhi2, Unigene21695\_JingChaoZuZhi2, Unigene22008\_JingChaoZuZhi2, Unigene22708\_JingChaoZuZhi2, Unigene23089\_JingChaoZuZhi2, Unigene24668\_JingChaoZuZhi2, Unigene27057\_JingChaoZuZhi2, Unigene28711\_JingChaoZuZhi2, Unigene32049\_JingChaoZuZhi2, Unigene3307\_JingChaoZuZhi2, Unigene33281\_JingChaoZuZhi2, Unigene34624\_JingChaoZuZhi2, Unigene41670\_JingChaoZuZhi2, Unigene42406\_JingChaoZuZhi2, Unigene4625\_JingChaoZuZhi2, Unigene47548\_JingChaoZuZhi2, Unigene48813\_JingChaoZuZhi2, Unigene48986\_JingChaoZuZhi2, Unigene5102\_JingChaoZuZhi2, Unigene53160\_JingChaoZuZhi2, Unigene5671\_JingChaoZuZhi2, Unigene58704\_JingChaoZuZhi2, Unigene59127\_JingChaoZuZhi2, Unigene63247\_JingChaoZuZhi2, Unigene63885\_JingChaoZuZhi2, Unigene64880\_JingChaoZuZhi2, Unigene6546\_JingChaoZuZhi2, Unigene66472\_JingChaoZuZhi2, Unigene67959\_JingChaoZuZhi2, Unigene68206\_JingChaoZuZhi2, Unigene70125\_JingChaoZuZhi2, Unigene72534\_JingChaoZuZhi2, Unigene74056\_JingChaoZuZhi2, Unigene74498\_JingChaoZuZhi2, Unigene74609\_JingChaoZuZhi2, Unigene76614\_JingChaoZuZhi2, Unigene77305\_JingChaoZuZhi2, Unigene77359\_JingChaoZuZhi2, Unigene77890\_JingChaoZuZhi2, Unigene80385\_JingChaoZuZhi2, Unigene80927\_JingChaoZuZhi2, Unigene82688\_JingChaoZuZhi2, Unigene85495\_JingChaoZuZhi2, Unigene85510\_JingChaoZuZhi2, Unigene86416\_JingChaoZuZhi2, Unigene88719\_JingChaoZuZhi2, Unigene88755\_JingChaoZuZhi2, Unigene89310\_JingChaoZuZhi2, Unigene91904\_JingChaoZuZhi2, Unigene94071\_JingChaoZuZhi2, Unigene94827\_JingChaoZuZhi2, Unigene95098\_JingChaoZuZhi2, Unigene95295\_JingChaoZuZhi2, Unigene95734\_JingChaoZuZhi2 |
| 144 | Leishmaniasis | Unigene10318\_JingChaoZuZhi2, Unigene11251\_JingChaoZuZhi2, Unigene11446\_JingChaoZuZhi2, Unigene11732\_JingChaoZuZhi2, Unigene1298\_JingChaoZuZhi2, Unigene1301\_JingChaoZuZhi2, Unigene13332\_JingChaoZuZhi2, Unigene14109\_JingChaoZuZhi2, Unigene14635\_JingChaoZuZhi2, Unigene16873\_JingChaoZuZhi2, Unigene17062\_JingChaoZuZhi2, Unigene17178\_JingChaoZuZhi2, Unigene18515\_JingChaoZuZhi2, Unigene18715\_JingChaoZuZhi2, Unigene19235\_JingChaoZuZhi2, Unigene19404\_JingChaoZuZhi2, Unigene20120\_JingChaoZuZhi2, Unigene20411\_JingChaoZuZhi2, Unigene21472\_JingChaoZuZhi2, Unigene21578\_JingChaoZuZhi2, Unigene21697\_JingChaoZuZhi2, Unigene21996\_JingChaoZuZhi2, Unigene22482\_JingChaoZuZhi2, Unigene22604\_JingChaoZuZhi2, Unigene22779\_JingChaoZuZhi2, Unigene25870\_JingChaoZuZhi2, Unigene260\_JingChaoZuZhi2, Unigene2628\_JingChaoZuZhi2, Unigene26730\_JingChaoZuZhi2, Unigene29201\_JingChaoZuZhi2, Unigene2960\_JingChaoZuZhi2, Unigene35243\_JingChaoZuZhi2, Unigene47155\_JingChaoZuZhi2, Unigene4853\_JingChaoZuZhi2, Unigene48843\_JingChaoZuZhi2, Unigene5099\_JingChaoZuZhi2, Unigene5314\_JingChaoZuZhi2, Unigene58550\_JingChaoZuZhi2, Unigene62841\_JingChaoZuZhi2, Unigene64164\_JingChaoZuZhi2, Unigene6459\_JingChaoZuZhi2, Unigene64830\_JingChaoZuZhi2, Unigene69071\_JingChaoZuZhi2, Unigene70723\_JingChaoZuZhi2, Unigene72033\_JingChaoZuZhi2, Unigene75840\_JingChaoZuZhi2, Unigene78508\_JingChaoZuZhi2, Unigene79860\_JingChaoZuZhi2, Unigene79911\_JingChaoZuZhi2, Unigene8191\_JingChaoZuZhi2, Unigene84519\_JingChaoZuZhi2, Unigene85615\_JingChaoZuZhi2, Unigene85880\_JingChaoZuZhi2, Unigene87332\_JingChaoZuZhi2, Unigene88942\_JingChaoZuZhi2, Unigene91661\_JingChaoZuZhi2, Unigene93912\_JingChaoZuZhi2, Unigene94236\_JingChaoZuZhi2, Unigene94561\_JingChaoZuZhi2, Unigene94619\_JingChaoZuZhi2, Unigene9517\_JingChaoZuZhi2, Unigene95677\_JingChaoZuZhi2, Unigene96121\_JingChaoZuZhi2 |
| 145 | Arachidonic acid metabolism | Unigene11055\_JingChaoZuZhi2, Unigene11803\_JingChaoZuZhi2, Unigene12260\_JingChaoZuZhi2, Unigene13420\_JingChaoZuZhi2, Unigene13869\_JingChaoZuZhi2, Unigene14339\_JingChaoZuZhi2, Unigene14357\_JingChaoZuZhi2, Unigene14448\_JingChaoZuZhi2, Unigene15425\_JingChaoZuZhi2, Unigene15892\_JingChaoZuZhi2, Unigene15893\_JingChaoZuZhi2, Unigene15942\_JingChaoZuZhi2, Unigene16524\_JingChaoZuZhi2, Unigene17709\_JingChaoZuZhi2, Unigene18029\_JingChaoZuZhi2, Unigene18229\_JingChaoZuZhi2, Unigene18666\_JingChaoZuZhi2, Unigene19778\_JingChaoZuZhi2, Unigene19904\_JingChaoZuZhi2, Unigene20411\_JingChaoZuZhi2, Unigene21025\_JingChaoZuZhi2, Unigene21407\_JingChaoZuZhi2, Unigene22904\_JingChaoZuZhi2, Unigene26954\_JingChaoZuZhi2, Unigene2909\_JingChaoZuZhi2, Unigene2916\_JingChaoZuZhi2, Unigene29995\_JingChaoZuZhi2, Unigene42329\_JingChaoZuZhi2, Unigene43723\_JingChaoZuZhi2, Unigene45532\_JingChaoZuZhi2, Unigene47722\_JingChaoZuZhi2, Unigene48173\_JingChaoZuZhi2, Unigene53831\_JingChaoZuZhi2, Unigene55355\_JingChaoZuZhi2, Unigene58437\_JingChaoZuZhi2, Unigene62924\_JingChaoZuZhi2, Unigene67328\_JingChaoZuZhi2, Unigene68151\_JingChaoZuZhi2, Unigene72098\_JingChaoZuZhi2, Unigene74594\_JingChaoZuZhi2, Unigene74736\_JingChaoZuZhi2, Unigene76182\_JingChaoZuZhi2, Unigene78508\_JingChaoZuZhi2, Unigene79976\_JingChaoZuZhi2, Unigene80211\_JingChaoZuZhi2, Unigene80948\_JingChaoZuZhi2, Unigene81236\_JingChaoZuZhi2, Unigene81959\_JingChaoZuZhi2, Unigene83913\_JingChaoZuZhi2, Unigene85035\_JingChaoZuZhi2, Unigene85937\_JingChaoZuZhi2, Unigene87025\_JingChaoZuZhi2, Unigene87416\_JingChaoZuZhi2, Unigene88068\_JingChaoZuZhi2, Unigene90393\_JingChaoZuZhi2, Unigene9140\_JingChaoZuZhi2, Unigene92281\_JingChaoZuZhi2, Unigene92737\_JingChaoZuZhi2, Unigene94614\_JingChaoZuZhi2, Unigene95174\_JingChaoZuZhi2, Unigene9\_JingChaoZuZhi2 |
| 146 | Nicotinate and nicotinamide metabolism | Unigene10769\_JingChaoZuZhi2, Unigene10979\_JingChaoZuZhi2, Unigene11773\_JingChaoZuZhi2, Unigene12286\_JingChaoZuZhi2, Unigene12758\_JingChaoZuZhi2, Unigene15770\_JingChaoZuZhi2, Unigene173\_JingChaoZuZhi2, Unigene19355\_JingChaoZuZhi2, Unigene19436\_JingChaoZuZhi2, Unigene19530\_JingChaoZuZhi2, Unigene19555\_JingChaoZuZhi2, Unigene19950\_JingChaoZuZhi2, Unigene19978\_JingChaoZuZhi2, Unigene20452\_JingChaoZuZhi2, Unigene21270\_JingChaoZuZhi2, Unigene21859\_JingChaoZuZhi2, Unigene22065\_JingChaoZuZhi2, Unigene22392\_JingChaoZuZhi2, Unigene30651\_JingChaoZuZhi2, Unigene30662\_JingChaoZuZhi2, Unigene34189\_JingChaoZuZhi2, Unigene35740\_JingChaoZuZhi2, Unigene3580\_JingChaoZuZhi2, Unigene40598\_JingChaoZuZhi2, Unigene46994\_JingChaoZuZhi2, Unigene50977\_JingChaoZuZhi2, Unigene52773\_JingChaoZuZhi2, Unigene5449\_JingChaoZuZhi2, Unigene55583\_JingChaoZuZhi2, Unigene61121\_JingChaoZuZhi2, Unigene65278\_JingChaoZuZhi2, Unigene65525\_JingChaoZuZhi2, Unigene65835\_JingChaoZuZhi2, Unigene66205\_JingChaoZuZhi2, Unigene6802\_JingChaoZuZhi2, Unigene7206\_JingChaoZuZhi2, Unigene74409\_JingChaoZuZhi2, Unigene75690\_JingChaoZuZhi2, Unigene76261\_JingChaoZuZhi2, Unigene76865\_JingChaoZuZhi2, Unigene77749\_JingChaoZuZhi2, Unigene78757\_JingChaoZuZhi2, Unigene80500\_JingChaoZuZhi2, Unigene82683\_JingChaoZuZhi2, Unigene84894\_JingChaoZuZhi2, Unigene86295\_JingChaoZuZhi2, Unigene86915\_JingChaoZuZhi2, Unigene8970\_JingChaoZuZhi2, Unigene90008\_JingChaoZuZhi2, Unigene90034\_JingChaoZuZhi2, Unigene91504\_JingChaoZuZhi2, Unigene91943\_JingChaoZuZhi2, Unigene92272\_JingChaoZuZhi2, Unigene93354\_JingChaoZuZhi2, Unigene94086\_JingChaoZuZhi2, Unigene94908\_JingChaoZuZhi2, Unigene95037\_JingChaoZuZhi2, Unigene95162\_JingChaoZuZhi2 |
| 147 | Linoleic acid metabolism | Unigene11055\_JingChaoZuZhi2, Unigene12260\_JingChaoZuZhi2, Unigene13068\_JingChaoZuZhi2, Unigene13160\_JingChaoZuZhi2, Unigene13420\_JingChaoZuZhi2, Unigene13869\_JingChaoZuZhi2, Unigene14132\_JingChaoZuZhi2, Unigene14357\_JingChaoZuZhi2, Unigene14448\_JingChaoZuZhi2, Unigene15425\_JingChaoZuZhi2, Unigene15942\_JingChaoZuZhi2, Unigene18029\_JingChaoZuZhi2, Unigene18114\_JingChaoZuZhi2, Unigene18510\_JingChaoZuZhi2, Unigene18666\_JingChaoZuZhi2, Unigene19904\_JingChaoZuZhi2, Unigene20264\_JingChaoZuZhi2, Unigene20504\_JingChaoZuZhi2, Unigene21025\_JingChaoZuZhi2, Unigene21407\_JingChaoZuZhi2, Unigene22492\_JingChaoZuZhi2, Unigene26954\_JingChaoZuZhi2, Unigene29995\_JingChaoZuZhi2, Unigene42329\_JingChaoZuZhi2, Unigene43723\_JingChaoZuZhi2, Unigene45532\_JingChaoZuZhi2, Unigene47722\_JingChaoZuZhi2, Unigene48343\_JingChaoZuZhi2, Unigene50305\_JingChaoZuZhi2, Unigene53447\_JingChaoZuZhi2, Unigene55355\_JingChaoZuZhi2, Unigene56370\_JingChaoZuZhi2, Unigene59427\_JingChaoZuZhi2, Unigene62924\_JingChaoZuZhi2, Unigene67328\_JingChaoZuZhi2, Unigene68151\_JingChaoZuZhi2, Unigene70488\_JingChaoZuZhi2, Unigene72098\_JingChaoZuZhi2, Unigene74594\_JingChaoZuZhi2, Unigene75891\_JingChaoZuZhi2, Unigene76182\_JingChaoZuZhi2, Unigene81630\_JingChaoZuZhi2, Unigene81878\_JingChaoZuZhi2, Unigene83273\_JingChaoZuZhi2, Unigene83913\_JingChaoZuZhi2, Unigene85539\_JingChaoZuZhi2, Unigene85937\_JingChaoZuZhi2, Unigene88068\_JingChaoZuZhi2, Unigene88383\_JingChaoZuZhi2, Unigene90317\_JingChaoZuZhi2, Unigene90376\_JingChaoZuZhi2, Unigene92281\_JingChaoZuZhi2, Unigene92737\_JingChaoZuZhi2, Unigene92757\_JingChaoZuZhi2, Unigene93255\_JingChaoZuZhi2, Unigene9\_JingChaoZuZhi2 |
| 148 | Limonene and pinene degradation | Unigene10011\_JingChaoZuZhi2, Unigene13160\_JingChaoZuZhi2, Unigene13640\_JingChaoZuZhi2, Unigene16352\_JingChaoZuZhi2, Unigene18358\_JingChaoZuZhi2, Unigene18409\_JingChaoZuZhi2, Unigene18553\_JingChaoZuZhi2, Unigene18773\_JingChaoZuZhi2, Unigene20192\_JingChaoZuZhi2, Unigene20264\_JingChaoZuZhi2, Unigene21049\_JingChaoZuZhi2, Unigene21176\_JingChaoZuZhi2, Unigene21821\_JingChaoZuZhi2, Unigene2235\_JingChaoZuZhi2, Unigene22793\_JingChaoZuZhi2, Unigene44759\_JingChaoZuZhi2, Unigene46619\_JingChaoZuZhi2, Unigene50305\_JingChaoZuZhi2, Unigene5068\_JingChaoZuZhi2, Unigene51532\_JingChaoZuZhi2, Unigene51751\_JingChaoZuZhi2, Unigene52259\_JingChaoZuZhi2, Unigene53447\_JingChaoZuZhi2, Unigene61664\_JingChaoZuZhi2, Unigene62515\_JingChaoZuZhi2, Unigene63649\_JingChaoZuZhi2, Unigene63715\_JingChaoZuZhi2, Unigene64217\_JingChaoZuZhi2, Unigene64903\_JingChaoZuZhi2, Unigene66337\_JingChaoZuZhi2, Unigene67613\_JingChaoZuZhi2, Unigene70209\_JingChaoZuZhi2, Unigene72476\_JingChaoZuZhi2, Unigene79414\_JingChaoZuZhi2, Unigene80881\_JingChaoZuZhi2, Unigene81656\_JingChaoZuZhi2, Unigene81878\_JingChaoZuZhi2, Unigene82129\_JingChaoZuZhi2, Unigene83273\_JingChaoZuZhi2, Unigene84063\_JingChaoZuZhi2, Unigene84343\_JingChaoZuZhi2, Unigene8454\_JingChaoZuZhi2, Unigene84851\_JingChaoZuZhi2, Unigene87044\_JingChaoZuZhi2, Unigene87485\_JingChaoZuZhi2, Unigene90127\_JingChaoZuZhi2, Unigene91700\_JingChaoZuZhi2, Unigene93334\_JingChaoZuZhi2, Unigene93600\_JingChaoZuZhi2, Unigene93681\_JingChaoZuZhi2, Unigene93910\_JingChaoZuZhi2, Unigene94837\_JingChaoZuZhi2, Unigene95174\_JingChaoZuZhi2, Unigene95959\_JingChaoZuZhi2, Unigene95985\_JingChaoZuZhi2, Unigene96031\_JingChaoZuZhi2 |
| 149 | Biosynthesis of unsaturated fatty acids | Unigene11456\_JingChaoZuZhi2, Unigene14132\_JingChaoZuZhi2, Unigene14715\_JingChaoZuZhi2, Unigene16663\_JingChaoZuZhi2, Unigene16879\_JingChaoZuZhi2, Unigene16899\_JingChaoZuZhi2, Unigene17884\_JingChaoZuZhi2, Unigene18163\_JingChaoZuZhi2, Unigene18409\_JingChaoZuZhi2, Unigene18553\_JingChaoZuZhi2, Unigene18846\_JingChaoZuZhi2, Unigene18963\_JingChaoZuZhi2, Unigene19490\_JingChaoZuZhi2, Unigene20187\_JingChaoZuZhi2, Unigene2054\_JingChaoZuZhi2, Unigene22352\_JingChaoZuZhi2, Unigene22492\_JingChaoZuZhi2, Unigene22568\_JingChaoZuZhi2, Unigene22793\_JingChaoZuZhi2, Unigene35187\_JingChaoZuZhi2, Unigene38419\_JingChaoZuZhi2, Unigene41233\_JingChaoZuZhi2, Unigene45150\_JingChaoZuZhi2, Unigene45176\_JingChaoZuZhi2, Unigene49819\_JingChaoZuZhi2, Unigene51532\_JingChaoZuZhi2, Unigene59548\_JingChaoZuZhi2, Unigene61162\_JingChaoZuZhi2, Unigene61664\_JingChaoZuZhi2, Unigene62962\_JingChaoZuZhi2, Unigene63715\_JingChaoZuZhi2, Unigene64903\_JingChaoZuZhi2, Unigene65441\_JingChaoZuZhi2, Unigene73451\_JingChaoZuZhi2, Unigene74590\_JingChaoZuZhi2, Unigene75891\_JingChaoZuZhi2, Unigene78854\_JingChaoZuZhi2, Unigene79135\_JingChaoZuZhi2, Unigene80446\_JingChaoZuZhi2, Unigene8213\_JingChaoZuZhi2, Unigene82170\_JingChaoZuZhi2, Unigene82435\_JingChaoZuZhi2, Unigene84124\_JingChaoZuZhi2, Unigene84343\_JingChaoZuZhi2, Unigene8454\_JingChaoZuZhi2, Unigene85989\_JingChaoZuZhi2, Unigene87485\_JingChaoZuZhi2, Unigene88377\_JingChaoZuZhi2, Unigene90949\_JingChaoZuZhi2, Unigene91374\_JingChaoZuZhi2, Unigene93681\_JingChaoZuZhi2, Unigene93810\_JingChaoZuZhi2, Unigene94422\_JingChaoZuZhi2, Unigene95603\_JingChaoZuZhi2, Unigene96129\_JingChaoZuZhi2, Unigene9847\_JingChaoZuZhi2 |
| 150 | Sphingolipid metabolism | Unigene10032\_JingChaoZuZhi2, Unigene11681\_JingChaoZuZhi2, Unigene13565\_JingChaoZuZhi2, Unigene14018\_JingChaoZuZhi2, Unigene16679\_JingChaoZuZhi2, Unigene19051\_JingChaoZuZhi2, Unigene19251\_JingChaoZuZhi2, Unigene19928\_JingChaoZuZhi2, Unigene20393\_JingChaoZuZhi2, Unigene20531\_JingChaoZuZhi2, Unigene21230\_JingChaoZuZhi2, Unigene21363\_JingChaoZuZhi2, Unigene21541\_JingChaoZuZhi2, Unigene22449\_JingChaoZuZhi2, Unigene24793\_JingChaoZuZhi2, Unigene2535\_JingChaoZuZhi2, Unigene26787\_JingChaoZuZhi2, Unigene27220\_JingChaoZuZhi2, Unigene42020\_JingChaoZuZhi2, Unigene44246\_JingChaoZuZhi2, Unigene45407\_JingChaoZuZhi2, Unigene53167\_JingChaoZuZhi2, Unigene53875\_JingChaoZuZhi2, Unigene55114\_JingChaoZuZhi2, Unigene56089\_JingChaoZuZhi2, Unigene57061\_JingChaoZuZhi2, Unigene57130\_JingChaoZuZhi2, Unigene6078\_JingChaoZuZhi2, Unigene60942\_JingChaoZuZhi2, Unigene64563\_JingChaoZuZhi2, Unigene6573\_JingChaoZuZhi2, Unigene66615\_JingChaoZuZhi2, Unigene70190\_JingChaoZuZhi2, Unigene71724\_JingChaoZuZhi2, Unigene72924\_JingChaoZuZhi2, Unigene7314\_JingChaoZuZhi2, Unigene74505\_JingChaoZuZhi2, Unigene76480\_JingChaoZuZhi2, Unigene77482\_JingChaoZuZhi2, Unigene77755\_JingChaoZuZhi2, Unigene80895\_JingChaoZuZhi2, Unigene80932\_JingChaoZuZhi2, Unigene82258\_JingChaoZuZhi2, Unigene82464\_JingChaoZuZhi2, Unigene83138\_JingChaoZuZhi2, Unigene83308\_JingChaoZuZhi2, Unigene85693\_JingChaoZuZhi2, Unigene88047\_JingChaoZuZhi2, Unigene88704\_JingChaoZuZhi2, Unigene91460\_JingChaoZuZhi2, Unigene92149\_JingChaoZuZhi2, Unigene92153\_JingChaoZuZhi2, Unigene94772\_JingChaoZuZhi2, Unigene95657\_JingChaoZuZhi2, Unigene95911\_JingChaoZuZhi2, Unigene9750\_JingChaoZuZhi2 |
| 151 | Ether lipid metabolism | Unigene11055\_JingChaoZuZhi2, Unigene11721\_JingChaoZuZhi2, Unigene12260\_JingChaoZuZhi2, Unigene13289\_JingChaoZuZhi2, Unigene13420\_JingChaoZuZhi2, Unigene13869\_JingChaoZuZhi2, Unigene13946\_JingChaoZuZhi2, Unigene14357\_JingChaoZuZhi2, Unigene15216\_JingChaoZuZhi2, Unigene15425\_JingChaoZuZhi2, Unigene15770\_JingChaoZuZhi2, Unigene15942\_JingChaoZuZhi2, Unigene16681\_JingChaoZuZhi2, Unigene17049\_JingChaoZuZhi2, Unigene18313\_JingChaoZuZhi2, Unigene19904\_JingChaoZuZhi2, Unigene20179\_JingChaoZuZhi2, Unigene20896\_JingChaoZuZhi2, Unigene21407\_JingChaoZuZhi2, Unigene2275\_JingChaoZuZhi2, Unigene3700\_JingChaoZuZhi2, Unigene42329\_JingChaoZuZhi2, Unigene43723\_JingChaoZuZhi2, Unigene44246\_JingChaoZuZhi2, Unigene45532\_JingChaoZuZhi2, Unigene48331\_JingChaoZuZhi2, Unigene49664\_JingChaoZuZhi2, Unigene59480\_JingChaoZuZhi2, Unigene66246\_JingChaoZuZhi2, Unigene67328\_JingChaoZuZhi2, Unigene68151\_JingChaoZuZhi2, Unigene71103\_JingChaoZuZhi2, Unigene72016\_JingChaoZuZhi2, Unigene72098\_JingChaoZuZhi2, Unigene72136\_JingChaoZuZhi2, Unigene72565\_JingChaoZuZhi2, Unigene7283\_JingChaoZuZhi2, Unigene74216\_JingChaoZuZhi2, Unigene74594\_JingChaoZuZhi2, Unigene76182\_JingChaoZuZhi2, Unigene76480\_JingChaoZuZhi2, Unigene76957\_JingChaoZuZhi2, Unigene79166\_JingChaoZuZhi2, Unigene80895\_JingChaoZuZhi2, Unigene88068\_JingChaoZuZhi2, Unigene8952\_JingChaoZuZhi2, Unigene9128\_JingChaoZuZhi2, Unigene92394\_JingChaoZuZhi2, Unigene92737\_JingChaoZuZhi2, Unigene93106\_JingChaoZuZhi2, Unigene94691\_JingChaoZuZhi2, Unigene95399\_JingChaoZuZhi2, Unigene95663\_JingChaoZuZhi2, Unigene95939\_JingChaoZuZhi2, Unigene9\_JingChaoZuZhi2 |
| 152 | Pentose phosphate pathway | Unigene12419\_JingChaoZuZhi2, Unigene12745\_JingChaoZuZhi2, Unigene13837\_JingChaoZuZhi2, Unigene15418\_JingChaoZuZhi2, Unigene16107\_JingChaoZuZhi2, Unigene16281\_JingChaoZuZhi2, Unigene16788\_JingChaoZuZhi2, Unigene19181\_JingChaoZuZhi2, Unigene20936\_JingChaoZuZhi2, Unigene21122\_JingChaoZuZhi2, Unigene22123\_JingChaoZuZhi2, Unigene22503\_JingChaoZuZhi2, Unigene28844\_JingChaoZuZhi2, Unigene38078\_JingChaoZuZhi2, Unigene42414\_JingChaoZuZhi2, Unigene44590\_JingChaoZuZhi2, Unigene45888\_JingChaoZuZhi2, Unigene46843\_JingChaoZuZhi2, Unigene48626\_JingChaoZuZhi2, Unigene4954\_JingChaoZuZhi2, Unigene49766\_JingChaoZuZhi2, Unigene49963\_JingChaoZuZhi2, Unigene50456\_JingChaoZuZhi2, Unigene50485\_JingChaoZuZhi2, Unigene5345\_JingChaoZuZhi2, Unigene55360\_JingChaoZuZhi2, Unigene55918\_JingChaoZuZhi2, Unigene56745\_JingChaoZuZhi2, Unigene5750\_JingChaoZuZhi2, Unigene59697\_JingChaoZuZhi2, Unigene65498\_JingChaoZuZhi2, Unigene71339\_JingChaoZuZhi2, Unigene74987\_JingChaoZuZhi2, Unigene76385\_JingChaoZuZhi2, Unigene76593\_JingChaoZuZhi2, Unigene81511\_JingChaoZuZhi2, Unigene82808\_JingChaoZuZhi2, Unigene85477\_JingChaoZuZhi2, Unigene86120\_JingChaoZuZhi2, Unigene89279\_JingChaoZuZhi2, Unigene89289\_JingChaoZuZhi2, Unigene90862\_JingChaoZuZhi2, Unigene90875\_JingChaoZuZhi2, Unigene92442\_JingChaoZuZhi2, Unigene95296\_JingChaoZuZhi2, Unigene95350\_JingChaoZuZhi2, Unigene95594\_JingChaoZuZhi2, Unigene95656\_JingChaoZuZhi2, Unigene95953\_JingChaoZuZhi2, Unigene96197\_JingChaoZuZhi2, Unigene96220\_JingChaoZuZhi2, Unigene9753\_JingChaoZuZhi2 |
| 153 | Homologous recombination | Unigene10165\_JingChaoZuZhi2, Unigene10802\_JingChaoZuZhi2, Unigene11473\_JingChaoZuZhi2, Unigene11948\_JingChaoZuZhi2, Unigene12340\_JingChaoZuZhi2, Unigene12617\_JingChaoZuZhi2, Unigene13986\_JingChaoZuZhi2, Unigene1404\_JingChaoZuZhi2, Unigene14231\_JingChaoZuZhi2, Unigene14972\_JingChaoZuZhi2, Unigene15296\_JingChaoZuZhi2, Unigene16657\_JingChaoZuZhi2, Unigene1738\_JingChaoZuZhi2, Unigene1913\_JingChaoZuZhi2, Unigene19463\_JingChaoZuZhi2, Unigene21673\_JingChaoZuZhi2, Unigene2354\_JingChaoZuZhi2, Unigene2443\_JingChaoZuZhi2, Unigene32183\_JingChaoZuZhi2, Unigene3299\_JingChaoZuZhi2, Unigene3498\_JingChaoZuZhi2, Unigene37179\_JingChaoZuZhi2, Unigene460\_JingChaoZuZhi2, Unigene4628\_JingChaoZuZhi2, Unigene4631\_JingChaoZuZhi2, Unigene47688\_JingChaoZuZhi2, Unigene50890\_JingChaoZuZhi2, Unigene57042\_JingChaoZuZhi2, Unigene62018\_JingChaoZuZhi2, Unigene64812\_JingChaoZuZhi2, Unigene69029\_JingChaoZuZhi2, Unigene71482\_JingChaoZuZhi2, Unigene75961\_JingChaoZuZhi2, Unigene77174\_JingChaoZuZhi2, Unigene77653\_JingChaoZuZhi2, Unigene78372\_JingChaoZuZhi2, Unigene78962\_JingChaoZuZhi2, Unigene80058\_JingChaoZuZhi2, Unigene81843\_JingChaoZuZhi2, Unigene82183\_JingChaoZuZhi2, Unigene83447\_JingChaoZuZhi2, Unigene84437\_JingChaoZuZhi2, Unigene88770\_JingChaoZuZhi2, Unigene90116\_JingChaoZuZhi2, Unigene91096\_JingChaoZuZhi2, Unigene92094\_JingChaoZuZhi2, Unigene92525\_JingChaoZuZhi2, Unigene92803\_JingChaoZuZhi2, Unigene94259\_JingChaoZuZhi2, Unigene94583\_JingChaoZuZhi2, Unigene95084\_JingChaoZuZhi2, Unigene95381\_JingChaoZuZhi2 |
| 154 | Melanoma | Unigene10422\_JingChaoZuZhi2, Unigene11000\_JingChaoZuZhi2, Unigene11961\_JingChaoZuZhi2, Unigene12577\_JingChaoZuZhi2, Unigene13176\_JingChaoZuZhi2, Unigene14912\_JingChaoZuZhi2, Unigene15228\_JingChaoZuZhi2, Unigene17247\_JingChaoZuZhi2, Unigene17467\_JingChaoZuZhi2, Unigene18672\_JingChaoZuZhi2, Unigene19273\_JingChaoZuZhi2, Unigene20013\_JingChaoZuZhi2, Unigene21513\_JingChaoZuZhi2, Unigene2456\_JingChaoZuZhi2, Unigene2790\_JingChaoZuZhi2, Unigene3665\_JingChaoZuZhi2, Unigene3931\_JingChaoZuZhi2, Unigene39876\_JingChaoZuZhi2, Unigene42981\_JingChaoZuZhi2, Unigene4377\_JingChaoZuZhi2, Unigene48751\_JingChaoZuZhi2, Unigene49798\_JingChaoZuZhi2, Unigene50713\_JingChaoZuZhi2, Unigene51285\_JingChaoZuZhi2, Unigene54470\_JingChaoZuZhi2, Unigene56642\_JingChaoZuZhi2, Unigene57488\_JingChaoZuZhi2, Unigene5758\_JingChaoZuZhi2, Unigene6028\_JingChaoZuZhi2, Unigene62786\_JingChaoZuZhi2, Unigene64583\_JingChaoZuZhi2, Unigene69565\_JingChaoZuZhi2, Unigene70549\_JingChaoZuZhi2, Unigene72521\_JingChaoZuZhi2, Unigene75317\_JingChaoZuZhi2, Unigene76400\_JingChaoZuZhi2, Unigene78574\_JingChaoZuZhi2, Unigene80143\_JingChaoZuZhi2, Unigene8191\_JingChaoZuZhi2, Unigene84930\_JingChaoZuZhi2, Unigene86206\_JingChaoZuZhi2, Unigene86526\_JingChaoZuZhi2, Unigene876\_JingChaoZuZhi2, Unigene88748\_JingChaoZuZhi2, Unigene92112\_JingChaoZuZhi2, Unigene93807\_JingChaoZuZhi2, Unigene94685\_JingChaoZuZhi2, Unigene95791\_JingChaoZuZhi2, Unigene96281\_JingChaoZuZhi2, Unigene96294\_JingChaoZuZhi2, Unigene9902\_JingChaoZuZhi2 |
| 155 | Cytosolic DNA-sensing pathway | Unigene10340\_JingChaoZuZhi2, Unigene11723\_JingChaoZuZhi2, Unigene14268\_JingChaoZuZhi2, Unigene14446\_JingChaoZuZhi2, Unigene15290\_JingChaoZuZhi2, Unigene1628\_JingChaoZuZhi2, Unigene17221\_JingChaoZuZhi2, Unigene17788\_JingChaoZuZhi2, Unigene18508\_JingChaoZuZhi2, Unigene19579\_JingChaoZuZhi2, Unigene20157\_JingChaoZuZhi2, Unigene20924\_JingChaoZuZhi2, Unigene20991\_JingChaoZuZhi2, Unigene21057\_JingChaoZuZhi2, Unigene21697\_JingChaoZuZhi2, Unigene22126\_JingChaoZuZhi2, Unigene22466\_JingChaoZuZhi2, Unigene26274\_JingChaoZuZhi2, Unigene30849\_JingChaoZuZhi2, Unigene3187\_JingChaoZuZhi2, Unigene34338\_JingChaoZuZhi2, Unigene42364\_JingChaoZuZhi2, Unigene4461\_JingChaoZuZhi2, Unigene44745\_JingChaoZuZhi2, Unigene47824\_JingChaoZuZhi2, Unigene50927\_JingChaoZuZhi2, Unigene52751\_JingChaoZuZhi2, Unigene54320\_JingChaoZuZhi2, Unigene57880\_JingChaoZuZhi2, Unigene61659\_JingChaoZuZhi2, Unigene67958\_JingChaoZuZhi2, Unigene7459\_JingChaoZuZhi2, Unigene77570\_JingChaoZuZhi2, Unigene78224\_JingChaoZuZhi2, Unigene83132\_JingChaoZuZhi2, Unigene84024\_JingChaoZuZhi2, Unigene84463\_JingChaoZuZhi2, Unigene85958\_JingChaoZuZhi2, Unigene8778\_JingChaoZuZhi2, Unigene88451\_JingChaoZuZhi2, Unigene89454\_JingChaoZuZhi2, Unigene8973\_JingChaoZuZhi2, Unigene90953\_JingChaoZuZhi2, Unigene91206\_JingChaoZuZhi2, Unigene91513\_JingChaoZuZhi2, Unigene92908\_JingChaoZuZhi2, Unigene94118\_JingChaoZuZhi2, Unigene94457\_JingChaoZuZhi2, Unigene95476\_JingChaoZuZhi2 |
| 156 | Collecting duct acid secretion | Unigene11730\_JingChaoZuZhi2, Unigene14104\_JingChaoZuZhi2, Unigene15172\_JingChaoZuZhi2, Unigene16938\_JingChaoZuZhi2, Unigene18977\_JingChaoZuZhi2, Unigene20651\_JingChaoZuZhi2, Unigene21404\_JingChaoZuZhi2, Unigene21993\_JingChaoZuZhi2, Unigene21999\_JingChaoZuZhi2, Unigene22914\_JingChaoZuZhi2, Unigene2519\_JingChaoZuZhi2, Unigene25228\_JingChaoZuZhi2, Unigene26267\_JingChaoZuZhi2, Unigene33026\_JingChaoZuZhi2, Unigene38924\_JingChaoZuZhi2, Unigene39299\_JingChaoZuZhi2, Unigene40303\_JingChaoZuZhi2, Unigene49757\_JingChaoZuZhi2, Unigene55368\_JingChaoZuZhi2, Unigene5545\_JingChaoZuZhi2, Unigene56557\_JingChaoZuZhi2, Unigene63391\_JingChaoZuZhi2, Unigene66535\_JingChaoZuZhi2, Unigene70308\_JingChaoZuZhi2, Unigene73896\_JingChaoZuZhi2, Unigene78518\_JingChaoZuZhi2, Unigene80275\_JingChaoZuZhi2, Unigene81692\_JingChaoZuZhi2, Unigene83846\_JingChaoZuZhi2, Unigene84686\_JingChaoZuZhi2, Unigene85789\_JingChaoZuZhi2, Unigene86634\_JingChaoZuZhi2, Unigene87347\_JingChaoZuZhi2, Unigene87834\_JingChaoZuZhi2, Unigene88262\_JingChaoZuZhi2, Unigene88774\_JingChaoZuZhi2, Unigene89128\_JingChaoZuZhi2, Unigene89595\_JingChaoZuZhi2, Unigene91344\_JingChaoZuZhi2, Unigene91490\_JingChaoZuZhi2, Unigene92452\_JingChaoZuZhi2, Unigene93785\_JingChaoZuZhi2, Unigene9446\_JingChaoZuZhi2, Unigene94552\_JingChaoZuZhi2, Unigene95063\_JingChaoZuZhi2, Unigene95331\_JingChaoZuZhi2, Unigene96002\_JingChaoZuZhi2, Unigene96229\_JingChaoZuZhi2 |
| 157 | alpha-Linolenic acid metabolism | Unigene11055\_JingChaoZuZhi2, Unigene12083\_JingChaoZuZhi2, Unigene12260\_JingChaoZuZhi2, Unigene13420\_JingChaoZuZhi2, Unigene13869\_JingChaoZuZhi2, Unigene14357\_JingChaoZuZhi2, Unigene15425\_JingChaoZuZhi2, Unigene15942\_JingChaoZuZhi2, Unigene16663\_JingChaoZuZhi2, Unigene16899\_JingChaoZuZhi2, Unigene18846\_JingChaoZuZhi2, Unigene19904\_JingChaoZuZhi2, Unigene20071\_JingChaoZuZhi2, Unigene2058\_JingChaoZuZhi2, Unigene20760\_JingChaoZuZhi2, Unigene21407\_JingChaoZuZhi2, Unigene22352\_JingChaoZuZhi2, Unigene35187\_JingChaoZuZhi2, Unigene42329\_JingChaoZuZhi2, Unigene43723\_JingChaoZuZhi2, Unigene45176\_JingChaoZuZhi2, Unigene45532\_JingChaoZuZhi2, Unigene49819\_JingChaoZuZhi2, Unigene59548\_JingChaoZuZhi2, Unigene60117\_JingChaoZuZhi2, Unigene61162\_JingChaoZuZhi2, Unigene62962\_JingChaoZuZhi2, Unigene65441\_JingChaoZuZhi2, Unigene67328\_JingChaoZuZhi2, Unigene68151\_JingChaoZuZhi2, Unigene72098\_JingChaoZuZhi2, Unigene72974\_JingChaoZuZhi2, Unigene74594\_JingChaoZuZhi2, Unigene76182\_JingChaoZuZhi2, Unigene78854\_JingChaoZuZhi2, Unigene79135\_JingChaoZuZhi2, Unigene80446\_JingChaoZuZhi2, Unigene8213\_JingChaoZuZhi2, Unigene82435\_JingChaoZuZhi2, Unigene84124\_JingChaoZuZhi2, Unigene85989\_JingChaoZuZhi2, Unigene88068\_JingChaoZuZhi2, Unigene88377\_JingChaoZuZhi2, Unigene89232\_JingChaoZuZhi2, Unigene90531\_JingChaoZuZhi2, Unigene92737\_JingChaoZuZhi2, Unigene93810\_JingChaoZuZhi2, Unigene9\_JingChaoZuZhi2 |
| 158 | Other glycan degradation (no map in kegg database) | Unigene11681\_JingChaoZuZhi2, Unigene12412\_JingChaoZuZhi2, Unigene12520\_JingChaoZuZhi2, Unigene14812\_JingChaoZuZhi2, Unigene15633\_JingChaoZuZhi2, Unigene16761\_JingChaoZuZhi2, Unigene16955\_JingChaoZuZhi2, Unigene19251\_JingChaoZuZhi2, Unigene22281\_JingChaoZuZhi2, Unigene2535\_JingChaoZuZhi2, Unigene26787\_JingChaoZuZhi2, Unigene27220\_JingChaoZuZhi2, Unigene35048\_JingChaoZuZhi2, Unigene42\_JingChaoZuZhi2, Unigene4438\_JingChaoZuZhi2, Unigene45407\_JingChaoZuZhi2, Unigene46156\_JingChaoZuZhi2, Unigene49877\_JingChaoZuZhi2, Unigene51610\_JingChaoZuZhi2, Unigene60942\_JingChaoZuZhi2, Unigene62146\_JingChaoZuZhi2, Unigene67946\_JingChaoZuZhi2, Unigene7059\_JingChaoZuZhi2, Unigene71248\_JingChaoZuZhi2, Unigene72266\_JingChaoZuZhi2, Unigene72924\_JingChaoZuZhi2, Unigene74009\_JingChaoZuZhi2, Unigene76927\_JingChaoZuZhi2, Unigene78418\_JingChaoZuZhi2, Unigene78720\_JingChaoZuZhi2, Unigene80358\_JingChaoZuZhi2, Unigene80848\_JingChaoZuZhi2, Unigene80928\_JingChaoZuZhi2, Unigene81654\_JingChaoZuZhi2, Unigene82258\_JingChaoZuZhi2, Unigene83023\_JingChaoZuZhi2, Unigene85353\_JingChaoZuZhi2, Unigene86174\_JingChaoZuZhi2, Unigene86892\_JingChaoZuZhi2, Unigene87062\_JingChaoZuZhi2, Unigene87341\_JingChaoZuZhi2, Unigene88528\_JingChaoZuZhi2, Unigene91242\_JingChaoZuZhi2, Unigene91460\_JingChaoZuZhi2, Unigene92149\_JingChaoZuZhi2, Unigene94655\_JingChaoZuZhi2, Unigene95275\_JingChaoZuZhi2, Unigene96039\_JingChaoZuZhi2 |
| 159 | Renin-angiotensin system | Unigene1478\_JingChaoZuZhi2, Unigene16073\_JingChaoZuZhi2, Unigene16549\_JingChaoZuZhi2, Unigene16633\_JingChaoZuZhi2, Unigene16764\_JingChaoZuZhi2, Unigene20429\_JingChaoZuZhi2, Unigene21281\_JingChaoZuZhi2, Unigene22035\_JingChaoZuZhi2, Unigene22293\_JingChaoZuZhi2, Unigene26903\_JingChaoZuZhi2, Unigene29179\_JingChaoZuZhi2, Unigene3061\_JingChaoZuZhi2, Unigene34264\_JingChaoZuZhi2, Unigene35574\_JingChaoZuZhi2, Unigene39201\_JingChaoZuZhi2, Unigene41749\_JingChaoZuZhi2, Unigene42718\_JingChaoZuZhi2, Unigene45216\_JingChaoZuZhi2, Unigene4542\_JingChaoZuZhi2, Unigene49977\_JingChaoZuZhi2, Unigene50252\_JingChaoZuZhi2, Unigene54066\_JingChaoZuZhi2, Unigene55482\_JingChaoZuZhi2, Unigene5714\_JingChaoZuZhi2, Unigene57596\_JingChaoZuZhi2, Unigene60905\_JingChaoZuZhi2, Unigene62191\_JingChaoZuZhi2, Unigene64846\_JingChaoZuZhi2, Unigene65447\_JingChaoZuZhi2, Unigene65960\_JingChaoZuZhi2, Unigene66772\_JingChaoZuZhi2, Unigene69427\_JingChaoZuZhi2, Unigene73882\_JingChaoZuZhi2, Unigene75580\_JingChaoZuZhi2, Unigene76514\_JingChaoZuZhi2, Unigene78367\_JingChaoZuZhi2, Unigene81935\_JingChaoZuZhi2, Unigene81947\_JingChaoZuZhi2, Unigene82234\_JingChaoZuZhi2, Unigene8784\_JingChaoZuZhi2, Unigene89805\_JingChaoZuZhi2, Unigene90999\_JingChaoZuZhi2, Unigene91115\_JingChaoZuZhi2, Unigene91914\_JingChaoZuZhi2, Unigene95550\_JingChaoZuZhi2, Unigene96201\_JingChaoZuZhi2, Unigene96284\_JingChaoZuZhi2 |
| 160 | Thyroid cancer | Unigene10876\_JingChaoZuZhi2, Unigene12202\_JingChaoZuZhi2, Unigene14534\_JingChaoZuZhi2, Unigene16229\_JingChaoZuZhi2, Unigene16551\_JingChaoZuZhi2, Unigene18438\_JingChaoZuZhi2, Unigene19150\_JingChaoZuZhi2, Unigene19836\_JingChaoZuZhi2, Unigene21513\_JingChaoZuZhi2, Unigene21956\_JingChaoZuZhi2, Unigene22054\_JingChaoZuZhi2, Unigene3452\_JingChaoZuZhi2, Unigene3665\_JingChaoZuZhi2, Unigene3957\_JingChaoZuZhi2, Unigene46160\_JingChaoZuZhi2, Unigene50645\_JingChaoZuZhi2, Unigene5798\_JingChaoZuZhi2, Unigene6028\_JingChaoZuZhi2, Unigene61911\_JingChaoZuZhi2, Unigene63449\_JingChaoZuZhi2, Unigene64227\_JingChaoZuZhi2, Unigene6785\_JingChaoZuZhi2, Unigene6857\_JingChaoZuZhi2, Unigene70100\_JingChaoZuZhi2, Unigene70549\_JingChaoZuZhi2, Unigene8191\_JingChaoZuZhi2, Unigene84930\_JingChaoZuZhi2, Unigene8649\_JingChaoZuZhi2, Unigene87122\_JingChaoZuZhi2, Unigene87261\_JingChaoZuZhi2, Unigene876\_JingChaoZuZhi2, Unigene88040\_JingChaoZuZhi2, Unigene88470\_JingChaoZuZhi2, Unigene88544\_JingChaoZuZhi2, Unigene88550\_JingChaoZuZhi2, Unigene90495\_JingChaoZuZhi2, Unigene90927\_JingChaoZuZhi2, Unigene92794\_JingChaoZuZhi2, Unigene93199\_JingChaoZuZhi2, Unigene93257\_JingChaoZuZhi2, Unigene94272\_JingChaoZuZhi2, Unigene94656\_JingChaoZuZhi2, Unigene95463\_JingChaoZuZhi2, Unigene95485\_JingChaoZuZhi2, Unigene96234\_JingChaoZuZhi2, Unigene9643\_JingChaoZuZhi2 |
| 161 | SNARE interactions in vesicular transport | Unigene10503\_JingChaoZuZhi2, Unigene1149\_JingChaoZuZhi2, Unigene14144\_JingChaoZuZhi2, Unigene15130\_JingChaoZuZhi2, Unigene15415\_JingChaoZuZhi2, Unigene15598\_JingChaoZuZhi2, Unigene15811\_JingChaoZuZhi2, Unigene16388\_JingChaoZuZhi2, Unigene17813\_JingChaoZuZhi2, Unigene19951\_JingChaoZuZhi2, Unigene21553\_JingChaoZuZhi2, Unigene21713\_JingChaoZuZhi2, Unigene21778\_JingChaoZuZhi2, Unigene22583\_JingChaoZuZhi2, Unigene24425\_JingChaoZuZhi2, Unigene3022\_JingChaoZuZhi2, Unigene31416\_JingChaoZuZhi2, Unigene3245\_JingChaoZuZhi2, Unigene32999\_JingChaoZuZhi2, Unigene3345\_JingChaoZuZhi2, Unigene39962\_JingChaoZuZhi2, Unigene44625\_JingChaoZuZhi2, Unigene48077\_JingChaoZuZhi2, Unigene52058\_JingChaoZuZhi2, Unigene54233\_JingChaoZuZhi2, Unigene5724\_JingChaoZuZhi2, Unigene6863\_JingChaoZuZhi2, Unigene7398\_JingChaoZuZhi2, Unigene74093\_JingChaoZuZhi2, Unigene7441\_JingChaoZuZhi2, Unigene75316\_JingChaoZuZhi2, Unigene7571\_JingChaoZuZhi2, Unigene79595\_JingChaoZuZhi2, Unigene79649\_JingChaoZuZhi2, Unigene80070\_JingChaoZuZhi2, Unigene83355\_JingChaoZuZhi2, Unigene88916\_JingChaoZuZhi2, Unigene91391\_JingChaoZuZhi2, Unigene91953\_JingChaoZuZhi2, Unigene92867\_JingChaoZuZhi2, Unigene93319\_JingChaoZuZhi2, Unigene93840\_JingChaoZuZhi2, Unigene94150\_JingChaoZuZhi2, Unigene94839\_JingChaoZuZhi2, Unigene95180\_JingChaoZuZhi2, Unigene95363\_JingChaoZuZhi2 |
| 162 | Glycosaminoglycan biosynthesis - chondroitin sulfate | Unigene11412\_JingChaoZuZhi2, Unigene11872\_JingChaoZuZhi2, Unigene12356\_JingChaoZuZhi2, Unigene14419\_JingChaoZuZhi2, Unigene15537\_JingChaoZuZhi2, Unigene15802\_JingChaoZuZhi2, Unigene16945\_JingChaoZuZhi2, Unigene20729\_JingChaoZuZhi2, Unigene20786\_JingChaoZuZhi2, Unigene21828\_JingChaoZuZhi2, Unigene22222\_JingChaoZuZhi2, Unigene22726\_JingChaoZuZhi2, Unigene26507\_JingChaoZuZhi2, Unigene31036\_JingChaoZuZhi2, Unigene42144\_JingChaoZuZhi2, Unigene44707\_JingChaoZuZhi2, Unigene55539\_JingChaoZuZhi2, Unigene63067\_JingChaoZuZhi2, Unigene69579\_JingChaoZuZhi2, Unigene70315\_JingChaoZuZhi2, Unigene72508\_JingChaoZuZhi2, Unigene75124\_JingChaoZuZhi2, Unigene75651\_JingChaoZuZhi2, Unigene79757\_JingChaoZuZhi2, Unigene79879\_JingChaoZuZhi2, Unigene80328\_JingChaoZuZhi2, Unigene80635\_JingChaoZuZhi2, Unigene81997\_JingChaoZuZhi2, Unigene82901\_JingChaoZuZhi2, Unigene84224\_JingChaoZuZhi2, Unigene85261\_JingChaoZuZhi2, Unigene86319\_JingChaoZuZhi2, Unigene8781\_JingChaoZuZhi2, Unigene88888\_JingChaoZuZhi2, Unigene89341\_JingChaoZuZhi2, Unigene8984\_JingChaoZuZhi2, Unigene91654\_JingChaoZuZhi2, Unigene91818\_JingChaoZuZhi2, Unigene9314\_JingChaoZuZhi2, Unigene93980\_JingChaoZuZhi2, Unigene95247\_JingChaoZuZhi2, Unigene95298\_JingChaoZuZhi2, Unigene95525\_JingChaoZuZhi2, Unigene95655\_JingChaoZuZhi2 |
| 163 | Bladder cancer | Unigene10286\_JingChaoZuZhi2, Unigene10339\_JingChaoZuZhi2, Unigene10422\_JingChaoZuZhi2, Unigene12271\_JingChaoZuZhi2, Unigene14151\_JingChaoZuZhi2, Unigene14433\_JingChaoZuZhi2, Unigene16516\_JingChaoZuZhi2, Unigene17467\_JingChaoZuZhi2, Unigene18309\_JingChaoZuZhi2, Unigene18760\_JingChaoZuZhi2, Unigene20297\_JingChaoZuZhi2, Unigene21513\_JingChaoZuZhi2, Unigene24196\_JingChaoZuZhi2, Unigene2790\_JingChaoZuZhi2, Unigene35236\_JingChaoZuZhi2, Unigene3665\_JingChaoZuZhi2, Unigene39637\_JingChaoZuZhi2, Unigene39876\_JingChaoZuZhi2, Unigene4024\_JingChaoZuZhi2, Unigene49798\_JingChaoZuZhi2, Unigene51285\_JingChaoZuZhi2, Unigene5364\_JingChaoZuZhi2, Unigene54470\_JingChaoZuZhi2, Unigene56642\_JingChaoZuZhi2, Unigene5758\_JingChaoZuZhi2, Unigene6028\_JingChaoZuZhi2, Unigene62786\_JingChaoZuZhi2, Unigene6785\_JingChaoZuZhi2, Unigene70549\_JingChaoZuZhi2, Unigene7068\_JingChaoZuZhi2, Unigene72521\_JingChaoZuZhi2, Unigene78574\_JingChaoZuZhi2, Unigene80305\_JingChaoZuZhi2, Unigene8191\_JingChaoZuZhi2, Unigene83700\_JingChaoZuZhi2, Unigene84930\_JingChaoZuZhi2, Unigene876\_JingChaoZuZhi2, Unigene88343\_JingChaoZuZhi2, Unigene91038\_JingChaoZuZhi2, Unigene92839\_JingChaoZuZhi2, Unigene93284\_JingChaoZuZhi2, Unigene96084\_JingChaoZuZhi2, Unigene96281\_JingChaoZuZhi2, Unigene9902\_JingChaoZuZhi2 |
| 164 | Glycosylphosphatidylinositol(GPI)-anchor biosynthesis | Unigene13191\_JingChaoZuZhi2, Unigene13693\_JingChaoZuZhi2, Unigene13740\_JingChaoZuZhi2, Unigene19853\_JingChaoZuZhi2, Unigene21065\_JingChaoZuZhi2, Unigene26170\_JingChaoZuZhi2, Unigene29994\_JingChaoZuZhi2, Unigene36973\_JingChaoZuZhi2, Unigene44544\_JingChaoZuZhi2, Unigene47424\_JingChaoZuZhi2, Unigene52572\_JingChaoZuZhi2, Unigene55656\_JingChaoZuZhi2, Unigene59038\_JingChaoZuZhi2, Unigene59570\_JingChaoZuZhi2, Unigene60894\_JingChaoZuZhi2, Unigene63705\_JingChaoZuZhi2, Unigene63917\_JingChaoZuZhi2, Unigene64505\_JingChaoZuZhi2, Unigene68045\_JingChaoZuZhi2, Unigene69639\_JingChaoZuZhi2, Unigene70779\_JingChaoZuZhi2, Unigene74612\_JingChaoZuZhi2, Unigene7537\_JingChaoZuZhi2, Unigene75459\_JingChaoZuZhi2, Unigene75828\_JingChaoZuZhi2, Unigene76892\_JingChaoZuZhi2, Unigene7899\_JingChaoZuZhi2, Unigene82817\_JingChaoZuZhi2, Unigene83704\_JingChaoZuZhi2, Unigene85449\_JingChaoZuZhi2, Unigene85591\_JingChaoZuZhi2, Unigene8712\_JingChaoZuZhi2, Unigene88429\_JingChaoZuZhi2, Unigene88523\_JingChaoZuZhi2, Unigene90656\_JingChaoZuZhi2, Unigene91995\_JingChaoZuZhi2, Unigene94932\_JingChaoZuZhi2, Unigene95081\_JingChaoZuZhi2, Unigene95083\_JingChaoZuZhi2, Unigene95090\_JingChaoZuZhi2, Unigene9523\_JingChaoZuZhi2, Unigene96268\_JingChaoZuZhi2, Unigene9634\_JingChaoZuZhi2 |
| 165 | Proximal tubule bicarbonate reclamation | Unigene14802\_JingChaoZuZhi2, Unigene18977\_JingChaoZuZhi2, Unigene20651\_JingChaoZuZhi2, Unigene21792\_JingChaoZuZhi2, Unigene21866\_JingChaoZuZhi2, Unigene28522\_JingChaoZuZhi2, Unigene30780\_JingChaoZuZhi2, Unigene31660\_JingChaoZuZhi2, Unigene32031\_JingChaoZuZhi2, Unigene39299\_JingChaoZuZhi2, Unigene42033\_JingChaoZuZhi2, Unigene42036\_JingChaoZuZhi2, Unigene49757\_JingChaoZuZhi2, Unigene51478\_JingChaoZuZhi2, Unigene5545\_JingChaoZuZhi2, Unigene56513\_JingChaoZuZhi2, Unigene5799\_JingChaoZuZhi2, Unigene63391\_JingChaoZuZhi2, Unigene69271\_JingChaoZuZhi2, Unigene70147\_JingChaoZuZhi2, Unigene72182\_JingChaoZuZhi2, Unigene73896\_JingChaoZuZhi2, Unigene77255\_JingChaoZuZhi2, Unigene80275\_JingChaoZuZhi2, Unigene80504\_JingChaoZuZhi2, Unigene81692\_JingChaoZuZhi2, Unigene83531\_JingChaoZuZhi2, Unigene83602\_JingChaoZuZhi2, Unigene83716\_JingChaoZuZhi2, Unigene84232\_JingChaoZuZhi2, Unigene84340\_JingChaoZuZhi2, Unigene91175\_JingChaoZuZhi2, Unigene91344\_JingChaoZuZhi2, Unigene93218\_JingChaoZuZhi2, Unigene94081\_JingChaoZuZhi2, Unigene94279\_JingChaoZuZhi2, Unigene9446\_JingChaoZuZhi2, Unigene94745\_JingChaoZuZhi2, Unigene95063\_JingChaoZuZhi2, Unigene95776\_JingChaoZuZhi2, Unigene95958\_JingChaoZuZhi2, Unigene96206\_JingChaoZuZhi2 |
| 166 | Porphyrin and chlorophyll metabolism | Unigene16415\_JingChaoZuZhi2, Unigene17670\_JingChaoZuZhi2, Unigene18991\_JingChaoZuZhi2, Unigene19140\_JingChaoZuZhi2, Unigene19811\_JingChaoZuZhi2, Unigene20357\_JingChaoZuZhi2, Unigene20676\_JingChaoZuZhi2, Unigene20742\_JingChaoZuZhi2, Unigene21967\_JingChaoZuZhi2, Unigene22121\_JingChaoZuZhi2, Unigene23389\_JingChaoZuZhi2, Unigene248\_JingChaoZuZhi2, Unigene54669\_JingChaoZuZhi2, Unigene552\_JingChaoZuZhi2, Unigene57061\_JingChaoZuZhi2, Unigene57130\_JingChaoZuZhi2, Unigene5957\_JingChaoZuZhi2, Unigene60406\_JingChaoZuZhi2, Unigene65843\_JingChaoZuZhi2, Unigene684\_JingChaoZuZhi2, Unigene70615\_JingChaoZuZhi2, Unigene71976\_JingChaoZuZhi2, Unigene73857\_JingChaoZuZhi2, Unigene79198\_JingChaoZuZhi2, Unigene79698\_JingChaoZuZhi2, Unigene80285\_JingChaoZuZhi2, Unigene82503\_JingChaoZuZhi2, Unigene82818\_JingChaoZuZhi2, Unigene84385\_JingChaoZuZhi2, Unigene85814\_JingChaoZuZhi2, Unigene85939\_JingChaoZuZhi2, Unigene87046\_JingChaoZuZhi2, Unigene88475\_JingChaoZuZhi2, Unigene89186\_JingChaoZuZhi2, Unigene89887\_JingChaoZuZhi2, Unigene92799\_JingChaoZuZhi2, Unigene93355\_JingChaoZuZhi2, Unigene93542\_JingChaoZuZhi2, Unigene95345\_JingChaoZuZhi2, Unigene95482\_JingChaoZuZhi2, Unigene95612\_JingChaoZuZhi2 |
| 167 | Glycosaminoglycan degradation | Unigene11462\_JingChaoZuZhi2, Unigene11681\_JingChaoZuZhi2, Unigene144\_JingChaoZuZhi2, Unigene16795\_JingChaoZuZhi2, Unigene18991\_JingChaoZuZhi2, Unigene19140\_JingChaoZuZhi2, Unigene19251\_JingChaoZuZhi2, Unigene19428\_JingChaoZuZhi2, Unigene22838\_JingChaoZuZhi2, Unigene33238\_JingChaoZuZhi2, Unigene44000\_JingChaoZuZhi2, Unigene4431\_JingChaoZuZhi2, Unigene47051\_JingChaoZuZhi2, Unigene47300\_JingChaoZuZhi2, Unigene51610\_JingChaoZuZhi2, Unigene52142\_JingChaoZuZhi2, Unigene523\_JingChaoZuZhi2, Unigene59000\_JingChaoZuZhi2, Unigene59210\_JingChaoZuZhi2, Unigene5957\_JingChaoZuZhi2, Unigene63747\_JingChaoZuZhi2, Unigene67946\_JingChaoZuZhi2, Unigene7059\_JingChaoZuZhi2, Unigene71248\_JingChaoZuZhi2, Unigene72924\_JingChaoZuZhi2, Unigene77903\_JingChaoZuZhi2, Unigene79288\_JingChaoZuZhi2, Unigene80848\_JingChaoZuZhi2, Unigene81654\_JingChaoZuZhi2, Unigene81661\_JingChaoZuZhi2, Unigene83023\_JingChaoZuZhi2, Unigene84626\_JingChaoZuZhi2, Unigene85353\_JingChaoZuZhi2, Unigene87007\_JingChaoZuZhi2, Unigene87046\_JingChaoZuZhi2, Unigene88528\_JingChaoZuZhi2, Unigene89882\_JingChaoZuZhi2, Unigene93425\_JingChaoZuZhi2, Unigene93837\_JingChaoZuZhi2, Unigene93936\_JingChaoZuZhi2, Unigene96039\_JingChaoZuZhi2 |
| 168 | Pentose and glucuronate interconversions | Unigene13176\_JingChaoZuZhi2, Unigene18991\_JingChaoZuZhi2, Unigene19140\_JingChaoZuZhi2, Unigene19778\_JingChaoZuZhi2, Unigene22591\_JingChaoZuZhi2, Unigene24676\_JingChaoZuZhi2, Unigene2474\_JingChaoZuZhi2, Unigene3955\_JingChaoZuZhi2, Unigene42670\_JingChaoZuZhi2, Unigene4377\_JingChaoZuZhi2, Unigene45887\_JingChaoZuZhi2, Unigene49290\_JingChaoZuZhi2, Unigene53809\_JingChaoZuZhi2, Unigene54539\_JingChaoZuZhi2, Unigene57061\_JingChaoZuZhi2, Unigene57130\_JingChaoZuZhi2, Unigene5957\_JingChaoZuZhi2, Unigene59735\_JingChaoZuZhi2, Unigene59979\_JingChaoZuZhi2, Unigene69601\_JingChaoZuZhi2, Unigene71976\_JingChaoZuZhi2, Unigene7200\_JingChaoZuZhi2, Unigene73451\_JingChaoZuZhi2, Unigene74590\_JingChaoZuZhi2, Unigene76593\_JingChaoZuZhi2, Unigene79198\_JingChaoZuZhi2, Unigene80141\_JingChaoZuZhi2, Unigene80285\_JingChaoZuZhi2, Unigene82270\_JingChaoZuZhi2, Unigene82808\_JingChaoZuZhi2, Unigene82818\_JingChaoZuZhi2, Unigene84385\_JingChaoZuZhi2, Unigene87046\_JingChaoZuZhi2, Unigene87676\_JingChaoZuZhi2, Unigene88238\_JingChaoZuZhi2, Unigene90268\_JingChaoZuZhi2, Unigene91969\_JingChaoZuZhi2, Unigene93877\_JingChaoZuZhi2, Unigene94925\_JingChaoZuZhi2, Unigene95057\_JingChaoZuZhi2, Unigene95818\_JingChaoZuZhi2 |
| 169 | Glycosaminoglycan biosynthesis - heparan sulfate | Unigene14419\_JingChaoZuZhi2, Unigene15802\_JingChaoZuZhi2, Unigene16845\_JingChaoZuZhi2, Unigene19052\_JingChaoZuZhi2, Unigene21828\_JingChaoZuZhi2, Unigene25319\_JingChaoZuZhi2, Unigene26507\_JingChaoZuZhi2, Unigene29618\_JingChaoZuZhi2, Unigene3336\_JingChaoZuZhi2, Unigene39331\_JingChaoZuZhi2, Unigene47814\_JingChaoZuZhi2, Unigene48636\_JingChaoZuZhi2, Unigene48958\_JingChaoZuZhi2, Unigene49623\_JingChaoZuZhi2, Unigene49703\_JingChaoZuZhi2, Unigene50361\_JingChaoZuZhi2, Unigene51688\_JingChaoZuZhi2, Unigene54009\_JingChaoZuZhi2, Unigene54707\_JingChaoZuZhi2, Unigene60781\_JingChaoZuZhi2, Unigene68920\_JingChaoZuZhi2, Unigene70315\_JingChaoZuZhi2, Unigene72508\_JingChaoZuZhi2, Unigene73561\_JingChaoZuZhi2, Unigene74517\_JingChaoZuZhi2, Unigene75008\_JingChaoZuZhi2, Unigene77779\_JingChaoZuZhi2, Unigene78060\_JingChaoZuZhi2, Unigene80008\_JingChaoZuZhi2, Unigene81997\_JingChaoZuZhi2, Unigene82901\_JingChaoZuZhi2, Unigene84220\_JingChaoZuZhi2, Unigene84224\_JingChaoZuZhi2, Unigene88937\_JingChaoZuZhi2, Unigene8984\_JingChaoZuZhi2, Unigene91818\_JingChaoZuZhi2, Unigene91950\_JingChaoZuZhi2, Unigene93980\_JingChaoZuZhi2, Unigene95298\_JingChaoZuZhi2, Unigene95655\_JingChaoZuZhi2, Unigene95797\_JingChaoZuZhi2 |
| 170 | O-Glycan biosynthesis | Unigene10829\_JingChaoZuZhi2, Unigene12129\_JingChaoZuZhi2, Unigene14913\_JingChaoZuZhi2, Unigene21158\_JingChaoZuZhi2, Unigene22197\_JingChaoZuZhi2, Unigene30697\_JingChaoZuZhi2, Unigene30782\_JingChaoZuZhi2, Unigene3128\_JingChaoZuZhi2, Unigene35544\_JingChaoZuZhi2, Unigene37589\_JingChaoZuZhi2, Unigene38285\_JingChaoZuZhi2, Unigene40527\_JingChaoZuZhi2, Unigene40607\_JingChaoZuZhi2, Unigene41427\_JingChaoZuZhi2, Unigene50284\_JingChaoZuZhi2, Unigene50538\_JingChaoZuZhi2, Unigene53966\_JingChaoZuZhi2, Unigene55268\_JingChaoZuZhi2, Unigene56961\_JingChaoZuZhi2, Unigene57511\_JingChaoZuZhi2, Unigene61380\_JingChaoZuZhi2, Unigene62994\_JingChaoZuZhi2, Unigene634\_JingChaoZuZhi2, Unigene69314\_JingChaoZuZhi2, Unigene75625\_JingChaoZuZhi2, Unigene81536\_JingChaoZuZhi2, Unigene82158\_JingChaoZuZhi2, Unigene84523\_JingChaoZuZhi2, Unigene85742\_JingChaoZuZhi2, Unigene86198\_JingChaoZuZhi2, Unigene86568\_JingChaoZuZhi2, Unigene87931\_JingChaoZuZhi2, Unigene88795\_JingChaoZuZhi2, Unigene88996\_JingChaoZuZhi2, Unigene90926\_JingChaoZuZhi2, Unigene94913\_JingChaoZuZhi2, Unigene95192\_JingChaoZuZhi2, Unigene95361\_JingChaoZuZhi2, Unigene95887\_JingChaoZuZhi2, Unigene96106\_JingChaoZuZhi2 |
| 171 | One carbon pool by folate | Unigene10394\_JingChaoZuZhi2, Unigene11433\_JingChaoZuZhi2, Unigene19246\_JingChaoZuZhi2, Unigene19644\_JingChaoZuZhi2, Unigene19893\_JingChaoZuZhi2, Unigene21092\_JingChaoZuZhi2, Unigene21391\_JingChaoZuZhi2, Unigene30051\_JingChaoZuZhi2, Unigene31282\_JingChaoZuZhi2, Unigene3871\_JingChaoZuZhi2, Unigene43658\_JingChaoZuZhi2, Unigene44196\_JingChaoZuZhi2, Unigene45512\_JingChaoZuZhi2, Unigene60108\_JingChaoZuZhi2, Unigene61679\_JingChaoZuZhi2, Unigene6246\_JingChaoZuZhi2, Unigene67411\_JingChaoZuZhi2, Unigene69155\_JingChaoZuZhi2, Unigene69752\_JingChaoZuZhi2, Unigene74390\_JingChaoZuZhi2, Unigene76424\_JingChaoZuZhi2, Unigene79622\_JingChaoZuZhi2, Unigene80222\_JingChaoZuZhi2, Unigene80622\_JingChaoZuZhi2, Unigene8175\_JingChaoZuZhi2, Unigene83854\_JingChaoZuZhi2, Unigene83939\_JingChaoZuZhi2, Unigene83966\_JingChaoZuZhi2, Unigene84279\_JingChaoZuZhi2, Unigene84532\_JingChaoZuZhi2, Unigene84851\_JingChaoZuZhi2, Unigene87480\_JingChaoZuZhi2, Unigene87664\_JingChaoZuZhi2, Unigene88792\_JingChaoZuZhi2, Unigene90127\_JingChaoZuZhi2, Unigene90392\_JingChaoZuZhi2, Unigene92027\_JingChaoZuZhi2, Unigene93414\_JingChaoZuZhi2, Unigene9528\_JingChaoZuZhi2, Unigene95765\_JingChaoZuZhi2 |
| 172 | Taste transduction | Unigene12938\_JingChaoZuZhi2, Unigene13770\_JingChaoZuZhi2, Unigene13996\_JingChaoZuZhi2, Unigene14360\_JingChaoZuZhi2, Unigene15651\_JingChaoZuZhi2, Unigene16991\_JingChaoZuZhi2, Unigene18307\_JingChaoZuZhi2, Unigene19730\_JingChaoZuZhi2, Unigene21815\_JingChaoZuZhi2, Unigene22426\_JingChaoZuZhi2, Unigene35952\_JingChaoZuZhi2, Unigene39832\_JingChaoZuZhi2, Unigene41333\_JingChaoZuZhi2, Unigene48499\_JingChaoZuZhi2, Unigene51991\_JingChaoZuZhi2, Unigene54367\_JingChaoZuZhi2, Unigene56709\_JingChaoZuZhi2, Unigene65445\_JingChaoZuZhi2, Unigene66540\_JingChaoZuZhi2, Unigene68300\_JingChaoZuZhi2, Unigene70087\_JingChaoZuZhi2, Unigene7456\_JingChaoZuZhi2, Unigene76495\_JingChaoZuZhi2, Unigene77149\_JingChaoZuZhi2, Unigene77588\_JingChaoZuZhi2, Unigene78078\_JingChaoZuZhi2, Unigene78235\_JingChaoZuZhi2, Unigene79689\_JingChaoZuZhi2, Unigene81901\_JingChaoZuZhi2, Unigene82368\_JingChaoZuZhi2, Unigene83166\_JingChaoZuZhi2, Unigene87607\_JingChaoZuZhi2, Unigene88385\_JingChaoZuZhi2, Unigene88773\_JingChaoZuZhi2, Unigene89807\_JingChaoZuZhi2, Unigene90228\_JingChaoZuZhi2, Unigene91527\_JingChaoZuZhi2, Unigene91739\_JingChaoZuZhi2, Unigene95741\_JingChaoZuZhi2, Unigene95916\_JingChaoZuZhi2 |
| 173 | Protein export | Unigene15846\_JingChaoZuZhi2, Unigene18547\_JingChaoZuZhi2, Unigene22843\_JingChaoZuZhi2, Unigene26293\_JingChaoZuZhi2, Unigene28672\_JingChaoZuZhi2, Unigene36859\_JingChaoZuZhi2, Unigene37667\_JingChaoZuZhi2, Unigene38873\_JingChaoZuZhi2, Unigene64670\_JingChaoZuZhi2, Unigene700\_JingChaoZuZhi2, Unigene72904\_JingChaoZuZhi2, Unigene74882\_JingChaoZuZhi2, Unigene74963\_JingChaoZuZhi2, Unigene77102\_JingChaoZuZhi2, Unigene77479\_JingChaoZuZhi2, Unigene82758\_JingChaoZuZhi2, Unigene83352\_JingChaoZuZhi2, Unigene84705\_JingChaoZuZhi2, Unigene85585\_JingChaoZuZhi2, Unigene86975\_JingChaoZuZhi2, Unigene87594\_JingChaoZuZhi2, Unigene88129\_JingChaoZuZhi2, Unigene88646\_JingChaoZuZhi2, Unigene89103\_JingChaoZuZhi2, Unigene89354\_JingChaoZuZhi2, Unigene89939\_JingChaoZuZhi2, Unigene90940\_JingChaoZuZhi2, Unigene91769\_JingChaoZuZhi2, Unigene92322\_JingChaoZuZhi2, Unigene92631\_JingChaoZuZhi2, Unigene92768\_JingChaoZuZhi2, Unigene93217\_JingChaoZuZhi2, Unigene94764\_JingChaoZuZhi2, Unigene95185\_JingChaoZuZhi2, Unigene95449\_JingChaoZuZhi2, Unigene95496\_JingChaoZuZhi2, Unigene9549\_JingChaoZuZhi2, Unigene95917\_JingChaoZuZhi2, Unigene96059\_JingChaoZuZhi2 |
| 174 | Steroid hormone biosynthesis | Unigene13068\_JingChaoZuZhi2, Unigene13160\_JingChaoZuZhi2, Unigene14132\_JingChaoZuZhi2, Unigene14448\_JingChaoZuZhi2, Unigene17089\_JingChaoZuZhi2, Unigene18163\_JingChaoZuZhi2, Unigene18284\_JingChaoZuZhi2, Unigene18576\_JingChaoZuZhi2, Unigene20264\_JingChaoZuZhi2, Unigene20504\_JingChaoZuZhi2, Unigene22492\_JingChaoZuZhi2, Unigene48173\_JingChaoZuZhi2, Unigene48343\_JingChaoZuZhi2, Unigene50305\_JingChaoZuZhi2, Unigene53447\_JingChaoZuZhi2, Unigene56370\_JingChaoZuZhi2, Unigene57061\_JingChaoZuZhi2, Unigene57130\_JingChaoZuZhi2, Unigene59427\_JingChaoZuZhi2, Unigene60494\_JingChaoZuZhi2, Unigene66888\_JingChaoZuZhi2, Unigene71976\_JingChaoZuZhi2, Unigene75891\_JingChaoZuZhi2, Unigene79198\_JingChaoZuZhi2, Unigene80285\_JingChaoZuZhi2, Unigene81630\_JingChaoZuZhi2, Unigene81878\_JingChaoZuZhi2, Unigene82170\_JingChaoZuZhi2, Unigene82297\_JingChaoZuZhi2, Unigene82818\_JingChaoZuZhi2, Unigene83273\_JingChaoZuZhi2, Unigene84385\_JingChaoZuZhi2, Unigene85539\_JingChaoZuZhi2, Unigene88383\_JingChaoZuZhi2, Unigene90949\_JingChaoZuZhi2, Unigene92281\_JingChaoZuZhi2, Unigene93039\_JingChaoZuZhi2, Unigene93255\_JingChaoZuZhi2, Unigene94422\_JingChaoZuZhi2 |
| 175 | Ascorbate and aldarate metabolism | Unigene13176\_JingChaoZuZhi2, Unigene18358\_JingChaoZuZhi2, Unigene21049\_JingChaoZuZhi2, Unigene21176\_JingChaoZuZhi2, Unigene2235\_JingChaoZuZhi2, Unigene4377\_JingChaoZuZhi2, Unigene44961\_JingChaoZuZhi2, Unigene45887\_JingChaoZuZhi2, Unigene46908\_JingChaoZuZhi2, Unigene53809\_JingChaoZuZhi2, Unigene57061\_JingChaoZuZhi2, Unigene57130\_JingChaoZuZhi2, Unigene63649\_JingChaoZuZhi2, Unigene67613\_JingChaoZuZhi2, Unigene68452\_JingChaoZuZhi2, Unigene71976\_JingChaoZuZhi2, Unigene72476\_JingChaoZuZhi2, Unigene73451\_JingChaoZuZhi2, Unigene74590\_JingChaoZuZhi2, Unigene79198\_JingChaoZuZhi2, Unigene80285\_JingChaoZuZhi2, Unigene80881\_JingChaoZuZhi2, Unigene82129\_JingChaoZuZhi2, Unigene82270\_JingChaoZuZhi2, Unigene82818\_JingChaoZuZhi2, Unigene84063\_JingChaoZuZhi2, Unigene84385\_JingChaoZuZhi2, Unigene84455\_JingChaoZuZhi2, Unigene84851\_JingChaoZuZhi2, Unigene87044\_JingChaoZuZhi2, Unigene88238\_JingChaoZuZhi2, Unigene90127\_JingChaoZuZhi2, Unigene91700\_JingChaoZuZhi2, Unigene93334\_JingChaoZuZhi2, Unigene93877\_JingChaoZuZhi2, Unigene94837\_JingChaoZuZhi2, Unigene95959\_JingChaoZuZhi2, Unigene95985\_JingChaoZuZhi2, Unigene96031\_JingChaoZuZhi2 |
| 176 | Valine, leucine and isoleucine biosynthesis | Unigene10831\_JingChaoZuZhi2, Unigene11349\_JingChaoZuZhi2, Unigene15755\_JingChaoZuZhi2, Unigene15856\_JingChaoZuZhi2, Unigene16069\_JingChaoZuZhi2, Unigene16081\_JingChaoZuZhi2, Unigene17125\_JingChaoZuZhi2, Unigene21393\_JingChaoZuZhi2, Unigene2321\_JingChaoZuZhi2, Unigene36183\_JingChaoZuZhi2, Unigene44292\_JingChaoZuZhi2, Unigene45733\_JingChaoZuZhi2, Unigene490\_JingChaoZuZhi2, Unigene5057\_JingChaoZuZhi2, Unigene52097\_JingChaoZuZhi2, Unigene56572\_JingChaoZuZhi2, Unigene57635\_JingChaoZuZhi2, Unigene59449\_JingChaoZuZhi2, Unigene62056\_JingChaoZuZhi2, Unigene62948\_JingChaoZuZhi2, Unigene65838\_JingChaoZuZhi2, Unigene65915\_JingChaoZuZhi2, Unigene6846\_JingChaoZuZhi2, Unigene69185\_JingChaoZuZhi2, Unigene69398\_JingChaoZuZhi2, Unigene69404\_JingChaoZuZhi2, Unigene72032\_JingChaoZuZhi2, Unigene73946\_JingChaoZuZhi2, Unigene74244\_JingChaoZuZhi2, Unigene74650\_JingChaoZuZhi2, Unigene77756\_JingChaoZuZhi2, Unigene79003\_JingChaoZuZhi2, Unigene8020\_JingChaoZuZhi2, Unigene84539\_JingChaoZuZhi2, Unigene91997\_JingChaoZuZhi2, Unigene93422\_JingChaoZuZhi2, Unigene93786\_JingChaoZuZhi2, Unigene95470\_JingChaoZuZhi2, Unigene96021\_JingChaoZuZhi2 |
| 177 | Regulation of autophagy | Unigene11742\_JingChaoZuZhi2, Unigene12422\_JingChaoZuZhi2, Unigene14108\_JingChaoZuZhi2, Unigene14452\_JingChaoZuZhi2, Unigene15165\_JingChaoZuZhi2, Unigene15386\_JingChaoZuZhi2, Unigene18176\_JingChaoZuZhi2, Unigene18300\_JingChaoZuZhi2, Unigene18729\_JingChaoZuZhi2, Unigene1878\_JingChaoZuZhi2, Unigene19187\_JingChaoZuZhi2, Unigene19615\_JingChaoZuZhi2, Unigene19701\_JingChaoZuZhi2, Unigene21020\_JingChaoZuZhi2, Unigene21132\_JingChaoZuZhi2, Unigene2337\_JingChaoZuZhi2, Unigene34259\_JingChaoZuZhi2, Unigene47248\_JingChaoZuZhi2, Unigene50201\_JingChaoZuZhi2, Unigene58478\_JingChaoZuZhi2, Unigene61559\_JingChaoZuZhi2, Unigene69069\_JingChaoZuZhi2, Unigene69666\_JingChaoZuZhi2, Unigene73252\_JingChaoZuZhi2, Unigene74690\_JingChaoZuZhi2, Unigene75410\_JingChaoZuZhi2, Unigene77172\_JingChaoZuZhi2, Unigene79268\_JingChaoZuZhi2, Unigene86344\_JingChaoZuZhi2, Unigene86995\_JingChaoZuZhi2, Unigene89979\_JingChaoZuZhi2, Unigene91648\_JingChaoZuZhi2, Unigene93442\_JingChaoZuZhi2, Unigene94007\_JingChaoZuZhi2, Unigene94883\_JingChaoZuZhi2, Unigene94898\_JingChaoZuZhi2, Unigene9782\_JingChaoZuZhi2, Unigene9995\_JingChaoZuZhi2 |
| 178 | Selenoamino acid metabolism | Unigene10192\_JingChaoZuZhi2, Unigene11153\_JingChaoZuZhi2, Unigene19048\_JingChaoZuZhi2, Unigene19801\_JingChaoZuZhi2, Unigene20143\_JingChaoZuZhi2, Unigene21805\_JingChaoZuZhi2, Unigene22601\_JingChaoZuZhi2, Unigene22904\_JingChaoZuZhi2, Unigene289\_JingChaoZuZhi2, Unigene38309\_JingChaoZuZhi2, Unigene46569\_JingChaoZuZhi2, Unigene48770\_JingChaoZuZhi2, Unigene50289\_JingChaoZuZhi2, Unigene58985\_JingChaoZuZhi2, Unigene60177\_JingChaoZuZhi2, Unigene63447\_JingChaoZuZhi2, Unigene64994\_JingChaoZuZhi2, Unigene69787\_JingChaoZuZhi2, Unigene74736\_JingChaoZuZhi2, Unigene76502\_JingChaoZuZhi2, Unigene76685\_JingChaoZuZhi2, Unigene7755\_JingChaoZuZhi2, Unigene8159\_JingChaoZuZhi2, Unigene8173\_JingChaoZuZhi2, Unigene81795\_JingChaoZuZhi2, Unigene87966\_JingChaoZuZhi2, Unigene90393\_JingChaoZuZhi2, Unigene90730\_JingChaoZuZhi2, Unigene94614\_JingChaoZuZhi2, Unigene94622\_JingChaoZuZhi2, Unigene94707\_JingChaoZuZhi2, Unigene95481\_JingChaoZuZhi2, Unigene95575\_JingChaoZuZhi2, Unigene95591\_JingChaoZuZhi2, Unigene95673\_JingChaoZuZhi2 |
| 179 | Histidine metabolism | Unigene10192\_JingChaoZuZhi2, Unigene11153\_JingChaoZuZhi2, Unigene18358\_JingChaoZuZhi2, Unigene19048\_JingChaoZuZhi2, Unigene21049\_JingChaoZuZhi2, Unigene21176\_JingChaoZuZhi2, Unigene2235\_JingChaoZuZhi2, Unigene36078\_JingChaoZuZhi2, Unigene50289\_JingChaoZuZhi2, Unigene58985\_JingChaoZuZhi2, Unigene63649\_JingChaoZuZhi2, Unigene64994\_JingChaoZuZhi2, Unigene67613\_JingChaoZuZhi2, Unigene68015\_JingChaoZuZhi2, Unigene69787\_JingChaoZuZhi2, Unigene72476\_JingChaoZuZhi2, Unigene76685\_JingChaoZuZhi2, Unigene7755\_JingChaoZuZhi2, Unigene80881\_JingChaoZuZhi2, Unigene8173\_JingChaoZuZhi2, Unigene81795\_JingChaoZuZhi2, Unigene82129\_JingChaoZuZhi2, Unigene84063\_JingChaoZuZhi2, Unigene84851\_JingChaoZuZhi2, Unigene87044\_JingChaoZuZhi2, Unigene87664\_JingChaoZuZhi2, Unigene90127\_JingChaoZuZhi2, Unigene91700\_JingChaoZuZhi2, Unigene93334\_JingChaoZuZhi2, Unigene94622\_JingChaoZuZhi2, Unigene94837\_JingChaoZuZhi2, Unigene95673\_JingChaoZuZhi2, Unigene95959\_JingChaoZuZhi2, Unigene95985\_JingChaoZuZhi2, Unigene96031\_JingChaoZuZhi2 |
| 180 | Pantothenate and CoA biosynthesis | Unigene14716\_JingChaoZuZhi2, Unigene15461\_JingChaoZuZhi2, Unigene15770\_JingChaoZuZhi2, Unigene16010\_JingChaoZuZhi2, Unigene17138\_JingChaoZuZhi2, Unigene18000\_JingChaoZuZhi2, Unigene18665\_JingChaoZuZhi2, Unigene22392\_JingChaoZuZhi2, Unigene22416\_JingChaoZuZhi2, Unigene2553\_JingChaoZuZhi2, Unigene2730\_JingChaoZuZhi2, Unigene36386\_JingChaoZuZhi2, Unigene37317\_JingChaoZuZhi2, Unigene42890\_JingChaoZuZhi2, Unigene43926\_JingChaoZuZhi2, Unigene44292\_JingChaoZuZhi2, Unigene53362\_JingChaoZuZhi2, Unigene59836\_JingChaoZuZhi2, Unigene662\_JingChaoZuZhi2, Unigene67981\_JingChaoZuZhi2, Unigene68063\_JingChaoZuZhi2, Unigene69209\_JingChaoZuZhi2, Unigene70824\_JingChaoZuZhi2, Unigene71235\_JingChaoZuZhi2, Unigene76915\_JingChaoZuZhi2, Unigene79590\_JingChaoZuZhi2, Unigene85640\_JingChaoZuZhi2, Unigene86295\_JingChaoZuZhi2, Unigene90292\_JingChaoZuZhi2, Unigene90999\_JingChaoZuZhi2, Unigene91914\_JingChaoZuZhi2, Unigene93589\_JingChaoZuZhi2, Unigene93666\_JingChaoZuZhi2, Unigene95155\_JingChaoZuZhi2 |
| 181 | Nitrogen metabolism | Unigene10394\_JingChaoZuZhi2, Unigene11433\_JingChaoZuZhi2, Unigene15992\_JingChaoZuZhi2, Unigene18977\_JingChaoZuZhi2, Unigene20163\_JingChaoZuZhi2, Unigene20651\_JingChaoZuZhi2, Unigene21428\_JingChaoZuZhi2, Unigene22601\_JingChaoZuZhi2, Unigene30780\_JingChaoZuZhi2, Unigene39299\_JingChaoZuZhi2, Unigene42036\_JingChaoZuZhi2, Unigene46280\_JingChaoZuZhi2, Unigene49757\_JingChaoZuZhi2, Unigene52911\_JingChaoZuZhi2, Unigene54075\_JingChaoZuZhi2, Unigene5545\_JingChaoZuZhi2, Unigene63391\_JingChaoZuZhi2, Unigene69271\_JingChaoZuZhi2, Unigene70672\_JingChaoZuZhi2, Unigene7110\_JingChaoZuZhi2, Unigene71728\_JingChaoZuZhi2, Unigene73896\_JingChaoZuZhi2, Unigene76438\_JingChaoZuZhi2, Unigene80275\_JingChaoZuZhi2, Unigene83716\_JingChaoZuZhi2, Unigene84416\_JingChaoZuZhi2, Unigene85428\_JingChaoZuZhi2, Unigene89496\_JingChaoZuZhi2, Unigene89813\_JingChaoZuZhi2, Unigene90147\_JingChaoZuZhi2, Unigene91344\_JingChaoZuZhi2, Unigene9446\_JingChaoZuZhi2, Unigene95063\_JingChaoZuZhi2, Unigene95958\_JingChaoZuZhi2 |
| 182 | Non-homologous end-joining | Unigene10165\_JingChaoZuZhi2, Unigene13756\_JingChaoZuZhi2, Unigene13986\_JingChaoZuZhi2, Unigene14776\_JingChaoZuZhi2, Unigene14972\_JingChaoZuZhi2, Unigene15428\_JingChaoZuZhi2, Unigene16657\_JingChaoZuZhi2, Unigene17164\_JingChaoZuZhi2, Unigene1833\_JingChaoZuZhi2, Unigene20041\_JingChaoZuZhi2, Unigene20352\_JingChaoZuZhi2, Unigene21061\_JingChaoZuZhi2, Unigene4628\_JingChaoZuZhi2, Unigene55127\_JingChaoZuZhi2, Unigene58089\_JingChaoZuZhi2, Unigene61015\_JingChaoZuZhi2, Unigene63320\_JingChaoZuZhi2, Unigene6477\_JingChaoZuZhi2, Unigene66737\_JingChaoZuZhi2, Unigene69295\_JingChaoZuZhi2, Unigene71542\_JingChaoZuZhi2, Unigene77688\_JingChaoZuZhi2, Unigene78008\_JingChaoZuZhi2, Unigene78372\_JingChaoZuZhi2, Unigene78458\_JingChaoZuZhi2, Unigene78962\_JingChaoZuZhi2, Unigene79229\_JingChaoZuZhi2, Unigene79887\_JingChaoZuZhi2, Unigene84706\_JingChaoZuZhi2, Unigene87923\_JingChaoZuZhi2, Unigene89756\_JingChaoZuZhi2, Unigene90116\_JingChaoZuZhi2, Unigene94779\_JingChaoZuZhi2, Unigene95113\_JingChaoZuZhi2 |
| 183 | Maturity onset diabetes of the young | Unigene11606\_JingChaoZuZhi2, Unigene14374\_JingChaoZuZhi2, Unigene16551\_JingChaoZuZhi2, Unigene16884\_JingChaoZuZhi2, Unigene19704\_JingChaoZuZhi2, Unigene27995\_JingChaoZuZhi2, Unigene28893\_JingChaoZuZhi2, Unigene3776\_JingChaoZuZhi2, Unigene43929\_JingChaoZuZhi2, Unigene48978\_JingChaoZuZhi2, Unigene50934\_JingChaoZuZhi2, Unigene50998\_JingChaoZuZhi2, Unigene5306\_JingChaoZuZhi2, Unigene580\_JingChaoZuZhi2, Unigene58209\_JingChaoZuZhi2, Unigene64281\_JingChaoZuZhi2, Unigene64529\_JingChaoZuZhi2, Unigene65775\_JingChaoZuZhi2, Unigene73344\_JingChaoZuZhi2, Unigene73475\_JingChaoZuZhi2, Unigene75121\_JingChaoZuZhi2, Unigene75\_JingChaoZuZhi2, Unigene77137\_JingChaoZuZhi2, Unigene8234\_JingChaoZuZhi2, Unigene83450\_JingChaoZuZhi2, Unigene83637\_JingChaoZuZhi2, Unigene86404\_JingChaoZuZhi2, Unigene89285\_JingChaoZuZhi2, Unigene89457\_JingChaoZuZhi2, Unigene90738\_JingChaoZuZhi2, Unigene90903\_JingChaoZuZhi2, Unigene93060\_JingChaoZuZhi2, Unigene93317\_JingChaoZuZhi2 |
| 184 | Autoimmune thyroid disease | Unigene14842\_JingChaoZuZhi2, Unigene15780\_JingChaoZuZhi2, Unigene18921\_JingChaoZuZhi2, Unigene21759\_JingChaoZuZhi2, Unigene22546\_JingChaoZuZhi2, Unigene28889\_JingChaoZuZhi2, Unigene39577\_JingChaoZuZhi2, Unigene39829\_JingChaoZuZhi2, Unigene42346\_JingChaoZuZhi2, Unigene42610\_JingChaoZuZhi2, Unigene46219\_JingChaoZuZhi2, Unigene51012\_JingChaoZuZhi2, Unigene51705\_JingChaoZuZhi2, Unigene5240\_JingChaoZuZhi2, Unigene5657\_JingChaoZuZhi2, Unigene58354\_JingChaoZuZhi2, Unigene61189\_JingChaoZuZhi2, Unigene63226\_JingChaoZuZhi2, Unigene65294\_JingChaoZuZhi2, Unigene67589\_JingChaoZuZhi2, Unigene68108\_JingChaoZuZhi2, Unigene68200\_JingChaoZuZhi2, Unigene70505\_JingChaoZuZhi2, Unigene78298\_JingChaoZuZhi2, Unigene78957\_JingChaoZuZhi2, Unigene79577\_JingChaoZuZhi2, Unigene8202\_JingChaoZuZhi2, Unigene8248\_JingChaoZuZhi2, Unigene92135\_JingChaoZuZhi2, Unigene92942\_JingChaoZuZhi2 |
| 185 | Terpenoid backbone biosynthesis | Unigene10302\_JingChaoZuZhi2, Unigene11312\_JingChaoZuZhi2, Unigene12101\_JingChaoZuZhi2, Unigene12387\_JingChaoZuZhi2, Unigene14300\_JingChaoZuZhi2, Unigene17217\_JingChaoZuZhi2, Unigene17773\_JingChaoZuZhi2, Unigene17995\_JingChaoZuZhi2, Unigene21877\_JingChaoZuZhi2, Unigene25672\_JingChaoZuZhi2, Unigene27219\_JingChaoZuZhi2, Unigene31040\_JingChaoZuZhi2, Unigene3632\_JingChaoZuZhi2, Unigene42651\_JingChaoZuZhi2, Unigene5663\_JingChaoZuZhi2, Unigene58763\_JingChaoZuZhi2, Unigene5897\_JingChaoZuZhi2, Unigene6247\_JingChaoZuZhi2, Unigene65214\_JingChaoZuZhi2, Unigene75514\_JingChaoZuZhi2, Unigene81774\_JingChaoZuZhi2, Unigene83110\_JingChaoZuZhi2, Unigene83851\_JingChaoZuZhi2, Unigene8442\_JingChaoZuZhi2, Unigene84790\_JingChaoZuZhi2, Unigene84876\_JingChaoZuZhi2, Unigene85224\_JingChaoZuZhi2, Unigene87857\_JingChaoZuZhi2, Unigene90188\_JingChaoZuZhi2, Unigene9973\_JingChaoZuZhi2 |
| 186 | Glyoxylate and dicarboxylate metabolism | Unigene10133\_JingChaoZuZhi2, Unigene10384\_JingChaoZuZhi2, Unigene11119\_JingChaoZuZhi2, Unigene11150\_JingChaoZuZhi2, Unigene11892\_JingChaoZuZhi2, Unigene22325\_JingChaoZuZhi2, Unigene2306\_JingChaoZuZhi2, Unigene33822\_JingChaoZuZhi2, Unigene42373\_JingChaoZuZhi2, Unigene44116\_JingChaoZuZhi2, Unigene51682\_JingChaoZuZhi2, Unigene53419\_JingChaoZuZhi2, Unigene67411\_JingChaoZuZhi2, Unigene69155\_JingChaoZuZhi2, Unigene69752\_JingChaoZuZhi2, Unigene70893\_JingChaoZuZhi2, Unigene7591\_JingChaoZuZhi2, Unigene84417\_JingChaoZuZhi2, Unigene84532\_JingChaoZuZhi2, Unigene85584\_JingChaoZuZhi2, Unigene87054\_JingChaoZuZhi2, Unigene92990\_JingChaoZuZhi2, Unigene94125\_JingChaoZuZhi2, Unigene94464\_JingChaoZuZhi2, Unigene94745\_JingChaoZuZhi2, Unigene95384\_JingChaoZuZhi2, Unigene95648\_JingChaoZuZhi2, Unigene9748\_JingChaoZuZhi2 |
| 187 | Phenylalanine metabolism | Unigene10827\_JingChaoZuZhi2, Unigene17667\_JingChaoZuZhi2, Unigene18788\_JingChaoZuZhi2, Unigene19552\_JingChaoZuZhi2, Unigene196\_JingChaoZuZhi2, Unigene22546\_JingChaoZuZhi2, Unigene2431\_JingChaoZuZhi2, Unigene34150\_JingChaoZuZhi2, Unigene38448\_JingChaoZuZhi2, Unigene4854\_JingChaoZuZhi2, Unigene5240\_JingChaoZuZhi2, Unigene62603\_JingChaoZuZhi2, Unigene66806\_JingChaoZuZhi2, Unigene70659\_JingChaoZuZhi2, Unigene71936\_JingChaoZuZhi2, Unigene72476\_JingChaoZuZhi2, Unigene80142\_JingChaoZuZhi2, Unigene8075\_JingChaoZuZhi2, Unigene83634\_JingChaoZuZhi2, Unigene85687\_JingChaoZuZhi2, Unigene8689\_JingChaoZuZhi2, Unigene89277\_JingChaoZuZhi2, Unigene93088\_JingChaoZuZhi2, Unigene93264\_JingChaoZuZhi2, Unigene93334\_JingChaoZuZhi2, Unigene95198\_JingChaoZuZhi2, Unigene95658\_JingChaoZuZhi2, Unigene95695\_JingChaoZuZhi2 |
| 188 | Type I diabetes mellitus | Unigene11120\_JingChaoZuZhi2, Unigene11755\_JingChaoZuZhi2, Unigene13380\_JingChaoZuZhi2, Unigene14717\_JingChaoZuZhi2, Unigene14842\_JingChaoZuZhi2, Unigene15834\_JingChaoZuZhi2, Unigene16897\_JingChaoZuZhi2, Unigene17763\_JingChaoZuZhi2, Unigene35450\_JingChaoZuZhi2, Unigene39577\_JingChaoZuZhi2, Unigene46219\_JingChaoZuZhi2, Unigene47206\_JingChaoZuZhi2, Unigene51705\_JingChaoZuZhi2, Unigene55906\_JingChaoZuZhi2, Unigene63226\_JingChaoZuZhi2, Unigene65294\_JingChaoZuZhi2, Unigene68108\_JingChaoZuZhi2, Unigene68200\_JingChaoZuZhi2, Unigene70505\_JingChaoZuZhi2, Unigene72849\_JingChaoZuZhi2, Unigene78957\_JingChaoZuZhi2, Unigene8202\_JingChaoZuZhi2, Unigene8248\_JingChaoZuZhi2, Unigene82594\_JingChaoZuZhi2, Unigene91946\_JingChaoZuZhi2 |
| 189 | Fatty acid biosynthesis | Unigene11719\_JingChaoZuZhi2, Unigene14603\_JingChaoZuZhi2, Unigene15115\_JingChaoZuZhi2, Unigene17691\_JingChaoZuZhi2, Unigene20129\_JingChaoZuZhi2, Unigene2342\_JingChaoZuZhi2, Unigene24985\_JingChaoZuZhi2, Unigene31336\_JingChaoZuZhi2, Unigene3769\_JingChaoZuZhi2, Unigene47888\_JingChaoZuZhi2, Unigene52040\_JingChaoZuZhi2, Unigene57015\_JingChaoZuZhi2, Unigene58224\_JingChaoZuZhi2, Unigene60906\_JingChaoZuZhi2, Unigene61304\_JingChaoZuZhi2, Unigene62415\_JingChaoZuZhi2, Unigene67673\_JingChaoZuZhi2, Unigene6862\_JingChaoZuZhi2, Unigene75617\_JingChaoZuZhi2, Unigene77649\_JingChaoZuZhi2, Unigene80971\_JingChaoZuZhi2, Unigene85473\_JingChaoZuZhi2, Unigene89444\_JingChaoZuZhi2, Unigene92251\_JingChaoZuZhi2 |
| 190 | Steroid biosynthesis | Unigene10268\_JingChaoZuZhi2, Unigene12083\_JingChaoZuZhi2, Unigene13538\_JingChaoZuZhi2, Unigene18929\_JingChaoZuZhi2, Unigene20631\_JingChaoZuZhi2, Unigene33308\_JingChaoZuZhi2, Unigene4072\_JingChaoZuZhi2, Unigene42157\_JingChaoZuZhi2, Unigene44278\_JingChaoZuZhi2, Unigene46619\_JingChaoZuZhi2, Unigene51085\_JingChaoZuZhi2, Unigene52259\_JingChaoZuZhi2, Unigene73018\_JingChaoZuZhi2, Unigene79315\_JingChaoZuZhi2, Unigene79515\_JingChaoZuZhi2, Unigene86307\_JingChaoZuZhi2, Unigene86770\_JingChaoZuZhi2, Unigene89232\_JingChaoZuZhi2, Unigene90190\_JingChaoZuZhi2, Unigene90531\_JingChaoZuZhi2, Unigene91755\_JingChaoZuZhi2, Unigene93513\_JingChaoZuZhi2, Unigene95693\_JingChaoZuZhi2 |
| 191 | Fatty acid elongation in mitochondria | Unigene11808\_JingChaoZuZhi2, Unigene16863\_JingChaoZuZhi2, Unigene18179\_JingChaoZuZhi2, Unigene18409\_JingChaoZuZhi2, Unigene18553\_JingChaoZuZhi2, Unigene21821\_JingChaoZuZhi2, Unigene22793\_JingChaoZuZhi2, Unigene50371\_JingChaoZuZhi2, Unigene5068\_JingChaoZuZhi2, Unigene51532\_JingChaoZuZhi2, Unigene61664\_JingChaoZuZhi2, Unigene63715\_JingChaoZuZhi2, Unigene64903\_JingChaoZuZhi2, Unigene66337\_JingChaoZuZhi2, Unigene84343\_JingChaoZuZhi2, Unigene8454\_JingChaoZuZhi2, Unigene87485\_JingChaoZuZhi2, Unigene90838\_JingChaoZuZhi2, Unigene93681\_JingChaoZuZhi2, Unigene93843\_JingChaoZuZhi2, Unigene95269\_JingChaoZuZhi2, Unigene9580\_JingChaoZuZhi2, Unigene95986\_JingChaoZuZhi2 |
| 192 | Glycosphingolipid biosynthesis - globo series | Unigene10002\_JingChaoZuZhi2, Unigene15728\_JingChaoZuZhi2, Unigene19620\_JingChaoZuZhi2, Unigene19754\_JingChaoZuZhi2, Unigene36907\_JingChaoZuZhi2, Unigene37067\_JingChaoZuZhi2, Unigene37453\_JingChaoZuZhi2, Unigene51610\_JingChaoZuZhi2, Unigene67946\_JingChaoZuZhi2, Unigene7059\_JingChaoZuZhi2, Unigene71248\_JingChaoZuZhi2, Unigene76668\_JingChaoZuZhi2, Unigene80848\_JingChaoZuZhi2, Unigene81654\_JingChaoZuZhi2, Unigene83023\_JingChaoZuZhi2, Unigene85353\_JingChaoZuZhi2, Unigene88528\_JingChaoZuZhi2, Unigene92869\_JingChaoZuZhi2, Unigene94286\_JingChaoZuZhi2, Unigene95596\_JingChaoZuZhi2, Unigene96039\_JingChaoZuZhi2, Unigene96163\_JingChaoZuZhi2 |
| 193 | Primary immunodeficiency | Unigene15428\_JingChaoZuZhi2, Unigene17164\_JingChaoZuZhi2, Unigene20426\_JingChaoZuZhi2, Unigene22466\_JingChaoZuZhi2, Unigene38133\_JingChaoZuZhi2, Unigene44797\_JingChaoZuZhi2, Unigene45962\_JingChaoZuZhi2, Unigene6211\_JingChaoZuZhi2, Unigene63374\_JingChaoZuZhi2, Unigene63458\_JingChaoZuZhi2, Unigene64957\_JingChaoZuZhi2, Unigene69295\_JingChaoZuZhi2, Unigene70342\_JingChaoZuZhi2, Unigene76369\_JingChaoZuZhi2, Unigene78008\_JingChaoZuZhi2, Unigene78458\_JingChaoZuZhi2, Unigene7850\_JingChaoZuZhi2, Unigene79887\_JingChaoZuZhi2, Unigene79990\_JingChaoZuZhi2, Unigene94762\_JingChaoZuZhi2 |
| 194 | Sulfur metabolism | Unigene17089\_JingChaoZuZhi2, Unigene18284\_JingChaoZuZhi2, Unigene18526\_JingChaoZuZhi2, Unigene18576\_JingChaoZuZhi2, Unigene19859\_JingChaoZuZhi2, Unigene20422\_JingChaoZuZhi2, Unigene20583\_JingChaoZuZhi2, Unigene20729\_JingChaoZuZhi2, Unigene22726\_JingChaoZuZhi2, Unigene44707\_JingChaoZuZhi2, Unigene60494\_JingChaoZuZhi2, Unigene6250\_JingChaoZuZhi2, Unigene79757\_JingChaoZuZhi2, Unigene8781\_JingChaoZuZhi2, Unigene88888\_JingChaoZuZhi2, Unigene90730\_JingChaoZuZhi2, Unigene93039\_JingChaoZuZhi2, Unigene93876\_JingChaoZuZhi2, Unigene95247\_JingChaoZuZhi2, Unigene95591\_JingChaoZuZhi2 |
| 195 | Folate biosynthesis | Unigene10542\_JingChaoZuZhi2, Unigene10763\_JingChaoZuZhi2, Unigene13388\_JingChaoZuZhi2, Unigene15358\_JingChaoZuZhi2, Unigene21698\_JingChaoZuZhi2, Unigene22297\_JingChaoZuZhi2, Unigene31032\_JingChaoZuZhi2, Unigene42846\_JingChaoZuZhi2, Unigene4334\_JingChaoZuZhi2, Unigene64991\_JingChaoZuZhi2, Unigene72384\_JingChaoZuZhi2, Unigene73297\_JingChaoZuZhi2, Unigene77892\_JingChaoZuZhi2, Unigene8175\_JingChaoZuZhi2, Unigene8512\_JingChaoZuZhi2, Unigene86322\_JingChaoZuZhi2, Unigene91429\_JingChaoZuZhi2, Unigene91556\_JingChaoZuZhi2, Unigene93043\_JingChaoZuZhi2, Unigene93804\_JingChaoZuZhi2 |
| 196 | Ubiquinone and other terpenoid-quinone biosynthesis | Unigene10827\_JingChaoZuZhi2, Unigene14414\_JingChaoZuZhi2, Unigene17667\_JingChaoZuZhi2, Unigene21507\_JingChaoZuZhi2, Unigene2431\_JingChaoZuZhi2, Unigene34150\_JingChaoZuZhi2, Unigene4763\_JingChaoZuZhi2, Unigene4854\_JingChaoZuZhi2, Unigene56290\_JingChaoZuZhi2, Unigene71936\_JingChaoZuZhi2, Unigene729\_JingChaoZuZhi2, Unigene78672\_JingChaoZuZhi2, Unigene7966\_JingChaoZuZhi2, Unigene80142\_JingChaoZuZhi2, Unigene8075\_JingChaoZuZhi2, Unigene85687\_JingChaoZuZhi2, Unigene8689\_JingChaoZuZhi2, Unigene90658\_JingChaoZuZhi2, Unigene93088\_JingChaoZuZhi2 |
| 197 | Glycosaminoglycan biosynthesis - keratan sulfate (no map in kegg database) | Unigene10002\_JingChaoZuZhi2, Unigene1071\_JingChaoZuZhi2, Unigene11412\_JingChaoZuZhi2, Unigene12736\_JingChaoZuZhi2, Unigene13003\_JingChaoZuZhi2, Unigene21754\_JingChaoZuZhi2, Unigene22403\_JingChaoZuZhi2, Unigene22474\_JingChaoZuZhi2, Unigene45708\_JingChaoZuZhi2, Unigene69176\_JingChaoZuZhi2, Unigene80218\_JingChaoZuZhi2, Unigene80736\_JingChaoZuZhi2, Unigene86608\_JingChaoZuZhi2, Unigene87999\_JingChaoZuZhi2, Unigene88709\_JingChaoZuZhi2, Unigene92869\_JingChaoZuZhi2, Unigene94957\_JingChaoZuZhi2, Unigene95555\_JingChaoZuZhi2 |
| 198 | Glycosphingolipid biosynthesis - lacto and neolacto series | Unigene10002\_JingChaoZuZhi2, Unigene12516\_JingChaoZuZhi2, Unigene12736\_JingChaoZuZhi2, Unigene13003\_JingChaoZuZhi2, Unigene15201\_JingChaoZuZhi2, Unigene21754\_JingChaoZuZhi2, Unigene22403\_JingChaoZuZhi2, Unigene22474\_JingChaoZuZhi2, Unigene3366\_JingChaoZuZhi2, Unigene45708\_JingChaoZuZhi2, Unigene69176\_JingChaoZuZhi2, Unigene76668\_JingChaoZuZhi2, Unigene86608\_JingChaoZuZhi2, Unigene87999\_JingChaoZuZhi2, Unigene88709\_JingChaoZuZhi2, Unigene92869\_JingChaoZuZhi2, Unigene95596\_JingChaoZuZhi2, Unigene95908\_JingChaoZuZhi2 |
| 199 | Glycosphingolipid biosynthesis - ganglio series | Unigene11681\_JingChaoZuZhi2, Unigene15201\_JingChaoZuZhi2, Unigene19251\_JingChaoZuZhi2, Unigene27301\_JingChaoZuZhi2, Unigene51610\_JingChaoZuZhi2, Unigene67946\_JingChaoZuZhi2, Unigene7059\_JingChaoZuZhi2, Unigene71248\_JingChaoZuZhi2, Unigene72924\_JingChaoZuZhi2, Unigene80848\_JingChaoZuZhi2, Unigene81654\_JingChaoZuZhi2, Unigene83023\_JingChaoZuZhi2, Unigene85353\_JingChaoZuZhi2, Unigene88528\_JingChaoZuZhi2, Unigene90891\_JingChaoZuZhi2, Unigene95102\_JingChaoZuZhi2, Unigene96039\_JingChaoZuZhi2 |
| 200 | Thiamine metabolism | Unigene19497\_JingChaoZuZhi2, Unigene19751\_JingChaoZuZhi2, Unigene20521\_JingChaoZuZhi2, Unigene20862\_JingChaoZuZhi2, Unigene22824\_JingChaoZuZhi2, Unigene2480\_JingChaoZuZhi2, Unigene41513\_JingChaoZuZhi2, Unigene69255\_JingChaoZuZhi2, Unigene73037\_JingChaoZuZhi2, Unigene75125\_JingChaoZuZhi2, Unigene77528\_JingChaoZuZhi2, Unigene79258\_JingChaoZuZhi2, Unigene80261\_JingChaoZuZhi2, Unigene84345\_JingChaoZuZhi2, Unigene90557\_JingChaoZuZhi2, Unigene95509\_JingChaoZuZhi2, Unigene95865\_JingChaoZuZhi2 |
| 201 | O-Mannosyl glycan biosynthesis (no map in kegg database) | Unigene1916\_JingChaoZuZhi2, Unigene20227\_JingChaoZuZhi2, Unigene25007\_JingChaoZuZhi2, Unigene52678\_JingChaoZuZhi2, Unigene55607\_JingChaoZuZhi2, Unigene64483\_JingChaoZuZhi2, Unigene70467\_JingChaoZuZhi2, Unigene71093\_JingChaoZuZhi2, Unigene7935\_JingChaoZuZhi2, Unigene81017\_JingChaoZuZhi2, Unigene81645\_JingChaoZuZhi2, Unigene85497\_JingChaoZuZhi2, Unigene89170\_JingChaoZuZhi2, Unigene89407\_JingChaoZuZhi2, Unigene95900\_JingChaoZuZhi2 |
| 202 | Primary bile acid biosynthesis | Unigene13176\_JingChaoZuZhi2, Unigene18846\_JingChaoZuZhi2, Unigene22072\_JingChaoZuZhi2, Unigene23634\_JingChaoZuZhi2, Unigene52259\_JingChaoZuZhi2, Unigene52\_JingChaoZuZhi2, Unigene54350\_JingChaoZuZhi2, Unigene73451\_JingChaoZuZhi2, Unigene74590\_JingChaoZuZhi2, Unigene78083\_JingChaoZuZhi2, Unigene79414\_JingChaoZuZhi2, Unigene90859\_JingChaoZuZhi2, Unigene93275\_JingChaoZuZhi2, Unigene93924\_JingChaoZuZhi2 |
| 203 | Synthesis and degradation of ketone bodies | Unigene11312\_JingChaoZuZhi2, Unigene17217\_JingChaoZuZhi2, Unigene17995\_JingChaoZuZhi2, Unigene27219\_JingChaoZuZhi2, Unigene44775\_JingChaoZuZhi2, Unigene5663\_JingChaoZuZhi2, Unigene58763\_JingChaoZuZhi2, Unigene64673\_JingChaoZuZhi2, Unigene70703\_JingChaoZuZhi2, Unigene79637\_JingChaoZuZhi2, Unigene8442\_JingChaoZuZhi2, Unigene85224\_JingChaoZuZhi2, Unigene89941\_JingChaoZuZhi2, Unigene96087\_JingChaoZuZhi2 |
| 204 | Cyanoamino acid metabolism | Unigene22904\_JingChaoZuZhi2, Unigene44196\_JingChaoZuZhi2, Unigene45512\_JingChaoZuZhi2, Unigene74736\_JingChaoZuZhi2, Unigene76424\_JingChaoZuZhi2, Unigene80622\_JingChaoZuZhi2, Unigene83634\_JingChaoZuZhi2, Unigene83939\_JingChaoZuZhi2, Unigene90392\_JingChaoZuZhi2, Unigene90393\_JingChaoZuZhi2, Unigene92027\_JingChaoZuZhi2, Unigene94614\_JingChaoZuZhi2, Unigene95695\_JingChaoZuZhi2 |
| 205 | Caffeine metabolism | Unigene12675\_JingChaoZuZhi2, Unigene12758\_JingChaoZuZhi2, Unigene18351\_JingChaoZuZhi2, Unigene18666\_JingChaoZuZhi2, Unigene19643\_JingChaoZuZhi2, Unigene36522\_JingChaoZuZhi2, Unigene47722\_JingChaoZuZhi2, Unigene5449\_JingChaoZuZhi2, Unigene54768\_JingChaoZuZhi2, Unigene63375\_JingChaoZuZhi2, Unigene63485\_JingChaoZuZhi2, Unigene87018\_JingChaoZuZhi2 |
| 206 | Allograft rejection | Unigene14842\_JingChaoZuZhi2, Unigene39577\_JingChaoZuZhi2, Unigene46219\_JingChaoZuZhi2, Unigene51705\_JingChaoZuZhi2, Unigene63226\_JingChaoZuZhi2, Unigene65294\_JingChaoZuZhi2, Unigene68108\_JingChaoZuZhi2, Unigene68200\_JingChaoZuZhi2, Unigene70505\_JingChaoZuZhi2, Unigene78957\_JingChaoZuZhi2, Unigene8202\_JingChaoZuZhi2, Unigene8248\_JingChaoZuZhi2 |
| 207 | Graft-versus-host disease | Unigene14842\_JingChaoZuZhi2, Unigene39577\_JingChaoZuZhi2, Unigene46219\_JingChaoZuZhi2, Unigene51705\_JingChaoZuZhi2, Unigene63226\_JingChaoZuZhi2, Unigene65294\_JingChaoZuZhi2, Unigene68108\_JingChaoZuZhi2, Unigene68200\_JingChaoZuZhi2, Unigene70505\_JingChaoZuZhi2, Unigene78957\_JingChaoZuZhi2, Unigene8202\_JingChaoZuZhi2, Unigene8248\_JingChaoZuZhi2 |
| 208 | Taurine and hypotaurine metabolism | Unigene21318\_JingChaoZuZhi2, Unigene22904\_JingChaoZuZhi2, Unigene32391\_JingChaoZuZhi2, Unigene35450\_JingChaoZuZhi2, Unigene47206\_JingChaoZuZhi2, Unigene55906\_JingChaoZuZhi2, Unigene72849\_JingChaoZuZhi2, Unigene74736\_JingChaoZuZhi2, Unigene82594\_JingChaoZuZhi2, Unigene90393\_JingChaoZuZhi2, Unigene94614\_JingChaoZuZhi2 |
| 209 | Lysine biosynthesis | Unigene18117\_JingChaoZuZhi2, Unigene21099\_JingChaoZuZhi2, Unigene53467\_JingChaoZuZhi2, Unigene55983\_JingChaoZuZhi2, Unigene6396\_JingChaoZuZhi2, Unigene80636\_JingChaoZuZhi2, Unigene81640\_JingChaoZuZhi2, Unigene95846\_JingChaoZuZhi2, Unigene95941\_JingChaoZuZhi2 |
| 210 | Insect hormone biosynthesis | Unigene10268\_JingChaoZuZhi2, Unigene13640\_JingChaoZuZhi2, Unigene45706\_JingChaoZuZhi2, Unigene52\_JingChaoZuZhi2, Unigene62924\_JingChaoZuZhi2, Unigene72767\_JingChaoZuZhi2, Unigene86307\_JingChaoZuZhi2 |
| 211 | Phenylalanine, tyrosine and tryptophan biosynthesis | Unigene18788\_JingChaoZuZhi2, Unigene38448\_JingChaoZuZhi2, Unigene70659\_JingChaoZuZhi2, Unigene8689\_JingChaoZuZhi2, Unigene89277\_JingChaoZuZhi2, Unigene93264\_JingChaoZuZhi2, Unigene95658\_JingChaoZuZhi2 |
| 212 | Butirosin and neomycin biosynthesis | Unigene1045\_JingChaoZuZhi2, Unigene16762\_JingChaoZuZhi2, Unigene48948\_JingChaoZuZhi2, Unigene70140\_JingChaoZuZhi2, Unigene77374\_JingChaoZuZhi2, Unigene79539\_JingChaoZuZhi2, Unigene83440\_JingChaoZuZhi2 |
| 213 | Lipoic acid metabolism | Unigene22727\_JingChaoZuZhi2, Unigene51504\_JingChaoZuZhi2, Unigene7767\_JingChaoZuZhi2, Unigene78070\_JingChaoZuZhi2, Unigene92638\_JingChaoZuZhi2, Unigene9977\_JingChaoZuZhi2 |
| 214 | Asthma | Unigene1422\_JingChaoZuZhi2, Unigene28889\_JingChaoZuZhi2, Unigene39829\_JingChaoZuZhi2, Unigene42610\_JingChaoZuZhi2, Unigene67589\_JingChaoZuZhi2, Unigene79577\_JingChaoZuZhi2 |
| 215 | D-Glutamine and D-glutamate metabolism | Unigene30780\_JingChaoZuZhi2, Unigene42036\_JingChaoZuZhi2, Unigene69271\_JingChaoZuZhi2, Unigene83716\_JingChaoZuZhi2, Unigene95958\_JingChaoZuZhi2 |
| 216 | Vitamin B6 metabolism | Unigene12758\_JingChaoZuZhi2, Unigene21557\_JingChaoZuZhi2, Unigene5449\_JingChaoZuZhi2, Unigene5496\_JingChaoZuZhi2, Unigene77193\_JingChaoZuZhi2 |
| 217 | Biotin metabolism | Unigene14424\_JingChaoZuZhi2, Unigene54387\_JingChaoZuZhi2, Unigene89040\_JingChaoZuZhi2 |
| 218 | D-Arginine and D-ornithine metabolism (no map in kegg database) | Unigene26104\_JingChaoZuZhi2, Unigene47407\_JingChaoZuZhi2 |
| 219 | Intestinal immune network for IgA production | Unigene19404\_JingChaoZuZhi2, Unigene85072\_JingChaoZuZhi2 |
| 220 | Polyketide sugar unit biosynthesis | Unigene95998\_JingChaoZuZhi2 |
